# Analysis of gene expression in the postmortem brain of neurotypical Black Americans reveals contributions of genetic ancestry

**DOI:** 10.1038/s41593-024-01636-0

**Published:** 2024-05-20

**Authors:** Kynon J. M. Benjamin, Qiang Chen, Nicholas J. Eagles, Louise A. Huuki-Myers, Leonardo Collado-Torres, Joshua M. Stolz, Geo Pertea, Joo Heon Shin, Apuã C. M. Paquola, Thomas M. Hyde, Joel E. Kleinman, Andrew E. Jaffe, Shizhong Han, Daniel R. Weinberger

**Affiliations:** 1 Lieber Institute for Brain Development, Baltimore, MD USA; 2 Department of Neurology, Johns Hopkins University School of Medicine, Baltimore, MD USA; 3 Department of Psychiatry and Behavioral Sciences, Johns Hopkins University School of Medicine, Baltimore, MD USA; 4 Center for Computational Biology, Johns Hopkins University, Baltimore, MD USA; 5 Department of Neuroscience, Johns Hopkins University School of Medicine, Baltimore, MD USA; 6 Neumora Therapeutics, Watertown, MA USA; 7 Department of Genetic Medicine, Johns Hopkins University School of Medicine, Baltimore, MD USA

**Keywords:** Gene expression, Stroke

## Abstract

Ancestral differences in genomic variation affect the regulation of gene expression; however, most gene expression studies have been limited to European ancestry samples or adjusted to identify ancestry-independent associations. Here, we instead examined the impact of genetic ancestry on gene expression and DNA methylation in the postmortem brain tissue of admixed Black American neurotypical individuals to identify ancestry-dependent and ancestry-independent contributions. Ancestry-associated differentially expressed genes (DEGs), transcripts and gene networks, while notably not implicating neurons, are enriched for genes related to the immune response and vascular tissue and explain up to 26% of heritability for ischemic stroke, 27% of heritability for Parkinson disease and 30% of heritability for Alzheimer’s disease. Ancestry-associated DEGs also show general enrichment for the heritability of diverse immune-related traits but depletion for psychiatric-related traits. We also compared Black and non-Hispanic white Americans, confirming most ancestry-associated DEGs. Our results delineate the extent to which genetic ancestry affects differences in gene expression in the human brain and the implications for brain illness risk.

## Abstract (web summary)

Studying gene expression in admixed Black Americans, Benjamin et al. reveal genetic ancestry-linked differences impacting immune and vascular genes and potentially influencing neurological disease risk. These findings highlight the importance of considering ancestry in brain research.

## Main

Race-based health disparities have endured for centuries^1^. In neuroscience and genomics, individuals with recent African genetic ancestry (AA) account for less than 5% of large-scale research cohorts for brain disorders but are 20% more likely to experience a major mental health crisis^2,3^. Insights gained from genome-wide association studies (GWAS) about disease risk are promising for clinical applications (for example, drug targets for new therapeutics and polygenic risk prediction). However, most GWAS of brain-related illness lack diversity with regard to the inclusion of individuals of AA, who account for less than 5% of GWAS participants^4^, despite AA individuals having more extensive genetic variation than any other population. This lack of diversity limits the accuracy of genetic risk prediction and hinders the development of effective personalized neurotherapeutics for individuals of non-European genetic ancestry^5^. While diversity in large-scale GWAS has increased in recent years (for example, the 1000 Genomes Project^6^, the All of Us research program^7^, the Trans-Omics for Precision Medicine (TOPMed) program^8^ and the Human Heredity and Health in Africa Consortium^9^), population-based genetic association studies do not directly elucidate potential biological mechanisms of risk variants. Cross-ancestry expression quantitative trait loci (eQTLs) have focused on improved fine mapping while leaving unanswered the question of how gene expression and epigenetic regulation are parsed specifically by ancestry^10^.

To bridge this gap, we need studies of the biological impact of genetic variation on molecular traits (for example, mRNA and DNA methylation) in disease-relevant tissues of diverse populations. An obvious impediment to undertaking this task is the limited availability of brain tissue from AA individuals. Currently, the most widely used resource for human postmortem tissue is the Genotype-Tissue Expression Project (GTEx), which has RNA sequencing (RNA-seq) and single-nucleotide polymorphism (SNP) genotype data from 13 brain regions (114–209 individuals per region). However, most GTEx brain samples are of European genetic ancestry (EA); for some brain regions, GTEx has no individuals of non-EA. In comparison, the BrainSeq Consortium, a collaboration between seven pharmaceutical companies and the Lieber Institute for Brain Development (LIBD), includes 784 samples from Black Americans (BAs) across 587 unique individuals, with a mean age of 44. While reports from this consortium and other large-scale analyses in the brain—including from the hippocampus, caudate nucleus, dorsolateral prefrontal cortex (DLPFC) and granule cells of the dentate gyrus—have samples of diverse genetic ancestry^10–16^, they have typically been ‘adjusted’ for ancestry status, which limits our understanding of ancestry-specific effects in the brain.

**Fig. 1:**
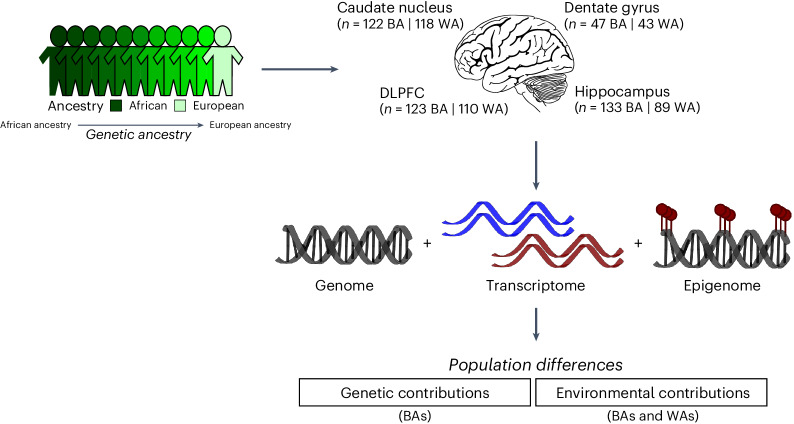
Study design for the examination of the genetic and nongenetic contributions to genetic ancestry-associated gene expression differences.

In this study, we used the LIBD RNA-seq, SNP genotype and whole-genome bisulfite sequencing (WGBS) datasets to evaluate differences in genetic ancestry in gene expression in the human brain (Fig. 1). We identified transcriptional features associated with genetic ancestry (African or European) in admixed neurotypical BA donors (*n* = 151). We quantified the contributions of common genetic variations to differences in genetic ancestry using a total of 425 samples, including the caudate nucleus (*n* = 122), dentate gyrus (*n* = 47), DLPFC (*n* = 123) and hippocampus (*n* = 133). Additionally, we examined the influence of genetic ancestry on DNA methylation using WGBS data of the admixed BA donors from the caudate nucleus (*n* = 89), DLPFC (*n* = 69) and hippocampus (*n* = 69). To confirm the genetic ancestry-associated differences in gene expression, we further examined transcriptional and DNA methylation differences in individuals of limited admixture (BAs ≥ 0.8 AA and white Americans (WAs) > 0.99 EA).

## Results

### Enrichment of immune response genes in genetic ancestry differentially expressed genes

We selectively examined the admixed BA population (151 unique individuals; Table 1) to (1) characterize transcriptional changes associated with African or European genetic ancestry in neurotypical adults (age > 17) and (2) limit the potential confounding effects of systematic environmental factors that may differ between BAs and non-Hispanic WAs. We used RNA-seq data from the caudate nucleus (*n* = 122), dentate gyrus (*n* *=* 47), DLPFC (*n* = 123) and hippocampus (*n* = 133). The admixed BA donors showed a varied proportion of EA (STRUCTURE^17^; EA mean = 0.21, range = 0–0.62; Supplementary Fig. 1) consistent with previous reports and the history of the slave trade^18,19^. We used these continuous genetic ancestry estimates to identify differentially expressed features (genes, transcripts, exons and junctions) linearly correlated with ancestry proportion and adjusted for sex, age and RNA quality. This RNA quality adjustment included experiment-based RNA degradation metrics that account for batch effect and cell composition^12,20^. To increase detection power and improve effect size estimation, we applied the multivariate adaptive shrinkage (‘mash’^21^) method, which leverages the correlation structure of genetic ancestry effects across brain regions (Methods).

**Table 1:** BA sample characteristics for adult (age > 17) neurotypical control postmortem caudate nucleus, dentate gyrus, DLPFC and hippocampus (10–12)

Characteristic	Brain region			
	Caudate nucleus	Dentate gyrus	DLPFC	Hippocampus
	*n* = 122	*n* = 47	*n* = 123	*n* = 133
Sex, *n* (%)				
Female	50 (41)	16 (34)	48 (39)	53 (40)
Male	72 (59)	31 (66)	75 (61)	80 (60)
Age, mean (s.d.)	46 (15)	46 (16)	44 (15)	43 (15)
RNA integrity number, mean (s.d.)	7.83 (0.80)	5.45 (1.22)	7.70 (0.89)	7.72 (0.98)

**Fig. 2:**
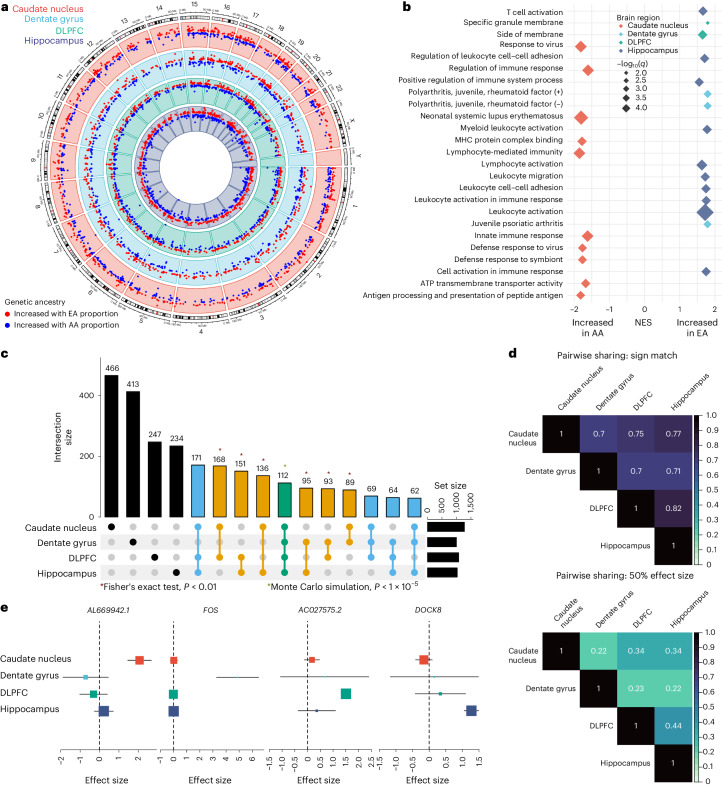
Extensive ancestry-associated expression changes across brain regions. **a**, Circos plot showing global ancestry DEGs across the caudate nucleus (red), dentate gyrus (blue), DLPFC (green) and hippocampus (purple). **b**, GSEA of differential expression analysis across brain regions, highlighting terms associated with increased African or European ancestry proportions based on normalized enrichment score (NES) direction of effect. **c**. UpSet plot showing large overlap between brain regions. Green is shared across the four brain regions; blue is shared across three brain regions; orange is shared between two brain regions; and black is unique to a specific brain region. The single asterisk indicates significant pairwise enrichment (two-sided Fisher’s exact test; *P* = 2.0 × 10^−135^ (caudate nucleus versus dentate gyrus), 4.9 × 10^−324^ (caudate nucleus versus DLPFC), 2.8 × 10^−288^ (caudate nucleus versus hippocampus), 1.8 × 10^−166^ (dentate gyrus versus DLPFC), 9.8 × 10^−169^ (dentate gyrus versus hippocampus) and approximately 0 (DLPFC versus hippocampus) or significant overlap between all four brain regions (Monte Carlo simulation). **d**, Heatmaps of the proportion of global ancestry DEG sharing with concordant direction (top, sign match) and within a factor 0.5 effect size (bottom) **e**, Metaplot showing examples of brain region-specific ancestry effects.

Of the 16,820 genes tested, we identified 2,570 (15%; 1,437 of which were protein-coding) unique differentially expressed genes (DEGs) based on global ancestry variation (local false sign rate (LFSR) < 0.05; Fig. 2a, Supplementary Table 1 and Supplementary Data 1) across the caudate nucleus (*n* = 1,273 DEGs), dentate gyrus (*n* = 997), DLPFC (*n* = 1,075) and hippocampus (*n* = 1,025). While this number increased when we examined local ancestry (9,906 (62% of genes tested); 6,982 protein-coding genes; Supplementary Table 2) across the caudate nucleus (*n* = 6,657 DEGs), dentate gyrus (*n* = 4,154), DLPFC (*n* = 6,148) and hippocampus (*n* = 7,006), effect sizes between global-ancestry and local-ancestry DEGs showed significant positive correlations (all Spearman rho > 0.57, *P* < 0.01; Supplementary Fig. 3) across all brain regions. When examining isoform-level associations (transcripts, exons and junctions), we found an additional 8,012 unique global ancestry-associated DEGs (LFSR < 0.05; Supplementary Fig. 2, Supplementary Table 1 and Supplementary Data 1) and 6,629 unique local ancestry-associated DEGs (LFSR < 0.05; Supplementary Table 2 and Supplementary Data 2) in these BAs. Similarly, we found that isoform-level local ancestry differentially expressed features showed a significant positive correlation in effect sizes compared with global ancestry differentially expressed features (Spearman, Supplementary Fig. 3). We also confirmed most of these ancestry-associated DEGs in a binary comparison of BAs and non-Hispanic WAs (Supplementary Note, Supplementary Figs. 42–45 and Supplementary Tables 5 and 6).

To evaluate the functional aspects of these genetic ancestry-associated DEGs (global and local ancestry), we performed gene set enrichment analysis (GSEA) for each brain region. Notably, while neuronal gene sets were not enriched, we observed significant enrichment (GSEA and hypergeometric testing, *q* < 0.05) for terms primarily related to the immune response, including innate, adaptive and virus responses (Supplementary Data 3, Fig. 2b and Supplementary Fig. 4). Interestingly, the caudate nucleus showed an opposite direction of effect compared with the dentate gyrus, DLPFC and hippocampus. Specifically, the caudate nucleus showed enrichment of the immune response associated with DEGs upregulated in the AA proportion, while the dentate gyrus, DLPFC and hippocampus showed enrichment for immune-related pathways associated with DEGs upregulated in the EA proportion (Fig. 2b and Supplementary Fig. 5). While not significant, we observed the same pattern of opposite directionality of effect for immune-related pathways with local ancestry-associated DEGs (Supplementary Fig. 6). The binary combined analysis (BAs and WAs) also revealed similar immune response enrichment with directionality dependent on brain region (Supplementary Note).

Expanding our analysis to the isoform level (transcripts, exons and junctions), we also found a significant association with immune-related pathways, with consistent upregulation for the AA proportion in the caudate nucleus and the EA proportion in the dentate gyrus, DLPFC and hippocampus. Additionally, we found significant enrichment of these DEGs for genes with population differences in macrophages^18^ associated with the innate immune response to infection (Fisher’s exact test, false discovery rate (FDR) < 0.05; Supplementary Fig. 7). Moreover, we found significant enrichment (Fisher’s exact test, FDR < 0.01) for global ancestry-associated DEGs in gene coexpression network modules (weighted gene coexpression network analysis^22^; Supplementary Fig. 8). Like our DEG analysis, immune response pathway enrichment in these modules showed a consistent opposite direction of effect based on brain region (Supplementary Fig. 9 and Supplementary Data 4 and 5).

Observing immune response pathway enrichment in bulk tissue, we performed cell type^23,24^ enrichment analysis to evaluate the cellular context of these ancestry-associated DEGs. We found significant enrichment of global ancestry DEGs (Fisher’s exact test, FDR < 0.05; Supplementary Figs. 10 and 11a) for genes specifically expressed in brain immune cells (that is, glia and microglia) and neurovasculature cells (that is, pericyte, endothelial and vascular tissue), but not peripheral immune cells. We also observed enrichment for distinct glial subtypes^25^ (Supplementary Fig. 12). Local ancestry-associated DEGs showed significant enrichment for brain and non-brain immune cells (Fisher’s exact test, FDR < 0.05; Supplementary Figs. 11b and 13), potentially due to the larger number of detected DEGs. Even so, we found that the level of enrichment of non-brain immune cells (global and local) on average was smaller than brain immune cells. We consistently found significant depletion of DEGs (global and local) for neuronal cell types. Moreover, we observed immune-related pathways and associated cell types (that is, microglia and perivascular macrophages) for DEGs upregulated with increasing AA proportion in the caudate nucleus and upregulated with increasing EA proportion in the dentate gyrus, DLPFC and hippocampus. Although we found some differences in glial cell subtypes^25^ (analysis of variance (ANOVA), FDR < 0.05; Supplementary Fig. 14) using publicly available single-cell data from brain regions with similar compositions^26^, no specific glial subtype^25^ showed directionality of the ancestry effect (Supplementary Fig. 12). Altogether, these results suggest that ancestry-associated DEGs in the human brain are strongly associated with a brain-specific immune response, with the direction of effects varying according to brain region.

### Sharing of ancestry-associated DEGs across brain regions

To understand the regional specificity of global ancestry-associated differentially expressed features, we compared DEGs from each brain region and observed extensive sharing across regions. Specifically, we observed 1,210 DEGs (47.1%) shared between at least two brain regions, where all pairwise overlaps demonstrated significant enrichment (Fisher’s exact test, *P* < 0.01; Fig. 2c). Moreover, 478 DEGs (18.6%) were shared among at least three brain regions, with 112 (4.4%) of these DEGs (Monte Carlo simulation, *P* < 1 × 10^−5^) shared across all four brain regions.

Interestingly, 27 of the 112 shared DEGs (24%) showed a discordant direction of effect in at least one of the four brain regions. This correlated well with the pairwise correlation of shared DEGs that shared the direction of effect (70% to 82%; Fig. 2d). However, this proportion of sharing dropped substantially when effect size was considered (0.22–0.44; Fig. 2d). Correspondingly, we found a large number of brain region-specific DEGs (1,360 (52.9%); Fig. 2e), which increased with isoform-level analysis (transcript (63.6%), exon (67.6%) and junction (69.7%)). This aligns with other studies showing isoform-level brain region specificity^27^.

### Limited role of major histocompatibility complex region and immune cells in ancestry differential expression

Given the primary enrichment signal for immune-related pathways and cell types, we next investigated if immune variation was driving the observed transcriptional changes. Initially, we examined the enrichment of ancestry-associated DEGs for the major histocompatibility complex (MHC) region. We found global ancestry-associated DEGs of the caudate nucleus, DLPFC and hippocampus enriched for human leukocyte antigen (HLA) class II, while the dentate gyrus was enriched for Zinc-finger proteins associated with the extended MHC class I region (Fisher’s exact test, FDR < 0.05; Supplementary Fig. 15). While we found limited enrichment of local ancestry-associated DEGs for gene clusters of the MHC region across brain regions, we still observed significant enrichment of HLA class II genes for the caudate nucleus similar to global ancestry DEGs (Fisher’s exact test, FDR < 0.05; Supplementary Fig. 16).

Next, we reexamined functional enrichment of ancestry-associated DEGs after removing the MHC region (that is, HLA-specific genes, MHC region and extended MHC region) to determine if the MHC region drove enrichment of immune-related pathways. After excluding the HLA genes, we still observed strong enrichment of immune-related pathways (Supplementary Fig. 17). Similarly, excluding the MHC (Supplementary Fig. 18) and the extended MHC region (Supplementary Fig. 19) also showed immune-related enrichment across brain regions. This pattern held for local ancestry DEGs (Supplementary Fig. 20), suggesting that the extended MHC region does not drive ancestry-associated DEG enrichment of immune-related pathways.

Although the MHC region did not appear to drive our immune enrichment, immune variation, either from HLA gene diversity or glial cell composition, could still contribute to our observed transcriptional changes. We next assessed the contributions of HLA variation or glial cell composition to these expression changes. Adding glial cell composition (astrocytes, microglia, macrophages, oligodendrocytes, oligodendrocyte progenitor cells (OPCs) and T cells) as covariates in our differential expression model showed a minimal effect, as evidenced by a high degree of correlation of effect sizes with the original model (Spearman rho from 0.81 to 0.92; Supplementary Fig. 21a). For HLA variation, we added the first five principal components of imputed HLA alleles (explaining 66% of the variance) as covariates, which similarly showed minimal change in effect sizes (Spearman rho from 0.83 to 0.87; Supplementary Fig. 21b). These sensitivity analyses collectively suggest that immune variation contributes only minimally to transcriptional changes for global ancestry-associated DEGs.

### Ancestry-associated DEGs are evolutionarily less constrained

**Fig. 3:**
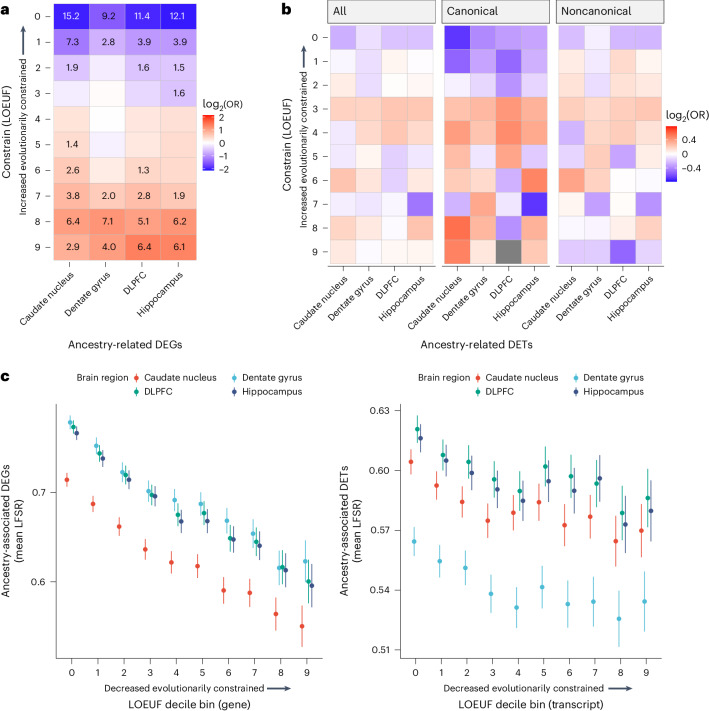
Ancestry-associated genes and canonical transcripts are evolutionarily less constrained. **a**, Significant depletion of ancestry DEGs for evolutionarily constrained genes (canonical transcripts) across brain regions. Significant depletion and enrichments (two-sided Fisher’s exact test, FDR-corrected *P*, −log_10_-transformed) are annotated within the tiles. Odds ratios (ORs) were log_2_-transformed to highlight depletion (blue) and enrichment (red). **b**, A similar trend of depletion of ancestry DETs (all, canonical and noncanonical) for evolutionarily constrained transcripts across brain regions. ORs were log_2_-transformed to highlight depletion (blue) and enrichment (red). **c**, The mean of ancestry-associated differential expression (that is, genes and transcripts) LFSR as a function of loss-of-function observed/expected upper bound fraction (LOEUF). The decile shows a significant negative correlation for genes (left; caudate nucleus (*n* = 122), dentate gyrus (*n* = 47), DLPFC (*n* = 123) and hippocampus (*n* = 133): two-sided Pearson correlation, *r* = −0.20, −0.20, −0.21 and −0.21; *P* = 3.0 × 10^−122^, 7.6 × 10^−113^, 8.6 × 10^−126^ and 1.2 × 10^−122^) and transcripts (right; caudate nucleus (*n* = 122), dentate gyrus (*n* = 47), DLPFC (*n* = 123) and hippocampus (*n* = 133): two-sided Pearson correlation, *r* = −0.05, −0.05, −0.04 and −0.04; *P* = 8.6 × 10^−13^, 1.7 × 10^–11^, 9.0 × 10^−11^ and 3.2 × 10^−^^10^). The error bars correspond to the 95% confidence intervals.

With consistent significant enrichment of DEGs and coexpression modules for the immune response, we hypothesized that these DEGs, with uniquely adaptable cellular biology, would be more likely tolerant of phenotypic consequences of gene disruption and thus be evolutionarily less constrained. To test this, we examined the gene and transcript constraint scores^28^ of the global ancestry-associated DEGs. We found a significant depletion of DEGs for highly constrained genes (Fisher’s exact test, FDR < 0.0001; Fig. 3a). At the transcript level, we found a similar trend (Fig. 3b) with differentially expressed transcripts (DETs) associated with less constrained genes. Furthermore, we observed a significant negative correlation with the DEG signal (LFSR), and gene and transcript constraint scores (Pearson correlation, *P* < 0.0001; Fig. 3c). These results suggest that ancestry-associated differentially expressed features are associated with more rapidly evolving genes as previously seen in immunity-related genes^29,30^.

### Influence of genetic variants on ancestry differential expression in the brain

**Fig. 4:**
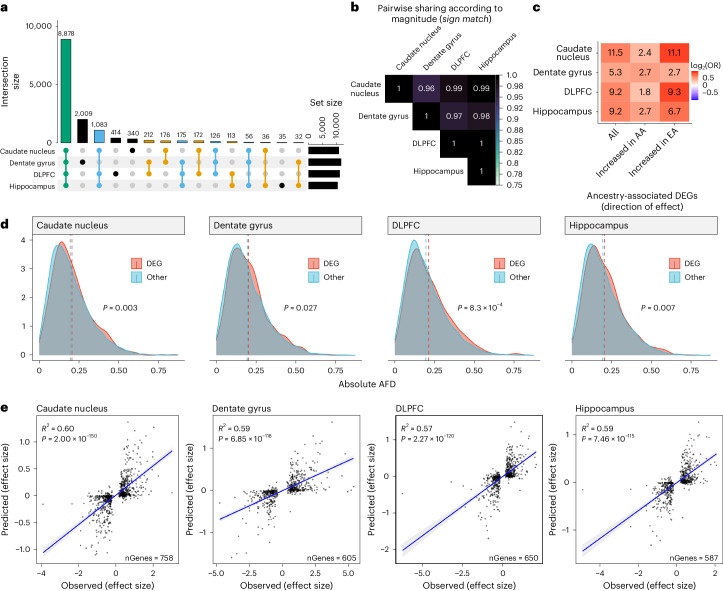
Genetic contribution of genetic ancestry differences in expression across the brain. **a**, UpSet plot showing large overlap of eGenes between brain regions. **b**, Heatmap of the proportion of global ancestry DEG sharing with concordant direction (sign match). **c**, Significant enrichment of ancestry-associated DEGs for eGenes (unique gene associated with an eQTL) across brain regions separated by the direction of effect (increase in AA or EA proportion). **d**, Density plot showing a significant increase in absolute AFDs (one-sided Mann–Whitney *U*-test, *P* < 0.05) for global ancestry-associated DEGs (red) compared with non-DEGs (blue) across brain regions. A dashed line marks the mean absolute AFD. Absolute AFD was calculated as the average absolute AFD across a gene using a significant eQTL (LFSR < 0.05). **e**, Correlation (two-sided Spearman) of elastic net predicted (*y* axis) versus observed (*x* axis) ancestry-associated differences in expression among ancestry-associated DEGs with an eQTL across brain regions. A fitted trend line is shown in blue as the mean value ± s.d. The s.d. is shaded in light gray.

To assess the role of genetic variation in global ancestry-associated DEGs, we initially mapped main effect *cis*-eQTLs in BAs (*n* = 120, 45, 121 and 131 for the caudate nucleus, dentate gyrus, DLPFC and hippocampus, respectively) examining genetic variants within ±500 kb of each feature (gene, transcript, exon and junction). To improve detection, we applied mash and identified at least one *cis*-eQTL for 13,857 genes (‘eGenes’) across brain regions (LFSR < 0.05; *n* = 10,867 for the caudate nucleus; *n* = 11,664 for the dentate gyrus; *n* = 11,173 for the DLPFC; and *n* = 10,408 for the hippocampus; Supplementary Table 3 and Supplementary Data 6). Most of these eGenes (64.1%; Fig. 4a) were shared across all brain regions with only about 0.25–14.5% showing brain region specificity. However, when considering the direction of effect, more than 96% showed sign matching across brain regions (Fig. 4b).

We also examined eQTLs whose effects may vary based on genetic ancestry (interaction between variant and global ancestry proportion), identifying at least one ancestry-dependent *cis*-eQTL for 943 unique genes across brain regions (LFSR < 0.05, *n* = 531, 942, 573 and 531 for the caudate nucleus, dentate gyrus, DLPFC and hippocampus, respectively; Supplementary Fig. 22, Supplementary Table 4 and Supplementary Data 7). Most of these eGenes (510 (54.1%) eGenes) were shared across the four brain regions (Supplementary Fig. 23). This relatively limited detection of ancestry-dependent eQTLs supports other work showing high correlation of causal effects across local ancestry of admixed individuals^31^.

We next tested whether these eGenes (main effect and ancestry-dependent) were likely to be differentially expressed by genetic ancestry. Across brain regions, we found significant enrichment (Fisher’s exact test, FDR < 0.05) of these eGenes (LFSR < 0.05) with ancestry-associated DEGs (LFSR < 0.05; Fig. 4c and Supplementary Fig. 23c). Given the potential correlation of genotypes with eGenes and ancestry inference, we also examined allele frequency differences (AFDs) between DEGs and non-DEGs. We found a significant increase in AFDs for DEGs compared with non-DEGs (Mann–Whitney *U*-test, *P* < 0.05; Fig. 4d and Supplementary Fig. 24) across brain regions. These results suggest that a genetic component is probably influencing these expression differences, potentially because of divergence in allele frequencies.

To test this possibility, we imputed gene expression from genotypes using an elastic net model and examined the correlation between the observed genetic ancestry effect from our ancestry differential expression analysis and the predicted genetic ancestry effect computed from the predicted expression. eGenes showed higher prediction accuracy than non-eGenes, with eGenes exhibiting an ancestry difference in gene expression showing a stronger genetic component (higher *R*^2^) across brain regions (Supplementary Fig. 25). Furthermore, the imputed gene expression explained an average of 59.5%, 58.7%, 56.8% and 56.8% of the variance in genetic ancestry effect sizes across the caudate nucleus, dentate gyrus, DLPFC and hippocampus, respectively (Fig. 4e). This variance was generally increased at the isoform level (transcript *R*^*2*^ = 50.8% ± 7.0%; exon *R*^*2*^ = 61.6% ± 4.1%; and junction *R*^*2*^ = 62.6% ± 5.1%; Supplementary Fig. 26). In contrast, the genetic variant for the top main effect eQTL associated with these genes explained on average approximately 20% of the variance in genetic ancestry effect sizes with a proportion similar to the isoform level (Supplementary Fig. 27). Thus, genetic variants contributed to nearly 60% of the observed genetic ancestry in gene expression; variant effects on alternative splicing were even greater.

### DNA methylation-based contributions to global ancestry differential expression

**Fig. 5:**
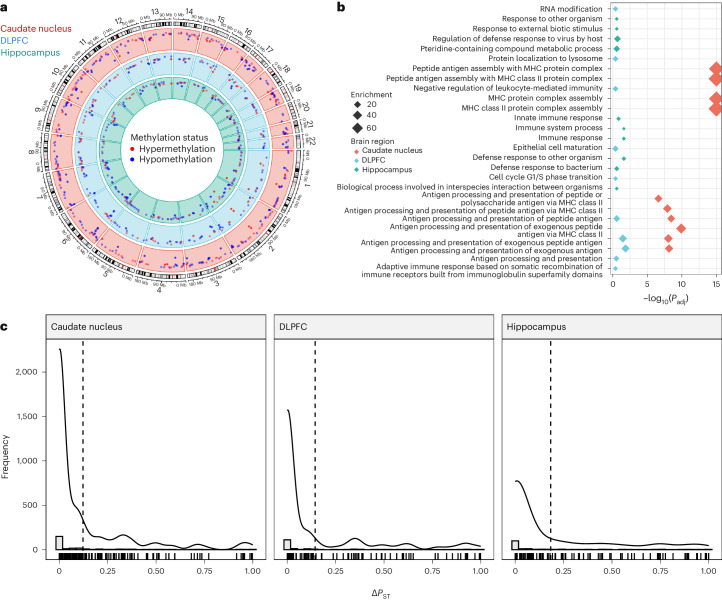
DNA methylation-based contributions to global ancestry-associated differential expression. **a**, Circos plot showing local ancestry-associated DMRs across the caudate nucleus (red), DLPFC (blue) and hippocampus (green). Methylation status is annotated in red for hypermethylation and blue for hypomethylation. **b**, Gene term enrichment (hypergeometric and FDR-corrected) of DMRs across brain regions. **c**, Histograms showing the distribution of Δ*P*_ST_ associated with the impact of unknown environmental factors as captured by residualized VMR (corrected according to local ancestry, age, sex and unknown biological factors captured by principal component analysis (PCA)) for nearby global ancestry-associated DEGs. A dashed line marks the mean Δ*P*_ST_. A solid line shows the density overlay.

To identify DEGs potentially driven by environmental factors, we used DNA methylation as an environmental proxy in BAs. We first identified the top 1% of variable CpGs probably driven by unknown environmental factors. We identified these CpGs by removing variation attributable to technical and biological factors captured by the top five DNA methylation principal components, while preserving variation due to global ancestry. We then grouped these top variable CpGs into variable methylated regions (VMRs) for the caudate nucleus (89 samples, 12,051 VMRs), DLPFC (69 samples, 9,701 VMRs) and hippocampus (69 samples, 9,924 VMRs). In contrast to our differential expression analysis, we found few global ancestry differentially methylated regions (DMRs) (FDR < 0.05; *n* = 3, 1 and 8 for the caudate nucleus, DLPFC and hippocampus, respectively). However, we identified a larger number of local ancestry-associated DMRs (FDR < 0.05; *n* = 494, 260 and 265 for the caudate nucleus, DLPFC and hippocampus, respectively; Fig. 5a).

We reasoned that the difference in DMRs linked to global and local ancestry can be explained both biologically and statistically. Biologically, DNA methylation is more influenced by local genetic variations. Statistically, local ancestry is more variable than global ancestry, which results in a higher power to detect DNA methylation differences and smaller s.d. values in the estimated effect size (Supplementary Fig. 28 and Supplementary Data 8). Even so, we found a significant correlation between local and global ancestry-associated DMRs (Supplementary Fig. 29). Functional enrichment analysis of local ancestry-associated DMRs showed significant enrichment for immune functions across brain regions (hypergeometric, FDR < 0.05; Fig. 5b and Supplementary Data 9), consistent with ancestry-associated DEGs.

We next regressed out known biological factors (local ancestry, age, sex), potential batch effects and other unknown biological factors (top five principal components of DNA methylation) for each VMR. We used *P*_ST_ estimates^18^ to provide a measure of proportion of overall gene expression variance explained by between-population differences. *P*_ST_ values ranged from 0 to 1, where values close to 1 imply that the majority of expression variance is due to differences between populations. We defined Δ*P*_ST_ as the difference between *P*_ST_ values before and after regressing out the effect of VMRs associated with each gene, quantifying the proportion of ancestry-associated DEGs probably due to environmental exposure. Across brain regions, we found that the average Δ*P*_ST_ was 15% (12.2%, 14.4% and 18.3% for the caudate nucleus, DLPFC and hippocampus, respectively, Fig. 5c). Altogether, these results imply that unknown environmental exposure reflected in DNA methylation contributes relatively little to the observed, primarily immune-related expression differences in our BA neurotypical sample.

### Ancestry DEGs are linked with immune-related and brain-related traits

**Fig. 6:**
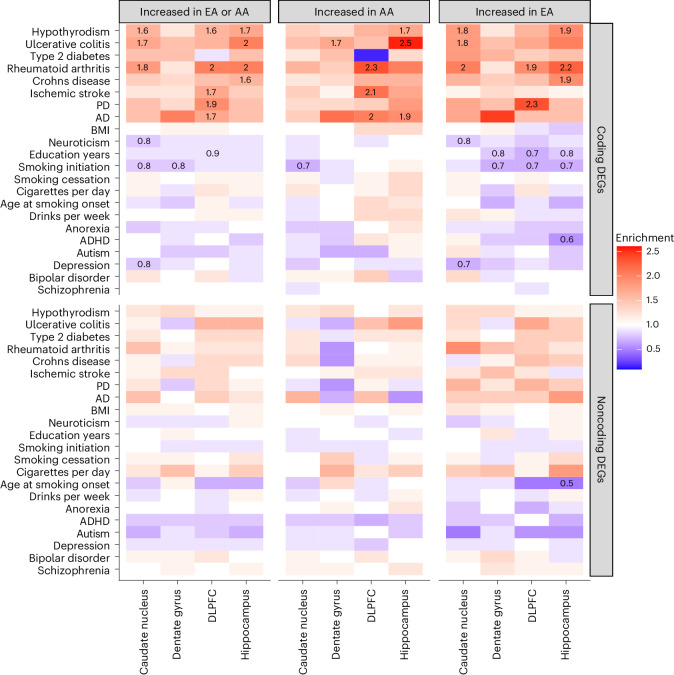
Global ancestry-associated DEGs stratified according to coding or noncoding DEGs show general enrichment for heritability of several neurological and immune-related traits, but depletion for brain-related behavioral traits. Heatmap for ancestry-associated DEGs that show enrichment (red) or depletion (blue) for heritability of brain-related and immune-related traits from S-LDSC analysis. Significant enrichment for heritability traits disappears when limited to noncoding DEGs. Numbers within the tiles are the levels of enrichment (>1) or depletion (<1) that are significant after multiple testing correction (FDR < 0.05). Left, Results for all DEGs in each brain region. Middle and right, Results for DEGs increased with AA or EA proportions for each brain region, respectively.

We reasoned that ancestry-associated DEGs may contain risk genes that explain susceptibility to brain-related illnesses based on ancestry. To explore this hypothesis, we conducted stratified linkage disequilibrium (LD) score (S-LDSC^32^) regression to assess the polygenic contributions of global ancestry-associated DEGs to 17 brain-related traits (for example, attention-deficit/hyperactivity disorder (ADHD), autism, body mass index BMI), depression and schizophrenia) and five immune-related traits as a positive control. Overall, we observed enrichment for heritability of neurological disorders and immune-related traits but not for psychiatric disorders and behavioral traits (Fig. 6, Supplementary Fig. 30 and Supplementary Data 10). This also included limited enrichment of peripheral immune function^33–35^ (Fisher’s exact test, FDR < 0.05; Supplementary Fig. 31), which is consistent with our previous finding of a stronger association with brain immune cell types compared to non-brain immune cell types (Supplementary Fig. 12).

Specifically, we found enrichment for heritability of ischemic stroke (enrichment fold = 1.5, FDR = 0.009) for ancestry-associated DEGs in the DLPFC, accounting for 26% of total heritability (Supplementary Fig. 30). This enrichment was mainly driven by protein-coding DEGs associated with an increase in AA proportion (DEGs: enrichment fold = 1.7, FDR = 0.013; protein-coding: enrichment fold = 2.1, FDR = 0.011), but not in EA (all DEGs: enrichment fold = 1.2, *P* = 0.2). Moreover, our cell type enrichment analysis showed that the DEGs associated with increased AA proportion were enriched for vascular smooth muscle cells, endothelial cells and pericytes (Supplementary Fig. 10), all of which may contribute to the vascular pathology implicated in stroke.

We also found enrichment for heritability of Parkinson disease (PD) (enrichment fold = 1.6, FDR = 0.025) in the DLPFC, accounting for 27% of disease heritability (Supplementary Fig. 30). This enrichment, however, was primarily driven by DEGs that were increased with EA proportion (DEG: enrichment fold = 1.9, FDR = 0.032; protein-coding: enrichment fold = 2.3, FDR = 0.038; Fig. 6), but not AA proportion (enrichment fold = 1.3, *P* = 0.23). Cell type enrichment analysis showed a similar pattern of enrichment for microglia, astrocytes and OPCs (Supplementary Fig. 10). Interestingly, we also found ancestry-associated glial cell subtypes (that is, astrocyte (AST7) and oligodendrocyte (OPC1) lineage) significantly enriched for PD heritability (enrichment fold > 2.0, FDR < 0.01; Supplementary Fig. 32), suggesting a potential role for specific glial subtypes in the pathogenesis of PD.

Furthermore, we observed enrichment for heritability of Alzheimer’s disease (AD) for ancestry-associated DEGs across the DLPFC, hippocampus and caudate nucleus accounting for 26%, 23% and 30% of total heritability, respectively (Supplementary Fig. 30). These enrichments were mainly driven by protein-coding DEGs associated with an increase in AA proportion for the DLPFC (enrichment fold = 2.0, FDR = 0.013; Fig. 6) and hippocampus (enrichment fold = 1.9, FDR = 0.02; Fig. 6). We found the opposite effect with an increase in EA proportion for the caudate nucleus when considering all DEGs (Supplementary Fig. 30), which disappeared when we considered only protein-coding DEGs (Fig. 6). Cell type enrichment analysis of astrocytes, however, showed ancestry-specific effects consistent with our finding for the caudate nucleus (increased EA proportion; Supplementary Fig. 10). Moreover, we found ancestry-associated glial cell subtypes (that is, microglia (MG0) and astrocytes (AST1 and AST7)) significantly enriched for AD heritability (enrichment fold > 2.2, FDR < 0.01; Supplementary Fig. 32) and ancestry-associated DEG enrichment for multiple activated microglia states^36^ (Supplementary Fig. 33a). These microglia states were associated with mouse AD-associated microglial genes and AD GWAS signals (Supplementary Fig. 33b), as well as late-response AD-related genes (Supplementary Fig. 34).

In marked contrast, we observed significant depletion in heritability for several brain-related traits (for example, education years, smoking initiation, age at smoking onset, schizophrenia and depression; enrichment fold < 1, FDR < 0.05; Fig. 6, Supplementary Fig. 30 and Supplementary Data 10). This depletion aligned with our observations that ancestry-associated DEGs were less associated with the neuronal functions implicated in psychiatric disorders and behavioral traits.

## Discussion

We have provided a detailed characterization of how genetic ancestry influences gene expression and DNA methylation in the human brain. Using admixed BA donors, we identified thousands of genomic features associated with global genetic ancestry, revealing their evolutionary adaptability. Approximately 60% of these ancestry-associated DEGs are associated with genetic variations. Our findings consistently highlight enrichment for immune response pathways and absence of neuronal functions. We also found similar trends with local genetic ancestry. Given that expression heritability is dominated (about 70%) by many small *trans* effects^37,38^, we focused primarily on global genetic ancestry.

Interestingly, the enrichment direction for immune-related pathways varied according to brain region, increasing with AA proportion in the caudate nucleus and with EA proportion in the other brain regions. Therefore, there is no simple ‘up or down’ bias in functional associations across brain regions. For example, if AA proportion is a risk factor for immune response in the caudate nucleus, then by the same reasoning AA proportion would be a protecting factor for immune response in the hippocampus and DLPFC. We considered that differences in directionality across regions may reflect variation in cell composition because the caudate nucleus was the only brain region without a laminar architecture. However, laminar architecture in the brain has generally implicated neuronal biology^39^, which was not the case in this study (that is, enrichment of immune-related pathways).

Notably, we found a striking enrichment of heritability for neurological disorders among ancestry-associated DEGs. For instance, small-vessel and ischemic stroke are 50% more frequent in BAs, and Black men are up to 70% more likely to die from stroke compared to non-Hispanic white men^40,41^. In this study, we showed heritability for ischemic stroke driven by ancestry-associated DEGs with an increased AA proportion in the DLPFC. Similarly, we observed a nearly twofold enrichment for AD heritability also increased with AA proportion in the DLPFC and hippocampus. This observation echoes the fact that AD is twice as prevalent in BAs^42,43^. However, general enrichment of DEGs for AD in the caudate nucleus associated with an increase in EA proportion highlights the potential regional complexity of the disorder in the brain as the caudate nucleus is not generally considered a site of AD pathology. Conversely, heritability of PD—more prevalent in non-Hispanic WAs^44^—showed enrichment among DEGs with an increase in EA proportion. Ancestral DEGs enriched heritability for several immune disorders and traits but not specifically with either ancestry across the brain. It is noteworthy that the DEGs are not linked with heritability of psychiatric disorders and related behavioral traits, perhaps consistent with genes associated with these traits being especially enriched in neurons, which were again conspicuously lacking in DEGs based on ancestry.

To highlight VMRs enriched for environmental influence, we focused on the top 1% VMRs and looked for ancestry-associated DMRs within these genomic regions. Consistent with the differential expression analysis, we found local ancestry DMRs enriched for genomic regions linked to immune functions. Using VMRs as an environmental proxy to examine the effect of environmental exposures on DEGs, we found that they explained, on average, roughly 15% of population differences in gene expression. Although we used local ancestry to adjust for genetic background, we cannot confirm that methylation variation is solely attributed to environmental factors nor can we ensure that methylation captures all environmental factors. A limitation of this study is the lack of social determinants of health information, which could have directly measured specific environmental exposures instead of using DNA methylation as a proxy. Nevertheless, our analyses demonstrate the potential to limit the impact of systematic environmental factors by leveraging admixture populations for genetic ancestry analyses.

Immune-related pathway enrichment is not unexpected: a previous study showed population differences in macrophages associated with the innate immune response to infection^18^. Furthermore, genetic variation is well documented as an important contributor to immune variation^45–47^ and immune cell function^33–35^. This research is particularly relevant for neuropsychiatric disorders (including schizophrenia, autism spectrum disorder and AD) where the immune system has been implicated^48–50^. Many of these neuropsychiatric disorders also show a racial health disparity^42,51–53^. Our detailed investigation of immune function found little evidence that the MHC region, HLA variation or glial cell composition drove immune response pathway enrichment. Additionally, we found stronger enrichment of brain immune compared with peripheral immune cell types, suggesting a potential involvement of brain-specific immune responses in these DEGs. Altogether, our findings lay the groundwork for further investigation of therapeutic interventions involving the immune response—therapeutic interventions that could address these health disparities.

In summary, we have provided a detailed examination of the genetic and environmental contributions to genetic ancestry transcriptional changes in the brain. We leveraged genetic diversity within an admixed population to limit environmental confounders, resulting in converging evidence of the immune response in genetic ancestry-associated transcriptional changes in the brain. The research we have provided substantively furthers our understanding of the contribution of genetic ancestry in the brain, opening new avenues to the development of ancestry-aware therapeutics and paving the way for equitable, personalized medicine.

## Methods

The research described in this article complies with all relevant ethical regulations. Additionally, all specimens used in this study were obtained using oral informed consent. We obtained informed consent from the next of kin under protocol nos. 12–24 (Department of Health and Mental Hygiene for the Office of the Chief Medical Examiner for the State of Maryland) and no. 20111080 (Western Institutional Review Board for the Offices of the Chief Medical Examiner for Kalamazoo Michigan, University of North Dakota in Grand Forks North Dakota and Santa Clara County California). We obtained samples from the Clinical Brain Disorder Branch at the National Institute of Mental Health (NIMH) from the Northern Virginia and District of Columbia Medical Examiners’ Office, according to National Institutes of Health institutional review board guidelines (protocol no. 90-M-0142). The LIBD received the tissues by donation under the terms of a material transfer agreement. The institutional review board of the University of Maryland and the State of Maryland approved the study protocols that collected these brain tissues^10–12^. Details of case selection, curation, diagnosis, anatomical localization and dissection can be found in previous publications from our research group^10–12^.

### BrainSeq consortium RNA-seq data processing

We surveyed covariates, FASTQ files, SNP array genotypes, RNA degradation metrics obtained with the qSVA methodology^20^, phenotype information and raw counts (gene, transcript, exon and exon–exon junction) for the caudate nucleus, dentate gyrus, DLPFC and hippocampus from the BrainSeq Consortium^10,12^ and research.libd.org/dg_hippo_paper/data.html (ref. ^11^).

### BrainSeq consortium genotype imputation

#### General imputation

Samples were genotyped and imputed as part of the full LIBD cohort, using procedures described previously^10,12,13^. Briefly, samples were genotyped on four different types of Illumina microarrays over the years (HumanHap650, Human1M, HumanOmni2.5 or HumanOmni5-Quad BeadChips). We merged samples genotyped by the same type of microarray and followed standard preimputation quality control (QC) to remove low-quality (Hardy–Weinberg equilibrium *P* < 1 × 10^−^^6^) and low-frequency (minor allele frequency (MAF) < 0.005) variants. We converted genotype positions from hg19 to hg38 with LiftOver^54^. Once converted, we imputed genotypes, separately according to genotyping array, on the TOPMed imputation server^8,55,56^ using the Haplotype Reference Consortium reference panels. We phased genotypes per chromosome using eagle (v.2.4)^57^. We performed post-imputation QC of each imputed dataset for Black and non-Hispanic WA samples separately.

We filtered out variants with low-quality imputation scores (*R*^*2*^ < 0.8) and removed variants with (1) MAF < 0.05, (2) missing call frequencies > 0.1 or (3) Hardy–Weinberg equilibrium *P* *<* 1 × 10^−10^ using PLINK2 (v.2.00a3LM)^58^. We then merged the imputed genotypes across four genotyping platforms based on overlapping filtered imputed variants. This resulted in 6,225,756 and 6,097,532 common variants for Black and non-Hispanic WA donors, respectively.

#### HLA imputation

For HLA allele imputation, we extracted the extended MHC region on chromosome 6 from preimputed quality checked genotypes (hg38) according to genotype array (see ‘General imputation’) with PLINK2. We performed HLA imputation on the Michigan Imputation Server^55^ using the four-digit, multiethnic HLA imputation reference panel^59^ (v.2). Like general imputation, we phased genotypes using eagle on the server. After imputation, we filtered low-quality imputation scores (*R*^*2*^ < 0.7) per genotype array with BCFtools (v.1.13)^60^. We then merged the imputed genotypes across the four genotyping arrays with BCFtools and extracted HLA alleles from the VCF file. This resulted in a total of 2,850 HLA alleles.

### BrainSeq consortium DNA methylation data processing

We generated WGBS datasets in our previous studies for three adult brain regions (DLPFC, hippocampus and caudate nucleus). Details about study samples, data generation and data processing have been described in our previous reports^14,61^. Briefly, we assessed QC with FastQC. After an assessment with FastQC, we removed adapter content with TrimGalore^62^. We aligned trimmed reads with Arioc^63^ to the hg38 genome build (GRCh38.p12) and removed duplicate alignments with SAMBLASTER^64^. After removing duplicates, we filtered alignments with SAMtools^65^ (v.1.9) to include only primary alignments with a mapping quality ≥ 5. From these filtered alignments, we extracted methylation data using the Bismark methylation extractor^66^. After methylation extraction, we processed and combined DNA methylation proportions across samples using bsseq (v.1.18)^67^, an R/Bioconductor package. We locally smoothed methylation data with BSmooth using default parameters. We filtered the resulting CpG data to remove (1) CpGs within the blacklist regions and (2) CpGs with coverage < 3.

### Sample selection and details

We selected samples per brain region using five common inclusion criteria: (1) RiboZero RNA-seq library preparation; (2) recent African ancestry; (3) TOPMed-imputed genotypes available; (4) adults (aged > 17 years); and (5) diagnosis of neurotypical control. This resulted in a total of 425 samples from 151 unique individuals across the caudate nucleus (*n* = 121, 50 female and 72 male), dentate gyrus (*n* = 47, 16 female and 32 male), DLPFC (*n* = 123, 48 female and 75 male) and hippocampus (*n* = 133, 53 female and 80 male). Participant details including age, sex and RNA integrity number are summarized in Table 1. Individual-level details are provided in Supplementary Data 11.

### Estimation of genome-wide admixture levels

We estimated the admixture proportion for each individual based on SNPs that were informative for ancestry using the STRUCTURE program (v.2.3.4)^17^. We selected 1,634 such SNPs based on genetic information downloaded from the 1000 Genomes CEU (Northern Europeans from Utah) and AFR (African ancestry superpopulation, including Esan, Gambian, Luhyu, Mende and Yoruba populations) samples. Markers were chosen based on the following criteria: (1) absolute difference (*δ*) in allele frequency between the two ancestry populations > 0.5; (2) *r*^2^ between each pair of SNPs < 0.1 within each population; (3) *P* > 0.01 to test the Hardy–Weinberg equilibrium within each population; and (4) successfully imputed in our brain samples (info > 0.8). The structure was run within a two-ancestry population model with 5,000 burn-in and 10,000 iterations.

### Estimation of local ancestry

We used RFMix (v.2.03-r0)^68^, a discriminative modeling approach for rapid and robust local-ancestry inferences, to infer local ancestry in our admixed samples using the European and African ancestry samples from the 1000 Genomes Project^69^ as reference. We extracted the posterior probability of African ancestry at each SNP per haplotype from the forward–backward output of RFMix. Local ancestry for a genomic region was then estimated as the average African ancestry across all SNPs within the region. As RFMix also computed and output a global ancestry estimate for each sample, we compared global ancestry estimates between STRUCTURE and RFMix and observed a high correlation between estimates from the two programs (Spearman rho = 0.99).

### Differential expression analysis

#### Cell type deconvolution analysis

Deconvolution was performed with the ReferenceBasedDecomposition function of the R package BisqueRNA (v.1.0.4)^70^, using the use.overlap = FALSE option. The single-cell reference dataset used was the single-nucleus RNA-seq from the 10X protocol, which includes tissue from eight donors and five brain regions^26^. The ten cell types considered in the deconvolution of the tissue were astrocytes, endothelial cells, microglia, macrophages, mural cells, oligodendrocytes, OPCs, T cells, excitatory neurons and inhibitory neurons. Marker genes were selected by first filtering for genes common between the bulk and reference data and then calculating the ratio of the mean expression of each gene in the target cell type over the highest mean expression of that gene in a nontarget cell type. The 25 genes with the highest ratios for each cell type were selected as markers.

#### QC and identification of relevant confounders

To evaluate potential sources of confounding for expression and genetic ancestry, we first correlated the technical and RNA quality variables available from the downloaded R variables and removed highly correlated variables (Pearson *r* > 0.95) present in two or more brain regions. After this, we retained variables common across the four brain regions. In addition to these variables, we also accounted for hidden variables using the downloaded qSVA (Supplementary Fig. 35 and equation (1), *k* = 13, 6, 9 and 14, for the caudate nucleus, dentate gyrus, DLPFC and hippocampus, respectively). We found that qSVs were also accurately correct for observed variables like batch effect and cell type composition^12,20^:

1 $$\begin{array}{l}E\left(Y\right)={\beta }_{0}+{\beta }_{1}{\mathrm{ancestry}}+{\beta }_{2}{\mathrm{sex}}+{\beta }_{3}{\mathrm{age}}+{\beta }_{4}{{\mathrm{mito}}\,{\mathrm{rate}}}+{\beta }_{5}{{\mathrm{rRNA}}\,{\mathrm{rate}}}\\\qquad+\,{\beta }_{6}{{\mathrm{total}}\,{\mathrm{assigned}}\,{\mathrm{genes}}}+{\beta }_{7}{{\mathrm{overall}}\,{\mathrm{mapping}}\,{\mathrm{rate}}}+\mathop{\sum }\limits_{i=1}^{k}{\gamma }_{i}{qS}{V}_{i}\end{array}$$

Given the potential influence of cell composition on gene expression, we also examined the cell type proportion associated with genetic ancestry and any potential confounding effects on gene expression. To this end, we performed cell type deconvolution (Supplementary Data 12). When we examined the BA population, we found that most cell types across brain regions showed no correlation with genetic ancestry (Supplementary Fig. 36); only oligodendrocytes in the DLPFC showed a significant association (Spearman *P* < 0.05) with genetic ancestry. In contrast, when we included non-Hispanic WA donors, we found that seven of the ten cell types showed a significant association (Spearman *P* < 0.05) with genetic ancestry in at least one brain region (Supplementary Fig. 37). These cell type proportions also showed high correlation with confounders (Supplementary Fig. 38). As such, our model also accounted for cell type proportions for each brain region (Supplementary Fig. 39).

#### Global ancestry-associated differential expression analysis

We performed differential expression analysis using mash modeling in R. Initially, we determined the effect size and the s.e. of the effect size using limma-voom modeling as described previously^12^. Briefly, we filtered low-expressing genes using filterByExpr from edgeR (v.3.40.2)^71,72^ and normalized library size. Next, we applied voom normalization^73^ as a model of genetic ancestry adjusted for age and RNA quality (mitochondria mapping, gene assignment, genome mapping and rRNA mapping rates, and hidden variance using qSVA; equation (1)). After voom normalization, we fitted the model using eBayes and extracted out the effect size (log fold change) and s.e. of the effect size from the model (equation (2)) by brain region for each feature (gene, transcript, exon and junction):

2 $${\mathrm{S.E.}}=\frac{{\rm{\sigma }}}{\sqrt{n}}$$

Next, we implemented mash modeling using mashr (v.0.2.57)^21^ for each feature using the limma-voom-extracted effect sizes and s.e. across brain regions. We learned the correlation structure across the brain regions and used all features as an unbiased representation of the results to account for overlapping samples. After this, we calculated the canonical covariances. A strong set of features was determined condition by condition using mash_1by1; data-driven covariance was calculated with the strong set of features. Once calculated, we fitted the mash model to the full set of features and computed the posterior summaries for all features. Features were considered significant if they had an LFSR < 0.05.

#### Local ancestry-associated differential expression analysis

For local ancestry differential expression analysis, we first calculated a local African ancestry score per feature (that is, gene, transcript, exon and junction). Then, we averaged all haplotypes within a 200-kbp window of each feature using the RFMix results. Following this estimate of local African ancestry per feature, we applied a separate linear model per feature using equation (1) modified for local ancestry. We limited our analysis to features tested for global ancestry differential expression. As each model was per feature, we replaced voom-normalized with counts per million log-normalized counts. We fitted our model with limma (v.3.46.0; R v.4.2) lmFit and extracted the effect size and s.e. for downstream mash modeling as described in ‘Global ancestry-associated differential expression analysis’. We compared the local and global ancestry differential expression results and found a large overlap (Supplementary Fig. 40).

#### Expression residualization

For residualized expression, we regressed out covariates from voom-normalized expression using a null model (equation (3)) and applied *z*-score normalization as described previously^12^:

3 $$\begin{array}{l}E\left(Y\right)={\beta }_{0}+{\beta }_{1}{\mathrm{sex}}+{\beta }_{2}{\mathrm{age}}+{\beta }_{3}{{\mathrm{mito}}\,{\mathrm{rate}}}+{\beta }_{4}{{\mathrm{rRNA}}\,{\mathrm{rate}}}\\\qquad+\,{\beta }_{5}{{\mathrm{total}}\,{\mathrm{assigned}}\,{\mathrm{genes}}}+{\beta }_{6}{{\mathrm{overall}}\,{\mathrm{mapping}}\,{\mathrm{rate}}}+\mathop{\sum }\limits_{i=1}^{k}{\gamma }_{i}{qS}{V}_{i}\end{array}$$

#### MHC region enrichment

To examine the contribution of the MHC region to immune-related pathway enrichment, we extracted genes within the MHC from the hg38 annotation (GENCODE v.25). Specifically, we extracted genes from the MHC region (chromosome 6: 28510120–33480577) and the extended MHC region (chromosome 6: 25726063–33400644) using PyRanges (v.0.0.127)^74^ and gtfparse (v.2.0.1). We further subset the extended MHC region for any gene names that started with HLA. After this, we assessed enrichment for the MHC regions (that is, the MHC region, the extended MHC region and the HLA genes) using a two-sided Fisher’s exact test. We corrected for multiple testing with the Benjamini–Hochberg method.

#### Public data comparison and enrichment analysis

For public data comparison, we downloaded the ancestry-associated DEGs in immune cells^18^ and immune function GWAS prioritized genes^33–35^. We assessed enrichment with our ancestry-associated DEGs using a two-sided Fisher’s exact test and corrected for multiple testing with the Benjamini–Hochberg method.

#### Single-cell specificity and cell type enrichment analysis

To understand the cellular context of ancestry-associated DEGs in the human brain, we performed cell type enrichment analysis by leveraging existing gene expression data from 39 broad categories of cell types from the mouse central and peripheral nervous system^23^. Specifically, we examined the overlap between DEGs and cell-type-specific genes for each cell type defined in a previous study^75^. We assessed enrichment for each brain cell type using a two-sided Fisher’s exact test. We corrected for multiple testing with Benjamini–Hochberg method.

We next expanded our cell type enrichment analysis to single-cell datasets with glial (that is, astrocyte, microglia and oligodendrocyte) subtype annotation and non-brain immune cells (that is, peripheral blood mononuclear cells (PBMCs)). For the glial subpopulations, we downloaded human postmortem hippocampus astrocyte, microglia and oligodendrocyte lineage single-cell data^25^ from the UCSC cell browser^76^. For PBMCs, we downloaded human PBMC single-cell data^24^ from *Zenodo* (10.5281/zenodo.4273999).

To calculate cell type specificity, we adapted the cell type specificity code from github.com/jbryois/scRNA_disease/blob/master/Code_Paper/Code_Zeisel/get_Zeisel_Lvl4_input.md (ref. ^75^) for these additional datasets. Briefly, we converted Seurat objects^77^ into SingleCellExperiment (v.1.23.0)^78^ in R (v.4.3). Next, we aggregated mean counts across annotated cell types with scuttle (v.1.11.2 (ref. ^79^); sciwheel.com/work/citation?ids=3436659&pre=&suf=&sa=0). After aggregation, we removed genes with zero expression and applied transcripts per million (TPM) normalization. Across all cell types, we calculated a specificity score for each gene defined as the proportion of total expression of a gene. To assign marker genes based on cell specificity, we filtered out genes with less than one TPM and selected the top 10% of genes based on the specificity score for each cell type. We used these marker genes to assess the enrichment of ancestry-associated DEGs using a two-sided Fisher’s exact test and corrected for multiple testing with the Benjamini–Hochberg method.

For disease single-cell enrichment, we downloaded marker genes and AD differential expression results for each microglial state^36^ from compbio.mit.edu/microglia_states/. For the enrichment analysis, we applied a two-sided Fisher’s exact test using all annotated genes as a universe. We corrected for multiple testing using the Benjamini–Hochberg method.

#### Glial cell composition across multiple brain regions

To investigate glial cell composition across the caudate nucleus, DLPFC and hippocampus, we downloaded single-cell datasets for multiple brain regions^26^ similar to ours (that is, nucleus accumbens, DLPFC and hippocampus). To integrate the single-cell data for three brain regions, we modified the across-region analysis script from github.com/LieberInstitute/10xPilot_snRNAseq-human/blob/master/10x_across-regions-analyses_step02_MNT.R. Specifically, we cleaned the annotated datasets, removing the precalculated metrics. After this, we combined the data and normalized them with multiBatchNorm from the batchelor R package (v.1.17.2)^80^. Next, we subset the dataset specifically for annotated glial cells (that is, the microglia, astrocyte and oligodendrocyte lineage).

To annotate the glia subpopulation to the multiple brain region dataset, we first converted R objects to H5AD files using zellkonverter (v.1.8.0; github.com/theislab/zellkonverter). We integrated the multi-brain region combined dataset^26^ with the glia subpopulation dataset^25^ using single-cell variational inference^81^ from scvi-tools (v.0.20.1)^82^ per glia subpopulation. After integration, we transferred the glia subpopulation annotations to the multi-brain region dataset with single-cell annotation using variational inference (scANVI^83^) from scvi-tools. We visualized the glia subpopulation clustering after removing batch effects from the PCA subspace with fastMNN from the batchelor package and applying *t*-distributed stochastic neighbor embedding using the scater package (v.1.28.0)^79^.

To test differences in glial cell composition across brain regions, we applied the propeller function from the speckle package in R (v.1.1.0)^84^, with arcsin-transformed counts. The propeller function was corrected for multiple testing.

#### Binary contrast of BAs and non-Hispanic WAs

For internal validation of global ancestry-associated differential expression features (that is, gene, transcript, exon and junction), we performed differential expression analysis with a combination of BAs and WAs using mash. As with ‘Global ancestry-associated differential expression analysis’, we determined the effect size and s.e. of the effect size using limma-voom modeling. We replaced the continuous variable genetic ancestry with the binary, self-reported race. Additionally, we selected individuals with limited admixture by including: (1) Black Americans with African genetic ancestry ≥ 0.8; and (2) WAs with European genetic ancestry > 0.99. To limit the influence of the larger sample size compared to ‘Global ancestry-associated differential expression analysis’, we randomly sampled ten times without replacement to approximate the sample size of the admixed BA-only analysis. After extraction of the effect sizes and s.e., we implemented mash modeling for each feature across brain regions as described in the *‘*Global ancestry-associated differential expression analysis’ section.

#### Immune variation modeling

To remove the potential effect of immune variation, we added HLA variation (equation (4)) or glial cell proportion (astrocytes, microglia, macrophages, oligodendrocytes, OPCs and T cells equation (5)) to our differential expression model as covariates. Previously, we found that only the oligodendrocytes in the DLPFC showed a significant association (Spearman *P* < 0.05; Supplementary Fig. 36) with genetic ancestry (see ‘QC and identification of relevant confounders’). Given the potential correlation between HLA variation and global genetic ancestry, we first examined the association of HLA variation with global genetic ancestry. For this, we first generated HLA variation principal components by applying PCA on the 2,850 HLA imputed alleles. We found a limited correlation between the ten principal components and global genetic ancestry (Spearman *P* < 0.05; Supplementary Fig. 41).

4 $$\begin{array}{l}E\left(Y\,\right)={\beta }_{0}+{\beta }_{1}{\mathrm{ancestry}}+{\beta }_{2}{\mathrm{sex}}+{\beta }_{3}{\mathrm{age}}+{\beta }_{4}{{\mathrm{mito}}\,{\mathrm{rate}}}+{\beta }_{5}{{\mathrm{rRNA}}\,{\mathrm{rate}}}\\\qquad +{\beta }_{6}{{\mathrm{total}}\,{\mathrm{assigned}}\,{\mathrm{genes}}}+{\beta }_{7}{{\mathrm{overall}}\,{\mathrm{mapping}}\,{\mathrm{rate}}}\\\qquad+\mathop{\sum }\limits_{i=1}^{k}{\gamma }_{i}{qS}{V}_{i}+\mathop{\sum }\limits_{j=1}^{5}{\sigma }_{j}{\mathrm{HLA}}_{j}\end{array}$$

5 $$\begin{array}{l}E\left(Y\,\right)={\beta }_{0}+{\beta }_{1}{\mathrm{ancestry}}+{\beta }_{2}{\mathrm{sex}}+{\beta }_{3}{\mathrm{age}}+{\beta }_{4}{{\mathrm{mito}}\,{\mathrm{rate}}}+{\beta }_{5}{{\mathrm{rRNA}}\,{\mathrm{rate}}}\\\qquad\quad+{\beta }_{6}{{\mathrm{total}}\,{\mathrm{assigned}}\,{\mathrm{genes}}}+{\beta }_{7}{{\mathrm{overall}}\,{\mathrm{mapping}}\,{\mathrm{rate}}}+\mathop{\sum }\limits_{i=1}^{k}{\gamma }_{i}{qS}{V}_{i}\\\qquad\quad+{\beta }_{8}{\mathrm{astrocyte}}+{\beta }_{9}{\mathrm{macrophage}}+{\beta }_{10}{\mathrm{microglia}}+{\beta }_{11}{{\mathrm{T}}\,{\mathrm{cell}}}\\\qquad\quad+{\beta }_{12}{\mathrm{oligodendrocyte}}+{\beta }_{13}{\mathrm{OPC}}\end{array}$$

### Weighted correlation network analysis

We performed a signed-network WGCNA (v.1.72)^22^ analysis using residualized expression to generate the coexpression network with neurotypical control individuals (*n* = 151 BAs) in a single block according to brain region. For this analysis, we filtered genes and outlier individuals with the WGCNA function goodSamplesGenes. After this, we applied additional sample filtering based on sample expression with a total *z*-normalized distance of 2.5 or greater from other samples. After evaluating power and network connectivity for each brain region, we selected a soft power of 12.

For network construction, we used bicor correlation and the following parameters: (1) mergeCutHeight set to 0.3 for the dentate gyrus and default values for the caudate nucleus, DLPFC and hippocampus; and (2) minModuleSizeset to 30 for the dentate gyrus and default values for the caudate nucleus, DLPFC and hippocampus. We set all other parameters to default values. The coexpression network was made using Pearson correlation values for the caudate nucleus (117 samples; 19,883 genes), dentate gyrus (46 samples; 18,747 genes), DLPFC (121 samples; 20,070 genes) and hippocampus (128 samples; 19,794 genes). We determined significant associations with ancestry using a linear model that correlated ancestry proportions (see ‘Estimation of genome-wide admixture levels’) with module eigengenes.

For each module, we calculated overlap enrichment or depletion with ancestry-associated DEGs (FDR < 0.05) separated by direction of effect (such as DEGs that are upregulated in AA, upregulated in EA or upregulated in either ancestry) using a two-sided Fisher’s exact test in Python with the SciPy^85^ stats module. *P* values were corrected using the statsmodels^86^ stats module with the Benjamini–Hochberg method in Python.

When we examined the most significantly enriched modules for ancestry-associated DEGs upregulated in BAs across brain regions, we found the cyan module (enriched for response to virus) for the caudate nucleus; the pink module (enriched for wound healing and cell migration) for the dentate gyrus; the saddle brown module (enriched for cellular response to viruses) for the DLPFC; and the yellow module (enriched for cilium movement and assembly) for the hippocampus (Supplementary Fig. 7a and Supplementary Data 4). In contrast, when we examined the most significantly enriched modules for ancestry-associated DEGs downregulated in proportion to BAs across brain regions, we found the green yellow module (enriched for inflammatory response) for the caudate nucleus; the saddle brown module (enriched for immune response) for the dentate gyrus; the pink module (enriched for immune response) for the DLPFC; and the blue module (enriched for immune response) for the hippocampus (Supplementary Fig. 7b and Supplementary Data 4). Although the caudate nucleus and DLPFC showed modules enriched for the immune response for both directions of effect, the most significantly enriched non-gray module (two-sided Fisher’s exact test) was associated with a specific direction of effect consistent with differential expression analysis for the caudate nucleus (cyan module, DEGs upregulated in African ancestry) and DLPFC (pink module, DEGs downregulated in African ancestry).

### Gene term enrichment analysis

#### Differential expression analysis: gene term enrichment and hypergeometric analysis

We determined significant enrichment for gene sets using the GSEA^87,88^, which is less susceptible to gene length bias because it uses permutation enrichment within gene sets. In this study, we performed GSEA with the GO gene set database from the clusterProfiler package (v.4.6.2)^89^ and DisGeNET gene set database^90^ from the DOSE package (v.3.24.2)^91^. We defined the gene set ‘universe’ as all unique genes tested for differential expression. When examining isoform-level enrichment (transcript, exon or junction), we selected, for each unique gene, the feature with the largest absolute effect size. For the GO gene set database, the minimal gene set size (minGSSize) was set to ten, the maximum gene set size (maxGSSize) was set to 500, and the *P* cutoff was set to 0.05. For theDisGeNET gene set database, minGSSize was set to five and the *P* cutoff to 0.05. We used the default settings for all other parameters.

For hypergeometric analysis, we used enrichGO and enrichDGN from the clusterProfiler and DOSE packages, respectively. Like the GSEA analysis, we defined the gene set ‘universe’ as all unique genes tested for differential expression.

#### Coexpression network analysis: gene term enrichment

For the gene term enrichment analysis, we used the GOATOOLS Python package (v.1.2.3)^92^, using hypergeometric tests with the GO database. Like ‘Differential expression analysis: gene term enrichment and hypergeometric analysis’, we defined the gene set universe as all unique genes tested from differential expression analysis.

### Enrichment of evolutionary constraint

For the evolutionary constraint enrichment analysis, we downloaded the Genome Aggregation Database (gnomAD) v.2 gene-level and transcript-level loss-of-function (LOF) metrics^28^. We assessed enrichment with the LOEUF using the decile bins. Additionally, we tested the correlation between ancestry-associated differentially expressed features (that is, genes and transcripts) and the LOEUF with a two-sided Pearson correlation. We corrected both statistical tests for multiple testing using the Benjamini–Hochberg method.

### eQTL analysis

We performed all *cis*-eQTL mapping for neurotypical controls (BAs, aged > 17 years; Table 1) using tensorQTL (v.1.0.7), which leverages graphics processing units to substantially increase computational speed^93^. Initially, we filtered low expression as described previously^12^ using the GTEx Python script (that is, eqtl_prepare_expression.py) with modifications for isoform-level genomic features (that is, transcripts, exons and junctions). This script retained features with expression estimates greater than 0.1 TPM in at least 20% of samples and aligned read counts of six or more. Additionally, this script used Python functions defined by rnaseqnorm.py to normalize counts with TMM, a Python port of the edgeR function.

To generate the TPM files as input for eqtl_prepare_expression.py, we used effective length (equation (6)). For genes and exons, we calculated effective length (equation (7)) using mean insert size from the Picard tools CollectInsertSizeMetrics tool (v.2.20.1; broadinstitute.github.io/picard/). For junctions, we fixed the effective length at 100. After calculating the effective length, we dropped any feature with an effective length less than or equal to one:

6 $${\mathrm{TPM}}=1e6\times \frac{{\mathrm{Count}}/{\mathrm{effective}}\,{\mathrm{length}}}{\varSigma\,({\mathrm{count}}/{\mathrm{effective}}\,{\mathrm{length}})}$$

7 $${\mathrm{Effective}}\,{\mathrm{length}}={\mathrm{length}}-({\mathrm{mean}}\,{\mathrm{insert}}\,{\mathrm{size}})+1$$

#### Main effect analysis

For main effect *cis*-eQTL mapping, we quantified the effects of unobserved confounding variables on expression after adjusting for sex, population stratification (SNP principal components 1–5) and *k* unobserved confounding variables on expression. We determined these variables using the num.sv function (vfilter set to 50,000) from sva, an R/Bioconductor package (v.3.34.0)^94^ and PCA of expression for each feature. To identify *cis*-eQTL, we implemented nominal mapping, adjusting for covariates with a mapping window within 0.5 Mb of the transcription start site of each feature and an MAF ≥ 0.01. tensorQTL used a two-sided *t*-test to estimate the nominal *P* value for each variant–gene pair. To generate a subset of ‘strong’ signals for downstream mash modeling in R, we also performed adaptive permutations. After this, empirical *P* values were corrected for multiple testing across features using Storey’s *q* value method^95,96^. This resulted in a file with the top variant for each feature. In addition to this permutation analysis, we also performed conditional analysis. This resulted in additional feature–variant pairs to generate our set of ‘strong’ associations for mash modeling.

#### Ancestry-dependent interaction analysis

For genetic ancestry-dependent *cis*-eQTL mapping, we used the confounders generated from the main effect analysis but removed variables associated with population stratification (SNP principal components 1–5). To identify genetic ancestry-dependent *cis*-eQTL, we implemented nominal mapping, adjusting for covariates with a mapping window within 0.5 Mb of the transcription start site of each feature and an MAF ≥ 0.05. To generate a subset of strong signals for downstream mash modeling, we performed eigenMT^97^ by setting run_eigenmt to True. This resulted in a file with the top variant for each feature.

For plotting, we generated residualized expression for BAs and non-Hispanic WAs for the caudate nucleus (*n* = 233), dentate gyrus (*n* = 85), DLPFC (*n* = 204) and hippocampus (*n* = 236). After the main effect analysis, we generated covariates and normalized expression for this multi-ancestry population. With this, we applied lmFit from limma to normalize expression and covariates, excluding the variable of interest (global ancestry). Subsequently, we applied the residuals function in R (v.4.0.3) to regress out the covariates from the normalized expression.

#### Integration with mash modeling in R

To assess sharing across brain regions and to increase our power to detect main and interacting eQTL effects within admixed BA-only individuals, we used the multivariate adaptive shrinkage framework as described previously^12^. We extracted the effect sizes and s.e. for these effect sizes from the nominal results for either the main or interacting *cis*-eQTL. To specify a correlation structure across brain regions (that is, overlapping sample donors), we used the estimate_null_correlation_simple function before fitting the mash model. The mash model included both the canonical covariance matrices and the data-driven covariance matrices learned from our data.

We defined the data-driven covariance matrices as the top four principal components from the PCA performed on the ‘strong’ signals. For gene-level analysis, we defined a set of ‘strong’ tests running a simple condition-by-condition (mash_1by1) analysis as described in ‘Global ancestry-associated differential expression analysis’. For the isoform-level analysis (that is, transcripts, exons and junctions), we defined a set of ‘strong’ tests using either the results from permutation or the eigenMT analyses. Specifically, for the main effect analysis, the set of ‘strong’ tests was selected if a feature–variant pair was present in at least one brain region within the permutation or conditional analyses. For the interaction analysis, we selected the set of ‘strong’ tests if a feature–variant pair was present in at least one brain region from the eigenMT top associations.

To learn the mixture weights and scaling for the main and interacting effects, we initially fitted the mash model with a random set (that is, unbiased representation of the results) of the nominal eQTL results (that is, 5% for gene–variant pairs and 1% for transcript–variant, exon–variant and junction–variant pairs). We next fitted these mixture weights and scaling to all of the main and interacting eQTL results in chunks. After model fitting, we extracted posterior summaries and measures of significance (that is, the LFSR). We considered main and interacting eQTLs significant if the LFSR < 0.05.

### Absolute AFD

To calculate the absolute AFDs, we first calculated the allele frequency within the 1000 Genome Project AFR (superpopulation) and EUR (superpopulation) reference genome using PLINK per chromosome. Before allele frequency calculation, we filtered SNPs based on an MAF of 0.01 for AFR and 0.005 for EUR. To calculate the differences between the two superpopulations, we matched SNP and reference alleles before calculating AFDs (equation (8)). We assessed absolute AFDs for ancestry-associated DEGs compared with other eGenes using two methods: (1) top SNP per gene; and (2) average SNPs across the gene:

8 $${\mathrm{AFD}}=\left|{\mathrm{AFR}}-{\mathrm{EUR}}\right|$$

### Genetic control of ancestry effects on expression

We estimated the predicted *cis*-genetic population differences in expression by first computing predicted expression from genotype dosage (0, 1 or 2; see below). With these predicted expression values, we performed differential expression for genetic ancestry using a model analogous to equation (1) (see ‘Global ancestry-associated differential expression analysis’) to obtain predicted genetic ancestry effects. We extracted the observed population differences in expression from the effect sizes estimated after applying mash as described in ‘Global ancestry-associated differential expression analysis’.

#### Expression residualization for prediction models

To generate residualized expression for our prediction models, we fitted a linear model with lmFit from limma to normalize expression (see ‘eQTL analysis’) and covariates (see ‘Global ancestry-associated differential expression analysis’; equation (3)). Using this model, we regressed out covariates from normalized expression using the residuals function in R (v.4.0.3).

#### Calculating predicted expression using genetic variants in a linear model

For our linear model, we extracted the posterior effect size of the top genetic variant from the mash model for each feature (gene, transcript, exon and junction). We imputed residualized expression using an individual’s genotype dosage (*j*) and feature (*i*) posterior effect size (equation (9)) using PyTorch (v.1.11.0+cu113)^98^:

9 $${\mathrm{Predicted}}\,{\mathrm{expression}}_{i}={{\mathrm{effect}}\,{\mathrm{size}}\,{({\mathrm{eQTL}})}_{j} \times{\mathrm{genotype}}}_{j}$$

#### Calculating predicted expression using genetic variants in an elastic net model

We selected all genetic variants within ±500 kb of the gene body. We removed variants with missing genotypes and filtered variants based on an MAF threshold of 0.01 and a Hardy–Weinberg equilibrium below a *P* value of 1 × 10^−5^. We used an elastic net model, ideal for relatively smaller sample sizes. For our elastic net model, we fitted a sparse linear regression model using big_spLinReg from the bigstatsr R package (v.1.5.12)^99^. We tuned the alpha parameter using a sequence of 20 alphas (that is, 0.05–1 using a 0.05 step size). Additionally, we used four sets for the cross-model selection and averaging procedure. We averaged feature weights for genetic variants across *k*-folds (five folds for each of the caudate nucleus, DLPFC and hippocampus; and three folds for the dentate gyrus). We imputed residualized expression with these feature weights (*i*) and an individual’s genotype dosage (*j*) (equation (10)). We calculated the correlation coefficient (*r*) using Pearson correlation on the test samples for each *k*-fold:

10 $${\mathrm{Predicted}}\,{\mathrm{expression}}_{i}=\sum_j{\mathrm{variant}}\,{\mathrm{weight}}_{j} \times {\mathrm{genotype}}_{j}$$

### LD score regression

We performed S-LDSC (v.1.0.1)^32^ to evaluate global ancestry-associated DEGs for their enrichment for heritability of complex traits, mainly focusing on 17 brain and five immune-related traits as a positive control. We downloaded GWAS summary statistics of each trait from the sources listed in Supplementary Data 13. Following recommendations from the LDSC resource website (alkesgroup.broadinstitute.org/LDSCORE/), we ran S-LDSC for each list of candidate genes. We used the baseline LD model (v.2.2), which included 97 annotations, to control for the LD between variants with other functional annotations in the genome. To remove other potential confounding factors in our analysis, we also included one annotation of all protein-coding genes.

To capture the regulatory regions of each gene, we defined gene intervals as a region spanning 500 kb upstream of the gene’s start position and 50 kb downstream of its end position. We used HapMap Project Phase 3 SNPs as regression SNPs and 1000 Genomes Project SNPs of EA samples as reference SNPs. We downloaded all SNPs from the LDSC resource website.

We ran S-LDSC for all ancestry-associated DEGs and conducted separate runs for DEGs of protein-coding and noncoding genes. For cell type-specific enrichment, we used glia subpopulation specificity markers generated in *‘*Single-cell specificity and cell type enrichment analysis’.

### Differential methylation and contribution to ancestry differential expression

#### VMR analysis

To identify environmentally driven VMRs, we used only our admixed BA neurotypical individuals (caudate nucleus (*n* = 89), DLPFC (*n* = 69) and hippocampus (*n* = 69)). We considered approximately 24 million CpGs that had sequencing coverage of more than five reads in more than 80% samples of each brain region. We also excluded CpGs within ENCODE ‘blacklist’ regions from the analysis. We selected the top one million variable CpGs to compute principal components based on smoothed DNA methylation levels while removing variation due to the global ancestry of our primary variable of interest. Specifically, we regressed out global ancestry from each variable CpG; the residual DNA methylation was used for PCA. To capture CpGs whose variation of DNA methylation level was potentially driven by unknown environmental factors, we computed the s.d. for residualized DNA methylation levels of each CpG after regressing out the top five principal components to remove variations due to batch effects and biological factors. We then selected the top 1% variable CpGs to call the VMRs for each brain region using the regionFinder3 function of bsseq and VMRs, retaining VMRs with more than five CpGs for further analysis. We estimated the DNA methylation level of each VMR by the total number of reads supporting methylated cytosine divided by the total number of reads supporting either methylated or unmethylated cytosine in the region.

#### Differentially methylated region analysis

For differentially methylated region analysis, we applied a linear model on VMRs (see ‘VMR analysis’) as a function of: (1) global genetic ancestry; (2) local genetic ancestry; (3) sex; (4) age; and (5) top five principal components of DNA methylation derived from the top one million variable CpGs. We corrected both statistical tests for multiple testing using the Benjamini–Hochberg method.

#### Functional enrichment analysis

We associated biological functions to global ancestry-associated DMRs using rGREAT (v.2.0.2)^100^, an R/Bioconductor package. Specifically, we selected significant DMRs (FDR < 0.05) and converted them into a genomic range format with plyranges (v.1.18.0)^101^, an R/Bioconductor package. After this conversion and filtering, we applied the ‘great’ function from rGREAT with the Molecular Signatures Database Canonical Pathway C5 (ref. ^88^) Gene Ontology database with background set to human genome (hg18) autosomal chromosomes. We extracted the enrichment results using the getEnrichmentTable function and plotted region–gene associations with the plotRegionGeneAssociation function from the rGREAT package.

#### Evaluating the environmental impact of global ancestry-associated DEGs

To evaluate the impact of unknown environmental factors on global ancestry-associated DEGs, we first annotated the VMRs using annotate_regions and the basic gene hg38 annotation from the R/Bioconduction package annotatr (v.1.24.0)^102^, after converting to genomic ranges with plyranges. After annotation, we estimated *P*_ST_^18^. *P*_ST_ is essentially the partial coefficient of determination. As such, we estimated the *P*_ST_ statistic for each gene with equation (11). We calculated the *P*_ST_ statistics for ancestry before and after including the residualized VMRs annotated to an ancestry-associated DEG. The residual was derived from the raw DNA methylation levels of each VMR by regressing out known biological factors (local ancestry, age, sex), as well as potential batch effects and other unknown biological factors captured by the top five principal components of DNA methylation levels. After this, we calculated Δ*P*_ST_ to extract the fraction of change associated with the environment (equation (12)):

11 $${R}_{{\mathrm{partial}}}^{2}=\frac{{\mathrm{SSE}}\left({\mathrm{reduced}}\right)-{\mathrm{SSE}}\left({\mathrm{full}}\right)}{{\mathrm{SSE}}\left({\mathrm{reduced}}\right)}$$

12 $$\Delta {P}_{{\mathrm{ST}}}=\frac{{P_{\mathrm{ST}}}-{P_{\mathrm{ST}}}\,_{{\mathrm{VMR}}}}{{P_{\mathrm{ST}}}}$$

### Graphics

We used R to generate all plots (R version 4.1, 4.2 and 4.3). We generated UpSet plots using ComplexHeatmap (v.2.10.0)^103^. To generate the circos plots, we used circlize (v.0.4.15)^104^. We generated enrichment heatmaps, gene term enrichment, error plots, box plots, distribution plots and scatterplots using a combination of ggplot2 (v.3.3.6)^105^ and ggpubr (v.0.4.15)^106^. For the pairwise comparison plots, we used corrplot (v.0.92)^107^. We generated metaplots using the mashr function mash_plot_meta. We generated Venn diagrams with ggvenn (v.0.1.10).

### Reporting summary

Further information on research design is available in the Nature Portfolio Reporting Summary linked to this article.

## Online content

Any methods, additional references, Nature Portfolio reporting summaries, source data, extended data, supplementary information, acknowledgements, peer review information; details of author contributions and competing interests; and statements of data and code availability are available at 10.1038/s41593-024-01636-0.

### Supplementary information

Supplementary Information Supplementary Methods, Figs. 1–45, Tables 1–6 and Data 3–15.

Reporting Summary

Supplementary Data 3 Excel file of Gene Ontology (GO) term enrichment and gene set enrichment analysis (GSEA) for genetic-ancestry (continuous) DEGs across the caudate nucleus, dentate gyrus, DLPFC and hippocampus.

Supplementary Data 4 Excel file of GO term enrichment for genetic ancestry-associated WGCNA modules across brain regions.

Supplementary Data 5 Compressed directory of ancestry-associated DEGs enriched for WGCNA module functional enrichment results (that is, GO term enrichment) for the caudate nucleus, dentate gyrus, DLPFC and hippocampus.

Supplementary Data 7 Compressed text file of genetic ancestry-dependent eQTL results (LFSR < 0.05), variant–feature pairs across the caudate nucleus, dentate gyrus, DLPFC and hippocampus for four features (gene, transcript, exon, junction).

Supplementary Data 8 Compressed directory of PDF of scatter plots comparing DNA methylation association with local and global ancestry for the caudate nucleus, DLPFC and hippocampus. Plots are annotated with the genetic ancestry DMR test results.

Supplementary Data 9 Excel file of GO term enrichment for genetic ancestry differential methylation regions across the caudate nucleus, DLPFC and hippocampus.

Supplementary Data 10 Excel file of stratified LD score regression of admixed Black American differential expression analysis separated by direction of effect (all DEGs, upregulated in AA or upregulated in EA) for genes (SNP proportion > 0.01) across the caudate nucleus, dentate gyrus, DLPFC and hippocampus.

Supplementary Data 11 CSV file of individual-level subject information including information on sex, age and self-identified ethnicity for the caudate nucleus, dentate gyrus, DLPFC and hippocampus.

Supplementary Data 13 Excel file of GWAS summary statistics for the heritability enrichment analysis.

Supplementary Data 15 Excel file of GO term enrichment and GSEA for internal validation of genetic-ancestry (binary) DEGs across the caudate nucleus, dentate gyrus, DLPFC and hippocampus for four features (gene, transcript, exon, junction).

## Data Availability

Publicly available BrainSeq Consortium total RNA DLPFC and hippocampus RangedSummarizedExperiment R Objects with processed counts are available at eqtl.brainseq.org/phase2/. Publicly available BrainSeq Consortium total RNA caudate RangedSummarizedExperiment R Objects with processed counts are available at erwinpaquolalab.libd.org/caudate_eqtl/. Publicly available dentate gyrus RangedSummarizedExperiment R Objects with processed counts and phenotype information are available at research.libd.org/dg_hippo_paper/data.html. Analysis-ready genotype data will be shared with researchers who obtain database of Genotypes and Phenotypes (dbGaP) accession no. phs000979.v3.p2. FASTQ files for total RNA DLPFC and hippocampus are available via the LIBD Globus collections jhpce#bsp2-dlpfc and jhpce#bsp2-hippo at research.libd.org/globus/. FASTQ files for the dentate gyrus are available via the Sequence Read Archive (accession no. SRP241159). FASTQ files for the caudate nucleus are available via dbGaP accession no. phs003495.v1.p1. DNA methylation data are available at github.com/LieberInstitute/aanri_phase1 (ref. ^108^). Supplementary Data 1, 2, 6, 11 and 13 are hosted on *Zenodo* (https://zenodo.org/doi/10.5281/zenodo.7777821). We used publicly available single-cell datasets. Glial subpopulation single-cell data from the human postmortem hippocampus astrocyte, microglia and oligodendrocyte lineages is available from the UCSC cell browser (‘Human Hippocampus Lifespan’ collection). The human PBMC single-cell data are available from *Zenodo* (10.5281/zenodo.4273999)^109^. Multiple human brain region single-cell datasets (that is, DLPFC, hippocampus, nucleus accumbens, amygdala and subgenual anterior cingulate cortex) are available according to brain region from GitHub (github.com/LieberInstitute/10xPilot_snRNAseq-human). Human microglial state dynamics in AD single-cell data are available from compbio.mit.edu/microglia_states/. We downloaded the following additional publicly available genotype data sources. We downloaded the LOF variant information from the gnomAD v.2 website (gnomad.broadinstitute.org/downloads) via Google Cloud Public Datasets storage (https://storage.googleapis.com/gcp-public-data--gnomad/release/2.1.1/constraint/gnomad.v2.1.1.lof_metrics.by_transcript.txt.bgz). We downloaded genotype references for the 1000 Genomes Project from www.internationalgenome.org/data/. We downloaded the HapMap Project Phase 3 SNPs from www.broadinstitute.org/medical-and-population-genetics/hapmap-3. We downloaded all SNPs from the LDSC resource website at data.broadinstitute.org/alkesgroup/LDSCORE/w_hm3.snplist.bz2.

## Code availability

All code and Jupyter Notebooks (v.6.0.2) are available through GitHub at github.com/LieberInstitute/aanri_phase1 with more details on *Zenodo* (10.5281/zenodo.7777821)^108^.

## References

[1] Bailey ZD (2017). Structural racism and health inequities in the USA: evidence and interventions. Lancet.

[2] Gurdasani D, Barroso I, Zeggini E, Sandhu MS (2019). Genomics of disease risk in globally diverse populations. Nat. Rev. Genet..

[3] Sirugo G, Williams SM, Tishkoff SA (2019). The missing diversity in human genetic studies. Cell.

[4] Weinberger DR, Dzirasa K, Crumpton-Young LL (2020). Missing in action: African ancestry brain research. Neuron.

[5] Bentley AR, Callier SL, Rotimi CN (2020). Evaluating the promise of inclusion of African ancestry populations in genomics. NPJ Genom. Med..

[6] Auton A (2015). A global reference for human genetic variation. Nature.

[7] Bick AG (2024). Genomic data in the All of Us Research Program. Nature.

[8] Taliun D (2021). Sequencing of 53,831 diverse genomes from the NHLBI TOPMed Program. Nature.

[9] Rotimi C (2014). Research capacity. Enabling the genomic revolution in Africa. Science.

[10] Collado-Torres L (2019). Regional heterogeneity in gene expression, regulation, and coherence in the frontal cortex and hippocampus across development and schizophrenia. Neuron.

[11] Jaffe AE (2020). Profiling gene expression in the human dentate gyrus granule cell layer reveals insights into schizophrenia and its genetic risk. Nat. Neurosci..

[12] Benjamin KJM (2022). Analysis of the caudate nucleus transcriptome in individuals with schizophrenia highlights effects of antipsychotics and new risk genes. Nat. Neurosci..

[13] Jaffe AE (2018). Developmental and genetic regulation of the human cortex transcriptome illuminate schizophrenia pathogenesis. Nat. Neurosci..

[14] Perzel Mandell KA (2021). Genome-wide sequencing-based identification of methylation quantitative trait loci and their role in schizophrenia risk. Nat. Commun..

[15] Fromer M (2016). Gene expression elucidates functional impact of polygenic risk for schizophrenia. Nat. Neurosci..

[16] Gandal MJ (2018). Transcriptome-wide isoform-level dysregulation in ASD, schizophrenia, and bipolar disorder. Science.

[17] Pritchard JK, Stephens M, Donnelly P (2000). Inference of population structure using multilocus genotype data. Genetics.

[18] Nédélec Y (2016). Genetic ancestry and natural selection drive population differences in immune responses to pathogens. Cell.

[19] Tishkoff SA (2009). The genetic structure and history of Africans and African Americans. Science.

[20] Jaffe AE (2017). qSVA framework for RNA quality correction in differential expression analysis. Proc. Natl Acad. Sci. USA.

[21] Urbut SM, Wang G, Carbonetto P, Stephens M (2019). Flexible statistical methods for estimating and testing effects in genomic studies with multiple conditions. Nat. Genet..

[22] Langfelder P, Horvath S (2008). WGCNA: an R package for weighted correlation network analysis. BMC Bioinformatics.

[23] Zeisel A (2018). Molecular architecture of the mouse nervous system. Cell.

[24] Randolph HE (2021). Genetic ancestry effects on the response to viral infection are pervasive but cell type specific. Science.

[25] Su Y (2022). A single-cell transcriptome atlas of glial diversity in the human hippocampus across the postnatal lifespan. Cell Stem Cell.

[26] Tran MN (2021). Single-nucleus transcriptome analysis reveals cell-type-specific molecular signatures across reward circuitry in the human brain. Neuron.

[27] Kang HJ (2011). Spatio-temporal transcriptome of the human brain. Nature.

[28] Karczewski KJ (2020). The mutational constraint spectrum quantified from variation in 141,456 humans. Nature.

[29] De S, Lopez-Bigas N, Teichmann SA (2008). Patterns of evolutionary constraints on genes in humans. BMC Evol. Biol..

[30] Quintana-Murci L, Clark AG (2013). Population genetic tools for dissecting innate immunity in humans. Nat. Rev. Immunol..

[31] Hou K (2023). Causal effects on complex traits are similar for common variants across segments of different continental ancestries within admixed individuals. Nat. Genet..

[32] Gazal S (2017). Linkage disequilibrium-dependent architecture of human complex traits shows action of negative selection. Nat. Genet..

[33] Orrù V (2013). Genetic variants regulating immune cell levels in health and disease. Cell.

[34] Orrù V (2020). Complex genetic signatures in immune cells underlie autoimmunity and inform therapy. Nat. Genet..

[35] Patin E (2018). Natural variation in the parameters of innate immune cells is preferentially driven by genetic factors. Nat. Immunol..

[36] Sun N (2023). Human microglial state dynamics in Alzheimer’s disease progression. Cell.

[37] Liu X, Li YI, Pritchard JK (2019). Trans effects on gene expression can drive omnigenic inheritance. Cell.

[38] Albert FW, Bloom JS, Siegel J, Day L, Kruglyak L (2018). Genetics of *trans*-regulatory variation in gene expression. eLife.

[39] Maynard KR (2021). Transcriptome-scale spatial gene expression in the human dorsolateral prefrontal cortex. Nat. Neurosci..

[40] Virani SS (2021). Heart disease and stroke statistics—2021 update: a report from the American Heart Association. Circulation.

[41] Prapiadou S, Demel SL, Hyacinth HI (2021). Genetic and genomic epidemiology of stroke in people of African ancestry. Genes.

[42] Alzheimer’s Association (2010). 2010 Alzheimer’s disease facts and figures. Alzheimers Dement..

[43] Power MC (2021). Trends in relative incidence and prevalence of dementia across non-Hispanic Black and White individuals in the United States, 2000–2016. JAMA Neurol..

[44] Kessler II (1972). Epidemiologic studies of Parkinson’s disease. II. A hospital-based survey. Am. J. Epidemiol..

[45] Colbran LL (2019). Inferred divergent gene regulation in archaic hominins reveals potential phenotypic differences. Nat. Ecol. Evol..

[46] Liston A, Carr EJ, Linterman MA (2016). Shaping variation in the human immune system. Trends Immunol..

[47] Mangino M, Roederer M, Beddall MH, Nestle FO, Spector TD (2017). Innate and adaptive immune traits are differentially affected by genetic and environmental factors. Nat. Commun..

[48] Debnath M (2015). Adaptive immunity in schizophrenia: functional implications of T cells in the etiology, course and treatment. J. Neuroimmune Pharmacol..

[49] Li X (2009). Elevated immune response in the brain of autistic patients. J. Neuroimmunol..

[50] Jevtic S, Sengar AS, Salter MW, McLaurin J (2017). The role of the immune system in Alzheimer disease: etiology and treatment. Ageing Res. Rev..

[51] Heun-Johnson H (2021). Association between race/ethnicity and disparities in health care use before first-episode psychosis among privately insured young patients. JAMA Psychiatry.

[52] Hemming JP (2011). Racial and socioeconomic disparities in parkinsonism. Arch. Neurol..

[53] Roman-Urrestarazu A (2021). Association of race/ethnicity and social disadvantage with autism prevalence in 7 million school children in England. JAMA Pediatr..

[54] Kent WJ (2002). The human genome browser at UCSC. Genome Res..

[55] Das S (2016). Next-generation genotype imputation service and methods. Nat. Genet..

[56] Fuchsberger C, Abecasis GR, Hinds DA (2015). minimac2: faster genotype imputation. Bioinformatics.

[57] Loh P-R (2016). Reference-based phasing using the Haplotype Reference Consortium panel. Nat. Genet..

[58] Chang, C. PLINK 2.0 alpha. http://www.cog-genomics.org/plink/2.0/ (2021).

[59] Luo Y (2021). A high-resolution HLA reference panel capturing global population diversity enables multi-ancestry fine-mapping in HIV host response. Nat. Genet..

[60] Danecek P (2021). Twelve years of SAMtools and BCFtools. Gigascience.

[61] Perzel Mandell KA (2022). Molecular phenotypes associated with antipsychotic drugs in the human caudate nucleus. Mol. Psychiatry.

[62] Krueger, F., James, F., Ewels, P., Afyounian, E. & Schuster-Boeckler, B. TrimGalore: a wrapper around Cutadapt and FastQC to consistently apply adapter and quality trimming to FastQ files, with extra functionality for RRBS data. *Zenodo*10.5281/zenodo.5127899 (2021).

[63] Wilton R, Li X, Feinberg AP, Szalay AS (2018). Arioc: GPU-accelerated alignment of short bisulfite-treated reads. Bioinformatics.

[64] Faust GG, Hall IM (2014). SAMBLASTER: fast duplicate marking and structural variant read extraction. Bioinformatics.

[65] Li H (2009). The Sequence Alignment/Map format and SAMtools. Bioinformatics.

[66] Krueger F, Andrews SR (2011). Bismark: a flexible aligner and methylation caller for Bisulfite-Seq applications. Bioinformatics.

[67] Hansen KD, Langmead B, Irizarry RA (2012). BSmooth: from whole genome bisulfite sequencing reads to differentially methylated regions. Genome Biol..

[68] Maples BK, Gravel S, Kenny EE, Bustamante CD (2013). RFMix: a discriminative modeling approach for rapid and robust local-ancestry inference. Am. J. Hum. Genet..

[69] Fairley S, Lowy-Gallego E, Perry E, Flicek P (2020). The International Genome Sample Resource (IGSR) collection of open human genomic variation resources. Nucleic Acids Res..

[70] Jew B (2020). Accurate estimation of cell composition in bulk expression through robust integration of single-cell information. Nat. Commun..

[71] Robinson MD, McCarthy DJ, Smyth GK (2010). edgeR: a Bioconductor package for differential expression analysis of digital gene expression data. Bioinformatics.

[72] McCarthy DJ, Chen Y, Smyth GK (2012). Differential expression analysis of multifactor RNA-Seq experiments with respect to biological variation. Nucleic Acids Res..

[73] Law CW, Chen Y, Shi W, Smyth GK (2014). voom: precision weights unlock linear model analysis tools for RNA-seq read counts. Genome Biol..

[74] Stovner EB, Sætrom P (2020). PyRanges: efficient comparison of genomic intervals in Python. Bioinformatics.

[75] Bryois J (2020). Genetic identification of cell types underlying brain complex traits yields insights into the etiology of Parkinson’s disease. Nat. Genet..

[76] Speir ML (2021). UCSC Cell Browser: visualize your single-cell data. Bioinformatics.

[77] Hao Y (2021). Integrated analysis of multimodal single-cell data. Cell.

[78] Amezquita RA (2020). Orchestrating single-cell analysis with Bioconductor. Nat. Methods.

[79] McCarthy DJ, Campbell KR, Lun ATL, Wills QF (2017). Scater: pre-processing, quality control, normalization and visualization of single-cell RNA-seq data in R. Bioinformatics.

[80] Haghverdi L, Lun ATL, Morgan MD, Marioni JC (2018). Batch effects in single-cell RNA-sequencing data are corrected by matching mutual nearest neighbors. Nat. Biotechnol..

[81] Lopez R, Regier J, Cole MB, Jordan MI, Yosef N (2018). Deep generative modeling for single-cell transcriptomics. Nat. Methods.

[82] Gayoso A (2022). A Python library for probabilistic analysis of single-cell omics data. Nat. Biotechnol..

[83] Xu C (2021). Probabilistic harmonization and annotation of single-cell transcriptomics data with deep generative models. Mol. Syst. Biol..

[84] Phipson B (2022). Propeller: testing for differences in cell type proportions in single cell data. Bioinformatics.

[85] Virtanen P (2020). SciPy 1.0: fundamental algorithms for scientific computing in Python. Nat. Methods.

[86] Seabold, S. & Perktold, J. Statsmodels: econometric and statistical modeling with Python. In *Proc. 9th Python in Science Conference* 92–96 (SciPy, 2010).

[87] Mootha VK (2003). PGC-1α-responsive genes involved in oxidative phosphorylation are coordinately downregulated in human diabetes. Nat. Genet..

[88] Subramanian A (2005). Gene set enrichment analysis: a knowledge-based approach for interpreting genome-wide expression profiles. Proc. Natl Acad. Sci. USA.

[89] Yu G, Wang L-G, Han Y, He Q-Y (2012). clusterProfiler: an R package for comparing biological themes among gene clusters. OMICS.

[90] Piñero J (2015). DisGeNET: a discovery platform for the dynamical exploration of human diseases and their genes. Database.

[91] Yu G, Wang L-G, Yan G-R, He Q-Y (2015). DOSE: an R/Bioconductor package for disease ontology semantic and enrichment analysis. Bioinformatics.

[92] Klopfenstein DV (2018). GOATOOLS: A Python library for Gene Ontology analyses. Sci. Rep..

[93] Taylor-Weiner A (2019). Scaling computational genomics to millions of individuals with GPUs. Genome Biol..

[94] Leek JT, Johnson WE, Parker HS, Jaffe AE, Storey JD (2012). The sva package for removing batch effects and other unwanted variation in high-throughput experiments. Bioinformatics.

[95] Storey JD, Tibshirani R (2003). Statistical significance for genomewide studies. Proc. Natl Acad. Sci. USA.

[96] Storey, J. D., Bass, A. J., Dabney, A. & Robinson, D. qvalue: Q-value estimation for false discovery rate control http://github.com/jdstorey/qvalue (2020).

[97] Davis JR (2016). An efficient multiple-testing adjustment for eQTL studies that accounts for linkage disequilibrium between variants. Am. J. Hum. Genet..

[98] Paszke, A. et al. PyTorch: an imperative style, high-performance deep learning library. *Proceedings of the 33rd International Conference on Neural Information Processing Systems* 721 (Curran Associates, 2019).

[99] Privé F, Aschard H, Ziyatdinov A, Blum MGB (2018). Efficient analysis of large-scale genome-wide data with two R packages: bigstatsr and bigsnpr. Bioinformatics.

[100] Gu Z, Hübschmann D (2023). rGREAT: an R/Bioconductor package for functional enrichment on genomic regions. Bioinformatics.

[101] Lee S, Cook D, Lawrence M (2019). plyranges: a grammar of genomic data transformation. Genome Biol..

[102] Cavalcante RG, Sartor MA (2017). annotatr: genomic regions in context. Bioinformatics.

[103] Gu Z, Eils R, Schlesner M (2016). Complex heatmaps reveal patterns and correlations in multidimensional genomic data. Bioinformatics.

[104] Gu Z, Gu L, Eils R, Schlesner M, Brors B (2014). circlize implements and enhances circular visualization in R. Bioinformatics.

[105] Wickham, H. *Ggplot2—Elegant Graphics for Data Analysis* (Springer, 2016).

[106] Kassambara, A. ggpubr: ‘ggplot2’ based publication ready plots. (v.0.4.15) https://CRAN.R-project.org/package=ggpubr (2020).

[107] Wei, T. & Simko, V. R package corrplot: Visualization of a correlation matrix. (v.0.92) https://github.com/taiyun/corrplot (2021).

[108] Benjamin, K. J. Git repository for Lieber Institute genetic ancestry in the brain study. *Zenodo* https://zenodo.org/doi/10.5281/zenodo.8403712 (2024).

[109] Randolph, H. E. Influenza A response variation scripts. *Zenodo*10.5281/zenodo.4273999 (2021).

