# Supplementary material for: Analysis of gene expression in the postmortem brain of neurotypical Black Americans reveals contributions of genetic ancestry
Source: Nat Neurosci. 2024 May 20;27(6):1064–74. doi: 10.1038/s41593-024-01636-0 (PMC11156587; doi:10.1038/s41593-024-01636-0)
Supplement: Supplementary file 1 — Supplementary Methods, Figs. 1–45, Tables 1–6 and Data 3–15. [file 41593_2024_1636_MOESM1_ESM.pdf]

nature neuroscience

Article https://doi.org/10.1038/s41593-024-01636-0

Analysis of gene expression in the postmortem brain of neurotypical Black Americans reveals contributions of genetic ancestry

In the format provided by the authors and unedited

Supplementary Information

Supplementary Note

DEGs in a binary contrast of Black and non-Hispanic white Americans largely confirms admixed Black American-only analysis

To extend our analysis of DEGs driven by genetic ancestry, we performed a binary analysis using combined Black and non-Hispanic white American samples (**Table S5**) – the latter showing very little admixture of African ancestry (STRUCTURE; African ancestry mean = 0.03, range = 0-0.16; **Fig. S1**). For this analysis, we selected individuals with relatively limited admixture (Black Americans ≥ 0.8 AA and white Americans > 0.99 EA). To limit the influence of the larger sample size for this binary analysis (Black American vs white American), we randomly sampled ten times without replacement to approximate the admixed Black American-only analysis sample size (caudate, n=122 [61 each]; dentate gyrus, n=46 [23 each]; DLPFC, n=124 [62 each]; and hippocampus, n=134 [67 each]). We identified more than double as many global ancestry-associated DEGs (5,324 unique genes, median lfsr < 0.05; **Fig. S42A**, **Table S6**, and **Data S14**) representing 28% of all genes tested across the caudate (n=2,877), dentate gyrus (n=2,219), DLPFC (n=3,318), and hippocampus (n=2,818) with similar immune system enrichment patterns (**Fig. S42B** and **Data S15**).

We next compared the binary analysis DE results (genes, transcripts, exons, and junctions) with the admixed Black American-only results. While we found a significant overlap of ancestry-associated DE features (Fisher's exact test, p-value < 0.0001), approximately 72% of features (3847 unique genes) were unique to the binary DE results (**Fig. S43**). Even so, effect sizes from binary analysis were significantly correlated (Spearman, rho = 0.43 to 0.49, p-value < 0.0001; **Fig. S44**) with effect sizes from admixed Black American-only analysis across features and brain regions, with a stronger correlation for shared features (Spearman, rho = 0.60 to 0.66, p-value < 0.0001; **Fig. S45**). These results confirm most of the ancestry-associated DEGs in the Black American sample alone, while also highlighting additional ancestry-related factors that influence gene expression, presumably including environmental (i.e., epigenetic) events.

We performed this combined analysis with white Americans as an internal validation. We found significant overlap between this and our Black American-only analyses (i.e., DE), but a dramatic increase in the extent of differentially expressed features. Additionally, this combined analysis (Black and white Americans) revealed similar enrichment of the immune response, again in analogous alternating directionality depending on brain region. While these results implicate environmental exposures that might reflect systematic differences between the two ancestral groups, disambiguating genetics from environmental factors in this context is challenging. We, therefore, chose to examine the environmental impact on our Black American-only global ancestry-associated DEGs. To this end, we identified thousands of VMRs across the brain in this context.

Supplementary Figures

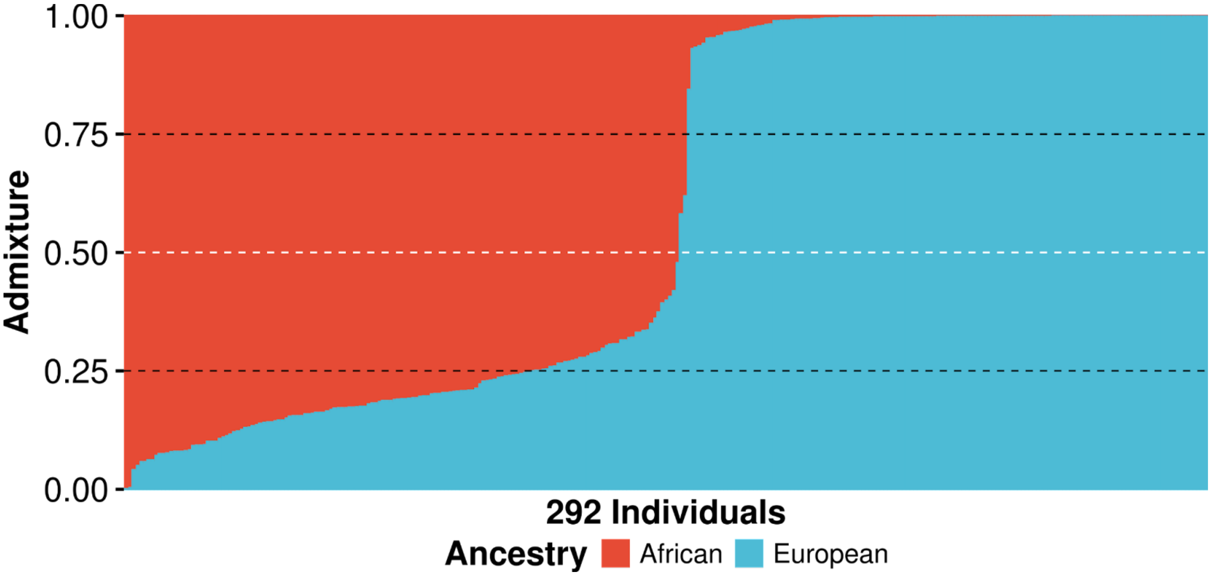

Fig. S1: Genetic ancestry estimates for Black and White American neurotypical individuals. Histogram showing estimated African and European ancestry for each unique neurotypical individual across the caudate, dentate gyrus, DLPFC, and hippocampus.

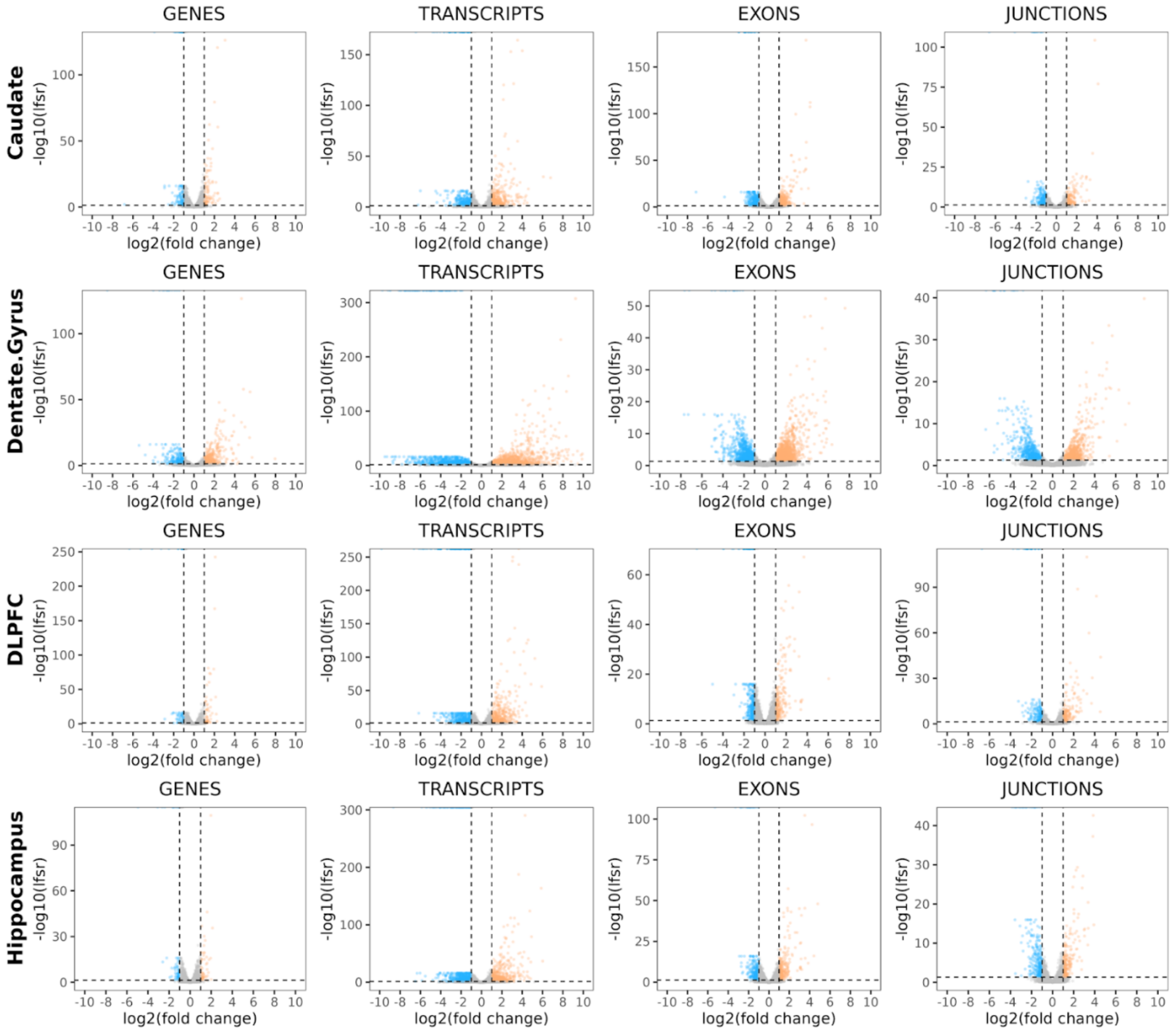

Fig. S2: Extensive global ancestry-associated differential expression across brain regions and features (i.e., gene, transcript, exon, and junction). Volcano plot of effect size ($\log_2$ of fold change) estimated from mash modeling and significance ($-\log_{10}$ of lfsr) with features associated with increased AA (blue) or EA (orange) proportions.

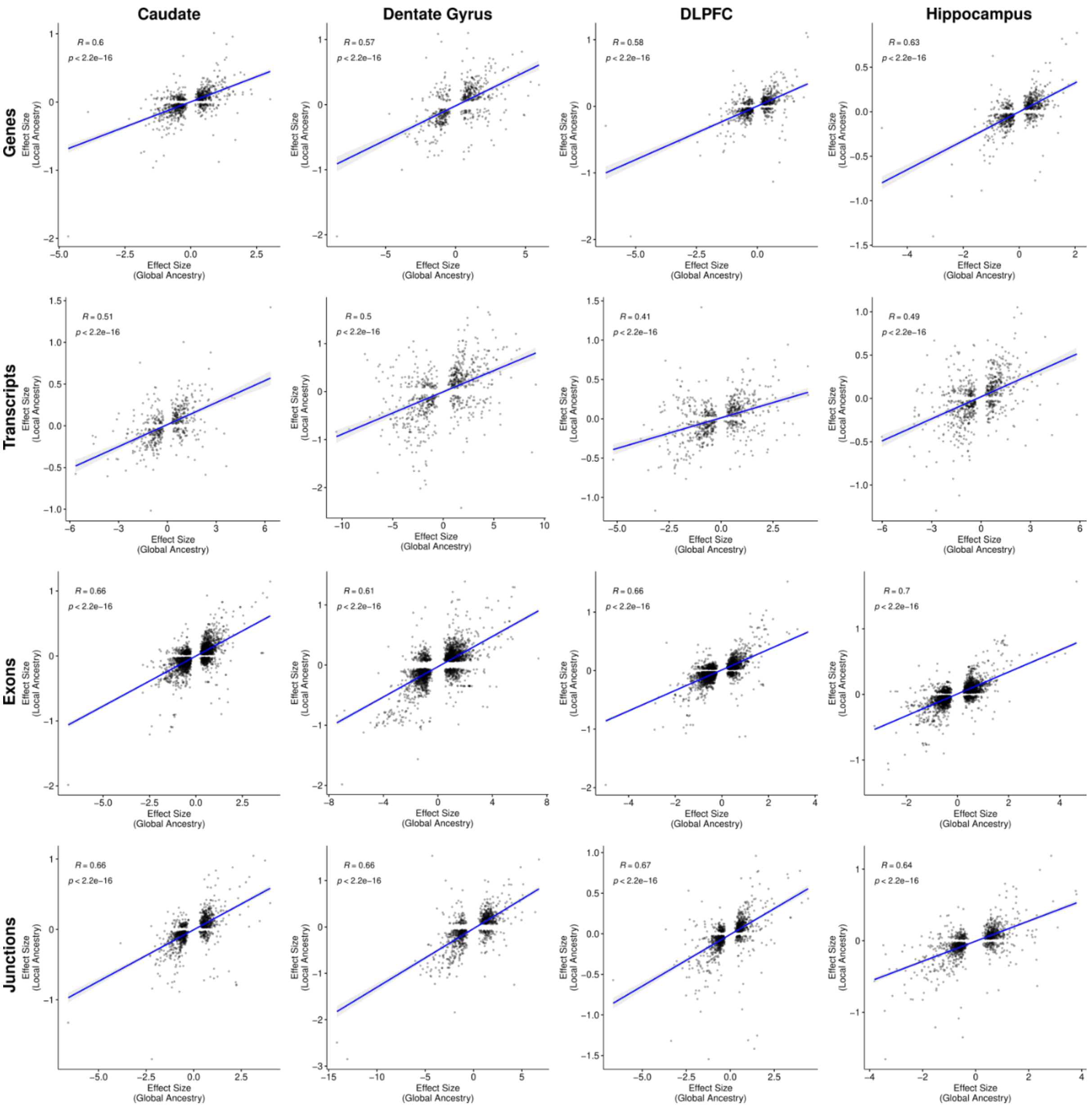

Fig. S3: Significant correlation between global and local ancestry shared features with local ancestry showing smaller effect sizes. Correlation (two-sided, Spearman) of local ancestry-associated DE features (i.e., gene, transcript, exon, and junction) effect sizes (y-axis) versus global ancestry-associated DE features effect sizes (x-axis) across brain regions. Exact p-values: the caudate (genes: 1.6e-91, transcripts: 2.4e-35, exons: ~0, and junctions: 7.9e-148), dentate gyrus (genes: 2.3e-49, transcripts: 7.3e-49, exons: 4.2e-251, and junctions: 2.4e-119), DLPFC (genes: 3.6e-68, transcripts: 3.5e-29, exons: ~0, and junctions: 1.4e-151), and hippocampus (genes: 1.6e-83, transcripts: 2.1e-47, exons: ~0, and junctions: 4.8e-144). A fitted trend line is presented in blue as the mean values +/- standard deviation. The standard deviation is shaded in light gray.

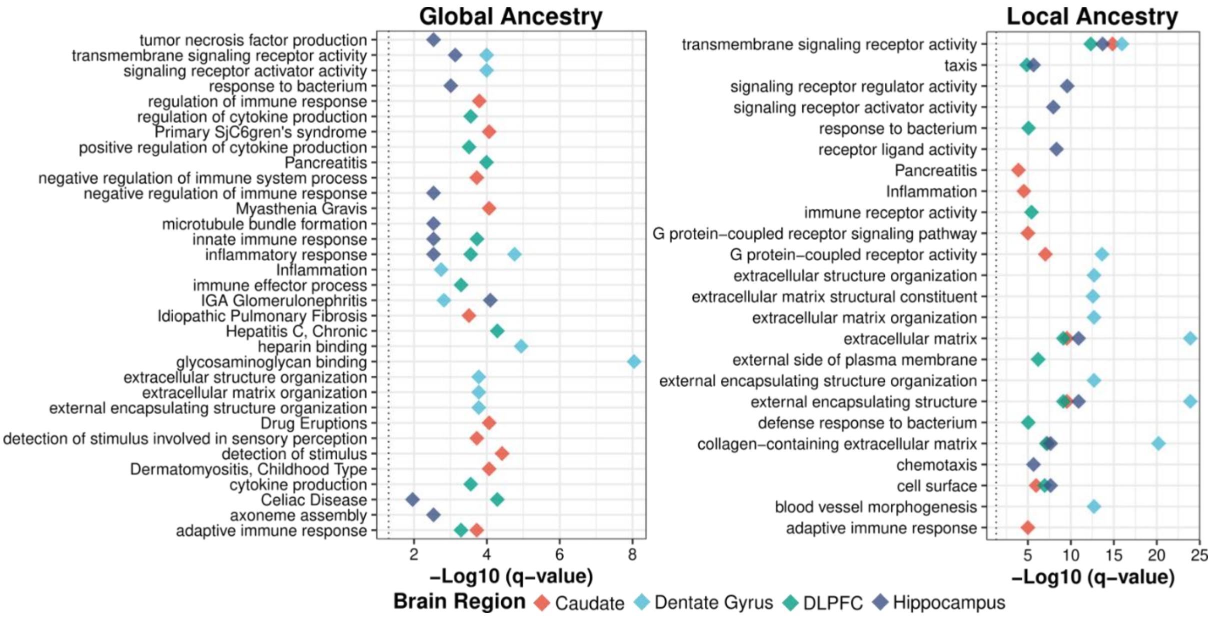

Fig. S4: Significant enrichment of immune-related pathways for ancestry-associated DEGs. GO enrichment (hypergeometric, q-value < 0.05) of all global (left) and local (right) ancestry-associated DEGs across brain regions, highlighting terms associated with immune response.

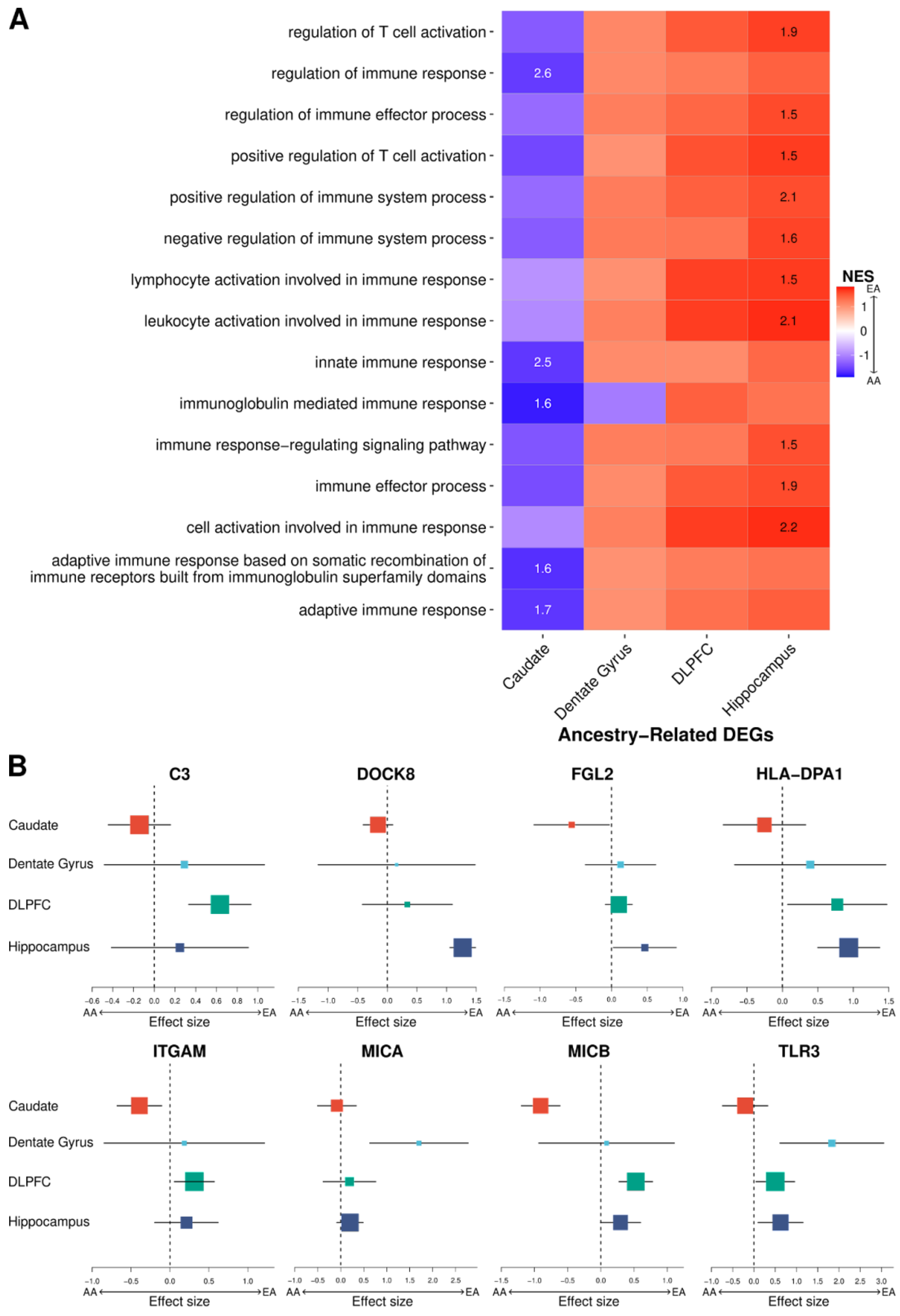

Fig. S5: Immune-related pathways show consistent direction of effect expression across brain regions. A. Heatmap showing direction of effect (increase AA proportion [blue] or increased EA proportion [red]) associated with immune-related GO terms across brain regions. Significant enrichments (GSEA, q-values < 0.05; -$\log_{10}$ transformed) annotated within tiles. **B.** Metaplot showing examples of immune-related genes associated with significantly enriched pathways (GSEA enrichment analysis) across brain regions.

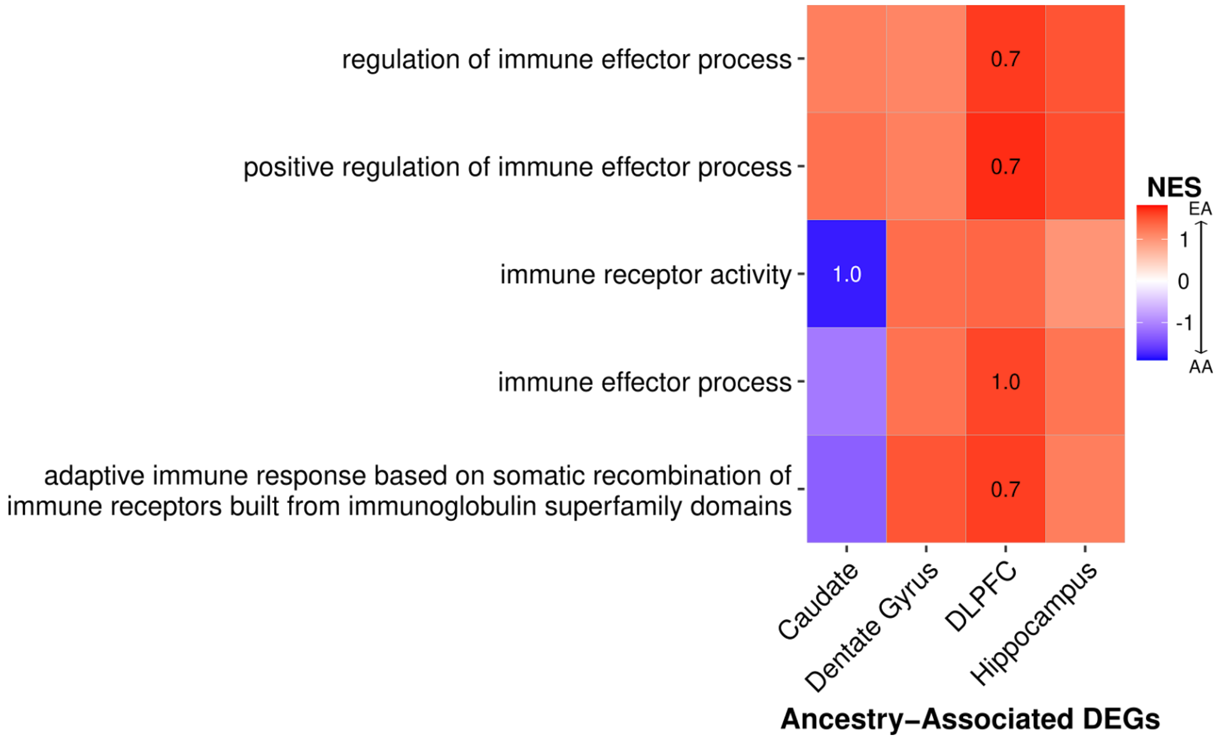

Fig. S6: Local ancestry expression show similar pattern of direction of effect in immune-related pathways across brain regions as global ancestry. Heatmap showing direction of effect (increase AA proportion [blue] or increased EA proportion [red]) associated with immune-related GO terms across brain regions. Enrichment trends (GSEA, q-values < 0.25; -$\log_{10}$ transformed) annotated within tiles.

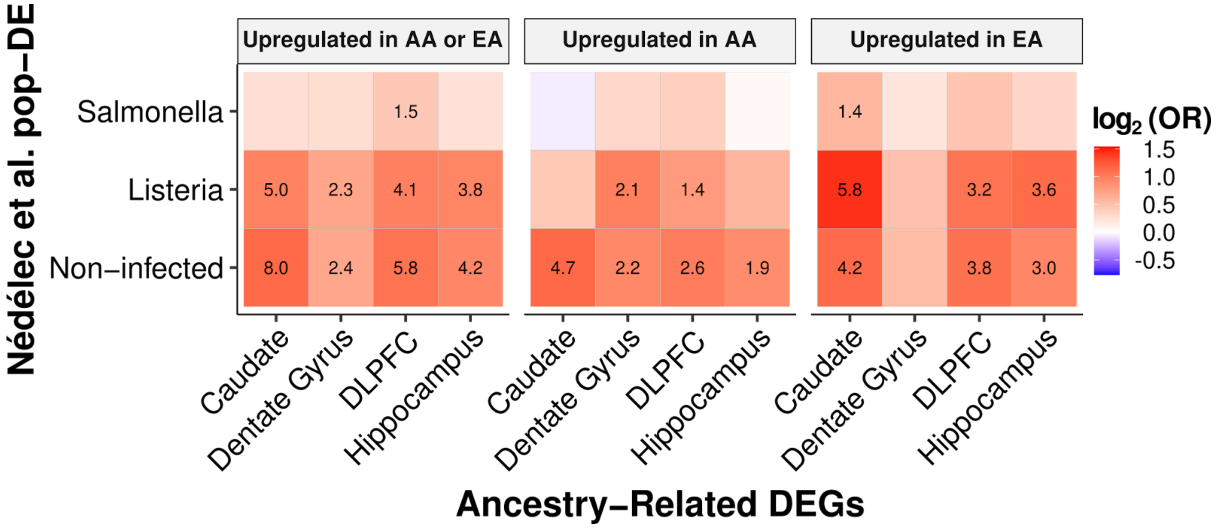

Fig. S7: Significant enrichment of ancestry-associated DEGs with population differences in immune responses. Heatmaps showing significant enrichment (red; two-sided, Fisher's exact test with p-values corrected for multiple testing with Benjamini-Hochberg) of ancestry-associated DEGs (adjusted p-value < 0.05) and population differences in primary macrophages (*18*) separated by infection status and direction of effect. Significant enrichments (two-sided, Fisher's exact test with FDR corrected p-values -$\log_{10}$ transformed) annotated within tiles.

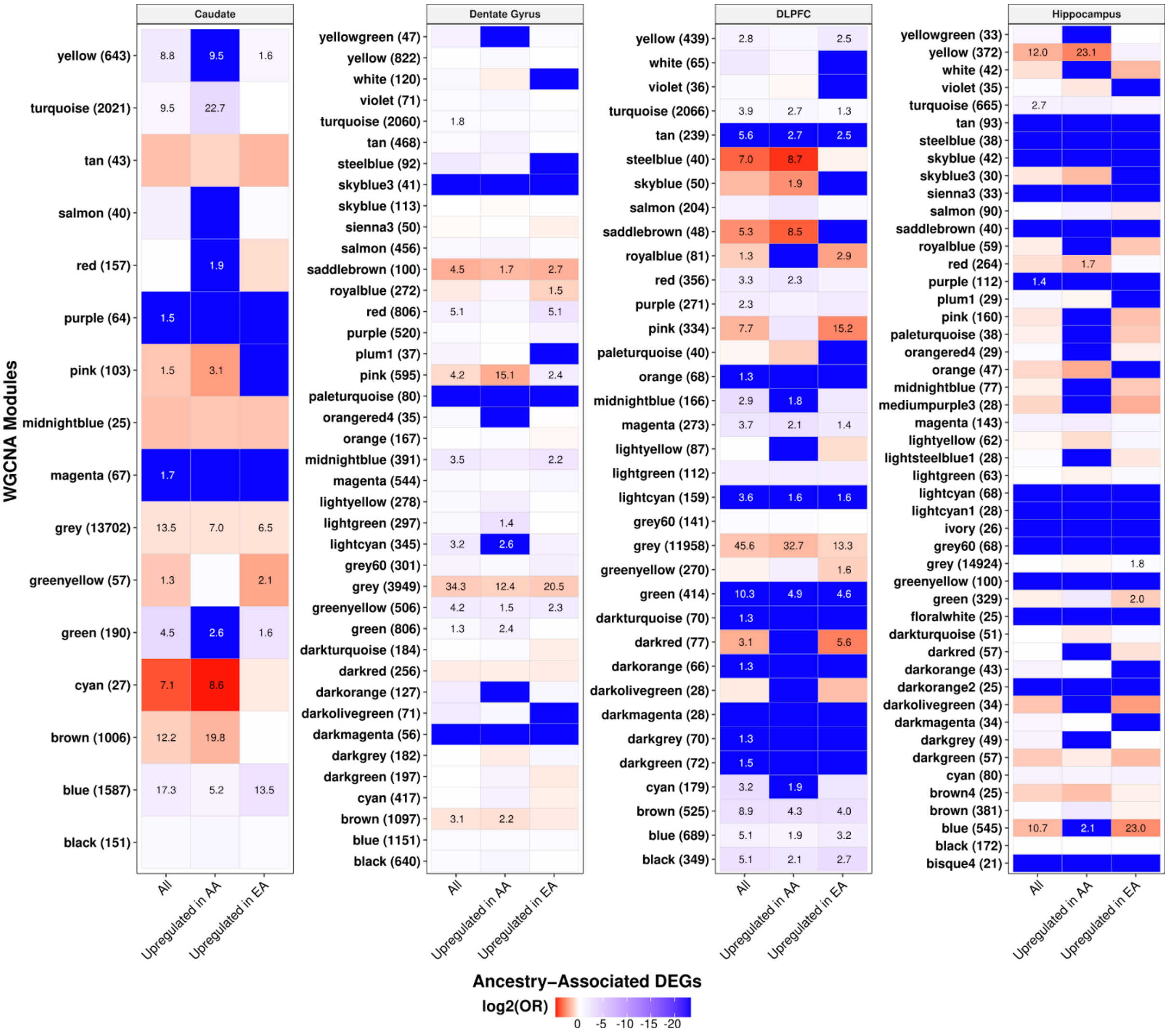

Fig. S8: Extensive enrichment of gene co-expression modules with ancestry-associated DEGs in admixed Black American individuals across brain regions. Heatmap of enrichment analysis (two-sided, Fisher's exact test with p-values corrected for multiple testing with Benjamini-Hochberg) showing significant enrichment (red) and depletion (blue) across WGCNA modules for ancestry-associated DEGs (adjusted p-value < 0.05) separated by direction of effect. Significant enrichments (two-sided, Fisher's exact test with FDR corrected p-values -$\log_{10}$ transformed) annotated within tiles.

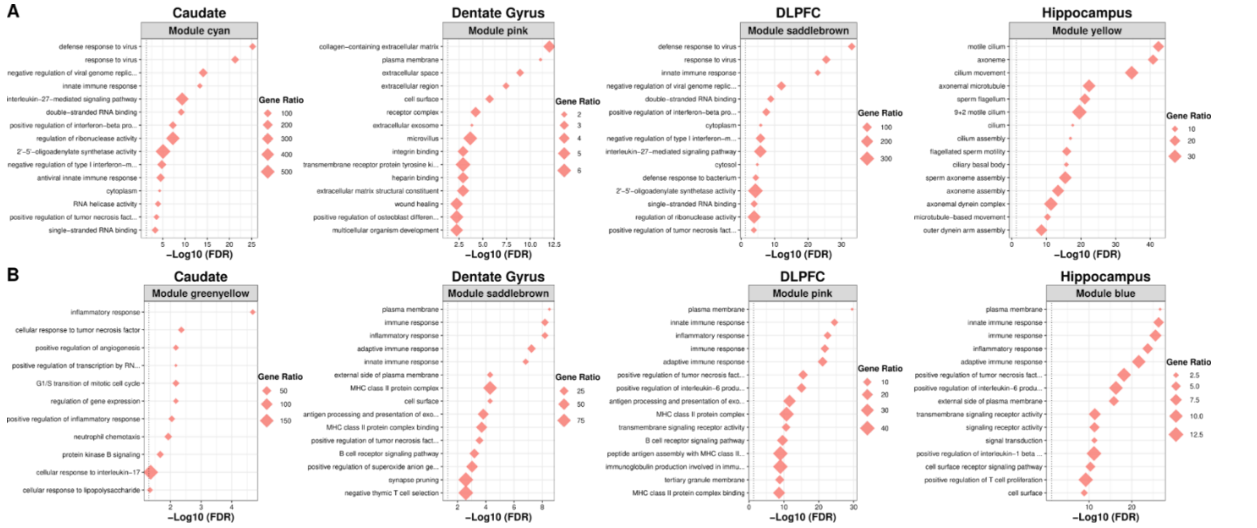

Fig. S9: Functional enrichment of gene co-expression network modules and ancestry-associated DEGs in admixed Black American across brain regions. Top 15 enriched GO terms for most significantly enriched WGCNA module for ancestry-associated DEGs that show **A.** upregulation with increasing AA proportion or **B.** upregulation with increasing EA proportion.

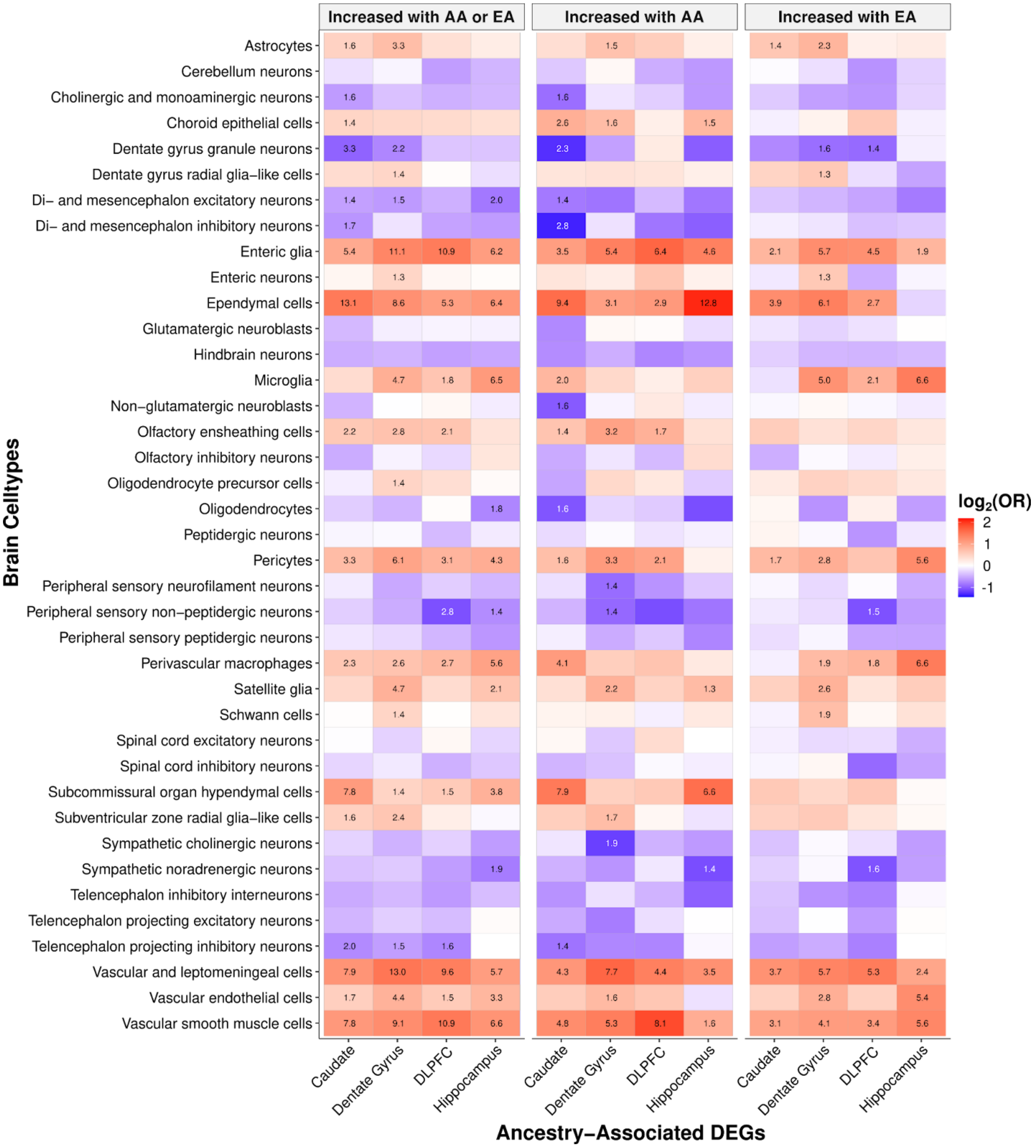

Fig. S10: Global ancestry-associated DEGs show significant enrichment of immune-related cell types (i.e., microglia and macrophages). Heatmap showing enrichment analysis (two-sided, Fisher's exact test with p-values corrected for multiple testing with Benjamini-Hochberg) of significantly enriched (red) or depleted (blue) across brain cell types (*24*) for ancestry-associated DEGs (adjusted p-value < 0.05) separated by direction of effect. Significant enrichments (two-sided, Fisher's exact test with FDR corrected p-values -log10 transformed) annotated within tiles.

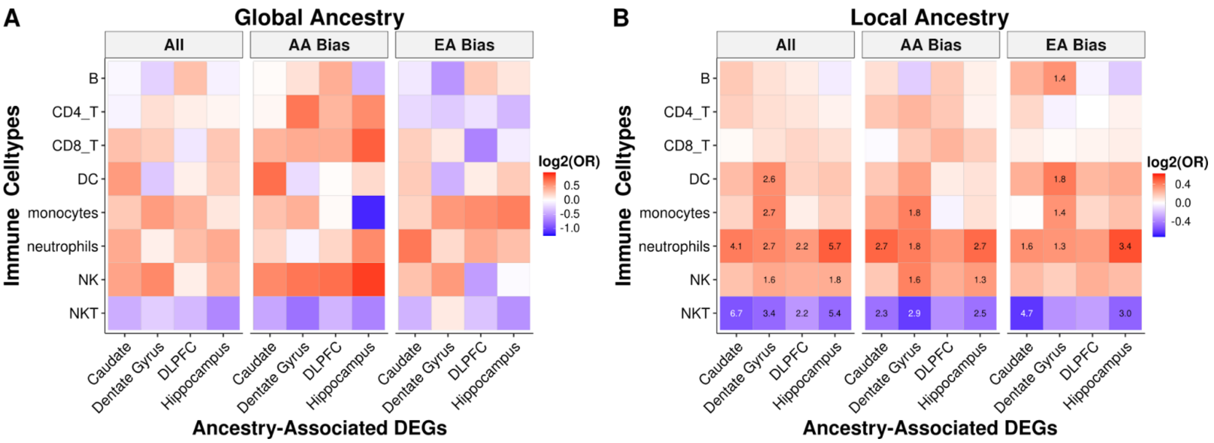

Fig. S11: Enrichment of non-brain immune cell types for local but not global ancestry DEGs. Heatmap showing enrichment analysis (two-sided, Fisher's exact test with p-values corrected for multiple testing with Benjamini-Hochberg) of significantly enriched (red) or depleted (blue) across peripheral blood mononuclear cells (PBMCs) cell types (*25*) for ancestry-associated DEGs (adjusted p-value < 0.05) separated by direction of effect. Significant enrichments (two-sided, Fisher's exact test with FDR corrected p-values -log10 transformed) annotated within tiles.

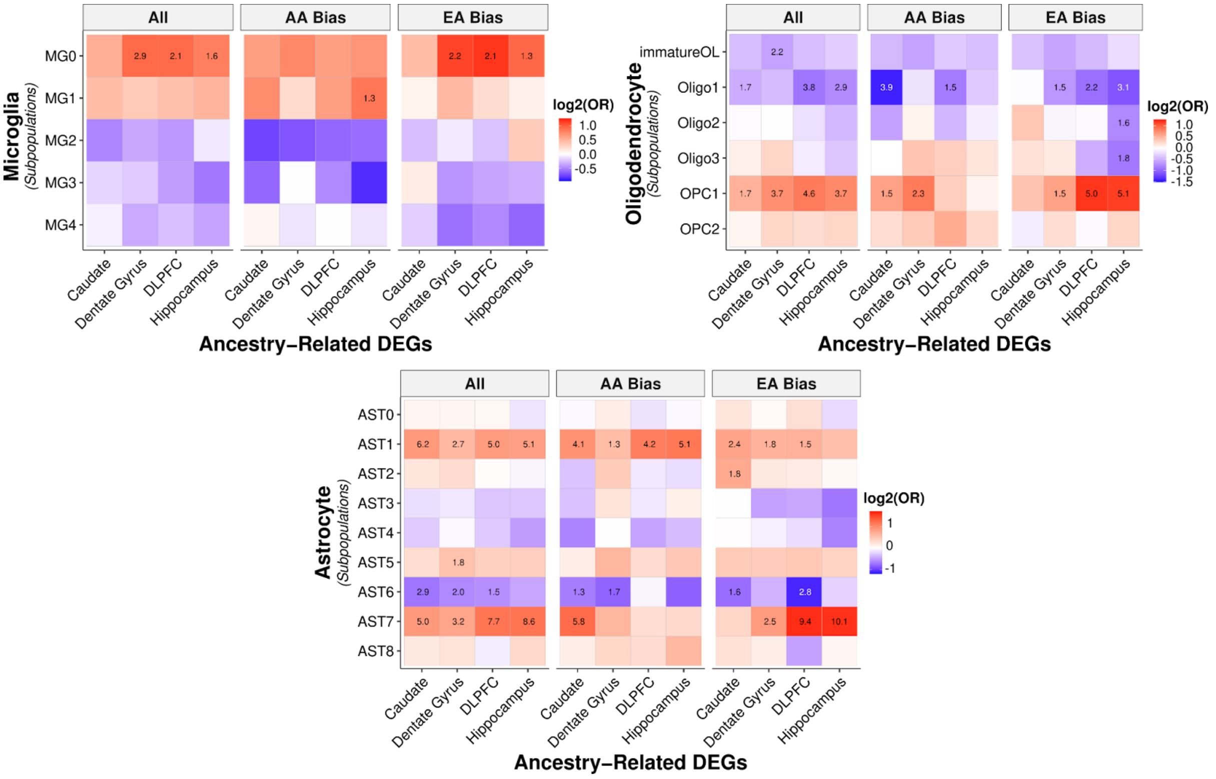

Fig. S12: Distinct, but not specific enrichment of global ancestry-associated DEGs for glial cells. Heatmap showing enrichment analysis (two-sided, Fisher's exact test with p-values corrected for multiple testing with Benjamini-Hochberg) of significantly enriched (red) or depleted (blue) across brain immune cell subtypes (*26*) for ancestry-associated DEGs (adjusted p-value < 0.05) separated by direction of effect. Significant enrichments (two-sided, Fisher's exact test with FDR corrected p-values -log10 transformed) annotated within tiles.

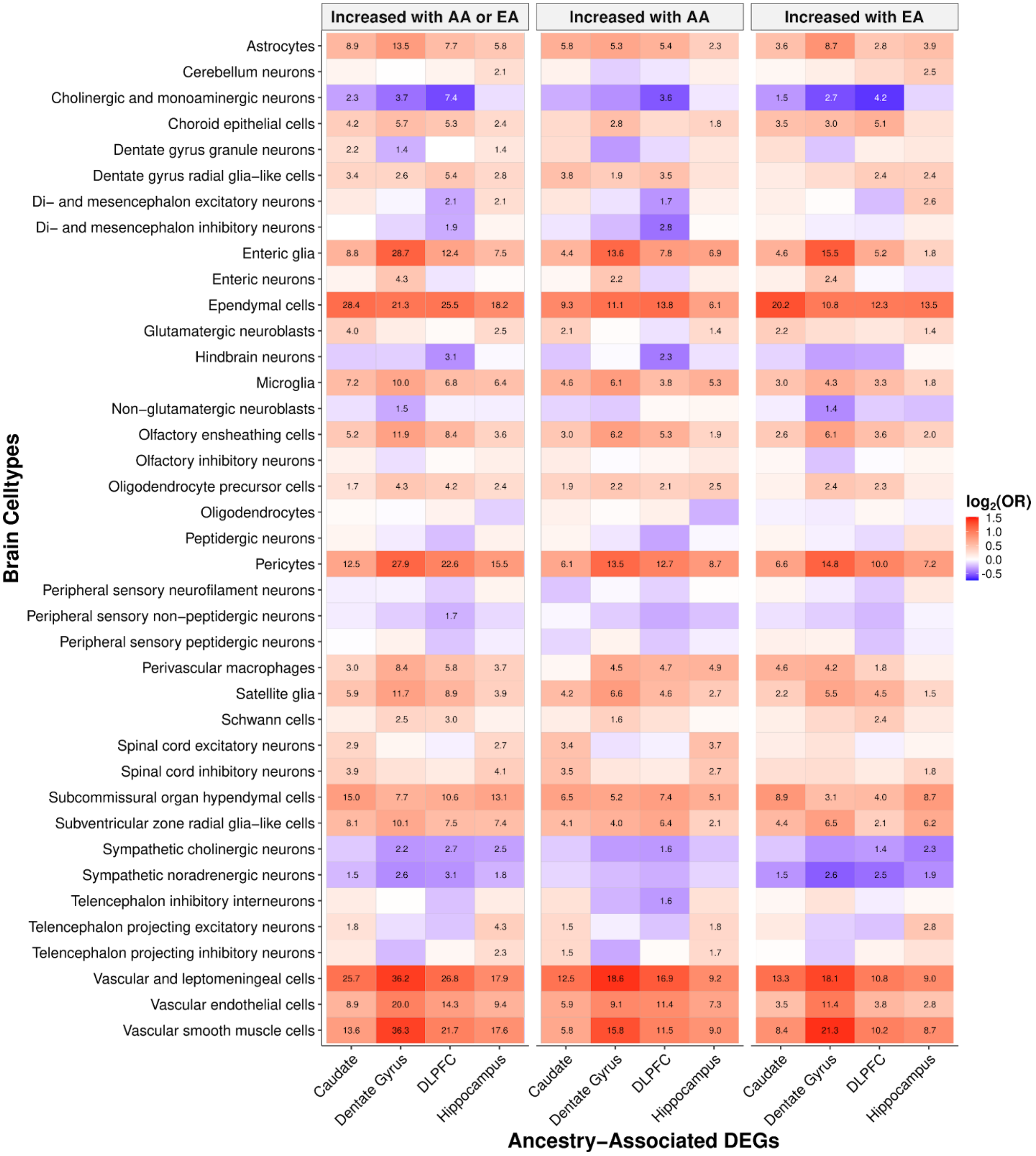

Fig. S13: Local ancestry-associated DEGs show significant enrichment of immune-related cell types (i.e., microglia and macrophages). Heatmap showing enrichment analysis (two-sided, Fisher's exact test with p-values corrected for multiple testing with Benjamini-Hochberg) of significantly enriched (red) or depleted (blue) across brain cell types (*24*) for ancestry-associated DEGs (adjusted p-value < 0.05) separated by direction of effect. Significant enrichments (two-sided, Fisher's exact test with FDR corrected p-values -log10 transformed) annotated within tiles.

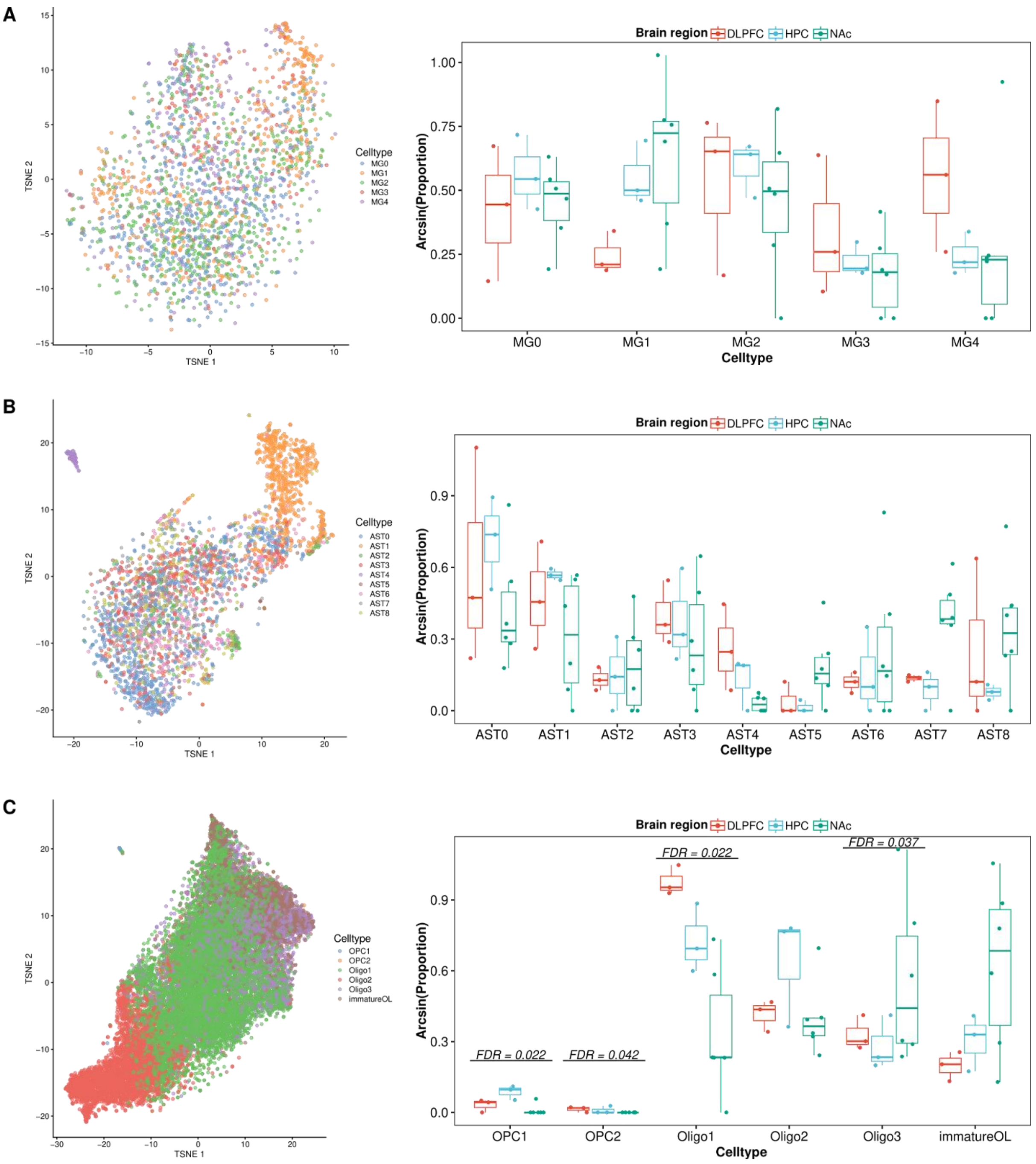

Fig. S14: The majority of glial cell composition is not significantly different across brain regions. t-SNE and cell proportion box plots (two-way, ANOVA) of single-cell data from the DLPFC (n=3), hippocampus (HPC; n=3), and nucleus accumbens (NAc; n=8) (*27*) after annotating for **A.** microglia subpopulations, **B.** astrocyte subpopulation, and **C.** oligodendrocyte lineage (*26*).Box plots show the median and first and third quartiles, and whiskers extend to 1.5× the interquartile range. Y-axis is arcsine transformed counts.

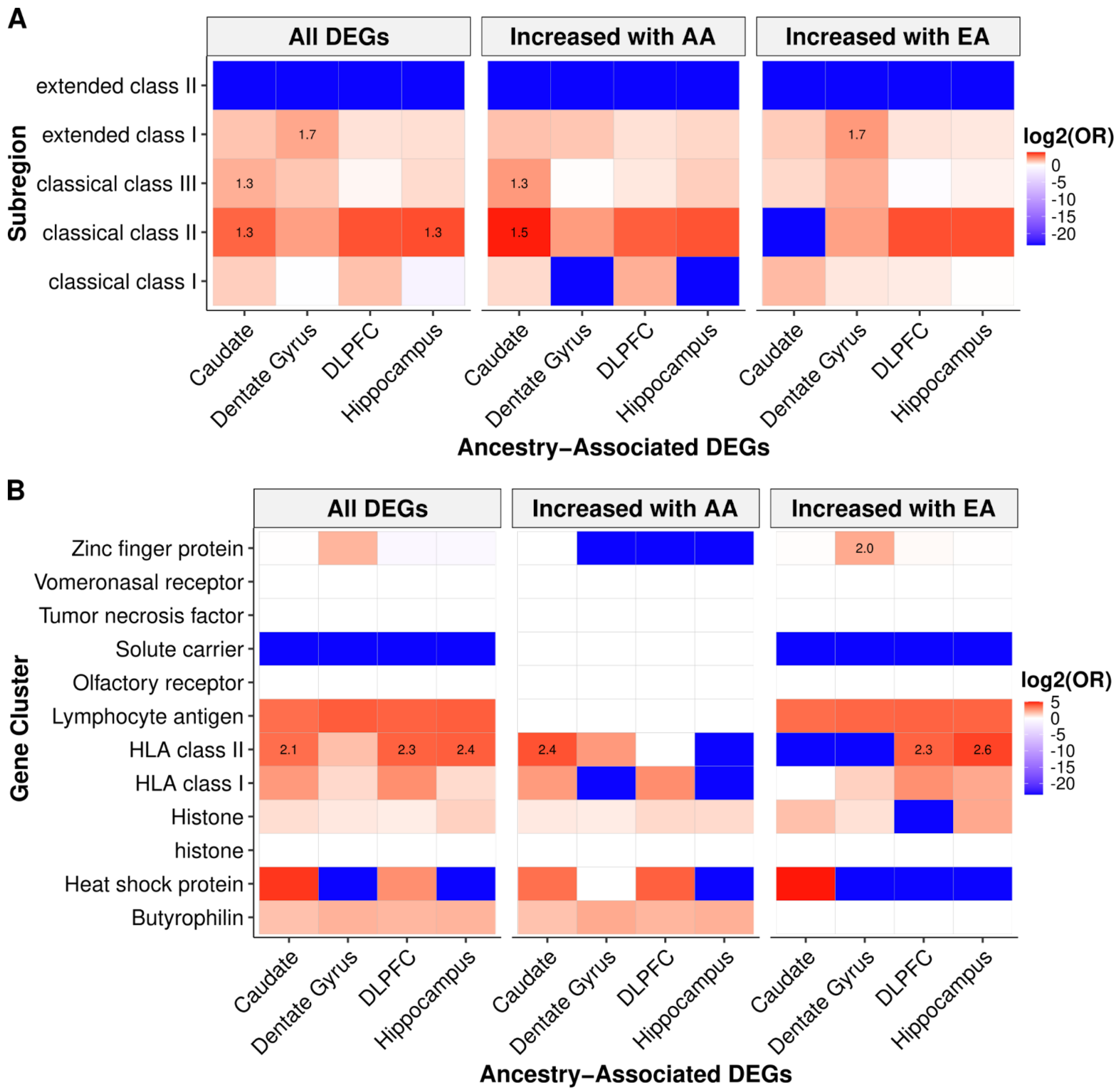

Fig. S15: Global ancestry-associated DEGs are significantly enriched for HLA class II genes. Heatmap showing enrichment analysis (two-sided, Fisher's exact test with p-values corrected for multiple testing with Benjamini-Hochberg) of significantly enriched (red) or depleted (blue) across extended MHC region for ancestry-associated DEGs (adjusted p-value < 0.05) separated by direction of effect. **A.** Subregions of the extended MHC region. **B.** Gene clusters of the extended MHC region. Significant enrichments (two-sided, Fisher's exact test with FDR corrected p-values -log10 transformed) annotated within tiles.

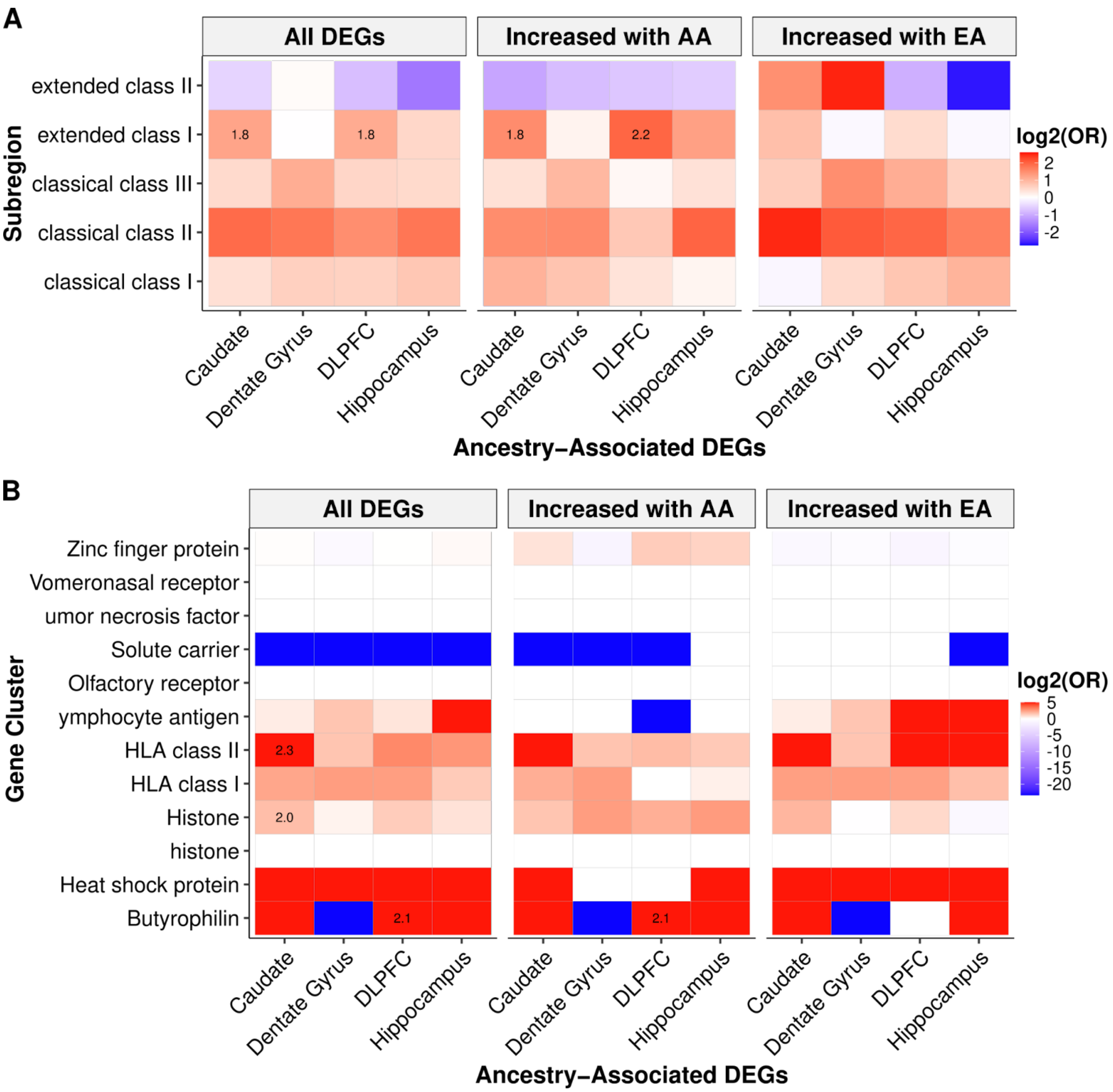

Fig. S16: Local ancestry-associated DEGs are significantly enriched for HLA class II genes. Heatmap showing enrichment analysis (two-sided, Fisher's exact test with p-values corrected for multiple testing with Benjamini-Hochberg) of significantly enriched (red) or depleted (blue) across extended MHC region for ancestry-associated DEGs (adjusted p-value < 0.05) separated by direction of effect. **A.** Subregions of the extended MHC region. **B.** Gene clusters of the extended MHC region. Significant enrichments (two-sided, Fisher's exact test with FDR corrected p-values -log10 transformed) annotated within tiles.

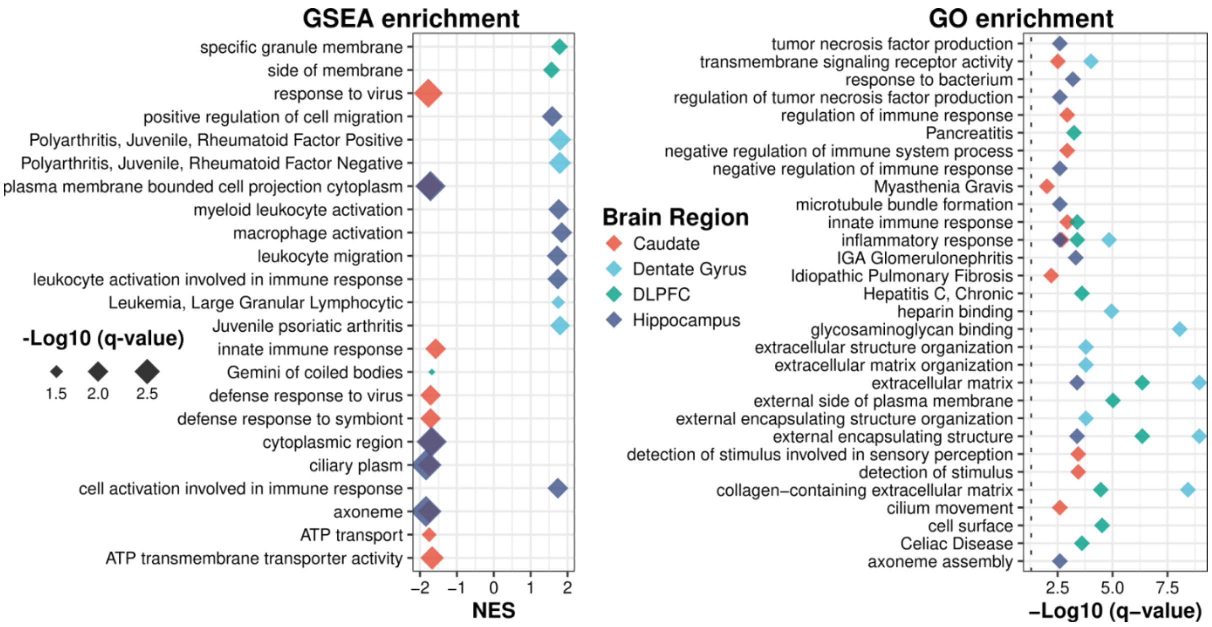

Fig. S17: HLA genes do not drive enrichment for immune-related pathways of global ancestry-associated DEGs. GSEA and GO enrichment of DEG without HLA genes across brain regions. GSEA analysis highlighting terms associated with increased AA (African ancestry) or EA (European ancestry) proportions. GO enrichment including all DEGs.

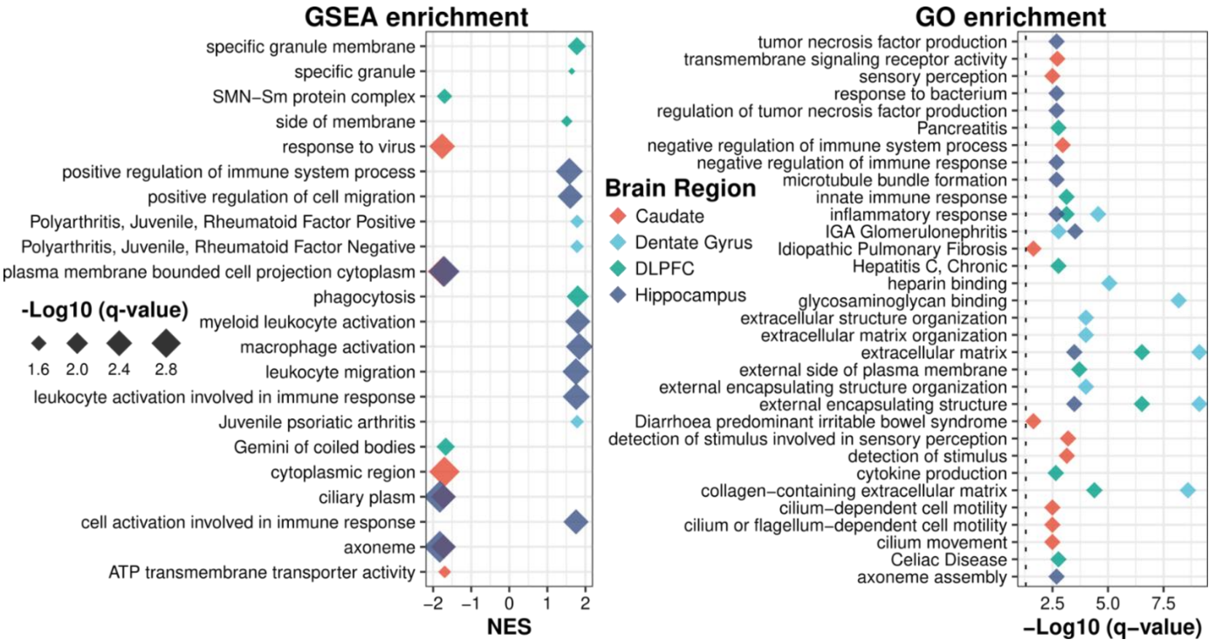

Fig. S18: MHC region does not drive enrichment for immune-related pathways of global ancestry-associated DEGs. GSEA and GO enrichment of DEG without MHC region across brain regions. GSEA analysis highlighting terms associated with increased AA (African ancestry) or EA (European ancestry) proportions. GO enrichment including all DEGs.

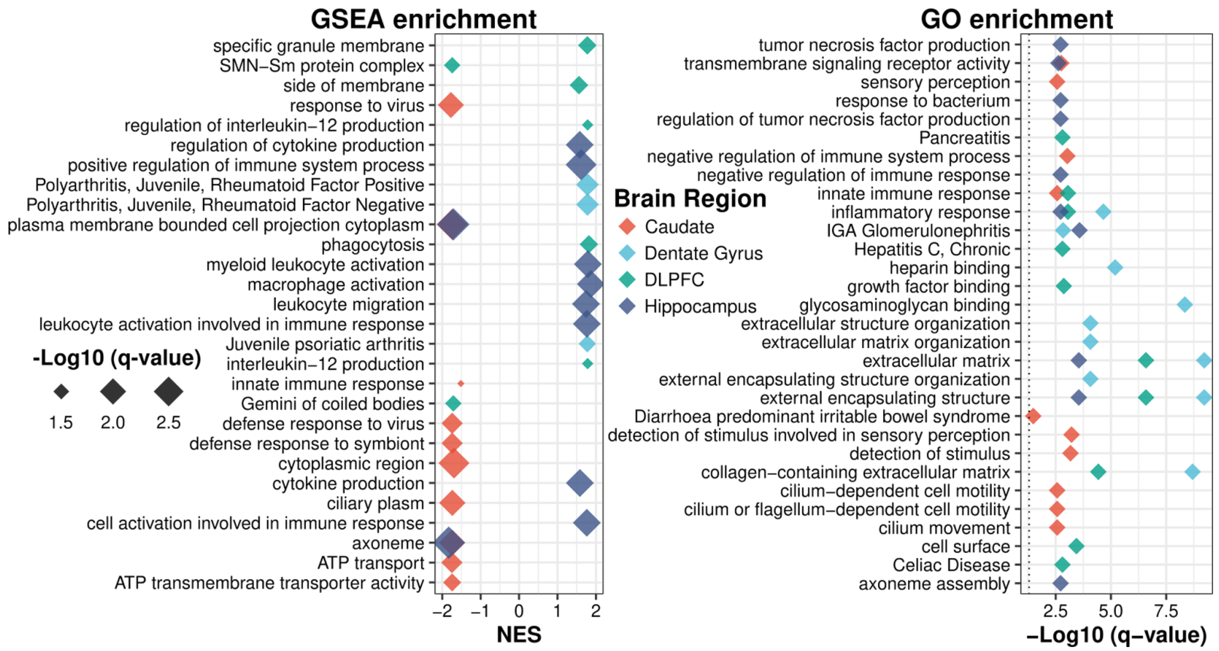

Fig. S19: Extended MHC region does not drive enrichment for immune-related pathways of global ancestry-associated DEGs. GSEA and GO enrichment of DEG without extended MHC region across brain regions. GSEA analysis highlighting terms associated with increased AA (African ancestry) or EA (European ancestry) proportions. GO enrichment including all DEGs.

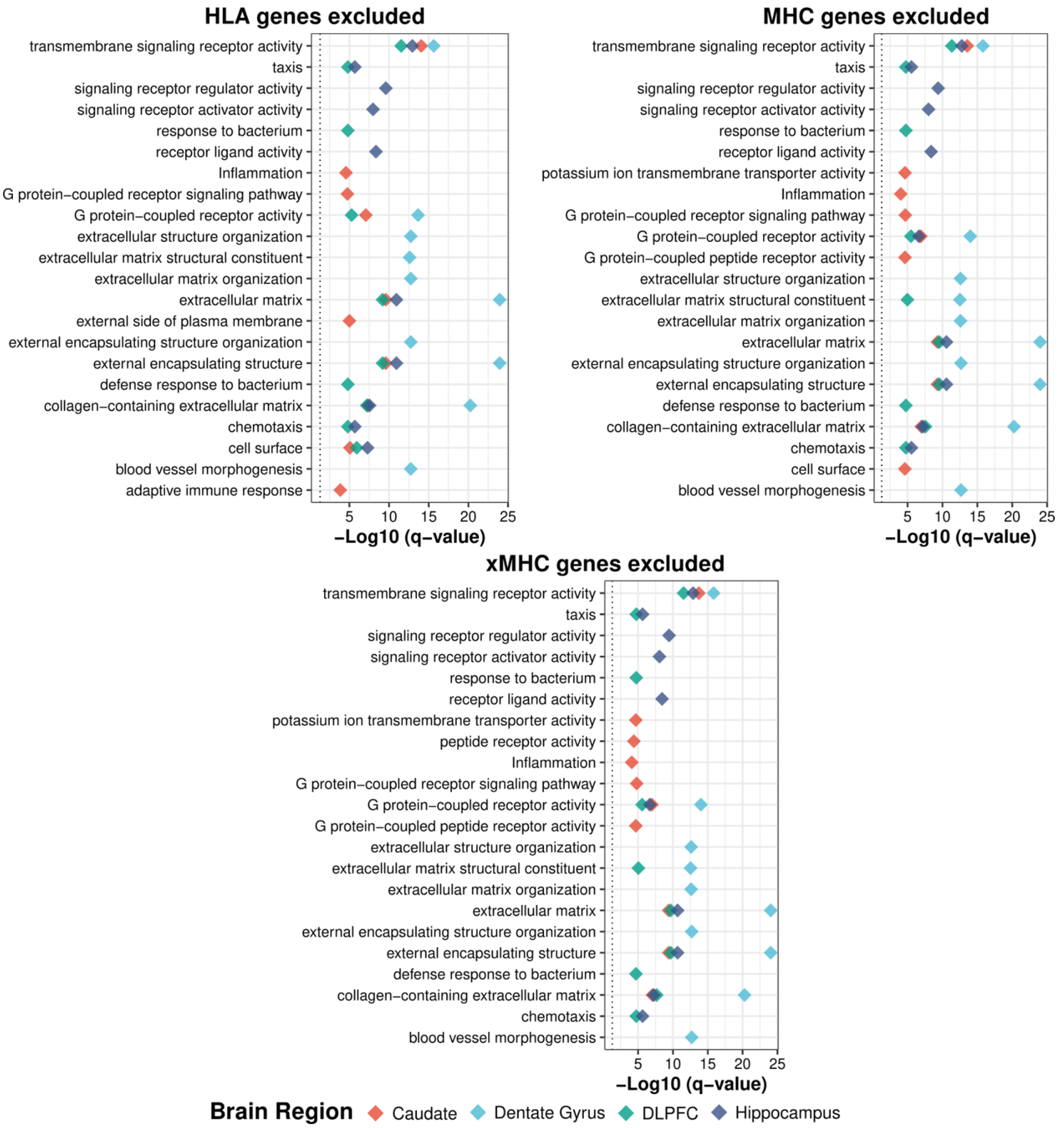

Fig. S20: Similar to global ancestry, extended MHC region does not drive enrichment for immune-related pathways of local ancestry-associated DEGs. GO enrichment of DEG without extended MHC region across brain regions. GO enrichment including all DEGs.

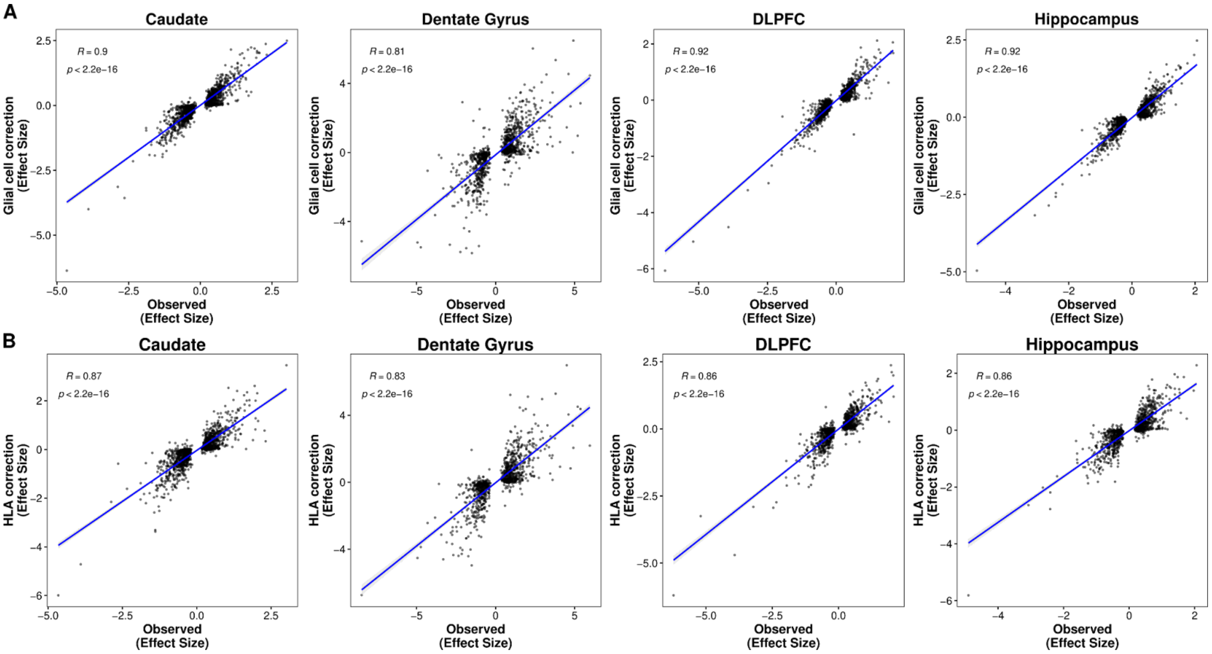

Fig. S21: Immune variation contributes only minimally to transcriptional changes of ancestry-associated DEGs. Scatter plot of global ancestry-associated DEGs showing correlation of effect sizes from general model (x-axis) and model with covariates (y-axis) associated with **A.** glial cell proportion (two-sided, Spearman; p-value is approximately 0 for the caudate, DLPFC, and hippocampus, p-value = 4.4e-232) or **B.** HLA variation (two-sided, Spearman; p-value = ~0, 1.0e-251, 1.85e-307, and 2.4e-301 for the caudate, dentate gyrus, DLPFC, and hippocampus). A fitted trend line is presented in blue as the mean values +/- standard deviation. The standard deviation is shaded in light gray.

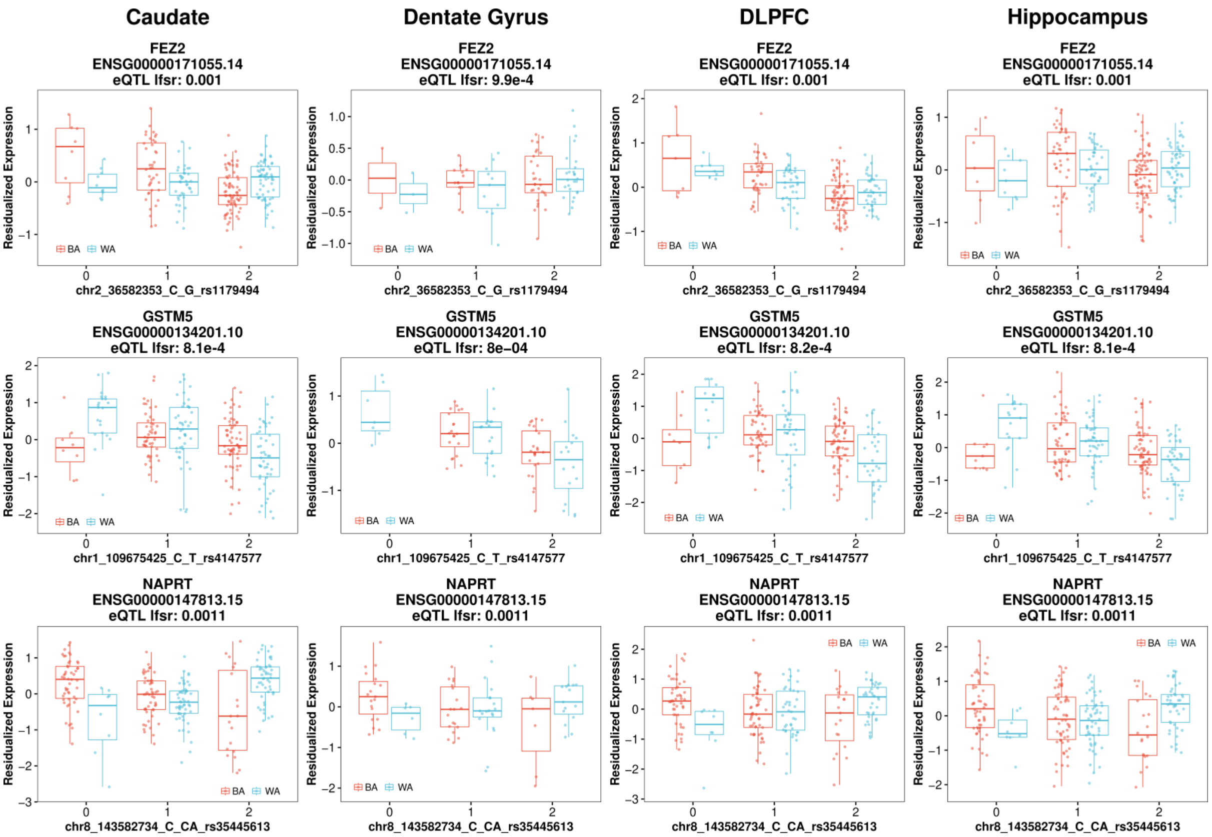

Fig. S22: Ancestry-dependent eQTL examples showing shared direction of effect across brain regions. Box plot of the most significant variant per eGene showing ancestry-dependent eQTL using combined (Black [red] and white [blue] Americans) for the caudate (n=233), dentate gyrus (n=85), DLPFC (n=204), and hippocampus (n=236). Box plots show the median and first and third quartiles, and whiskers extend to 1.5× the interquartile range.

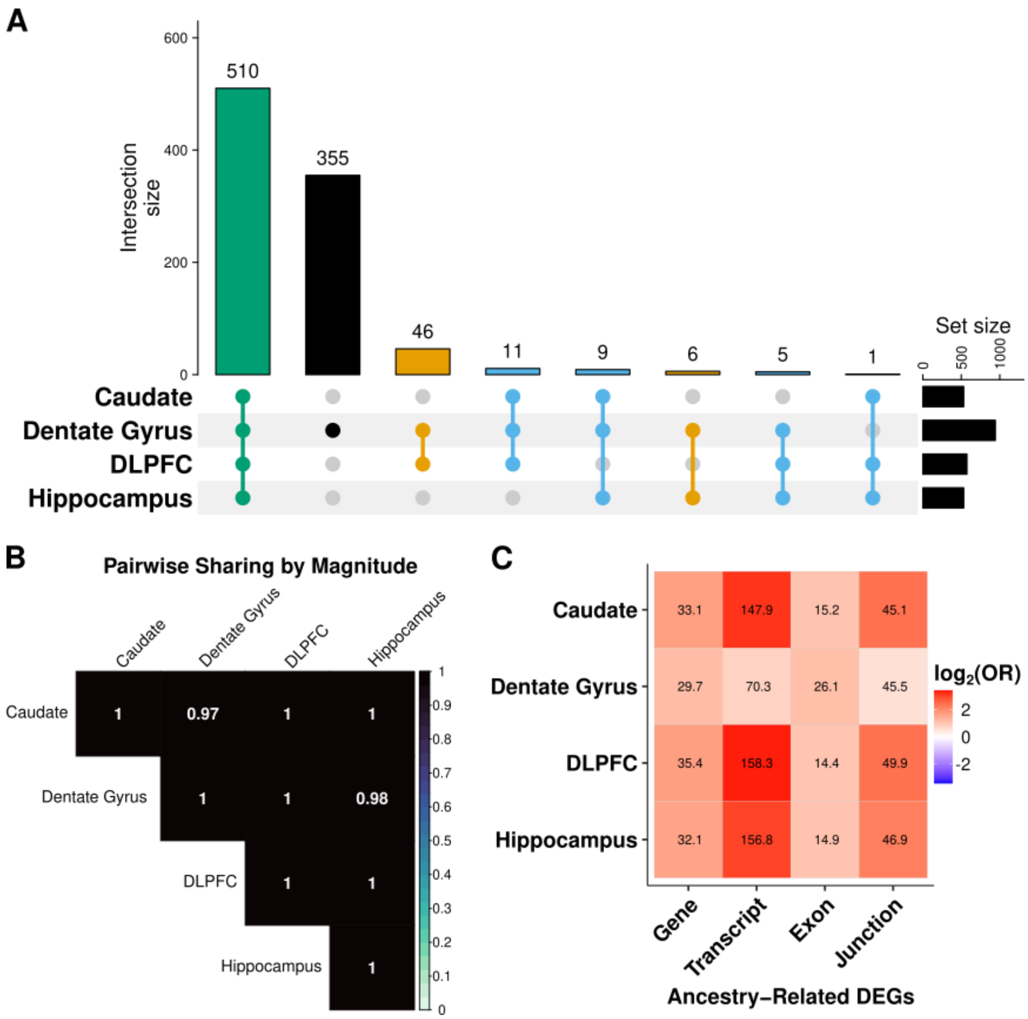

Fig. S23: **Ancestry-dependent eQTL shared across brain regions and enriched for main effect eQTL. A.** UpSet plot showing sharing of eGenes across brain regions. **B.** Heatmap showing significant sign matched. **C.** Enrichment heatmap of ancestry-dependent eGenes with ancestry-associated DEGs; significant enrichments (two-sided, Fisher's exact test with FDR corrected p-values $-\log_{10}$ transformed) annotated within tiles.

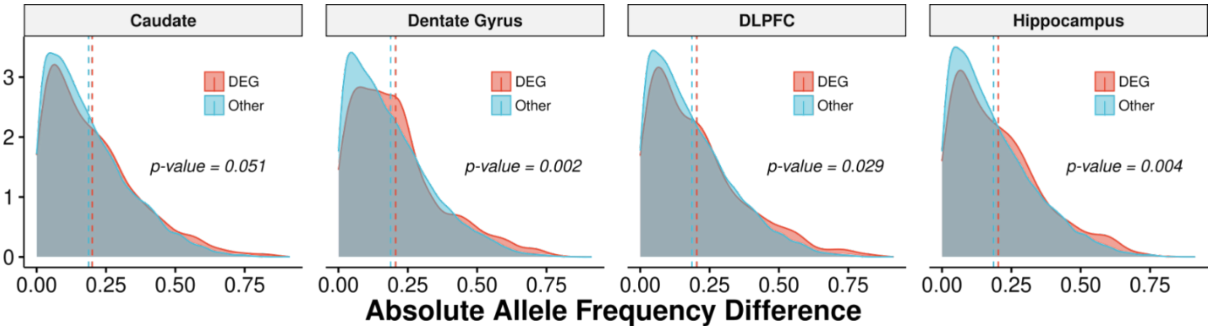

Fig. S24: Significant increase of absolute allele frequency difference for ancestry-associated DEGs compared with non-DEGs. Density plot showing a significant increase in absolute allele frequency differences (AFD; one-sided, Mann-Whitney U, p-value < 0.05) for global ancestry-associated DEGs (red) compared with non-DEGs (blue) across brain regions. A dashed line marks the mean absolute AFD. Absolute AFD is calculated from the most significant SNP per gene.

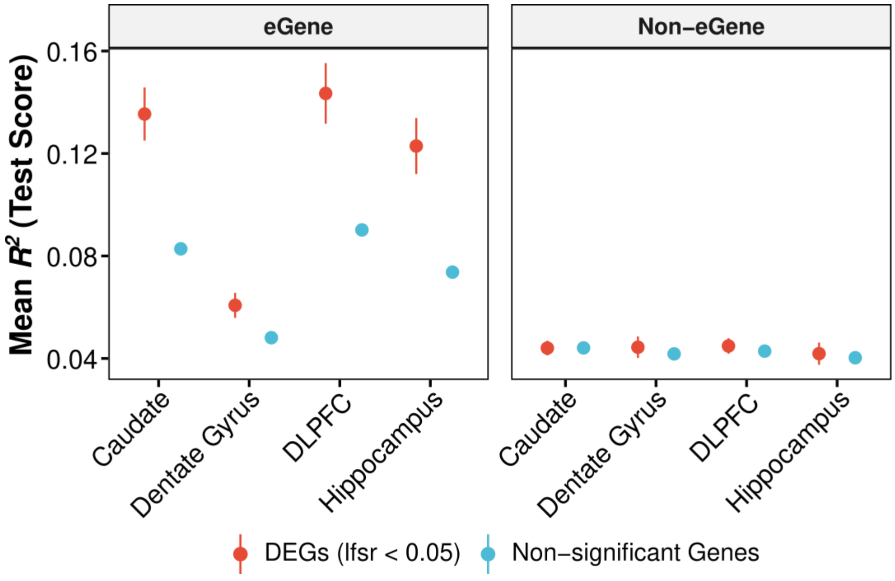

Fig. S25: Summary of test R2 for elastic net model separated by genes with an eQTL and those without. The mean R^2 of eGenes are plotted on the left for the caudate (n=760 DEGs and 8,080 non-significant genes), dentate gyrus (n=605 DEGs and 8,963 non-significant genes), DLPFC (n=652 DEGs and 8,448 non-significant genes), and hippocampus (n=589 DEGs and 7,839 non-significant genes). Similarly, the mean R^2 of non-eGenes are plotted for the caudate (n=464 DEGs and 7,286 non-significant genes), dentate gyrus (n=362 DEGs and 6,644 non-significant genes), DLPFC (n=371 DEGs and 7,139 non-significant genes), and hippocampus (n=404 DEGs and 7,821 non-significant genes). Error bars correspond to 95% confidence intervals. eGenes refer to unique genes associated with an eQTL, while DEGs denote global ancestry-associated differential expressed genes.

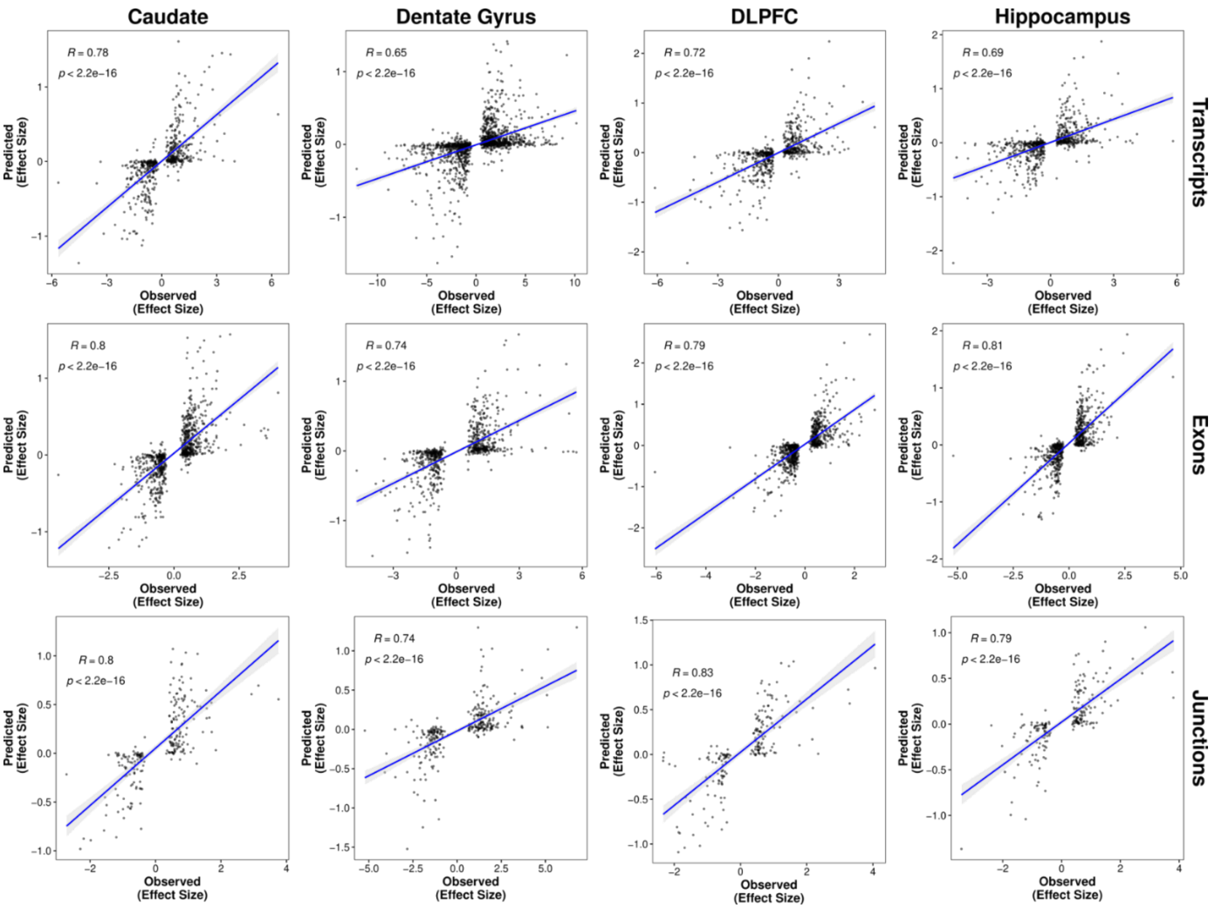

Fig. S26: Elastic net model captures more genetic contribution of genetic ancestry-associated expression changes in the brain on the isoform level. Correlation (two-sided, Spearman) between elastic net predicted (y-axis) and observed (x-axis) global ancestry-associated differences in expression for ancestry-associated DE features (i.e., transcripts, exons, and junctions) with an eQTL across brain regions. Exact p-values: transcripts (caudate: 4.7e-112, dentate gyrus: 8.9e-163, DLPFC: 6.0e-99, and hippocampus: 1.2e-93), exons (caudate: 6.4e-175, dentate gyrus: 1.9e-118, DLPFC: 1.4e-166, and hippocampus: 8.6e-156), and junctions (caudate: 4.5e-51, dentate gyrus: 4.2e-52, DLPFC: 1.6e-50, and hippocampus: 2.3e-44). A fitted trend line is presented in blue as the mean values +/- standard deviation. The standard deviation is shaded in light gray.

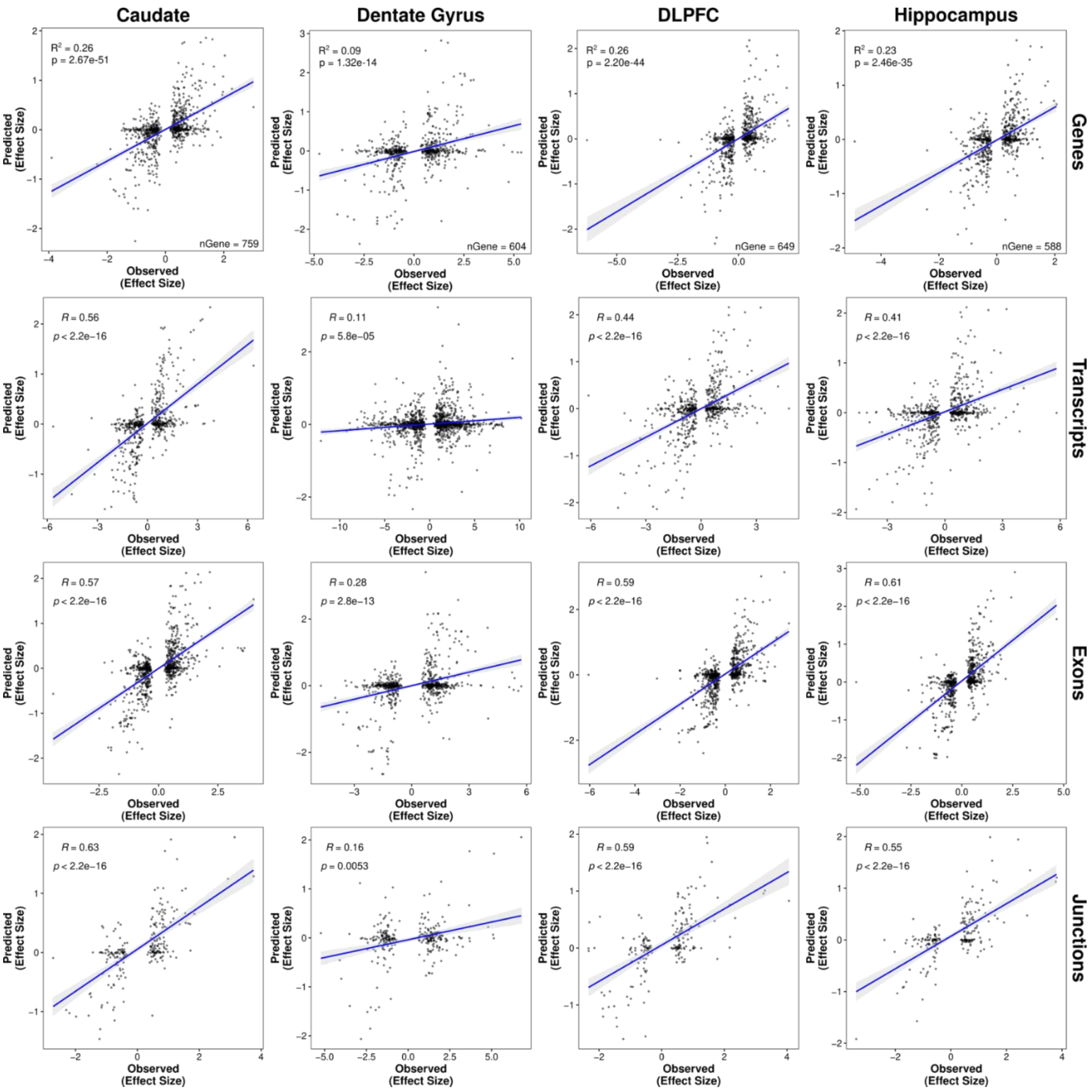

Fig. S27: The most significant eQTL explains roughly 20% of genetic ancestry expression differences in the brain. Correlation (two-sided, Spearman) of cis-predicted (y-axis) versus observed (x-axis) global ancestry-associated differences in expression among ancestry-associated DE features (i.e., gene, transcript, exon, and junction) with an eQTL across brain regions. Exact p-values: the caudate (genes: 2.7e-51, transcripts: 2.5e-45, exons: 2.0e-69, and junctions: 2.2e-26), dentate gyrus (genes: 1.3e-14, transcripts: 5.8e-05, exons: 2.2e-13, and junctions: 5.3e-03), DLPFC (genes: 2.2e-44, transcripts: 6.4e-31, exons: 1.2e-73, and junctions: 7.5e-20), and hippocampus (genes: 2.4e-35, transcripts: 3.1e-27, exons: 3.2e-70, and junctions: 2.0e-17). A fitted trend line is presented in blue as the mean values +/- standard deviation. The standard deviation is shaded in light gray.

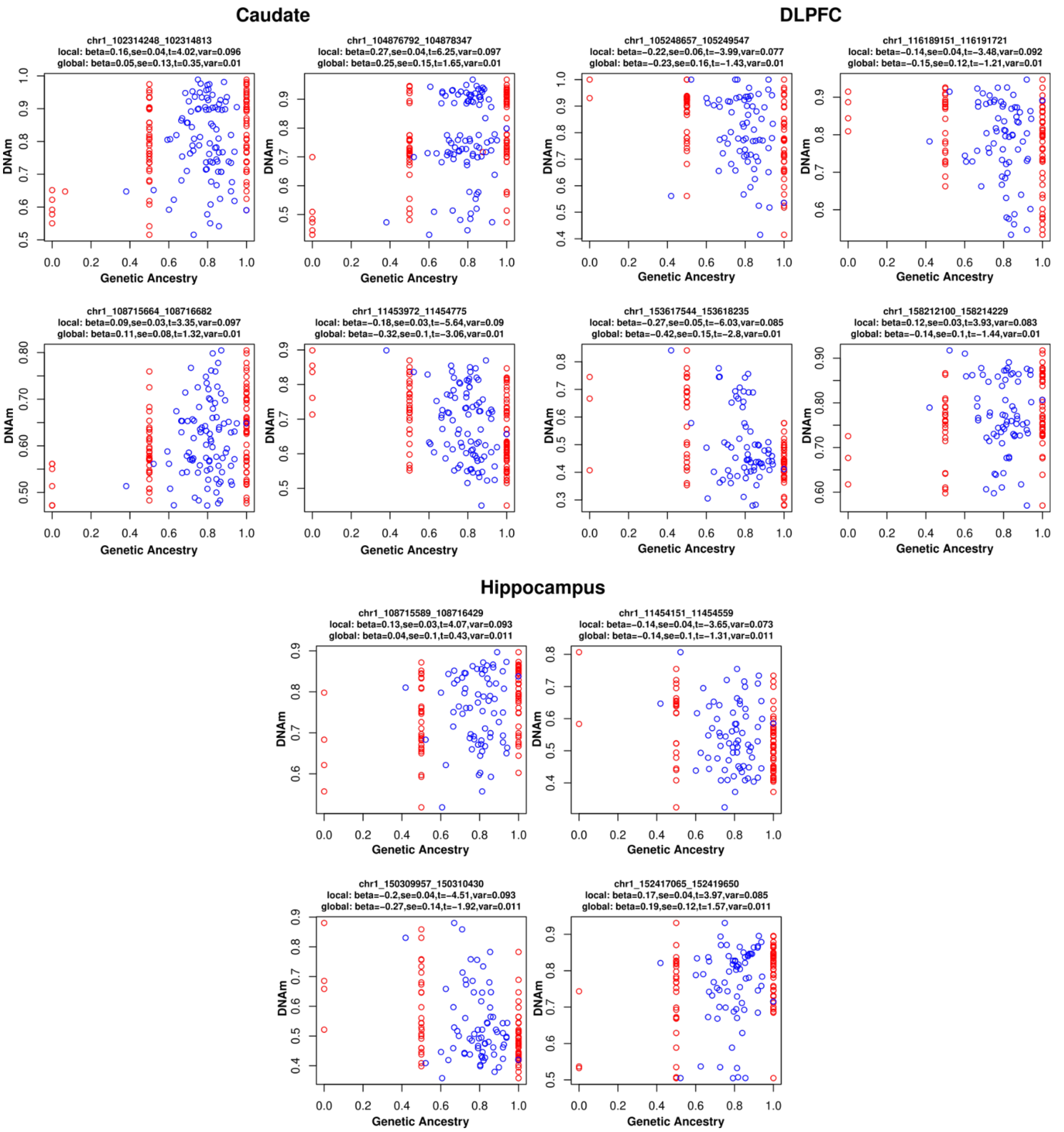

Fig. S28: Local ancestry is more variable than global ancestry. Example scatter plots showing local (red) and global (blue) ancestry associated with DNAm across brain regions. DMR test results (effect size [beta], standard error [se], and variance [var]) annotated on top of each example VMR.

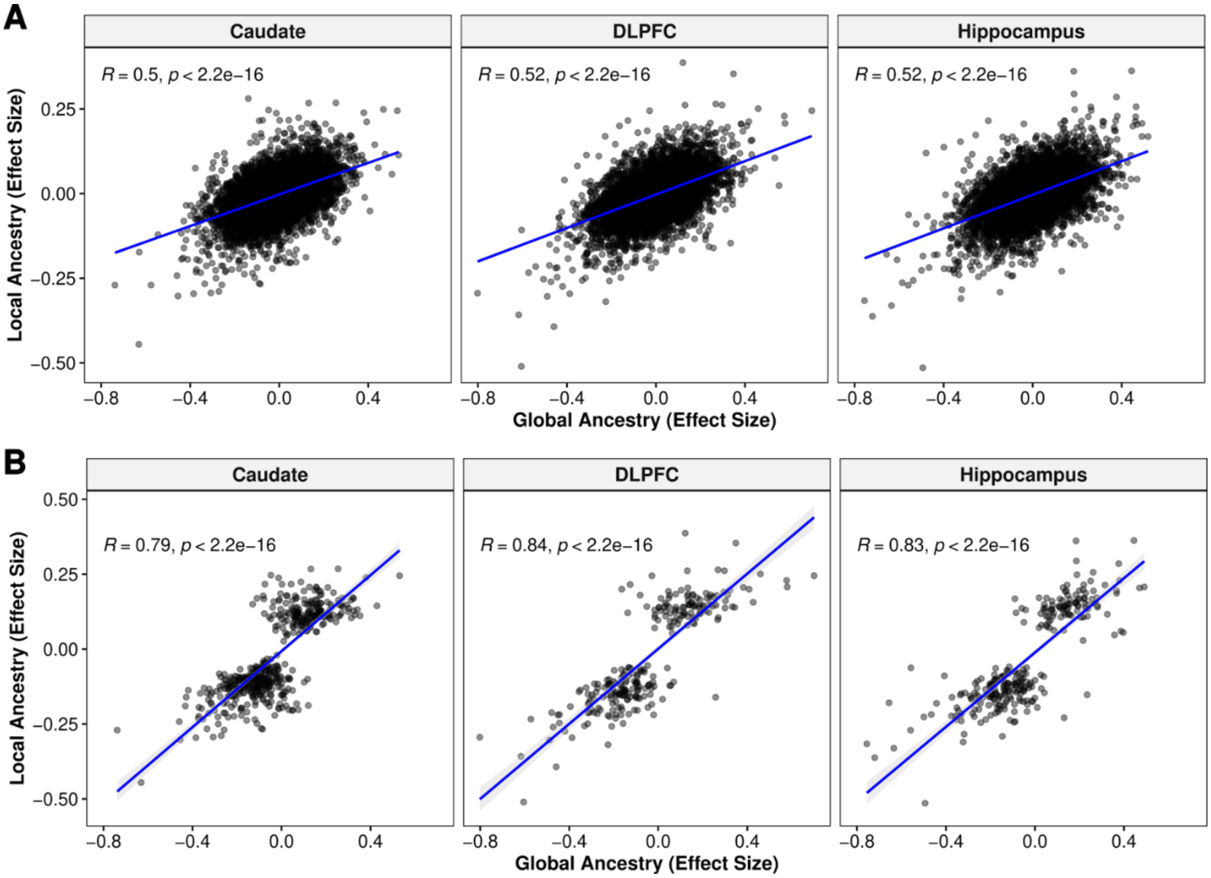

Fig. S29: Significant correlation of DNAm levels between local and global ancestry-associated DMRs across brain regions. Scatter plot showing correlation (two-sided, Spearman) between global (x-axis) and local (y-axis) for **A.** all ancestry-associated DMRs (p-values ~ 0 for the caudate, DLPFC, and hippocampus) and **B.** significant ancestry-associated DMRs (p-values = 1.1e-90, 5.6e-65, and 6.1e-63 for the caudate, DLPFC, and hippocampus, respectively). A fitted trend line is presented in blue as the mean values +/- standard deviation. The standard deviation is shaded in light gray.

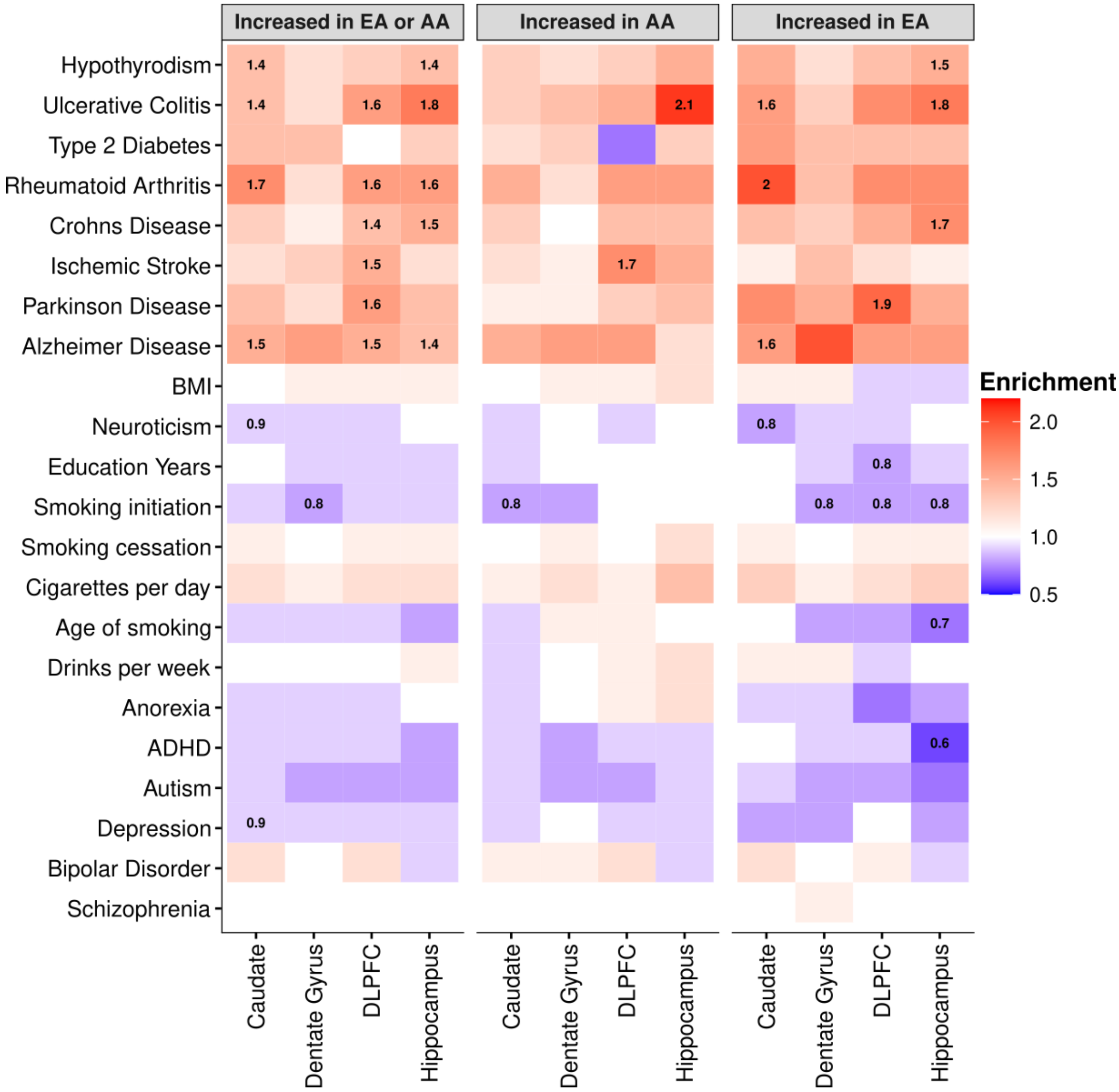

Fig. S30: **Global ancestry-associated DEGs show general enrichment for heritability of neurological and immune-related traits.** Heatmap for ancestry-associated DEGs that show no enrichment (red) nor depletion (blue) for heritability of brain- and immune-related traits from S-LDSC analysis. Numbers within tiles are levels of enrichment (> 1) or depletion (< 1) that are significant after multiple testing correction (FDR < 0.05). The left panel shows results for all DEG in each brain region. The middle and right panels show results for DEG increased with AA or EA proportions for each brain region, respectively.

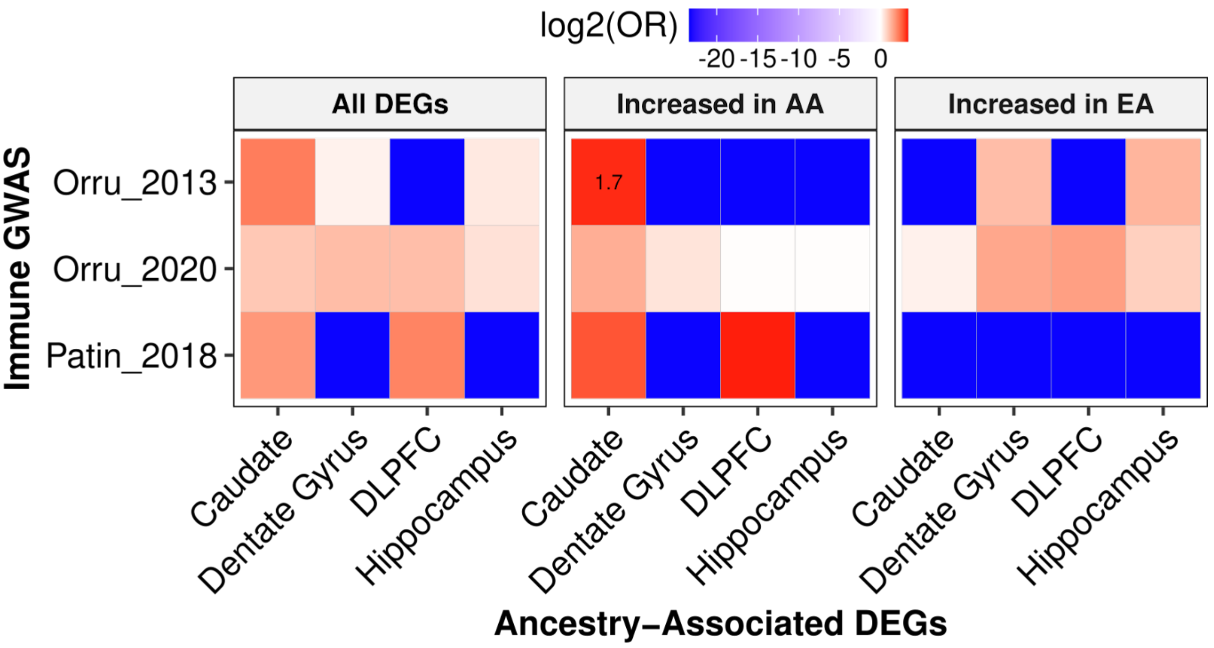

Fig. S31: Limited enrichment of non-brain immune function GWAS prioritized genes with global ancestry-associated DEGs. Heatmap showing enrichment analysis (two-sided, Fisher's exact test with p-values corrected for multiple testing with Benjamini-Hochberg) of significantly enriched (red) or depleted (blue) immune function GWAS prioritized genes for ancestry-associated DEGs (lfsr < 0.05) separated by direction of effect. Significant enrichments (-log10 transformed) annotated within tiles.

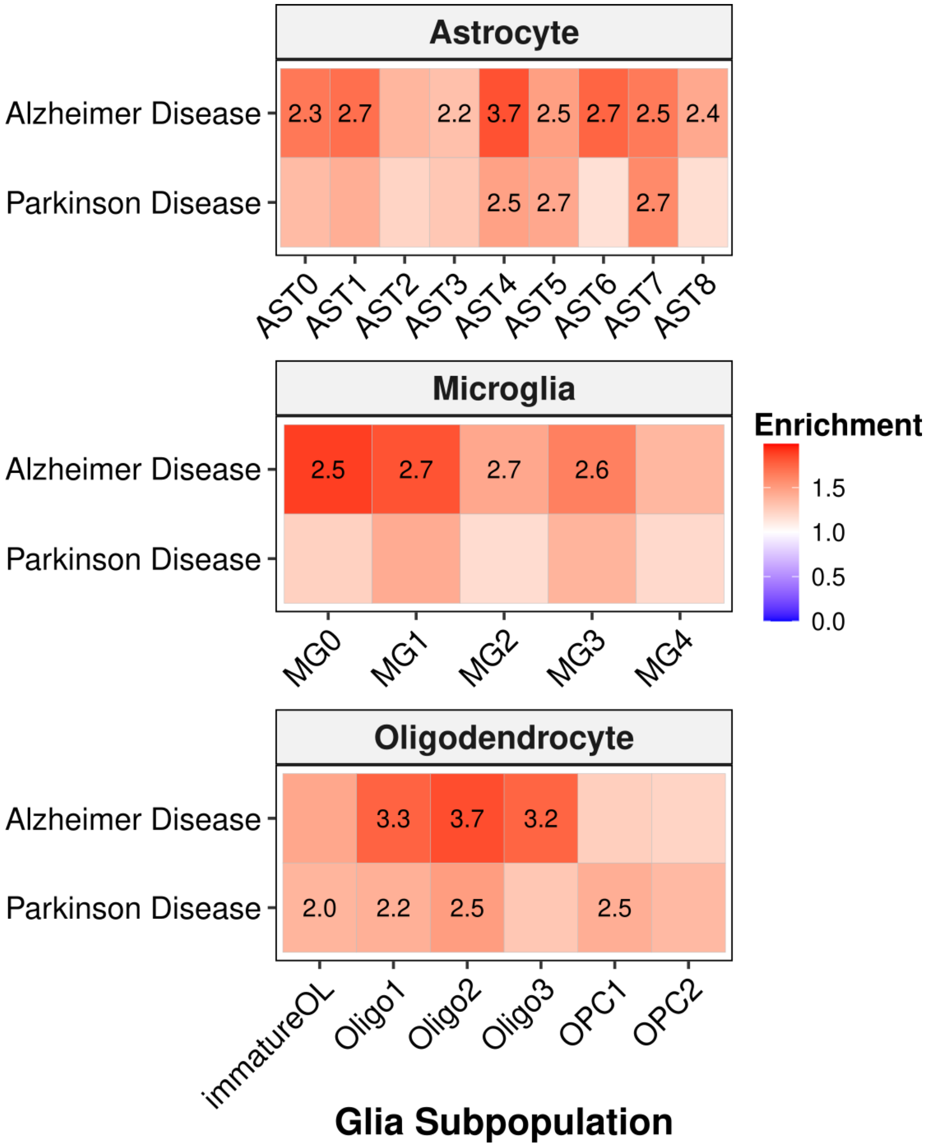

Fig. S32: Ancestry-associated glial cell subpopulations show general enrichment for heritability of Alzheimer's and Parkinson's diseases. Heatmap for glial cell subpopulations that show enrichment (red) for heritability of Alzheimer's and Parkinson's diseases from S-LDSC analysis. Numbers within tiles are levels of enrichment (> 1) or depletion (< 1) that are significant after multiple testing correction (FDR < 0.01).

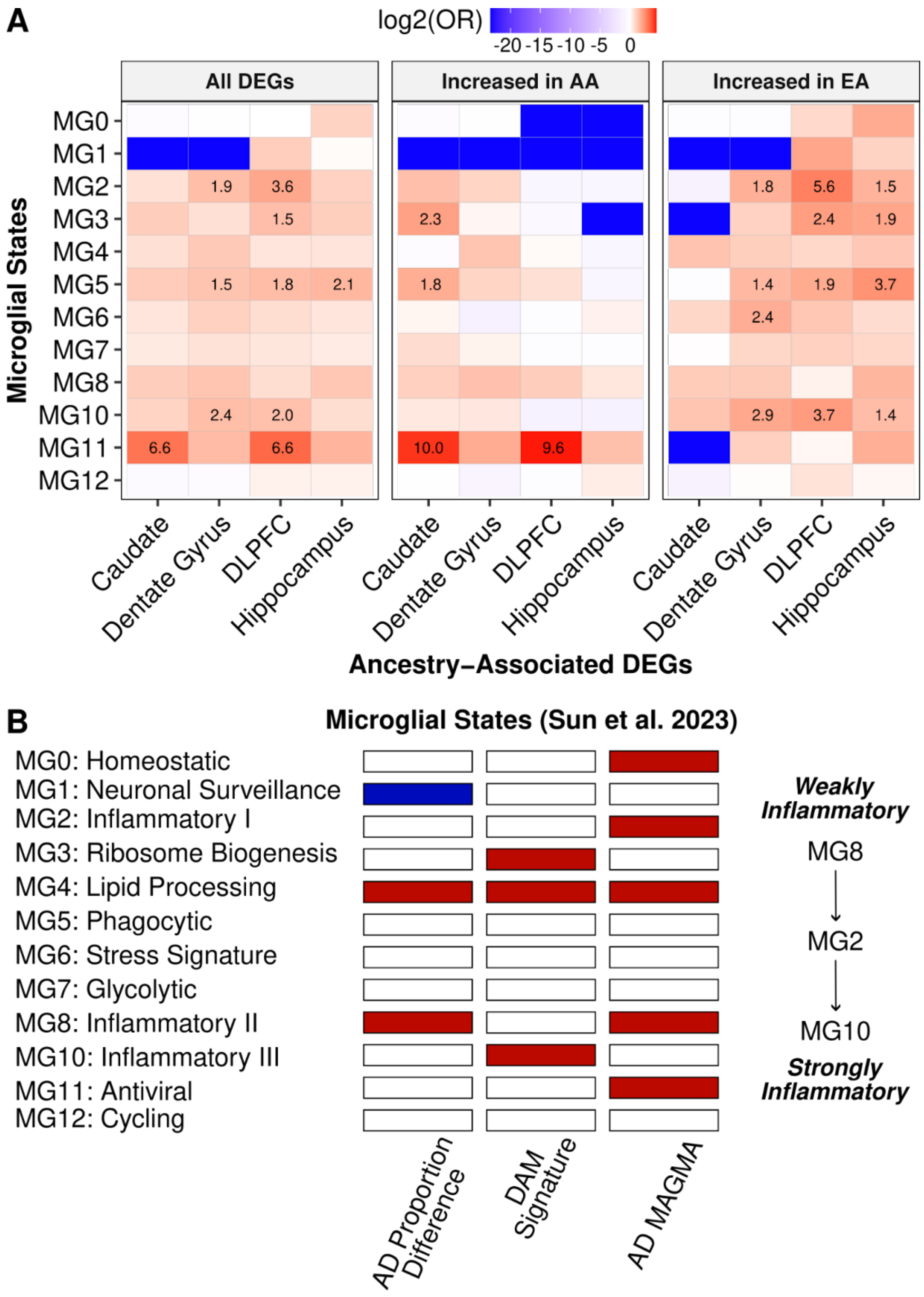

Fig. S33: Significant enrichment of ancestry-associated DEGs for activated microglia and disease-associated microglia. A. Heatmap showing enrichment analysis (two-sided, Fisher's exact test) of significantly enriched (red) or depleted (blue) microglia states (*40*) for ancestry-associated DEGs (lfsr < 0.05) separated by direction of effect. Significant enrichments (-$\log_{10}$ transformed) annotated within tiles. **B.** Annotation of microglia states (*40*) for Alzheimer's disease (AD): significant cell proportion differences, disease-associated microglia [DAM] signature in mouse models, and genetic association via MAGMA enrichment. Depletion is annotated in blue and enrichment in red.

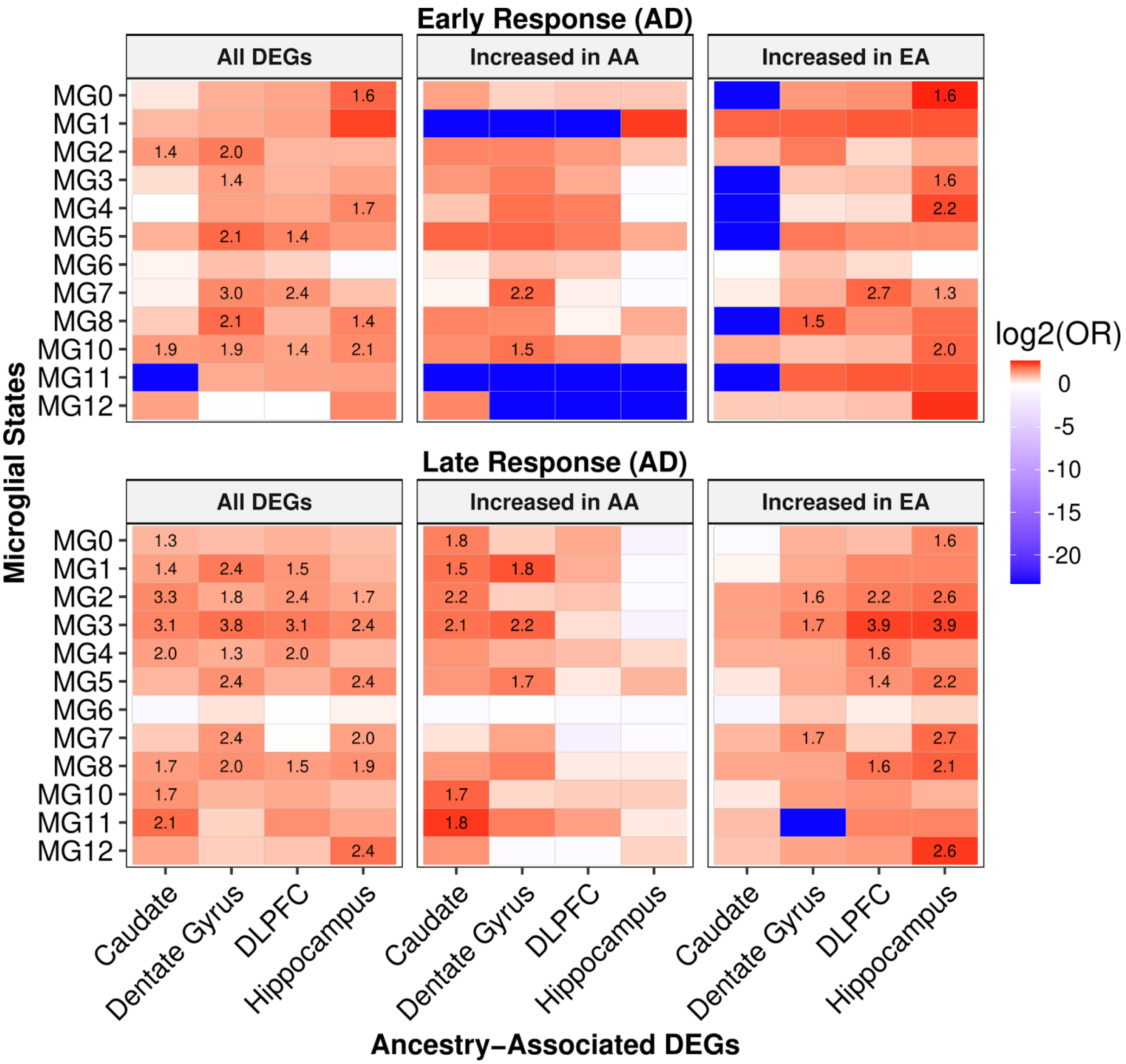

Fig. S34: Ancestry-associated DEGs are primarily enriched for microglial states associated with late-response Alzheimer's disease-related DEGs. Heatmap showing enrichment analysis (two-sided, Fisher's exact test) of ancestry DEGs (lfsr < 0.05) with cell type-specific Alzheimer's disease (AD) DEGs (*40*) separated response stage for ancestry-associated DEGs (lfsr < 0.05) separated by direction of effect. Early response is Alzheimer's DEGs detected between neurotypical control and early Alzhiemer's individual. Late response is Alzheimer's DEGs detected between early and late Alzheimer's individuals. Alzheimer's stage defined in (*40*). Significantly enriched (red) or depleted (blue) tiles are annotated with -$\log_{10}$(FDR).

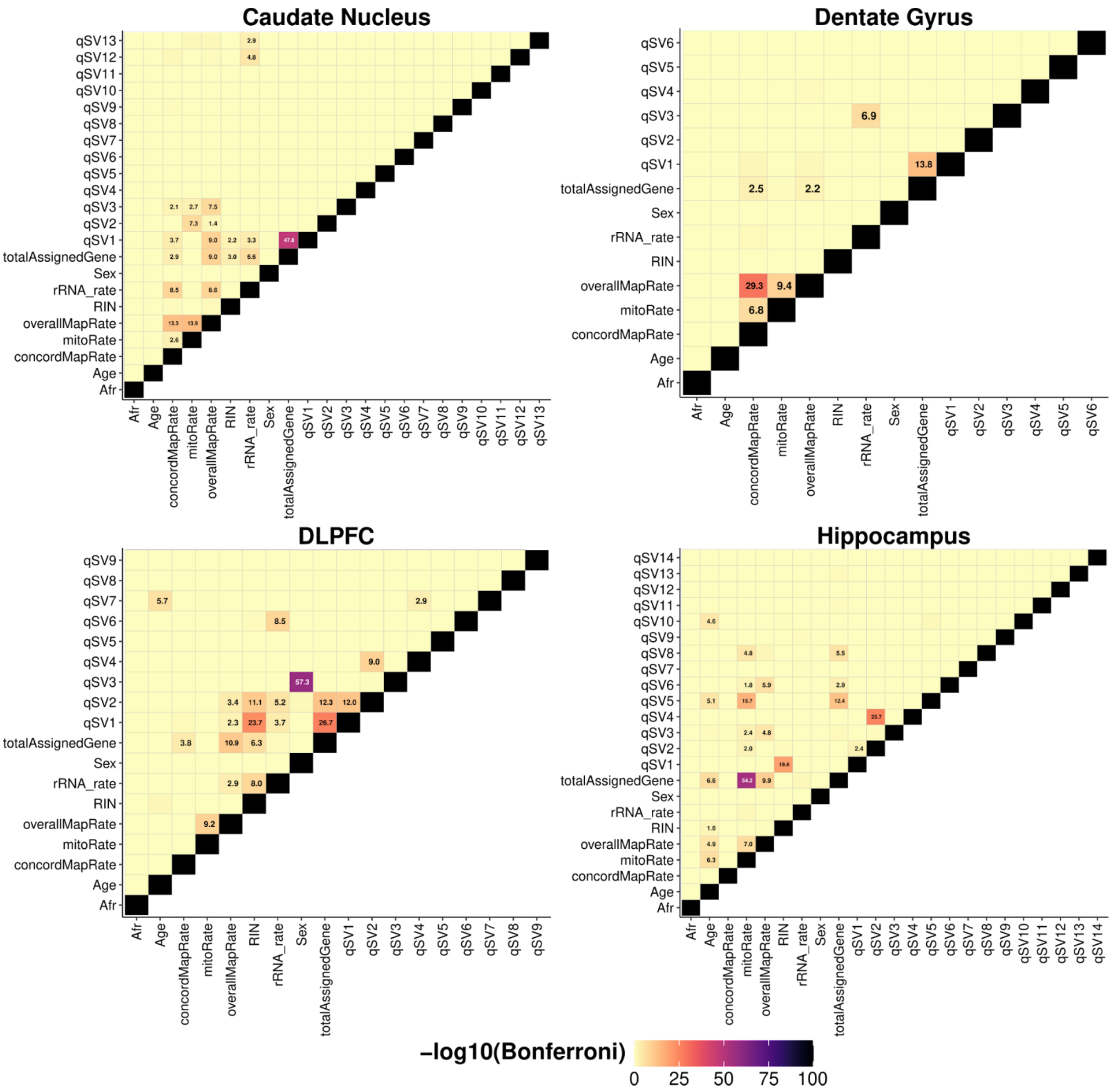

Fig. S35: Limited correlation between covariates across brain regions. Heatmap showing correlation between covariates across brain regions (linear regression, Bonferroni corrected p-values). Significant correlations (-$\log_{10}$ transformed) are denoted in each tile.

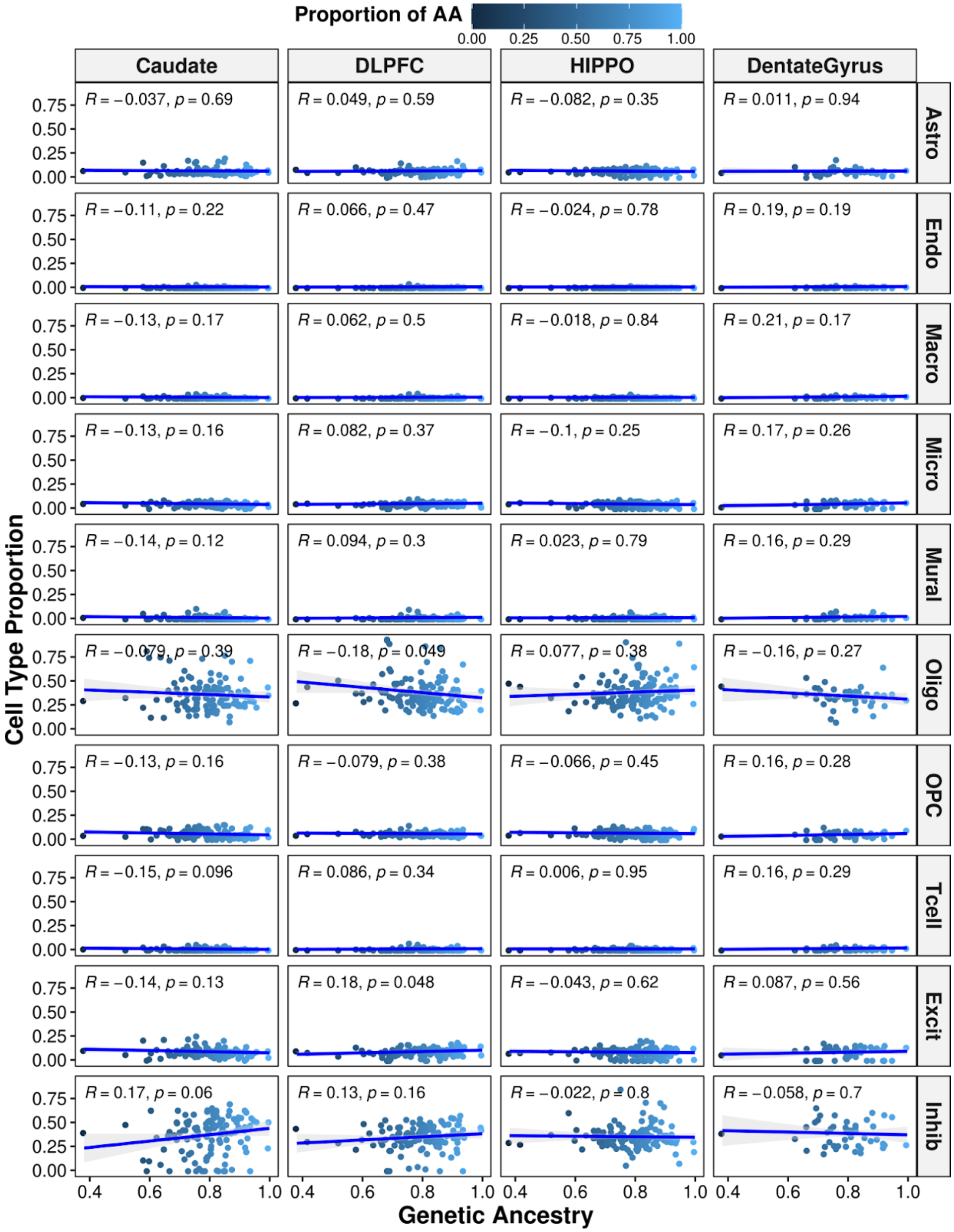

Fig. S36: Cell-type proportions show no correlation with genetic ancestry in Black American donors across brain regions. Scatter plot showing no correlation (two-sided, Spearman) between genetic ancestry and cell-type proportion across the brain. A fitted trend line is presented in blue as the mean values +/- standard deviation. The standard deviation is shaded in light gray. Astro: astrocytes. Endo: endothelial. Micro: microglia. Macro: macrophage. Mural: mural cells. Oligo: oligodendrocytes. OPC: oligodendrocyte progenitor cells. Tcell: T cells. Excit: excitatory neurons. Inhib: inhibitory neurons.

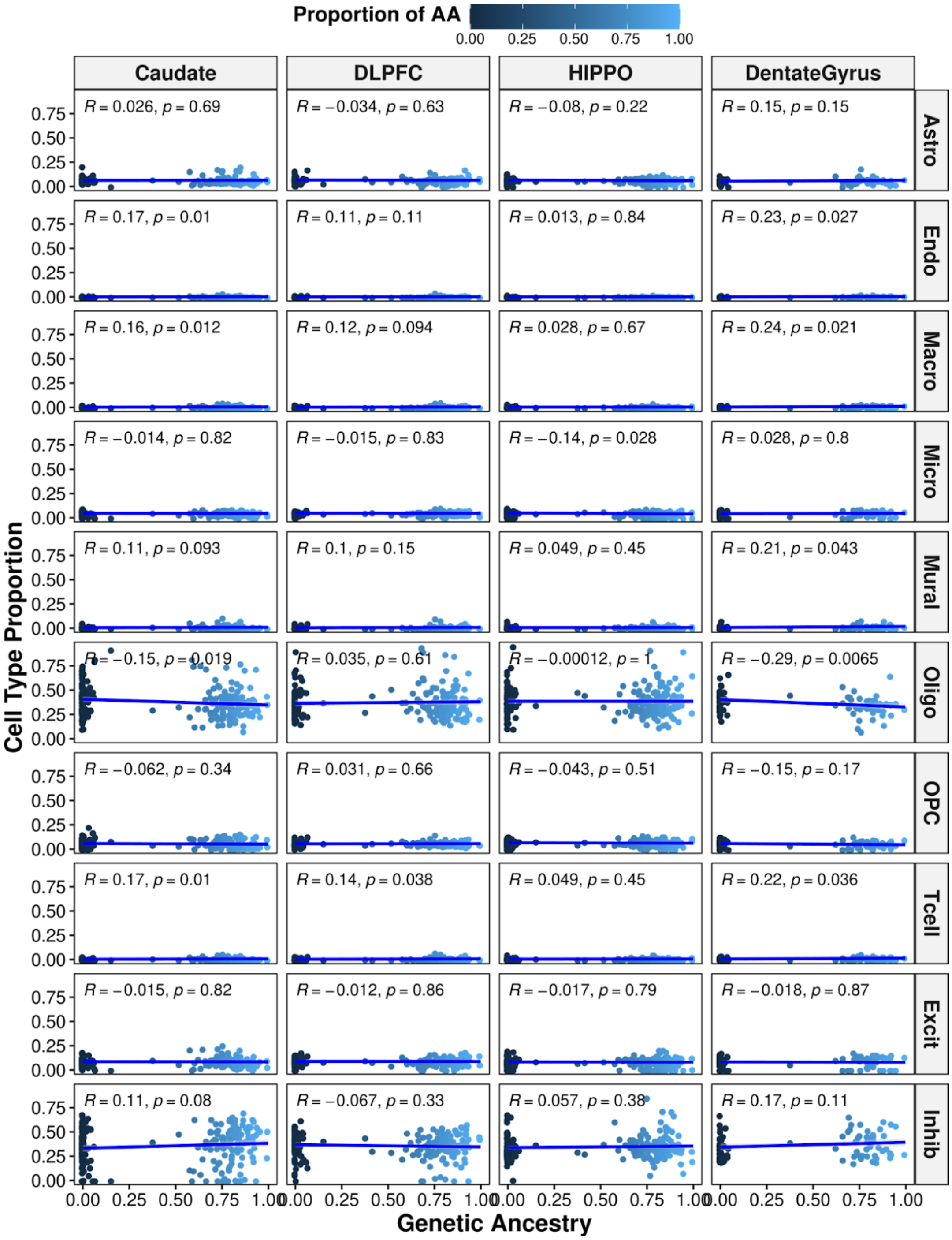

Fig. S37: Significant correlation of several cell-type proportions with genetic ancestry in Black American and White American donors across brain regions. Scatter plot showing correlation (two-sided, Spearman) between genetic ancestry and cell-type proportion across the brain. A fitted trend line is presented in blue as the mean values +/- standard deviation. The standard deviation is shaded in light gray. Astro: astrocytes. Endo: endothelial. Micro: microglia. Macro: macrophage. Mural: mural cells. Oligo: oligodendrocytes. OPC: oligodendrocyte progenitor cells. Tcell: T cells. Excit: excitatory neurons. Inhib: inhibitory neurons.

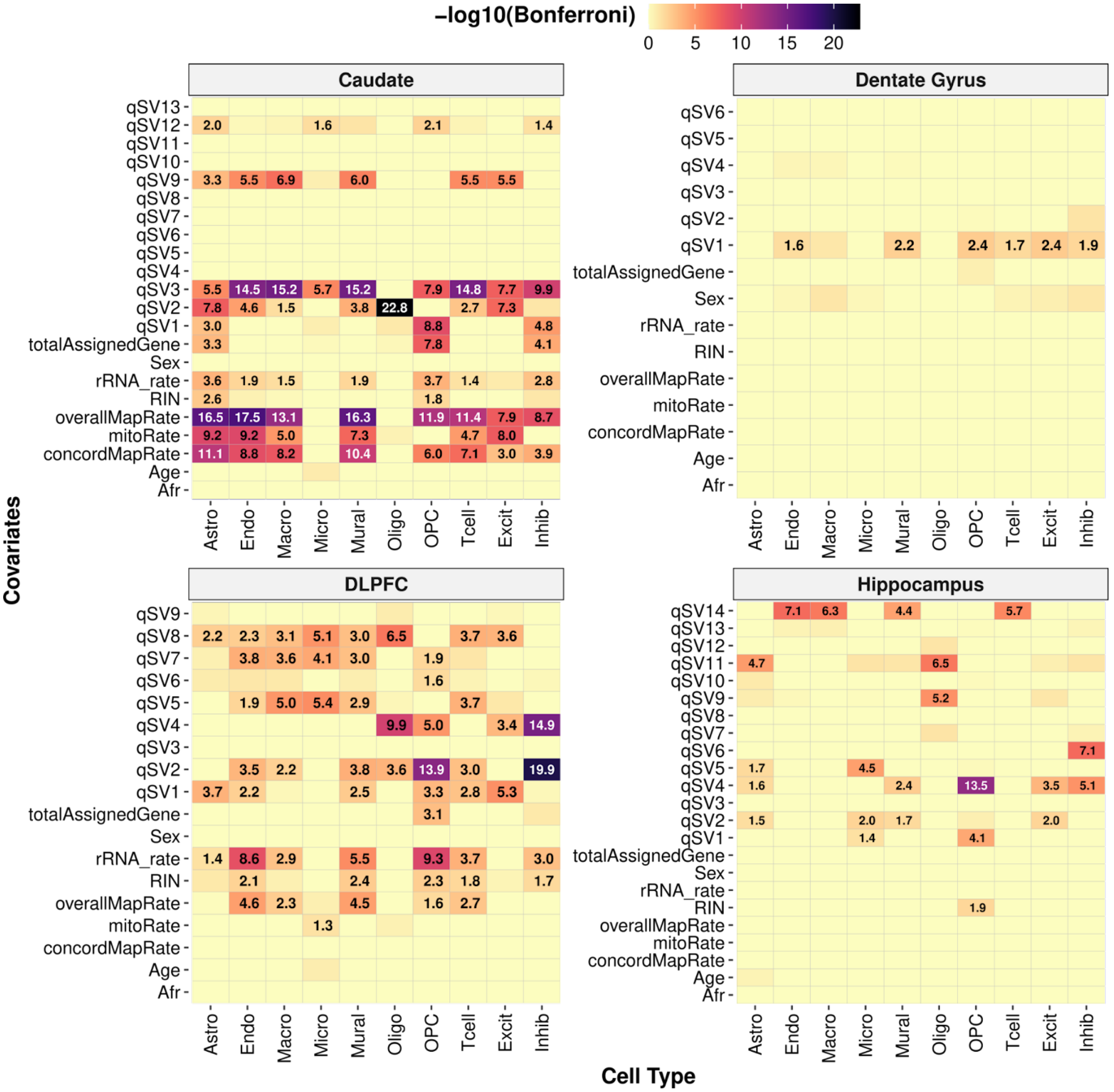

Fig. S38: Significant correlation between cell-type proportion and model covariations. Heatmap of correlation between covariates and cell-type proportion across the brain (linear regression, Bonferroni corrected p-values). Significant correlations (-$\log_{10}$ transformed) are denoted in each tile. Astro: astrocytes. Endo: endothelial. Micro: microglia. Macro: macrophage. Mural: mural cells. Oligo: oligodendrocytes. OPC: oligodendrocyte progenitor cells. Tcell: T cells. Excit: excitatory neurons. Inhib: inhibitory neurons.

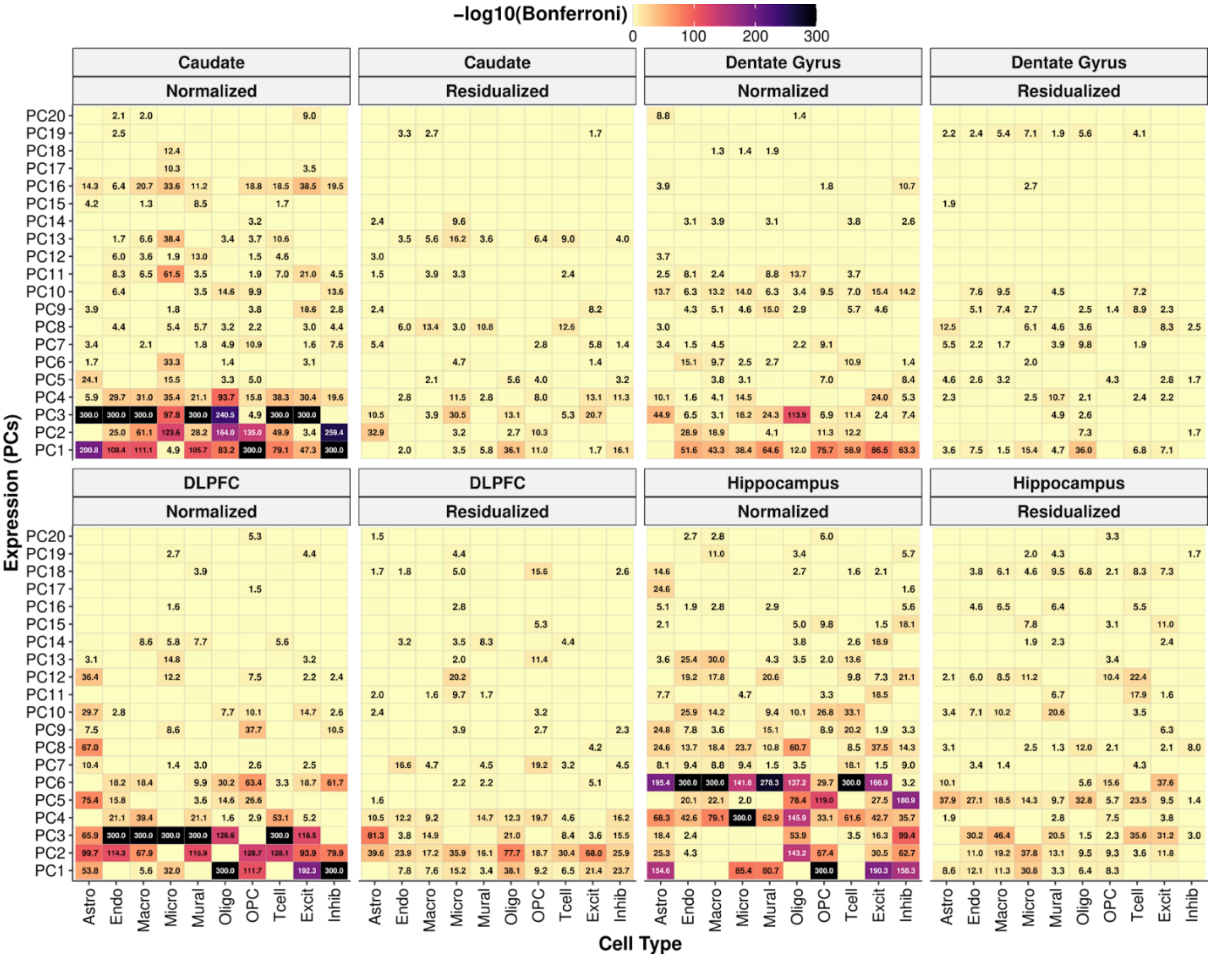

Fig. S39: Cell-type proportion partially corrected for by covariates. Heatmap of correlation between PCA of gene expression and cell-type proportions before (normalized) and after (residualized) adjusting for covariates including qSVs across brain regions (linear regression, Bonferroni corrected p-values). Significant correlations (-$\log_{10}$ transformed) are denoted in each tile. Astro: astrocytes. Endo: endothelial. Micro: microglia. Macro: macrophage. Mural: mural cells. Oligo: oligodendrocytes. OPC: oligodendrocyte progenitor cells. Tcell: T cells. Excit: excitatory neurons. Inhib: inhibitory neurons.

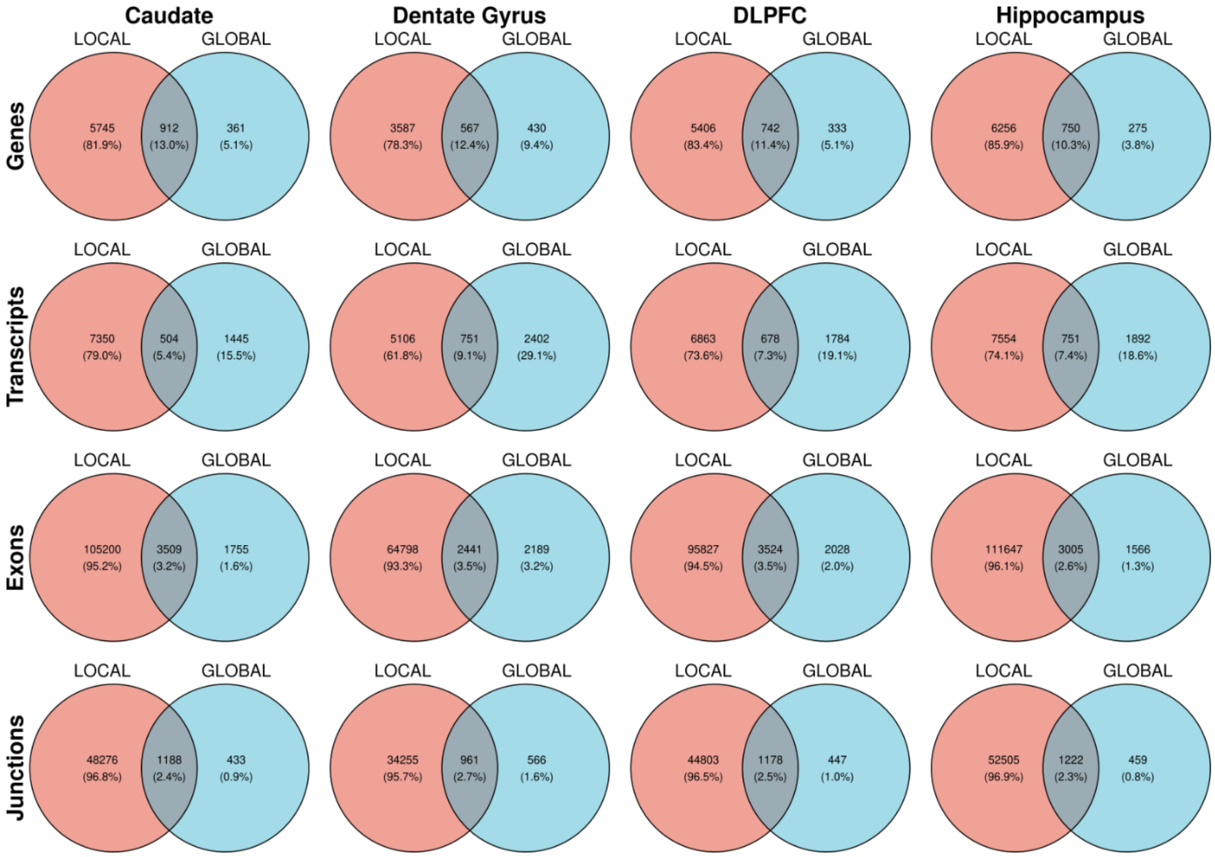

Fig. S40: Significant overlap of global ancestry-associated DE features with local ancestry analysis. Venn diagram showing the overlap between local ancestry-associated DE features (i.e., gene, transcript, exon, and junction) and global ancestry-associated DE features across brain regions. Significant overlap tested with two-sided, Fisher's exact test (FDR < 0.05).

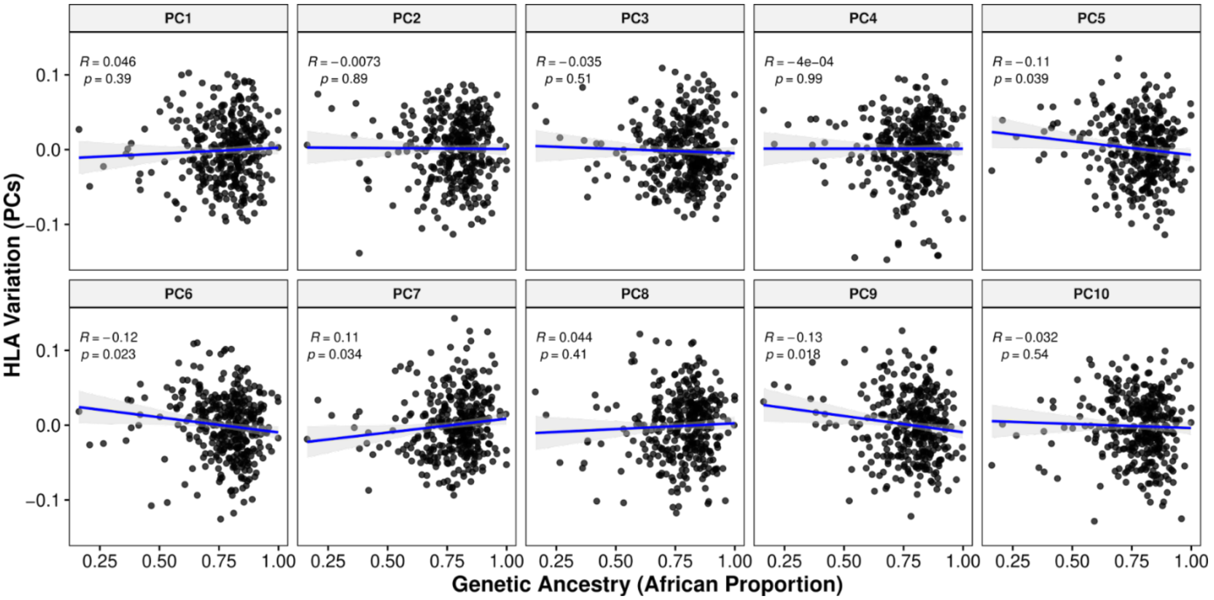

Fig. S41: Little to no correlation between HLA variation PCs and global genetic ancestry. Scatter plot showing correlation (two-sided, Spearman) between genetic ancestry and HLA variation PCs. A fitted trend line is presented in blue as the mean values +/- standard deviation. The standard deviation is shaded in light gray.

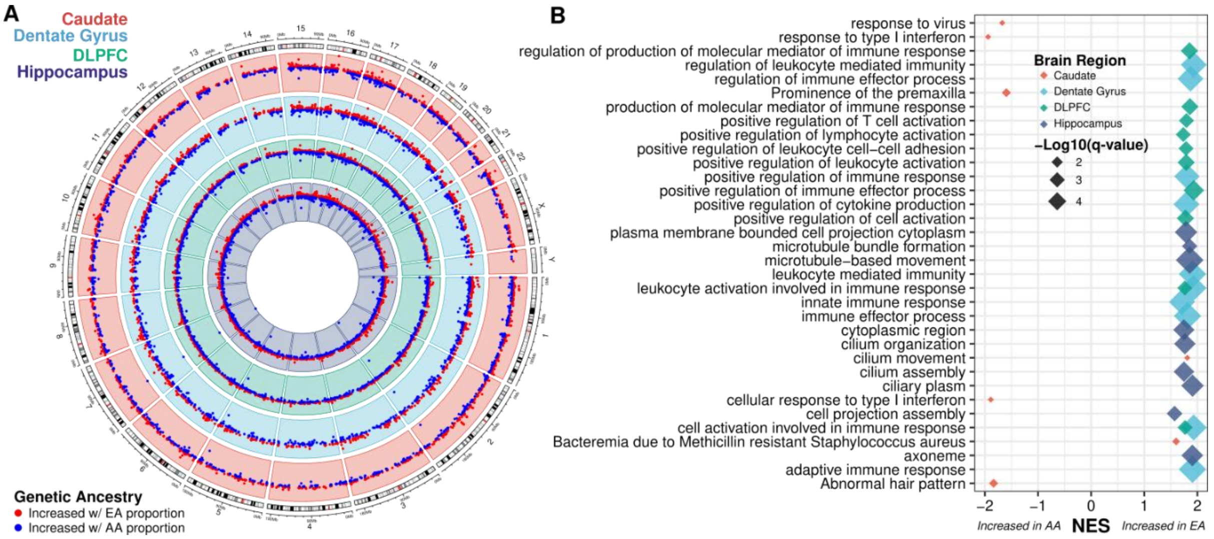

Fig. S42: Extensive ancestry-associated expression changes across the brain highlighting impact of environment. A. Circos plot showing ancestry DEGs from binary internal replication analysis across the caudate (red), dentate gyrus (blue), DLPFC (green), and hippocampus (purple). **B.** Gene set enrichment analysis of differential expression analysis across brain regions, highlighting terms associated with increased AA (African ancestry) or EA (European ancestry) proportions.

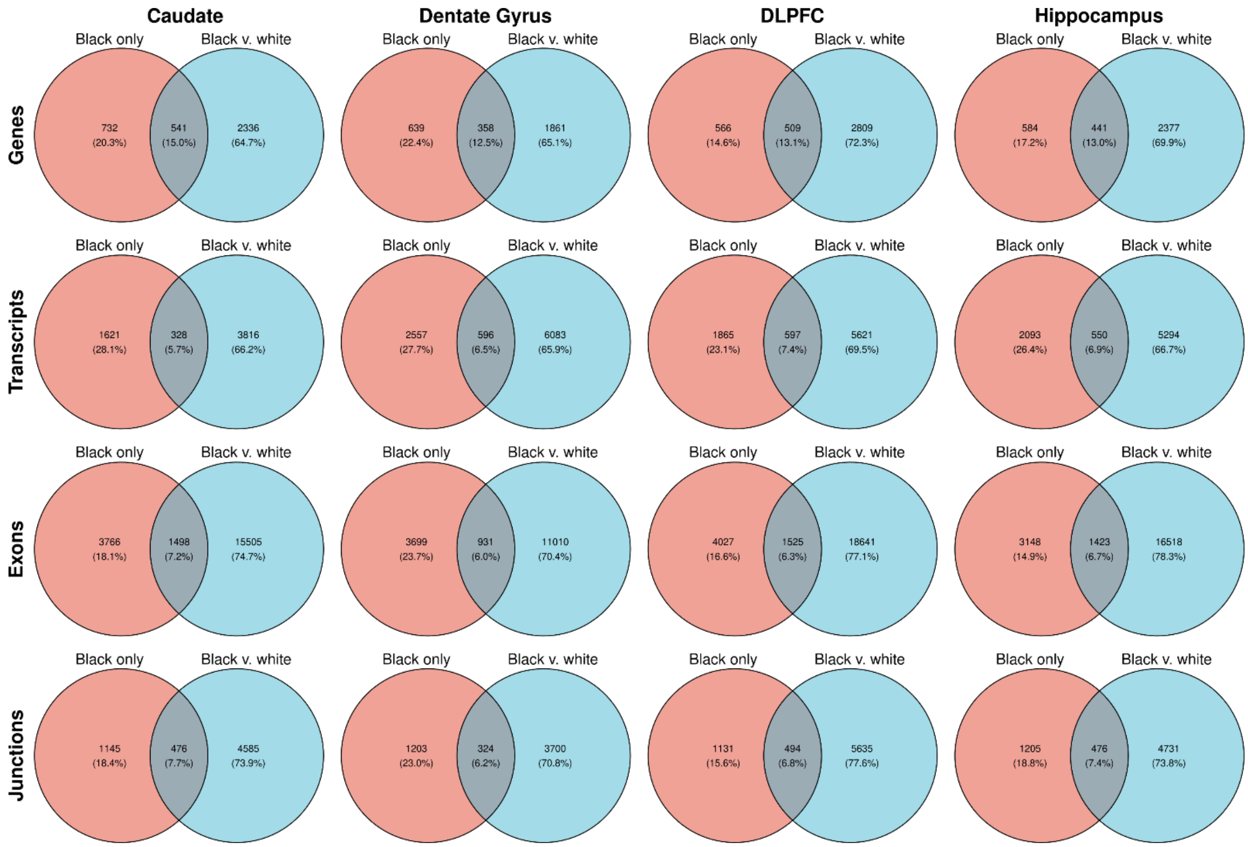

Fig. S43: Black versus non-Hispanic white American binary analysis potentially confounded by environmental factors. Venn diagram showing the overlap of global ancestry-associated DE features (i.e., gene, transcript, exon, and junction) between within Black and other (Black v. white Americans) DE analysis across brain regions.

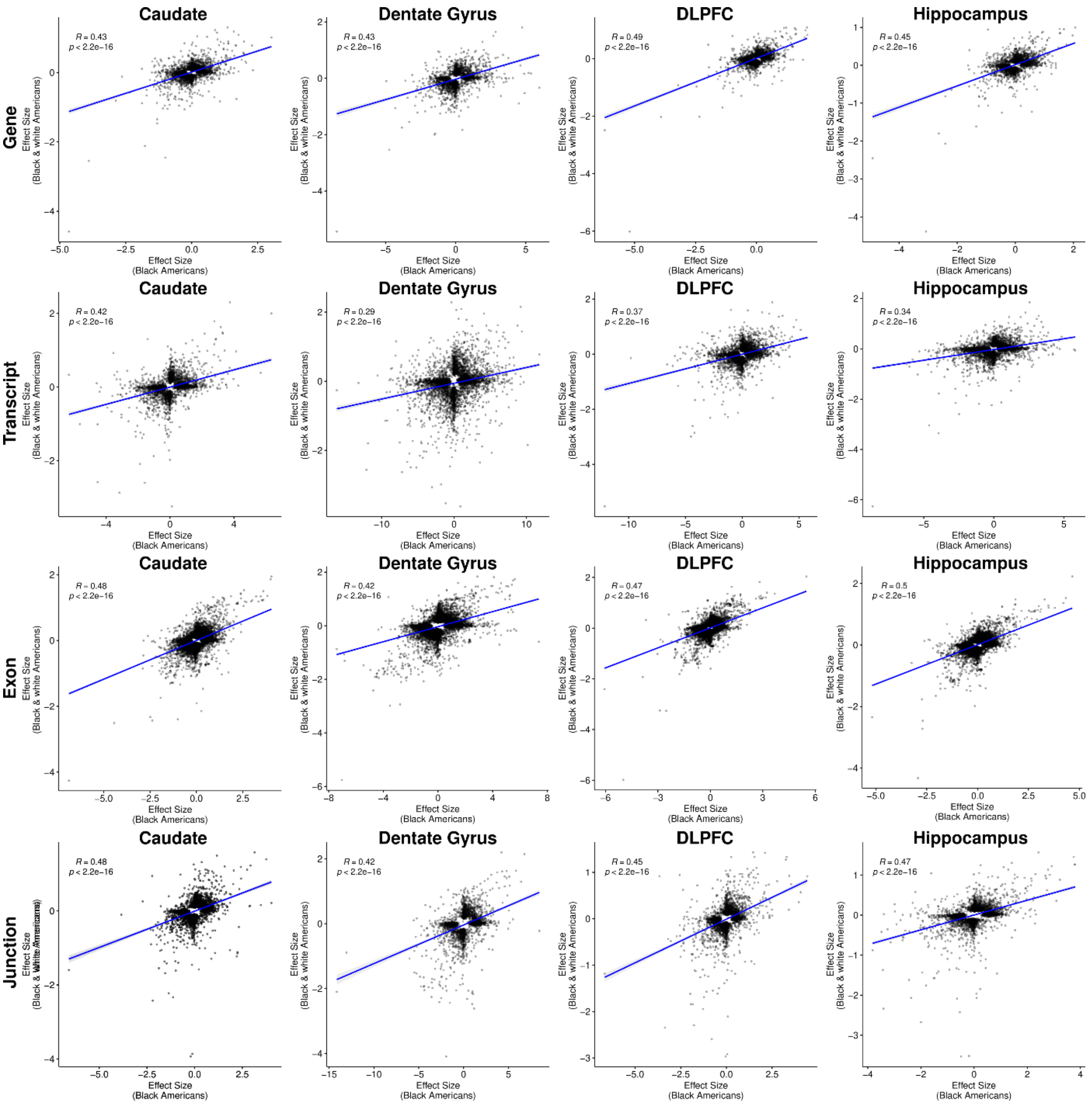

Fig. S44: Significant correlation of effect sizes between genetic ancestry association in admixed Black American individuals and self-reported race between Black and non-Hispanic white Americans. Scatter plot showing significant correlation (two-sided, Spearman correlation) between Black American-only analysis (x-axis) and combined analysis (Black and white Americans; y-axis) for each brain region and feature (i.e., gene, transcript, exon, and junction). Exact p-values: the caudate (genes: 7.0e-129, transcripts: 9.8e-131, exons: ~0, and junctions: 1.2e-215), dentate gyrus (genes: 1.3e-97, transcripts: 3.7e-87, exons: ~0, and junctions: 3.3e-122), DLPFC (genes: 1.7e-182, transcripts: 1.6e-124, exons: ~0, and junctions: 2.3e-209), and hippocampus (genes: 3.6e-134, transcripts: 1.9e-107, exons: ~0, and junctions: 4.4e-205). A fitted trend line is presented in blue as the mean values +/- standard deviation. The standard deviation is shaded in light gray.

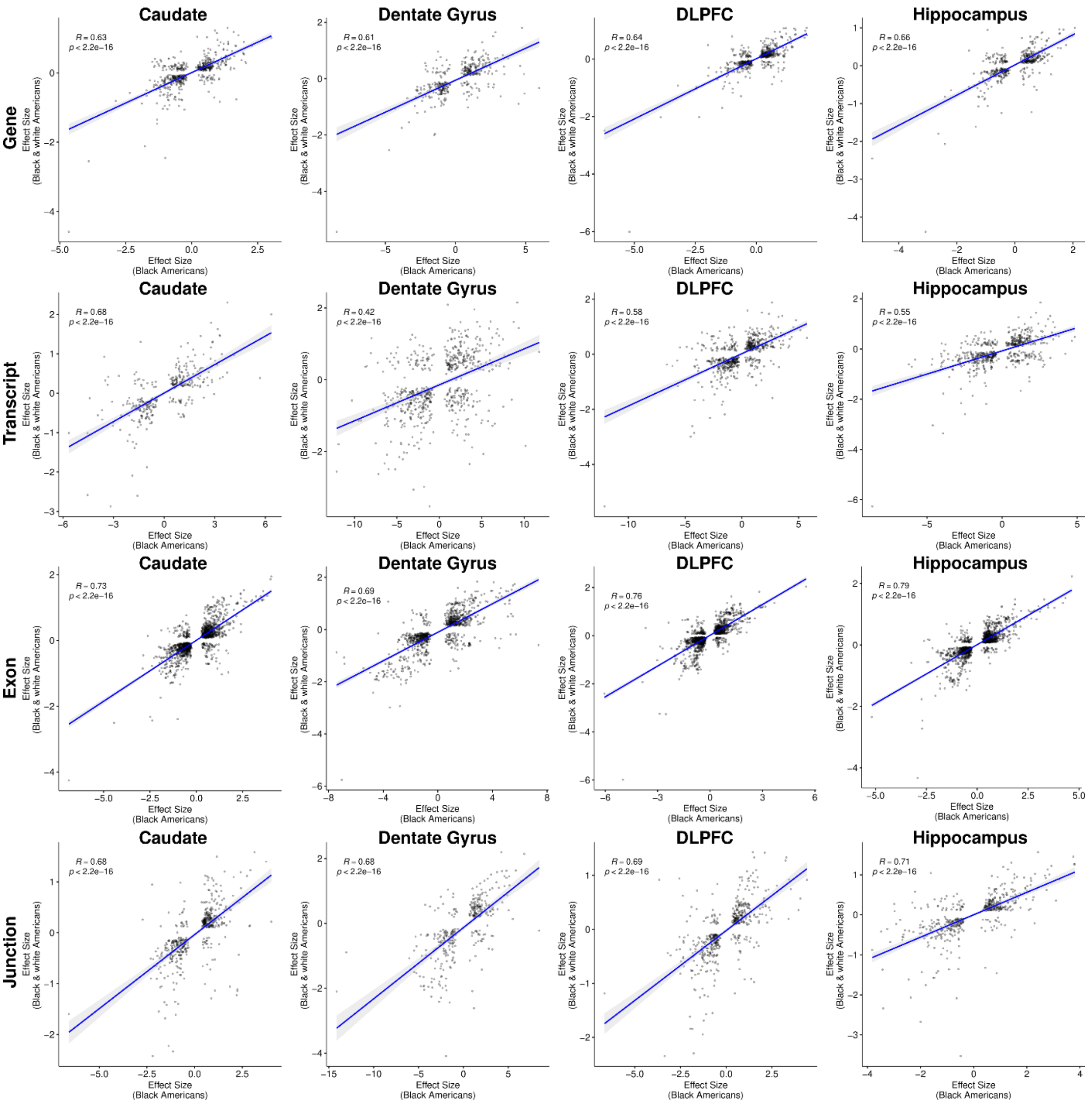

Fig. S45: Increased correlation of effect sizes for shared features between genetic ancestry association in admixed Black American individuals and self-reported race between Black and non-Hispanic white Americans. Scatter plot showing significant correlation (two-sided, Spearman correlation) between Black American-only analysis (x-axis) and combined analysis (Black and white Americans; y-axis) for each brain region and feature (i.e., gene, transcript, exon, and junction). Exact p-values: the caudate (genes: 3.9e-62, transcripts: 21.6e-45, exons: 2.0e-250, and junctions: 1.4e-64), dentate gyrus (genes: 3.5e-37, transcripts: 2.6e-26, exons: 3.7e-135, and junctions: 7.0e-46), DLPFC (genes: 5.1e-60, transcripts: 3.5e-55, exons: 1.5e-292, and junctions: 7.5e-72), and hippocampus (genes: 1.4e-56, transcripts: 1.1e-44, exons: 2.7e-304, and junctions: 3.1e-73). A fitted trend line is presented in blue as the mean values +/- standard deviation. The standard deviation is shaded in light gray.

Supplementary Tables

Table S1. Summary of global ancestry-associated differential expression results (lfsr < 0.05) by feature (gene, transcript, exon, and exon-exon junction) for ancestry differences within admixed AA (n=151) in the caudate (n=122), dentate gyrus (n=47), DLPFC (n=123), and hippocampus (n=133). The number of unique genes associated with transcript, exon, or junction is in parentheses.

Brain Region	Gene	Transcript (Geneid)	Exon (Geneid)	Junction (Geneid)
Caudate	1,273	1,949 (1,728)	5,264 (2,991)	1,621 (1,116)
Dentate Gyrus	997	3,153 (2,701)	4,630 (2,737)	1,527 (1,105)
DLPFC	1,075	2,462 (2,126)	5,552 (3,140)	1,625 (1,138)
Hippocampus	1,025	2,643 (2,263)	4,571 (2,717)	1,681 (1,177)

Table S2. Summary of local ancestry-associated differential expression results (lfsr < 0.05) by feature (gene, transcript, exon, and exon-exon junction) for ancestry differences within admixed AA (n=149) in the caudate (n=120), dentate gyrus (n=45), DLPFC (n=121), and hippocampus (n=131). The number of unique genes associated with transcript, exon, or junction is in parentheses.

Brain Region	Gene	Transcript (Geneid)	Exon (Geneid)	Junction (Geneid)
Caudate	6,657	7,854 (5,746)	108,709 (12,287)	49,464 (8,385)
Dentate Gyrus	4,154	5,857 (4,613)	67,239 (10,974)	35,216 (7,918)
DLPFC	6,148	7,541 (5,561)	99,351 (12,051)	45,981 (8,282)
Hippocampus	7,006	8,305 (5,996)	114,652 (12,411)	53,727 (8,510)

Table S3: Summary of main effect cis-eQTL (lfsr < 0.05) in Black American admixed individuals (n=148) by feature (gene, transcript, exon, and exon-exon junction) across the caudate (n=120), dentate gyrus (n=45), DLPFC (n=121), and hippocampus (n=131). eFeature: unique feature. eGene: unique gene ID.

		Caudate	Dentate Gyrus	DLPFC	Hippocampus
**Gene**	*eQTL*	698,047	605,755	728,533	688,628
	eFeature	10,867	11,664	11,173	10,408
	eGene	10,867	11,664	11,173	10,408
**Transcript**	*eQTL*	934,240	1,062,967	968,814	940,405
	eFeature	17,759	32,342	18,422	17,581
	eGene	10,369	14,674	10,710	10,320
**Exon**	*eQTL*	1,503,349	1,532,590	1,551,027	1,461,400
	eFeature	29,203	37,894	30,091	27,560
	eGene	10,423	12,675	10,612	9,927
**Junction**	*eQTL*	502,183	601,181	496,775	480,246
	eFeature	11,135	25,694	10,831	10,165
	eGene	3,084	4,768	3,022	2,874

Table S4: Summary of ancestry-dependent cis-eQTL (lfsr < 0.05) in Black American admixed individuals (n=148) by feature (gene, transcript, exon, and exon-exon junction) across the caudate (n=120), dentate gyrus (n=45), DLPFC (n=121), and hippocampus (n=131). eFeature: unique feature. eGene: unique gene ID.

		**Caudate**	**Dentate Gyrus**	**DLPFC**	**Hippocampus**
**Gene**	*eQTL*	3,281	5,484	3,441	3,371
	eFeature	531	942	573	531
	eGene	531	942	573	531
**Transcript**	*eQTL*	3,849	28,102	3,853	4,315
	eFeature	617	4,443	619	716
	eGene	580	3,529	582	671
**Exon**	*eQTL*	2,353	4,346	2,189	2,196
	eFeature	510	773	483	483
	eGene	330	481	314	312
**Junction**	*eQTL*	2,452	61,494	2,609	3,111
	eFeature	436	8,427	508	613
	eGene	275	4,748	332	419

Table S5. Black and non-Hispanic white Americans sample breakdown for adult (age > 17) neurotypical control postmortem caudate, dentate gyrus, DLPFC, and hippocampus (*10–12*). Abbreviations: Female (F), Male (M), Black American (BA), non-Hispanic white American (WA), and RNA integrity number (RIN).

	Caudate		Dentate Gyrus		DLPFC		Hippocampus	
**Characteristic**	**BA** N = 122[1]	**WA** N = 118[1]	**BA** N = 47[1]	**WA** N = 43[1]	**BA** N = 123[1]	**WA** N = 89[1]	**BA** N = 133[1]	**WA** N = 110[1]
**Sex**								
Female	50 (41%)	21 (18%)	16 (34%)	10 (23%)	48 (39%)	18 (20%)	53 (40%)	21 (19%)
Male	72 (59%)	97 (82%)	31 (66%)	33 (77%)	75 (61%)	71 (80%)	80 (60%)	89 (81%)
**Age**	46 (15)	51 (17)	46 (16)	50 (13)	44 (15)	47 (14)	43 (15)	46 (15)
**RIN**	7.83 (0.80)	7.87 (0.79)	5.45 (1.22)	5.14 (1.22)	7.70 (0.89)	7.70 (0.88)	7.72 (0.98)	7.76 (0.96)

[1] n (%); Mean (SD)

Table S6. Summary of differential expression results (lfsr < 0.05) by feature (gene, transcript, exon, and exon-exon junction) for ancestry differences in the caudate (n=240), dentate gyrus (n = 90), DLPFC (n=212), and hippocampus (n=243). The number of unique genes associated with transcript, exon, or junction is in parentheses.

Brain Region	Gene	Transcript (Geneid)	Exon (Geneid)	Junction (Geneid)
caudate	4,238	7,752 (5,451)	30,858 (9,303)	9,426 (4,181)
Dentate Gyrus	3,395	9,758 (6,543)	23,407 (8,145)	7,770 (3,848)
DLPFC	4,226	9,396 (6,288)	31,834 (9,524)	9,467 (4,231)
Hippocampus	4,025	9,456 (6,370)	29,340 (9,095)	9,034 (4,071)

Supplementary Data

1. **Data S1. BrainSeq_ancestry_4features_4regions_allFeatures.txt.gz**: Compressed text file of differential expression analysis after mash modeling for global genetic ancestry (continuous) across the caudate, dentate gyrus, DLPFC, and hippocampus for four features (gene, transcript, exon, and junction).
2. **Data S2. BrainSeq_ancestry_local_4features_4regions_allFeatures.txt.gz**: Compressed text file of differential expression analysis after mash modeling for local genetic ancestry (continuous by feature) across the caudate, dentate gyrus, DLPFC, and hippocampus for four features (gene, transcript, exon, and junction).
3. **Data S3. DE_functional_enrichment_ancestry_AAonly.xlsx**: Excel file of GO-term enrichment and gene set enrichment analysis (GSEA) for genetic ancestry (continuous) differentially expressed genes across the caudate, dentate gyrus, DLPFC, and hippocampus.
4. **Data S4**. **WGCNA_functional_enrichment_analysis_ancestry_AAonly.xlsx**: Excel file of GO-term enrichment for genetic ancestry-associated WGCNA modules across brain regions.
5. **Data S5. WGCNA_DEG_enrichment_modules_GO_analysis.tar.gz**: Compressed directory of ancestry-associated DEGs enriched for WGCNA module functional enrichment results (i.e., GO-term enrichment) for the caudate, dentate gyrus, DLPFC, and hippocampus.
6. **Data S6. BrainSeq_main_eQTL_4features_4regions_significant.txt.gz**: Compressed text file of main effect eQTL results (lfsr < 0.05), variant-feature pairs across the caudate, dentate gyrus, DLPFC, and hippocampus for four features (gene, transcript, exon, and junction).
7. **Data S7. BrainSeq_ancestry_dependent_eQTL_4features_4regions_significant.txt.gz**: Compressed text file of genetic ancestry-dependent eQTL results (lfsr < 0.05), variant-feature pairs across the caudate, dentate gyrus, DLPFC, and hippocampus for four features (gene, transcript, exon, and junction).
8. **Data S8**. **BrainSeq_DMR_global_local_comparison.tar.gz**: Compressed directory of PDF of scatter plots comparing DNAm association with local and global ancestry for the caudate, DLPFC, and hippocampus. Plots are annotated with genetic ancestry DMR test results.
9. **Data S9**. **DMR_functional_enrichment_localAncestry_AAonly.xlsx**: Excel file of GO-term enrichment for genetic ancestry differential methylation regions across the caudate, DLPFC, and hippocampus.
10. **Data S10. BrainSeq_DEancestry_LDSC_AAonly.xlsx**: Excel file of stratified LD score regression of admixed Black American differential expression analysis separated by direction of effect (all DEGs, upregulated in AA, or upregulated in EA) for genes (SNP proportion > 0.01) across the caudate, dentate gyrus, DLPFC, and hippocampus.
11. **Data S11. merged_phenotypes.csv**: CSV file of individual level subject information including information on sex, age, and self-identified race for the caudate, dentate gyrus, DLPFC, and hippocampus.
12. **Data S12. BrainSeq_est_prop_Bisque.Rdata**: R variable containing estimated cell type proportions using Bisque for the caudate, dentate gyrus, DLPFC, and hippocampus.

13. **Data S13. gwas_summary_statistics_ldsc.xlsx**: Excel file of GWAS summary statistics for heritability enrichment analysis.
14. **Data S14. BrainSeq_ancestry_binary_4features_4regions_allFeatures.txt.gz**: Compressed text file of binary differential expression analysis (10 permutations) for genetic ancestry (binary) across the caudate, dentate gyrus, DLPFC, and hippocampus for four features (gene, transcript, exon, and junction).
15. **Data S15**. **DE_binary_validation_functional_enrichment_ancestry_AA_EA.xlsx**: Excel file of GO-term enrichment and GSEA for internal validation for genetic ancestry (binary) differentially expressed genes across the caudate, dentate gyrus, DLPFC, and hippocampus for four features (gene, transcript, exon, and junction).
