# Supplementary material for: Analysis of gene expression in the postmortem brain of neurotypical Black Americans reveals contributions of genetic ancestry
Source: Nat Neurosci. 2024 May 20;27(6):1064–74. doi: 10.1038/s41593-024-01636-0 (PMC11156587; doi:10.1038/s41593-024-01636-0)
Supplement: Supplementary file 2 — Reporting Summary [file 41593_2024_1636_MOESM2_ESM.pdf]

Corresponding author(s): Daniel R Weinberger
Shizhong Han
Kynon JM Benjamin

Last updated by author(s): Mar 4, 2024

Reporting Summary

Nature Portfolio wishes to improve the reproducibility of the work that we publish. This form provides structure for consistency and transparency in reporting. For further information on Nature Portfolio policies, see our Editorial Policies and the Editorial Policy Checklist.

Statistics

For all statistical analyses, confirm that the following items are present in the figure legend, table legend, main text, or Methods section.

n/a	Confirmed	
☐	☒	The exact sample size (*n*) for each experimental group/condition, given as a discrete number and unit of measurement
☒	☐	A statement on whether measurements were taken from distinct samples or whether the same sample was measured repeatedly
☐	☒	The statistical test(s) used AND whether they are one- or two-sided<br>*Only common tests should be described solely by name; describe more complex techniques in the Methods section.*
☐	☒	A description of all covariates tested
☐	☒	A description of any assumptions or corrections, such as tests of normality and adjustment for multiple comparisons
☐	☒	A full description of the statistical parameters including central tendency (e.g. means) or other basic estimates (e.g. regression coefficient) AND variation (e.g. standard deviation) or associated estimates of uncertainty (e.g. confidence intervals)
☐	☒	For null hypothesis testing, the test statistic (e.g. *F*, *t*, *r*) with confidence intervals, effect sizes, degrees of freedom and *P* value noted<br>*Give P values as exact values whenever suitable.*
☒	☐	For Bayesian analysis, information on the choice of priors and Markov chain Monte Carlo settings
☒	☐	For hierarchical and complex designs, identification of the appropriate level for tests and full reporting of outcomes
☐	☒	Estimates of effect sizes (e.g. Cohen's *d*, Pearson's *r*), indicating how they were calculated

Our web collection on statistics for biologists contains articles on many of the points above.

Software and code

Policy information about availability of computer code

Data collection

No software was used for data collection.

Data analysis

Code used for the analyses presented in this paper is available at https://github.com/LieberInstitute/aanri_phase1 (10.5281/zenodo.7777821).

Software packages used in data analysis include the following:
Genotype imputation: TOPMed Imputation server for general genotype imputation (Eagle v2.4), liftOver to convert genotypes from hg19 to hg38, PLINK (v2.00a3LM) for pre- and post-imputation quality control, and PLINK (v1.9) for MDS population stratification;
HLA imputation: PLINK2 (v2.00a3LM) for quality control, Michigan Imputation Server for reference panel imputation and genotype phasing, and BCFtools (v1.13) for post-imputation quality control;
WGBS data processing (done in previous publication [ref 14, 63], some versions are unknown): FastQC for quality control, Trim Galore to remove adaptor contect, Arioc to align reads, SAMBLASTER to remove duplicate alignments, samtools (v1.9) to filter alignments, Bismark methylation extractor was used to extract methylation data, and bsseq (v1.18) to process and combine DNA methylation proportions across samples
Admixture calculation (global and local): STRUCTURE (v2.3.4) for global admixture proportion estimations; RFIMX (v2.03-r0) was used to infer local ancestry;
Differential expression analysis: BisqueRNA (v1.0.4) was used for cell-type deconvolution; limma (R v4.2; v3.46.0) and edgeR (v3.40.2) for differential expression analysis (see GitHub for full environmental details);
mashr (v0.2.57) was used for differential expression and eQTL data;
WGCNA (R version 4.2; v1.72) for network analysis;
Functional gene term enrichment used gseGO and gseDGN (GSEA; clusterProfiler [v4.6.2] and DOSE [v3.24.2]; R v4.2) and enrichGO and

enrichDGN (hypergeometric; cluster Profiler and DOSE); GOATOOLS (v1.0.15) for network analysis gene-term enrichment;
Glial cell composition comparison of single-cell multiple brain region data: We normalized with batchelor (R v4.3; v1.17.2), integrated single cell datasets with scVI and annotated subpopulations with scANVI from scvi-tools (v0.20.1); composition differences using propeller from speckle (v1.1.0; R v4.3); tSNE mapping from scater (v1.28.0; R v4.3); conversion of H5AD files using zellkonverter (v1.8.0; R v4.3)
eQTL analysis: tensorQTL (v1.0.7) for eQTL mapping; Picard tools (v2.20.1) was used to extract mean insert size; eigenMT was implemented in tensorQTL to correct for multiple testing (interaction eQTL analysis);
Allele frequency differences were calculated in PLINK (v1.9);
Predicted expression: PyTorch (v1.11.0+cu113) was used to calculated predicted expression (top eQTL); bigstatsr (v1.5.12; R v4.2) was used to implement elastic net;
S-LDSC (v1.0.1) was used to perform enrichment of heritability of complex traits;
rGREAT (v2.0.2; R v4.2) was used to assess biological function of DMRs; plyranges (v1.18.0; R v4.2) formated DMRs into genomic ranges format; annotatr (v1.24.0; R v4.2) used to annotate VMRs/DMRs;
Graphics: We used R (v4.03; v4.2; v4.3) to generate all plots. We generated UpSet plots using ComplexHeatmap (v2.10.0); circos plot using circlize (v0.4.15); enrichment heatmaps, gene term enrichment, error plots, box plots, distribution plots, and scatterplots using a combination of ggplot2 (v3.3.6) and ggpubr (v0.4.15); pairwise comparison plots using corrplot (v0.92); meta plots using mashr; Venn diagrams using ggvenn (v0.1.10).
Jupyter Notebooks (v6.0.2) were used for visualization of some code and are available in the GitHub repository.

For manuscripts utilizing custom algorithms or software that are central to the research but not yet described in published literature, software must be made available to editors and reviewers. We strongly encourage code deposition in a community repository (e.g. GitHub). See the Nature Portfolio guidelines for submitting code & software for further information.

Data

Policy information about availability of data

All manuscripts must include a data availability statement. This statement should provide the following information, where applicable:

- Accession codes, unique identifiers, or web links for publicly available datasets
- A description of any restrictions on data availability
- For clinical datasets or third party data, please ensure that the statement adheres to our policy

Publicly available BrainSeq Consortium total RNA DLPFC and hippocampus RangedSummarizedExperiment R Objects with processed counts are available at http://eqtl.brainseq.org/phase2/. Publicly available BrainSeq Consortium total RNA caudate RangedSummarizedExperiment R Objects with processed counts are available at http://erwinpaquolalab.libd.org/caudate_eqtl/. Publicly available dentate gyrus RangedSummarizedExperiment R Objects with processed counts and phenotype information are available at http://research.libd.org/dg_hippo_paper/data.html. Analysis-ready genotype data will be shared with researchers that obtain dbGaP accession phs000979.v3.p2. FASTQ files for total RNA DLPFC and hippocampus are available via Globus collections jhpce#bsp2-dlpfc and jhpce#bsp2-hippo at https://research.libd.org/globus/. FASTQ files for the dentate gyrus are available via Sequence Read Archive (SRP241159). FASTQ files for the caudate nucleus are available via dbGaP accession phs003495.v1.p1. DNA methylation data is available at https://github.com/LieberInstitute/aanri_phase1.

We used publicly available single cell datasets. Glial subpopulation single-cell data from the human postmortem hippocampus astrocyte, microglia, and oligodendrocyte lineage is available from UCSC cell browser ("Human Hippocampus Lifespan" collection). The human PBMCs single-cell data is available from Zenodo (10.5281/zenodo.4273999). Multiple human brain region single-cell datasets (i.e., DLPFC, hippocampus, nucleus accumbens, amygdala, and subgenual anterior cingulate cortex) are available by brain region from GitHub (https://github.com/LieberInstitute/10xPilot_snRNAseq-human). Human microglial state dynamics in Alzhiemer's disease single-cell data is available from http://compbio.mit.edu/microglia_states/.

We downloaded the following additional publicly available genotype data sources. We downloaded the loss-of-function variant information from the genome aggregation database (gnomAD; version 2) website (https://gnomad.broadinstitute.org/downloads) via Google Cloud Public Datasets storage (https://storage.googleapis.com/gcp-public-data--gnomad/papers/2019-flagship-lof/v1.0/gnomad.v2.1.1.all_lofs.txt.bgz). We downloaded from https://www.internationalgenome.org/data/ genotype references for the 1000 Genomes Project. We downloaded HapMap Project Phase 3 SNPs from https://www.broadinstitute.org/medical-and-population-genetics/hapmap-3. We downloaded all SNPs from the LDSC resource website (https://data.broadinstitute.org/alkesgroup/LDSCORE/w_hm3.snplist.bz2).

Research involving human participants, their data, or biological material

Policy information about studies with human participants or human data. See also policy information about sex, gender (identity/presentation), and sexual orientation and race, ethnicity and racism.

Reporting on sex and gender	Sex/gender reported in this study are self-reported. No sex-specific analysis is reported in this study. A summary of sex/gender breakdown is found in Table 1. Unaggregated numbers are available upon request.
Reporting on race, ethnicity, or other socially relevant groupings	We selectively examined our admixed Black American population (151 unique individuals; Table 1) to 1) characterize transcriptional changes associated with African or European genetic ancestry in neurotypical adults (age > 17) and 2) limit potential confounding effects of systematic environmental factors that may differ between Black and White American samples. We used self-reported race to identify Black/African Americans and White Americans.<br><br>We define African ancestry (AA) as genetic similarity associated with individuals with recent African ancestry. We define European ancestry (EA) as genetic similarity associated with Northern Europeans from Utah. These are a continuous measures determined by STRUCTURE (global) or RFMIX (local). For STRUCTURE admixture proportion estimates, we used SNPs informative with respect to ancestry using the 1000 Genomes populations. For African ancestry superpopulation, we used Esan, Gambian, Luhyu, Mende, and Yoruba populations to estimate our Black American African ancestry. For European ancestry, we used the 1000 Genomes CEU (Northern Europeans from Utah).
Population characteristics	All samples are neurotypical controls. Mean age ranges from 43 to 46. Genetic similarity was measured using global population structure via multidimensional scaling (MDS). A full breakdown is found in Table S1. Unaggregated numbers are

	available upon request.
Recruitment	All specimens used in this study were obtained with informed consent from the next kin. See below for more details.
Ethics oversight	The research described herein complies with all relevant ethical regulations. Additionally, we declare that all specimens used in this study were obtained with informed consent. We obtained informed consent from the next kin under protocols No. 12-24 (the Department of Health and Mental Hygiene for the Office of the Chief Medical Examiner for the State of Maryland) and No. 20111080 (the Western Institutional Review Board for the Offices of the Chief Medical Examiner for Kalamazoo Michigan, University of North Dakota in Grand Forks North Dakota, and Santa Clara County California). We obtained samples at the Clinical Brain Disorder Branch (CBDB) at the National Institute of Mental Health (NIMH) from the Northern Virginia and District of Columbia Medical Examiners' Office, according to NIH Institutional Review Board guidelines (Protocol #90-M-0142). The LIBD received the tissues by donation under the terms of a material transfer agreement. The Institutional Review Board of the University of Maryland at Baltimore and the State of Maryland approved the study protocols that collected these brain regions (10–12). Details of case selection, curation, diagnosis, and anatomical localization and dissection can be found in previous publications from our research group (10–12).

Note that full information on the approval of the study protocol must also be provided in the manuscript.

Field-specific reporting

Please select the one below that is the best fit for your research. If you are not sure, read the appropriate sections before making your selection.

☒ Life sciences ☐ Behavioural & social sciences ☐ Ecological, evolutionary & environmental sciences

For a reference copy of the document with all sections, see nature.com/documents/nr-reporting-summary-flat.pdf

Life sciences study design

All studies must disclose on these points even when the disclosure is negative.

Sample size	We used all samples available based on experimental design from the LIBD repository. We quantify the contributions of common genetic variations to genetic ancestry differences using a total of 425 samples, including the caudate (n=122), dentate gyrus (n=47), DLPFC (n=123), and hippocampus (n=133). Additionally, we examine the influence of genetic ancestry on DNAm using WGBS data of the admixed Black American donors from the caudate (n=89), DLPFC (n=69), and hippocampus (n=69).
Data exclusions	We selected samples per brain region using five common inclusion criteria: 1) RiboZero RNA-sequencing library preparation, 2) recent African ancestry (self-reported race), 3) TOPMed imputed genotypes available, 4) adults (age > 17) and 5) diagnosis of neurotypical control. This resulted in a total of 425 samples from 151 unique individuals across the caudate (n=121), dentate gyrus (n=47), DLPFC (n=123), and hippocampus (n=133). Subject details are summarized in Table S1.
Replication	External validation was not possible as there are no postmortem brain datasets with suffienct samples sizes for self-report Black Americans. Of note, the PsychENCODE DLPFC data has significant overlap with LIBD brains and is not appropriate for external replication of this brain region. For internal validation of global ancestry-associated DE features (i.e., gene, transcript, exon, and junction), we performed differential expression analysis with a combination of Black and White American individuals using mash. To limit the influence of the larger sample size compared to "Global ancestry-associated differential expression analysis", we randomly sampled ten times without replacement to approximately the admixed Black American-only analysis sample size.
Randomization	This is an observational study from postmortem human brain tissues. As such, subjects were not randomized into outcome groups.
Blinding	Investigators were not blinded to group allocation since the study is observational.

Reporting for specific materials, systems and methods

We require information from authors about some types of materials, experimental systems and methods used in many studies. Here, indicate whether each material, system or method listed is relevant to your study. If you are not sure if a list item applies to your research, read the appropriate section before selecting a response.

Materials & experimental systems

n/a	Involved in the study	
☒	☐	Antibodies
☒	☐	Eukaryotic cell lines
☒	☐	Palaeontology and archaeology
☒	☐	Animals and other organisms
☒	☐	Clinical data
☒	☐	Dual use research of concern
☒	☐	Plants

Methods

n/a	Involved in the study	
☒	☐	ChIP-seq
☒	☐	Flow cytometry
☒	☐	MRI-based neuroimaging
