# Supplementary figures and images for: Analysis of gene expression in the postmortem brain of neurotypical Black Americans reveals contributions of genetic ancestry
Source: Nat Neurosci. 2024 May 20;27(6):1064–74. doi: 10.1038/s41593-024-01636-0 (PMC11156587; doi:10.1038/s41593-024-01636-0)

## Module greenyellow

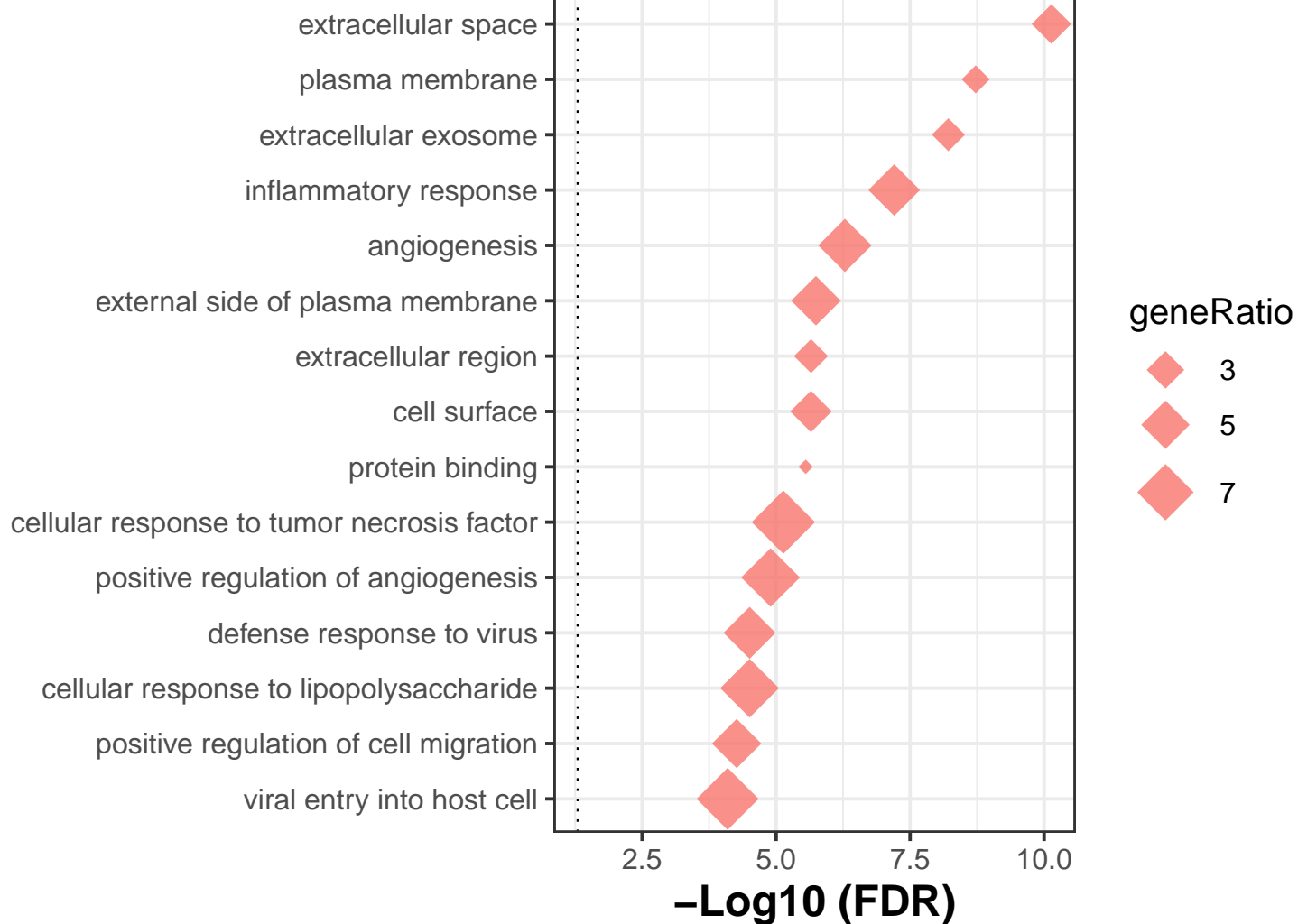

Supplement: Supplementary file 5 — Compressed directory of ancestry-associated DEGs enriched for WGCNA module functional enrichment results (that is, GO term enrichment) for the caudate nucleus, dentate gyrus, DLPFC and hippocampus. [file 41593_2024_1636_MOESM5_ESM.gz › wgcna_functional_enrichment/dlpfc/module_greenyellow_go_enrichment.pdf]

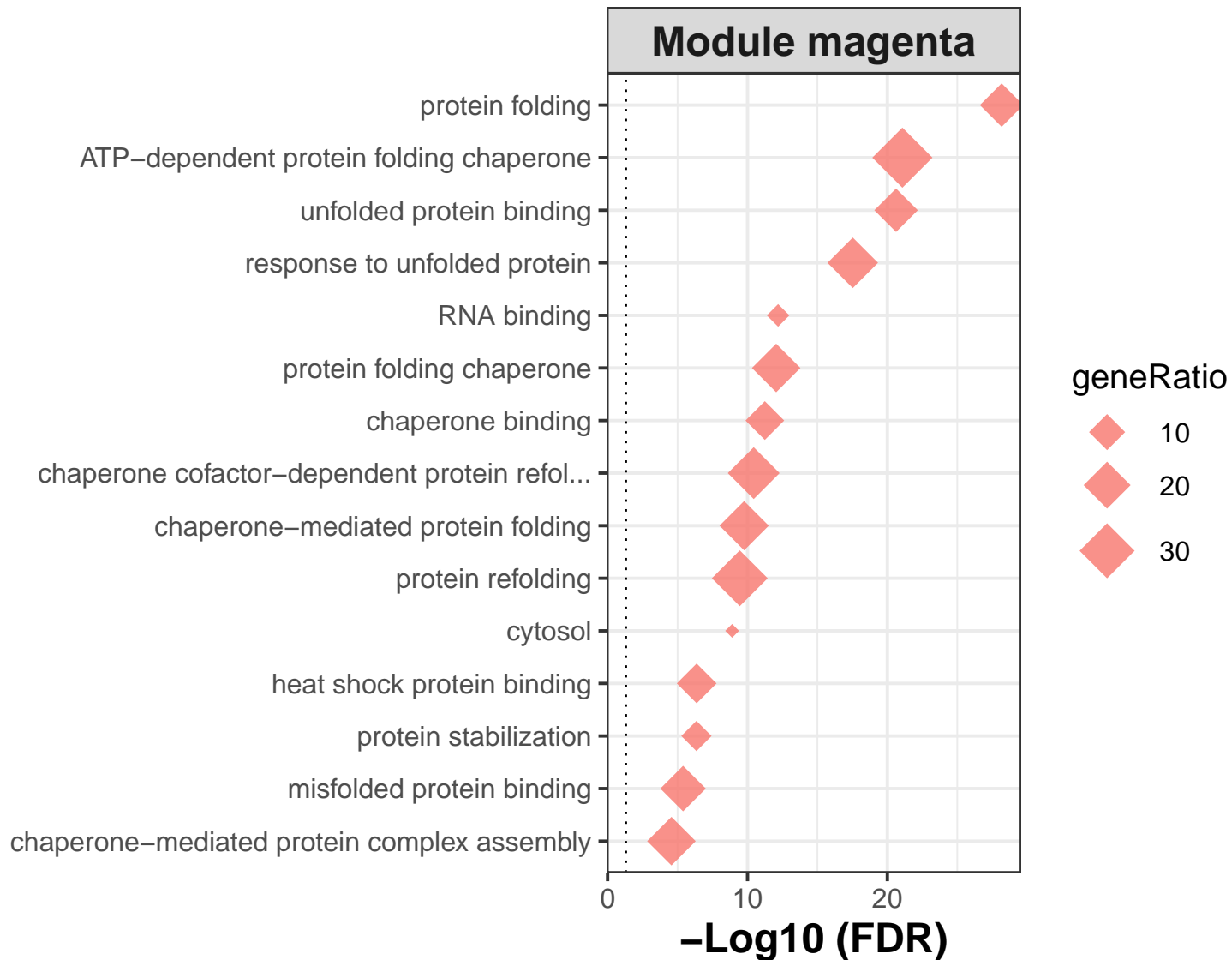

Supplement: Supplementary file 5 — Compressed directory of ancestry-associated DEGs enriched for WGCNA module functional enrichment results (that is, GO term enrichment) for the caudate nucleus, dentate gyrus, DLPFC and hippocampus. [file 41593_2024_1636_MOESM5_ESM.gz › wgcna_functional_enrichment/dlpfc/module_magenta_go_enrichment.pdf]

## Module darkorange

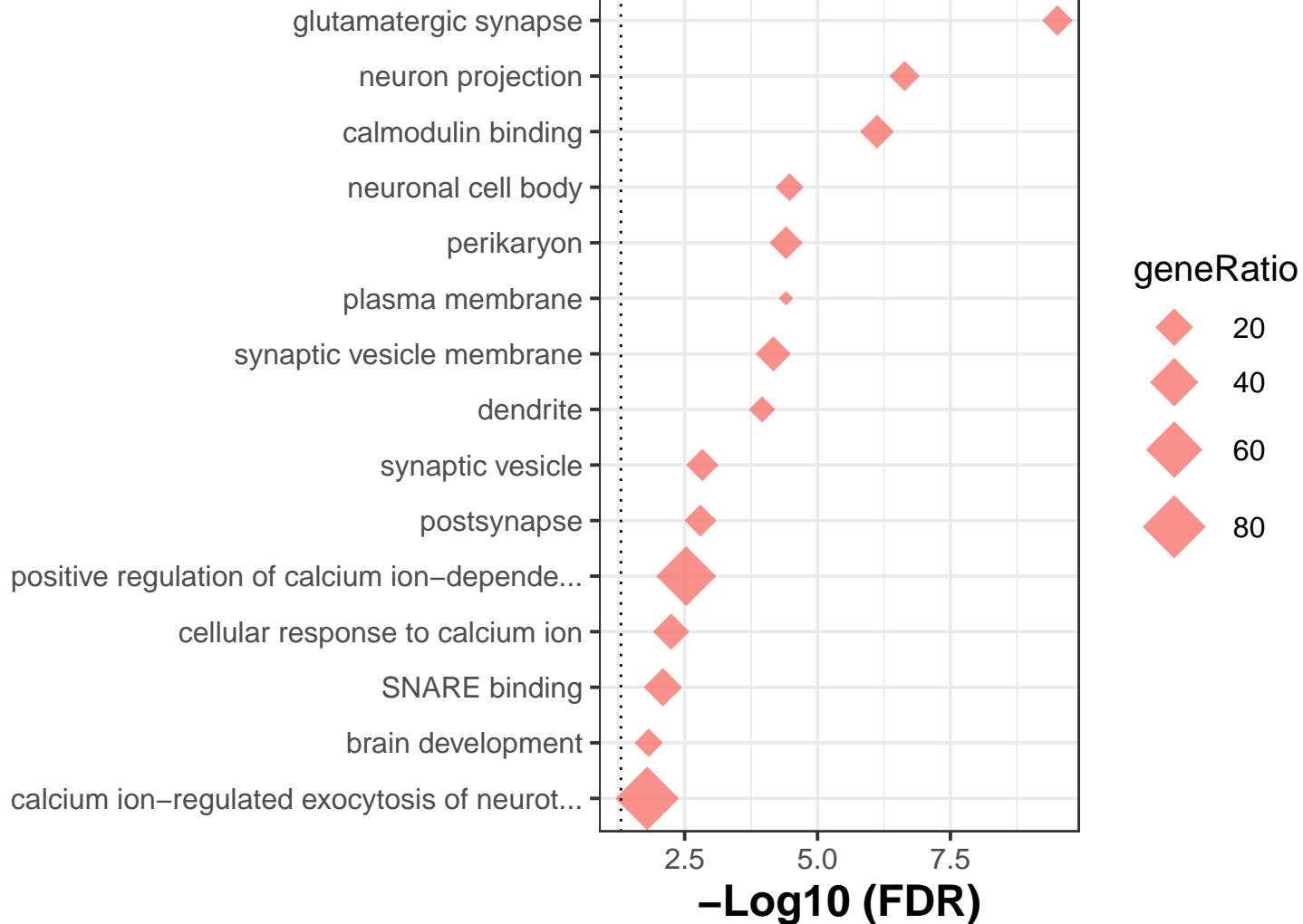

Supplement: Supplementary file 5 — Compressed directory of ancestry-associated DEGs enriched for WGCNA module functional enrichment results (that is, GO term enrichment) for the caudate nucleus, dentate gyrus, DLPFC and hippocampus. [file 41593_2024_1636_MOESM5_ESM.gz › wgcna_functional_enrichment/dlpfc/module_darkorange_go_enrichment.pdf]

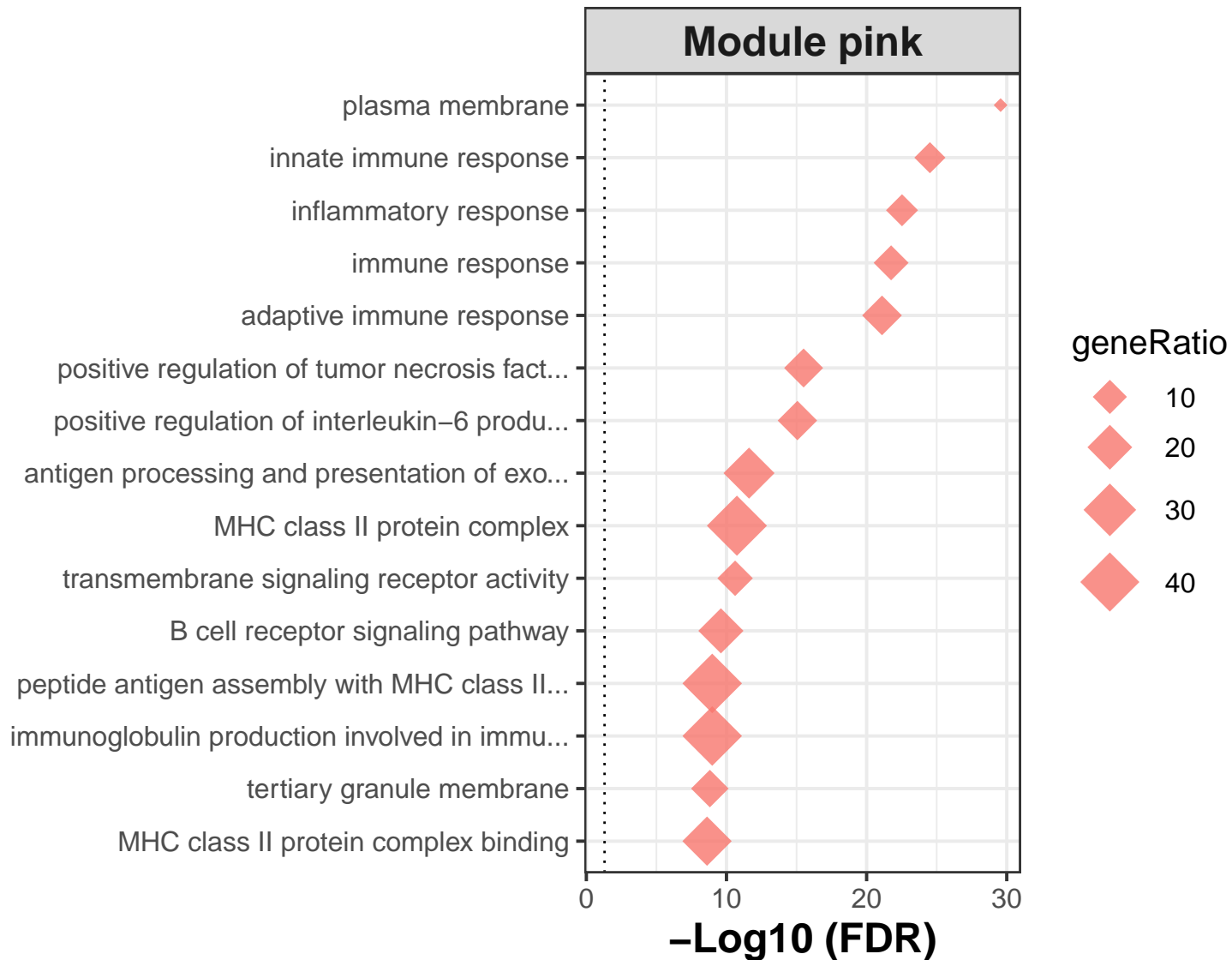

Supplement: Supplementary file 5 — Compressed directory of ancestry-associated DEGs enriched for WGCNA module functional enrichment results (that is, GO term enrichment) for the caudate nucleus, dentate gyrus, DLPFC and hippocampus. [file 41593_2024_1636_MOESM5_ESM.gz › wgcna_functional_enrichment/dlpfc/module_pink_go_enrichment.pdf]

## Module blue

mitochondrion

geneRatio

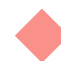

1.656315

1.50

1.75

2.00

2.25

**$-\text{Log}_{10}(\text{FDR})$**

Supplement: Supplementary file 5 — Compressed directory of ancestry-associated DEGs enriched for WGCNA module functional enrichment results (that is, GO term enrichment) for the caudate nucleus, dentate gyrus, DLPFC and hippocampus. [file 41593_2024_1636_MOESM5_ESM.gz › wgcna_functional_enrichment/dlpfc/module_blue_go_enrichment.pdf]

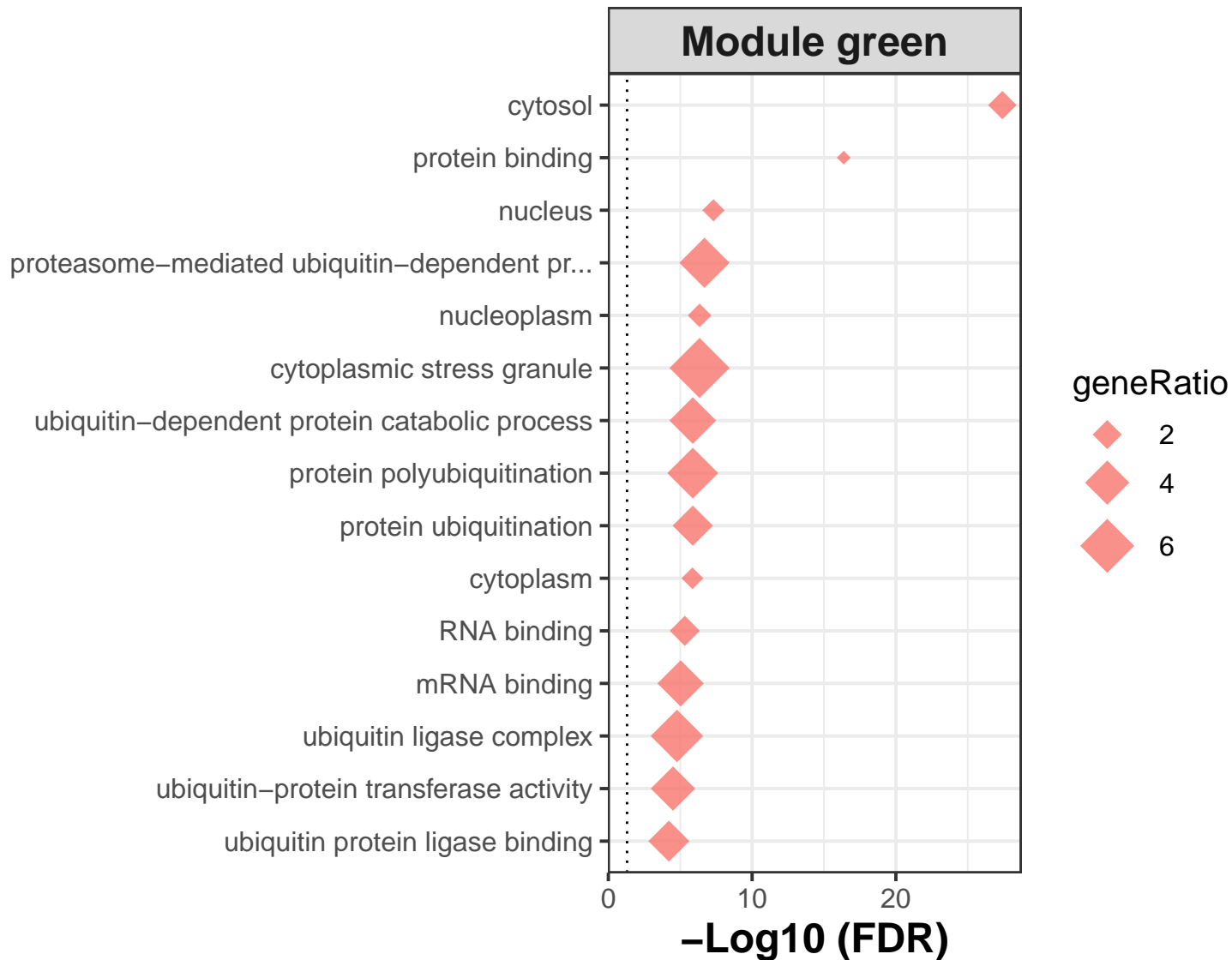

Supplement: Supplementary file 5 — Compressed directory of ancestry-associated DEGs enriched for WGCNA module functional enrichment results (that is, GO term enrichment) for the caudate nucleus, dentate gyrus, DLPFC and hippocampus. [file 41593_2024_1636_MOESM5_ESM.gz › wgcna_functional_enrichment/dlpfc/module_green_go_enrichment.pdf]

## Module midnightblue

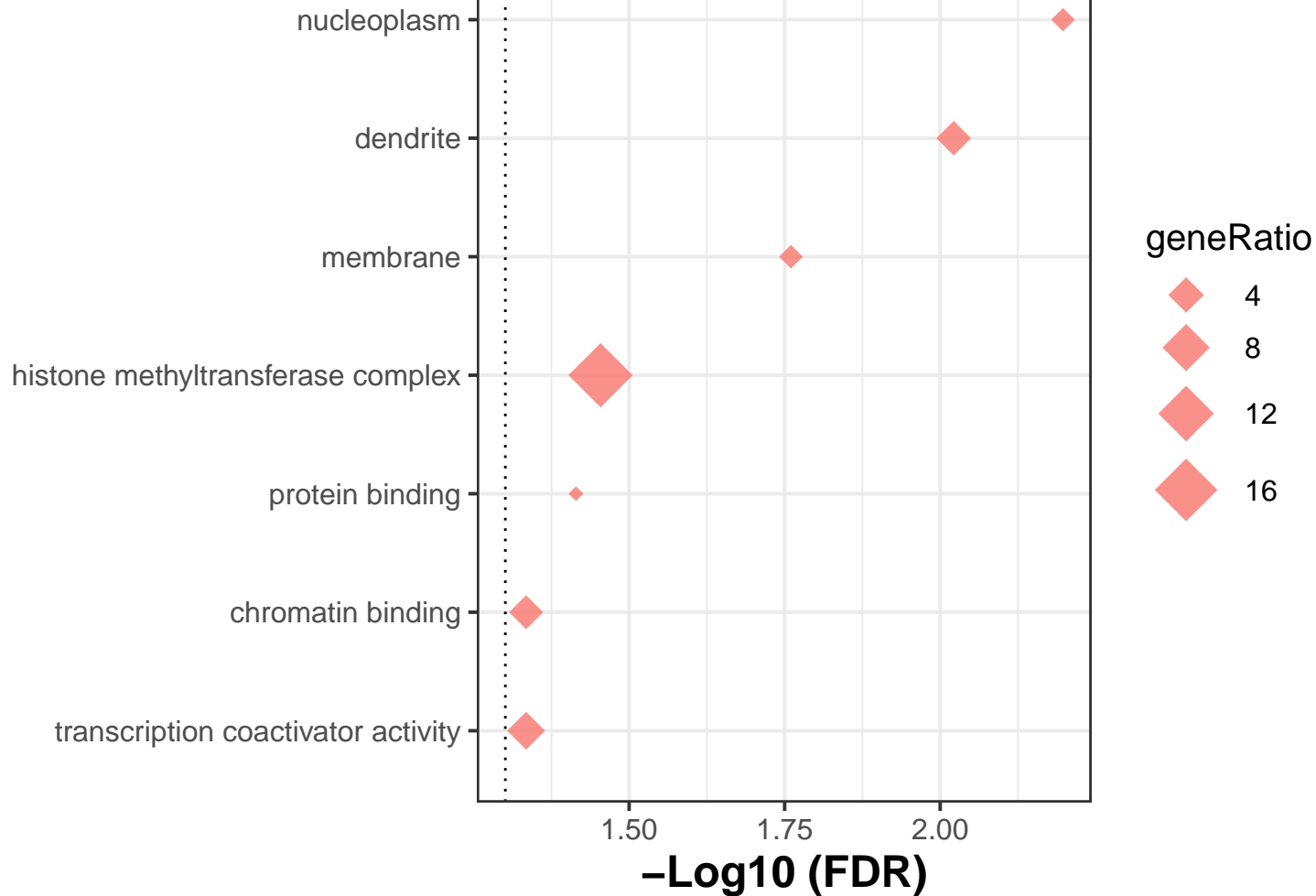

Supplement: Supplementary file 5 — Compressed directory of ancestry-associated DEGs enriched for WGCNA module functional enrichment results (that is, GO term enrichment) for the caudate nucleus, dentate gyrus, DLPFC and hippocampus. [file 41593_2024_1636_MOESM5_ESM.gz › wgcna_functional_enrichment/dlpfc/module_midnightblue_go_enrichment.pdf]

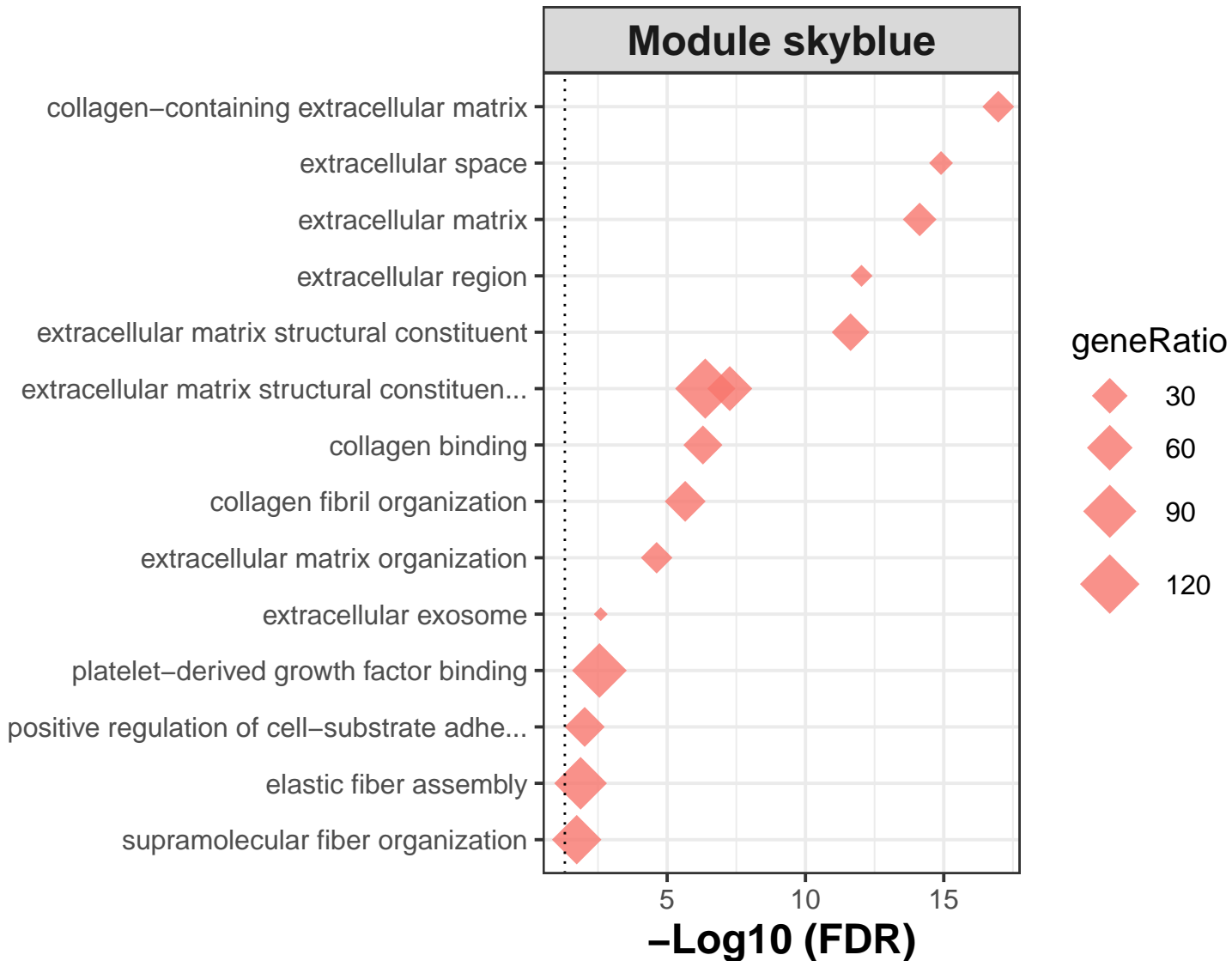

Supplement: Supplementary file 5 — Compressed directory of ancestry-associated DEGs enriched for WGCNA module functional enrichment results (that is, GO term enrichment) for the caudate nucleus, dentate gyrus, DLPFC and hippocampus. [file 41593_2024_1636_MOESM5_ESM.gz › wgcna_functional_enrichment/dlpfc/module_skyblue_go_enrichment.pdf]

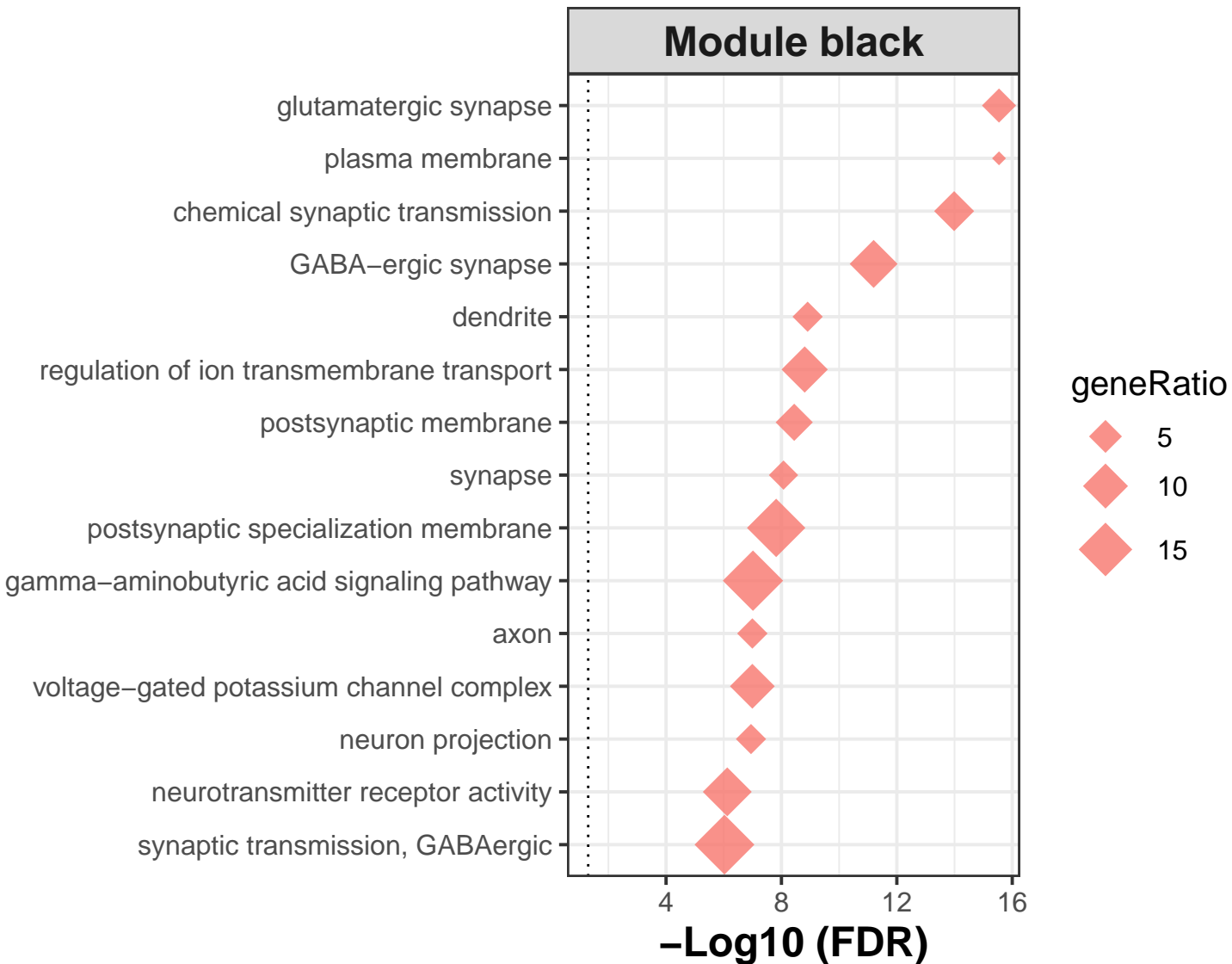

Supplement: Supplementary file 5 — Compressed directory of ancestry-associated DEGs enriched for WGCNA module functional enrichment results (that is, GO term enrichment) for the caudate nucleus, dentate gyrus, DLPFC and hippocampus. [file 41593_2024_1636_MOESM5_ESM.gz › wgcna_functional_enrichment/dlpfc/module_black_go_enrichment.pdf]

## Module darkgreen

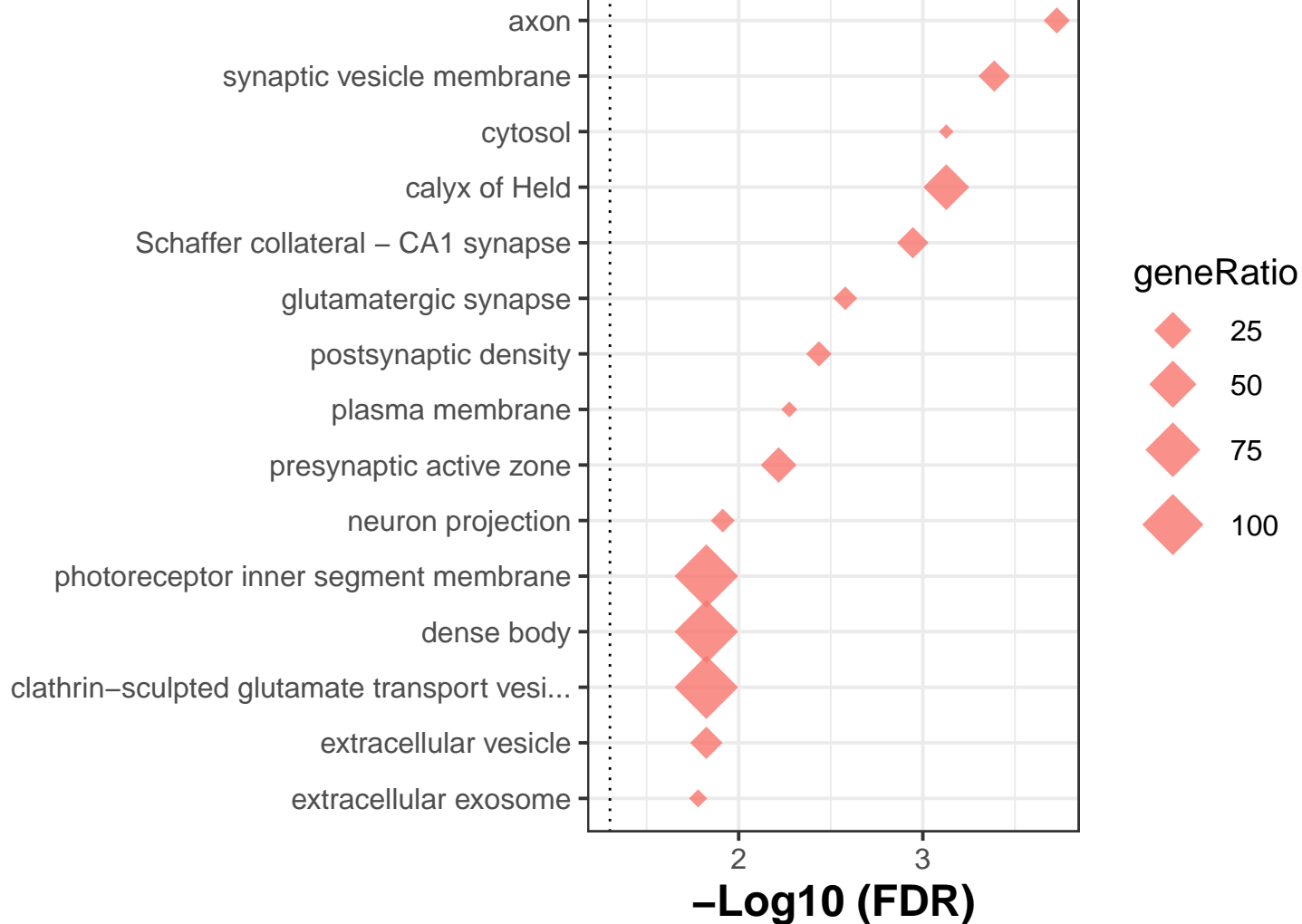

Supplement: Supplementary file 5 — Compressed directory of ancestry-associated DEGs enriched for WGCNA module functional enrichment results (that is, GO term enrichment) for the caudate nucleus, dentate gyrus, DLPFC and hippocampus. [file 41593_2024_1636_MOESM5_ESM.gz › wgcna_functional_enrichment/dlpfc/module_darkgreen_go_enrichment.pdf]

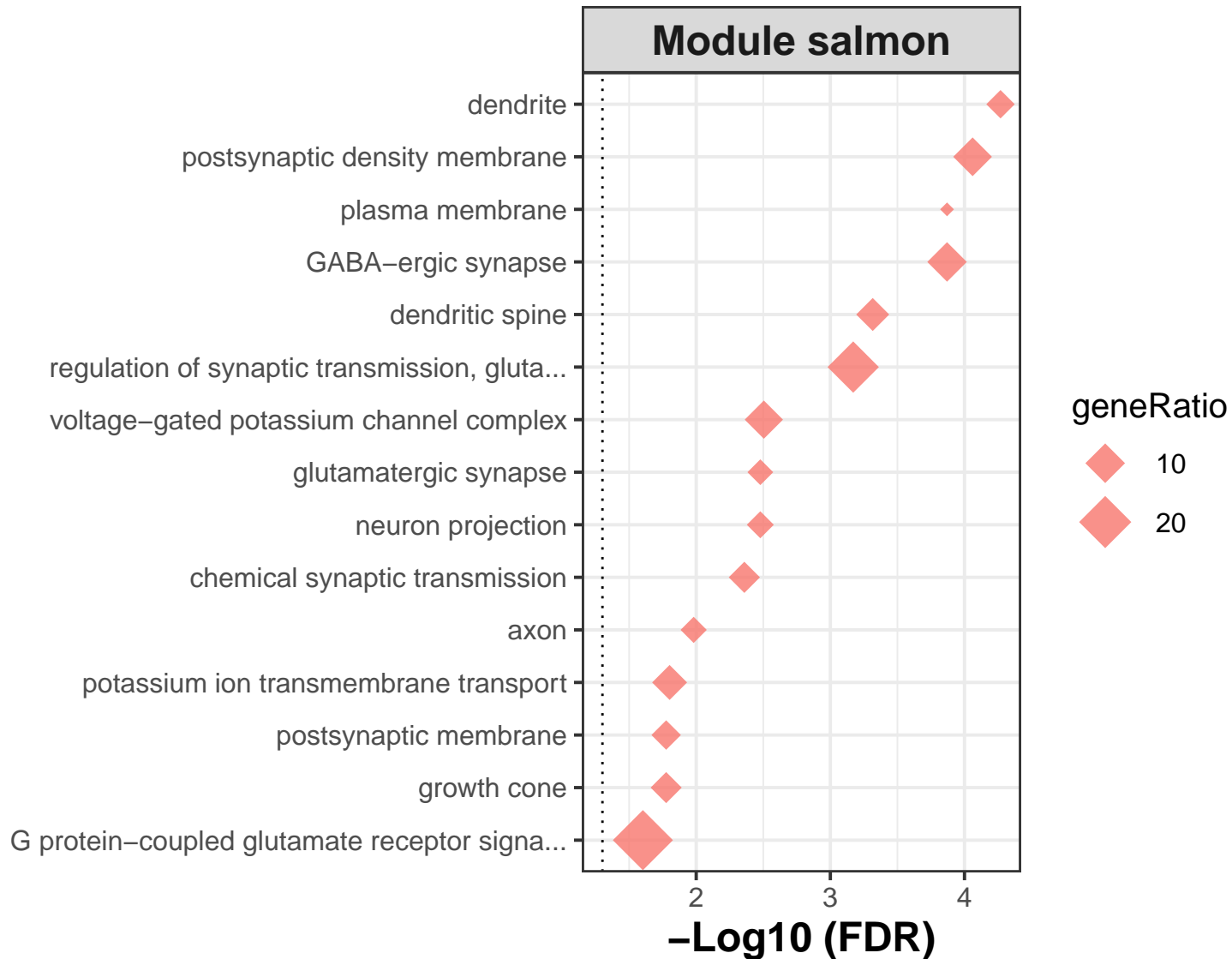

Supplement: Supplementary file 5 — Compressed directory of ancestry-associated DEGs enriched for WGCNA module functional enrichment results (that is, GO term enrichment) for the caudate nucleus, dentate gyrus, DLPFC and hippocampus. [file 41593_2024_1636_MOESM5_ESM.gz › wgcna_functional_enrichment/dlpfc/module_salmon_go_enrichment.pdf]

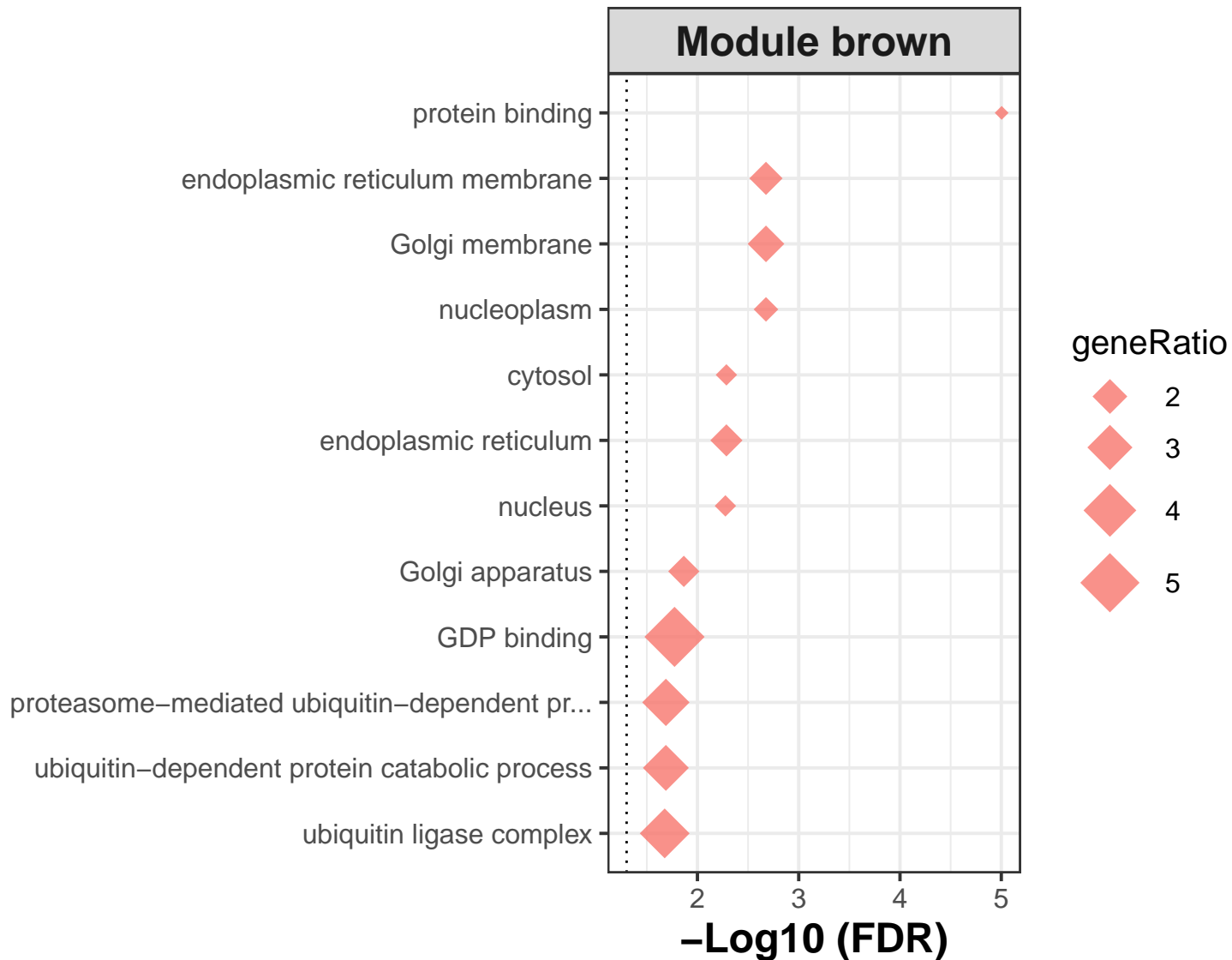

Supplement: Supplementary file 5 — Compressed directory of ancestry-associated DEGs enriched for WGCNA module functional enrichment results (that is, GO term enrichment) for the caudate nucleus, dentate gyrus, DLPFC and hippocampus. [file 41593_2024_1636_MOESM5_ESM.gz › wgcna_functional_enrichment/dlpfc/module_brown_go_enrichment.pdf]

## Module saddlebrown

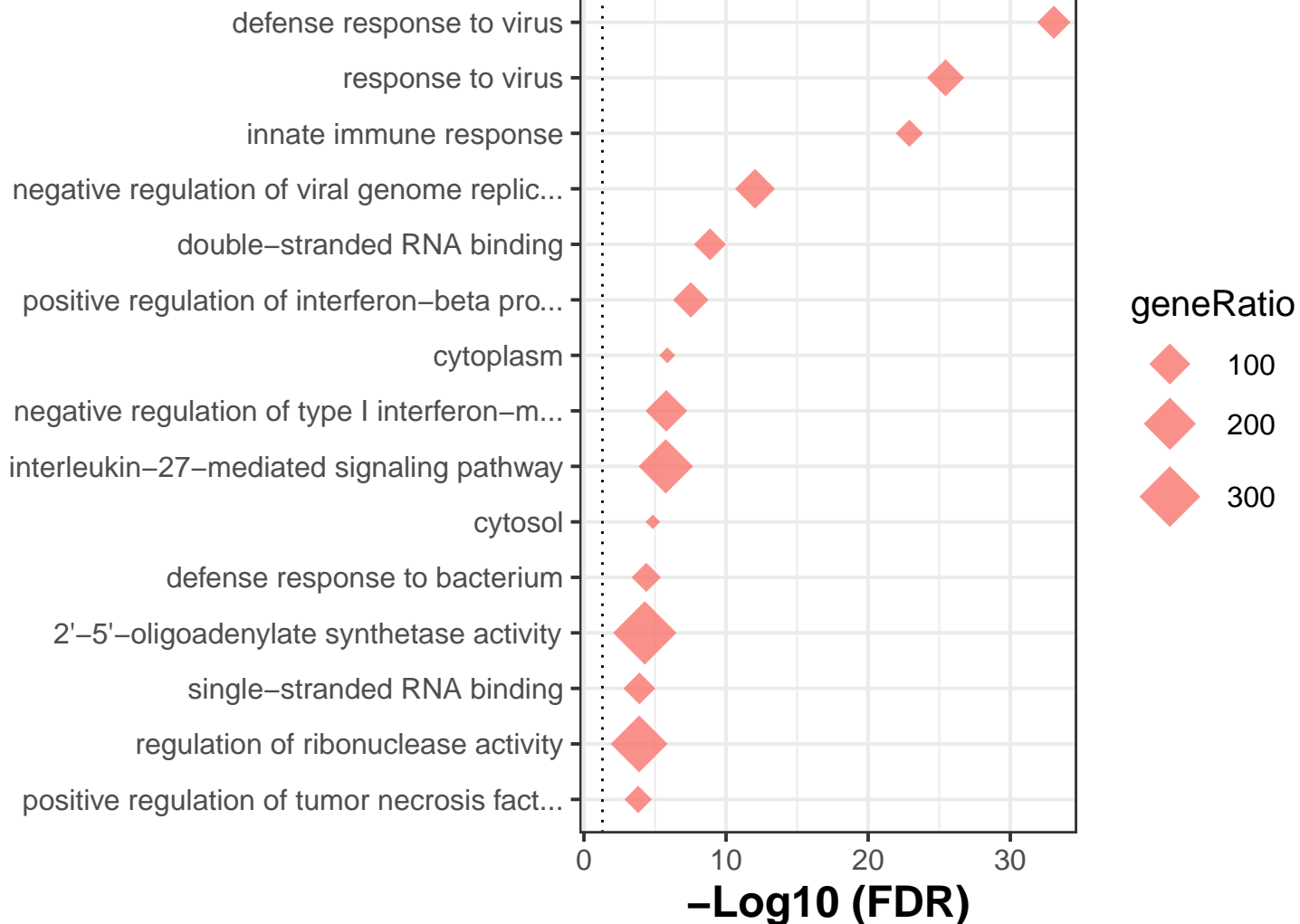

Supplement: Supplementary file 5 — Compressed directory of ancestry-associated DEGs enriched for WGCNA module functional enrichment results (that is, GO term enrichment) for the caudate nucleus, dentate gyrus, DLPFC and hippocampus. [file 41593_2024_1636_MOESM5_ESM.gz › wgcna_functional_enrichment/dlpfc/module_saddlebrown_go_enrichment.pdf]

## Module darkturquoise

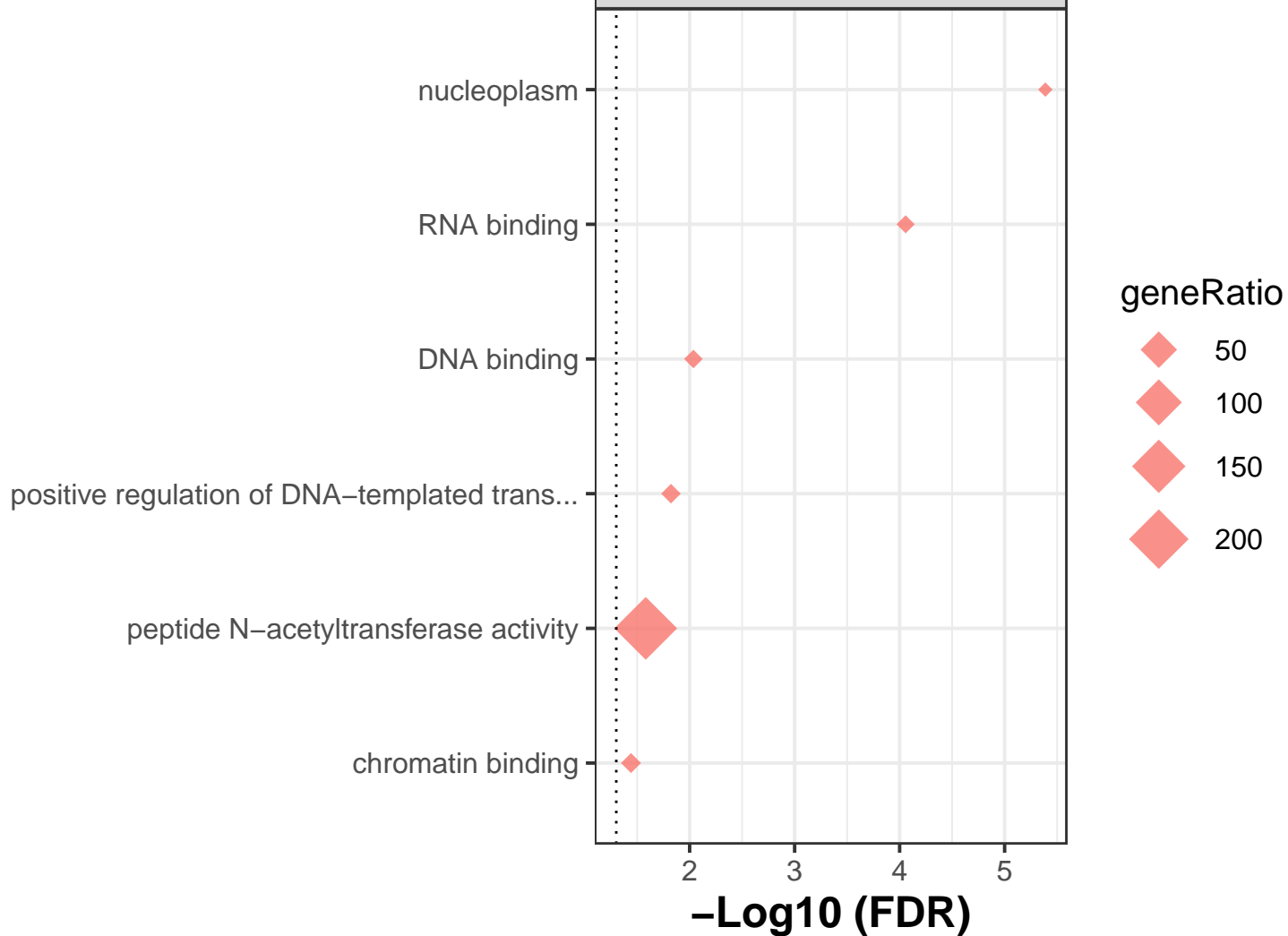

Supplement: Supplementary file 5 — Compressed directory of ancestry-associated DEGs enriched for WGCNA module functional enrichment results (that is, GO term enrichment) for the caudate nucleus, dentate gyrus, DLPFC and hippocampus. [file 41593_2024_1636_MOESM5_ESM.gz › wgcna_functional_enrichment/dlpfc/module_darkturquoise_go_enrichment.pdf]

## Module cyan

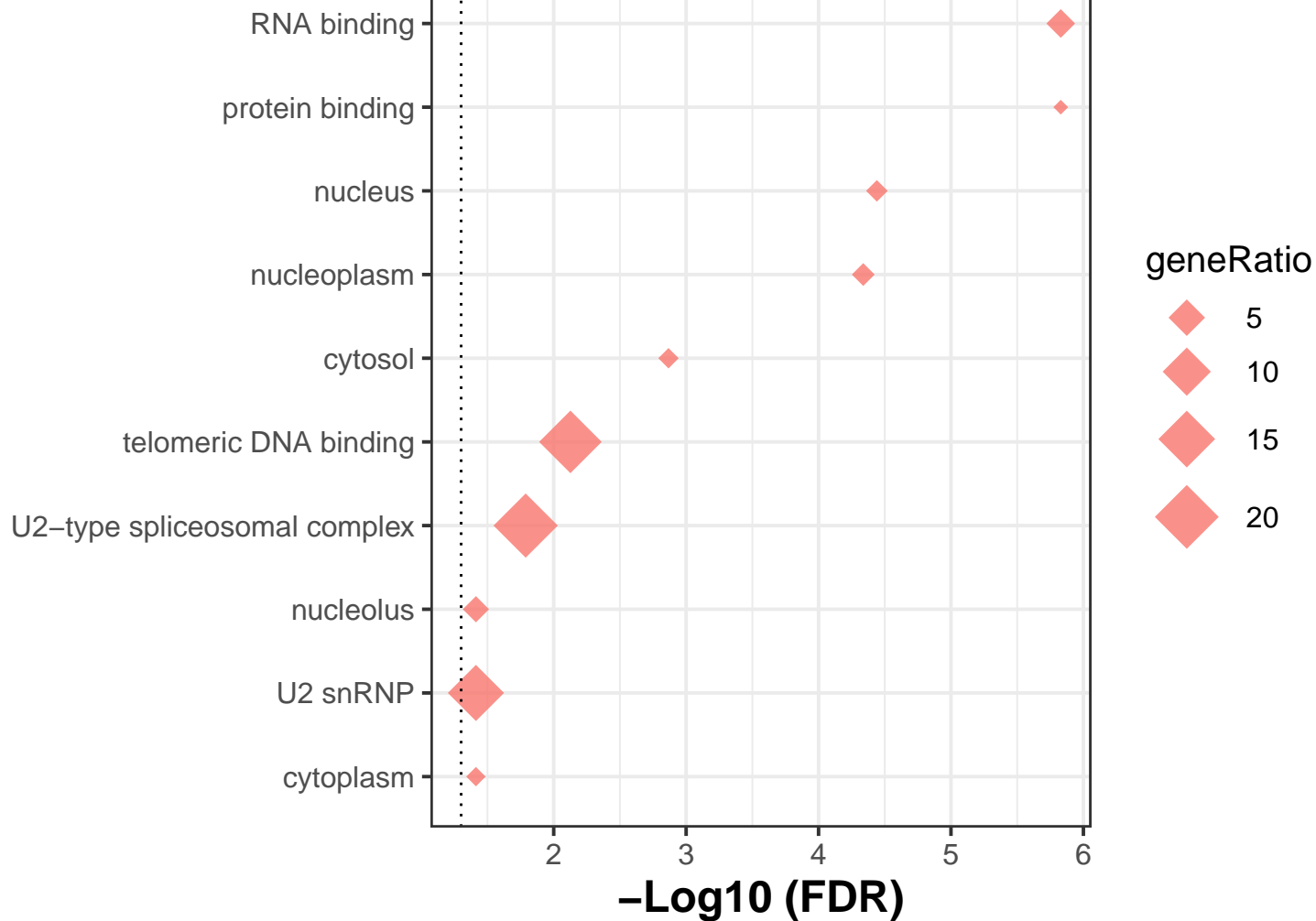

Supplement: Supplementary file 5 — Compressed directory of ancestry-associated DEGs enriched for WGCNA module functional enrichment results (that is, GO term enrichment) for the caudate nucleus, dentate gyrus, DLPFC and hippocampus. [file 41593_2024_1636_MOESM5_ESM.gz › wgcna_functional_enrichment/dlpfc/module_cyan_go_enrichment.pdf]

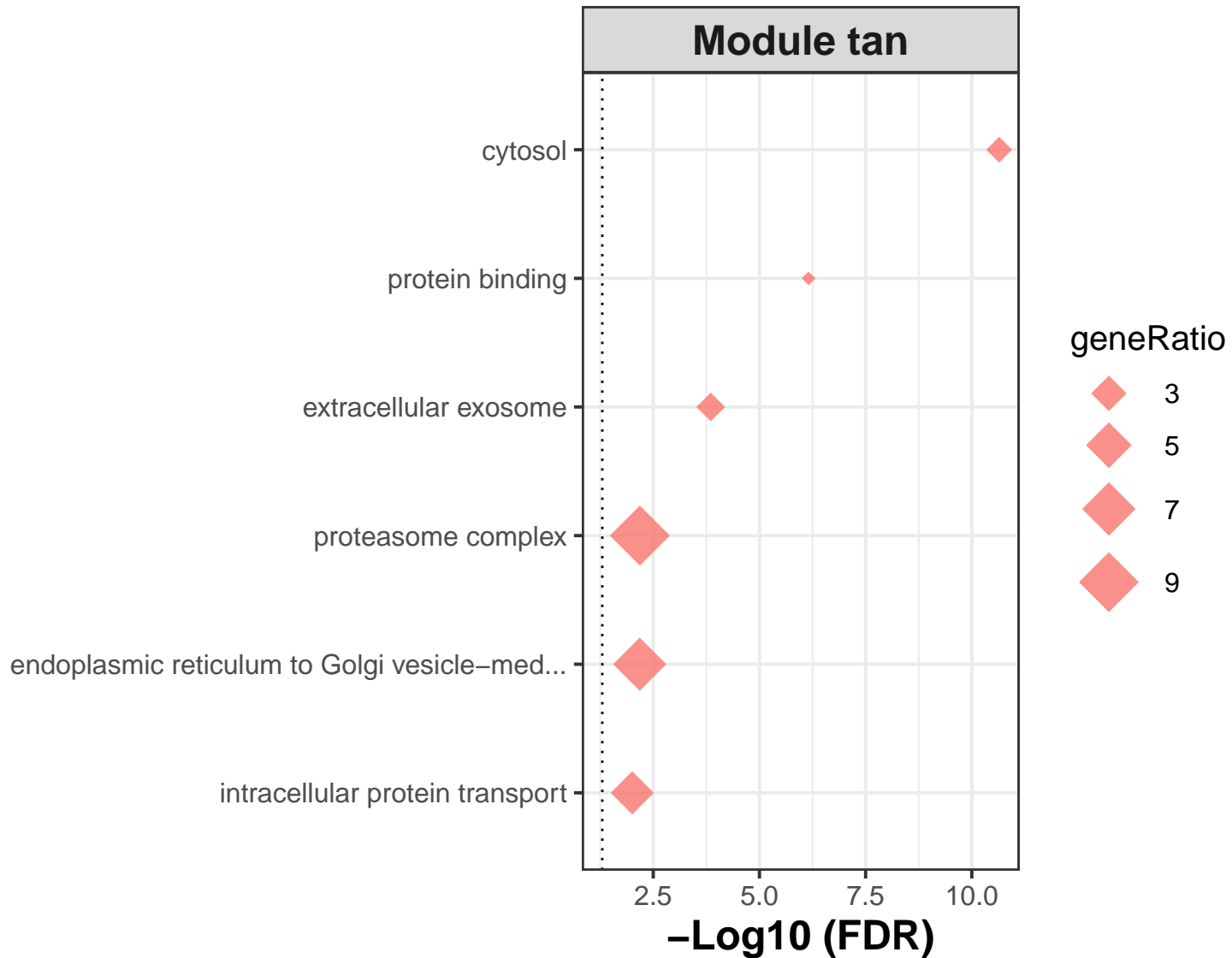

Supplement: Supplementary file 5 — Compressed directory of ancestry-associated DEGs enriched for WGCNA module functional enrichment results (that is, GO term enrichment) for the caudate nucleus, dentate gyrus, DLPFC and hippocampus. [file 41593_2024_1636_MOESM5_ESM.gz › wgcna_functional_enrichment/dlpfc/module_tan_go_enrichment.pdf]

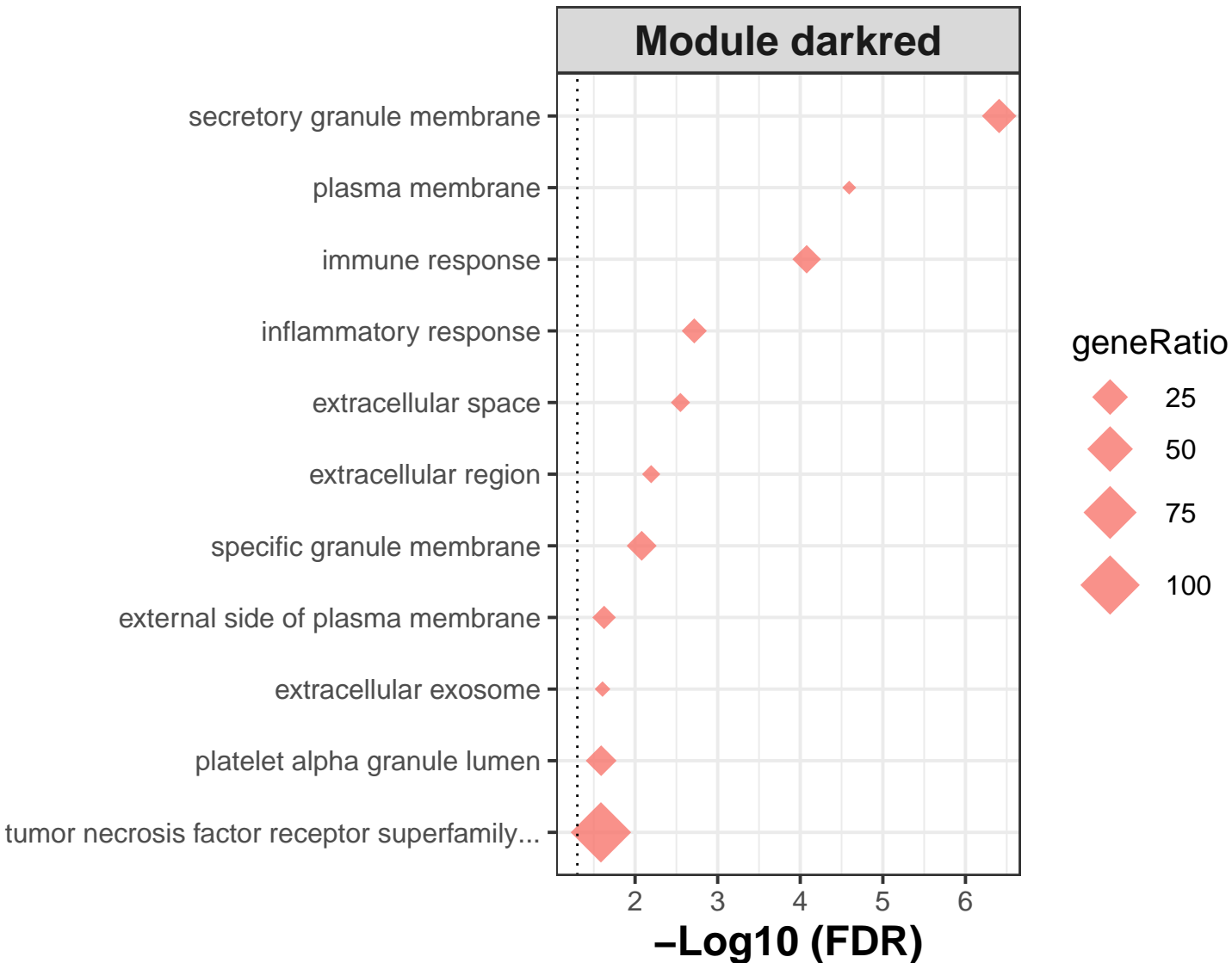

Supplement: Supplementary file 5 — Compressed directory of ancestry-associated DEGs enriched for WGCNA module functional enrichment results (that is, GO term enrichment) for the caudate nucleus, dentate gyrus, DLPFC and hippocampus. [file 41593_2024_1636_MOESM5_ESM.gz › wgcna_functional_enrichment/dlpfc/module_darkred_go_enrichment.pdf]

## Module palette turquoise

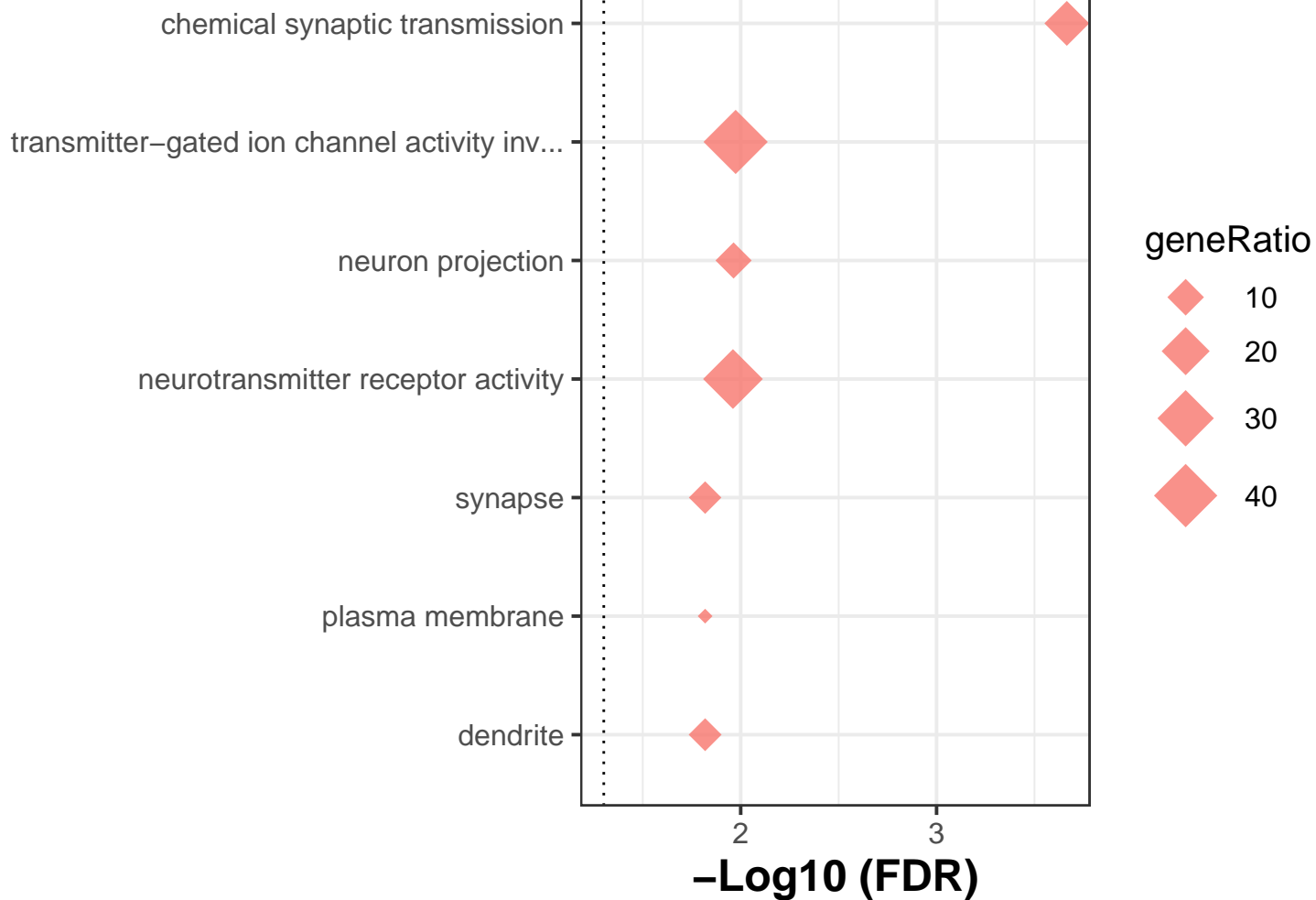

Supplement: Supplementary file 5 — Compressed directory of ancestry-associated DEGs enriched for WGCNA module functional enrichment results (that is, GO term enrichment) for the caudate nucleus, dentate gyrus, DLPFC and hippocampus. [file 41593_2024_1636_MOESM5_ESM.gz › wgcna_functional_enrichment/dlpfc/module_paleturquoise_go_enrichment.pdf]

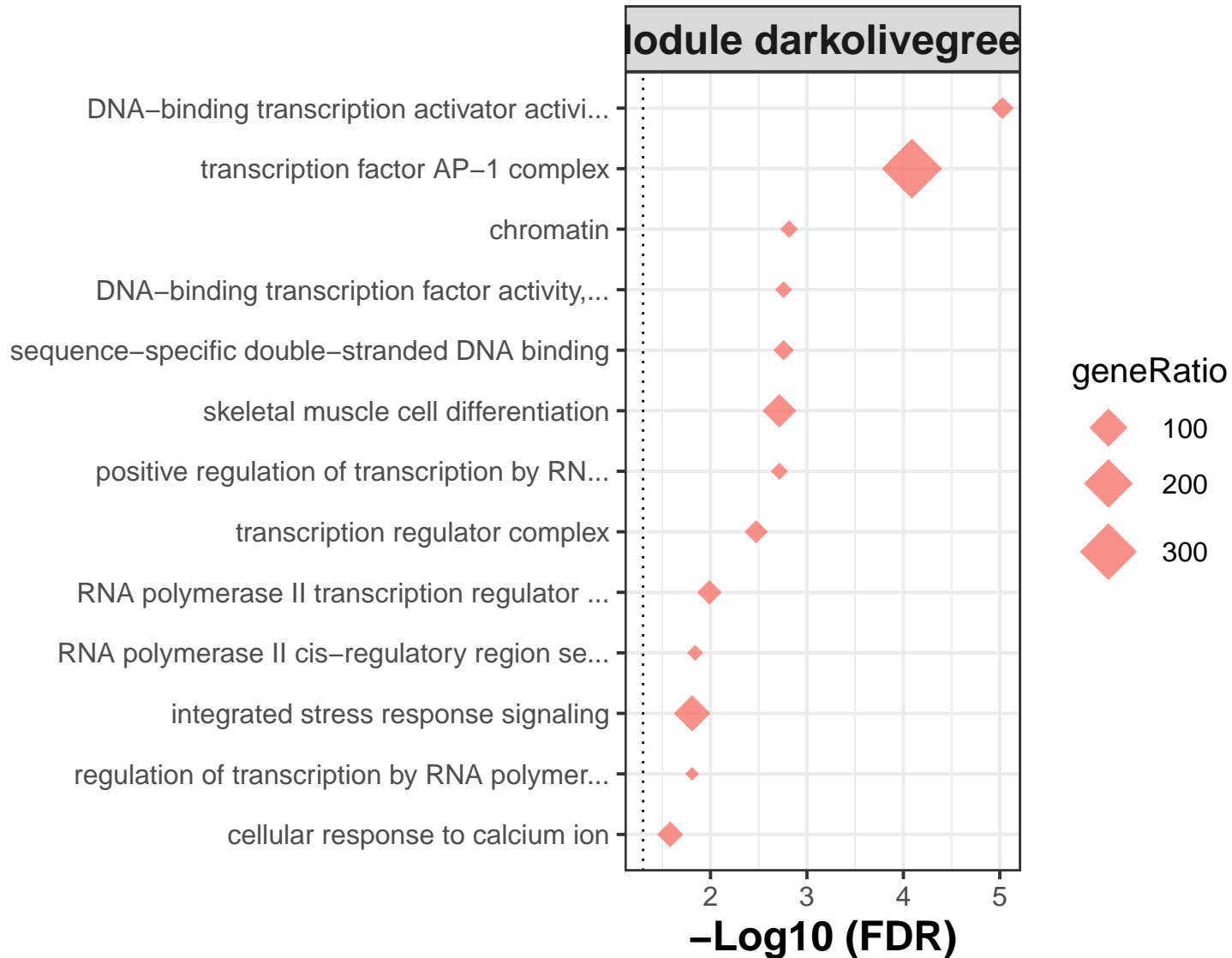

Supplement: Supplementary file 5 — Compressed directory of ancestry-associated DEGs enriched for WGCNA module functional enrichment results (that is, GO term enrichment) for the caudate nucleus, dentate gyrus, DLPFC and hippocampus. [file 41593_2024_1636_MOESM5_ESM.gz › wgcna_functional_enrichment/dlpfc/module_darkolivegreen_go_enrichment.pdf]

## Module darkgrey

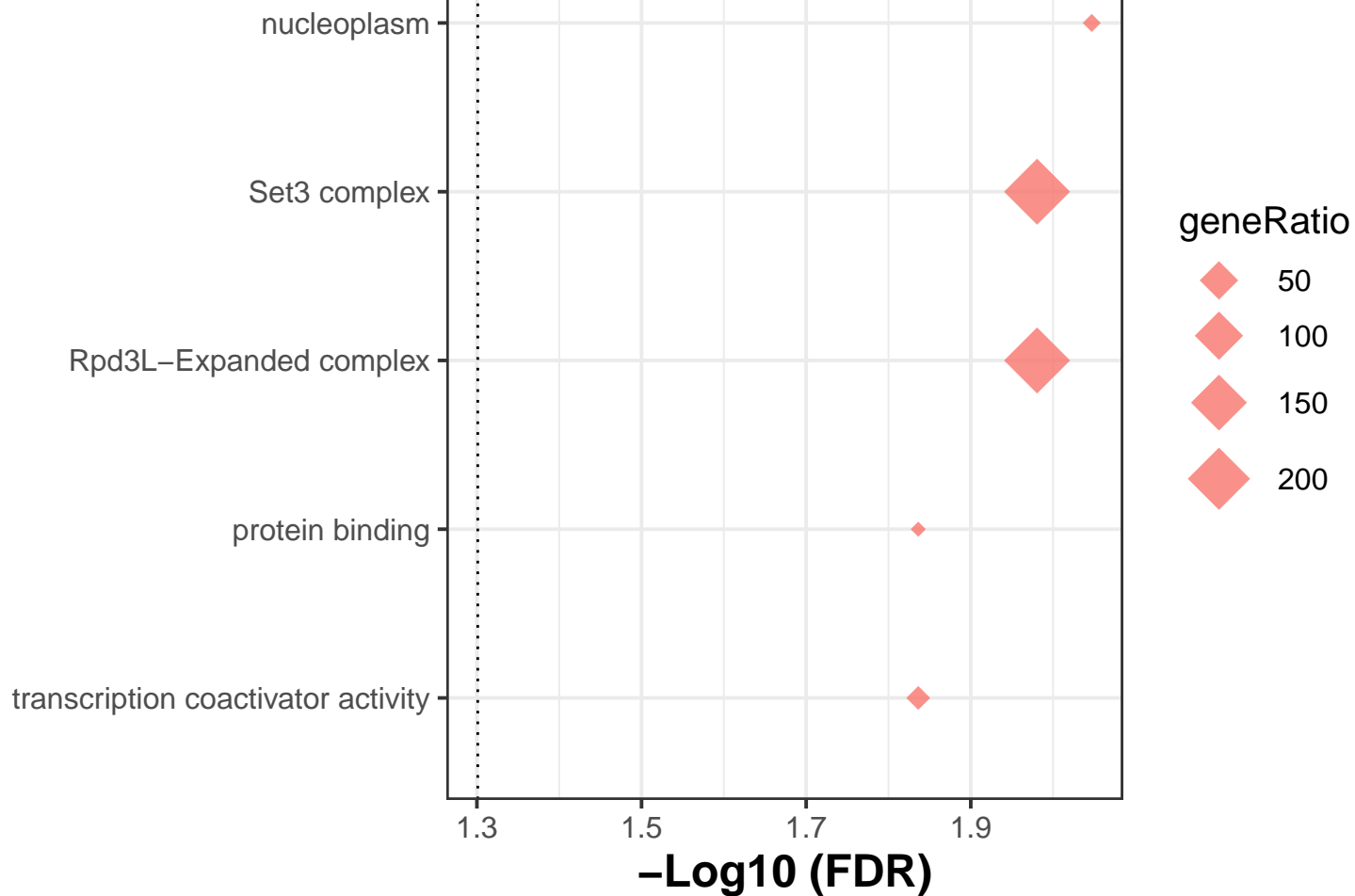

Supplement: Supplementary file 5 — Compressed directory of ancestry-associated DEGs enriched for WGCNA module functional enrichment results (that is, GO term enrichment) for the caudate nucleus, dentate gyrus, DLPFC and hippocampus. [file 41593_2024_1636_MOESM5_ESM.gz › wgcna_functional_enrichment/dlpfc/module_darkgrey_go_enrichment.pdf]

## Module royalblue

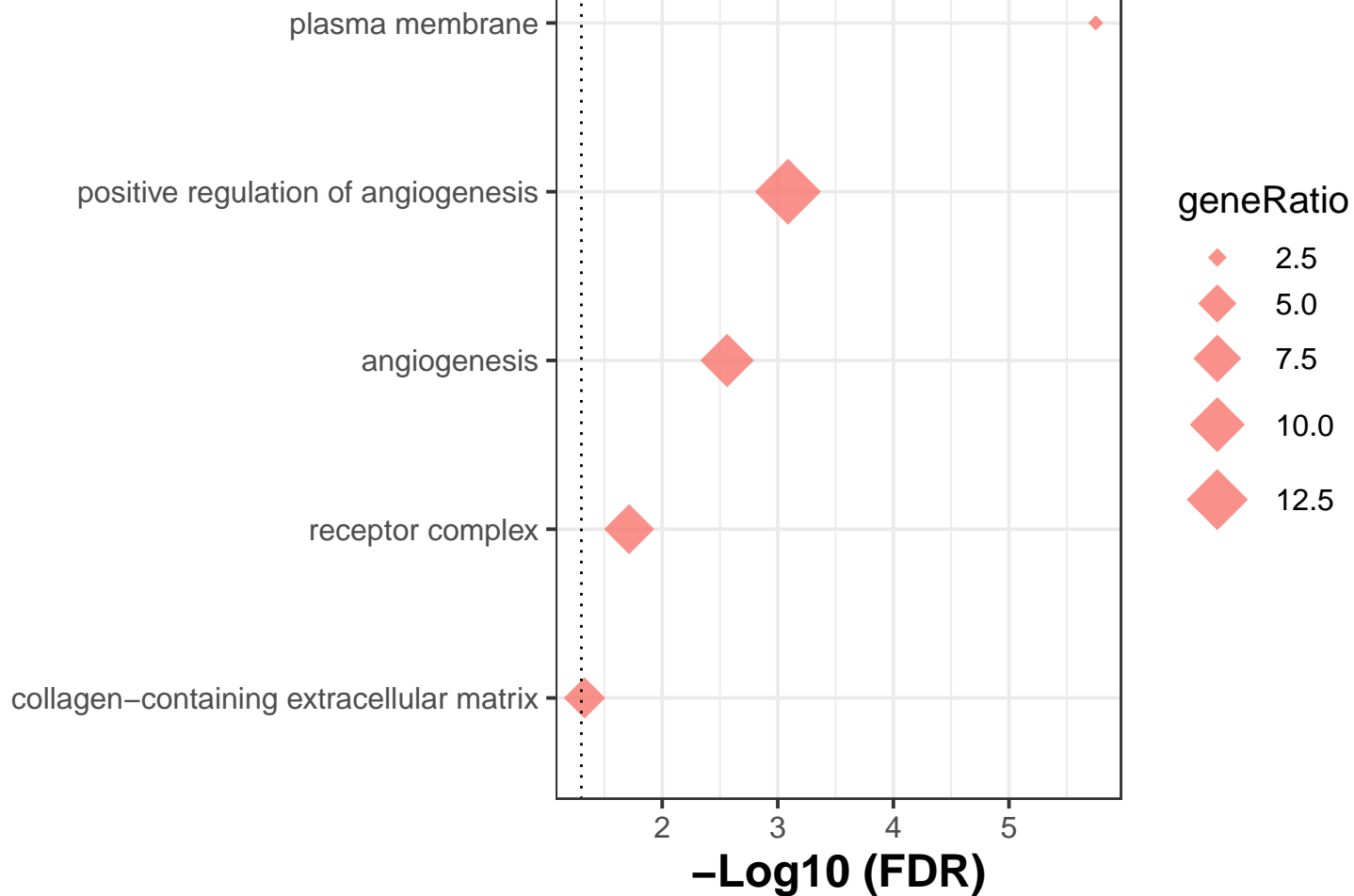

Supplement: Supplementary file 5 — Compressed directory of ancestry-associated DEGs enriched for WGCNA module functional enrichment results (that is, GO term enrichment) for the caudate nucleus, dentate gyrus, DLPFC and hippocampus. [file 41593_2024_1636_MOESM5_ESM.gz › wgcna_functional_enrichment/dlpfc/module_royalblue_go_enrichment.pdf]

## Module darkmagenta

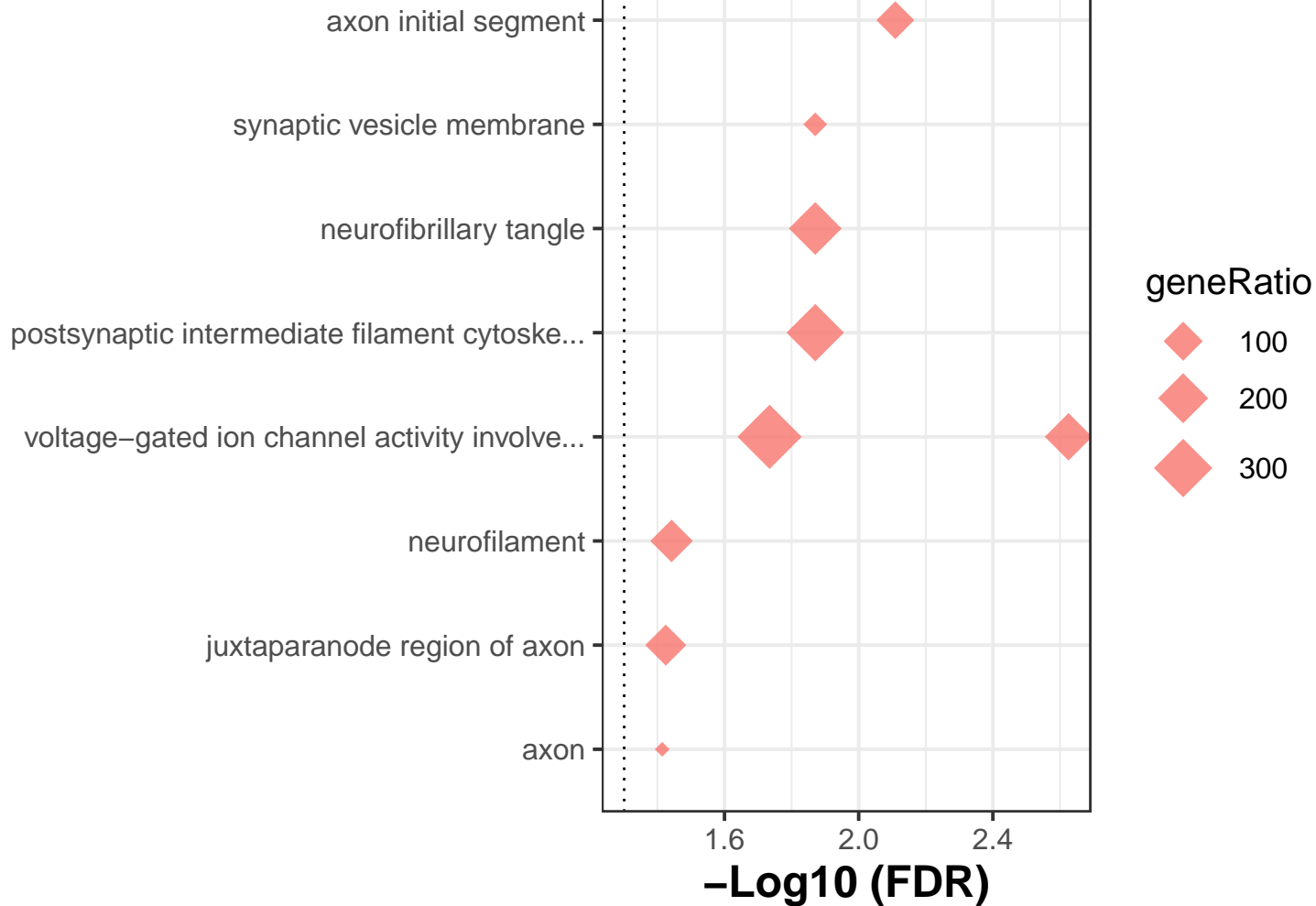

Supplement: Supplementary file 5 — Compressed directory of ancestry-associated DEGs enriched for WGCNA module functional enrichment results (that is, GO term enrichment) for the caudate nucleus, dentate gyrus, DLPFC and hippocampus. [file 41593_2024_1636_MOESM5_ESM.gz › wgcna_functional_enrichment/dlpfc/module_darkmagenta_go_enrichment.pdf]

## Module purple

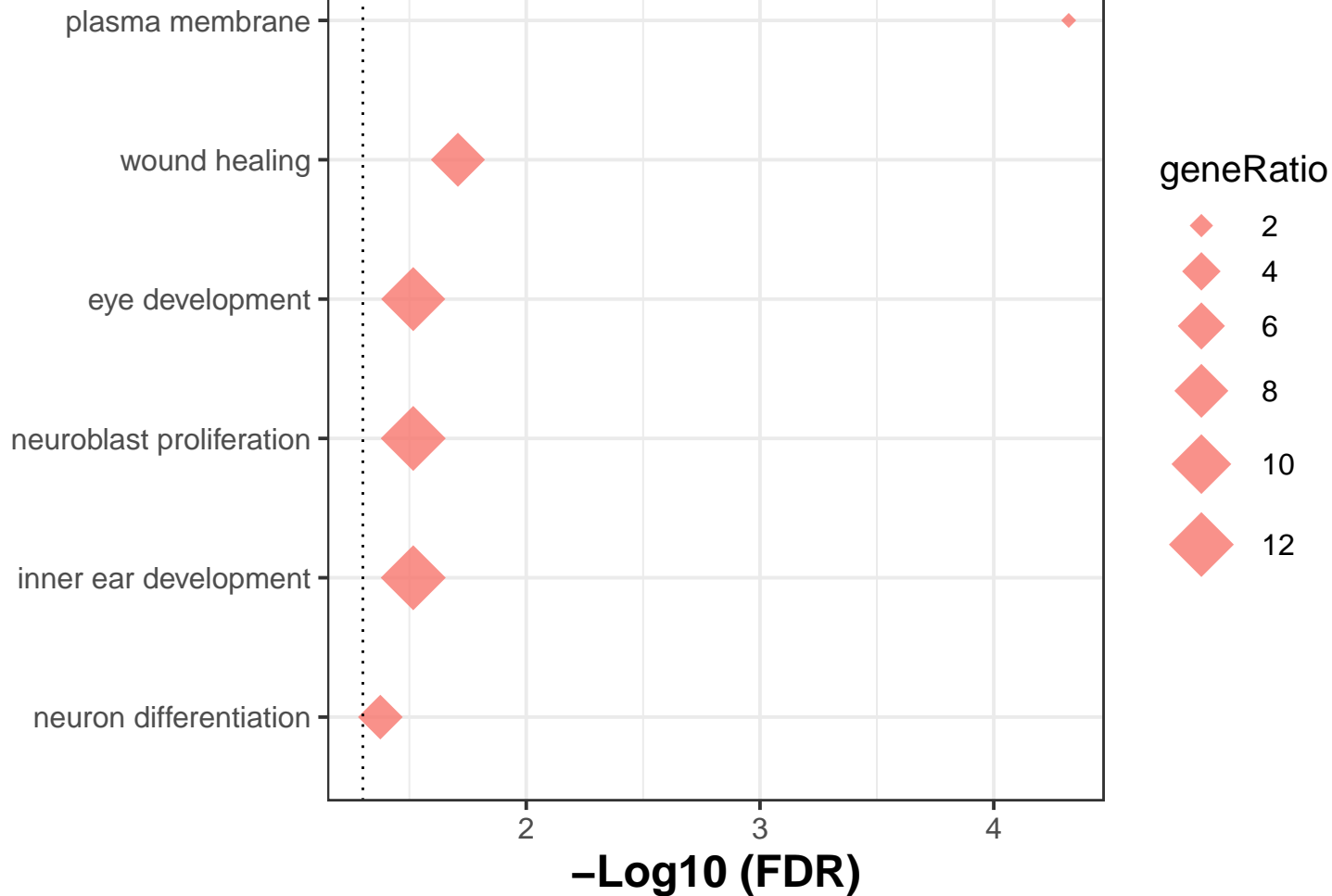

Supplement: Supplementary file 5 — Compressed directory of ancestry-associated DEGs enriched for WGCNA module functional enrichment results (that is, GO term enrichment) for the caudate nucleus, dentate gyrus, DLPFC and hippocampus. [file 41593_2024_1636_MOESM5_ESM.gz › wgcna_functional_enrichment/dlpfc/module_purple_go_enrichment.pdf]

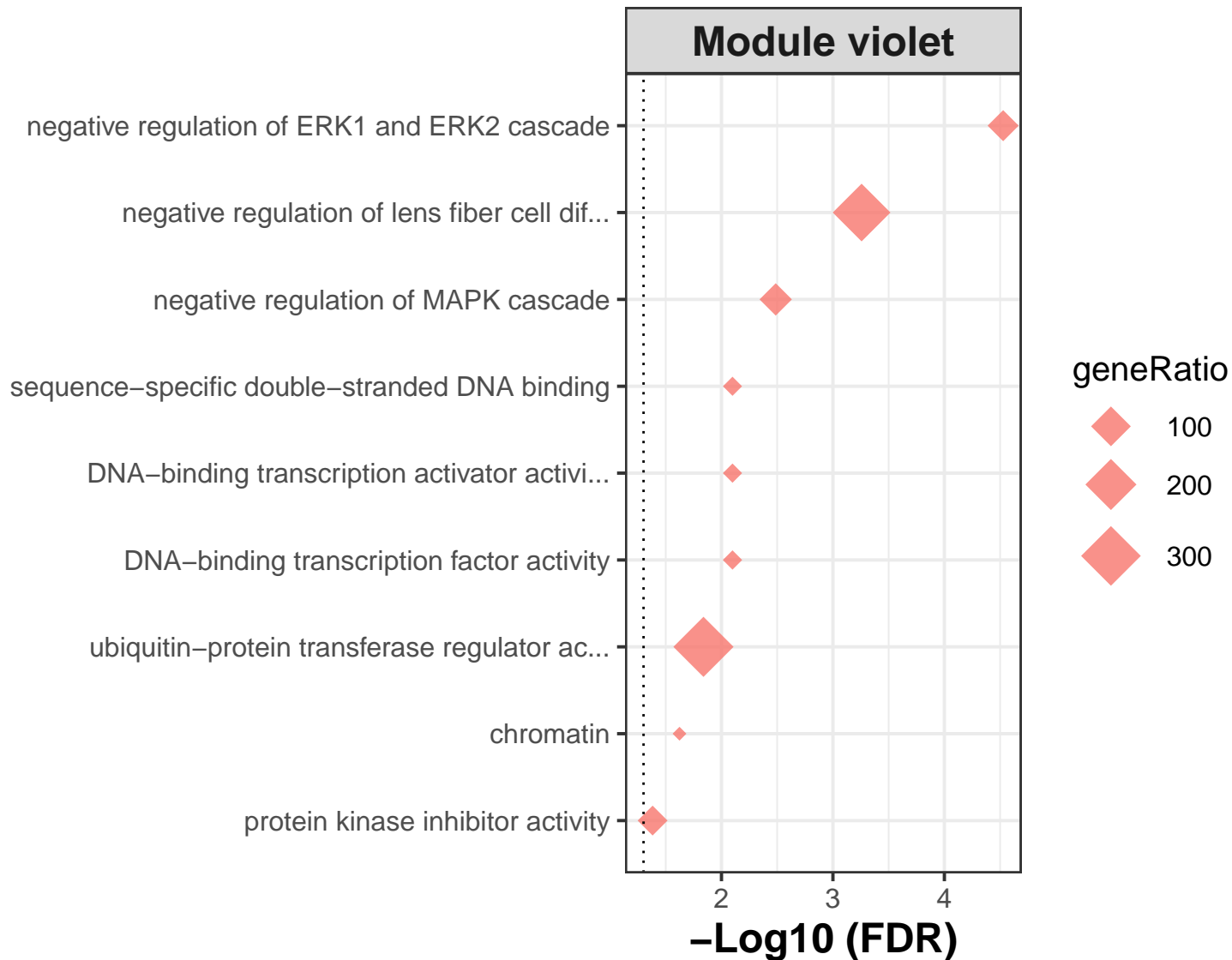

Supplement: Supplementary file 5 — Compressed directory of ancestry-associated DEGs enriched for WGCNA module functional enrichment results (that is, GO term enrichment) for the caudate nucleus, dentate gyrus, DLPFC and hippocampus. [file 41593_2024_1636_MOESM5_ESM.gz › wgcna_functional_enrichment/dlpfc/module_violet_go_enrichment.pdf]

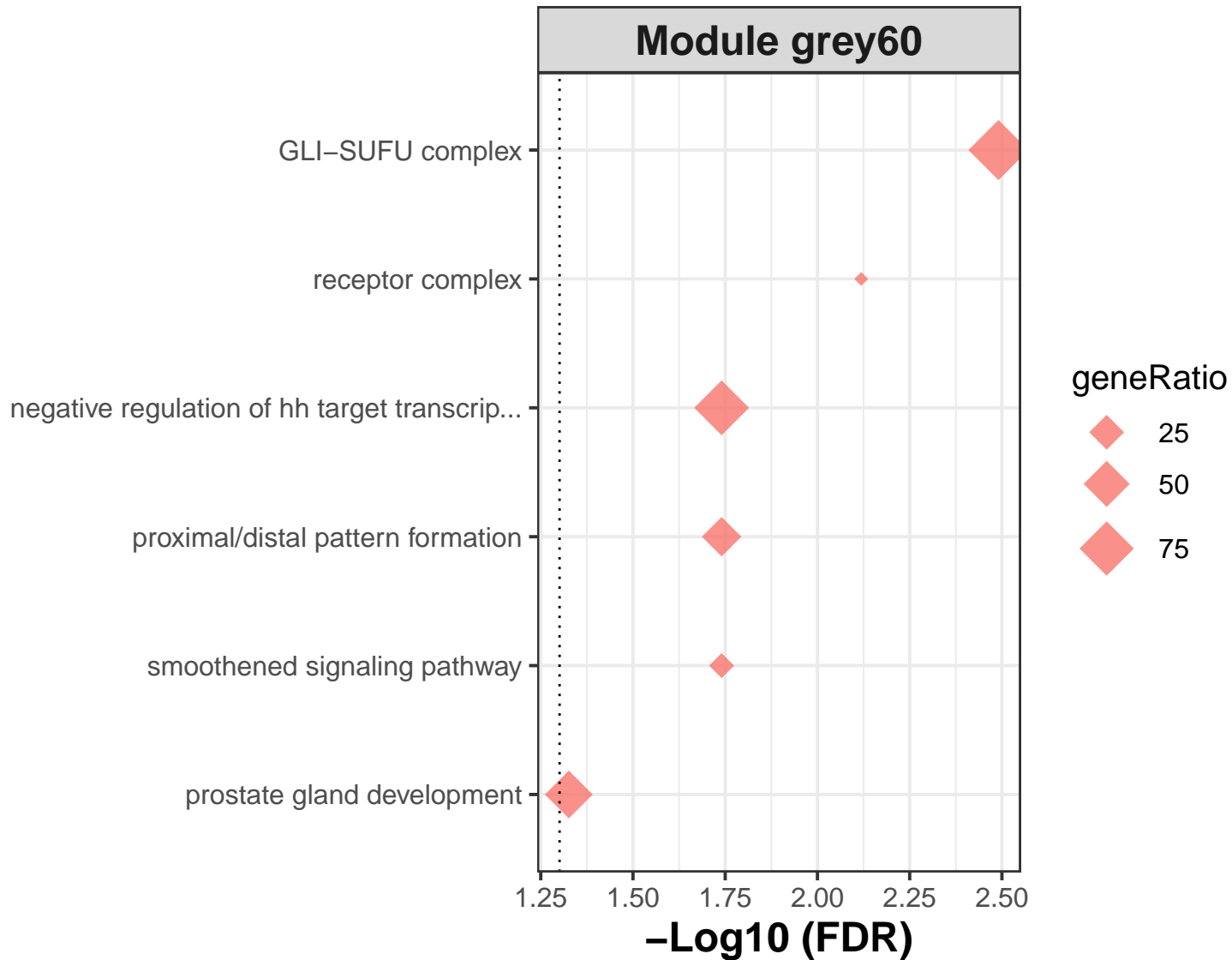

Supplement: Supplementary file 5 — Compressed directory of ancestry-associated DEGs enriched for WGCNA module functional enrichment results (that is, GO term enrichment) for the caudate nucleus, dentate gyrus, DLPFC and hippocampus. [file 41593_2024_1636_MOESM5_ESM.gz › wgcna_functional_enrichment/dlpfc/module_grey60_go_enrichment.pdf]

## Module orange

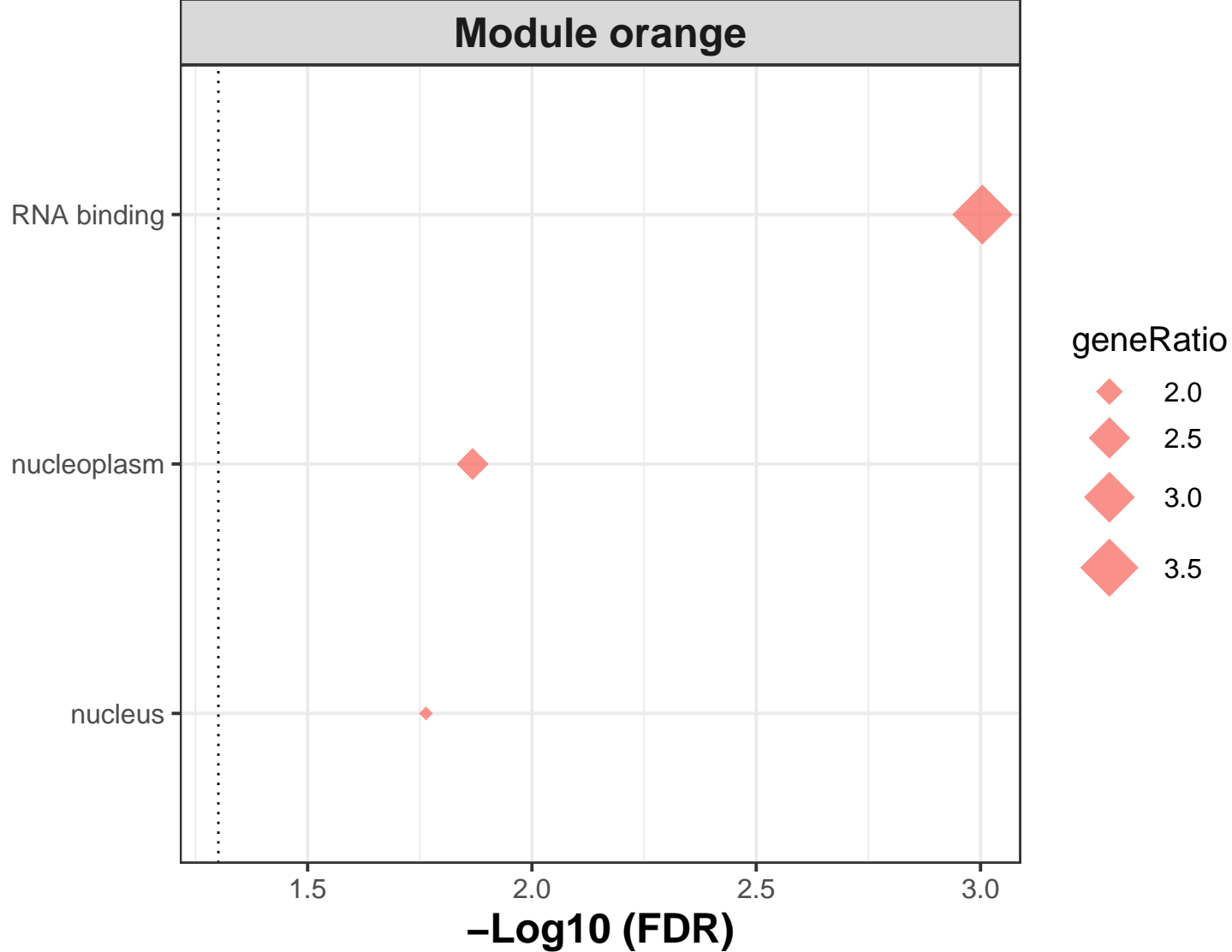

Supplement: Supplementary file 5 — Compressed directory of ancestry-associated DEGs enriched for WGCNA module functional enrichment results (that is, GO term enrichment) for the caudate nucleus, dentate gyrus, DLPFC and hippocampus. [file 41593_2024_1636_MOESM5_ESM.gz › wgcna_functional_enrichment/dlpfc/module_orange_go_enrichment.pdf]

## Module yellow

protein binding

geneRatio

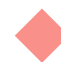

1.168444

1.50

1.75

2.00

2.25

**$-\text{Log}_{10}(\text{FDR})$**

Supplement: Supplementary file 5 — Compressed directory of ancestry-associated DEGs enriched for WGCNA module functional enrichment results (that is, GO term enrichment) for the caudate nucleus, dentate gyrus, DLPFC and hippocampus. [file 41593_2024_1636_MOESM5_ESM.gz › wgcna_functional_enrichment/dlpfc/module_yellow_go_enrichment.pdf]

## Module turquoise

plasma membrane

geneRatio

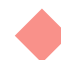

1.172992

1.301

1.302

1.303

1.304

1.305

1.306

**$-\text{Log}_{10}(\text{FDR})$**

Supplement: Supplementary file 5 — Compressed directory of ancestry-associated DEGs enriched for WGCNA module functional enrichment results (that is, GO term enrichment) for the caudate nucleus, dentate gyrus, DLPFC and hippocampus. [file 41593_2024_1636_MOESM5_ESM.gz › wgcna_functional_enrichment/dlpfc/module_turquoise_go_enrichment.pdf]

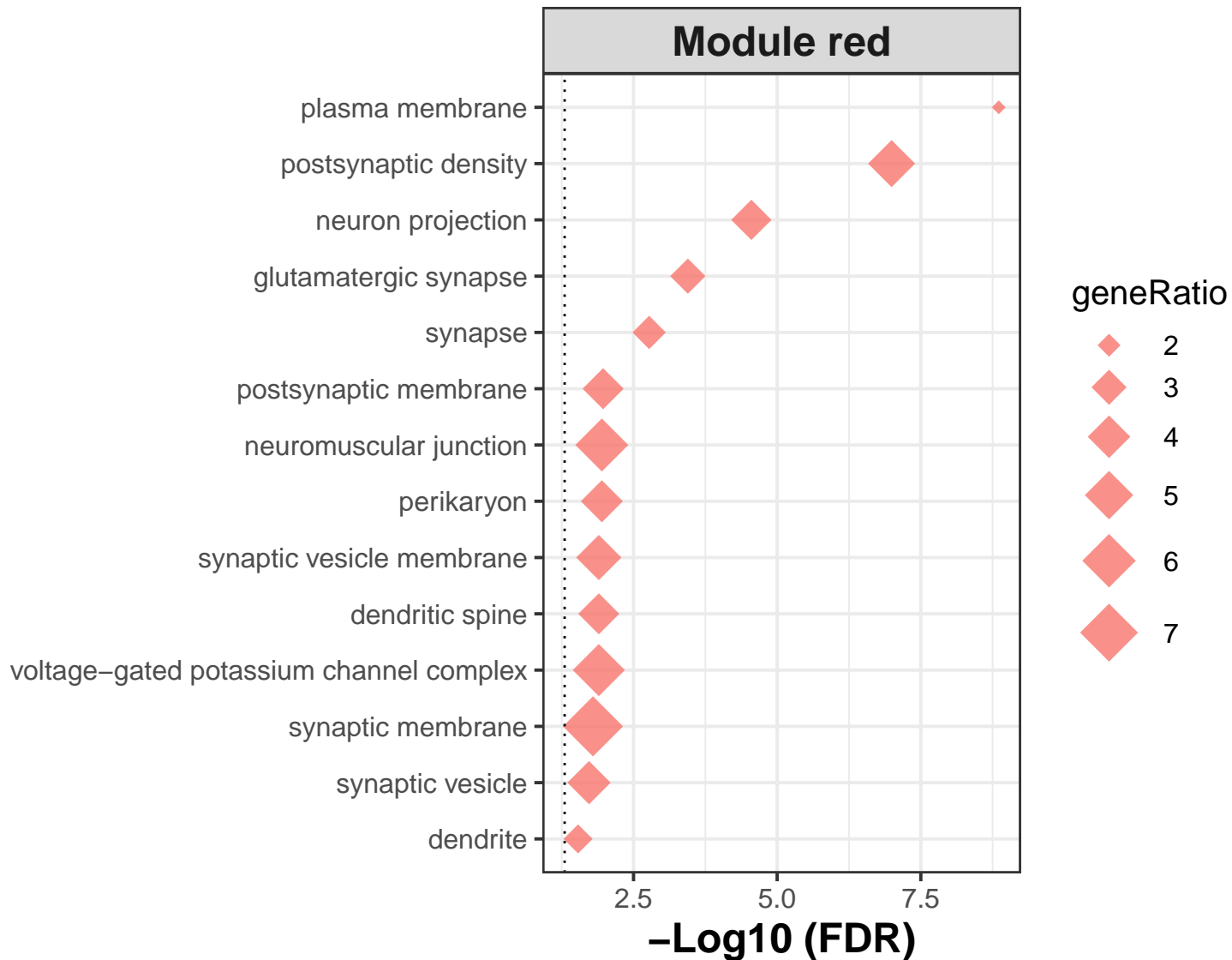

Supplement: Supplementary file 5 — Compressed directory of ancestry-associated DEGs enriched for WGCNA module functional enrichment results (that is, GO term enrichment) for the caudate nucleus, dentate gyrus, DLPFC and hippocampus. [file 41593_2024_1636_MOESM5_ESM.gz › wgcna_functional_enrichment/dlpfc/module_red_go_enrichment.pdf]

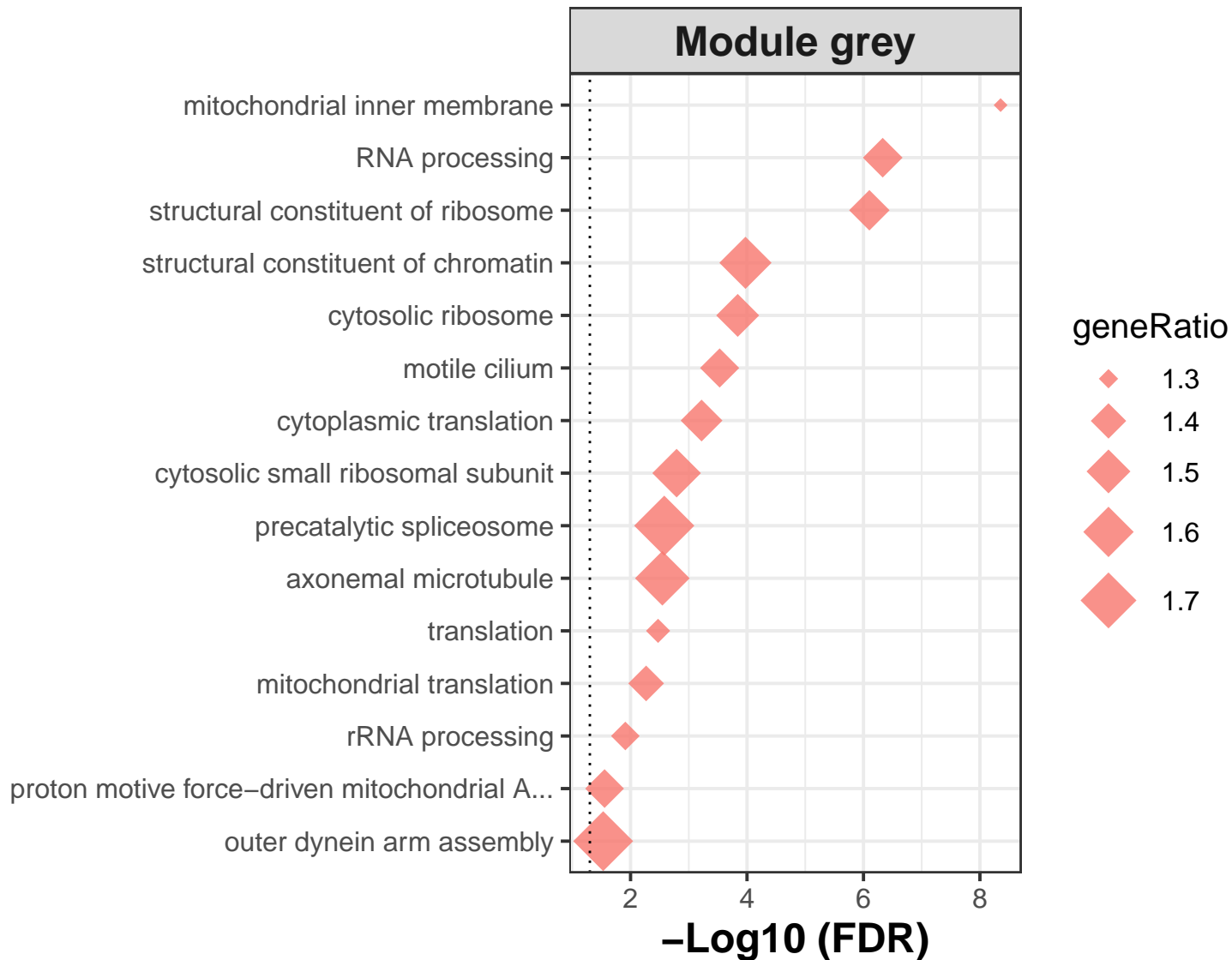

Supplement: Supplementary file 5 — Compressed directory of ancestry-associated DEGs enriched for WGCNA module functional enrichment results (that is, GO term enrichment) for the caudate nucleus, dentate gyrus, DLPFC and hippocampus. [file 41593_2024_1636_MOESM5_ESM.gz › wgcna_functional_enrichment/dlpfc/module_grey_go_enrichment.pdf]

## Module saddlebrown

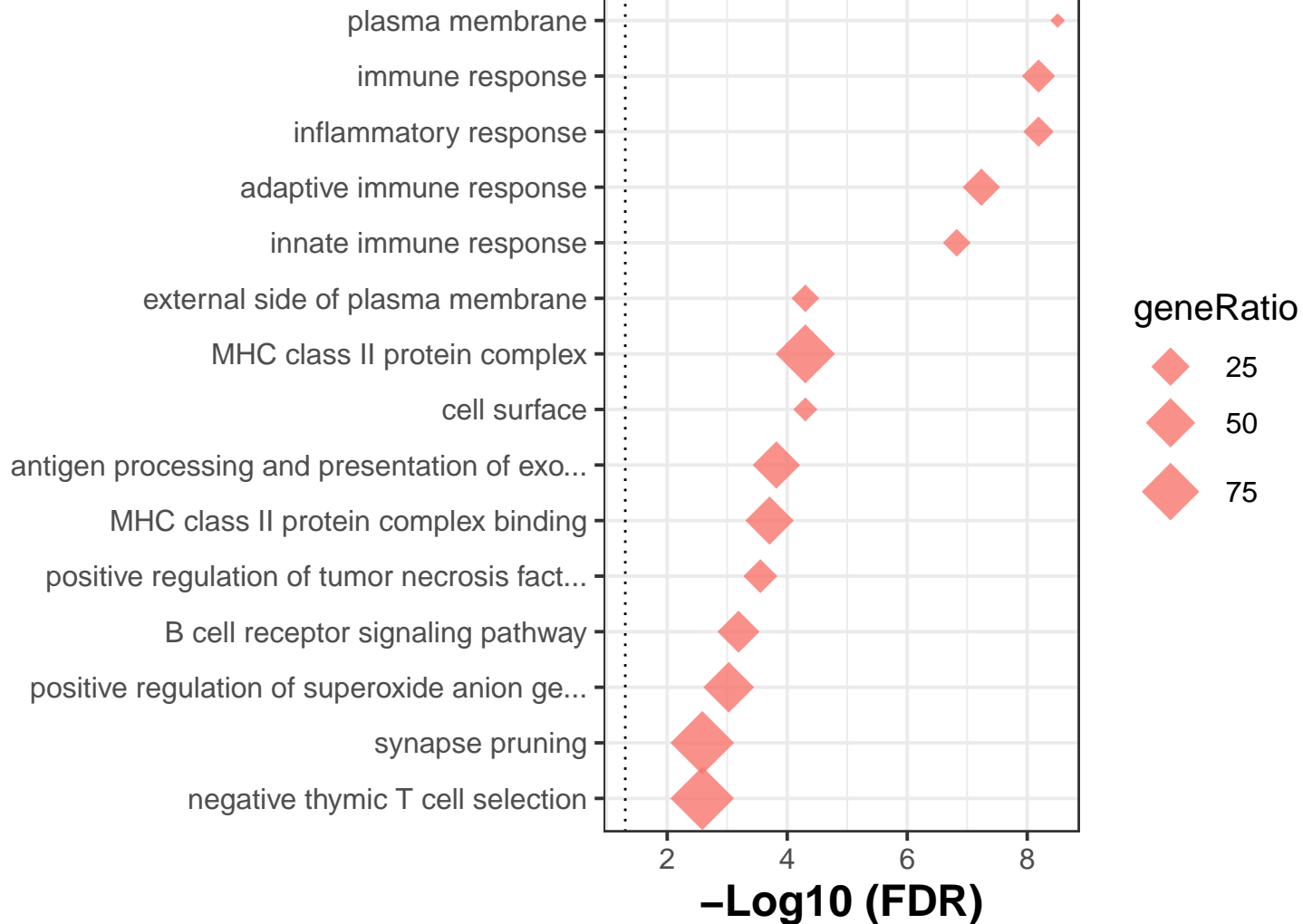

Supplement: Supplementary file 5 — Compressed directory of ancestry-associated DEGs enriched for WGCNA module functional enrichment results (that is, GO term enrichment) for the caudate nucleus, dentate gyrus, DLPFC and hippocampus. [file 41593_2024_1636_MOESM5_ESM.gz › wgcna_functional_enrichment/dentateGyrus/module_saddlebrown_go_enrichment.pdf]

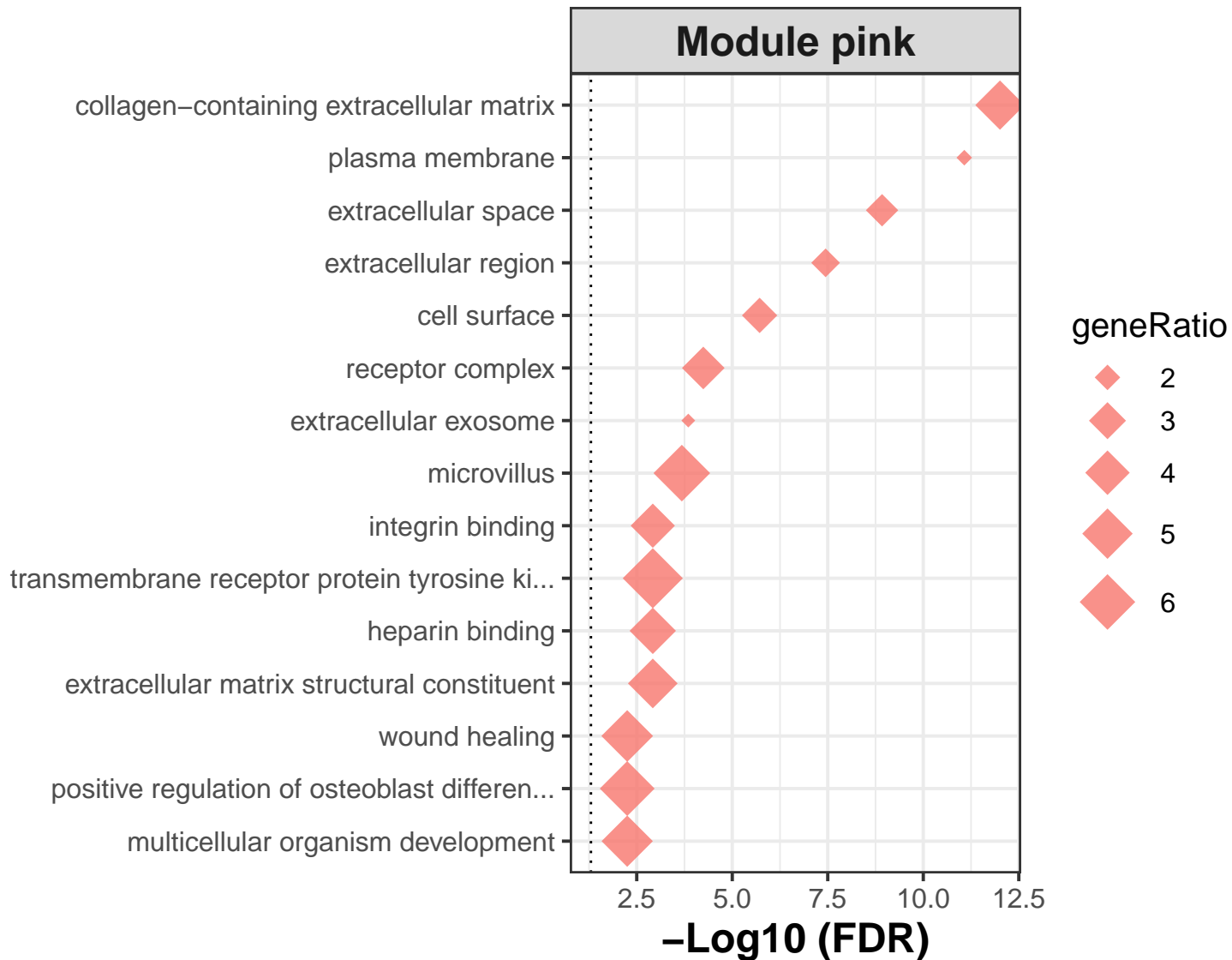

Supplement: Supplementary file 5 — Compressed directory of ancestry-associated DEGs enriched for WGCNA module functional enrichment results (that is, GO term enrichment) for the caudate nucleus, dentate gyrus, DLPFC and hippocampus. [file 41593_2024_1636_MOESM5_ESM.gz › wgcna_functional_enrichment/dentateGyrus/module_pink_go_enrichment.pdf]

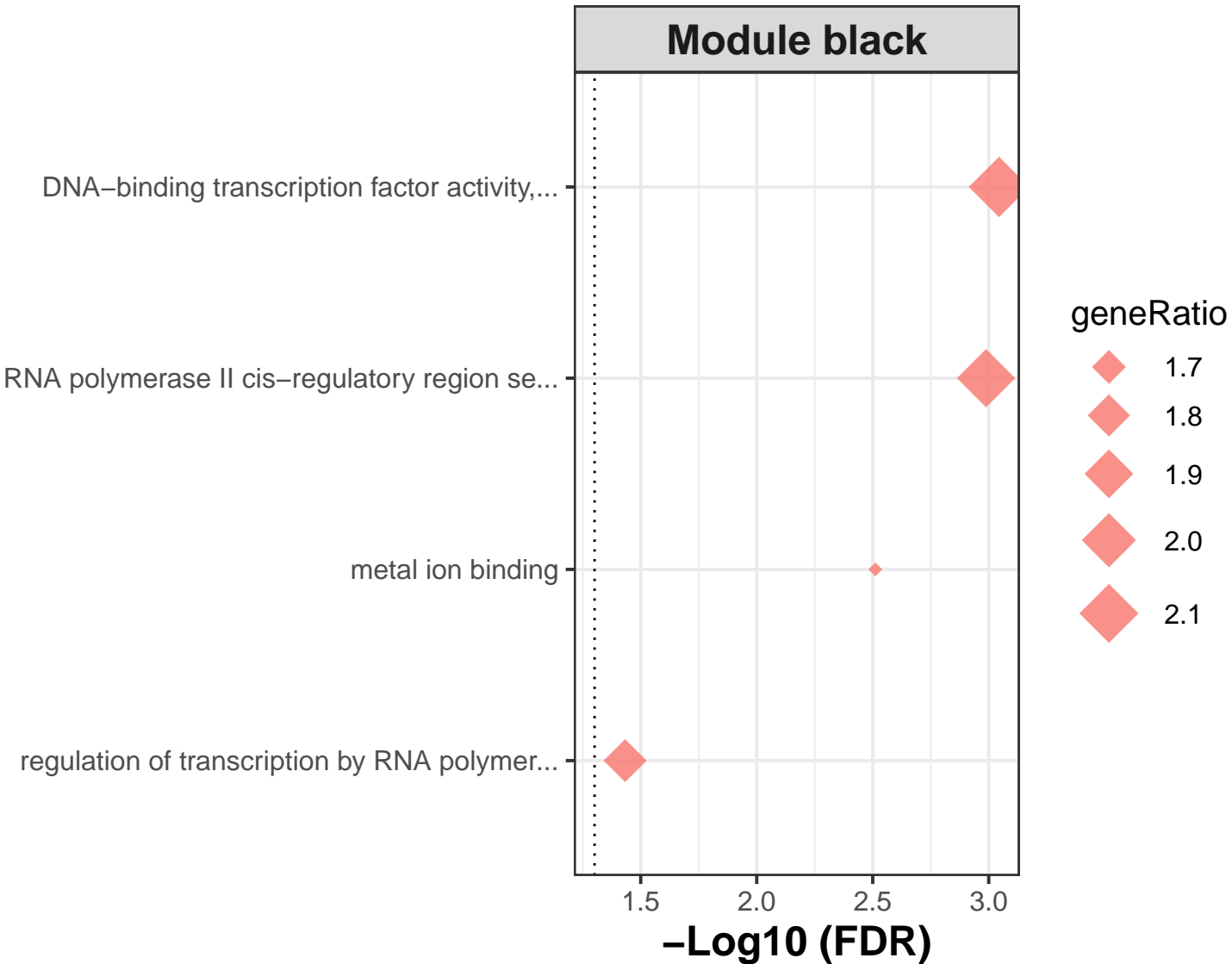

Supplement: Supplementary file 5 — Compressed directory of ancestry-associated DEGs enriched for WGCNA module functional enrichment results (that is, GO term enrichment) for the caudate nucleus, dentate gyrus, DLPFC and hippocampus. [file 41593_2024_1636_MOESM5_ESM.gz › wgcna_functional_enrichment/dentateGyrus/module_black_go_enrichment.pdf]

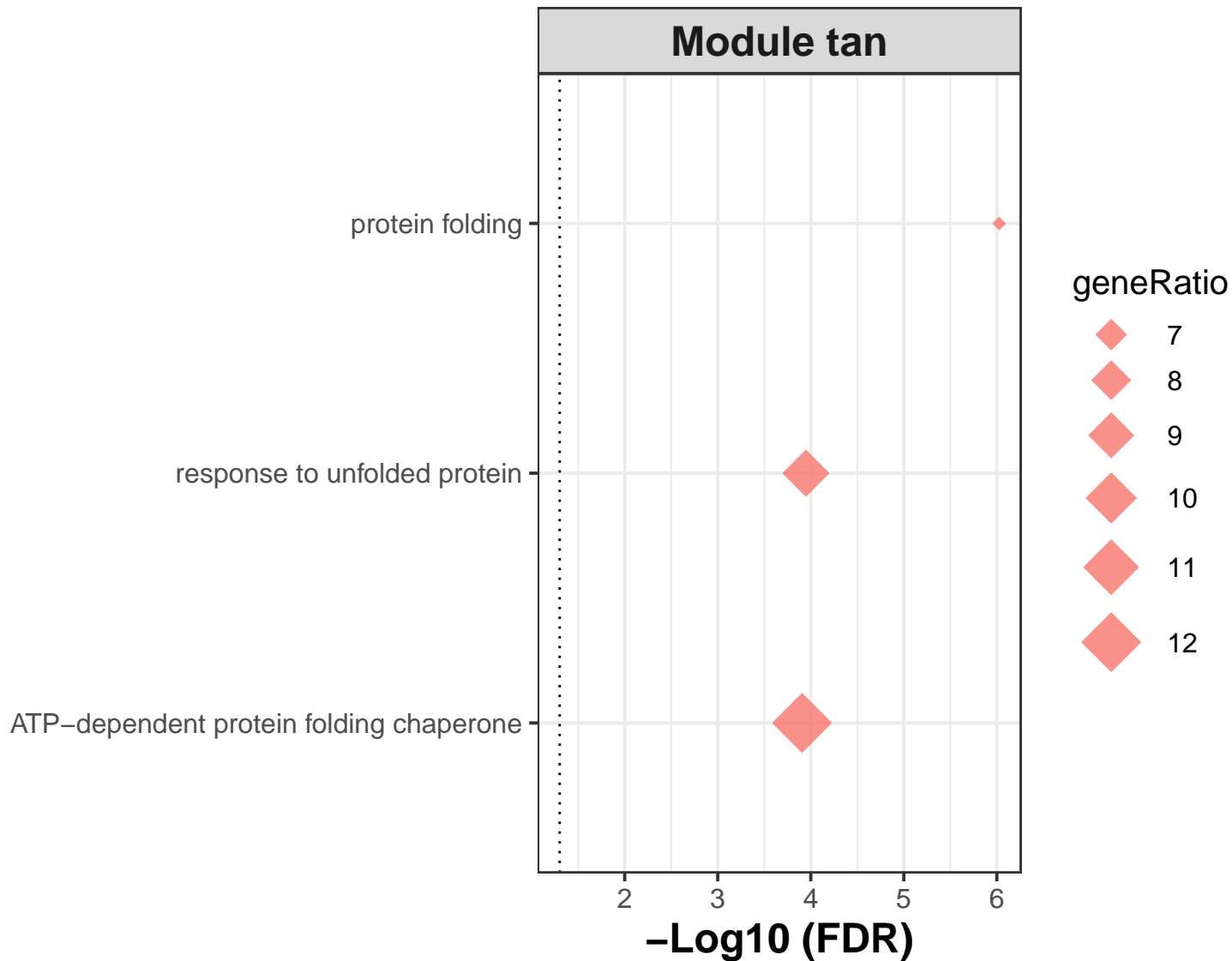

Supplement: Supplementary file 5 — Compressed directory of ancestry-associated DEGs enriched for WGCNA module functional enrichment results (that is, GO term enrichment) for the caudate nucleus, dentate gyrus, DLPFC and hippocampus. [file 41593_2024_1636_MOESM5_ESM.gz › wgcna_functional_enrichment/dentateGyrus/module_tan_go_enrichment.pdf]

## Module brown

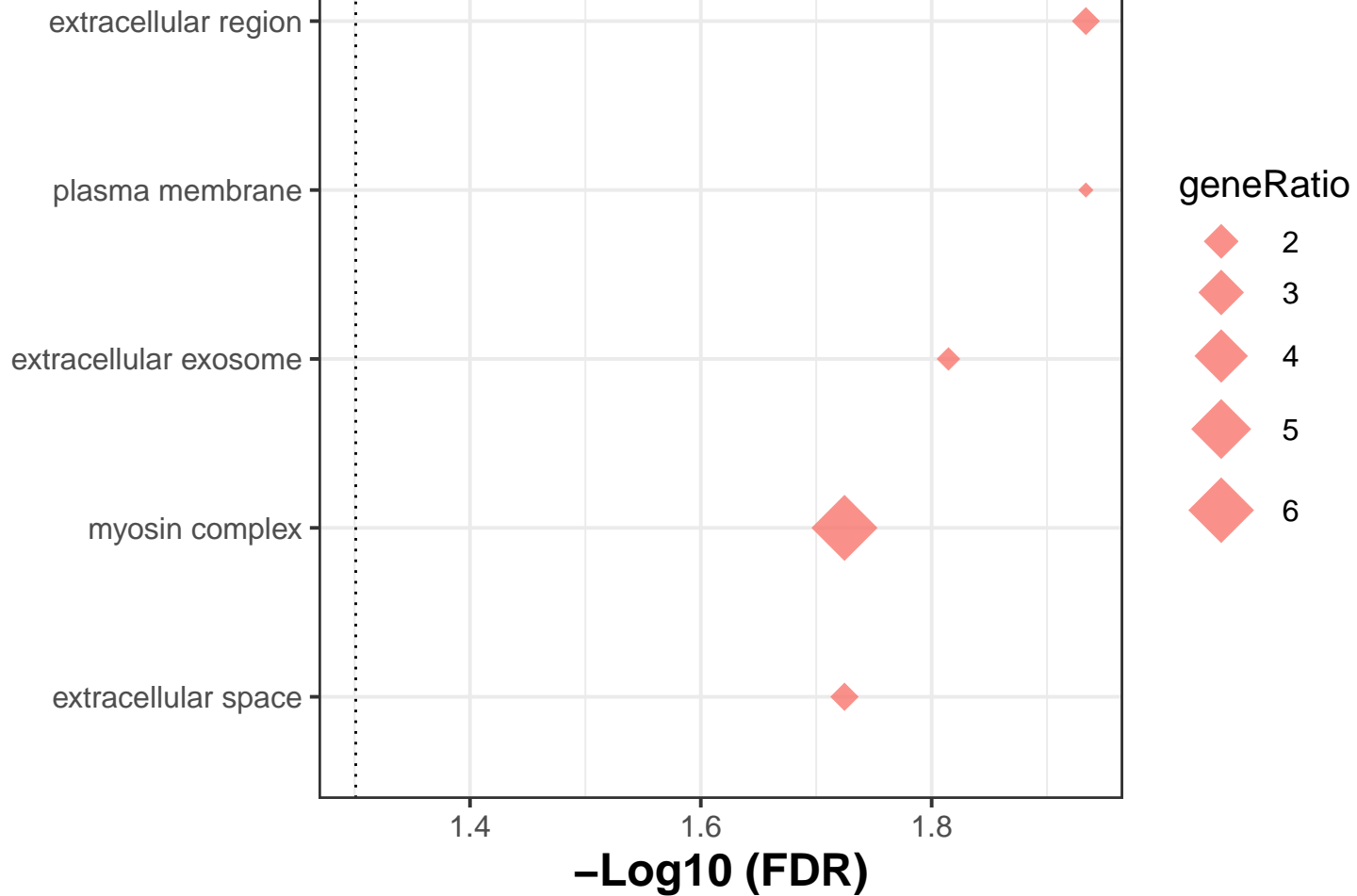

Supplement: Supplementary file 5 — Compressed directory of ancestry-associated DEGs enriched for WGCNA module functional enrichment results (that is, GO term enrichment) for the caudate nucleus, dentate gyrus, DLPFC and hippocampus. [file 41593_2024_1636_MOESM5_ESM.gz › wgcna_functional_enrichment/dentateGyrus/module_brown_go_enrichment.pdf]

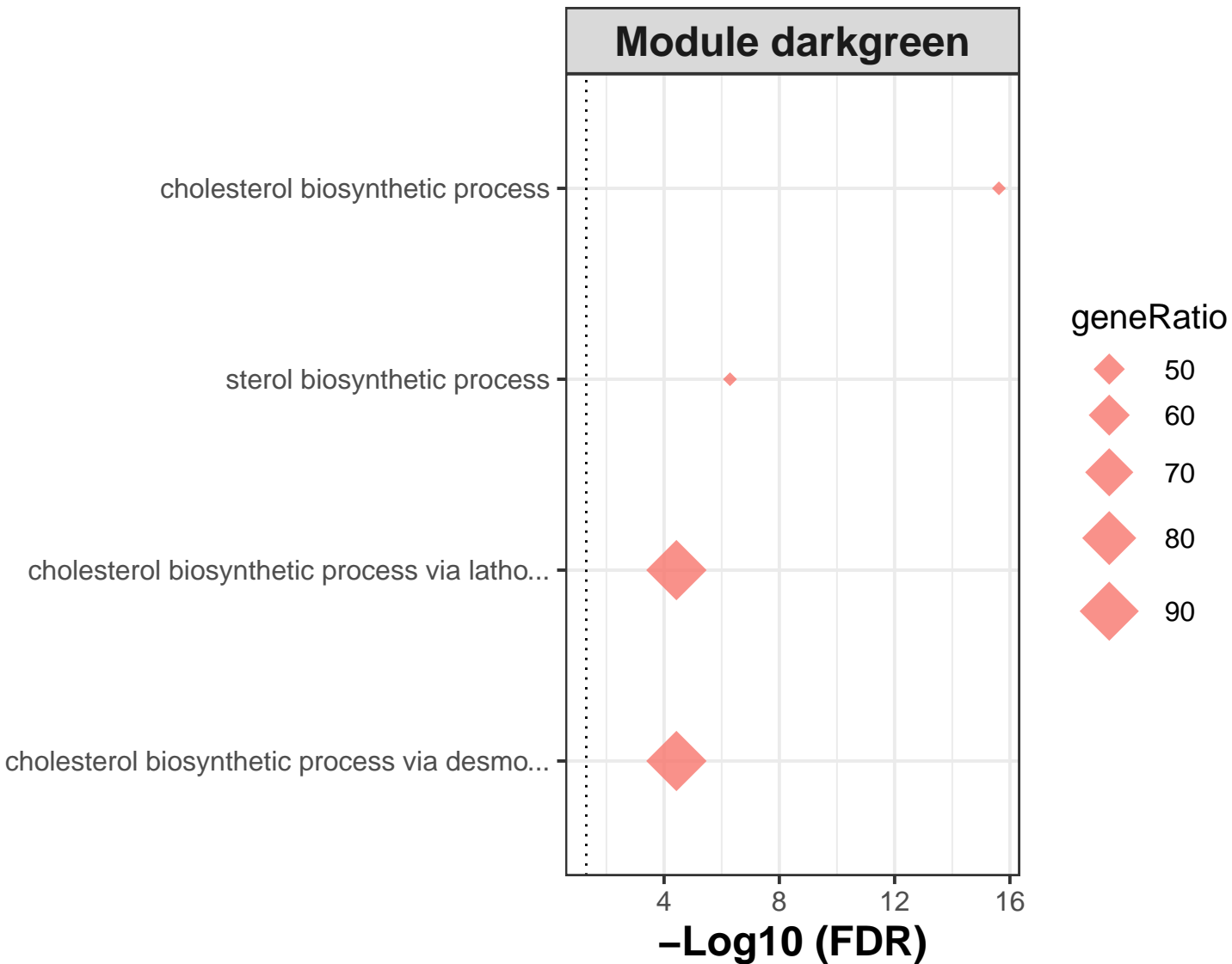

Supplement: Supplementary file 5 — Compressed directory of ancestry-associated DEGs enriched for WGCNA module functional enrichment results (that is, GO term enrichment) for the caudate nucleus, dentate gyrus, DLPFC and hippocampus. [file 41593_2024_1636_MOESM5_ESM.gz › wgcna_functional_enrichment/dentateGyrus/module_darkgreen_go_enrichment.pdf]

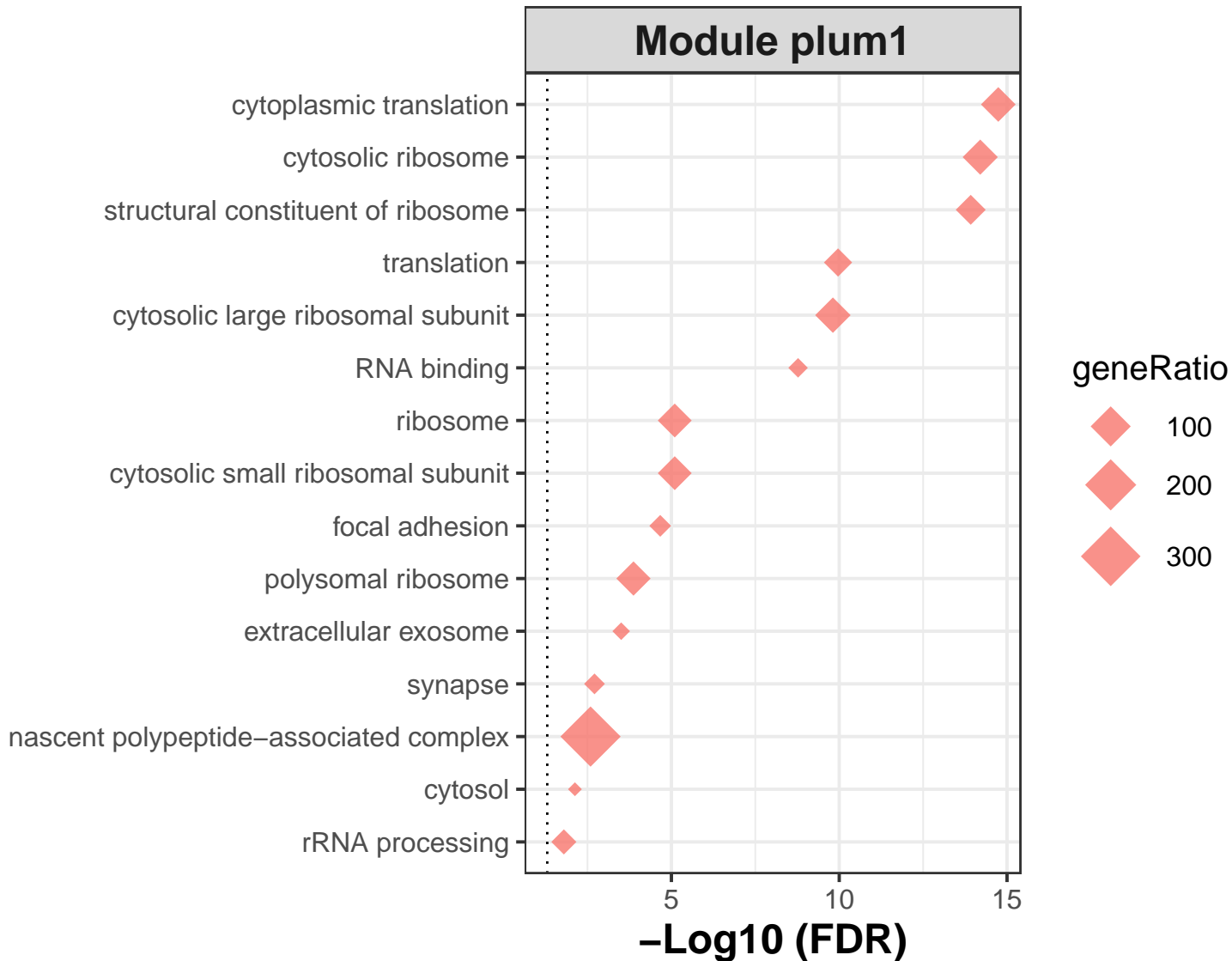

Supplement: Supplementary file 5 — Compressed directory of ancestry-associated DEGs enriched for WGCNA module functional enrichment results (that is, GO term enrichment) for the caudate nucleus, dentate gyrus, DLPFC and hippocampus. [file 41593_2024_1636_MOESM5_ESM.gz › wgcna_functional_enrichment/dentateGyrus/module_plum1_go_enrichment.pdf]

## Module greenyellow

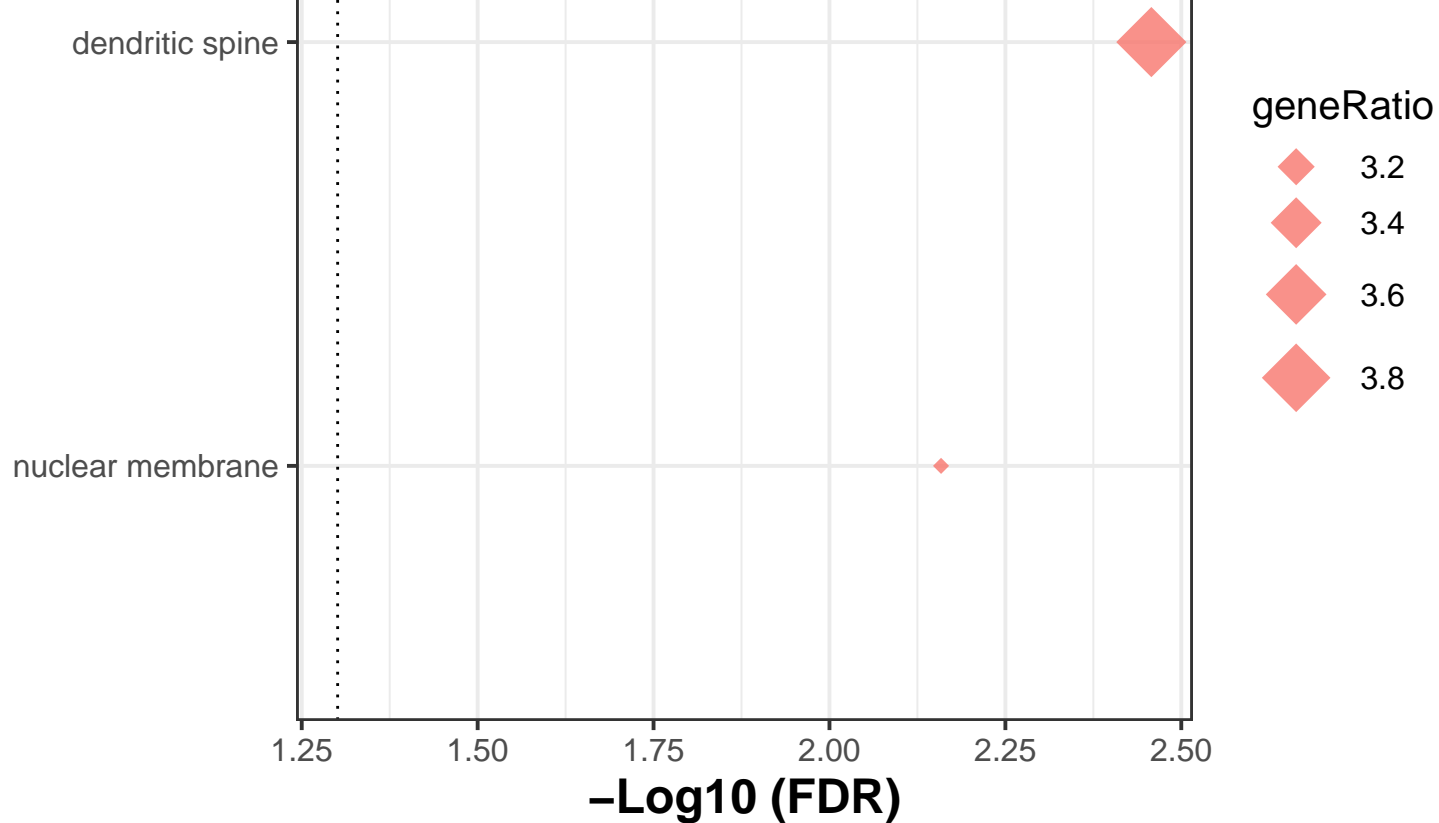

Supplement: Supplementary file 5 — Compressed directory of ancestry-associated DEGs enriched for WGCNA module functional enrichment results (that is, GO term enrichment) for the caudate nucleus, dentate gyrus, DLPFC and hippocampus. [file 41593_2024_1636_MOESM5_ESM.gz › wgcna_functional_enrichment/dentateGyrus/module_greenyellow_go_enrichment.pdf]

## Module red

lysosomal membrane

protein binding

geneRatio

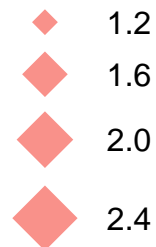

2

3

4

$-\text{Log}_{10}(\text{FDR})$

Supplement: Supplementary file 5 — Compressed directory of ancestry-associated DEGs enriched for WGCNA module functional enrichment results (that is, GO term enrichment) for the caudate nucleus, dentate gyrus, DLPFC and hippocampus. [file 41593_2024_1636_MOESM5_ESM.gz › wgcna_functional_enrichment/dentateGyrus/module_red_go_enrichment.pdf]

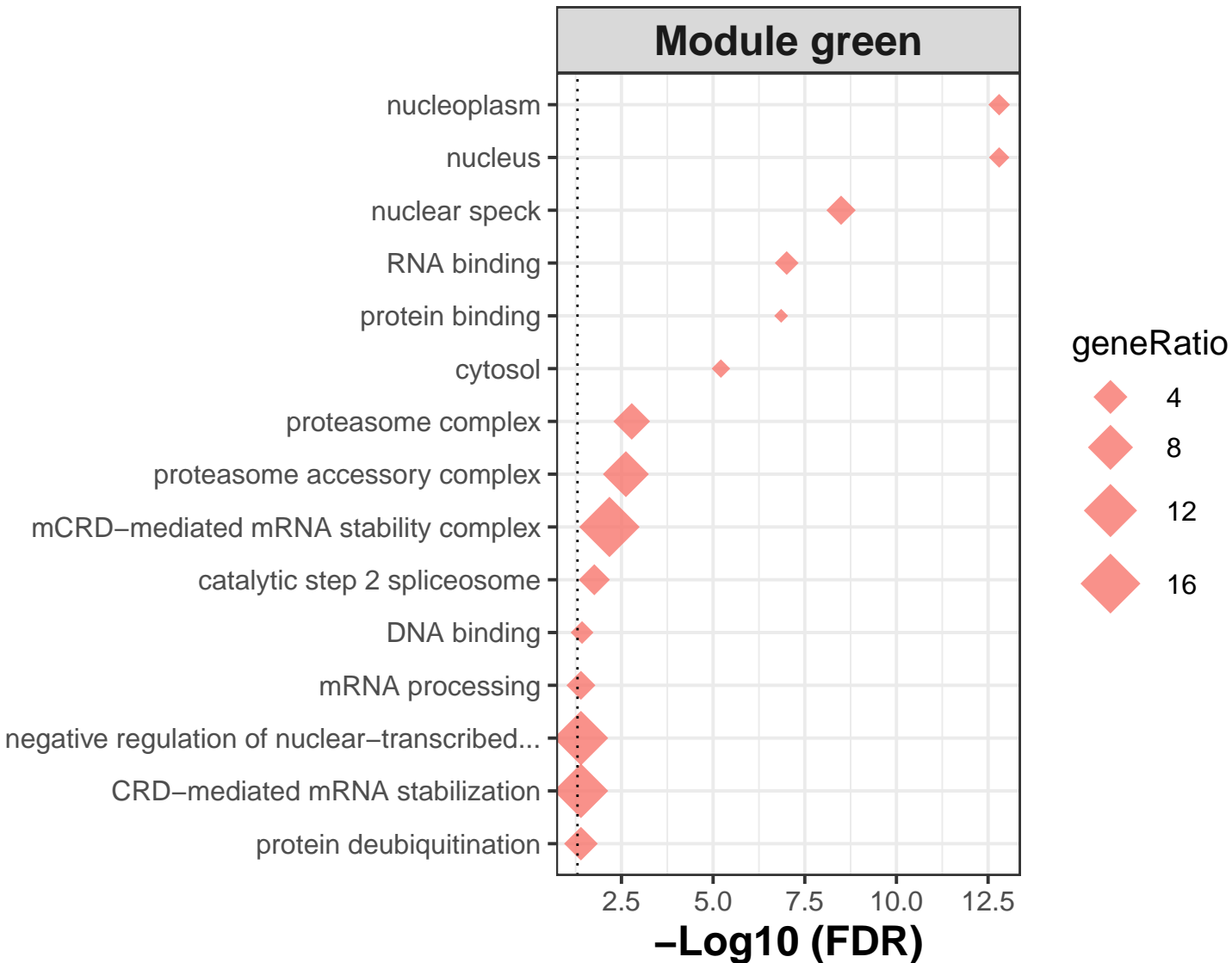

Supplement: Supplementary file 5 — Compressed directory of ancestry-associated DEGs enriched for WGCNA module functional enrichment results (that is, GO term enrichment) for the caudate nucleus, dentate gyrus, DLPFC and hippocampus. [file 41593_2024_1636_MOESM5_ESM.gz › wgcna_functional_enrichment/dentateGyrus/module_green_go_enrichment.pdf]

## Module darkmagenta

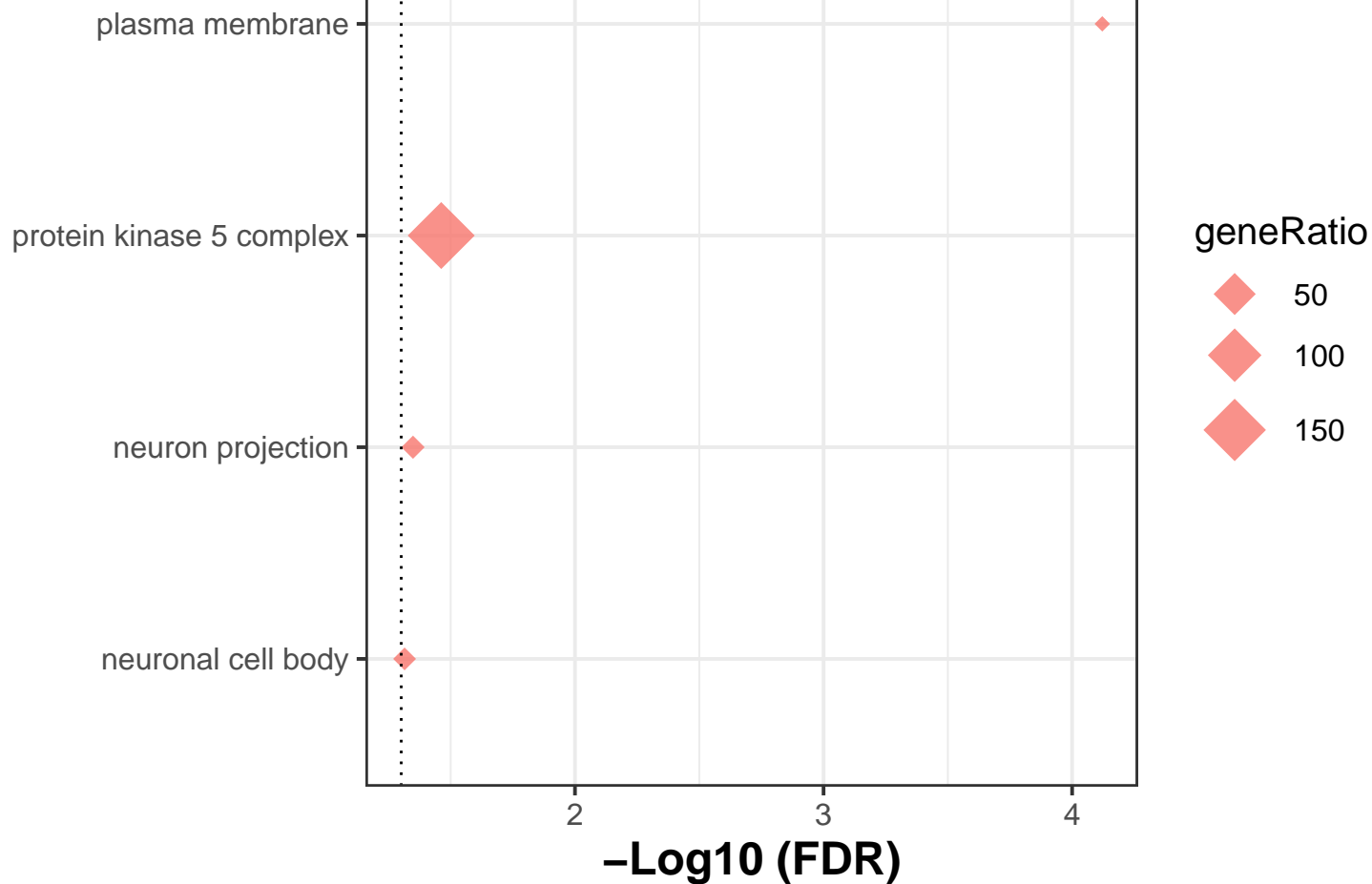

Supplement: Supplementary file 5 — Compressed directory of ancestry-associated DEGs enriched for WGCNA module functional enrichment results (that is, GO term enrichment) for the caudate nucleus, dentate gyrus, DLPFC and hippocampus. [file 41593_2024_1636_MOESM5_ESM.gz › wgcna_functional_enrichment/dentateGyrus/module_darkmagenta_go_enrichment.pdf]

## Module orangered4

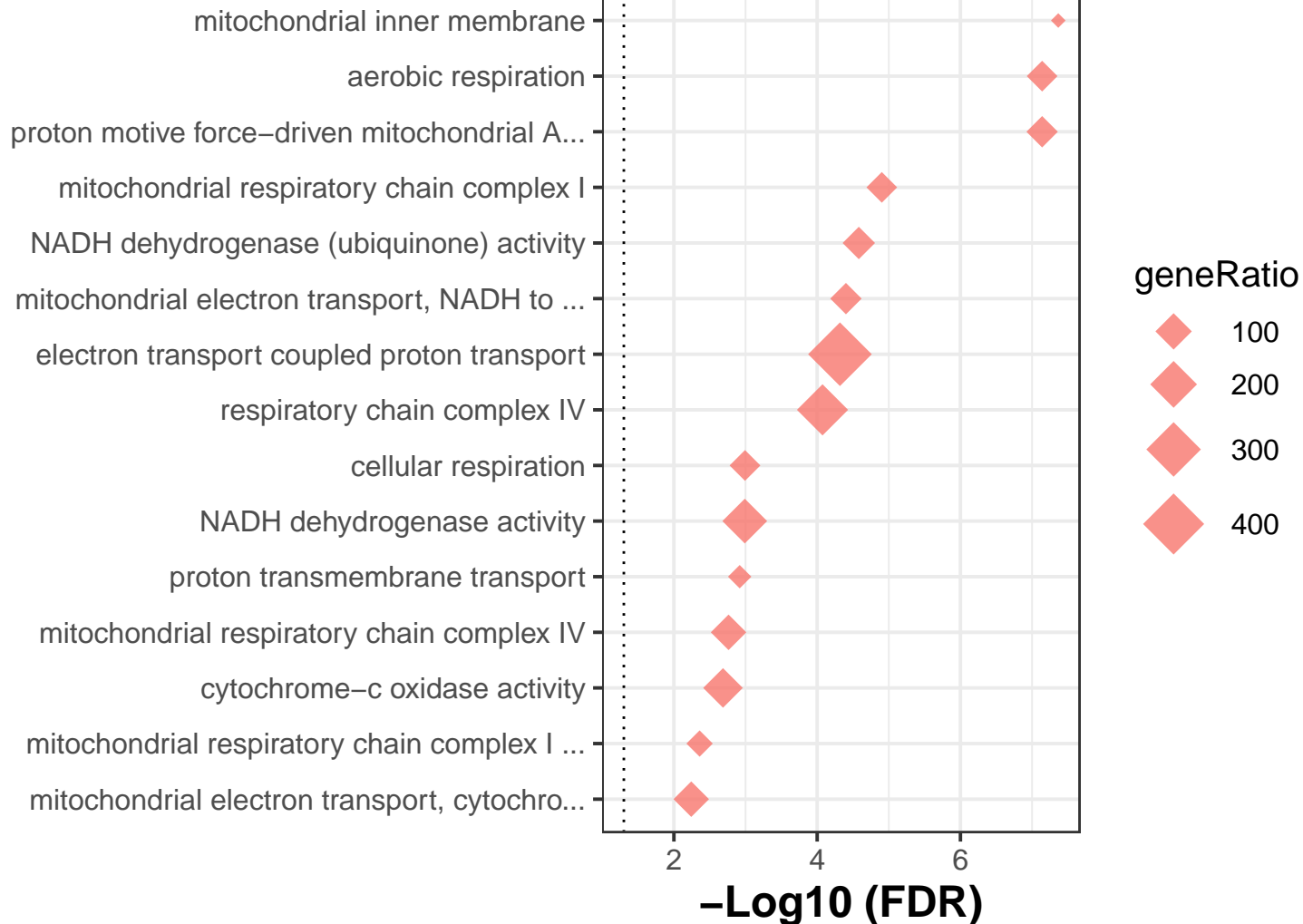

Supplement: Supplementary file 5 — Compressed directory of ancestry-associated DEGs enriched for WGCNA module functional enrichment results (that is, GO term enrichment) for the caudate nucleus, dentate gyrus, DLPFC and hippocampus. [file 41593_2024_1636_MOESM5_ESM.gz › wgcna_functional_enrichment/dentateGyrus/module_orangered4_go_enrichment.pdf]

## Module lightgreen

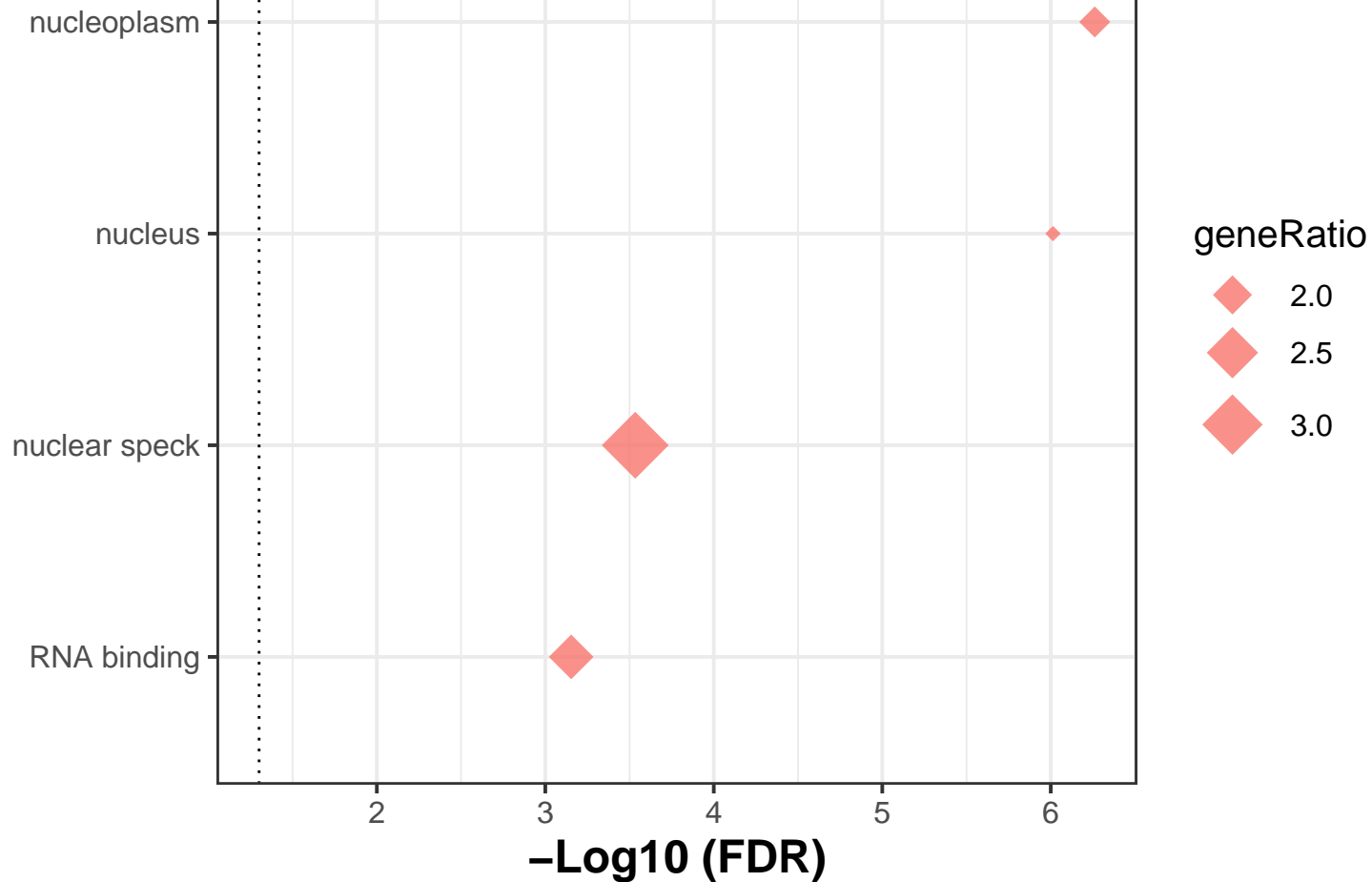

Supplement: Supplementary file 5 — Compressed directory of ancestry-associated DEGs enriched for WGCNA module functional enrichment results (that is, GO term enrichment) for the caudate nucleus, dentate gyrus, DLPFC and hippocampus. [file 41593_2024_1636_MOESM5_ESM.gz › wgcna_functional_enrichment/dentateGyrus/module_lightgreen_go_enrichment.pdf]

## Module midnightblue

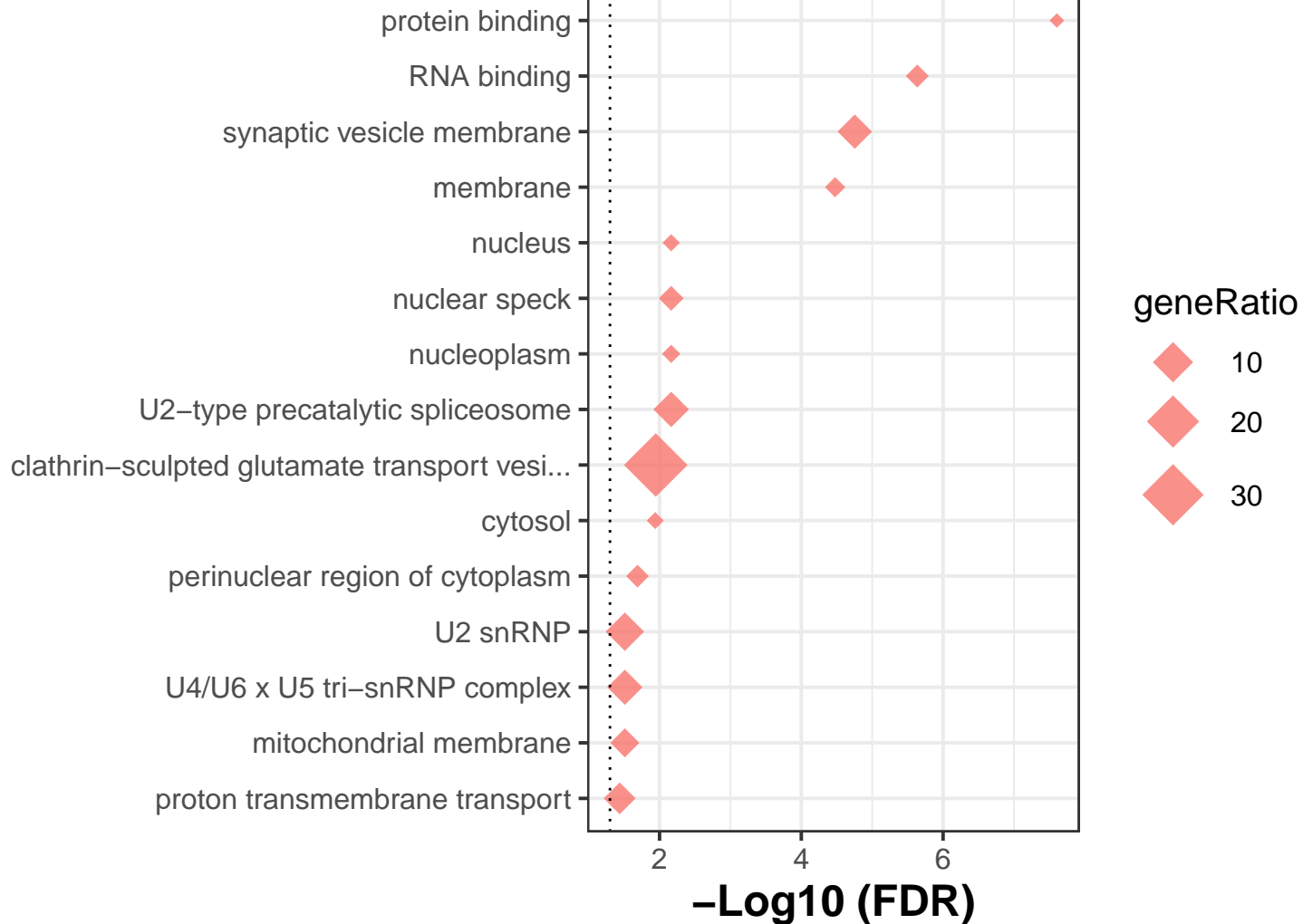

Supplement: Supplementary file 5 — Compressed directory of ancestry-associated DEGs enriched for WGCNA module functional enrichment results (that is, GO term enrichment) for the caudate nucleus, dentate gyrus, DLPFC and hippocampus. [file 41593_2024_1636_MOESM5_ESM.gz › wgcna_functional_enrichment/dentateGyrus/module_midnightblue_go_enrichment.pdf]

# Module skyblue3

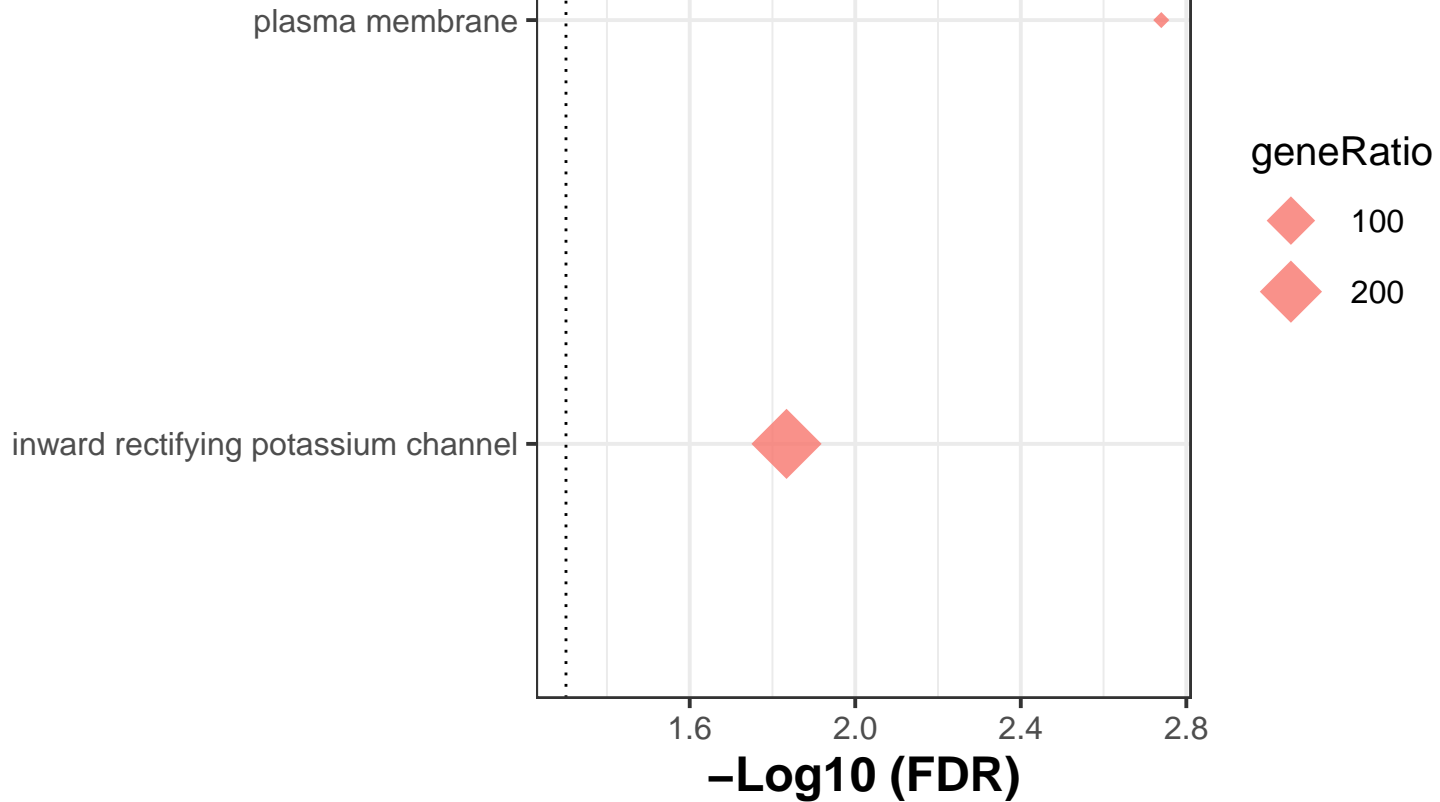

Supplement: Supplementary file 5 — Compressed directory of ancestry-associated DEGs enriched for WGCNA module functional enrichment results (that is, GO term enrichment) for the caudate nucleus, dentate gyrus, DLPFC and hippocampus. [file 41593_2024_1636_MOESM5_ESM.gz › wgcna_functional_enrichment/dentateGyrus/module_skyblue3_go_enrichment.pdf]

## Module grey

RNA processing

cytosolic ribosome

geneRatio

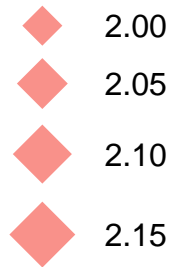

2

3

4

5

6

**$-\text{Log}_{10}(\text{FDR})$**

Supplement: Supplementary file 5 — Compressed directory of ancestry-associated DEGs enriched for WGCNA module functional enrichment results (that is, GO term enrichment) for the caudate nucleus, dentate gyrus, DLPFC and hippocampus. [file 41593_2024_1636_MOESM5_ESM.gz › wgcna_functional_enrichment/dentateGyrus/module_grey_go_enrichment.pdf]

## Module darkturquoise

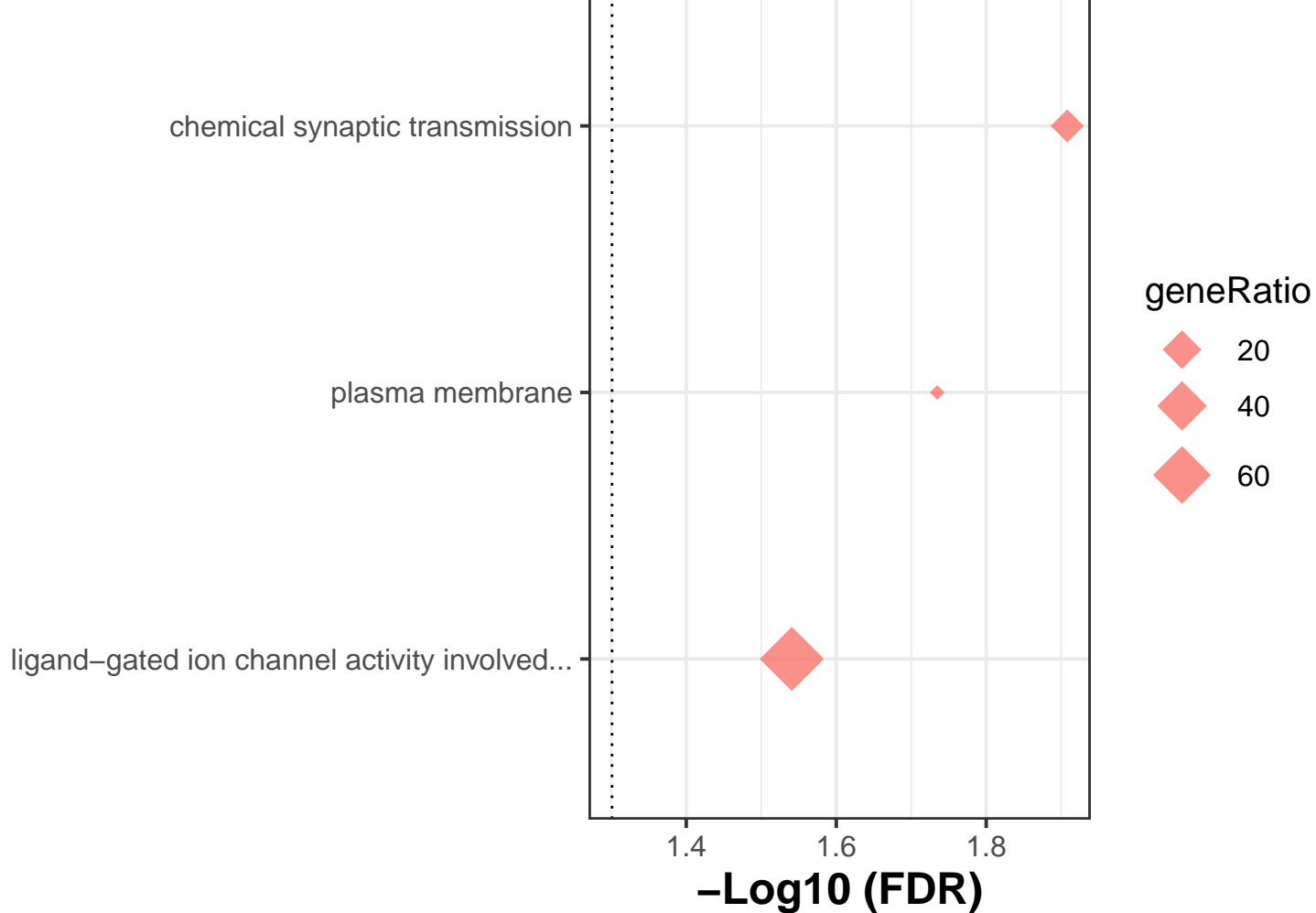

Supplement: Supplementary file 5 — Compressed directory of ancestry-associated DEGs enriched for WGCNA module functional enrichment results (that is, GO term enrichment) for the caudate nucleus, dentate gyrus, DLPFC and hippocampus. [file 41593_2024_1636_MOESM5_ESM.gz › wgcna_functional_enrichment/hippocampus/module_darkturquoise_go_enrichment.pdf]

## Module saddlebrown

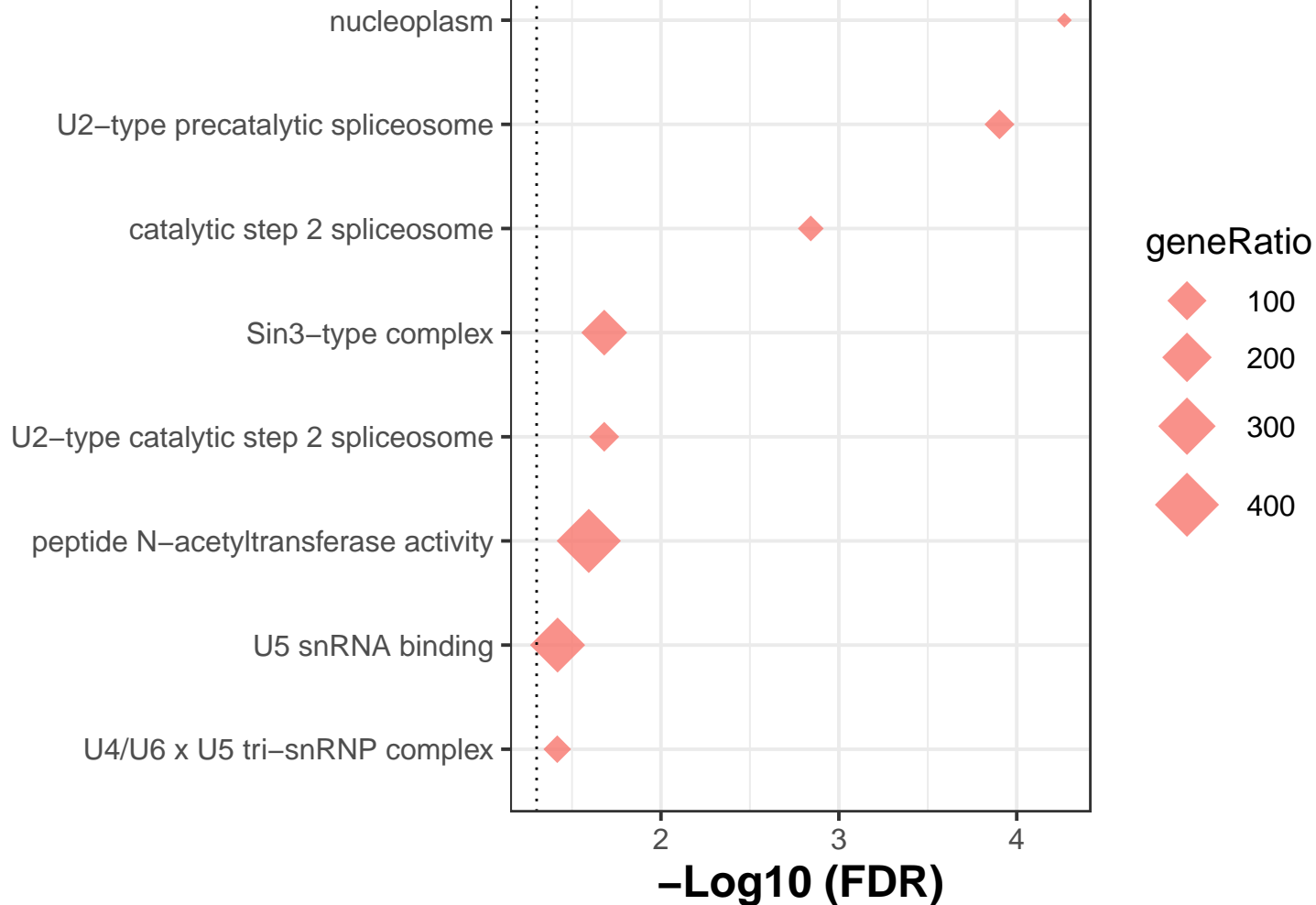

Supplement: Supplementary file 5 — Compressed directory of ancestry-associated DEGs enriched for WGCNA module functional enrichment results (that is, GO term enrichment) for the caudate nucleus, dentate gyrus, DLPFC and hippocampus. [file 41593_2024_1636_MOESM5_ESM.gz › wgcna_functional_enrichment/hippocampus/module_saddlebrown_go_enrichment.pdf]

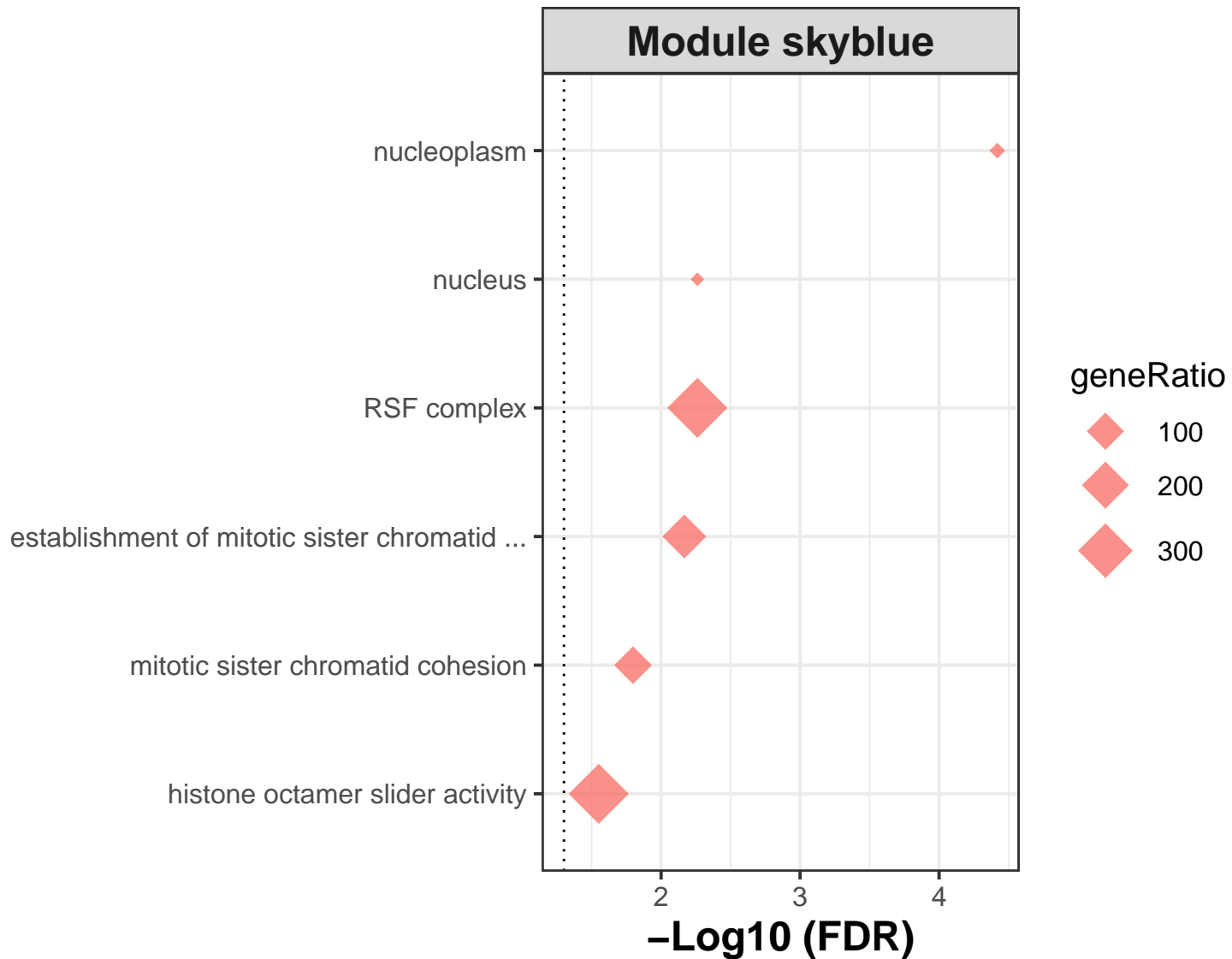

Supplement: Supplementary file 5 — Compressed directory of ancestry-associated DEGs enriched for WGCNA module functional enrichment results (that is, GO term enrichment) for the caudate nucleus, dentate gyrus, DLPFC and hippocampus. [file 41593_2024_1636_MOESM5_ESM.gz › wgcna_functional_enrichment/hippocampus/module_skyblue_go_enrichment.pdf]

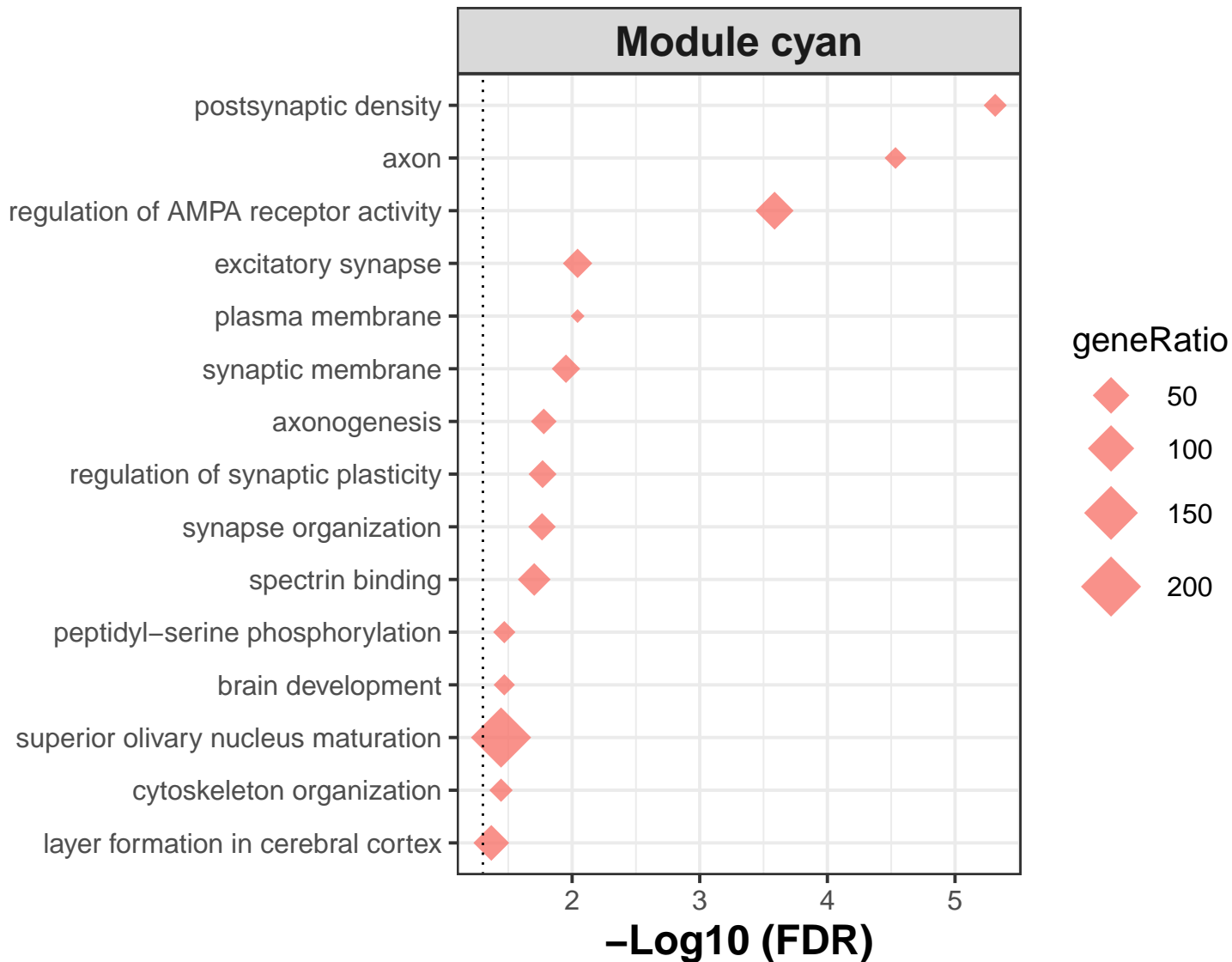

Supplement: Supplementary file 5 — Compressed directory of ancestry-associated DEGs enriched for WGCNA module functional enrichment results (that is, GO term enrichment) for the caudate nucleus, dentate gyrus, DLPFC and hippocampus. [file 41593_2024_1636_MOESM5_ESM.gz › wgcna_functional_enrichment/hippocampus/module_cyan_go_enrichment.pdf]

## Module midnightblue

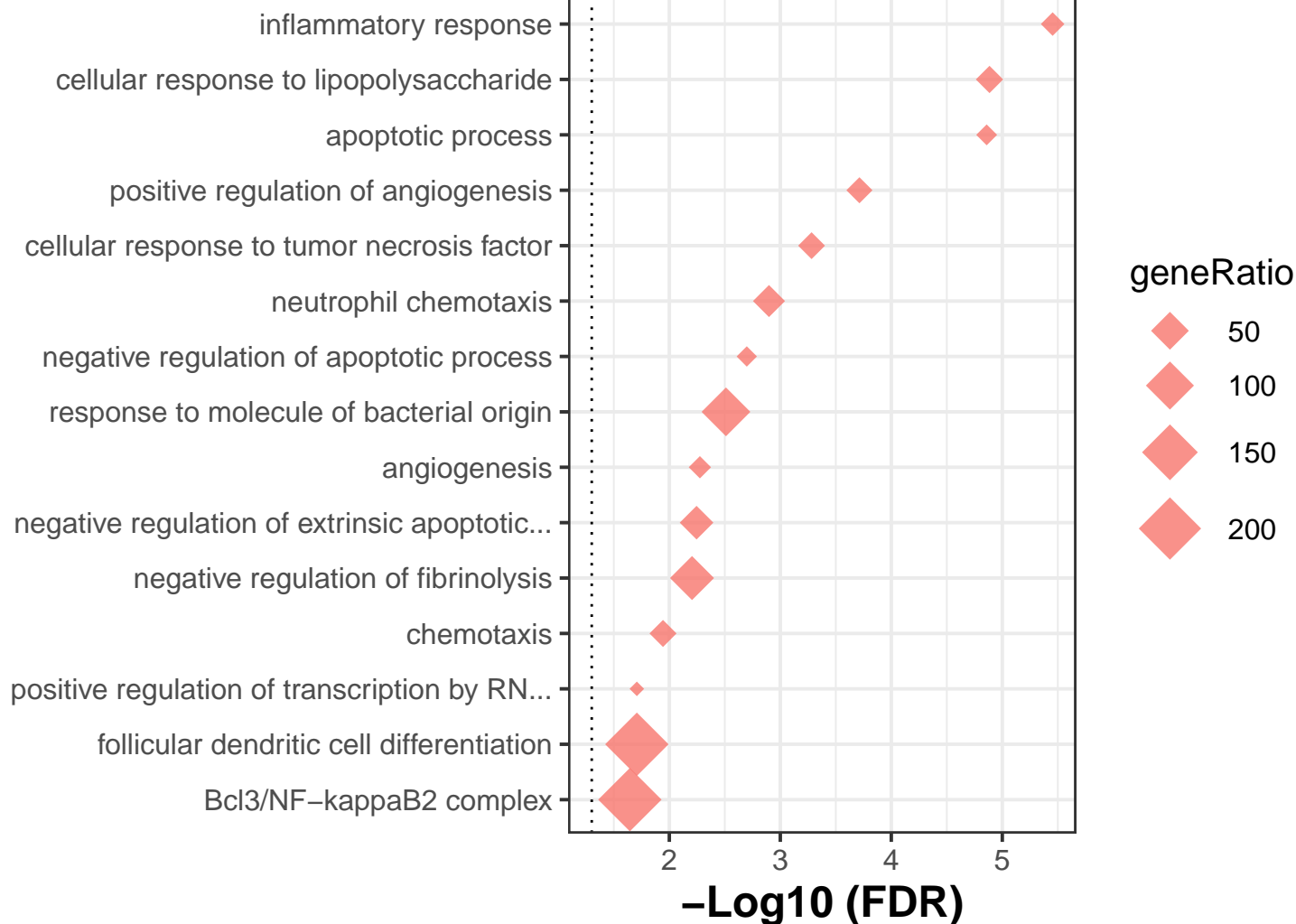

Supplement: Supplementary file 5 — Compressed directory of ancestry-associated DEGs enriched for WGCNA module functional enrichment results (that is, GO term enrichment) for the caudate nucleus, dentate gyrus, DLPFC and hippocampus. [file 41593_2024_1636_MOESM5_ESM.gz › wgcna_functional_enrichment/hippocampus/module_midnightblue_go_enrichment.pdf]

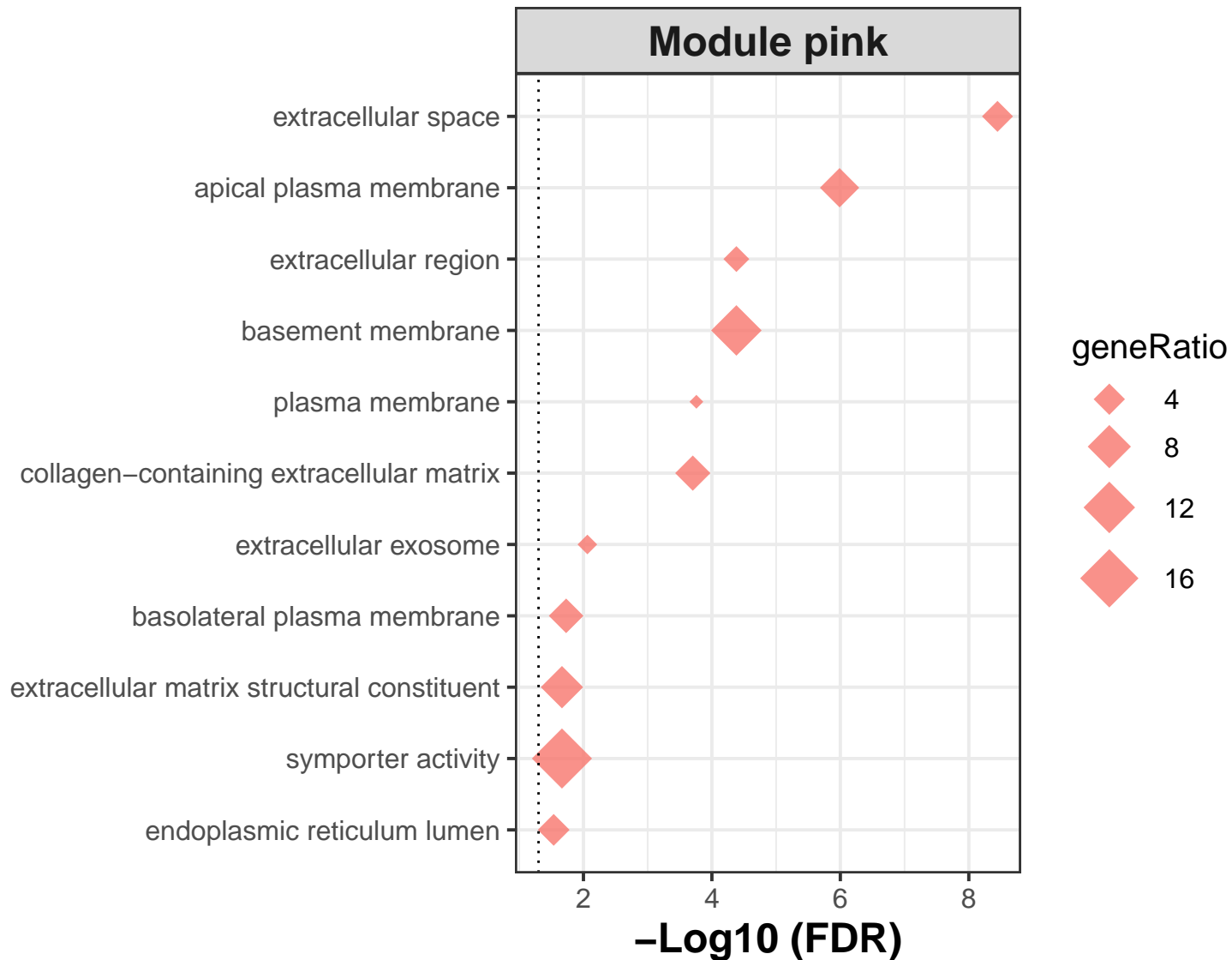

Supplement: Supplementary file 5 — Compressed directory of ancestry-associated DEGs enriched for WGCNA module functional enrichment results (that is, GO term enrichment) for the caudate nucleus, dentate gyrus, DLPFC and hippocampus. [file 41593_2024_1636_MOESM5_ESM.gz › wgcna_functional_enrichment/hippocampus/module_pink_go_enrichment.pdf]

## Module darkgreen

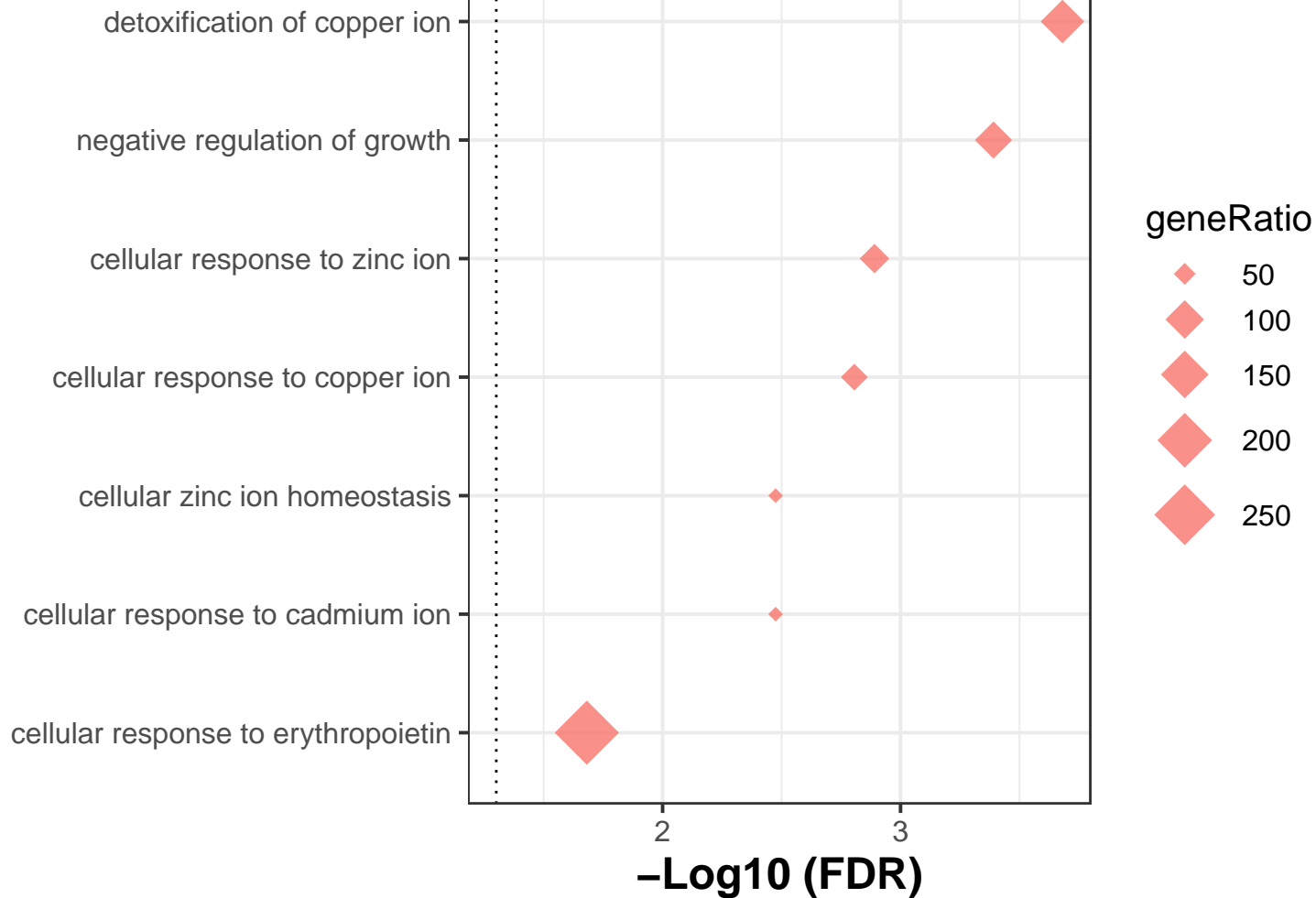

Supplement: Supplementary file 5 — Compressed directory of ancestry-associated DEGs enriched for WGCNA module functional enrichment results (that is, GO term enrichment) for the caudate nucleus, dentate gyrus, DLPFC and hippocampus. [file 41593_2024_1636_MOESM5_ESM.gz › wgcna_functional_enrichment/hippocampus/module_darkgreen_go_enrichment.pdf]

## Module steelblue

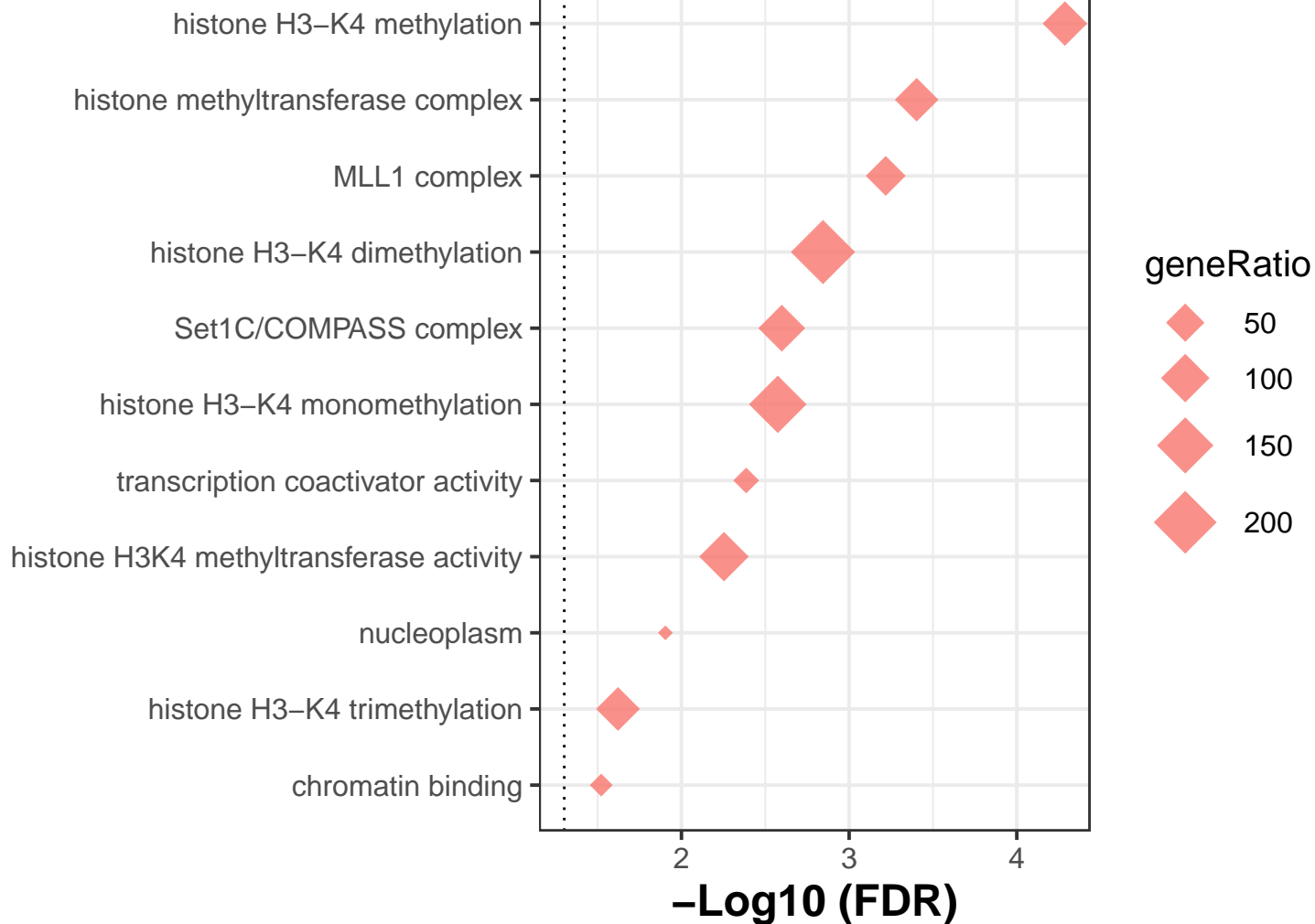

Supplement: Supplementary file 5 — Compressed directory of ancestry-associated DEGs enriched for WGCNA module functional enrichment results (that is, GO term enrichment) for the caudate nucleus, dentate gyrus, DLPFC and hippocampus. [file 41593_2024_1636_MOESM5_ESM.gz › wgcna_functional_enrichment/hippocampus/module_steelblue_go_enrichment.pdf]

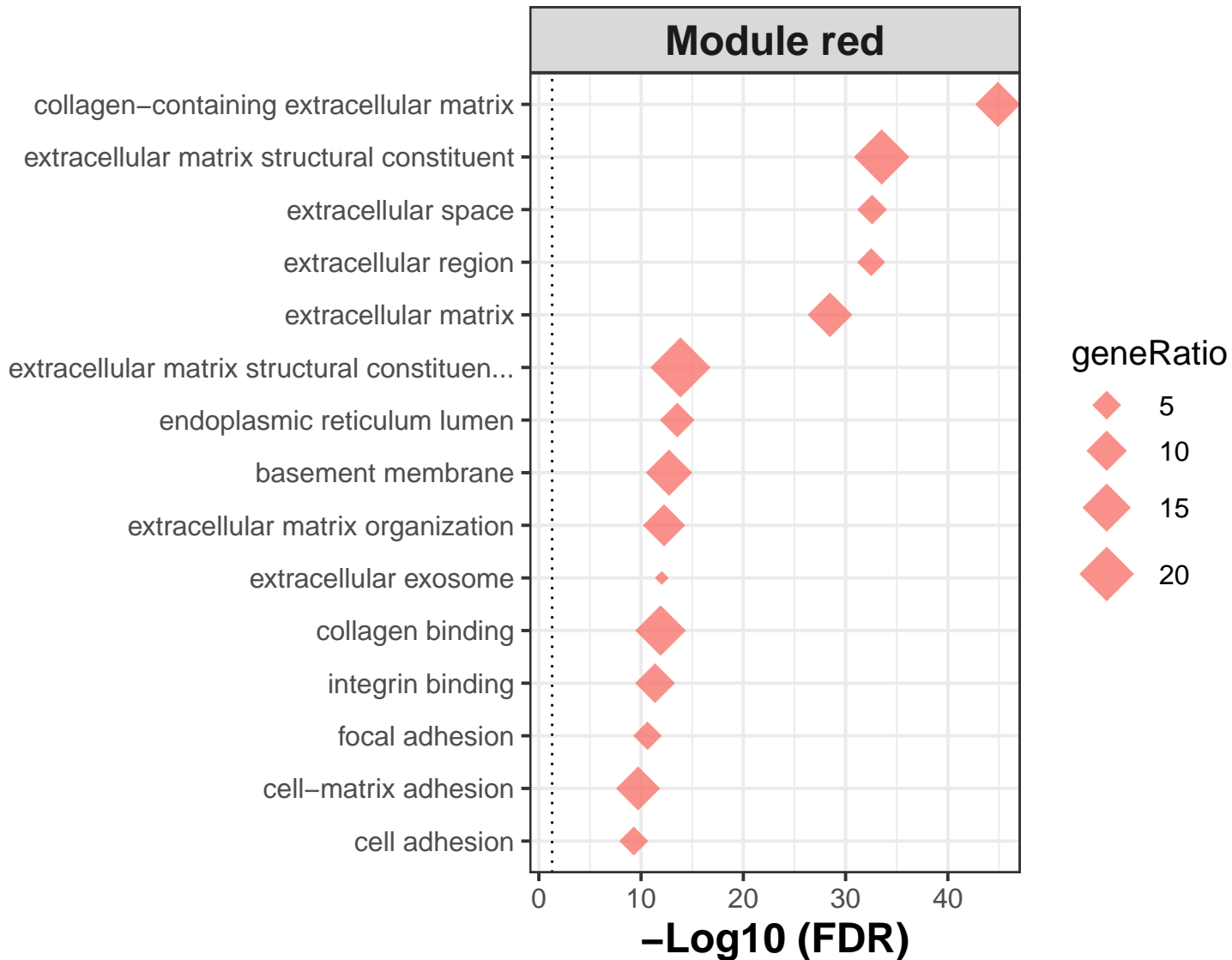

Supplement: Supplementary file 5 — Compressed directory of ancestry-associated DEGs enriched for WGCNA module functional enrichment results (that is, GO term enrichment) for the caudate nucleus, dentate gyrus, DLPFC and hippocampus. [file 41593_2024_1636_MOESM5_ESM.gz › wgcna_functional_enrichment/hippocampus/module_red_go_enrichment.pdf]

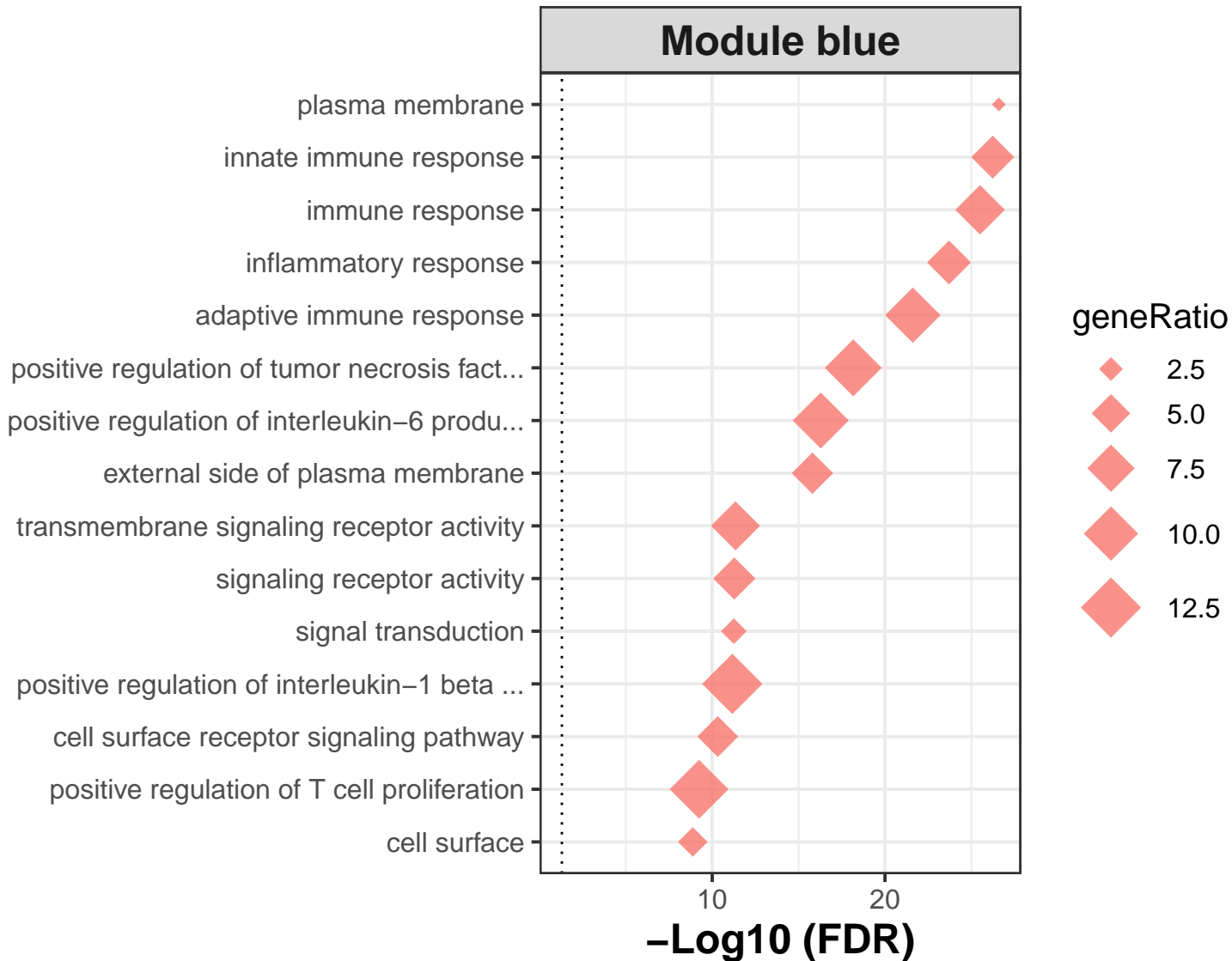

Supplement: Supplementary file 5 — Compressed directory of ancestry-associated DEGs enriched for WGCNA module functional enrichment results (that is, GO term enrichment) for the caudate nucleus, dentate gyrus, DLPFC and hippocampus. [file 41593_2024_1636_MOESM5_ESM.gz › wgcna_functional_enrichment/hippocampus/module_blue_go_enrichment.pdf]

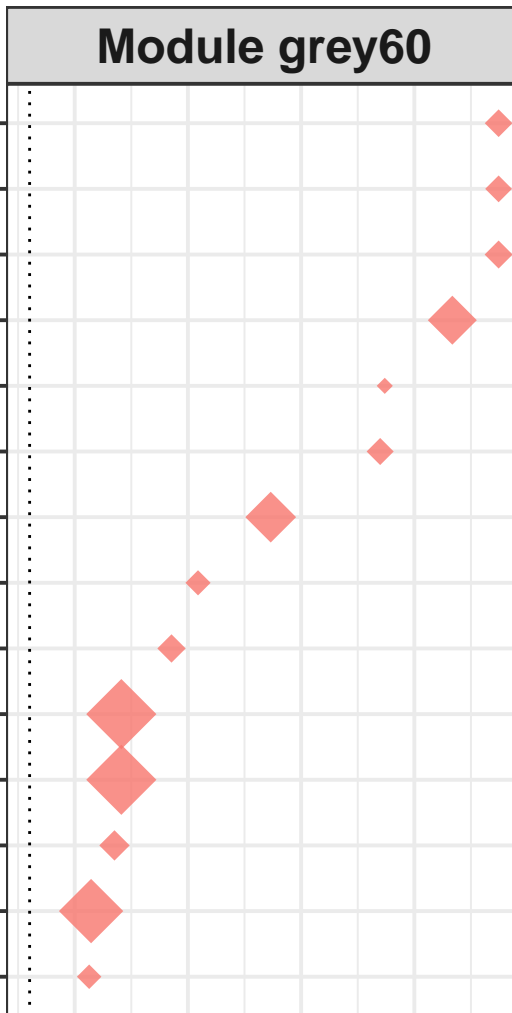

geneRatio

30

60

90

**-Log10 (FDR)**

Supplement: Supplementary file 5 — Compressed directory of ancestry-associated DEGs enriched for WGCNA module functional enrichment results (that is, GO term enrichment) for the caudate nucleus, dentate gyrus, DLPFC and hippocampus. [file 41593_2024_1636_MOESM5_ESM.gz › wgcna_functional_enrichment/hippocampus/module_grey60_go_enrichment.pdf]
