# Supplementary material for: Analysis of gene expression in the postmortem brain of neurotypical Black Americans reveals contributions of genetic ancestry
Source: Nat Neurosci. 2024 May 20;27(6):1064–74. doi: 10.1038/s41593-024-01636-0 (PMC11156587; doi:10.1038/s41593-024-01636-0)
Supplement: Supplementary file 7 — Compressed directory of PDF of scatter plots comparing DNA methylation association with local and global ancestry for the caudate nucleus, DLPFC and hippocampus. Plots are annotated with the genetic ancestry DMR test results. [file 41593_2024_1636_MOESM7_ESM.gz › DMR_global_local_comparison/DMR_global_local_compare_by_region_hippocampus.pdf]

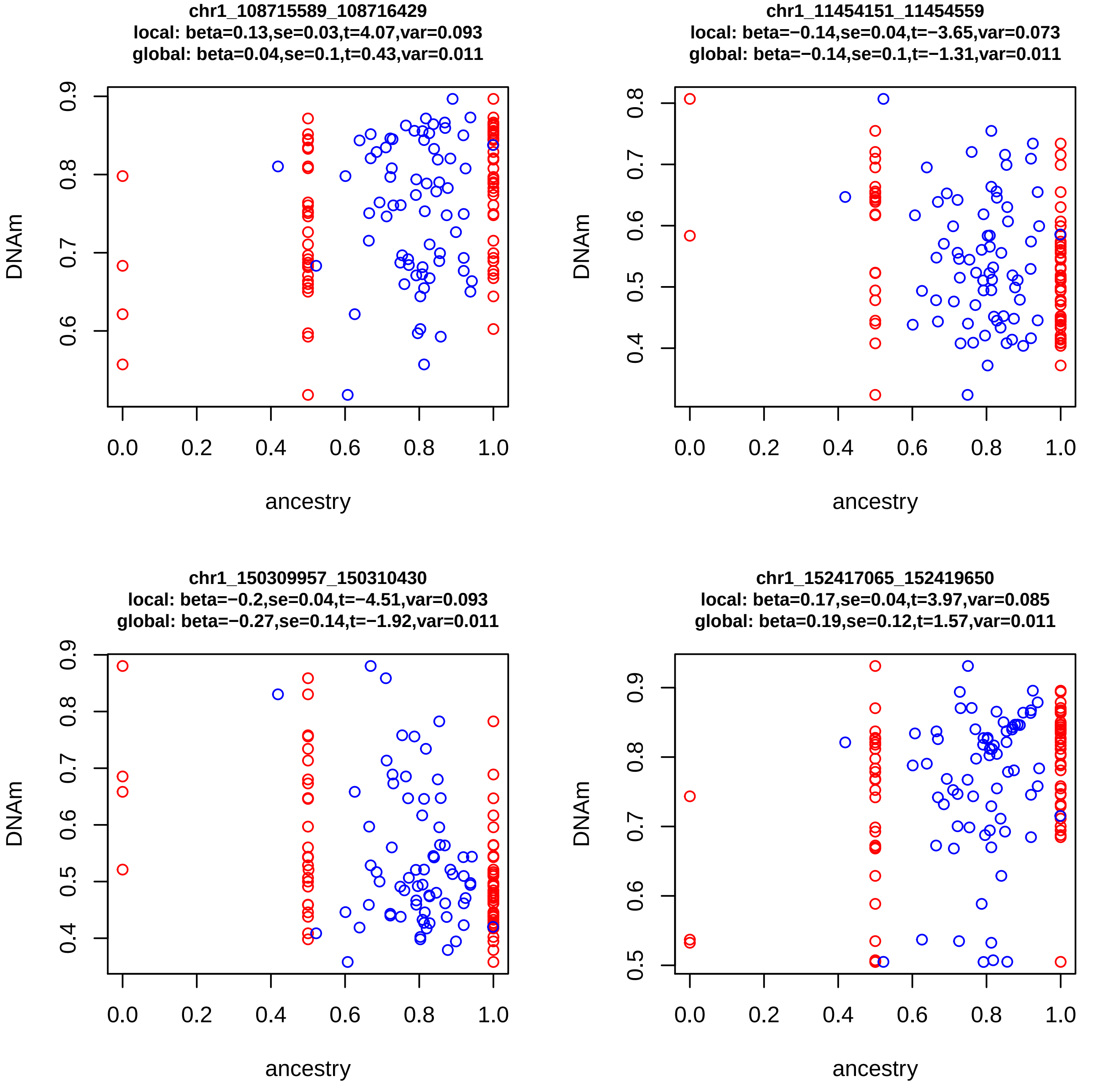
chr1_108715589_108716429
local: beta=0.13,se=0.03,t=4.07,var=0.093
global: beta=0.04,se=0.1,t=0.43,var=0.011
DNAm
ancestry
0.0 0.2 0.4 0.6 0.8 1.0
0.6 0.7 0.8 0.9
chr1_11454151_11454559
local: beta=−0.14,se=0.04,t=−3.65,var=0.073
global: beta=−0.14,se=0.1,t=−1.31,var=0.011
DNAm
ancestry
0.0 0.2 0.4 0.6 0.8 1.0
0.4 0.5 0.6 0.7 0.8
chr1_150309957_150310430
local: beta=−0.2,se=0.04,t=−4.51,var=0.093
global: beta=−0.27,se=0.14,t=−1.92,var=0.011
DNAm
ancestry
0.0 0.2 0.4 0.6 0.8 1.0
0.4 0.5 0.6 0.7 0.8 0.9
chr1_152417065_152419650
local: beta=0.17,se=0.04,t=3.97,var=0.085
global: beta=0.19,se=0.12,t=1.57,var=0.011
DNAm
ancestry
0.0 0.2 0.4 0.6 0.8 1.0
0.5 0.6 0.7 0.8 0.9

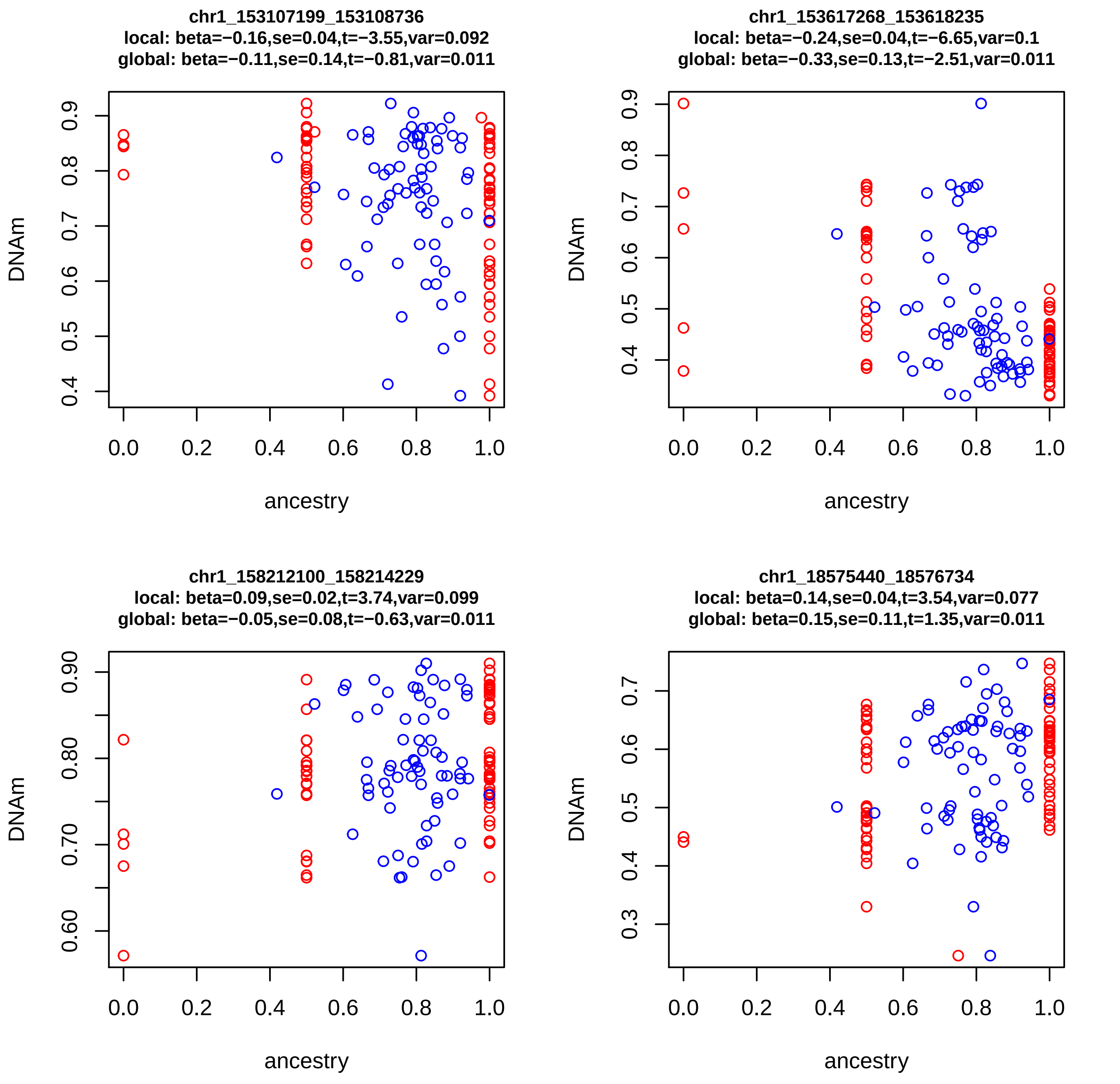
chr1_153107199_153108736
local: beta=−0.16,se=0.04,t=−3.55,var=0.092
global: beta=−0.11,se=0.14,t=−0.81,var=0.011
DNAm
ancestry
chr1_153617268_153618235
local: beta=−0.24,se=0.04,t=−6.65,var=0.1
global: beta=−0.33,se=0.13,t=−2.51,var=0.011
DNAm
ancestry
chr1_158212100_158214229
local: beta=0.09,se=0.02,t=3.74,var=0.099
global: beta=−0.05,se=0.08,t=−0.63,var=0.011
DNAm
ancestry
chr1_18575440_18576734
local: beta=0.14,se=0.04,t=3.54,var=0.077
global: beta=0.15,se=0.11,t=1.35,var=0.011
DNAm
ancestry

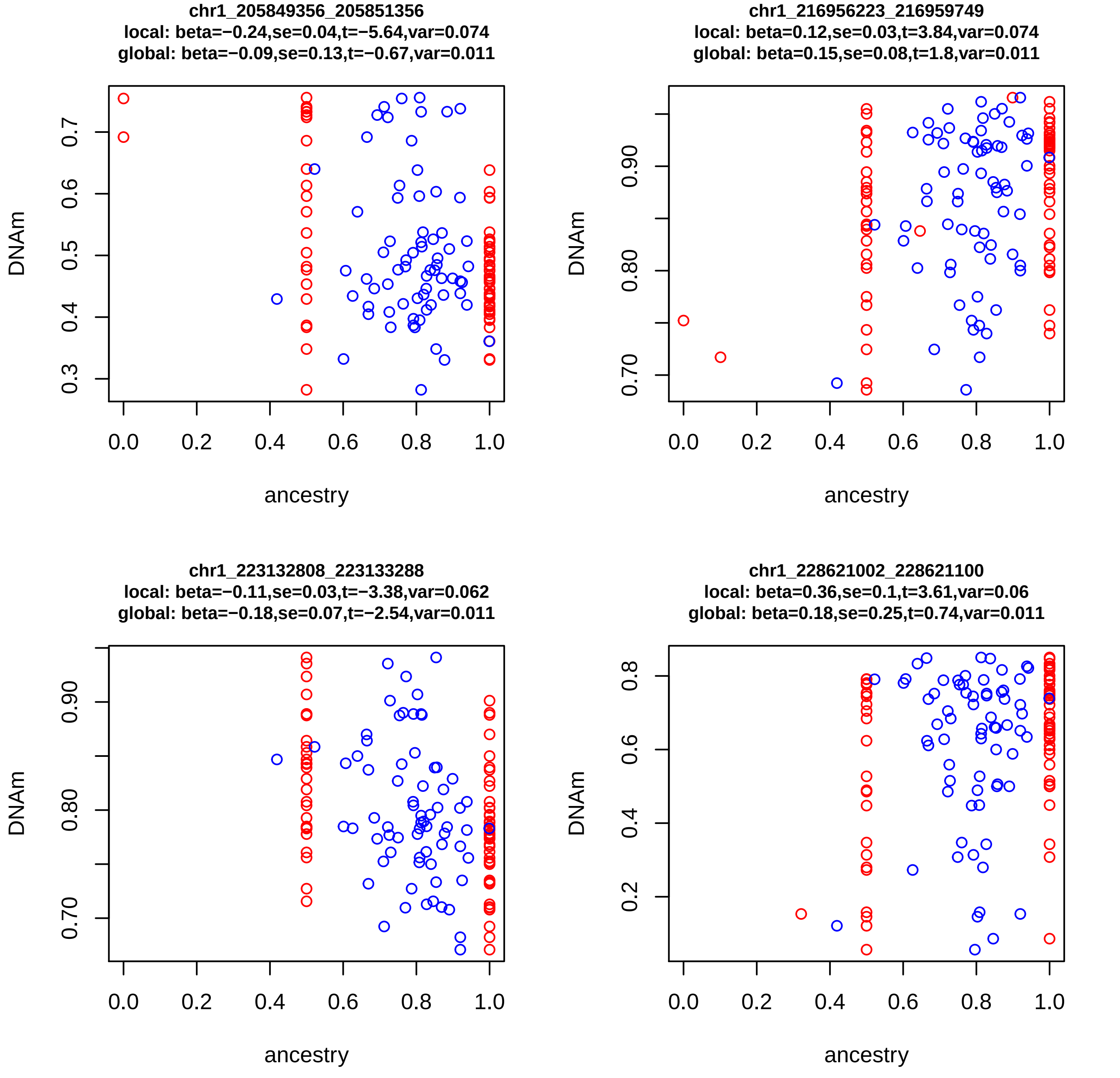
chr1_205849356_205851356
local: beta=−0.24,se=0.04,t=−5.64,var=0.074
global: beta=−0.09,se=0.13,t=−0.67,var=0.011
DNAm
ancestry
chr1_216956223_216959749
local: beta=0.12,se=0.03,t=3.84,var=0.074
global: beta=0.15,se=0.08,t=1.8,var=0.011
DNAm
ancestry
chr1_223132808_223133288
local: beta=−0.11,se=0.03,t=−3.38,var=0.062
global: beta=−0.18,se=0.07,t=−2.54,var=0.011
DNAm
ancestry
chr1_228621002_228621100
local: beta=0.36,se=0.1,t=3.61,var=0.06
global: beta=0.18,se=0.25,t=0.74,var=0.011
DNAm
ancestry

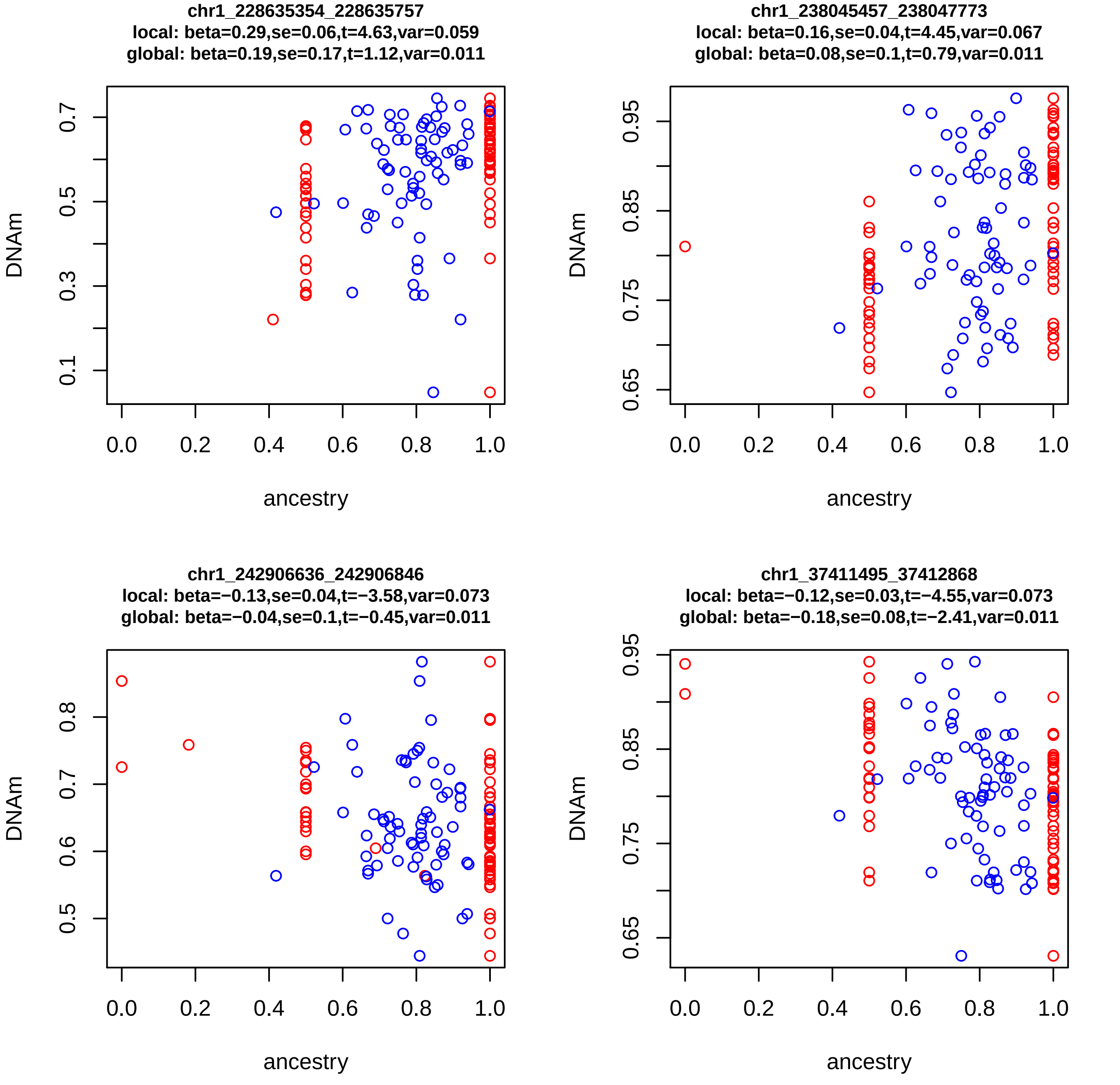

chr1_228635354_228635757
local: beta=0.29,se=0.06,t=4.63,var=0.059
global: beta=0.19,se=0.17,t=1.12,var=0.011
DNAm
0.1
0.3
0.5
0.7
0.0
0.2
0.4
0.6
0.8
1.0
ancestry
chr1_238045457_238047773
local: beta=0.16,se=0.04,t=4.45,var=0.067
global: beta=0.08,se=0.1,t=0.79,var=0.011
DNAm
0.65
0.75
0.85
0.95
0.0
0.2
0.4
0.6
0.8
1.0
ancestry
chr1_242906636_242906846
local: beta=−0.13,se=0.04,t=−3.58,var=0.073
global: beta=−0.04,se=0.1,t=−0.45,var=0.011
DNAm
0.5
0.6
0.7
0.8
0.0
0.2
0.4
0.6
0.8
1.0
ancestry
chr1_37411495_37412868
local: beta=−0.12,se=0.03,t=−4.55,var=0.073
global: beta=−0.18,se=0.08,t=−2.41,var=0.011
DNAm
0.65
0.75
0.85
0.95
0.0
0.2
0.4
0.6
0.8
1.0
ancestry

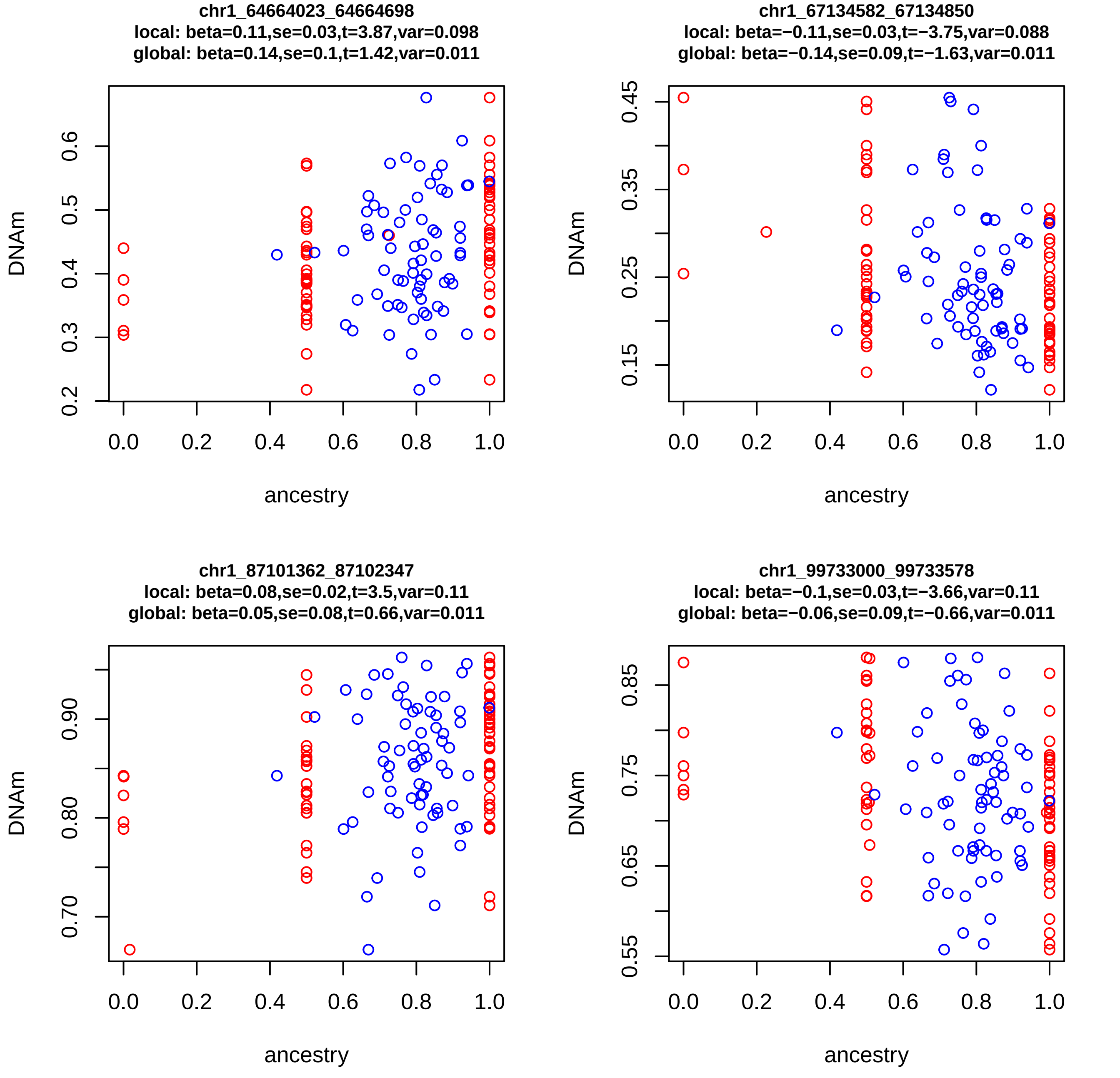
chr1_64664023_64664698
local: beta=0.11,se=0.03,t=3.87,var=0.098
global: beta=0.14,se=0.1,t=1.42,var=0.011
DNAm
0.2
0.3
0.4
0.5
0.6
ancestry
0.0
0.2
0.4
0.6
0.8
1.0
chr1_67134582_67134850
local: beta=−0.11,se=0.03,t=−3.75,var=0.088
global: beta=−0.14,se=0.09,t=−1.63,var=0.011
DNAm
0.15
0.25
0.35
0.45
ancestry
0.0
0.2
0.4
0.6
0.8
1.0
chr1_87101362_87102347
local: beta=0.08,se=0.02,t=3.5,var=0.11
global: beta=0.05,se=0.08,t=0.66,var=0.011
DNAm
0.70
0.80
0.90
ancestry
0.0
0.2
0.4
0.6
0.8
1.0
chr1_99733000_99733578
local: beta=−0.1,se=0.03,t=−3.66,var=0.11
global: beta=−0.06,se=0.09,t=−0.66,var=0.011
DNAm
0.55
0.65
0.75
0.85
ancestry
0.0
0.2
0.4
0.6
0.8
1.0

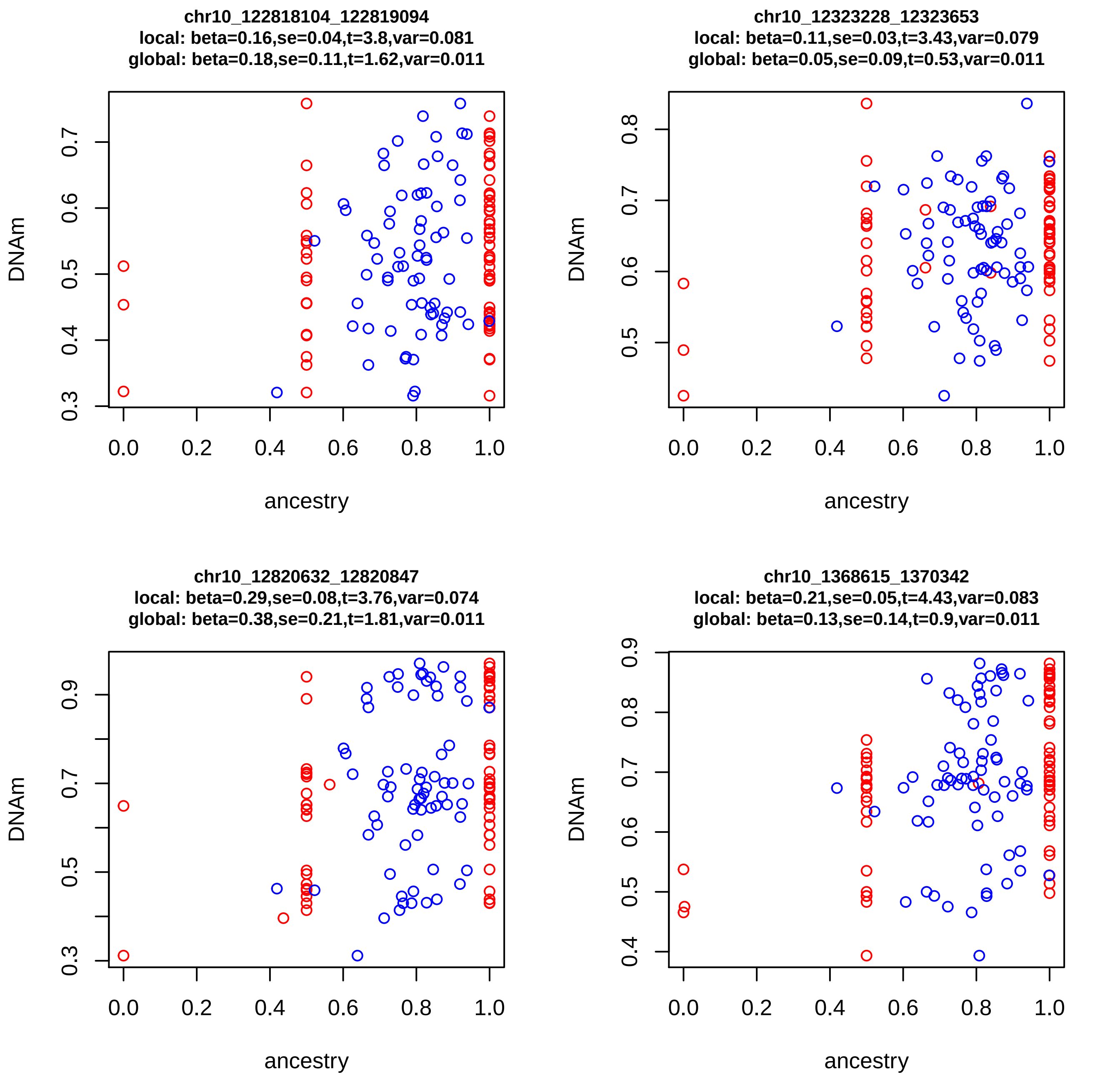
chr10_122818104_122819094
local: beta=0.16,se=0.04,t=3.8,var=0.081
global: beta=0.18,se=0.11,t=1.62,var=0.011
DNAm
ancestry
chr10_12323228_12323653
local: beta=0.11,se=0.03,t=3.43,var=0.079
global: beta=0.05,se=0.09,t=0.53,var=0.011
DNAm
ancestry
chr10_12820632_12820847
local: beta=0.29,se=0.08,t=3.76,var=0.074
global: beta=0.38,se=0.21,t=1.81,var=0.011
DNAm
ancestry
chr10_1368615_1370342
local: beta=0.21,se=0.05,t=4.43,var=0.083
global: beta=0.13,se=0.14,t=0.9,var=0.011
DNAm
ancestry

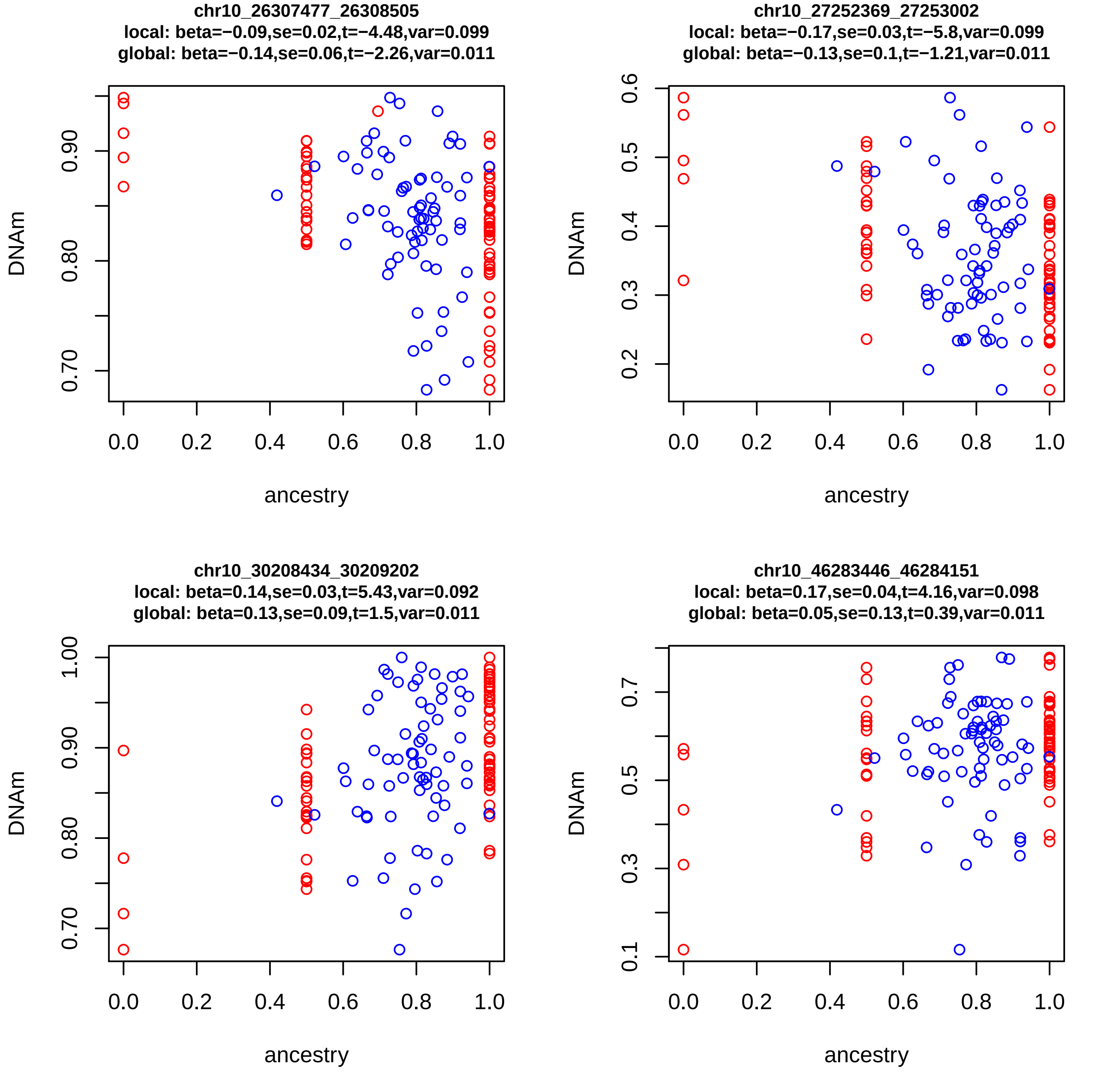
chr10_26307477_26308505
local: beta=−0.09,se=0.02,t=−4.48,var=0.099
global: beta=−0.14,se=0.06,t=−2.26,var=0.011
DNAm
ancestry
chr10_27252369_27253002
local: beta=−0.17,se=0.03,t=−5.8,var=0.099
global: beta=−0.13,se=0.1,t=−1.21,var=0.011
DNAm
ancestry
chr10_30208434_30209202
local: beta=0.14,se=0.03,t=5.43,var=0.092
global: beta=0.13,se=0.09,t=1.5,var=0.011
DNAm
ancestry
chr10_46283446_46284151
local: beta=0.17,se=0.04,t=4.16,var=0.098
global: beta=0.05,se=0.13,t=0.39,var=0.011
DNAm
ancestry

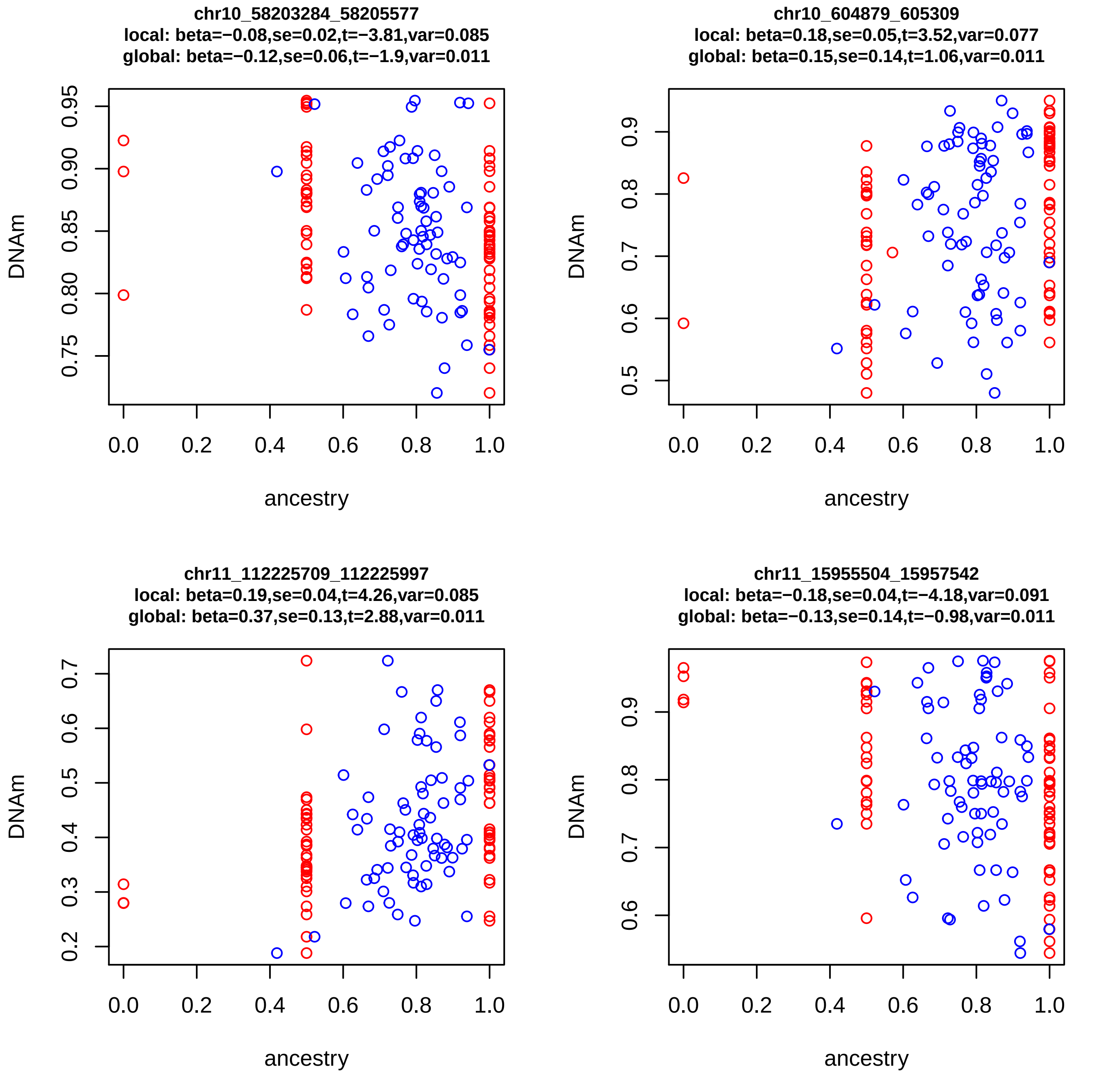
chr10_58203284_58205577
local: beta=−0.08,se=0.02,t=−3.81,var=0.085
global: beta=−0.12,se=0.06,t=−1.9,var=0.011
DNAm
0.75 0.80 0.85 0.90 0.95
0.0 0.2 0.4 0.6 0.8 1.0
ancestry
chr10_604879_605309
local: beta=0.18,se=0.05,t=3.52,var=0.077
global: beta=0.15,se=0.14,t=1.06,var=0.011
DNAm
0.5 0.6 0.7 0.8 0.9
0.0 0.2 0.4 0.6 0.8 1.0
ancestry
chr11_112225709_112225997
local: beta=0.19,se=0.04,t=4.26,var=0.085
global: beta=0.37,se=0.13,t=2.88,var=0.011
DNAm
0.2 0.3 0.4 0.5 0.6 0.7
0.0 0.2 0.4 0.6 0.8 1.0
ancestry
chr11_15955504_15957542
local: beta=−0.18,se=0.04,t=−4.18,var=0.091
global: beta=−0.13,se=0.14,t=−0.98,var=0.011
DNAm
0.6 0.7 0.8 0.9
0.0 0.2 0.4 0.6 0.8 1.0
ancestry

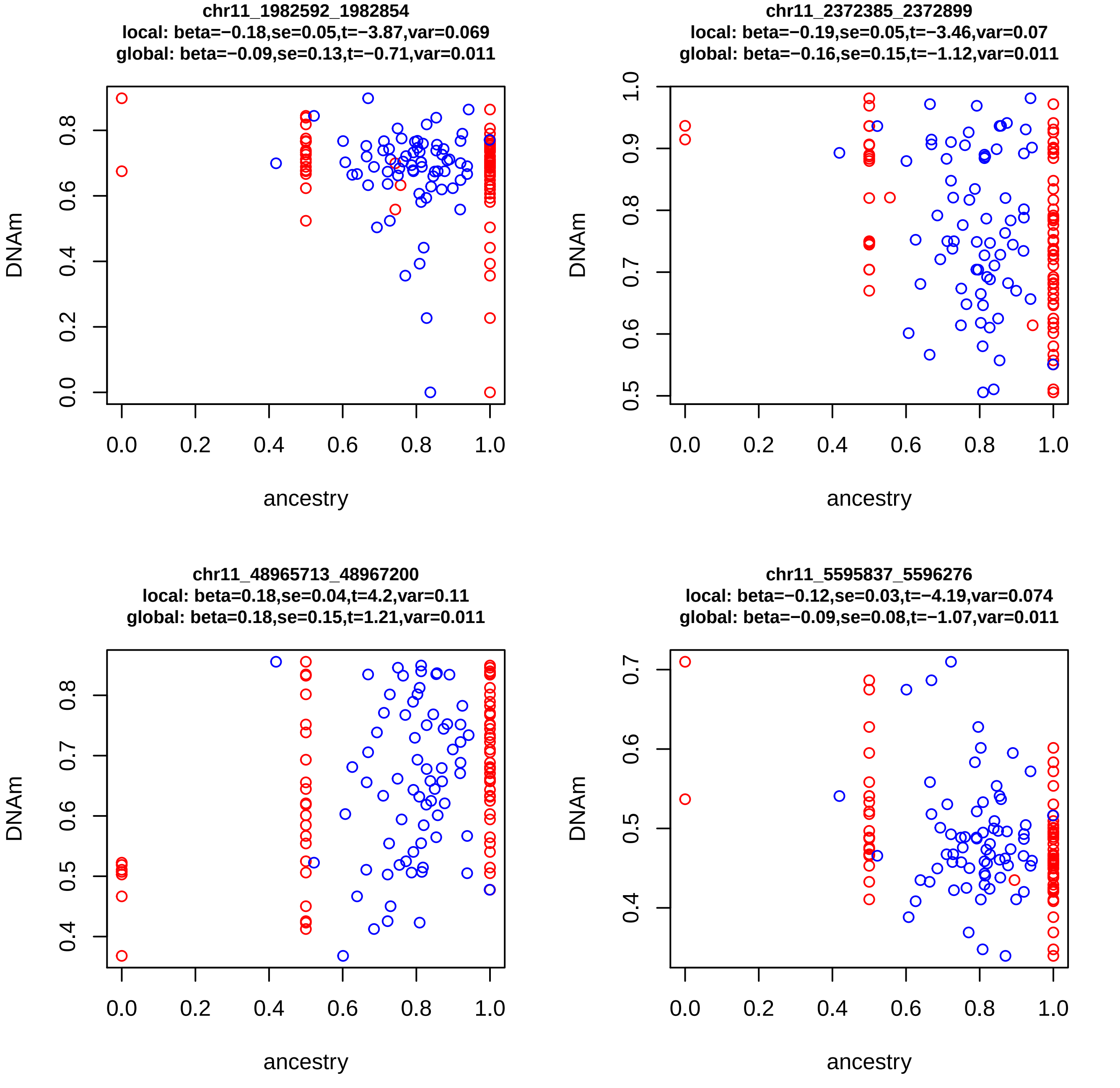
chr11_1982592_1982854
local: beta=−0.18,se=0.05,t=−3.87,var=0.069
global: beta=−0.09,se=0.13,t=−0.71,var=0.011
DNAm
ancestry
chr11_2372385_2372899
local: beta=−0.19,se=0.05,t=−3.46,var=0.07
global: beta=−0.16,se=0.15,t=−1.12,var=0.011
DNAm
ancestry
chr11_48965713_48967200
local: beta=0.18,se=0.04,t=4.2,var=0.11
global: beta=0.18,se=0.15,t=1.21,var=0.011
DNAm
ancestry
chr11_5595837_5596276
local: beta=−0.12,se=0.03,t=−4.19,var=0.074
global: beta=−0.09,se=0.08,t=−1.07,var=0.011
DNAm
ancestry

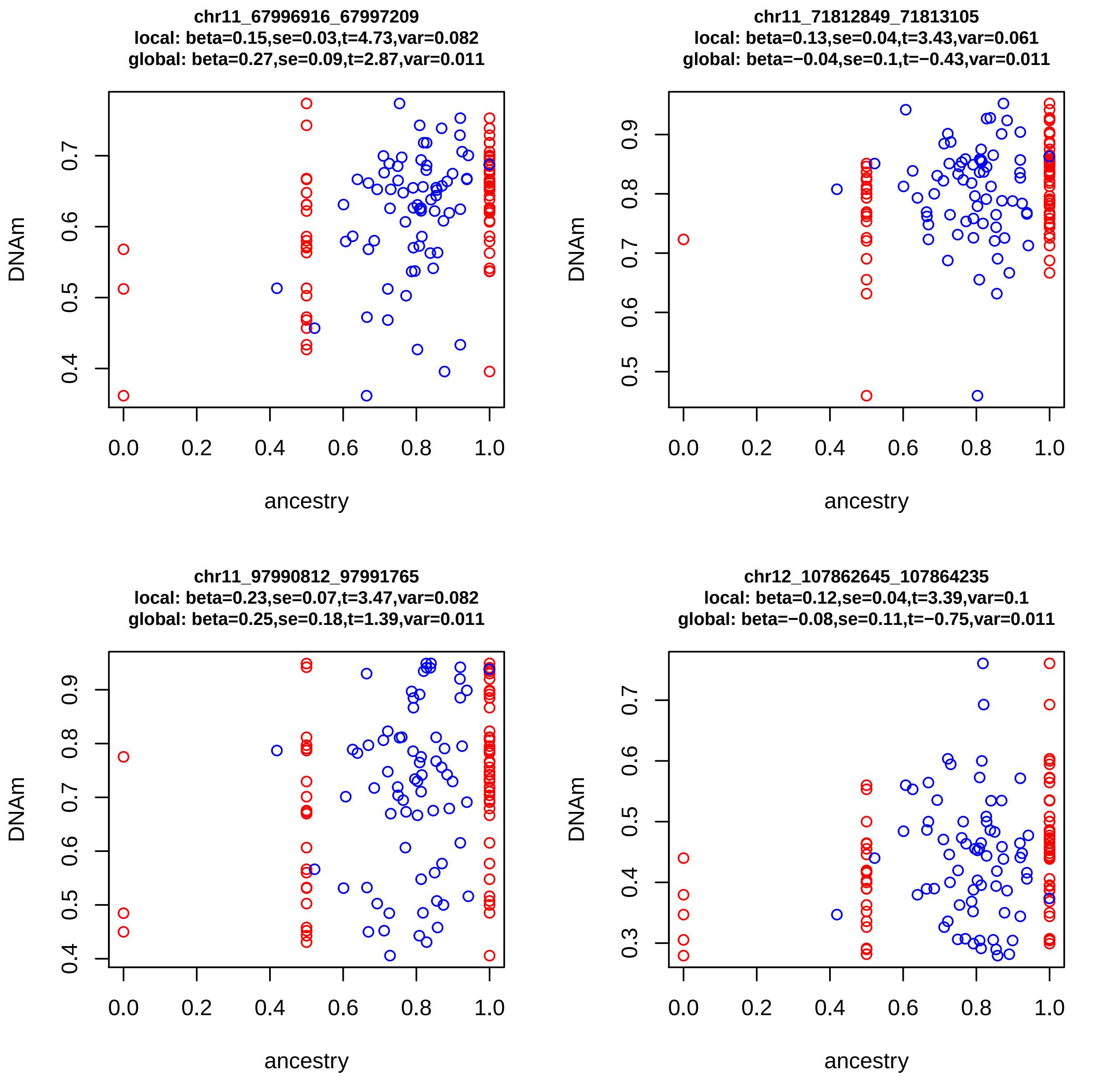
chr11_67996916_67997209
local: beta=0.15,se=0.03,t=4.73,var=0.082
global: beta=0.27,se=0.09,t=2.87,var=0.011
DNAm
ancestry
chr11_71812849_71813105
local: beta=0.13,se=0.04,t=3.43,var=0.061
global: beta=−0.04,se=0.1,t=−0.43,var=0.011
DNAm
ancestry
chr11_97990812_97991765
local: beta=0.23,se=0.07,t=3.47,var=0.082
global: beta=0.25,se=0.18,t=1.39,var=0.011
DNAm
ancestry
chr12_107862645_107864235
local: beta=0.12,se=0.04,t=3.39,var=0.1
global: beta=−0.08,se=0.11,t=−0.75,var=0.011
DNAm
ancestry

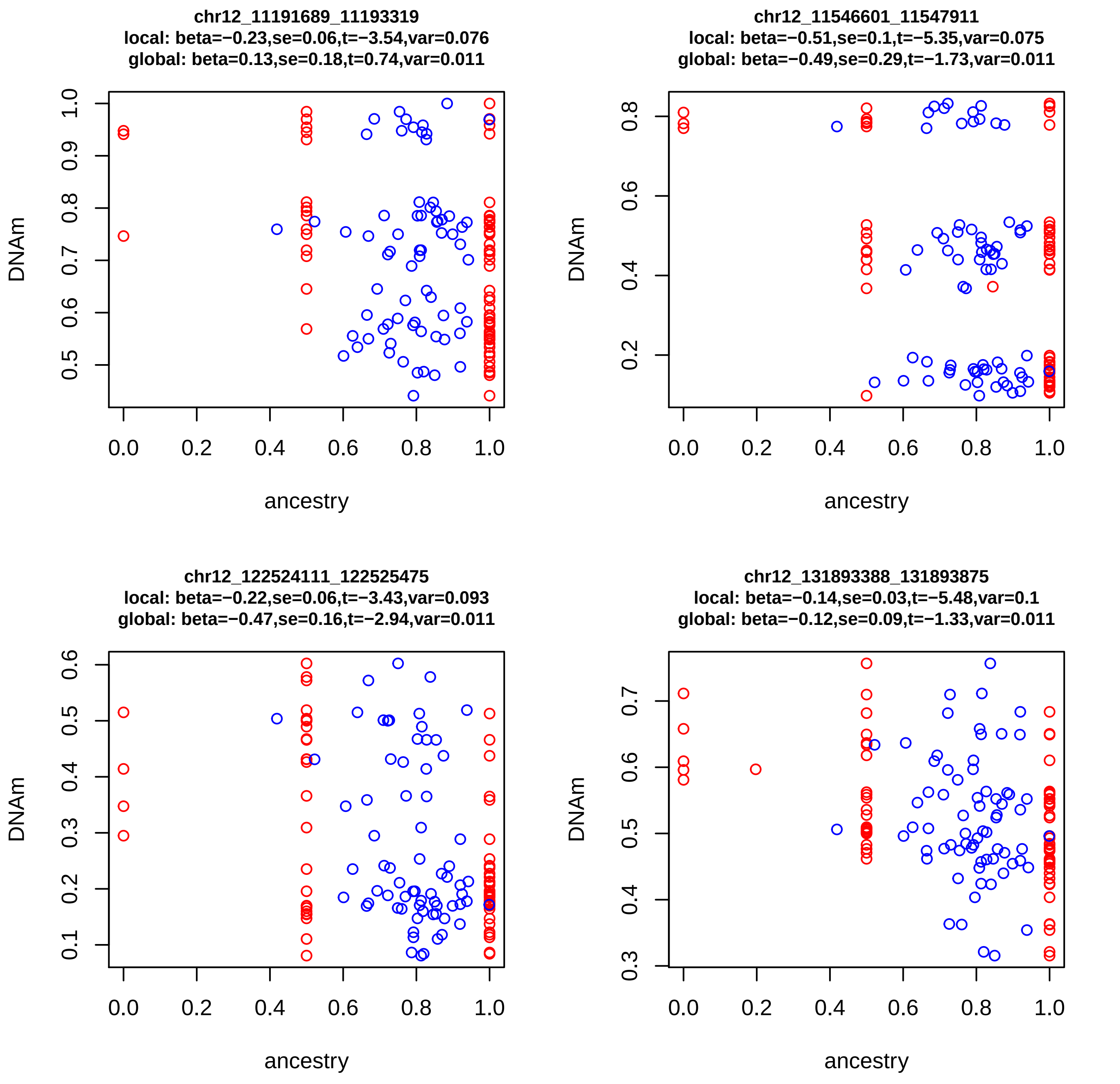
chr12_11191689_11193319
local: beta=−0.23,se=0.06,t=−3.54,var=0.076
global: beta=0.13,se=0.18,t=0.74,var=0.011
DNAm
ancestry
chr12_11546601_11547911
local: beta=−0.51,se=0.1,t=−5.35,var=0.075
global: beta=−0.49,se=0.29,t=−1.73,var=0.011
DNAm
ancestry
chr12_122524111_122525475
local: beta=−0.22,se=0.06,t=−3.43,var=0.093
global: beta=−0.47,se=0.16,t=−2.94,var=0.011
DNAm
ancestry
chr12_131893388_131893875
local: beta=−0.14,se=0.03,t=−5.48,var=0.1
global: beta=−0.12,se=0.09,t=−1.33,var=0.011
DNAm
ancestry

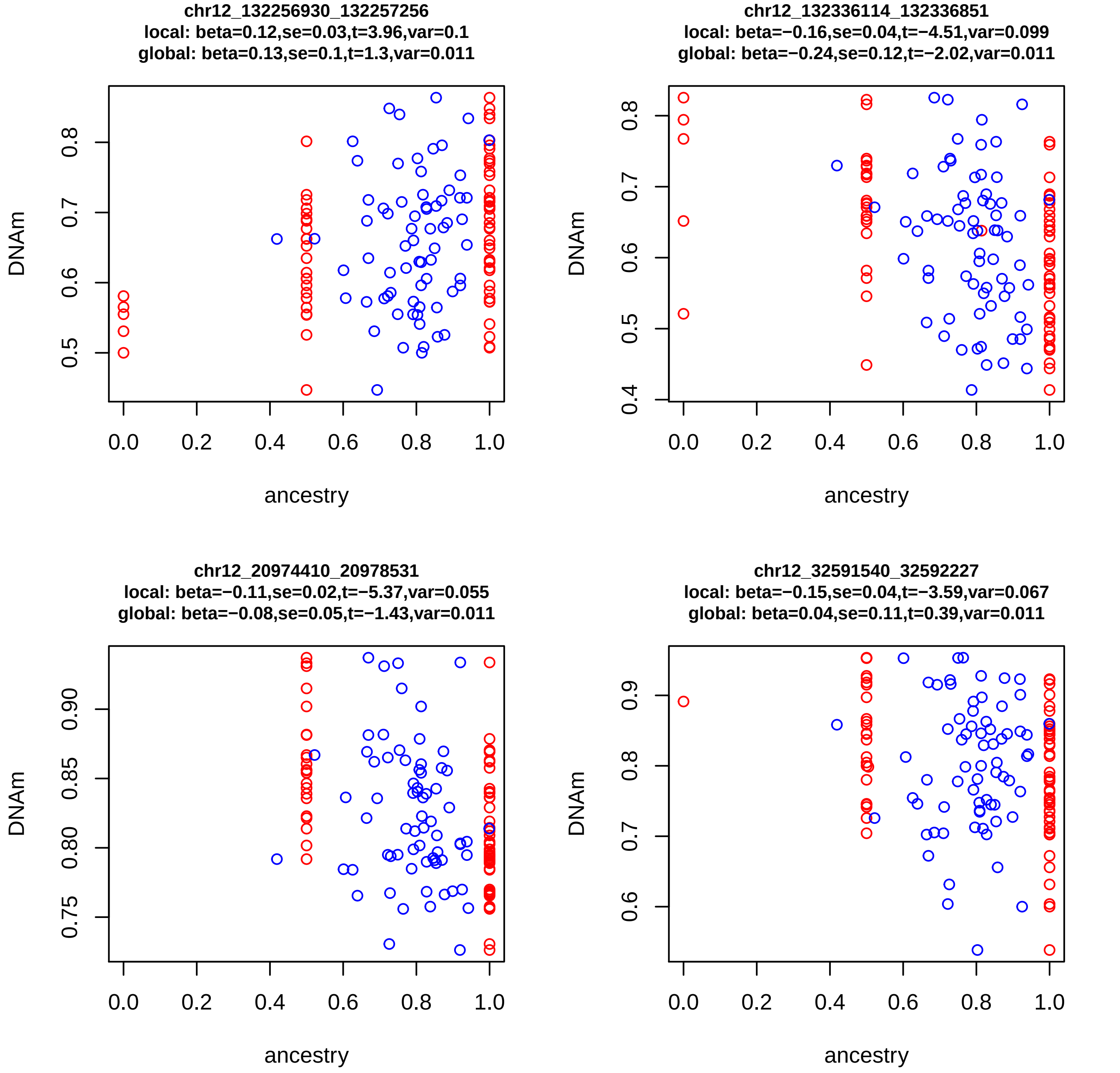
chr12_132256930_132257256
local: beta=0.12,se=0.03,t=3.96,var=0.1
global: beta=0.13,se=0.1,t=1.3,var=0.011
DNAm
ancestry
chr12_132336114_132336851
local: beta=−0.16,se=0.04,t=−4.51,var=0.099
global: beta=−0.24,se=0.12,t=−2.02,var=0.011
DNAm
ancestry
chr12_20974410_20978531
local: beta=−0.11,se=0.02,t=−5.37,var=0.055
global: beta=−0.08,se=0.05,t=−1.43,var=0.011
DNAm
ancestry
chr12_32591540_32592227
local: beta=−0.15,se=0.04,t=−3.59,var=0.067
global: beta=0.04,se=0.11,t=0.39,var=0.011
DNAm
ancestry

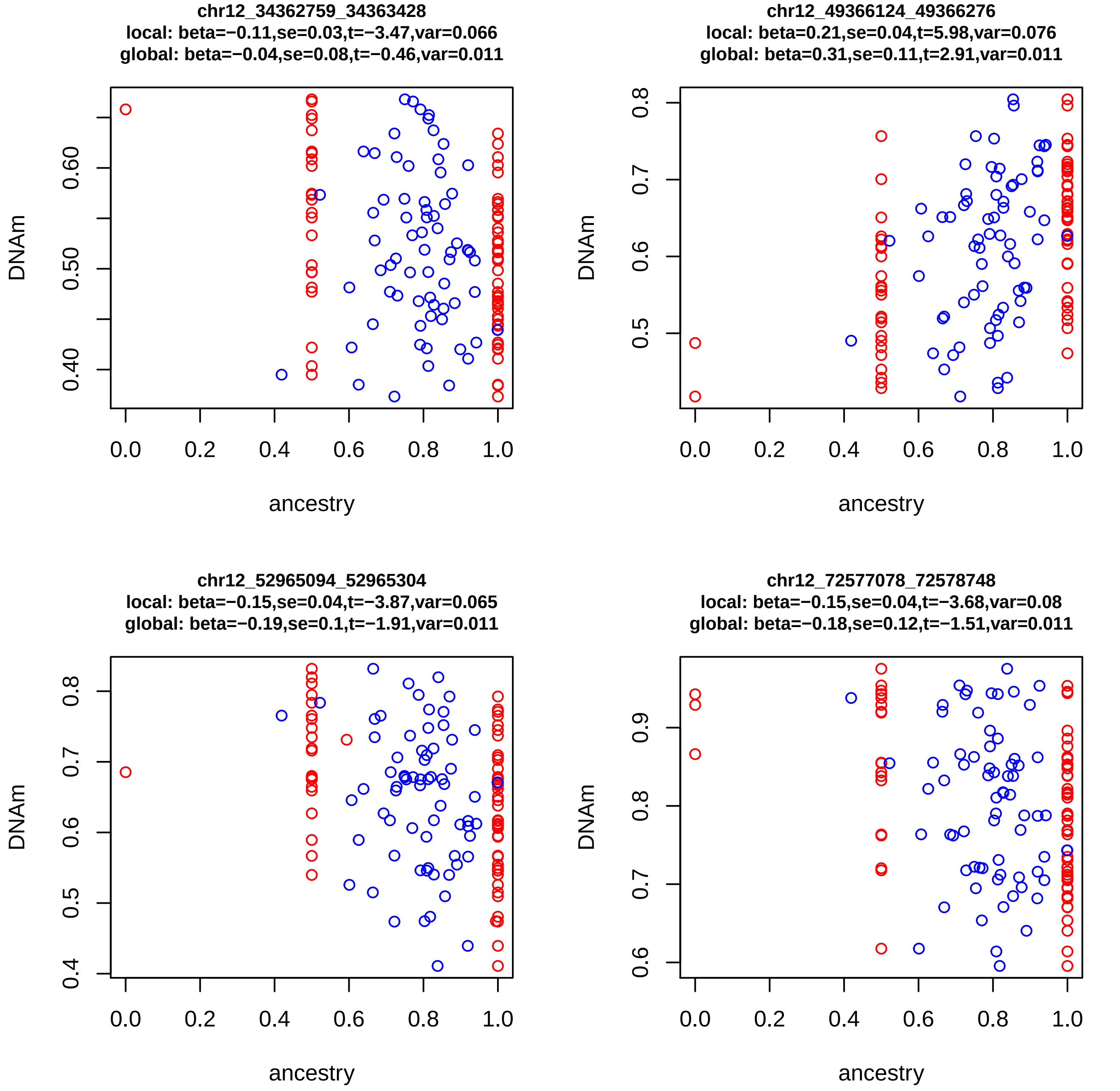
chr12_34362759_34363428
local: beta=−0.11,se=0.03,t=−3.47,var=0.066
global: beta=−0.04,se=0.08,t=−0.46,var=0.011
DNAm
0.40
0.50
0.60
0.0
0.2
0.4
0.6
0.8
1.0
ancestry
chr12_49366124_49366276
local: beta=0.21,se=0.04,t=5.98,var=0.076
global: beta=0.31,se=0.11,t=2.91,var=0.011
DNAm
0.5
0.6
0.7
0.8
ancestry
chr12_52965094_52965304
local: beta=−0.15,se=0.04,t=−3.87,var=0.065
global: beta=−0.19,se=0.1,t=−1.91,var=0.011
DNAm
0.4
0.5
0.6
0.7
0.8
ancestry
chr12_72577078_72578748
local: beta=−0.15,se=0.04,t=−3.68,var=0.08
global: beta=−0.18,se=0.12,t=−1.51,var=0.011
DNAm
0.6
0.7
0.8
0.9
ancestry

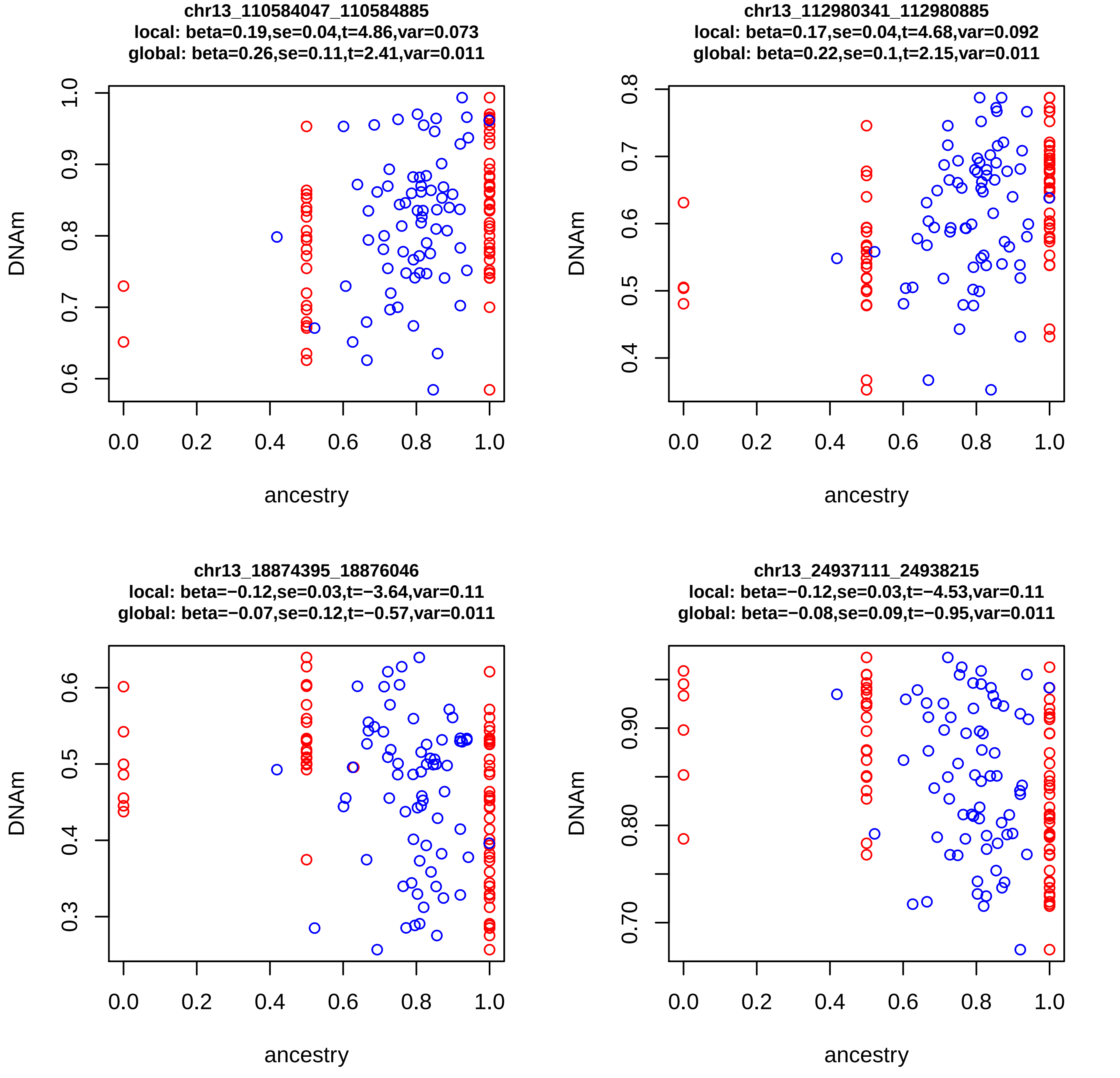
chr13_110584047_110584885
local: beta=0.19,se=0.04,t=4.86,var=0.073
global: beta=0.26,se=0.11,t=2.41,var=0.011
chr13_112980341_112980885
local: beta=0.17,se=0.04,t=4.68,var=0.092
global: beta=0.22,se=0.1,t=2.15,var=0.011
chr13_18874395_18876046
local: beta=−0.12,se=0.03,t=−3.64,var=0.11
global: beta=−0.07,se=0.12,t=−0.57,var=0.011
chr13_24937111_24938215
local: beta=−0.12,se=0.03,t=−4.53,var=0.11
global: beta=−0.08,se=0.09,t=−0.95,var=0.011
DNAm
ancestry

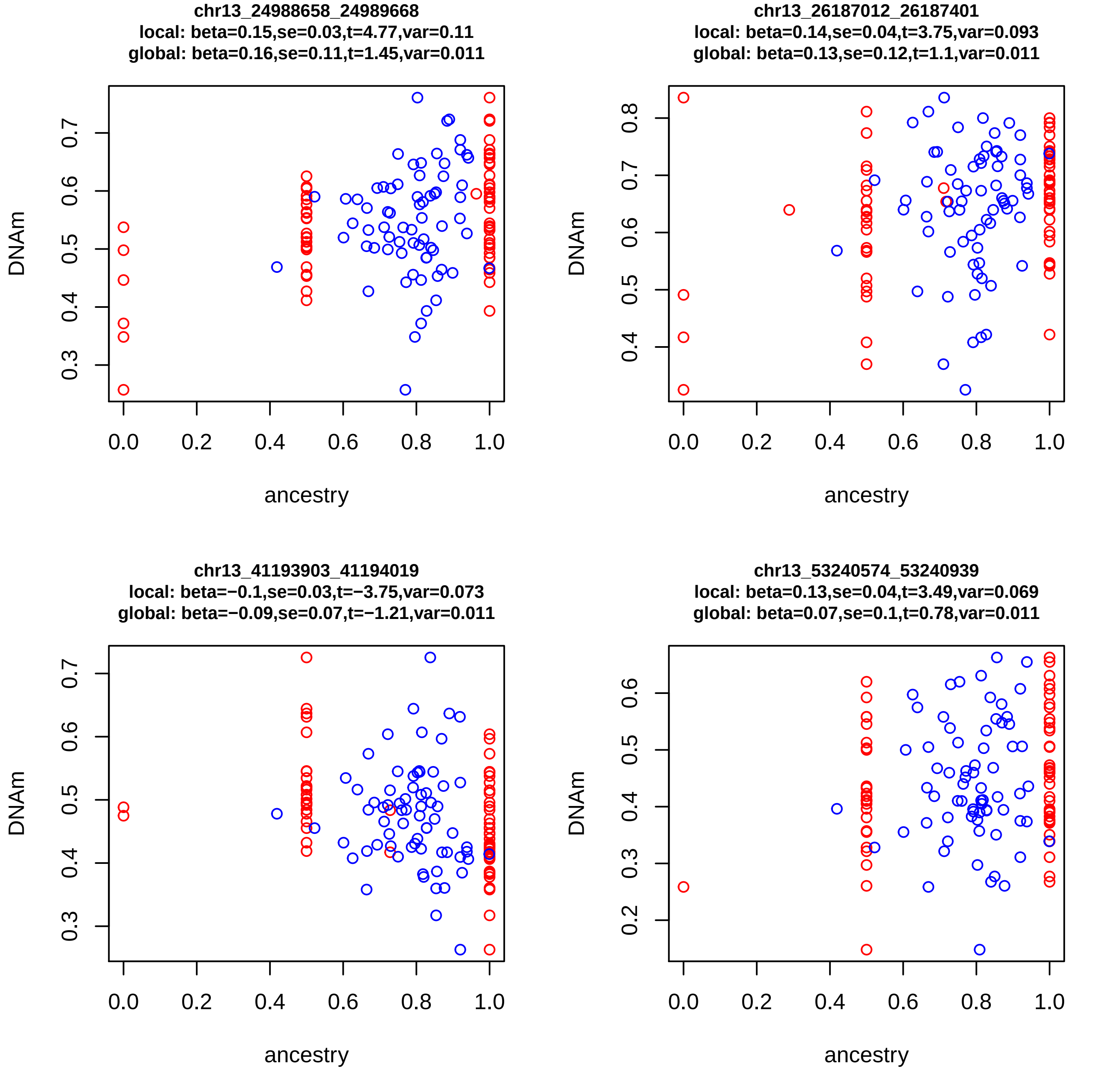
chr13_24988658_24989668
local: beta=0.15,se=0.03,t=4.77,var=0.11
global: beta=0.16,se=0.11,t=1.45,var=0.011
DNAm
ancestry
chr13_26187012_26187401
local: beta=0.14,se=0.04,t=3.75,var=0.093
global: beta=0.13,se=0.12,t=1.1,var=0.011
DNAm
ancestry
chr13_41193903_41194019
local: beta=−0.1,se=0.03,t=−3.75,var=0.073
global: beta=−0.09,se=0.07,t=−1.21,var=0.011
DNAm
ancestry
chr13_53240574_53240939
local: beta=0.13,se=0.04,t=3.49,var=0.069
global: beta=0.07,se=0.1,t=0.78,var=0.011
DNAm
ancestry

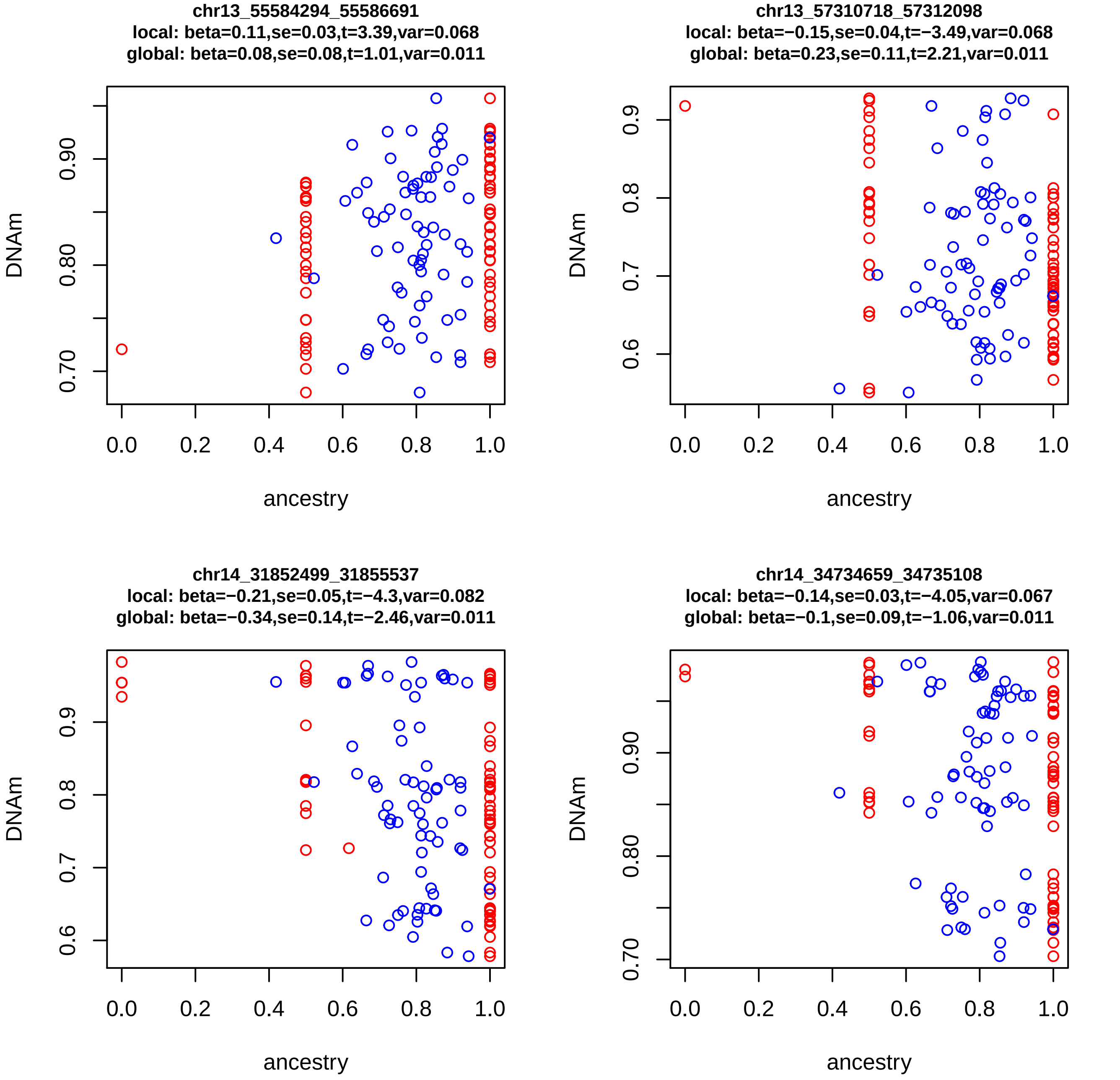
chr13_55584294_55586691
local: beta=0.11,se=0.03,t=3.39,var=0.068
global: beta=0.08,se=0.08,t=1.01,var=0.011
DNAm
ancestry
chr13_57310718_57312098
local: beta=−0.15,se=0.04,t=−3.49,var=0.068
global: beta=0.23,se=0.11,t=2.21,var=0.011
DNAm
ancestry
chr14_31852499_31855537
local: beta=−0.21,se=0.05,t=−4.3,var=0.082
global: beta=−0.34,se=0.14,t=−2.46,var=0.011
DNAm
ancestry
chr14_34734659_34735108
local: beta=−0.14,se=0.03,t=−4.05,var=0.067
global: beta=−0.1,se=0.09,t=−1.06,var=0.011
DNAm
ancestry

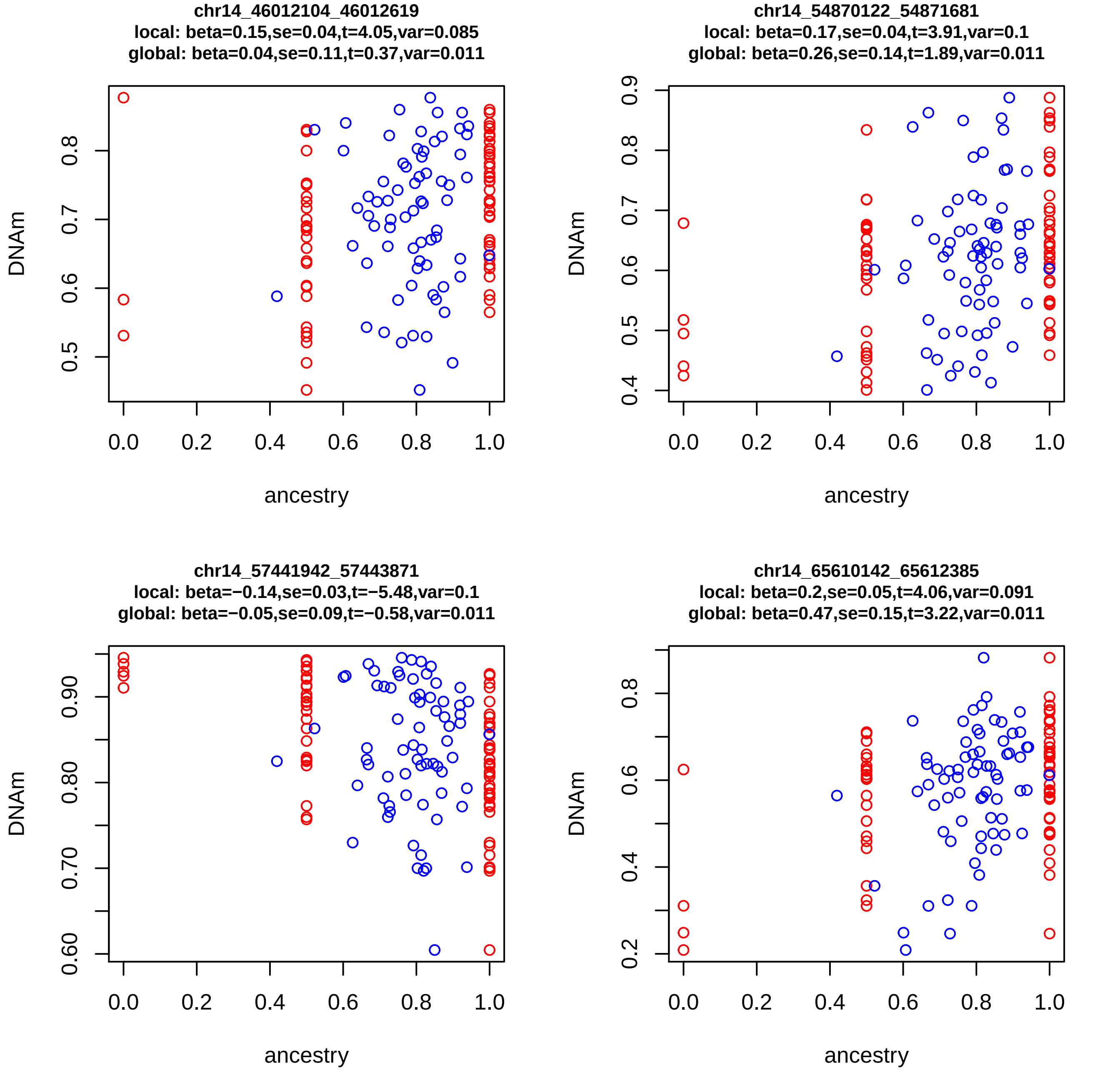
chr14_46012104_46012619
local: beta=0.15,se=0.04,t=4.05,var=0.085
global: beta=0.04,se=0.11,t=0.37,var=0.011
DNAm
ancestry
chr14_54870122_54871681
local: beta=0.17,se=0.04,t=3.91,var=0.1
global: beta=0.26,se=0.14,t=1.89,var=0.011
DNAm
ancestry
chr14_57441942_57443871
local: beta=−0.14,se=0.03,t=−5.48,var=0.1
global: beta=−0.05,se=0.09,t=−0.58,var=0.011
DNAm
ancestry
chr14_65610142_65612385
local: beta=0.2,se=0.05,t=4.06,var=0.091
global: beta=0.47,se=0.15,t=3.22,var=0.011
DNAm
ancestry

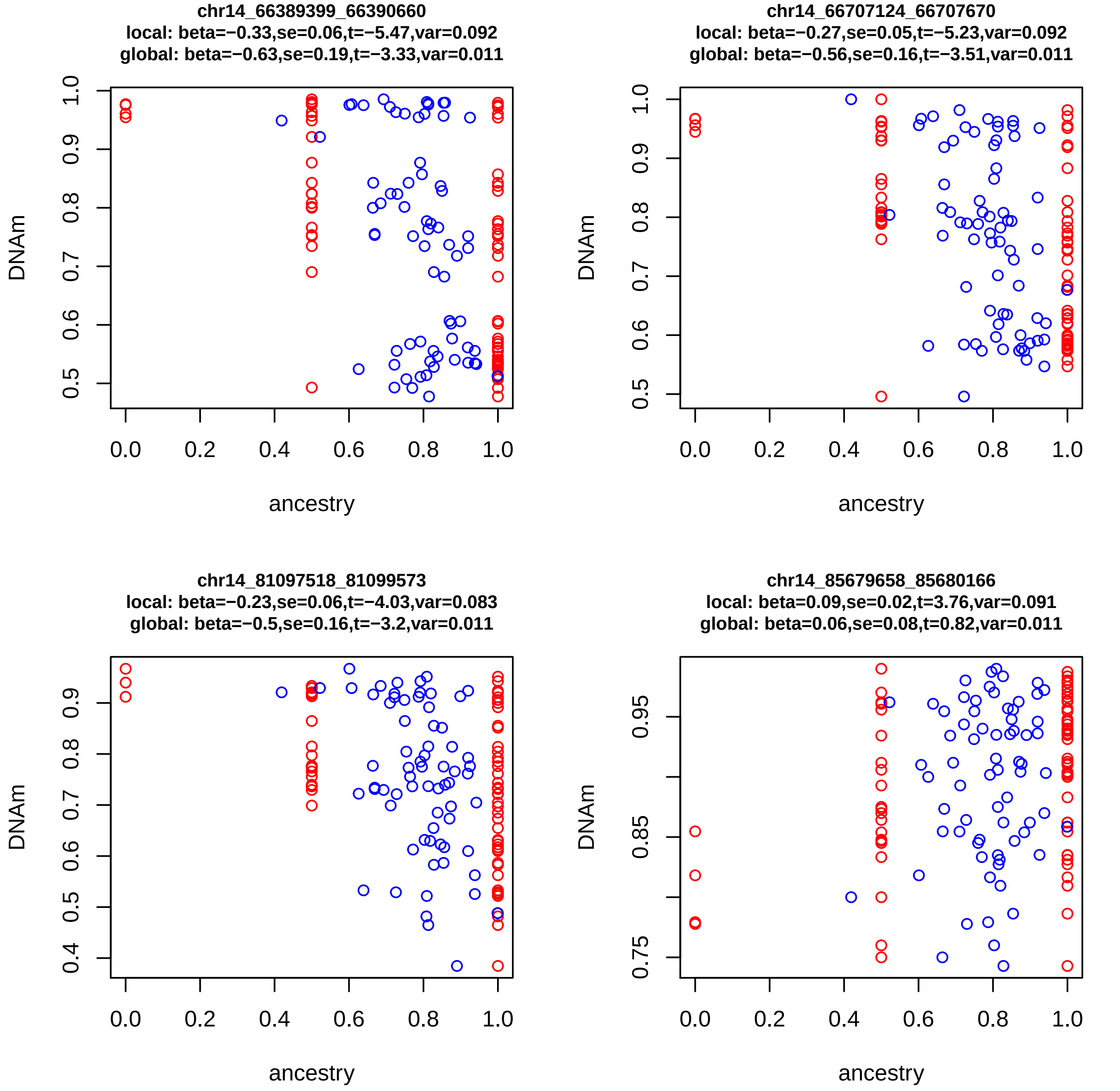
chr14_66389399_66390660
local: beta=−0.33,se=0.06,t=−5.47,var=0.092
global: beta=−0.63,se=0.19,t=−3.33,var=0.011
DNAm
ancestry
chr14_66707124_66707670
local: beta=−0.27,se=0.05,t=−5.23,var=0.092
global: beta=−0.56,se=0.16,t=−3.51,var=0.011
DNAm
ancestry
chr14_81097518_81099573
local: beta=−0.23,se=0.06,t=−4.03,var=0.083
global: beta=−0.5,se=0.16,t=−3.2,var=0.011
DNAm
ancestry
chr14_85679658_85680166
local: beta=0.09,se=0.02,t=3.76,var=0.091
global: beta=0.06,se=0.08,t=0.82,var=0.011
DNAm
ancestry

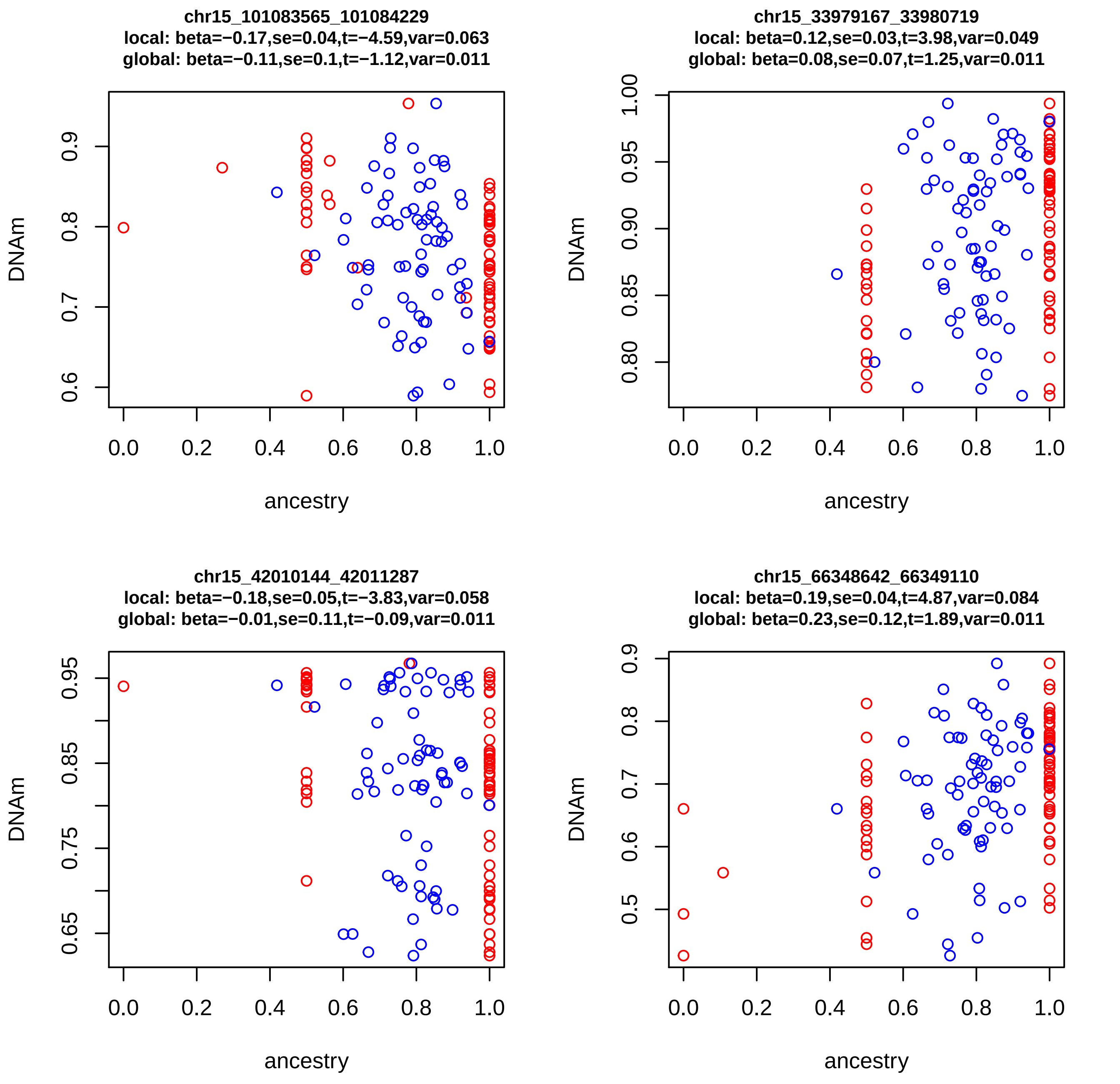
chr15_101083565_101084229
local: beta=−0.17,se=0.04,t=−4.59,var=0.063
global: beta=−0.11,se=0.1,t=−1.12,var=0.011
DNAm
ancestry
chr15_33979167_33980719
local: beta=0.12,se=0.03,t=3.98,var=0.049
global: beta=0.08,se=0.07,t=1.25,var=0.011
DNAm
ancestry
chr15_42010144_42011287
local: beta=−0.18,se=0.05,t=−3.83,var=0.058
global: beta=−0.01,se=0.11,t=−0.09,var=0.011
DNAm
ancestry
chr15_66348642_66349110
local: beta=0.19,se=0.04,t=4.87,var=0.084
global: beta=0.23,se=0.12,t=1.89,var=0.011
DNAm
ancestry

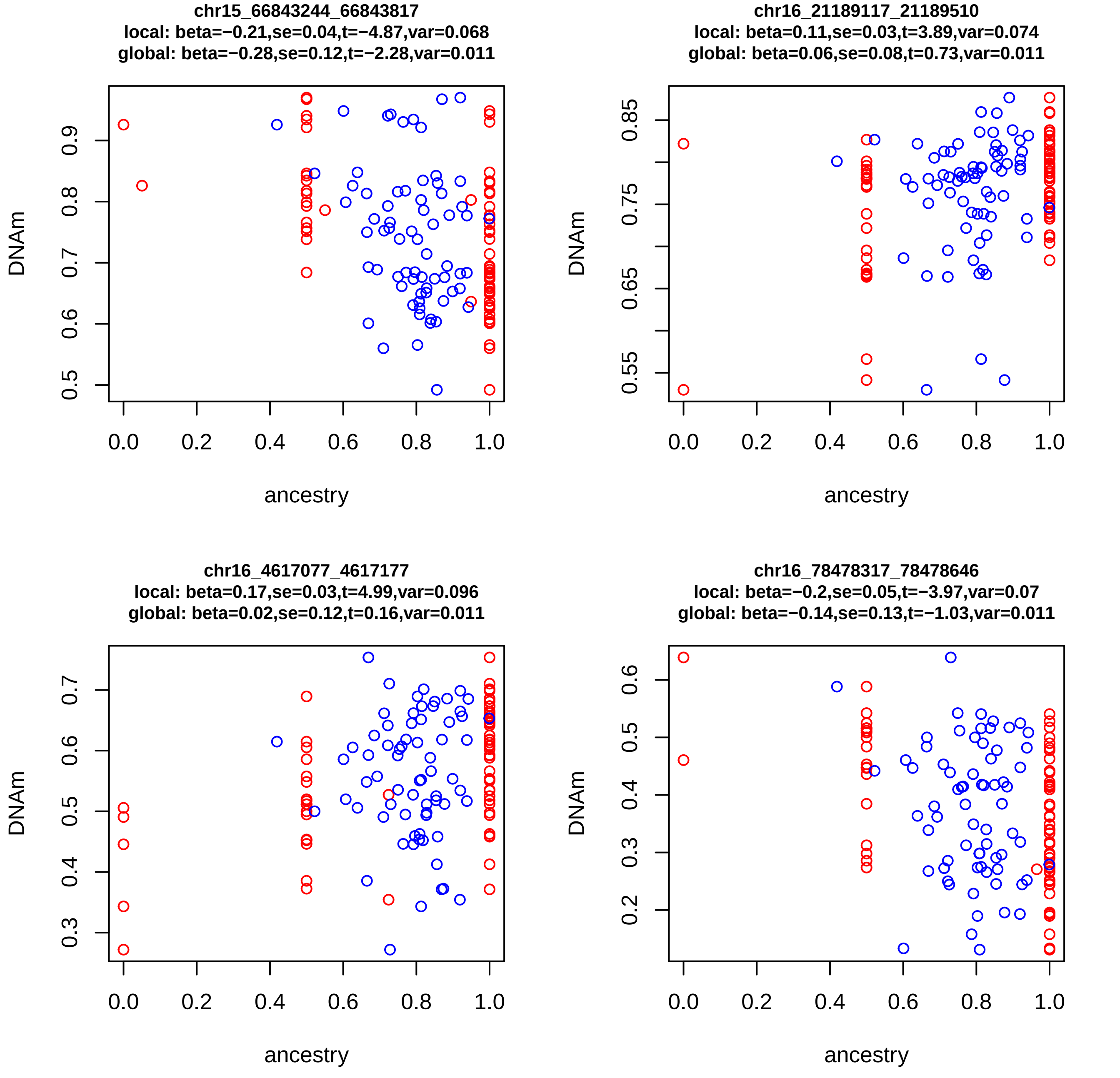
chr15_66843244_66843817
local: beta=−0.21,se=0.04,t=−4.87,var=0.068
global: beta=−0.28,se=0.12,t=−2.28,var=0.011
DNAm
ancestry
chr16_21189117_21189510
local: beta=0.11,se=0.03,t=3.89,var=0.074
global: beta=0.06,se=0.08,t=0.73,var=0.011
DNAm
ancestry
chr16_4617077_4617177
local: beta=0.17,se=0.03,t=4.99,var=0.096
global: beta=0.02,se=0.12,t=0.16,var=0.011
DNAm
ancestry
chr16_78478317_78478646
local: beta=−0.2,se=0.05,t=−3.97,var=0.07
global: beta=−0.14,se=0.13,t=−1.03,var=0.011
DNAm
ancestry

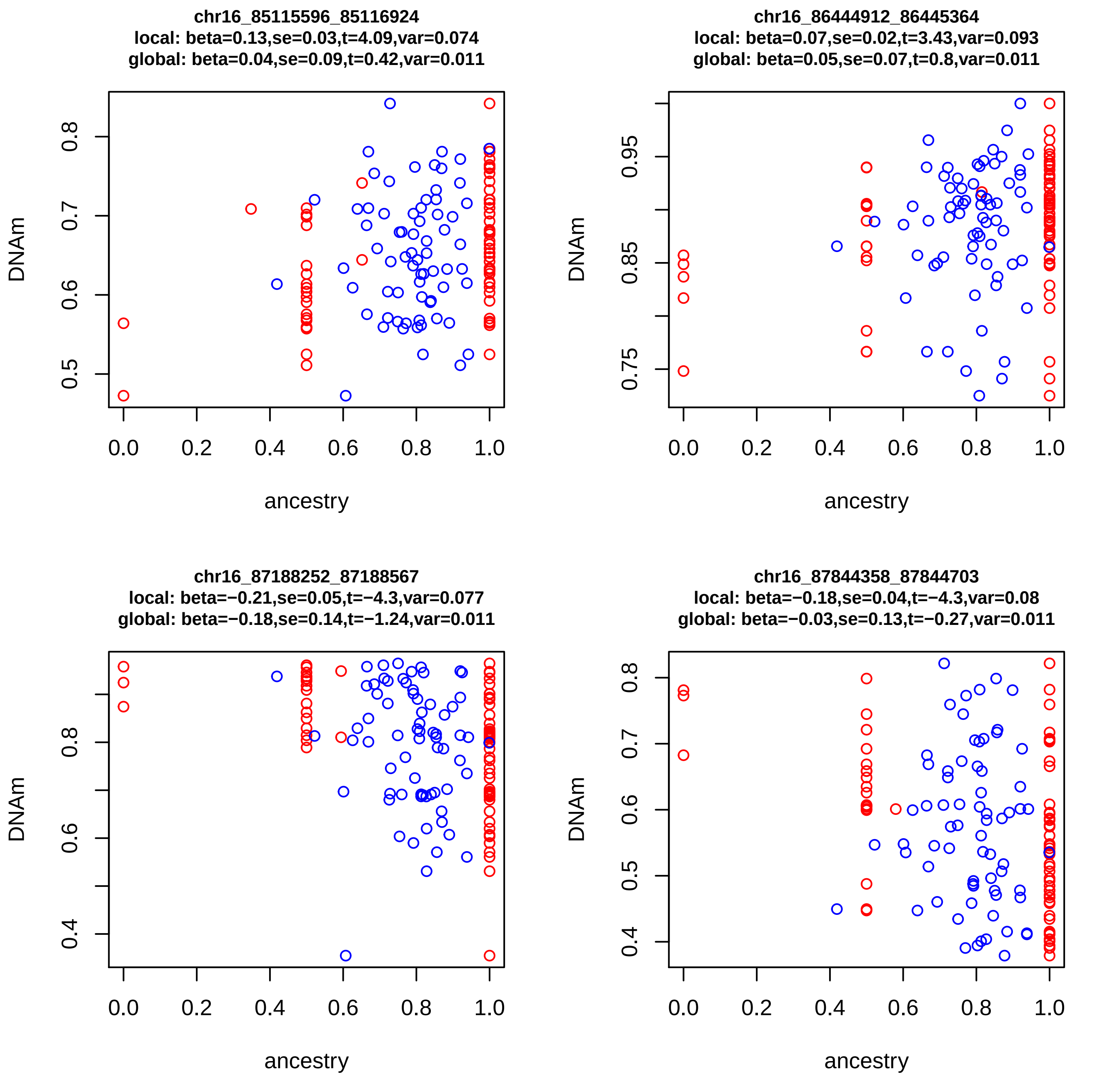
chr16_85115596_85116924
local: beta=0.13,se=0.03,t=4.09,var=0.074
global: beta=0.04,se=0.09,t=0.42,var=0.011
DNAm
ancestry
chr16_86444912_86445364
local: beta=0.07,se=0.02,t=3.43,var=0.093
global: beta=0.05,se=0.07,t=0.8,var=0.011
DNAm
ancestry
chr16_87188252_87188567
local: beta=−0.21,se=0.05,t=−4.3,var=0.077
global: beta=−0.18,se=0.14,t=−1.24,var=0.011
DNAm
ancestry
chr16_87844358_87844703
local: beta=−0.18,se=0.04,t=−4.3,var=0.08
global: beta=−0.03,se=0.13,t=−0.27,var=0.011
DNAm
ancestry

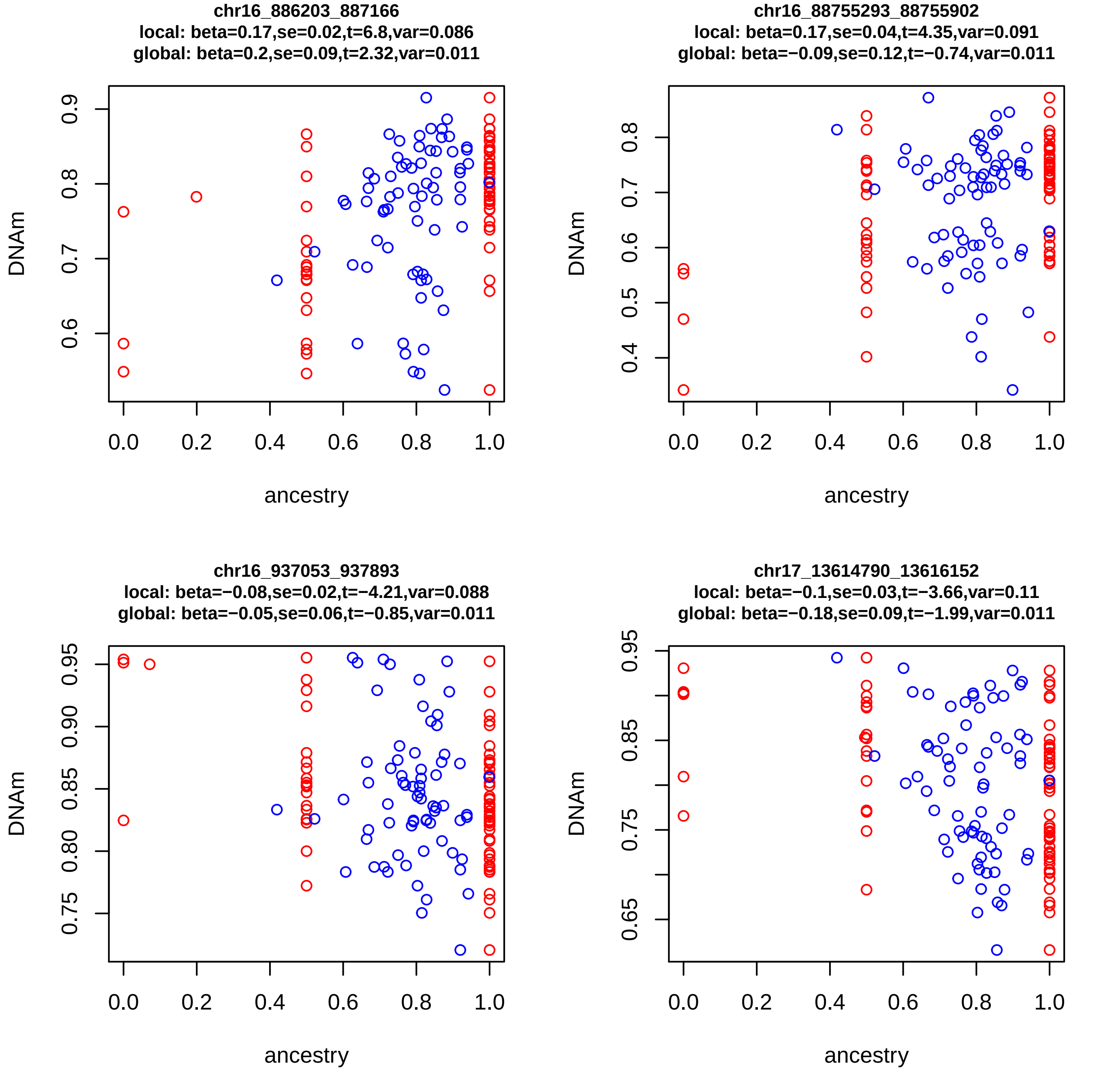
chr16_886203_887166
local: beta=0.17,se=0.02,t=6.8,var=0.086
global: beta=0.2,se=0.09,t=2.32,var=0.011
DNAm
ancestry
chr16_88755293_88755902
local: beta=0.17,se=0.04,t=4.35,var=0.091
global: beta=−0.09,se=0.12,t=−0.74,var=0.011
DNAm
ancestry
chr16_937053_937893
local: beta=−0.08,se=0.02,t=−4.21,var=0.088
global: beta=−0.05,se=0.06,t=−0.85,var=0.011
DNAm
ancestry
chr17_13614790_13616152
local: beta=−0.1,se=0.03,t=−3.66,var=0.11
global: beta=−0.18,se=0.09,t=−1.99,var=0.011
DNAm
ancestry

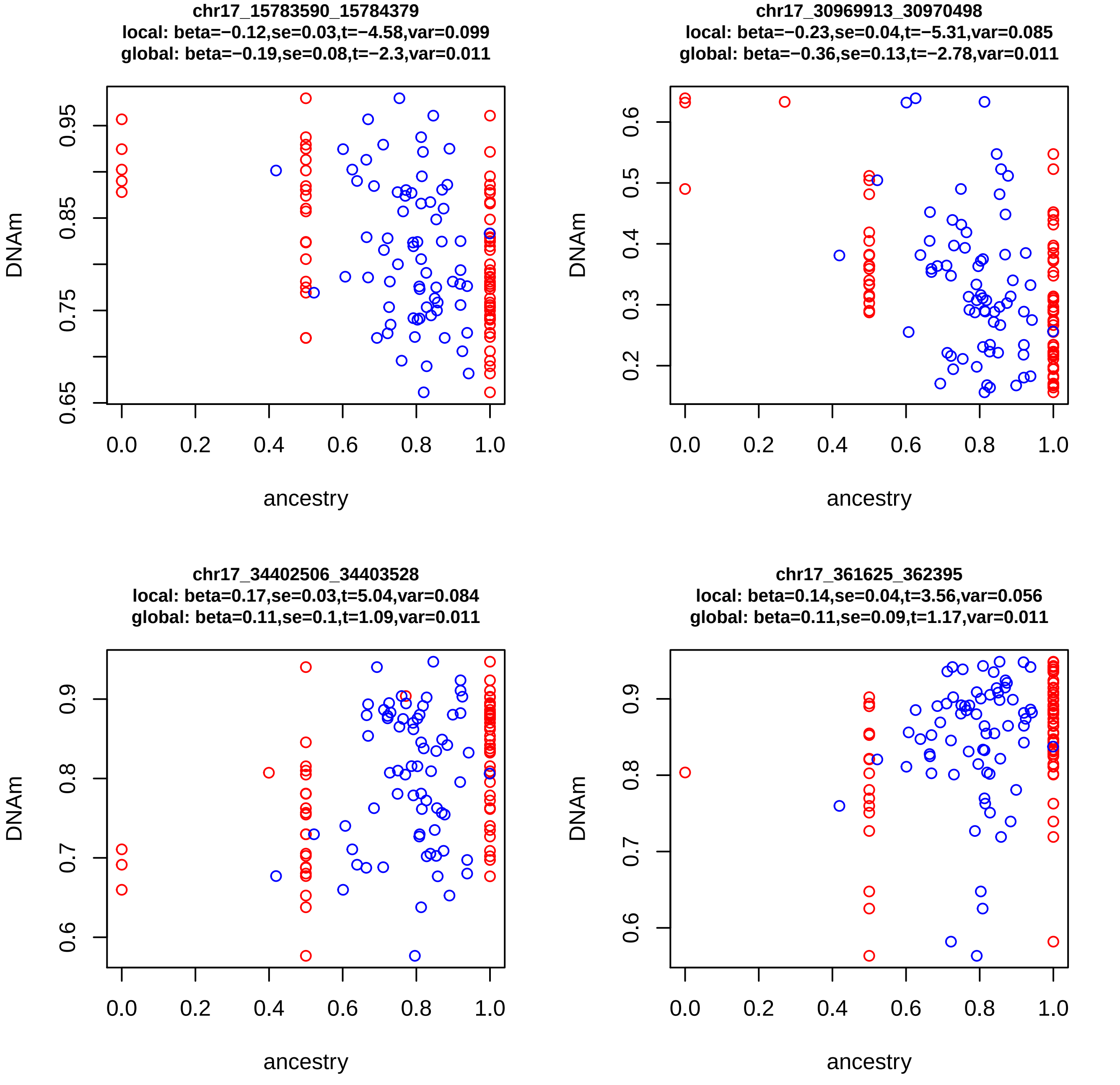
chr17_15783590_15784379
local: beta=−0.12,se=0.03,t=−4.58,var=0.099
global: beta=−0.19,se=0.08,t=−2.3,var=0.011
chr17_30969913_30970498
local: beta=−0.23,se=0.04,t=−5.31,var=0.085
global: beta=−0.36,se=0.13,t=−2.78,var=0.011
chr17_34402506_34403528
local: beta=0.17,se=0.03,t=5.04,var=0.084
global: beta=0.11,se=0.1,t=1.09,var=0.011
chr17_361625_362395
local: beta=0.14,se=0.04,t=3.56,var=0.056
global: beta=0.11,se=0.09,t=1.17,var=0.011
DNAm
ancestry
0.0
0.2
0.4
0.6
0.8
1.0
0.65
0.75
0.85
0.95
0.2
0.3
0.4
0.5
0.6
0.7
0.8
0.9

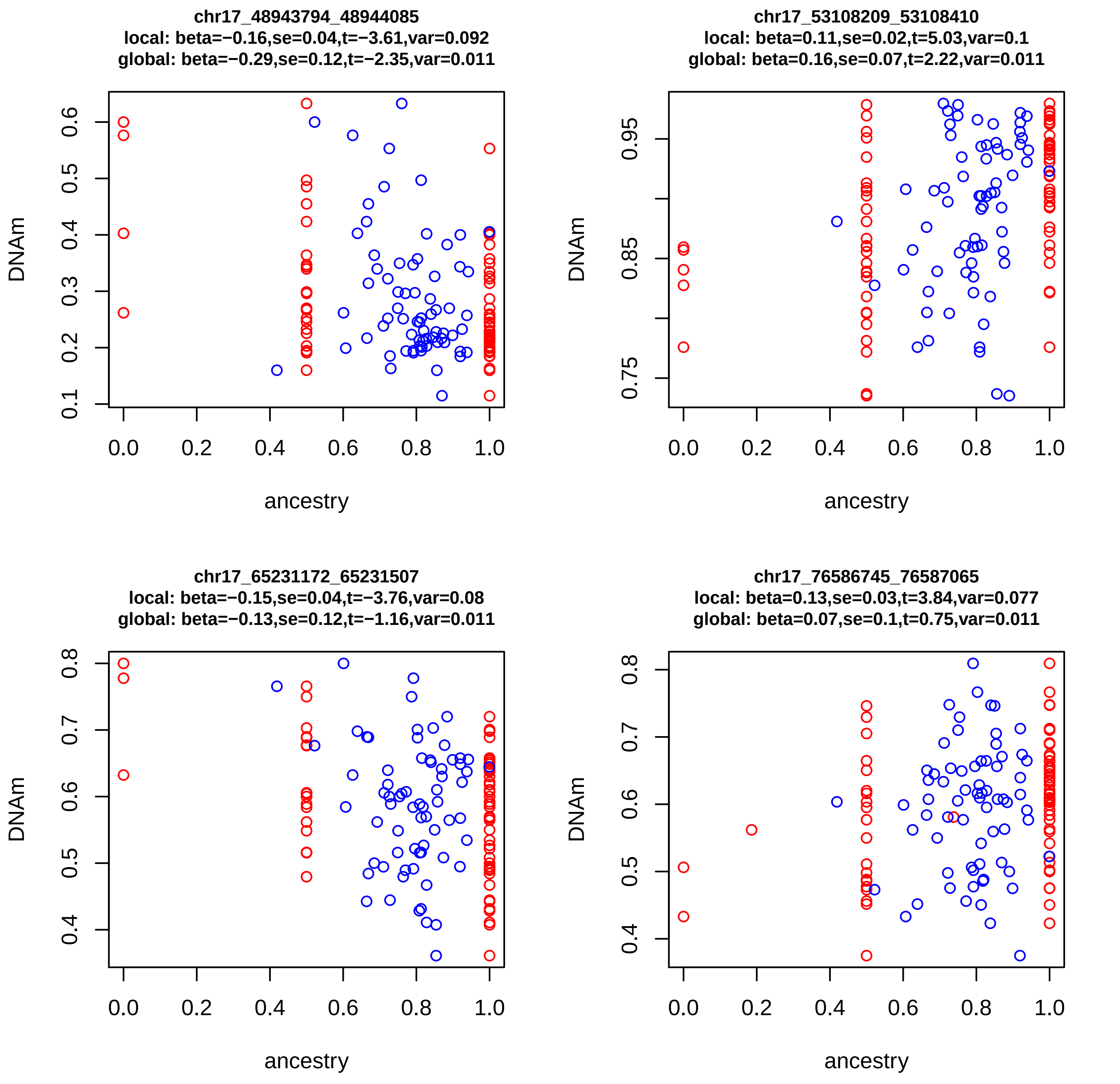

chr17_48943794_48944085
local: beta=−0.16,se=0.04,t=−3.61,var=0.092
global: beta=−0.29,se=0.12,t=−2.35,var=0.011
DNAm
ancestry
chr17_53108209_53108410
local: beta=0.11,se=0.02,t=5.03,var=0.1
global: beta=0.16,se=0.07,t=2.22,var=0.011
DNAm
ancestry
chr17_65231172_65231507
local: beta=−0.15,se=0.04,t=−3.76,var=0.08
global: beta=−0.13,se=0.12,t=−1.16,var=0.011
DNAm
ancestry
chr17_76586745_76587065
local: beta=0.13,se=0.03,t=3.84,var=0.077
global: beta=0.07,se=0.1,t=0.75,var=0.011
DNAm
ancestry

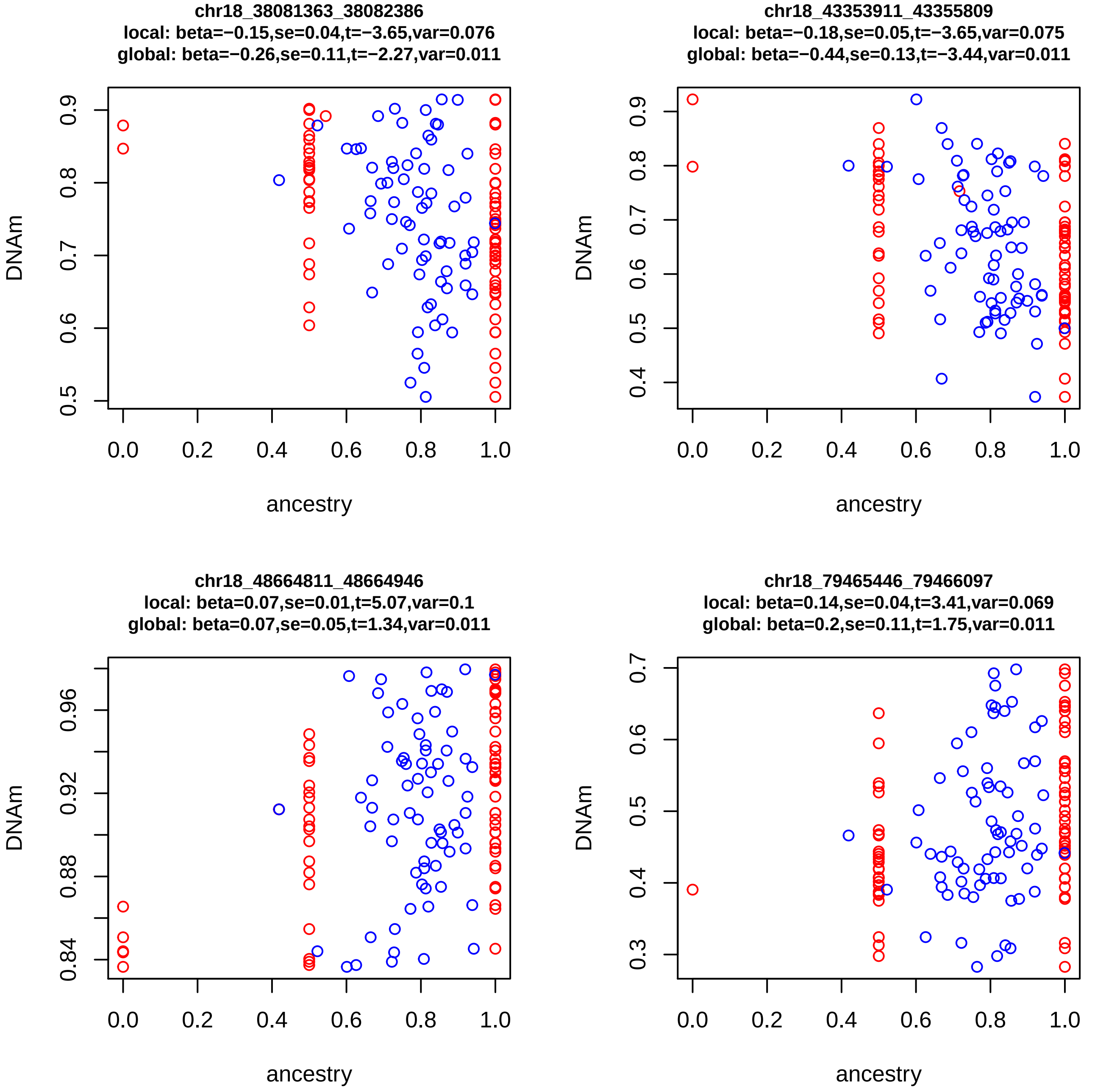

chr18_38081363_38082386
local: beta=−0.15,se=0.04,t=−3.65,var=0.076
global: beta=−0.26,se=0.11,t=−2.27,var=0.011
DNAm
ancestry
chr18_43353911_43355809
local: beta=−0.18,se=0.05,t=−3.65,var=0.075
global: beta=−0.44,se=0.13,t=−3.44,var=0.011
DNAm
ancestry
chr18_48664811_48664946
local: beta=0.07,se=0.01,t=5.07,var=0.1
global: beta=0.07,se=0.05,t=1.34,var=0.011
DNAm
ancestry
chr18_79465446_79466097
local: beta=0.14,se=0.04,t=3.41,var=0.069
global: beta=0.2,se=0.11,t=1.75,var=0.011
DNAm
ancestry

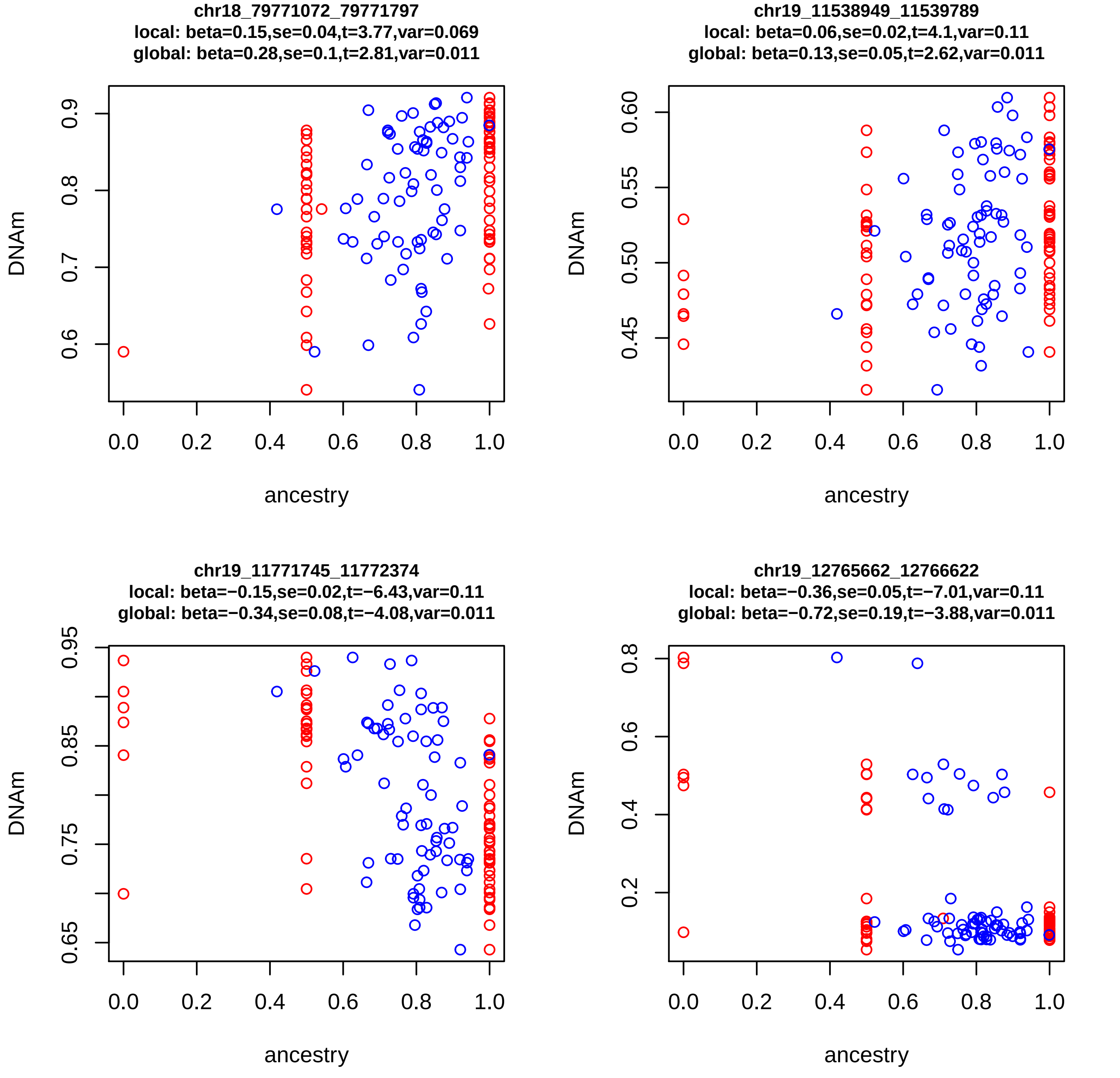
chr18_79771072_79771797
local: beta=0.15,se=0.04,t=3.77,var=0.069
global: beta=0.28,se=0.1,t=2.81,var=0.011
DNAm
0.6
0.7
0.8
0.9
0.0
0.2
0.4
0.6
0.8
1.0
ancestry
chr19_11538949_11539789
local: beta=0.06,se=0.02,t=4.1,var=0.11
global: beta=0.13,se=0.05,t=2.62,var=0.011
DNAm
0.45
0.50
0.55
0.60
0.0
0.2
0.4
0.6
0.8
1.0
ancestry
chr19_11771745_11772374
local: beta=−0.15,se=0.02,t=−6.43,var=0.11
global: beta=−0.34,se=0.08,t=−4.08,var=0.011
DNAm
0.65
0.75
0.85
0.95
0.0
0.2
0.4
0.6
0.8
1.0
ancestry
chr19_12765662_12766622
local: beta=−0.36,se=0.05,t=−7.01,var=0.11
global: beta=−0.72,se=0.19,t=−3.88,var=0.011
DNAm
0.2
0.4
0.6
0.8
0.0
0.2
0.4
0.6
0.8
1.0
ancestry

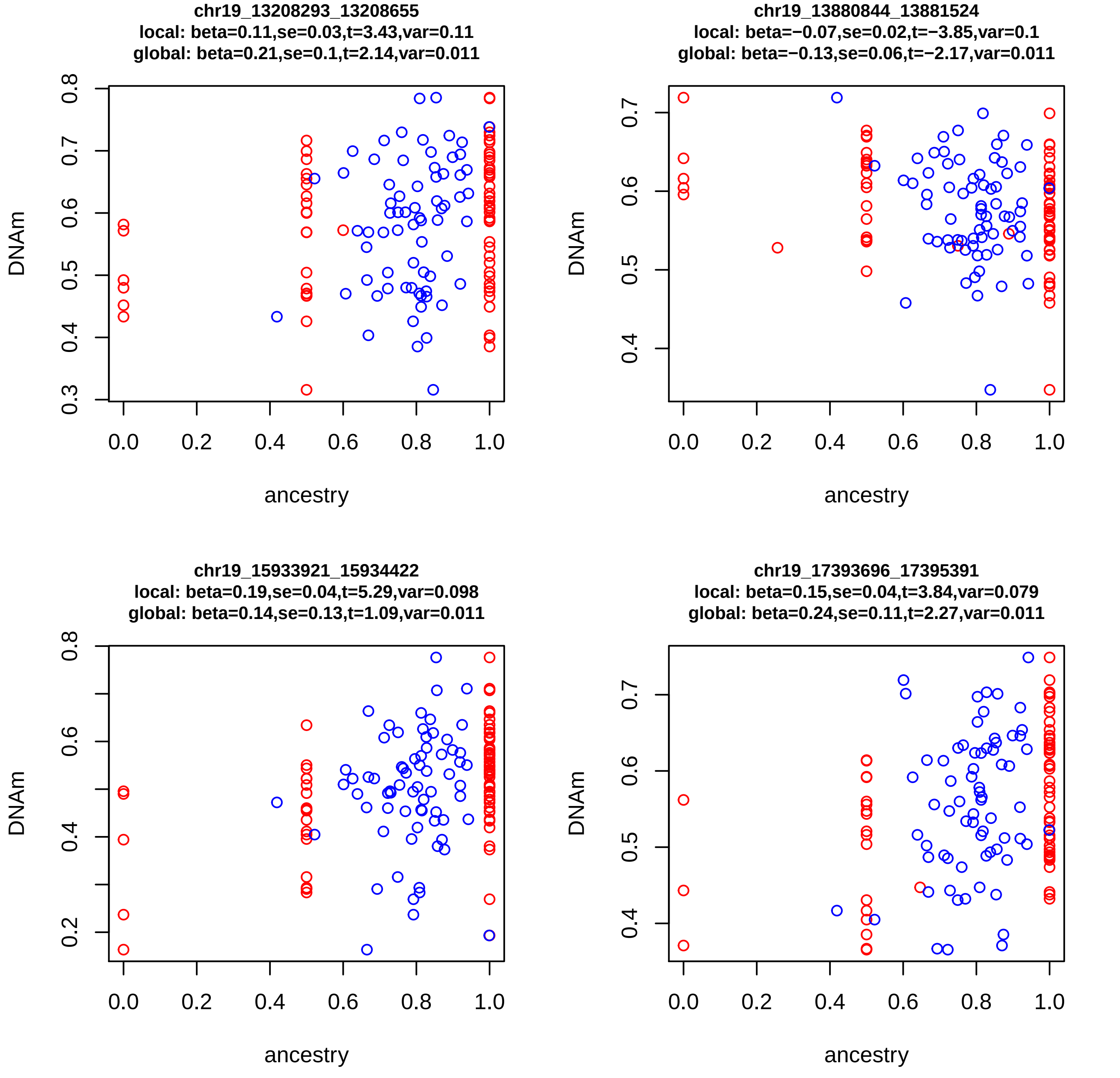

chr19_13208293_13208655
local: beta=0.11,se=0.03,t=3.43,var=0.11
global: beta=0.21,se=0.1,t=2.14,var=0.011
DNAm
ancestry
chr19_13880844_13881524
local: beta=−0.07,se=0.02,t=−3.85,var=0.1
global: beta=−0.13,se=0.06,t=−2.17,var=0.011
DNAm
ancestry
chr19_15933921_15934422
local: beta=0.19,se=0.04,t=5.29,var=0.098
global: beta=0.14,se=0.13,t=1.09,var=0.011
DNAm
ancestry
chr19_17393696_17395391
local: beta=0.15,se=0.04,t=3.84,var=0.079
global: beta=0.24,se=0.11,t=2.27,var=0.011
DNAm
ancestry

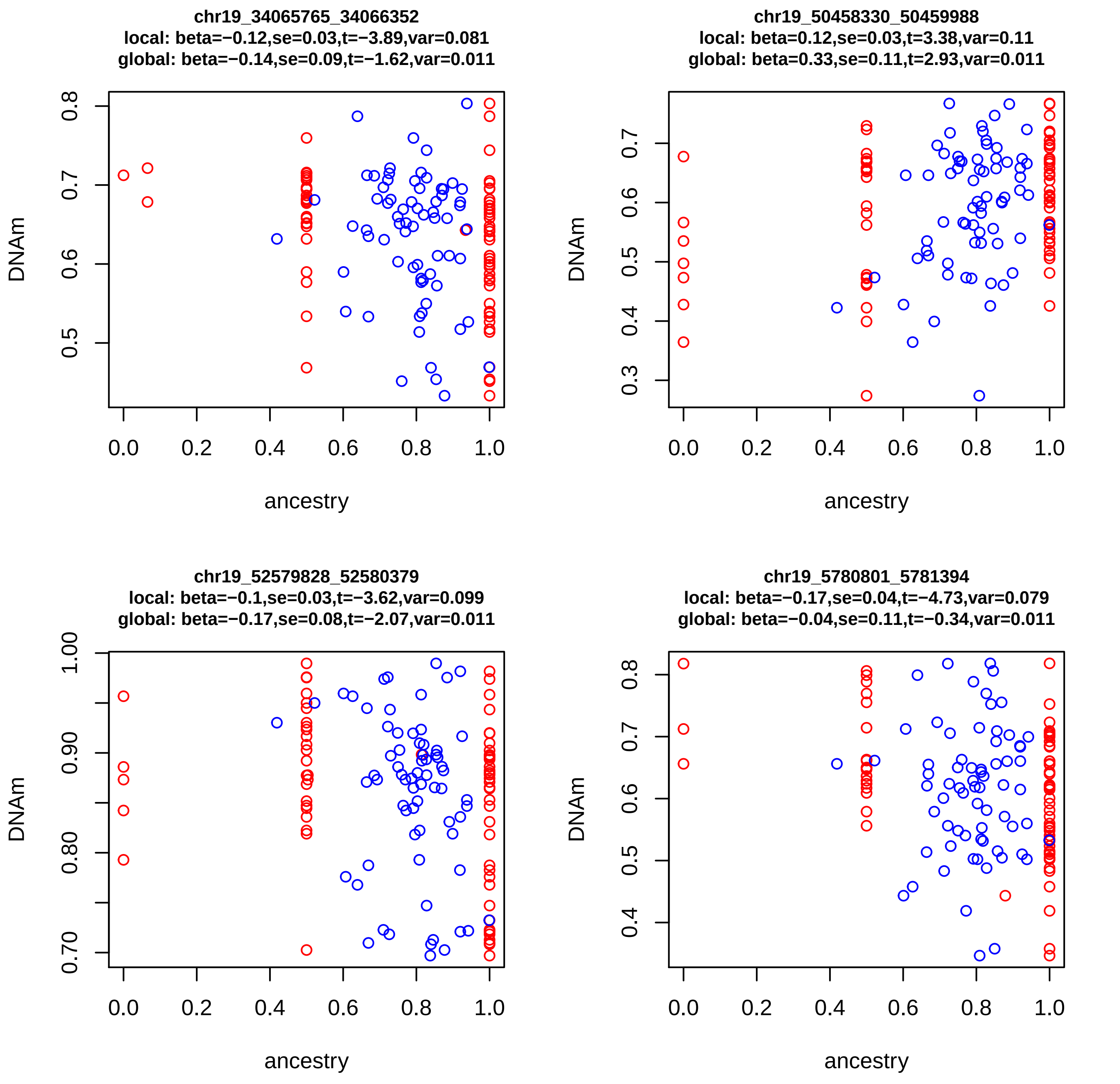

chr19_34065765_34066352
local: beta=−0.12,se=0.03,t=−3.89,var=0.081
global: beta=−0.14,se=0.09,t=−1.62,var=0.011
DNAm
ancestry
chr19_50458330_50459988
local: beta=0.12,se=0.03,t=3.38,var=0.11
global: beta=0.33,se=0.11,t=2.93,var=0.011
DNAm
ancestry
chr19_52579828_52580379
local: beta=−0.1,se=0.03,t=−3.62,var=0.099
global: beta=−0.17,se=0.08,t=−2.07,var=0.011
DNAm
ancestry
chr19_5780801_5781394
local: beta=−0.17,se=0.04,t=−4.73,var=0.079
global: beta=−0.04,se=0.11,t=−0.34,var=0.011
DNAm
ancestry

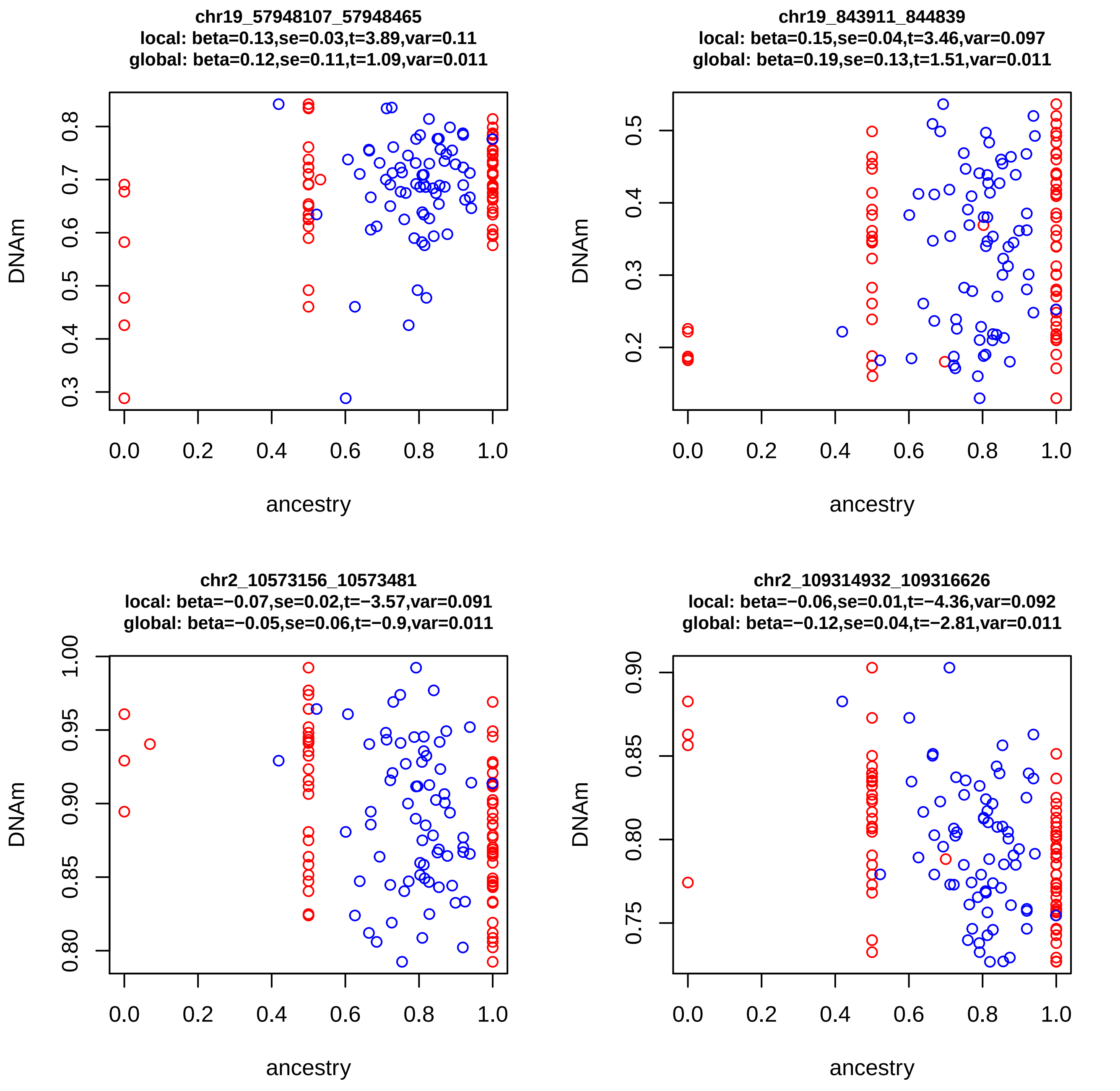
chr19_57948107_57948465
local: beta=0.13,se=0.03,t=3.89,var=0.11
global: beta=0.12,se=0.11,t=1.09,var=0.011
DNAm
0.3
0.4
0.5
0.6
0.7
0.8
0.0
0.2
0.4
0.6
0.8
1.0
ancestry
chr19_843911_844839
local: beta=0.15,se=0.04,t=3.46,var=0.097
global: beta=0.19,se=0.13,t=1.51,var=0.011
DNAm
0.2
0.3
0.4
0.5
0.0
0.2
0.4
0.6
0.8
1.0
ancestry
chr2_10573156_10573481
local: beta=−0.07,se=0.02,t=−3.57,var=0.091
global: beta=−0.05,se=0.06,t=−0.9,var=0.011
DNAm
0.80
0.85
0.90
0.95
1.00
0.0
0.2
0.4
0.6
0.8
1.0
ancestry
chr2_109314932_109316626
local: beta=−0.06,se=0.01,t=−4.36,var=0.092
global: beta=−0.12,se=0.04,t=−2.81,var=0.011
DNAm
0.75
0.80
0.85
0.90
0.0
0.2
0.4
0.6
0.8
1.0
ancestry

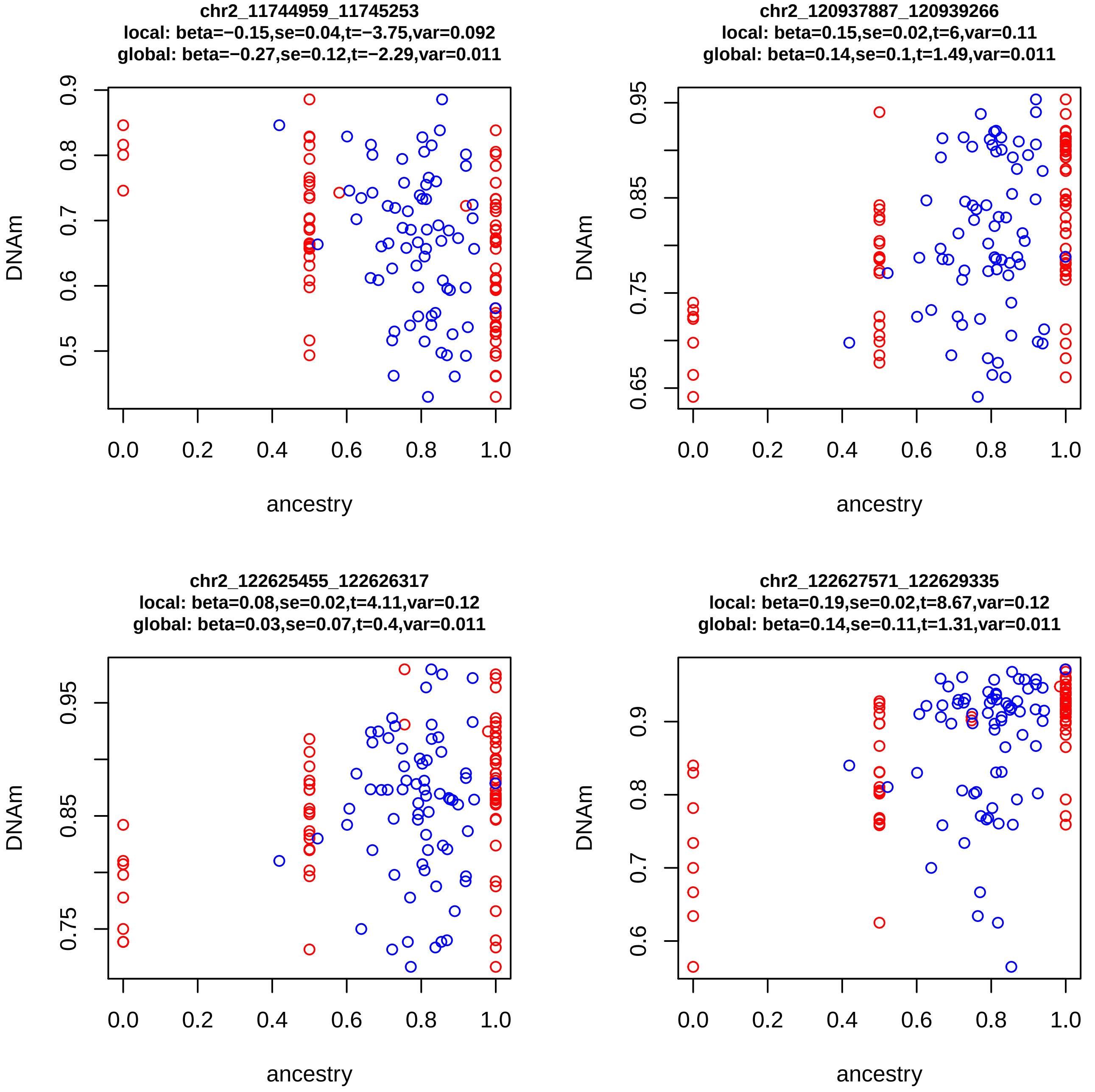
chr2_11744959_11745253
local: beta=−0.15,se=0.04,t=−3.75,var=0.092
global: beta=−0.27,se=0.12,t=−2.29,var=0.011
DNAm
0.5
0.6
0.7
0.8
0.9
0.0
0.2
0.4
0.6
0.8
1.0
ancestry
chr2_120937887_120939266
local: beta=0.15,se=0.02,t=6,var=0.11
global: beta=0.14,se=0.1,t=1.49,var=0.011
DNAm
0.65
0.75
0.85
0.95
0.0
0.2
0.4
0.6
0.8
1.0
ancestry
chr2_122625455_122626317
local: beta=0.08,se=0.02,t=4.11,var=0.12
global: beta=0.03,se=0.07,t=0.4,var=0.011
DNAm
0.75
0.85
0.95
0.0
0.2
0.4
0.6
0.8
1.0
ancestry
chr2_122627571_122629335
local: beta=0.19,se=0.02,t=8.67,var=0.12
global: beta=0.14,se=0.11,t=1.31,var=0.011
DNAm
0.6
0.7
0.8
0.9
0.0
0.2
0.4
0.6
0.8
1.0
ancestry

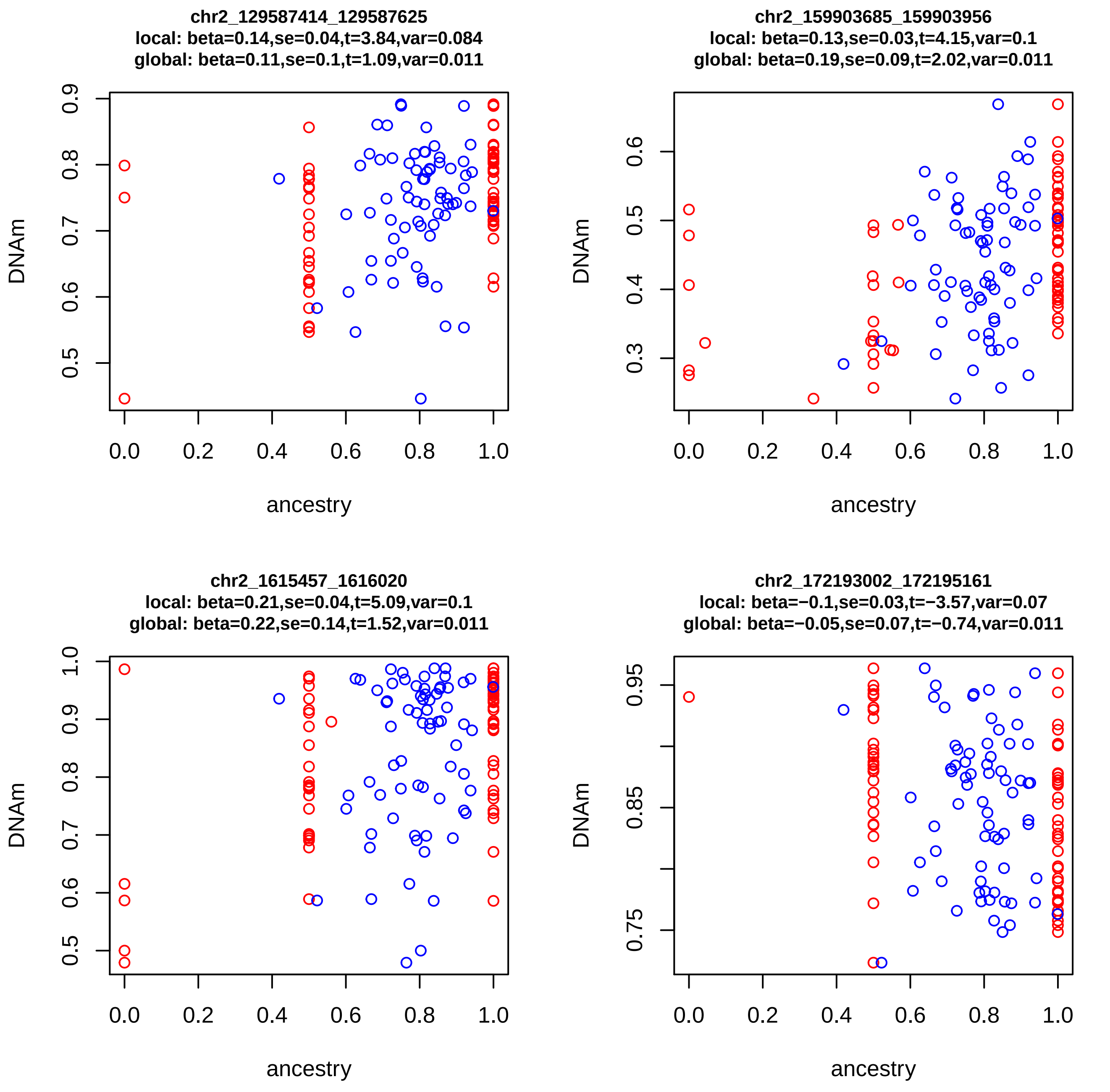
chr2_129587414_129587625
local: beta=0.14,se=0.04,t=3.84,var=0.084
global: beta=0.11,se=0.1,t=1.09,var=0.011
DNAm
ancestry
chr2_159903685_159903956
local: beta=0.13,se=0.03,t=4.15,var=0.1
global: beta=0.19,se=0.09,t=2.02,var=0.011
DNAm
ancestry
chr2_1615457_1616020
local: beta=0.21,se=0.04,t=5.09,var=0.1
global: beta=0.22,se=0.14,t=1.52,var=0.011
DNAm
ancestry
chr2_172193002_172195161
local: beta=−0.1,se=0.03,t=−3.57,var=0.07
global: beta=−0.05,se=0.07,t=−0.74,var=0.011
DNAm
ancestry

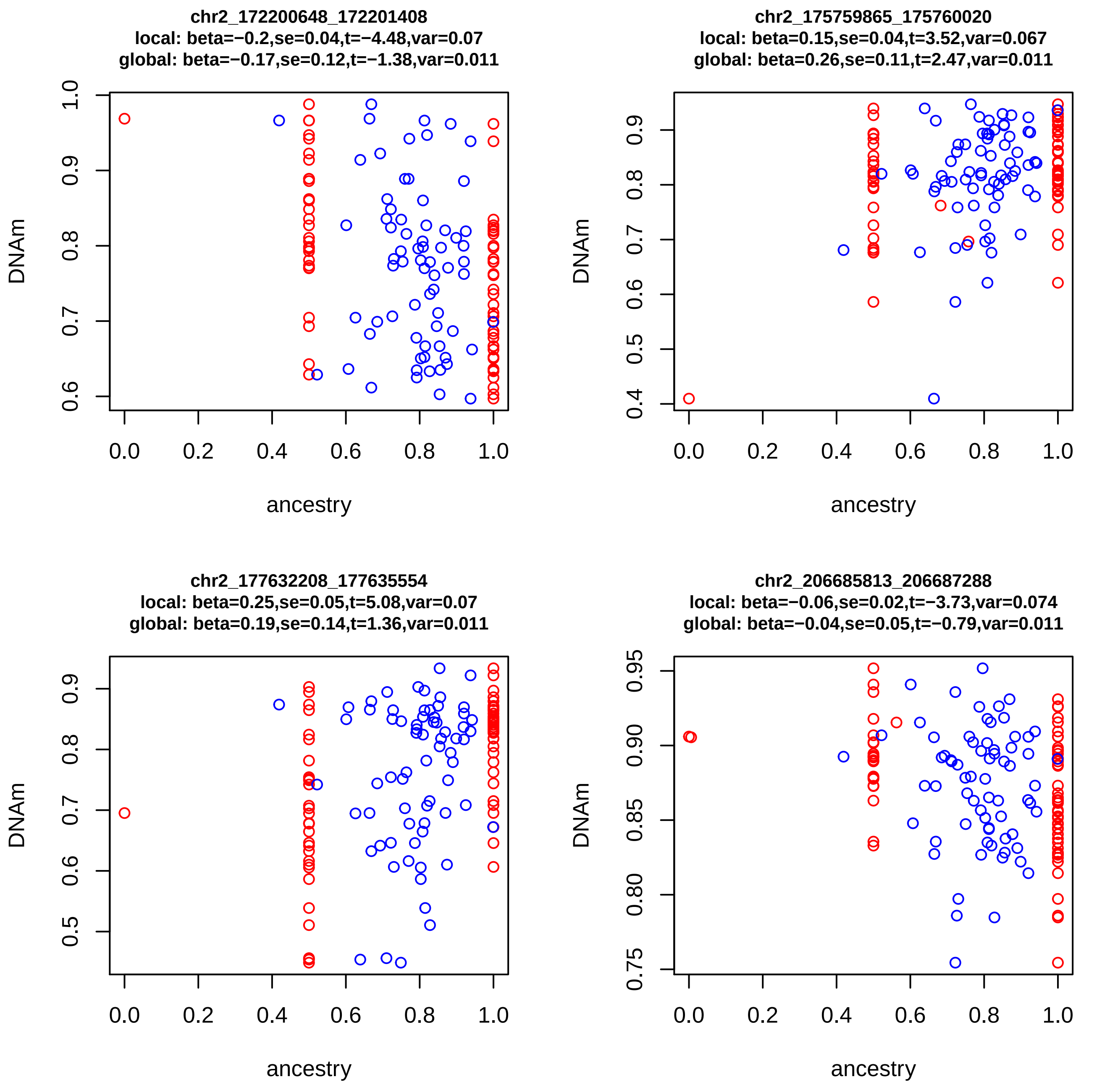

chr2_172200648_172201408
local: beta=−0.2,se=0.04,t=−4.48,var=0.07
global: beta=−0.17,se=0.12,t=−1.38,var=0.011
DNAm
ancestry
chr2_175759865_175760020
local: beta=0.15,se=0.04,t=3.52,var=0.067
global: beta=0.26,se=0.11,t=2.47,var=0.011
DNAm
ancestry
chr2_177632208_177635554
local: beta=0.25,se=0.05,t=5.08,var=0.07
global: beta=0.19,se=0.14,t=1.36,var=0.011
DNAm
ancestry
chr2_206685813_206687288
local: beta=−0.06,se=0.02,t=−3.73,var=0.074
global: beta=−0.04,se=0.05,t=−0.79,var=0.011
DNAm
ancestry

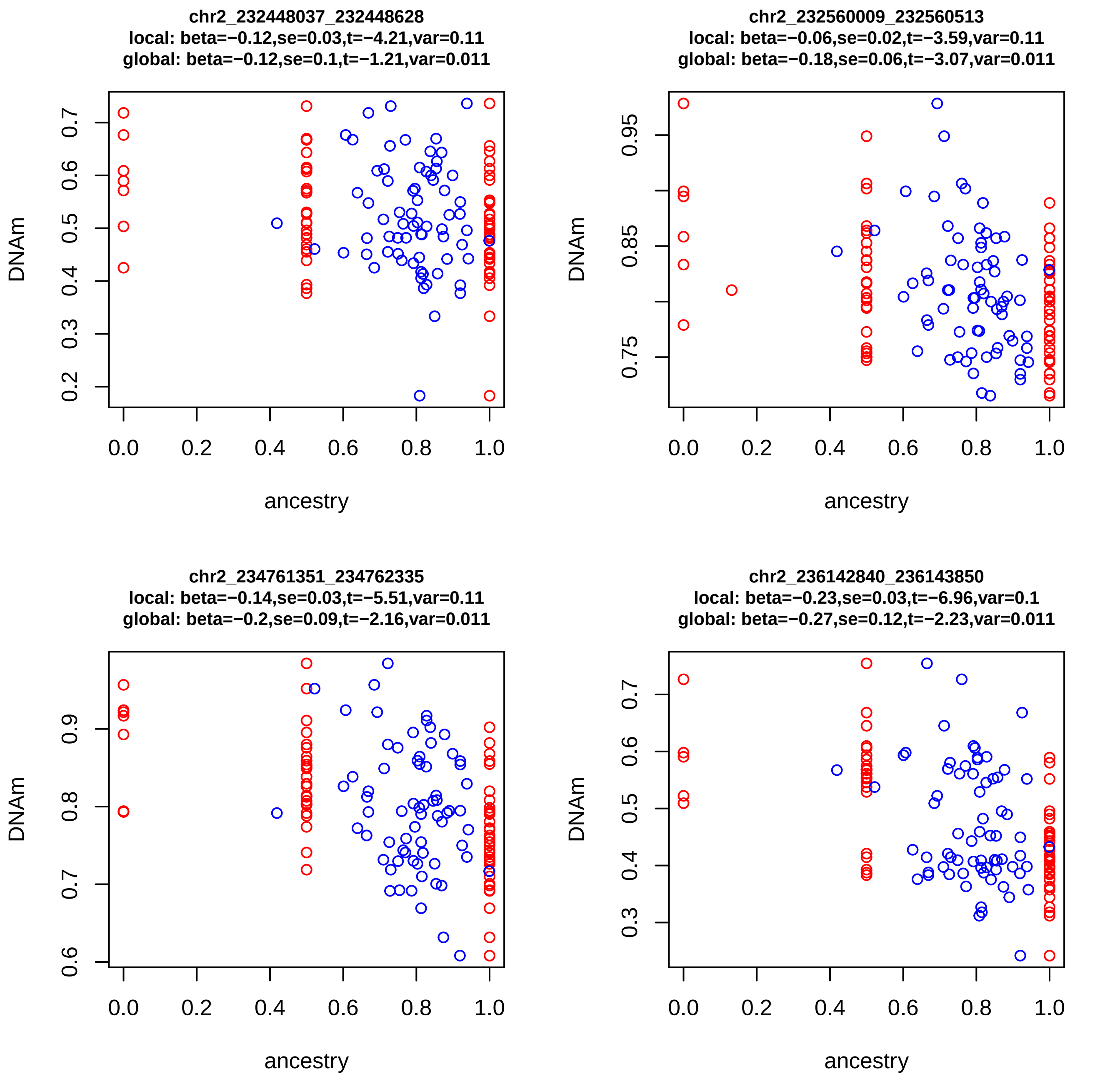
chr2_232448037_232448628
local: beta=−0.12,se=0.03,t=−4.21,var=0.11
global: beta=−0.12,se=0.1,t=−1.21,var=0.011
DNAm
ancestry
chr2_232560009_232560513
local: beta=−0.06,se=0.02,t=−3.59,var=0.11
global: beta=−0.18,se=0.06,t=−3.07,var=0.011
DNAm
ancestry
chr2_234761351_234762335
local: beta=−0.14,se=0.03,t=−5.51,var=0.11
global: beta=−0.2,se=0.09,t=−2.16,var=0.011
DNAm
ancestry
chr2_236142840_236143850
local: beta=−0.23,se=0.03,t=−6.96,var=0.1
global: beta=−0.27,se=0.12,t=−2.23,var=0.011
DNAm
ancestry

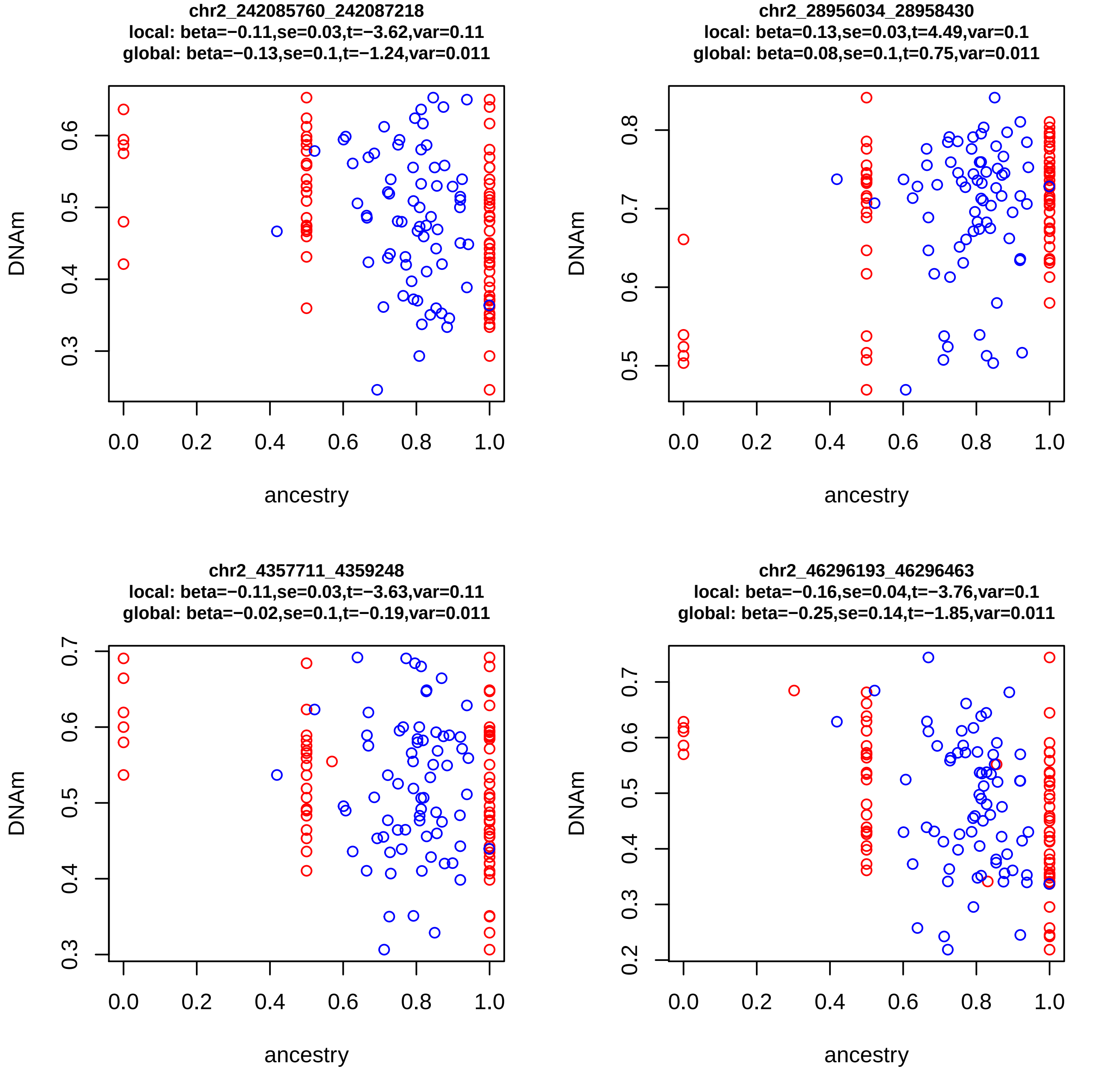
chr2_242085760_242087218
local: beta=−0.11,se=0.03,t=−3.62,var=0.11
global: beta=−0.13,se=0.1,t=−1.24,var=0.011
DNAm
0.3 0.4 0.5 0.6
ancestry
0.0 0.2 0.4 0.6 0.8 1.0
chr2_28956034_28958430
local: beta=0.13,se=0.03,t=4.49,var=0.1
global: beta=0.08,se=0.1,t=0.75,var=0.011
DNAm
0.5 0.6 0.7 0.8
ancestry
0.0 0.2 0.4 0.6 0.8 1.0
chr2_4357711_4359248
local: beta=−0.11,se=0.03,t=−3.63,var=0.11
global: beta=−0.02,se=0.1,t=−0.19,var=0.011
DNAm
0.3 0.4 0.5 0.6 0.7
ancestry
0.0 0.2 0.4 0.6 0.8 1.0
chr2_46296193_46296463
local: beta=−0.16,se=0.04,t=−3.76,var=0.1
global: beta=−0.25,se=0.14,t=−1.85,var=0.011
DNAm
0.2 0.3 0.4 0.5 0.6 0.7
ancestry
0.0 0.2 0.4 0.6 0.8 1.0

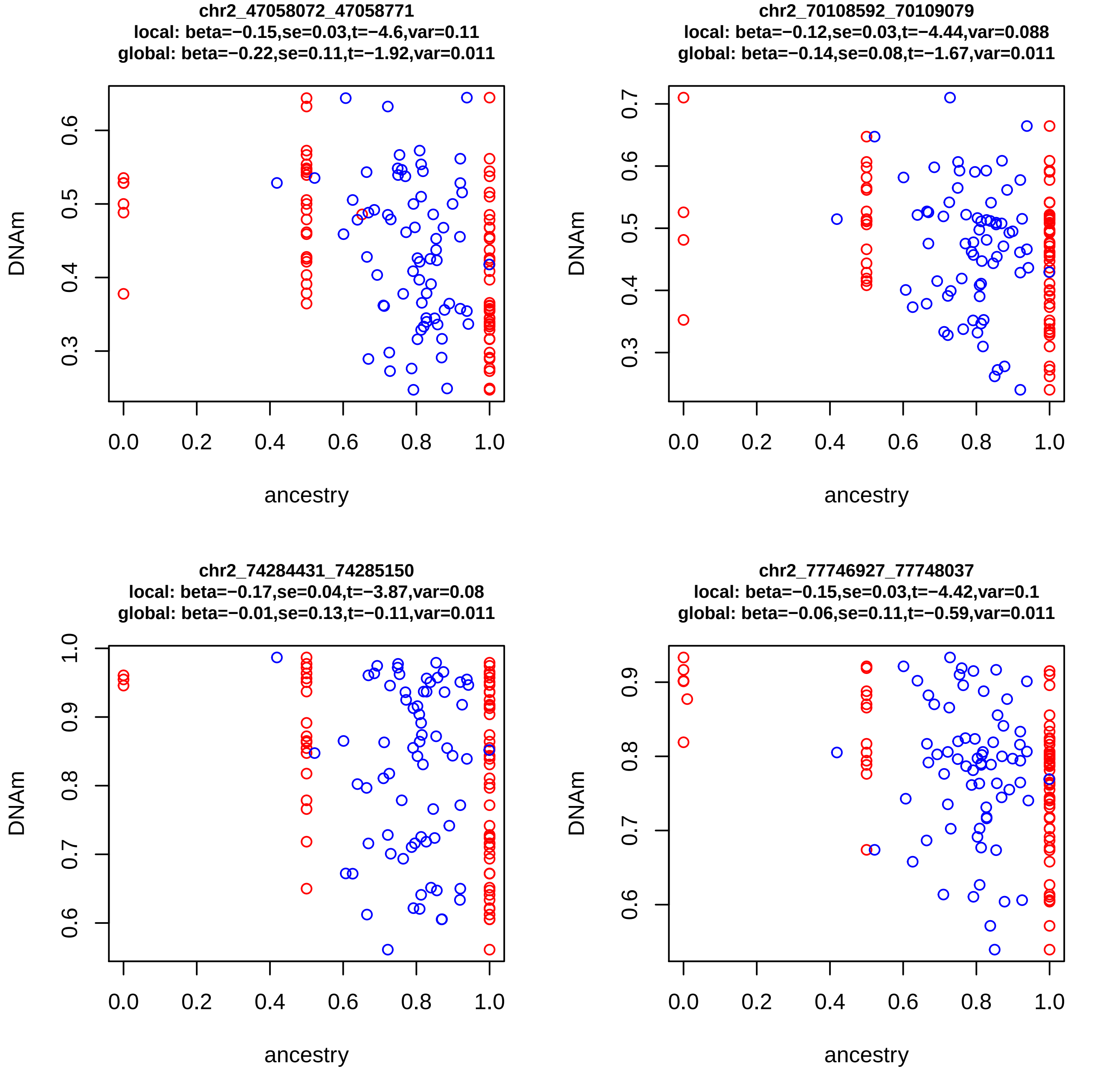
chr2_47058072_47058771
local: beta=−0.15,se=0.03,t=−4.6,var=0.11
global: beta=−0.22,se=0.11,t=−1.92,var=0.011
chr2_70108592_70109079
local: beta=−0.12,se=0.03,t=−4.44,var=0.088
global: beta=−0.14,se=0.08,t=−1.67,var=0.011
chr2_74284431_74285150
local: beta=−0.17,se=0.04,t=−3.87,var=0.08
global: beta=−0.01,se=0.13,t=−0.11,var=0.011
chr2_77746927_77748037
local: beta=−0.15,se=0.03,t=−4.42,var=0.1
global: beta=−0.06,se=0.11,t=−0.59,var=0.011
DNAm
ancestry
0.0
0.2
0.4
0.6
0.8
1.0

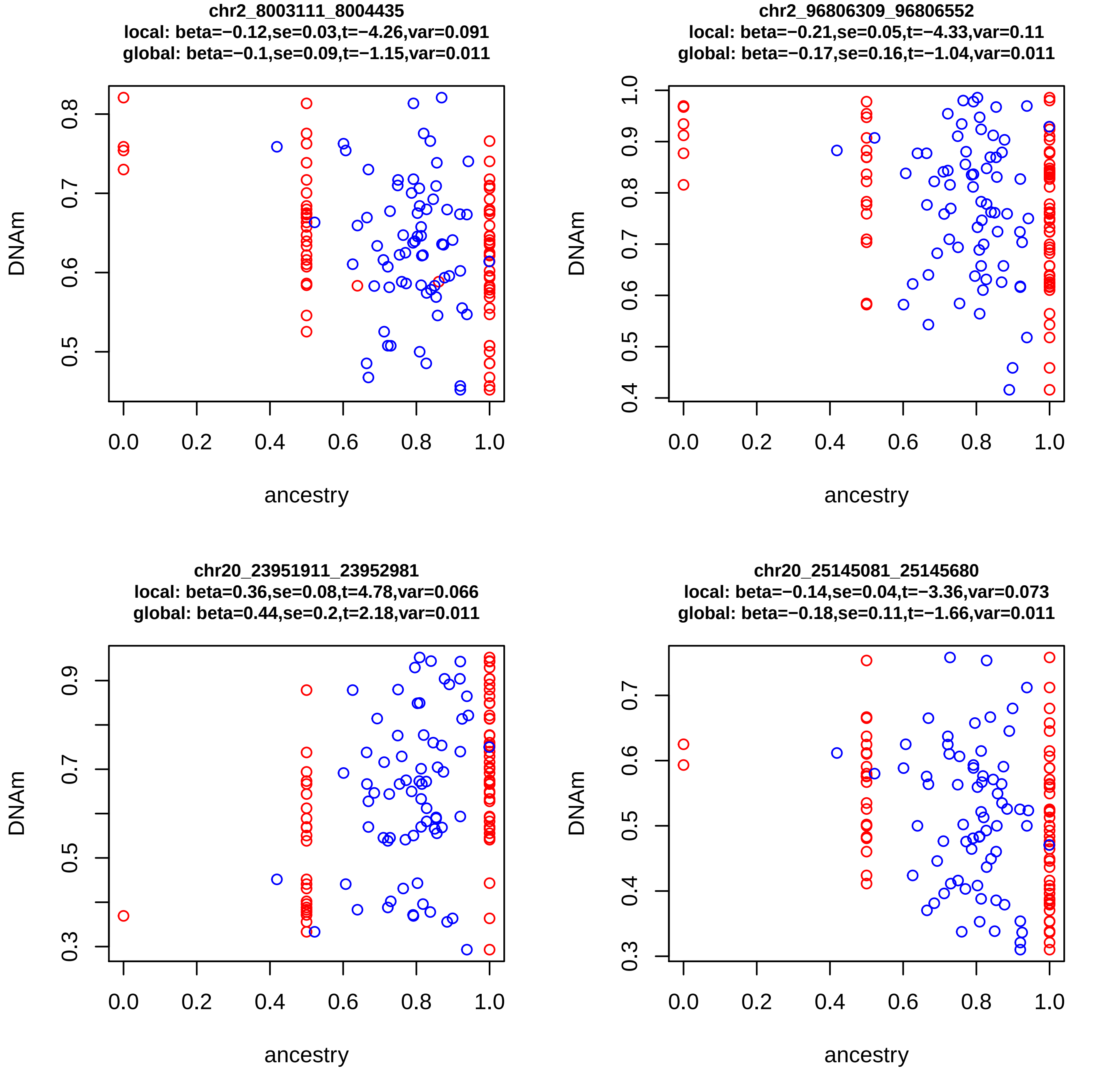
chr2_8003111_8004435
local: beta=−0.12,se=0.03,t=−4.26,var=0.091
global: beta=−0.1,se=0.09,t=−1.15,var=0.011
DNAm
ancestry
chr2_96806309_96806552
local: beta=−0.21,se=0.05,t=−4.33,var=0.11
global: beta=−0.17,se=0.16,t=−1.04,var=0.011
DNAm
ancestry
chr20_23951911_23952981
local: beta=0.36,se=0.08,t=4.78,var=0.066
global: beta=0.44,se=0.2,t=2.18,var=0.011
DNAm
ancestry
chr20_25145081_25145680
local: beta=−0.14,se=0.04,t=−3.36,var=0.073
global: beta=−0.18,se=0.11,t=−1.66,var=0.011
DNAm
ancestry

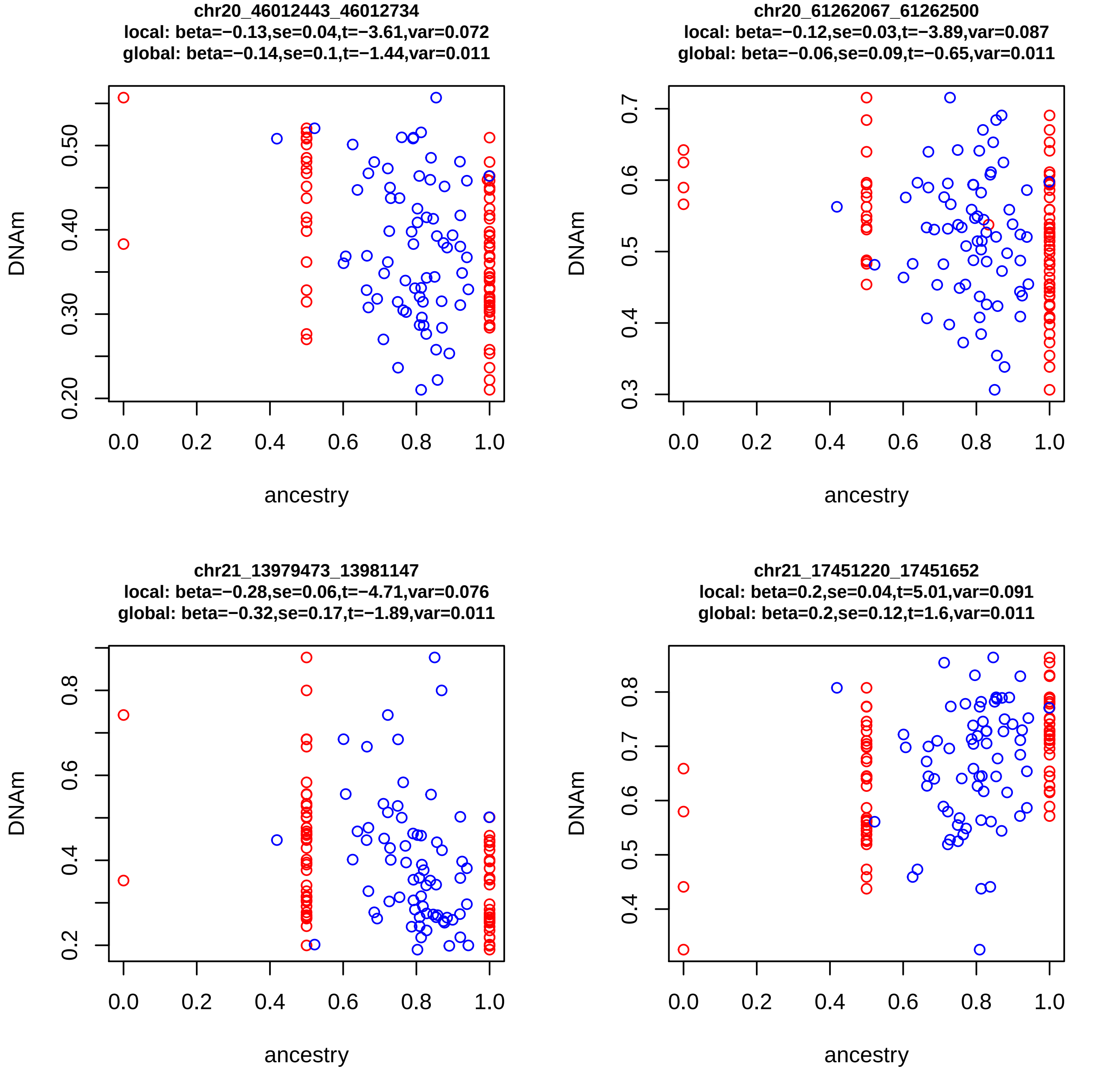
chr20_46012443_46012734
local: beta=−0.13,se=0.04,t=−3.61,var=0.072
global: beta=−0.14,se=0.1,t=−1.44,var=0.011
DNAm
0.20
0.30
0.40
0.50
ancestry
0.0
0.2
0.4
0.6
0.8
1.0
chr20_61262067_61262500
local: beta=−0.12,se=0.03,t=−3.89,var=0.087
global: beta=−0.06,se=0.09,t=−0.65,var=0.011
DNAm
0.3
0.4
0.5
0.6
0.7
ancestry
0.0
0.2
0.4
0.6
0.8
1.0
chr21_13979473_13981147
local: beta=−0.28,se=0.06,t=−4.71,var=0.076
global: beta=−0.32,se=0.17,t=−1.89,var=0.011
DNAm
0.2
0.4
0.6
0.8
ancestry
0.0
0.2
0.4
0.6
0.8
1.0
chr21_17451220_17451652
local: beta=0.2,se=0.04,t=5.01,var=0.091
global: beta=0.2,se=0.12,t=1.6,var=0.011
DNAm
0.4
0.5
0.6
0.7
0.8
ancestry
0.0
0.2
0.4
0.6
0.8
1.0

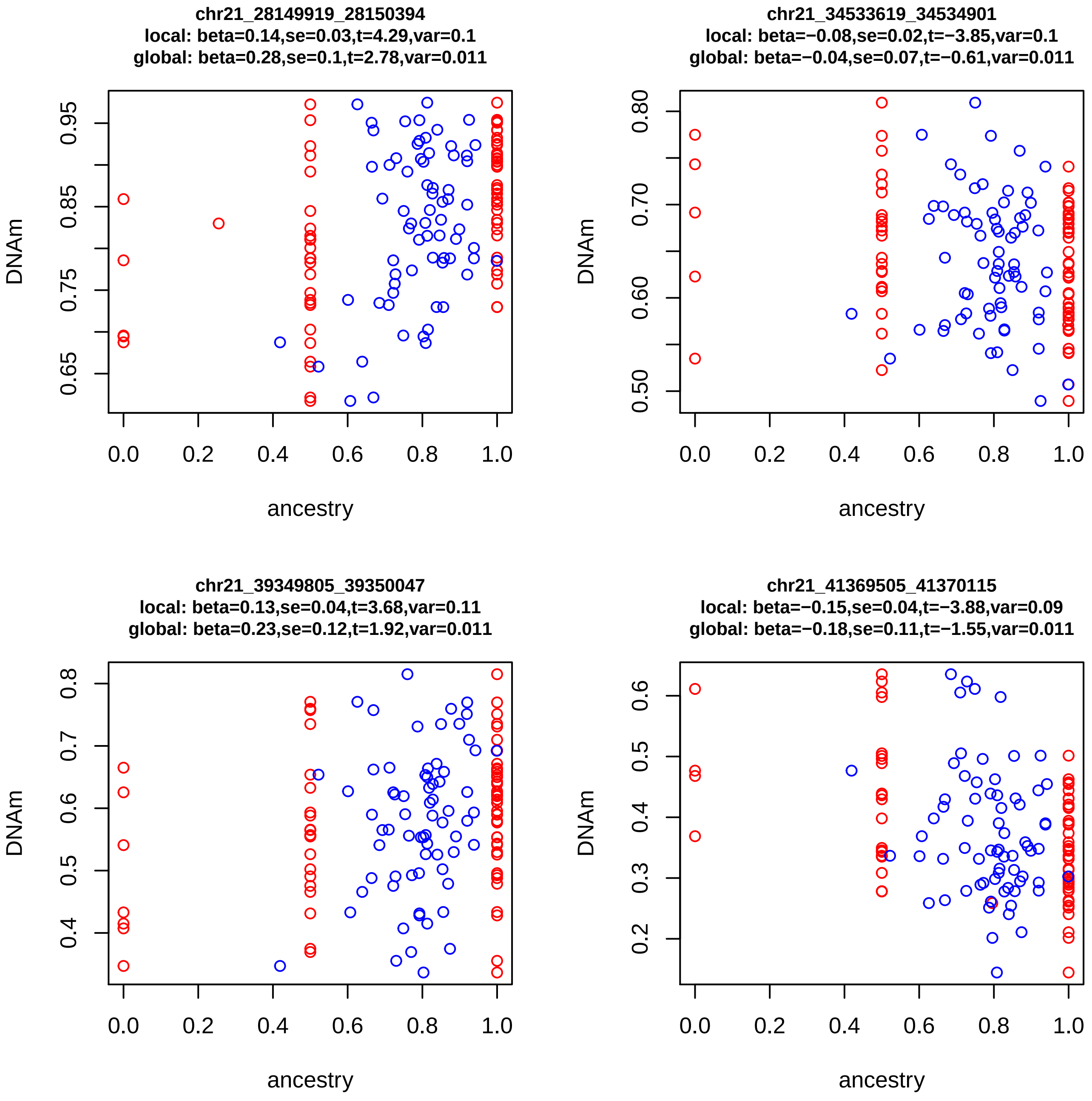
chr21_28149919_28150394
local: beta=0.14,se=0.03,t=4.29,var=0.1
global: beta=0.28,se=0.1,t=2.78,var=0.011
DNAm
0.65
0.75
0.85
0.95
0.0
0.2
0.4
0.6
0.8
1.0
ancestry
chr21_34533619_34534901
local: beta=−0.08,se=0.02,t=−3.85,var=0.1
global: beta=−0.04,se=0.07,t=−0.61,var=0.011
DNAm
0.50
0.60
0.70
0.80
0.0
0.2
0.4
0.6
0.8
1.0
ancestry
chr21_39349805_39350047
local: beta=0.13,se=0.04,t=3.68,var=0.11
global: beta=0.23,se=0.12,t=1.92,var=0.011
DNAm
0.4
0.5
0.6
0.7
0.8
0.0
0.2
0.4
0.6
0.8
1.0
ancestry
chr21_41369505_41370115
local: beta=−0.15,se=0.04,t=−3.88,var=0.09
global: beta=−0.18,se=0.11,t=−1.55,var=0.011
DNAm
0.2
0.3
0.4
0.5
0.6
0.0
0.2
0.4
0.6
0.8
1.0
ancestry

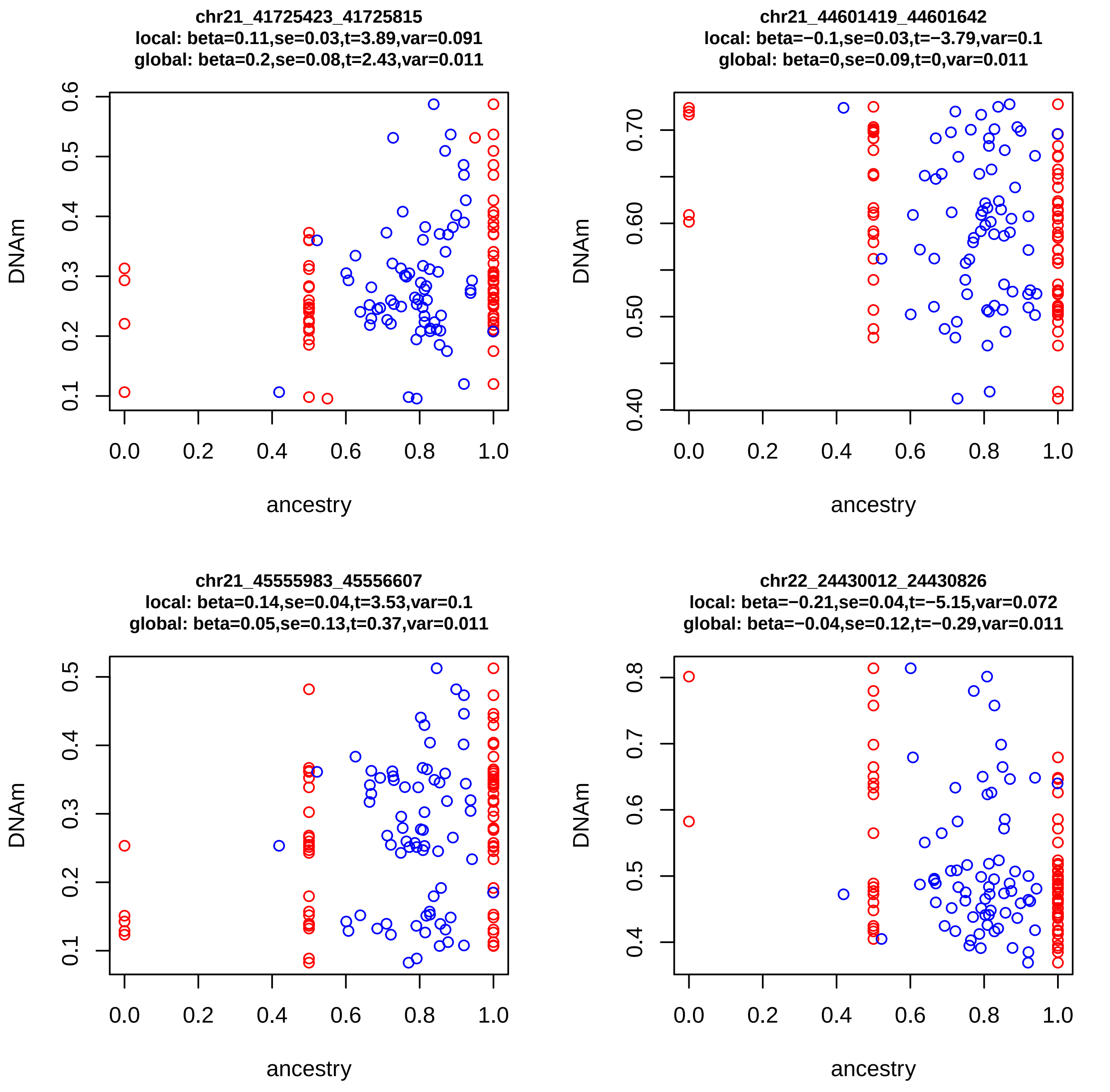
chr21_41725423_41725815
local: beta=0.11,se=0.03,t=3.89,var=0.091
global: beta=0.2,se=0.08,t=2.43,var=0.011
DNAm
ancestry
chr21_44601419_44601642
local: beta=−0.1,se=0.03,t=−3.79,var=0.1
global: beta=0,se=0.09,t=0,var=0.011
DNAm
ancestry
chr21_45555983_45556607
local: beta=0.14,se=0.04,t=3.53,var=0.1
global: beta=0.05,se=0.13,t=0.37,var=0.011
DNAm
ancestry
chr22_24430012_24430826
local: beta=−0.21,se=0.04,t=−5.15,var=0.072
global: beta=−0.04,se=0.12,t=−0.29,var=0.011
DNAm
ancestry

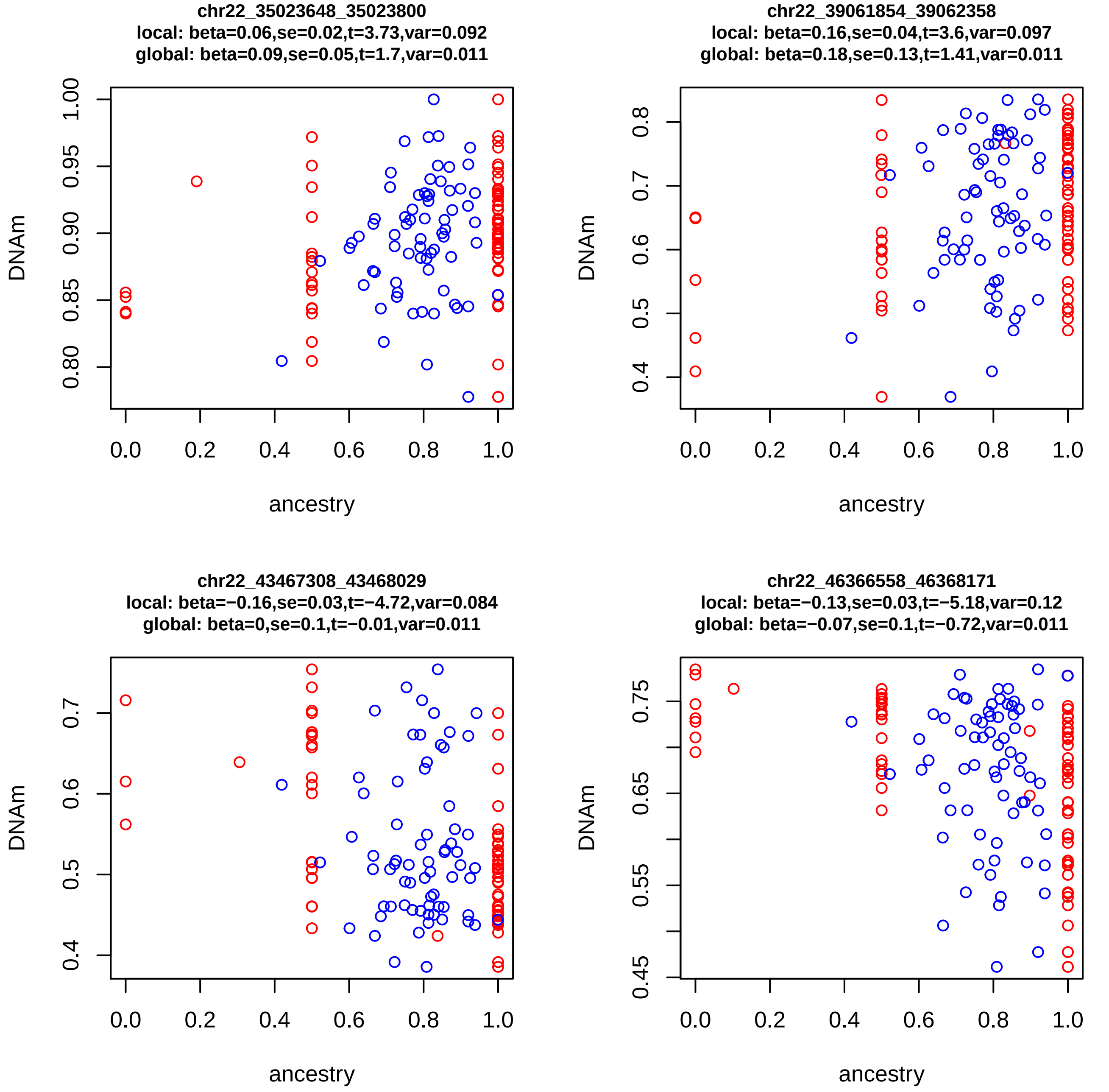
chr22_35023648_35023800
local: beta=0.06,se=0.02,t=3.73,var=0.092
global: beta=0.09,se=0.05,t=1.7,var=0.011
DNAm
0.80 0.85 0.90 0.95 1.00
ancestry
0.0 0.2 0.4 0.6 0.8 1.0
chr22_39061854_39062358
local: beta=0.16,se=0.04,t=3.6,var=0.097
global: beta=0.18,se=0.13,t=1.41,var=0.011
DNAm
0.4 0.5 0.6 0.7 0.8
ancestry
0.0 0.2 0.4 0.6 0.8 1.0
chr22_43467308_43468029
local: beta=−0.16,se=0.03,t=−4.72,var=0.084
global: beta=0,se=0.1,t=−0.01,var=0.011
DNAm
0.4 0.5 0.6 0.7
ancestry
0.0 0.2 0.4 0.6 0.8 1.0
chr22_46366558_46368171
local: beta=−0.13,se=0.03,t=−5.18,var=0.12
global: beta=−0.07,se=0.1,t=−0.72,var=0.011
DNAm
0.45 0.55 0.65 0.75
ancestry
0.0 0.2 0.4 0.6 0.8 1.0

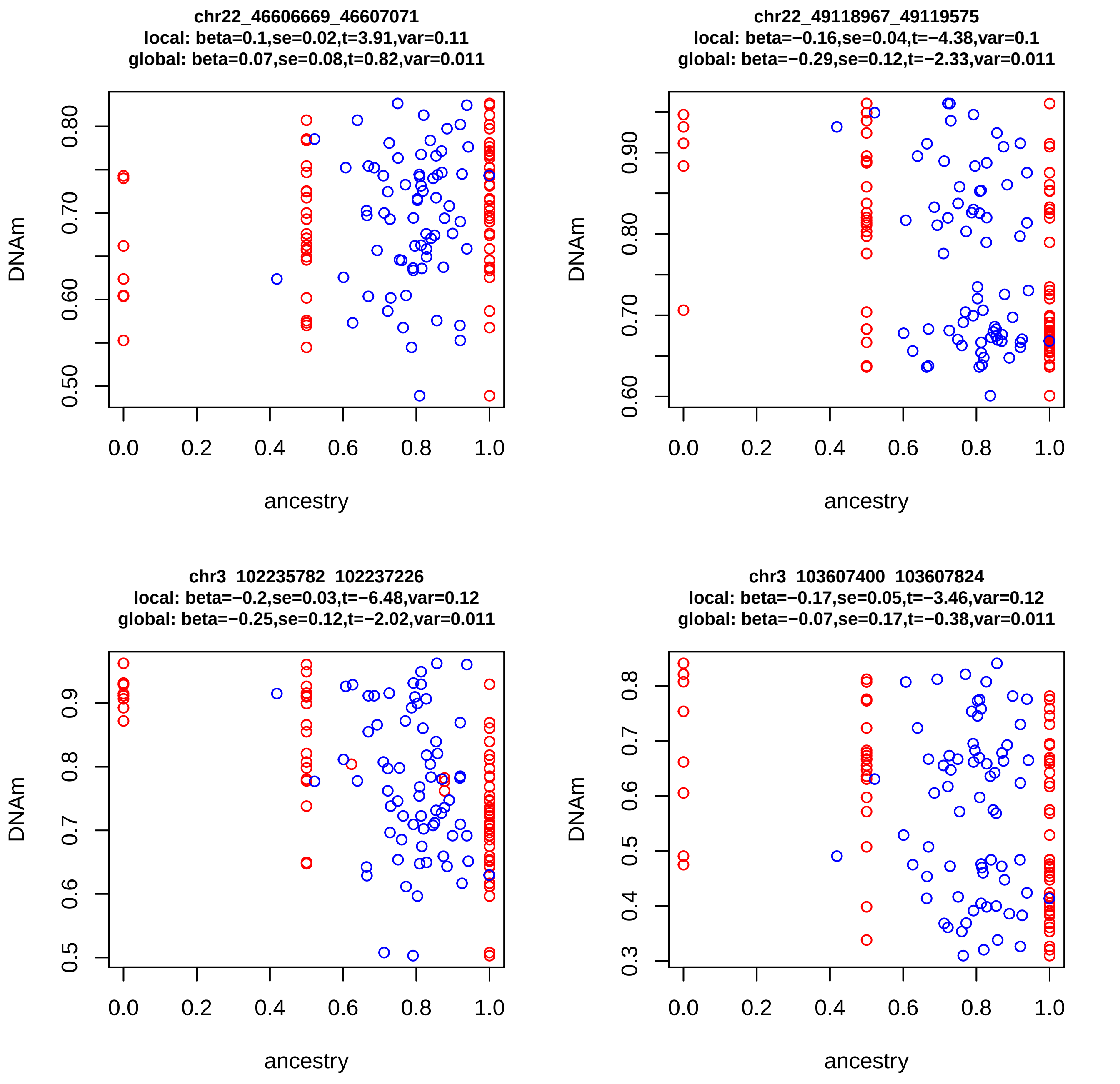
chr22_46606669_46607071
local: beta=0.1,se=0.02,t=3.91,var=0.11
global: beta=0.07,se=0.08,t=0.82,var=0.011
chr22_49118967_49119575
local: beta=−0.16,se=0.04,t=−4.38,var=0.1
global: beta=−0.29,se=0.12,t=−2.33,var=0.011
chr3_102235782_102237226
local: beta=−0.2,se=0.03,t=−6.48,var=0.12
global: beta=−0.25,se=0.12,t=−2.02,var=0.011
chr3_103607400_103607824
local: beta=−0.17,se=0.05,t=−3.46,var=0.12
global: beta=−0.07,se=0.17,t=−0.38,var=0.011
DNAm
ancestry

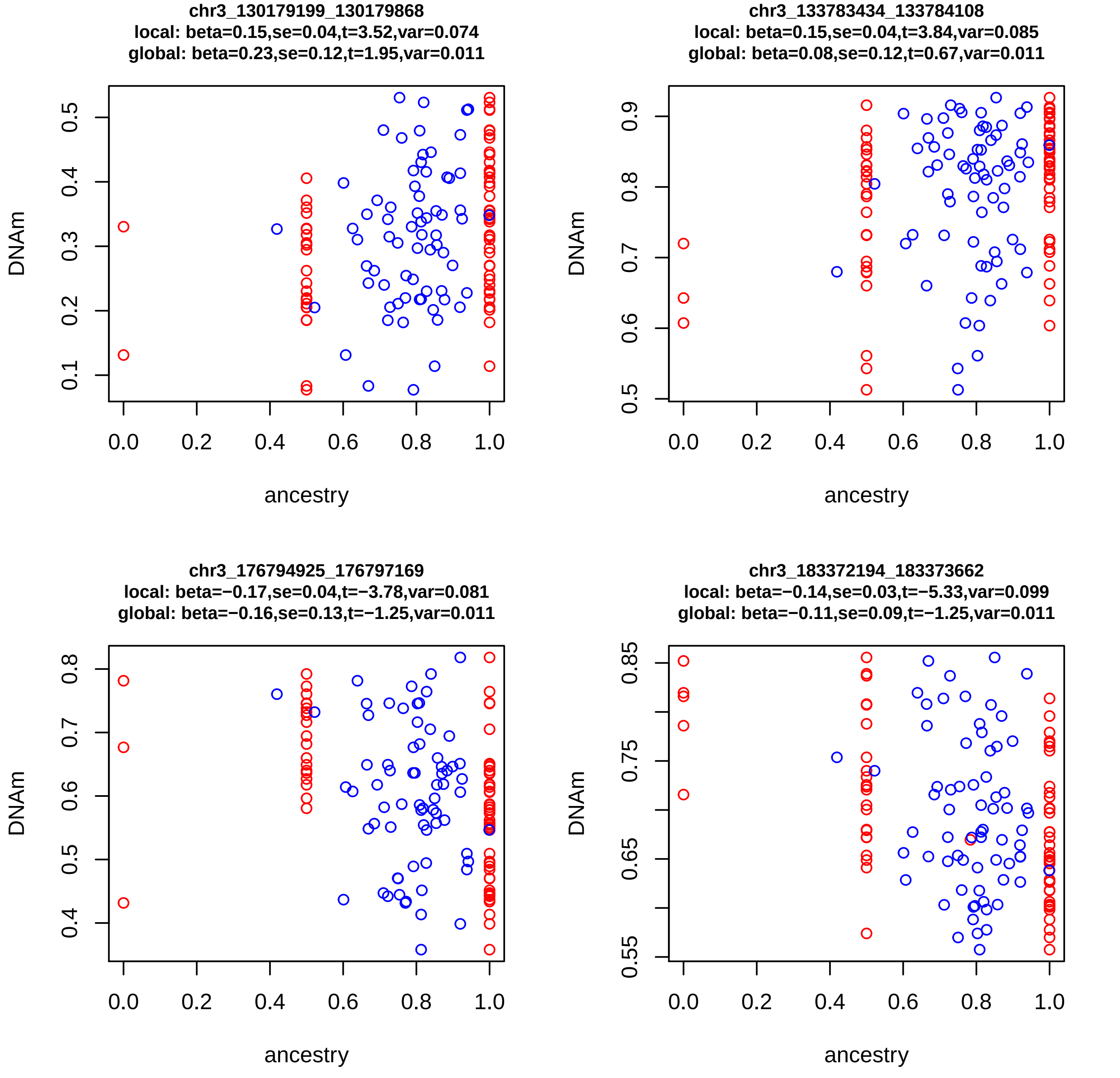
chr3_130179199_130179868
local: beta=0.15,se=0.04,t=3.52,var=0.074
global: beta=0.23,se=0.12,t=1.95,var=0.011
DNAm
ancestry
chr3_133783434_133784108
local: beta=0.15,se=0.04,t=3.84,var=0.085
global: beta=0.08,se=0.12,t=0.67,var=0.011
DNAm
ancestry
chr3_176794925_176797169
local: beta=−0.17,se=0.04,t=−3.78,var=0.081
global: beta=−0.16,se=0.13,t=−1.25,var=0.011
DNAm
ancestry
chr3_183372194_183373662
local: beta=−0.14,se=0.03,t=−5.33,var=0.099
global: beta=−0.11,se=0.09,t=−1.25,var=0.011
DNAm
ancestry

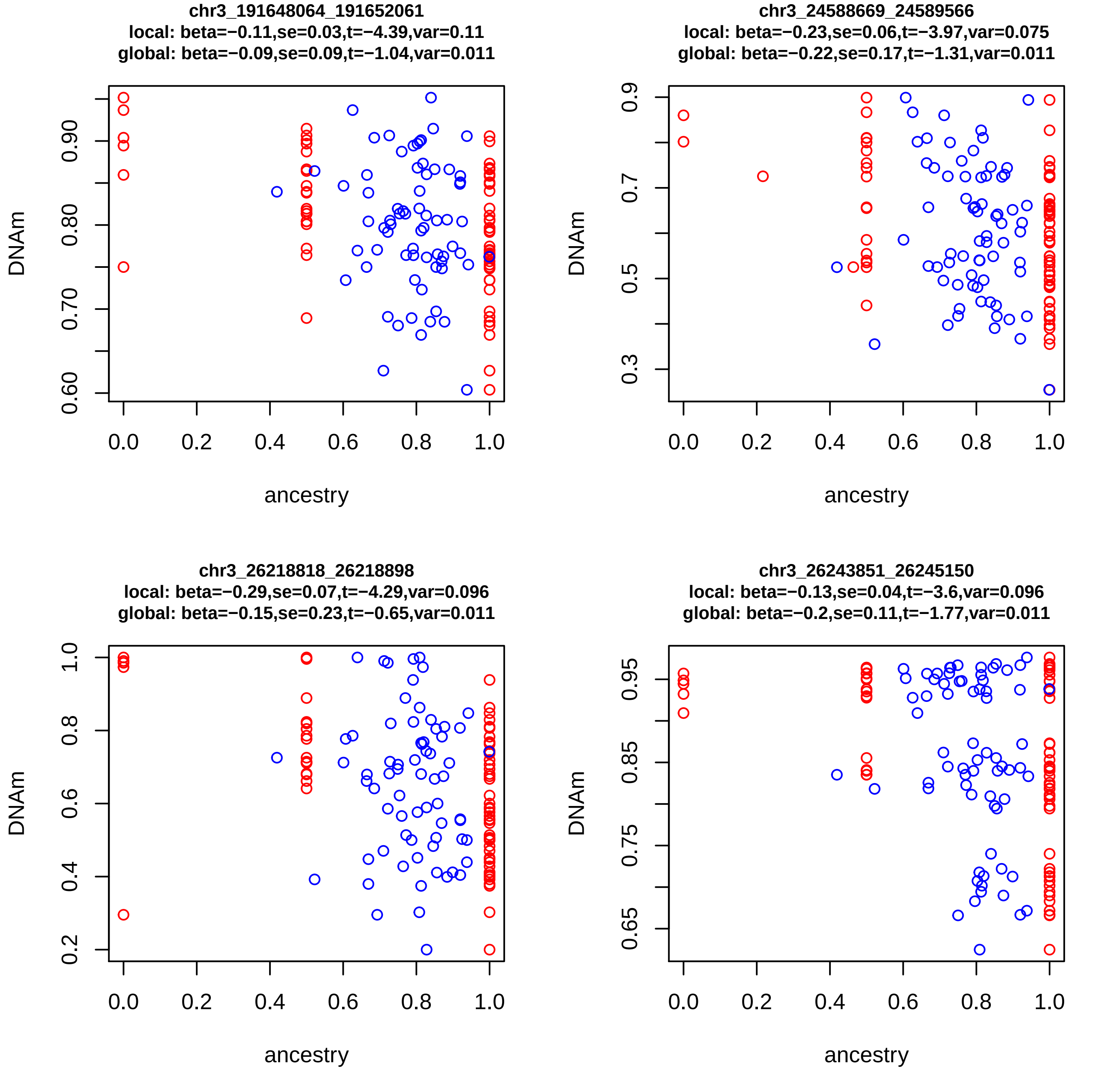
chr3_191648064_191652061
local: beta=−0.11,se=0.03,t=−4.39,var=0.11
global: beta=−0.09,se=0.09,t=−1.04,var=0.011
DNAm
ancestry
0.60
0.70
0.80
0.90
0.0
0.2
0.4
0.6
0.8
1.0
chr3_24588669_24589566
local: beta=−0.23,se=0.06,t=−3.97,var=0.075
global: beta=−0.22,se=0.17,t=−1.31,var=0.011
DNAm
ancestry
0.3
0.5
0.7
0.9
chr3_26218818_26218898
local: beta=−0.29,se=0.07,t=−4.29,var=0.096
global: beta=−0.15,se=0.23,t=−0.65,var=0.011
DNAm
ancestry
0.2
0.4
0.6
0.8
1.0
chr3_26243851_26245150
local: beta=−0.13,se=0.04,t=−3.6,var=0.096
global: beta=−0.2,se=0.11,t=−1.77,var=0.011
DNAm
ancestry
0.65
0.75
0.85
0.95

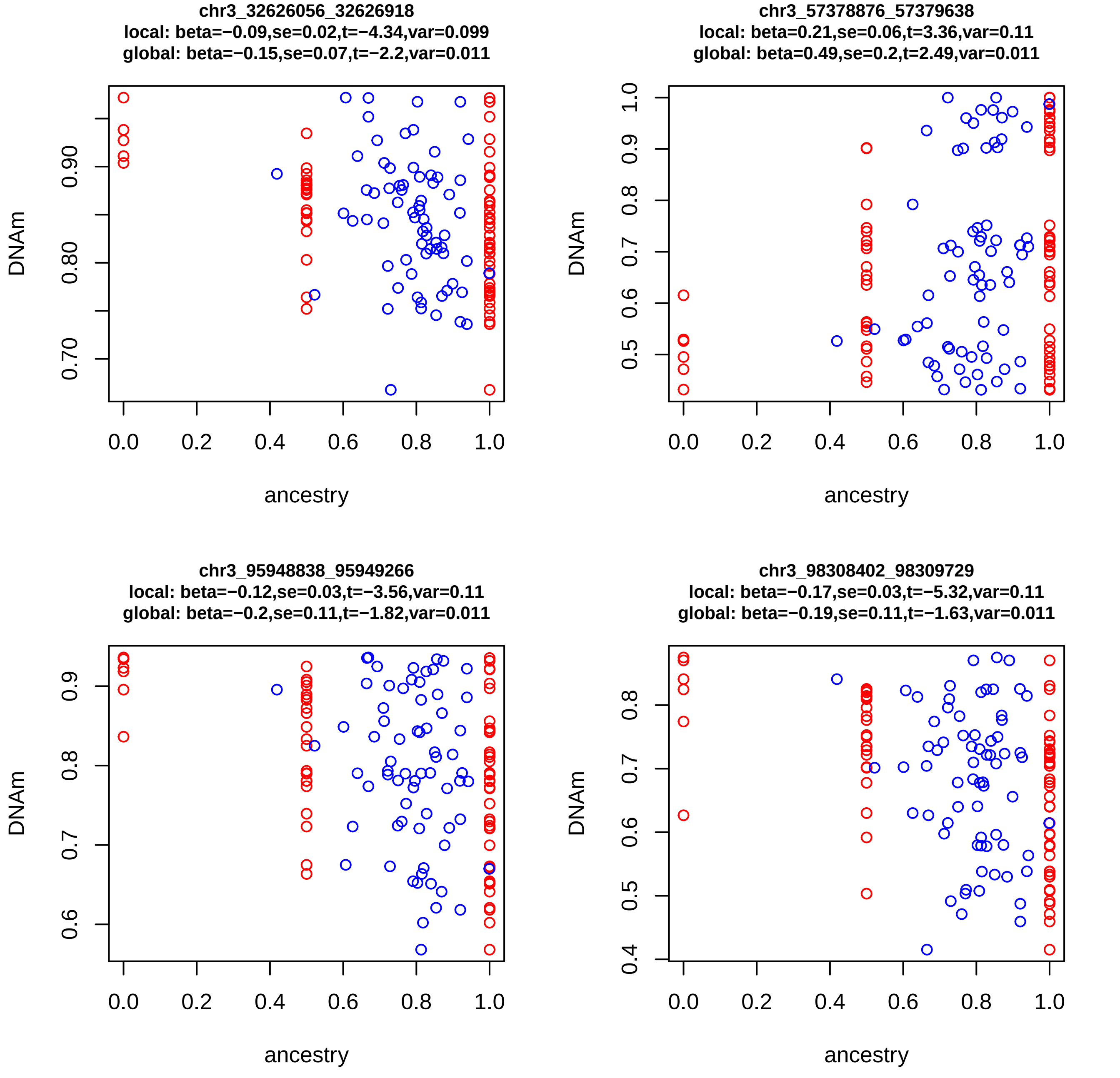
chr3_32626056_32626918
local: beta=−0.09,se=0.02,t=−4.34,var=0.099
global: beta=−0.15,se=0.07,t=−2.2,var=0.011
DNAm
ancestry
chr3_57378876_57379638
local: beta=0.21,se=0.06,t=3.36,var=0.11
global: beta=0.49,se=0.2,t=2.49,var=0.011
DNAm
ancestry
chr3_95948838_95949266
local: beta=−0.12,se=0.03,t=−3.56,var=0.11
global: beta=−0.2,se=0.11,t=−1.82,var=0.011
DNAm
ancestry
chr3_98308402_98309729
local: beta=−0.17,se=0.03,t=−5.32,var=0.11
global: beta=−0.19,se=0.11,t=−1.63,var=0.011
DNAm
ancestry

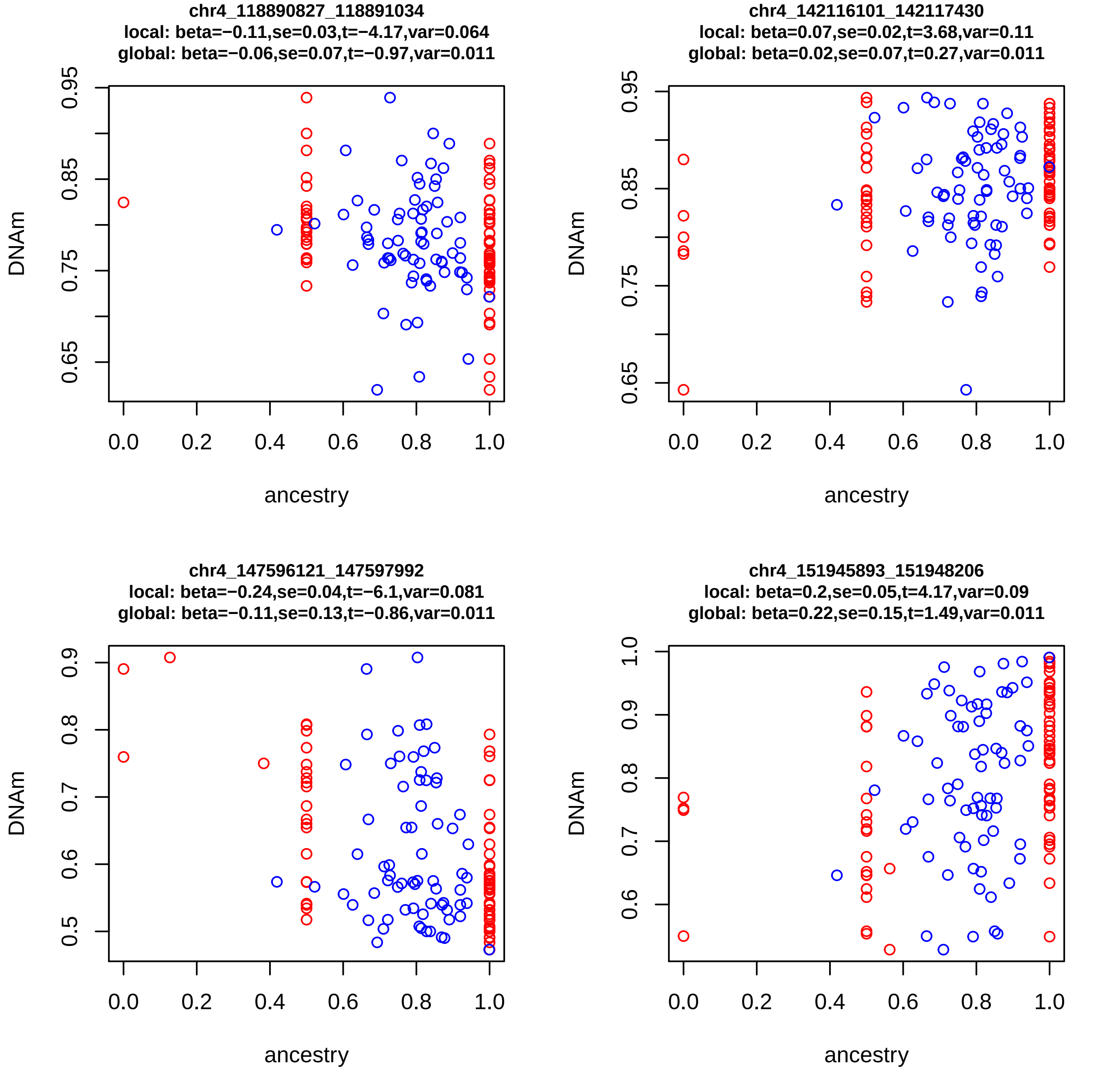
chr4_118890827_118891034
local: beta=−0.11,se=0.03,t=−4.17,var=0.064
global: beta=−0.06,se=0.07,t=−0.97,var=0.011
DNAm
ancestry
chr4_142116101_142117430
local: beta=0.07,se=0.02,t=3.68,var=0.11
global: beta=0.02,se=0.07,t=0.27,var=0.011
DNAm
ancestry
chr4_147596121_147597992
local: beta=−0.24,se=0.04,t=−6.1,var=0.081
global: beta=−0.11,se=0.13,t=−0.86,var=0.011
DNAm
ancestry
chr4_151945893_151948206
local: beta=0.2,se=0.05,t=4.17,var=0.09
global: beta=0.22,se=0.15,t=1.49,var=0.011
DNAm
ancestry

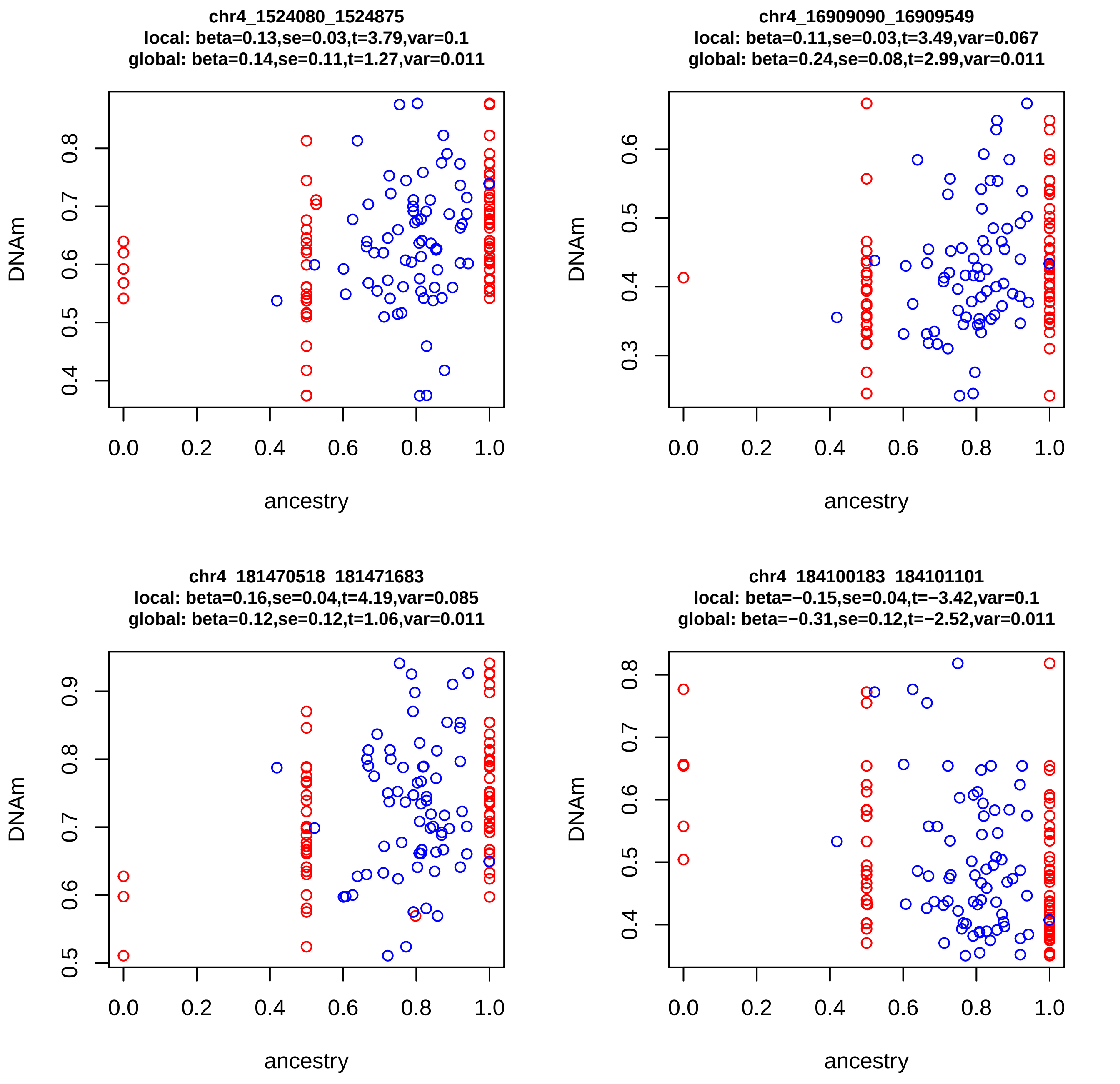
chr4_1524080_1524875
local: beta=0.13,se=0.03,t=3.79,var=0.1
global: beta=0.14,se=0.11,t=1.27,var=0.011
DNAm
0.4
0.5
0.6
0.7
0.8
0.0
0.2
0.4
0.6
0.8
1.0
ancestry
chr4_16909090_16909549
local: beta=0.11,se=0.03,t=3.49,var=0.067
global: beta=0.24,se=0.08,t=2.99,var=0.011
DNAm
0.3
0.4
0.5
0.6
0.0
0.2
0.4
0.6
0.8
1.0
ancestry
chr4_181470518_181471683
local: beta=0.16,se=0.04,t=4.19,var=0.085
global: beta=0.12,se=0.12,t=1.06,var=0.011
DNAm
0.5
0.6
0.7
0.8
0.9
0.0
0.2
0.4
0.6
0.8
1.0
ancestry
chr4_184100183_184101101
local: beta=−0.15,se=0.04,t=−3.42,var=0.1
global: beta=−0.31,se=0.12,t=−2.52,var=0.011
DNAm
0.4
0.5
0.6
0.7
0.8
0.0
0.2
0.4
0.6
0.8
1.0
ancestry

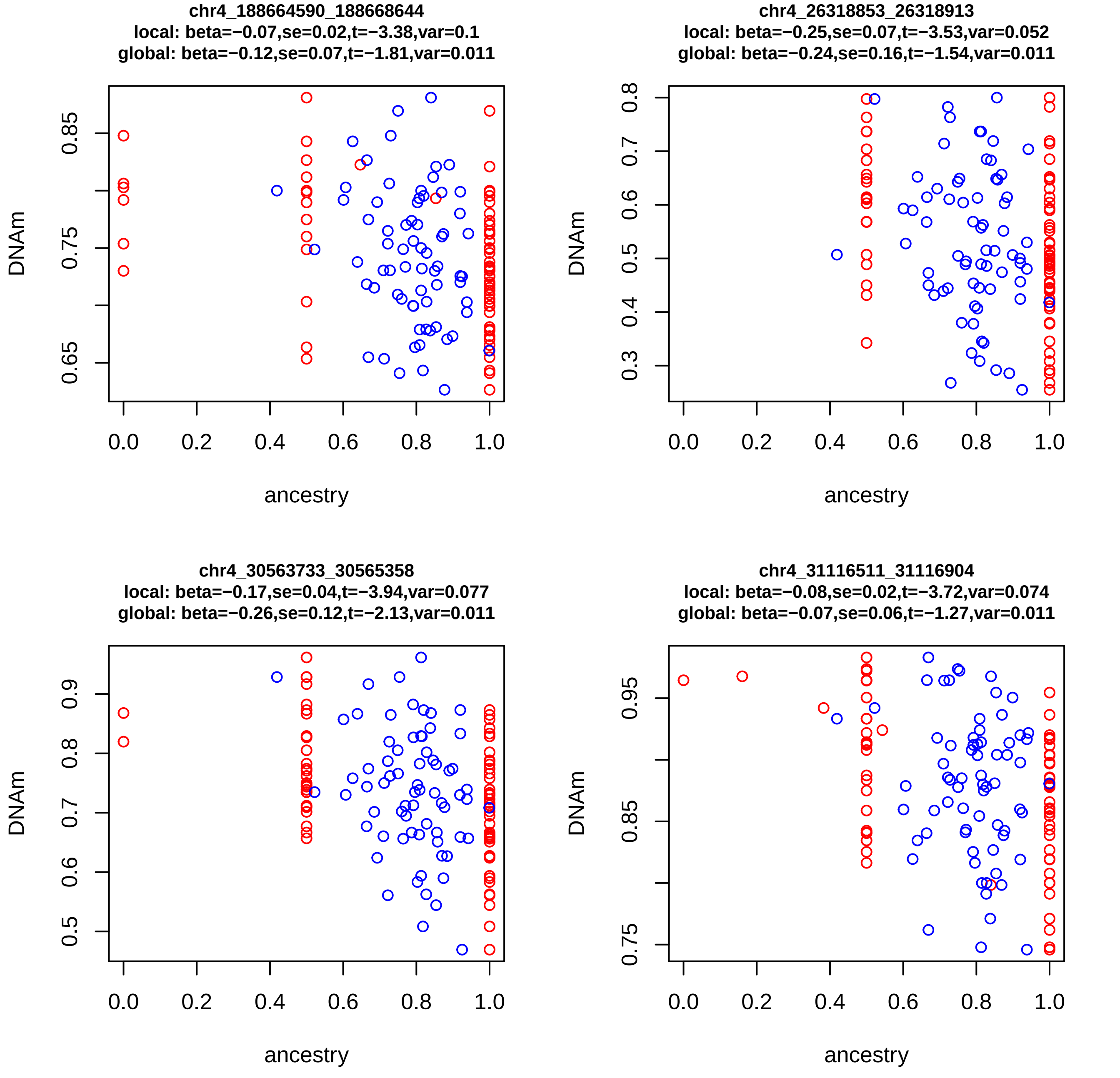
chr4_188664590_188668644
local: beta=−0.07,se=0.02,t=−3.38,var=0.1
global: beta=−0.12,se=0.07,t=−1.81,var=0.011
DNAm
0.65
0.75
0.85
ancestry
0.0
0.2
0.4
0.6
0.8
1.0
chr4_26318853_26318913
local: beta=−0.25,se=0.07,t=−3.53,var=0.052
global: beta=−0.24,se=0.16,t=−1.54,var=0.011
DNAm
0.3
0.4
0.5
0.6
0.7
0.8
ancestry
0.0
0.2
0.4
0.6
0.8
1.0
chr4_30563733_30565358
local: beta=−0.17,se=0.04,t=−3.94,var=0.077
global: beta=−0.26,se=0.12,t=−2.13,var=0.011
DNAm
0.5
0.6
0.7
0.8
0.9
ancestry
0.0
0.2
0.4
0.6
0.8
1.0
chr4_31116511_31116904
local: beta=−0.08,se=0.02,t=−3.72,var=0.074
global: beta=−0.07,se=0.06,t=−1.27,var=0.011
DNAm
0.75
0.85
0.95
ancestry
0.0
0.2
0.4
0.6
0.8
1.0

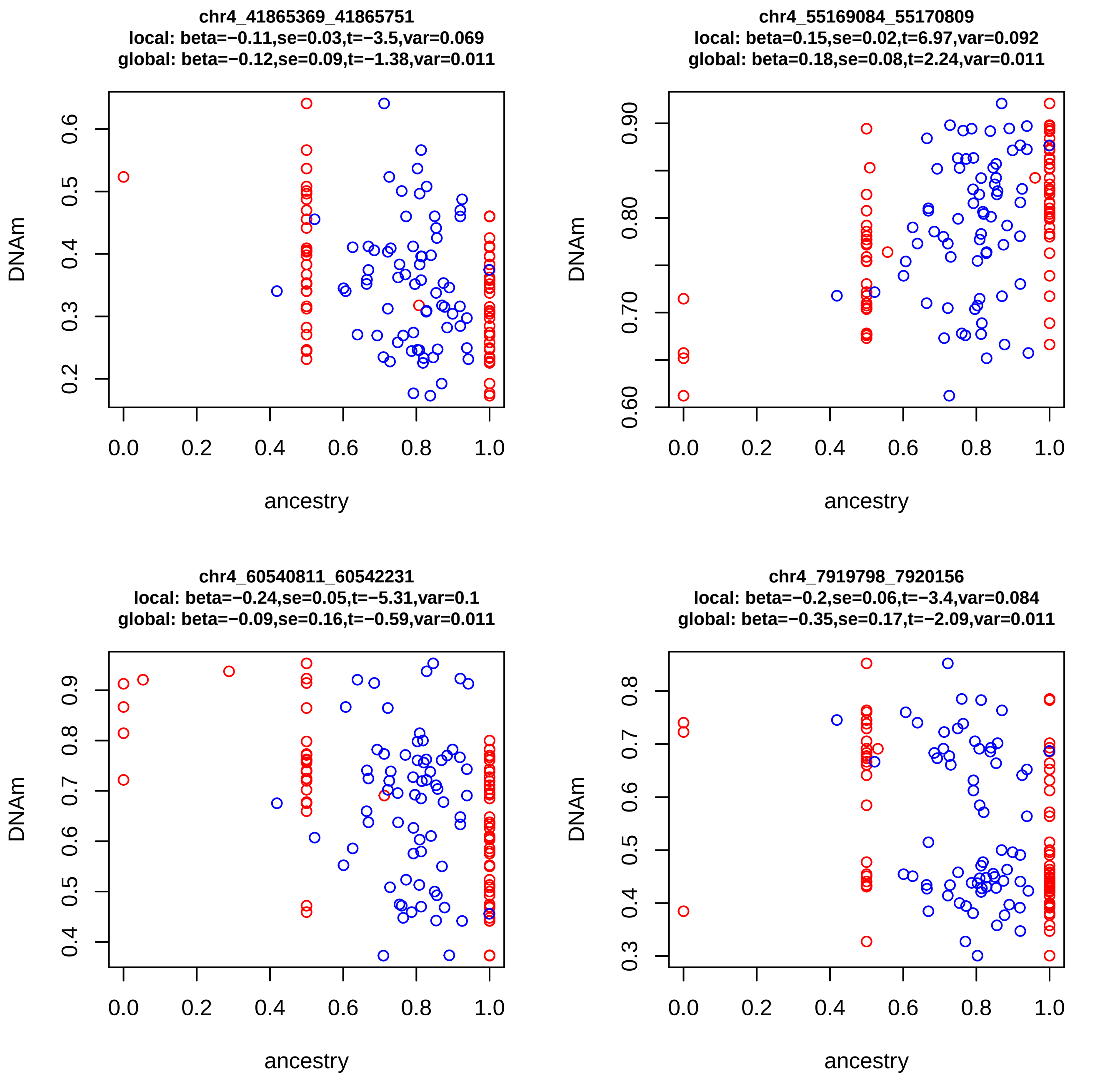
chr4_41865369_41865751
local: beta=−0.11,se=0.03,t=−3.5,var=0.069
global: beta=−0.12,se=0.09,t=−1.38,var=0.011
DNAm
ancestry
chr4_55169084_55170809
local: beta=0.15,se=0.02,t=6.97,var=0.092
global: beta=0.18,se=0.08,t=2.24,var=0.011
DNAm
ancestry
chr4_60540811_60542231
local: beta=−0.24,se=0.05,t=−5.31,var=0.1
global: beta=−0.09,se=0.16,t=−0.59,var=0.011
DNAm
ancestry
chr4_7919798_7920156
local: beta=−0.2,se=0.06,t=−3.4,var=0.084
global: beta=−0.35,se=0.17,t=−2.09,var=0.011
DNAm
ancestry

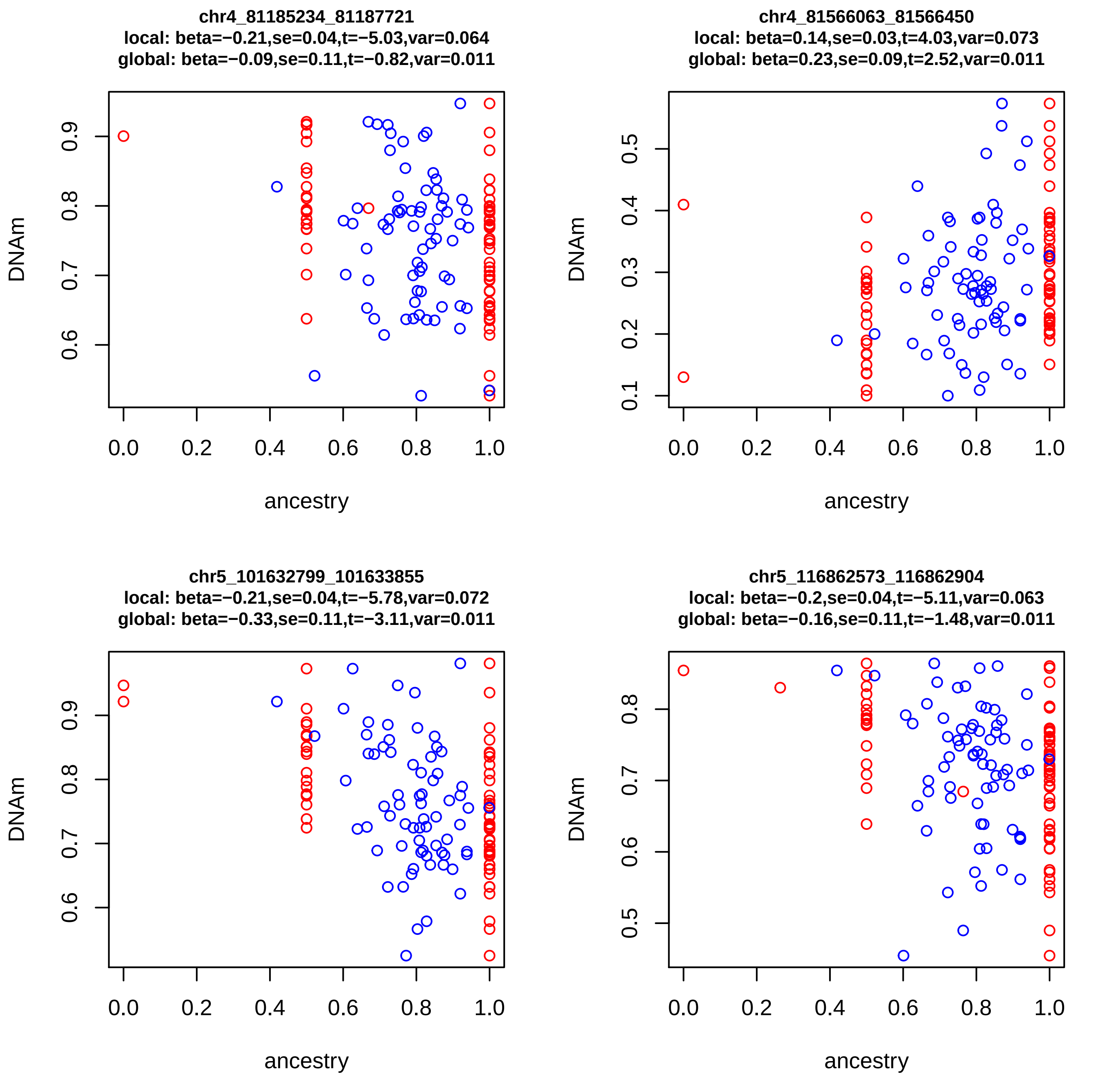
chr4_81185234_81187721
local: beta=−0.21,se=0.04,t=−5.03,var=0.064
global: beta=−0.09,se=0.11,t=−0.82,var=0.011
chr4_81566063_81566450
local: beta=0.14,se=0.03,t=4.03,var=0.073
global: beta=0.23,se=0.09,t=2.52,var=0.011
chr5_101632799_101633855
local: beta=−0.21,se=0.04,t=−5.78,var=0.072
global: beta=−0.33,se=0.11,t=−3.11,var=0.011
chr5_116862573_116862904
local: beta=−0.2,se=0.04,t=−5.11,var=0.063
global: beta=−0.16,se=0.11,t=−1.48,var=0.011
DNAm
ancestry

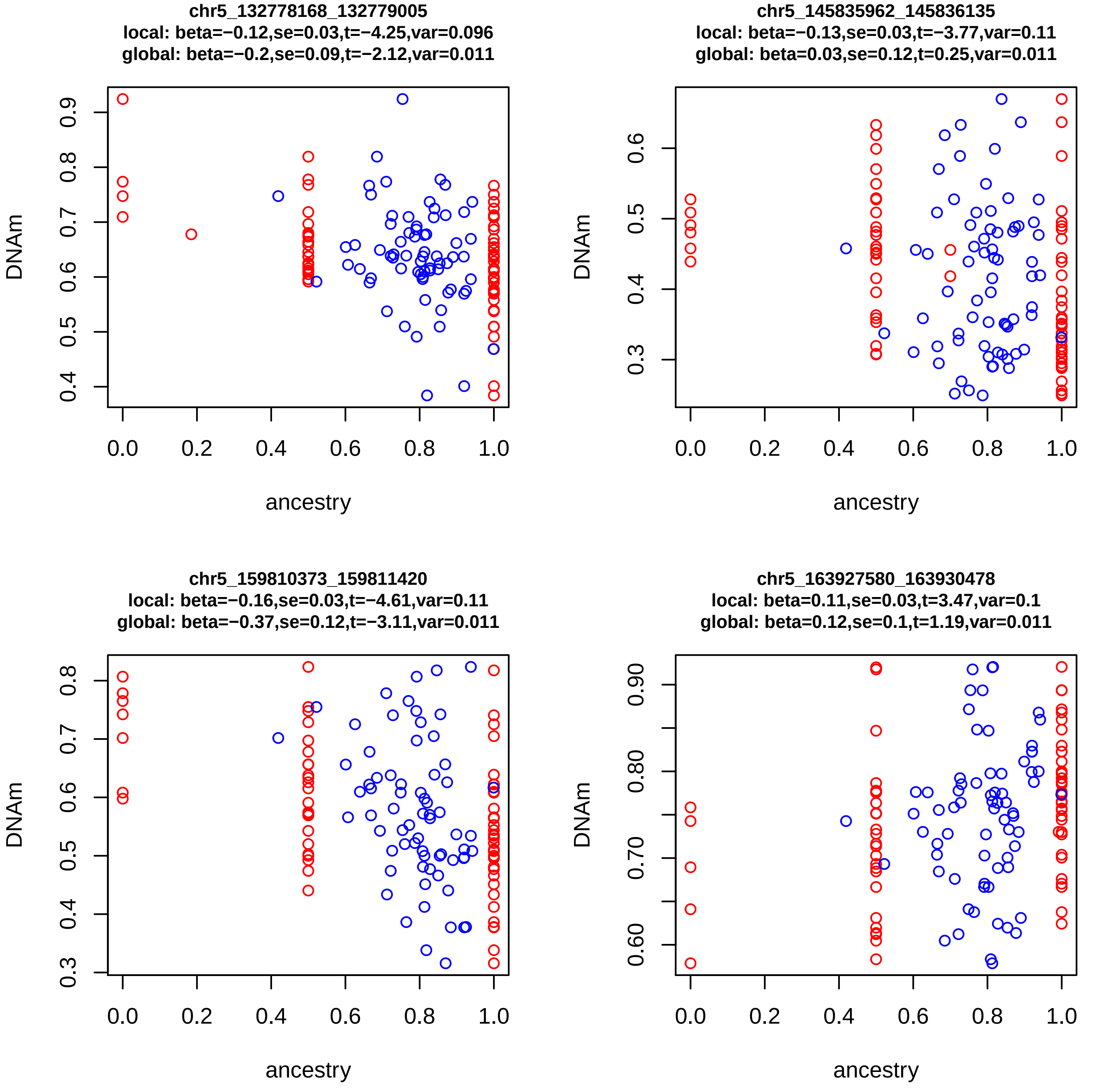
chr5_132778168_132779005
local: beta=−0.12,se=0.03,t=−4.25,var=0.096
global: beta=−0.2,se=0.09,t=−2.12,var=0.011
DNAm
ancestry
chr5_145835962_145836135
local: beta=−0.13,se=0.03,t=−3.77,var=0.11
global: beta=0.03,se=0.12,t=0.25,var=0.011
DNAm
ancestry
chr5_159810373_159811420
local: beta=−0.16,se=0.03,t=−4.61,var=0.11
global: beta=−0.37,se=0.12,t=−3.11,var=0.011
DNAm
ancestry
chr5_163927580_163930478
local: beta=0.11,se=0.03,t=3.47,var=0.1
global: beta=0.12,se=0.1,t=1.19,var=0.011
DNAm
ancestry

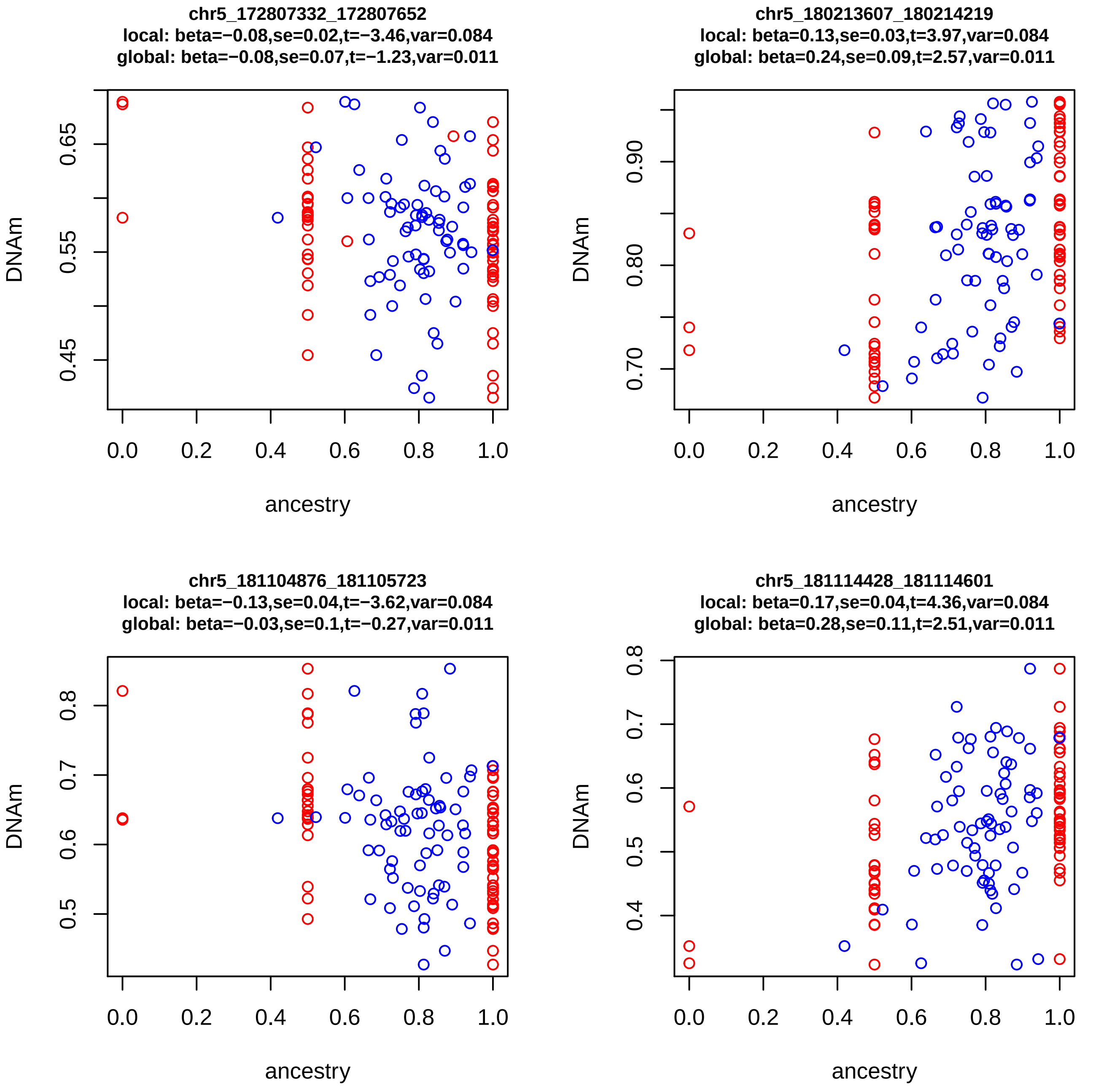
chr5_172807332_172807652
local: beta=−0.08,se=0.02,t=−3.46,var=0.084
global: beta=−0.08,se=0.07,t=−1.23,var=0.011
DNAm
0.45
0.55
0.65
0.0
0.2
0.4
0.6
0.8
1.0
ancestry
chr5_180213607_180214219
local: beta=0.13,se=0.03,t=3.97,var=0.084
global: beta=0.24,se=0.09,t=2.57,var=0.011
DNAm
0.70
0.80
0.90
0.0
0.2
0.4
0.6
0.8
1.0
ancestry
chr5_181104876_181105723
local: beta=−0.13,se=0.04,t=−3.62,var=0.084
global: beta=−0.03,se=0.1,t=−0.27,var=0.011
DNAm
0.5
0.6
0.7
0.8
0.0
0.2
0.4
0.6
0.8
1.0
ancestry
chr5_181114428_181114601
local: beta=0.17,se=0.04,t=4.36,var=0.084
global: beta=0.28,se=0.11,t=2.51,var=0.011
DNAm
0.4
0.5
0.6
0.7
0.8
0.0
0.2
0.4
0.6
0.8
1.0
ancestry

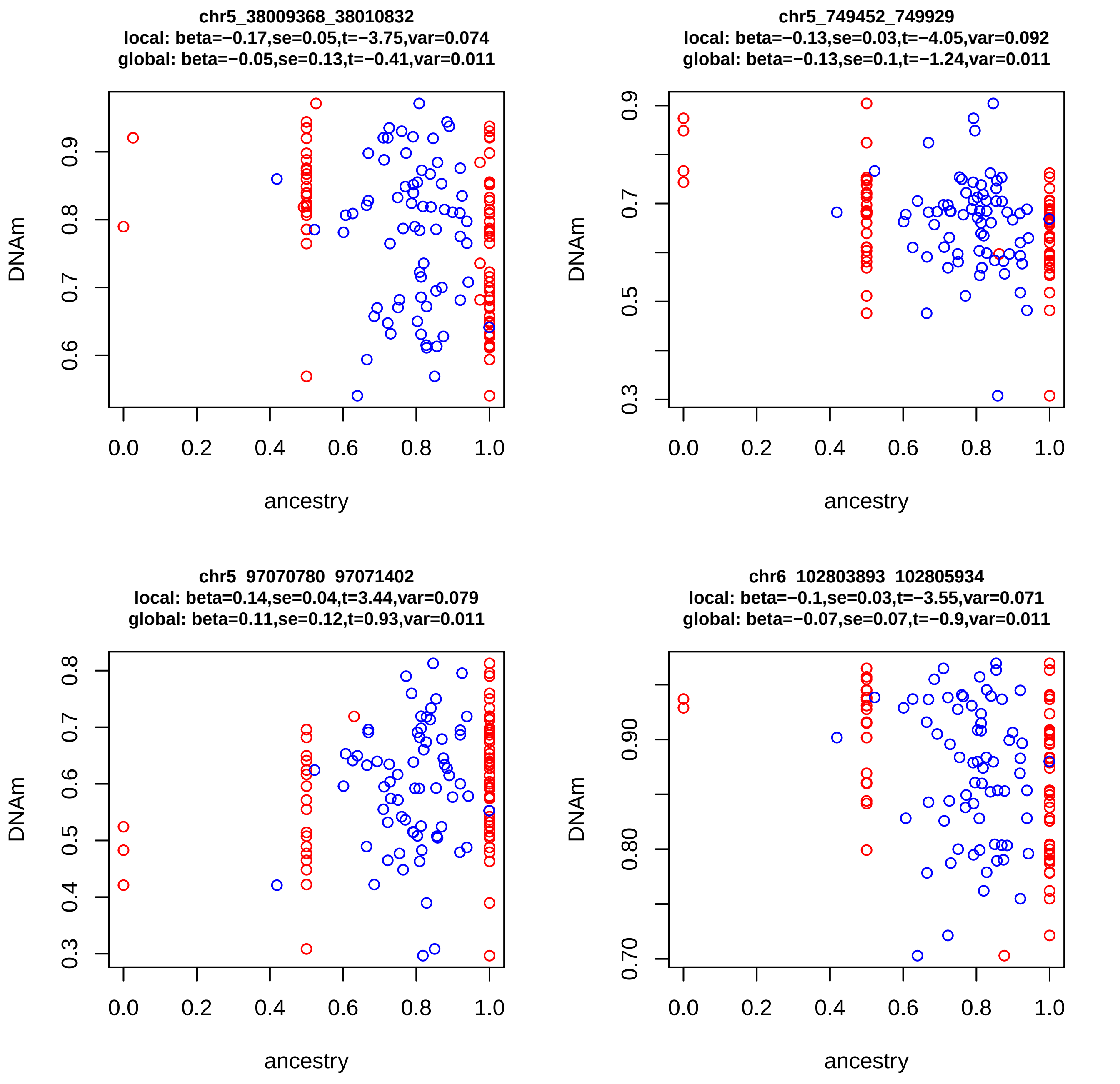
chr5_38009368_38010832
local: beta=−0.17,se=0.05,t=−3.75,var=0.074
global: beta=−0.05,se=0.13,t=−0.41,var=0.011
DNAm
0.6
0.7
0.8
0.9
0.0
0.2
0.4
0.6
0.8
1.0
ancestry
chr5_749452_749929
local: beta=−0.13,se=0.03,t=−4.05,var=0.092
global: beta=−0.13,se=0.1,t=−1.24,var=0.011
DNAm
0.3
0.5
0.7
0.9
0.0
0.2
0.4
0.6
0.8
1.0
ancestry
chr5_97070780_97071402
local: beta=0.14,se=0.04,t=3.44,var=0.079
global: beta=0.11,se=0.12,t=0.93,var=0.011
DNAm
0.3
0.4
0.5
0.6
0.7
0.8
0.0
0.2
0.4
0.6
0.8
1.0
ancestry
chr6_102803893_102805934
local: beta=−0.1,se=0.03,t=−3.55,var=0.071
global: beta=−0.07,se=0.07,t=−0.9,var=0.011
DNAm
0.70
0.80
0.90
0.0
0.2
0.4
0.6
0.8
1.0
ancestry

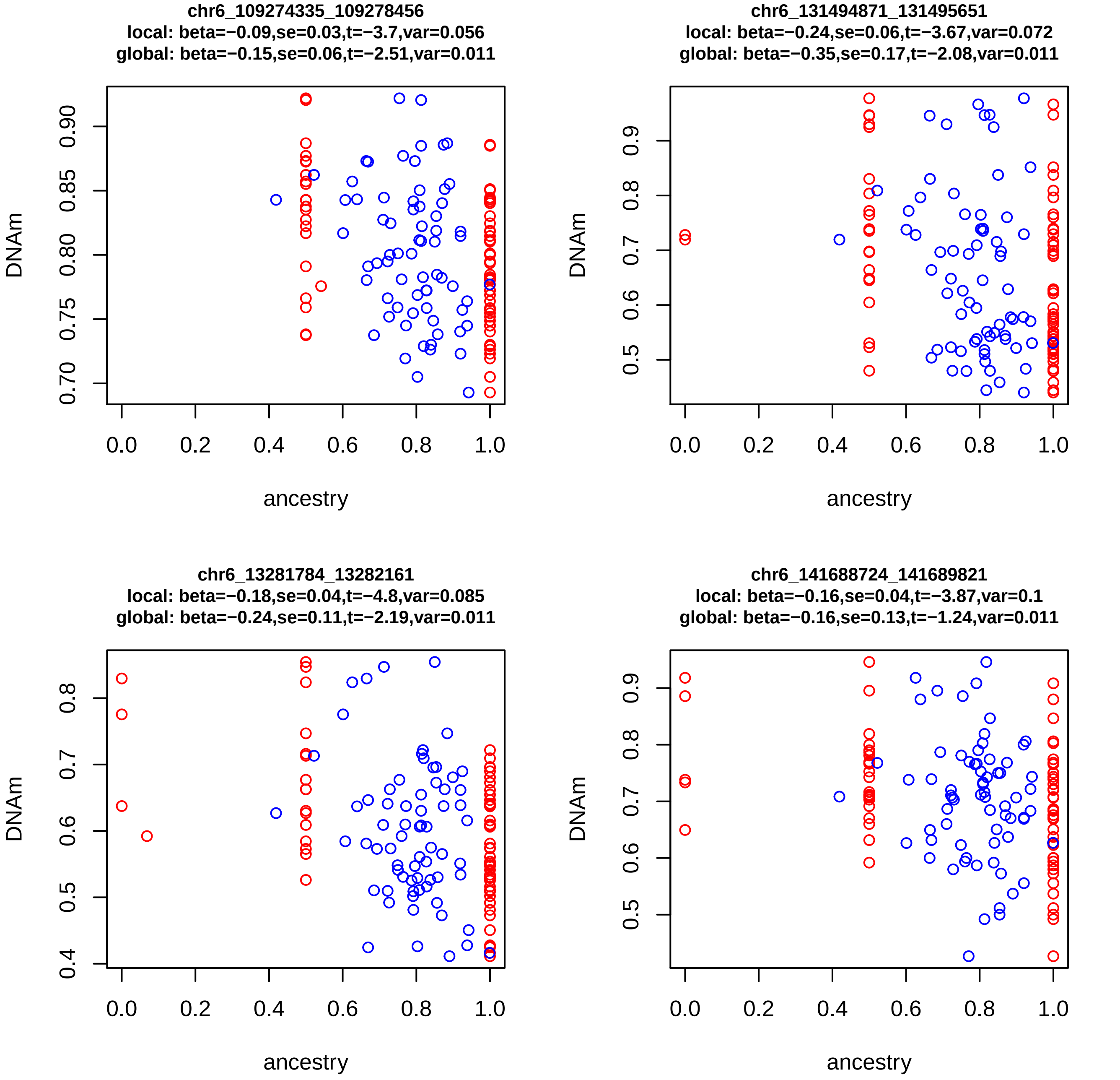
chr6_109274335_109278456
local: beta=−0.09,se=0.03,t=−3.7,var=0.056
global: beta=−0.15,se=0.06,t=−2.51,var=0.011
chr6_131494871_131495651
local: beta=−0.24,se=0.06,t=−3.67,var=0.072
global: beta=−0.35,se=0.17,t=−2.08,var=0.011
chr6_13281784_13282161
local: beta=−0.18,se=0.04,t=−4.8,var=0.085
global: beta=−0.24,se=0.11,t=−2.19,var=0.011
chr6_141688724_141689821
local: beta=−0.16,se=0.04,t=−3.87,var=0.1
global: beta=−0.16,se=0.13,t=−1.24,var=0.011
DNAm
ancestry

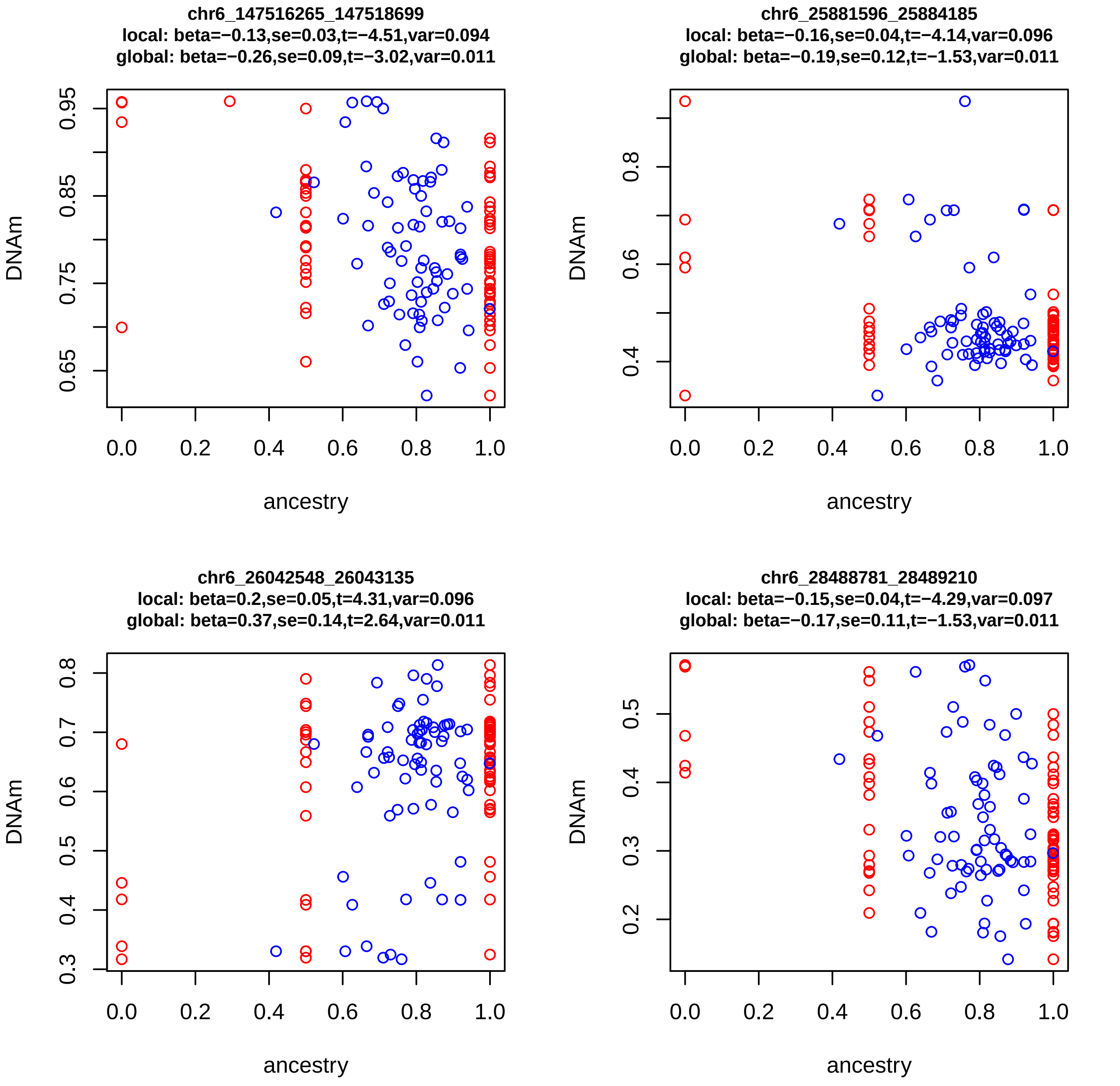
chr6_147516265_147518699
local: beta=−0.13,se=0.03,t=−4.51,var=0.094
global: beta=−0.26,se=0.09,t=−3.02,var=0.011
DNAm
0.65
0.75
0.85
0.95
ancestry
0.0
0.2
0.4
0.6
0.8
1.0
chr6_25881596_25884185
local: beta=−0.16,se=0.04,t=−4.14,var=0.096
global: beta=−0.19,se=0.12,t=−1.53,var=0.011
DNAm
0.4
0.6
0.8
ancestry
0.0
0.2
0.4
0.6
0.8
1.0
chr6_26042548_26043135
local: beta=0.2,se=0.05,t=4.31,var=0.096
global: beta=0.37,se=0.14,t=2.64,var=0.011
DNAm
0.3
0.4
0.5
0.6
0.7
0.8
ancestry
0.0
0.2
0.4
0.6
0.8
1.0
chr6_28488781_28489210
local: beta=−0.15,se=0.04,t=−4.29,var=0.097
global: beta=−0.17,se=0.11,t=−1.53,var=0.011
DNAm
0.2
0.3
0.4
0.5
ancestry
0.0
0.2
0.4
0.6
0.8
1.0

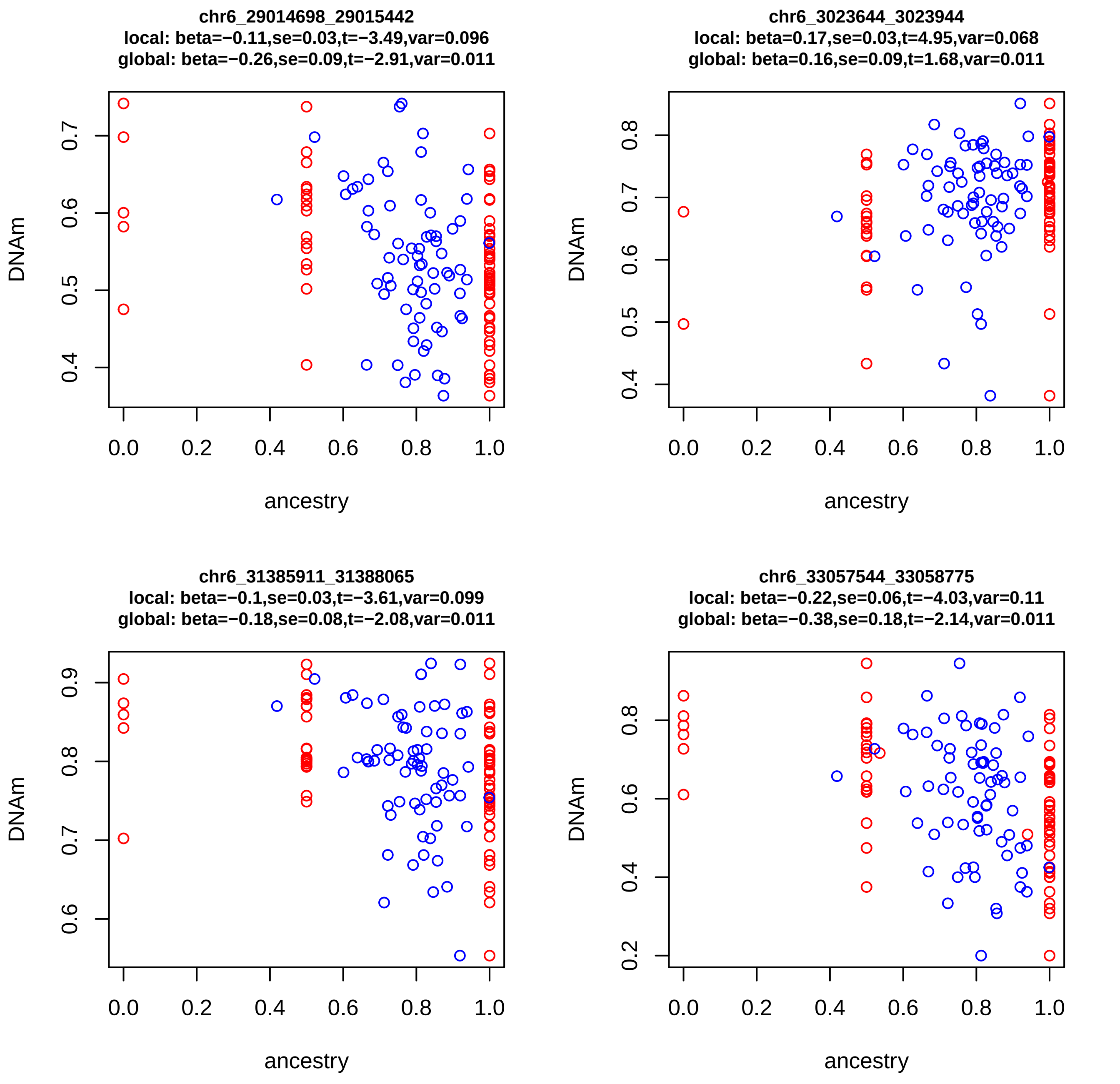
chr6_29014698_29015442
local: beta=−0.11,se=0.03,t=−3.49,var=0.096
global: beta=−0.26,se=0.09,t=−2.91,var=0.011
chr6_3023644_3023944
local: beta=0.17,se=0.03,t=4.95,var=0.068
global: beta=0.16,se=0.09,t=1.68,var=0.011
chr6_31385911_31388065
local: beta=−0.1,se=0.03,t=−3.61,var=0.099
global: beta=−0.18,se=0.08,t=−2.08,var=0.011
chr6_33057544_33058775
local: beta=−0.22,se=0.06,t=−4.03,var=0.11
global: beta=−0.38,se=0.18,t=−2.14,var=0.011
DNAm
ancestry

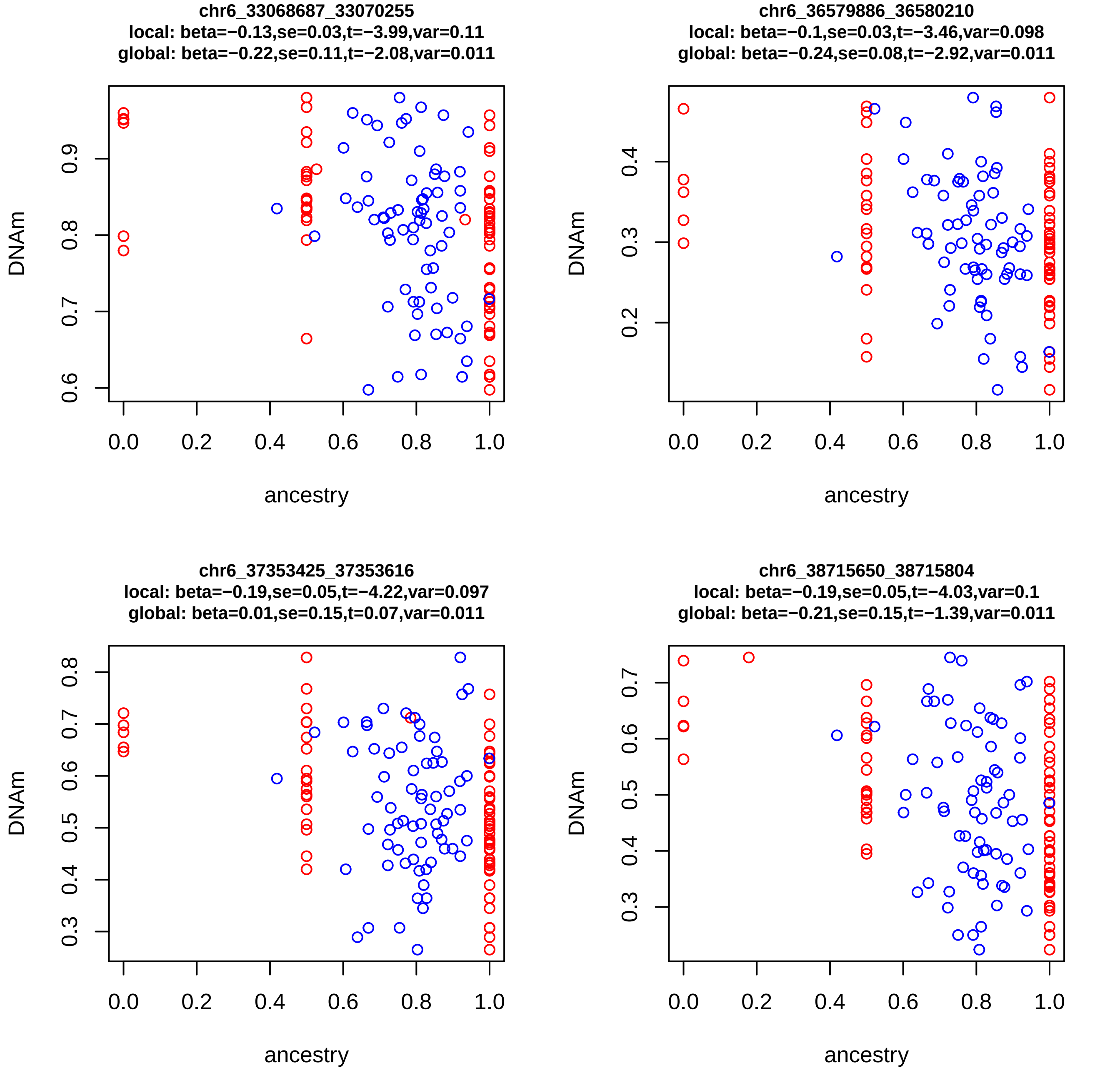

chr6_33068687_33070255
local: beta=−0.13,se=0.03,t=−3.99,var=0.11
global: beta=−0.22,se=0.11,t=−2.08,var=0.011
DNAm
0.6
0.7
0.8
0.9
0.0
0.2
0.4
0.6
0.8
1.0
ancestry
chr6_36579886_36580210
local: beta=−0.1,se=0.03,t=−3.46,var=0.098
global: beta=−0.24,se=0.08,t=−2.92,var=0.011
DNAm
0.2
0.3
0.4
0.0
0.2
0.4
0.6
0.8
1.0
ancestry
chr6_37353425_37353616
local: beta=−0.19,se=0.05,t=−4.22,var=0.097
global: beta=0.01,se=0.15,t=0.07,var=0.011
DNAm
0.3
0.4
0.5
0.6
0.7
0.8
0.0
0.2
0.4
0.6
0.8
1.0
ancestry
chr6_38715650_38715804
local: beta=−0.19,se=0.05,t=−4.03,var=0.1
global: beta=−0.21,se=0.15,t=−1.39,var=0.011
DNAm
0.3
0.4
0.5
0.6
0.7
0.0
0.2
0.4
0.6
0.8
1.0
ancestry

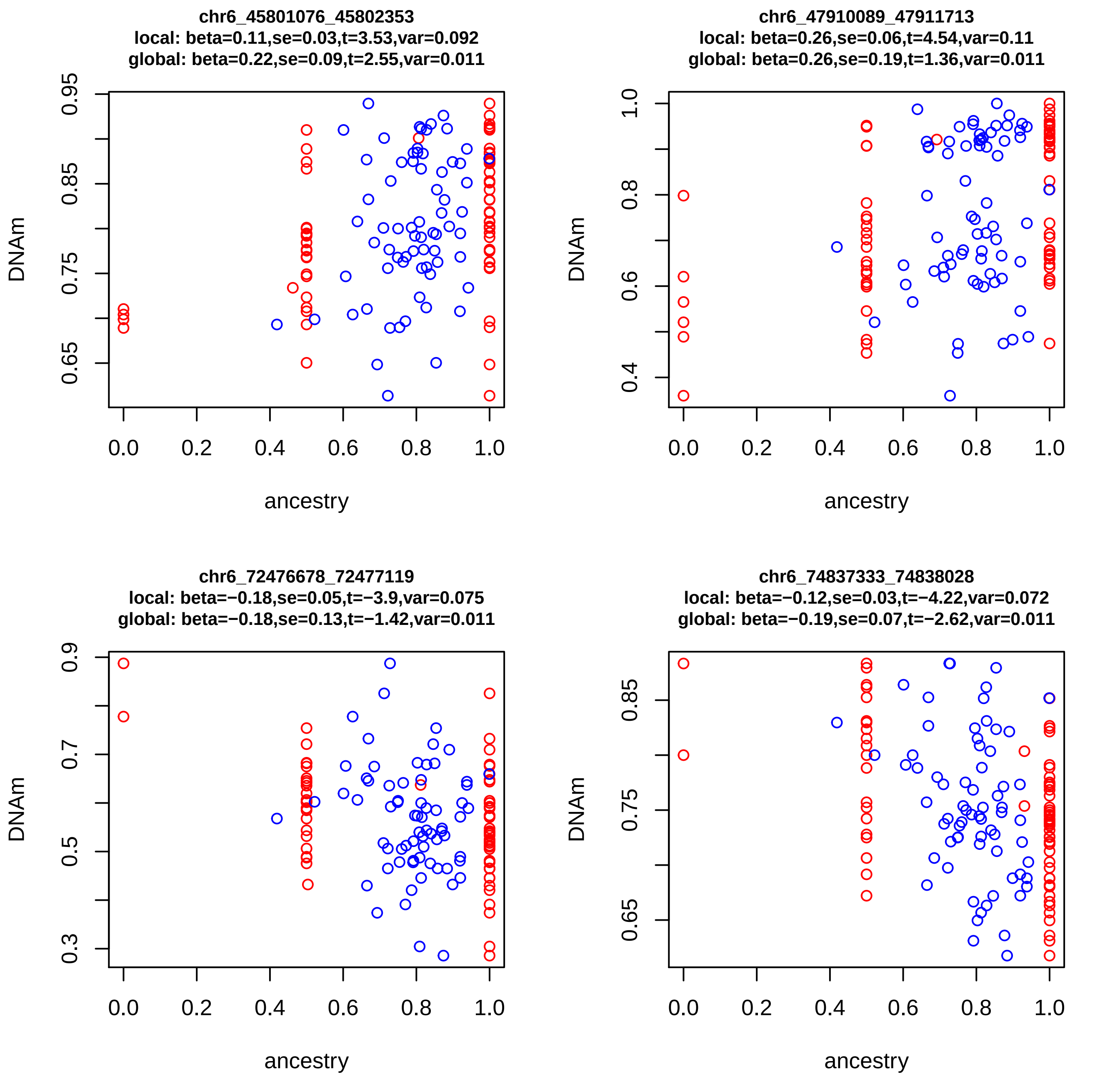
chr6_45801076_45802353
local: beta=0.11,se=0.03,t=3.53,var=0.092
global: beta=0.22,se=0.09,t=2.55,var=0.011
DNAm
ancestry
chr6_47910089_47911713
local: beta=0.26,se=0.06,t=4.54,var=0.11
global: beta=0.26,se=0.19,t=1.36,var=0.011
DNAm
ancestry
chr6_72476678_72477119
local: beta=−0.18,se=0.05,t=−3.9,var=0.075
global: beta=−0.18,se=0.13,t=−1.42,var=0.011
DNAm
ancestry
chr6_74837333_74838028
local: beta=−0.12,se=0.03,t=−4.22,var=0.072
global: beta=−0.19,se=0.07,t=−2.62,var=0.011
DNAm
ancestry

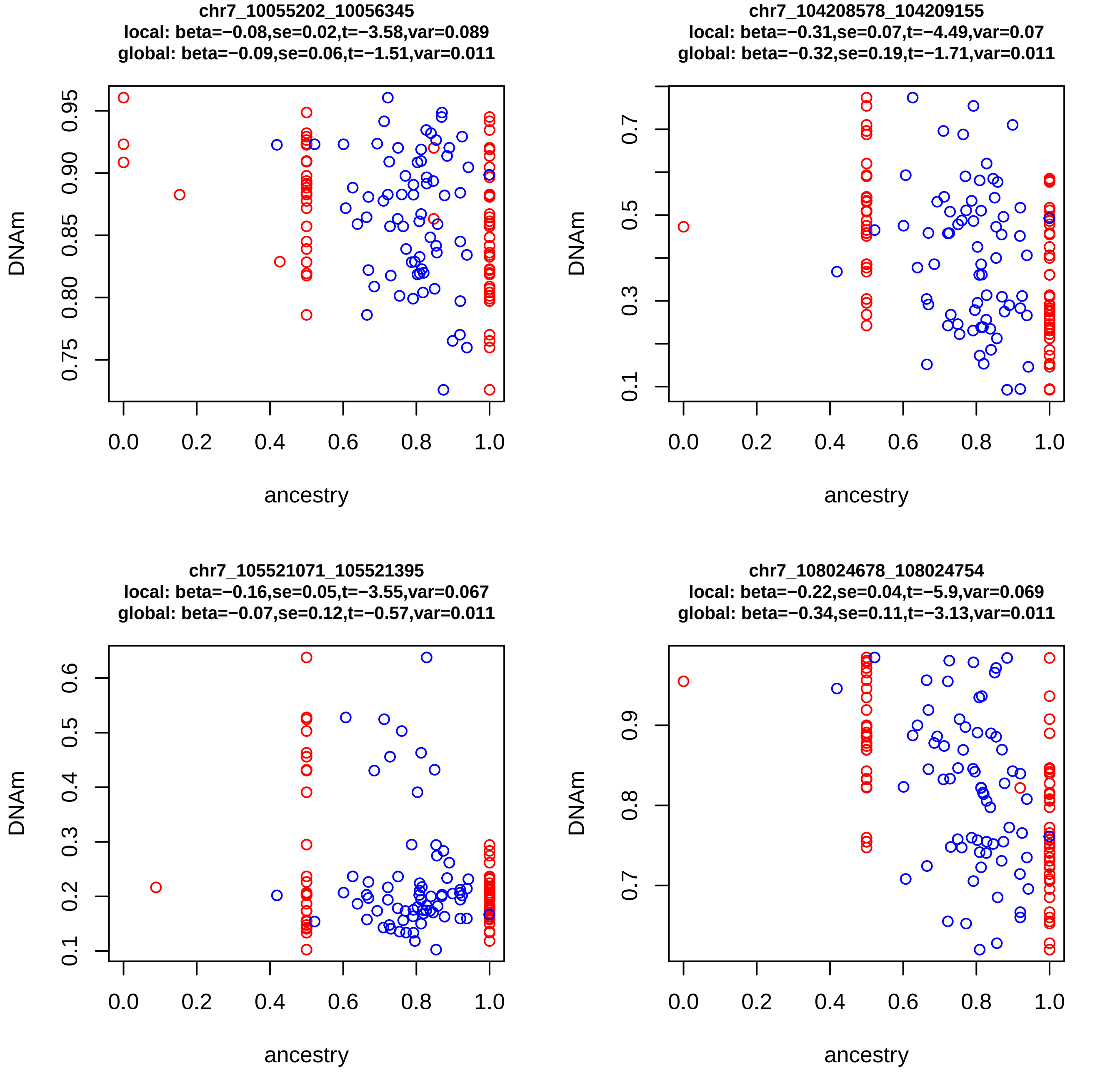
chr7_10055202_10056345
local: beta=−0.08,se=0.02,t=−3.58,var=0.089
global: beta=−0.09,se=0.06,t=−1.51,var=0.011
DNAm
0.75
0.80
0.85
0.90
0.95
0.0
0.2
0.4
0.6
0.8
1.0
ancestry
chr7_104208578_104209155
local: beta=−0.31,se=0.07,t=−4.49,var=0.07
global: beta=−0.32,se=0.19,t=−1.71,var=0.011
DNAm
0.1
0.3
0.5
0.7
0.0
0.2
0.4
0.6
0.8
1.0
ancestry
chr7_105521071_105521395
local: beta=−0.16,se=0.05,t=−3.55,var=0.067
global: beta=−0.07,se=0.12,t=−0.57,var=0.011
DNAm
0.1
0.2
0.3
0.4
0.5
0.6
0.0
0.2
0.4
0.6
0.8
1.0
ancestry
chr7_108024678_108024754
local: beta=−0.22,se=0.04,t=−5.9,var=0.069
global: beta=−0.34,se=0.11,t=−3.13,var=0.011
DNAm
0.7
0.8
0.9
0.0
0.2
0.4
0.6
0.8
1.0
ancestry

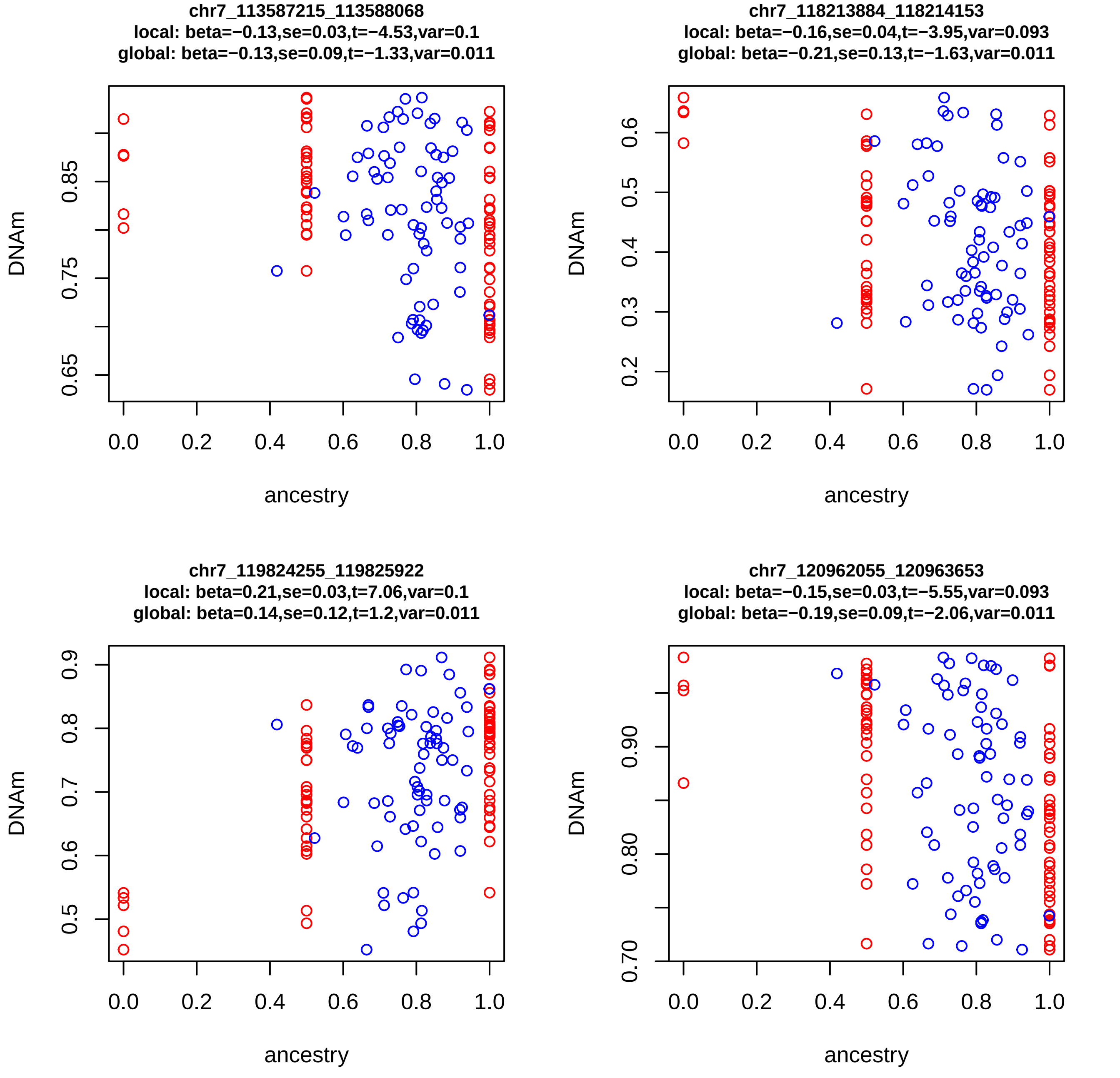
chr7_113587215_113588068
local: beta=−0.13,se=0.03,t=−4.53,var=0.1
global: beta=−0.13,se=0.09,t=−1.33,var=0.011
DNAm
0.65
0.75
0.85
ancestry
0.0
0.2
0.4
0.6
0.8
1.0
chr7_118213884_118214153
local: beta=−0.16,se=0.04,t=−3.95,var=0.093
global: beta=−0.21,se=0.13,t=−1.63,var=0.011
DNAm
0.2
0.3
0.4
0.5
0.6
ancestry
0.0
0.2
0.4
0.6
0.8
1.0
chr7_119824255_119825922
local: beta=0.21,se=0.03,t=7.06,var=0.1
global: beta=0.14,se=0.12,t=1.2,var=0.011
DNAm
0.5
0.6
0.7
0.8
0.9
ancestry
0.0
0.2
0.4
0.6
0.8
1.0
chr7_120962055_120963653
local: beta=−0.15,se=0.03,t=−5.55,var=0.093
global: beta=−0.19,se=0.09,t=−2.06,var=0.011
DNAm
0.70
0.80
0.90
ancestry
0.0
0.2
0.4
0.6
0.8
1.0

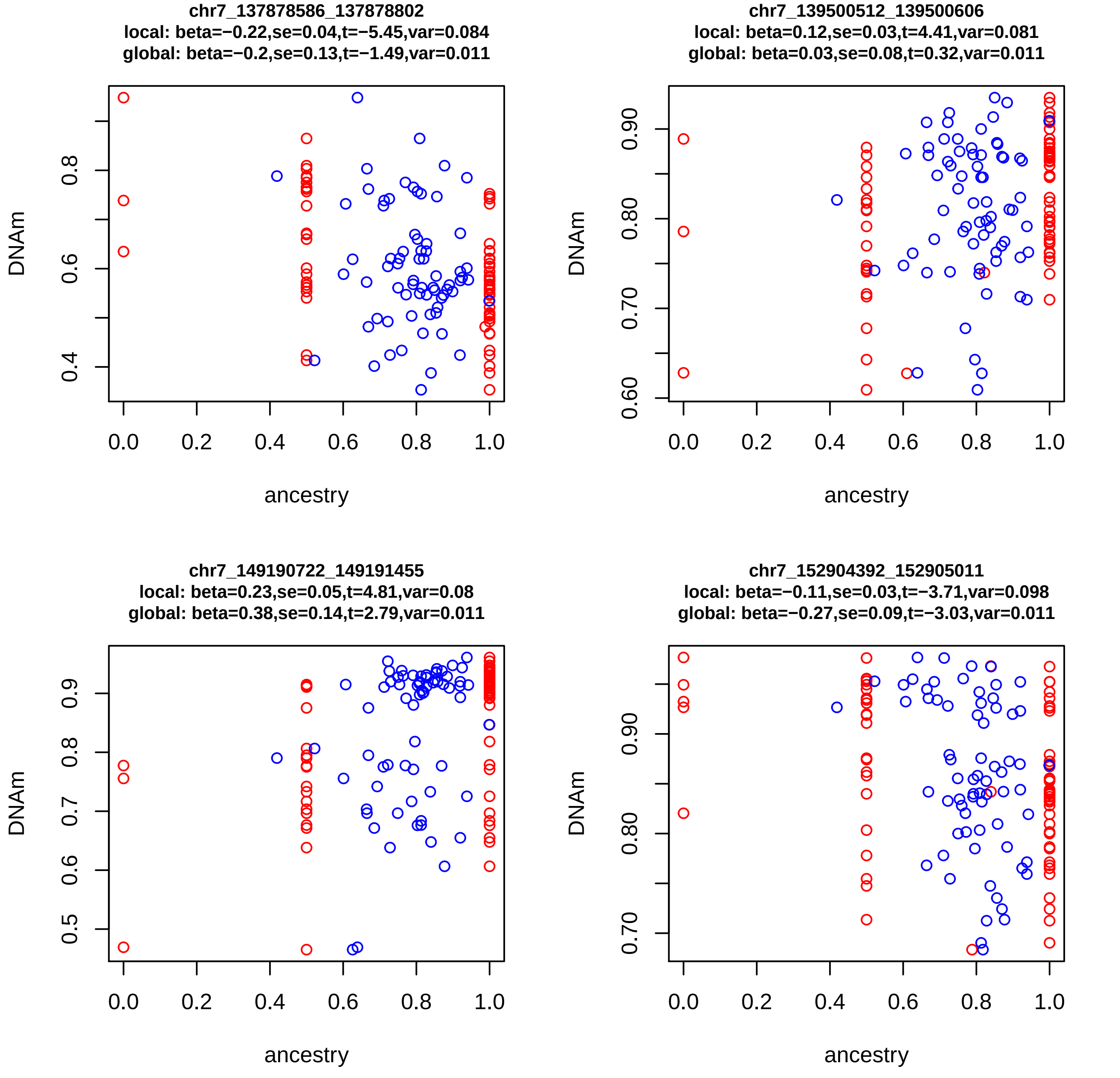
chr7_137878586_137878802
local: beta=−0.22,se=0.04,t=−5.45,var=0.084
global: beta=−0.2,se=0.13,t=−1.49,var=0.011
DNAm
ancestry
chr7_139500512_139500606
local: beta=0.12,se=0.03,t=4.41,var=0.081
global: beta=0.03,se=0.08,t=0.32,var=0.011
DNAm
ancestry
chr7_149190722_149191455
local: beta=0.23,se=0.05,t=4.81,var=0.08
global: beta=0.38,se=0.14,t=2.79,var=0.011
DNAm
ancestry
chr7_152904392_152905011
local: beta=−0.11,se=0.03,t=−3.71,var=0.098
global: beta=−0.27,se=0.09,t=−3.03,var=0.011
DNAm
ancestry

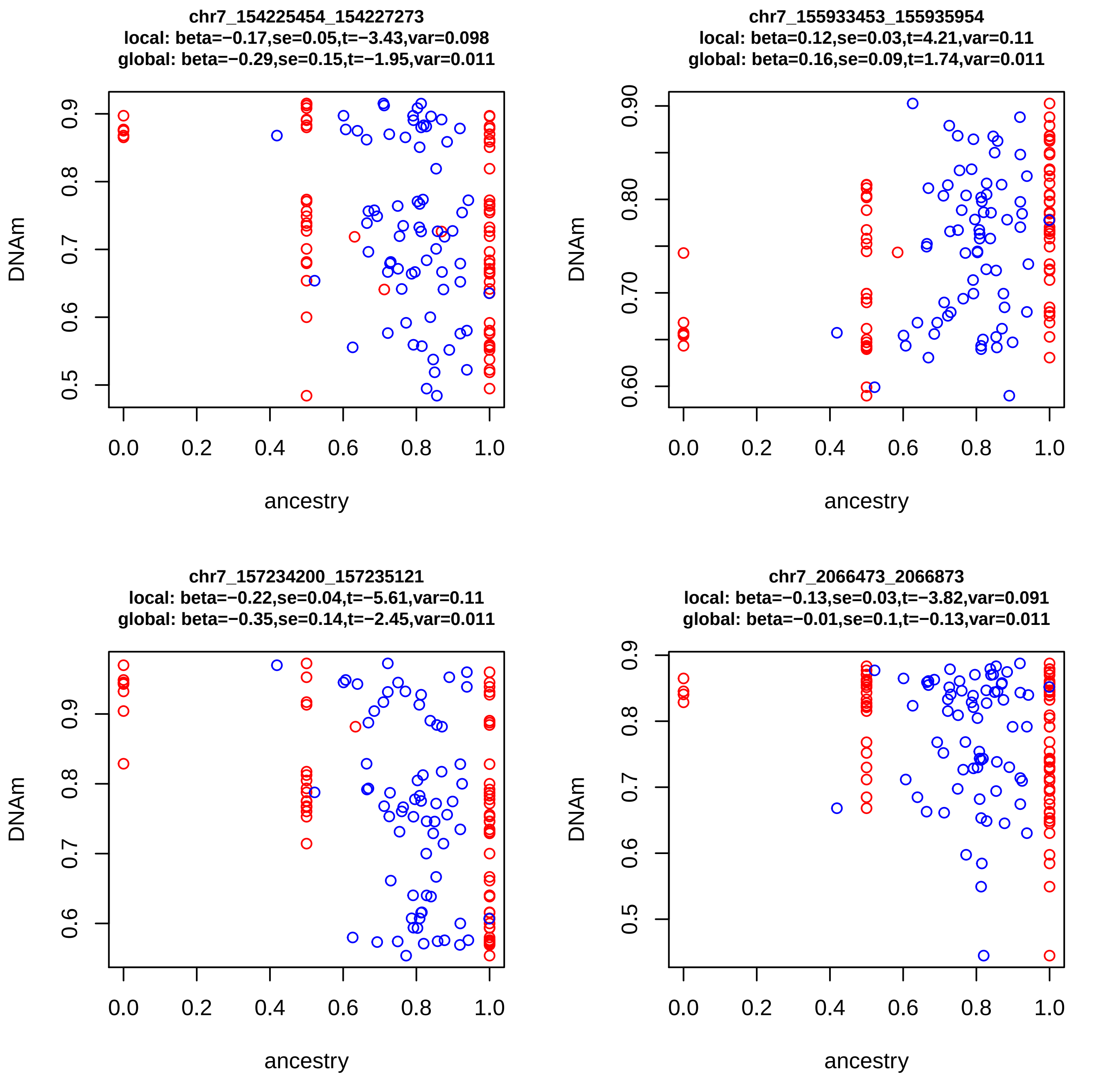
chr7_154225454_154227273
local: beta=−0.17,se=0.05,t=−3.43,var=0.098
global: beta=−0.29,se=0.15,t=−1.95,var=0.011
DNAm
ancestry
chr7_155933453_155935954
local: beta=0.12,se=0.03,t=4.21,var=0.11
global: beta=0.16,se=0.09,t=1.74,var=0.011
DNAm
ancestry
chr7_157234200_157235121
local: beta=−0.22,se=0.04,t=−5.61,var=0.11
global: beta=−0.35,se=0.14,t=−2.45,var=0.011
DNAm
ancestry
chr7_2066473_2066873
local: beta=−0.13,se=0.03,t=−3.82,var=0.091
global: beta=−0.01,se=0.1,t=−0.13,var=0.011
DNAm
ancestry

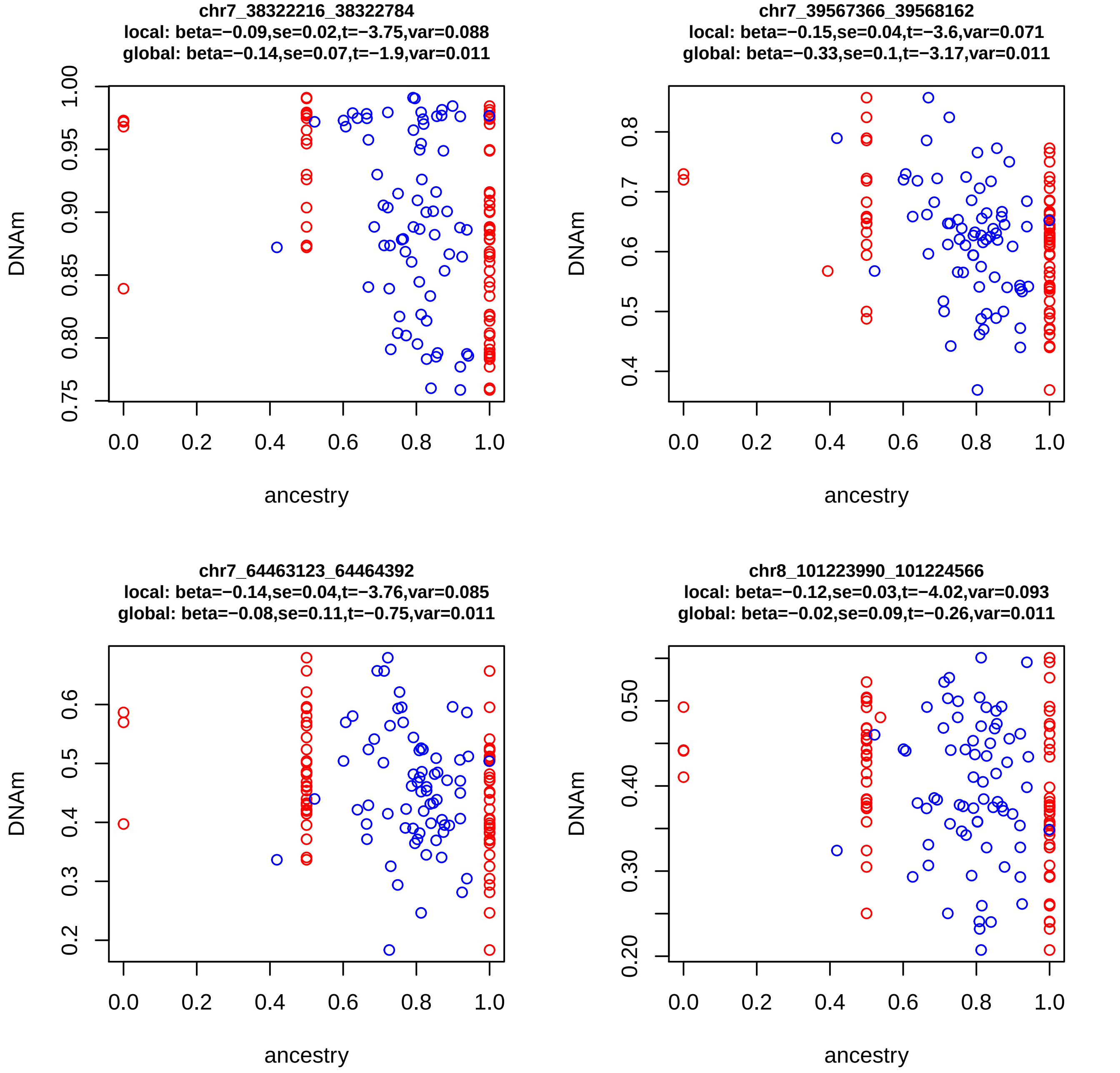
chr7_38322216_38322784
local: beta=−0.09,se=0.02,t=−3.75,var=0.088
global: beta=−0.14,se=0.07,t=−1.9,var=0.011
DNAm
0.75 0.80 0.85 0.90 0.95 1.00
0.0 0.2 0.4 0.6 0.8 1.0
ancestry
chr7_39567366_39568162
local: beta=−0.15,se=0.04,t=−3.6,var=0.071
global: beta=−0.33,se=0.1,t=−3.17,var=0.011
DNAm
0.4 0.5 0.6 0.7 0.8
0.0 0.2 0.4 0.6 0.8 1.0
ancestry
chr7_64463123_64464392
local: beta=−0.14,se=0.04,t=−3.76,var=0.085
global: beta=−0.08,se=0.11,t=−0.75,var=0.011
DNAm
0.2 0.3 0.4 0.5 0.6
0.0 0.2 0.4 0.6 0.8 1.0
ancestry
chr8_101223990_101224566
local: beta=−0.12,se=0.03,t=−4.02,var=0.093
global: beta=−0.02,se=0.09,t=−0.26,var=0.011
DNAm
0.20 0.30 0.40 0.50
0.0 0.2 0.4 0.6 0.8 1.0
ancestry

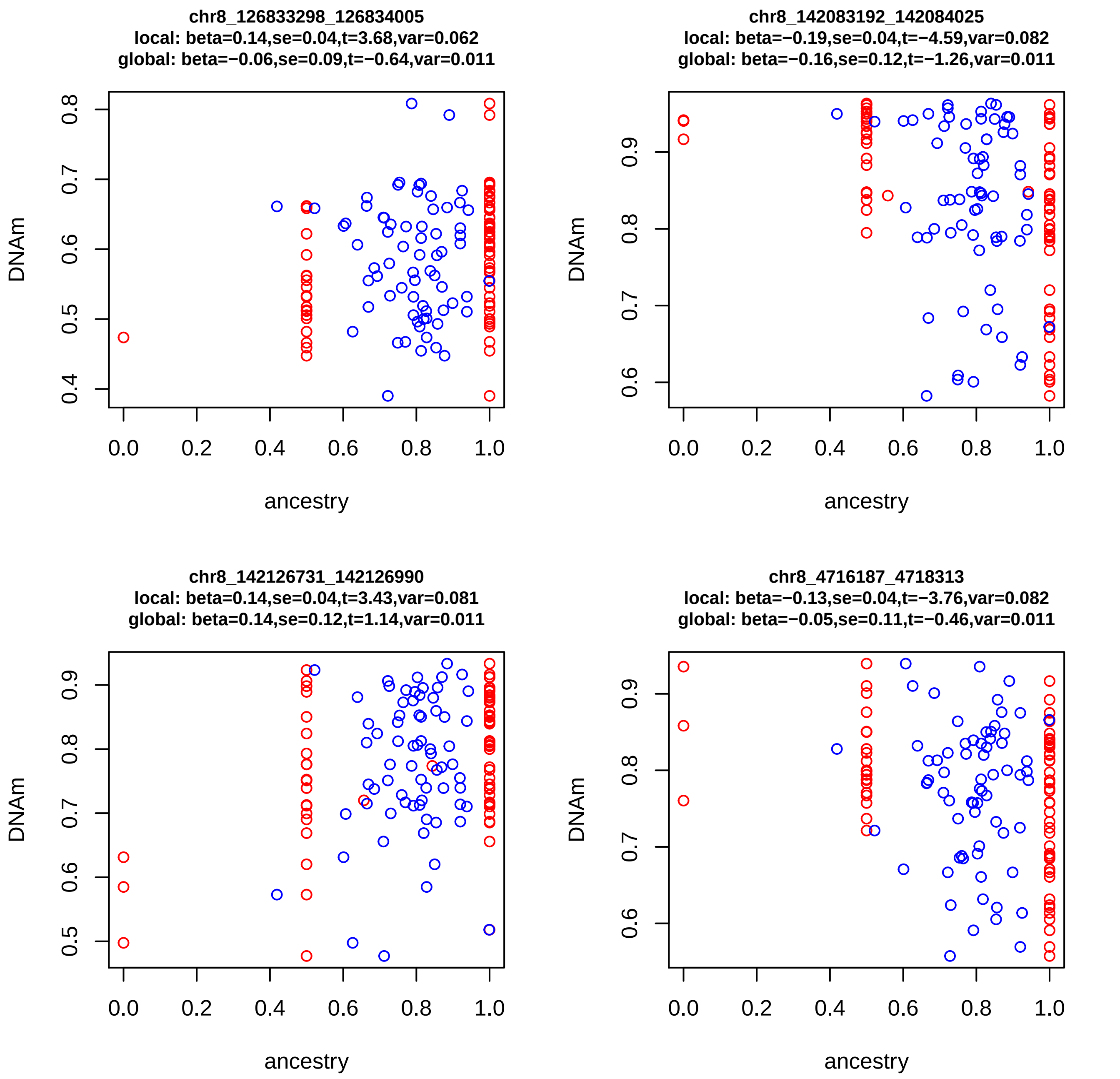
chr8_126833298_126834005
local: beta=0.14,se=0.04,t=3.68,var=0.062
global: beta=−0.06,se=0.09,t=−0.64,var=0.011
chr8_142083192_142084025
local: beta=−0.19,se=0.04,t=−4.59,var=0.082
global: beta=−0.16,se=0.12,t=−1.26,var=0.011
chr8_142126731_142126990
local: beta=0.14,se=0.04,t=3.43,var=0.081
global: beta=0.14,se=0.12,t=1.14,var=0.011
chr8_4716187_4718313
local: beta=−0.13,se=0.04,t=−3.76,var=0.082
global: beta=−0.05,se=0.11,t=−0.46,var=0.011
DNAm
ancestry
0.0
0.2
0.4
0.6
0.8
1.0

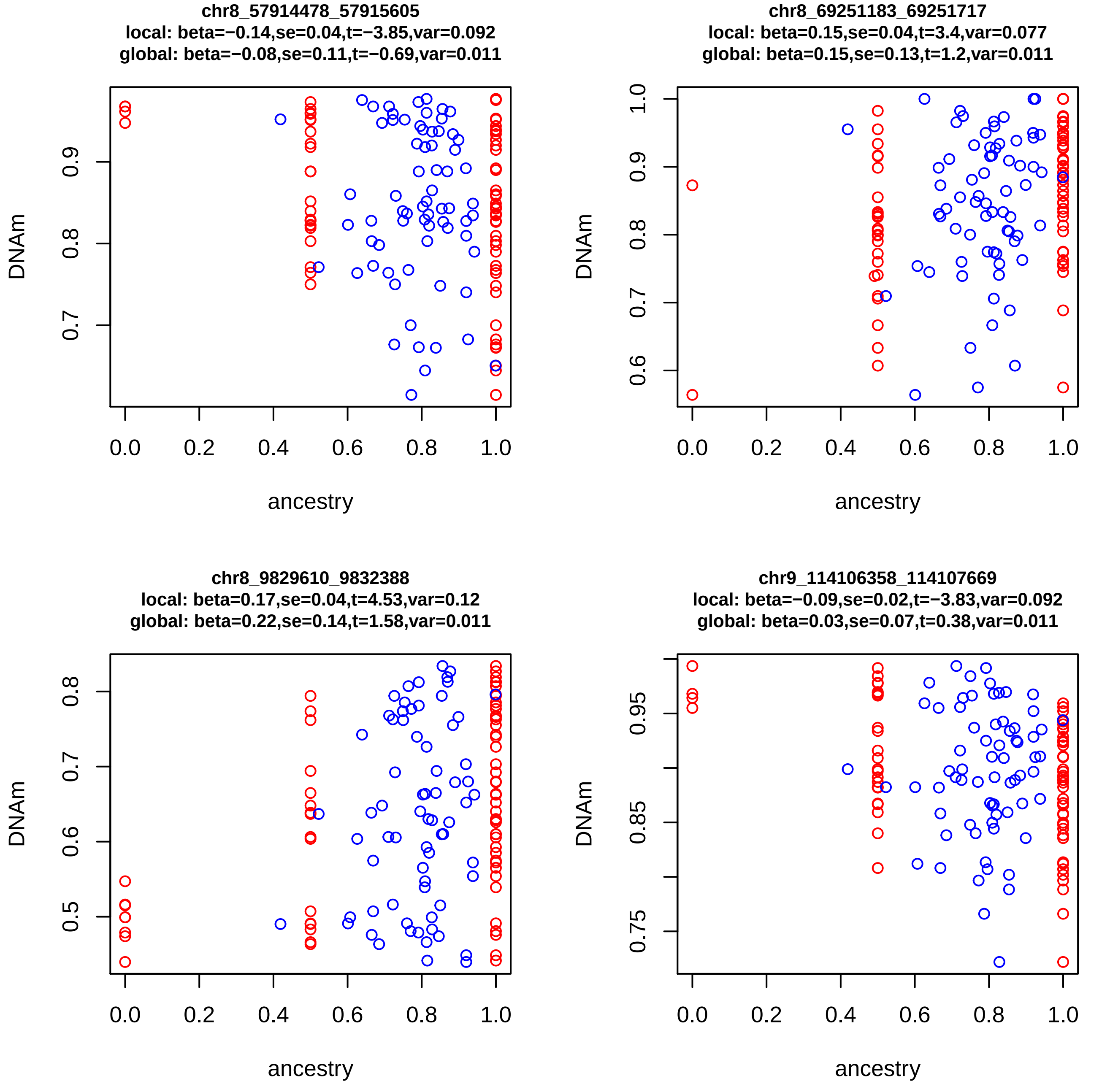
chr8_57914478_57915605
local: beta=−0.14,se=0.04,t=−3.85,var=0.092
global: beta=−0.08,se=0.11,t=−0.69,var=0.011
DNAm
ancestry
chr8_69251183_69251717
local: beta=0.15,se=0.04,t=3.4,var=0.077
global: beta=0.15,se=0.13,t=1.2,var=0.011
DNAm
ancestry
chr8_9829610_9832388
local: beta=0.17,se=0.04,t=4.53,var=0.12
global: beta=0.22,se=0.14,t=1.58,var=0.011
DNAm
ancestry
chr9_114106358_114107669
local: beta=−0.09,se=0.02,t=−3.83,var=0.092
global: beta=0.03,se=0.07,t=0.38,var=0.011
DNAm
ancestry

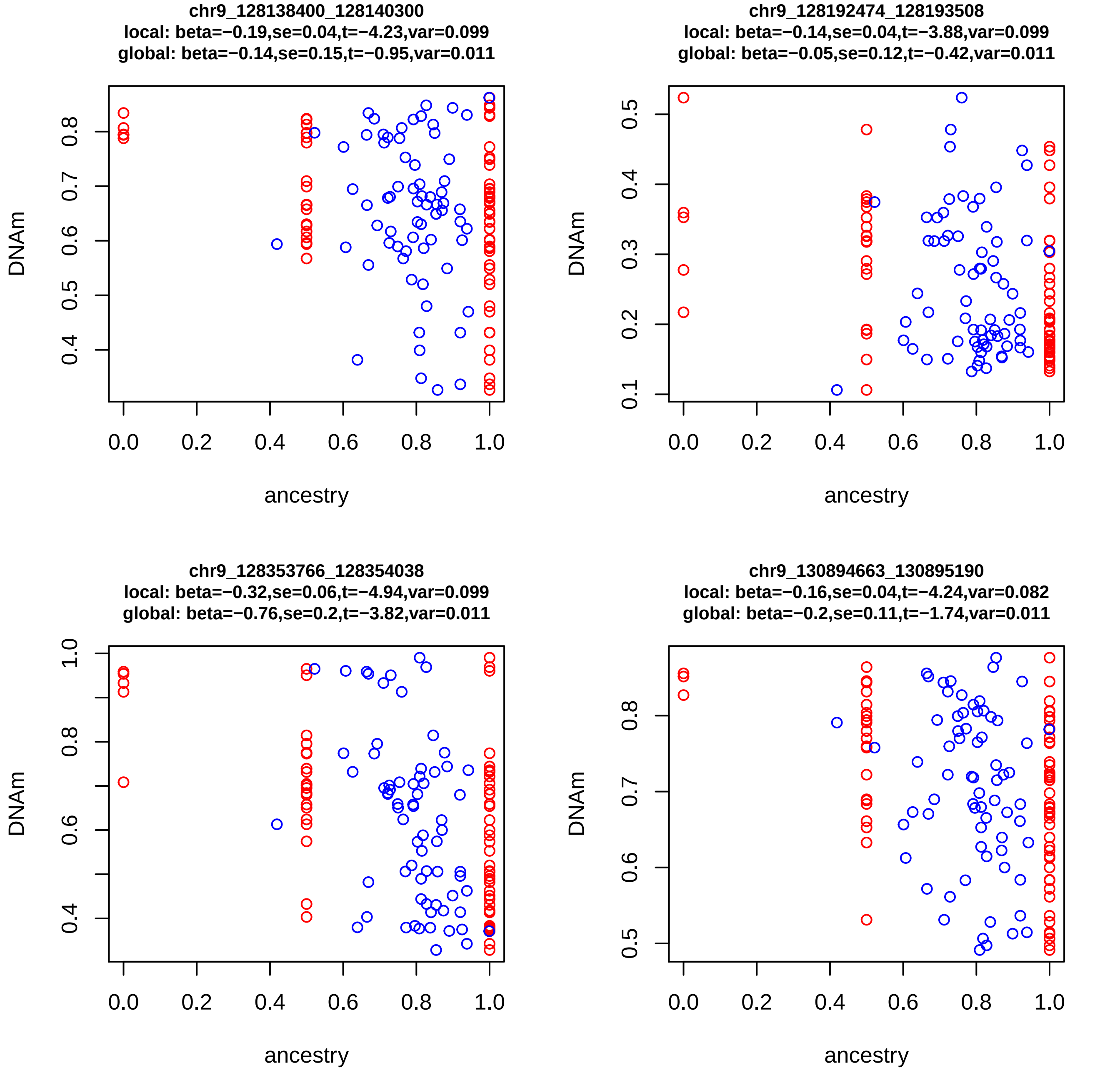
chr9_128138400_128140300
local: beta=−0.19,se=0.04,t=−4.23,var=0.099
global: beta=−0.14,se=0.15,t=−0.95,var=0.011
DNAm
ancestry
0.4
0.5
0.6
0.7
0.8
0.0
0.2
0.4
0.6
0.8
1.0
chr9_128192474_128193508
local: beta=−0.14,se=0.04,t=−3.88,var=0.099
global: beta=−0.05,se=0.12,t=−0.42,var=0.011
DNAm
ancestry
0.1
0.2
0.3
0.4
0.5
0.0
0.2
0.4
0.6
0.8
1.0
chr9_128353766_128354038
local: beta=−0.32,se=0.06,t=−4.94,var=0.099
global: beta=−0.76,se=0.2,t=−3.82,var=0.011
DNAm
ancestry
0.4
0.6
0.8
1.0
0.0
0.2
0.4
0.6
0.8
1.0
chr9_130894663_130895190
local: beta=−0.16,se=0.04,t=−4.24,var=0.082
global: beta=−0.2,se=0.11,t=−1.74,var=0.011
DNAm
ancestry
0.5
0.6
0.7
0.8
0.0
0.2
0.4
0.6
0.8
1.0

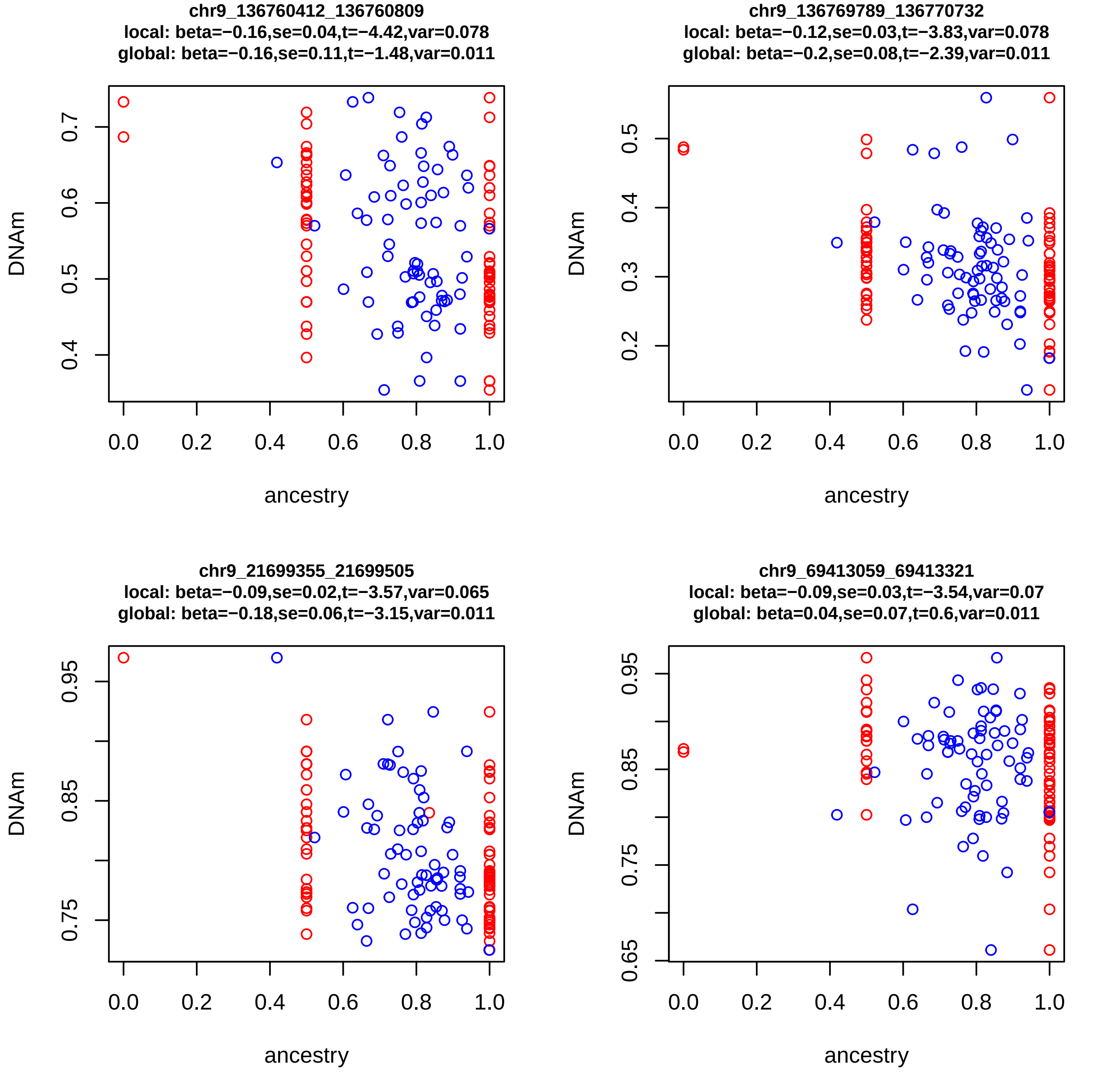
chr9_136760412_136760809
local: beta=−0.16,se=0.04,t=−4.42,var=0.078
global: beta=−0.16,se=0.11,t=−1.48,var=0.011
DNAm
ancestry
chr9_136769789_136770732
local: beta=−0.12,se=0.03,t=−3.83,var=0.078
global: beta=−0.2,se=0.08,t=−2.39,var=0.011
DNAm
ancestry
chr9_21699355_21699505
local: beta=−0.09,se=0.02,t=−3.57,var=0.065
global: beta=−0.18,se=0.06,t=−3.15,var=0.011
DNAm
ancestry
chr9_69413059_69413321
local: beta=−0.09,se=0.03,t=−3.54,var=0.07
global: beta=0.04,se=0.07,t=0.6,var=0.011
DNAm
ancestry

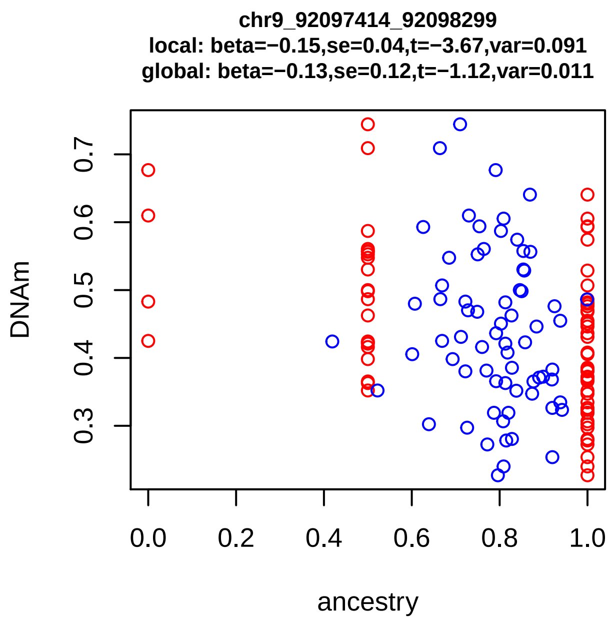

chr9_92097414_92098299
local: beta=−0.15,se=0.04,t=−3.67,var=0.091
global: beta=−0.13,se=0.12,t=−1.12,var=0.011
DNAm
0.3
0.4
0.5
0.6
0.7
0.0
0.2
0.4
0.6
0.8
1.0
ancestry
