# Supplementary material for: Analysis of gene expression in the postmortem brain of neurotypical Black Americans reveals contributions of genetic ancestry
Source: Nat Neurosci. 2024 May 20;27(6):1064–74. doi: 10.1038/s41593-024-01636-0 (PMC11156587; doi:10.1038/s41593-024-01636-0)
Supplement: Supplementary file 7 — Compressed directory of PDF of scatter plots comparing DNA methylation association with local and global ancestry for the caudate nucleus, DLPFC and hippocampus. Plots are annotated with the genetic ancestry DMR test results. [file 41593_2024_1636_MOESM7_ESM.gz › DMR_global_local_comparison/DMR_global_local_compare_by_region_dlpfc.pdf]

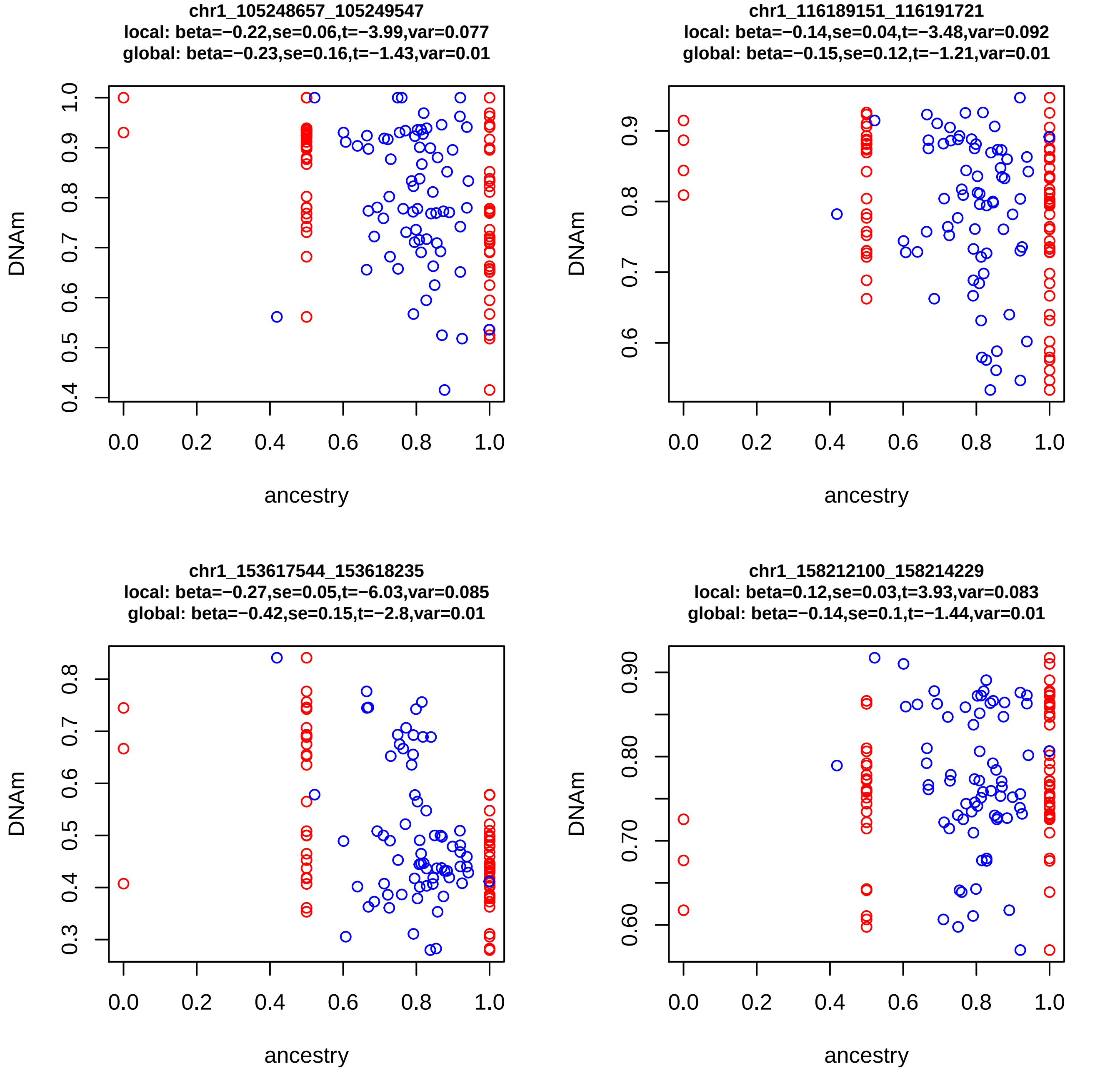
chr1_105248657_105249547
local: beta=−0.22,se=0.06,t=−3.99,var=0.077
global: beta=−0.23,se=0.16,t=−1.43,var=0.01
chr1_116189151_116191721
local: beta=−0.14,se=0.04,t=−3.48,var=0.092
global: beta=−0.15,se=0.12,t=−1.21,var=0.01
chr1_153617544_153618235
local: beta=−0.27,se=0.05,t=−6.03,var=0.085
global: beta=−0.42,se=0.15,t=−2.8,var=0.01
chr1_158212100_158214229
local: beta=0.12,se=0.03,t=3.93,var=0.083
global: beta=−0.14,se=0.1,t=−1.44,var=0.01
DNAm
ancestry
0.0
0.2
0.4
0.6
0.8
1.0

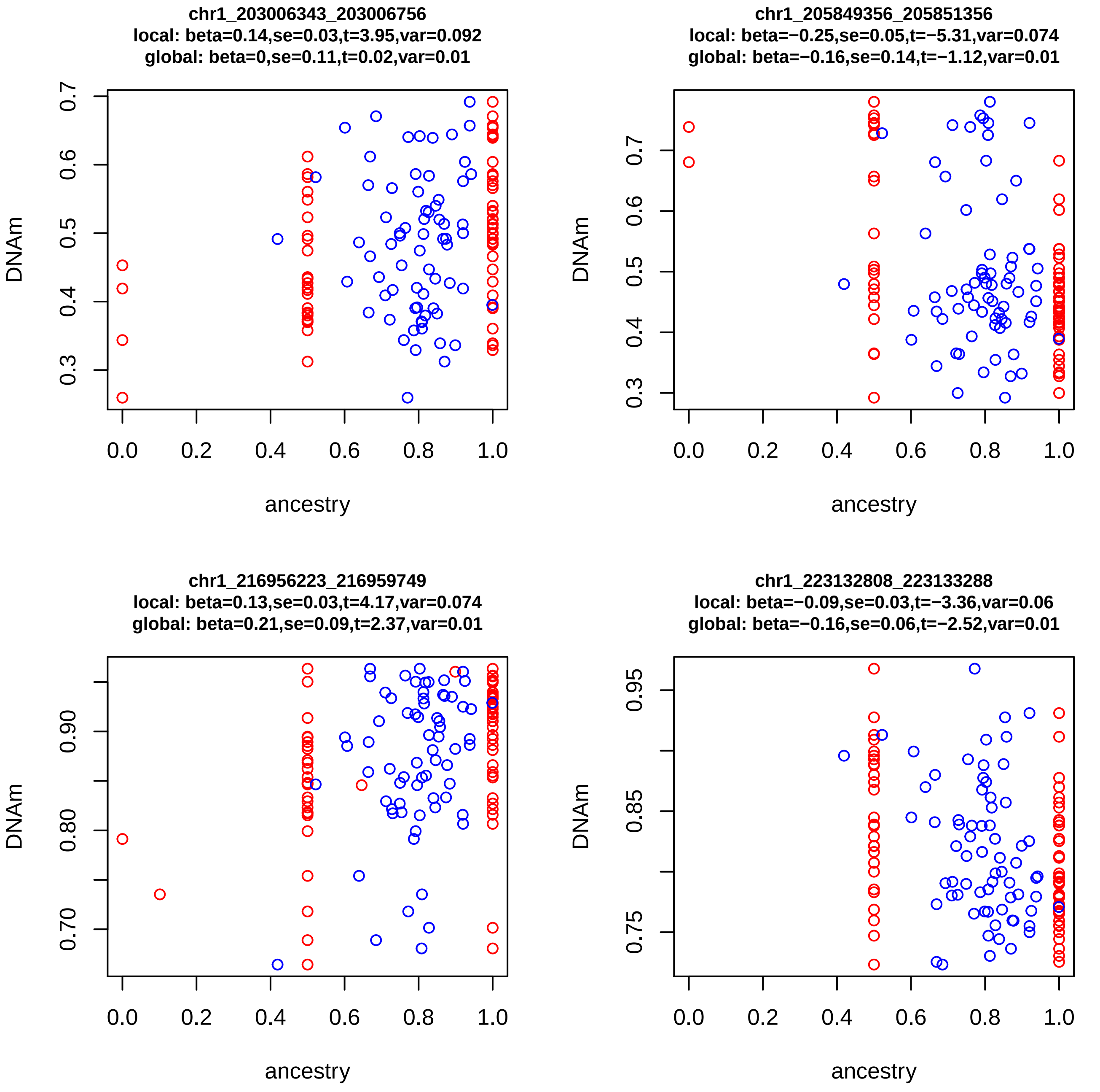
chr1_203006343_203006756
local: beta=0.14,se=0.03,t=3.95,var=0.092
global: beta=0,se=0.11,t=0.02,var=0.01
DNAm
ancestry
chr1_205849356_205851356
local: beta=−0.25,se=0.05,t=−5.31,var=0.074
global: beta=−0.16,se=0.14,t=−1.12,var=0.01
DNAm
ancestry
chr1_216956223_216959749
local: beta=0.13,se=0.03,t=4.17,var=0.074
global: beta=0.21,se=0.09,t=2.37,var=0.01
DNAm
ancestry
chr1_223132808_223133288
local: beta=−0.09,se=0.03,t=−3.36,var=0.06
global: beta=−0.16,se=0.06,t=−2.52,var=0.01
DNAm
ancestry

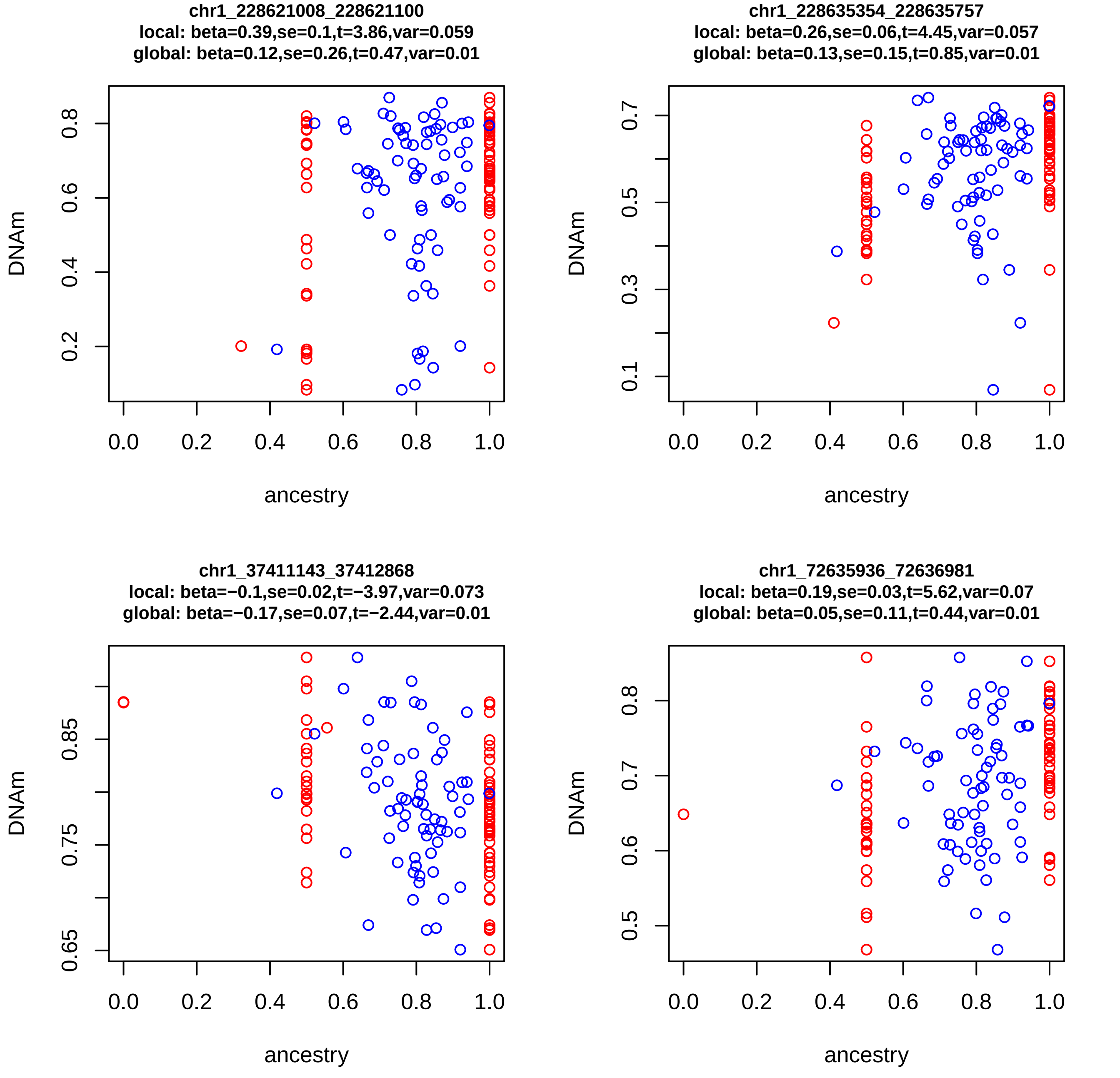
chr1_228621008_228621100
local: beta=0.39,se=0.1,t=3.86,var=0.059
global: beta=0.12,se=0.26,t=0.47,var=0.01
DNAm
0.2
0.4
0.6
0.8
0.0
0.2
0.4
0.6
0.8
1.0
ancestry
chr1_228635354_228635757
local: beta=0.26,se=0.06,t=4.45,var=0.057
global: beta=0.13,se=0.15,t=0.85,var=0.01
DNAm
0.1
0.3
0.5
0.7
0.0
0.2
0.4
0.6
0.8
1.0
ancestry
chr1_37411143_37412868
local: beta=−0.1,se=0.02,t=−3.97,var=0.073
global: beta=−0.17,se=0.07,t=−2.44,var=0.01
DNAm
0.65
0.75
0.85
0.0
0.2
0.4
0.6
0.8
1.0
ancestry
chr1_72635936_72636981
local: beta=0.19,se=0.03,t=5.62,var=0.07
global: beta=0.05,se=0.11,t=0.44,var=0.01
DNAm
0.5
0.6
0.7
0.8
0.0
0.2
0.4
0.6
0.8
1.0
ancestry

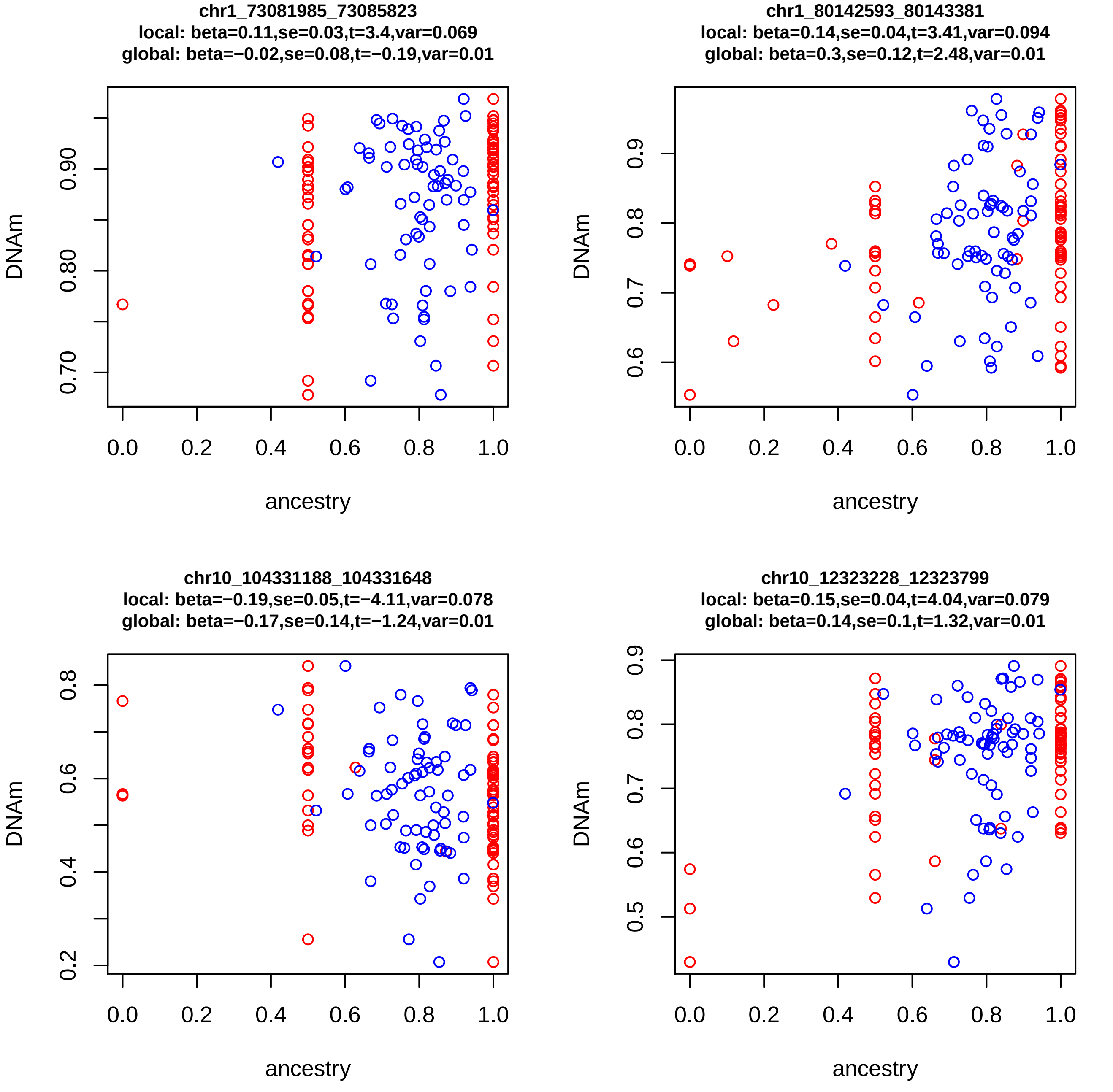
chr1_73081985_73085823
local: beta=0.11,se=0.03,t=3.4,var=0.069
global: beta=−0.02,se=0.08,t=−0.19,var=0.01
DNAm
0.70
0.80
0.90
0.0
0.2
0.4
0.6
0.8
1.0
ancestry
chr1_80142593_80143381
local: beta=0.14,se=0.04,t=3.41,var=0.094
global: beta=0.3,se=0.12,t=2.48,var=0.01
DNAm
0.6
0.7
0.8
0.9
0.0
0.2
0.4
0.6
0.8
1.0
ancestry
chr10_104331188_104331648
local: beta=−0.19,se=0.05,t=−4.11,var=0.078
global: beta=−0.17,se=0.14,t=−1.24,var=0.01
DNAm
0.2
0.4
0.6
0.8
0.0
0.2
0.4
0.6
0.8
1.0
ancestry
chr10_12323228_12323799
local: beta=0.15,se=0.04,t=4.04,var=0.079
global: beta=0.14,se=0.1,t=1.32,var=0.01
DNAm
0.5
0.6
0.7
0.8
0.9
0.0
0.2
0.4
0.6
0.8
1.0
ancestry

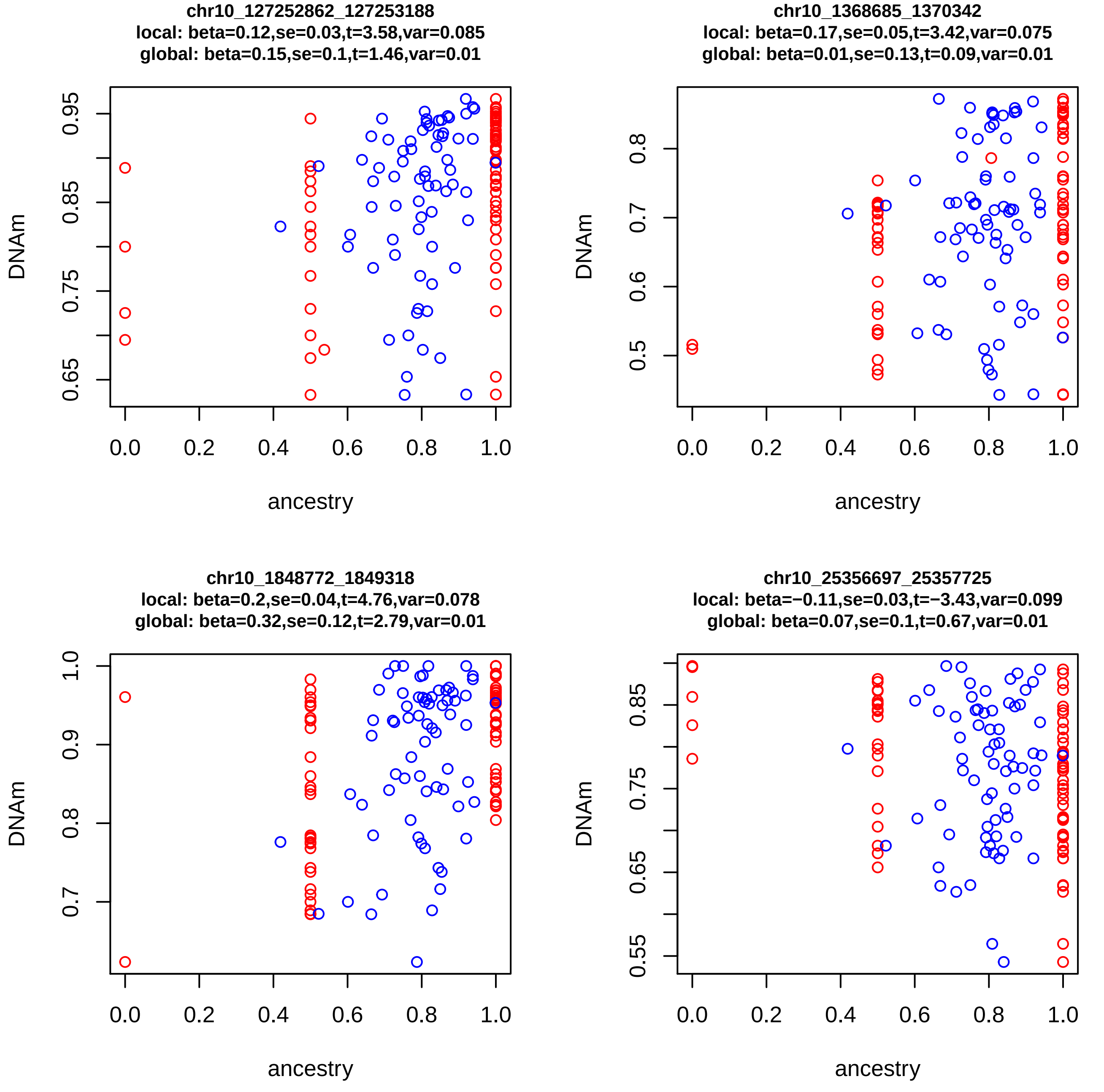
chr10_127252862_127253188
local: beta=0.12,se=0.03,t=3.58,var=0.085
global: beta=0.15,se=0.1,t=1.46,var=0.01
DNAm
ancestry
chr10_1368685_1370342
local: beta=0.17,se=0.05,t=3.42,var=0.075
global: beta=0.01,se=0.13,t=0.09,var=0.01
DNAm
ancestry
chr10_1848772_1849318
local: beta=0.2,se=0.04,t=4.76,var=0.078
global: beta=0.32,se=0.12,t=2.79,var=0.01
DNAm
ancestry
chr10_25356697_25357725
local: beta=−0.11,se=0.03,t=−3.43,var=0.099
global: beta=0.07,se=0.1,t=0.67,var=0.01
DNAm
ancestry

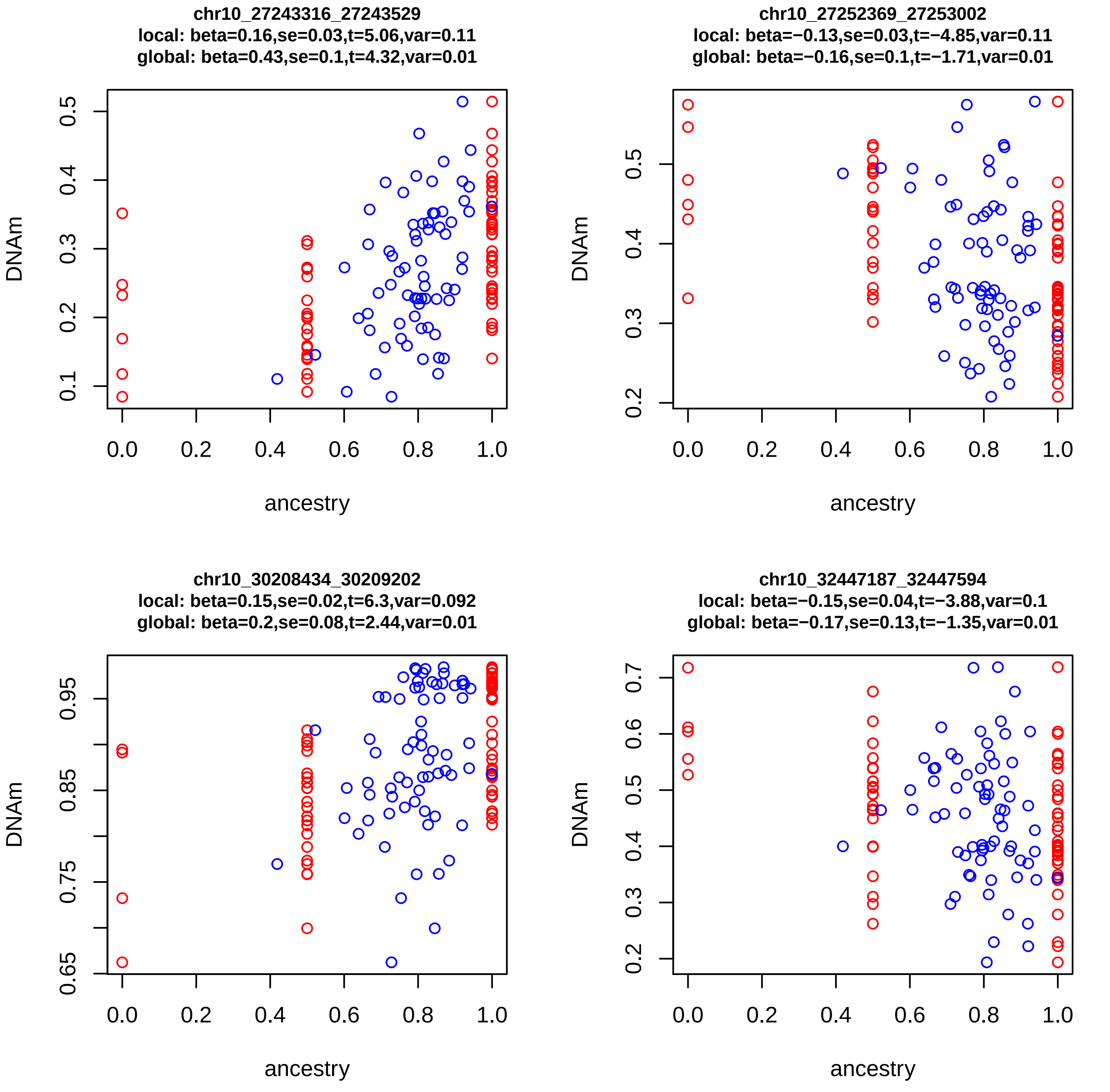
chr10_27243316_27243529
local: beta=0.16,se=0.03,t=5.06,var=0.11
global: beta=0.43,se=0.1,t=4.32,var=0.01
DNAm
0.1
0.2
0.3
0.4
0.5
0.0
0.2
0.4
0.6
0.8
1.0
ancestry
chr10_27252369_27253002
local: beta=−0.13,se=0.03,t=−4.85,var=0.11
global: beta=−0.16,se=0.1,t=−1.71,var=0.01
DNAm
0.2
0.3
0.4
0.5
0.0
0.2
0.4
0.6
0.8
1.0
ancestry
chr10_30208434_30209202
local: beta=0.15,se=0.02,t=6.3,var=0.092
global: beta=0.2,se=0.08,t=2.44,var=0.01
DNAm
0.65
0.75
0.85
0.95
0.0
0.2
0.4
0.6
0.8
1.0
ancestry
chr10_32447187_32447594
local: beta=−0.15,se=0.04,t=−3.88,var=0.1
global: beta=−0.17,se=0.13,t=−1.35,var=0.01
DNAm
0.2
0.3
0.4
0.5
0.6
0.7
0.0
0.2
0.4
0.6
0.8
1.0
ancestry

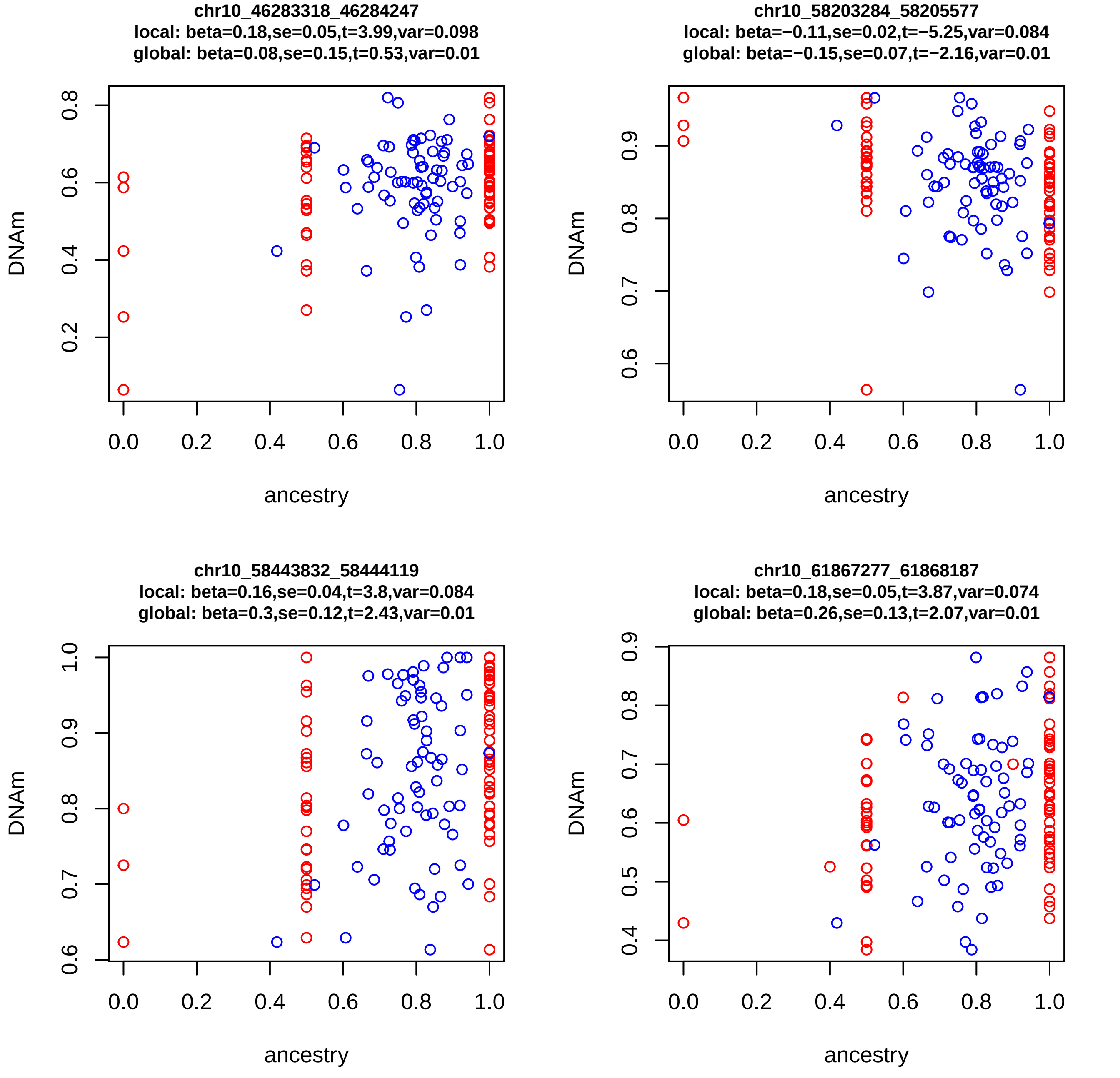
chr10_46283318_46284247
local: beta=0.18,se=0.05,t=3.99,var=0.098
global: beta=0.08,se=0.15,t=0.53,var=0.01
DNAm
ancestry
chr10_58203284_58205577
local: beta=−0.11,se=0.02,t=−5.25,var=0.084
global: beta=−0.15,se=0.07,t=−2.16,var=0.01
DNAm
ancestry
chr10_58443832_58444119
local: beta=0.16,se=0.04,t=3.8,var=0.084
global: beta=0.3,se=0.12,t=2.43,var=0.01
DNAm
ancestry
chr10_61867277_61868187
local: beta=0.18,se=0.05,t=3.87,var=0.074
global: beta=0.26,se=0.13,t=2.07,var=0.01
DNAm
ancestry

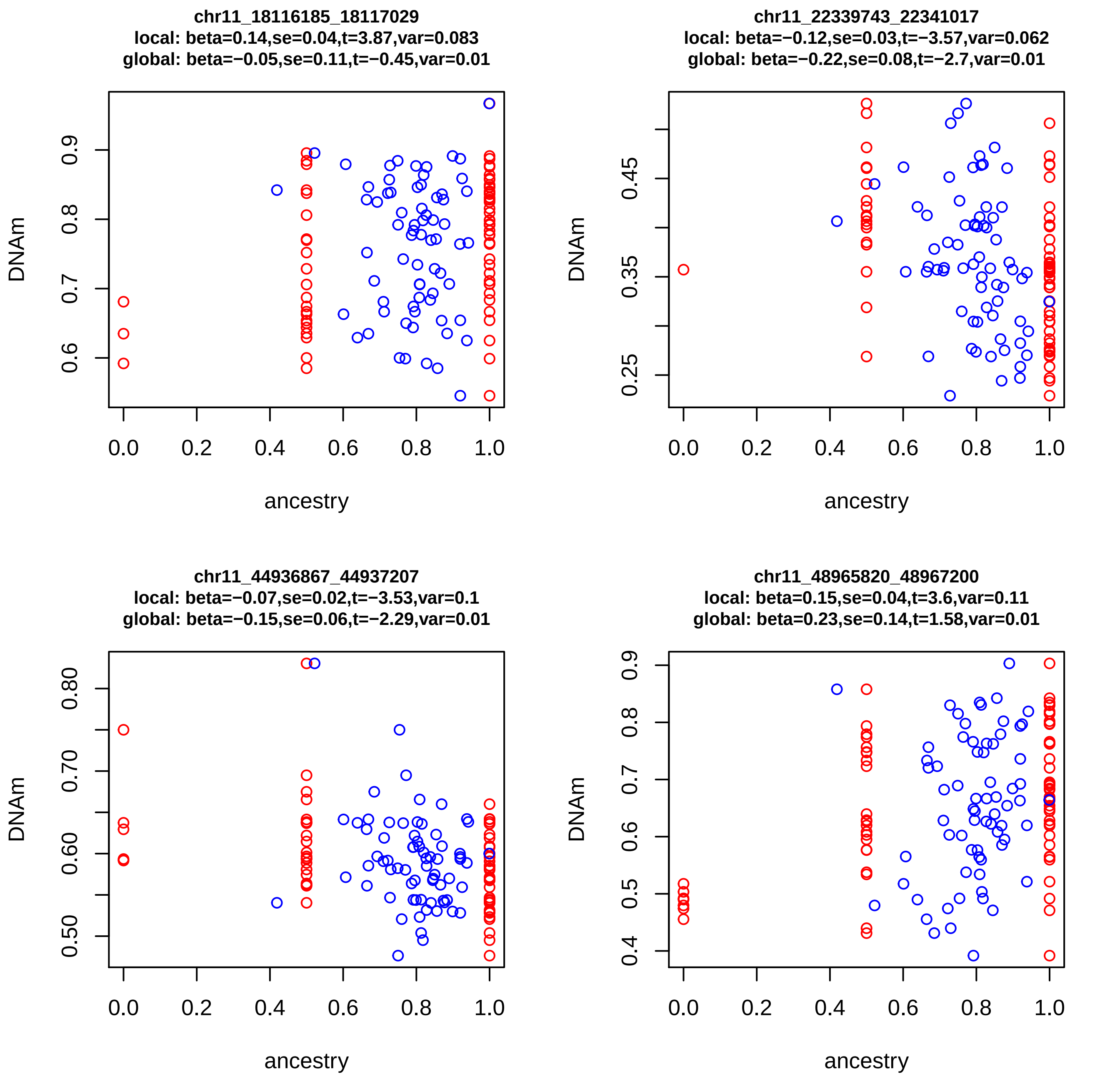
chr11_18116185_18117029
local: beta=0.14,se=0.04,t=3.87,var=0.083
global: beta=−0.05,se=0.11,t=−0.45,var=0.01
DNAm
0.6
0.7
0.8
0.9
0.0
0.2
0.4
0.6
0.8
1.0
ancestry
chr11_22339743_22341017
local: beta=−0.12,se=0.03,t=−3.57,var=0.062
global: beta=−0.22,se=0.08,t=−2.7,var=0.01
DNAm
0.25
0.35
0.45
0.0
0.2
0.4
0.6
0.8
1.0
ancestry
chr11_44936867_44937207
local: beta=−0.07,se=0.02,t=−3.53,var=0.1
global: beta=−0.15,se=0.06,t=−2.29,var=0.01
DNAm
0.50
0.60
0.70
0.80
0.0
0.2
0.4
0.6
0.8
1.0
ancestry
chr11_48965820_48967200
local: beta=0.15,se=0.04,t=3.6,var=0.11
global: beta=0.23,se=0.14,t=1.58,var=0.01
DNAm
0.4
0.5
0.6
0.7
0.8
0.9
0.0
0.2
0.4
0.6
0.8
1.0
ancestry

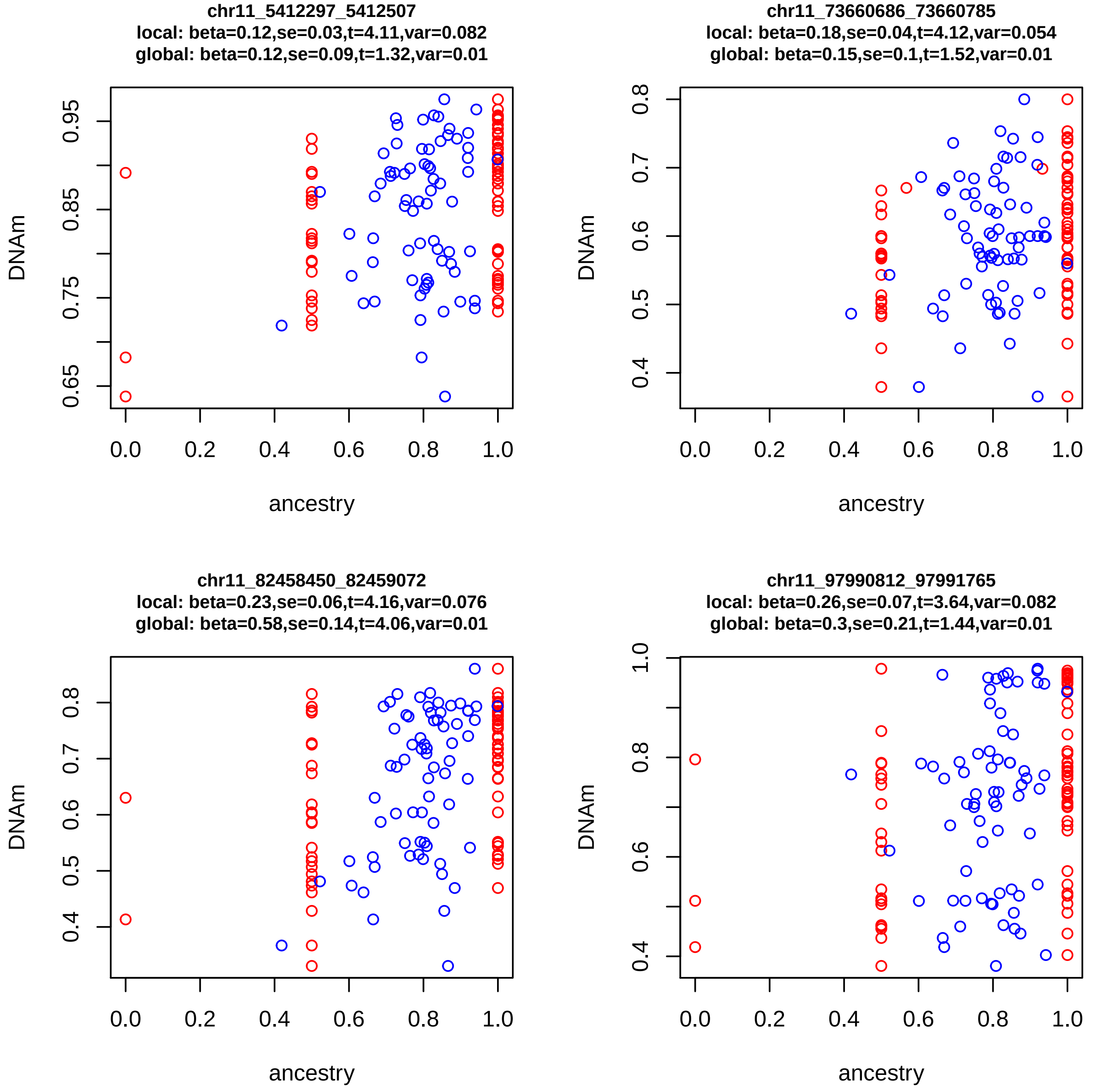
chr11_5412297_5412507
local: beta=0.12,se=0.03,t=4.11,var=0.082
global: beta=0.12,se=0.09,t=1.32,var=0.01
DNAm
ancestry
0.65
0.75
0.85
0.95
0.0
0.2
0.4
0.6
0.8
1.0
chr11_73660686_73660785
local: beta=0.18,se=0.04,t=4.12,var=0.054
global: beta=0.15,se=0.1,t=1.52,var=0.01
DNAm
ancestry
0.4
0.5
0.6
0.7
0.8
0.0
0.2
0.4
0.6
0.8
1.0
chr11_82458450_82459072
local: beta=0.23,se=0.06,t=4.16,var=0.076
global: beta=0.58,se=0.14,t=4.06,var=0.01
DNAm
ancestry
0.4
0.5
0.6
0.7
0.8
0.0
0.2
0.4
0.6
0.8
1.0
chr11_97990812_97991765
local: beta=0.26,se=0.07,t=3.64,var=0.082
global: beta=0.3,se=0.21,t=1.44,var=0.01
DNAm
ancestry
0.4
0.6
0.8
1.0
0.0
0.2
0.4
0.6
0.8
1.0

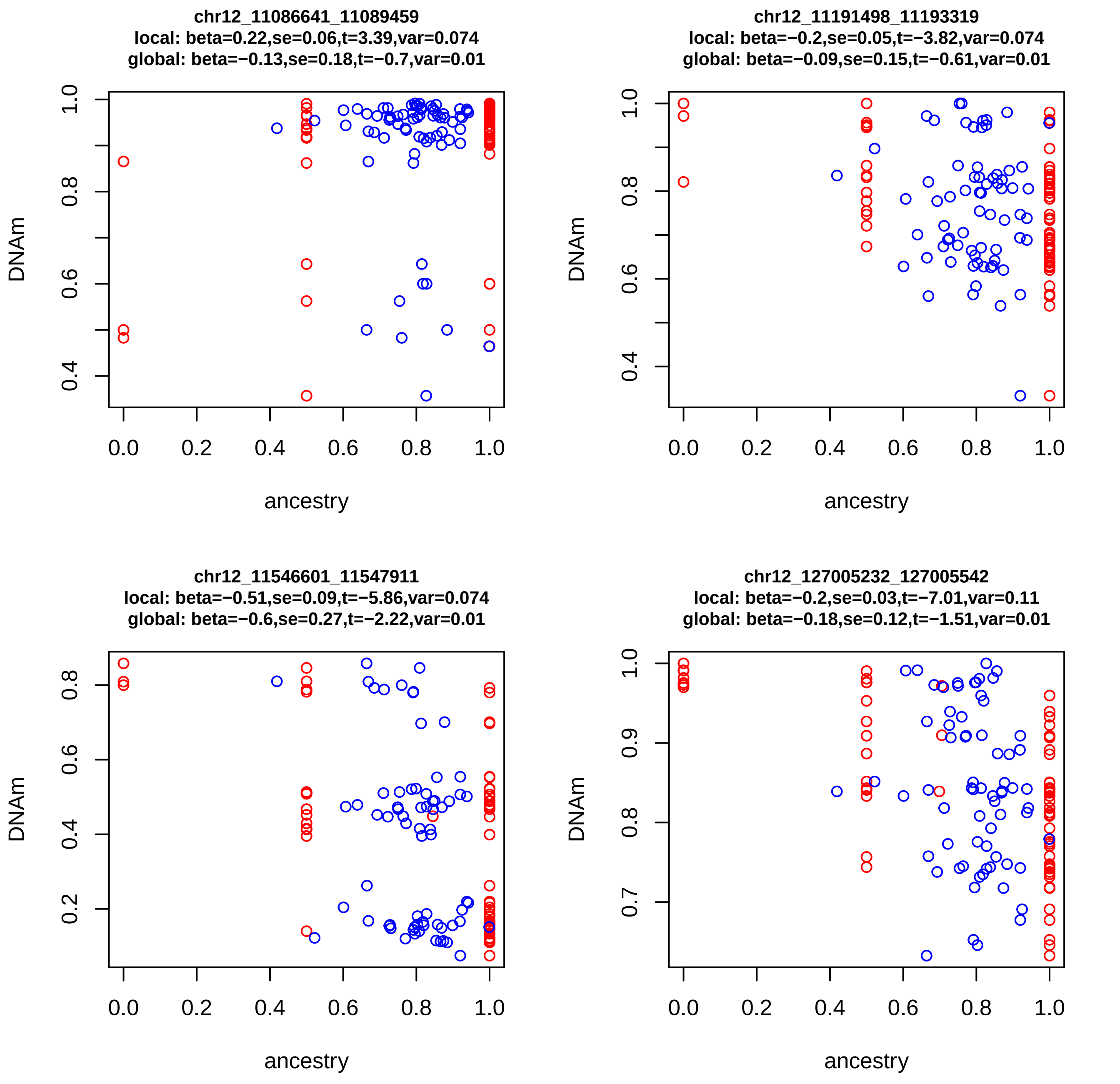
chr12_11086641_11089459
local: beta=0.22,se=0.06,t=3.39,var=0.074
global: beta=−0.13,se=0.18,t=−0.7,var=0.01
DNAm
ancestry
chr12_11191498_11193319
local: beta=−0.2,se=0.05,t=−3.82,var=0.074
global: beta=−0.09,se=0.15,t=−0.61,var=0.01
DNAm
ancestry
chr12_11546601_11547911
local: beta=−0.51,se=0.09,t=−5.86,var=0.074
global: beta=−0.6,se=0.27,t=−2.22,var=0.01
DNAm
ancestry
chr12_127005232_127005542
local: beta=−0.2,se=0.03,t=−7.01,var=0.11
global: beta=−0.18,se=0.12,t=−1.51,var=0.01
DNAm
ancestry

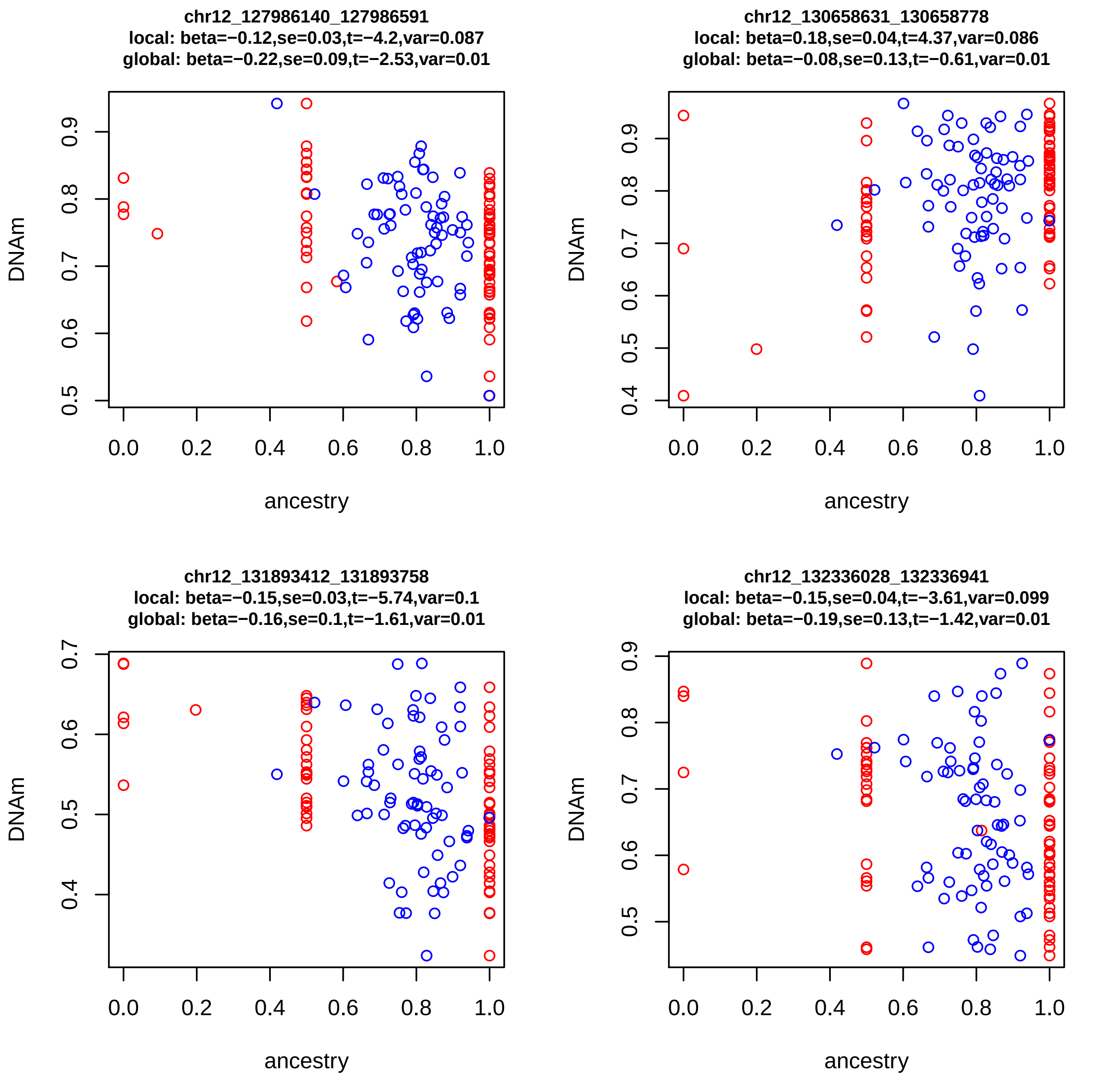
chr12_127986140_127986591
local: beta=−0.12,se=0.03,t=−4.2,var=0.087
global: beta=−0.22,se=0.09,t=−2.53,var=0.01
DNAm
ancestry
chr12_130658631_130658778
local: beta=0.18,se=0.04,t=4.37,var=0.086
global: beta=−0.08,se=0.13,t=−0.61,var=0.01
DNAm
ancestry
chr12_131893412_131893758
local: beta=−0.15,se=0.03,t=−5.74,var=0.1
global: beta=−0.16,se=0.1,t=−1.61,var=0.01
DNAm
ancestry
chr12_132336028_132336941
local: beta=−0.15,se=0.04,t=−3.61,var=0.099
global: beta=−0.19,se=0.13,t=−1.42,var=0.01
DNAm
ancestry

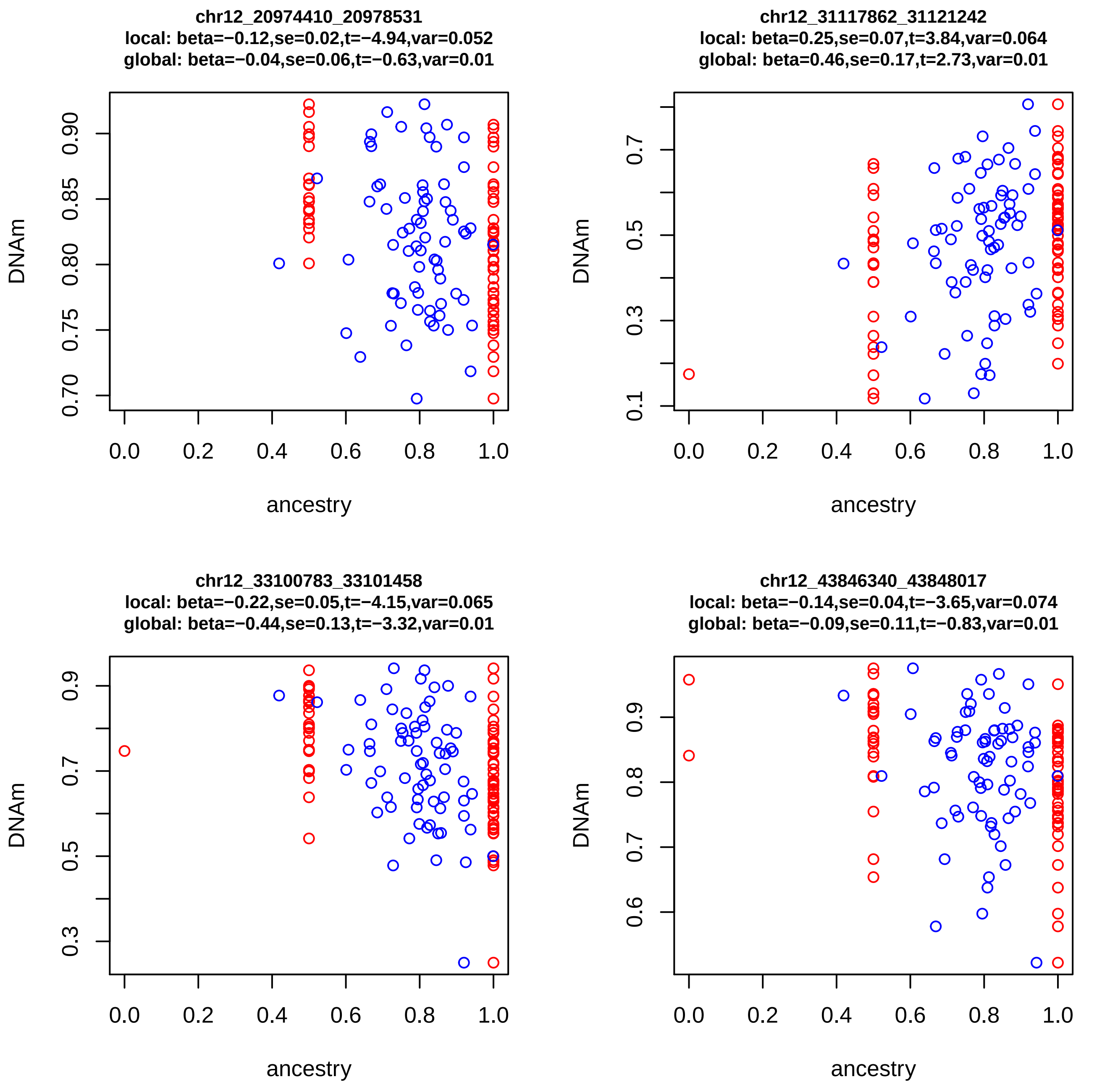

chr12_20974410_20978531
local: beta=−0.12,se=0.02,t=−4.94,var=0.052
global: beta=−0.04,se=0.06,t=−0.63,var=0.01
DNAm
ancestry
chr12_31117862_31121242
local: beta=0.25,se=0.07,t=3.84,var=0.064
global: beta=0.46,se=0.17,t=2.73,var=0.01
DNAm
ancestry
chr12_33100783_33101458
local: beta=−0.22,se=0.05,t=−4.15,var=0.065
global: beta=−0.44,se=0.13,t=−3.32,var=0.01
DNAm
ancestry
chr12_43846340_43848017
local: beta=−0.14,se=0.04,t=−3.65,var=0.074
global: beta=−0.09,se=0.11,t=−0.83,var=0.01
DNAm
ancestry

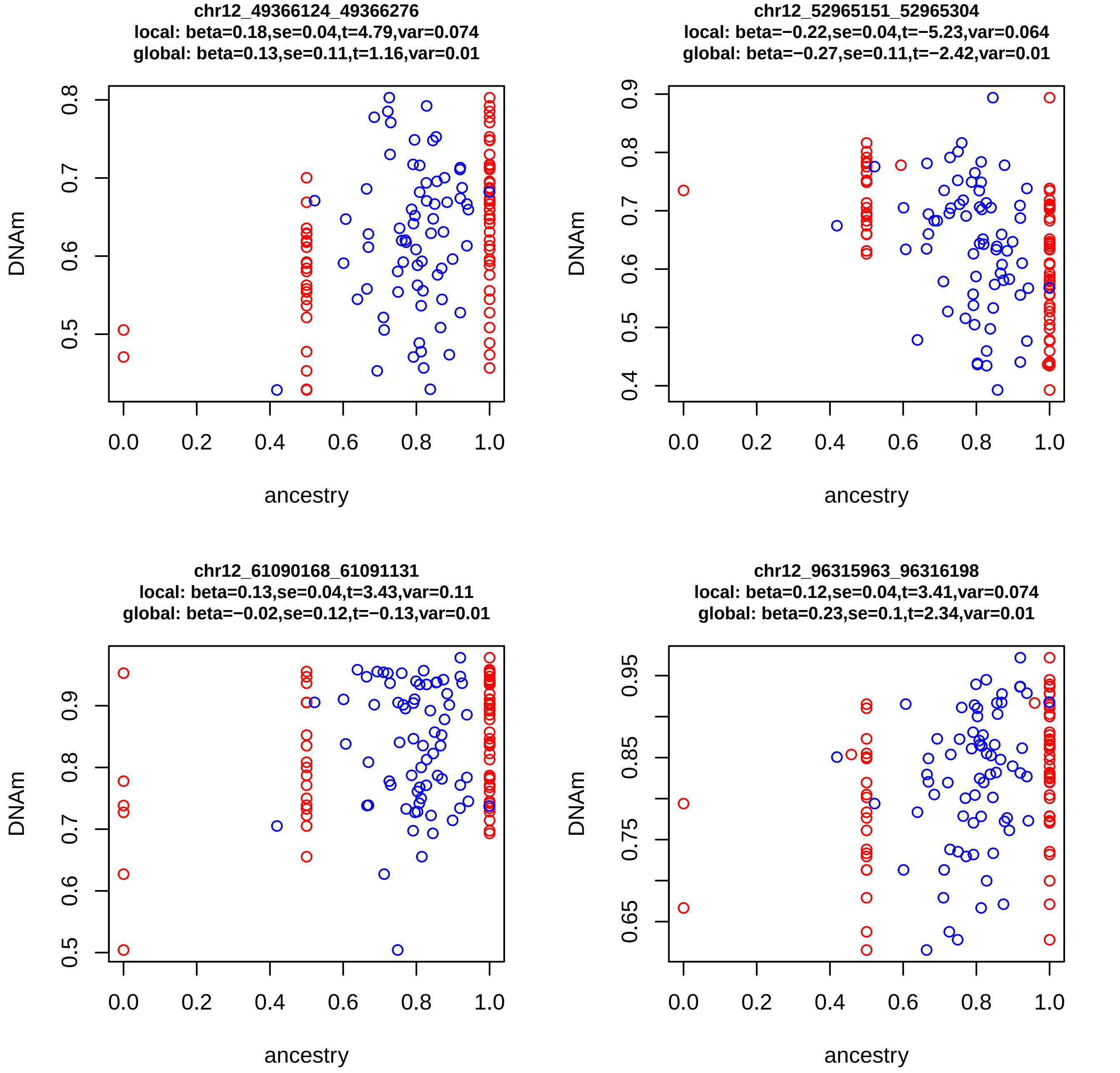
chr12_49366124_49366276
local: beta=0.18,se=0.04,t=4.79,var=0.074
global: beta=0.13,se=0.11,t=1.16,var=0.01
DNAm
ancestry
chr12_52965151_52965304
local: beta=−0.22,se=0.04,t=−5.23,var=0.064
global: beta=−0.27,se=0.11,t=−2.42,var=0.01
DNAm
ancestry
chr12_61090168_61091131
local: beta=0.13,se=0.04,t=3.43,var=0.11
global: beta=−0.02,se=0.12,t=−0.13,var=0.01
DNAm
ancestry
chr12_96315963_96316198
local: beta=0.12,se=0.04,t=3.41,var=0.074
global: beta=0.23,se=0.1,t=2.34,var=0.01
DNAm
ancestry

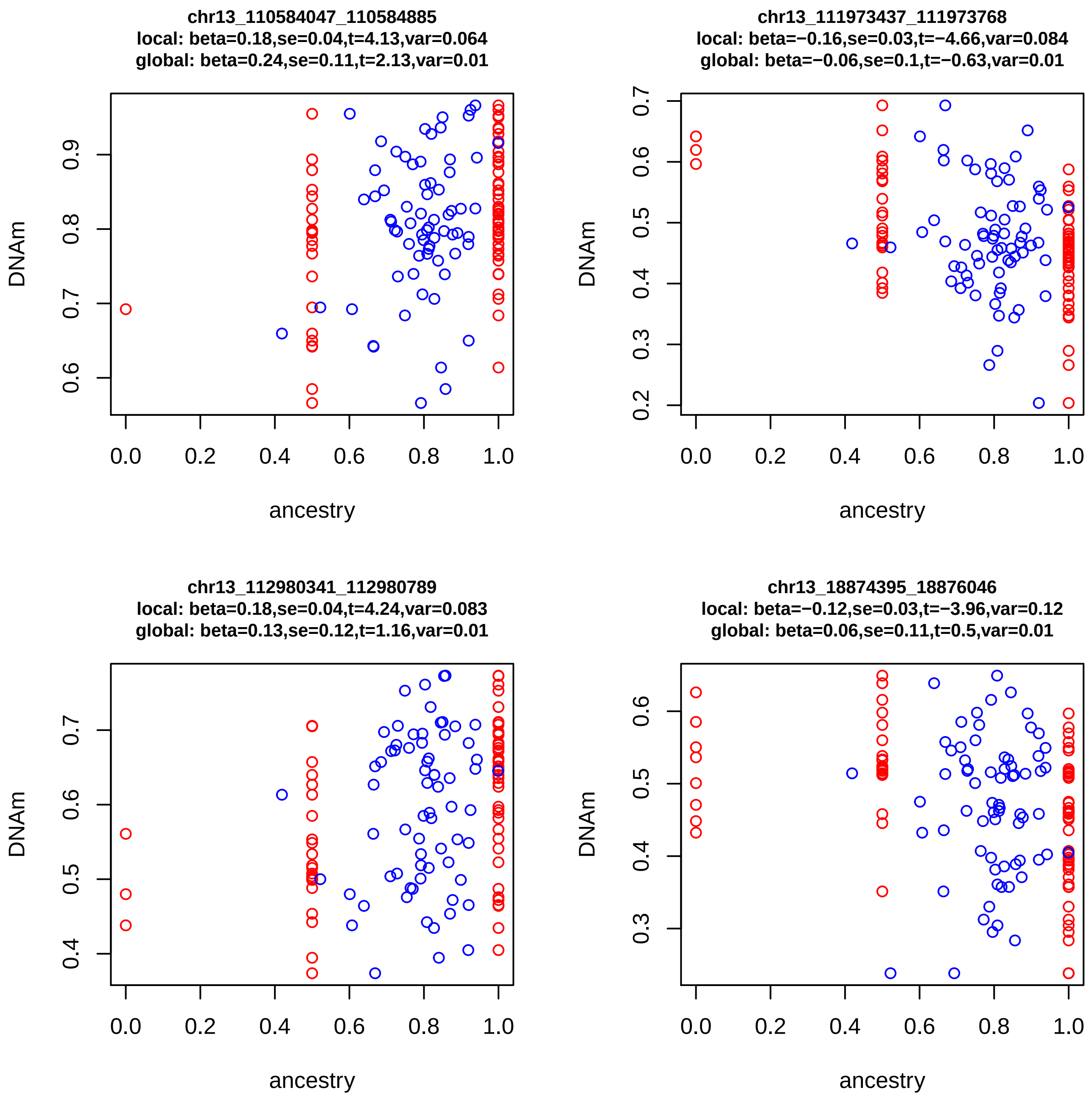
chr13_110584047_110584885
local: beta=0.18,se=0.04,t=4.13,var=0.064
global: beta=0.24,se=0.11,t=2.13,var=0.01
DNAm
0.6
0.7
0.8
0.9
0.0
0.2
0.4
0.6
0.8
1.0
ancestry
chr13_111973437_111973768
local: beta=−0.16,se=0.03,t=−4.66,var=0.084
global: beta=−0.06,se=0.1,t=−0.63,var=0.01
DNAm
0.2
0.3
0.4
0.5
0.6
0.7
0.0
0.2
0.4
0.6
0.8
1.0
ancestry
chr13_112980341_112980789
local: beta=0.18,se=0.04,t=4.24,var=0.083
global: beta=0.13,se=0.12,t=1.16,var=0.01
DNAm
0.4
0.5
0.6
0.7
0.0
0.2
0.4
0.6
0.8
1.0
ancestry
chr13_18874395_18876046
local: beta=−0.12,se=0.03,t=−3.96,var=0.12
global: beta=0.06,se=0.11,t=0.5,var=0.01
DNAm
0.3
0.4
0.5
0.6
0.0
0.2
0.4
0.6
0.8
1.0
ancestry

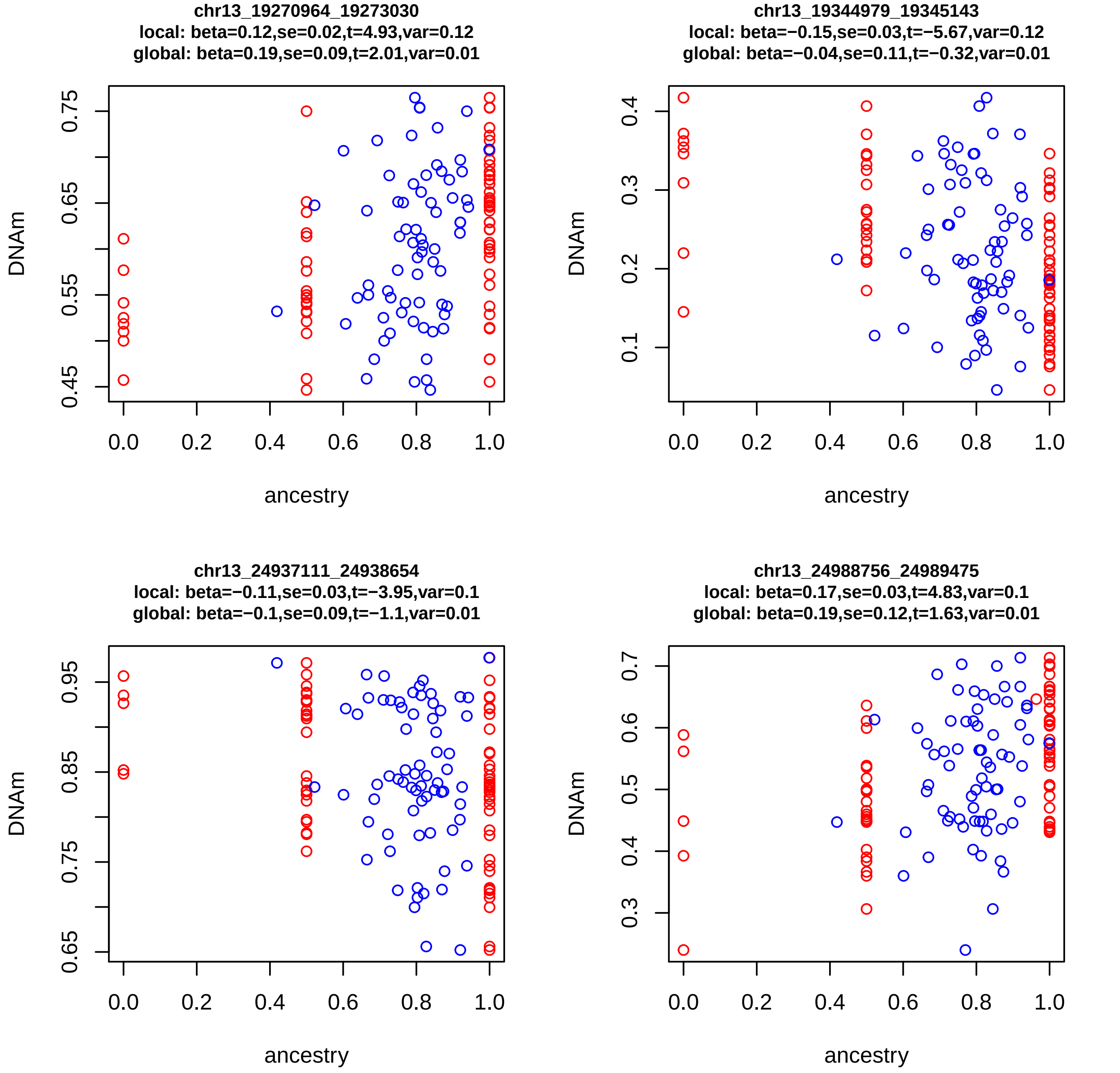
chr13_19270964_19273030
local: beta=0.12,se=0.02,t=4.93,var=0.12
global: beta=0.19,se=0.09,t=2.01,var=0.01
DNAm
0.45
0.55
0.65
0.75
0.0
0.2
0.4
0.6
0.8
1.0
ancestry
chr13_19344979_19345143
local: beta=−0.15,se=0.03,t=−5.67,var=0.12
global: beta=−0.04,se=0.11,t=−0.32,var=0.01
DNAm
0.1
0.2
0.3
0.4
0.0
0.2
0.4
0.6
0.8
1.0
ancestry
chr13_24937111_24938654
local: beta=−0.11,se=0.03,t=−3.95,var=0.1
global: beta=−0.1,se=0.09,t=−1.1,var=0.01
DNAm
0.65
0.75
0.85
0.95
0.0
0.2
0.4
0.6
0.8
1.0
ancestry
chr13_24988756_24989475
local: beta=0.17,se=0.03,t=4.83,var=0.1
global: beta=0.19,se=0.12,t=1.63,var=0.01
DNAm
0.3
0.4
0.5
0.6
0.7
0.0
0.2
0.4
0.6
0.8
1.0
ancestry

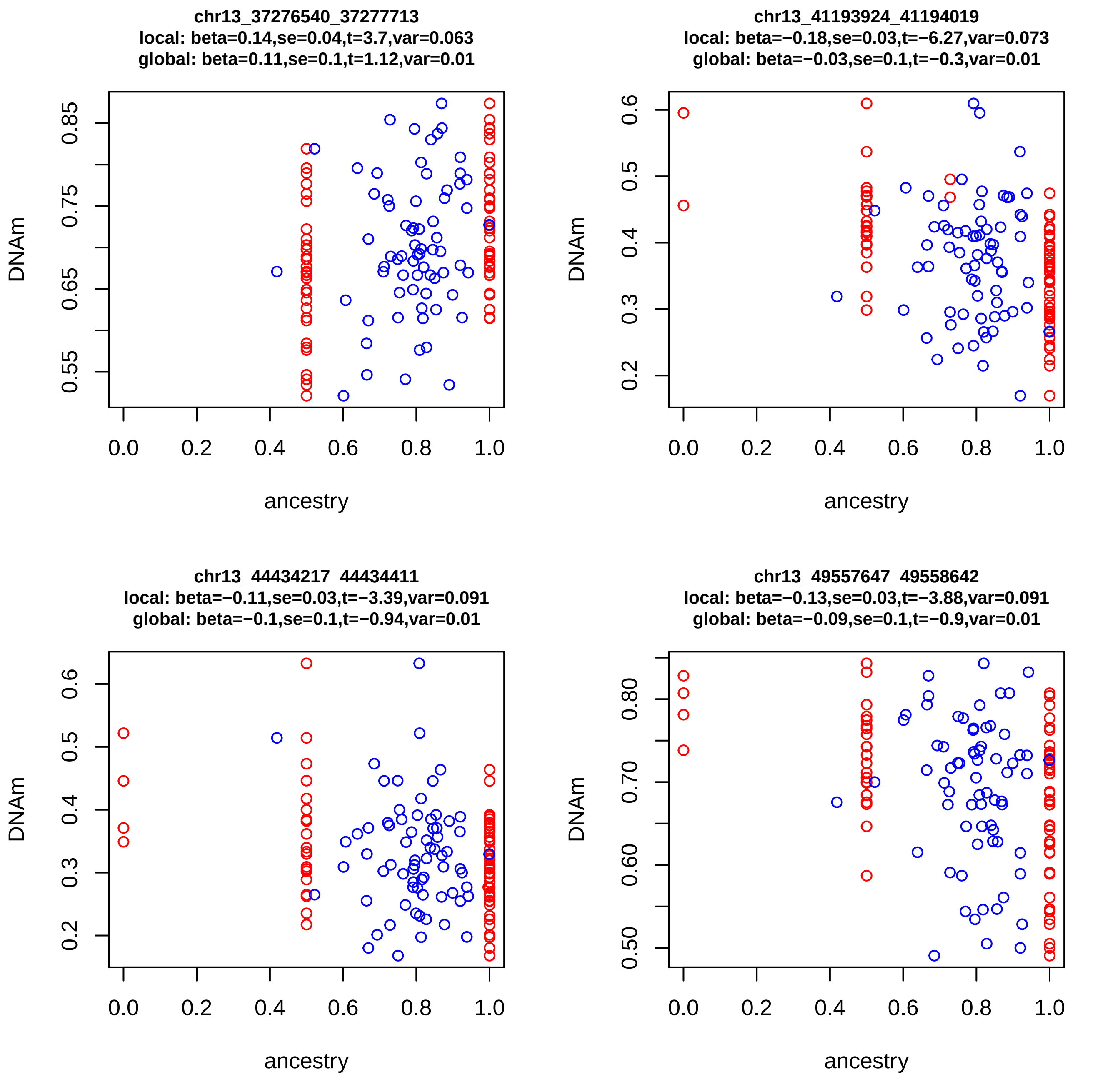
chr13_37276540_37277713
local: beta=0.14,se=0.04,t=3.7,var=0.063
global: beta=0.11,se=0.1,t=1.12,var=0.01
DNAm
ancestry
chr13_41193924_41194019
local: beta=−0.18,se=0.03,t=−6.27,var=0.073
global: beta=−0.03,se=0.1,t=−0.3,var=0.01
DNAm
ancestry
chr13_44434217_44434411
local: beta=−0.11,se=0.03,t=−3.39,var=0.091
global: beta=−0.1,se=0.1,t=−0.94,var=0.01
DNAm
ancestry
chr13_49557647_49558642
local: beta=−0.13,se=0.03,t=−3.88,var=0.091
global: beta=−0.09,se=0.1,t=−0.9,var=0.01
DNAm
ancestry

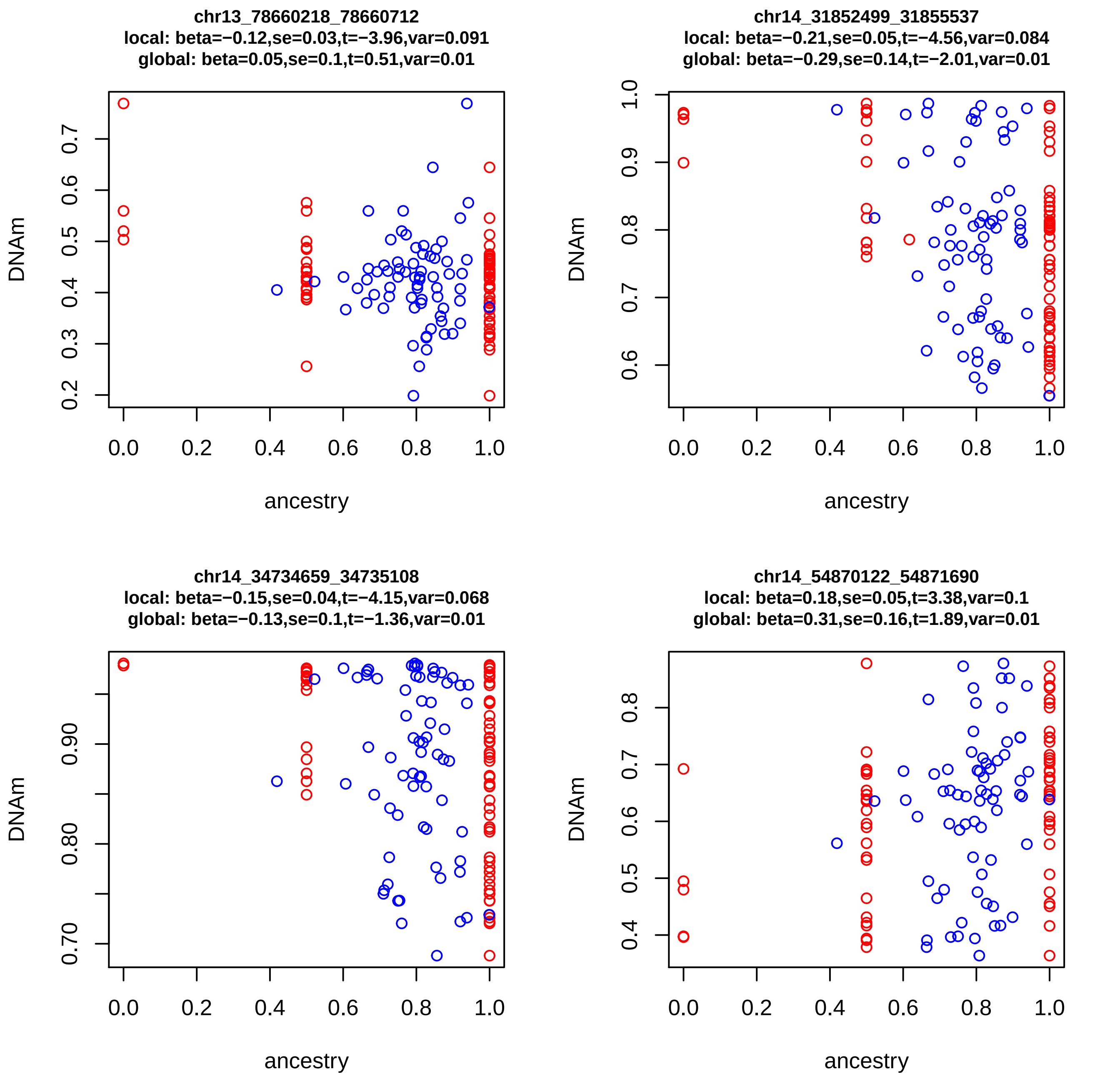
chr13_78660218_78660712
local: beta=−0.12,se=0.03,t=−3.96,var=0.091
global: beta=0.05,se=0.1,t=0.51,var=0.01
chr14_31852499_31855537
local: beta=−0.21,se=0.05,t=−4.56,var=0.084
global: beta=−0.29,se=0.14,t=−2.01,var=0.01
chr14_34734659_34735108
local: beta=−0.15,se=0.04,t=−4.15,var=0.068
global: beta=−0.13,se=0.1,t=−1.36,var=0.01
chr14_54870122_54871690
local: beta=0.18,se=0.05,t=3.38,var=0.1
global: beta=0.31,se=0.16,t=1.89,var=0.01
DNAm
ancestry
0.0
0.2
0.4
0.6
0.8
1.0

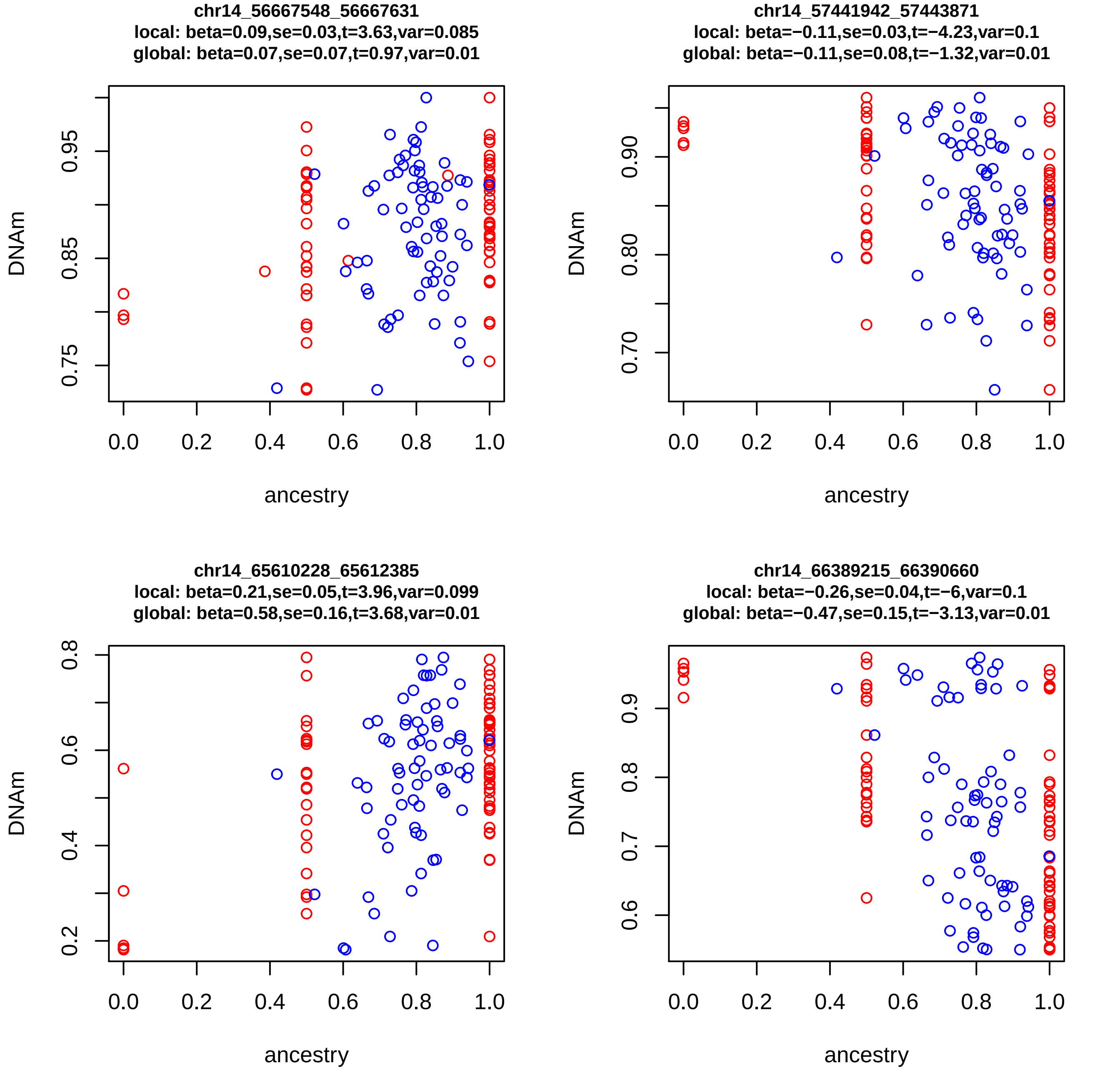
chr14_56667548_56667631
local: beta=0.09,se=0.03,t=3.63,var=0.085
global: beta=0.07,se=0.07,t=0.97,var=0.01
DNAm
ancestry
chr14_57441942_57443871
local: beta=−0.11,se=0.03,t=−4.23,var=0.1
global: beta=−0.11,se=0.08,t=−1.32,var=0.01
DNAm
ancestry
chr14_65610228_65612385
local: beta=0.21,se=0.05,t=3.96,var=0.099
global: beta=0.58,se=0.16,t=3.68,var=0.01
DNAm
ancestry
chr14_66389215_66390660
local: beta=−0.26,se=0.04,t=−6,var=0.1
global: beta=−0.47,se=0.15,t=−3.13,var=0.01
DNAm
ancestry

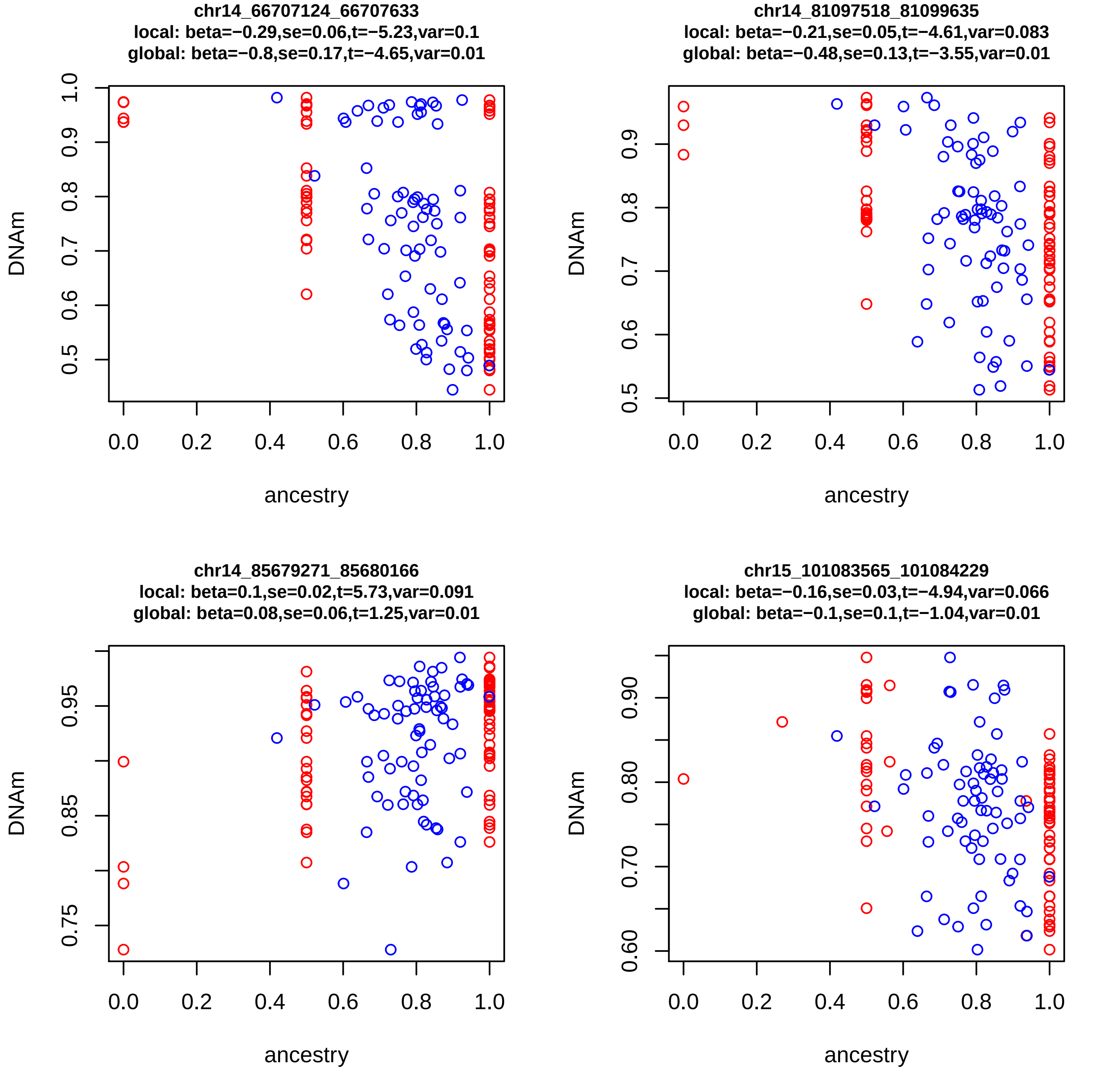

chr14_66707124_66707633
local: beta=−0.29,se=0.06,t=−5.23,var=0.1
global: beta=−0.8,se=0.17,t=−4.65,var=0.01
DNAm
ancestry
chr14_81097518_81099635
local: beta=−0.21,se=0.05,t=−4.61,var=0.083
global: beta=−0.48,se=0.13,t=−3.55,var=0.01
DNAm
ancestry
chr14_85679271_85680166
local: beta=0.1,se=0.02,t=5.73,var=0.091
global: beta=0.08,se=0.06,t=1.25,var=0.01
DNAm
ancestry
chr15_101083565_101084229
local: beta=−0.16,se=0.03,t=−4.94,var=0.066
global: beta=−0.1,se=0.1,t=−1.04,var=0.01
DNAm
ancestry

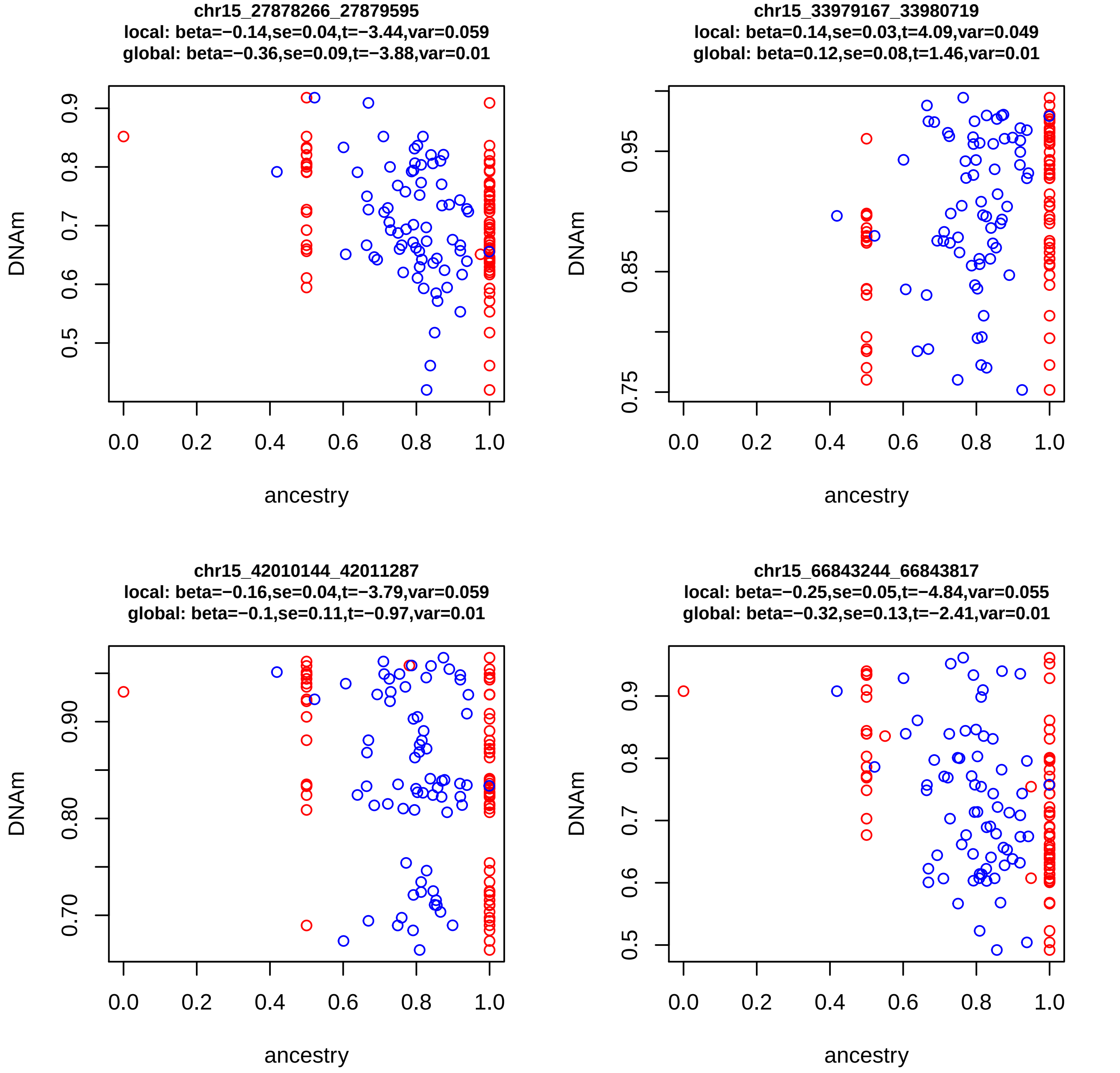

chr15_27878266_27879595
local: beta=−0.14,se=0.04,t=−3.44,var=0.059
global: beta=−0.36,se=0.09,t=−3.88,var=0.01
DNAm
0.5
0.6
0.7
0.8
0.9
0.0
0.2
0.4
0.6
0.8
1.0
ancestry
chr15_33979167_33980719
local: beta=0.14,se=0.03,t=4.09,var=0.049
global: beta=0.12,se=0.08,t=1.46,var=0.01
DNAm
0.75
0.85
0.95
0.0
0.2
0.4
0.6
0.8
1.0
ancestry
chr15_42010144_42011287
local: beta=−0.16,se=0.04,t=−3.79,var=0.059
global: beta=−0.1,se=0.11,t=−0.97,var=0.01
DNAm
0.70
0.80
0.90
0.0
0.2
0.4
0.6
0.8
1.0
ancestry
chr15_66843244_66843817
local: beta=−0.25,se=0.05,t=−4.84,var=0.055
global: beta=−0.32,se=0.13,t=−2.41,var=0.01
DNAm
0.5
0.6
0.7
0.8
0.9
0.0
0.2
0.4
0.6
0.8
1.0
ancestry

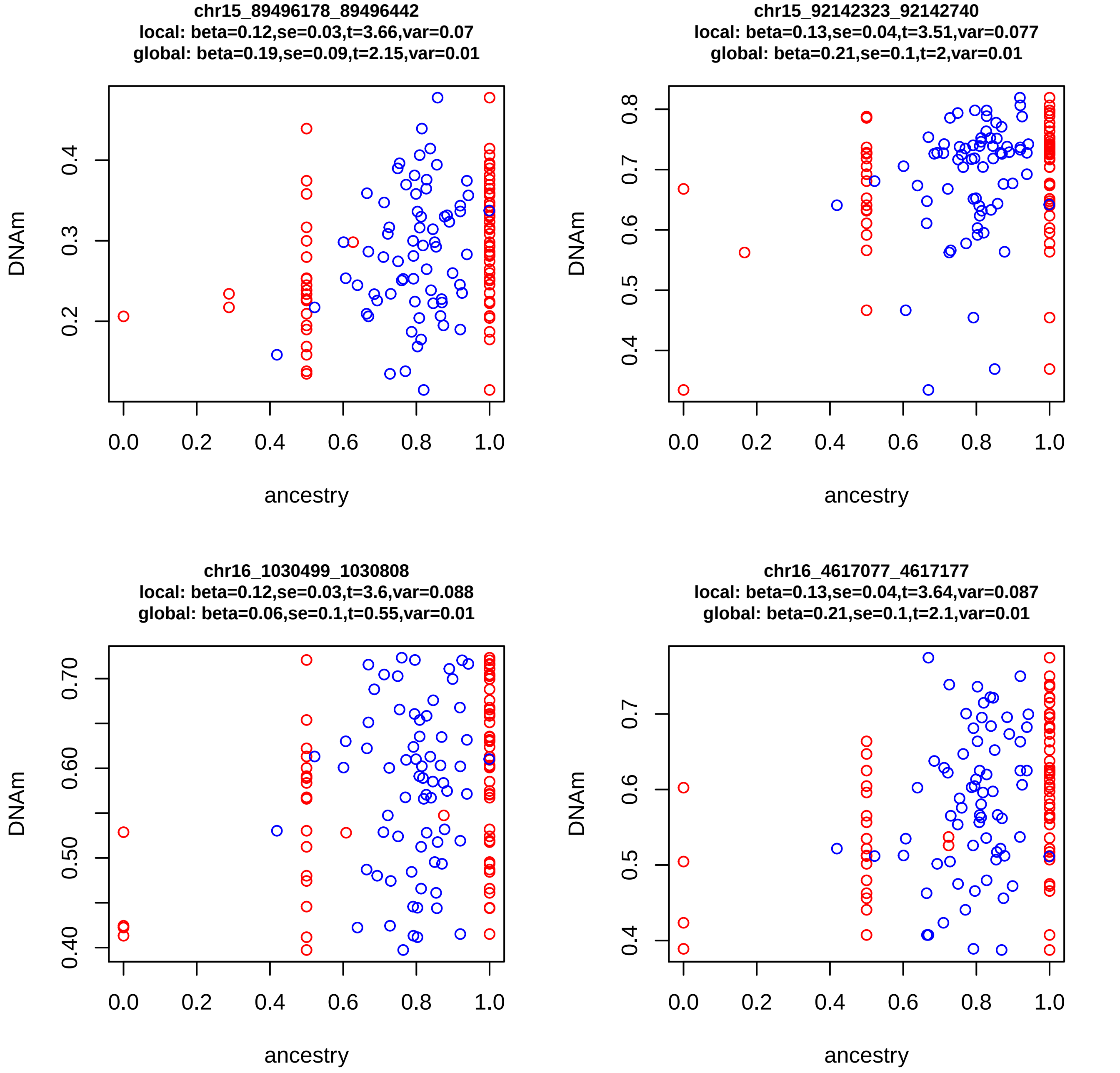
chr15_89496178_89496442
local: beta=0.12,se=0.03,t=3.66,var=0.07
global: beta=0.19,se=0.09,t=2.15,var=0.01
DNAm
0.2
0.3
0.4
0.0
0.2
0.4
0.6
0.8
1.0
ancestry
chr15_92142323_92142740
local: beta=0.13,se=0.04,t=3.51,var=0.077
global: beta=0.21,se=0.1,t=2,var=0.01
DNAm
0.4
0.5
0.6
0.7
0.8
0.0
0.2
0.4
0.6
0.8
1.0
ancestry
chr16_1030499_1030808
local: beta=0.12,se=0.03,t=3.6,var=0.088
global: beta=0.06,se=0.1,t=0.55,var=0.01
DNAm
0.40
0.50
0.60
0.70
0.0
0.2
0.4
0.6
0.8
1.0
ancestry
chr16_4617077_4617177
local: beta=0.13,se=0.04,t=3.64,var=0.087
global: beta=0.21,se=0.1,t=2.1,var=0.01
DNAm
0.4
0.5
0.6
0.7
0.0
0.2
0.4
0.6
0.8
1.0
ancestry

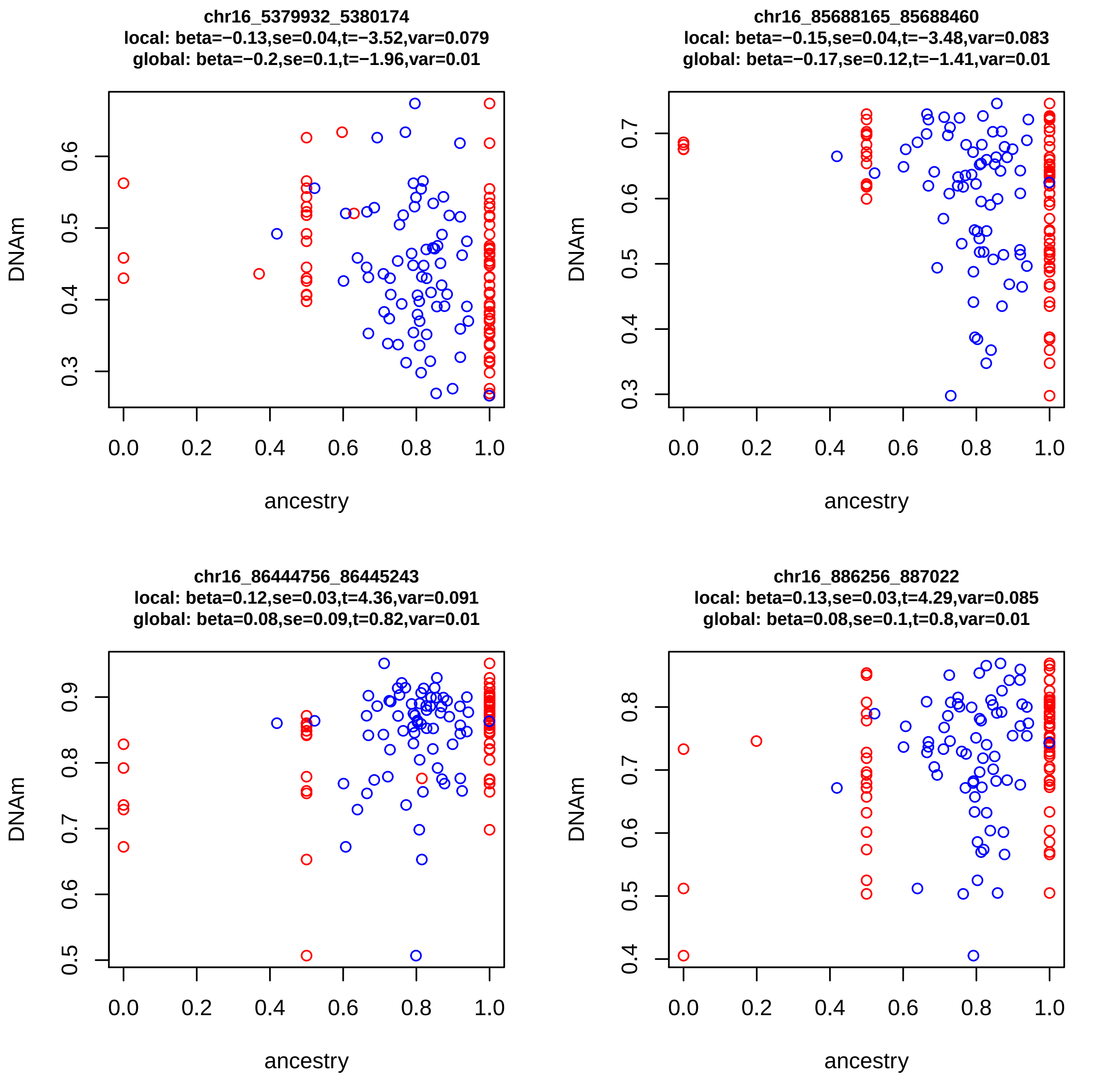
chr16_5379932_5380174
local: beta=−0.13,se=0.04,t=−3.52,var=0.079
global: beta=−0.2,se=0.1,t=−1.96,var=0.01
chr16_85688165_85688460
local: beta=−0.15,se=0.04,t=−3.48,var=0.083
global: beta=−0.17,se=0.12,t=−1.41,var=0.01
chr16_86444756_86445243
local: beta=0.12,se=0.03,t=4.36,var=0.091
global: beta=0.08,se=0.09,t=0.82,var=0.01
chr16_886256_887022
local: beta=0.13,se=0.03,t=4.29,var=0.085
global: beta=0.08,se=0.1,t=0.8,var=0.01
DNAm
ancestry

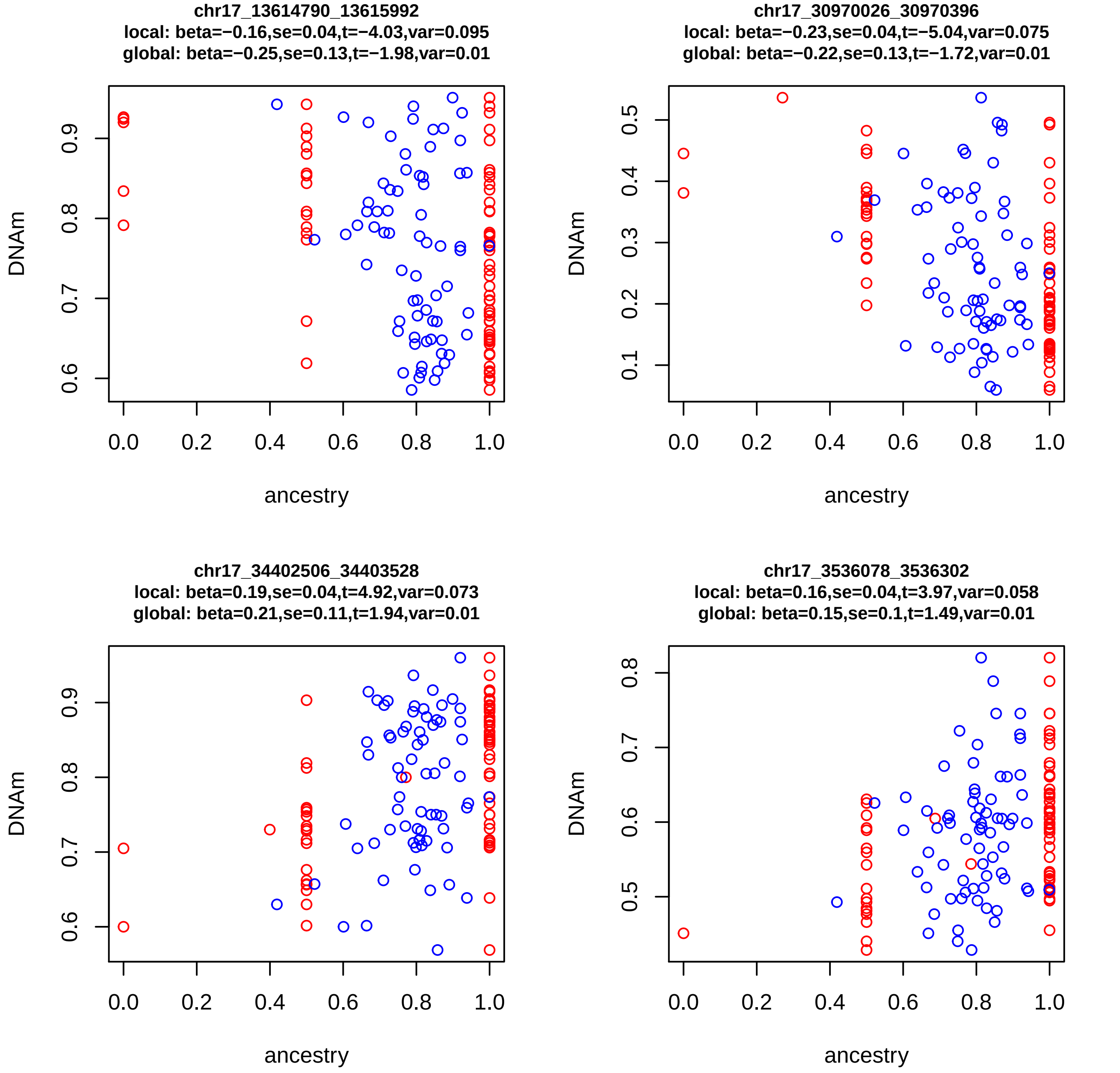
chr17_13614790_13615992
local: beta=−0.16,se=0.04,t=−4.03,var=0.095
global: beta=−0.25,se=0.13,t=−1.98,var=0.01
DNAm
ancestry
chr17_30970026_30970396
local: beta=−0.23,se=0.04,t=−5.04,var=0.075
global: beta=−0.22,se=0.13,t=−1.72,var=0.01
DNAm
ancestry
chr17_34402506_34403528
local: beta=0.19,se=0.04,t=4.92,var=0.073
global: beta=0.21,se=0.11,t=1.94,var=0.01
DNAm
ancestry
chr17_3536078_3536302
local: beta=0.16,se=0.04,t=3.97,var=0.058
global: beta=0.15,se=0.1,t=1.49,var=0.01
DNAm
ancestry

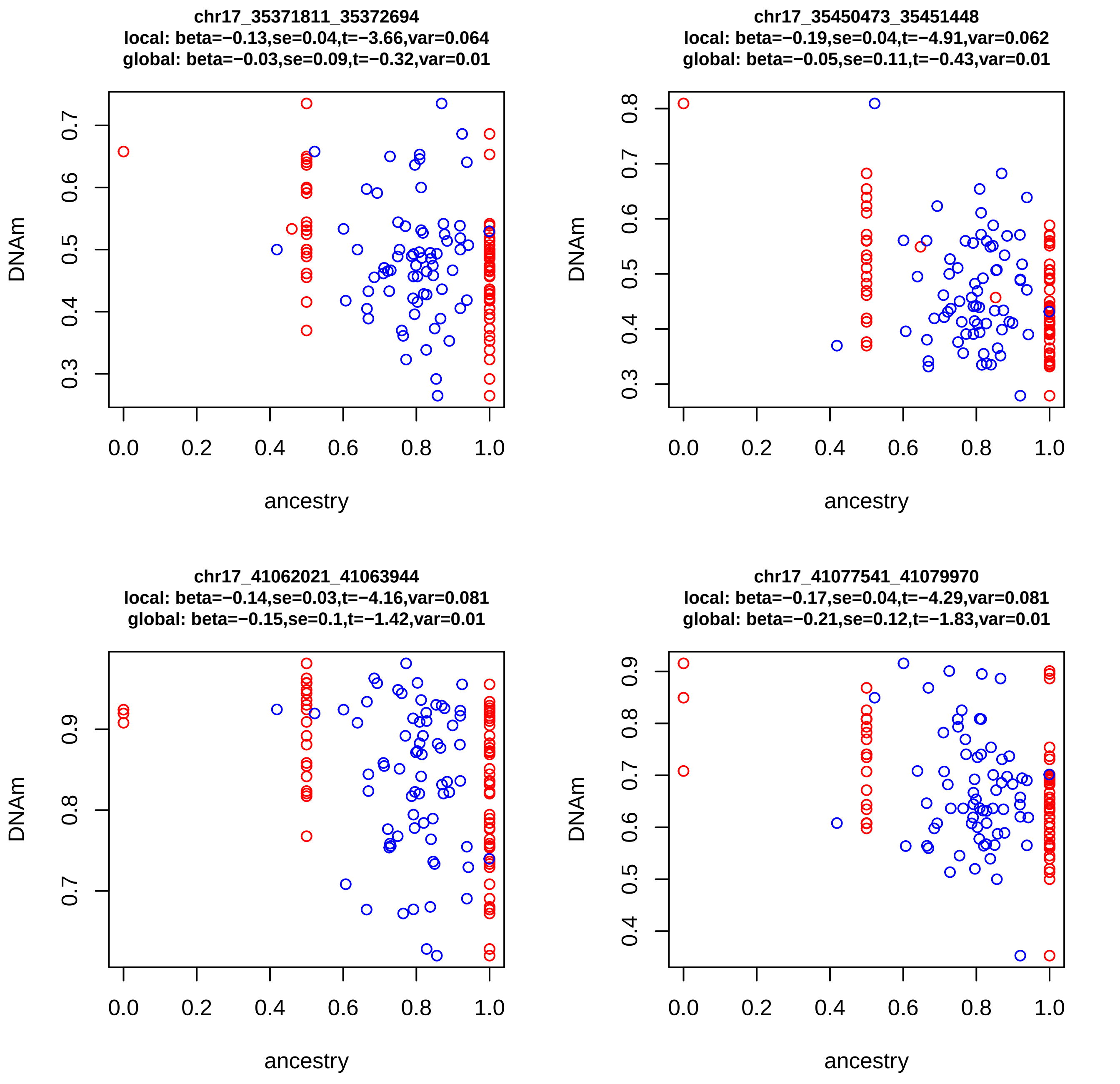

chr17_35371811_35372694
local: beta=−0.13,se=0.04,t=−3.66,var=0.064
global: beta=−0.03,se=0.09,t=−0.32,var=0.01
chr17_35450473_35451448
local: beta=−0.19,se=0.04,t=−4.91,var=0.062
global: beta=−0.05,se=0.11,t=−0.43,var=0.01
chr17_41062021_41063944
local: beta=−0.14,se=0.03,t=−4.16,var=0.081
global: beta=−0.15,se=0.1,t=−1.42,var=0.01
chr17_41077541_41079970
local: beta=−0.17,se=0.04,t=−4.29,var=0.081
global: beta=−0.21,se=0.12,t=−1.83,var=0.01
DNAm
ancestry

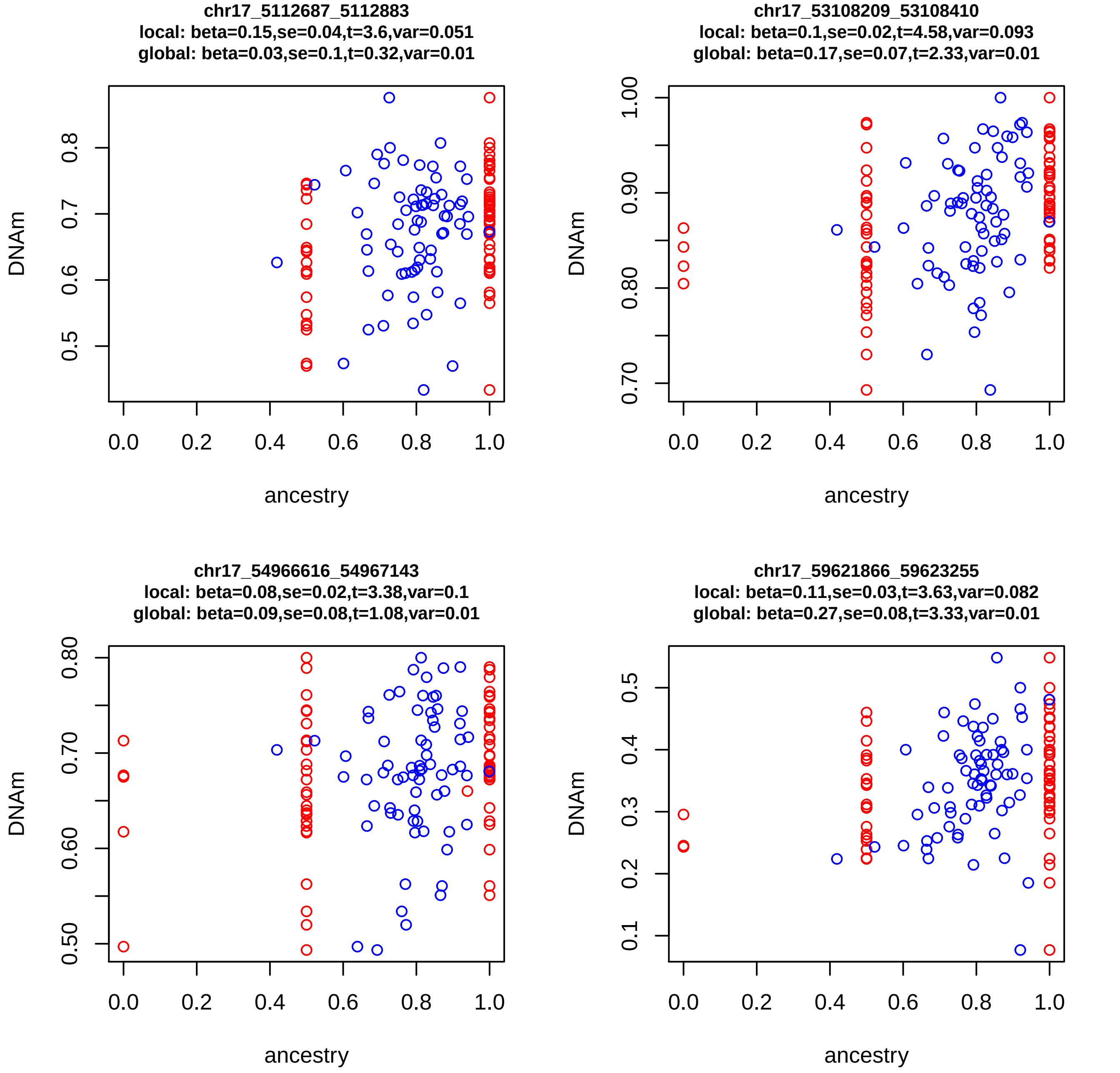
chr17_5112687_5112883
local: beta=0.15,se=0.04,t=3.6,var=0.051
global: beta=0.03,se=0.1,t=0.32,var=0.01
DNAm
ancestry
chr17_53108209_53108410
local: beta=0.1,se=0.02,t=4.58,var=0.093
global: beta=0.17,se=0.07,t=2.33,var=0.01
DNAm
ancestry
chr17_54966616_54967143
local: beta=0.08,se=0.02,t=3.38,var=0.1
global: beta=0.09,se=0.08,t=1.08,var=0.01
DNAm
ancestry
chr17_59621866_59623255
local: beta=0.11,se=0.03,t=3.63,var=0.082
global: beta=0.27,se=0.08,t=3.33,var=0.01
DNAm
ancestry

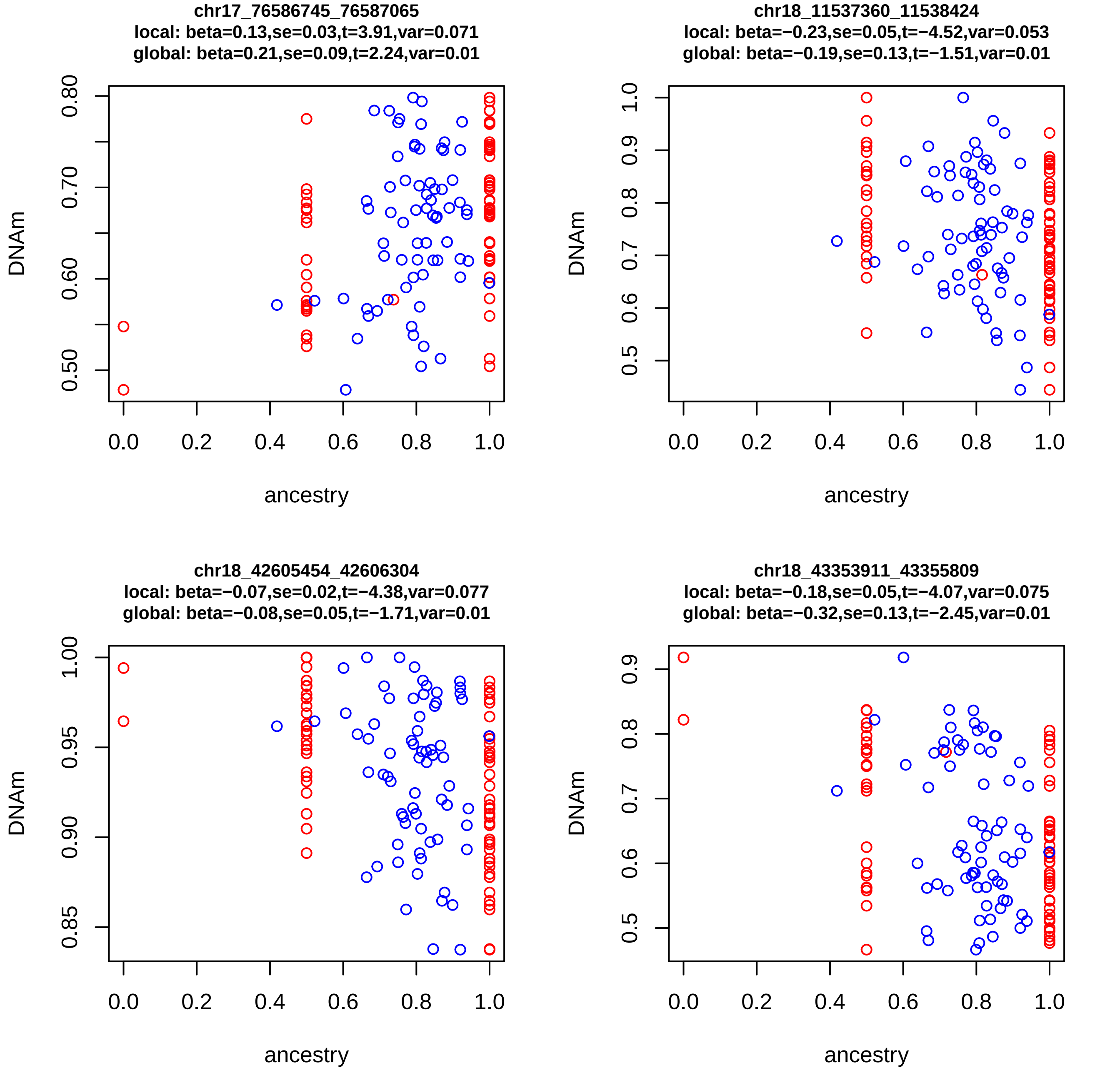
chr17_76586745_76587065
local: beta=0.13,se=0.03,t=3.91,var=0.071
global: beta=0.21,se=0.09,t=2.24,var=0.01
DNAm
ancestry
chr18_11537360_11538424
local: beta=−0.23,se=0.05,t=−4.52,var=0.053
global: beta=−0.19,se=0.13,t=−1.51,var=0.01
DNAm
ancestry
chr18_42605454_42606304
local: beta=−0.07,se=0.02,t=−4.38,var=0.077
global: beta=−0.08,se=0.05,t=−1.71,var=0.01
DNAm
ancestry
chr18_43353911_43355809
local: beta=−0.18,se=0.05,t=−4.07,var=0.075
global: beta=−0.32,se=0.13,t=−2.45,var=0.01
DNAm
ancestry

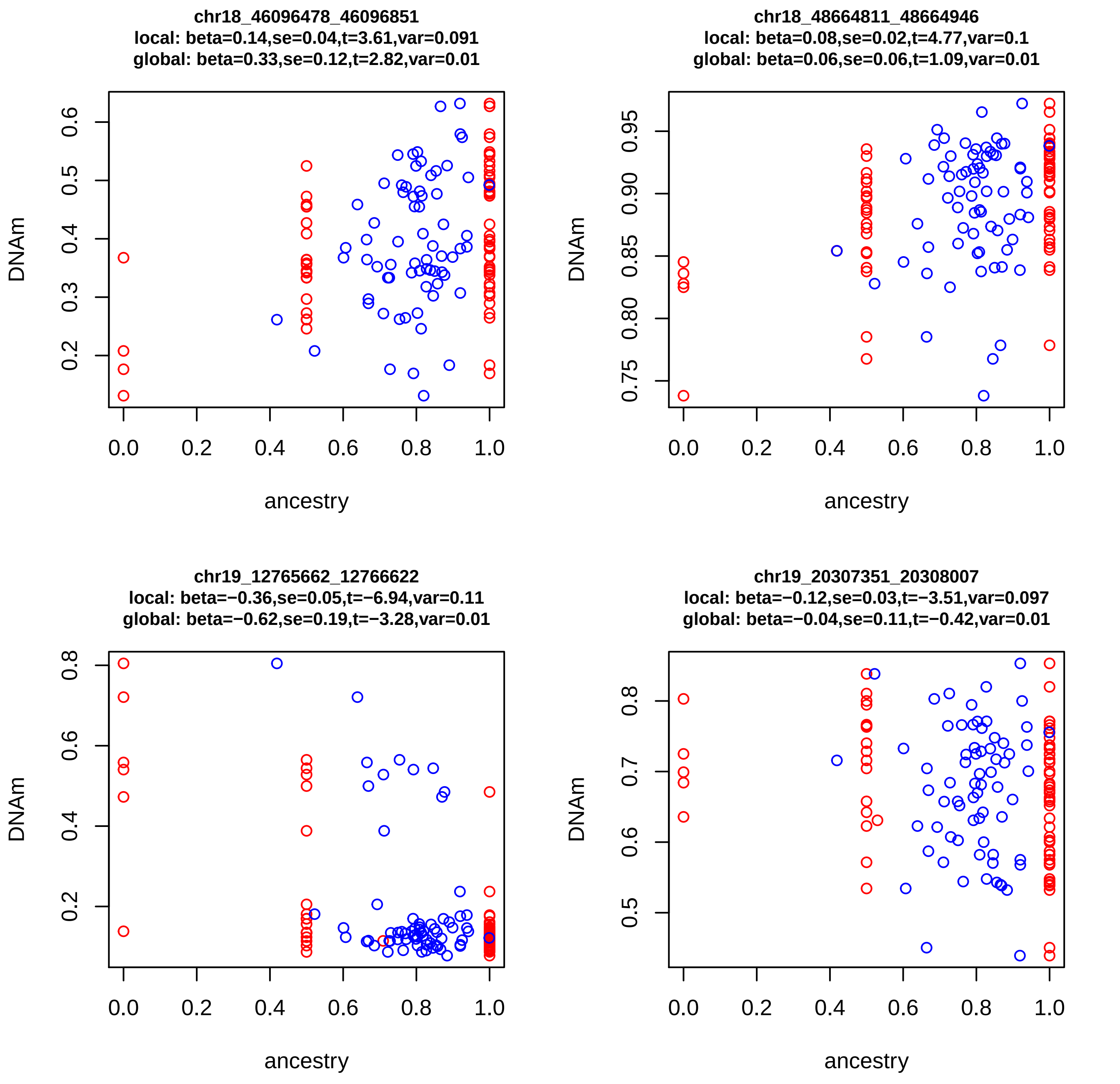
chr18_46096478_46096851
local: beta=0.14,se=0.04,t=3.61,var=0.091
global: beta=0.33,se=0.12,t=2.82,var=0.01
DNAm
ancestry
chr18_48664811_48664946
local: beta=0.08,se=0.02,t=4.77,var=0.1
global: beta=0.06,se=0.06,t=1.09,var=0.01
DNAm
ancestry
chr19_12765662_12766622
local: beta=−0.36,se=0.05,t=−6.94,var=0.11
global: beta=−0.62,se=0.19,t=−3.28,var=0.01
DNAm
ancestry
chr19_20307351_20308007
local: beta=−0.12,se=0.03,t=−3.51,var=0.097
global: beta=−0.04,se=0.11,t=−0.42,var=0.01
DNAm
ancestry

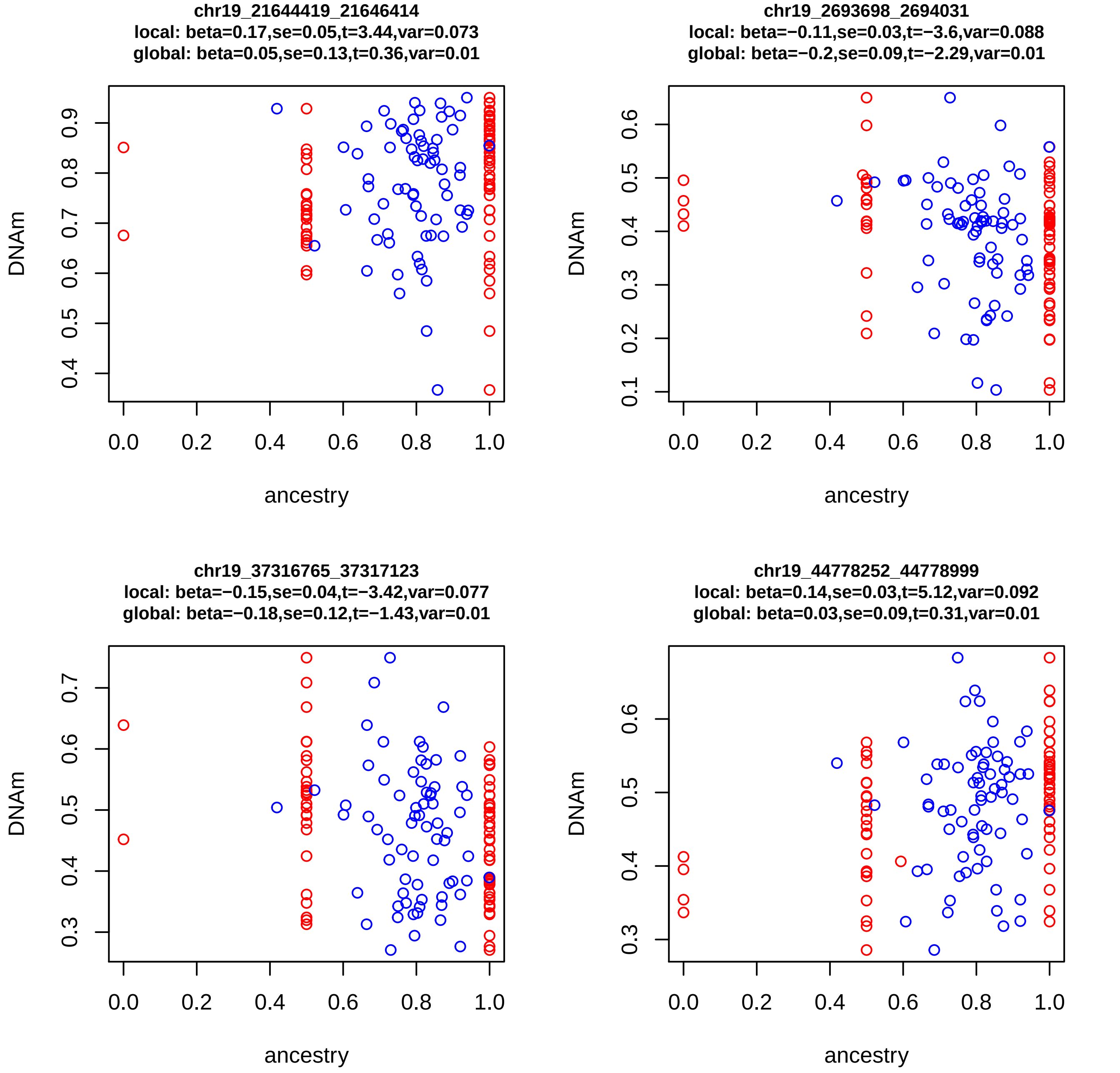
chr19_21644419_21646414
local: beta=0.17,se=0.05,t=3.44,var=0.073
global: beta=0.05,se=0.13,t=0.36,var=0.01
DNAm
0.4 0.5 0.6 0.7 0.8 0.9
ancestry
0.0 0.2 0.4 0.6 0.8 1.0
chr19_2693698_2694031
local: beta=−0.11,se=0.03,t=−3.6,var=0.088
global: beta=−0.2,se=0.09,t=−2.29,var=0.01
DNAm
0.1 0.2 0.3 0.4 0.5 0.6
ancestry
0.0 0.2 0.4 0.6 0.8 1.0
chr19_37316765_37317123
local: beta=−0.15,se=0.04,t=−3.42,var=0.077
global: beta=−0.18,se=0.12,t=−1.43,var=0.01
DNAm
0.3 0.4 0.5 0.6 0.7
ancestry
0.0 0.2 0.4 0.6 0.8 1.0
chr19_44778252_44778999
local: beta=0.14,se=0.03,t=5.12,var=0.092
global: beta=0.03,se=0.09,t=0.31,var=0.01
DNAm
0.3 0.4 0.5 0.6
ancestry
0.0 0.2 0.4 0.6 0.8 1.0

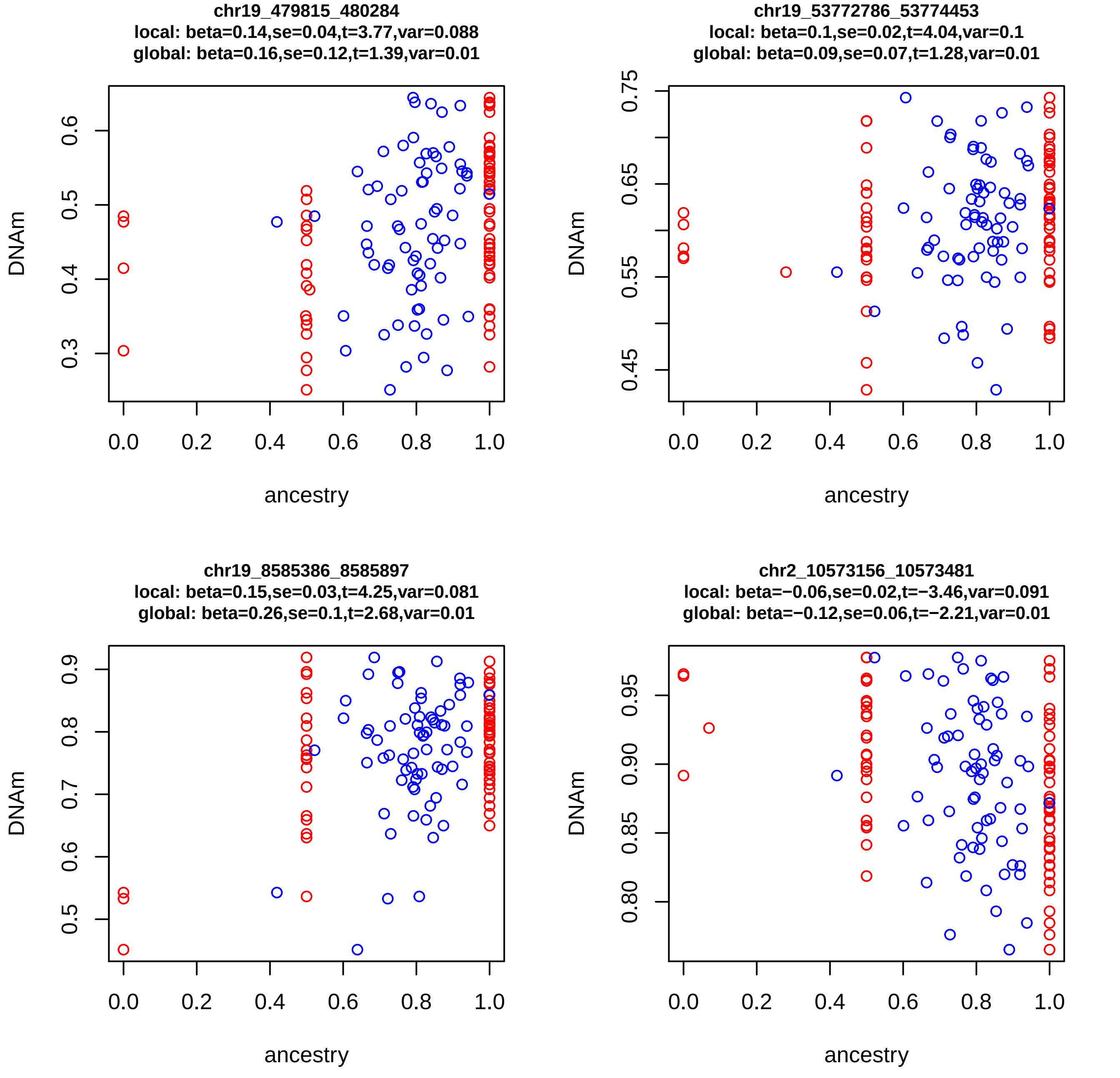
chr19_479815_480284
local: beta=0.14,se=0.04,t=3.77,var=0.088
global: beta=0.16,se=0.12,t=1.39,var=0.01
chr19_53772786_53774453
local: beta=0.1,se=0.02,t=4.04,var=0.1
global: beta=0.09,se=0.07,t=1.28,var=0.01
chr19_8585386_8585897
local: beta=0.15,se=0.03,t=4.25,var=0.081
global: beta=0.26,se=0.1,t=2.68,var=0.01
chr2_10573156_10573481
local: beta=−0.06,se=0.02,t=−3.46,var=0.091
global: beta=−0.12,se=0.06,t=−2.21,var=0.01
DNAm
ancestry

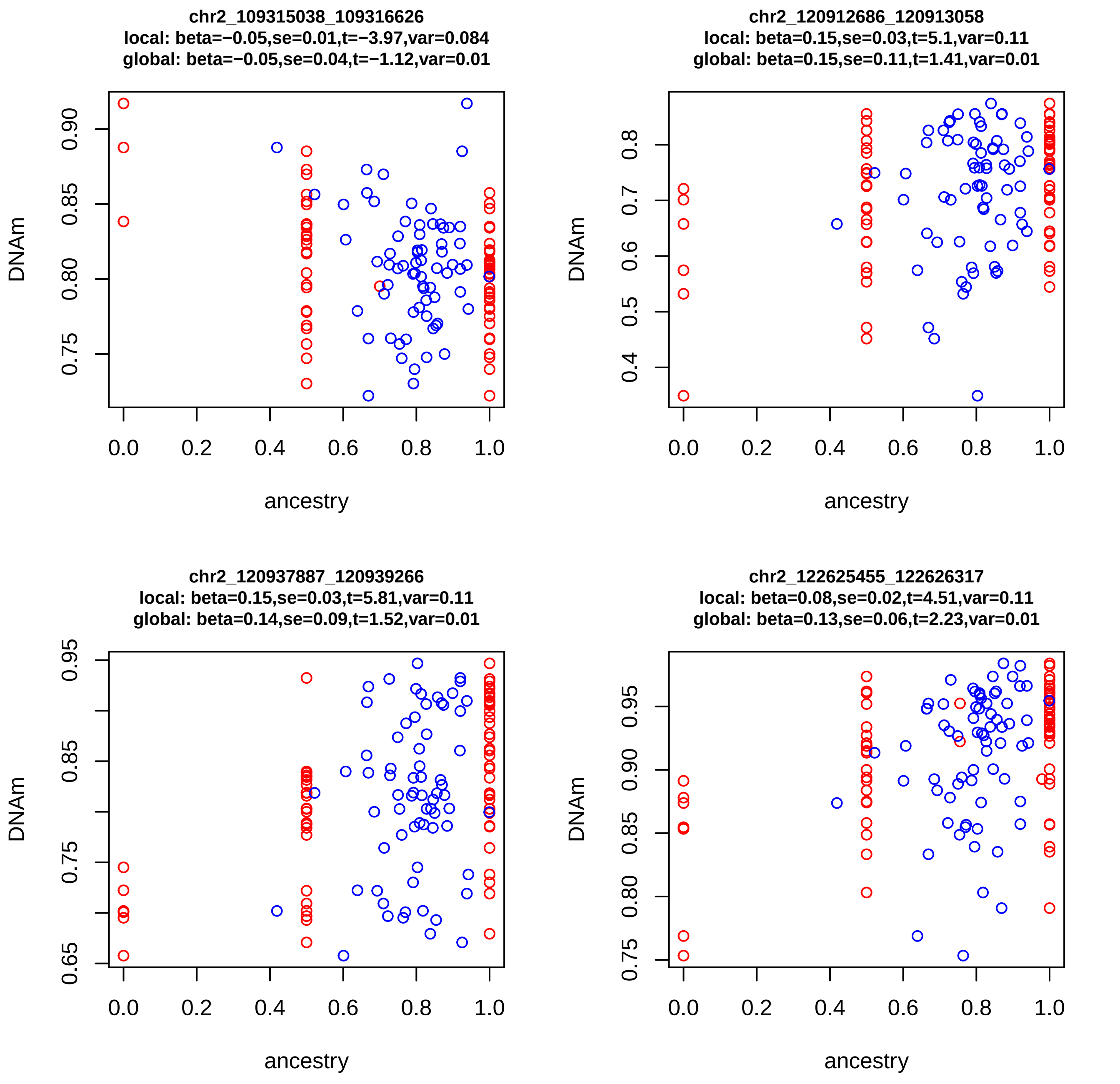
chr2_109315038_109316626
local: beta=−0.05,se=0.01,t=−3.97,var=0.084
global: beta=−0.05,se=0.04,t=−1.12,var=0.01
DNAm
0.75
0.80
0.85
0.90
ancestry
0.0
0.2
0.4
0.6
0.8
1.0
chr2_120912686_120913058
local: beta=0.15,se=0.03,t=5.1,var=0.11
global: beta=0.15,se=0.11,t=1.41,var=0.01
DNAm
0.4
0.5
0.6
0.7
0.8
ancestry
0.0
0.2
0.4
0.6
0.8
1.0
chr2_120937887_120939266
local: beta=0.15,se=0.03,t=5.81,var=0.11
global: beta=0.14,se=0.09,t=1.52,var=0.01
DNAm
0.65
0.75
0.85
0.95
ancestry
0.0
0.2
0.4
0.6
0.8
1.0
chr2_122625455_122626317
local: beta=0.08,se=0.02,t=4.51,var=0.11
global: beta=0.13,se=0.06,t=2.23,var=0.01
DNAm
0.75
0.80
0.85
0.90
0.95
ancestry
0.0
0.2
0.4
0.6
0.8
1.0

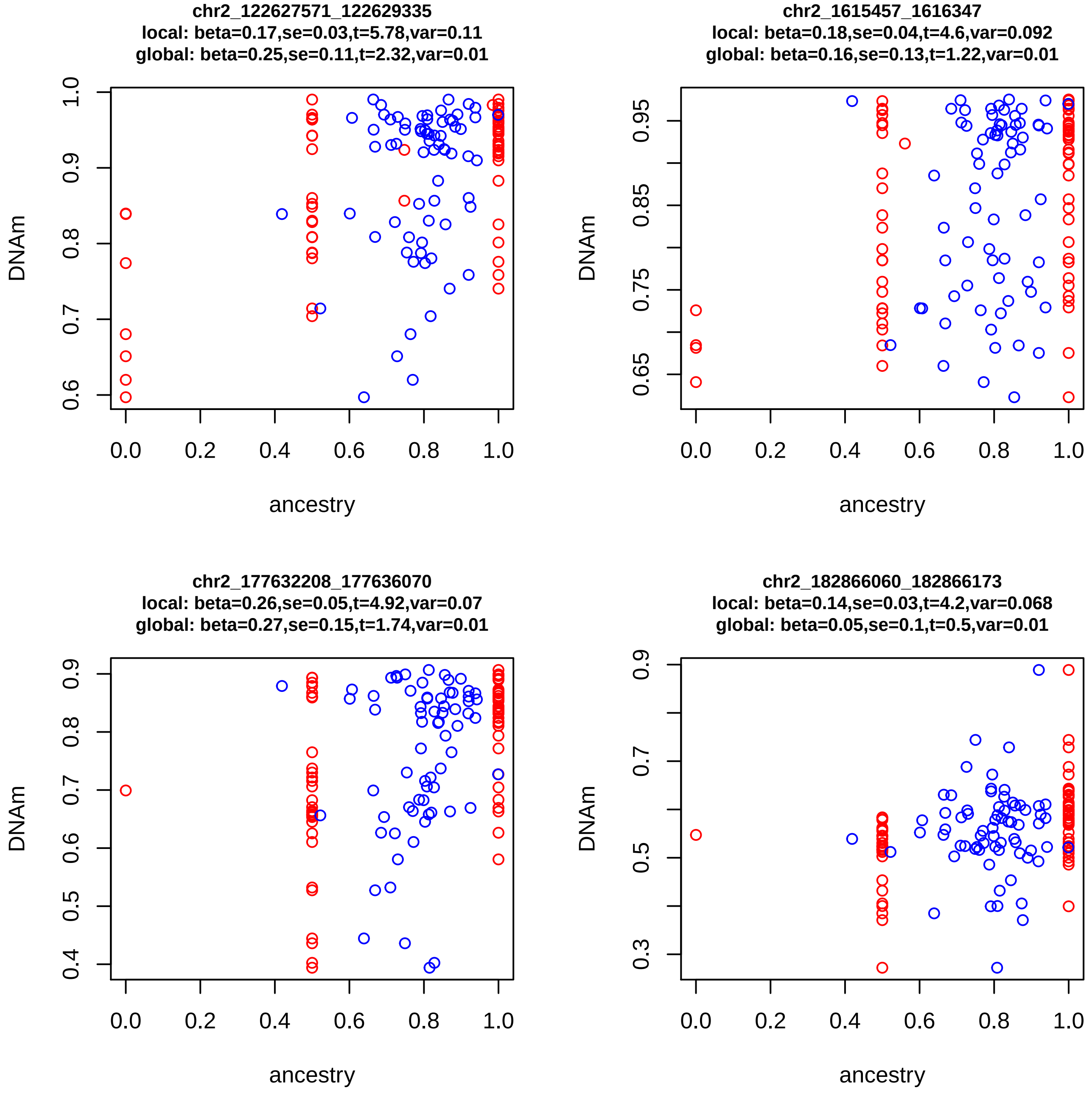
chr2_122627571_122629335
local: beta=0.17,se=0.03,t=5.78,var=0.11
global: beta=0.25,se=0.11,t=2.32,var=0.01
DNAm
ancestry
0.0 0.2 0.4 0.6 0.8 1.0
0.6 0.7 0.8 0.9 1.0
chr2_1615457_1616347
local: beta=0.18,se=0.04,t=4.6,var=0.092
global: beta=0.16,se=0.13,t=1.22,var=0.01
DNAm
ancestry
0.0 0.2 0.4 0.6 0.8 1.0
0.65 0.75 0.85 0.95
chr2_177632208_177636070
local: beta=0.26,se=0.05,t=4.92,var=0.07
global: beta=0.27,se=0.15,t=1.74,var=0.01
DNAm
ancestry
0.0 0.2 0.4 0.6 0.8 1.0
0.4 0.5 0.6 0.7 0.8 0.9
chr2_182866060_182866173
local: beta=0.14,se=0.03,t=4.2,var=0.068
global: beta=0.05,se=0.1,t=0.5,var=0.01
DNAm
ancestry
0.0 0.2 0.4 0.6 0.8 1.0
0.3 0.5 0.7 0.9

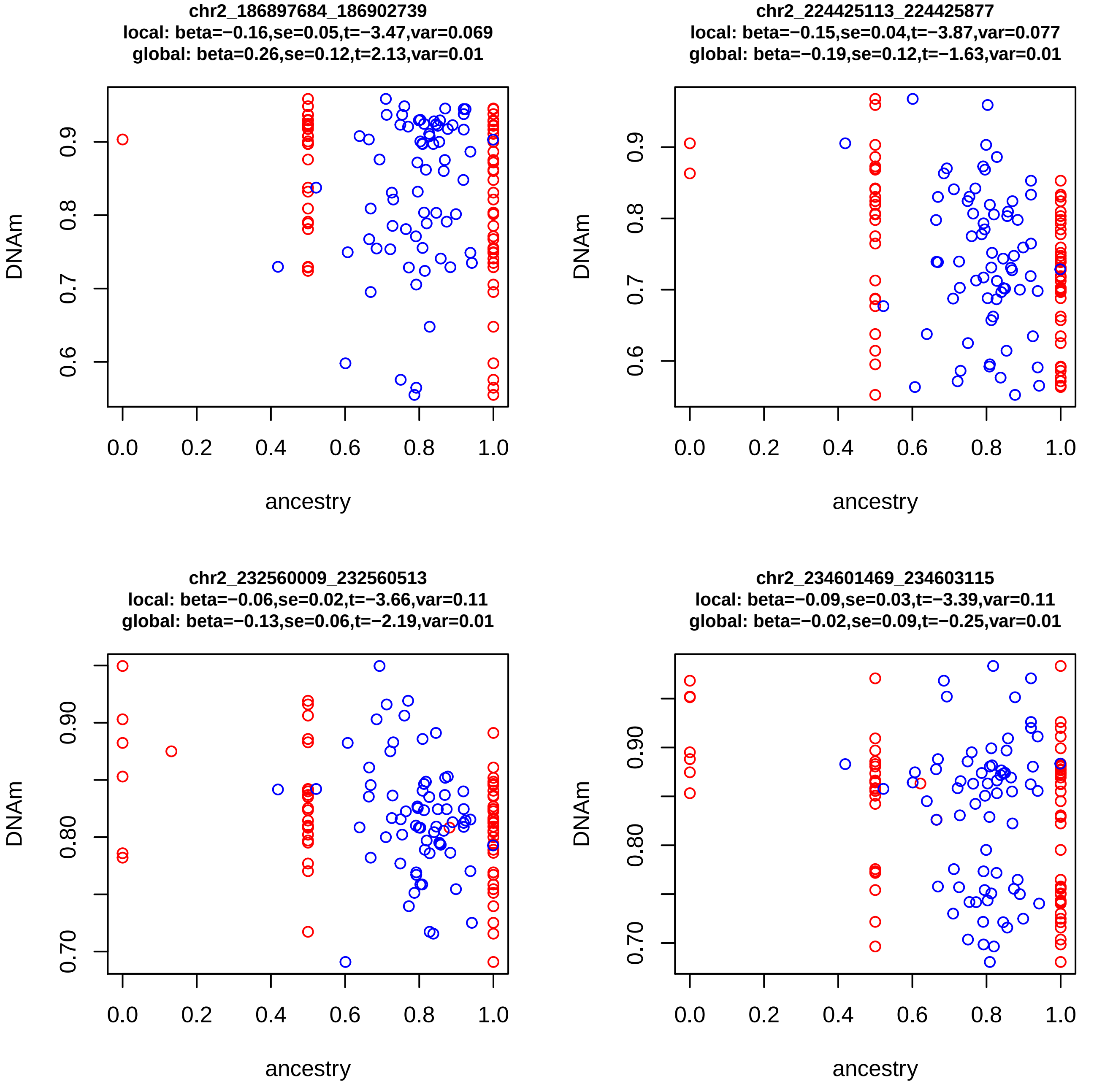
chr2_186897684_186902739
local: beta=−0.16,se=0.05,t=−3.47,var=0.069
global: beta=0.26,se=0.12,t=2.13,var=0.01
DNAm
ancestry
chr2_224425113_224425877
local: beta=−0.15,se=0.04,t=−3.87,var=0.077
global: beta=−0.19,se=0.12,t=−1.63,var=0.01
DNAm
ancestry
chr2_232560009_232560513
local: beta=−0.06,se=0.02,t=−3.66,var=0.11
global: beta=−0.13,se=0.06,t=−2.19,var=0.01
DNAm
ancestry
chr2_234601469_234603115
local: beta=−0.09,se=0.03,t=−3.39,var=0.11
global: beta=−0.02,se=0.09,t=−0.25,var=0.01
DNAm
ancestry

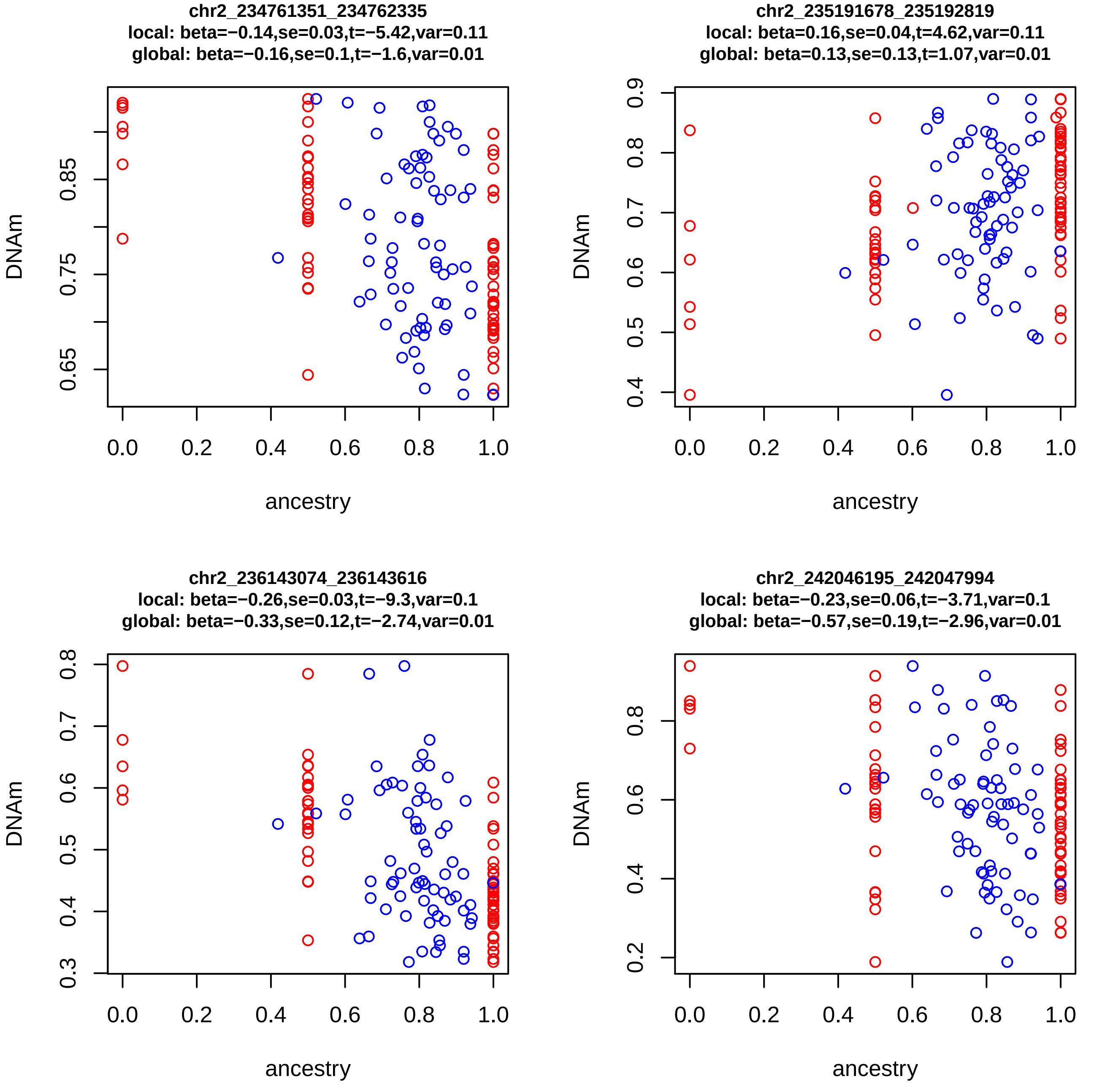
chr2_234761351_234762335
local: beta=−0.14,se=0.03,t=−5.42,var=0.11
global: beta=−0.16,se=0.1,t=−1.6,var=0.01
DNAm
0.65
0.75
0.85
ancestry
0.0
0.2
0.4
0.6
0.8
1.0
chr2_235191678_235192819
local: beta=0.16,se=0.04,t=4.62,var=0.11
global: beta=0.13,se=0.13,t=1.07,var=0.01
DNAm
0.4
0.5
0.6
0.7
0.8
0.9
ancestry
chr2_236143074_236143616
local: beta=−0.26,se=0.03,t=−9.3,var=0.1
global: beta=−0.33,se=0.12,t=−2.74,var=0.01
DNAm
0.3
0.4
0.5
0.6
0.7
0.8
ancestry
chr2_242046195_242047994
local: beta=−0.23,se=0.06,t=−3.71,var=0.1
global: beta=−0.57,se=0.19,t=−2.96,var=0.01
DNAm
0.2
0.4
0.6
0.8
ancestry

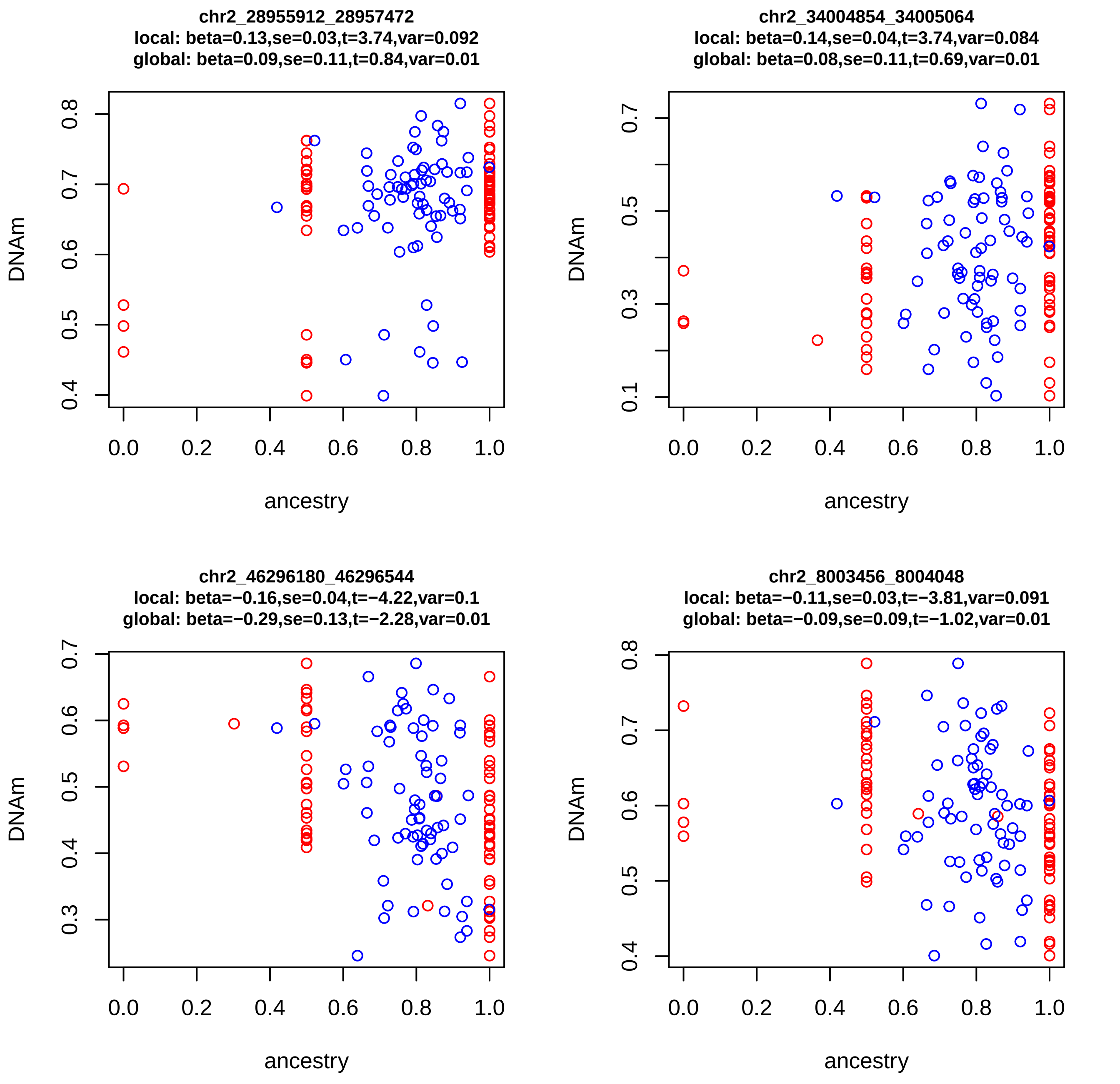
chr2_28955912_28957472
local: beta=0.13,se=0.03,t=3.74,var=0.092
global: beta=0.09,se=0.11,t=0.84,var=0.01
DNAm
ancestry
chr2_34004854_34005064
local: beta=0.14,se=0.04,t=3.74,var=0.084
global: beta=0.08,se=0.11,t=0.69,var=0.01
DNAm
ancestry
chr2_46296180_46296544
local: beta=−0.16,se=0.04,t=−4.22,var=0.1
global: beta=−0.29,se=0.13,t=−2.28,var=0.01
DNAm
ancestry
chr2_8003456_8004048
local: beta=−0.11,se=0.03,t=−3.81,var=0.091
global: beta=−0.09,se=0.09,t=−1.02,var=0.01
DNAm
ancestry

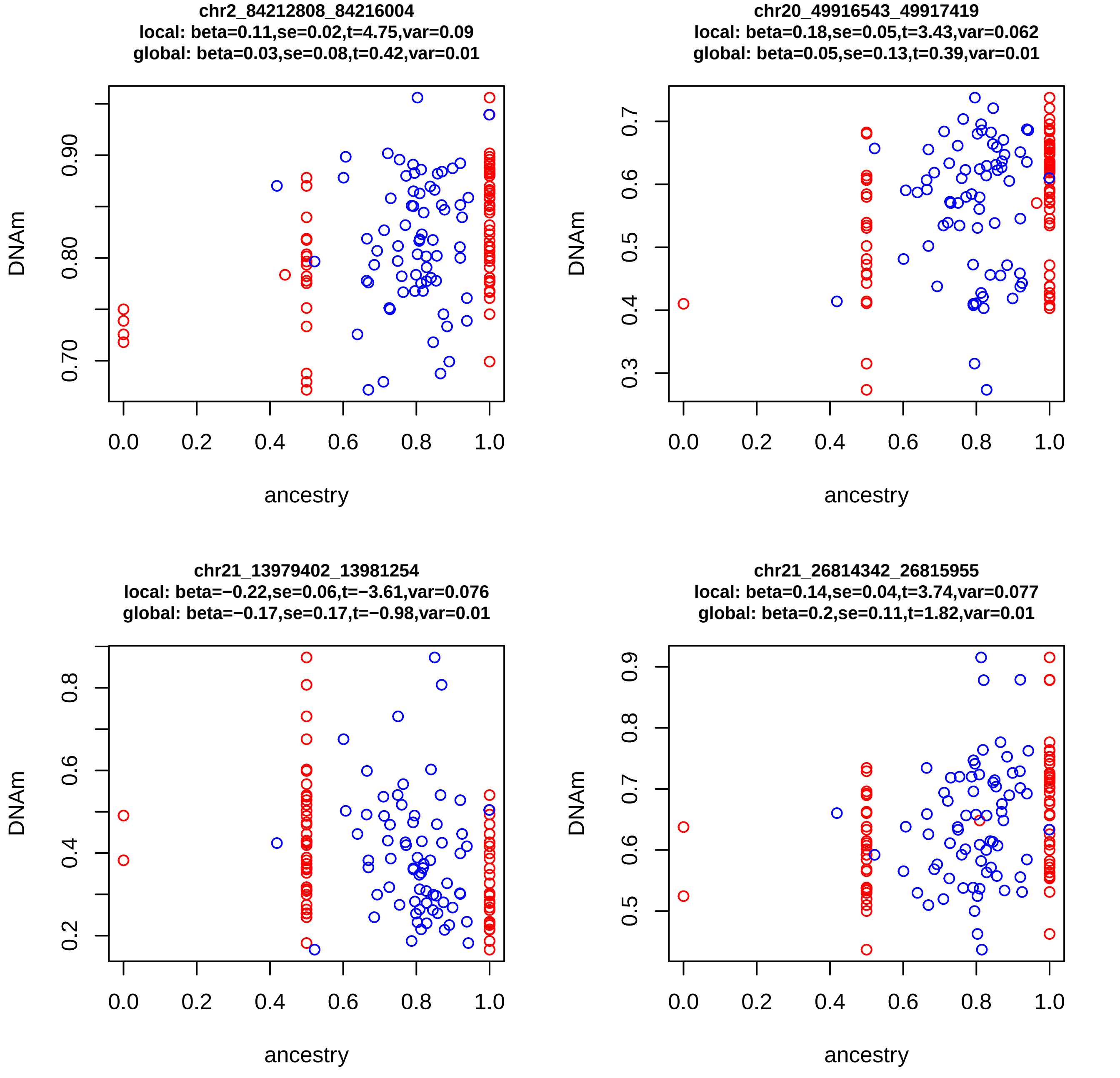
chr2_84212808_84216004
local: beta=0.11,se=0.02,t=4.75,var=0.09
global: beta=0.03,se=0.08,t=0.42,var=0.01
DNAm
0.70
0.80
0.90
0.0
0.2
0.4
0.6
0.8
1.0
ancestry
chr20_49916543_49917419
local: beta=0.18,se=0.05,t=3.43,var=0.062
global: beta=0.05,se=0.13,t=0.39,var=0.01
DNAm
0.3
0.4
0.5
0.6
0.7
ancestry
chr21_13979402_13981254
local: beta=−0.22,se=0.06,t=−3.61,var=0.076
global: beta=−0.17,se=0.17,t=−0.98,var=0.01
DNAm
0.2
0.4
0.6
0.8
ancestry
chr21_26814342_26815955
local: beta=0.14,se=0.04,t=3.74,var=0.077
global: beta=0.2,se=0.11,t=1.82,var=0.01
DNAm
0.5
0.6
0.7
0.8
0.9
ancestry

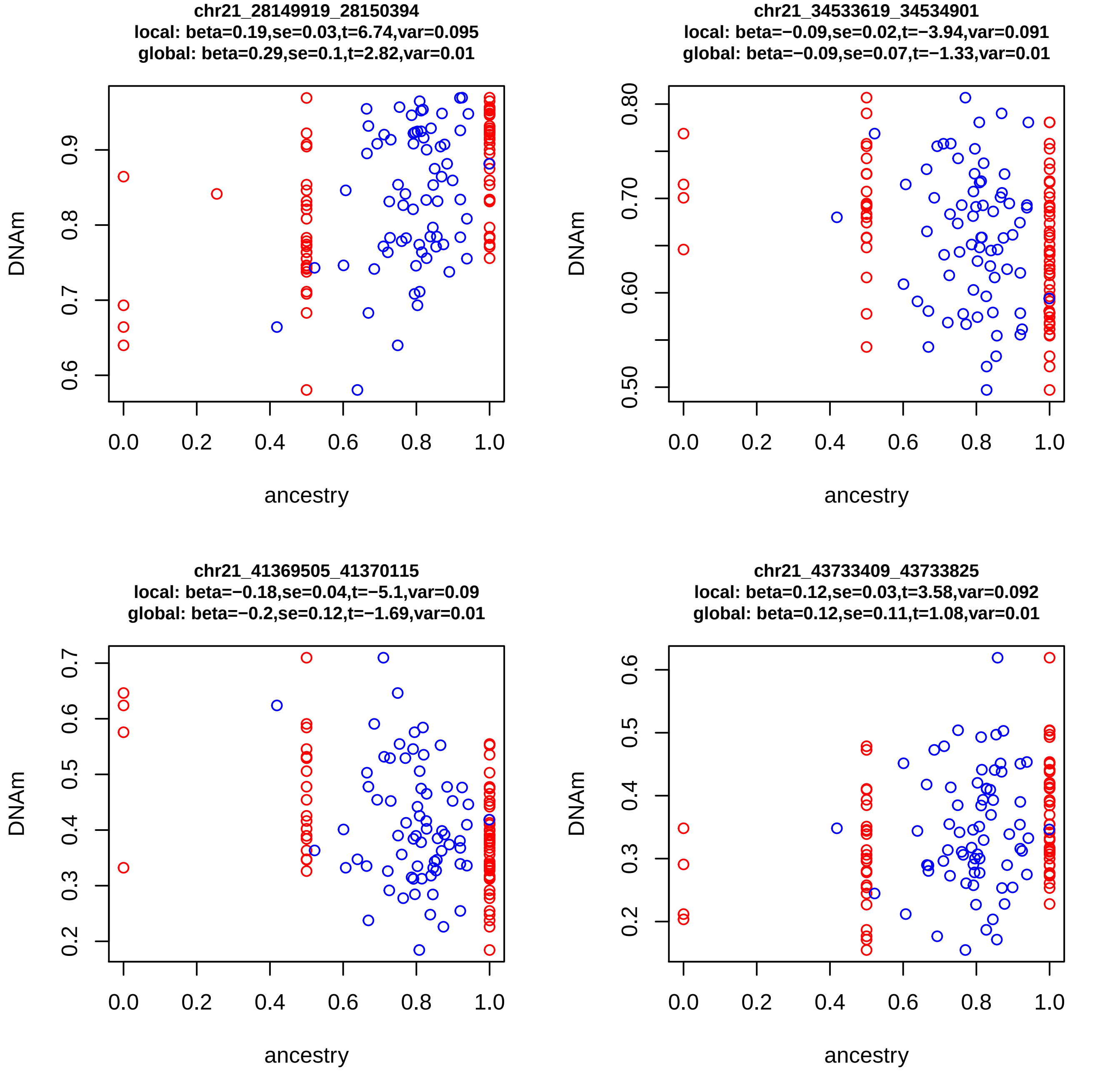
chr21_28149919_28150394
local: beta=0.19,se=0.03,t=6.74,var=0.095
global: beta=0.29,se=0.1,t=2.82,var=0.01
DNAm
ancestry
chr21_34533619_34534901
local: beta=−0.09,se=0.02,t=−3.94,var=0.091
global: beta=−0.09,se=0.07,t=−1.33,var=0.01
DNAm
ancestry
chr21_41369505_41370115
local: beta=−0.18,se=0.04,t=−5.1,var=0.09
global: beta=−0.2,se=0.12,t=−1.69,var=0.01
DNAm
ancestry
chr21_43733409_43733825
local: beta=0.12,se=0.03,t=3.58,var=0.092
global: beta=0.12,se=0.11,t=1.08,var=0.01
DNAm
ancestry

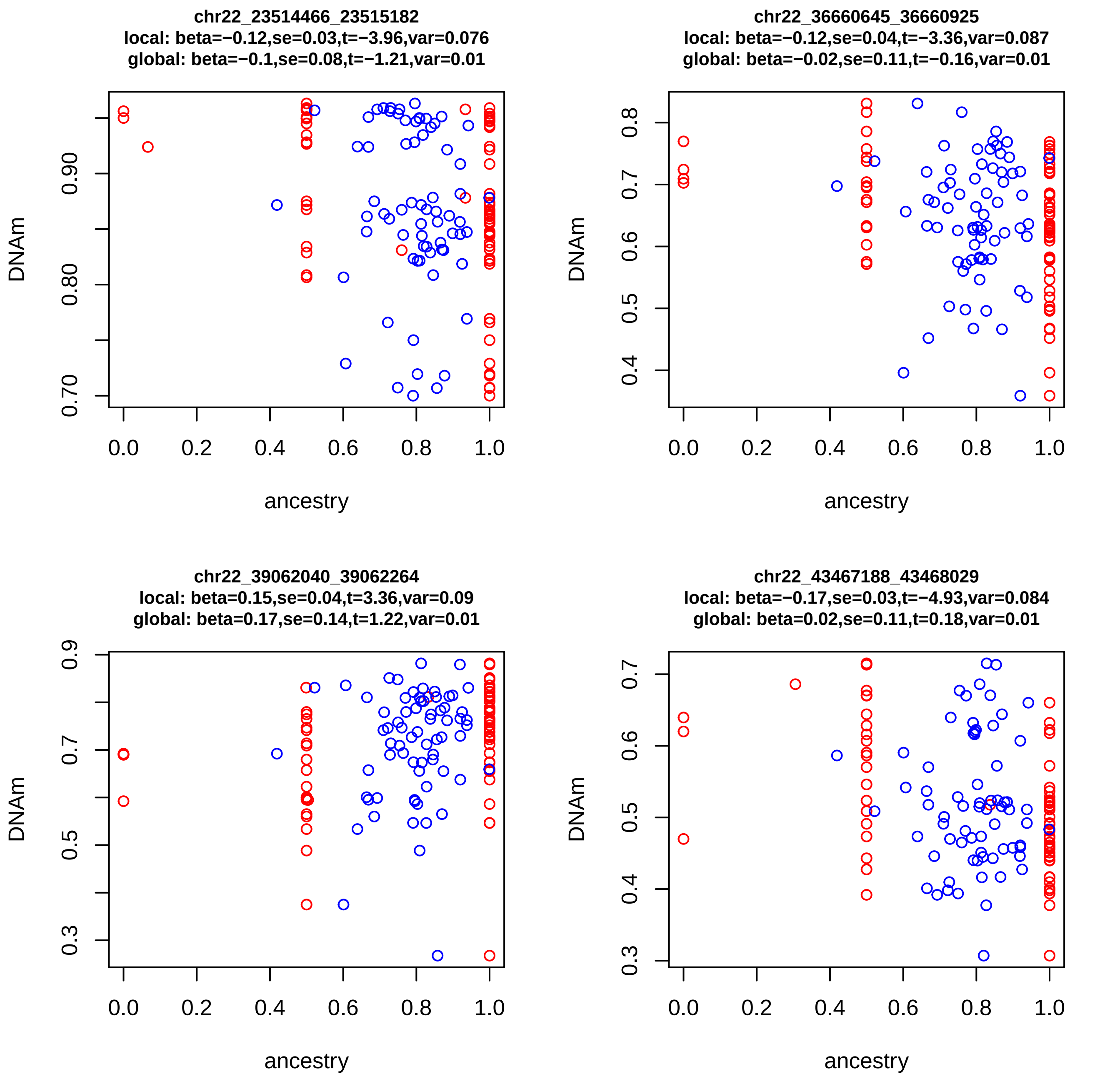
chr22_23514466_23515182
local: beta=−0.12,se=0.03,t=−3.96,var=0.076
global: beta=−0.1,se=0.08,t=−1.21,var=0.01
DNAm
0.70
0.80
0.90
0.0
0.2
0.4
0.6
0.8
1.0
ancestry
chr22_36660645_36660925
local: beta=−0.12,se=0.04,t=−3.36,var=0.087
global: beta=−0.02,se=0.11,t=−0.16,var=0.01
DNAm
0.4
0.5
0.6
0.7
0.8
ancestry
chr22_39062040_39062264
local: beta=0.15,se=0.04,t=3.36,var=0.09
global: beta=0.17,se=0.14,t=1.22,var=0.01
DNAm
0.3
0.5
0.7
0.9
ancestry
chr22_43467188_43468029
local: beta=−0.17,se=0.03,t=−4.93,var=0.084
global: beta=0.02,se=0.11,t=0.18,var=0.01
DNAm
0.3
0.4
0.5
0.6
0.7
ancestry

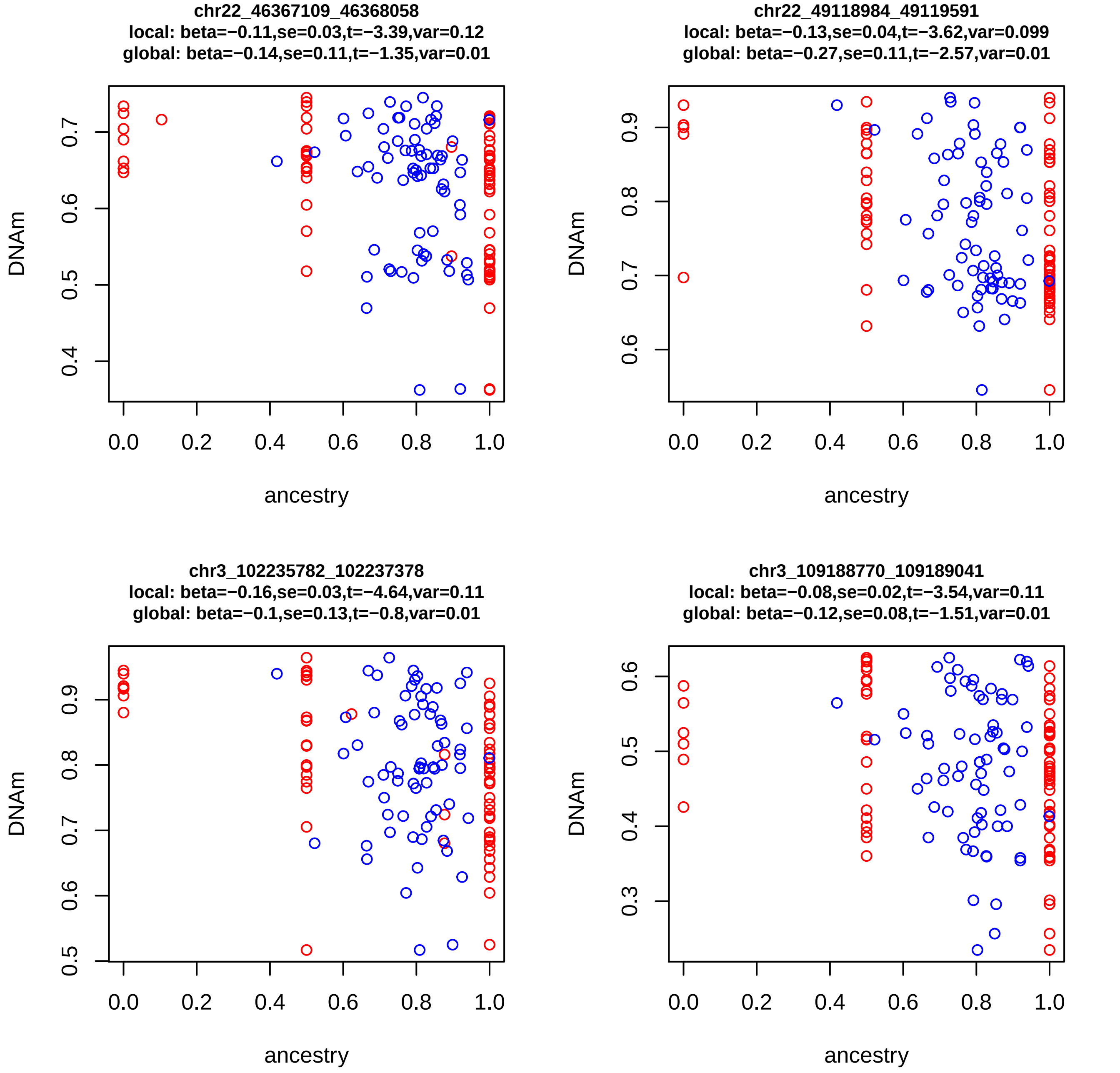
chr22_46367109_46368058
local: beta=−0.11,se=0.03,t=−3.39,var=0.12
global: beta=−0.14,se=0.11,t=−1.35,var=0.01
chr22_49118984_49119591
local: beta=−0.13,se=0.04,t=−3.62,var=0.099
global: beta=−0.27,se=0.11,t=−2.57,var=0.01
chr3_102235782_102237378
local: beta=−0.16,se=0.03,t=−4.64,var=0.11
global: beta=−0.1,se=0.13,t=−0.8,var=0.01
chr3_109188770_109189041
local: beta=−0.08,se=0.02,t=−3.54,var=0.11
global: beta=−0.12,se=0.08,t=−1.51,var=0.01
DNAm
ancestry

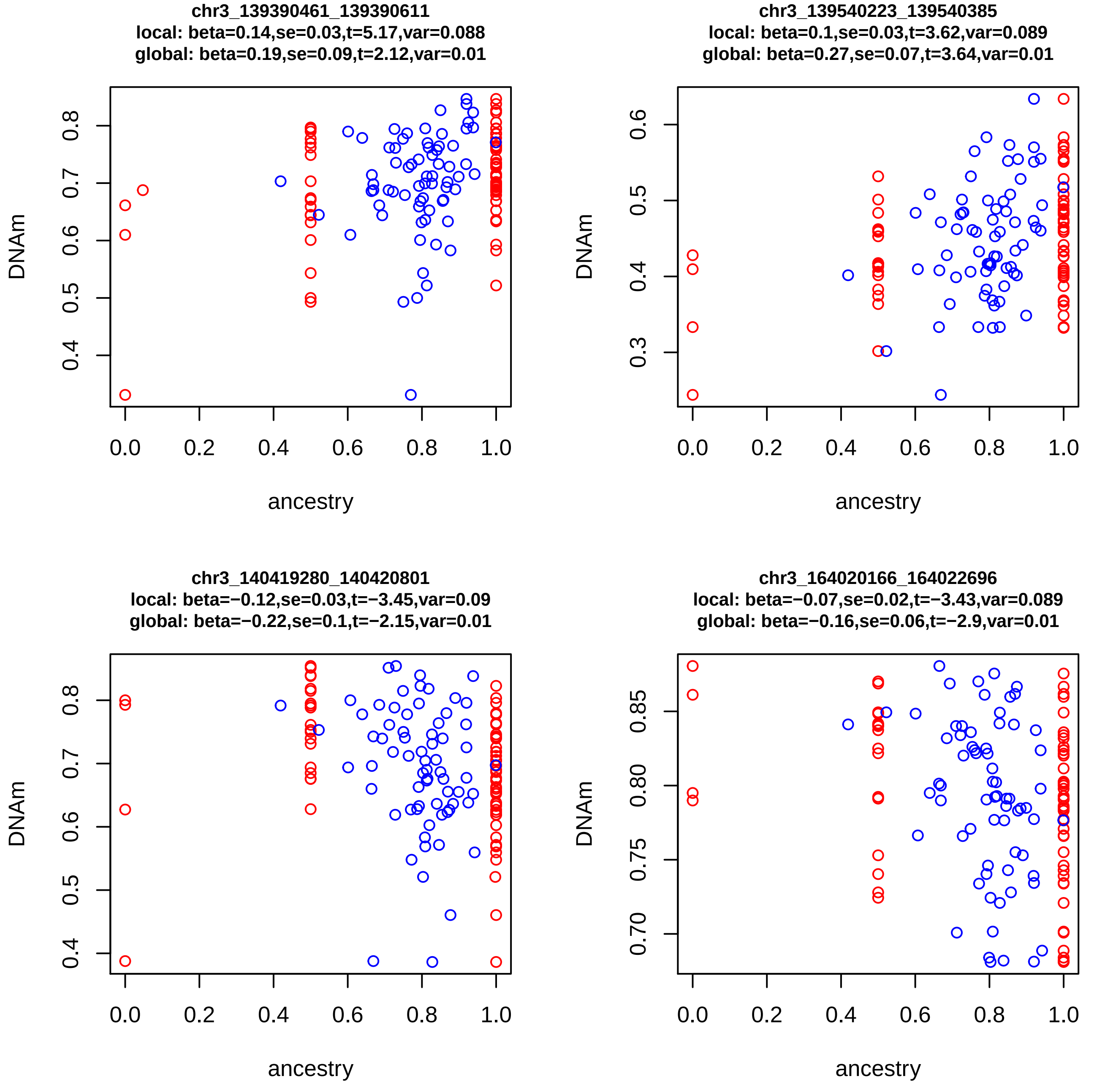
chr3_139390461_139390611
local: beta=0.14,se=0.03,t=5.17,var=0.088
global: beta=0.19,se=0.09,t=2.12,var=0.01
DNAm
0.4
0.5
0.6
0.7
0.8
0.0
0.2
0.4
0.6
0.8
1.0
ancestry
chr3_139540223_139540385
local: beta=0.1,se=0.03,t=3.62,var=0.089
global: beta=0.27,se=0.07,t=3.64,var=0.01
DNAm
0.3
0.4
0.5
0.6
0.0
0.2
0.4
0.6
0.8
1.0
ancestry
chr3_140419280_140420801
local: beta=−0.12,se=0.03,t=−3.45,var=0.09
global: beta=−0.22,se=0.1,t=−2.15,var=0.01
DNAm
0.4
0.5
0.6
0.7
0.8
0.0
0.2
0.4
0.6
0.8
1.0
ancestry
chr3_164020166_164022696
local: beta=−0.07,se=0.02,t=−3.43,var=0.089
global: beta=−0.16,se=0.06,t=−2.9,var=0.01
DNAm
0.70
0.75
0.80
0.85
0.0
0.2
0.4
0.6
0.8
1.0
ancestry

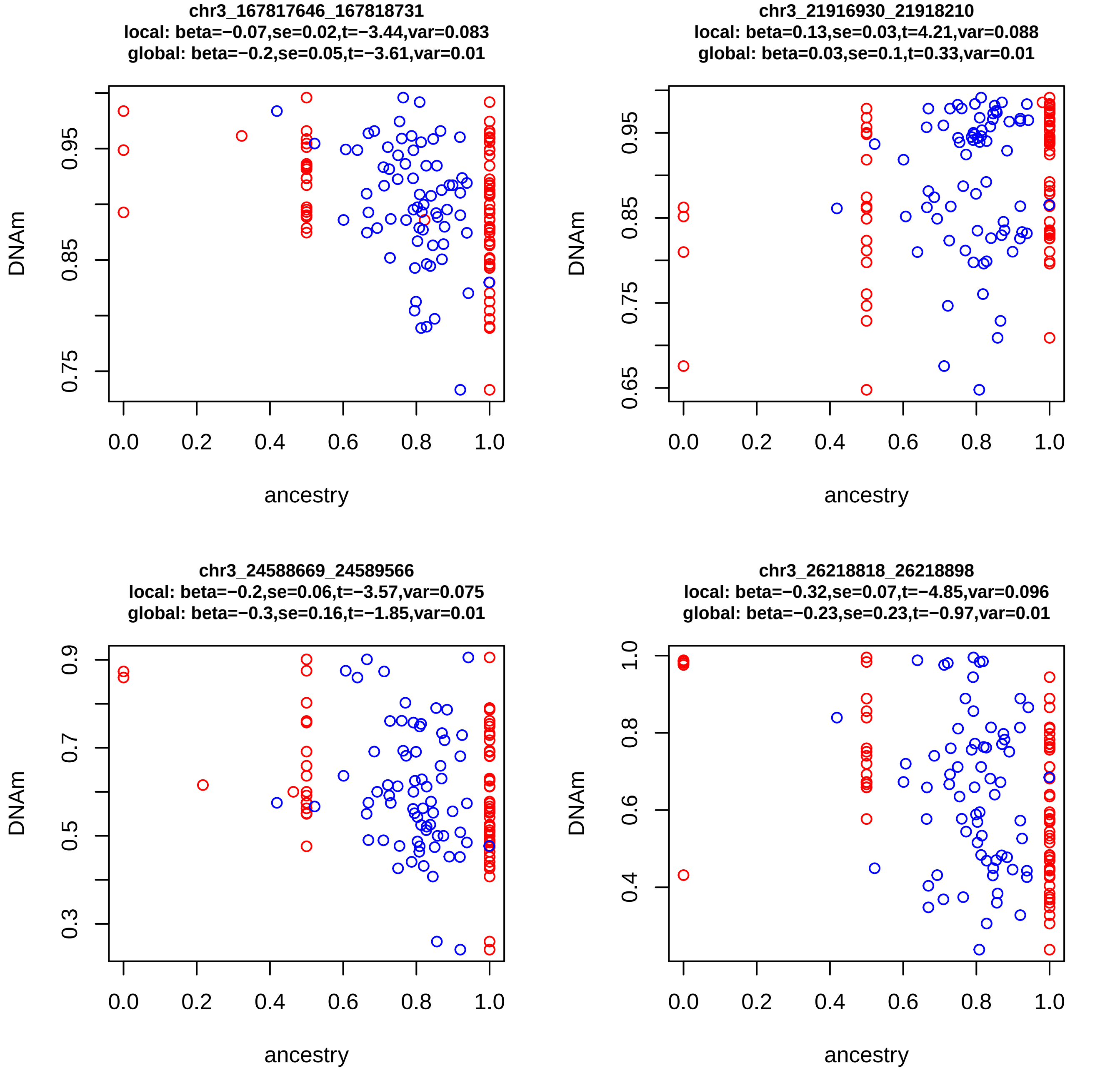
chr3_167817646_167818731
local: beta=−0.07,se=0.02,t=−3.44,var=0.083
global: beta=−0.2,se=0.05,t=−3.61,var=0.01
DNAm
ancestry
0.75
0.85
0.95
0.0
0.2
0.4
0.6
0.8
1.0
chr3_21916930_21918210
local: beta=0.13,se=0.03,t=4.21,var=0.088
global: beta=0.03,se=0.1,t=0.33,var=0.01
DNAm
ancestry
0.65
0.75
0.85
0.95
0.0
0.2
0.4
0.6
0.8
1.0
chr3_24588669_24589566
local: beta=−0.2,se=0.06,t=−3.57,var=0.075
global: beta=−0.3,se=0.16,t=−1.85,var=0.01
DNAm
ancestry
0.3
0.5
0.7
0.9
0.0
0.2
0.4
0.6
0.8
1.0
chr3_26218818_26218898
local: beta=−0.32,se=0.07,t=−4.85,var=0.096
global: beta=−0.23,se=0.23,t=−0.97,var=0.01
DNAm
ancestry
0.4
0.6
0.8
1.0
0.0
0.2
0.4
0.6
0.8
1.0

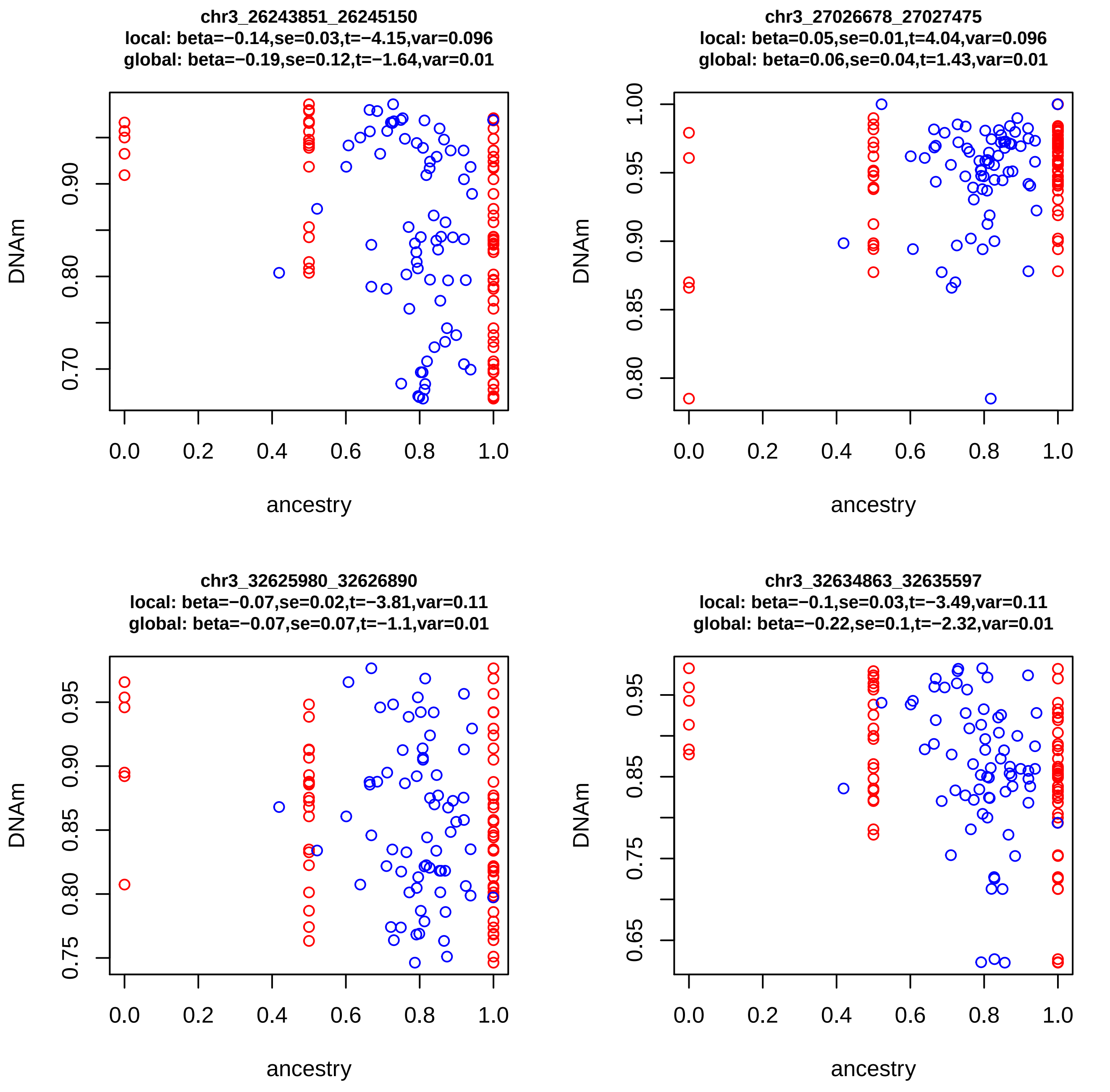
chr3_26243851_26245150
local: beta=−0.14,se=0.03,t=−4.15,var=0.096
global: beta=−0.19,se=0.12,t=−1.64,var=0.01
DNAm
0.70
0.80
0.90
ancestry
0.0
0.2
0.4
0.6
0.8
1.0
chr3_27026678_27027475
local: beta=0.05,se=0.01,t=4.04,var=0.096
global: beta=0.06,se=0.04,t=1.43,var=0.01
DNAm
0.80
0.85
0.90
0.95
1.00
ancestry
0.0
0.2
0.4
0.6
0.8
1.0
chr3_32625980_32626890
local: beta=−0.07,se=0.02,t=−3.81,var=0.11
global: beta=−0.07,se=0.07,t=−1.1,var=0.01
DNAm
0.75
0.80
0.85
0.90
0.95
ancestry
0.0
0.2
0.4
0.6
0.8
1.0
chr3_32634863_32635597
local: beta=−0.1,se=0.03,t=−3.49,var=0.11
global: beta=−0.22,se=0.1,t=−2.32,var=0.01
DNAm
0.65
0.75
0.85
0.95
ancestry
0.0
0.2
0.4
0.6
0.8
1.0

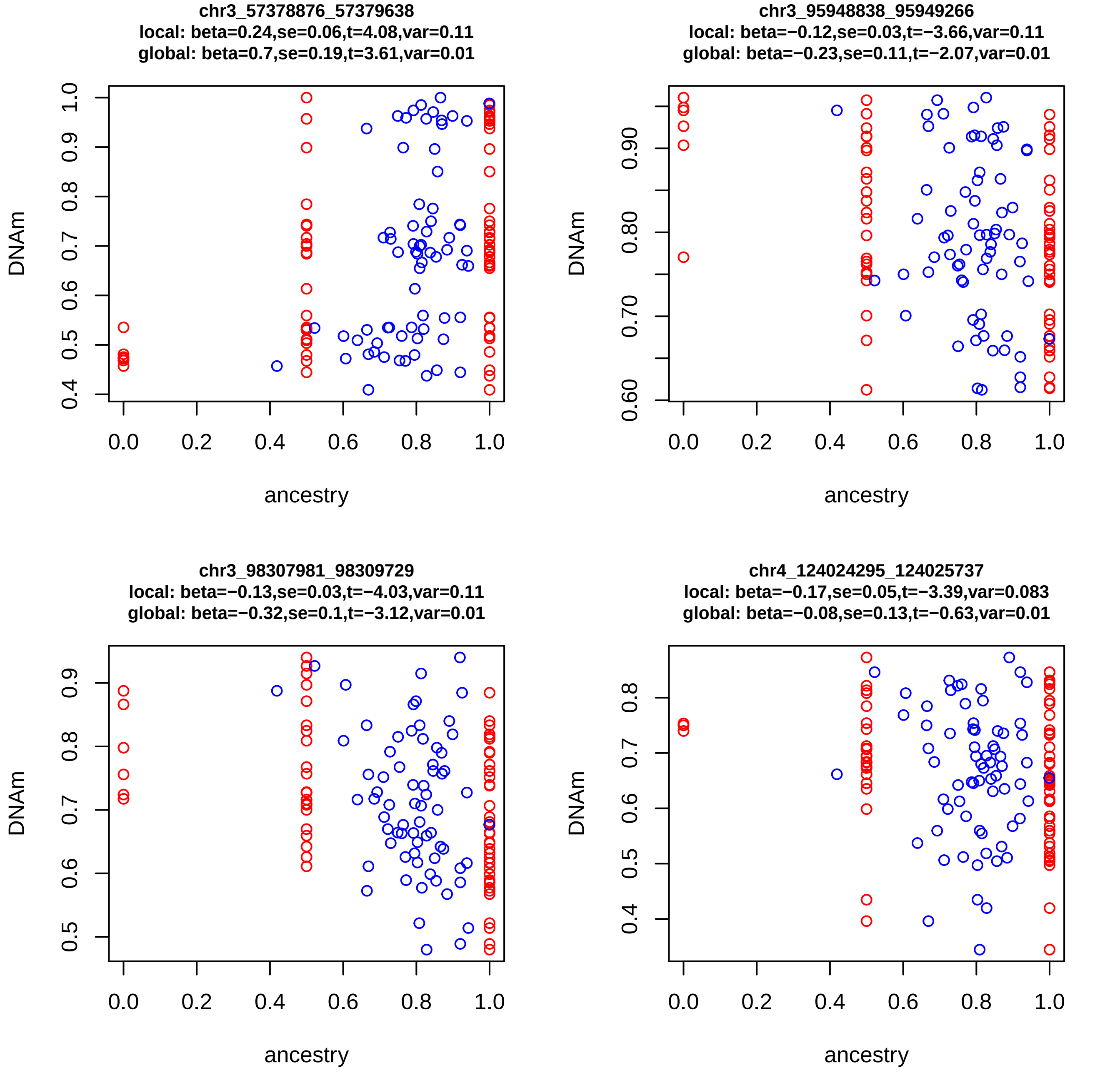
chr3_57378876_57379638
local: beta=0.24,se=0.06,t=4.08,var=0.11
global: beta=0.7,se=0.19,t=3.61,var=0.01
DNAm
ancestry
chr3_95948838_95949266
local: beta=−0.12,se=0.03,t=−3.66,var=0.11
global: beta=−0.23,se=0.11,t=−2.07,var=0.01
DNAm
ancestry
chr3_98307981_98309729
local: beta=−0.13,se=0.03,t=−4.03,var=0.11
global: beta=−0.32,se=0.1,t=−3.12,var=0.01
DNAm
ancestry
chr4_124024295_124025737
local: beta=−0.17,se=0.05,t=−3.39,var=0.083
global: beta=−0.08,se=0.13,t=−0.63,var=0.01
DNAm
ancestry

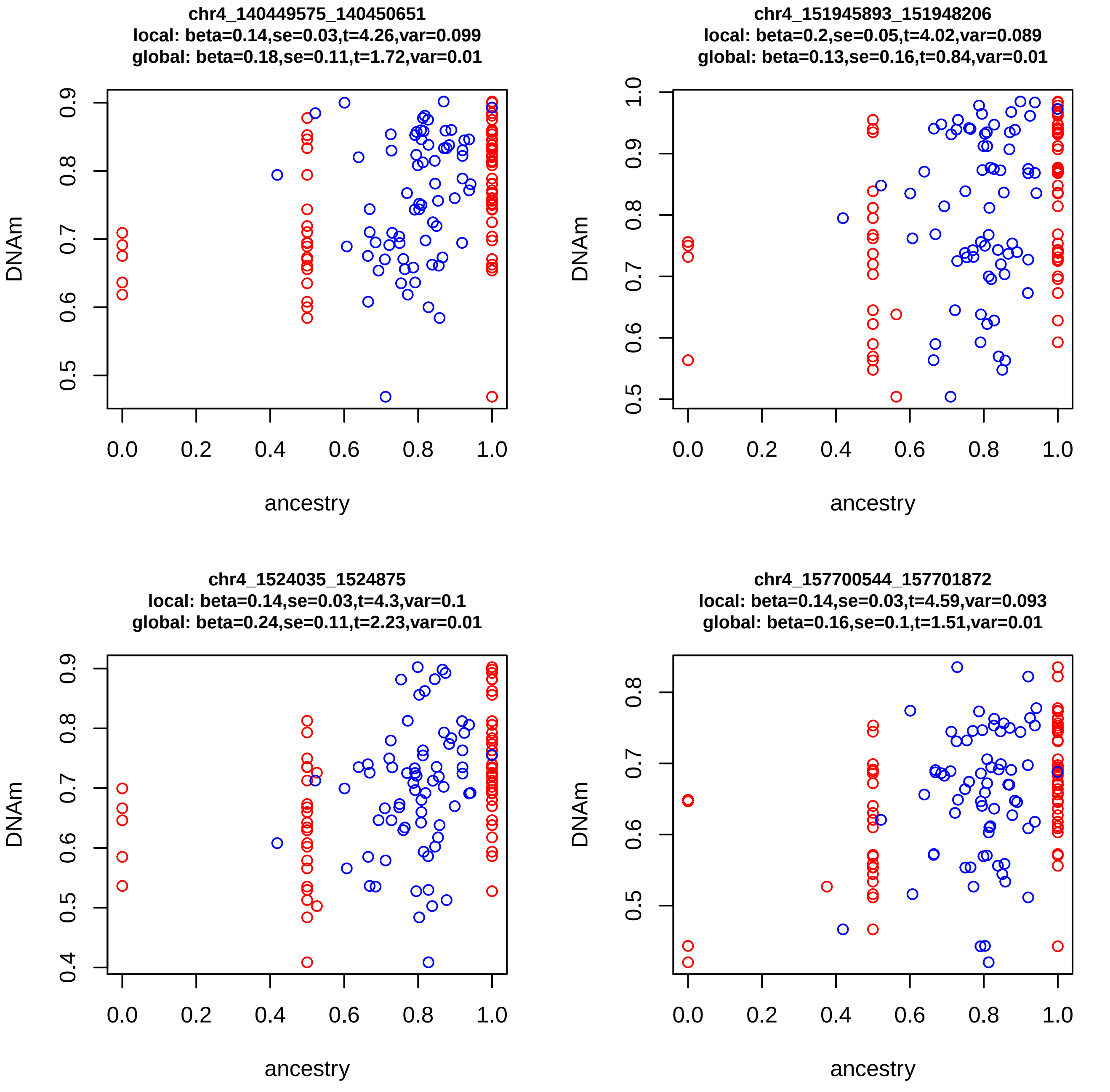
chr4_140449575_140450651
local: beta=0.14,se=0.03,t=4.26,var=0.099
global: beta=0.18,se=0.11,t=1.72,var=0.01
DNAm
ancestry
chr4_151945893_151948206
local: beta=0.2,se=0.05,t=4.02,var=0.089
global: beta=0.13,se=0.16,t=0.84,var=0.01
DNAm
ancestry
chr4_1524035_1524875
local: beta=0.14,se=0.03,t=4.3,var=0.1
global: beta=0.24,se=0.11,t=2.23,var=0.01
DNAm
ancestry
chr4_157700544_157701872
local: beta=0.14,se=0.03,t=4.59,var=0.093
global: beta=0.16,se=0.1,t=1.51,var=0.01
DNAm
ancestry

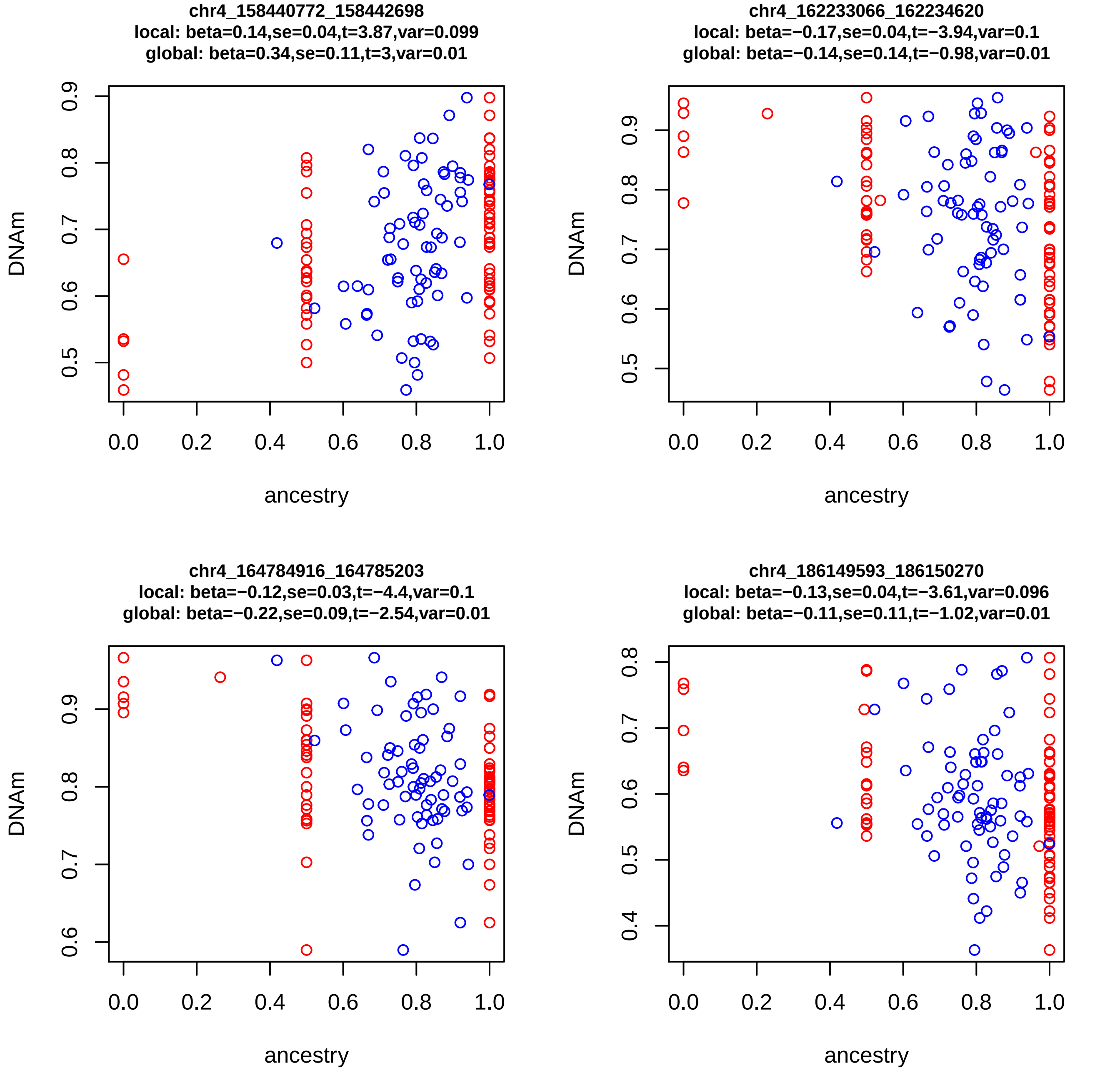
chr4_158440772_158442698
local: beta=0.14,se=0.04,t=3.87,var=0.099
global: beta=0.34,se=0.11,t=3,var=0.01
DNAm
ancestry
chr4_162233066_162234620
local: beta=−0.17,se=0.04,t=−3.94,var=0.1
global: beta=−0.14,se=0.14,t=−0.98,var=0.01
DNAm
ancestry
chr4_164784916_164785203
local: beta=−0.12,se=0.03,t=−4.4,var=0.1
global: beta=−0.22,se=0.09,t=−2.54,var=0.01
DNAm
ancestry
chr4_186149593_186150270
local: beta=−0.13,se=0.04,t=−3.61,var=0.096
global: beta=−0.11,se=0.11,t=−1.02,var=0.01
DNAm
ancestry

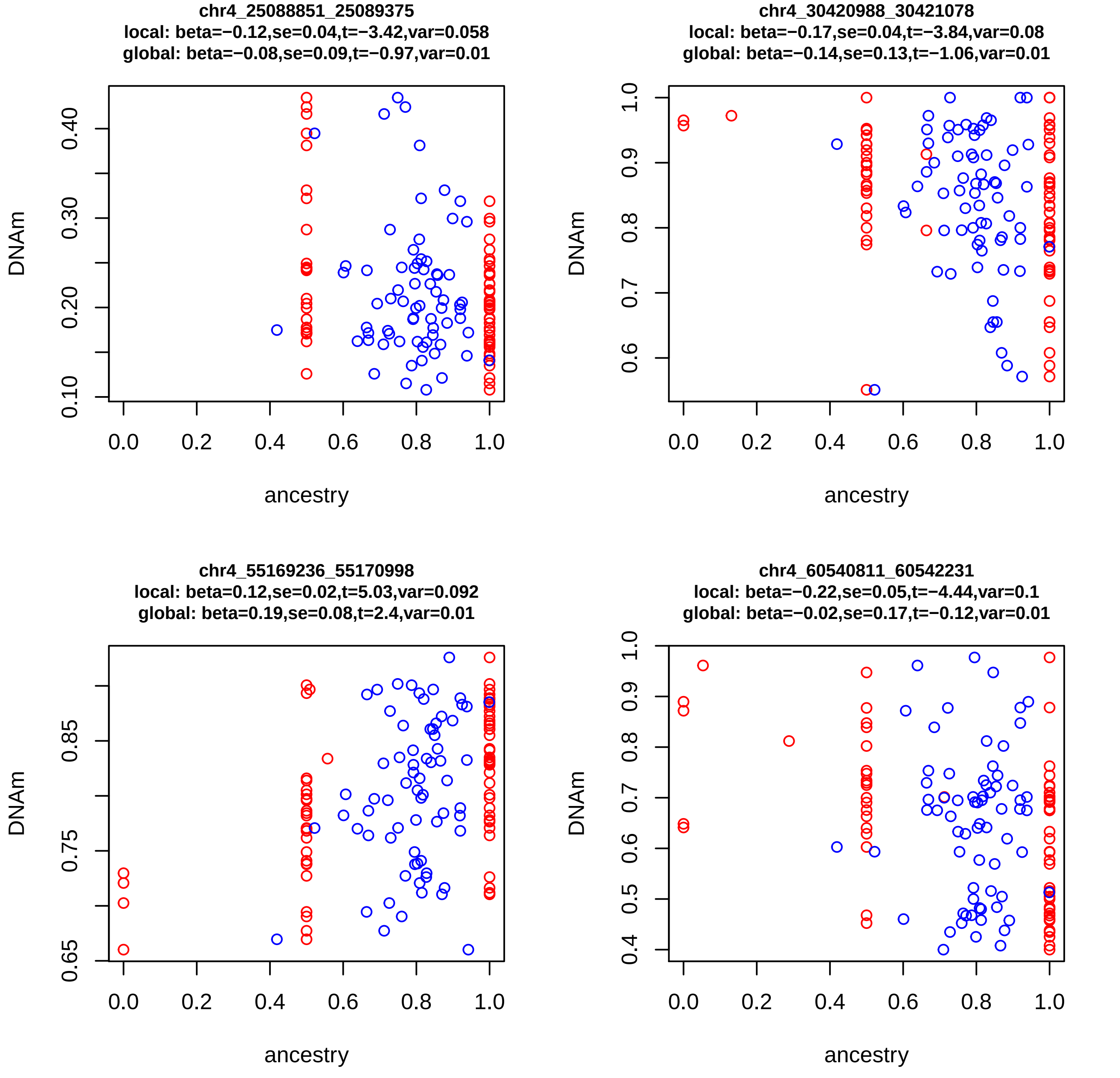
chr4_25088851_25089375
local: beta=−0.12,se=0.04,t=−3.42,var=0.058
global: beta=−0.08,se=0.09,t=−0.97,var=0.01
DNAm
ancestry
chr4_30420988_30421078
local: beta=−0.17,se=0.04,t=−3.84,var=0.08
global: beta=−0.14,se=0.13,t=−1.06,var=0.01
DNAm
ancestry
chr4_55169236_55170998
local: beta=0.12,se=0.02,t=5.03,var=0.092
global: beta=0.19,se=0.08,t=2.4,var=0.01
DNAm
ancestry
chr4_60540811_60542231
local: beta=−0.22,se=0.05,t=−4.44,var=0.1
global: beta=−0.02,se=0.17,t=−0.12,var=0.01
DNAm
ancestry

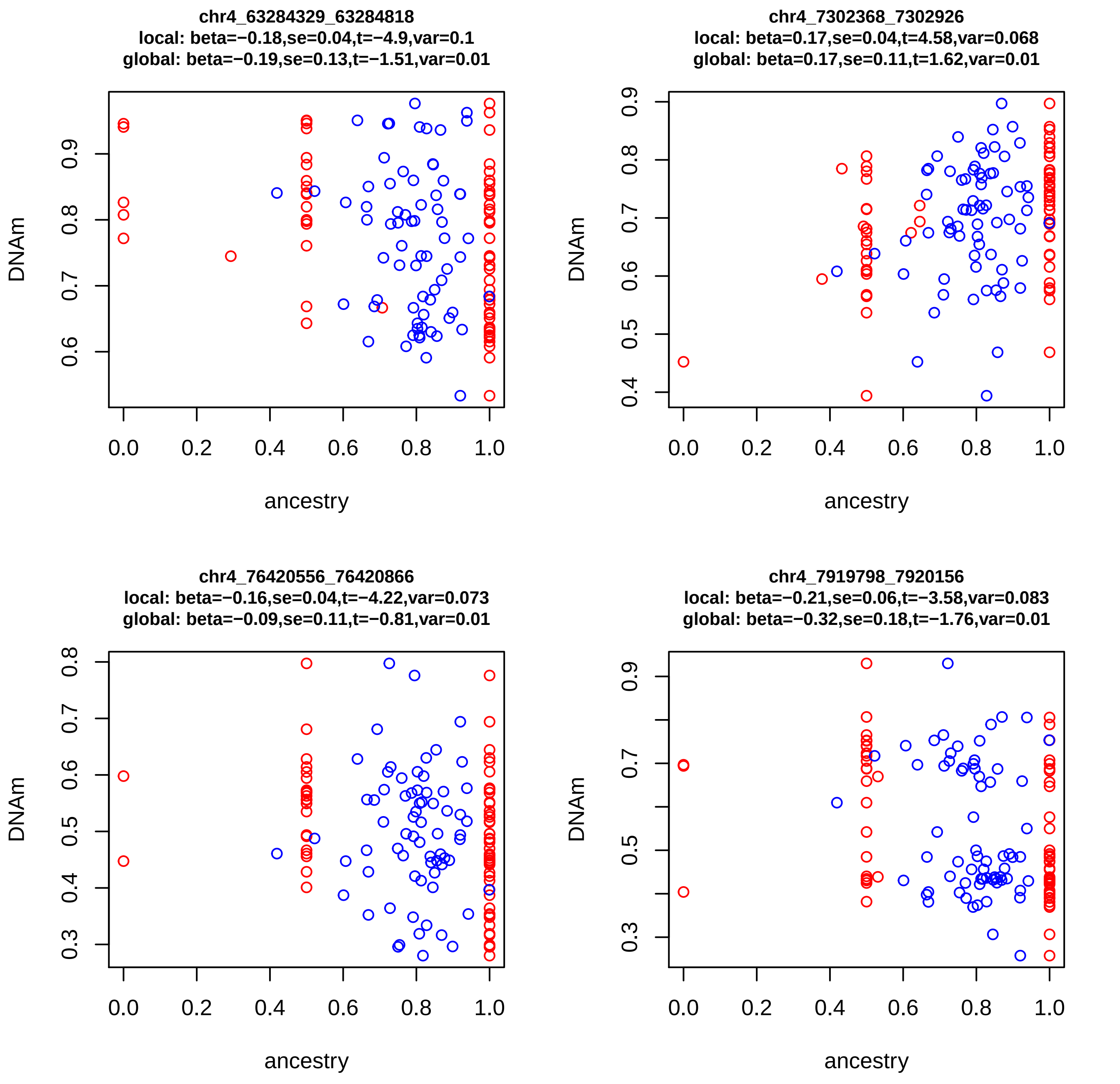
chr4_63284329_63284818
local: beta=−0.18,se=0.04,t=−4.9,var=0.1
global: beta=−0.19,se=0.13,t=−1.51,var=0.01
DNAm
0.6
0.7
0.8
0.9
0.0
0.2
0.4
0.6
0.8
1.0
ancestry
chr4_7302368_7302926
local: beta=0.17,se=0.04,t=4.58,var=0.068
global: beta=0.17,se=0.11,t=1.62,var=0.01
DNAm
0.4
0.5
0.6
0.7
0.8
0.9
0.0
0.2
0.4
0.6
0.8
1.0
ancestry
chr4_76420556_76420866
local: beta=−0.16,se=0.04,t=−4.22,var=0.073
global: beta=−0.09,se=0.11,t=−0.81,var=0.01
DNAm
0.3
0.4
0.5
0.6
0.7
0.8
0.0
0.2
0.4
0.6
0.8
1.0
ancestry
chr4_7919798_7920156
local: beta=−0.21,se=0.06,t=−3.58,var=0.083
global: beta=−0.32,se=0.18,t=−1.76,var=0.01
DNAm
0.3
0.5
0.7
0.9
0.0
0.2
0.4
0.6
0.8
1.0
ancestry

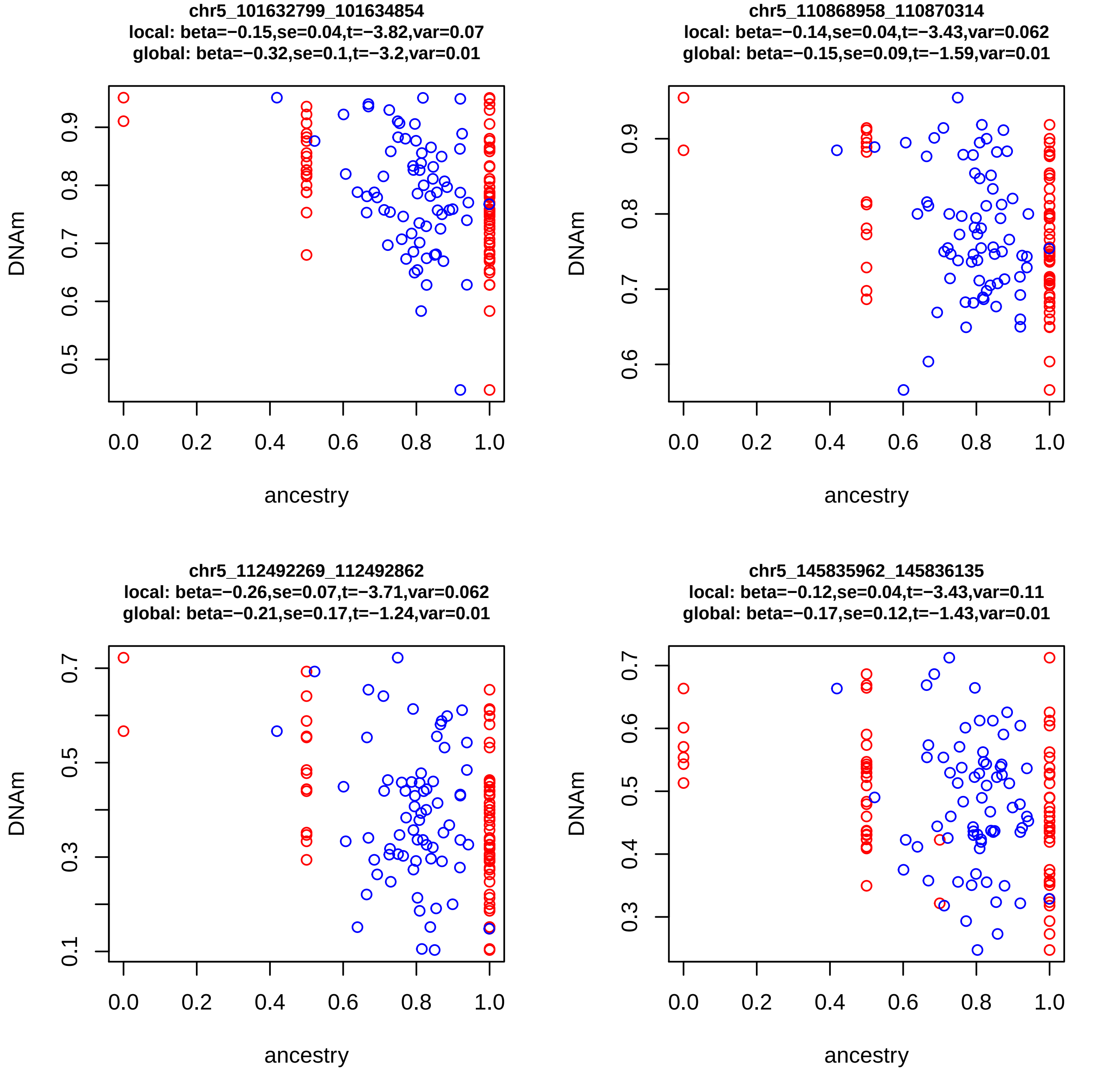
chr5_101632799_101634854
local: beta=−0.15,se=0.04,t=−3.82,var=0.07
global: beta=−0.32,se=0.1,t=−3.2,var=0.01
DNAm
0.5
0.6
0.7
0.8
0.9
0.0
0.2
0.4
0.6
0.8
1.0
ancestry
chr5_110868958_110870314
local: beta=−0.14,se=0.04,t=−3.43,var=0.062
global: beta=−0.15,se=0.09,t=−1.59,var=0.01
DNAm
0.6
0.7
0.8
0.9
0.0
0.2
0.4
0.6
0.8
1.0
ancestry
chr5_112492269_112492862
local: beta=−0.26,se=0.07,t=−3.71,var=0.062
global: beta=−0.21,se=0.17,t=−1.24,var=0.01
DNAm
0.1
0.3
0.5
0.7
0.0
0.2
0.4
0.6
0.8
1.0
ancestry
chr5_145835962_145836135
local: beta=−0.12,se=0.04,t=−3.43,var=0.11
global: beta=−0.17,se=0.12,t=−1.43,var=0.01
DNAm
0.3
0.4
0.5
0.6
0.7
0.0
0.2
0.4
0.6
0.8
1.0
ancestry

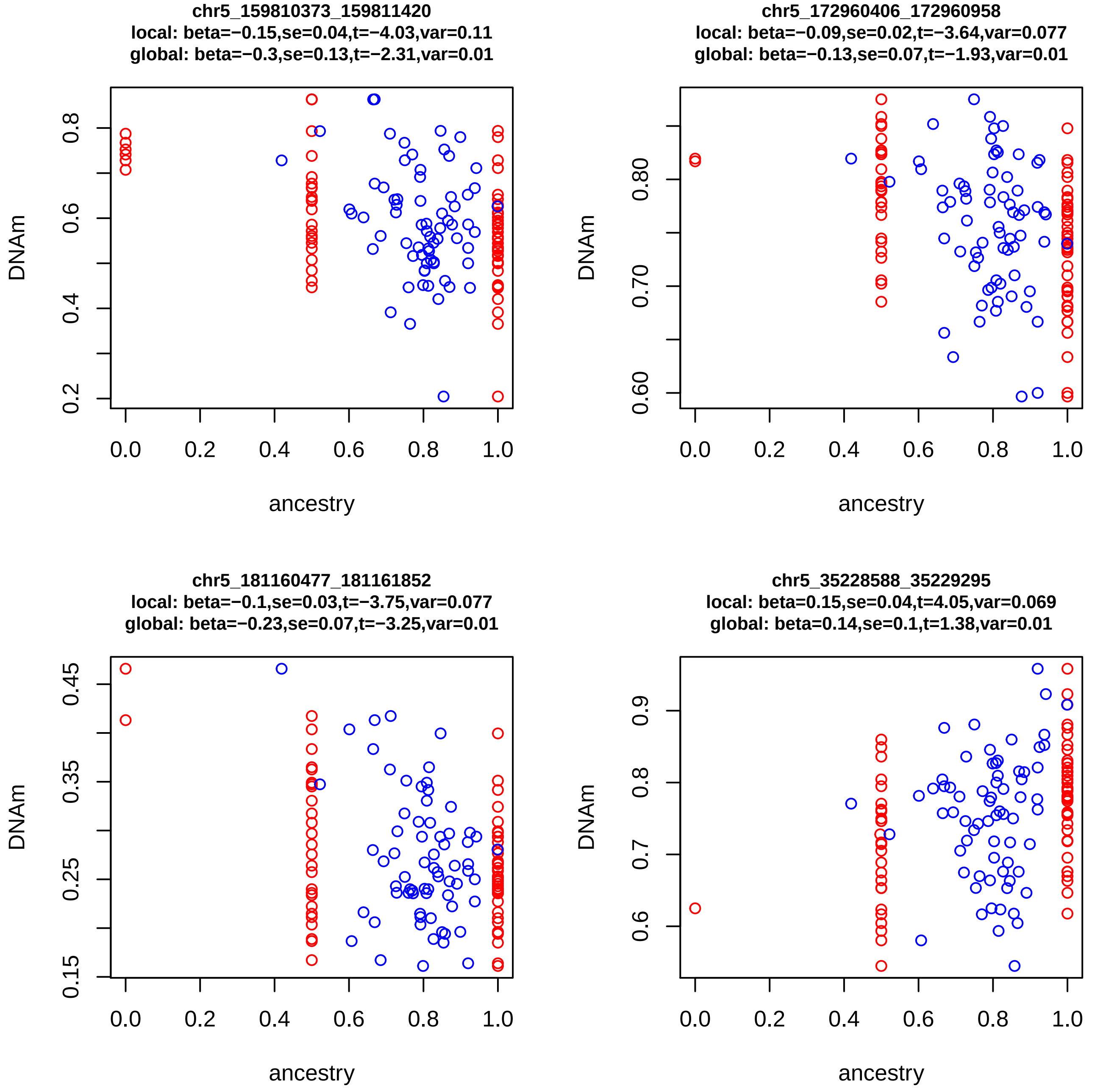
chr5_159810373_159811420
local: beta=−0.15,se=0.04,t=−4.03,var=0.11
global: beta=−0.3,se=0.13,t=−2.31,var=0.01
DNAm
0.2
0.4
0.6
0.8
0.0
0.2
0.4
0.6
0.8
1.0
ancestry
chr5_172960406_172960958
local: beta=−0.09,se=0.02,t=−3.64,var=0.077
global: beta=−0.13,se=0.07,t=−1.93,var=0.01
DNAm
0.60
0.70
0.80
0.0
0.2
0.4
0.6
0.8
1.0
ancestry
chr5_181160477_181161852
local: beta=−0.1,se=0.03,t=−3.75,var=0.077
global: beta=−0.23,se=0.07,t=−3.25,var=0.01
DNAm
0.15
0.25
0.35
0.45
0.0
0.2
0.4
0.6
0.8
1.0
ancestry
chr5_35228588_35229295
local: beta=0.15,se=0.04,t=4.05,var=0.069
global: beta=0.14,se=0.1,t=1.38,var=0.01
DNAm
0.6
0.7
0.8
0.9
0.0
0.2
0.4
0.6
0.8
1.0
ancestry

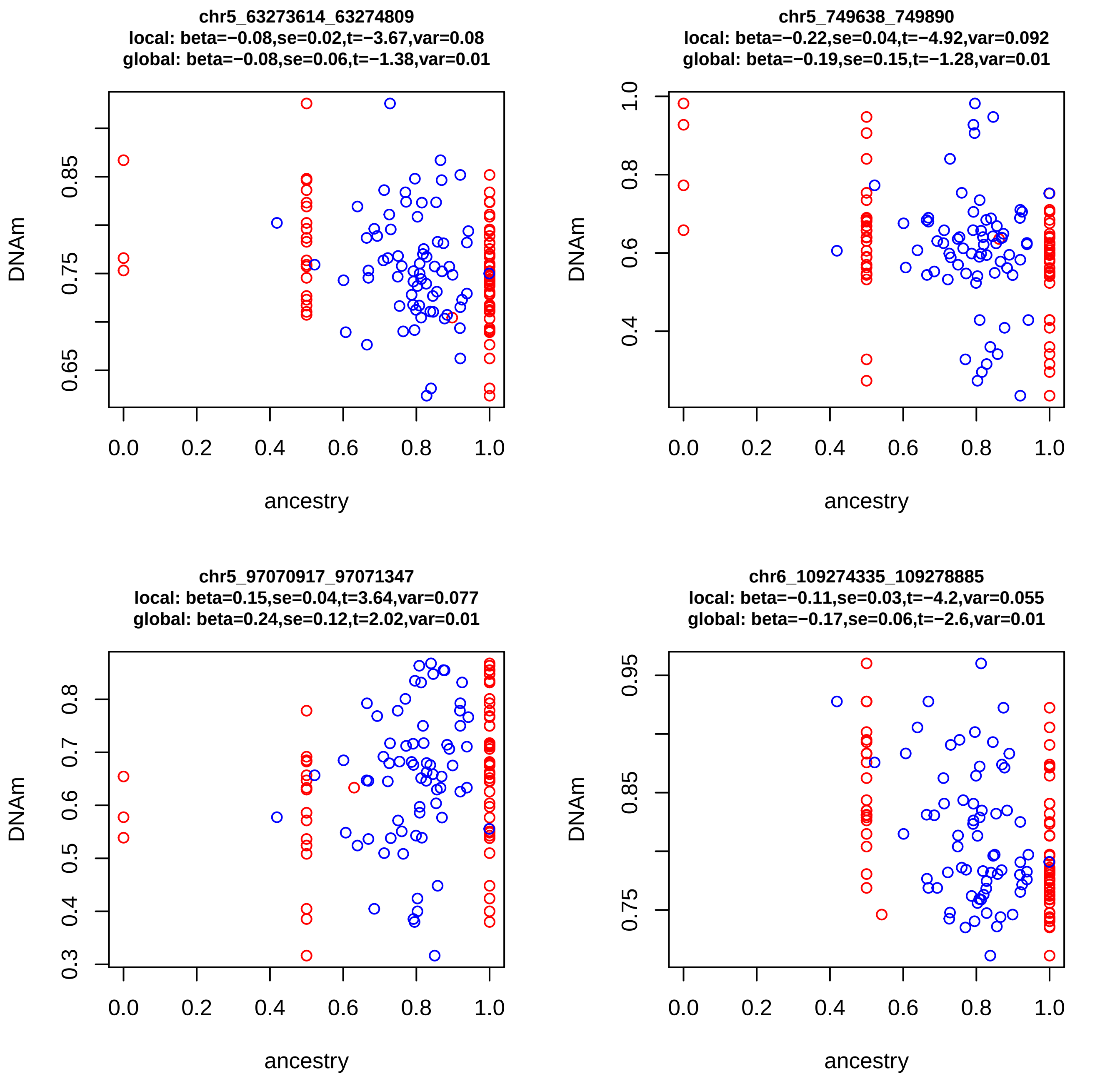
chr5_63273614_63274809
local: beta=−0.08,se=0.02,t=−3.67,var=0.08
global: beta=−0.08,se=0.06,t=−1.38,var=0.01
DNAm
0.65
0.75
0.85
0.0
0.2
0.4
0.6
0.8
1.0
ancestry
chr5_749638_749890
local: beta=−0.22,se=0.04,t=−4.92,var=0.092
global: beta=−0.19,se=0.15,t=−1.28,var=0.01
DNAm
0.4
0.6
0.8
1.0
0.0
0.2
0.4
0.6
0.8
1.0
ancestry
chr5_97070917_97071347
local: beta=0.15,se=0.04,t=3.64,var=0.077
global: beta=0.24,se=0.12,t=2.02,var=0.01
DNAm
0.3
0.4
0.5
0.6
0.7
0.8
0.0
0.2
0.4
0.6
0.8
1.0
ancestry
chr6_109274335_109278885
local: beta=−0.11,se=0.03,t=−4.2,var=0.055
global: beta=−0.17,se=0.06,t=−2.6,var=0.01
DNAm
0.75
0.85
0.95
0.0
0.2
0.4
0.6
0.8
1.0
ancestry

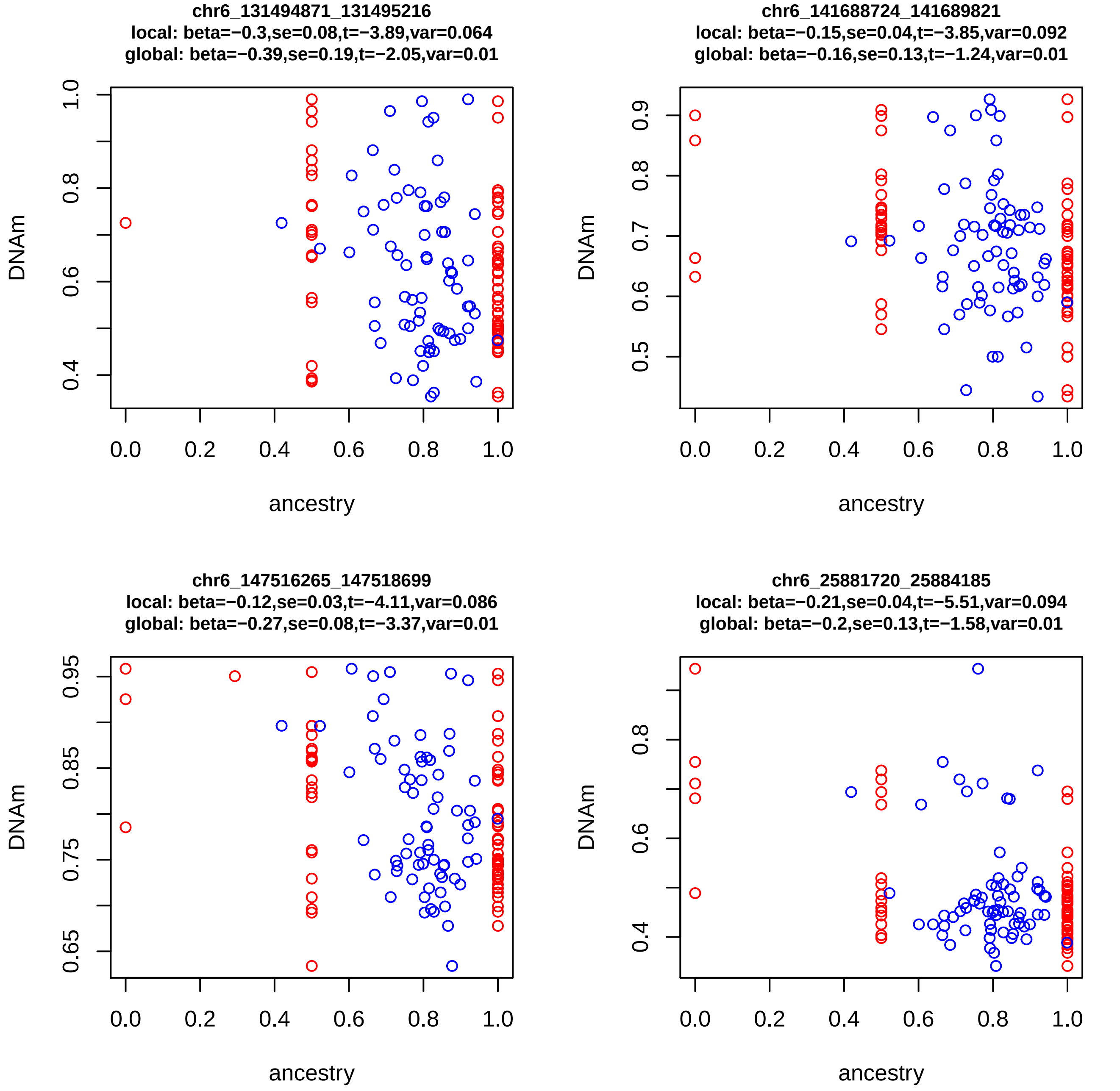
chr6_131494871_131495216
local: beta=−0.3,se=0.08,t=−3.89,var=0.064
global: beta=−0.39,se=0.19,t=−2.05,var=0.01
DNAm
0.4
0.6
0.8
1.0
0.0
0.2
0.4
0.6
0.8
1.0
ancestry
chr6_141688724_141689821
local: beta=−0.15,se=0.04,t=−3.85,var=0.092
global: beta=−0.16,se=0.13,t=−1.24,var=0.01
DNAm
0.5
0.6
0.7
0.8
0.9
0.0
0.2
0.4
0.6
0.8
1.0
ancestry
chr6_147516265_147518699
local: beta=−0.12,se=0.03,t=−4.11,var=0.086
global: beta=−0.27,se=0.08,t=−3.37,var=0.01
DNAm
0.65
0.75
0.85
0.95
0.0
0.2
0.4
0.6
0.8
1.0
ancestry
chr6_25881720_25884185
local: beta=−0.21,se=0.04,t=−5.51,var=0.094
global: beta=−0.2,se=0.13,t=−1.58,var=0.01
DNAm
0.4
0.6
0.8
0.0
0.2
0.4
0.6
0.8
1.0
ancestry

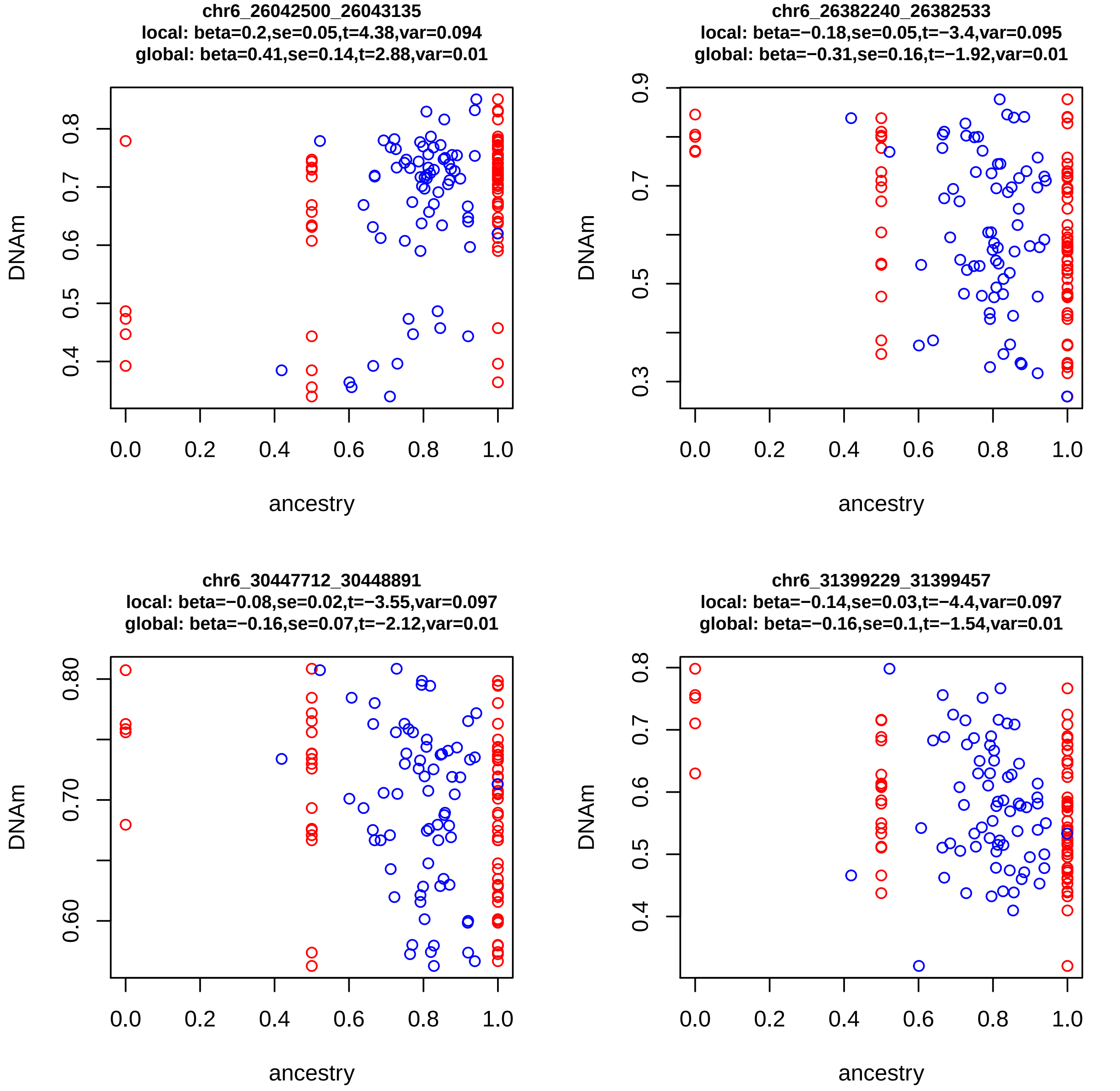
chr6_26042500_26043135
local: beta=0.2,se=0.05,t=4.38,var=0.094
global: beta=0.41,se=0.14,t=2.88,var=0.01
DNAm
ancestry
chr6_26382240_26382533
local: beta=−0.18,se=0.05,t=−3.4,var=0.095
global: beta=−0.31,se=0.16,t=−1.92,var=0.01
DNAm
ancestry
chr6_30447712_30448891
local: beta=−0.08,se=0.02,t=−3.55,var=0.097
global: beta=−0.16,se=0.07,t=−2.12,var=0.01
DNAm
ancestry
chr6_31399229_31399457
local: beta=−0.14,se=0.03,t=−4.4,var=0.097
global: beta=−0.16,se=0.1,t=−1.54,var=0.01
DNAm
ancestry

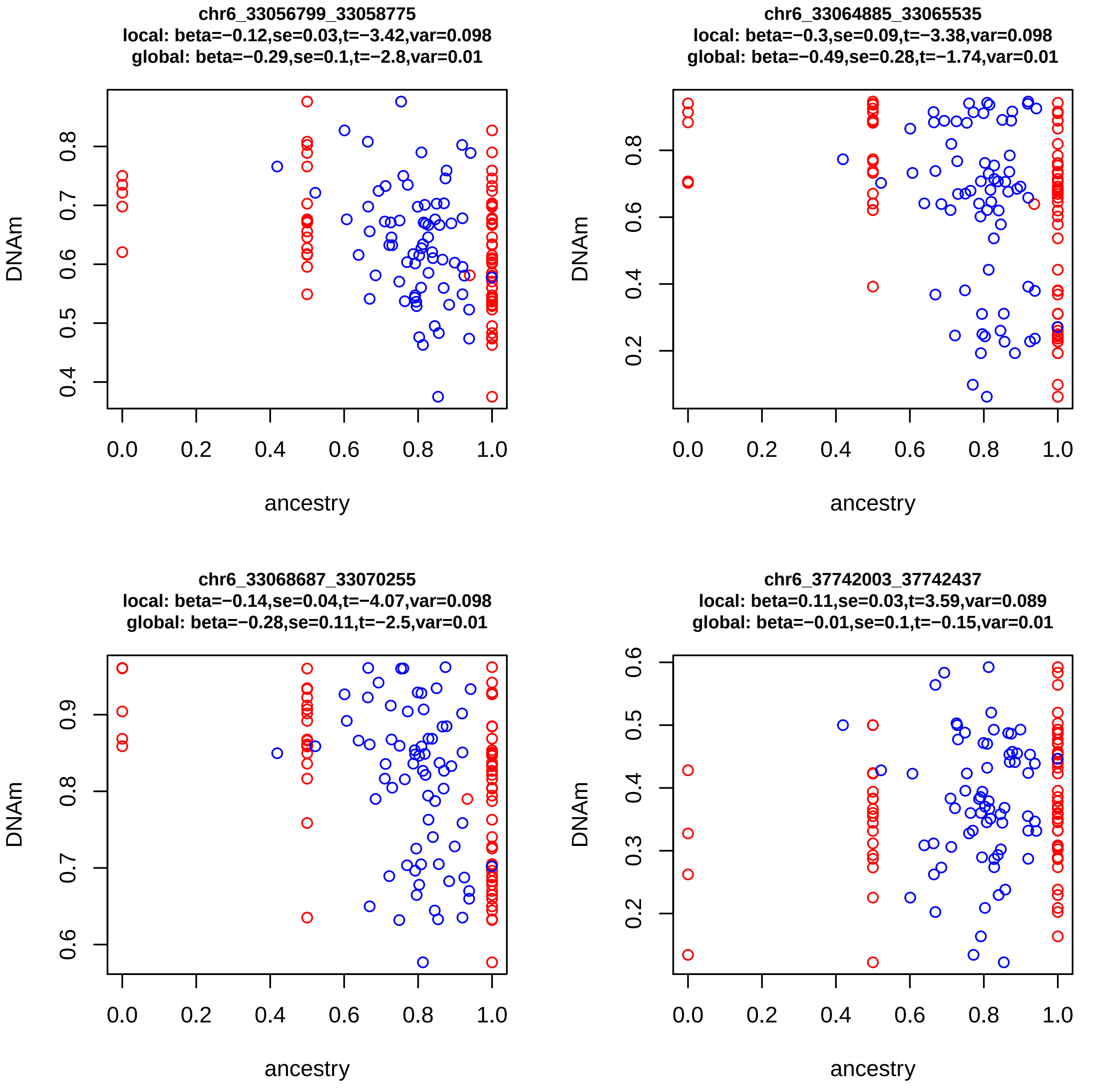
chr6_33056799_33058775
local: beta=−0.12,se=0.03,t=−3.42,var=0.098
global: beta=−0.29,se=0.1,t=−2.8,var=0.01
DNAm
ancestry
chr6_33064885_33065535
local: beta=−0.3,se=0.09,t=−3.38,var=0.098
global: beta=−0.49,se=0.28,t=−1.74,var=0.01
DNAm
ancestry
chr6_33068687_33070255
local: beta=−0.14,se=0.04,t=−4.07,var=0.098
global: beta=−0.28,se=0.11,t=−2.5,var=0.01
DNAm
ancestry
chr6_37742003_37742437
local: beta=0.11,se=0.03,t=3.59,var=0.089
global: beta=−0.01,se=0.1,t=−0.15,var=0.01
DNAm
ancestry

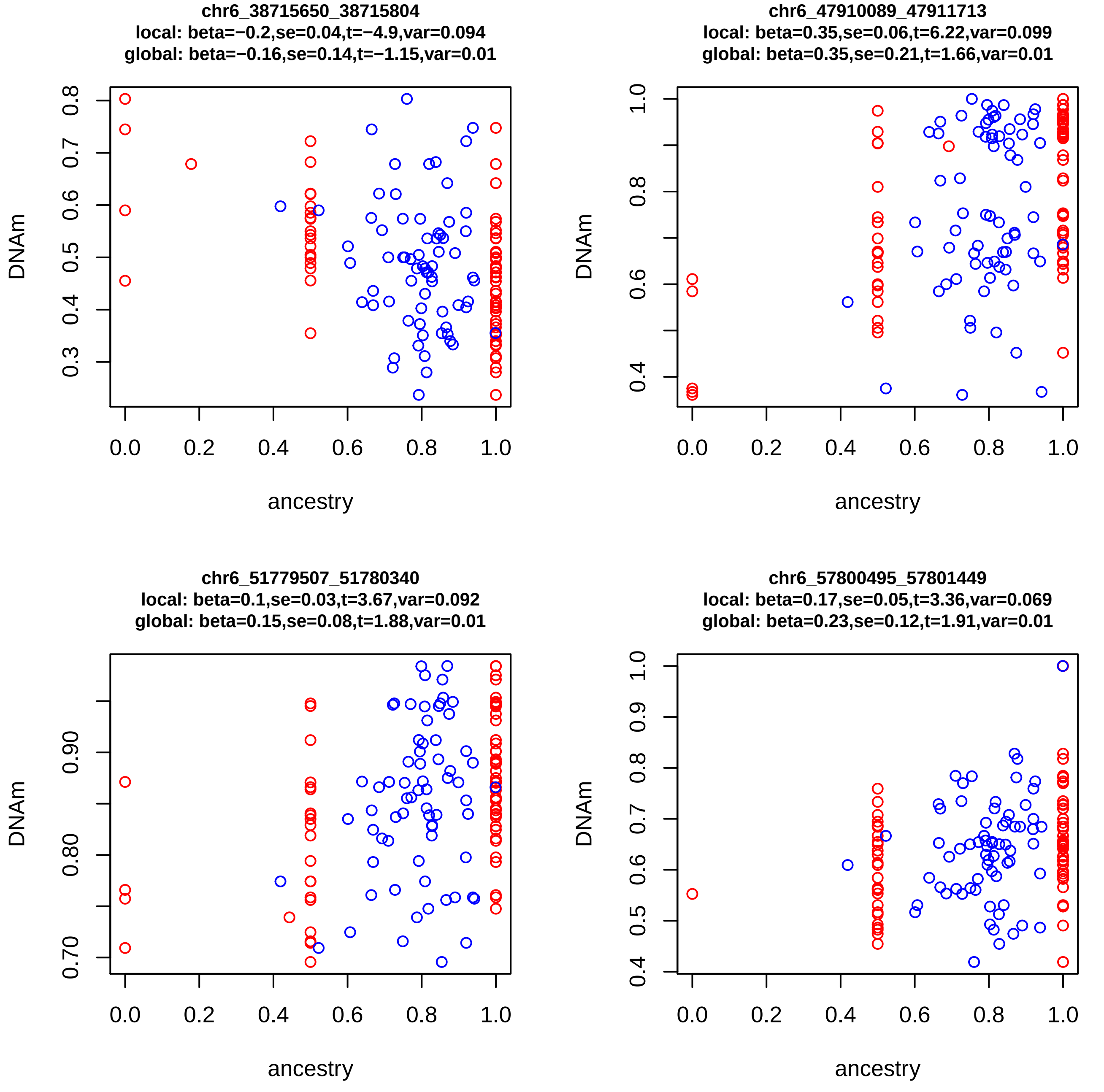
chr6_38715650_38715804
local: beta=−0.2,se=0.04,t=−4.9,var=0.094
global: beta=−0.16,se=0.14,t=−1.15,var=0.01
DNAm
0.3
0.4
0.5
0.6
0.7
0.8
0.0
0.2
0.4
0.6
0.8
1.0
ancestry
chr6_47910089_47911713
local: beta=0.35,se=0.06,t=6.22,var=0.099
global: beta=0.35,se=0.21,t=1.66,var=0.01
DNAm
0.4
0.6
0.8
1.0
0.0
0.2
0.4
0.6
0.8
1.0
ancestry
chr6_51779507_51780340
local: beta=0.1,se=0.03,t=3.67,var=0.092
global: beta=0.15,se=0.08,t=1.88,var=0.01
DNAm
0.70
0.80
0.90
0.0
0.2
0.4
0.6
0.8
1.0
ancestry
chr6_57800495_57801449
local: beta=0.17,se=0.05,t=3.36,var=0.069
global: beta=0.23,se=0.12,t=1.91,var=0.01
DNAm
0.4
0.5
0.6
0.7
0.8
0.9
1.0
0.0
0.2
0.4
0.6
0.8
1.0
ancestry

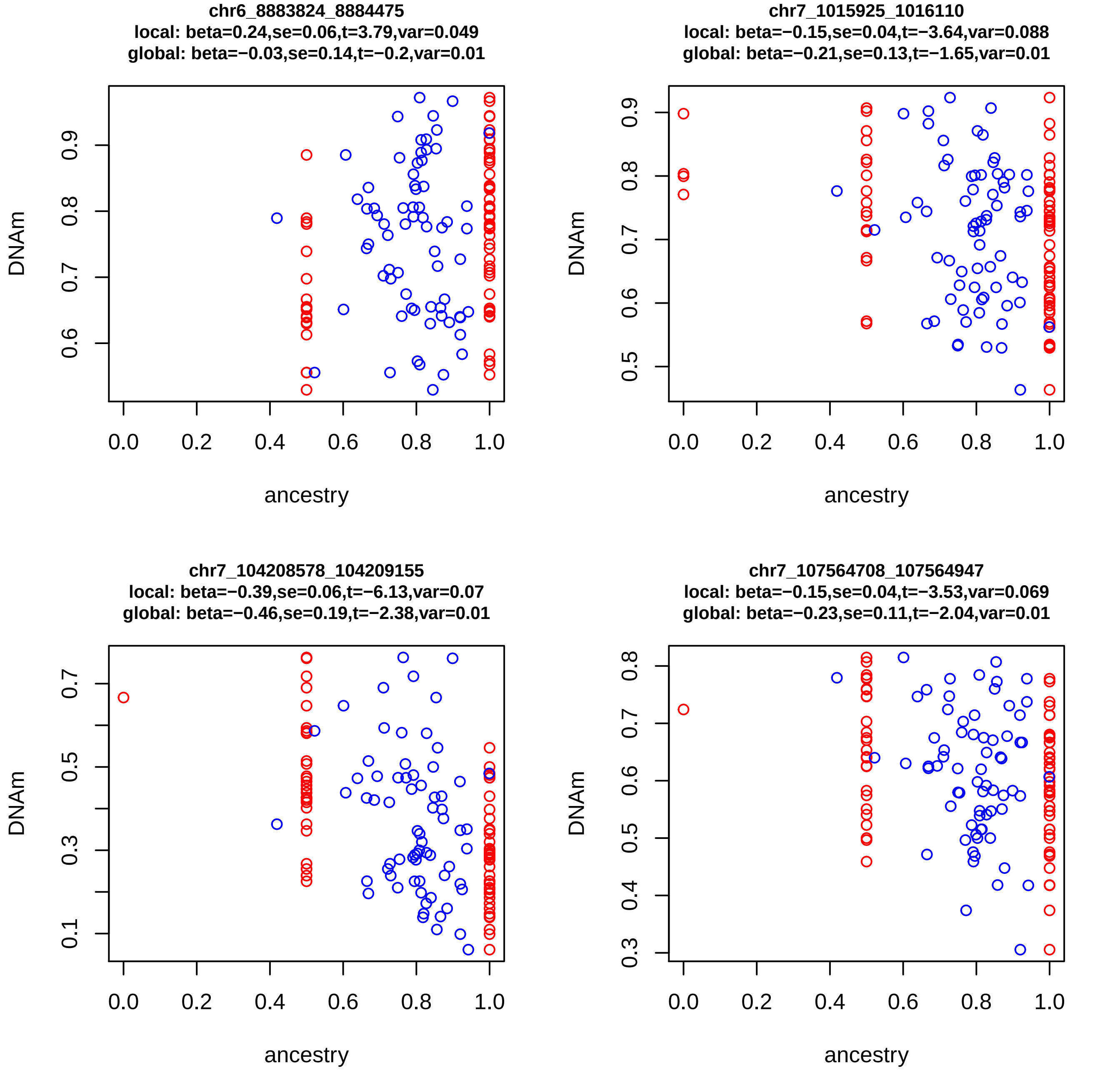
chr6_8883824_8884475
local: beta=0.24,se=0.06,t=3.79,var=0.049
global: beta=−0.03,se=0.14,t=−0.2,var=0.01
chr7_1015925_1016110
local: beta=−0.15,se=0.04,t=−3.64,var=0.088
global: beta=−0.21,se=0.13,t=−1.65,var=0.01
chr7_104208578_104209155
local: beta=−0.39,se=0.06,t=−6.13,var=0.07
global: beta=−0.46,se=0.19,t=−2.38,var=0.01
chr7_107564708_107564947
local: beta=−0.15,se=0.04,t=−3.53,var=0.069
global: beta=−0.23,se=0.11,t=−2.04,var=0.01
DNAm
ancestry
0.0
0.2
0.4
0.6
0.8
1.0

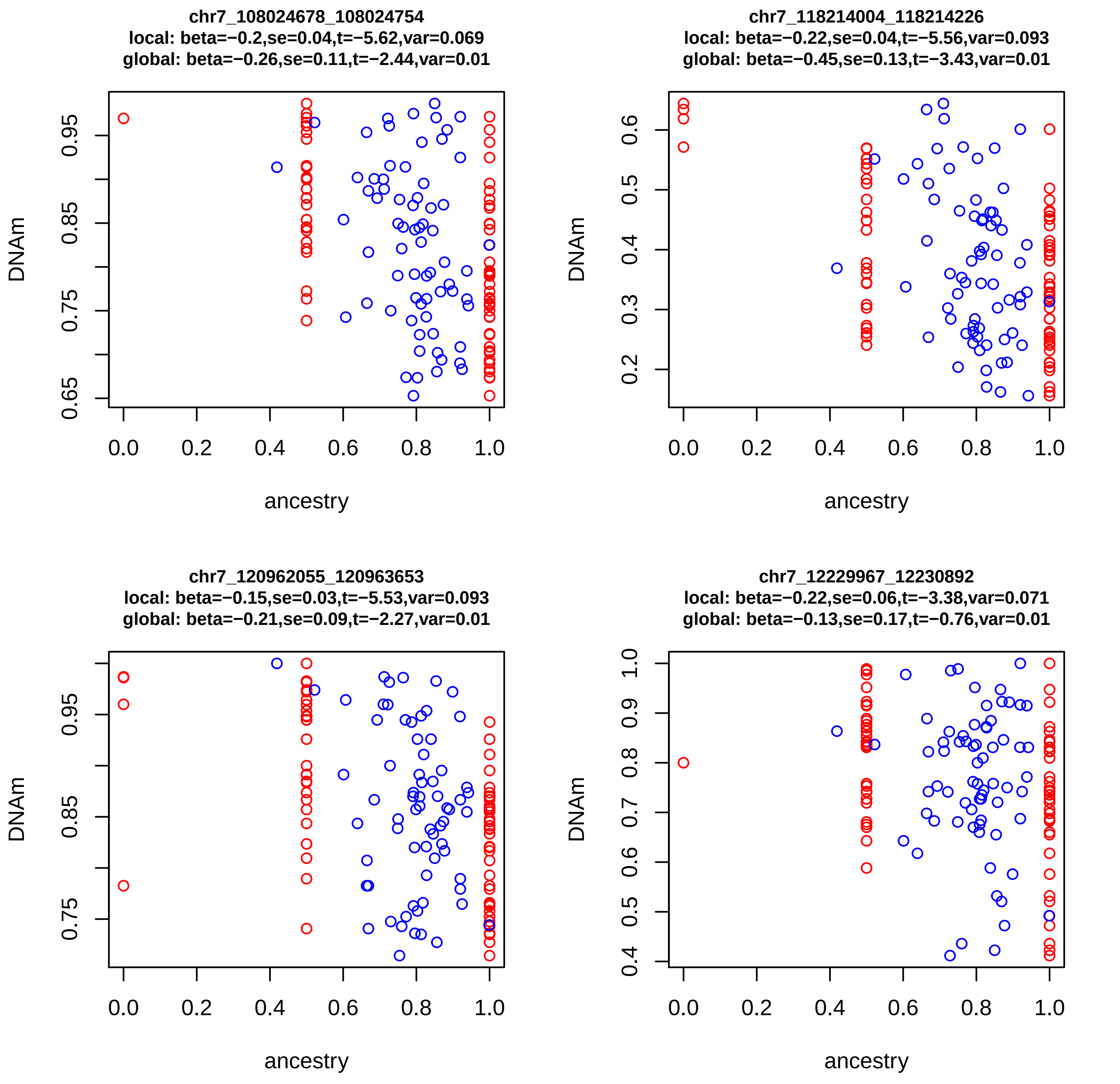

chr7_108024678_108024754
local: beta=−0.2,se=0.04,t=−5.62,var=0.069
global: beta=−0.26,se=0.11,t=−2.44,var=0.01
DNAm
0.65
0.75
0.85
0.95
0.0
0.2
0.4
0.6
0.8
1.0
ancestry
chr7_118214004_118214226
local: beta=−0.22,se=0.04,t=−5.56,var=0.093
global: beta=−0.45,se=0.13,t=−3.43,var=0.01
DNAm
0.2
0.3
0.4
0.5
0.6
ancestry
chr7_120962055_120963653
local: beta=−0.15,se=0.03,t=−5.53,var=0.093
global: beta=−0.21,se=0.09,t=−2.27,var=0.01
DNAm
0.75
0.85
0.95
ancestry
chr7_12229967_12230892
local: beta=−0.22,se=0.06,t=−3.38,var=0.071
global: beta=−0.13,se=0.17,t=−0.76,var=0.01
DNAm
0.4
0.5
0.6
0.7
0.8
0.9
1.0
ancestry

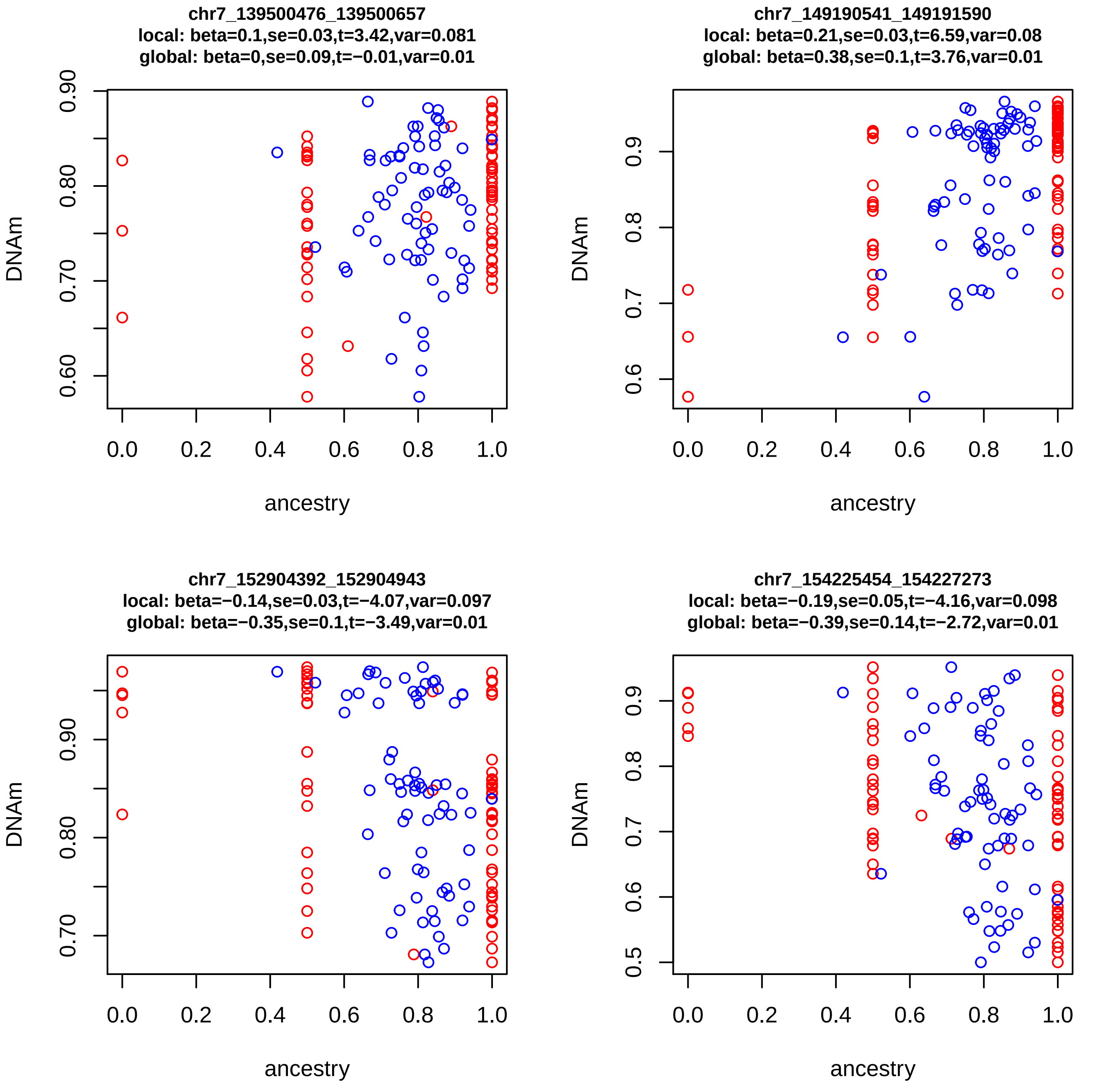
chr7_139500476_139500657
local: beta=0.1,se=0.03,t=3.42,var=0.081
global: beta=0,se=0.09,t=−0.01,var=0.01
chr7_149190541_149191590
local: beta=0.21,se=0.03,t=6.59,var=0.08
global: beta=0.38,se=0.1,t=3.76,var=0.01
chr7_152904392_152904943
local: beta=−0.14,se=0.03,t=−4.07,var=0.097
global: beta=−0.35,se=0.1,t=−3.49,var=0.01
chr7_154225454_154227273
local: beta=−0.19,se=0.05,t=−4.16,var=0.098
global: beta=−0.39,se=0.14,t=−2.72,var=0.01
DNAm
ancestry

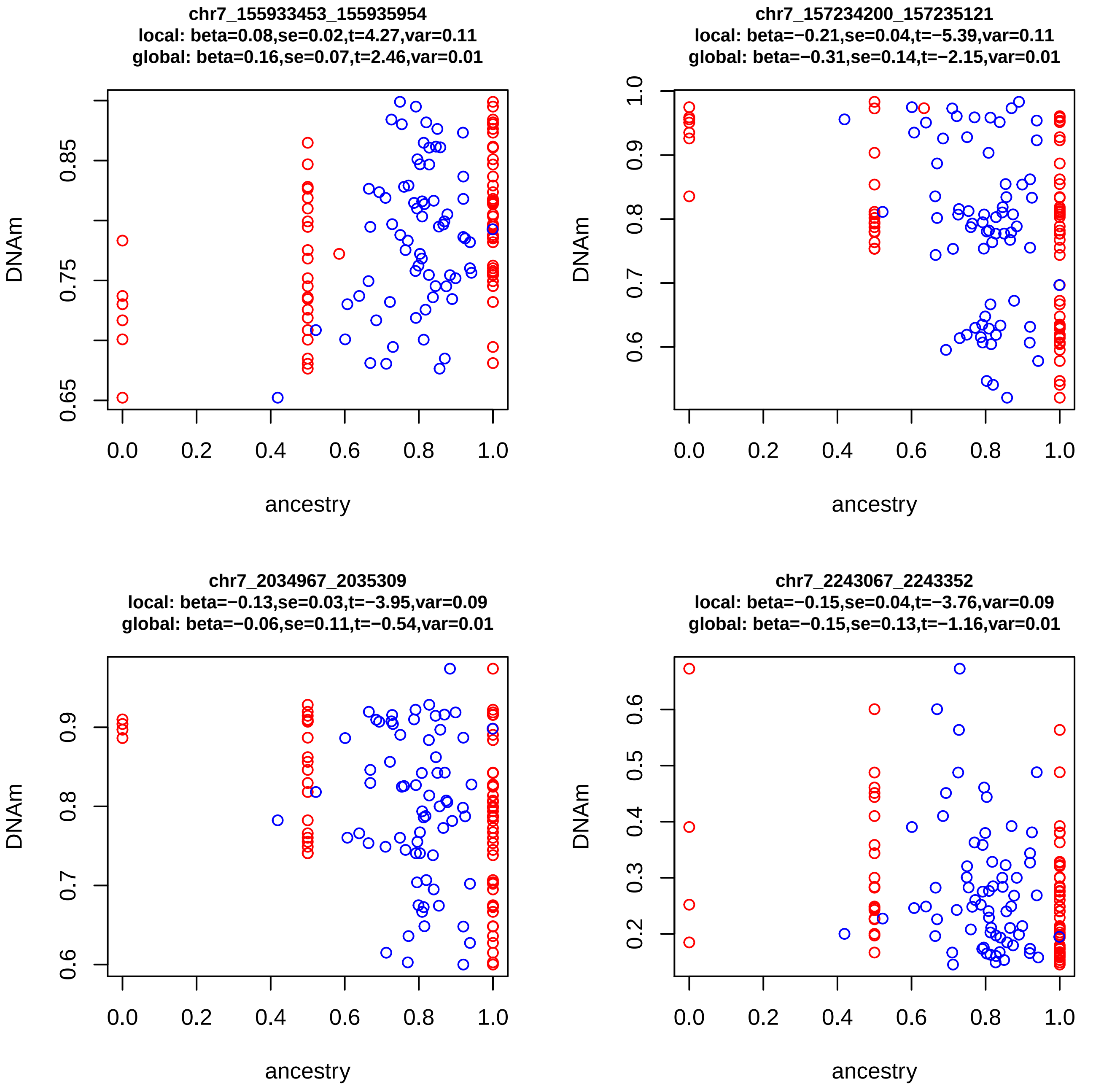
chr7_155933453_155935954
local: beta=0.08,se=0.02,t=4.27,var=0.11
global: beta=0.16,se=0.07,t=2.46,var=0.01
DNAm
0.65
0.75
0.85
0.0
0.2
0.4
0.6
0.8
1.0
ancestry
chr7_157234200_157235121
local: beta=−0.21,se=0.04,t=−5.39,var=0.11
global: beta=−0.31,se=0.14,t=−2.15,var=0.01
DNAm
0.6
0.7
0.8
0.9
1.0
0.0
0.2
0.4
0.6
0.8
1.0
ancestry
chr7_2034967_2035309
local: beta=−0.13,se=0.03,t=−3.95,var=0.09
global: beta=−0.06,se=0.11,t=−0.54,var=0.01
DNAm
0.6
0.7
0.8
0.9
0.0
0.2
0.4
0.6
0.8
1.0
ancestry
chr7_2243067_2243352
local: beta=−0.15,se=0.04,t=−3.76,var=0.09
global: beta=−0.15,se=0.13,t=−1.16,var=0.01
DNAm
0.2
0.3
0.4
0.5
0.6
0.0
0.2
0.4
0.6
0.8
1.0
ancestry

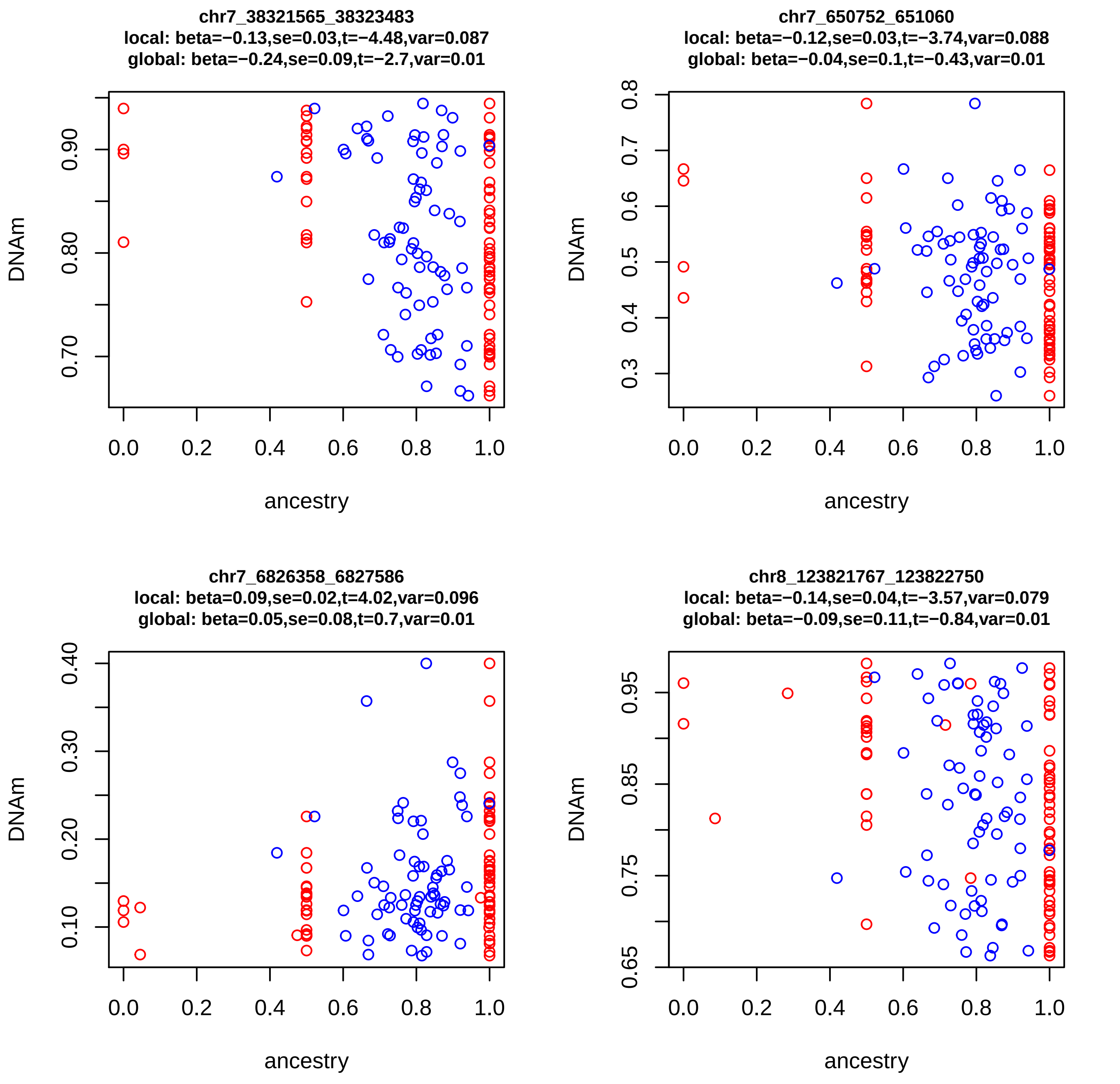
chr7_38321565_38323483
local: beta=−0.13,se=0.03,t=−4.48,var=0.087
global: beta=−0.24,se=0.09,t=−2.7,var=0.01
DNAm
0.70
0.80
0.90
0.0
0.2
0.4
0.6
0.8
1.0
ancestry
chr7_650752_651060
local: beta=−0.12,se=0.03,t=−3.74,var=0.088
global: beta=−0.04,se=0.1,t=−0.43,var=0.01
DNAm
0.3
0.4
0.5
0.6
0.7
0.8
0.0
0.2
0.4
0.6
0.8
1.0
ancestry
chr7_6826358_6827586
local: beta=0.09,se=0.02,t=4.02,var=0.096
global: beta=0.05,se=0.08,t=0.7,var=0.01
DNAm
0.10
0.20
0.30
0.40
0.0
0.2
0.4
0.6
0.8
1.0
ancestry
chr8_123821767_123822750
local: beta=−0.14,se=0.04,t=−3.57,var=0.079
global: beta=−0.09,se=0.11,t=−0.84,var=0.01
DNAm
0.65
0.75
0.85
0.95
0.0
0.2
0.4
0.6
0.8
1.0
ancestry

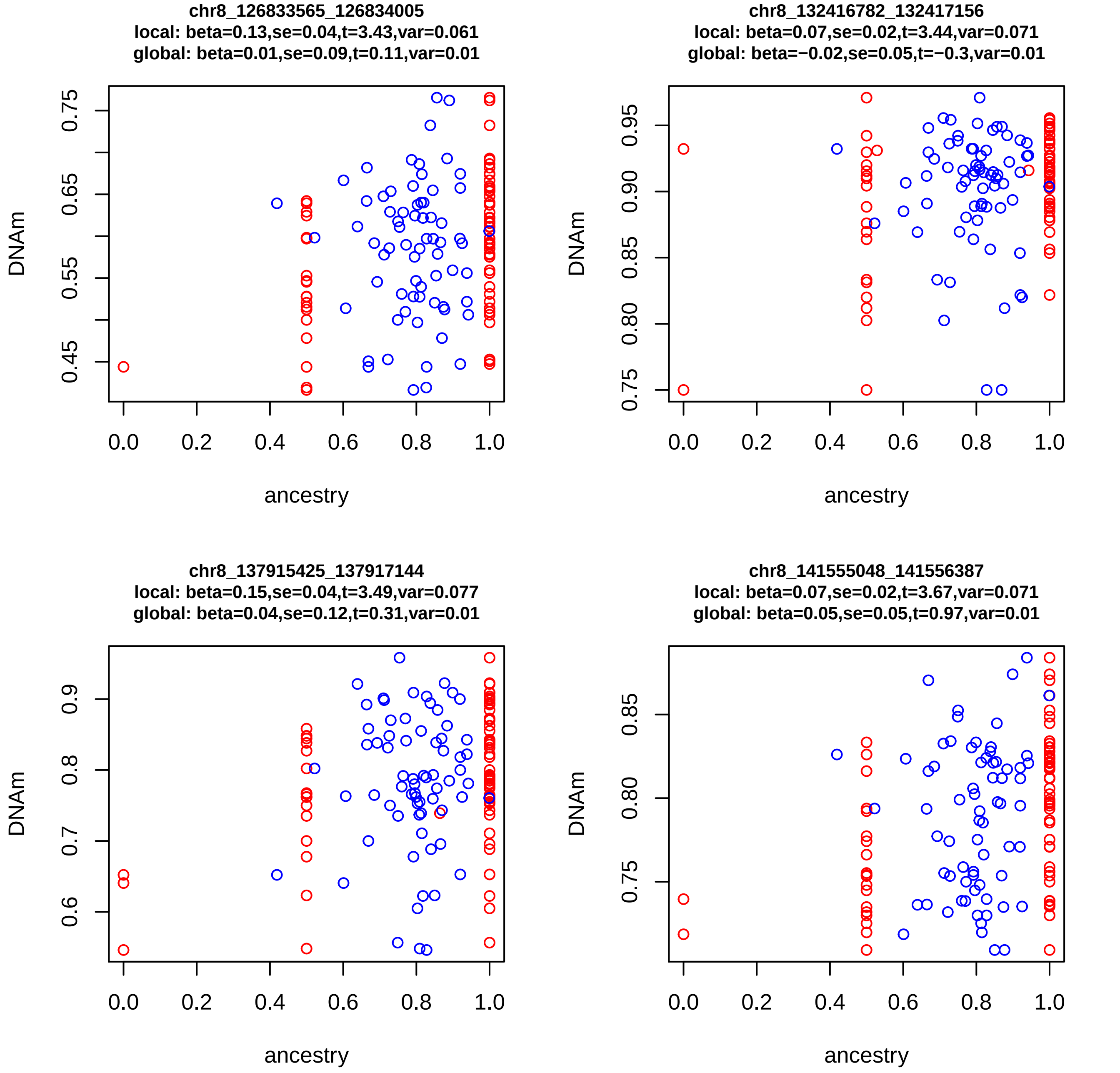
chr8_126833565_126834005
local: beta=0.13,se=0.04,t=3.43,var=0.061
global: beta=0.01,se=0.09,t=0.11,var=0.01
DNAm
0.45
0.55
0.65
0.75
0.0
0.2
0.4
0.6
0.8
1.0
ancestry
chr8_132416782_132417156
local: beta=0.07,se=0.02,t=3.44,var=0.071
global: beta=−0.02,se=0.05,t=−0.3,var=0.01
DNAm
0.75
0.80
0.85
0.90
0.95
0.0
0.2
0.4
0.6
0.8
1.0
ancestry
chr8_137915425_137917144
local: beta=0.15,se=0.04,t=3.49,var=0.077
global: beta=0.04,se=0.12,t=0.31,var=0.01
DNAm
0.6
0.7
0.8
0.9
0.0
0.2
0.4
0.6
0.8
1.0
ancestry
chr8_141555048_141556387
local: beta=0.07,se=0.02,t=3.67,var=0.071
global: beta=0.05,se=0.05,t=0.97,var=0.01
DNAm
0.75
0.80
0.85
0.0
0.2
0.4
0.6
0.8
1.0
ancestry

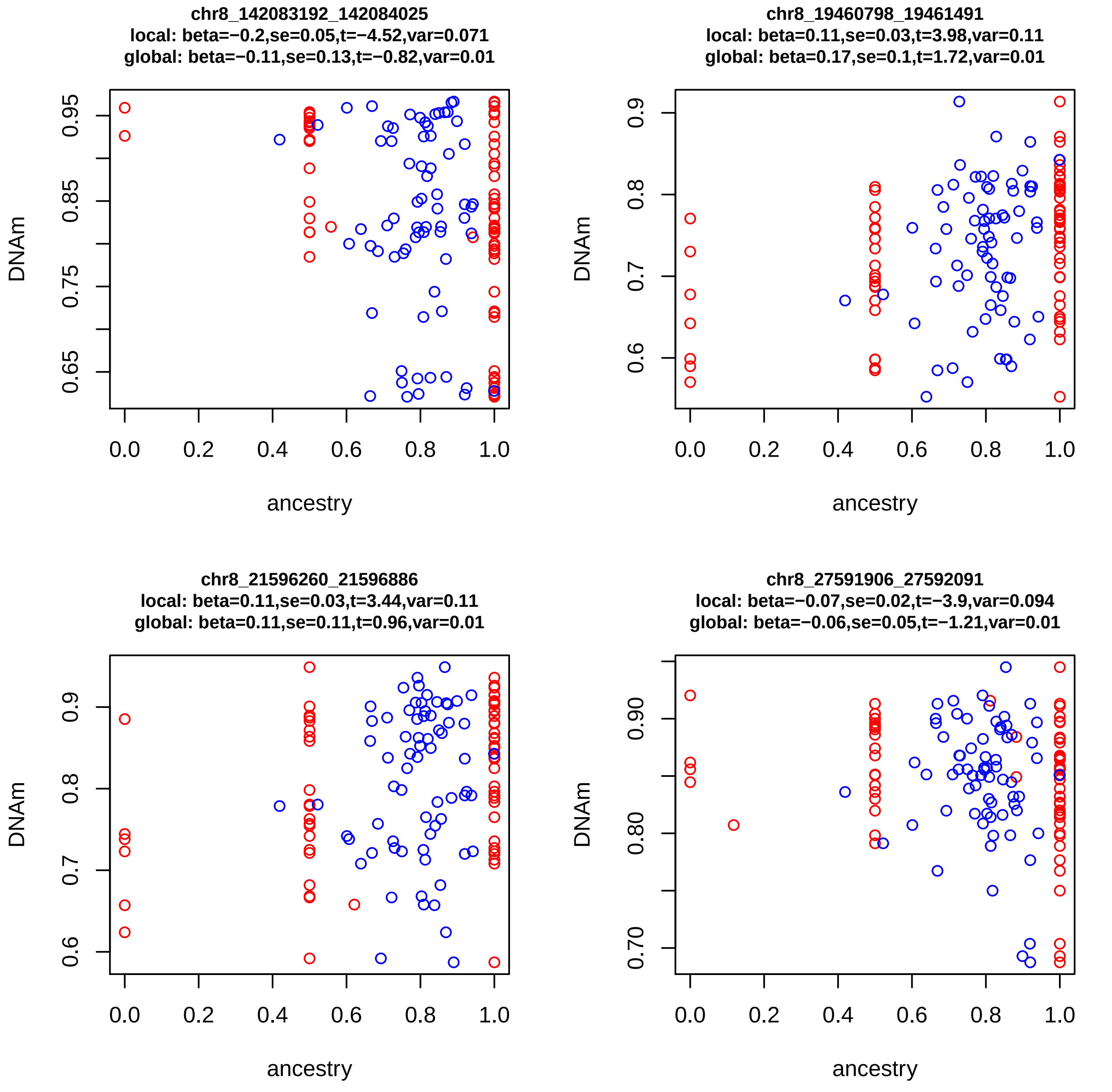
chr8_142083192_142084025
local: beta=−0.2,se=0.05,t=−4.52,var=0.071
global: beta=−0.11,se=0.13,t=−0.82,var=0.01
DNAm
0.65
0.75
0.85
0.95
0.0
0.2
0.4
0.6
0.8
1.0
ancestry
chr8_19460798_19461491
local: beta=0.11,se=0.03,t=3.98,var=0.11
global: beta=0.17,se=0.1,t=1.72,var=0.01
DNAm
0.6
0.7
0.8
0.9
0.0
0.2
0.4
0.6
0.8
1.0
ancestry
chr8_21596260_21596886
local: beta=0.11,se=0.03,t=3.44,var=0.11
global: beta=0.11,se=0.11,t=0.96,var=0.01
DNAm
0.6
0.7
0.8
0.9
0.0
0.2
0.4
0.6
0.8
1.0
ancestry
chr8_27591906_27592091
local: beta=−0.07,se=0.02,t=−3.9,var=0.094
global: beta=−0.06,se=0.05,t=−1.21,var=0.01
DNAm
0.70
0.80
0.90
0.0
0.2
0.4
0.6
0.8
1.0
ancestry

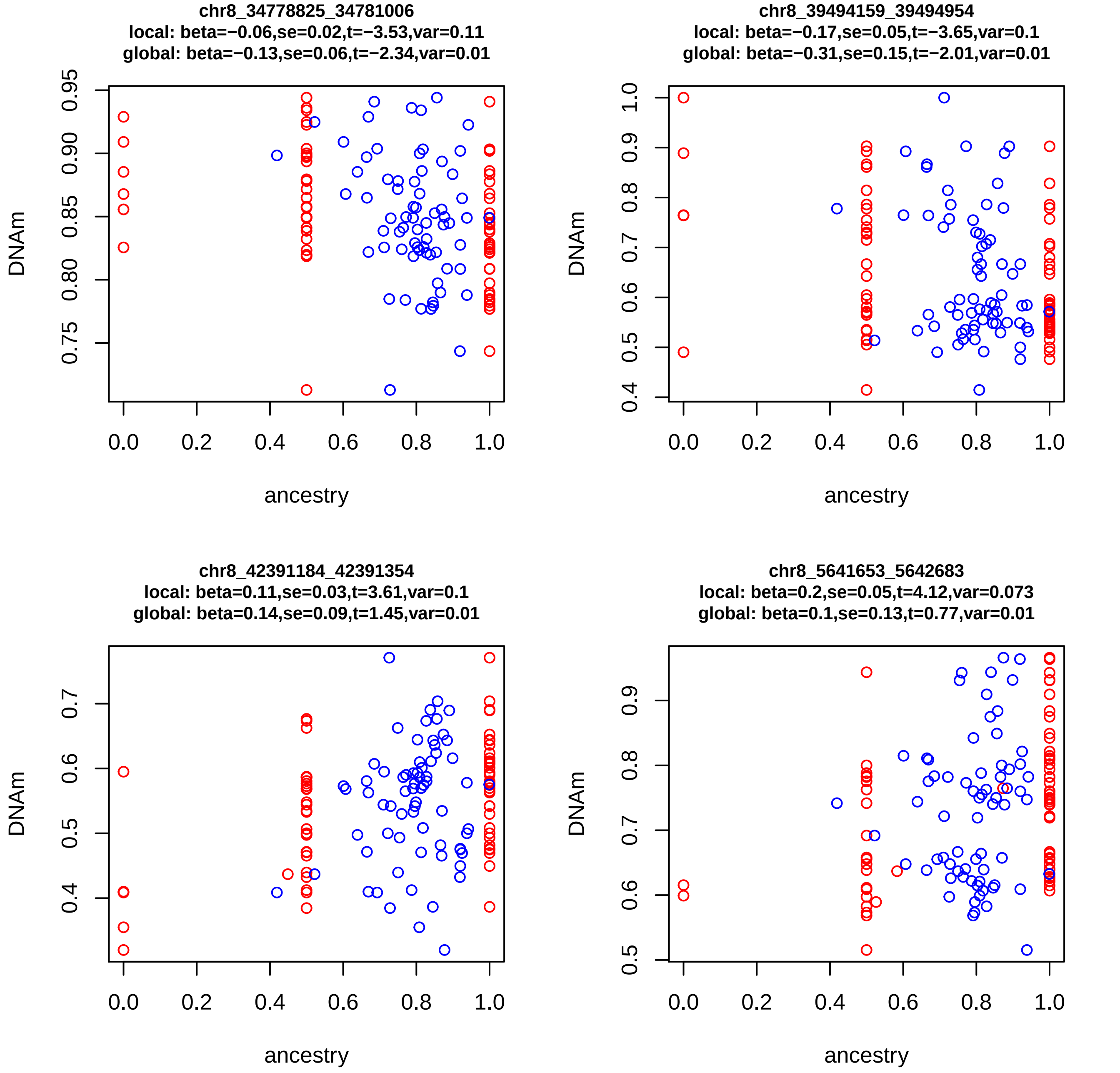
chr8_34778825_34781006
local: beta=−0.06,se=0.02,t=−3.53,var=0.11
global: beta=−0.13,se=0.06,t=−2.34,var=0.01
DNAm
ancestry
chr8_39494159_39494954
local: beta=−0.17,se=0.05,t=−3.65,var=0.1
global: beta=−0.31,se=0.15,t=−2.01,var=0.01
DNAm
ancestry
chr8_42391184_42391354
local: beta=0.11,se=0.03,t=3.61,var=0.1
global: beta=0.14,se=0.09,t=1.45,var=0.01
DNAm
ancestry
chr8_5641653_5642683
local: beta=0.2,se=0.05,t=4.12,var=0.073
global: beta=0.1,se=0.13,t=0.77,var=0.01
DNAm
ancestry

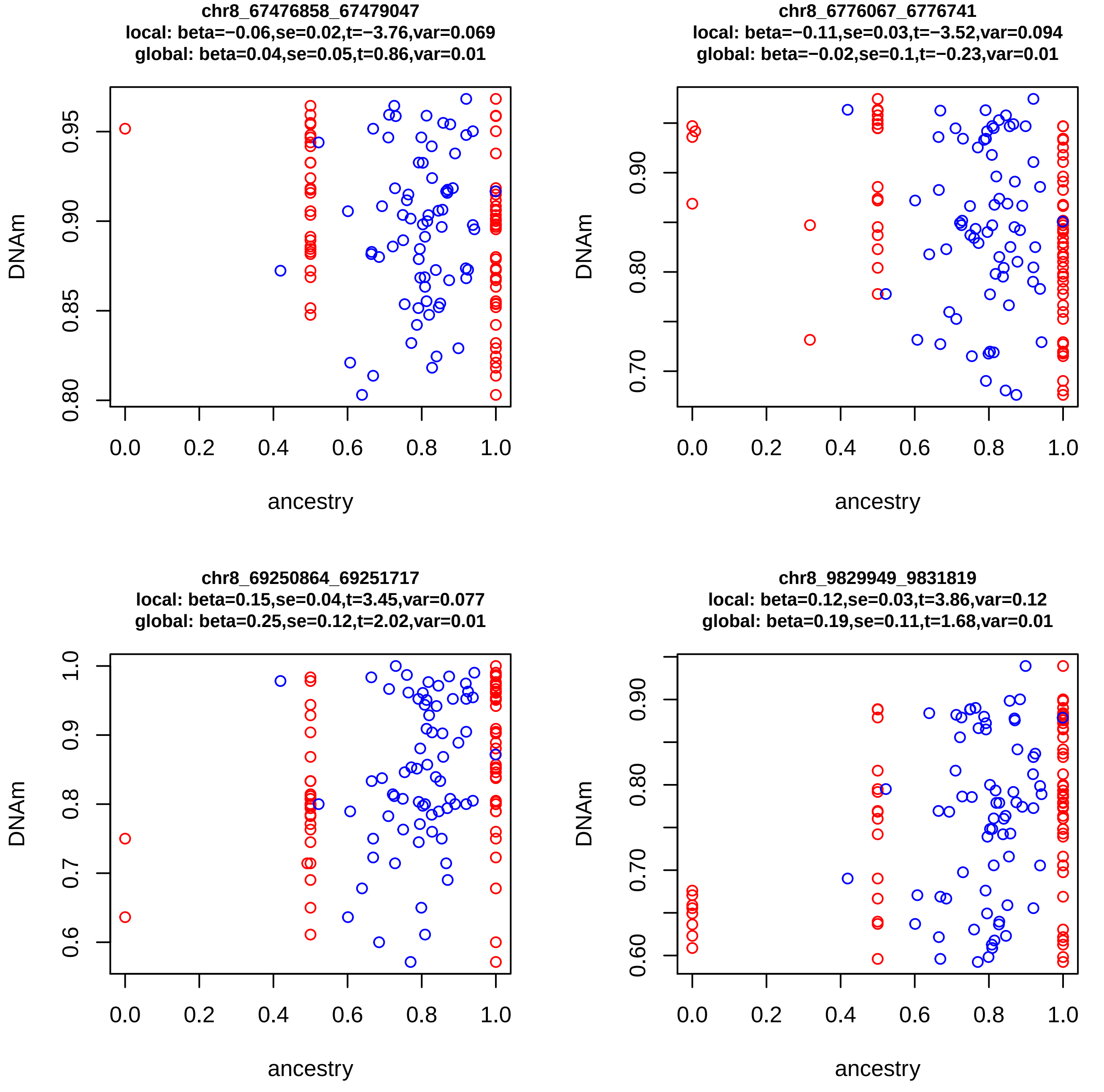
chr8_67476858_67479047
local: beta=−0.06,se=0.02,t=−3.76,var=0.069
global: beta=0.04,se=0.05,t=0.86,var=0.01
DNAm
0.80
0.85
0.90
0.95
0.0
0.2
0.4
0.6
0.8
1.0
ancestry
chr8_6776067_6776741
local: beta=−0.11,se=0.03,t=−3.52,var=0.094
global: beta=−0.02,se=0.1,t=−0.23,var=0.01
DNAm
0.70
0.80
0.90
0.0
0.2
0.4
0.6
0.8
1.0
ancestry
chr8_69250864_69251717
local: beta=0.15,se=0.04,t=3.45,var=0.077
global: beta=0.25,se=0.12,t=2.02,var=0.01
DNAm
0.6
0.7
0.8
0.9
1.0
0.0
0.2
0.4
0.6
0.8
1.0
ancestry
chr8_9829949_9831819
local: beta=0.12,se=0.03,t=3.86,var=0.12
global: beta=0.19,se=0.11,t=1.68,var=0.01
DNAm
0.60
0.70
0.80
0.90
0.0
0.2
0.4
0.6
0.8
1.0
ancestry

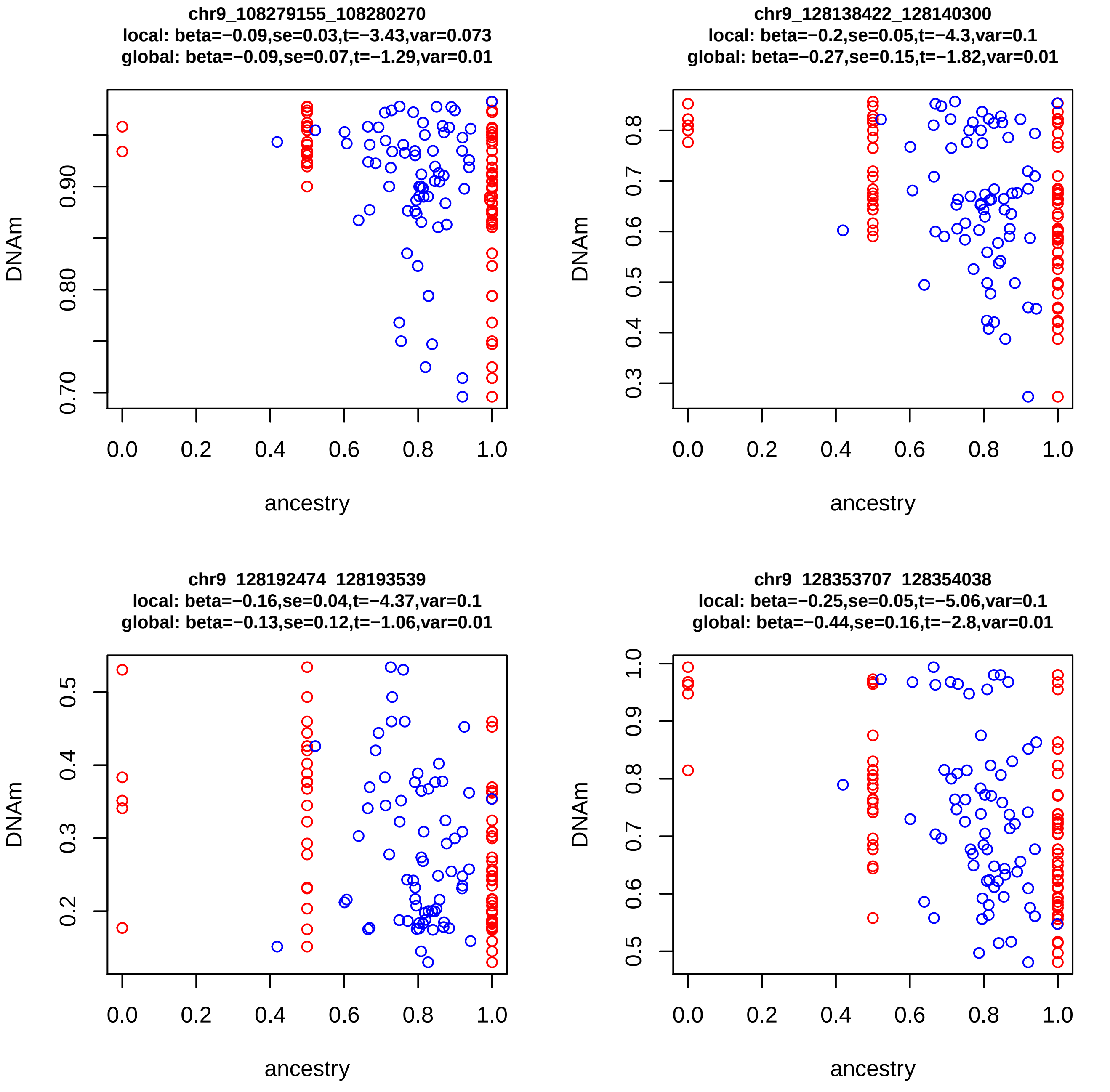
chr9_108279155_108280270
local: beta=−0.09,se=0.03,t=−3.43,var=0.073
global: beta=−0.09,se=0.07,t=−1.29,var=0.01
DNAm
ancestry
chr9_128138422_128140300
local: beta=−0.2,se=0.05,t=−4.3,var=0.1
global: beta=−0.27,se=0.15,t=−1.82,var=0.01
DNAm
ancestry
chr9_128192474_128193539
local: beta=−0.16,se=0.04,t=−4.37,var=0.1
global: beta=−0.13,se=0.12,t=−1.06,var=0.01
DNAm
ancestry
chr9_128353707_128354038
local: beta=−0.25,se=0.05,t=−5.06,var=0.1
global: beta=−0.44,se=0.16,t=−2.8,var=0.01
DNAm
ancestry

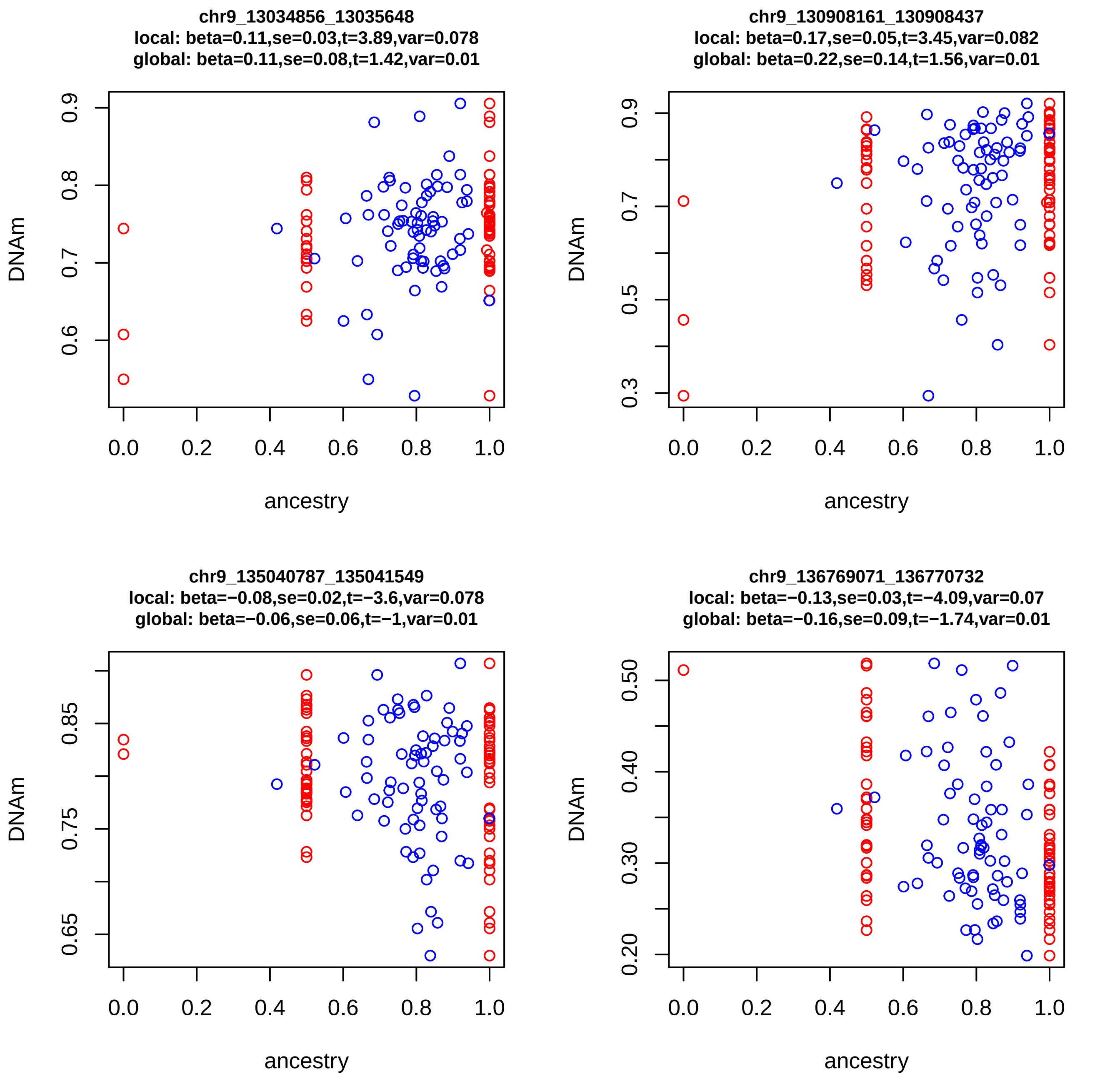
chr9_13034856_13035648
local: beta=0.11,se=0.03,t=3.89,var=0.078
global: beta=0.11,se=0.08,t=1.42,var=0.01
chr9_130908161_130908437
local: beta=0.17,se=0.05,t=3.45,var=0.082
global: beta=0.22,se=0.14,t=1.56,var=0.01
chr9_135040787_135041549
local: beta=−0.08,se=0.02,t=−3.6,var=0.078
global: beta=−0.06,se=0.06,t=−1,var=0.01
chr9_136769071_136770732
local: beta=−0.13,se=0.03,t=−4.09,var=0.07
global: beta=−0.16,se=0.09,t=−1.74,var=0.01
DNAm
ancestry
0.0
0.2
0.4
0.6
0.8
1.0

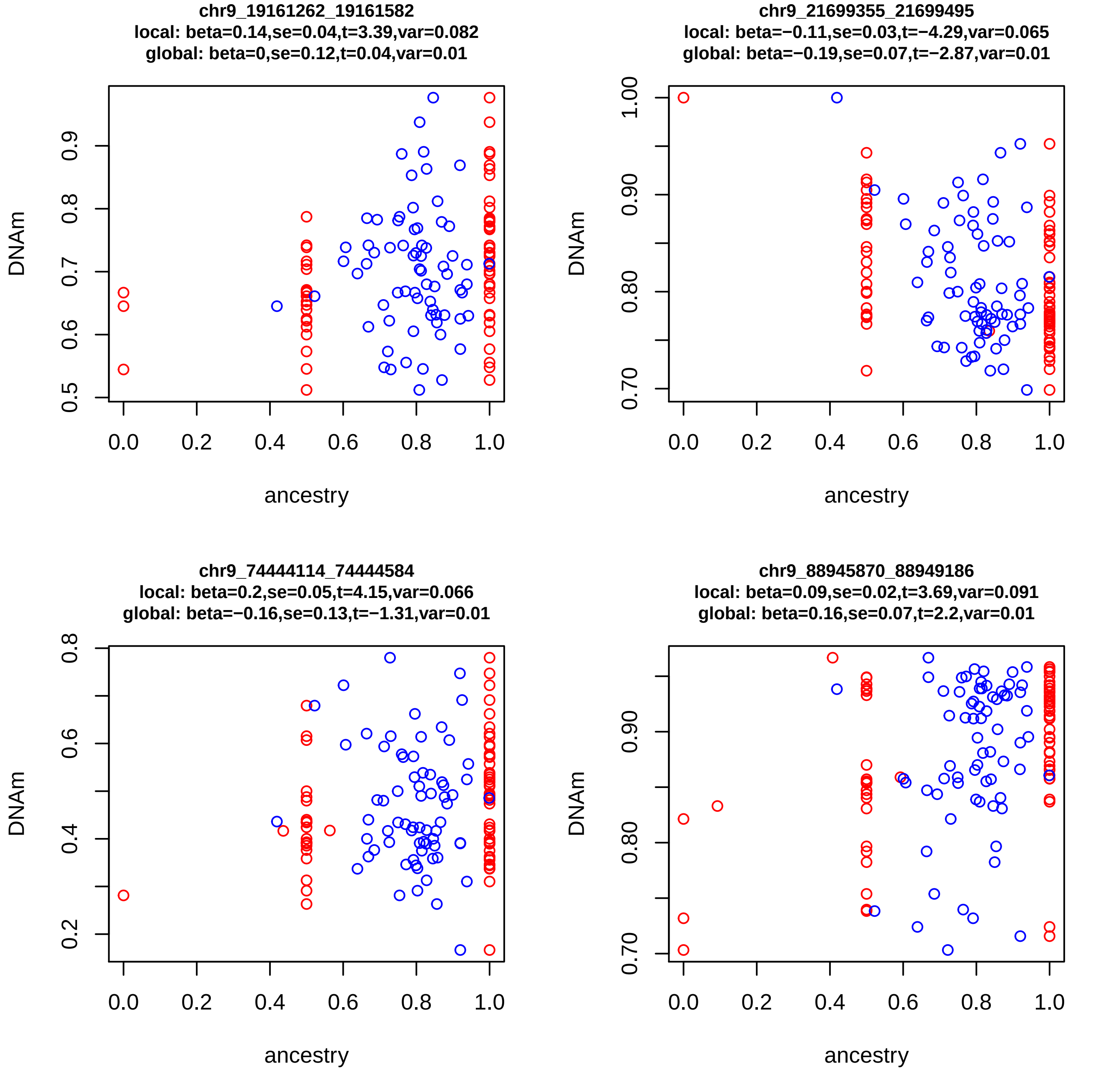
chr9_19161262_19161582
local: beta=0.14,se=0.04,t=3.39,var=0.082
global: beta=0,se=0.12,t=0.04,var=0.01
DNAm
ancestry
chr9_21699355_21699495
local: beta=−0.11,se=0.03,t=−4.29,var=0.065
global: beta=−0.19,se=0.07,t=−2.87,var=0.01
DNAm
ancestry
chr9_74444114_74444584
local: beta=0.2,se=0.05,t=4.15,var=0.066
global: beta=−0.16,se=0.13,t=−1.31,var=0.01
DNAm
ancestry
chr9_88945870_88949186
local: beta=0.09,se=0.02,t=3.69,var=0.091
global: beta=0.16,se=0.07,t=2.2,var=0.01
DNAm
ancestry
