# Supplementary material for: Analysis of gene expression in the postmortem brain of neurotypical Black Americans reveals contributions of genetic ancestry
Source: Nat Neurosci. 2024 May 20;27(6):1064–74. doi: 10.1038/s41593-024-01636-0 (PMC11156587; doi:10.1038/s41593-024-01636-0)
Supplement: Supplementary file 7 — Compressed directory of PDF of scatter plots comparing DNA methylation association with local and global ancestry for the caudate nucleus, DLPFC and hippocampus. Plots are annotated with the genetic ancestry DMR test results. [file 41593_2024_1636_MOESM7_ESM.gz › DMR_global_local_comparison/DMR_global_local_compare_by_region_caudate.pdf]

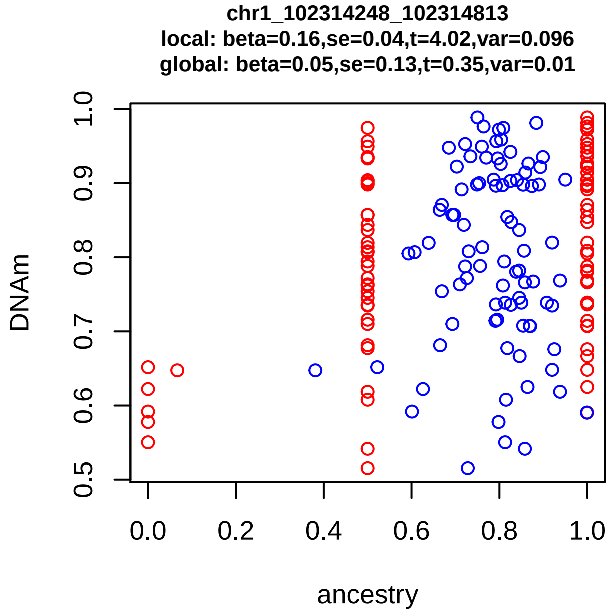
chr1_102314248_102314813
local: beta=0.16,se=0.04,t=4.02,var=0.096
global: beta=0.05,se=0.13,t=0.35,var=0.01
DNAm
0.5 0.6 0.7 0.8 0.9 1.0
0.0 0.2 0.4 0.6 0.8 1.0
ancestry

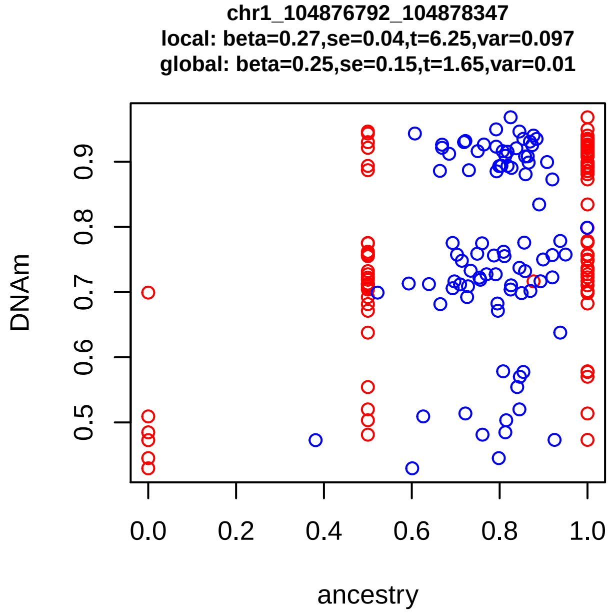
chr1_104876792_104878347
local: beta=0.27,se=0.04,t=6.25,var=0.097
global: beta=0.25,se=0.15,t=1.65,var=0.01
DNAm
0.5 0.6 0.7 0.8 0.9
0.0 0.2 0.4 0.6 0.8 1.0
ancestry

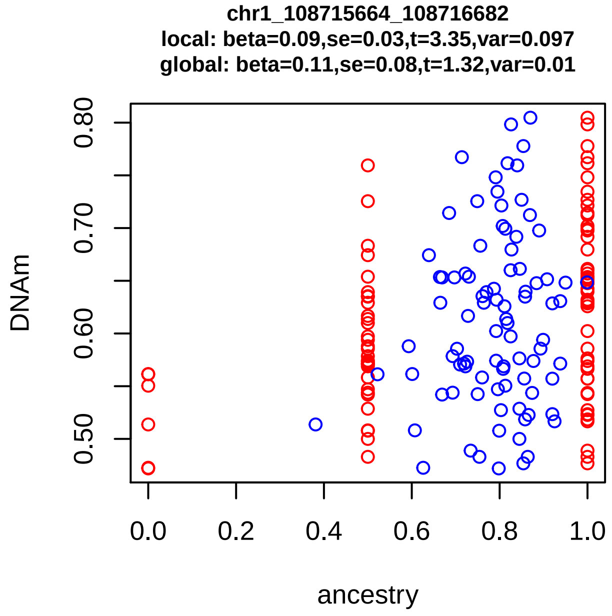
chr1_108715664_108716682
local: beta=0.09,se=0.03,t=3.35,var=0.097
global: beta=0.11,se=0.08,t=1.32,var=0.01
DNAm
0.50 0.60 0.70 0.80
0.0 0.2 0.4 0.6 0.8 1.0
ancestry

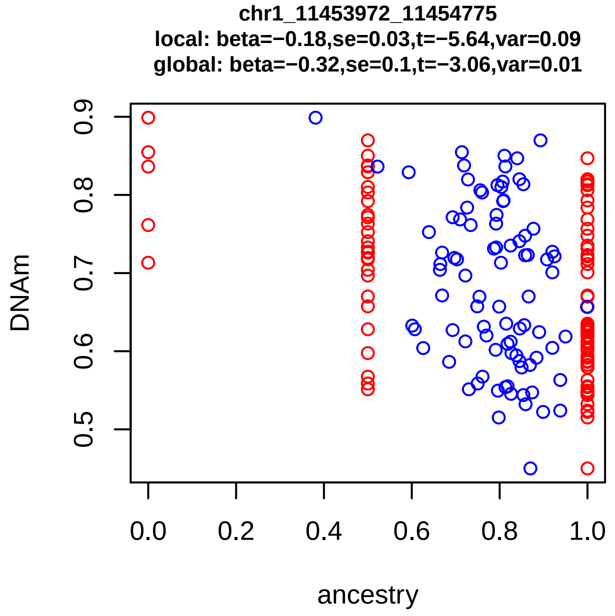
chr1_11453972_11454775
local: beta=−0.18,se=0.03,t=−5.64,var=0.09
global: beta=−0.32,se=0.1,t=−3.06,var=0.01
DNAm
0.5 0.6 0.7 0.8 0.9
0.0 0.2 0.4 0.6 0.8 1.0
ancestry

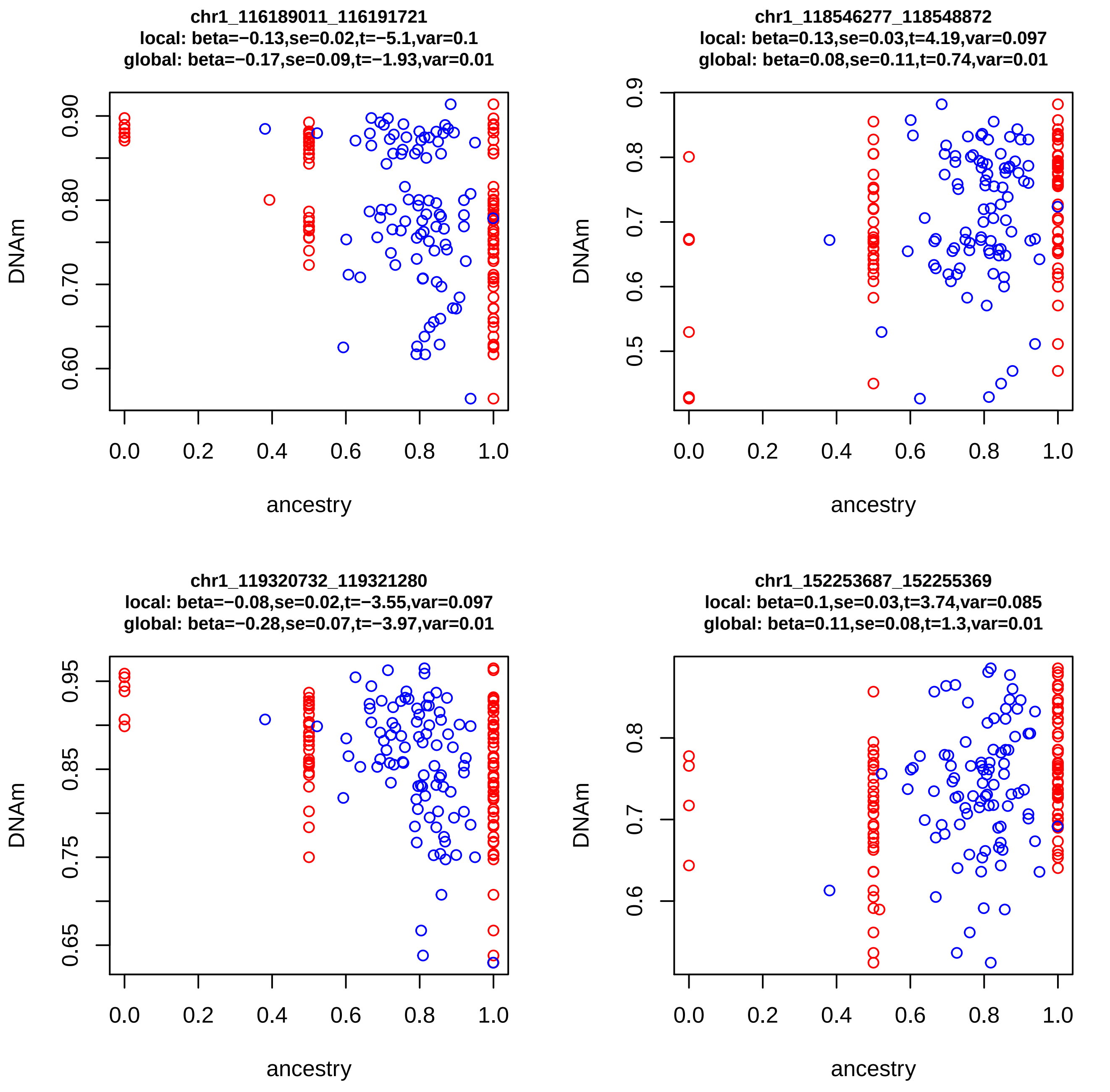
chr1_116189011_116191721
local: beta=−0.13,se=0.02,t=−5.1,var=0.1
global: beta=−0.17,se=0.09,t=−1.93,var=0.01
DNAm
0.60
0.70
0.80
0.90
0.0
0.2
0.4
0.6
0.8
1.0
ancestry
chr1_118546277_118548872
local: beta=0.13,se=0.03,t=4.19,var=0.097
global: beta=0.08,se=0.11,t=0.74,var=0.01
DNAm
0.5
0.6
0.7
0.8
0.9
ancestry
chr1_119320732_119321280
local: beta=−0.08,se=0.02,t=−3.55,var=0.097
global: beta=−0.28,se=0.07,t=−3.97,var=0.01
DNAm
0.65
0.75
0.85
0.95
ancestry
chr1_152253687_152255369
local: beta=0.1,se=0.03,t=3.74,var=0.085
global: beta=0.11,se=0.08,t=1.3,var=0.01
DNAm
0.6
0.7
0.8
ancestry

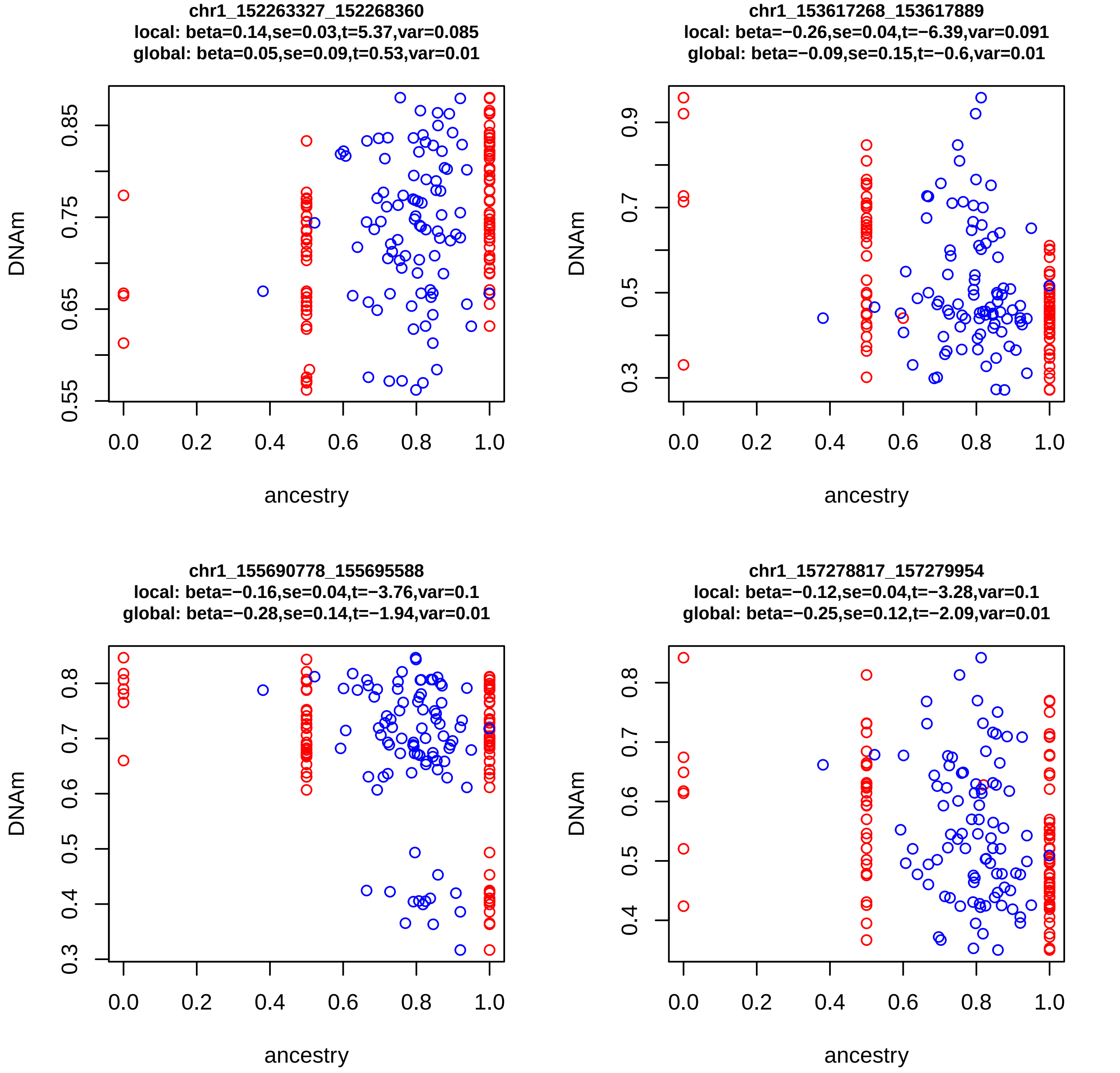
chr1_152263327_152268360
local: beta=0.14,se=0.03,t=5.37,var=0.085
global: beta=0.05,se=0.09,t=0.53,var=0.01
DNAm
0.55
0.65
0.75
0.85
0.0
0.2
0.4
0.6
0.8
1.0
ancestry
chr1_153617268_153617889
local: beta=−0.26,se=0.04,t=−6.39,var=0.091
global: beta=−0.09,se=0.15,t=−0.6,var=0.01
DNAm
0.3
0.5
0.7
0.9
0.0
0.2
0.4
0.6
0.8
1.0
ancestry
chr1_155690778_155695588
local: beta=−0.16,se=0.04,t=−3.76,var=0.1
global: beta=−0.28,se=0.14,t=−1.94,var=0.01
DNAm
0.3
0.4
0.5
0.6
0.7
0.8
0.0
0.2
0.4
0.6
0.8
1.0
ancestry
chr1_157278817_157279954
local: beta=−0.12,se=0.04,t=−3.28,var=0.1
global: beta=−0.25,se=0.12,t=−2.09,var=0.01
DNAm
0.4
0.5
0.6
0.7
0.8
0.0
0.2
0.4
0.6
0.8
1.0
ancestry

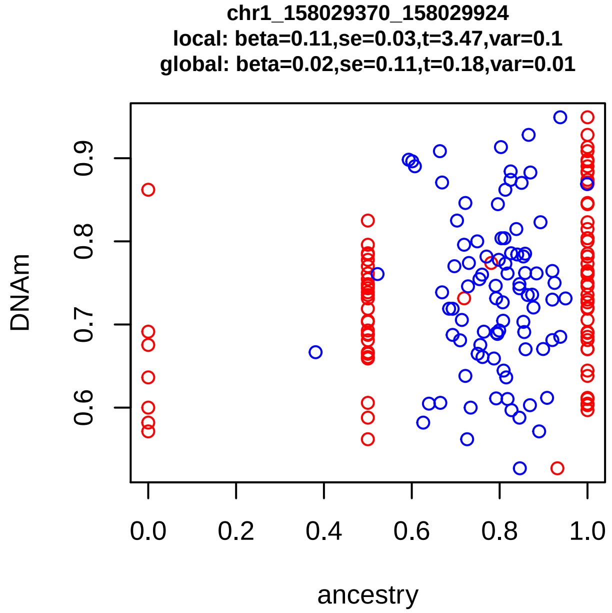
chr1_158029370_158029924
local: beta=0.11,se=0.03,t=3.47,var=0.1
global: beta=0.02,se=0.11,t=0.18,var=0.01
DNAm
0.6
0.7
0.8
0.9
0.0
0.2
0.4
0.6
0.8
1.0
ancestry

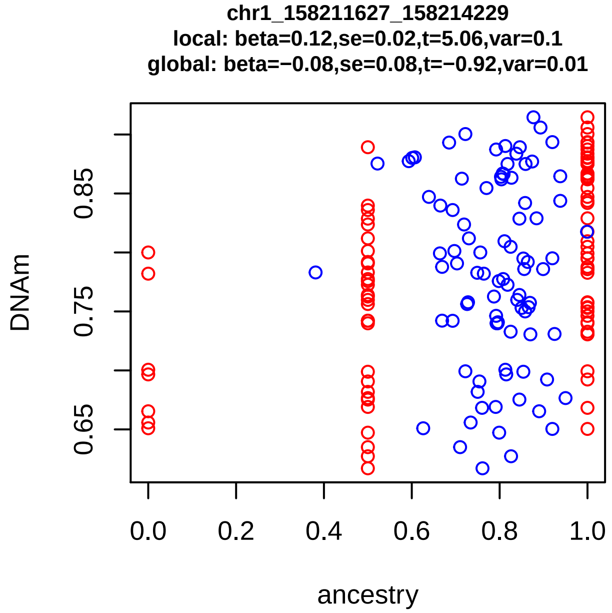
chr1_158211627_158214229
local: beta=0.12,se=0.02,t=5.06,var=0.1
global: beta=−0.08,se=0.08,t=−0.92,var=0.01
DNAm
0.65
0.75
0.85
0.0
0.2
0.4
0.6
0.8
1.0
ancestry

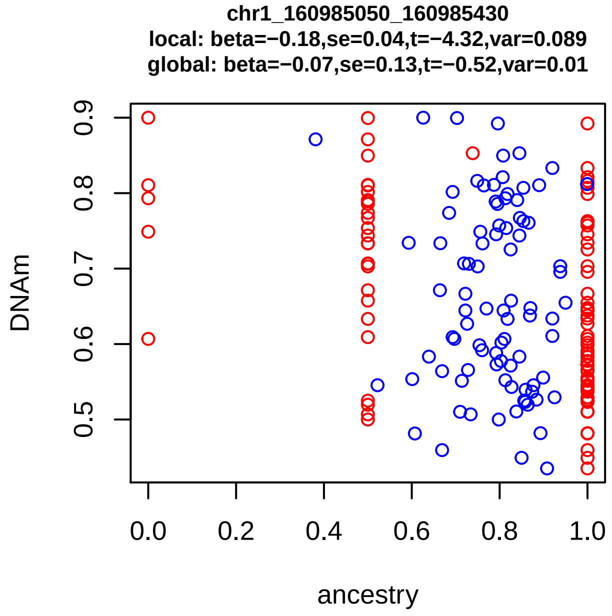
chr1_160985050_160985430
local: beta=−0.18,se=0.04,t=−4.32,var=0.089
global: beta=−0.07,se=0.13,t=−0.52,var=0.01
DNAm
0.5
0.6
0.7
0.8
0.9
0.0
0.2
0.4
0.6
0.8
1.0
ancestry

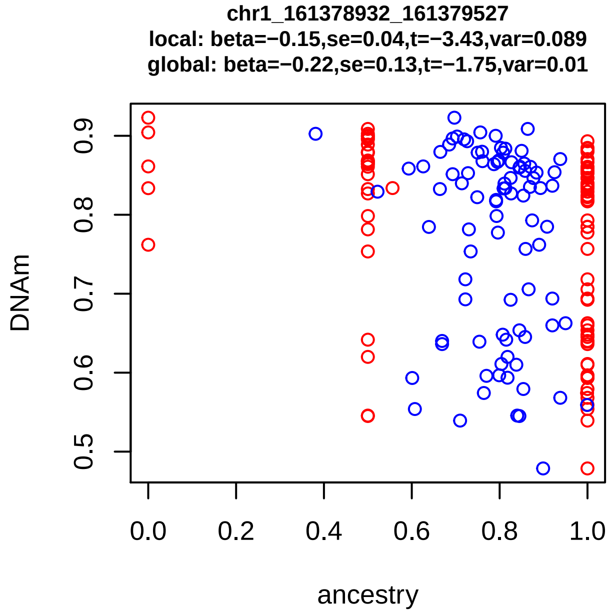
chr1_161378932_161379527
local: beta=−0.15,se=0.04,t=−3.43,var=0.089
global: beta=−0.22,se=0.13,t=−1.75,var=0.01
DNAm
0.5
0.6
0.7
0.8
0.9
0.0
0.2
0.4
0.6
0.8
1.0
ancestry

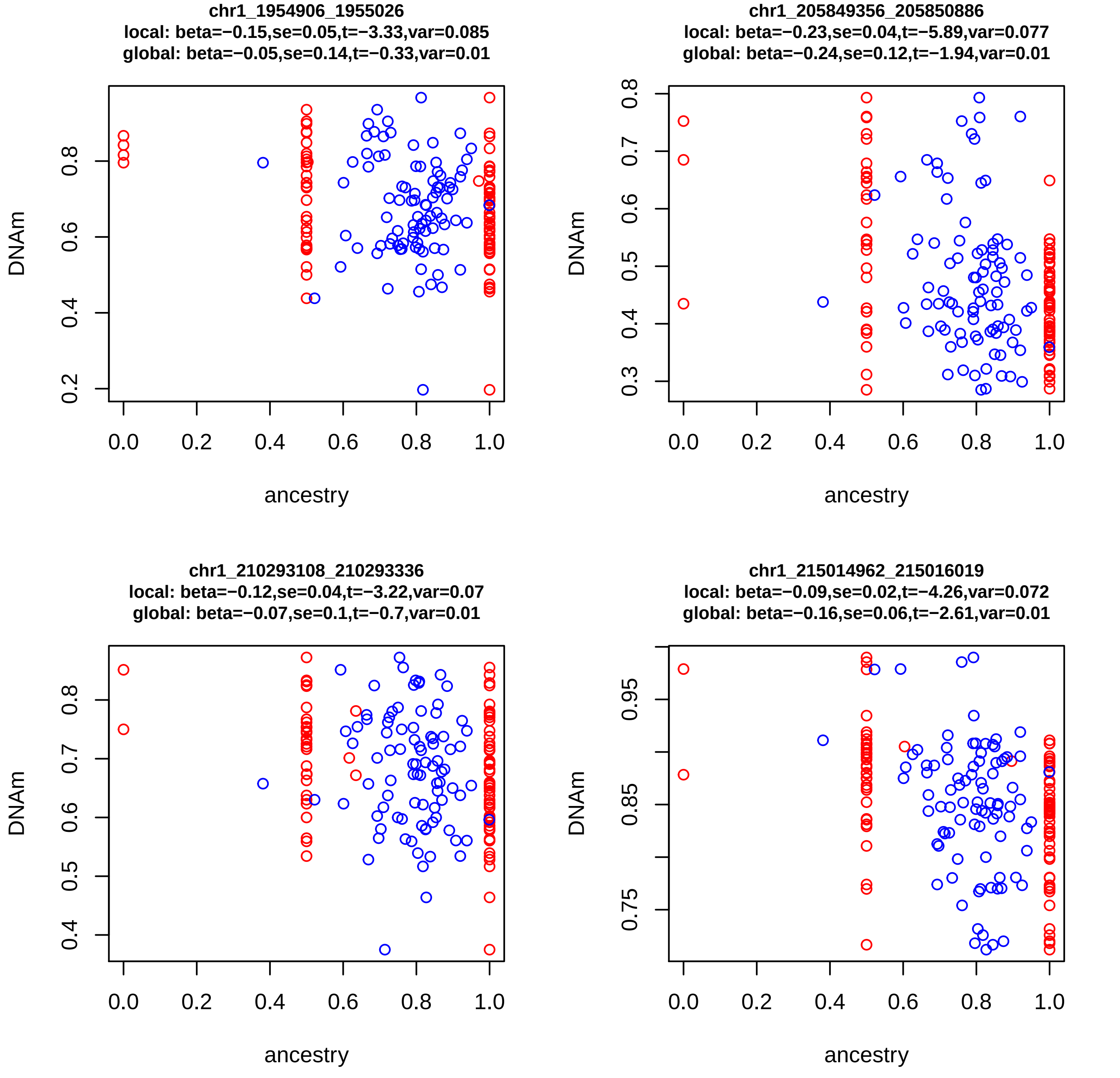
chr1_1954906_1955026
local: beta=−0.15,se=0.05,t=−3.33,var=0.085
global: beta=−0.05,se=0.14,t=−0.33,var=0.01
chr1_205849356_205850886
local: beta=−0.23,se=0.04,t=−5.89,var=0.077
global: beta=−0.24,se=0.12,t=−1.94,var=0.01
chr1_210293108_210293336
local: beta=−0.12,se=0.04,t=−3.22,var=0.07
global: beta=−0.07,se=0.1,t=−0.7,var=0.01
chr1_215014962_215016019
local: beta=−0.09,se=0.02,t=−4.26,var=0.072
global: beta=−0.16,se=0.06,t=−2.61,var=0.01
DNAm
ancestry
0.0
0.2
0.4
0.6
0.8
1.0
0.3
0.5
0.7
0.95
0.85
0.75

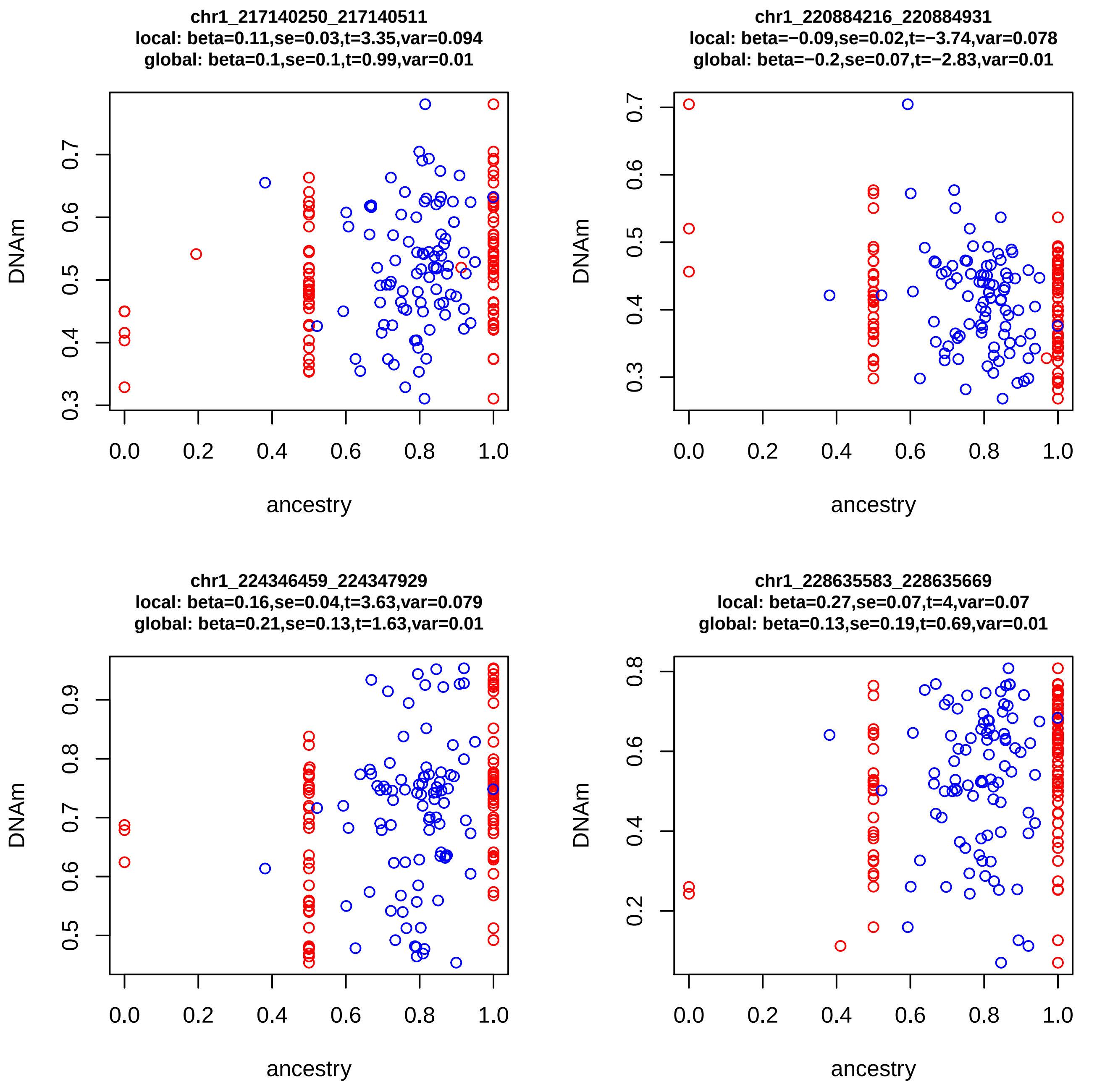
chr1_217140250_217140511
local: beta=0.11,se=0.03,t=3.35,var=0.094
global: beta=0.1,se=0.1,t=0.99,var=0.01
DNAm
ancestry
chr1_220884216_220884931
local: beta=−0.09,se=0.02,t=−3.74,var=0.078
global: beta=−0.2,se=0.07,t=−2.83,var=0.01
DNAm
ancestry
chr1_224346459_224347929
local: beta=0.16,se=0.04,t=3.63,var=0.079
global: beta=0.21,se=0.13,t=1.63,var=0.01
DNAm
ancestry
chr1_228635583_228635669
local: beta=0.27,se=0.07,t=4,var=0.07
global: beta=0.13,se=0.19,t=0.69,var=0.01
DNAm
ancestry

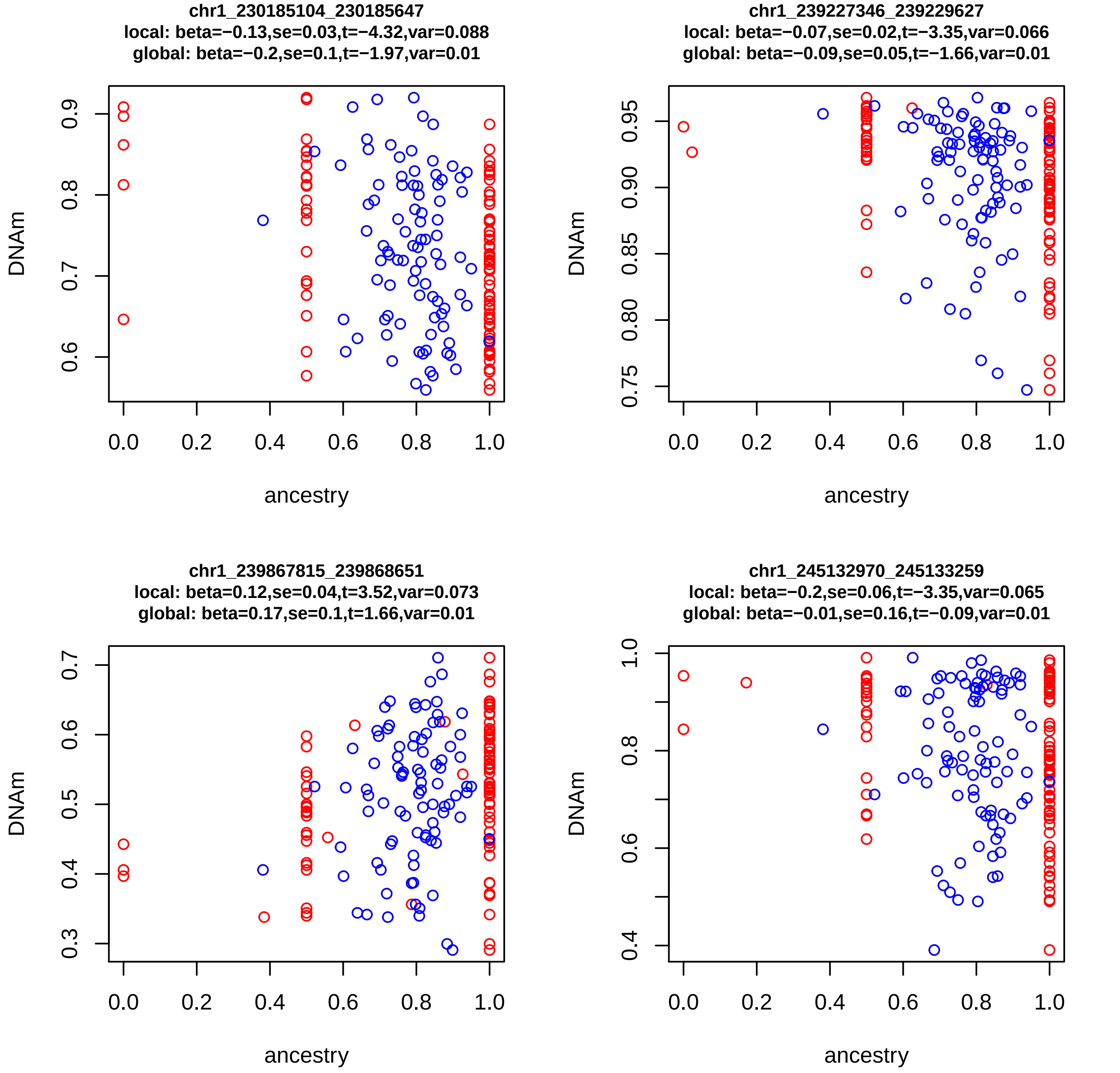
chr1_230185104_230185647
local: beta=−0.13,se=0.03,t=−4.32,var=0.088
global: beta=−0.2,se=0.1,t=−1.97,var=0.01
DNAm
ancestry
chr1_239227346_239229627
local: beta=−0.07,se=0.02,t=−3.35,var=0.066
global: beta=−0.09,se=0.05,t=−1.66,var=0.01
DNAm
ancestry
chr1_239867815_239868651
local: beta=0.12,se=0.04,t=3.52,var=0.073
global: beta=0.17,se=0.1,t=1.66,var=0.01
DNAm
ancestry
chr1_245132970_245133259
local: beta=−0.2,se=0.06,t=−3.35,var=0.065
global: beta=−0.01,se=0.16,t=−0.09,var=0.01
DNAm
ancestry

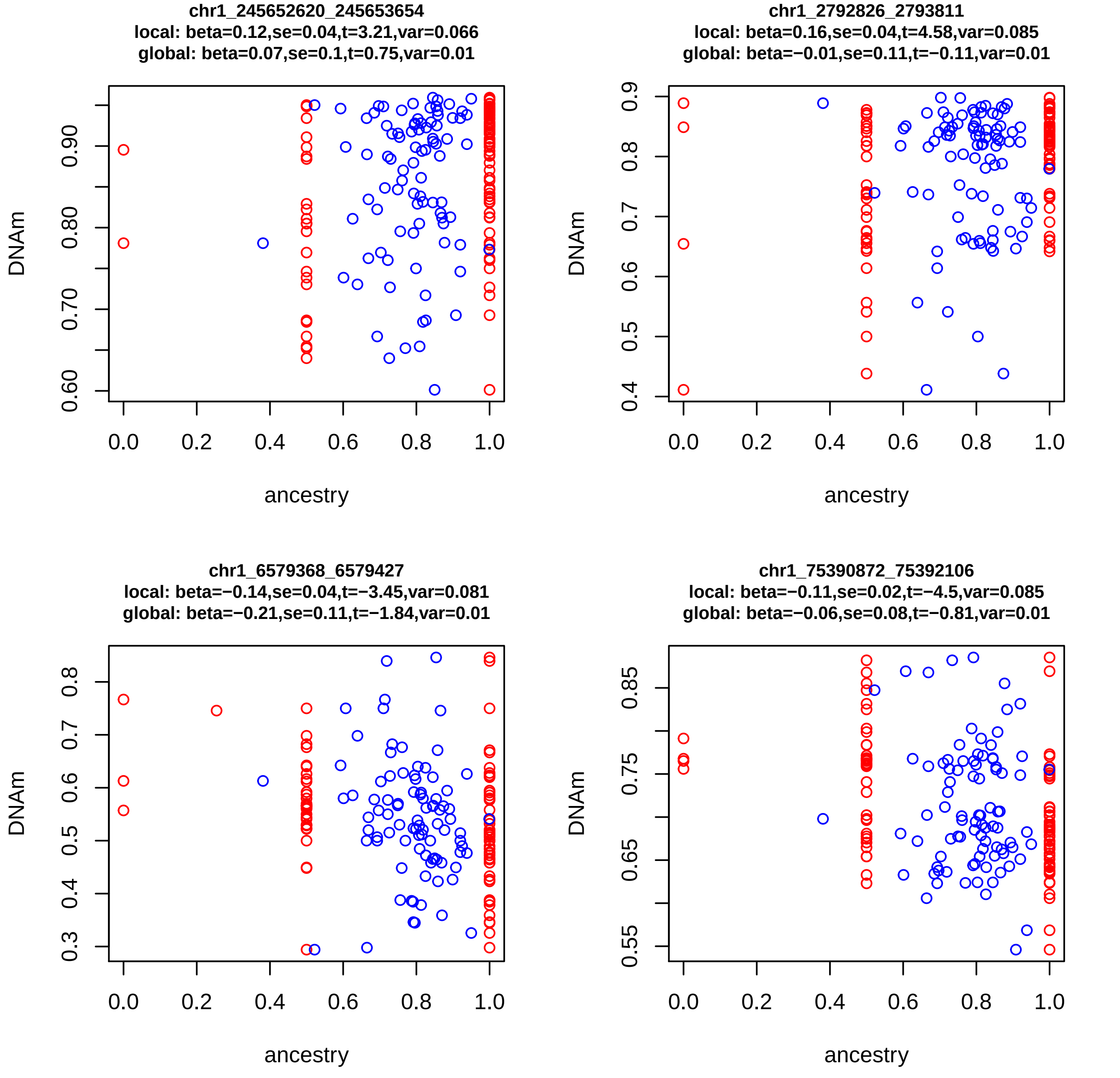
chr1_245652620_245653654
local: beta=0.12,se=0.04,t=3.21,var=0.066
global: beta=0.07,se=0.1,t=0.75,var=0.01
DNAm
0.60
0.70
0.80
0.90
0.0
0.2
0.4
0.6
0.8
1.0
ancestry
chr1_2792826_2793811
local: beta=0.16,se=0.04,t=4.58,var=0.085
global: beta=−0.01,se=0.11,t=−0.11,var=0.01
DNAm
0.4
0.5
0.6
0.7
0.8
0.9
0.0
0.2
0.4
0.6
0.8
1.0
ancestry
chr1_6579368_6579427
local: beta=−0.14,se=0.04,t=−3.45,var=0.081
global: beta=−0.21,se=0.11,t=−1.84,var=0.01
DNAm
0.3
0.4
0.5
0.6
0.7
0.8
0.0
0.2
0.4
0.6
0.8
1.0
ancestry
chr1_75390872_75392106
local: beta=−0.11,se=0.02,t=−4.5,var=0.085
global: beta=−0.06,se=0.08,t=−0.81,var=0.01
DNAm
0.55
0.65
0.75
0.85
0.0
0.2
0.4
0.6
0.8
1.0
ancestry

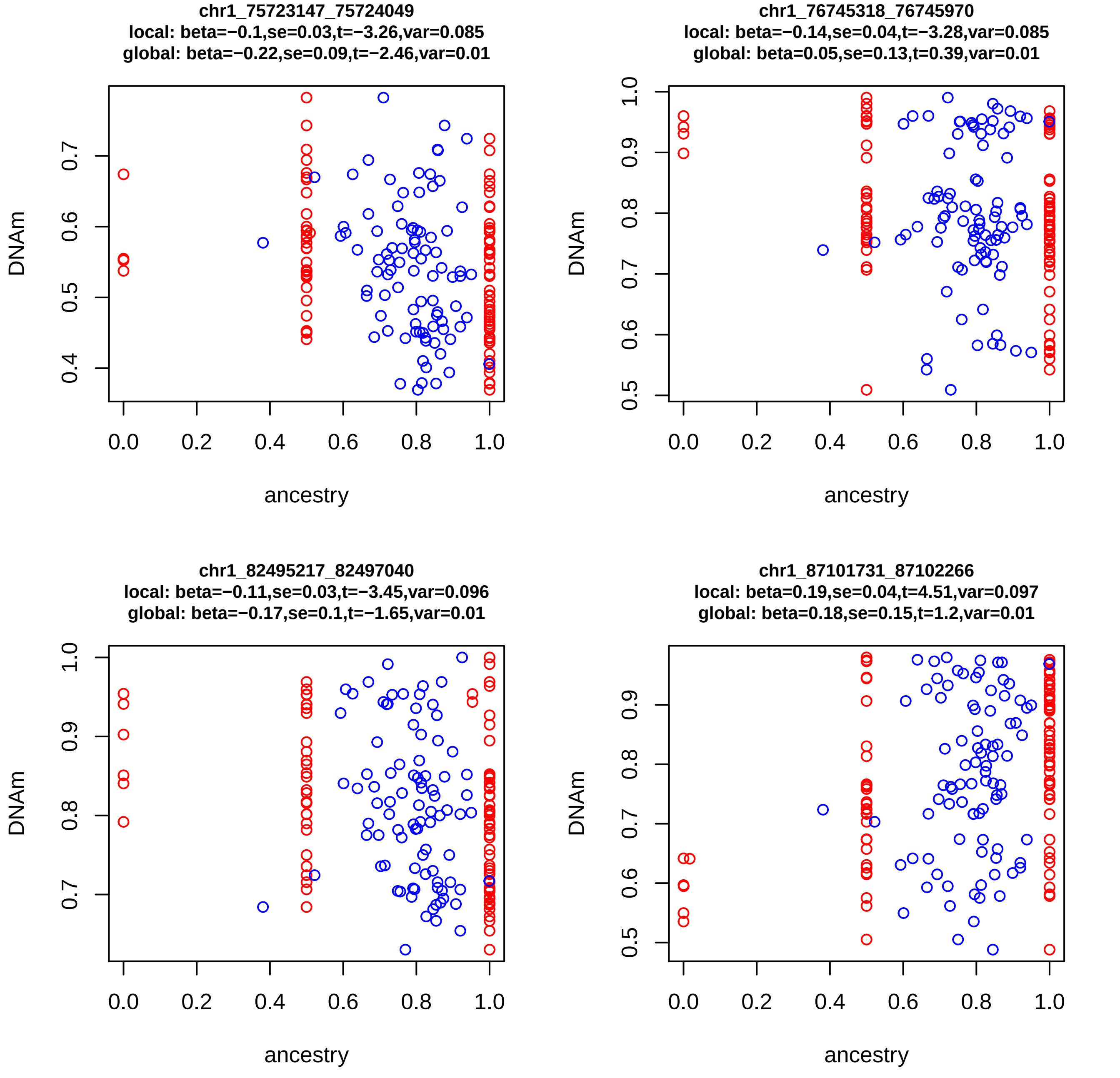
chr1_75723147_75724049
local: beta=−0.1,se=0.03,t=−3.26,var=0.085
global: beta=−0.22,se=0.09,t=−2.46,var=0.01
DNAm
ancestry
chr1_76745318_76745970
local: beta=−0.14,se=0.04,t=−3.28,var=0.085
global: beta=0.05,se=0.13,t=0.39,var=0.01
DNAm
ancestry
chr1_82495217_82497040
local: beta=−0.11,se=0.03,t=−3.45,var=0.096
global: beta=−0.17,se=0.1,t=−1.65,var=0.01
DNAm
ancestry
chr1_87101731_87102266
local: beta=0.19,se=0.04,t=4.51,var=0.097
global: beta=0.18,se=0.15,t=1.2,var=0.01
DNAm
ancestry

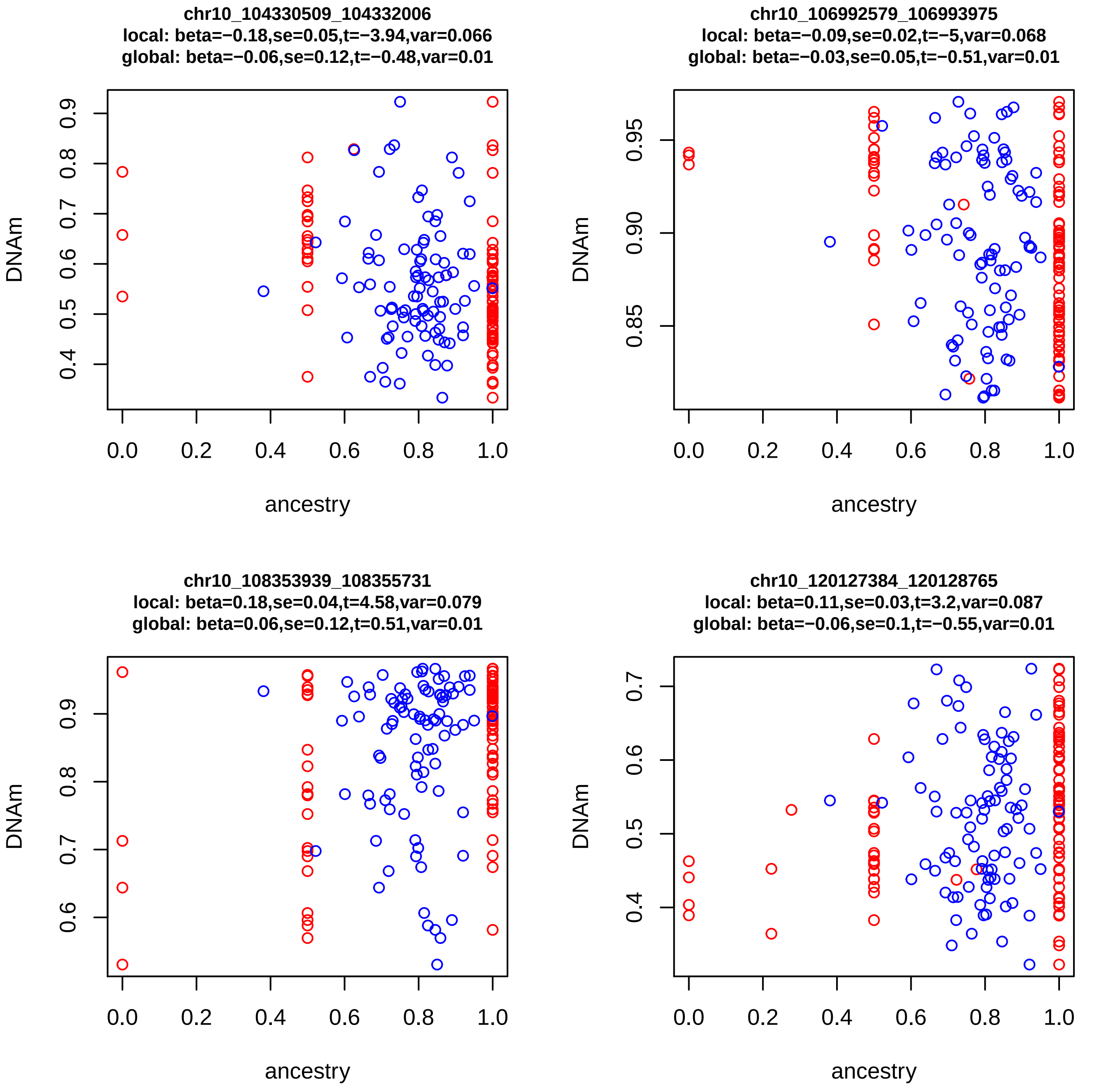
chr10_104330509_104332006
local: beta=−0.18,se=0.05,t=−3.94,var=0.066
global: beta=−0.06,se=0.12,t=−0.48,var=0.01
DNAm
ancestry
chr10_106992579_106993975
local: beta=−0.09,se=0.02,t=−5,var=0.068
global: beta=−0.03,se=0.05,t=−0.51,var=0.01
DNAm
ancestry
chr10_108353939_108355731
local: beta=0.18,se=0.04,t=4.58,var=0.079
global: beta=0.06,se=0.12,t=0.51,var=0.01
DNAm
ancestry
chr10_120127384_120128765
local: beta=0.11,se=0.03,t=3.2,var=0.087
global: beta=−0.06,se=0.1,t=−0.55,var=0.01
DNAm
ancestry

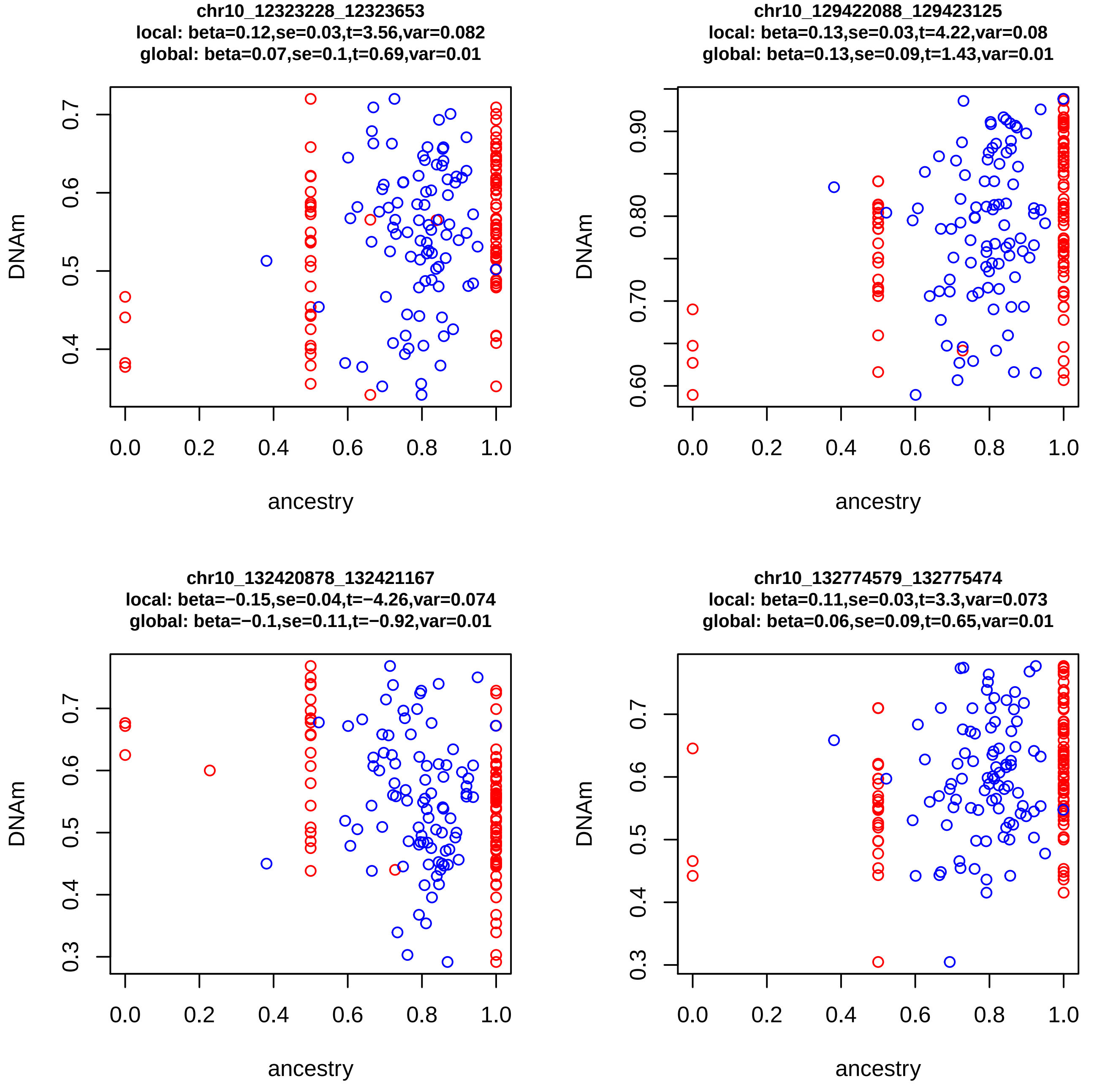

chr10_12323228_12323653
local: beta=0.12,se=0.03,t=3.56,var=0.082
global: beta=0.07,se=0.1,t=0.69,var=0.01
DNAm
ancestry
0.0
0.2
0.4
0.6
0.8
1.0
0.4
0.5
0.6
0.7
chr10_129422088_129423125
local: beta=0.13,se=0.03,t=4.22,var=0.08
global: beta=0.13,se=0.09,t=1.43,var=0.01
DNAm
ancestry
0.60
0.70
0.80
0.90
chr10_132420878_132421167
local: beta=−0.15,se=0.04,t=−4.26,var=0.074
global: beta=−0.1,se=0.11,t=−0.92,var=0.01
DNAm
ancestry
0.3
0.4
0.5
0.6
0.7
chr10_132774579_132775474
local: beta=0.11,se=0.03,t=3.3,var=0.073
global: beta=0.06,se=0.09,t=0.65,var=0.01
DNAm
ancestry
0.3
0.4
0.5
0.6
0.7

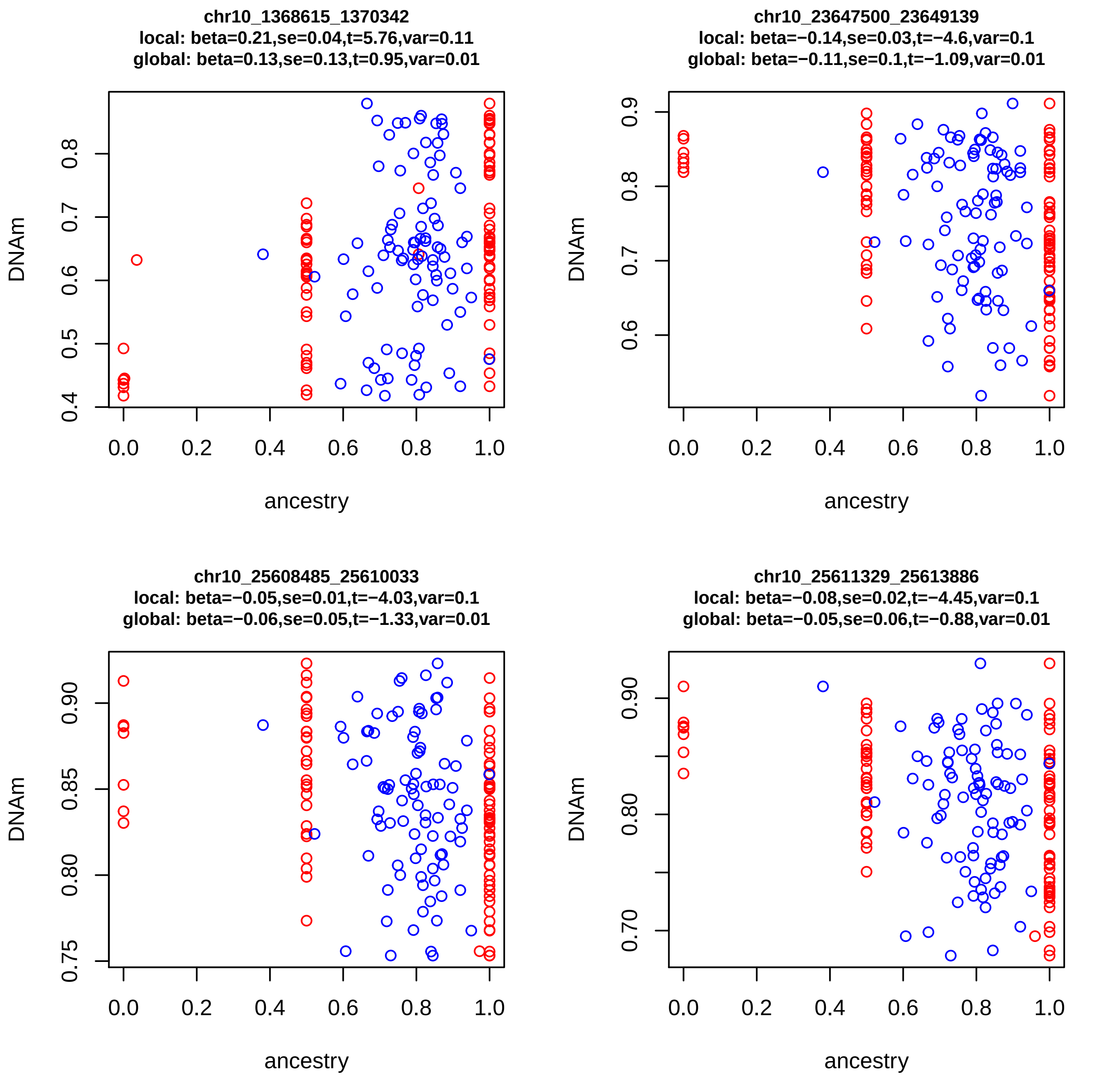
chr10_1368615_1370342
local: beta=0.21,se=0.04,t=5.76,var=0.11
global: beta=0.13,se=0.13,t=0.95,var=0.01
DNAm
0.4
0.5
0.6
0.7
0.8
ancestry
0.0
0.2
0.4
0.6
0.8
1.0
chr10_23647500_23649139
local: beta=−0.14,se=0.03,t=−4.6,var=0.1
global: beta=−0.11,se=0.1,t=−1.09,var=0.01
DNAm
0.6
0.7
0.8
0.9
ancestry
0.0
0.2
0.4
0.6
0.8
1.0
chr10_25608485_25610033
local: beta=−0.05,se=0.01,t=−4.03,var=0.1
global: beta=−0.06,se=0.05,t=−1.33,var=0.01
DNAm
0.75
0.80
0.85
0.90
ancestry
0.0
0.2
0.4
0.6
0.8
1.0
chr10_25611329_25613886
local: beta=−0.08,se=0.02,t=−4.45,var=0.1
global: beta=−0.05,se=0.06,t=−0.88,var=0.01
DNAm
0.70
0.80
0.90
ancestry
0.0
0.2
0.4
0.6
0.8
1.0

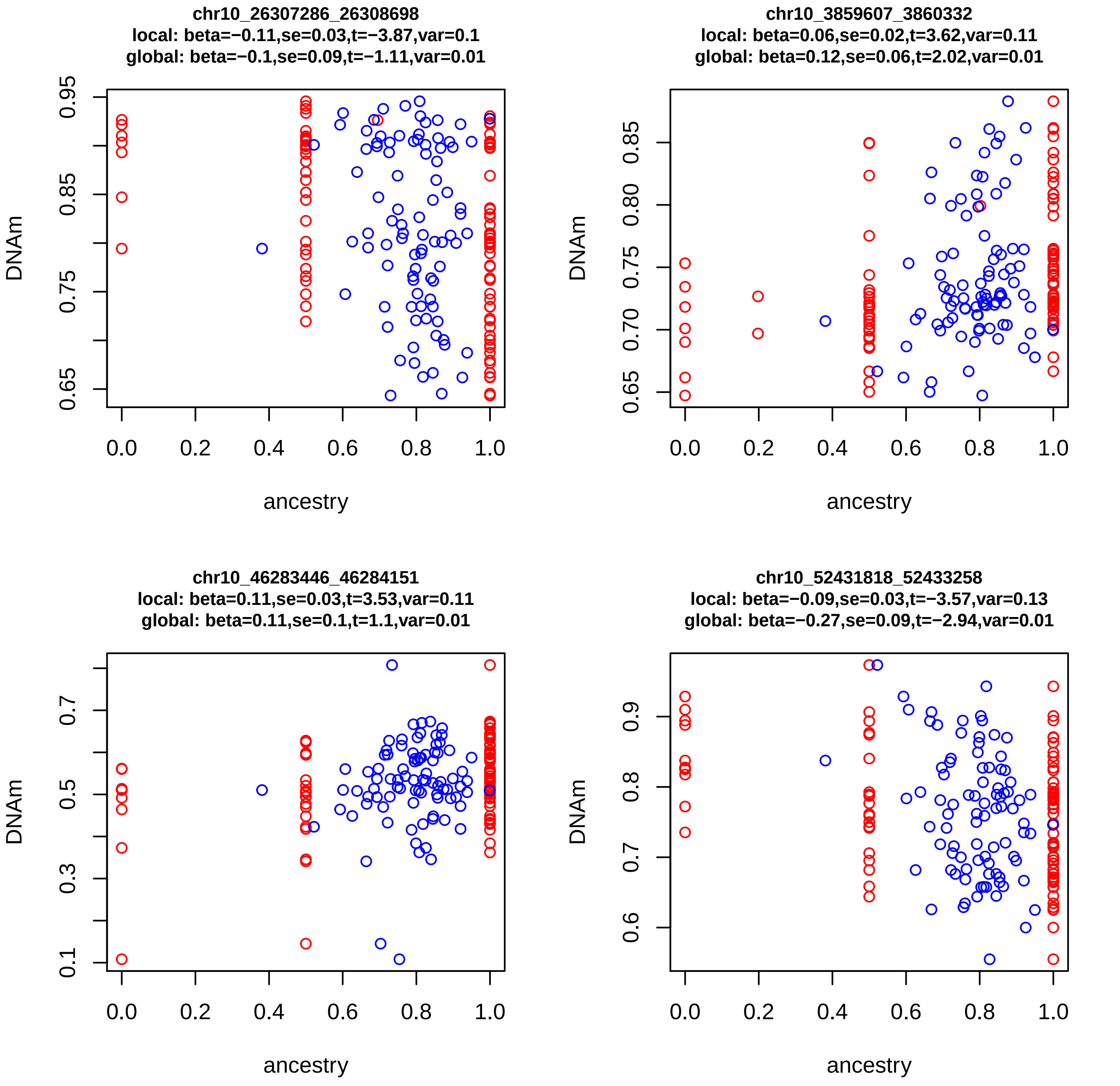
chr10_26307286_26308698
local: beta=−0.11,se=0.03,t=−3.87,var=0.1
global: beta=−0.1,se=0.09,t=−1.11,var=0.01
DNAm
0.65
0.75
0.85
0.95
0.0
0.2
0.4
0.6
0.8
1.0
ancestry
chr10_3859607_3860332
local: beta=0.06,se=0.02,t=3.62,var=0.11
global: beta=0.12,se=0.06,t=2.02,var=0.01
DNAm
0.65
0.70
0.75
0.80
0.85
0.0
0.2
0.4
0.6
0.8
1.0
ancestry
chr10_46283446_46284151
local: beta=0.11,se=0.03,t=3.53,var=0.11
global: beta=0.11,se=0.1,t=1.1,var=0.01
DNAm
0.1
0.3
0.5
0.7
0.0
0.2
0.4
0.6
0.8
1.0
ancestry
chr10_52431818_52433258
local: beta=−0.09,se=0.03,t=−3.57,var=0.13
global: beta=−0.27,se=0.09,t=−2.94,var=0.01
DNAm
0.6
0.7
0.8
0.9
0.0
0.2
0.4
0.6
0.8
1.0
ancestry

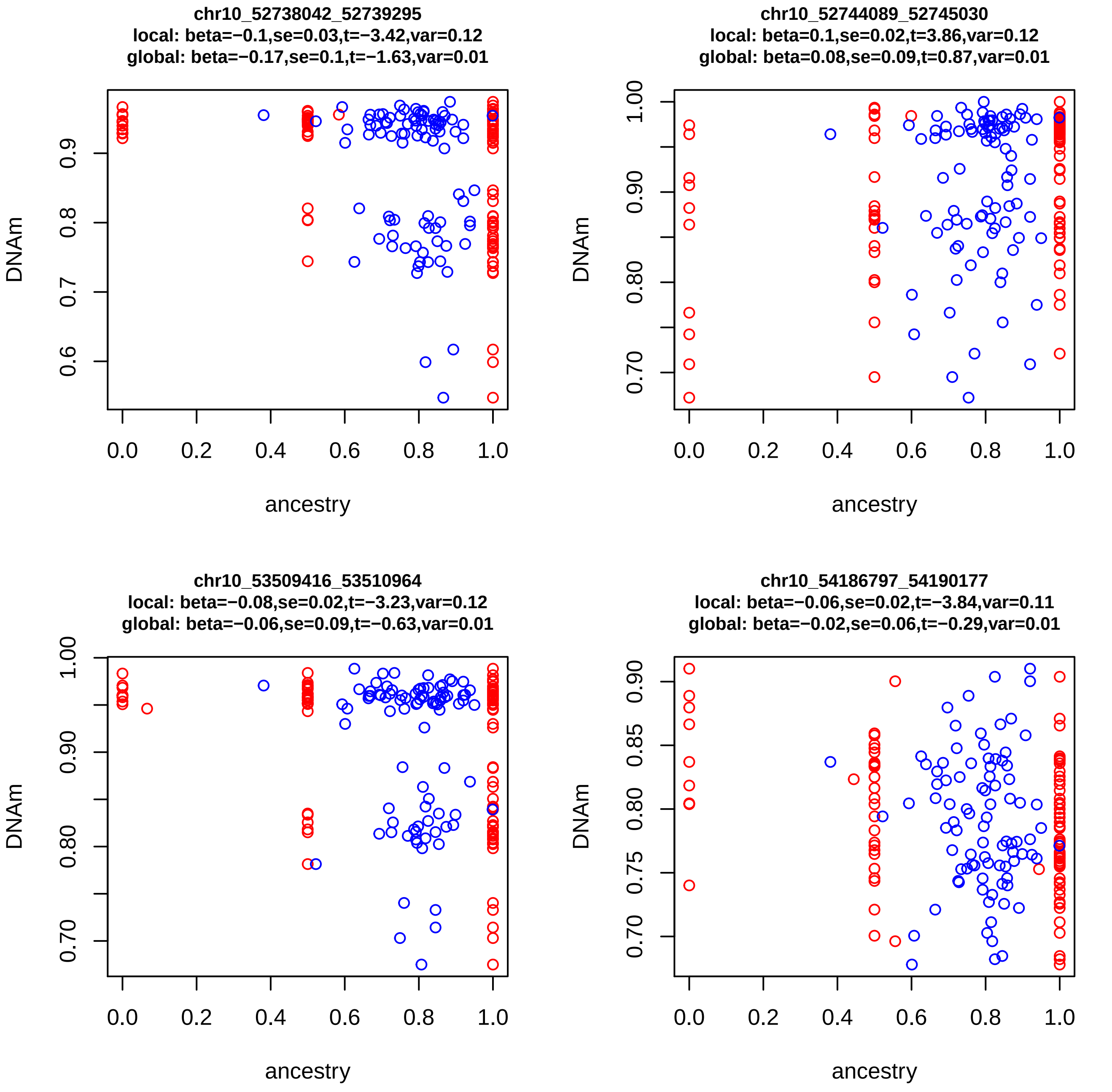
chr10_52738042_52739295
local: beta=−0.1,se=0.03,t=−3.42,var=0.12
global: beta=−0.17,se=0.1,t=−1.63,var=0.01
DNAm
ancestry
chr10_52744089_52745030
local: beta=0.1,se=0.02,t=3.86,var=0.12
global: beta=0.08,se=0.09,t=0.87,var=0.01
DNAm
ancestry
chr10_53509416_53510964
local: beta=−0.08,se=0.02,t=−3.23,var=0.12
global: beta=−0.06,se=0.09,t=−0.63,var=0.01
DNAm
ancestry
chr10_54186797_54190177
local: beta=−0.06,se=0.02,t=−3.84,var=0.11
global: beta=−0.02,se=0.06,t=−0.29,var=0.01
DNAm
ancestry

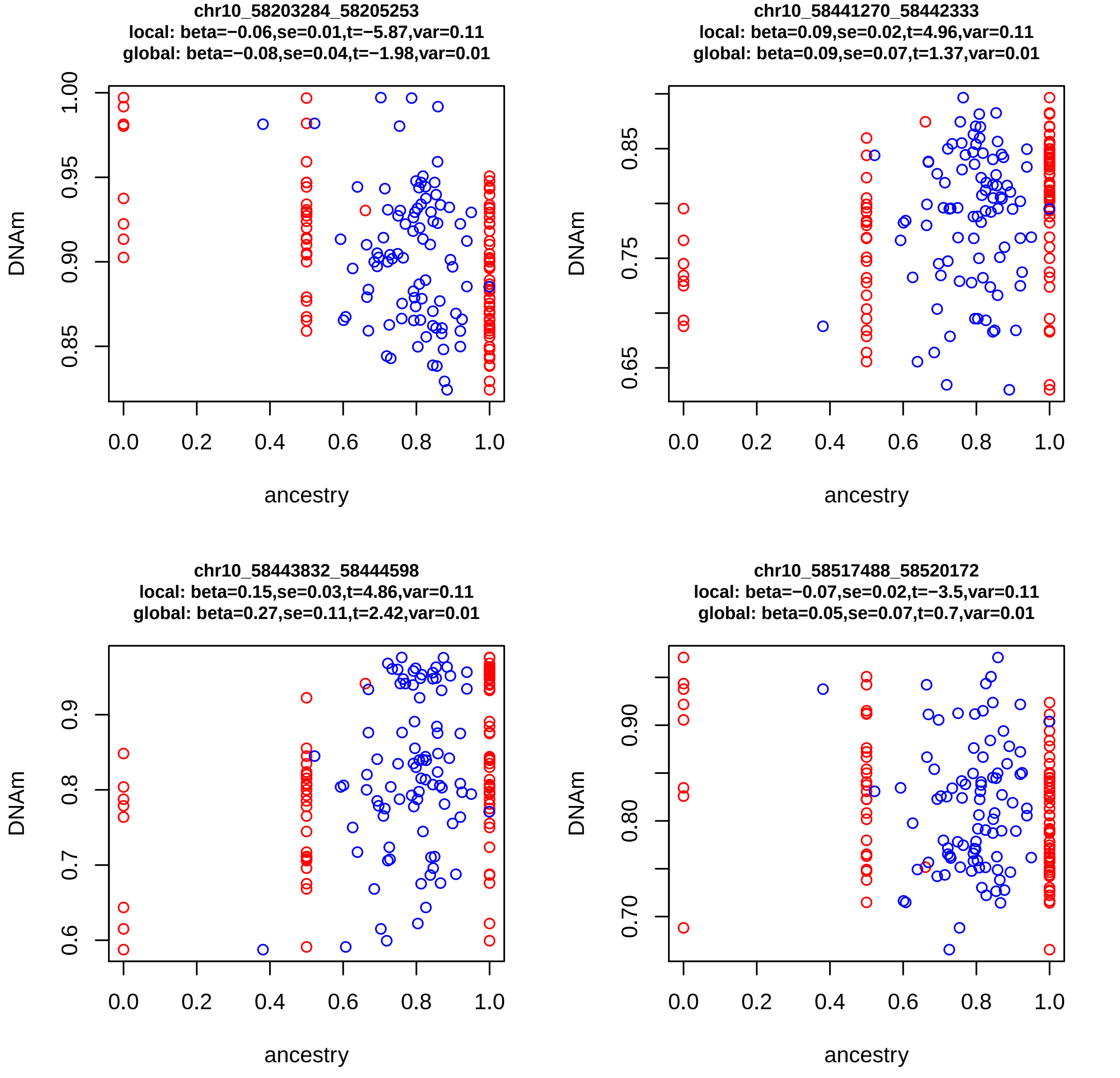
chr10_58203284_58205253
local: beta=−0.06,se=0.01,t=−5.87,var=0.11
global: beta=−0.08,se=0.04,t=−1.98,var=0.01
DNAm
1.00
0.95
0.90
0.85
ancestry
0.0
0.2
0.4
0.6
0.8
1.0
chr10_58441270_58442333
local: beta=0.09,se=0.02,t=4.96,var=0.11
global: beta=0.09,se=0.07,t=1.37,var=0.01
DNAm
0.85
0.75
0.65
ancestry
chr10_58443832_58444598
local: beta=0.15,se=0.03,t=4.86,var=0.11
global: beta=0.27,se=0.11,t=2.42,var=0.01
DNAm
0.9
0.8
0.7
0.6
ancestry
chr10_58517488_58520172
local: beta=−0.07,se=0.02,t=−3.5,var=0.11
global: beta=0.05,se=0.07,t=0.7,var=0.01
DNAm
0.90
0.80
0.70
ancestry

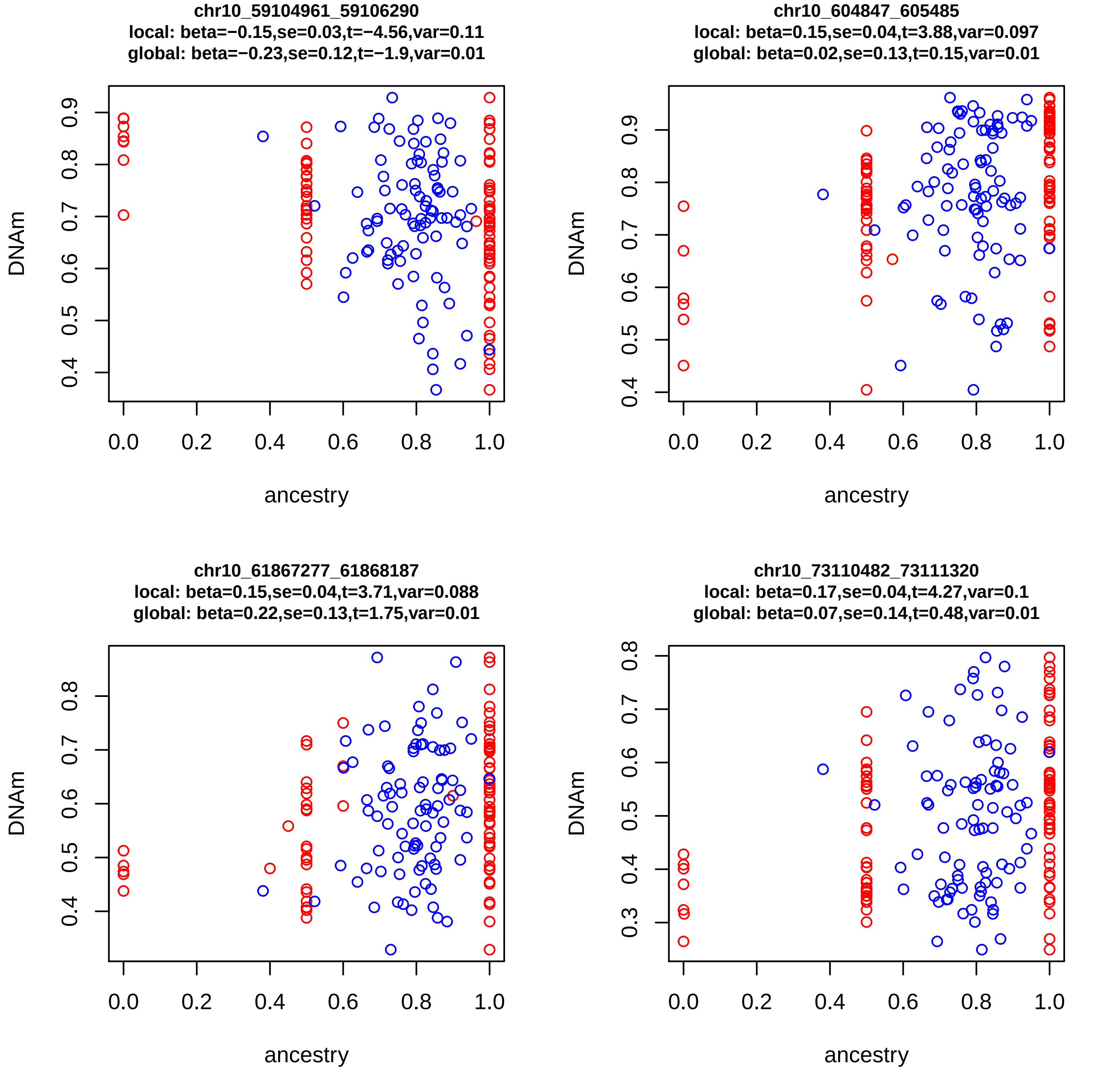

chr10_59104961_59106290
local: beta=−0.15,se=0.03,t=−4.56,var=0.11
global: beta=−0.23,se=0.12,t=−1.9,var=0.01
DNAm
ancestry
chr10_604847_605485
local: beta=0.15,se=0.04,t=3.88,var=0.097
global: beta=0.02,se=0.13,t=0.15,var=0.01
DNAm
ancestry
chr10_61867277_61868187
local: beta=0.15,se=0.04,t=3.71,var=0.088
global: beta=0.22,se=0.13,t=1.75,var=0.01
DNAm
ancestry
chr10_73110482_73111320
local: beta=0.17,se=0.04,t=4.27,var=0.1
global: beta=0.07,se=0.14,t=0.48,var=0.01
DNAm
ancestry

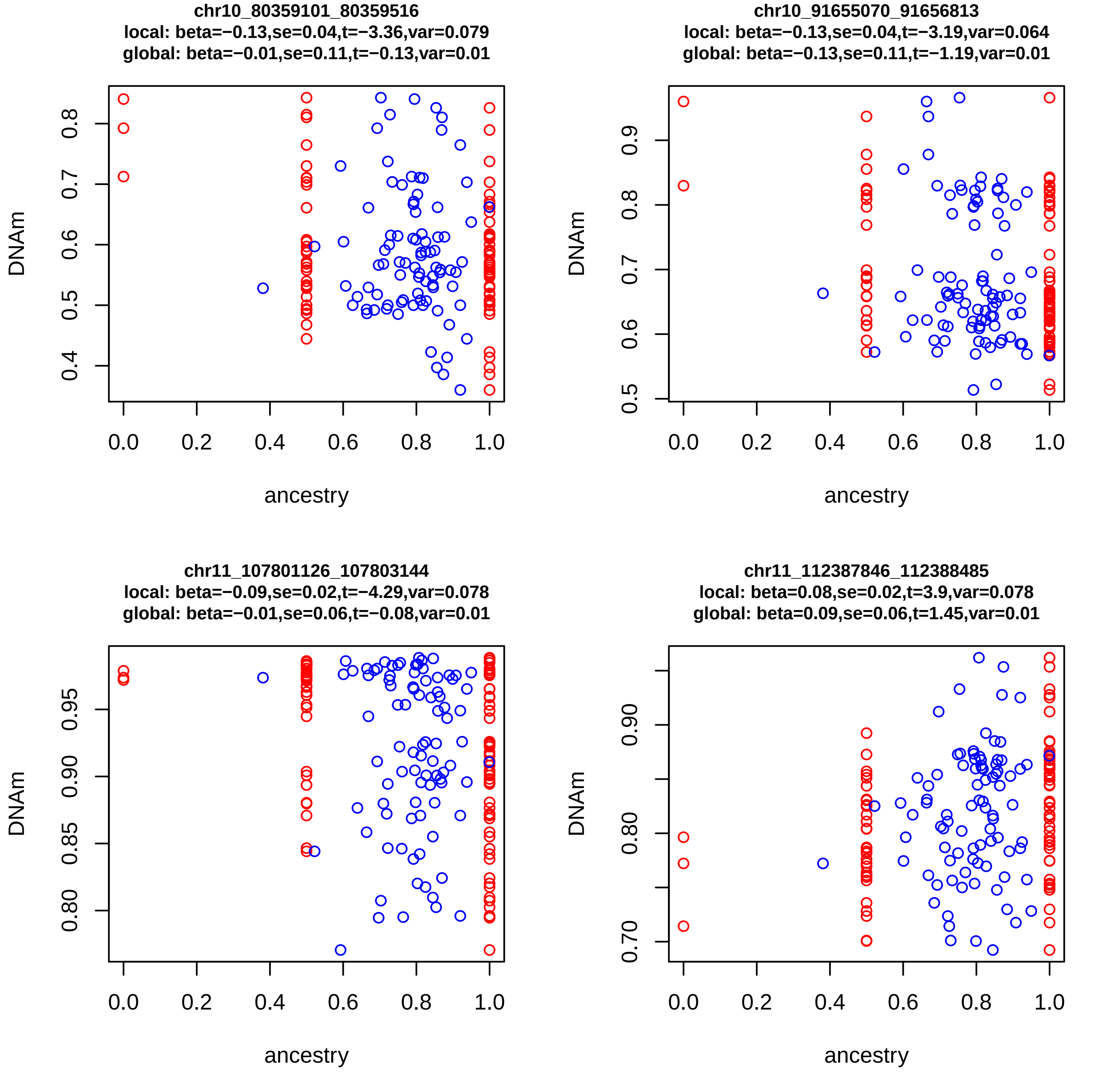
chr10_80359101_80359516
local: beta=−0.13,se=0.04,t=−3.36,var=0.079
global: beta=−0.01,se=0.11,t=−0.13,var=0.01
DNAm
0.4
0.5
0.6
0.7
0.8
0.0
0.2
0.4
0.6
0.8
1.0
ancestry
chr10_91655070_91656813
local: beta=−0.13,se=0.04,t=−3.19,var=0.064
global: beta=−0.13,se=0.11,t=−1.19,var=0.01
DNAm
0.5
0.6
0.7
0.8
0.9
0.0
0.2
0.4
0.6
0.8
1.0
ancestry
chr11_107801126_107803144
local: beta=−0.09,se=0.02,t=−4.29,var=0.078
global: beta=−0.01,se=0.06,t=−0.08,var=0.01
DNAm
0.80
0.85
0.90
0.95
0.0
0.2
0.4
0.6
0.8
1.0
ancestry
chr11_112387846_112388485
local: beta=0.08,se=0.02,t=3.9,var=0.078
global: beta=0.09,se=0.06,t=1.45,var=0.01
DNAm
0.70
0.80
0.90
0.0
0.2
0.4
0.6
0.8
1.0
ancestry

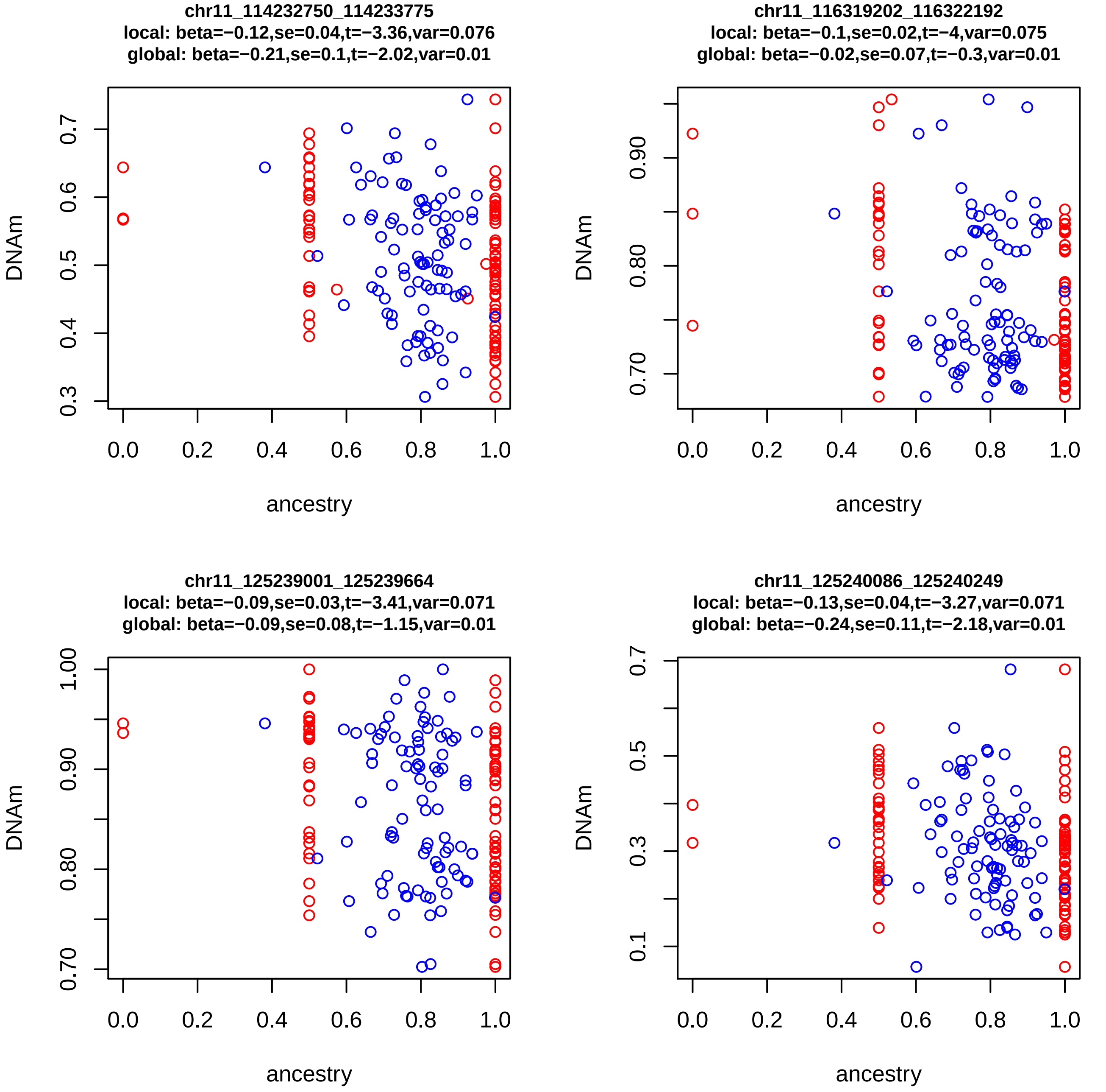
chr11_114232750_114233775
local: beta=−0.12,se=0.04,t=−3.36,var=0.076
global: beta=−0.21,se=0.1,t=−2.02,var=0.01
chr11_116319202_116322192
local: beta=−0.1,se=0.02,t=−4,var=0.075
global: beta=−0.02,se=0.07,t=−0.3,var=0.01
chr11_125239001_125239664
local: beta=−0.09,se=0.03,t=−3.41,var=0.071
global: beta=−0.09,se=0.08,t=−1.15,var=0.01
chr11_125240086_125240249
local: beta=−0.13,se=0.04,t=−3.27,var=0.071
global: beta=−0.24,se=0.11,t=−2.18,var=0.01
DNAm
ancestry
0.0
0.2
0.4
0.6
0.8
1.0

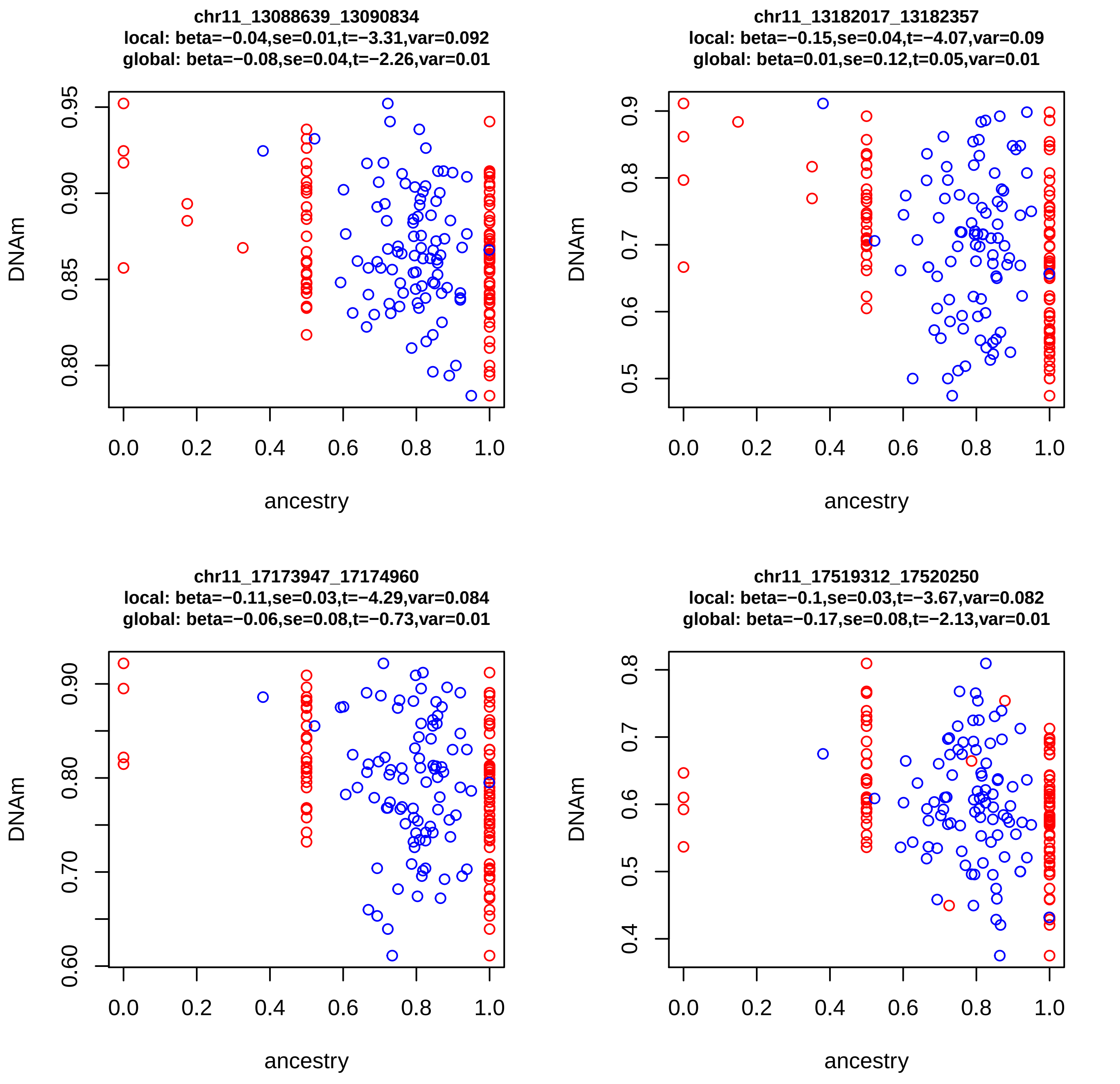
chr11_13088639_13090834
local: beta=−0.04,se=0.01,t=−3.31,var=0.092
global: beta=−0.08,se=0.04,t=−2.26,var=0.01
DNAm
ancestry
chr11_13182017_13182357
local: beta=−0.15,se=0.04,t=−4.07,var=0.09
global: beta=0.01,se=0.12,t=0.05,var=0.01
DNAm
ancestry
chr11_17173947_17174960
local: beta=−0.11,se=0.03,t=−4.29,var=0.084
global: beta=−0.06,se=0.08,t=−0.73,var=0.01
DNAm
ancestry
chr11_17519312_17520250
local: beta=−0.1,se=0.03,t=−3.67,var=0.082
global: beta=−0.17,se=0.08,t=−2.13,var=0.01
DNAm
ancestry

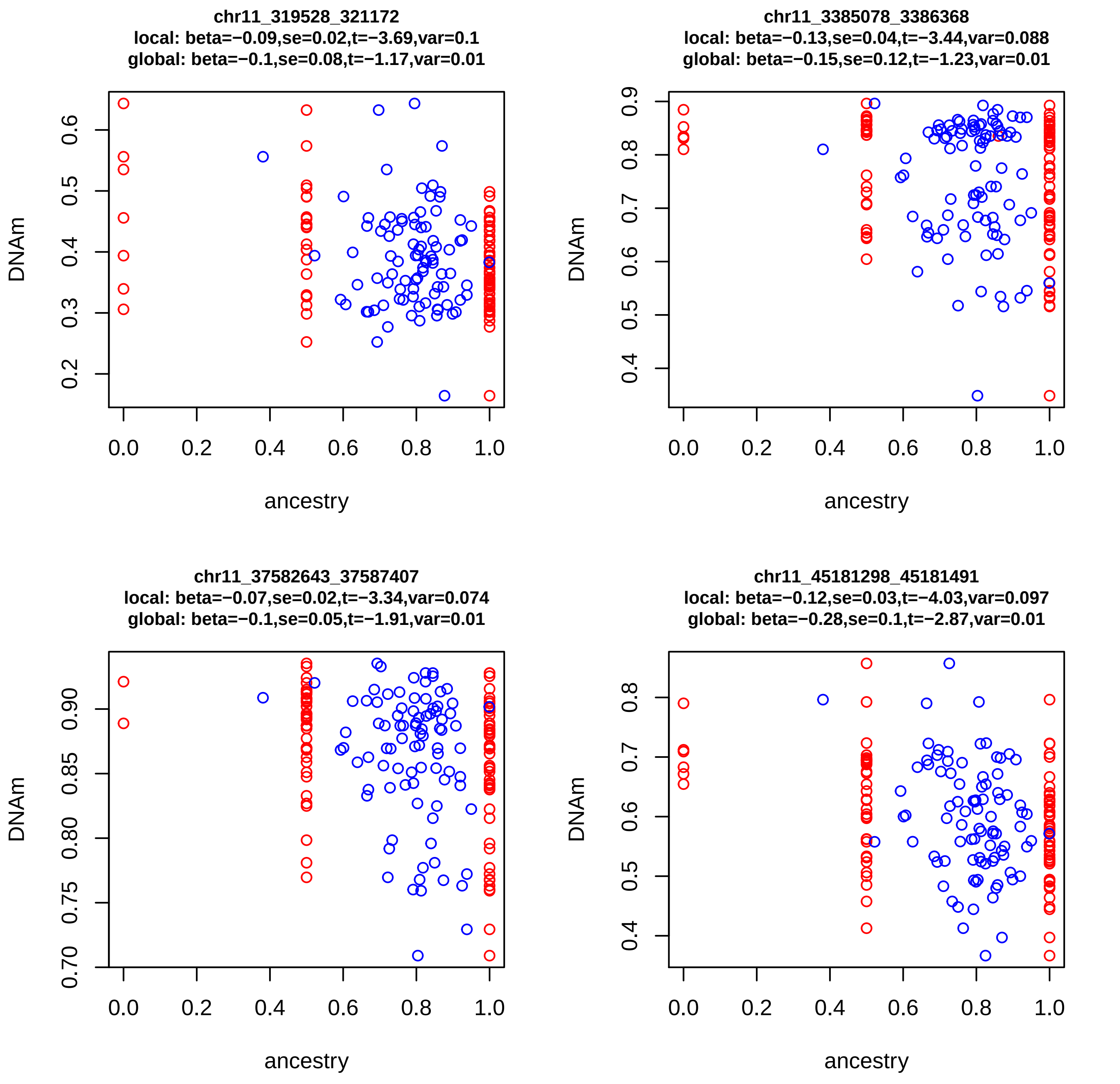

chr11_319528_321172
local: beta=−0.09,se=0.02,t=−3.69,var=0.1
global: beta=−0.1,se=0.08,t=−1.17,var=0.01
DNAm
ancestry
chr11_3385078_3386368
local: beta=−0.13,se=0.04,t=−3.44,var=0.088
global: beta=−0.15,se=0.12,t=−1.23,var=0.01
DNAm
ancestry
chr11_37582643_37587407
local: beta=−0.07,se=0.02,t=−3.34,var=0.074
global: beta=−0.1,se=0.05,t=−1.91,var=0.01
DNAm
ancestry
chr11_45181298_45181491
local: beta=−0.12,se=0.03,t=−4.03,var=0.097
global: beta=−0.28,se=0.1,t=−2.87,var=0.01
DNAm
ancestry

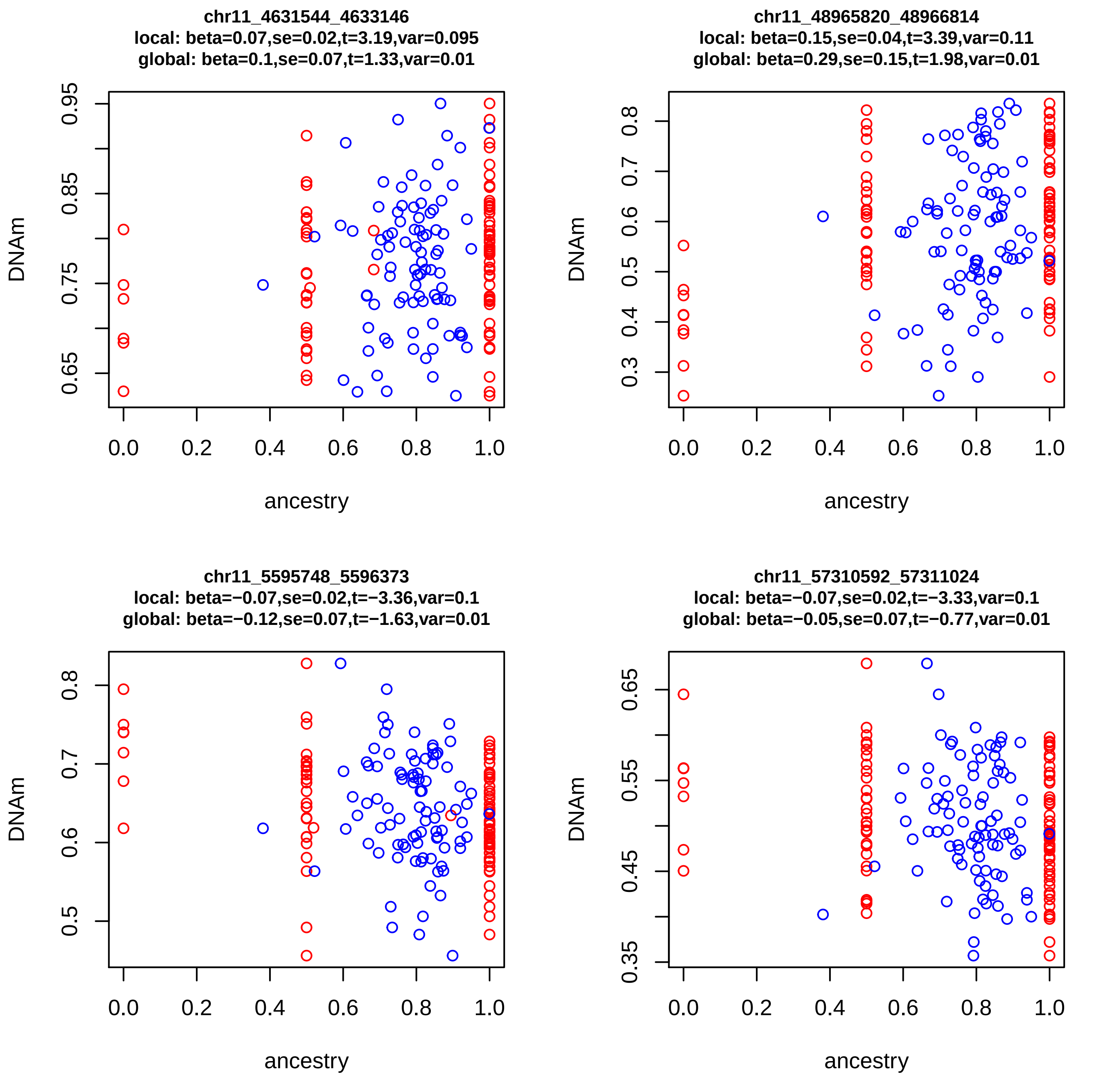

chr11_4631544_4633146
local: beta=0.07,se=0.02,t=3.19,var=0.095
global: beta=0.1,se=0.07,t=1.33,var=0.01
chr11_48965820_48966814
local: beta=0.15,se=0.04,t=3.39,var=0.11
global: beta=0.29,se=0.15,t=1.98,var=0.01
chr11_5595748_5596373
local: beta=−0.07,se=0.02,t=−3.36,var=0.1
global: beta=−0.12,se=0.07,t=−1.63,var=0.01
chr11_57310592_57311024
local: beta=−0.07,se=0.02,t=−3.33,var=0.1
global: beta=−0.05,se=0.07,t=−0.77,var=0.01
DNAm
ancestry
0.0
0.2
0.4
0.6
0.8
1.0
0.65
0.75
0.85
0.95
0.3
0.4
0.5
0.6
0.7
0.8
0.35
0.45
0.55

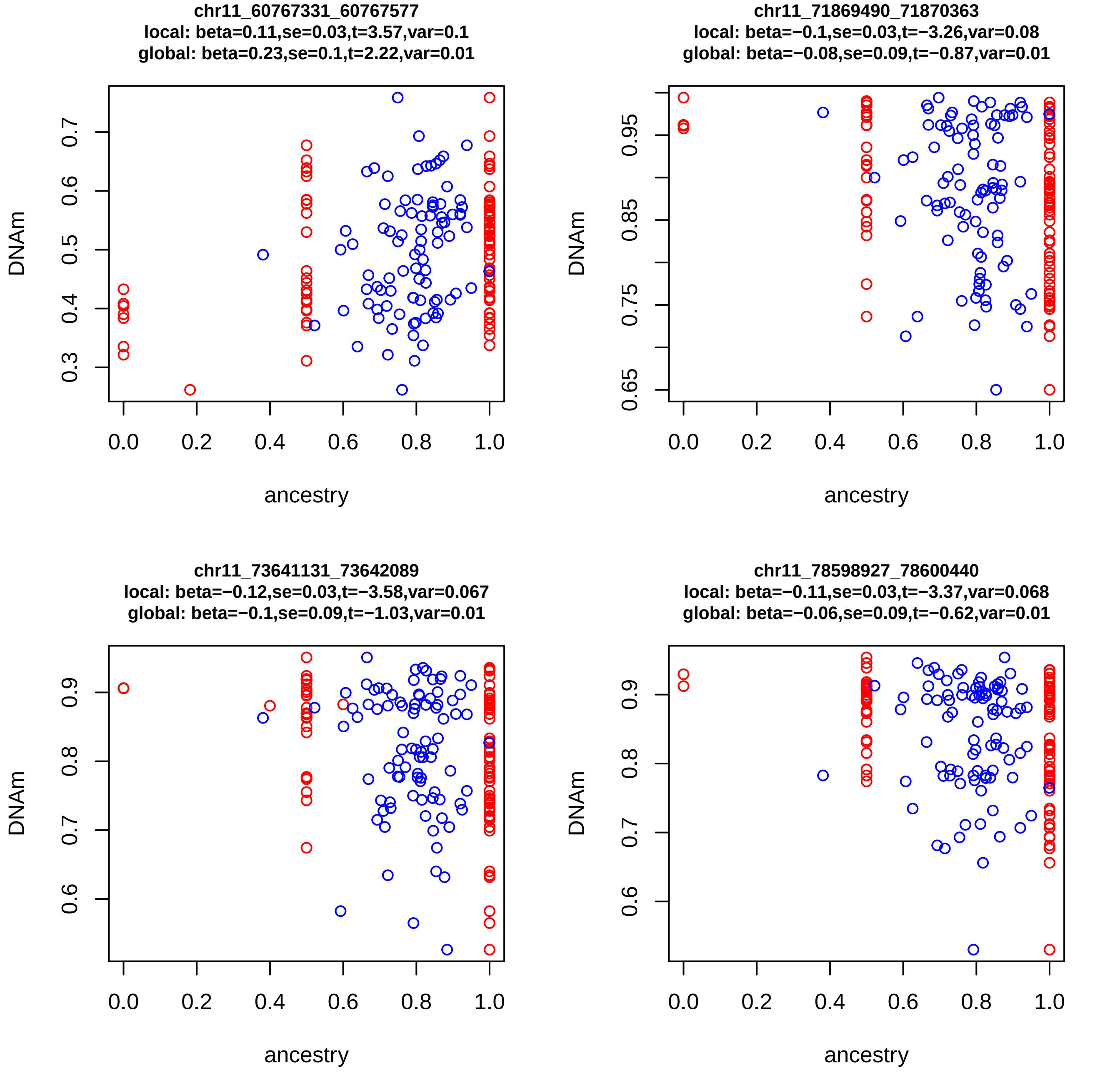
chr11_60767331_60767577
local: beta=0.11,se=0.03,t=3.57,var=0.1
global: beta=0.23,se=0.1,t=2.22,var=0.01
DNAm
ancestry
chr11_71869490_71870363
local: beta=−0.1,se=0.03,t=−3.26,var=0.08
global: beta=−0.08,se=0.09,t=−0.87,var=0.01
DNAm
ancestry
chr11_73641131_73642089
local: beta=−0.12,se=0.03,t=−3.58,var=0.067
global: beta=−0.1,se=0.09,t=−1.03,var=0.01
DNAm
ancestry
chr11_78598927_78600440
local: beta=−0.11,se=0.03,t=−3.37,var=0.068
global: beta=−0.06,se=0.09,t=−0.62,var=0.01
DNAm
ancestry

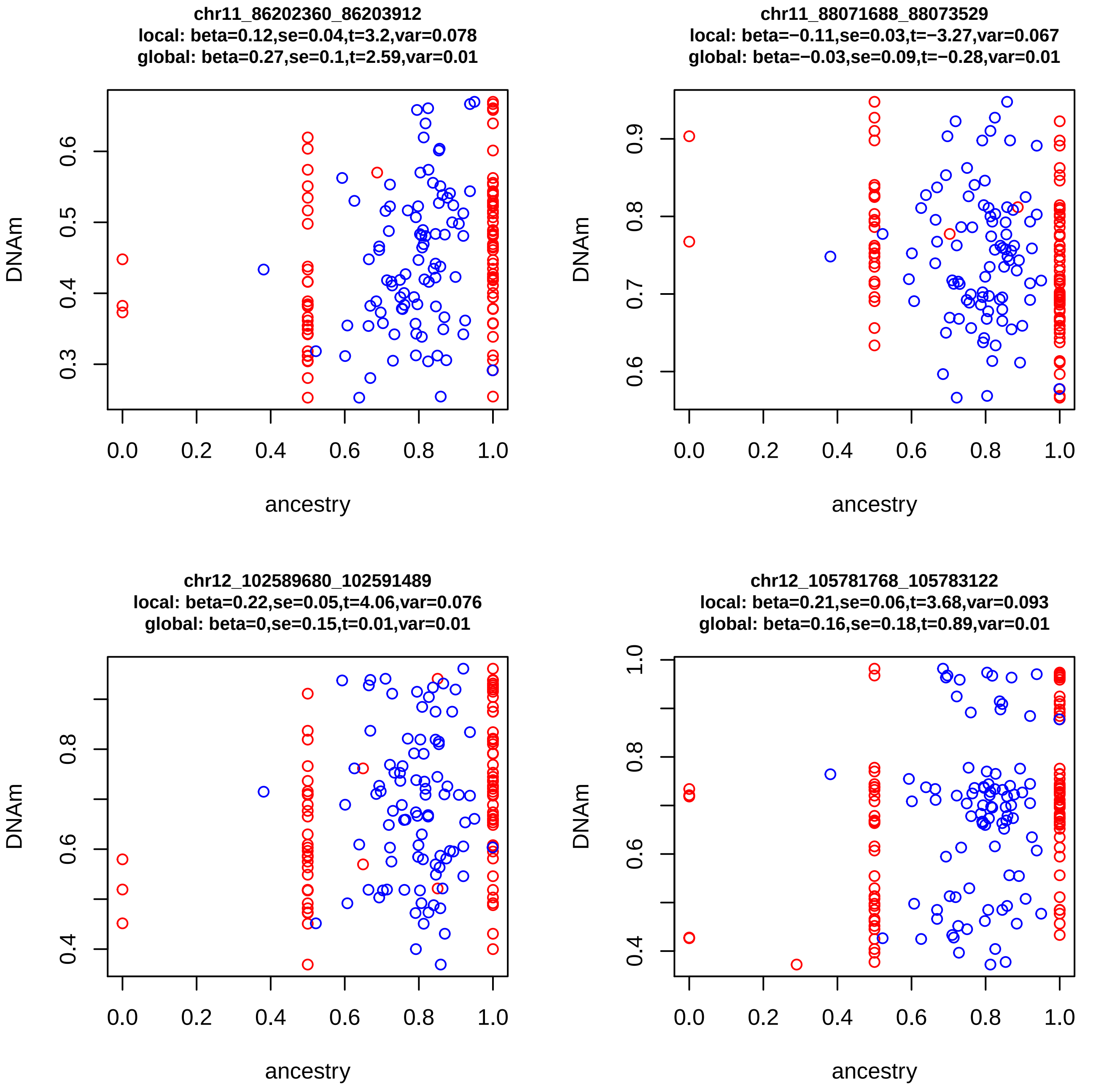
chr11_86202360_86203912
local: beta=0.12,se=0.04,t=3.2,var=0.078
global: beta=0.27,se=0.1,t=2.59,var=0.01
DNAm
ancestry
chr11_88071688_88073529
local: beta=−0.11,se=0.03,t=−3.27,var=0.067
global: beta=−0.03,se=0.09,t=−0.28,var=0.01
DNAm
ancestry
chr12_102589680_102591489
local: beta=0.22,se=0.05,t=4.06,var=0.076
global: beta=0,se=0.15,t=0.01,var=0.01
DNAm
ancestry
chr12_105781768_105783122
local: beta=0.21,se=0.06,t=3.68,var=0.093
global: beta=0.16,se=0.18,t=0.89,var=0.01
DNAm
ancestry

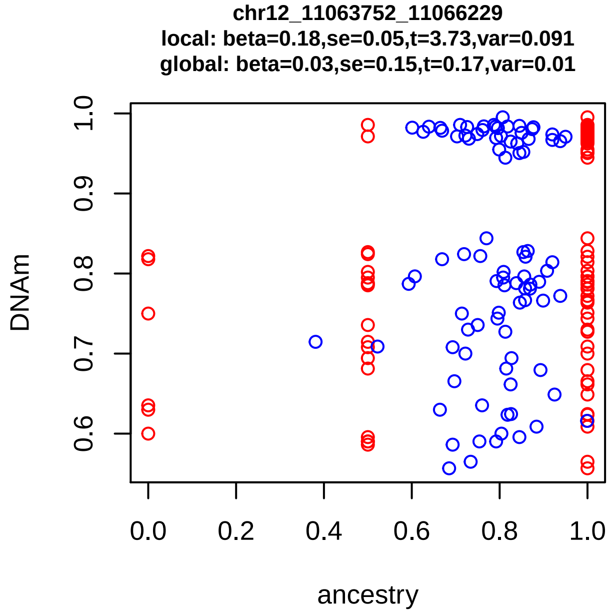
chr12_11063752_11066229
local: beta=0.18,se=0.05,t=3.73,var=0.091
global: beta=0.03,se=0.15,t=0.17,var=0.01
DNAm
ancestry

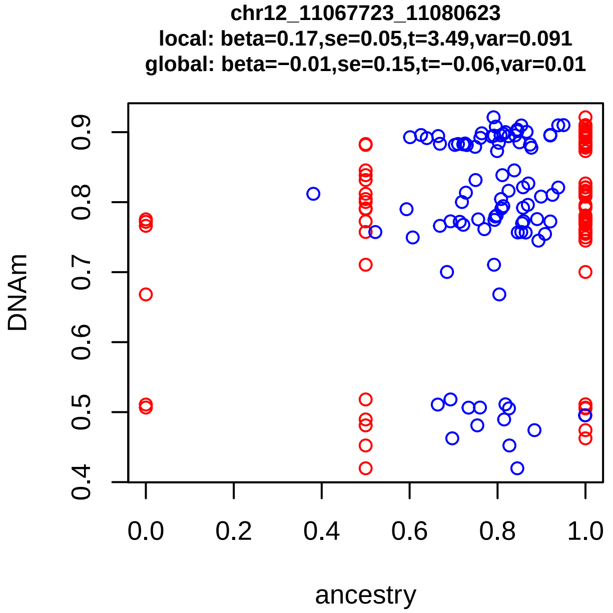
chr12_11067723_11080623
local: beta=0.17,se=0.05,t=3.49,var=0.091
global: beta=−0.01,se=0.15,t=−0.06,var=0.01
DNAm
ancestry

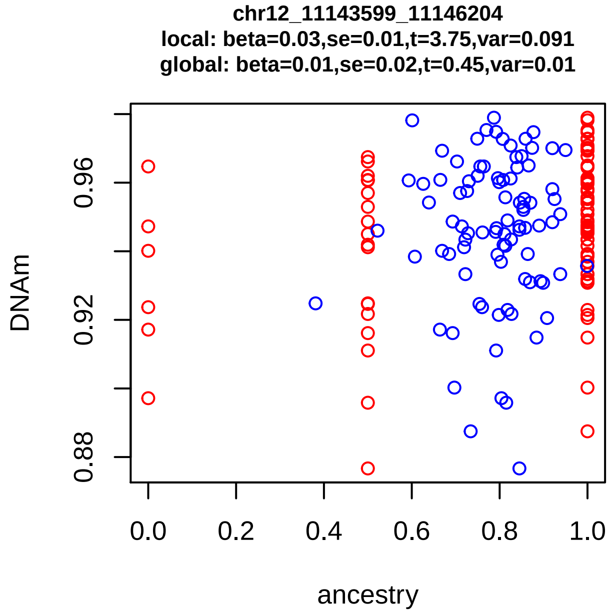
chr12_11143599_11146204
local: beta=0.03,se=0.01,t=3.75,var=0.091
global: beta=0.01,se=0.02,t=0.45,var=0.01
DNAm
ancestry

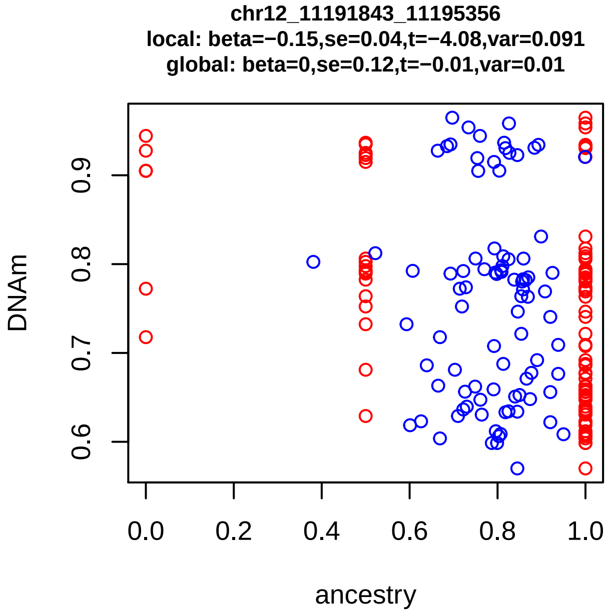
chr12_11191843_11195356
local: beta=−0.15,se=0.04,t=−4.08,var=0.091
global: beta=0,se=0.12,t=−0.01,var=0.01
DNAm
ancestry

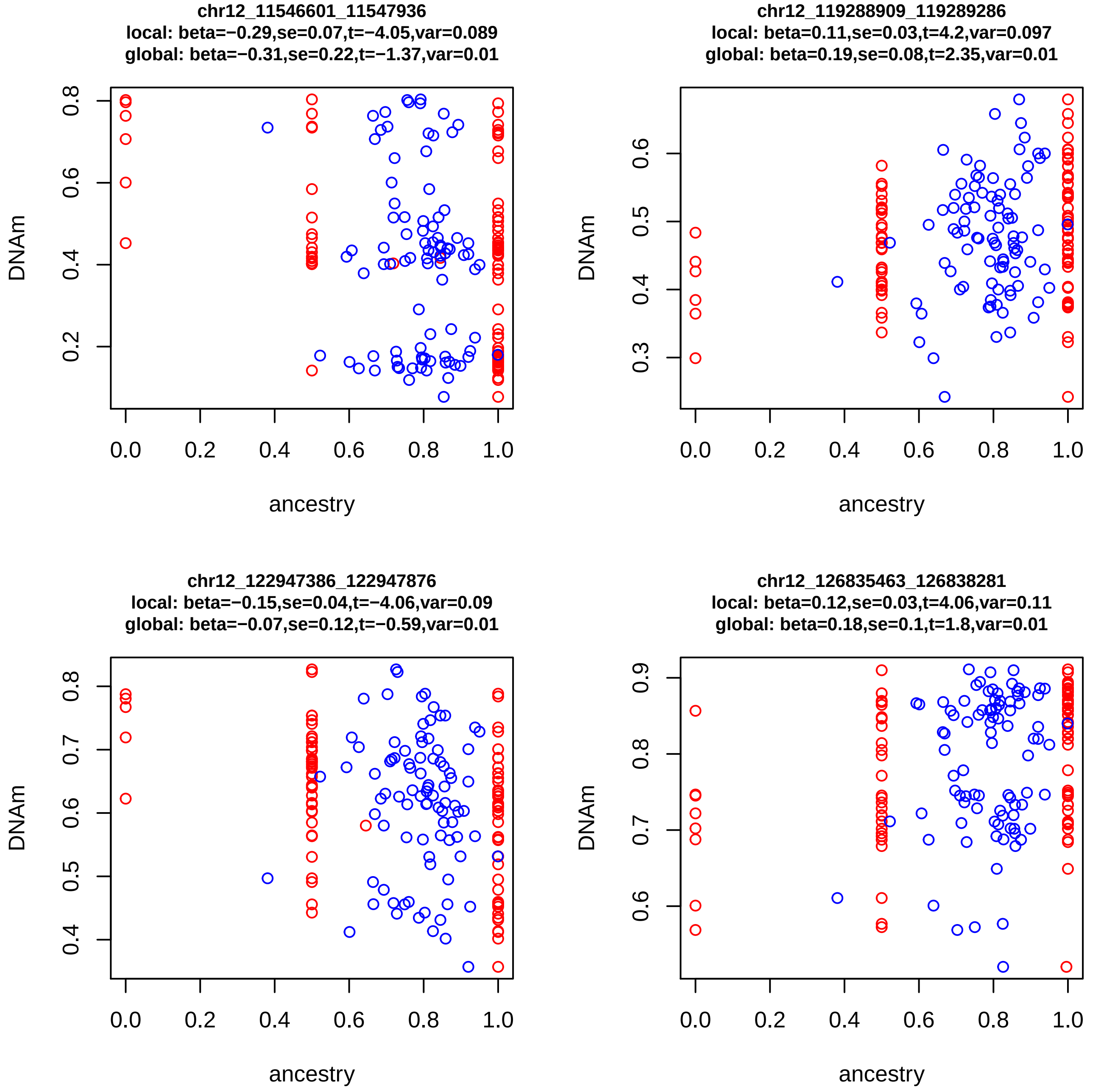
chr12_11546601_11547936
local: beta=−0.29,se=0.07,t=−4.05,var=0.089
global: beta=−0.31,se=0.22,t=−1.37,var=0.01
DNAm
0.2
0.4
0.6
0.8
0.0
0.2
0.4
0.6
0.8
1.0
ancestry
chr12_119288909_119289286
local: beta=0.11,se=0.03,t=4.2,var=0.097
global: beta=0.19,se=0.08,t=2.35,var=0.01
DNAm
0.3
0.4
0.5
0.6
0.0
0.2
0.4
0.6
0.8
1.0
ancestry
chr12_122947386_122947876
local: beta=−0.15,se=0.04,t=−4.06,var=0.09
global: beta=−0.07,se=0.12,t=−0.59,var=0.01
DNAm
0.4
0.5
0.6
0.7
0.8
0.0
0.2
0.4
0.6
0.8
1.0
ancestry
chr12_126835463_126838281
local: beta=0.12,se=0.03,t=4.06,var=0.11
global: beta=0.18,se=0.1,t=1.8,var=0.01
DNAm
0.6
0.7
0.8
0.9
0.0
0.2
0.4
0.6
0.8
1.0
ancestry

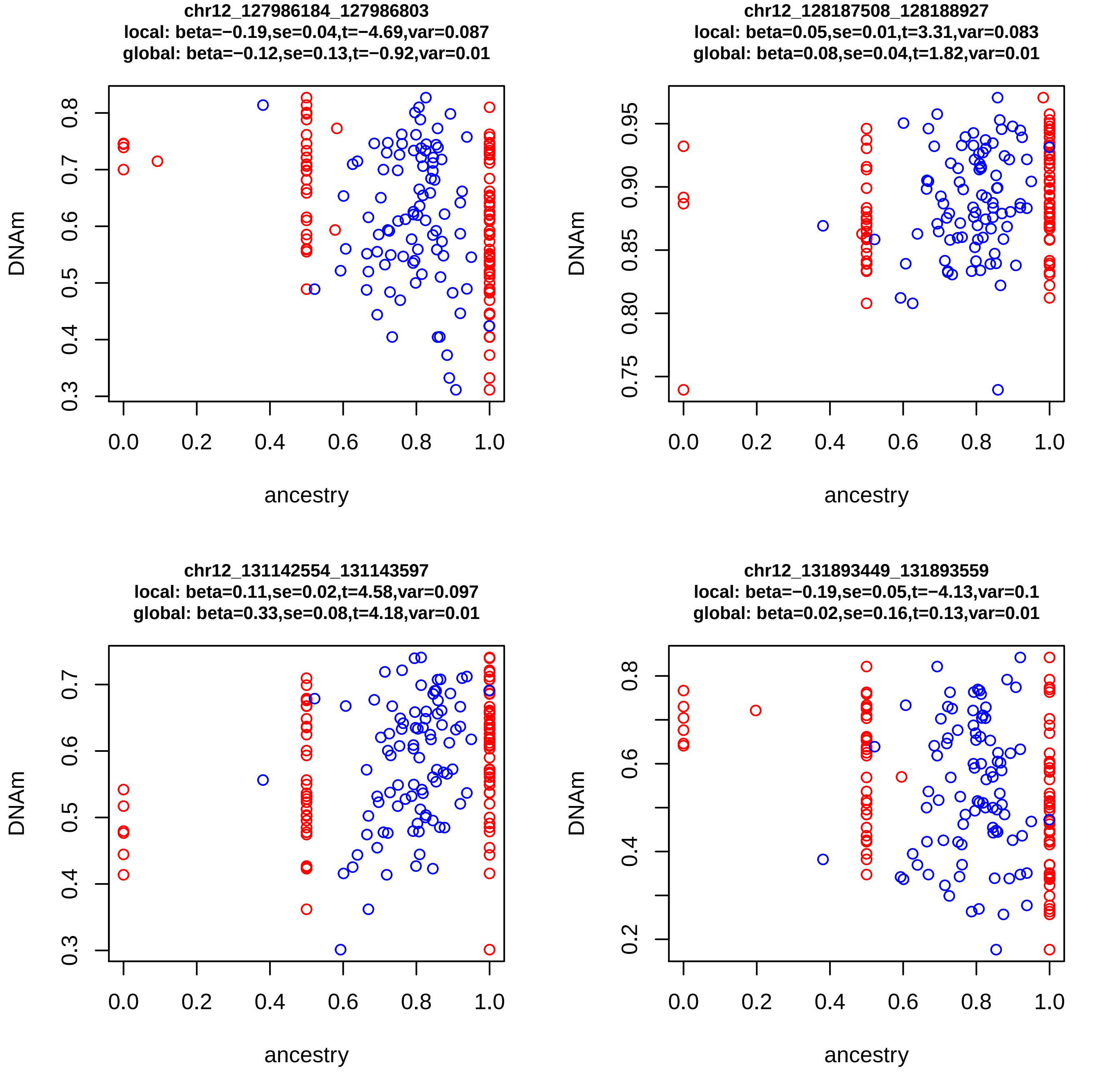

chr12_127986184_127986803
local: beta=−0.19,se=0.04,t=−4.69,var=0.087
global: beta=−0.12,se=0.13,t=−0.92,var=0.01
DNAm
ancestry
chr12_128187508_128188927
local: beta=0.05,se=0.01,t=3.31,var=0.083
global: beta=0.08,se=0.04,t=1.82,var=0.01
DNAm
ancestry
chr12_131142554_131143597
local: beta=0.11,se=0.02,t=4.58,var=0.097
global: beta=0.33,se=0.08,t=4.18,var=0.01
DNAm
ancestry
chr12_131893449_131893559
local: beta=−0.19,se=0.05,t=−4.13,var=0.1
global: beta=0.02,se=0.16,t=0.13,var=0.01
DNAm
ancestry

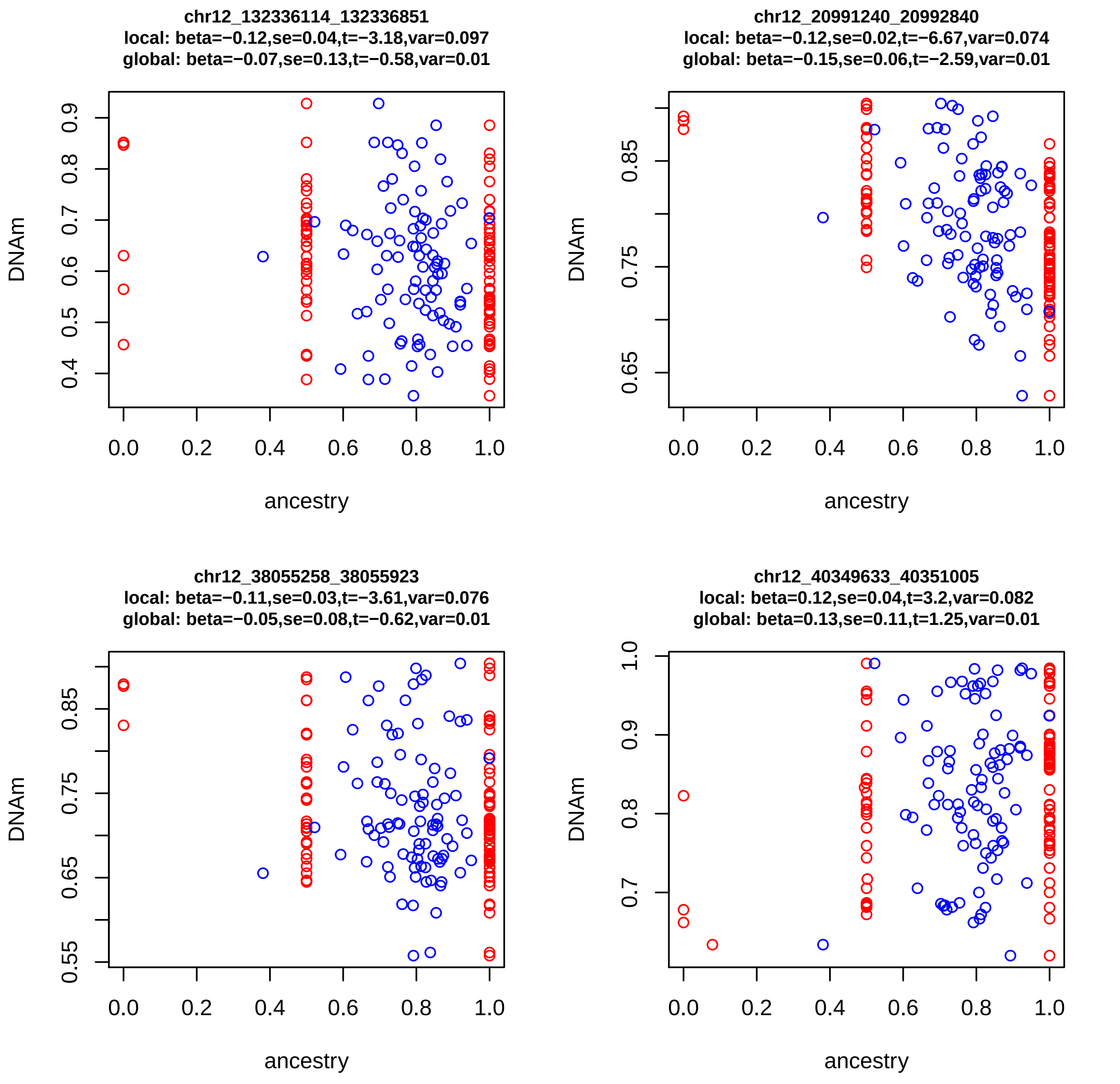

chr12_132336114_132336851
local: beta=−0.12,se=0.04,t=−3.18,var=0.097
global: beta=−0.07,se=0.13,t=−0.58,var=0.01
DNAm
ancestry
chr12_20991240_20992840
local: beta=−0.12,se=0.02,t=−6.67,var=0.074
global: beta=−0.15,se=0.06,t=−2.59,var=0.01
DNAm
ancestry
chr12_38055258_38055923
local: beta=−0.11,se=0.03,t=−3.61,var=0.076
global: beta=−0.05,se=0.08,t=−0.62,var=0.01
DNAm
ancestry
chr12_40349633_40351005
local: beta=0.12,se=0.04,t=3.2,var=0.082
global: beta=0.13,se=0.11,t=1.25,var=0.01
DNAm
ancestry

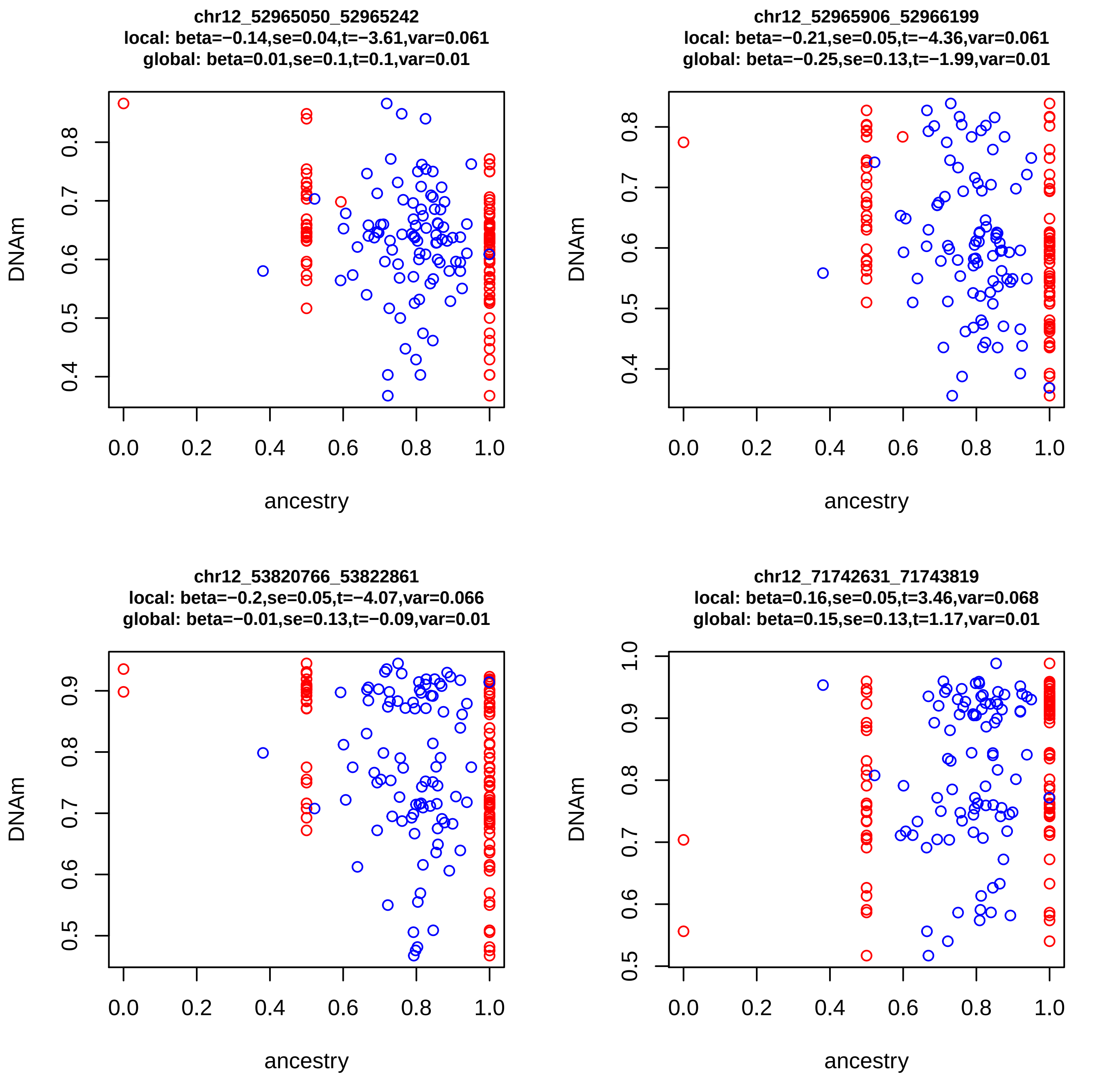
chr12_52965050_52965242
local: beta=−0.14,se=0.04,t=−3.61,var=0.061
global: beta=0.01,se=0.1,t=0.1,var=0.01
DNAm
0.4
0.5
0.6
0.7
0.8
0.0
0.2
0.4
0.6
0.8
1.0
ancestry
chr12_52965906_52966199
local: beta=−0.21,se=0.05,t=−4.36,var=0.061
global: beta=−0.25,se=0.13,t=−1.99,var=0.01
DNAm
0.4
0.5
0.6
0.7
0.8
0.0
0.2
0.4
0.6
0.8
1.0
ancestry
chr12_53820766_53822861
local: beta=−0.2,se=0.05,t=−4.07,var=0.066
global: beta=−0.01,se=0.13,t=−0.09,var=0.01
DNAm
0.5
0.6
0.7
0.8
0.9
0.0
0.2
0.4
0.6
0.8
1.0
ancestry
chr12_71742631_71743819
local: beta=0.16,se=0.05,t=3.46,var=0.068
global: beta=0.15,se=0.13,t=1.17,var=0.01
DNAm
0.5
0.6
0.7
0.8
0.9
1.0
0.0
0.2
0.4
0.6
0.8
1.0
ancestry

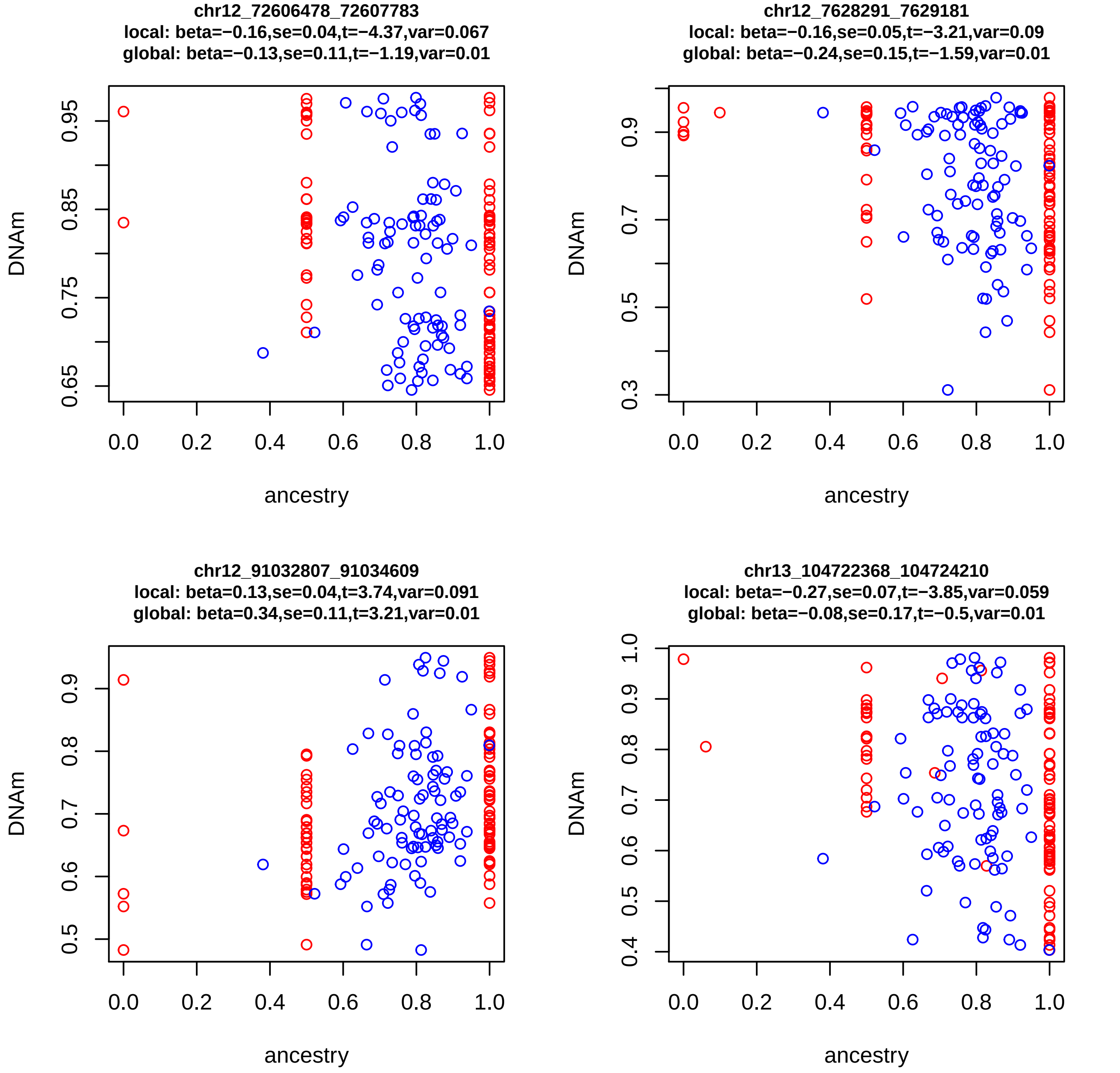

chr12_72606478_72607783
local: beta=−0.16,se=0.04,t=−4.37,var=0.067
global: beta=−0.13,se=0.11,t=−1.19,var=0.01
chr12_7628291_7629181
local: beta=−0.16,se=0.05,t=−3.21,var=0.09
global: beta=−0.24,se=0.15,t=−1.59,var=0.01
chr12_91032807_91034609
local: beta=0.13,se=0.04,t=3.74,var=0.091
global: beta=0.34,se=0.11,t=3.21,var=0.01
chr13_104722368_104724210
local: beta=−0.27,se=0.07,t=−3.85,var=0.059
global: beta=−0.08,se=0.17,t=−0.5,var=0.01
DNAm
ancestry
0.0
0.2
0.4
0.6
0.8
1.0
0.65
0.75
0.85
0.95
0.3
0.5
0.7
0.9
0.5
0.6
0.7
0.8
0.9
0.4
1.0

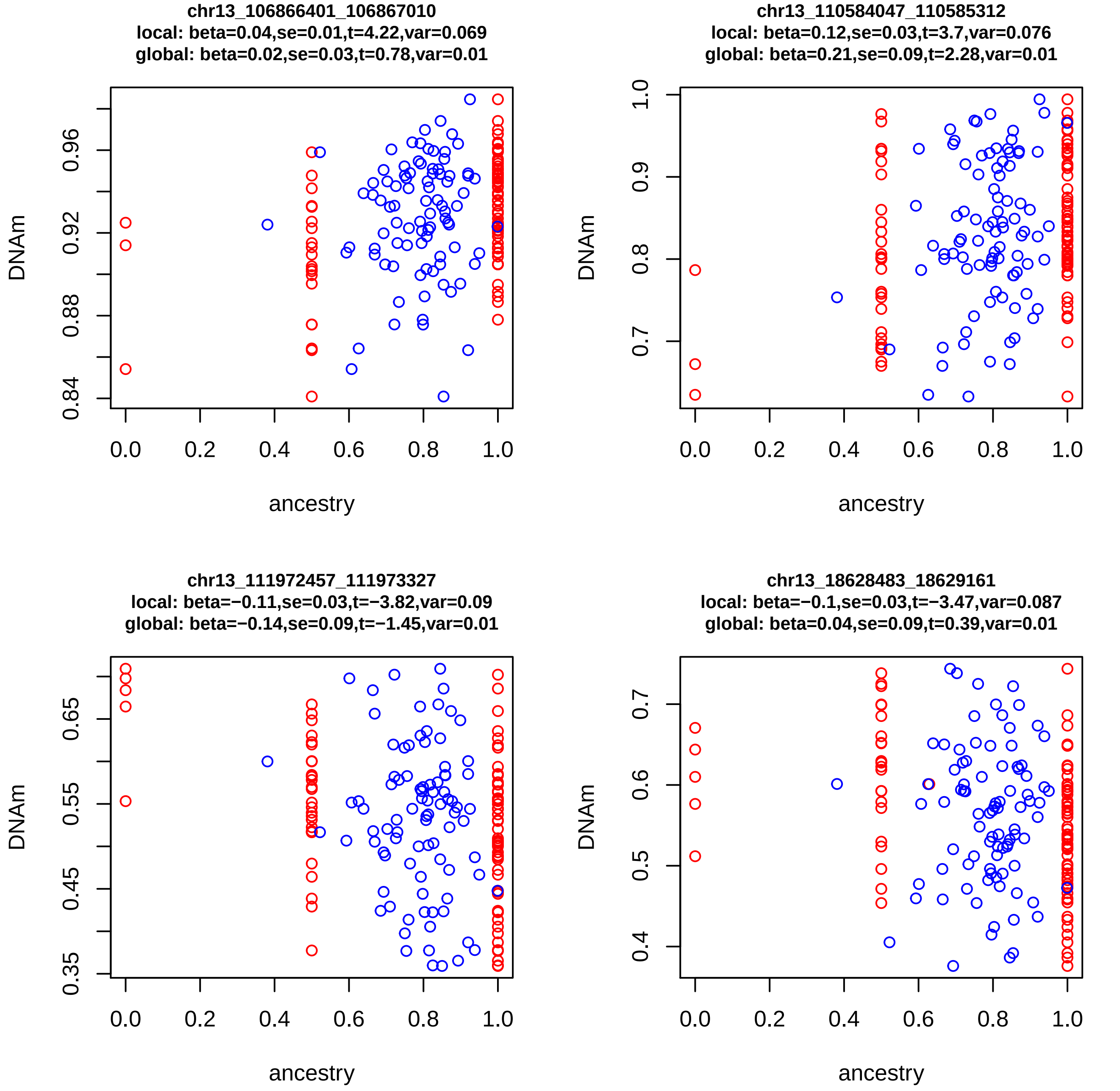
chr13_106866401_106867010
local: beta=0.04,se=0.01,t=4.22,var=0.069
global: beta=0.02,se=0.03,t=0.78,var=0.01
DNAm
0.84
0.88
0.92
0.96
ancestry
0.0
0.2
0.4
0.6
0.8
1.0
chr13_110584047_110585312
local: beta=0.12,se=0.03,t=3.7,var=0.076
global: beta=0.21,se=0.09,t=2.28,var=0.01
DNAm
0.7
0.8
0.9
1.0
ancestry
0.0
0.2
0.4
0.6
0.8
1.0
chr13_111972457_111973327
local: beta=−0.11,se=0.03,t=−3.82,var=0.09
global: beta=−0.14,se=0.09,t=−1.45,var=0.01
DNAm
0.35
0.45
0.55
0.65
ancestry
0.0
0.2
0.4
0.6
0.8
1.0
chr13_18628483_18629161
local: beta=−0.1,se=0.03,t=−3.47,var=0.087
global: beta=0.04,se=0.09,t=0.39,var=0.01
DNAm
0.4
0.5
0.6
0.7
ancestry
0.0
0.2
0.4
0.6
0.8
1.0

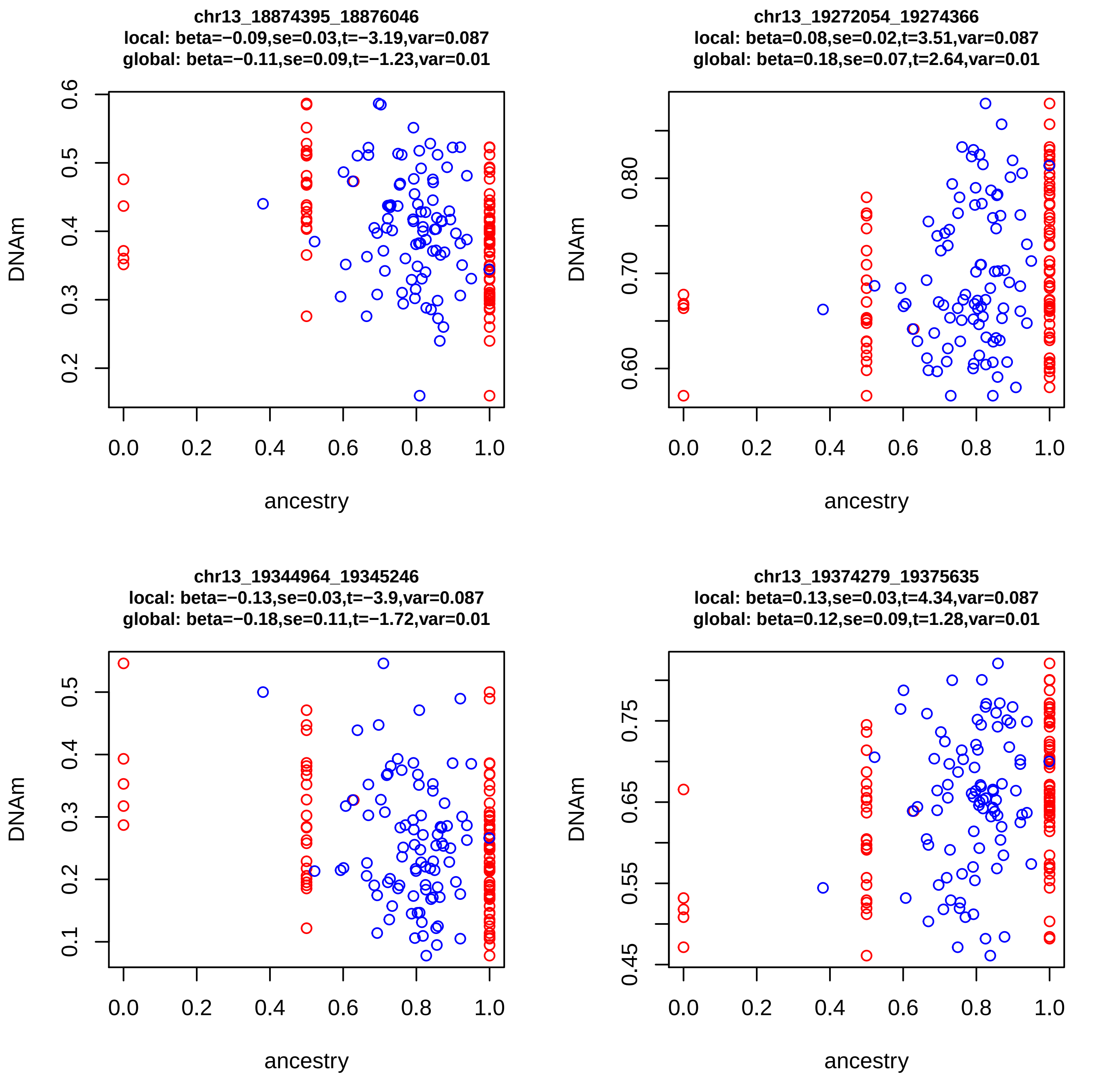
chr13_18874395_18876046
local: beta=−0.09,se=0.03,t=−3.19,var=0.087
global: beta=−0.11,se=0.09,t=−1.23,var=0.01
DNAm
ancestry
chr13_19272054_19274366
local: beta=0.08,se=0.02,t=3.51,var=0.087
global: beta=0.18,se=0.07,t=2.64,var=0.01
DNAm
ancestry
chr13_19344964_19345246
local: beta=−0.13,se=0.03,t=−3.9,var=0.087
global: beta=−0.18,se=0.11,t=−1.72,var=0.01
DNAm
ancestry
chr13_19374279_19375635
local: beta=0.13,se=0.03,t=4.34,var=0.087
global: beta=0.12,se=0.09,t=1.28,var=0.01
DNAm
ancestry

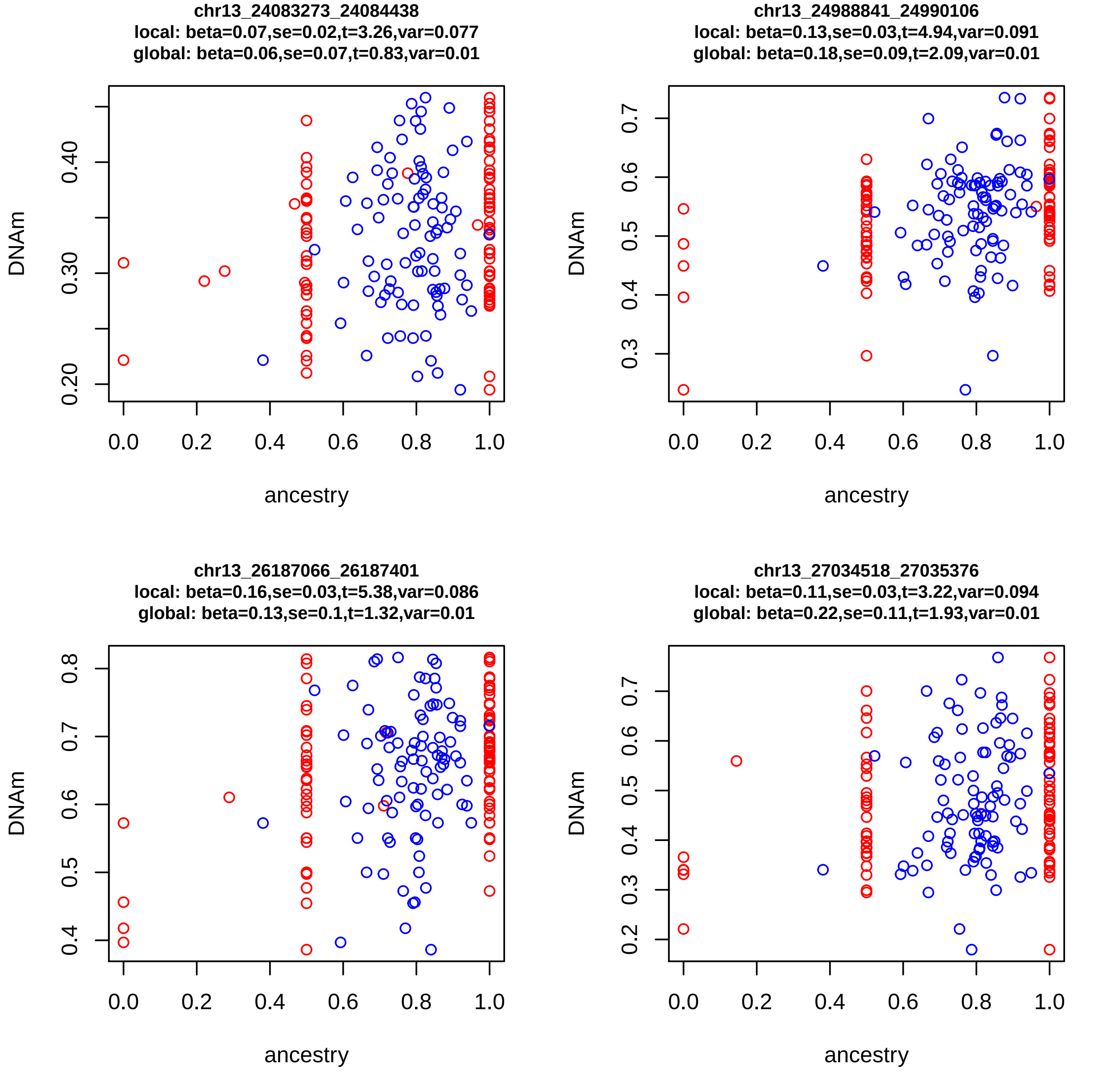
chr13_24083273_24084438
local: beta=0.07,se=0.02,t=3.26,var=0.077
global: beta=0.06,se=0.07,t=0.83,var=0.01
DNAm
ancestry
chr13_24988841_24990106
local: beta=0.13,se=0.03,t=4.94,var=0.091
global: beta=0.18,se=0.09,t=2.09,var=0.01
DNAm
ancestry
chr13_26187066_26187401
local: beta=0.16,se=0.03,t=5.38,var=0.086
global: beta=0.13,se=0.1,t=1.32,var=0.01
DNAm
ancestry
chr13_27034518_27035376
local: beta=0.11,se=0.03,t=3.22,var=0.094
global: beta=0.22,se=0.11,t=1.93,var=0.01
DNAm
ancestry

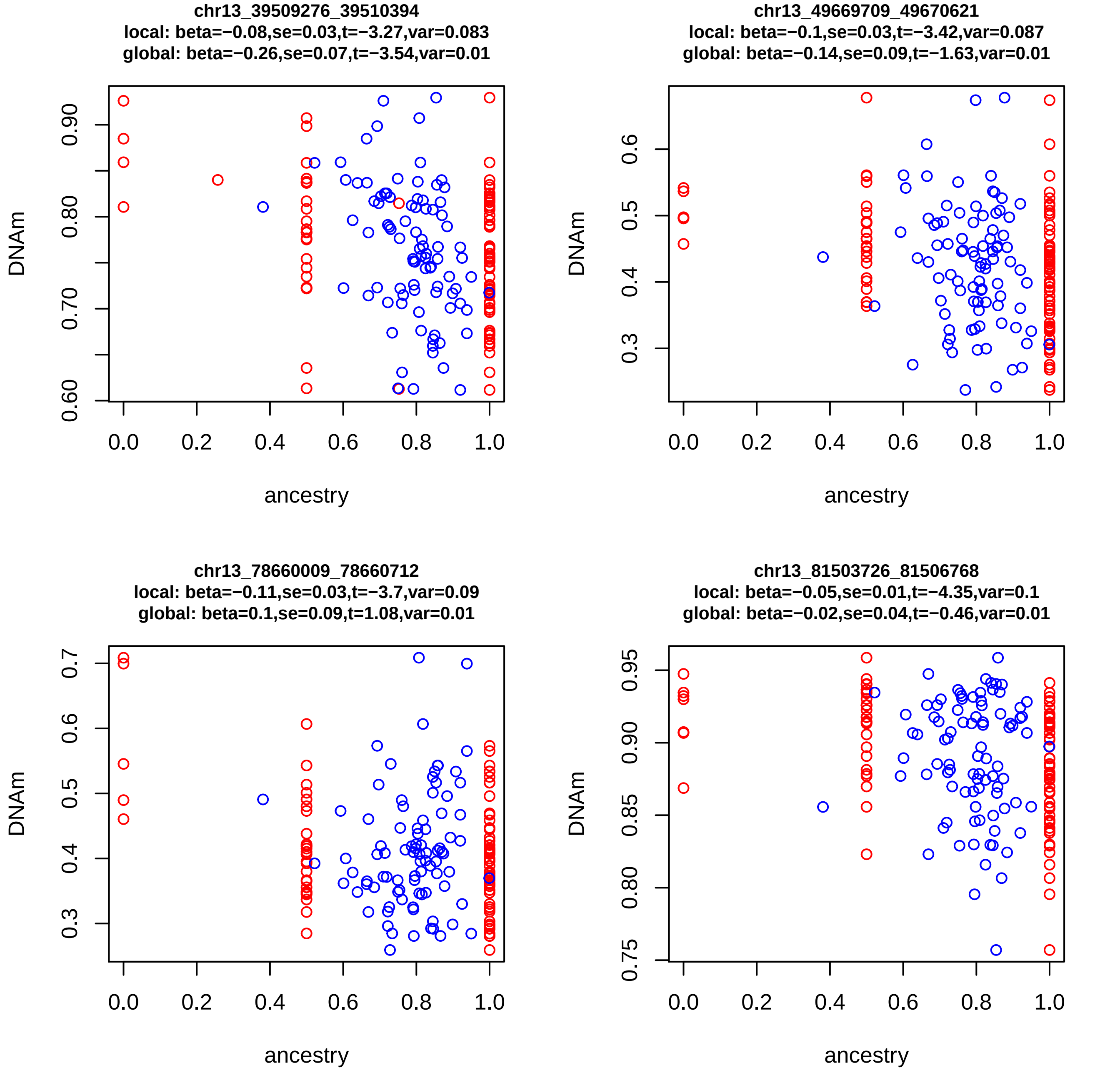
chr13_39509276_39510394
local: beta=−0.08,se=0.03,t=−3.27,var=0.083
global: beta=−0.26,se=0.07,t=−3.54,var=0.01
chr13_49669709_49670621
local: beta=−0.1,se=0.03,t=−3.42,var=0.087
global: beta=−0.14,se=0.09,t=−1.63,var=0.01
chr13_78660009_78660712
local: beta=−0.11,se=0.03,t=−3.7,var=0.09
global: beta=0.1,se=0.09,t=1.08,var=0.01
chr13_81503726_81506768
local: beta=−0.05,se=0.01,t=−4.35,var=0.1
global: beta=−0.02,se=0.04,t=−0.46,var=0.01
DNAm
ancestry

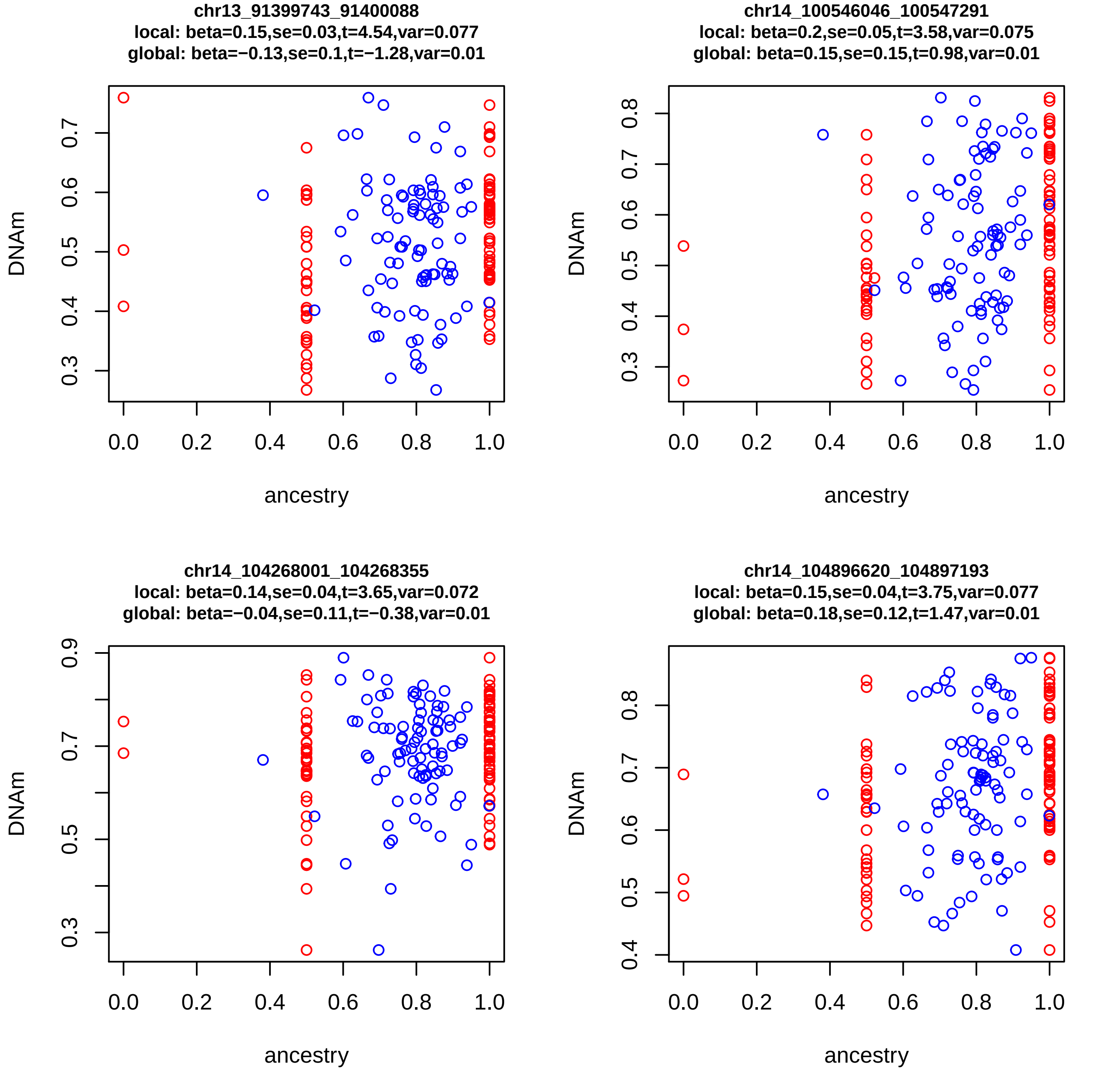
chr13_91399743_91400088
local: beta=0.15,se=0.03,t=4.54,var=0.077
global: beta=−0.13,se=0.1,t=−1.28,var=0.01
DNAm
0.3
0.4
0.5
0.6
0.7
0.0
0.2
0.4
0.6
0.8
1.0
ancestry
chr14_100546046_100547291
local: beta=0.2,se=0.05,t=3.58,var=0.075
global: beta=0.15,se=0.15,t=0.98,var=0.01
DNAm
0.3
0.4
0.5
0.6
0.7
0.8
0.0
0.2
0.4
0.6
0.8
1.0
ancestry
chr14_104268001_104268355
local: beta=0.14,se=0.04,t=3.65,var=0.072
global: beta=−0.04,se=0.11,t=−0.38,var=0.01
DNAm
0.3
0.5
0.7
0.9
0.0
0.2
0.4
0.6
0.8
1.0
ancestry
chr14_104896620_104897193
local: beta=0.15,se=0.04,t=3.75,var=0.077
global: beta=0.18,se=0.12,t=1.47,var=0.01
DNAm
0.4
0.5
0.6
0.7
0.8
0.0
0.2
0.4
0.6
0.8
1.0
ancestry

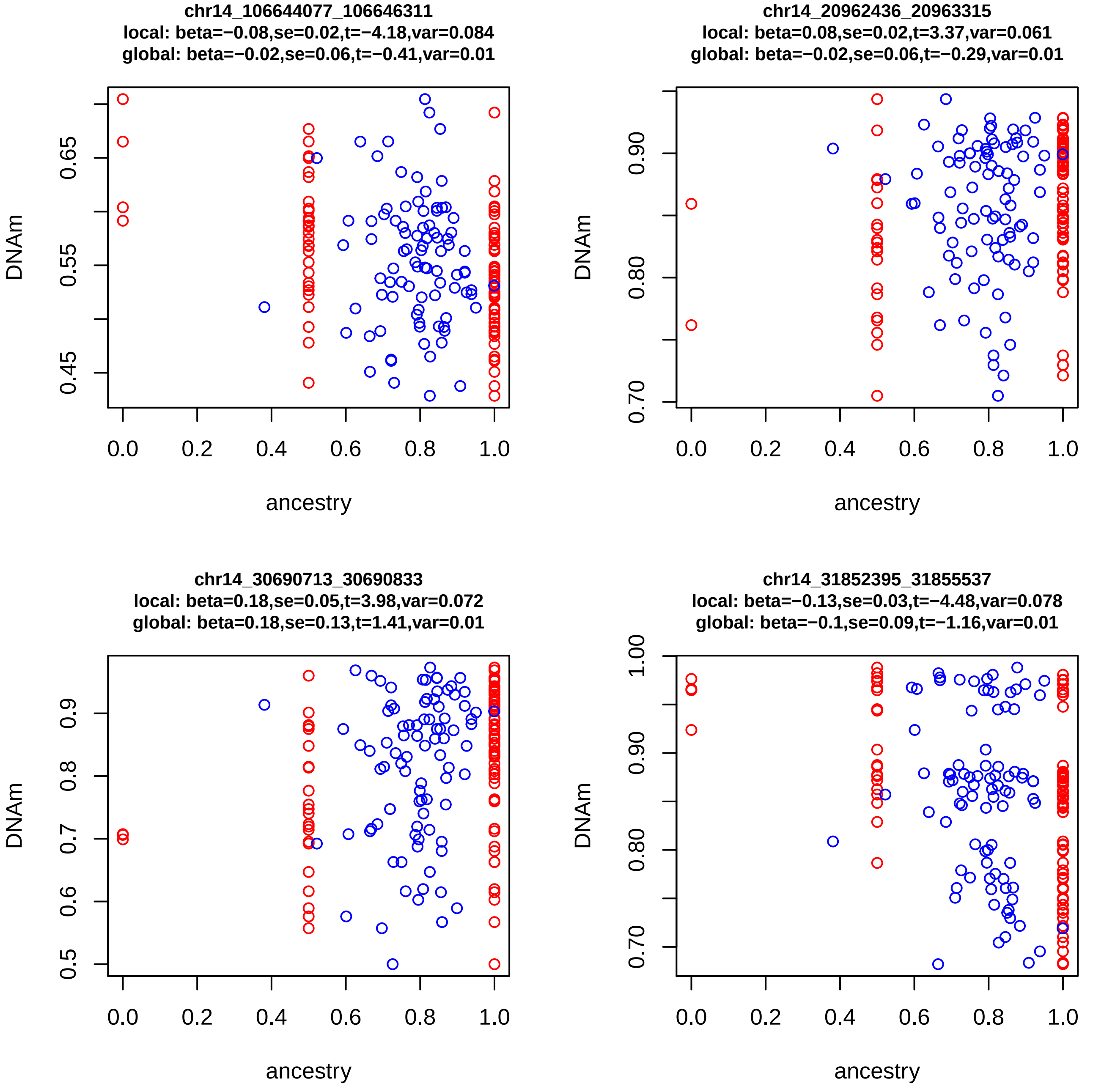
chr14_106644077_106646311
local: beta=−0.08,se=0.02,t=−4.18,var=0.084
global: beta=−0.02,se=0.06,t=−0.41,var=0.01
DNAm
0.45
0.55
0.65
ancestry
0.0
0.2
0.4
0.6
0.8
1.0
chr14_20962436_20963315
local: beta=0.08,se=0.02,t=3.37,var=0.061
global: beta=−0.02,se=0.06,t=−0.29,var=0.01
DNAm
0.70
0.80
0.90
ancestry
0.0
0.2
0.4
0.6
0.8
1.0
chr14_30690713_30690833
local: beta=0.18,se=0.05,t=3.98,var=0.072
global: beta=0.18,se=0.13,t=1.41,var=0.01
DNAm
0.5
0.6
0.7
0.8
0.9
ancestry
0.0
0.2
0.4
0.6
0.8
1.0
chr14_31852395_31855537
local: beta=−0.13,se=0.03,t=−4.48,var=0.078
global: beta=−0.1,se=0.09,t=−1.16,var=0.01
DNAm
0.70
0.80
0.90
1.00
ancestry
0.0
0.2
0.4
0.6
0.8
1.0

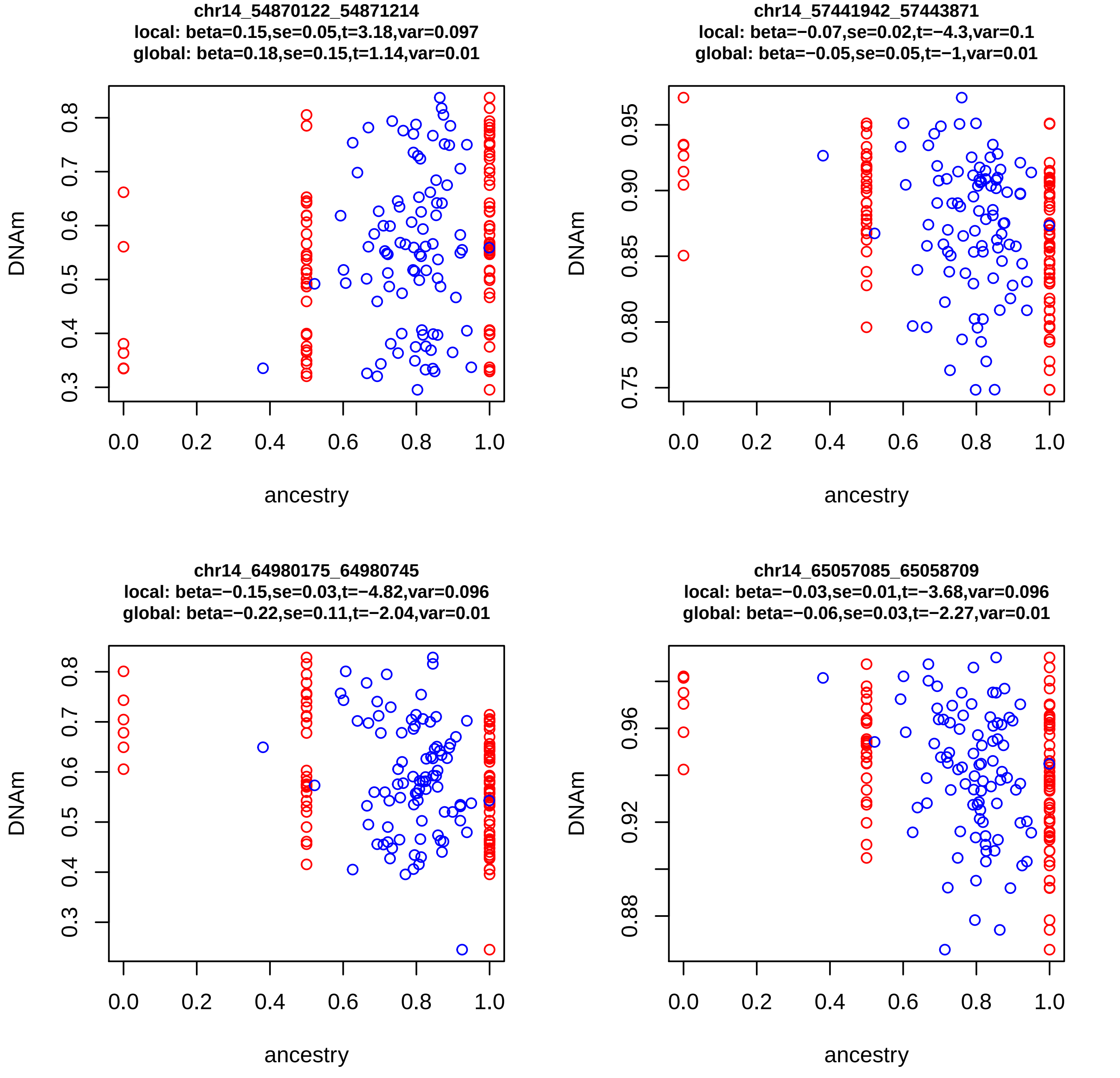
chr14_54870122_54871214
local: beta=0.15,se=0.05,t=3.18,var=0.097
global: beta=0.18,se=0.15,t=1.14,var=0.01
DNAm
0.3 0.4 0.5 0.6 0.7 0.8
0.0 0.2 0.4 0.6 0.8 1.0
ancestry
chr14_57441942_57443871
local: beta=−0.07,se=0.02,t=−4.3,var=0.1
global: beta=−0.05,se=0.05,t=−1,var=0.01
DNAm
0.75 0.80 0.85 0.90 0.95
0.0 0.2 0.4 0.6 0.8 1.0
ancestry
chr14_64980175_64980745
local: beta=−0.15,se=0.03,t=−4.82,var=0.096
global: beta=−0.22,se=0.11,t=−2.04,var=0.01
DNAm
0.3 0.4 0.5 0.6 0.7 0.8
0.0 0.2 0.4 0.6 0.8 1.0
ancestry
chr14_65057085_65058709
local: beta=−0.03,se=0.01,t=−3.68,var=0.096
global: beta=−0.06,se=0.03,t=−2.27,var=0.01
DNAm
0.88 0.92 0.96
0.0 0.2 0.4 0.6 0.8 1.0
ancestry

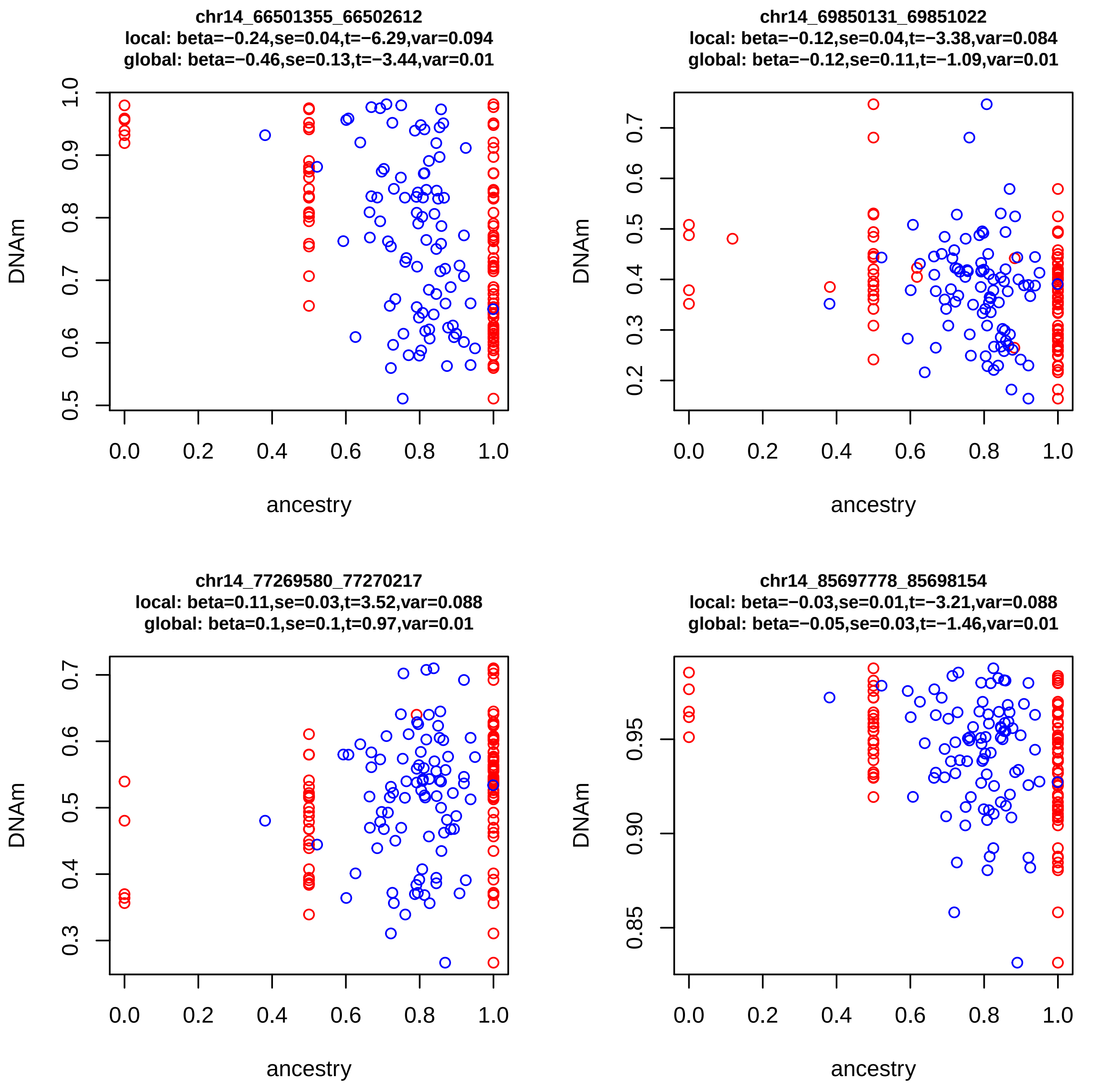
chr14_66501355_66502612
local: beta=−0.24,se=0.04,t=−6.29,var=0.094
global: beta=−0.46,se=0.13,t=−3.44,var=0.01
DNAm
ancestry
chr14_69850131_69851022
local: beta=−0.12,se=0.04,t=−3.38,var=0.084
global: beta=−0.12,se=0.11,t=−1.09,var=0.01
DNAm
ancestry
chr14_77269580_77270217
local: beta=0.11,se=0.03,t=3.52,var=0.088
global: beta=0.1,se=0.1,t=0.97,var=0.01
DNAm
ancestry
chr14_85697778_85698154
local: beta=−0.03,se=0.01,t=−3.21,var=0.088
global: beta=−0.05,se=0.03,t=−1.46,var=0.01
DNAm
ancestry

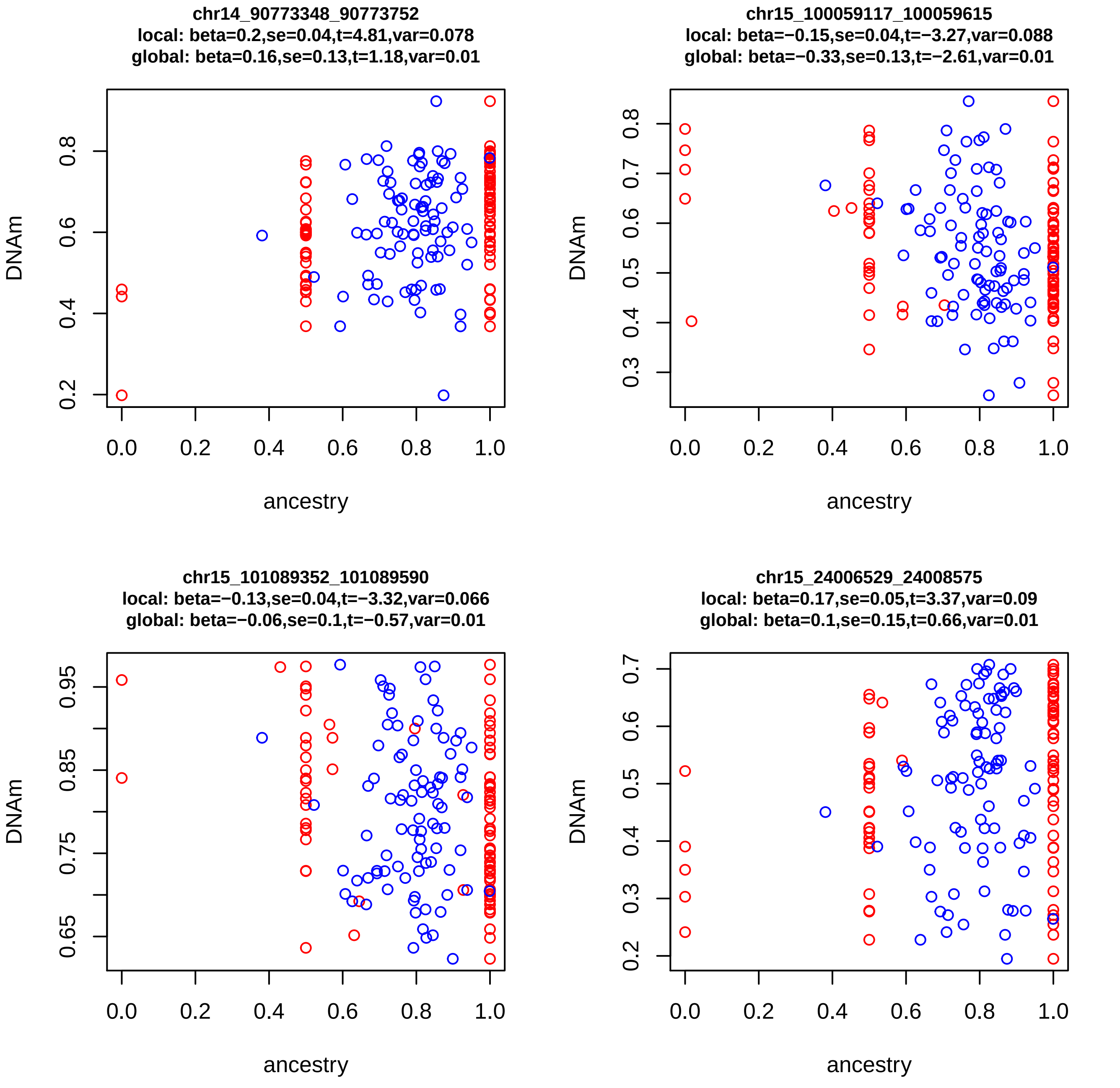

chr14_90773348_90773752
local: beta=0.2,se=0.04,t=4.81,var=0.078
global: beta=0.16,se=0.13,t=1.18,var=0.01
DNAm
0.2
0.4
0.6
0.8
0.0
0.2
0.4
0.6
0.8
1.0
ancestry
chr15_100059117_100059615
local: beta=−0.15,se=0.04,t=−3.27,var=0.088
global: beta=−0.33,se=0.13,t=−2.61,var=0.01
DNAm
0.3
0.4
0.5
0.6
0.7
0.8
0.0
0.2
0.4
0.6
0.8
1.0
ancestry
chr15_101089352_101089590
local: beta=−0.13,se=0.04,t=−3.32,var=0.066
global: beta=−0.06,se=0.1,t=−0.57,var=0.01
DNAm
0.65
0.75
0.85
0.95
0.0
0.2
0.4
0.6
0.8
1.0
ancestry
chr15_24006529_24008575
local: beta=0.17,se=0.05,t=3.37,var=0.09
global: beta=0.1,se=0.15,t=0.66,var=0.01
DNAm
0.2
0.3
0.4
0.5
0.6
0.7
0.0
0.2
0.4
0.6
0.8
1.0
ancestry

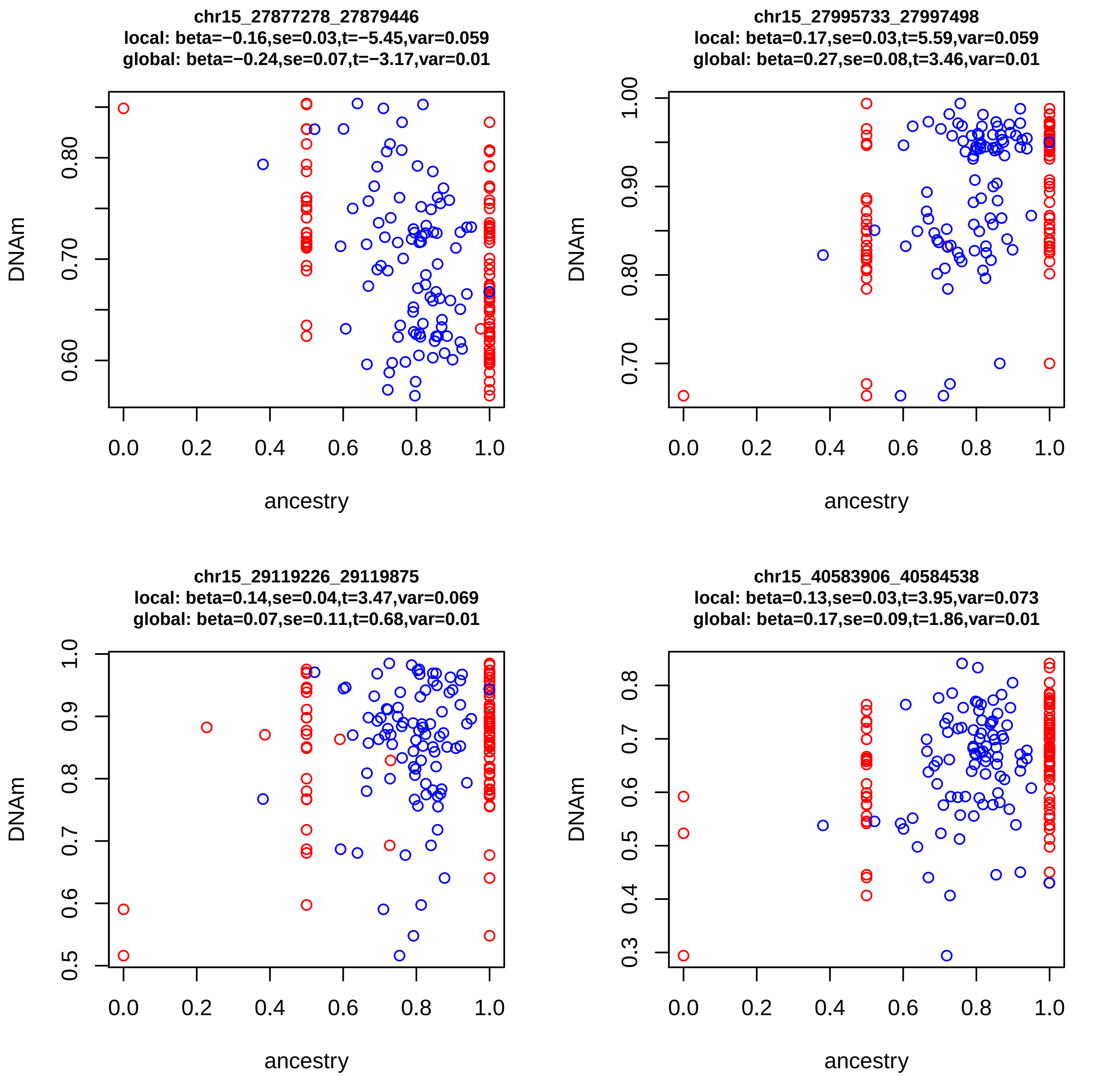
chr15_27877278_27879446
local: beta=−0.16,se=0.03,t=−5.45,var=0.059
global: beta=−0.24,se=0.07,t=−3.17,var=0.01
DNAm
ancestry
chr15_27995733_27997498
local: beta=0.17,se=0.03,t=5.59,var=0.059
global: beta=0.27,se=0.08,t=3.46,var=0.01
DNAm
ancestry
chr15_29119226_29119875
local: beta=0.14,se=0.04,t=3.47,var=0.069
global: beta=0.07,se=0.11,t=0.68,var=0.01
DNAm
ancestry
chr15_40583906_40584538
local: beta=0.13,se=0.03,t=3.95,var=0.073
global: beta=0.17,se=0.09,t=1.86,var=0.01
DNAm
ancestry

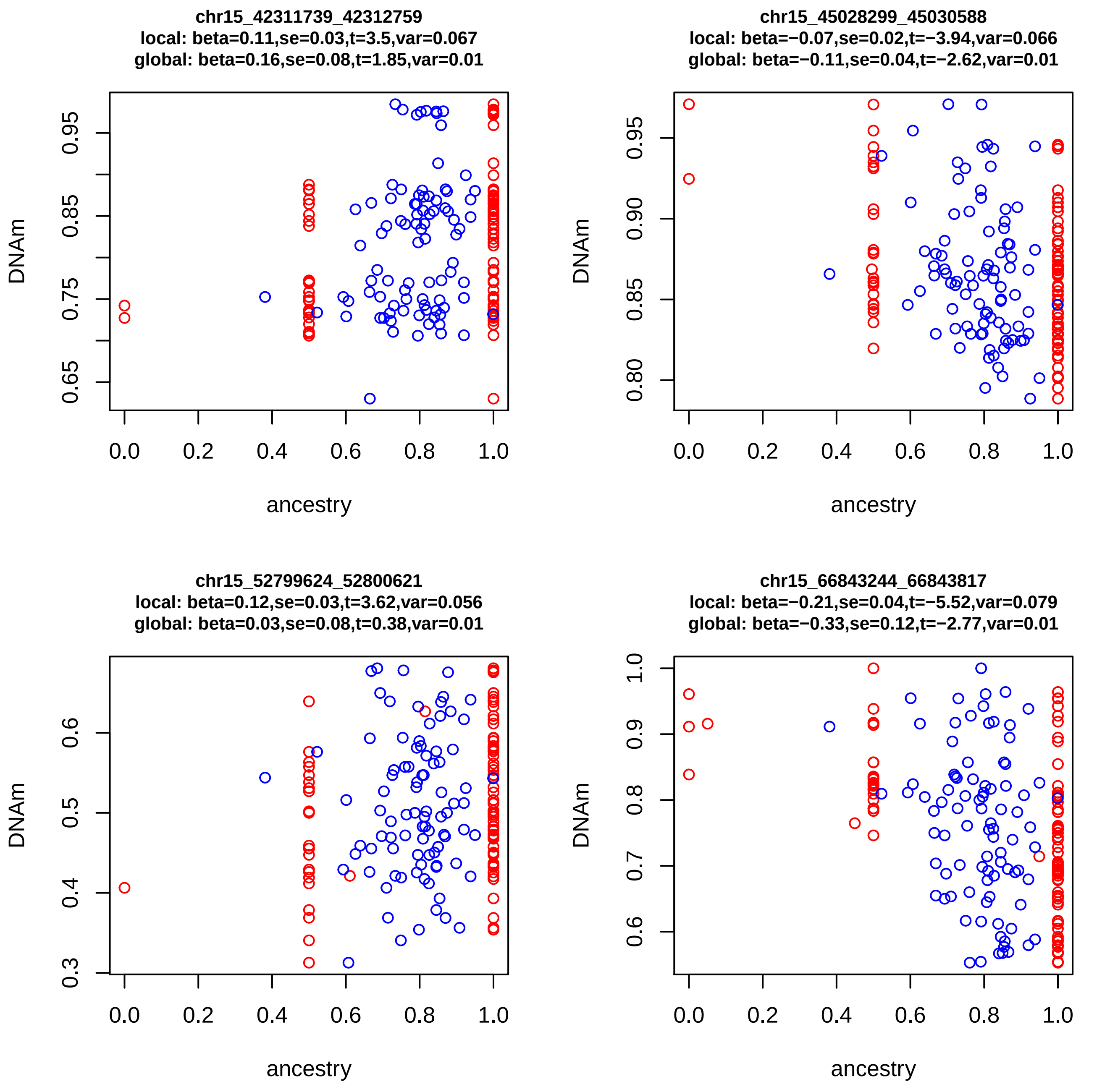
chr15_42311739_42312759
local: beta=0.11,se=0.03,t=3.5,var=0.067
global: beta=0.16,se=0.08,t=1.85,var=0.01
DNAm
ancestry
chr15_45028299_45030588
local: beta=−0.07,se=0.02,t=−3.94,var=0.066
global: beta=−0.11,se=0.04,t=−2.62,var=0.01
DNAm
ancestry
chr15_52799624_52800621
local: beta=0.12,se=0.03,t=3.62,var=0.056
global: beta=0.03,se=0.08,t=0.38,var=0.01
DNAm
ancestry
chr15_66843244_66843817
local: beta=−0.21,se=0.04,t=−5.52,var=0.079
global: beta=−0.33,se=0.12,t=−2.77,var=0.01
DNAm
ancestry

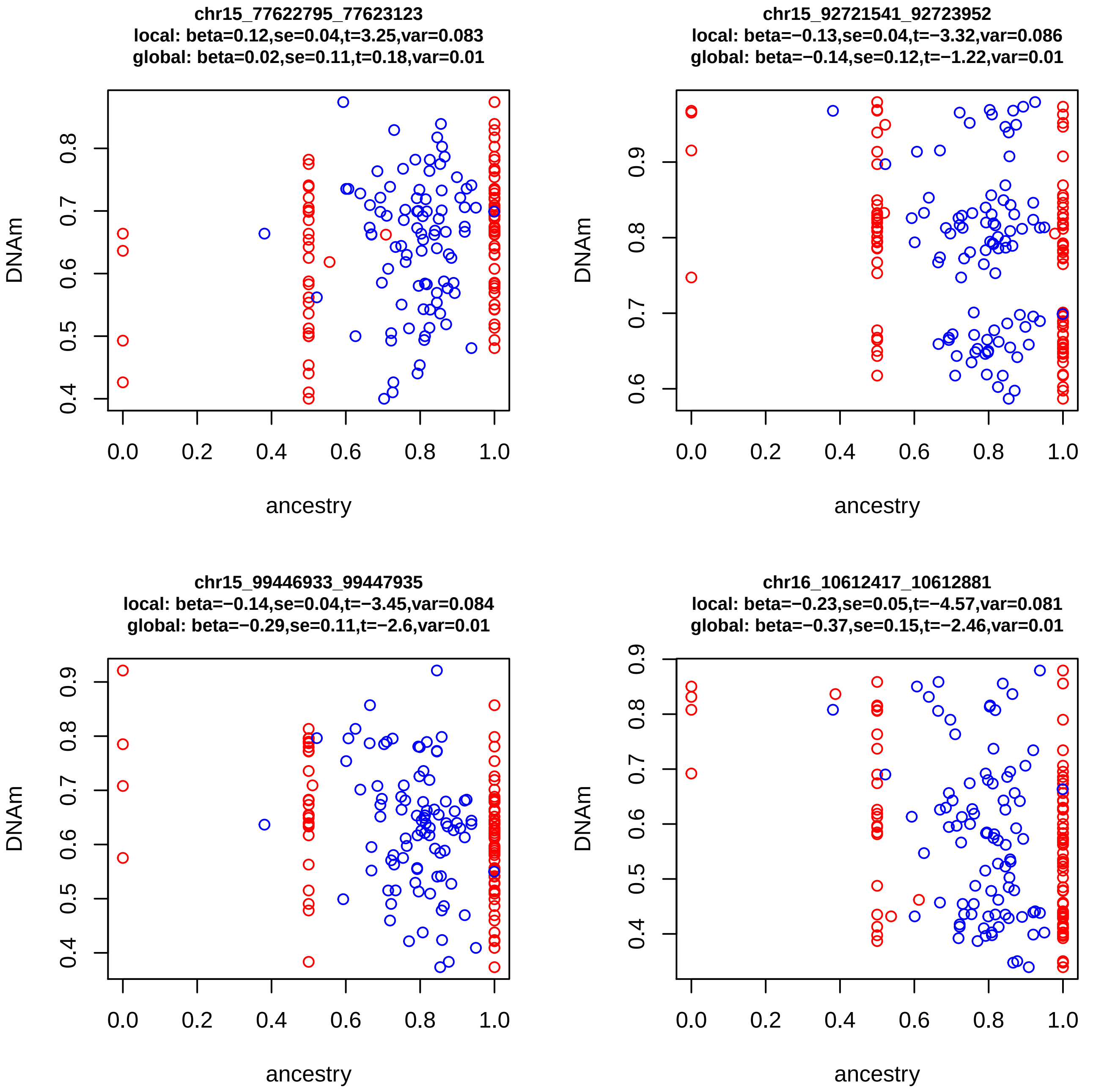
chr15_77622795_77623123
local: beta=0.12,se=0.04,t=3.25,var=0.083
global: beta=0.02,se=0.11,t=0.18,var=0.01
DNAm
0.4
0.5
0.6
0.7
0.8
0.0
0.2
0.4
0.6
0.8
1.0
ancestry
chr15_92721541_92723952
local: beta=−0.13,se=0.04,t=−3.32,var=0.086
global: beta=−0.14,se=0.12,t=−1.22,var=0.01
DNAm
0.6
0.7
0.8
0.9
0.0
0.2
0.4
0.6
0.8
1.0
ancestry
chr15_99446933_99447935
local: beta=−0.14,se=0.04,t=−3.45,var=0.084
global: beta=−0.29,se=0.11,t=−2.6,var=0.01
DNAm
0.4
0.5
0.6
0.7
0.8
0.9
0.0
0.2
0.4
0.6
0.8
1.0
ancestry
chr16_10612417_10612881
local: beta=−0.23,se=0.05,t=−4.57,var=0.081
global: beta=−0.37,se=0.15,t=−2.46,var=0.01
DNAm
0.4
0.5
0.6
0.7
0.8
0.9
0.0
0.2
0.4
0.6
0.8
1.0
ancestry

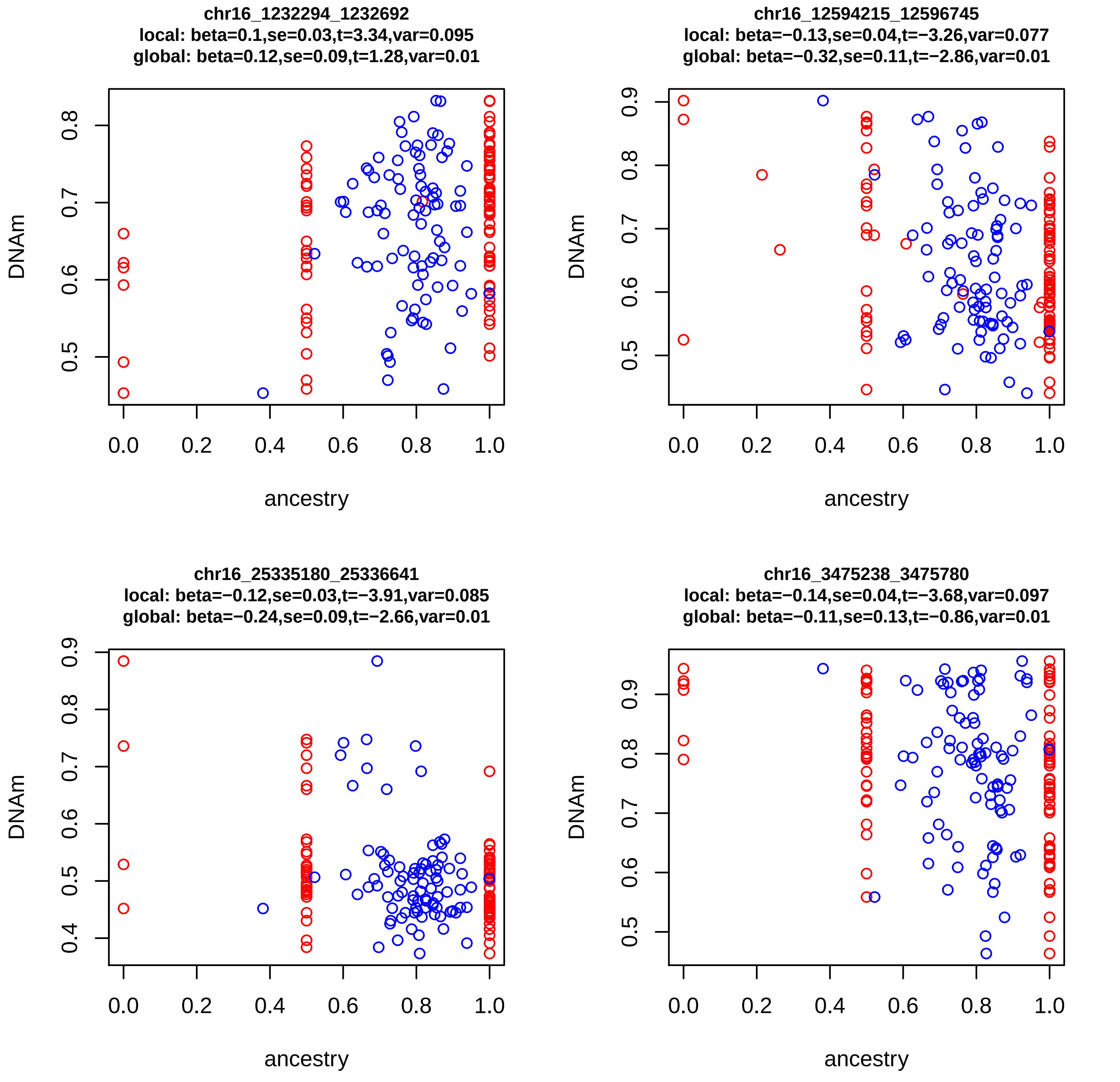
chr16_1232294_1232692
local: beta=0.1,se=0.03,t=3.34,var=0.095
global: beta=0.12,se=0.09,t=1.28,var=0.01
DNAm
ancestry
chr16_12594215_12596745
local: beta=−0.13,se=0.04,t=−3.26,var=0.077
global: beta=−0.32,se=0.11,t=−2.86,var=0.01
DNAm
ancestry
chr16_25335180_25336641
local: beta=−0.12,se=0.03,t=−3.91,var=0.085
global: beta=−0.24,se=0.09,t=−2.66,var=0.01
DNAm
ancestry
chr16_3475238_3475780
local: beta=−0.14,se=0.04,t=−3.68,var=0.097
global: beta=−0.11,se=0.13,t=−0.86,var=0.01
DNAm
ancestry

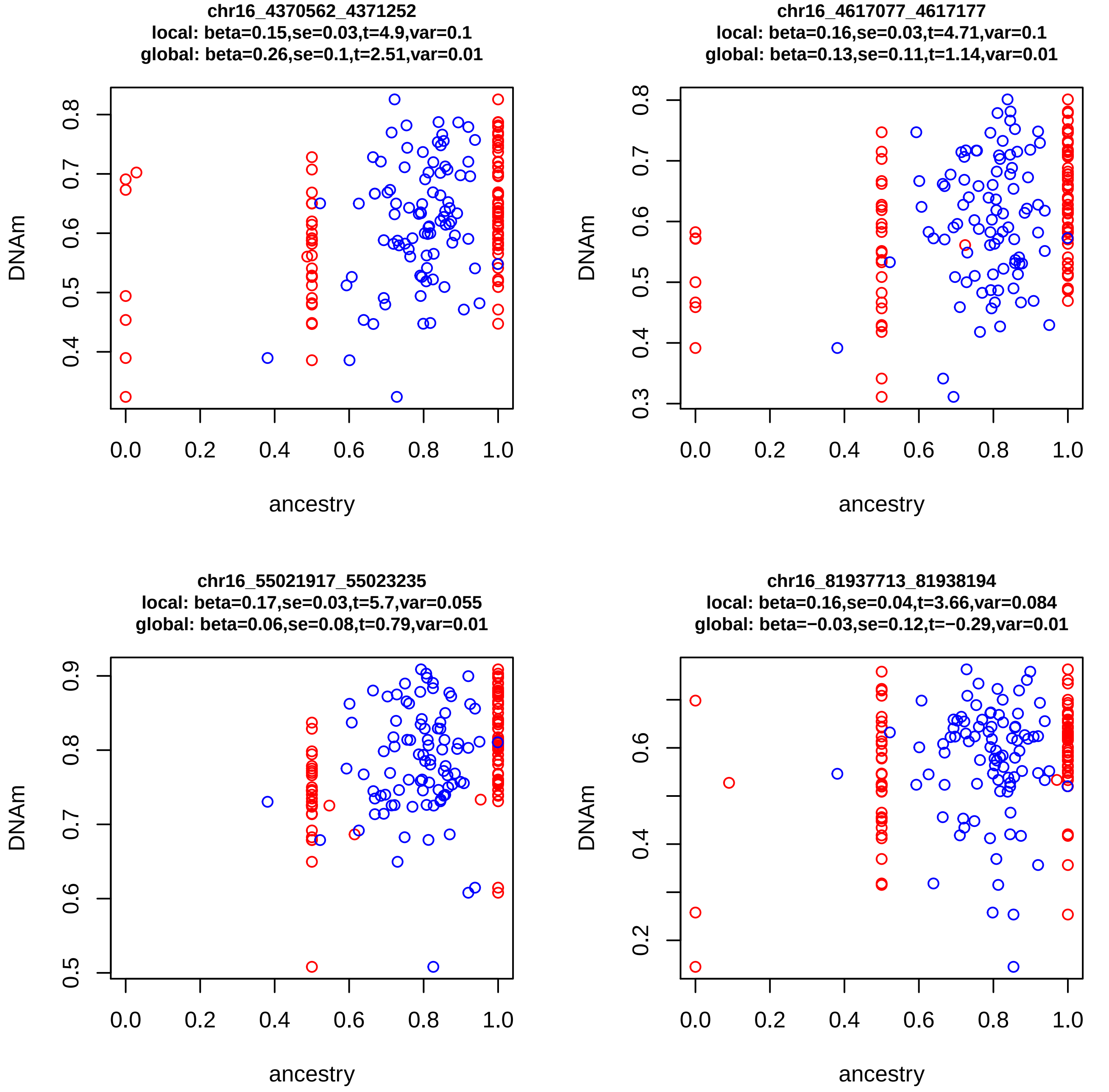
chr16_4370562_4371252
local: beta=0.15,se=0.03,t=4.9,var=0.1
global: beta=0.26,se=0.1,t=2.51,var=0.01
DNAm
0.4
0.5
0.6
0.7
0.8
0.0
0.2
0.4
0.6
0.8
1.0
ancestry
chr16_4617077_4617177
local: beta=0.16,se=0.03,t=4.71,var=0.1
global: beta=0.13,se=0.11,t=1.14,var=0.01
DNAm
0.3
0.4
0.5
0.6
0.7
0.8
0.0
0.2
0.4
0.6
0.8
1.0
ancestry
chr16_55021917_55023235
local: beta=0.17,se=0.03,t=5.7,var=0.055
global: beta=0.06,se=0.08,t=0.79,var=0.01
DNAm
0.5
0.6
0.7
0.8
0.9
0.0
0.2
0.4
0.6
0.8
1.0
ancestry
chr16_81937713_81938194
local: beta=0.16,se=0.04,t=3.66,var=0.084
global: beta=−0.03,se=0.12,t=−0.29,var=0.01
DNAm
0.2
0.4
0.6
0.0
0.2
0.4
0.6
0.8
1.0
ancestry

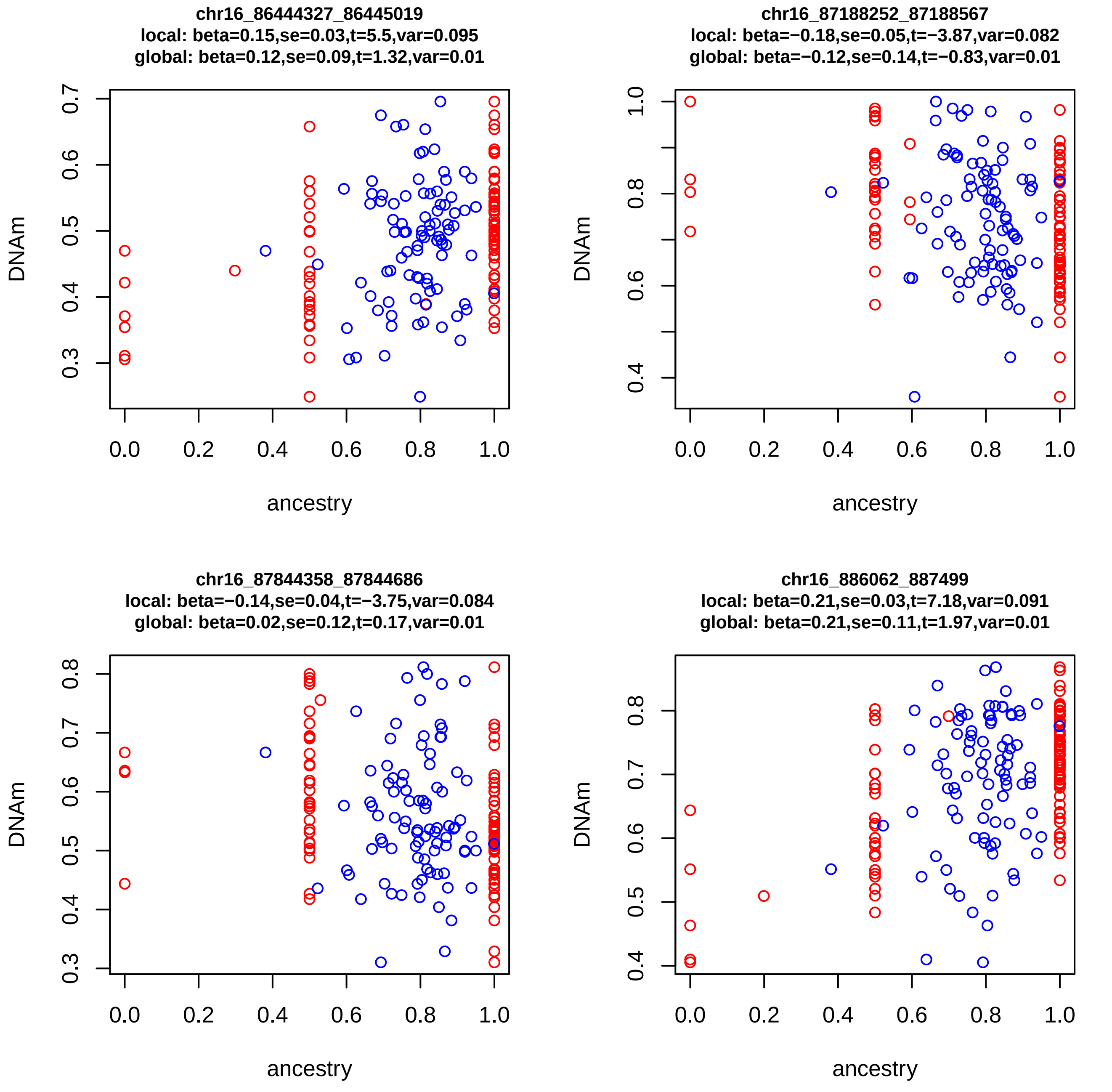
chr16_86444327_86445019
local: beta=0.15,se=0.03,t=5.5,var=0.095
global: beta=0.12,se=0.09,t=1.32,var=0.01
DNAm
ancestry
chr16_87188252_87188567
local: beta=−0.18,se=0.05,t=−3.87,var=0.082
global: beta=−0.12,se=0.14,t=−0.83,var=0.01
DNAm
ancestry
chr16_87844358_87844686
local: beta=−0.14,se=0.04,t=−3.75,var=0.084
global: beta=0.02,se=0.12,t=0.17,var=0.01
DNAm
ancestry
chr16_886062_887499
local: beta=0.21,se=0.03,t=7.18,var=0.091
global: beta=0.21,se=0.11,t=1.97,var=0.01
DNAm
ancestry

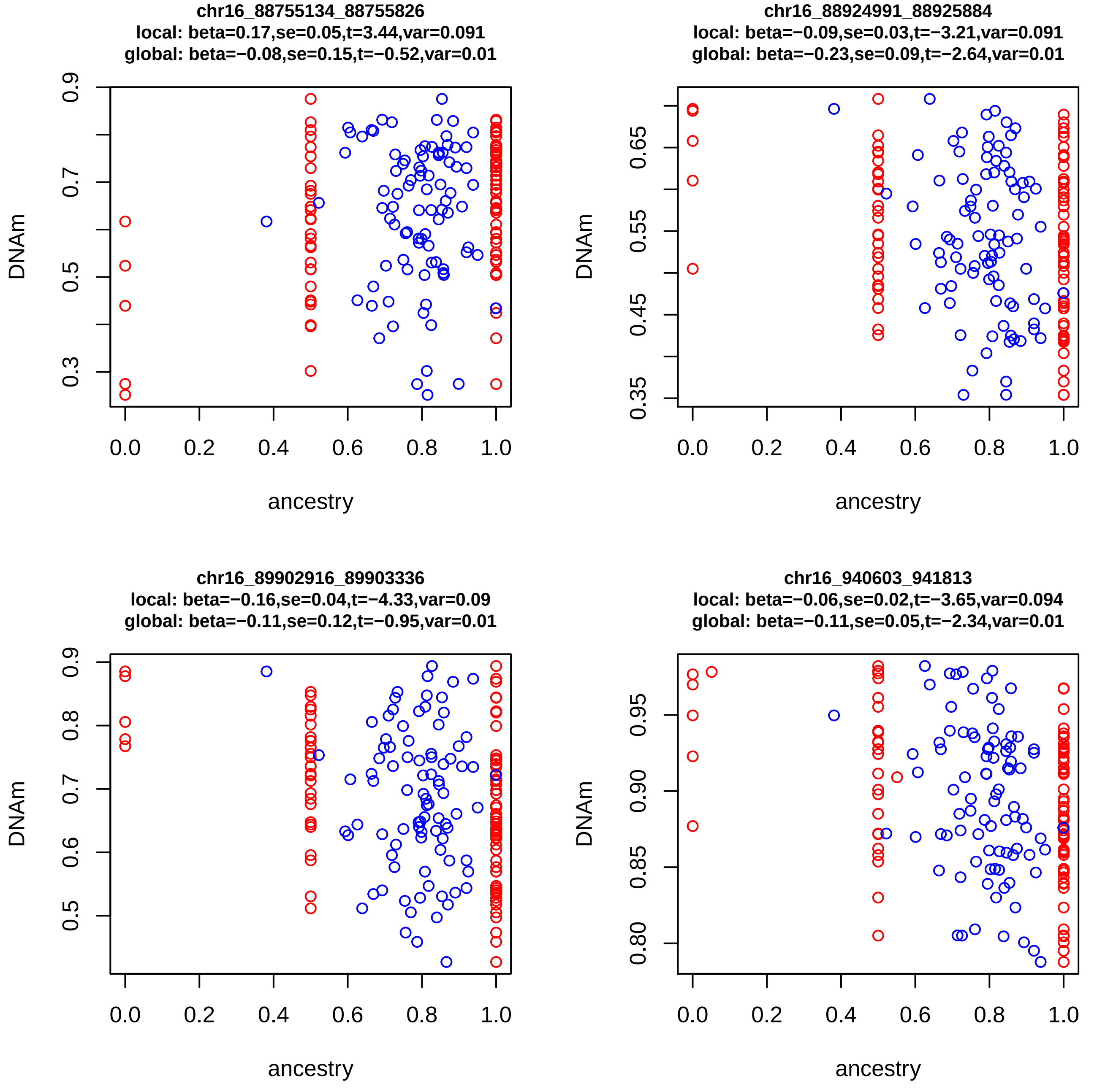

chr16_88755134_88755826
local: beta=0.17,se=0.05,t=3.44,var=0.091
global: beta=−0.08,se=0.15,t=−0.52,var=0.01
DNAm
ancestry
chr16_88924991_88925884
local: beta=−0.09,se=0.03,t=−3.21,var=0.091
global: beta=−0.23,se=0.09,t=−2.64,var=0.01
DNAm
ancestry
chr16_89902916_89903336
local: beta=−0.16,se=0.04,t=−4.33,var=0.09
global: beta=−0.11,se=0.12,t=−0.95,var=0.01
DNAm
ancestry
chr16_940603_941813
local: beta=−0.06,se=0.02,t=−3.65,var=0.094
global: beta=−0.11,se=0.05,t=−2.34,var=0.01
DNAm
ancestry

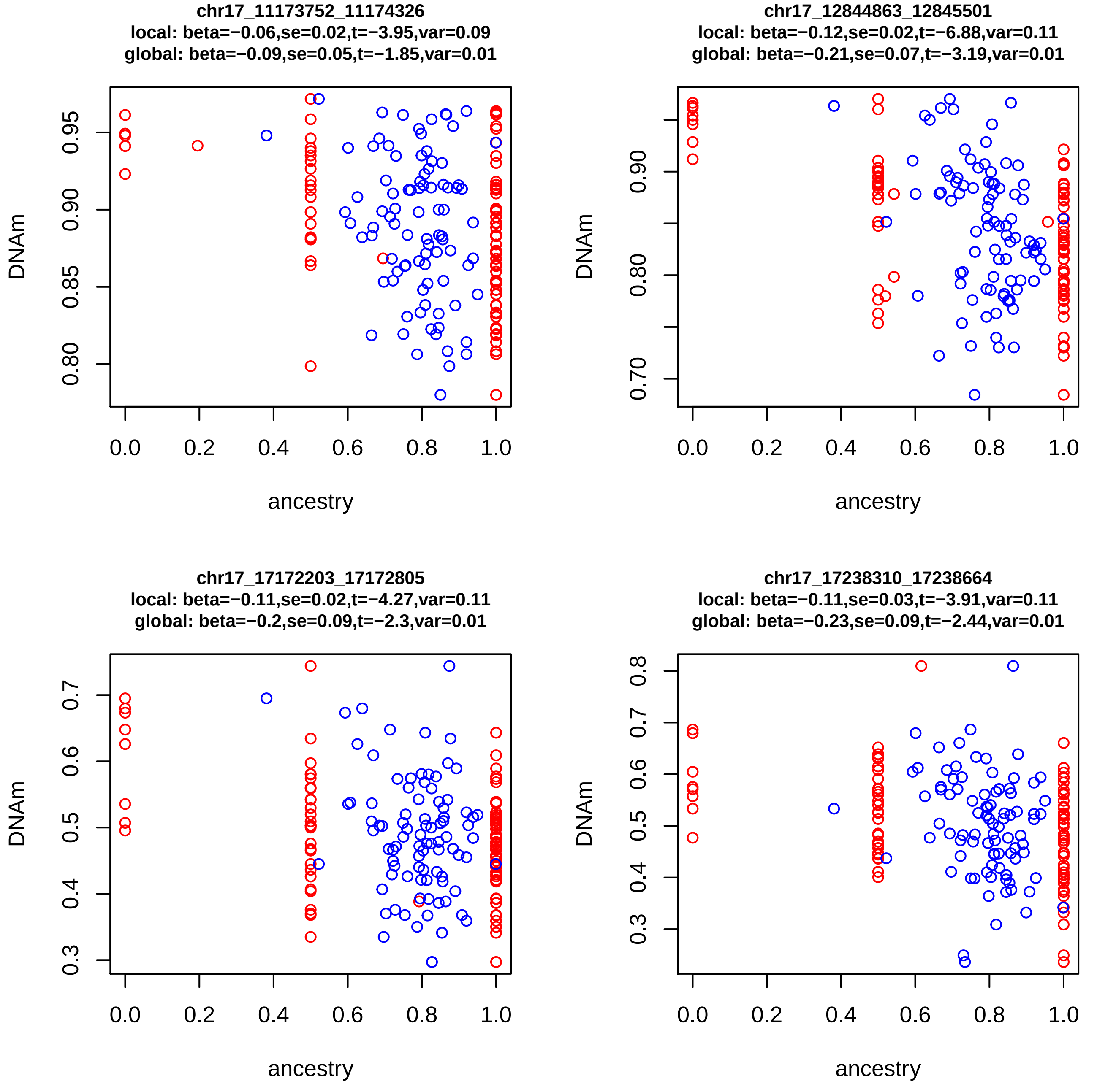

chr17_11173752_11174326
local: beta=−0.06,se=0.02,t=−3.95,var=0.09
global: beta=−0.09,se=0.05,t=−1.85,var=0.01
DNAm
ancestry
chr17_12844863_12845501
local: beta=−0.12,se=0.02,t=−6.88,var=0.11
global: beta=−0.21,se=0.07,t=−3.19,var=0.01
DNAm
ancestry
chr17_17172203_17172805
local: beta=−0.11,se=0.02,t=−4.27,var=0.11
global: beta=−0.2,se=0.09,t=−2.3,var=0.01
DNAm
ancestry
chr17_17238310_17238664
local: beta=−0.11,se=0.03,t=−3.91,var=0.11
global: beta=−0.23,se=0.09,t=−2.44,var=0.01
DNAm
ancestry

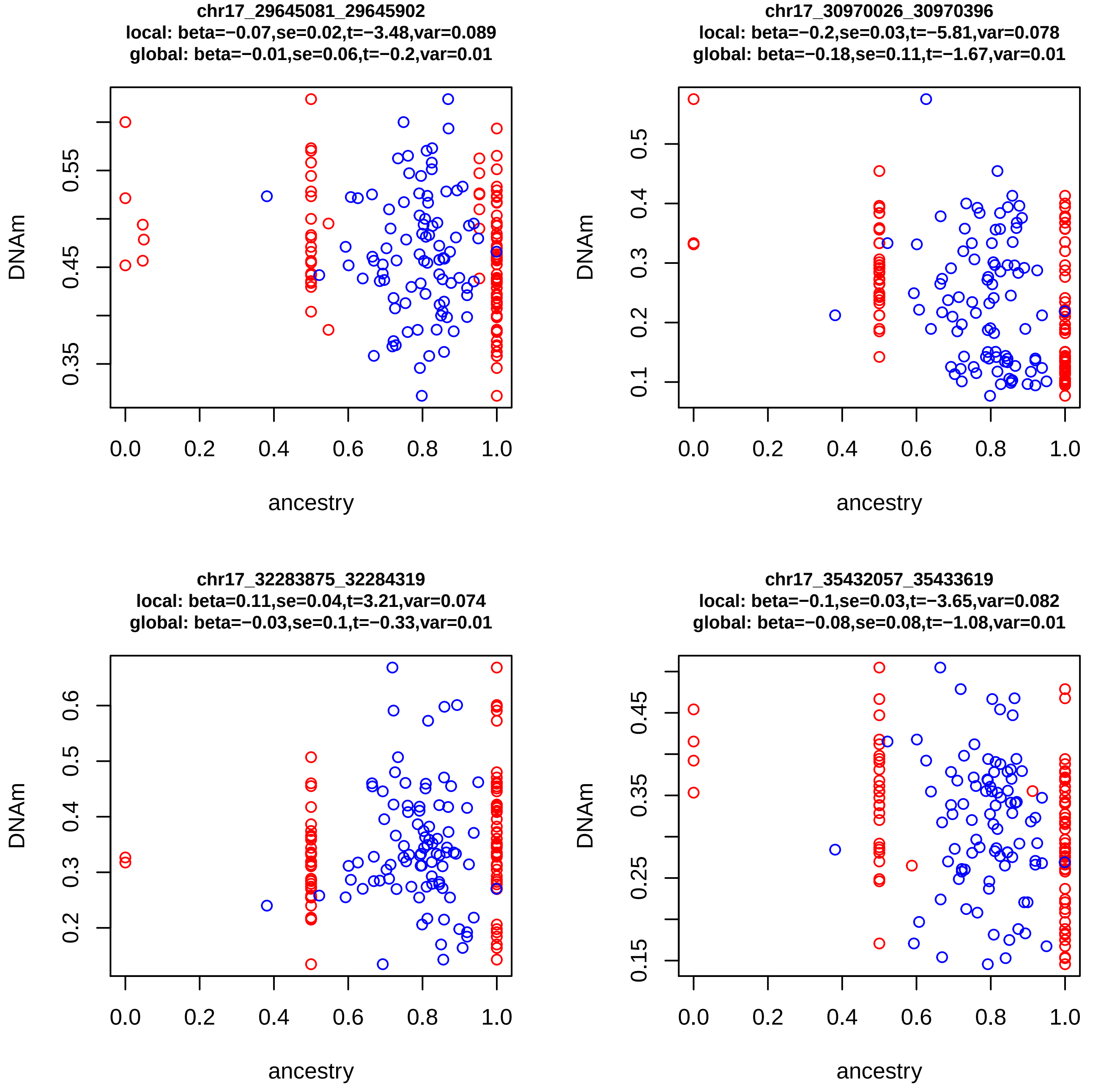
chr17_29645081_29645902
local: beta=−0.07,se=0.02,t=−3.48,var=0.089
global: beta=−0.01,se=0.06,t=−0.2,var=0.01
DNAm
0.35
0.45
0.55
ancestry
0.0
0.2
0.4
0.6
0.8
1.0
chr17_30970026_30970396
local: beta=−0.2,se=0.03,t=−5.81,var=0.078
global: beta=−0.18,se=0.11,t=−1.67,var=0.01
DNAm
0.1
0.2
0.3
0.4
0.5
ancestry
chr17_32283875_32284319
local: beta=0.11,se=0.04,t=3.21,var=0.074
global: beta=−0.03,se=0.1,t=−0.33,var=0.01
DNAm
0.2
0.3
0.4
0.5
0.6
ancestry
chr17_35432057_35433619
local: beta=−0.1,se=0.03,t=−3.65,var=0.082
global: beta=−0.08,se=0.08,t=−1.08,var=0.01
DNAm
0.15
0.25
0.35
0.45
ancestry

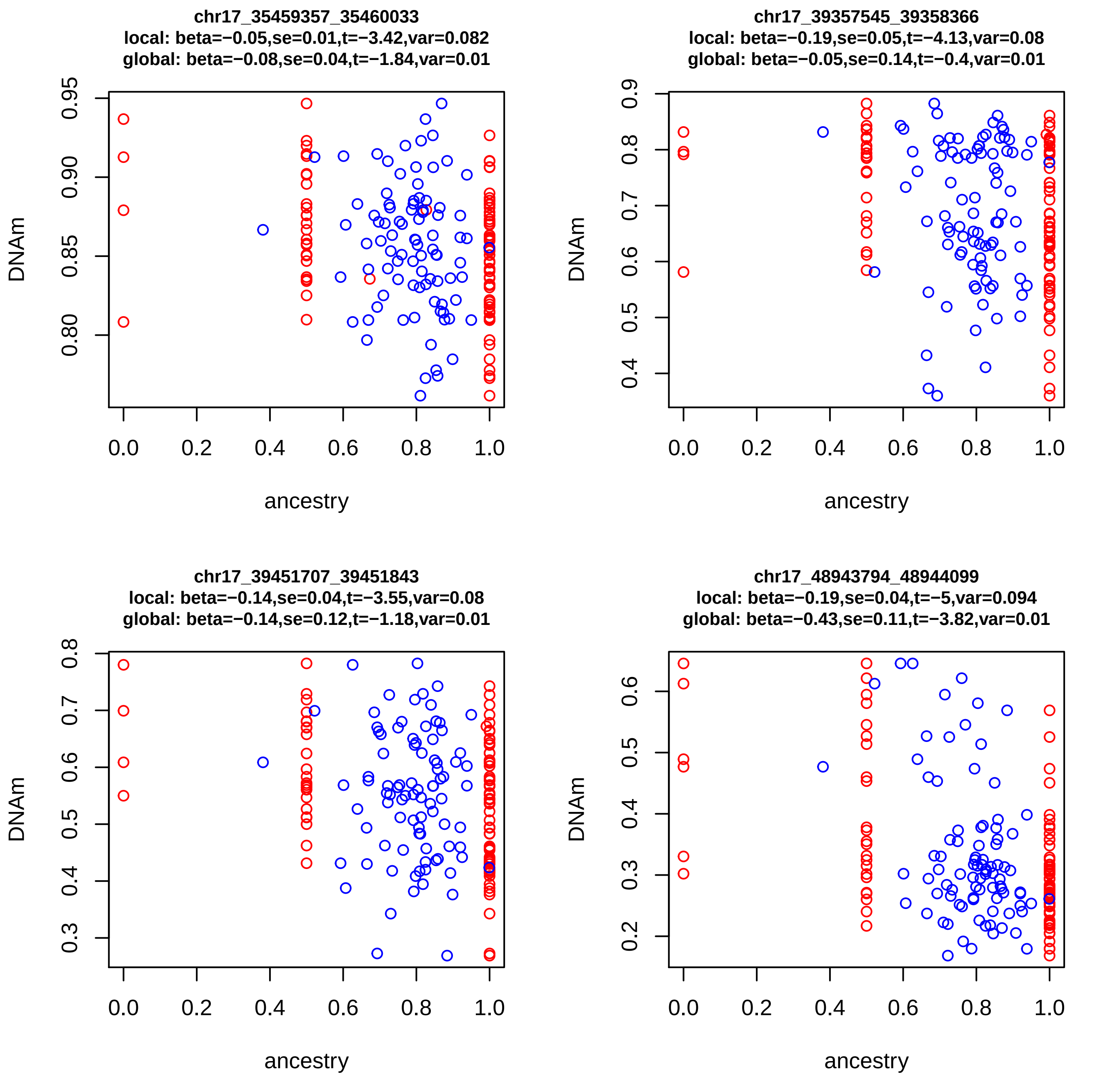
chr17_35459357_35460033
local: beta=−0.05,se=0.01,t=−3.42,var=0.082
global: beta=−0.08,se=0.04,t=−1.84,var=0.01
DNAm
0.80
0.85
0.90
0.95
0.0
0.2
0.4
0.6
0.8
1.0
ancestry
chr17_39357545_39358366
local: beta=−0.19,se=0.05,t=−4.13,var=0.08
global: beta=−0.05,se=0.14,t=−0.4,var=0.01
DNAm
0.4
0.5
0.6
0.7
0.8
0.9
0.0
0.2
0.4
0.6
0.8
1.0
ancestry
chr17_39451707_39451843
local: beta=−0.14,se=0.04,t=−3.55,var=0.08
global: beta=−0.14,se=0.12,t=−1.18,var=0.01
DNAm
0.3
0.4
0.5
0.6
0.7
0.8
0.0
0.2
0.4
0.6
0.8
1.0
ancestry
chr17_48943794_48944099
local: beta=−0.19,se=0.04,t=−5,var=0.094
global: beta=−0.43,se=0.11,t=−3.82,var=0.01
DNAm
0.2
0.3
0.4
0.5
0.6
0.0
0.2
0.4
0.6
0.8
1.0
ancestry

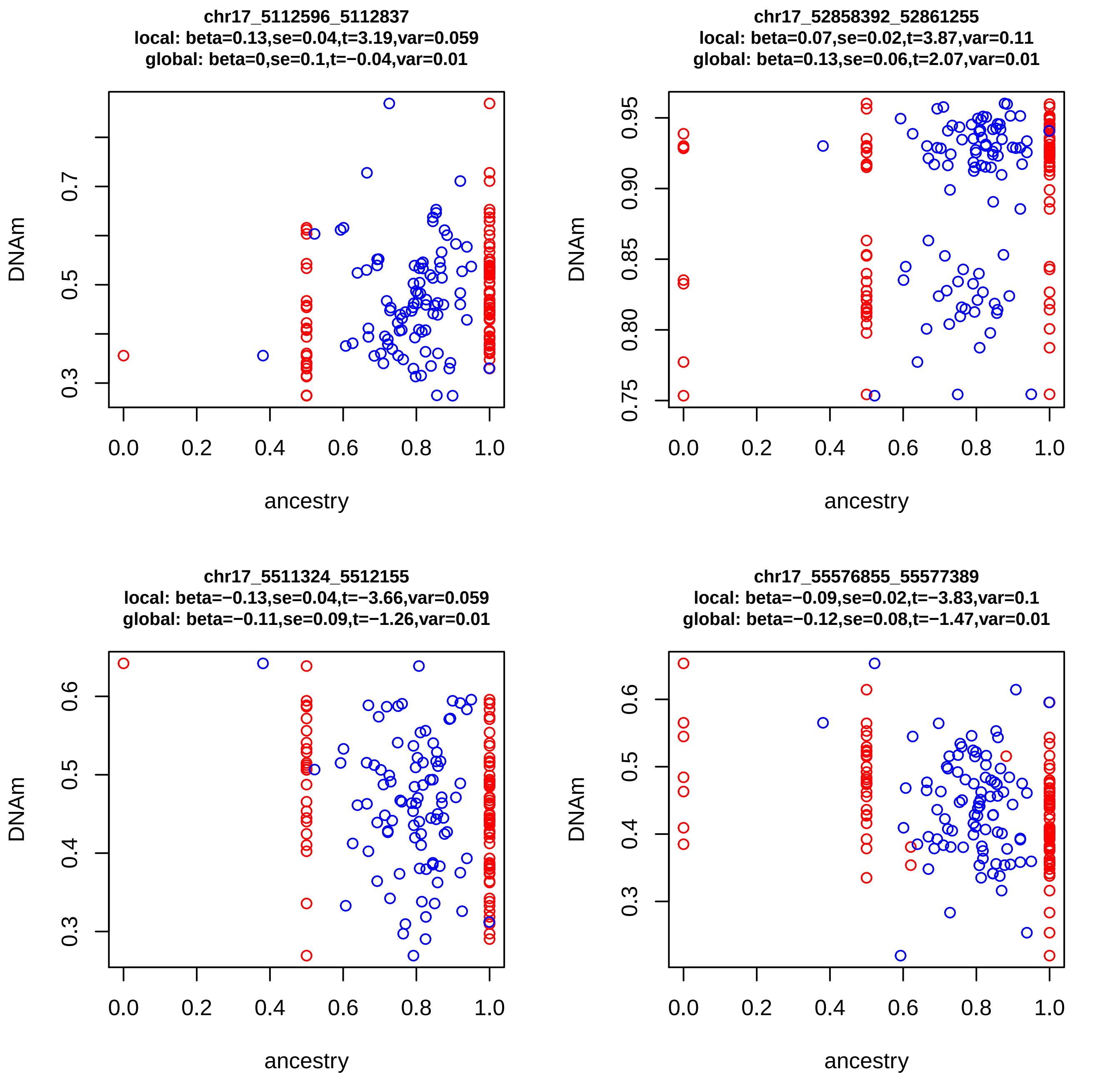
chr17_5112596_5112837
local: beta=0.13,se=0.04,t=3.19,var=0.059
global: beta=0,se=0.1,t=−0.04,var=0.01
DNAm
0.3
0.5
0.7
0.0
0.2
0.4
0.6
0.8
1.0
ancestry
chr17_52858392_52861255
local: beta=0.07,se=0.02,t=3.87,var=0.11
global: beta=0.13,se=0.06,t=2.07,var=0.01
DNAm
0.75
0.80
0.85
0.90
0.95
0.0
0.2
0.4
0.6
0.8
1.0
ancestry
chr17_5511324_5512155
local: beta=−0.13,se=0.04,t=−3.66,var=0.059
global: beta=−0.11,se=0.09,t=−1.26,var=0.01
DNAm
0.3
0.4
0.5
0.6
0.0
0.2
0.4
0.6
0.8
1.0
ancestry
chr17_55576855_55577389
local: beta=−0.09,se=0.02,t=−3.83,var=0.1
global: beta=−0.12,se=0.08,t=−1.47,var=0.01
DNAm
0.3
0.4
0.5
0.6
0.0
0.2
0.4
0.6
0.8
1.0
ancestry

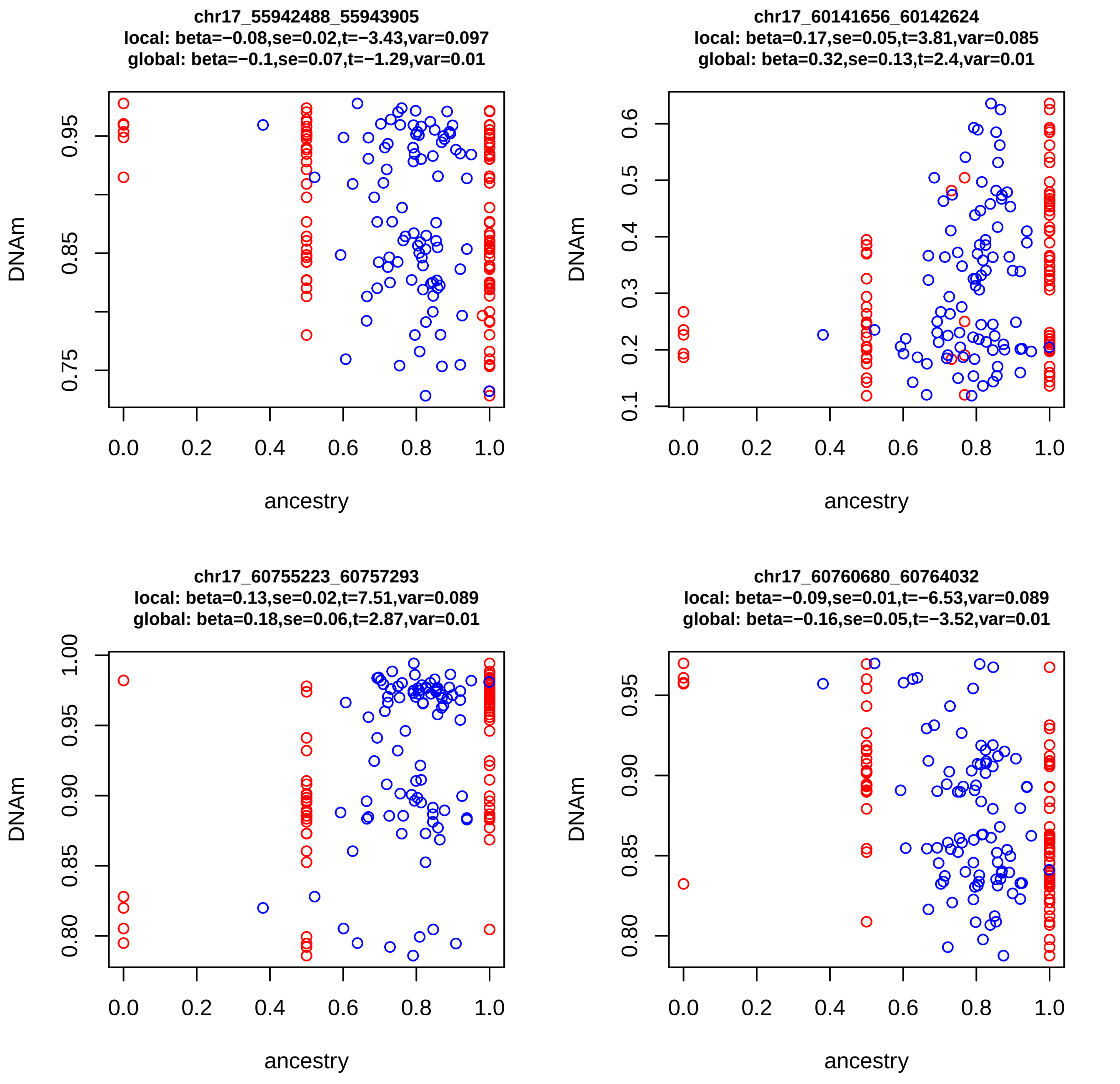
chr17_55942488_55943905
local: beta=−0.08,se=0.02,t=−3.43,var=0.097
global: beta=−0.1,se=0.07,t=−1.29,var=0.01
DNAm
0.75
0.85
0.95
ancestry
0.0
0.2
0.4
0.6
0.8
1.0
chr17_60141656_60142624
local: beta=0.17,se=0.05,t=3.81,var=0.085
global: beta=0.32,se=0.13,t=2.4,var=0.01
DNAm
0.1
0.2
0.3
0.4
0.5
0.6
ancestry
chr17_60755223_60757293
local: beta=0.13,se=0.02,t=7.51,var=0.089
global: beta=0.18,se=0.06,t=2.87,var=0.01
DNAm
0.80
0.85
0.90
0.95
1.00
ancestry
chr17_60760680_60764032
local: beta=−0.09,se=0.01,t=−6.53,var=0.089
global: beta=−0.16,se=0.05,t=−3.52,var=0.01
DNAm
0.80
0.85
0.90
0.95
ancestry

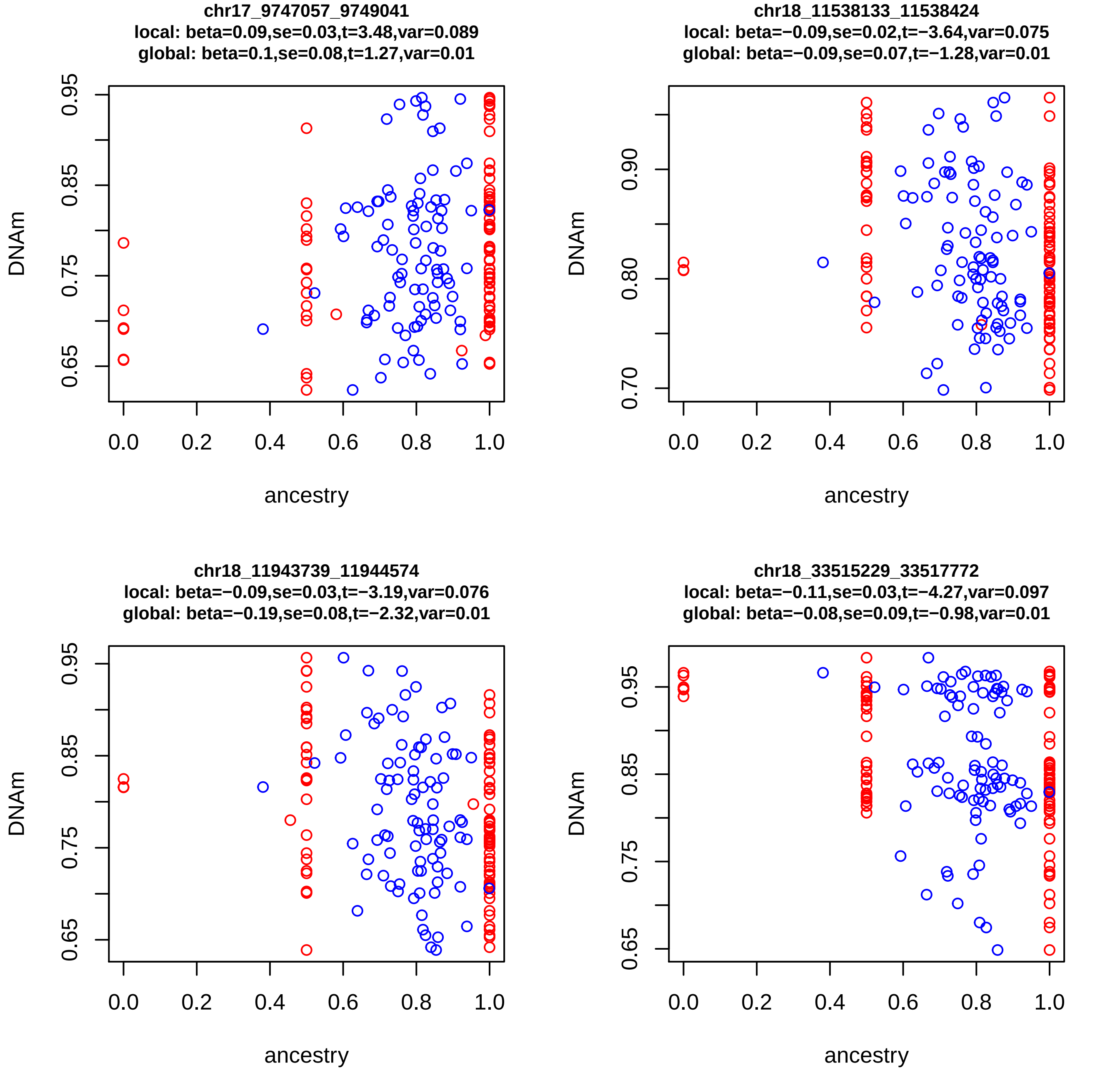
chr17_9747057_9749041
local: beta=0.09,se=0.03,t=3.48,var=0.089
global: beta=0.1,se=0.08,t=1.27,var=0.01
chr18_11538133_11538424
local: beta=−0.09,se=0.02,t=−3.64,var=0.075
global: beta=−0.09,se=0.07,t=−1.28,var=0.01
chr18_11943739_11944574
local: beta=−0.09,se=0.03,t=−3.19,var=0.076
global: beta=−0.19,se=0.08,t=−2.32,var=0.01
chr18_33515229_33517772
local: beta=−0.11,se=0.03,t=−4.27,var=0.097
global: beta=−0.08,se=0.09,t=−0.98,var=0.01
DNAm
ancestry
0.0
0.2
0.4
0.6
0.8
1.0
0.65
0.75
0.85
0.95
0.70
0.80
0.90

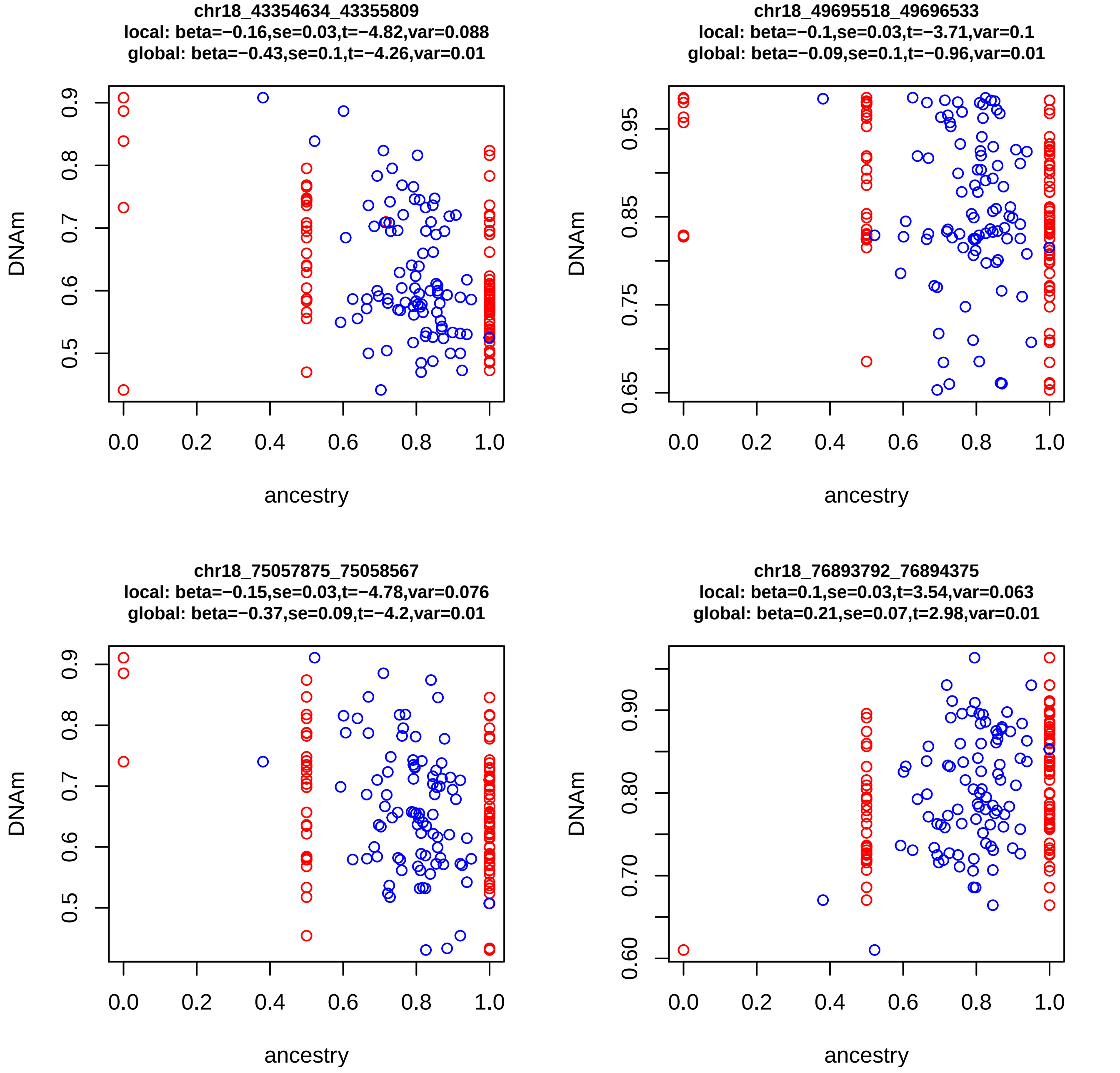

chr18_43354634_43355809
local: beta=−0.16,se=0.03,t=−4.82,var=0.088
global: beta=−0.43,se=0.1,t=−4.26,var=0.01
DNAm
ancestry
chr18_49695518_49696533
local: beta=−0.1,se=0.03,t=−3.71,var=0.1
global: beta=−0.09,se=0.1,t=−0.96,var=0.01
DNAm
ancestry
chr18_75057875_75058567
local: beta=−0.15,se=0.03,t=−4.78,var=0.076
global: beta=−0.37,se=0.09,t=−4.2,var=0.01
DNAm
ancestry
chr18_76893792_76894375
local: beta=0.1,se=0.03,t=3.54,var=0.063
global: beta=0.21,se=0.07,t=2.98,var=0.01
DNAm
ancestry

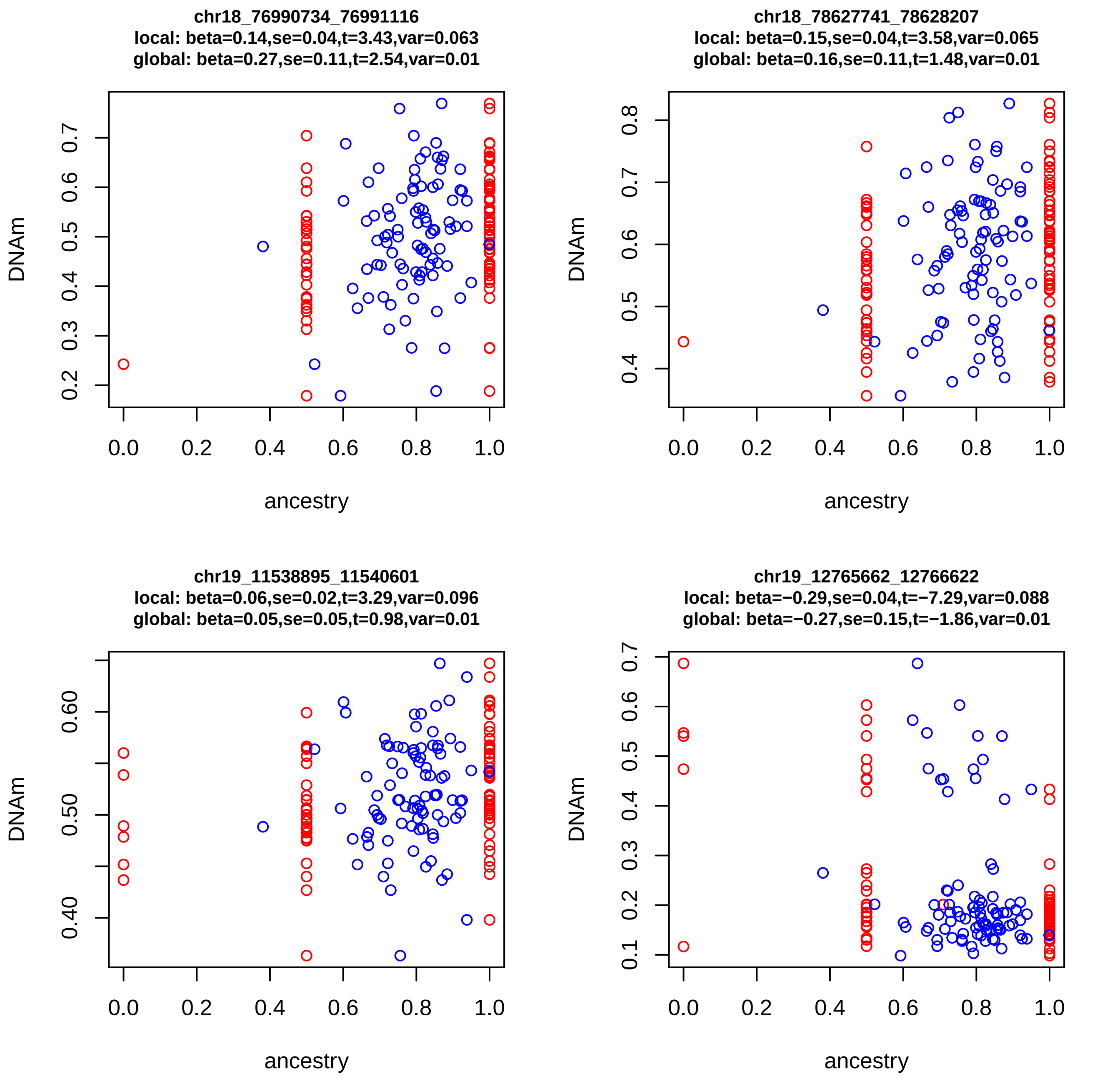
chr18_76990734_76991116
local: beta=0.14,se=0.04,t=3.43,var=0.063
global: beta=0.27,se=0.11,t=2.54,var=0.01
chr18_78627741_78628207
local: beta=0.15,se=0.04,t=3.58,var=0.065
global: beta=0.16,se=0.11,t=1.48,var=0.01
chr19_11538895_11540601
local: beta=0.06,se=0.02,t=3.29,var=0.096
global: beta=0.05,se=0.05,t=0.98,var=0.01
chr19_12765662_12766622
local: beta=−0.29,se=0.04,t=−7.29,var=0.088
global: beta=−0.27,se=0.15,t=−1.86,var=0.01
DNAm
ancestry

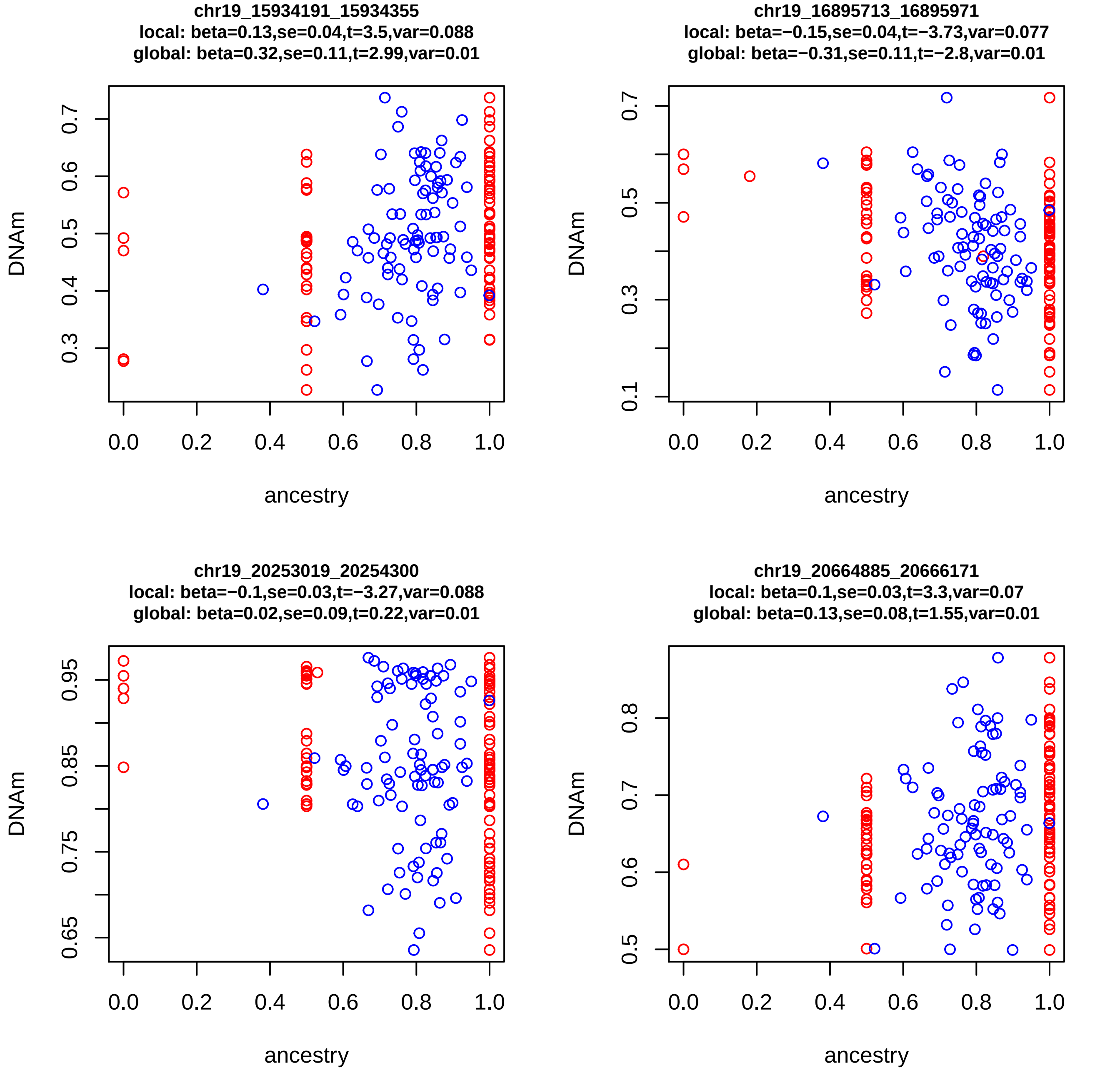
chr19_15934191_15934355
local: beta=0.13,se=0.04,t=3.5,var=0.088
global: beta=0.32,se=0.11,t=2.99,var=0.01
DNAm
ancestry
0.3
0.4
0.5
0.6
0.7
0.0
0.2
0.4
0.6
0.8
1.0
chr19_16895713_16895971
local: beta=−0.15,se=0.04,t=−3.73,var=0.077
global: beta=−0.31,se=0.11,t=−2.8,var=0.01
DNAm
ancestry
0.1
0.3
0.5
0.7
chr19_20253019_20254300
local: beta=−0.1,se=0.03,t=−3.27,var=0.088
global: beta=0.02,se=0.09,t=0.22,var=0.01
DNAm
ancestry
0.65
0.75
0.85
0.95
chr19_20664885_20666171
local: beta=0.1,se=0.03,t=3.3,var=0.07
global: beta=0.13,se=0.08,t=1.55,var=0.01
DNAm
ancestry
0.5
0.6
0.7
0.8

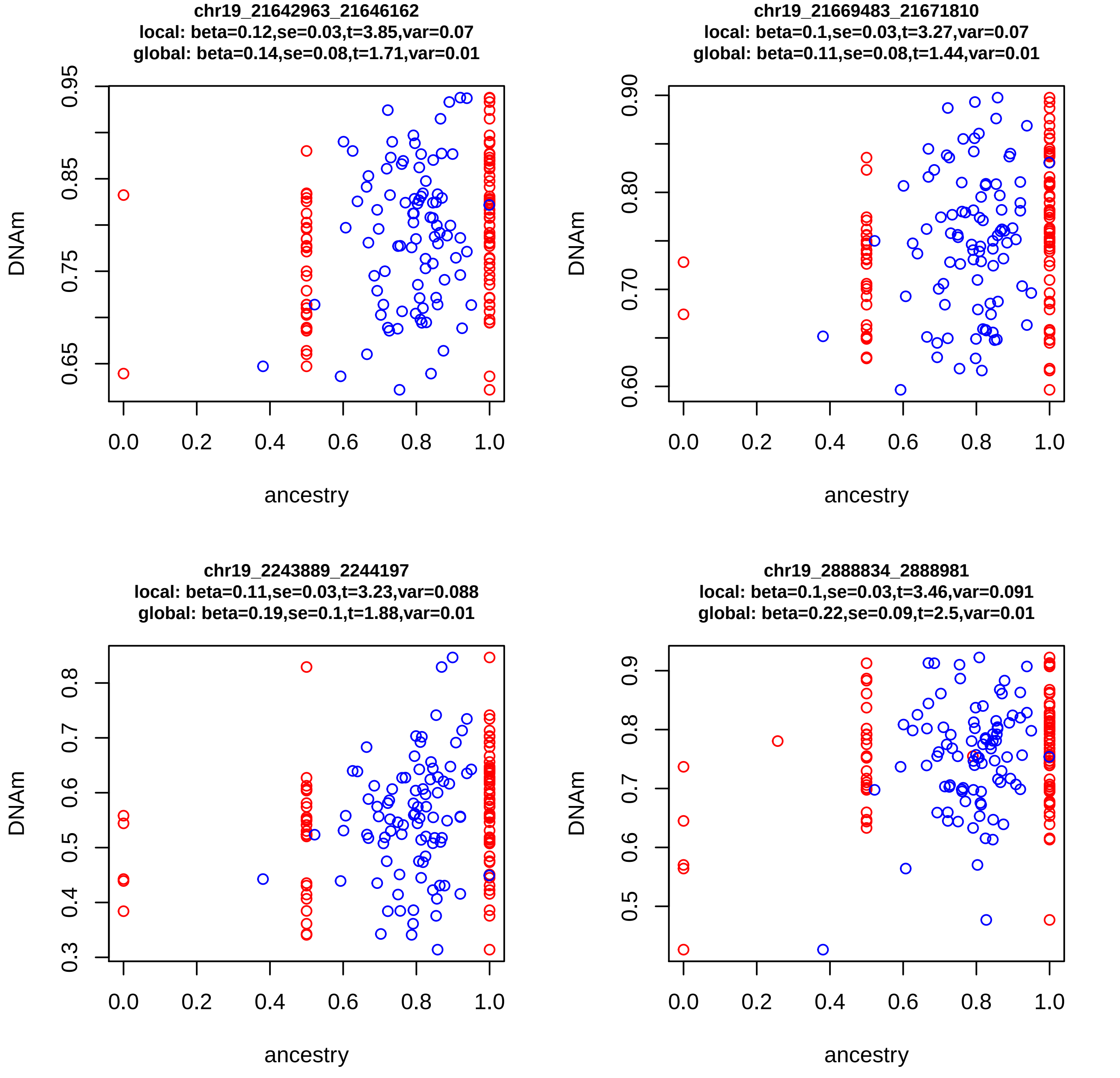
chr19_21642963_21646162
local: beta=0.12,se=0.03,t=3.85,var=0.07
global: beta=0.14,se=0.08,t=1.71,var=0.01
DNAm
0.65
0.75
0.85
0.95
0.0
0.2
0.4
0.6
0.8
1.0
ancestry
chr19_21669483_21671810
local: beta=0.1,se=0.03,t=3.27,var=0.07
global: beta=0.11,se=0.08,t=1.44,var=0.01
DNAm
0.60
0.70
0.80
0.90
0.0
0.2
0.4
0.6
0.8
1.0
ancestry
chr19_2243889_2244197
local: beta=0.11,se=0.03,t=3.23,var=0.088
global: beta=0.19,se=0.1,t=1.88,var=0.01
DNAm
0.3
0.4
0.5
0.6
0.7
0.8
0.0
0.2
0.4
0.6
0.8
1.0
ancestry
chr19_2888834_2888981
local: beta=0.1,se=0.03,t=3.46,var=0.091
global: beta=0.22,se=0.09,t=2.5,var=0.01
DNAm
0.5
0.6
0.7
0.8
0.9
0.0
0.2
0.4
0.6
0.8
1.0
ancestry

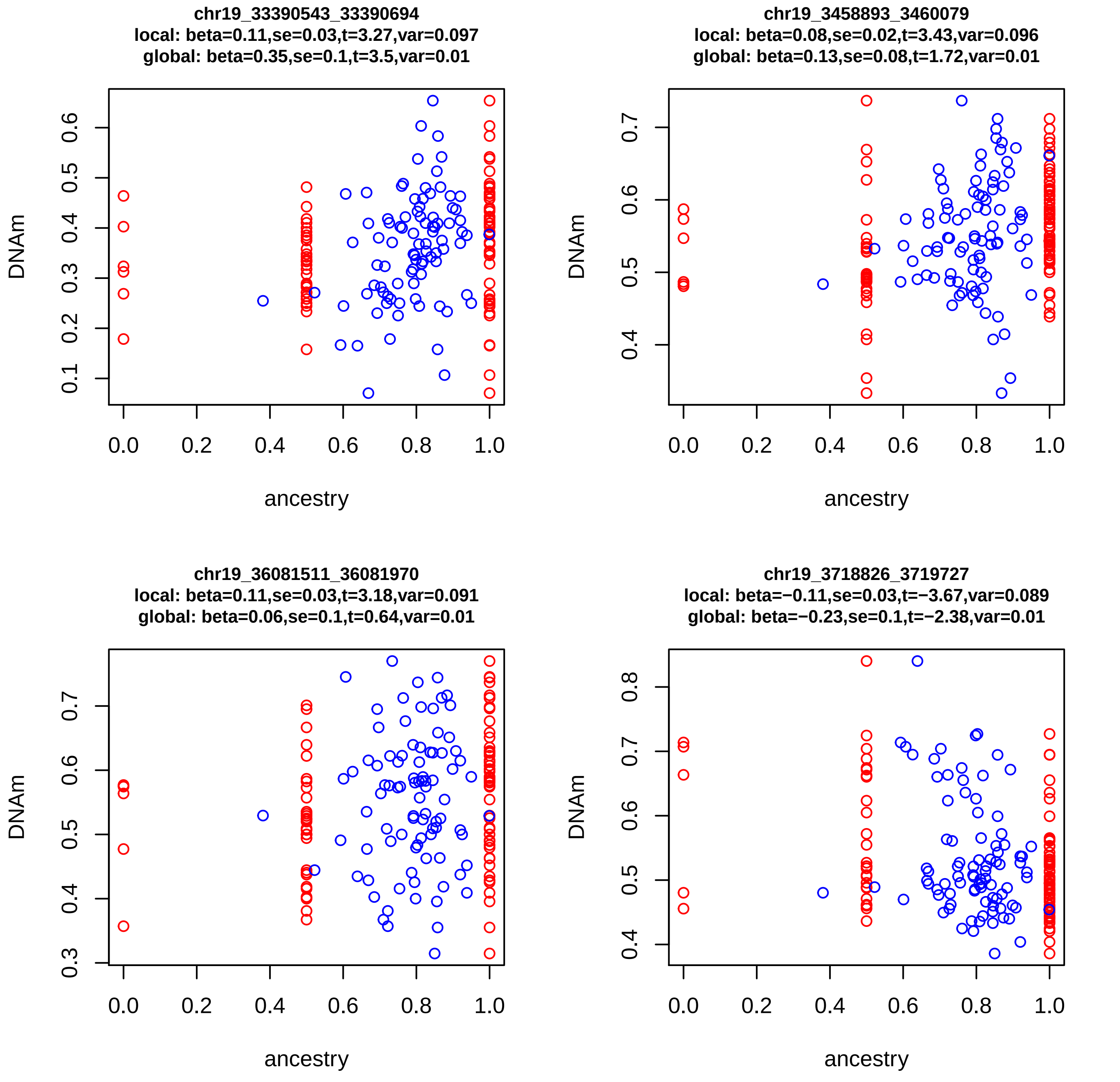
chr19_33390543_33390694
local: beta=0.11,se=0.03,t=3.27,var=0.097
global: beta=0.35,se=0.1,t=3.5,var=0.01
DNAm
ancestry
chr19_3458893_3460079
local: beta=0.08,se=0.02,t=3.43,var=0.096
global: beta=0.13,se=0.08,t=1.72,var=0.01
DNAm
ancestry
chr19_36081511_36081970
local: beta=0.11,se=0.03,t=3.18,var=0.091
global: beta=0.06,se=0.1,t=0.64,var=0.01
DNAm
ancestry
chr19_3718826_3719727
local: beta=−0.11,se=0.03,t=−3.67,var=0.089
global: beta=−0.23,se=0.1,t=−2.38,var=0.01
DNAm
ancestry

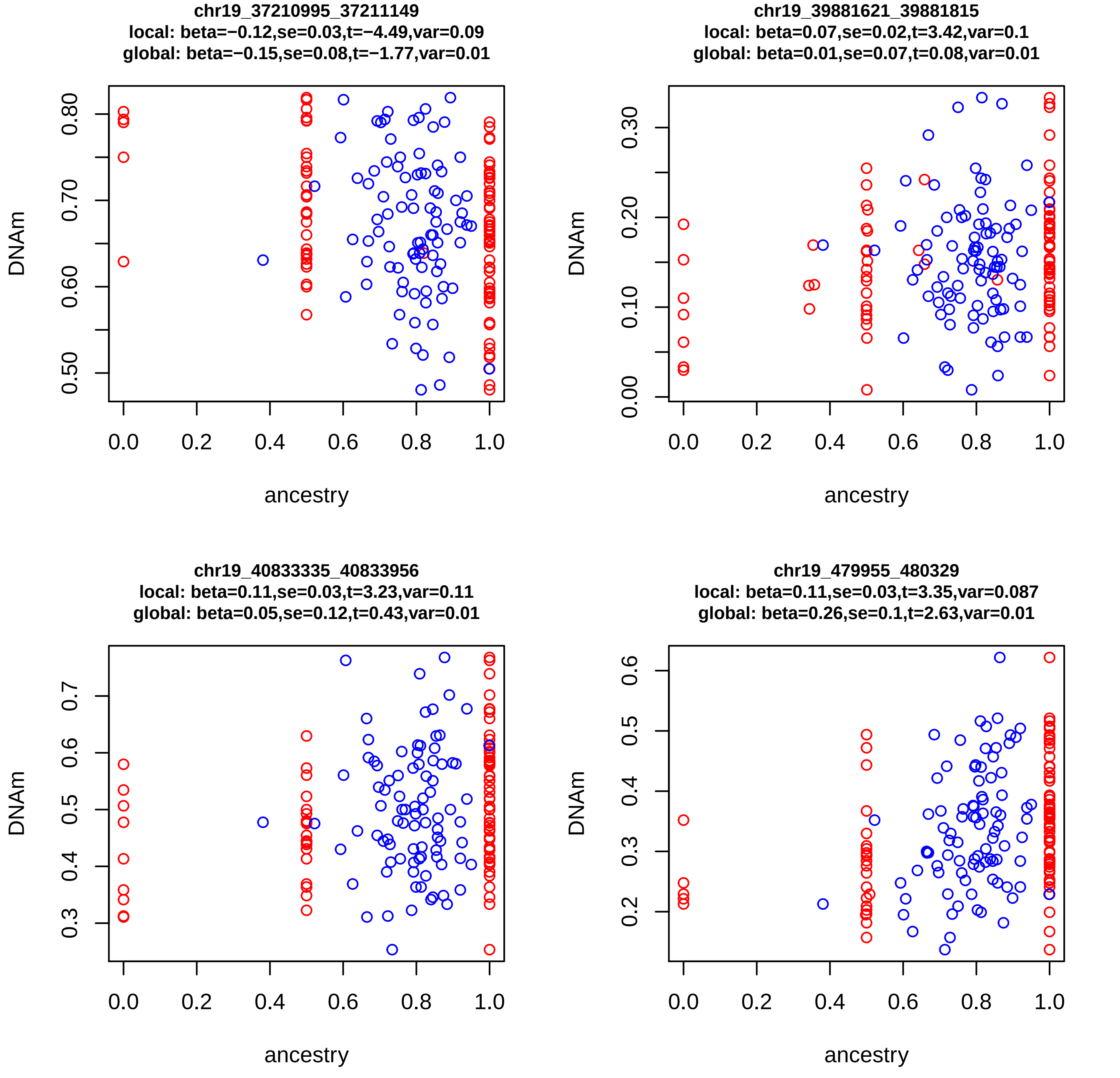
chr19_37210995_37211149
local: beta=−0.12,se=0.03,t=−4.49,var=0.09
global: beta=−0.15,se=0.08,t=−1.77,var=0.01
chr19_39881621_39881815
local: beta=0.07,se=0.02,t=3.42,var=0.1
global: beta=0.01,se=0.07,t=0.08,var=0.01
chr19_40833335_40833956
local: beta=0.11,se=0.03,t=3.23,var=0.11
global: beta=0.05,se=0.12,t=0.43,var=0.01
chr19_479955_480329
local: beta=0.11,se=0.03,t=3.35,var=0.087
global: beta=0.26,se=0.1,t=2.63,var=0.01
DNAm
ancestry

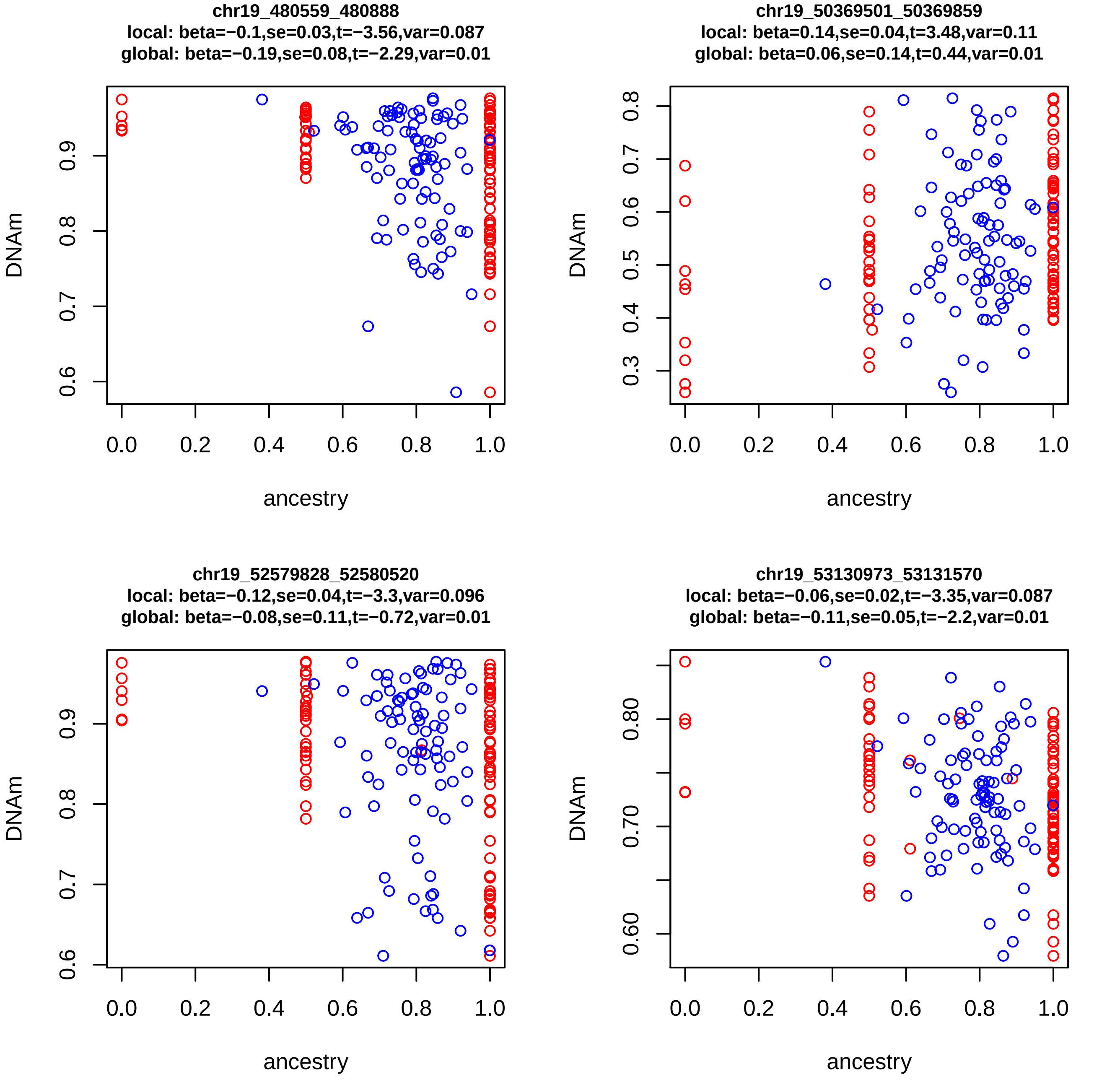
chr19_480559_480888
local: beta=−0.1,se=0.03,t=−3.56,var=0.087
global: beta=−0.19,se=0.08,t=−2.29,var=0.01
DNAm
0.6
0.7
0.8
0.9
0.0
0.2
0.4
0.6
0.8
1.0
ancestry
chr19_50369501_50369859
local: beta=0.14,se=0.04,t=3.48,var=0.11
global: beta=0.06,se=0.14,t=0.44,var=0.01
DNAm
0.3
0.4
0.5
0.6
0.7
0.8
0.0
0.2
0.4
0.6
0.8
1.0
ancestry
chr19_52579828_52580520
local: beta=−0.12,se=0.04,t=−3.3,var=0.096
global: beta=−0.08,se=0.11,t=−0.72,var=0.01
DNAm
0.6
0.7
0.8
0.9
0.0
0.2
0.4
0.6
0.8
1.0
ancestry
chr19_53130973_53131570
local: beta=−0.06,se=0.02,t=−3.35,var=0.087
global: beta=−0.11,se=0.05,t=−2.2,var=0.01
DNAm
0.60
0.70
0.80
0.0
0.2
0.4
0.6
0.8
1.0
ancestry

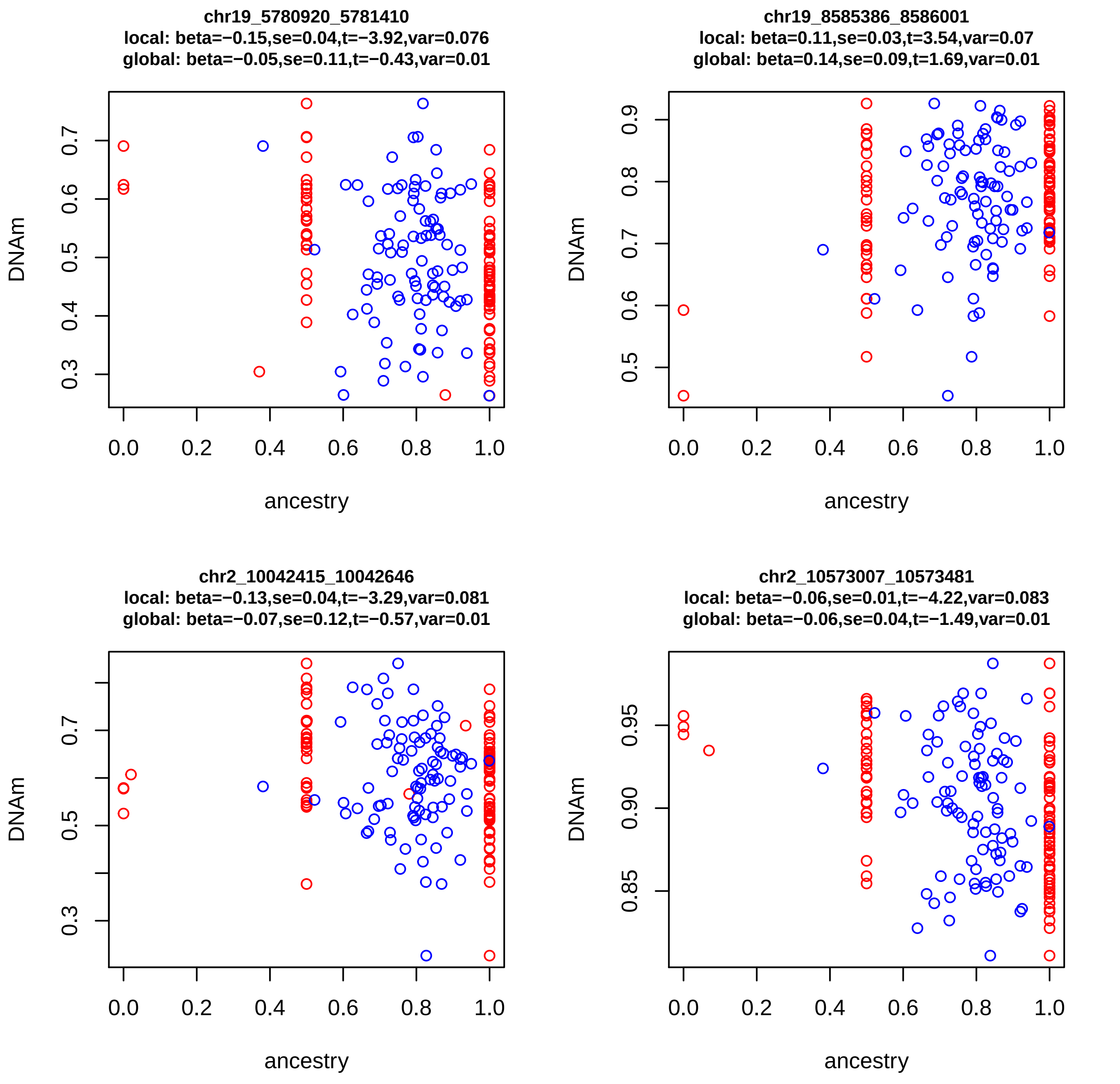

chr19_5780920_5781410
local: beta=−0.15,se=0.04,t=−3.92,var=0.076
global: beta=−0.05,se=0.11,t=−0.43,var=0.01
DNAm
ancestry
chr19_8585386_8586001
local: beta=0.11,se=0.03,t=3.54,var=0.07
global: beta=0.14,se=0.09,t=1.69,var=0.01
DNAm
ancestry
chr2_10042415_10042646
local: beta=−0.13,se=0.04,t=−3.29,var=0.081
global: beta=−0.07,se=0.12,t=−0.57,var=0.01
DNAm
ancestry
chr2_10573007_10573481
local: beta=−0.06,se=0.01,t=−4.22,var=0.083
global: beta=−0.06,se=0.04,t=−1.49,var=0.01
DNAm
ancestry

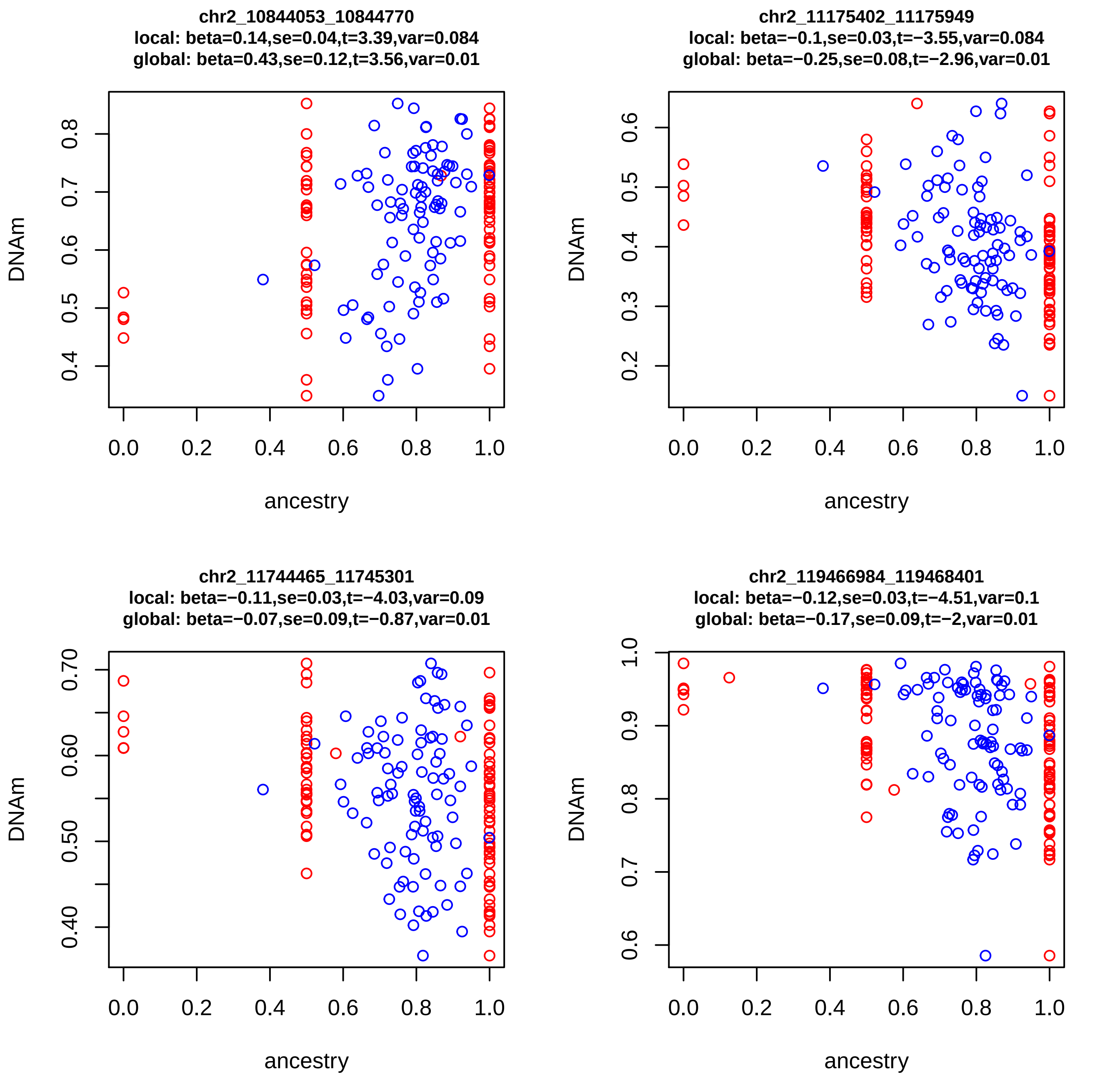
chr2_10844053_10844770
local: beta=0.14,se=0.04,t=3.39,var=0.084
global: beta=0.43,se=0.12,t=3.56,var=0.01
DNAm
0.4
0.5
0.6
0.7
0.8
0.0
0.2
0.4
0.6
0.8
1.0
ancestry
chr2_11175402_11175949
local: beta=−0.1,se=0.03,t=−3.55,var=0.084
global: beta=−0.25,se=0.08,t=−2.96,var=0.01
DNAm
0.2
0.3
0.4
0.5
0.6
0.0
0.2
0.4
0.6
0.8
1.0
ancestry
chr2_11744465_11745301
local: beta=−0.11,se=0.03,t=−4.03,var=0.09
global: beta=−0.07,se=0.09,t=−0.87,var=0.01
DNAm
0.40
0.50
0.60
0.70
0.0
0.2
0.4
0.6
0.8
1.0
ancestry
chr2_119466984_119468401
local: beta=−0.12,se=0.03,t=−4.51,var=0.1
global: beta=−0.17,se=0.09,t=−2,var=0.01
DNAm
0.6
0.7
0.8
0.9
1.0
0.0
0.2
0.4
0.6
0.8
1.0
ancestry

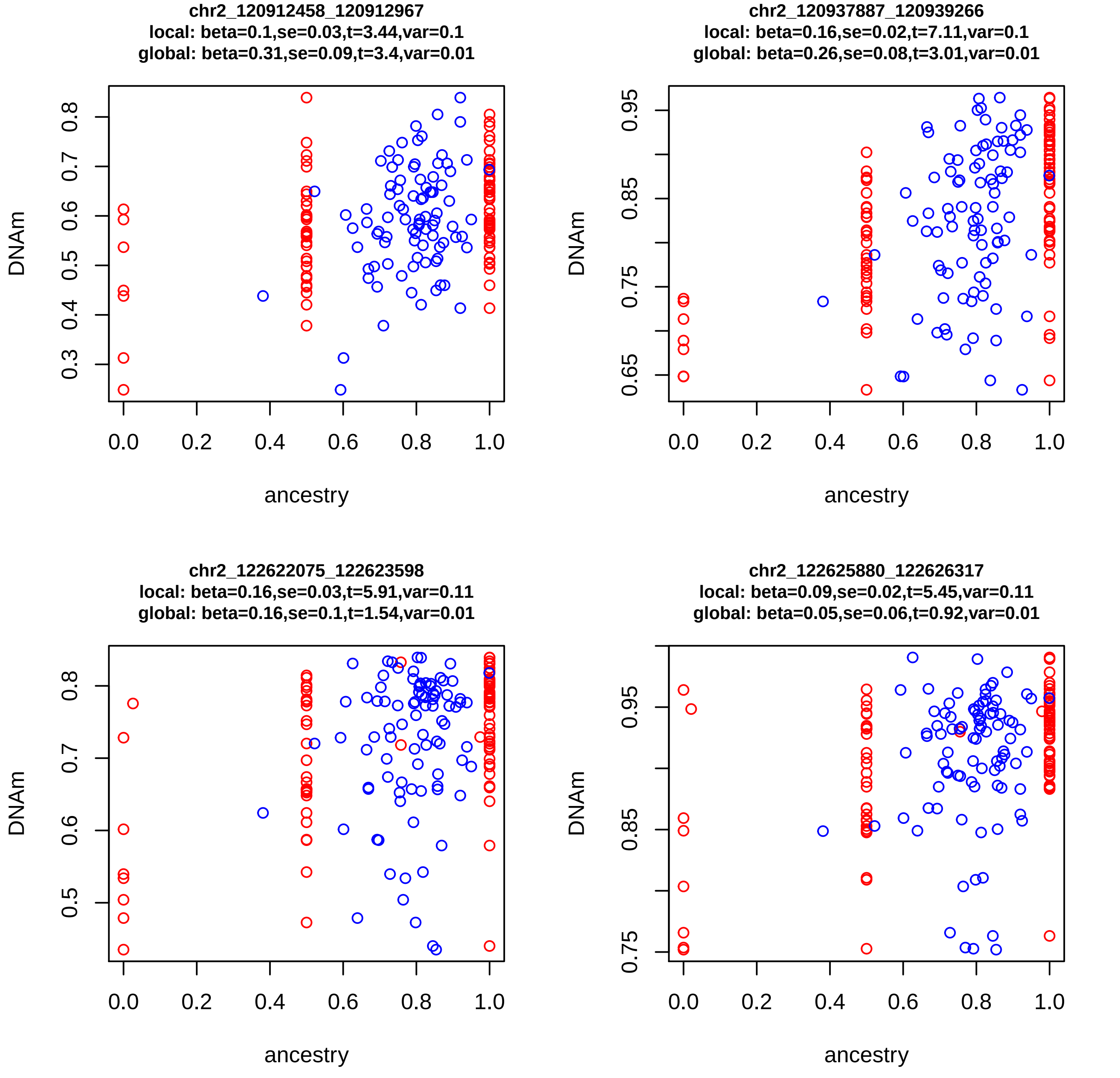
chr2_120912458_120912967
local: beta=0.1,se=0.03,t=3.44,var=0.1
global: beta=0.31,se=0.09,t=3.4,var=0.01
DNAm
ancestry
chr2_120937887_120939266
local: beta=0.16,se=0.02,t=7.11,var=0.1
global: beta=0.26,se=0.08,t=3.01,var=0.01
DNAm
ancestry
chr2_122622075_122623598
local: beta=0.16,se=0.03,t=5.91,var=0.11
global: beta=0.16,se=0.1,t=1.54,var=0.01
DNAm
ancestry
chr2_122625880_122626317
local: beta=0.09,se=0.02,t=5.45,var=0.11
global: beta=0.05,se=0.06,t=0.92,var=0.01
DNAm
ancestry

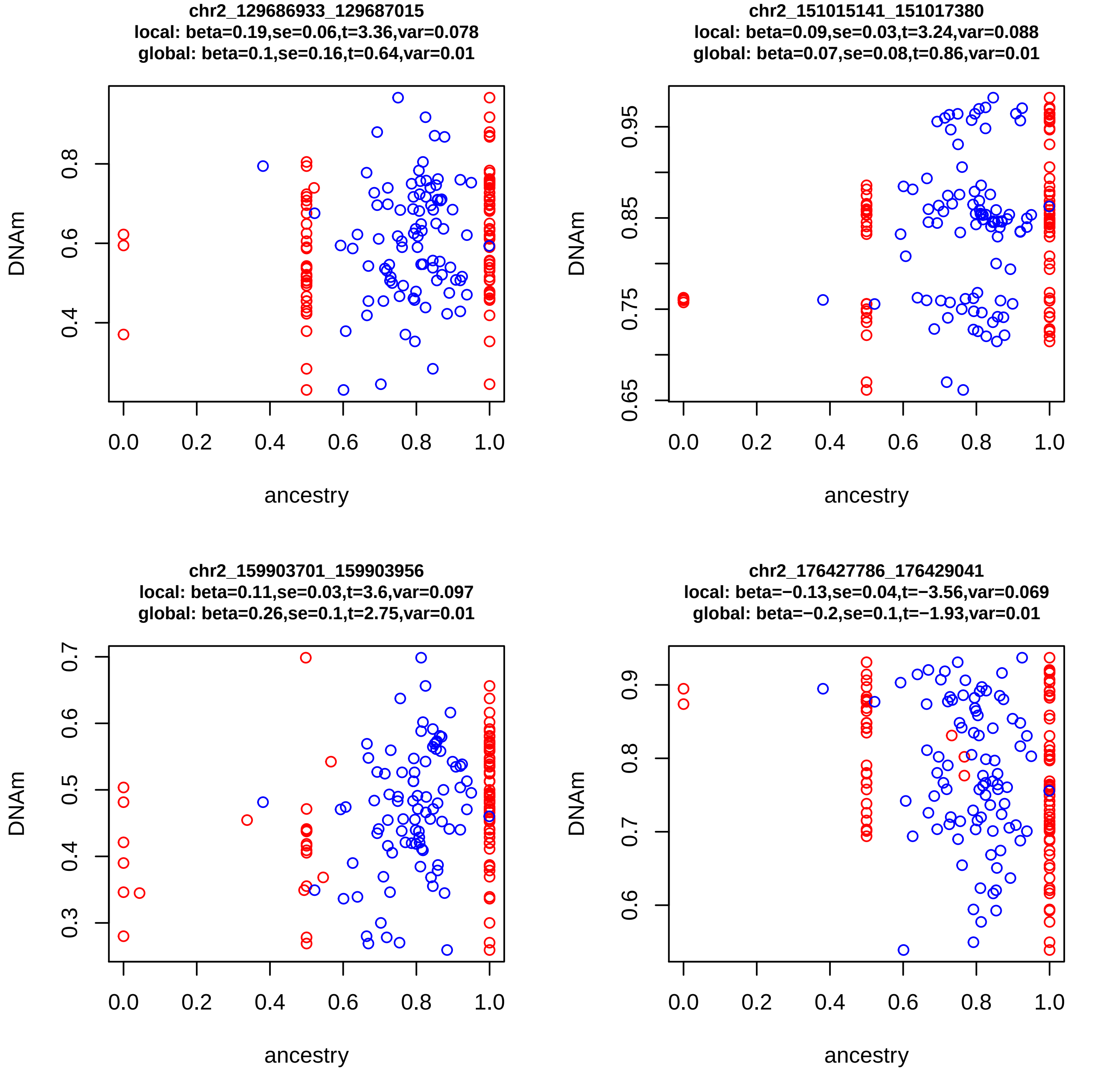
chr2_129686933_129687015
local: beta=0.19,se=0.06,t=3.36,var=0.078
global: beta=0.1,se=0.16,t=0.64,var=0.01
DNAm
ancestry
chr2_151015141_151017380
local: beta=0.09,se=0.03,t=3.24,var=0.088
global: beta=0.07,se=0.08,t=0.86,var=0.01
DNAm
ancestry
chr2_159903701_159903956
local: beta=0.11,se=0.03,t=3.6,var=0.097
global: beta=0.26,se=0.1,t=2.75,var=0.01
DNAm
ancestry
chr2_176427786_176429041
local: beta=−0.13,se=0.04,t=−3.56,var=0.069
global: beta=−0.2,se=0.1,t=−1.93,var=0.01
DNAm
ancestry

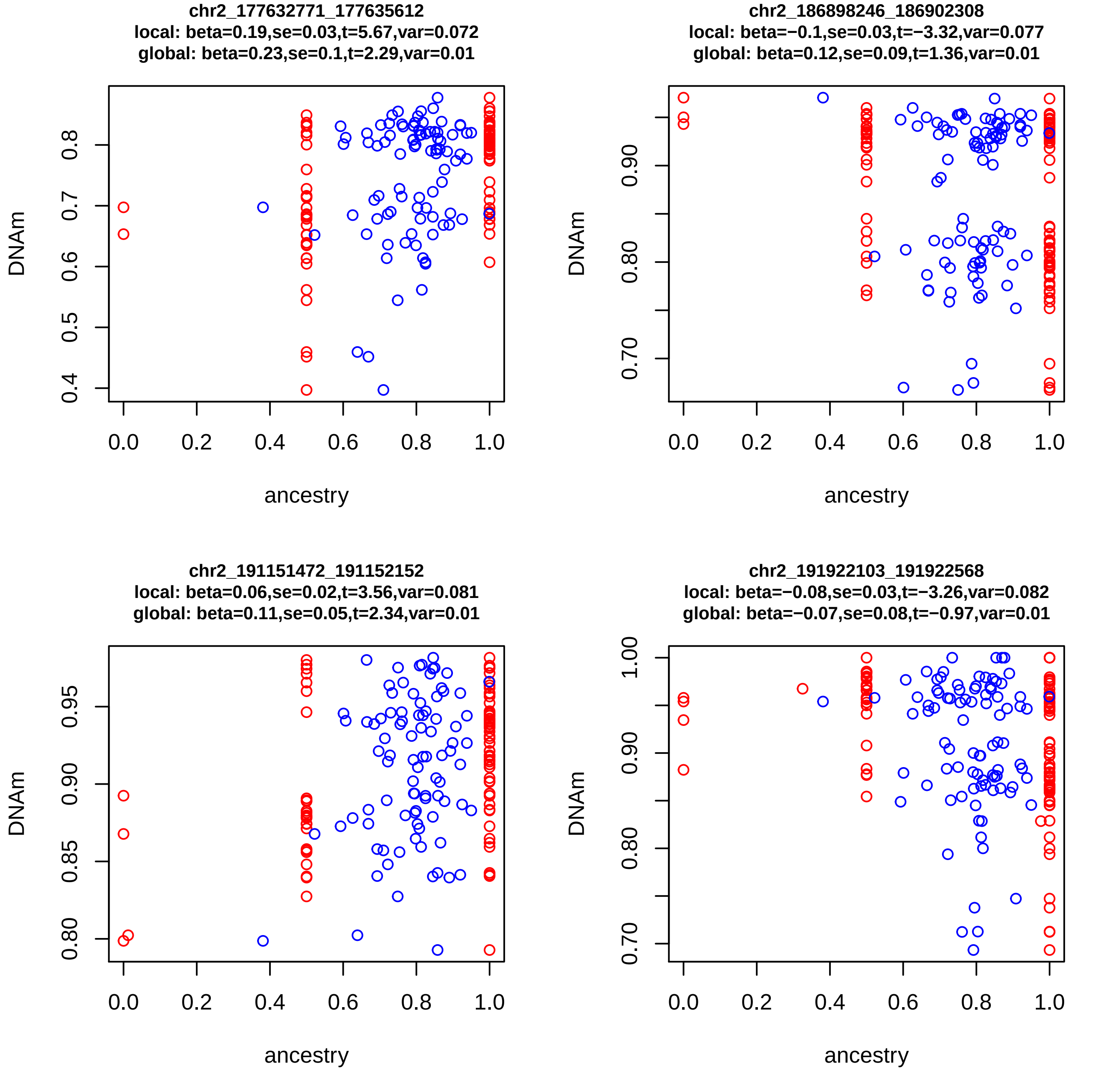
chr2_177632771_177635612
local: beta=0.19,se=0.03,t=5.67,var=0.072
global: beta=0.23,se=0.1,t=2.29,var=0.01
DNAm
0.4
0.5
0.6
0.7
0.8
ancestry
0.0
0.2
0.4
0.6
0.8
1.0
chr2_186898246_186902308
local: beta=−0.1,se=0.03,t=−3.32,var=0.077
global: beta=0.12,se=0.09,t=1.36,var=0.01
DNAm
0.70
0.80
0.90
ancestry
0.0
0.2
0.4
0.6
0.8
1.0
chr2_191151472_191152152
local: beta=0.06,se=0.02,t=3.56,var=0.081
global: beta=0.11,se=0.05,t=2.34,var=0.01
DNAm
0.80
0.85
0.90
0.95
ancestry
0.0
0.2
0.4
0.6
0.8
1.0
chr2_191922103_191922568
local: beta=−0.08,se=0.03,t=−3.26,var=0.082
global: beta=−0.07,se=0.08,t=−0.97,var=0.01
DNAm
0.70
0.80
0.90
1.00
ancestry
0.0
0.2
0.4
0.6
0.8
1.0

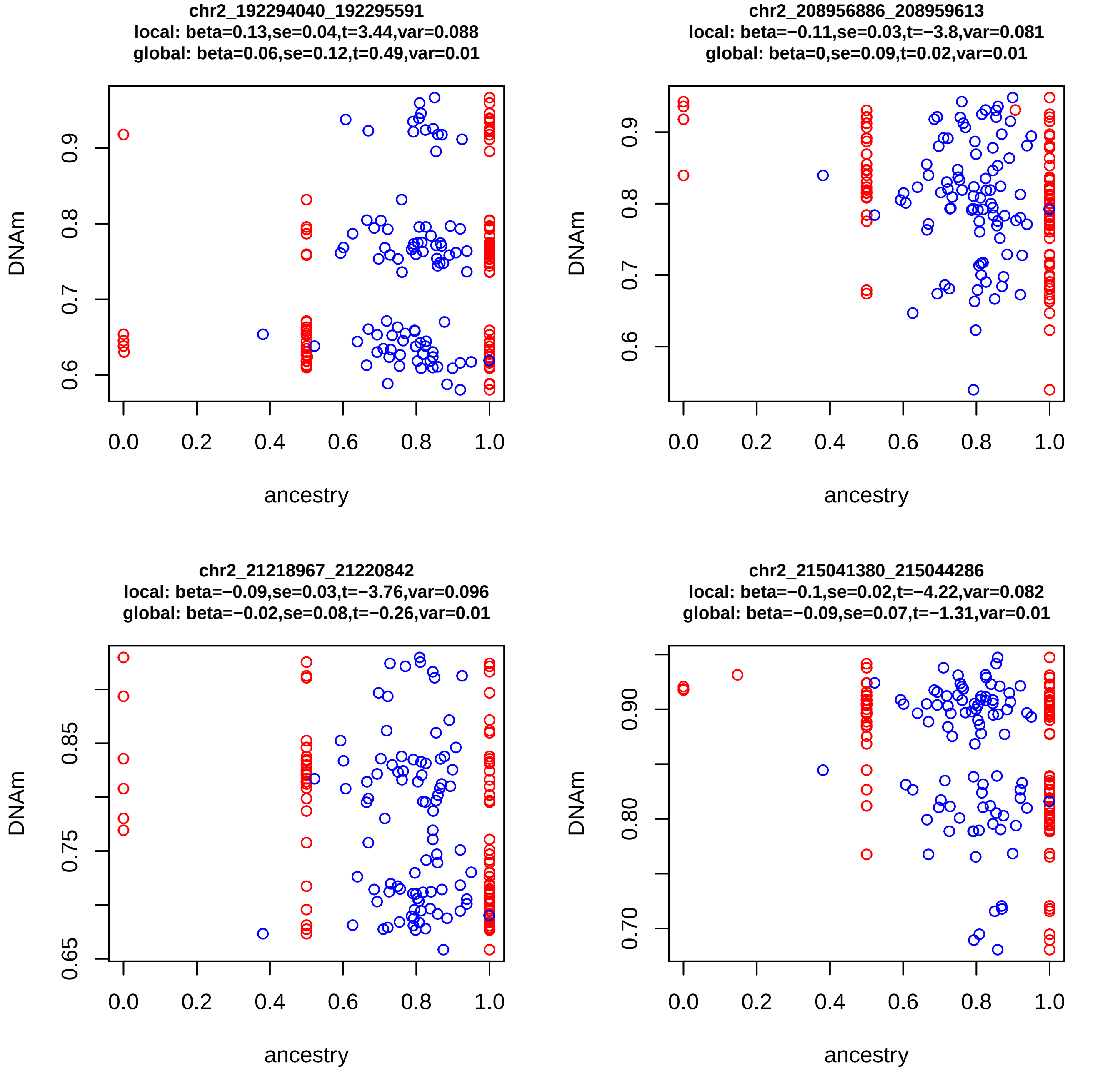
chr2_192294040_192295591
local: beta=0.13,se=0.04,t=3.44,var=0.088
global: beta=0.06,se=0.12,t=0.49,var=0.01
chr2_208956886_208959613
local: beta=−0.11,se=0.03,t=−3.8,var=0.081
global: beta=0,se=0.09,t=0.02,var=0.01
chr2_21218967_21220842
local: beta=−0.09,se=0.03,t=−3.76,var=0.096
global: beta=−0.02,se=0.08,t=−0.26,var=0.01
chr2_215041380_215044286
local: beta=−0.1,se=0.02,t=−4.22,var=0.082
global: beta=−0.09,se=0.07,t=−1.31,var=0.01
DNAm
ancestry

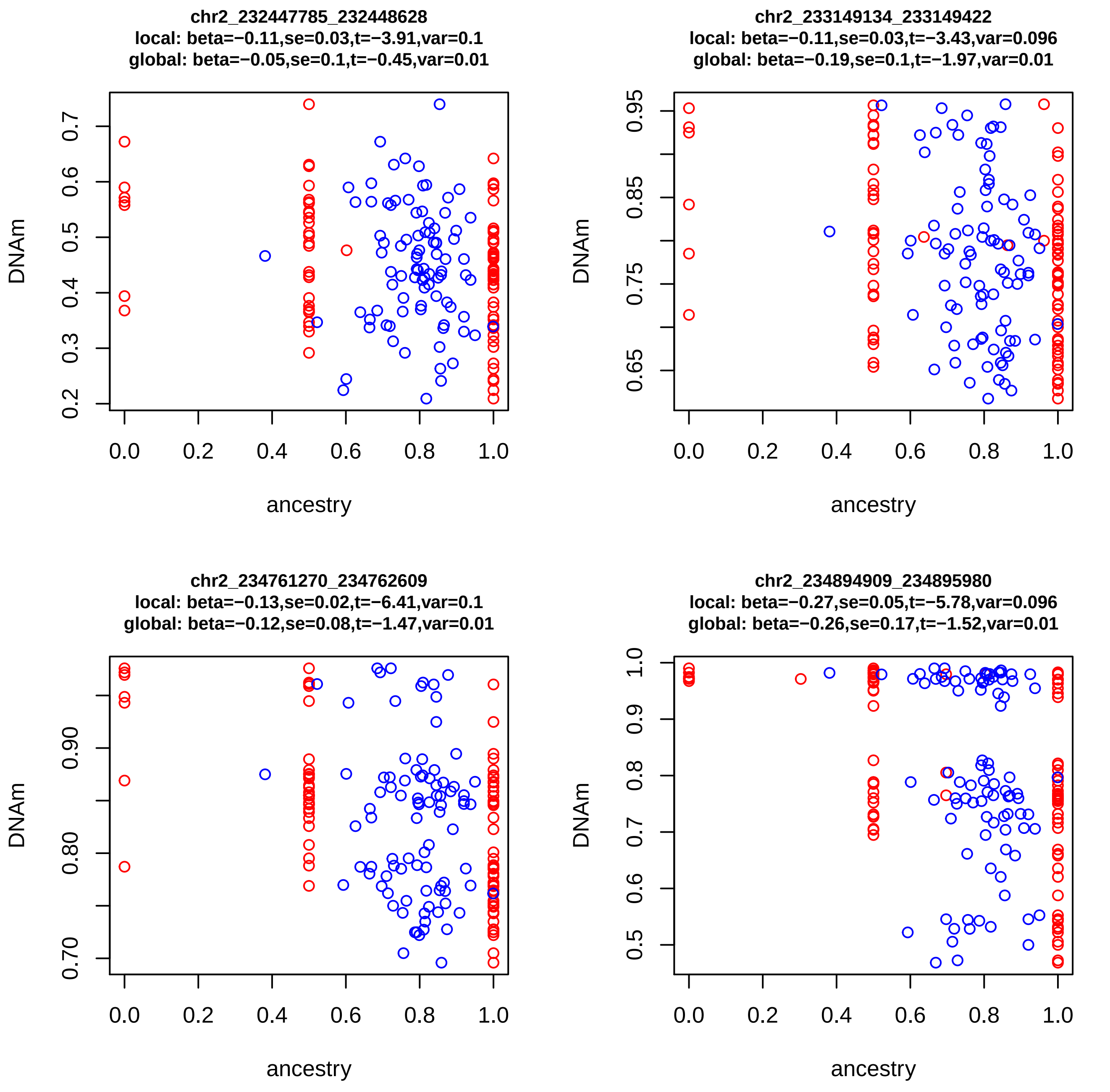
chr2_232447785_232448628
local: beta=−0.11,se=0.03,t=−3.91,var=0.1
global: beta=−0.05,se=0.1,t=−0.45,var=0.01
DNAm
0.2
0.3
0.4
0.5
0.6
0.7
0.0
0.2
0.4
0.6
0.8
1.0
ancestry
chr2_233149134_233149422
local: beta=−0.11,se=0.03,t=−3.43,var=0.096
global: beta=−0.19,se=0.1,t=−1.97,var=0.01
DNAm
0.65
0.75
0.85
0.95
ancestry
chr2_234761270_234762609
local: beta=−0.13,se=0.02,t=−6.41,var=0.1
global: beta=−0.12,se=0.08,t=−1.47,var=0.01
DNAm
0.70
0.80
0.90
ancestry
chr2_234894909_234895980
local: beta=−0.27,se=0.05,t=−5.78,var=0.096
global: beta=−0.26,se=0.17,t=−1.52,var=0.01
DNAm
0.5
0.6
0.7
0.8
0.9
1.0
ancestry

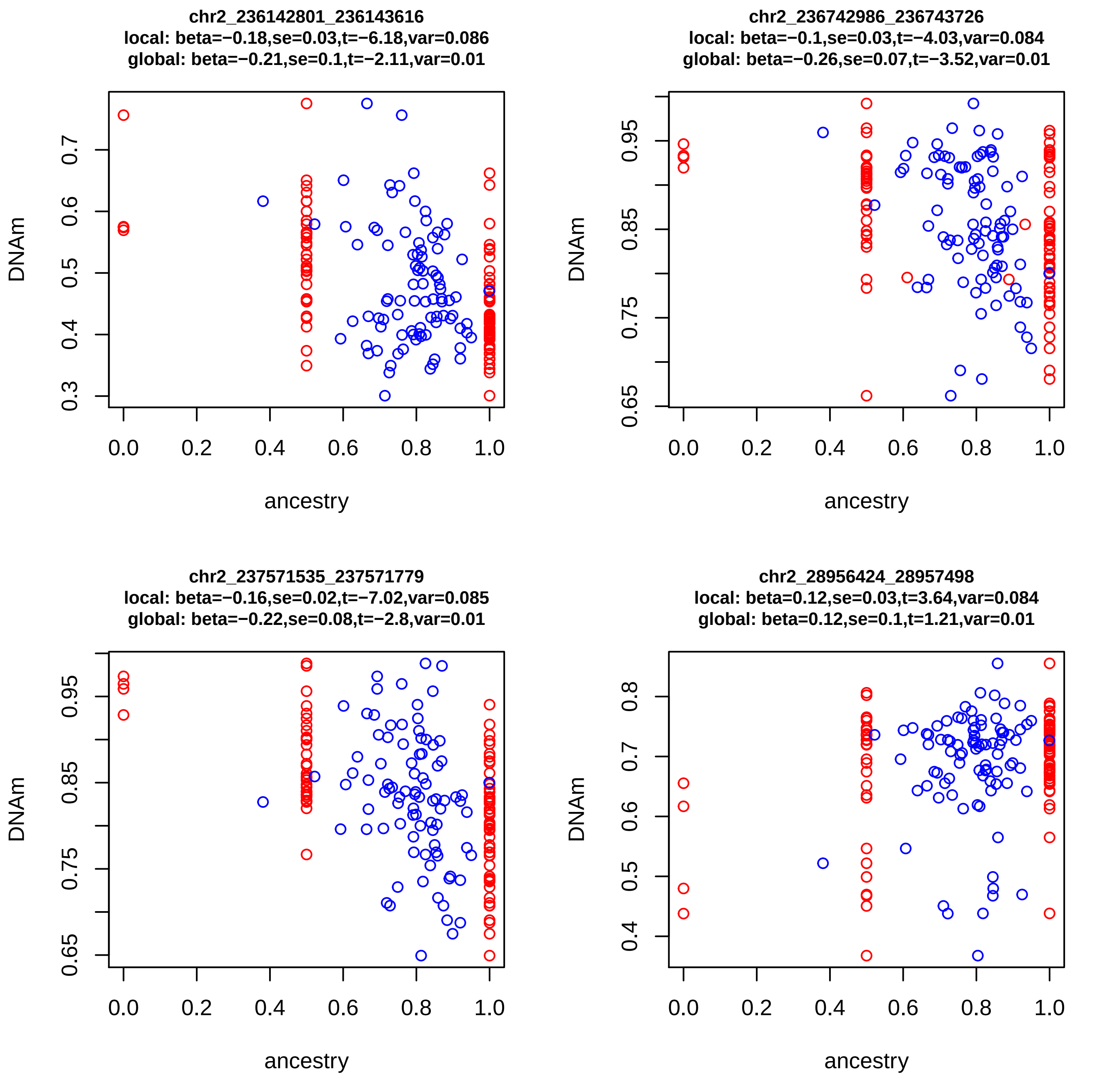
chr2_236142801_236143616
local: beta=−0.18,se=0.03,t=−6.18,var=0.086
global: beta=−0.21,se=0.1,t=−2.11,var=0.01
DNAm
ancestry
chr2_236742986_236743726
local: beta=−0.1,se=0.03,t=−4.03,var=0.084
global: beta=−0.26,se=0.07,t=−3.52,var=0.01
DNAm
ancestry
chr2_237571535_237571779
local: beta=−0.16,se=0.02,t=−7.02,var=0.085
global: beta=−0.22,se=0.08,t=−2.8,var=0.01
DNAm
ancestry
chr2_28956424_28957498
local: beta=0.12,se=0.03,t=3.64,var=0.084
global: beta=0.12,se=0.1,t=1.21,var=0.01
DNAm
ancestry

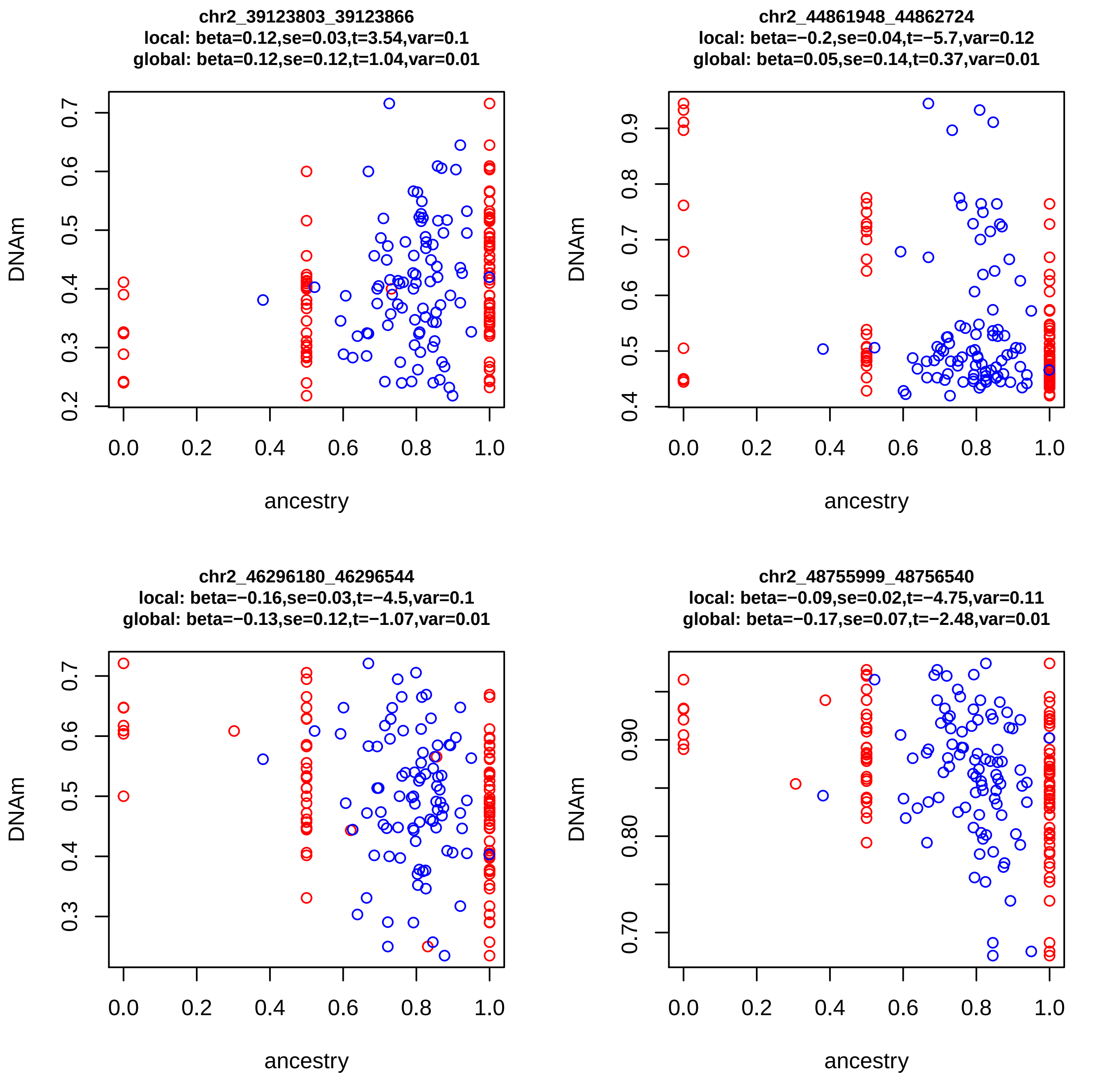
chr2_39123803_39123866
local: beta=0.12,se=0.03,t=3.54,var=0.1
global: beta=0.12,se=0.12,t=1.04,var=0.01
DNAm
ancestry
chr2_44861948_44862724
local: beta=−0.2,se=0.04,t=−5.7,var=0.12
global: beta=0.05,se=0.14,t=0.37,var=0.01
DNAm
ancestry
chr2_46296180_46296544
local: beta=−0.16,se=0.03,t=−4.5,var=0.1
global: beta=−0.13,se=0.12,t=−1.07,var=0.01
DNAm
ancestry
chr2_48755999_48756540
local: beta=−0.09,se=0.02,t=−4.75,var=0.11
global: beta=−0.17,se=0.07,t=−2.48,var=0.01
DNAm
ancestry

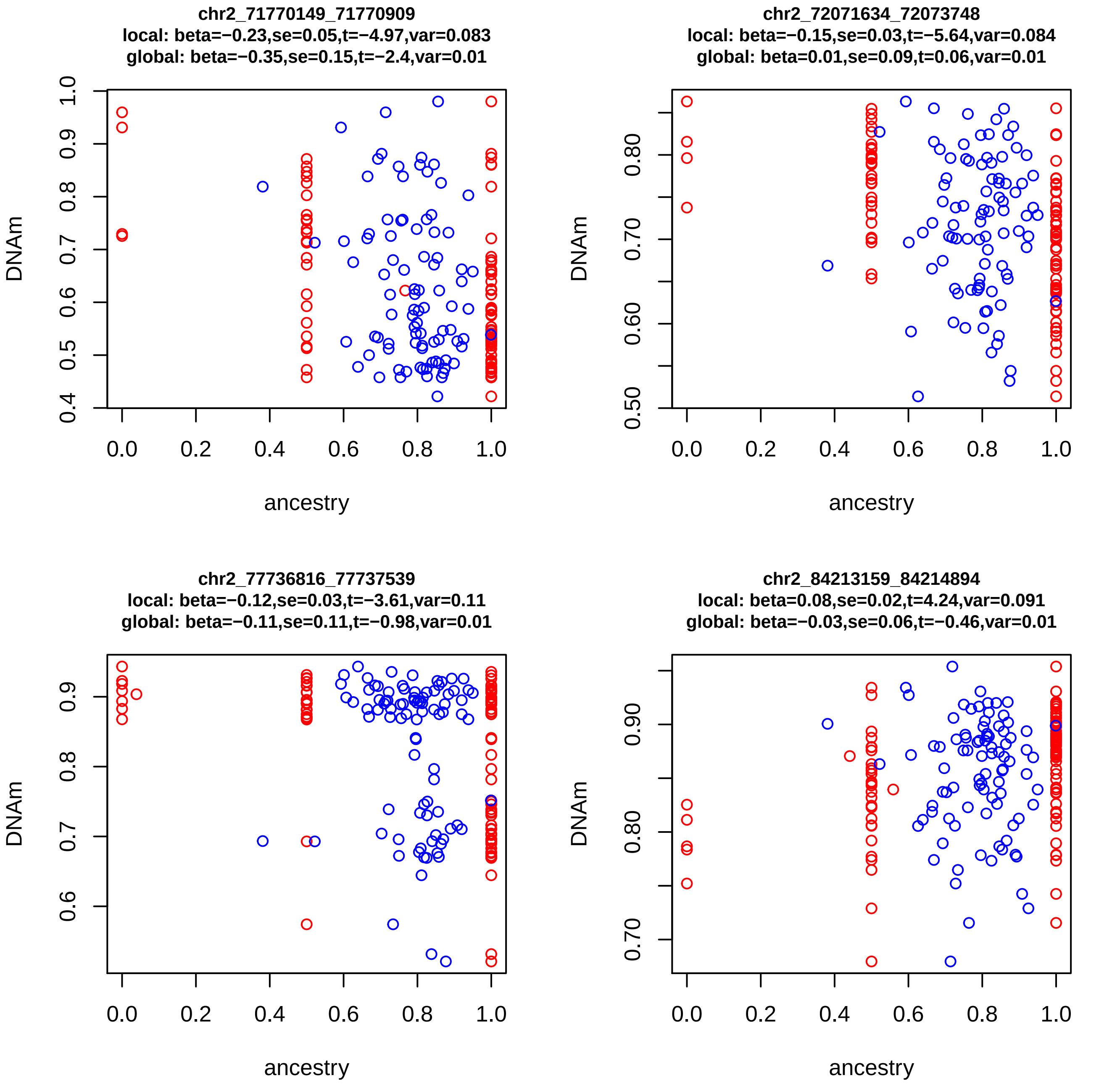
chr2_71770149_71770909
local: beta=−0.23,se=0.05,t=−4.97,var=0.083
global: beta=−0.35,se=0.15,t=−2.4,var=0.01
DNAm
0.4
0.5
0.6
0.7
0.8
0.9
1.0
0.0
0.2
0.4
0.6
0.8
1.0
ancestry
chr2_72071634_72073748
local: beta=−0.15,se=0.03,t=−5.64,var=0.084
global: beta=0.01,se=0.09,t=0.06,var=0.01
DNAm
0.50
0.60
0.70
0.80
0.0
0.2
0.4
0.6
0.8
1.0
ancestry
chr2_77736816_77737539
local: beta=−0.12,se=0.03,t=−3.61,var=0.11
global: beta=−0.11,se=0.11,t=−0.98,var=0.01
DNAm
0.6
0.7
0.8
0.9
0.0
0.2
0.4
0.6
0.8
1.0
ancestry
chr2_84213159_84214894
local: beta=0.08,se=0.02,t=4.24,var=0.091
global: beta=−0.03,se=0.06,t=−0.46,var=0.01
DNAm
0.70
0.80
0.90
0.0
0.2
0.4
0.6
0.8
1.0
ancestry

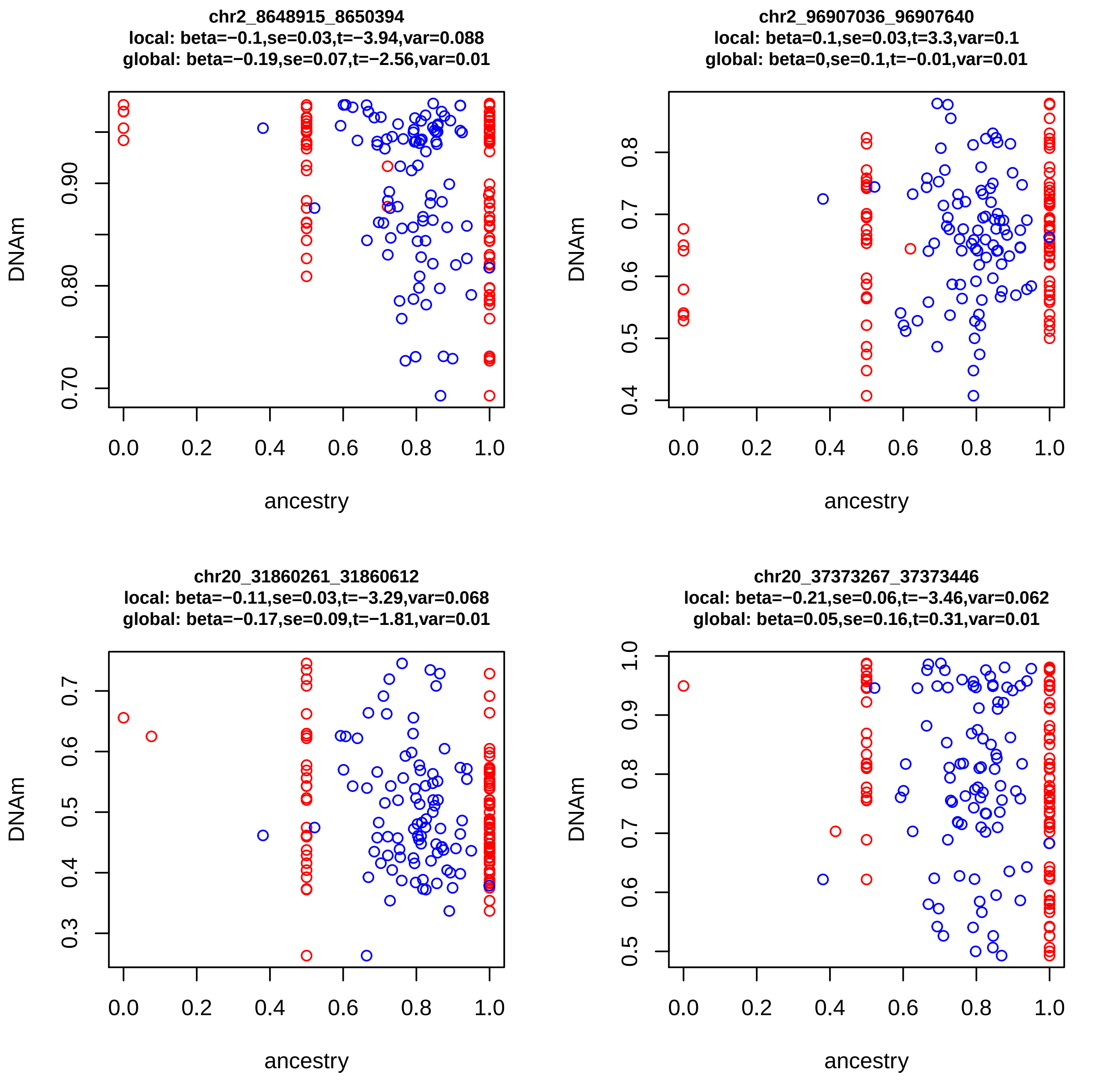
chr2_8648915_8650394
local: beta=−0.1,se=0.03,t=−3.94,var=0.088
global: beta=−0.19,se=0.07,t=−2.56,var=0.01
DNAm
0.70
0.80
0.90
0.0
0.2
0.4
0.6
0.8
1.0
ancestry
chr2_96907036_96907640
local: beta=0.1,se=0.03,t=3.3,var=0.1
global: beta=0,se=0.1,t=−0.01,var=0.01
DNAm
0.4
0.5
0.6
0.7
0.8
0.0
0.2
0.4
0.6
0.8
1.0
ancestry
chr20_31860261_31860612
local: beta=−0.11,se=0.03,t=−3.29,var=0.068
global: beta=−0.17,se=0.09,t=−1.81,var=0.01
DNAm
0.3
0.4
0.5
0.6
0.7
0.0
0.2
0.4
0.6
0.8
1.0
ancestry
chr20_37373267_37373446
local: beta=−0.21,se=0.06,t=−3.46,var=0.062
global: beta=0.05,se=0.16,t=0.31,var=0.01
DNAm
0.5
0.6
0.7
0.8
0.9
1.0
0.0
0.2
0.4
0.6
0.8
1.0
ancestry

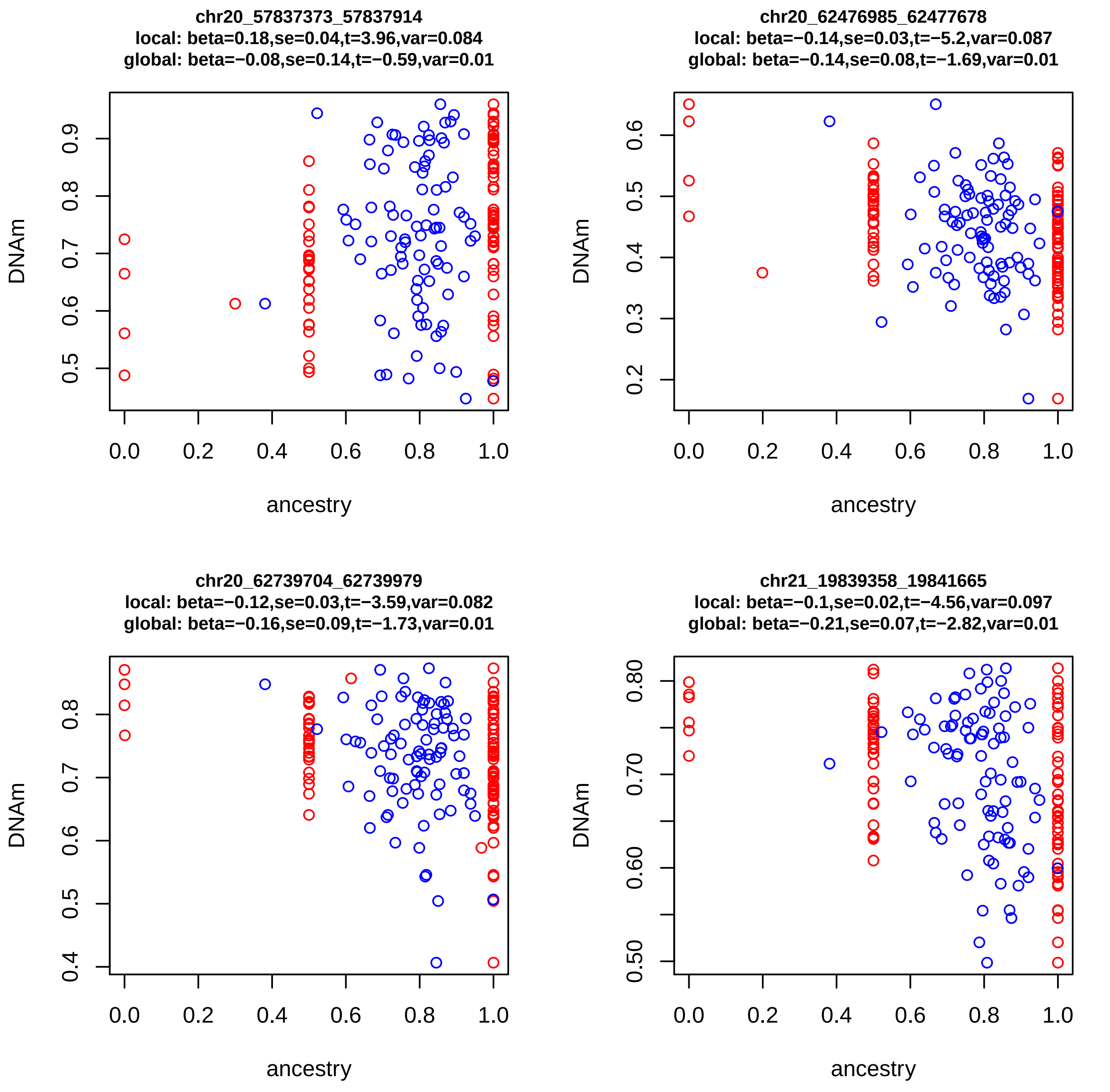
chr20_57837373_57837914
local: beta=0.18,se=0.04,t=3.96,var=0.084
global: beta=−0.08,se=0.14,t=−0.59,var=0.01
chr20_62476985_62477678
local: beta=−0.14,se=0.03,t=−5.2,var=0.087
global: beta=−0.14,se=0.08,t=−1.69,var=0.01
chr20_62739704_62739979
local: beta=−0.12,se=0.03,t=−3.59,var=0.082
global: beta=−0.16,se=0.09,t=−1.73,var=0.01
chr21_19839358_19841665
local: beta=−0.1,se=0.02,t=−4.56,var=0.097
global: beta=−0.21,se=0.07,t=−2.82,var=0.01
DNAm
ancestry

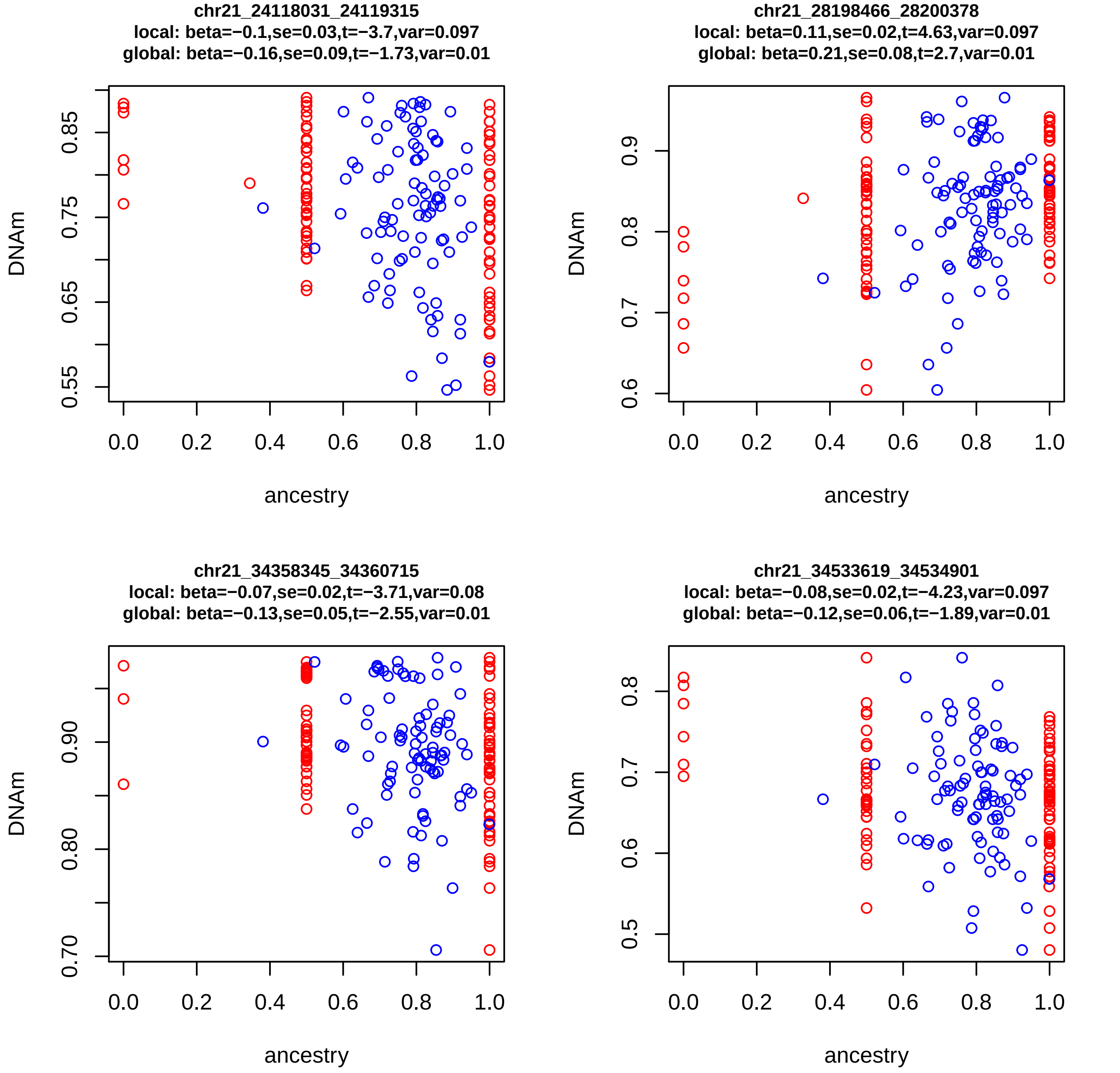
chr21_24118031_24119315
local: beta=−0.1,se=0.03,t=−3.7,var=0.097
global: beta=−0.16,se=0.09,t=−1.73,var=0.01
DNAm
0.55
0.65
0.75
0.85
0.0
0.2
0.4
0.6
0.8
1.0
ancestry
chr21_28198466_28200378
local: beta=0.11,se=0.02,t=4.63,var=0.097
global: beta=0.21,se=0.08,t=2.7,var=0.01
DNAm
0.6
0.7
0.8
0.9
0.0
0.2
0.4
0.6
0.8
1.0
ancestry
chr21_34358345_34360715
local: beta=−0.07,se=0.02,t=−3.71,var=0.08
global: beta=−0.13,se=0.05,t=−2.55,var=0.01
DNAm
0.70
0.80
0.90
0.0
0.2
0.4
0.6
0.8
1.0
ancestry
chr21_34533619_34534901
local: beta=−0.08,se=0.02,t=−4.23,var=0.097
global: beta=−0.12,se=0.06,t=−1.89,var=0.01
DNAm
0.5
0.6
0.7
0.8
0.0
0.2
0.4
0.6
0.8
1.0
ancestry

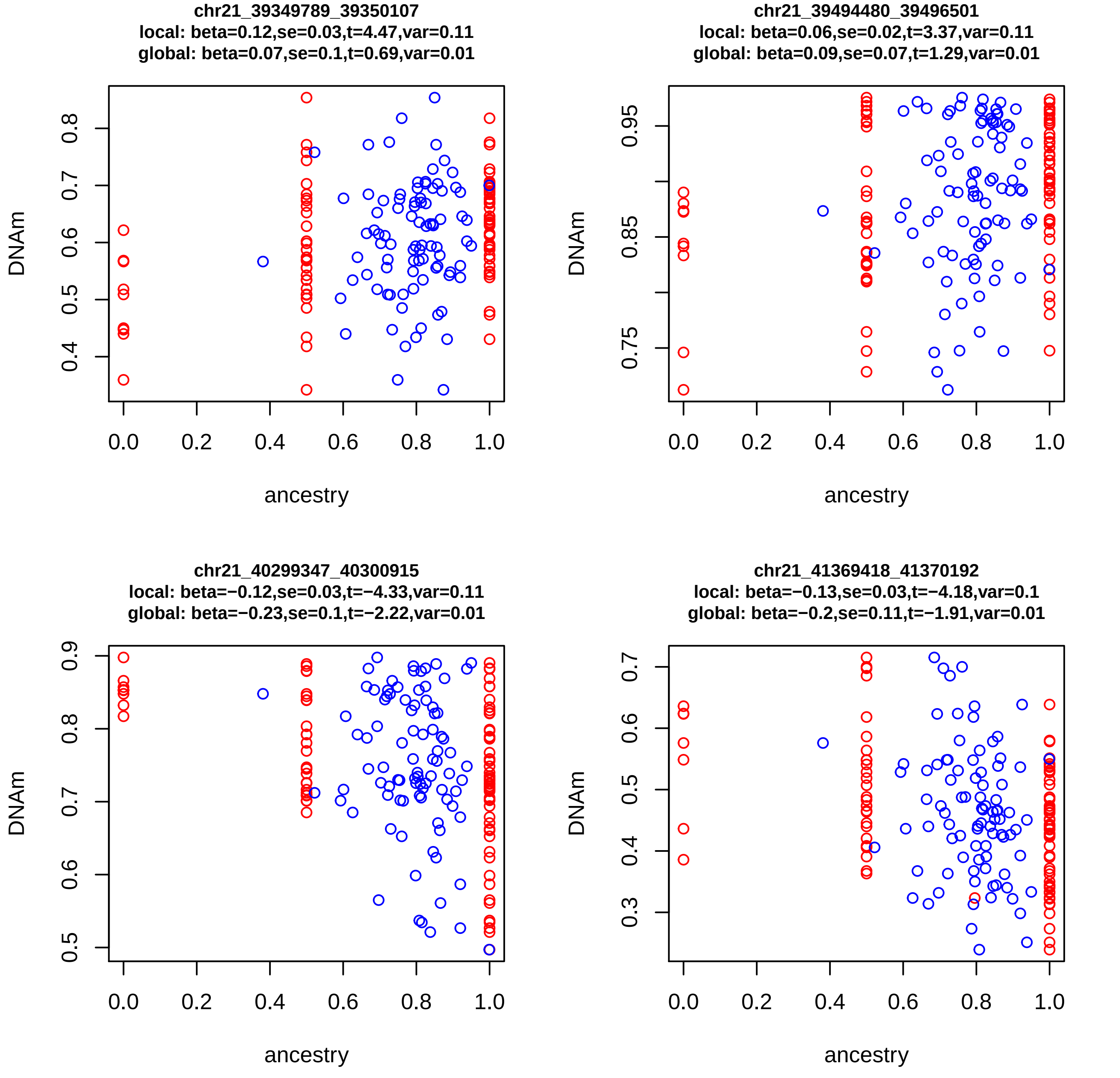
chr21_39349789_39350107
local: beta=0.12,se=0.03,t=4.47,var=0.11
global: beta=0.07,se=0.1,t=0.69,var=0.01
DNAm
ancestry
chr21_39494480_39496501
local: beta=0.06,se=0.02,t=3.37,var=0.11
global: beta=0.09,se=0.07,t=1.29,var=0.01
DNAm
ancestry
chr21_40299347_40300915
local: beta=−0.12,se=0.03,t=−4.33,var=0.11
global: beta=−0.23,se=0.1,t=−2.22,var=0.01
DNAm
ancestry
chr21_41369418_41370192
local: beta=−0.13,se=0.03,t=−4.18,var=0.1
global: beta=−0.2,se=0.11,t=−1.91,var=0.01
DNAm
ancestry

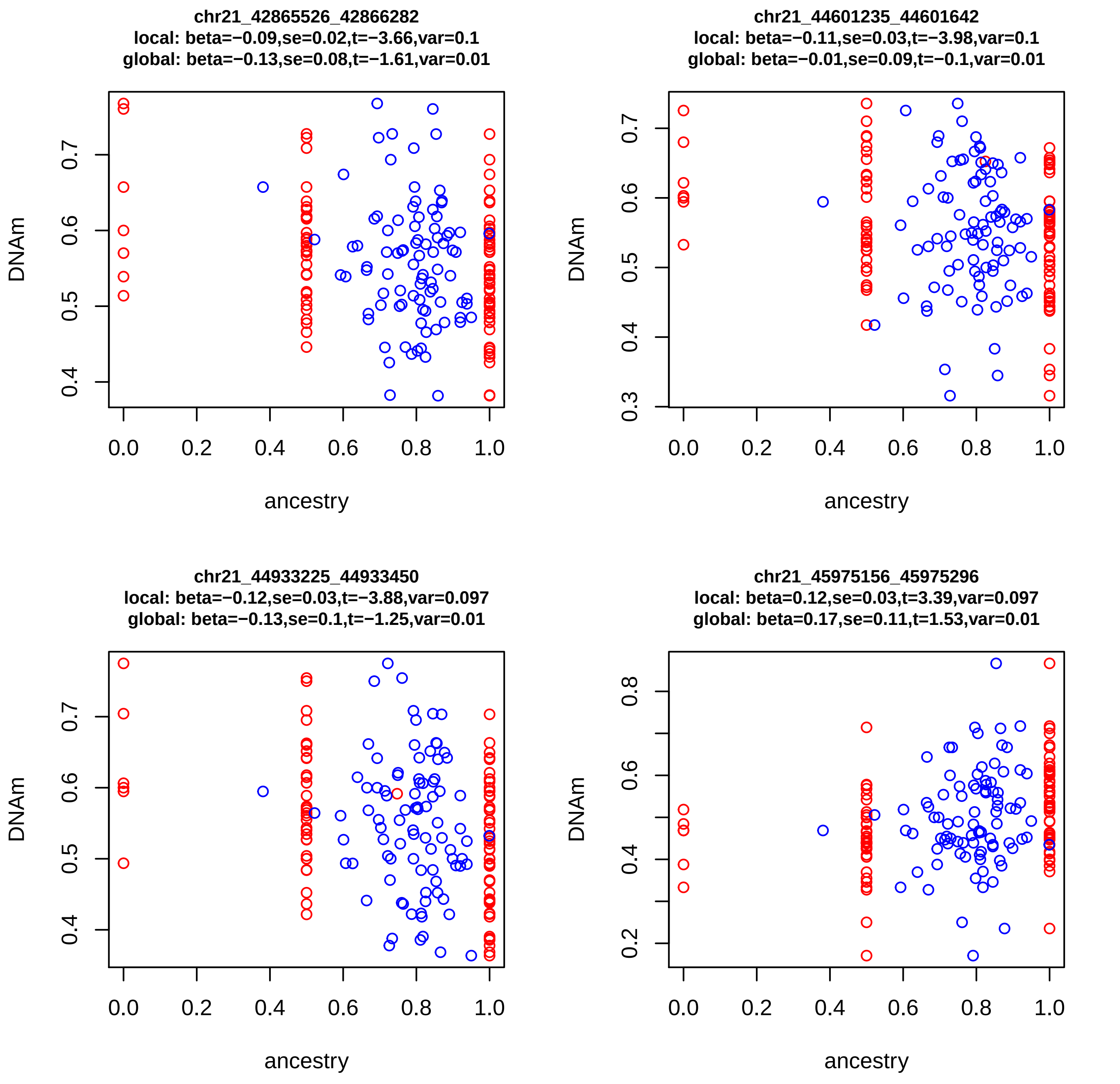
chr21_42865526_42866282
local: beta=−0.09,se=0.02,t=−3.66,var=0.1
global: beta=−0.13,se=0.08,t=−1.61,var=0.01
chr21_44601235_44601642
local: beta=−0.11,se=0.03,t=−3.98,var=0.1
global: beta=−0.01,se=0.09,t=−0.1,var=0.01
chr21_44933225_44933450
local: beta=−0.12,se=0.03,t=−3.88,var=0.097
global: beta=−0.13,se=0.1,t=−1.25,var=0.01
chr21_45975156_45975296
local: beta=0.12,se=0.03,t=3.39,var=0.097
global: beta=0.17,se=0.11,t=1.53,var=0.01
DNAm
ancestry

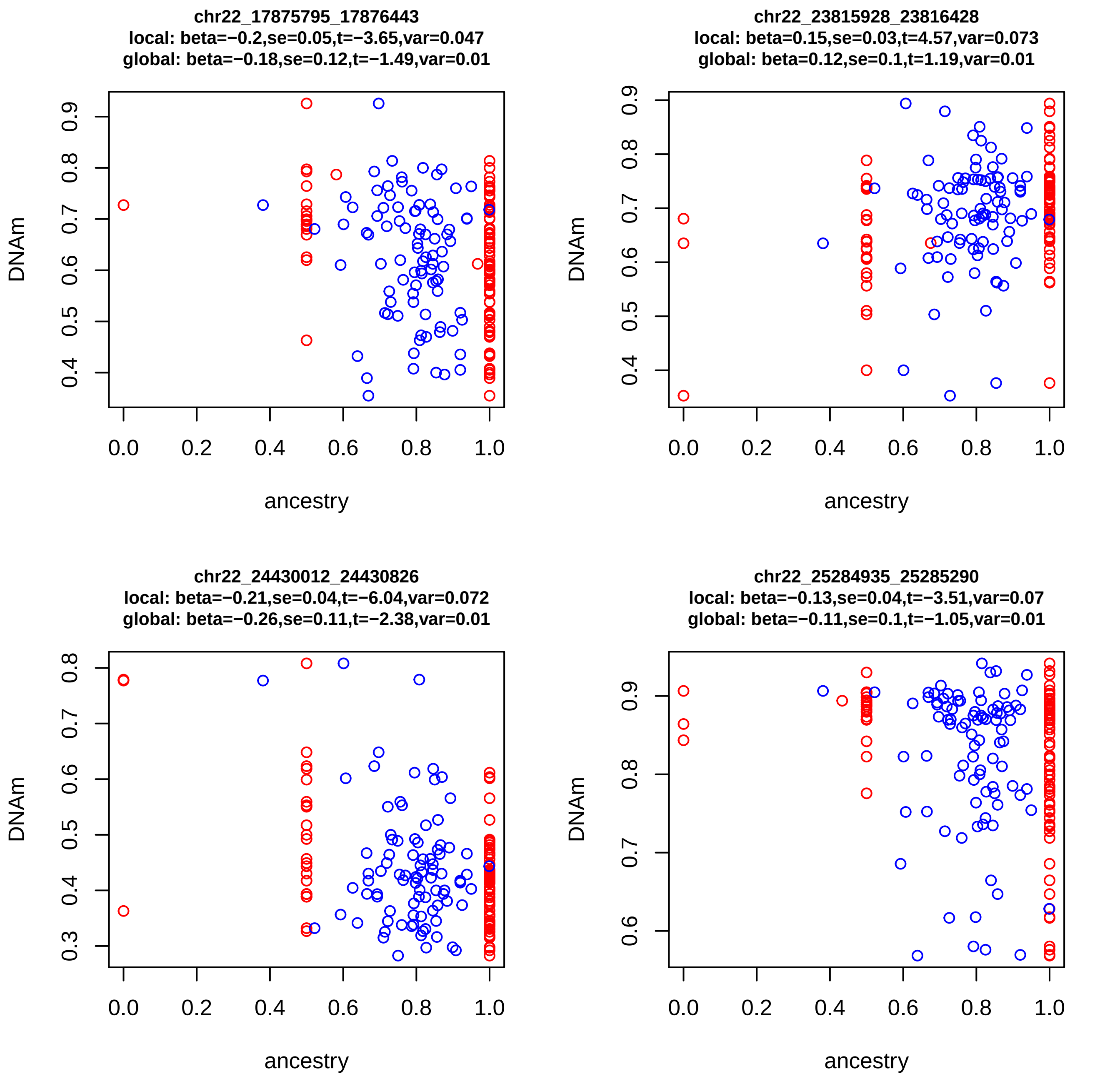
chr22_17875795_17876443
local: beta=−0.2,se=0.05,t=−3.65,var=0.047
global: beta=−0.18,se=0.12,t=−1.49,var=0.01
chr22_23815928_23816428
local: beta=0.15,se=0.03,t=4.57,var=0.073
global: beta=0.12,se=0.1,t=1.19,var=0.01
chr22_24430012_24430826
local: beta=−0.21,se=0.04,t=−6.04,var=0.072
global: beta=−0.26,se=0.11,t=−2.38,var=0.01
chr22_25284935_25285290
local: beta=−0.13,se=0.04,t=−3.51,var=0.07
global: beta=−0.11,se=0.1,t=−1.05,var=0.01
DNAm
ancestry

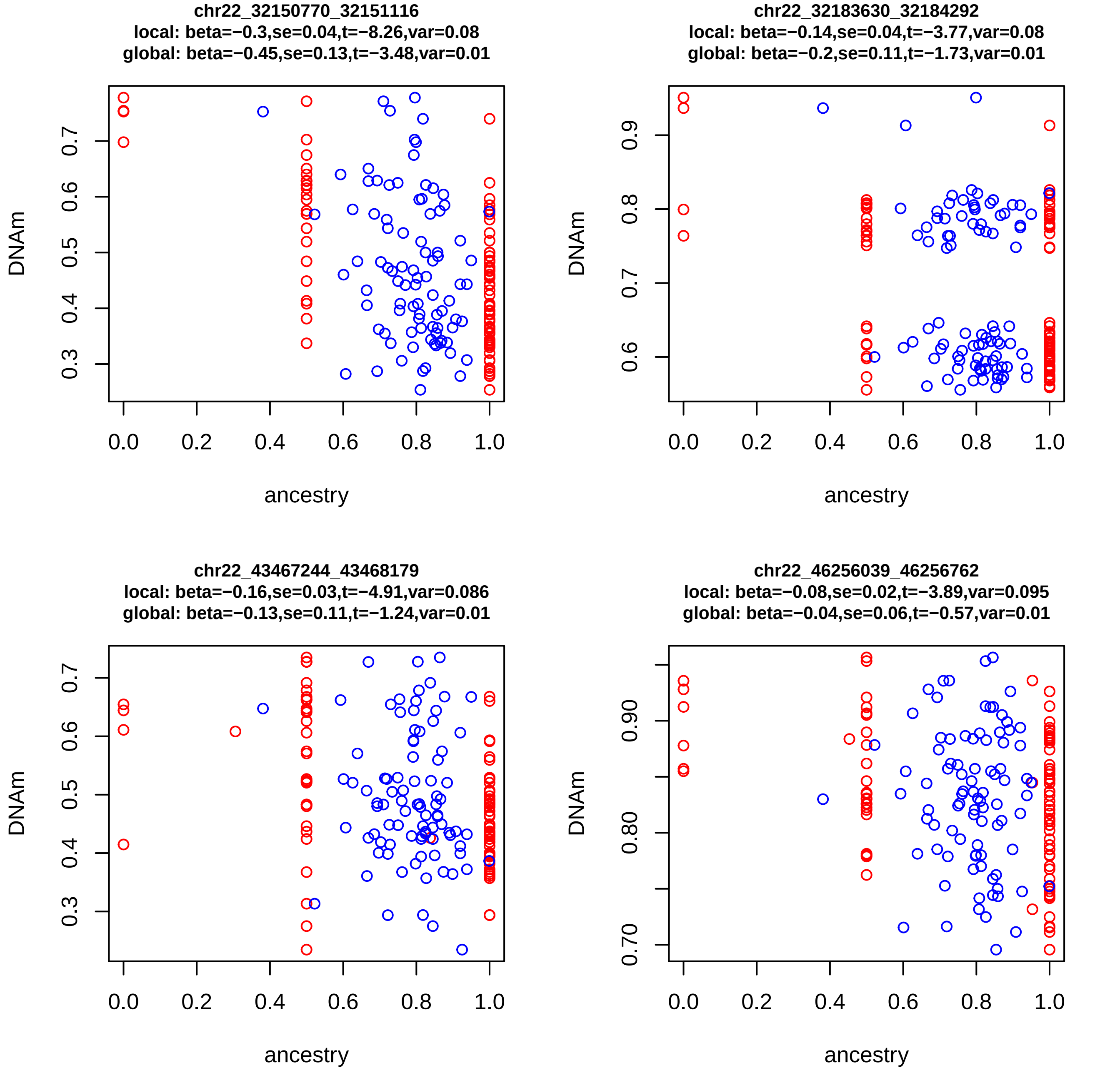
chr22_32150770_32151116
local: beta=−0.3,se=0.04,t=−8.26,var=0.08
global: beta=−0.45,se=0.13,t=−3.48,var=0.01
DNAm
ancestry
chr22_32183630_32184292
local: beta=−0.14,se=0.04,t=−3.77,var=0.08
global: beta=−0.2,se=0.11,t=−1.73,var=0.01
DNAm
ancestry
chr22_43467244_43468179
local: beta=−0.16,se=0.03,t=−4.91,var=0.086
global: beta=−0.13,se=0.11,t=−1.24,var=0.01
DNAm
ancestry
chr22_46256039_46256762
local: beta=−0.08,se=0.02,t=−3.89,var=0.095
global: beta=−0.04,se=0.06,t=−0.57,var=0.01
DNAm
ancestry

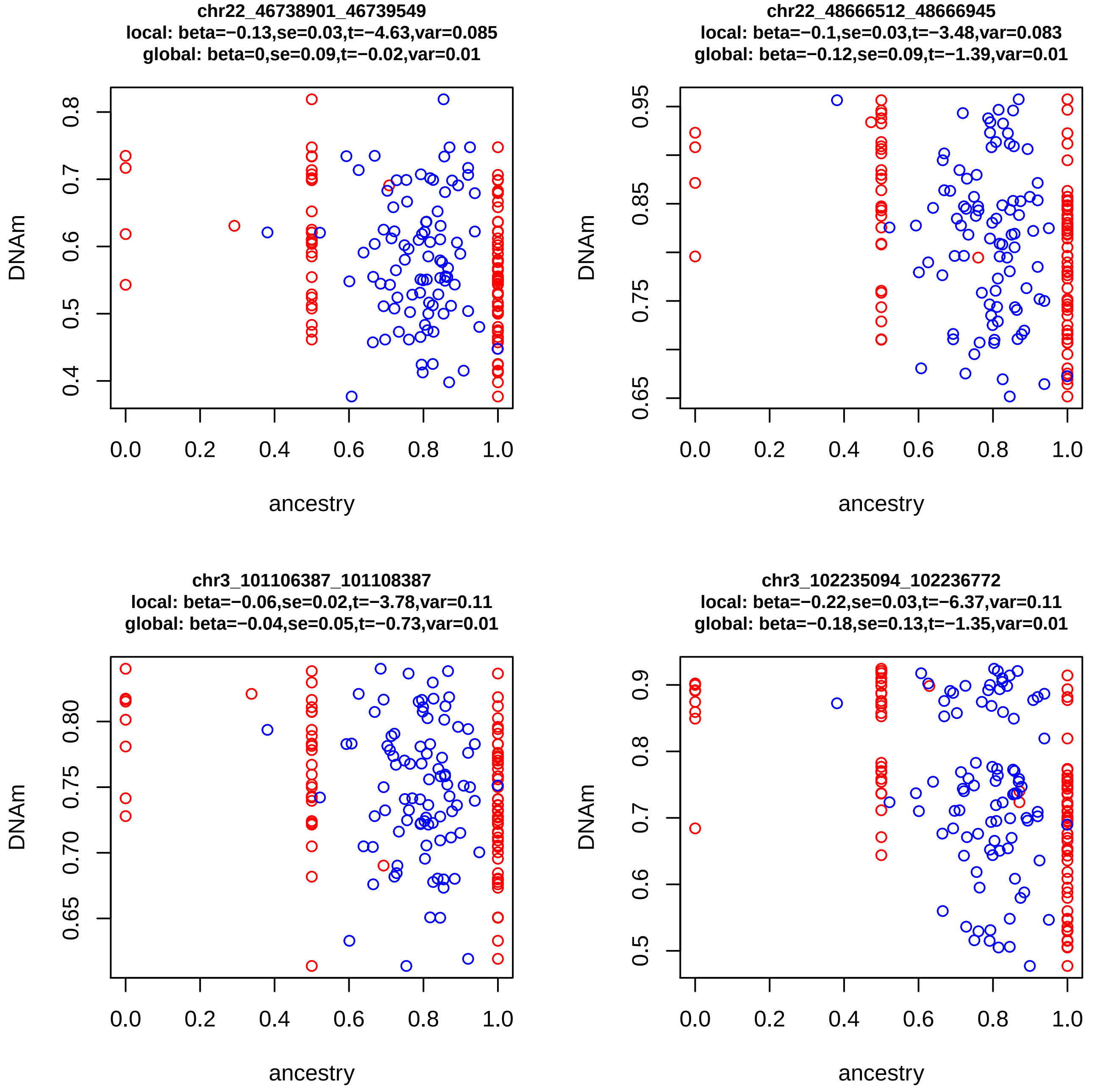
chr22_46738901_46739549
local: beta=−0.13,se=0.03,t=−4.63,var=0.085
global: beta=0,se=0.09,t=−0.02,var=0.01
DNAm
ancestry
chr22_48666512_48666945
local: beta=−0.1,se=0.03,t=−3.48,var=0.083
global: beta=−0.12,se=0.09,t=−1.39,var=0.01
DNAm
ancestry
chr3_101106387_101108387
local: beta=−0.06,se=0.02,t=−3.78,var=0.11
global: beta=−0.04,se=0.05,t=−0.73,var=0.01
DNAm
ancestry
chr3_102235094_102236772
local: beta=−0.22,se=0.03,t=−6.37,var=0.11
global: beta=−0.18,se=0.13,t=−1.35,var=0.01
DNAm
ancestry

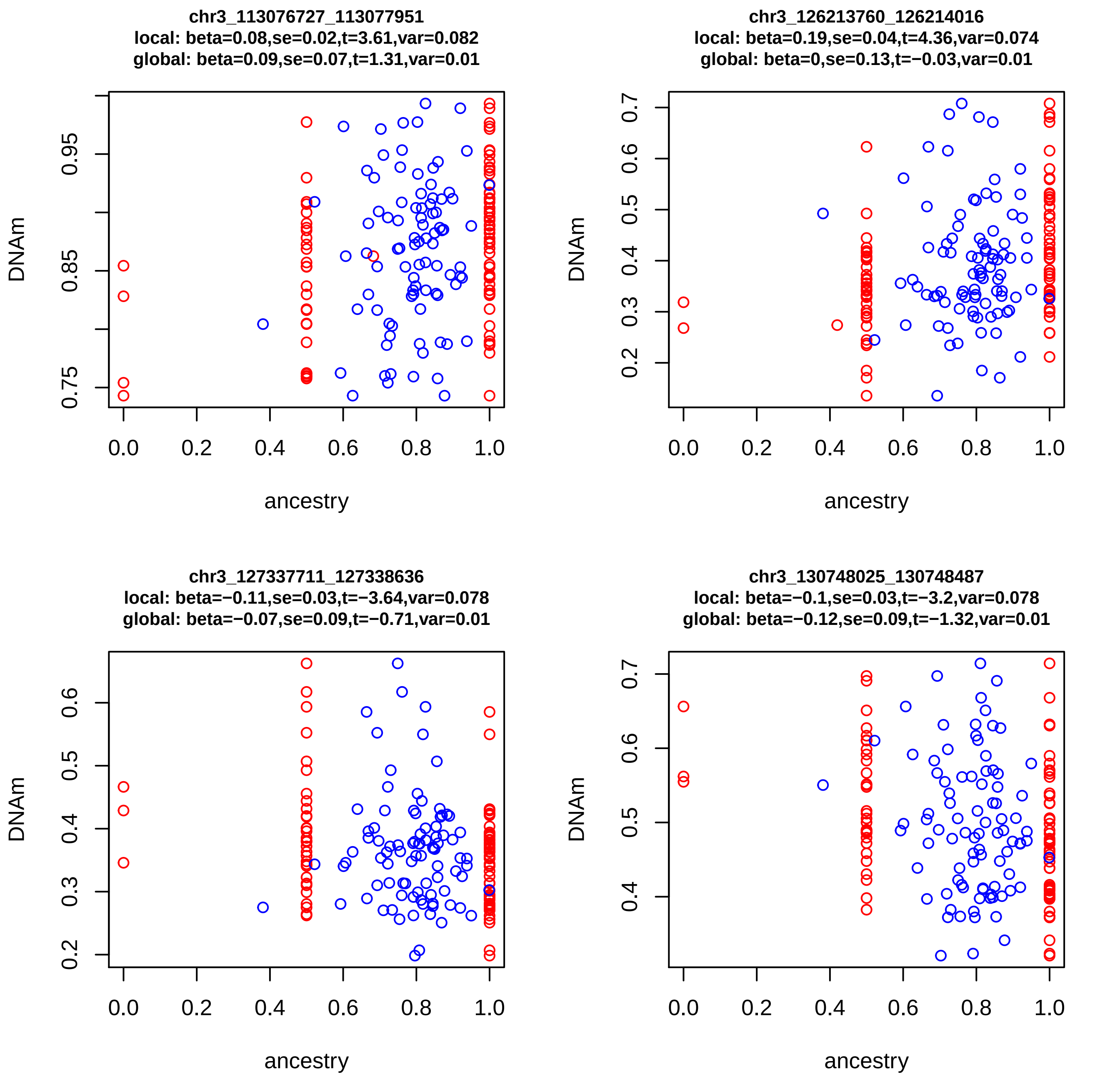
chr3_113076727_113077951
local: beta=0.08,se=0.02,t=3.61,var=0.082
global: beta=0.09,se=0.07,t=1.31,var=0.01
DNAm
ancestry
chr3_126213760_126214016
local: beta=0.19,se=0.04,t=4.36,var=0.074
global: beta=0,se=0.13,t=−0.03,var=0.01
DNAm
ancestry
chr3_127337711_127338636
local: beta=−0.11,se=0.03,t=−3.64,var=0.078
global: beta=−0.07,se=0.09,t=−0.71,var=0.01
DNAm
ancestry
chr3_130748025_130748487
local: beta=−0.1,se=0.03,t=−3.2,var=0.078
global: beta=−0.12,se=0.09,t=−1.32,var=0.01
DNAm
ancestry

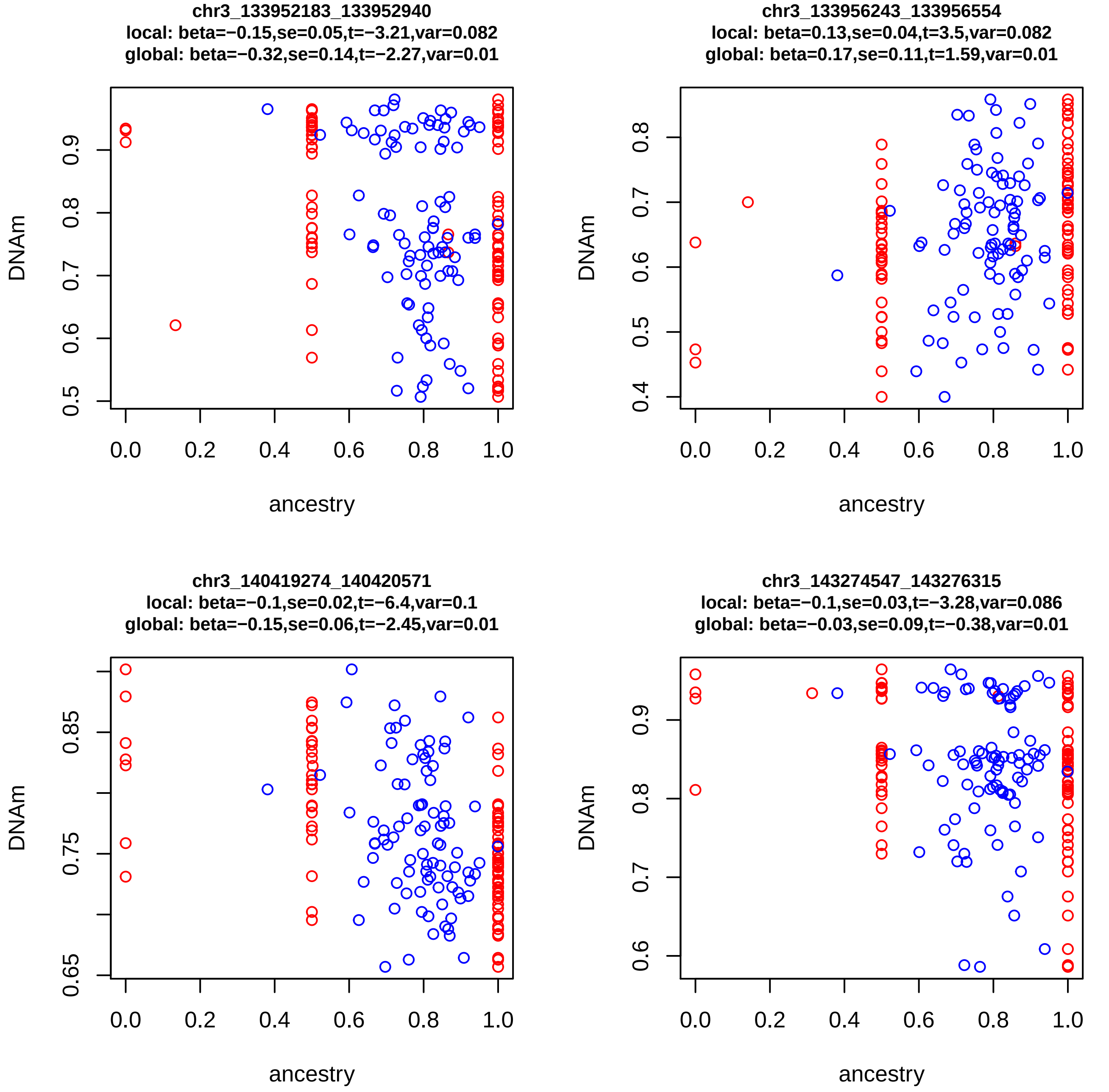

chr3_133952183_133952940
local: beta=−0.15,se=0.05,t=−3.21,var=0.082
global: beta=−0.32,se=0.14,t=−2.27,var=0.01
DNAm
ancestry
chr3_133956243_133956554
local: beta=0.13,se=0.04,t=3.5,var=0.082
global: beta=0.17,se=0.11,t=1.59,var=0.01
DNAm
ancestry
chr3_140419274_140420571
local: beta=−0.1,se=0.02,t=−6.4,var=0.1
global: beta=−0.15,se=0.06,t=−2.45,var=0.01
DNAm
ancestry
chr3_143274547_143276315
local: beta=−0.1,se=0.03,t=−3.28,var=0.086
global: beta=−0.03,se=0.09,t=−0.38,var=0.01
DNAm
ancestry

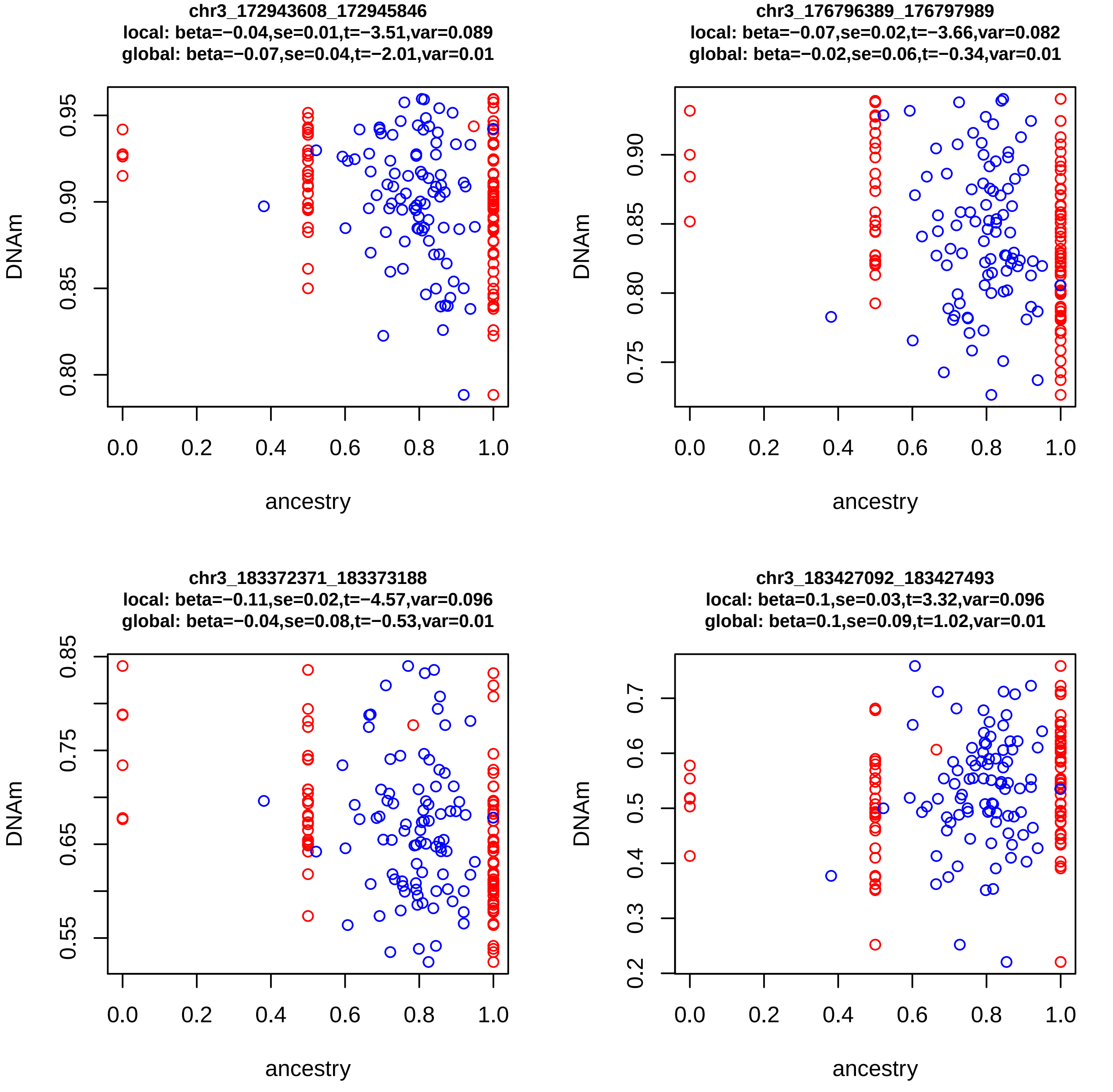
chr3_172943608_172945846
local: beta=−0.04,se=0.01,t=−3.51,var=0.089
global: beta=−0.07,se=0.04,t=−2.01,var=0.01
DNAm
0.80
0.85
0.90
0.95
0.0
0.2
0.4
0.6
0.8
1.0
ancestry
chr3_176796389_176797989
local: beta=−0.07,se=0.02,t=−3.66,var=0.082
global: beta=−0.02,se=0.06,t=−0.34,var=0.01
DNAm
0.75
0.80
0.85
0.90
0.0
0.2
0.4
0.6
0.8
1.0
ancestry
chr3_183372371_183373188
local: beta=−0.11,se=0.02,t=−4.57,var=0.096
global: beta=−0.04,se=0.08,t=−0.53,var=0.01
DNAm
0.55
0.65
0.75
0.85
0.0
0.2
0.4
0.6
0.8
1.0
ancestry
chr3_183427092_183427493
local: beta=0.1,se=0.03,t=3.32,var=0.096
global: beta=0.1,se=0.09,t=1.02,var=0.01
DNAm
0.2
0.3
0.4
0.5
0.6
0.7
0.0
0.2
0.4
0.6
0.8
1.0
ancestry

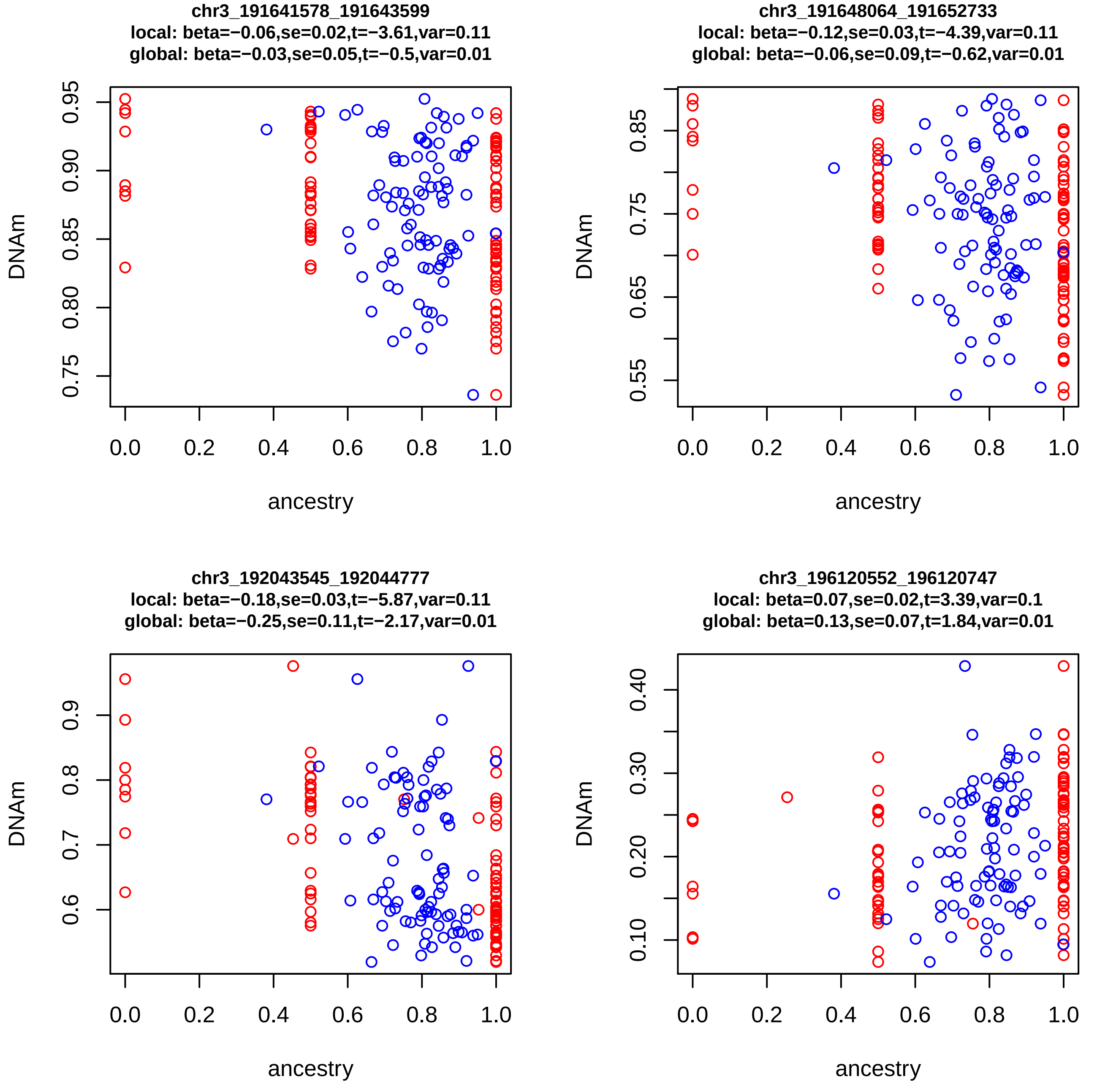
chr3_191641578_191643599
local: beta=−0.06,se=0.02,t=−3.61,var=0.11
global: beta=−0.03,se=0.05,t=−0.5,var=0.01
DNAm
0.75
0.80
0.85
0.90
0.95
0.0
0.2
0.4
0.6
0.8
1.0
ancestry
chr3_191648064_191652733
local: beta=−0.12,se=0.03,t=−4.39,var=0.11
global: beta=−0.06,se=0.09,t=−0.62,var=0.01
DNAm
0.55
0.65
0.75
0.85
0.0
0.2
0.4
0.6
0.8
1.0
ancestry
chr3_192043545_192044777
local: beta=−0.18,se=0.03,t=−5.87,var=0.11
global: beta=−0.25,se=0.11,t=−2.17,var=0.01
DNAm
0.6
0.7
0.8
0.9
0.0
0.2
0.4
0.6
0.8
1.0
ancestry
chr3_196120552_196120747
local: beta=0.07,se=0.02,t=3.39,var=0.1
global: beta=0.13,se=0.07,t=1.84,var=0.01
DNAm
0.10
0.20
0.30
0.40
0.0
0.2
0.4
0.6
0.8
1.0
ancestry

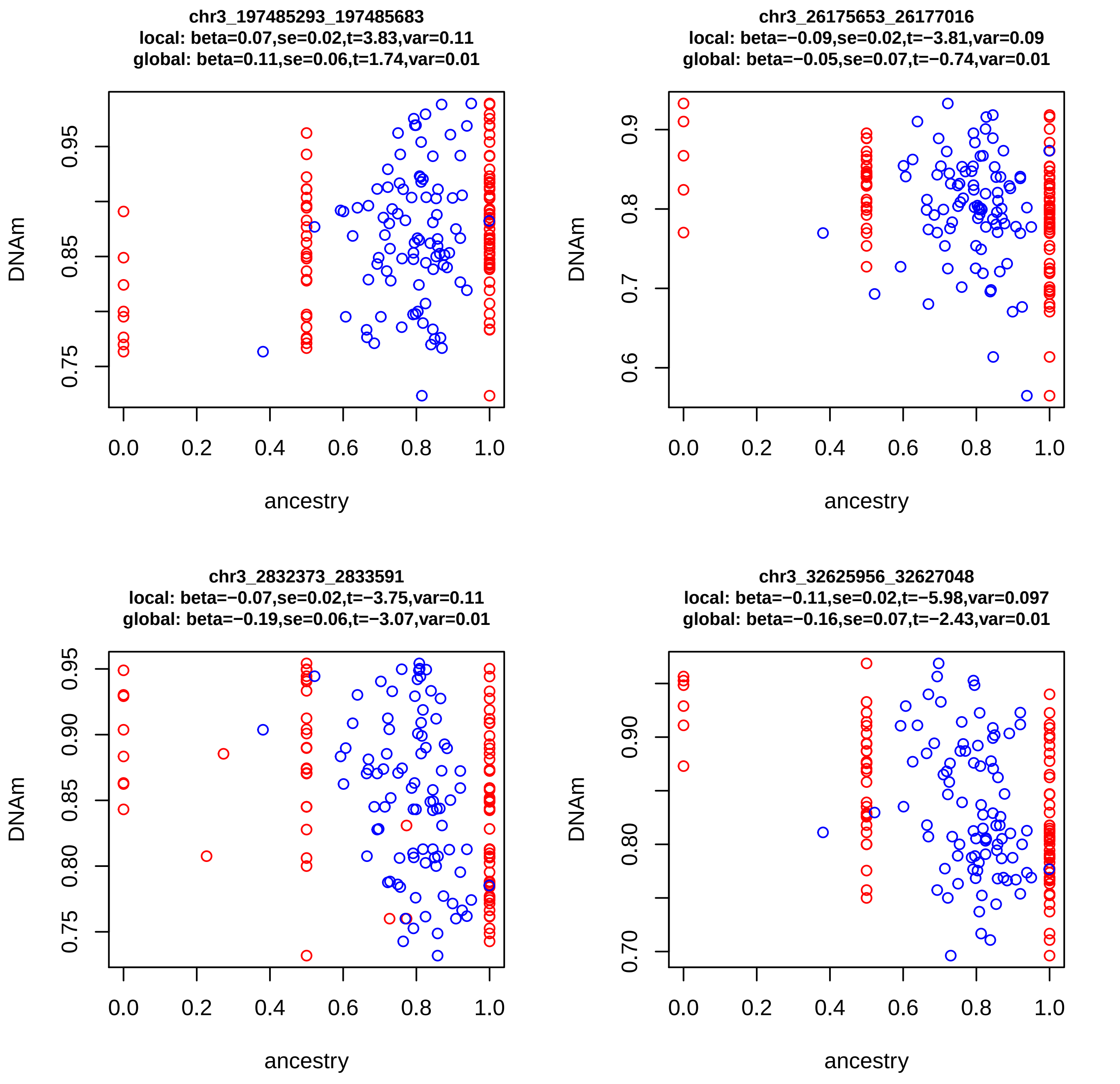
chr3_197485293_197485683
local: beta=0.07,se=0.02,t=3.83,var=0.11
global: beta=0.11,se=0.06,t=1.74,var=0.01
DNAm
ancestry
chr3_26175653_26177016
local: beta=−0.09,se=0.02,t=−3.81,var=0.09
global: beta=−0.05,se=0.07,t=−0.74,var=0.01
DNAm
ancestry
chr3_2832373_2833591
local: beta=−0.07,se=0.02,t=−3.75,var=0.11
global: beta=−0.19,se=0.06,t=−3.07,var=0.01
DNAm
ancestry
chr3_32625956_32627048
local: beta=−0.11,se=0.02,t=−5.98,var=0.097
global: beta=−0.16,se=0.07,t=−2.43,var=0.01
DNAm
ancestry

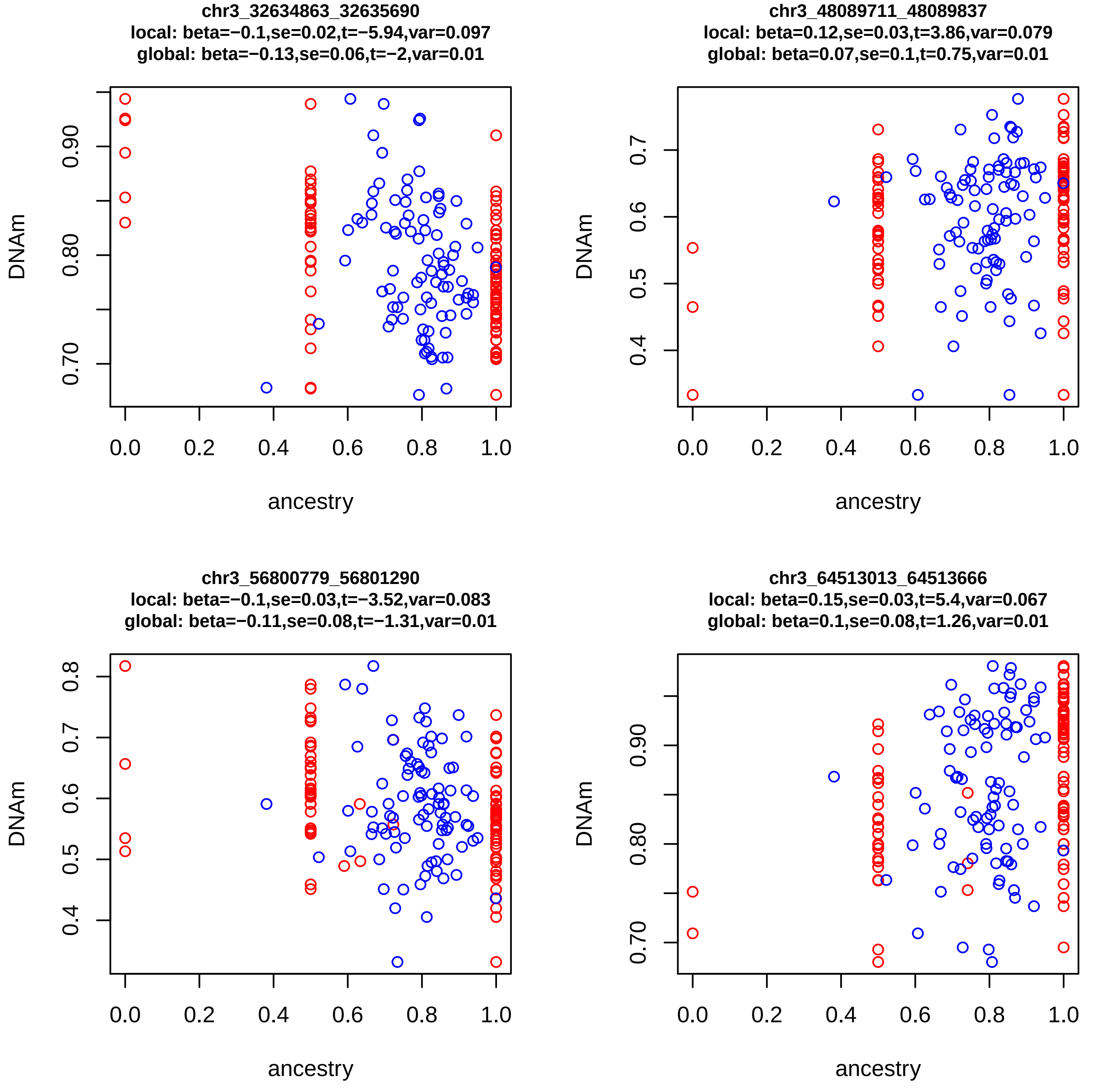
chr3_32634863_32635690
local: beta=−0.1,se=0.02,t=−5.94,var=0.097
global: beta=−0.13,se=0.06,t=−2,var=0.01
DNAm
0.70
0.80
0.90
ancestry
0.0
0.2
0.4
0.6
0.8
1.0
chr3_48089711_48089837
local: beta=0.12,se=0.03,t=3.86,var=0.079
global: beta=0.07,se=0.1,t=0.75,var=0.01
DNAm
0.4
0.5
0.6
0.7
ancestry
0.0
0.2
0.4
0.6
0.8
1.0
chr3_56800779_56801290
local: beta=−0.1,se=0.03,t=−3.52,var=0.083
global: beta=−0.11,se=0.08,t=−1.31,var=0.01
DNAm
0.4
0.5
0.6
0.7
0.8
ancestry
0.0
0.2
0.4
0.6
0.8
1.0
chr3_64513013_64513666
local: beta=0.15,se=0.03,t=5.4,var=0.067
global: beta=0.1,se=0.08,t=1.26,var=0.01
DNAm
0.70
0.80
0.90
ancestry
0.0
0.2
0.4
0.6
0.8
1.0

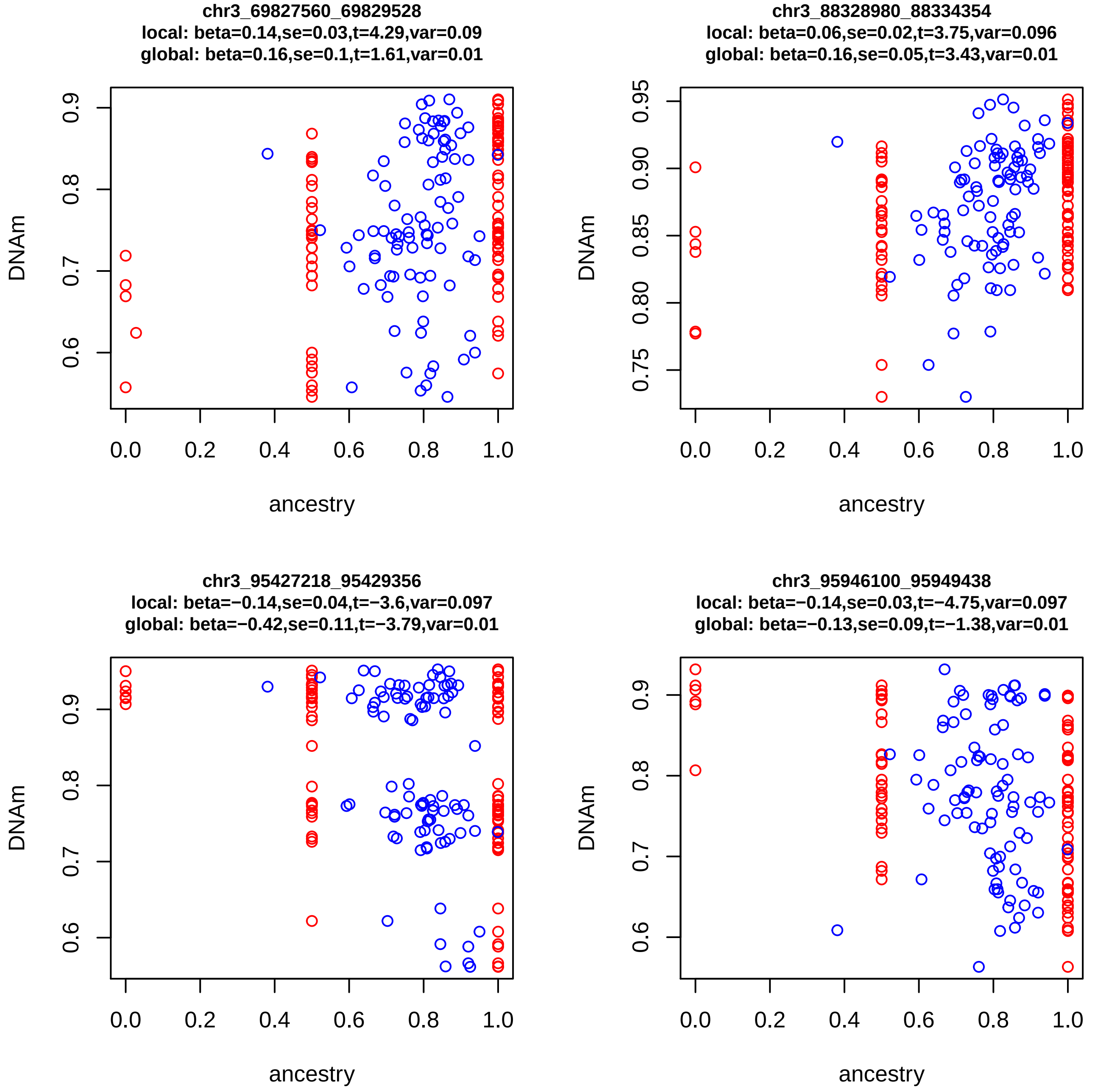
chr3_69827560_69829528
local: beta=0.14,se=0.03,t=4.29,var=0.09
global: beta=0.16,se=0.1,t=1.61,var=0.01
DNAm
ancestry
chr3_88328980_88334354
local: beta=0.06,se=0.02,t=3.75,var=0.096
global: beta=0.16,se=0.05,t=3.43,var=0.01
DNAm
ancestry
chr3_95427218_95429356
local: beta=−0.14,se=0.04,t=−3.6,var=0.097
global: beta=−0.42,se=0.11,t=−3.79,var=0.01
DNAm
ancestry
chr3_95946100_95949438
local: beta=−0.14,se=0.03,t=−4.75,var=0.097
global: beta=−0.13,se=0.09,t=−1.38,var=0.01
DNAm
ancestry

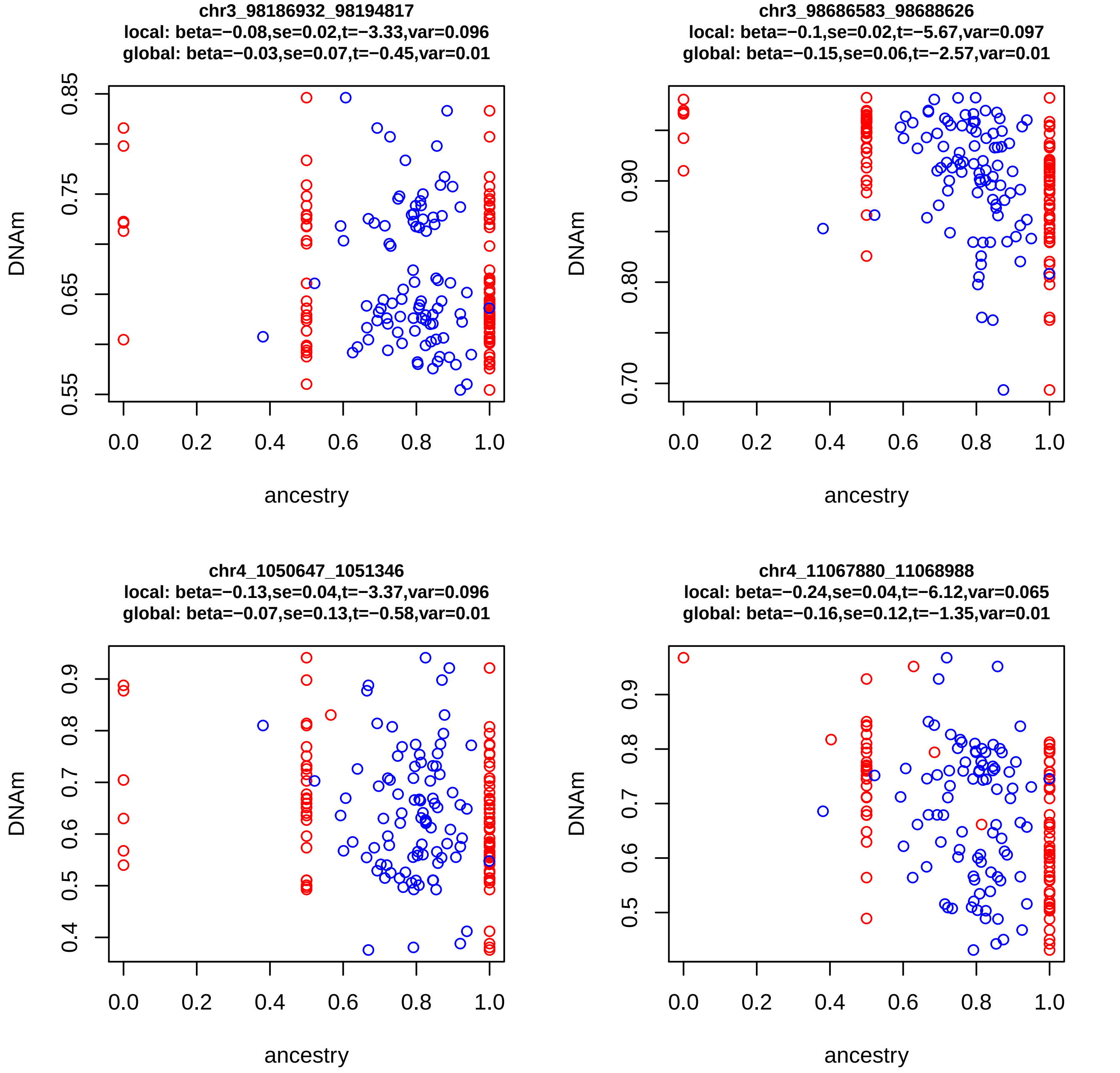
chr3_98186932_98194817
local: beta=−0.08,se=0.02,t=−3.33,var=0.096
global: beta=−0.03,se=0.07,t=−0.45,var=0.01
DNAm
0.55
0.65
0.75
0.85
ancestry
0.0
0.2
0.4
0.6
0.8
1.0
chr3_98686583_98688626
local: beta=−0.1,se=0.02,t=−5.67,var=0.097
global: beta=−0.15,se=0.06,t=−2.57,var=0.01
DNAm
0.70
0.80
0.90
ancestry
chr4_1050647_1051346
local: beta=−0.13,se=0.04,t=−3.37,var=0.096
global: beta=−0.07,se=0.13,t=−0.58,var=0.01
DNAm
0.4
0.5
0.6
0.7
0.8
0.9
ancestry
chr4_11067880_11068988
local: beta=−0.24,se=0.04,t=−6.12,var=0.065
global: beta=−0.16,se=0.12,t=−1.35,var=0.01
DNAm
0.5
0.6
0.7
0.8
0.9
ancestry

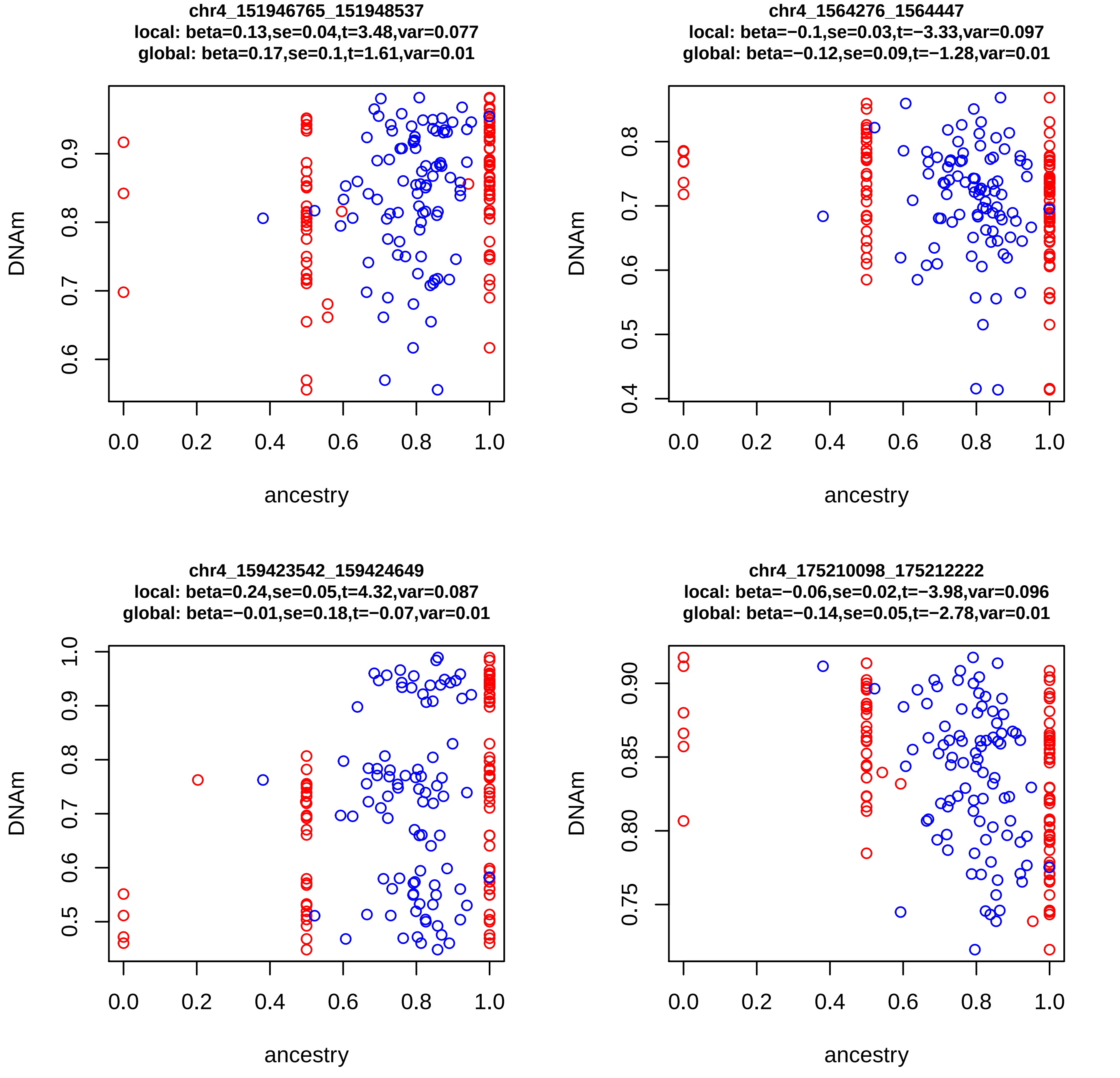
chr4_151946765_151948537
local: beta=0.13,se=0.04,t=3.48,var=0.077
global: beta=0.17,se=0.1,t=1.61,var=0.01
DNAm
ancestry
0.6
0.7
0.8
0.9
0.0
0.2
0.4
0.6
0.8
1.0
chr4_1564276_1564447
local: beta=−0.1,se=0.03,t=−3.33,var=0.097
global: beta=−0.12,se=0.09,t=−1.28,var=0.01
DNAm
ancestry
0.4
0.5
0.6
0.7
0.8
0.0
0.2
0.4
0.6
0.8
1.0
chr4_159423542_159424649
local: beta=0.24,se=0.05,t=4.32,var=0.087
global: beta=−0.01,se=0.18,t=−0.07,var=0.01
DNAm
ancestry
0.5
0.6
0.7
0.8
0.9
1.0
0.0
0.2
0.4
0.6
0.8
1.0
chr4_175210098_175212222
local: beta=−0.06,se=0.02,t=−3.98,var=0.096
global: beta=−0.14,se=0.05,t=−2.78,var=0.01
DNAm
ancestry
0.75
0.80
0.85
0.90
0.0
0.2
0.4
0.6
0.8
1.0

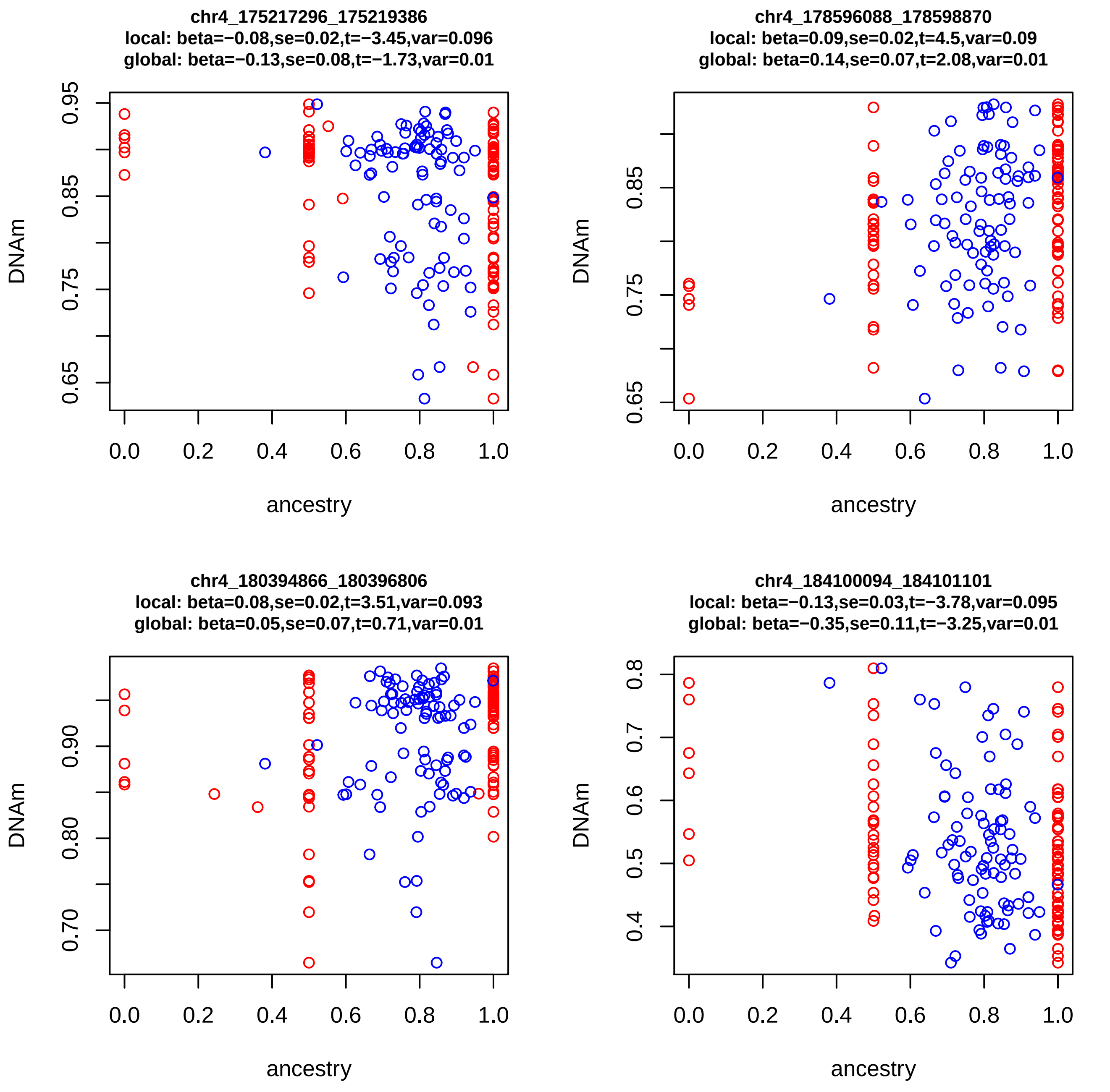
chr4_175217296_175219386
local: beta=−0.08,se=0.02,t=−3.45,var=0.096
global: beta=−0.13,se=0.08,t=−1.73,var=0.01
DNAm
ancestry
chr4_178596088_178598870
local: beta=0.09,se=0.02,t=4.5,var=0.09
global: beta=0.14,se=0.07,t=2.08,var=0.01
DNAm
ancestry
chr4_180394866_180396806
local: beta=0.08,se=0.02,t=3.51,var=0.093
global: beta=0.05,se=0.07,t=0.71,var=0.01
DNAm
ancestry
chr4_184100094_184101101
local: beta=−0.13,se=0.03,t=−3.78,var=0.095
global: beta=−0.35,se=0.11,t=−3.25,var=0.01
DNAm
ancestry

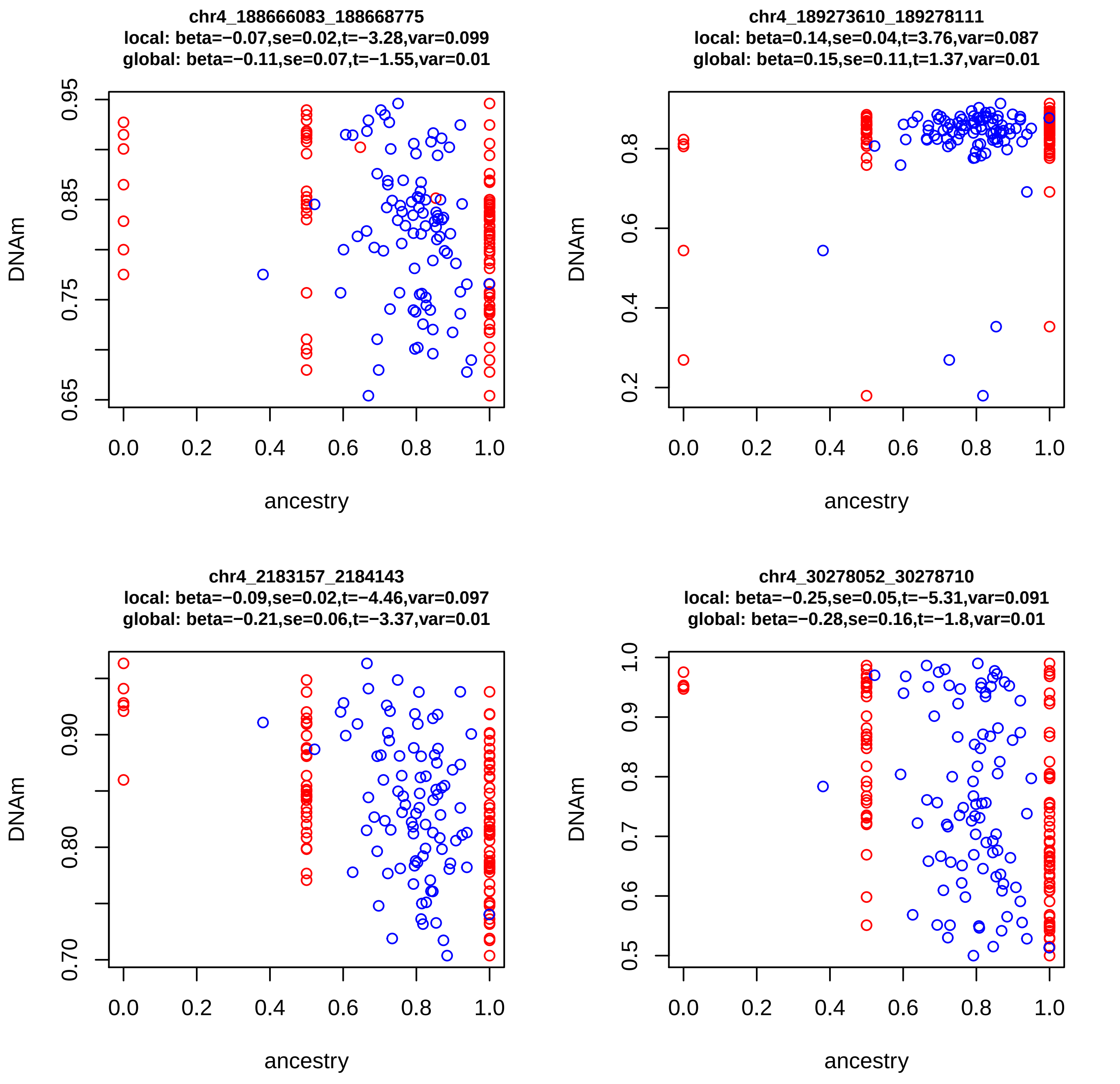
chr4_188666083_188668775
local: beta=−0.07,se=0.02,t=−3.28,var=0.099
global: beta=−0.11,se=0.07,t=−1.55,var=0.01
DNAm
0.65
0.75
0.85
0.95
0.0
0.2
0.4
0.6
0.8
1.0
ancestry
chr4_189273610_189278111
local: beta=0.14,se=0.04,t=3.76,var=0.087
global: beta=0.15,se=0.11,t=1.37,var=0.01
DNAm
0.2
0.4
0.6
0.8
0.0
0.2
0.4
0.6
0.8
1.0
ancestry
chr4_2183157_2184143
local: beta=−0.09,se=0.02,t=−4.46,var=0.097
global: beta=−0.21,se=0.06,t=−3.37,var=0.01
DNAm
0.70
0.80
0.90
0.0
0.2
0.4
0.6
0.8
1.0
ancestry
chr4_30278052_30278710
local: beta=−0.25,se=0.05,t=−5.31,var=0.091
global: beta=−0.28,se=0.16,t=−1.8,var=0.01
DNAm
0.5
0.6
0.7
0.8
0.9
1.0
0.0
0.2
0.4
0.6
0.8
1.0
ancestry

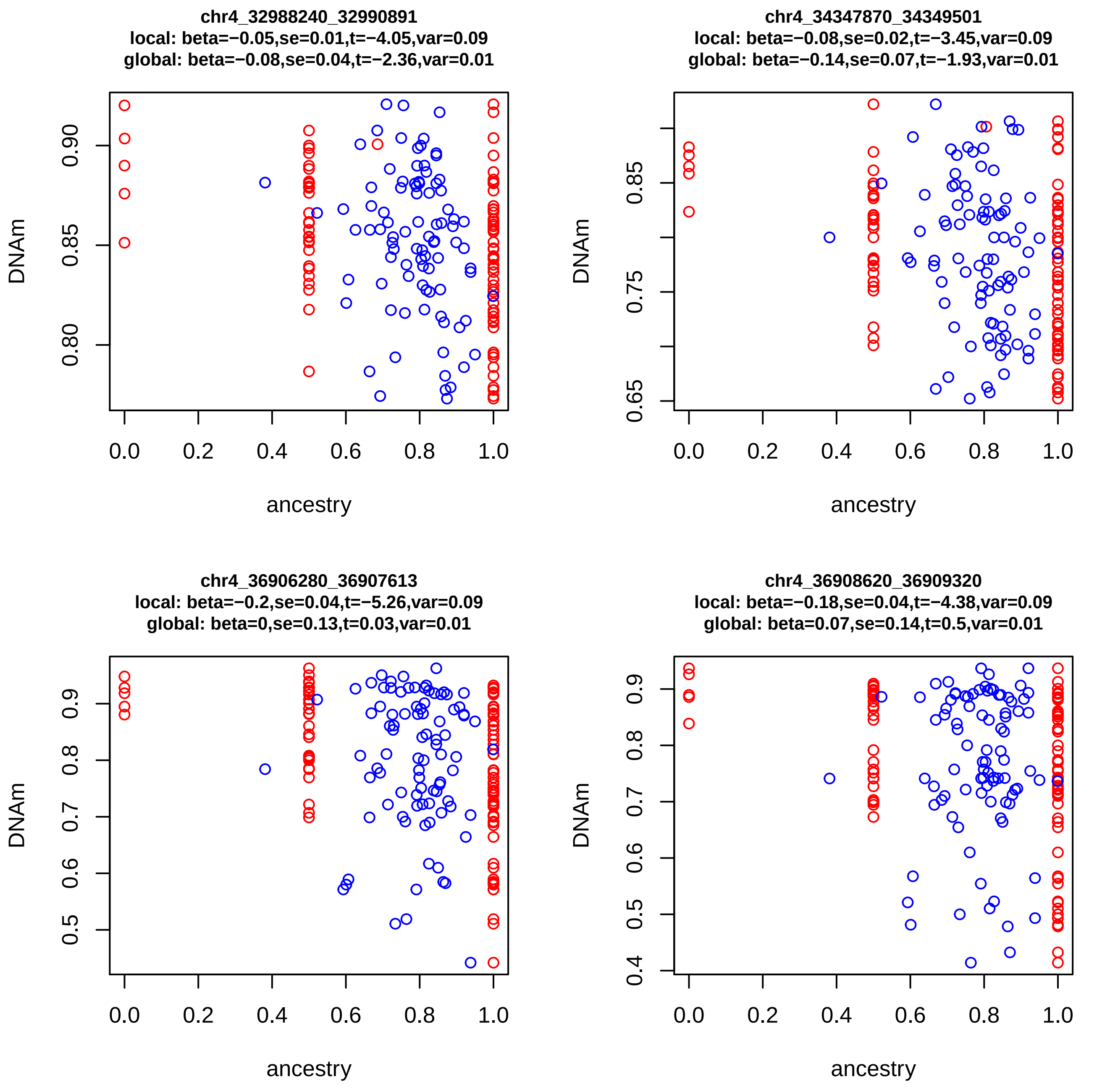
chr4_32988240_32990891
local: beta=−0.05,se=0.01,t=−4.05,var=0.09
global: beta=−0.08,se=0.04,t=−2.36,var=0.01
DNAm
0.80
0.85
0.90
0.0
0.2
0.4
0.6
0.8
1.0
ancestry
chr4_34347870_34349501
local: beta=−0.08,se=0.02,t=−3.45,var=0.09
global: beta=−0.14,se=0.07,t=−1.93,var=0.01
DNAm
0.65
0.75
0.85
0.0
0.2
0.4
0.6
0.8
1.0
ancestry
chr4_36906280_36907613
local: beta=−0.2,se=0.04,t=−5.26,var=0.09
global: beta=0,se=0.13,t=0.03,var=0.01
DNAm
0.5
0.6
0.7
0.8
0.9
0.0
0.2
0.4
0.6
0.8
1.0
ancestry
chr4_36908620_36909320
local: beta=−0.18,se=0.04,t=−4.38,var=0.09
global: beta=0.07,se=0.14,t=0.5,var=0.01
DNAm
0.4
0.5
0.6
0.7
0.8
0.9
0.0
0.2
0.4
0.6
0.8
1.0
ancestry

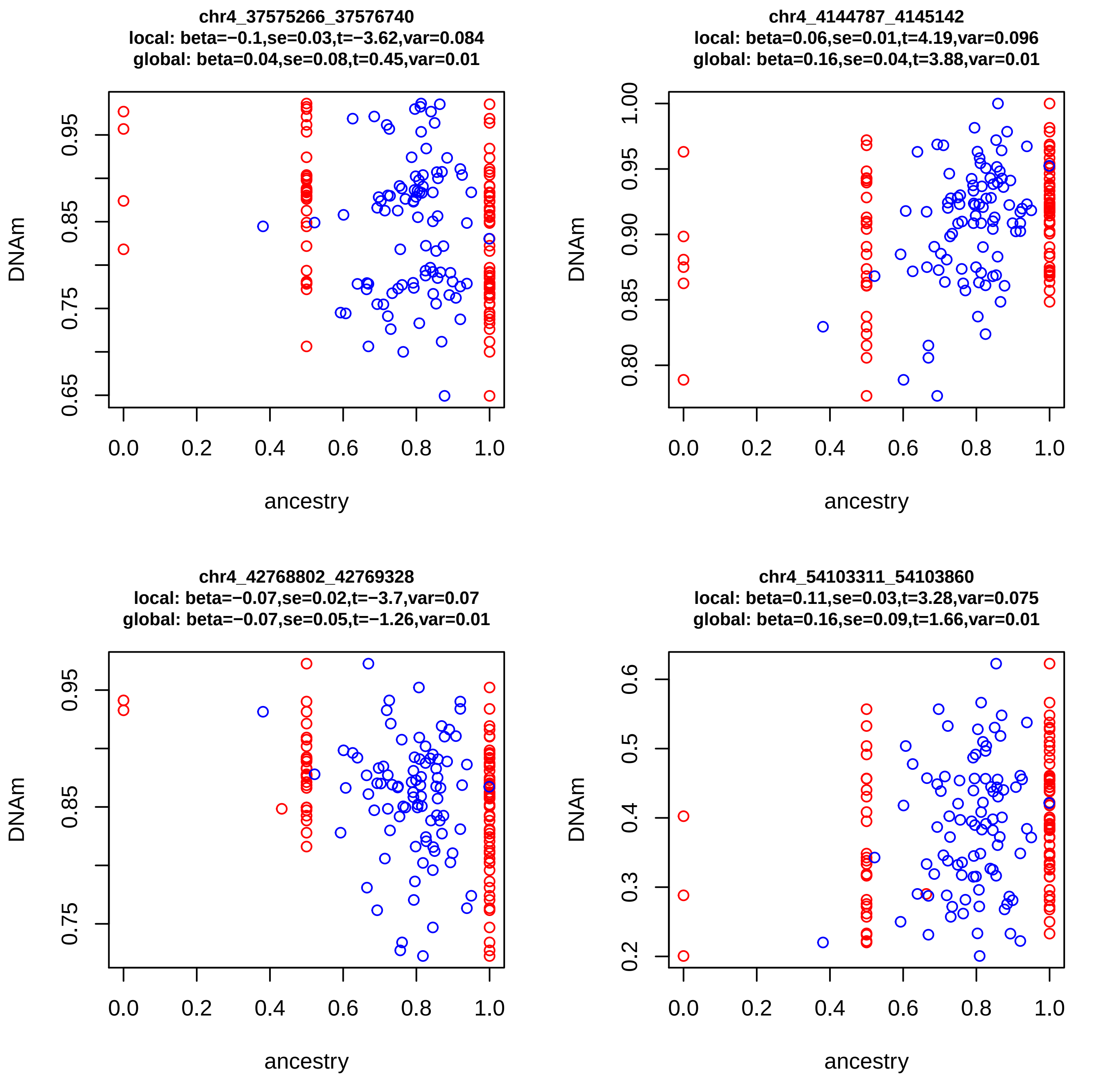
chr4_37575266_37576740
local: beta=−0.1,se=0.03,t=−3.62,var=0.084
global: beta=0.04,se=0.08,t=0.45,var=0.01
DNAm
0.65
0.75
0.85
0.95
ancestry
0.0
0.2
0.4
0.6
0.8
1.0
chr4_4144787_4145142
local: beta=0.06,se=0.01,t=4.19,var=0.096
global: beta=0.16,se=0.04,t=3.88,var=0.01
DNAm
0.80
0.85
0.90
0.95
1.00
ancestry
0.0
0.2
0.4
0.6
0.8
1.0
chr4_42768802_42769328
local: beta=−0.07,se=0.02,t=−3.7,var=0.07
global: beta=−0.07,se=0.05,t=−1.26,var=0.01
DNAm
0.75
0.85
0.95
ancestry
0.0
0.2
0.4
0.6
0.8
1.0
chr4_54103311_54103860
local: beta=0.11,se=0.03,t=3.28,var=0.075
global: beta=0.16,se=0.09,t=1.66,var=0.01
DNAm
0.2
0.3
0.4
0.5
0.6
ancestry
0.0
0.2
0.4
0.6
0.8
1.0

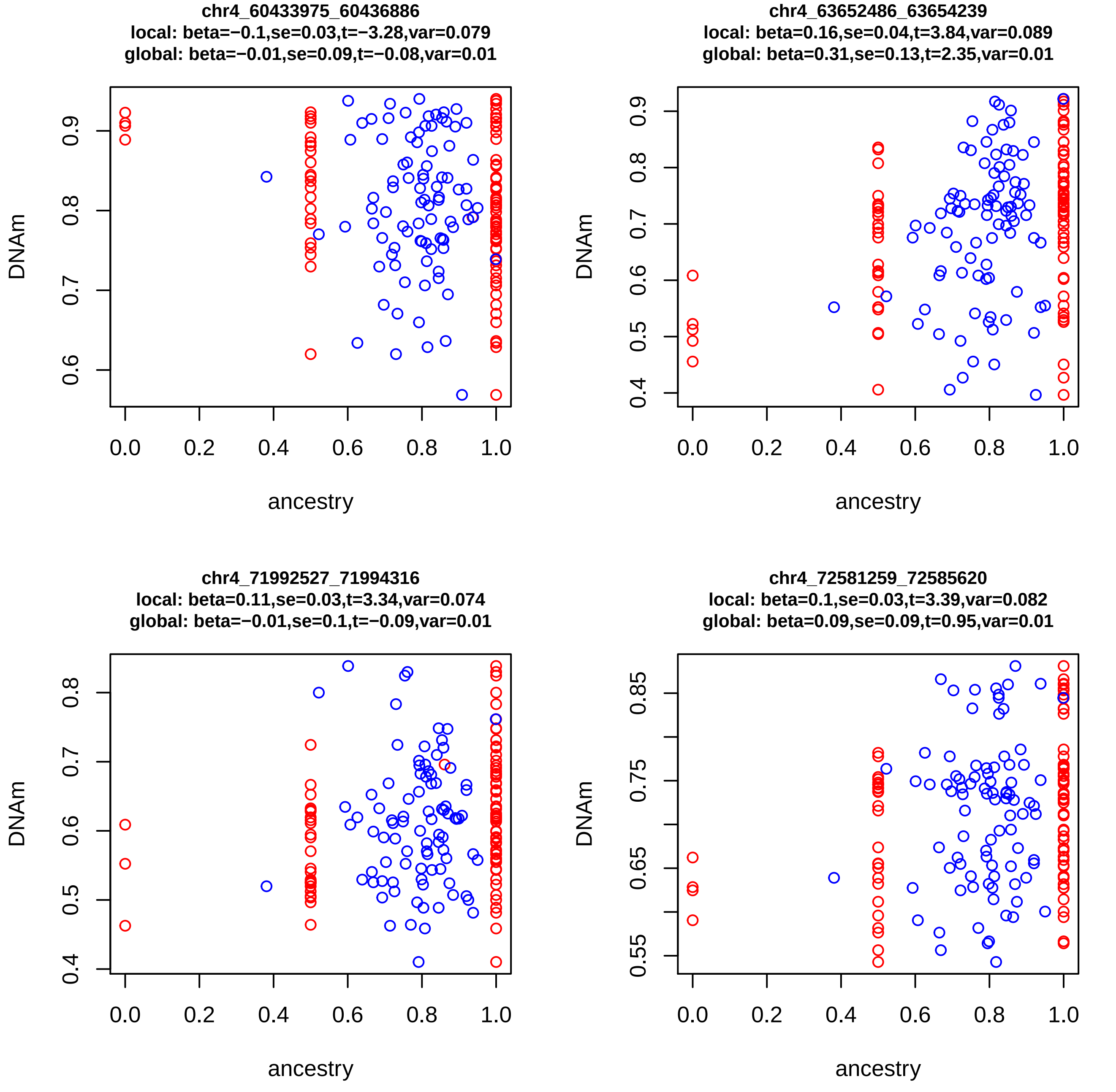
chr4_60433975_60436886
local: beta=−0.1,se=0.03,t=−3.28,var=0.079
global: beta=−0.01,se=0.09,t=−0.08,var=0.01
DNAm
ancestry
chr4_63652486_63654239
local: beta=0.16,se=0.04,t=3.84,var=0.089
global: beta=0.31,se=0.13,t=2.35,var=0.01
DNAm
ancestry
chr4_71992527_71994316
local: beta=0.11,se=0.03,t=3.34,var=0.074
global: beta=−0.01,se=0.1,t=−0.09,var=0.01
DNAm
ancestry
chr4_72581259_72585620
local: beta=0.1,se=0.03,t=3.39,var=0.082
global: beta=0.09,se=0.09,t=0.95,var=0.01
DNAm
ancestry

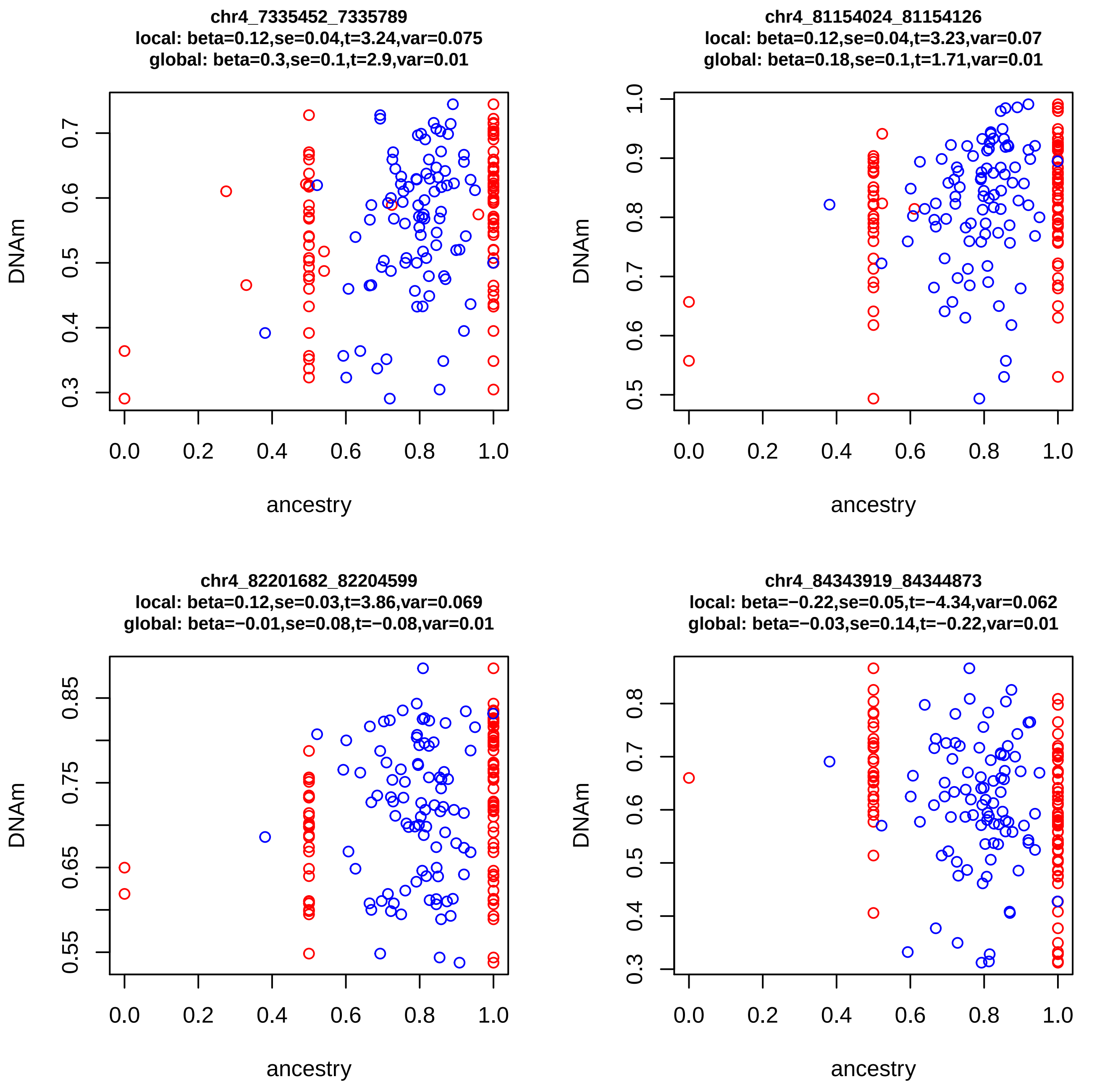
chr4_7335452_7335789
local: beta=0.12,se=0.04,t=3.24,var=0.075
global: beta=0.3,se=0.1,t=2.9,var=0.01
chr4_81154024_81154126
local: beta=0.12,se=0.04,t=3.23,var=0.07
global: beta=0.18,se=0.1,t=1.71,var=0.01
chr4_82201682_82204599
local: beta=0.12,se=0.03,t=3.86,var=0.069
global: beta=−0.01,se=0.08,t=−0.08,var=0.01
chr4_84343919_84344873
local: beta=−0.22,se=0.05,t=−4.34,var=0.062
global: beta=−0.03,se=0.14,t=−0.22,var=0.01
DNAm
ancestry

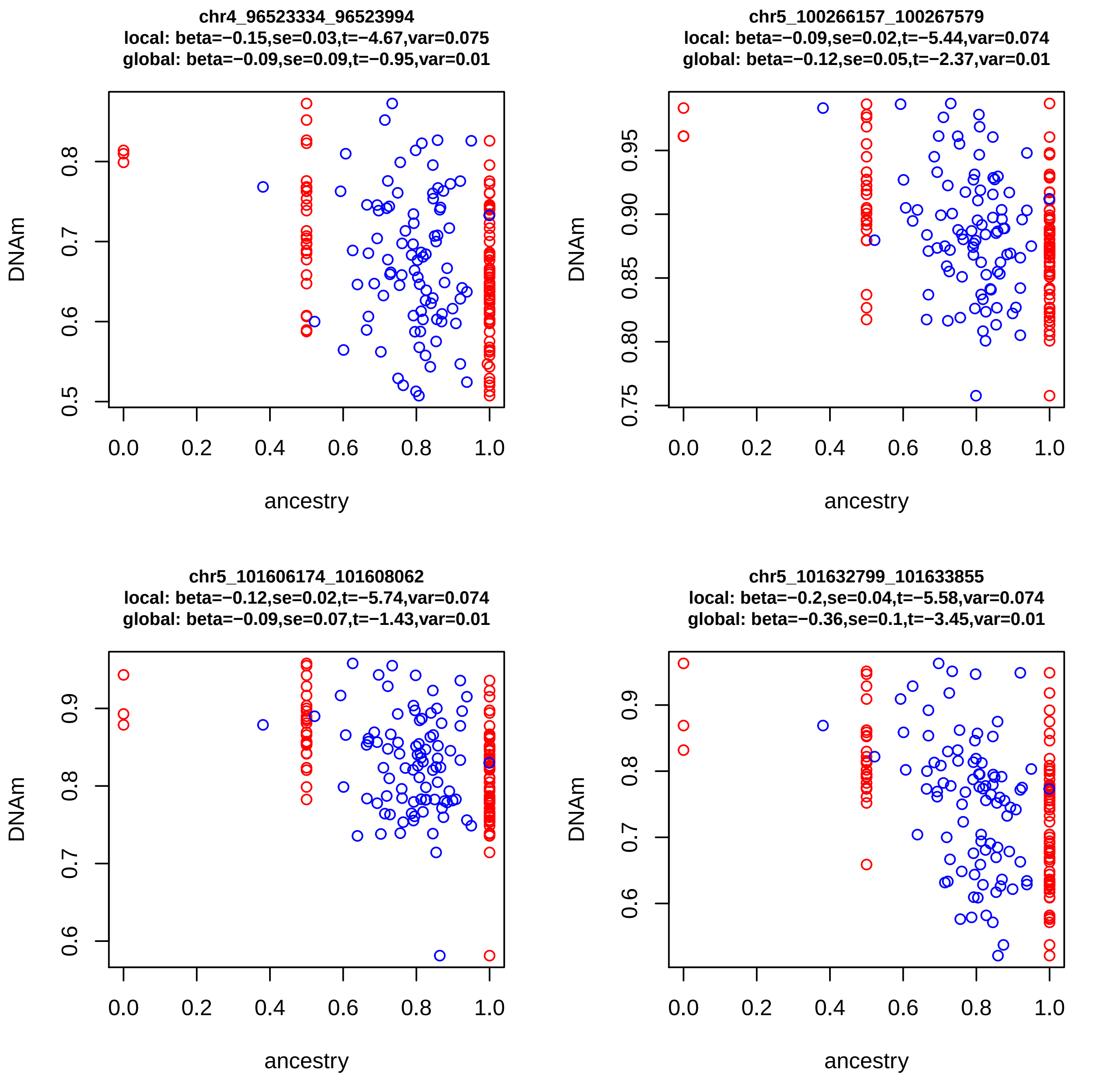
chr4_96523334_96523994
local: beta=−0.15,se=0.03,t=−4.67,var=0.075
global: beta=−0.09,se=0.09,t=−0.95,var=0.01
chr5_100266157_100267579
local: beta=−0.09,se=0.02,t=−5.44,var=0.074
global: beta=−0.12,se=0.05,t=−2.37,var=0.01
chr5_101606174_101608062
local: beta=−0.12,se=0.02,t=−5.74,var=0.074
global: beta=−0.09,se=0.07,t=−1.43,var=0.01
chr5_101632799_101633855
local: beta=−0.2,se=0.04,t=−5.58,var=0.074
global: beta=−0.36,se=0.1,t=−3.45,var=0.01
DNAm
ancestry

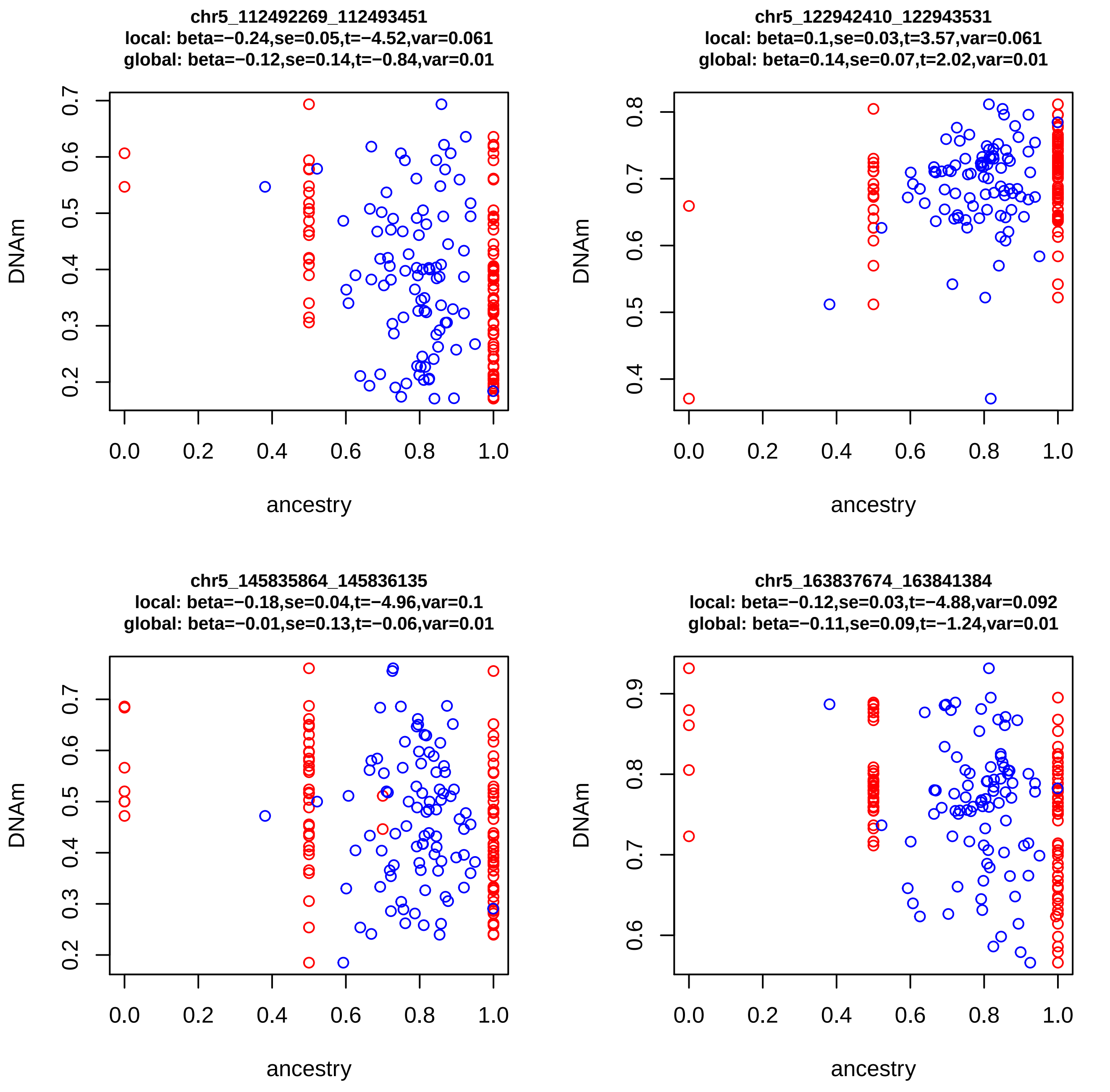
chr5_112492269_112493451
local: beta=−0.24,se=0.05,t=−4.52,var=0.061
global: beta=−0.12,se=0.14,t=−0.84,var=0.01
DNAm
ancestry
chr5_122942410_122943531
local: beta=0.1,se=0.03,t=3.57,var=0.061
global: beta=0.14,se=0.07,t=2.02,var=0.01
DNAm
ancestry
chr5_145835864_145836135
local: beta=−0.18,se=0.04,t=−4.96,var=0.1
global: beta=−0.01,se=0.13,t=−0.06,var=0.01
DNAm
ancestry
chr5_163837674_163841384
local: beta=−0.12,se=0.03,t=−4.88,var=0.092
global: beta=−0.11,se=0.09,t=−1.24,var=0.01
DNAm
ancestry

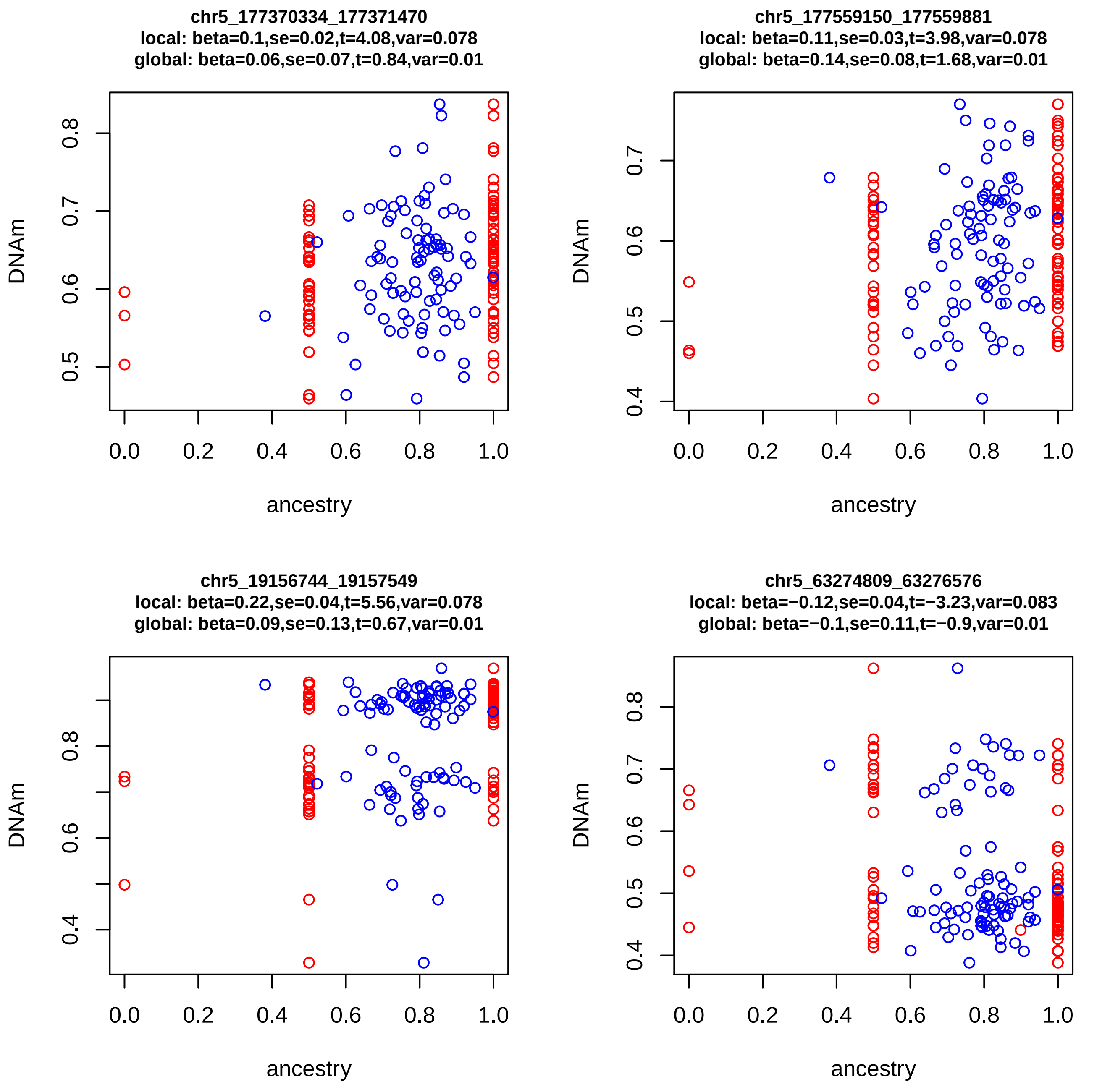
chr5_177370334_177371470
local: beta=0.1,se=0.02,t=4.08,var=0.078
global: beta=0.06,se=0.07,t=0.84,var=0.01
chr5_177559150_177559881
local: beta=0.11,se=0.03,t=3.98,var=0.078
global: beta=0.14,se=0.08,t=1.68,var=0.01
chr5_19156744_19157549
local: beta=0.22,se=0.04,t=5.56,var=0.078
global: beta=0.09,se=0.13,t=0.67,var=0.01
chr5_63274809_63276576
local: beta=−0.12,se=0.04,t=−3.23,var=0.083
global: beta=−0.1,se=0.11,t=−0.9,var=0.01
DNAm
ancestry
0.0
0.2
0.4
0.6
0.8
1.0

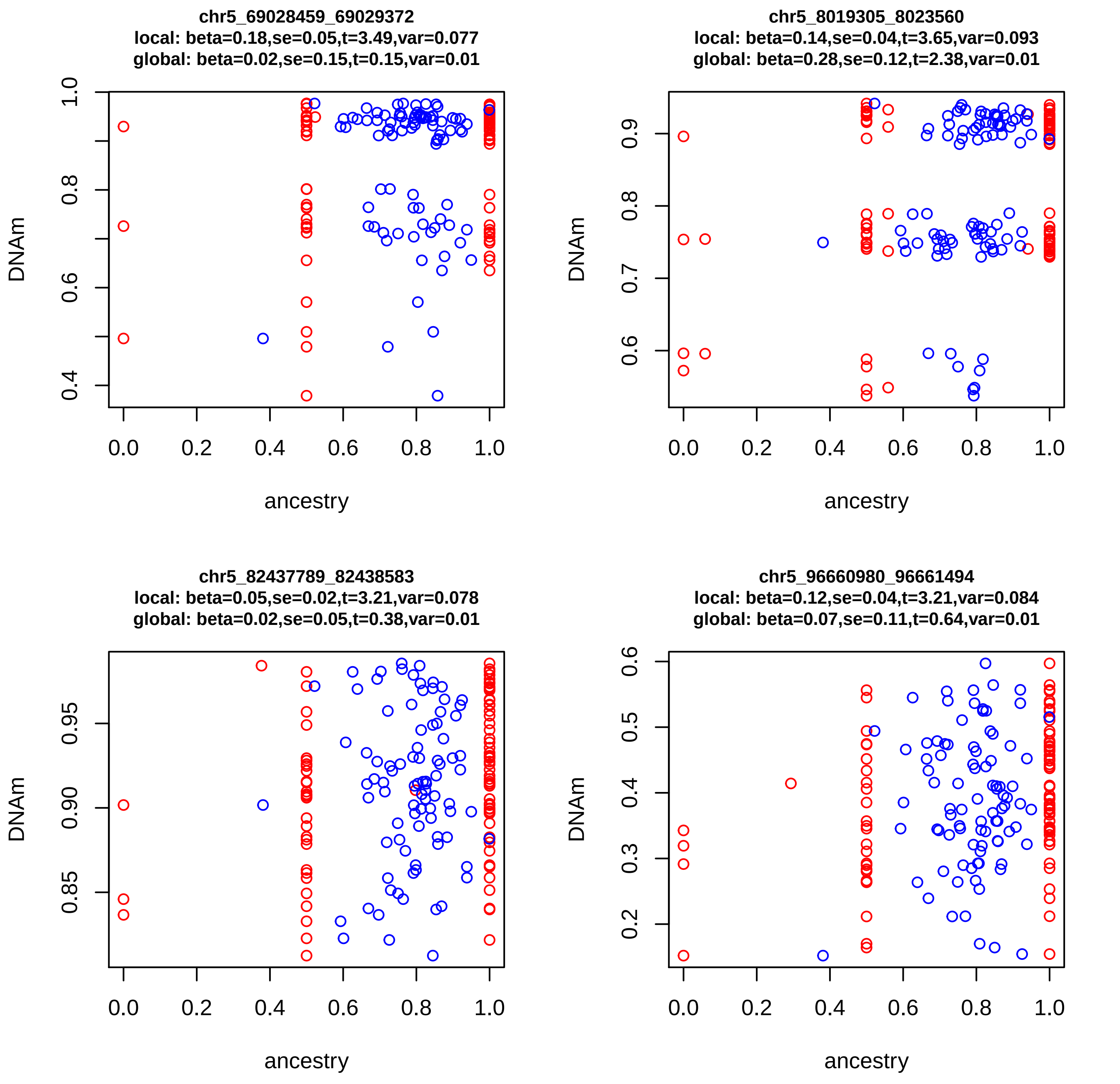
chr5_69028459_69029372
local: beta=0.18,se=0.05,t=3.49,var=0.077
global: beta=0.02,se=0.15,t=0.15,var=0.01
DNAm
ancestry
chr5_8019305_8023560
local: beta=0.14,se=0.04,t=3.65,var=0.093
global: beta=0.28,se=0.12,t=2.38,var=0.01
DNAm
ancestry
chr5_82437789_82438583
local: beta=0.05,se=0.02,t=3.21,var=0.078
global: beta=0.02,se=0.05,t=0.38,var=0.01
DNAm
ancestry
chr5_96660980_96661494
local: beta=0.12,se=0.04,t=3.21,var=0.084
global: beta=0.07,se=0.11,t=0.64,var=0.01
DNAm
ancestry

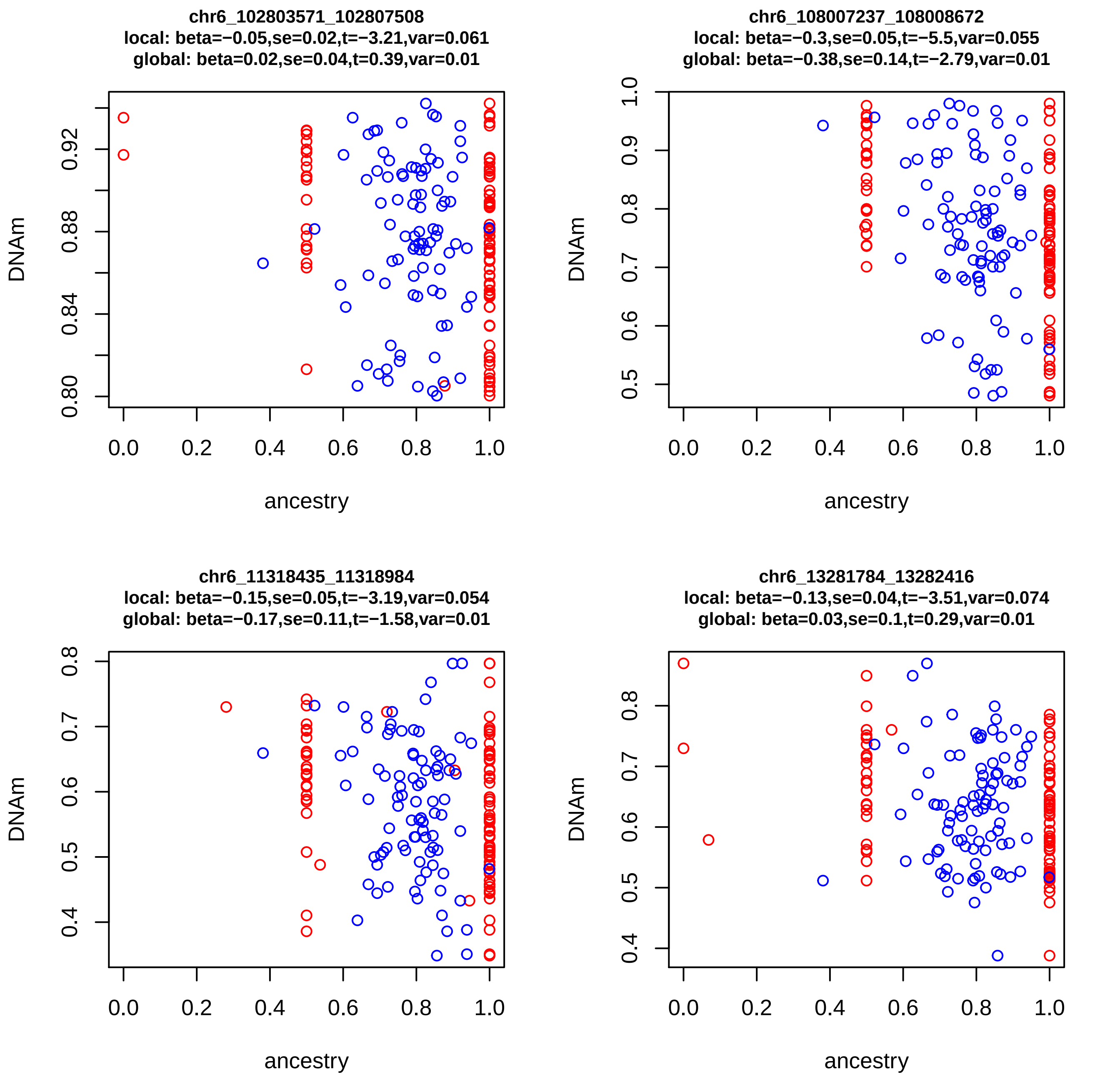
chr6_102803571_102807508
local: beta=−0.05,se=0.02,t=−3.21,var=0.061
global: beta=0.02,se=0.04,t=0.39,var=0.01
DNAm
ancestry
chr6_108007237_108008672
local: beta=−0.3,se=0.05,t=−5.5,var=0.055
global: beta=−0.38,se=0.14,t=−2.79,var=0.01
DNAm
ancestry
chr6_11318435_11318984
local: beta=−0.15,se=0.05,t=−3.19,var=0.054
global: beta=−0.17,se=0.11,t=−1.58,var=0.01
DNAm
ancestry
chr6_13281784_13282416
local: beta=−0.13,se=0.04,t=−3.51,var=0.074
global: beta=0.03,se=0.1,t=0.29,var=0.01
DNAm
ancestry

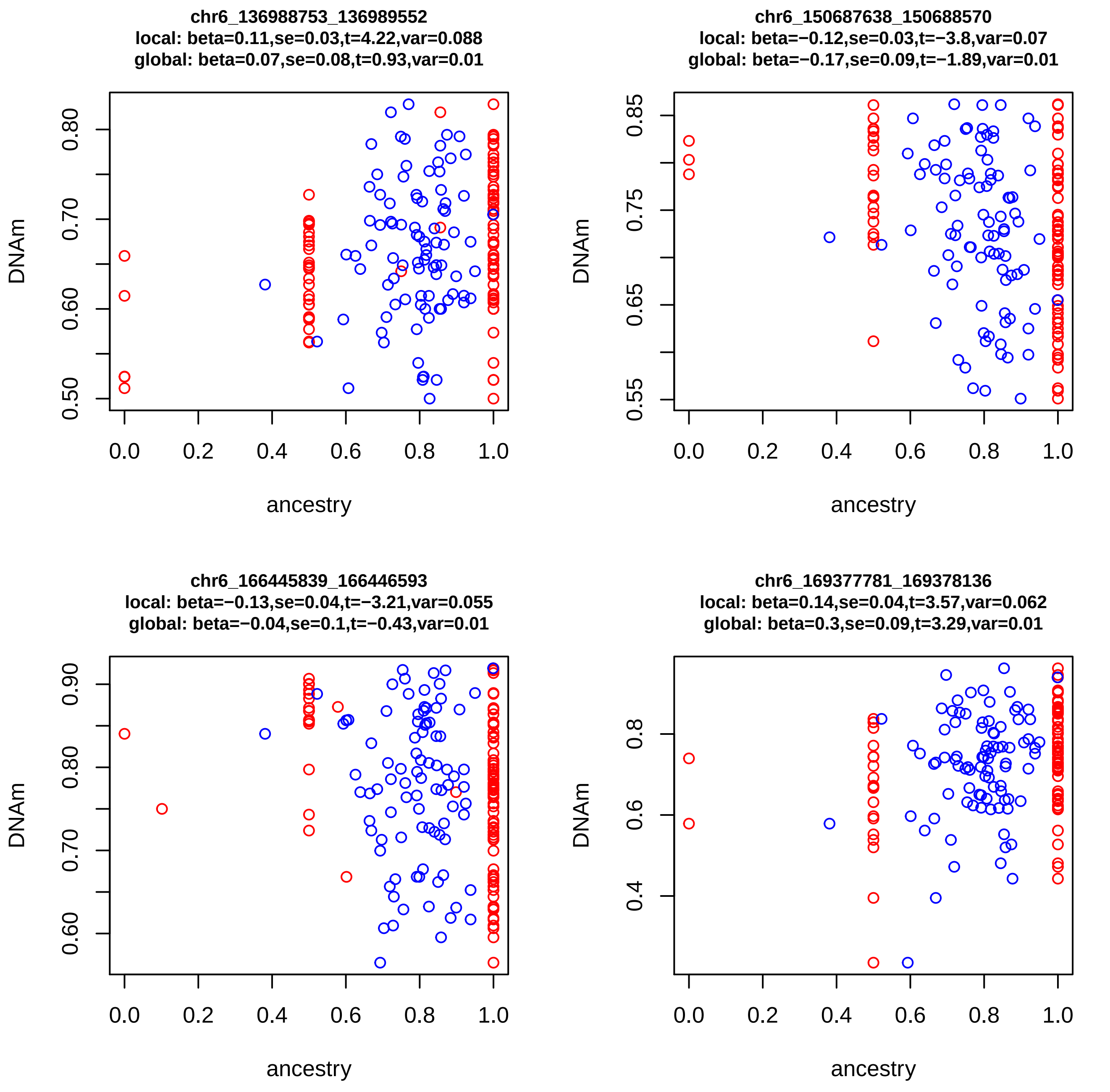
chr6_136988753_136989552
local: beta=0.11,se=0.03,t=4.22,var=0.088
global: beta=0.07,se=0.08,t=0.93,var=0.01
DNAm
ancestry
chr6_150687638_150688570
local: beta=−0.12,se=0.03,t=−3.8,var=0.07
global: beta=−0.17,se=0.09,t=−1.89,var=0.01
DNAm
ancestry
chr6_166445839_166446593
local: beta=−0.13,se=0.04,t=−3.21,var=0.055
global: beta=−0.04,se=0.1,t=−0.43,var=0.01
DNAm
ancestry
chr6_169377781_169378136
local: beta=0.14,se=0.04,t=3.57,var=0.062
global: beta=0.3,se=0.09,t=3.29,var=0.01
DNAm
ancestry

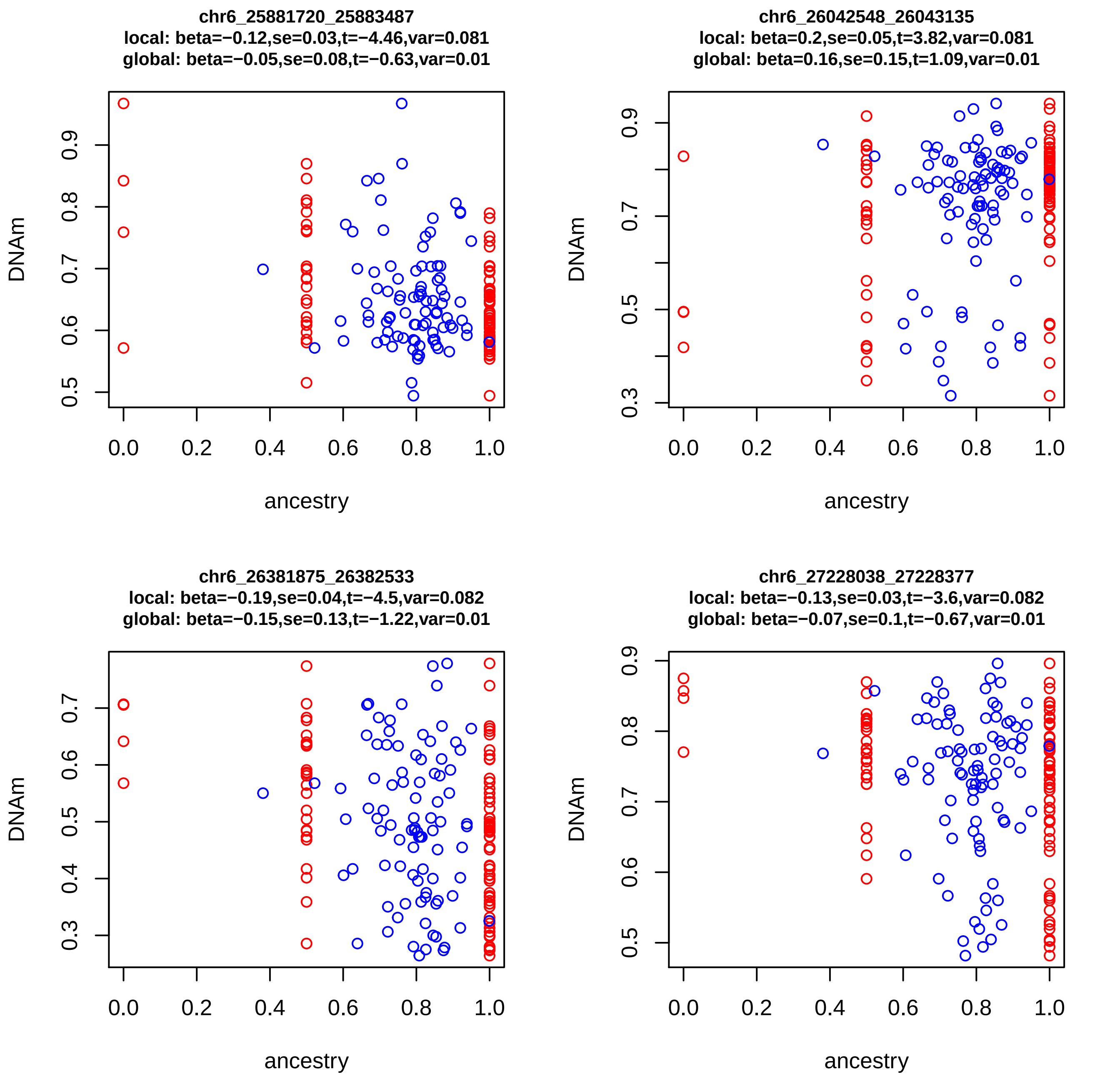
chr6_25881720_25883487
local: beta=−0.12,se=0.03,t=−4.46,var=0.081
global: beta=−0.05,se=0.08,t=−0.63,var=0.01
DNAm
ancestry
chr6_26042548_26043135
local: beta=0.2,se=0.05,t=3.82,var=0.081
global: beta=0.16,se=0.15,t=1.09,var=0.01
DNAm
ancestry
chr6_26381875_26382533
local: beta=−0.19,se=0.04,t=−4.5,var=0.082
global: beta=−0.15,se=0.13,t=−1.22,var=0.01
DNAm
ancestry
chr6_27228038_27228377
local: beta=−0.13,se=0.03,t=−3.6,var=0.082
global: beta=−0.07,se=0.1,t=−0.67,var=0.01
DNAm
ancestry

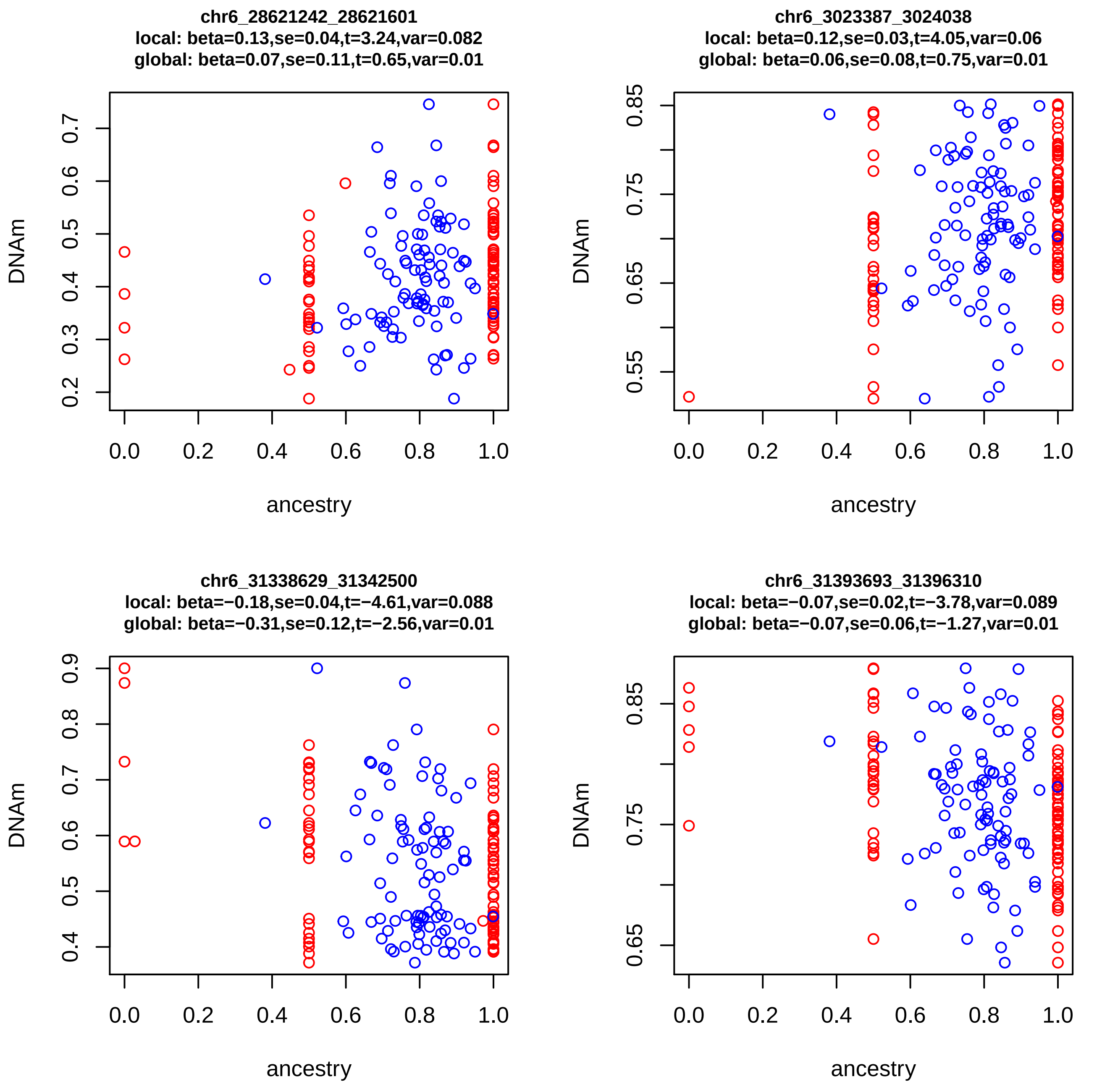
chr6_28621242_28621601
local: beta=0.13,se=0.04,t=3.24,var=0.082
global: beta=0.07,se=0.11,t=0.65,var=0.01
DNAm
ancestry
chr6_3023387_3024038
local: beta=0.12,se=0.03,t=4.05,var=0.06
global: beta=0.06,se=0.08,t=0.75,var=0.01
DNAm
ancestry
chr6_31338629_31342500
local: beta=−0.18,se=0.04,t=−4.61,var=0.088
global: beta=−0.31,se=0.12,t=−2.56,var=0.01
DNAm
ancestry
chr6_31393693_31396310
local: beta=−0.07,se=0.02,t=−3.78,var=0.089
global: beta=−0.07,se=0.06,t=−1.27,var=0.01
DNAm
ancestry

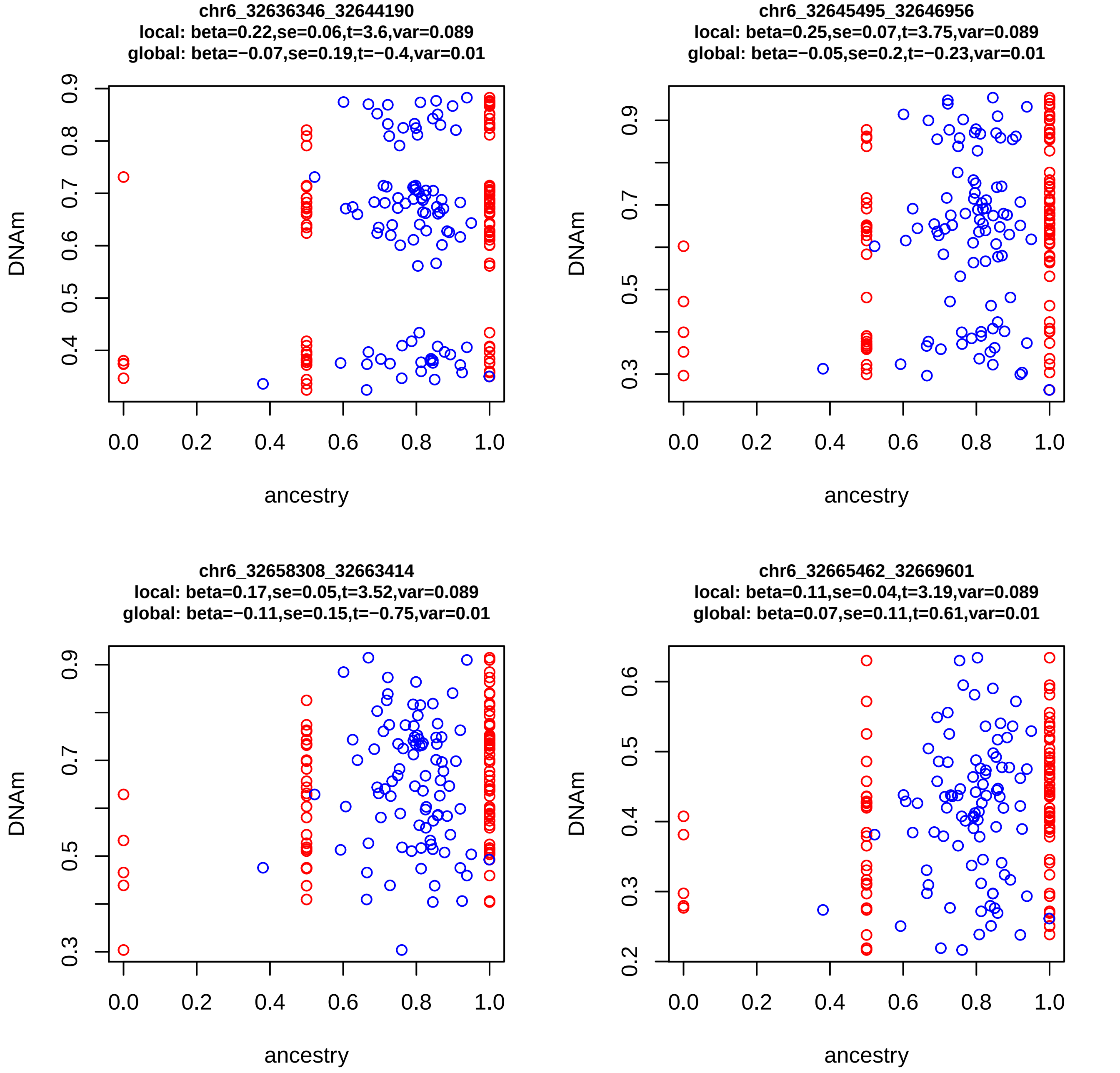
chr6_32636346_32644190
local: beta=0.22,se=0.06,t=3.6,var=0.089
global: beta=−0.07,se=0.19,t=−0.4,var=0.01
chr6_32645495_32646956
local: beta=0.25,se=0.07,t=3.75,var=0.089
global: beta=−0.05,se=0.2,t=−0.23,var=0.01
chr6_32658308_32663414
local: beta=0.17,se=0.05,t=3.52,var=0.089
global: beta=−0.11,se=0.15,t=−0.75,var=0.01
chr6_32665462_32669601
local: beta=0.11,se=0.04,t=3.19,var=0.089
global: beta=0.07,se=0.11,t=0.61,var=0.01
DNAm
ancestry
0.0
0.2
0.4
0.6
0.8
1.0

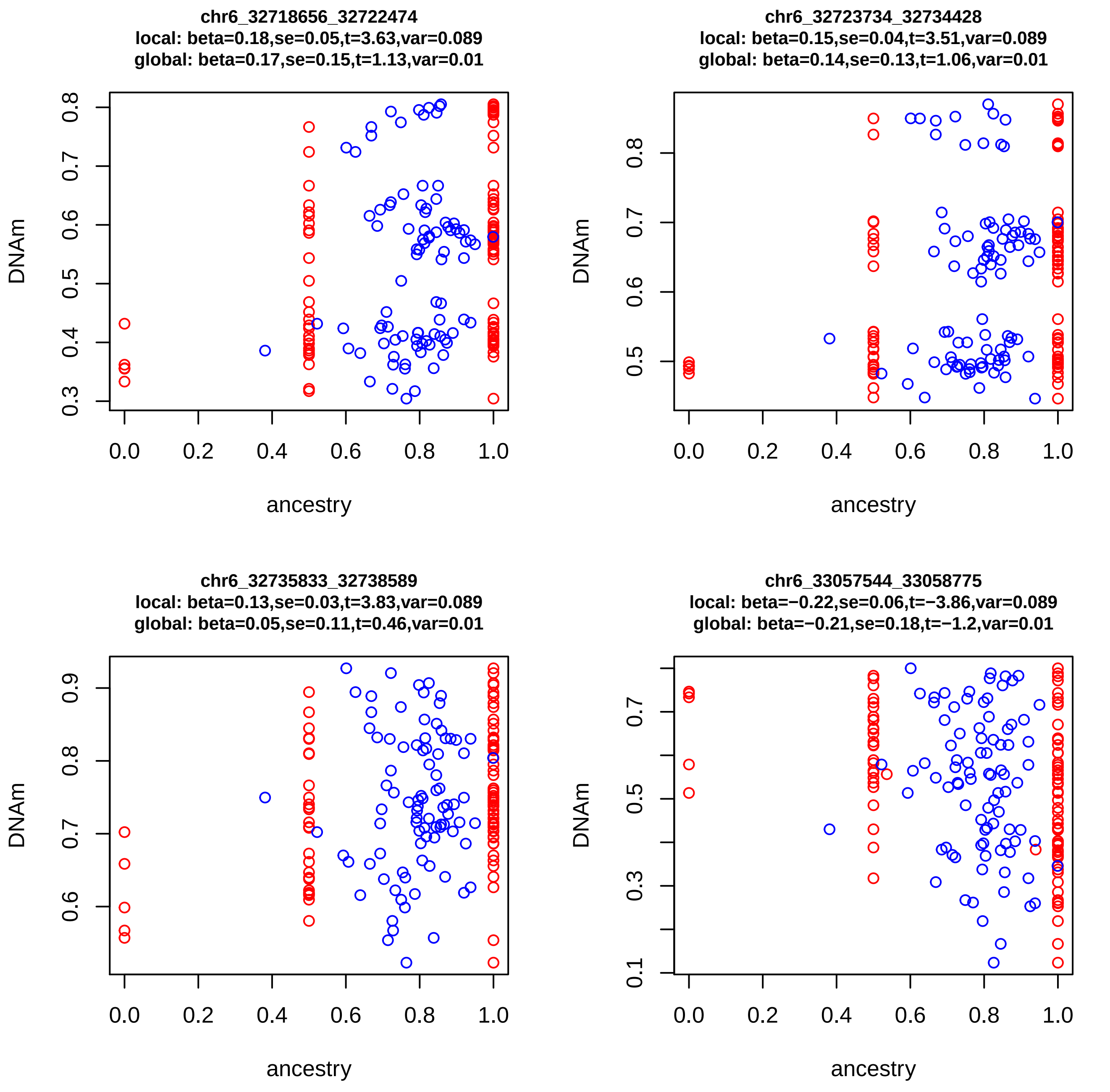
chr6_32718656_32722474
local: beta=0.18,se=0.05,t=3.63,var=0.089
global: beta=0.17,se=0.15,t=1.13,var=0.01
DNAm
ancestry
chr6_32723734_32734428
local: beta=0.15,se=0.04,t=3.51,var=0.089
global: beta=0.14,se=0.13,t=1.06,var=0.01
DNAm
ancestry
chr6_32735833_32738589
local: beta=0.13,se=0.03,t=3.83,var=0.089
global: beta=0.05,se=0.11,t=0.46,var=0.01
DNAm
ancestry
chr6_33057544_33058775
local: beta=−0.22,se=0.06,t=−3.86,var=0.089
global: beta=−0.21,se=0.18,t=−1.2,var=0.01
DNAm
ancestry

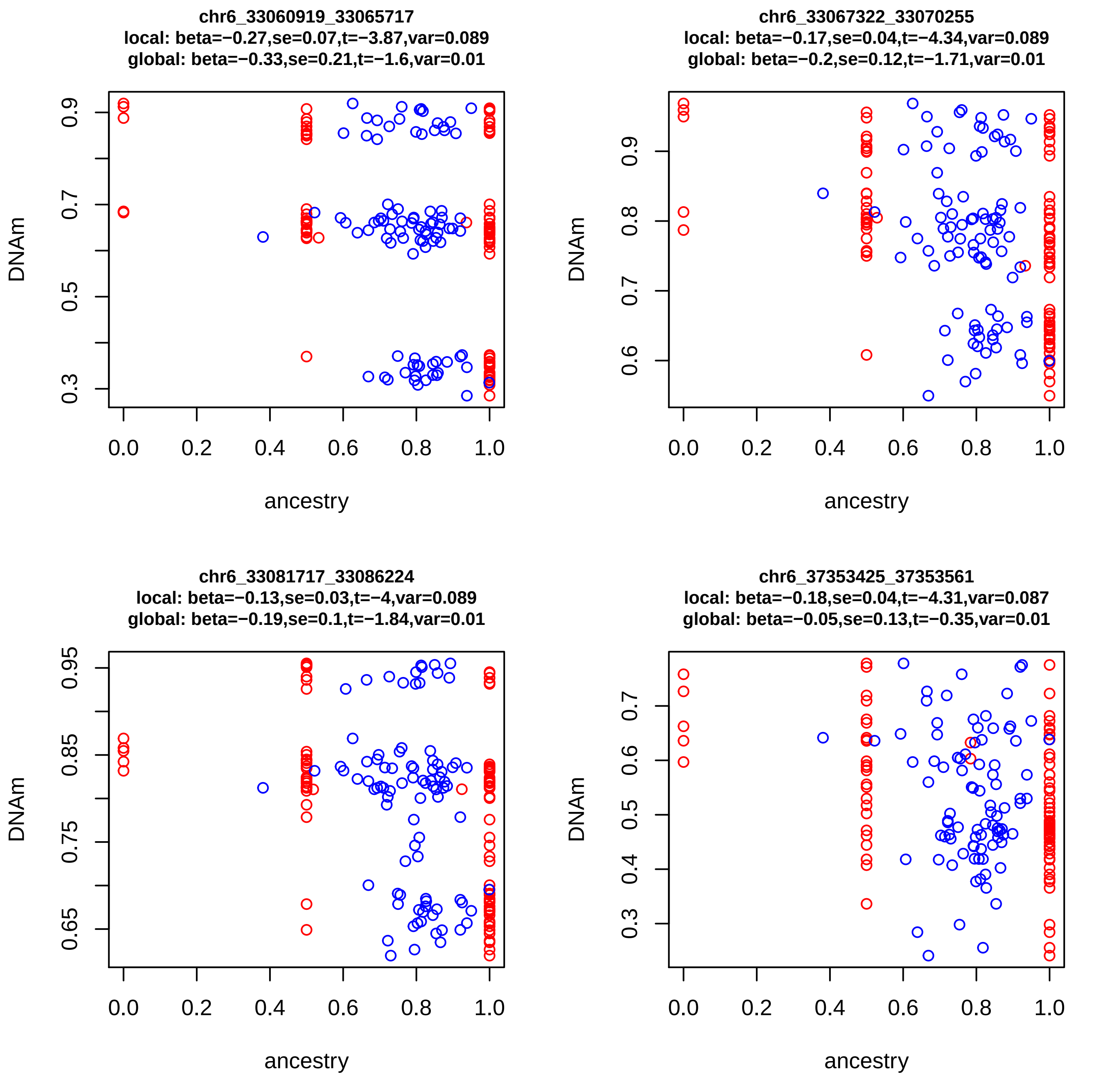
chr6_33060919_33065717
local: beta=−0.27,se=0.07,t=−3.87,var=0.089
global: beta=−0.33,se=0.21,t=−1.6,var=0.01
DNAm
ancestry
chr6_33067322_33070255
local: beta=−0.17,se=0.04,t=−4.34,var=0.089
global: beta=−0.2,se=0.12,t=−1.71,var=0.01
DNAm
ancestry
chr6_33081717_33086224
local: beta=−0.13,se=0.03,t=−4,var=0.089
global: beta=−0.19,se=0.1,t=−1.84,var=0.01
DNAm
ancestry
chr6_37353425_37353561
local: beta=−0.18,se=0.04,t=−4.31,var=0.087
global: beta=−0.05,se=0.13,t=−0.35,var=0.01
DNAm
ancestry

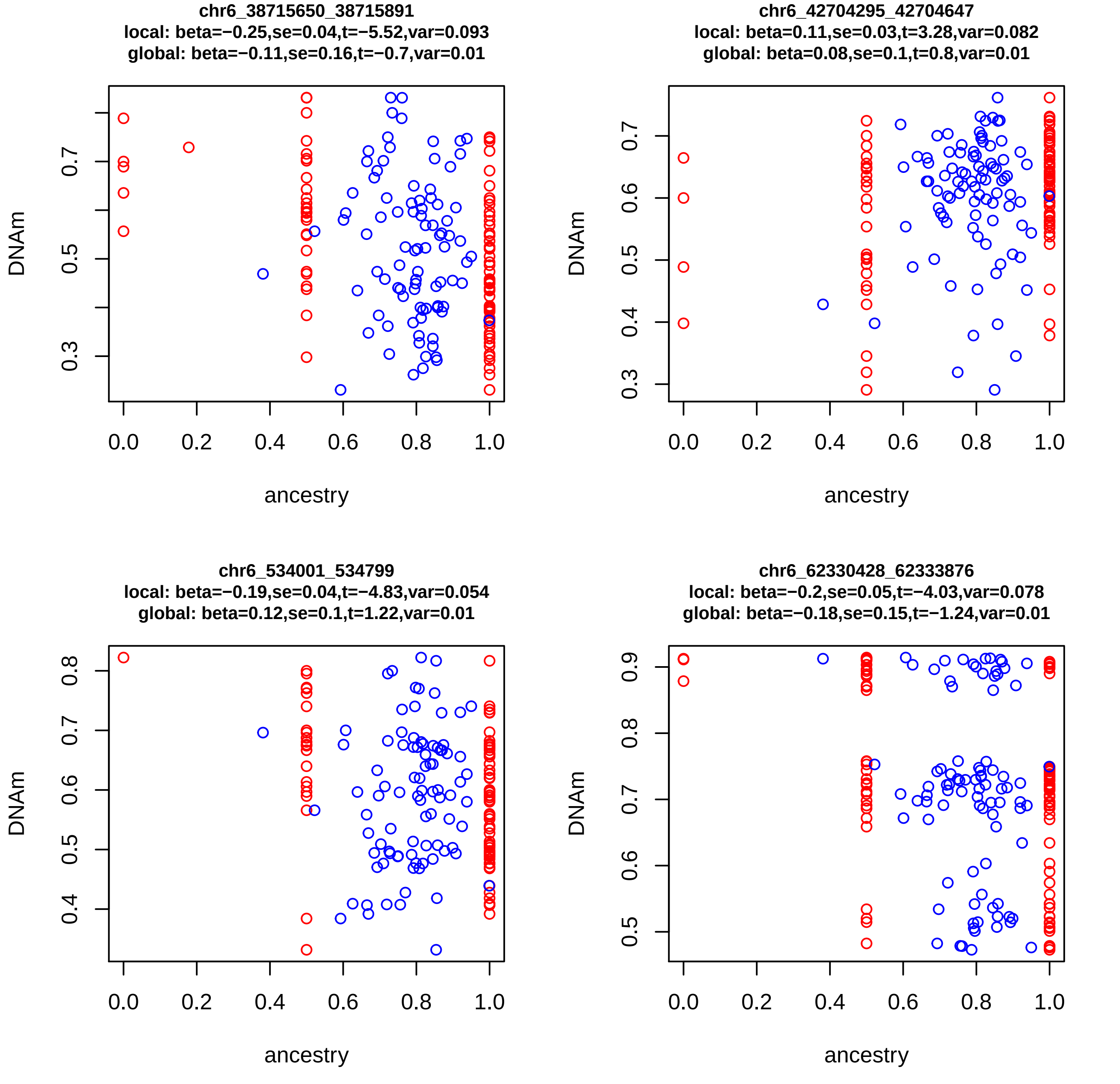
chr6_38715650_38715891
local: beta=−0.25,se=0.04,t=−5.52,var=0.093
global: beta=−0.11,se=0.16,t=−0.7,var=0.01
DNAm
0.3
0.5
0.7
0.0
0.2
0.4
0.6
0.8
1.0
ancestry
chr6_42704295_42704647
local: beta=0.11,se=0.03,t=3.28,var=0.082
global: beta=0.08,se=0.1,t=0.8,var=0.01
DNAm
0.3
0.4
0.5
0.6
0.7
0.0
0.2
0.4
0.6
0.8
1.0
ancestry
chr6_534001_534799
local: beta=−0.19,se=0.04,t=−4.83,var=0.054
global: beta=0.12,se=0.1,t=1.22,var=0.01
DNAm
0.4
0.5
0.6
0.7
0.8
0.0
0.2
0.4
0.6
0.8
1.0
ancestry
chr6_62330428_62333876
local: beta=−0.2,se=0.05,t=−4.03,var=0.078
global: beta=−0.18,se=0.15,t=−1.24,var=0.01
DNAm
0.5
0.6
0.7
0.8
0.9
0.0
0.2
0.4
0.6
0.8
1.0
ancestry

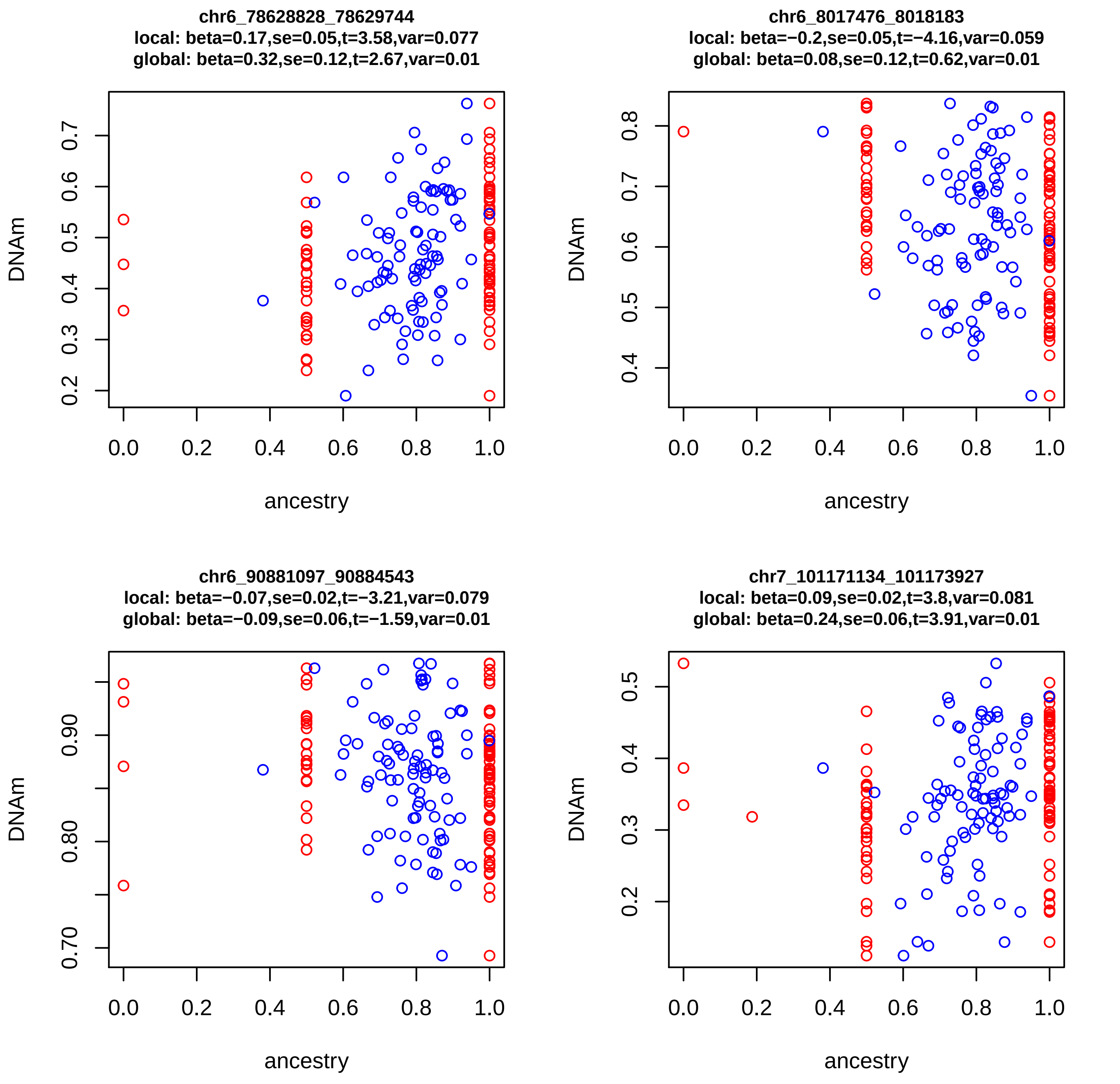
chr6_78628828_78629744
local: beta=0.17,se=0.05,t=3.58,var=0.077
global: beta=0.32,se=0.12,t=2.67,var=0.01
DNAm
ancestry
chr6_8017476_8018183
local: beta=−0.2,se=0.05,t=−4.16,var=0.059
global: beta=0.08,se=0.12,t=0.62,var=0.01
DNAm
ancestry
chr6_90881097_90884543
local: beta=−0.07,se=0.02,t=−3.21,var=0.079
global: beta=−0.09,se=0.06,t=−1.59,var=0.01
DNAm
ancestry
chr7_101171134_101173927
local: beta=0.09,se=0.02,t=3.8,var=0.081
global: beta=0.24,se=0.06,t=3.91,var=0.01
DNAm
ancestry

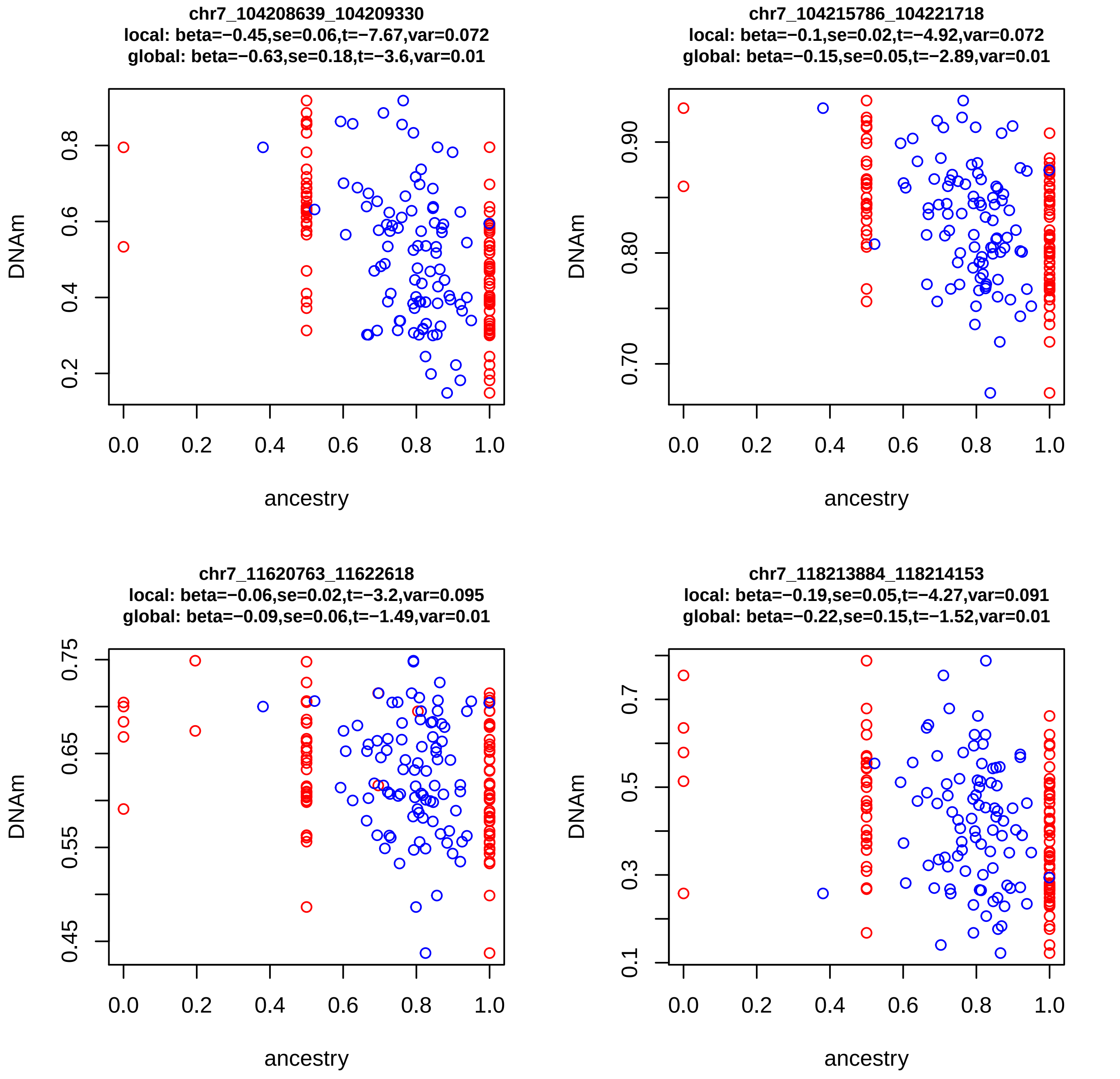
chr7_104208639_104209330
local: beta=−0.45,se=0.06,t=−7.67,var=0.072
global: beta=−0.63,se=0.18,t=−3.6,var=0.01
DNAm
0.2
0.4
0.6
0.8
0.0
0.2
0.4
0.6
0.8
1.0
ancestry
chr7_104215786_104221718
local: beta=−0.1,se=0.02,t=−4.92,var=0.072
global: beta=−0.15,se=0.05,t=−2.89,var=0.01
DNAm
0.70
0.80
0.90
0.0
0.2
0.4
0.6
0.8
1.0
ancestry
chr7_11620763_11622618
local: beta=−0.06,se=0.02,t=−3.2,var=0.095
global: beta=−0.09,se=0.06,t=−1.49,var=0.01
DNAm
0.45
0.55
0.65
0.75
0.0
0.2
0.4
0.6
0.8
1.0
ancestry
chr7_118213884_118214153
local: beta=−0.19,se=0.05,t=−4.27,var=0.091
global: beta=−0.22,se=0.15,t=−1.52,var=0.01
DNAm
0.1
0.3
0.5
0.7
0.0
0.2
0.4
0.6
0.8
1.0
ancestry

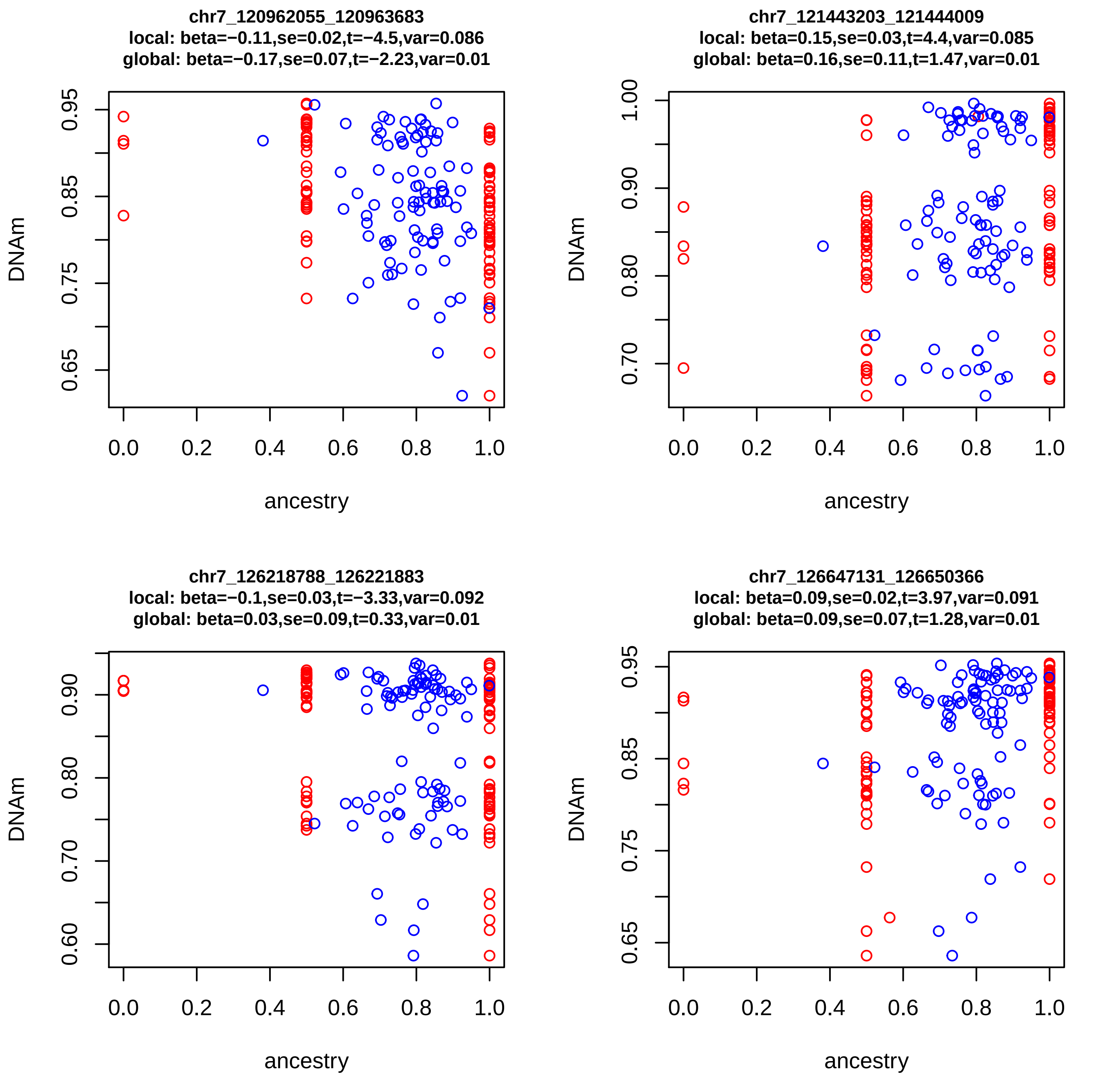
chr7_120962055_120963683
local: beta=−0.11,se=0.02,t=−4.5,var=0.086
global: beta=−0.17,se=0.07,t=−2.23,var=0.01
DNAm
ancestry
chr7_121443203_121444009
local: beta=0.15,se=0.03,t=4.4,var=0.085
global: beta=0.16,se=0.11,t=1.47,var=0.01
DNAm
ancestry
chr7_126218788_126221883
local: beta=−0.1,se=0.03,t=−3.33,var=0.092
global: beta=0.03,se=0.09,t=0.33,var=0.01
DNAm
ancestry
chr7_126647131_126650366
local: beta=0.09,se=0.02,t=3.97,var=0.091
global: beta=0.09,se=0.07,t=1.28,var=0.01
DNAm
ancestry

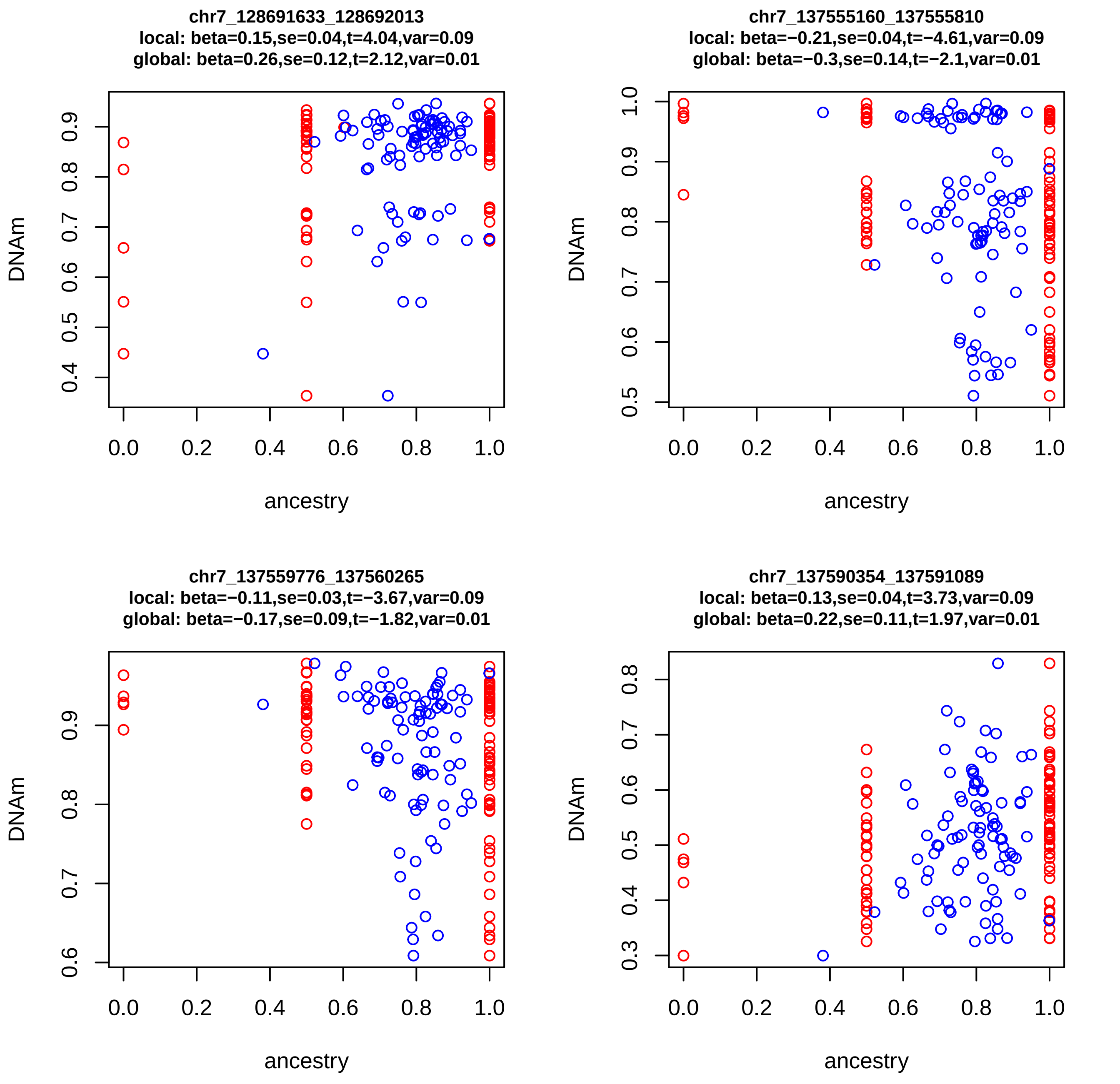
chr7_128691633_128692013
local: beta=0.15,se=0.04,t=4.04,var=0.09
global: beta=0.26,se=0.12,t=2.12,var=0.01
DNAm
ancestry
chr7_137555160_137555810
local: beta=−0.21,se=0.04,t=−4.61,var=0.09
global: beta=−0.3,se=0.14,t=−2.1,var=0.01
DNAm
ancestry
chr7_137559776_137560265
local: beta=−0.11,se=0.03,t=−3.67,var=0.09
global: beta=−0.17,se=0.09,t=−1.82,var=0.01
DNAm
ancestry
chr7_137590354_137591089
local: beta=0.13,se=0.04,t=3.73,var=0.09
global: beta=0.22,se=0.11,t=1.97,var=0.01
DNAm
ancestry

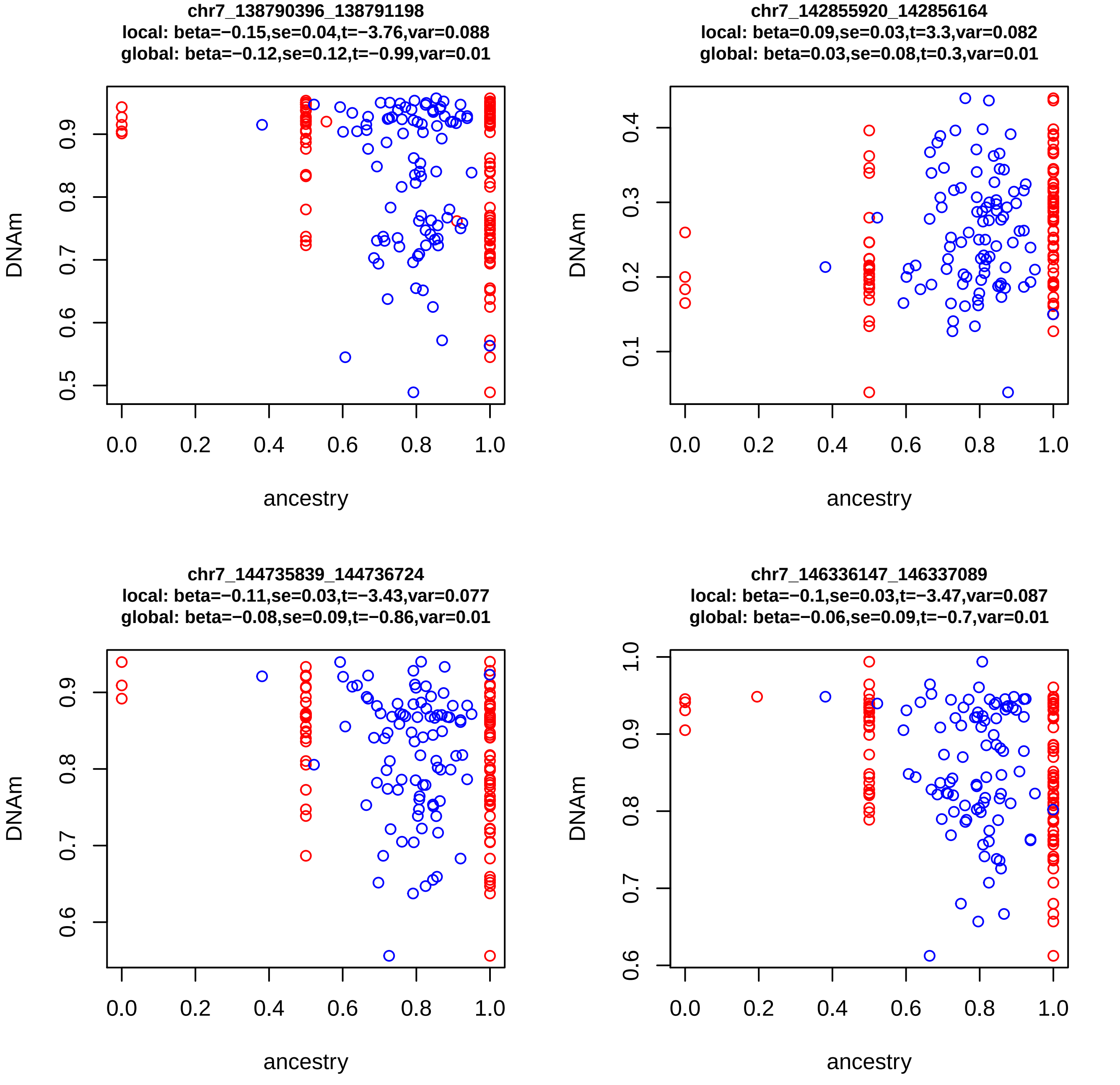
chr7_138790396_138791198
local: beta=−0.15,se=0.04,t=−3.76,var=0.088
global: beta=−0.12,se=0.12,t=−0.99,var=0.01
chr7_142855920_142856164
local: beta=0.09,se=0.03,t=3.3,var=0.082
global: beta=0.03,se=0.08,t=0.3,var=0.01
chr7_144735839_144736724
local: beta=−0.11,se=0.03,t=−3.43,var=0.077
global: beta=−0.08,se=0.09,t=−0.86,var=0.01
chr7_146336147_146337089
local: beta=−0.1,se=0.03,t=−3.47,var=0.087
global: beta=−0.06,se=0.09,t=−0.7,var=0.01
DNAm
ancestry

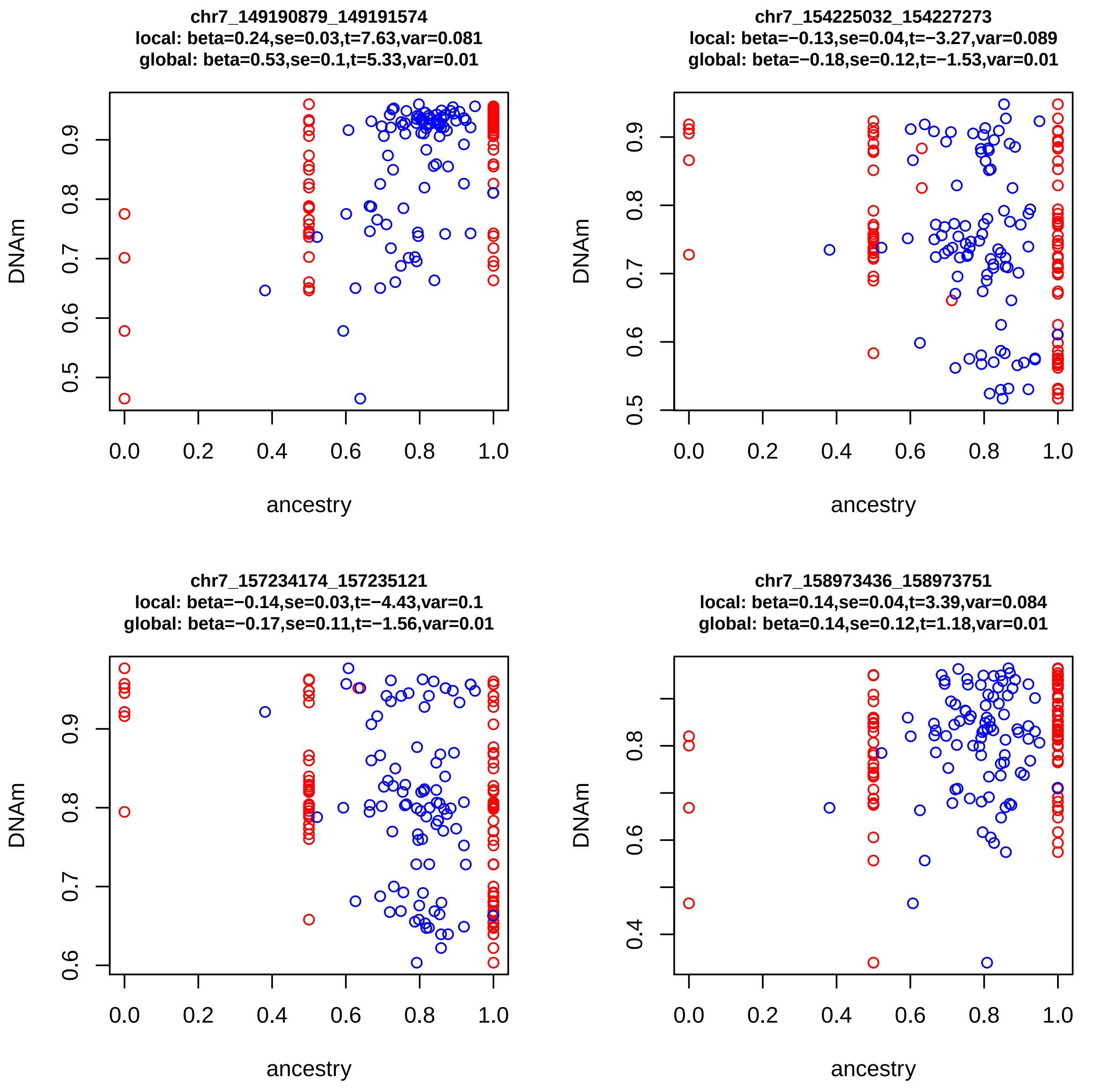
chr7_149190879_149191574
local: beta=0.24,se=0.03,t=7.63,var=0.081
global: beta=0.53,se=0.1,t=5.33,var=0.01
chr7_154225032_154227273
local: beta=−0.13,se=0.04,t=−3.27,var=0.089
global: beta=−0.18,se=0.12,t=−1.53,var=0.01
chr7_157234174_157235121
local: beta=−0.14,se=0.03,t=−4.43,var=0.1
global: beta=−0.17,se=0.11,t=−1.56,var=0.01
chr7_158973436_158973751
local: beta=0.14,se=0.04,t=3.39,var=0.084
global: beta=0.14,se=0.12,t=1.18,var=0.01
DNAm
ancestry
0.0
0.2
0.4
0.6
0.8
1.0

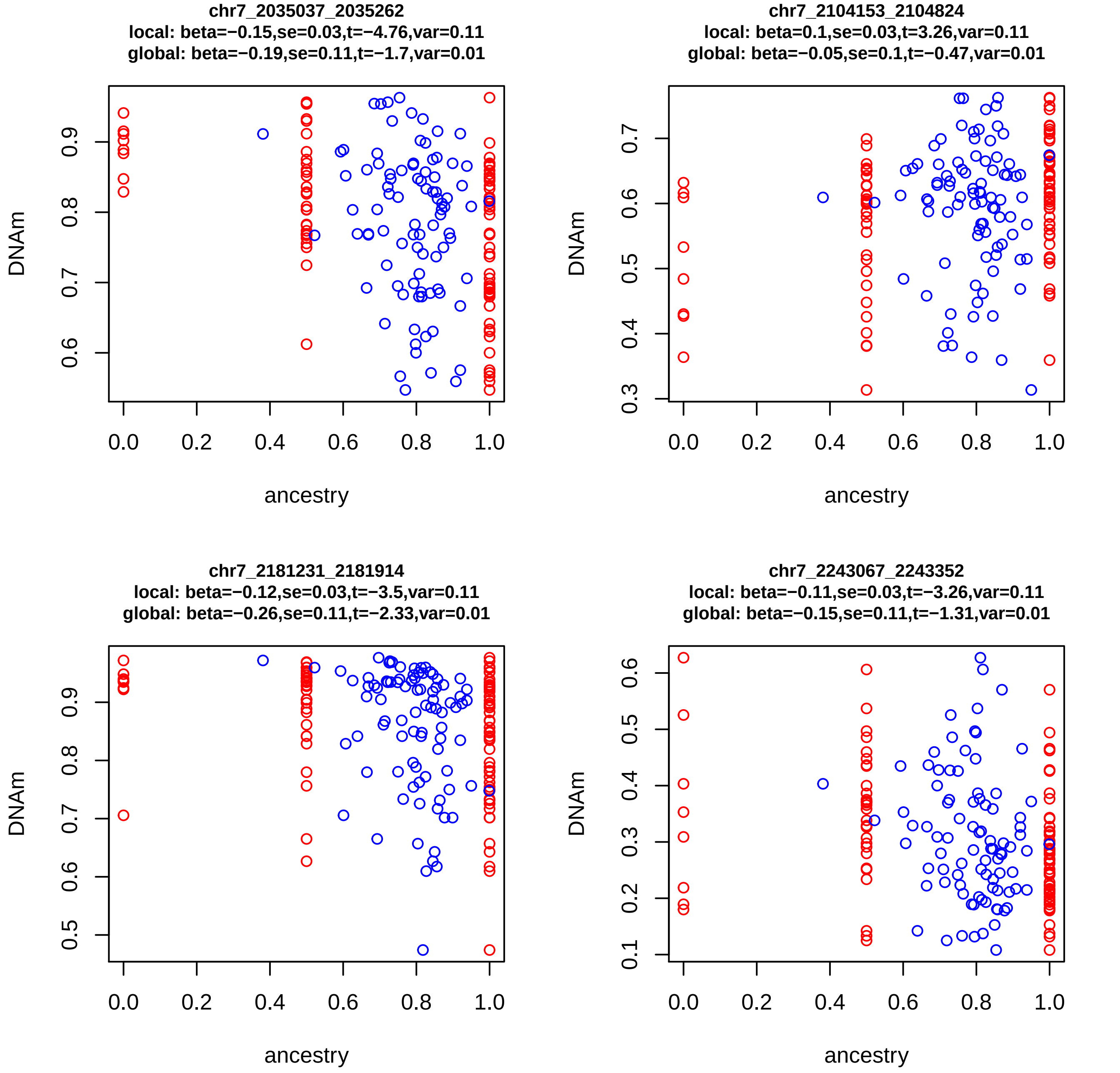
chr7_2035037_2035262
local: beta=−0.15,se=0.03,t=−4.76,var=0.11
global: beta=−0.19,se=0.11,t=−1.7,var=0.01
DNAm
ancestry
chr7_2104153_2104824
local: beta=0.1,se=0.03,t=3.26,var=0.11
global: beta=−0.05,se=0.1,t=−0.47,var=0.01
DNAm
ancestry
chr7_2181231_2181914
local: beta=−0.12,se=0.03,t=−3.5,var=0.11
global: beta=−0.26,se=0.11,t=−2.33,var=0.01
DNAm
ancestry
chr7_2243067_2243352
local: beta=−0.11,se=0.03,t=−3.26,var=0.11
global: beta=−0.15,se=0.11,t=−1.31,var=0.01
DNAm
ancestry

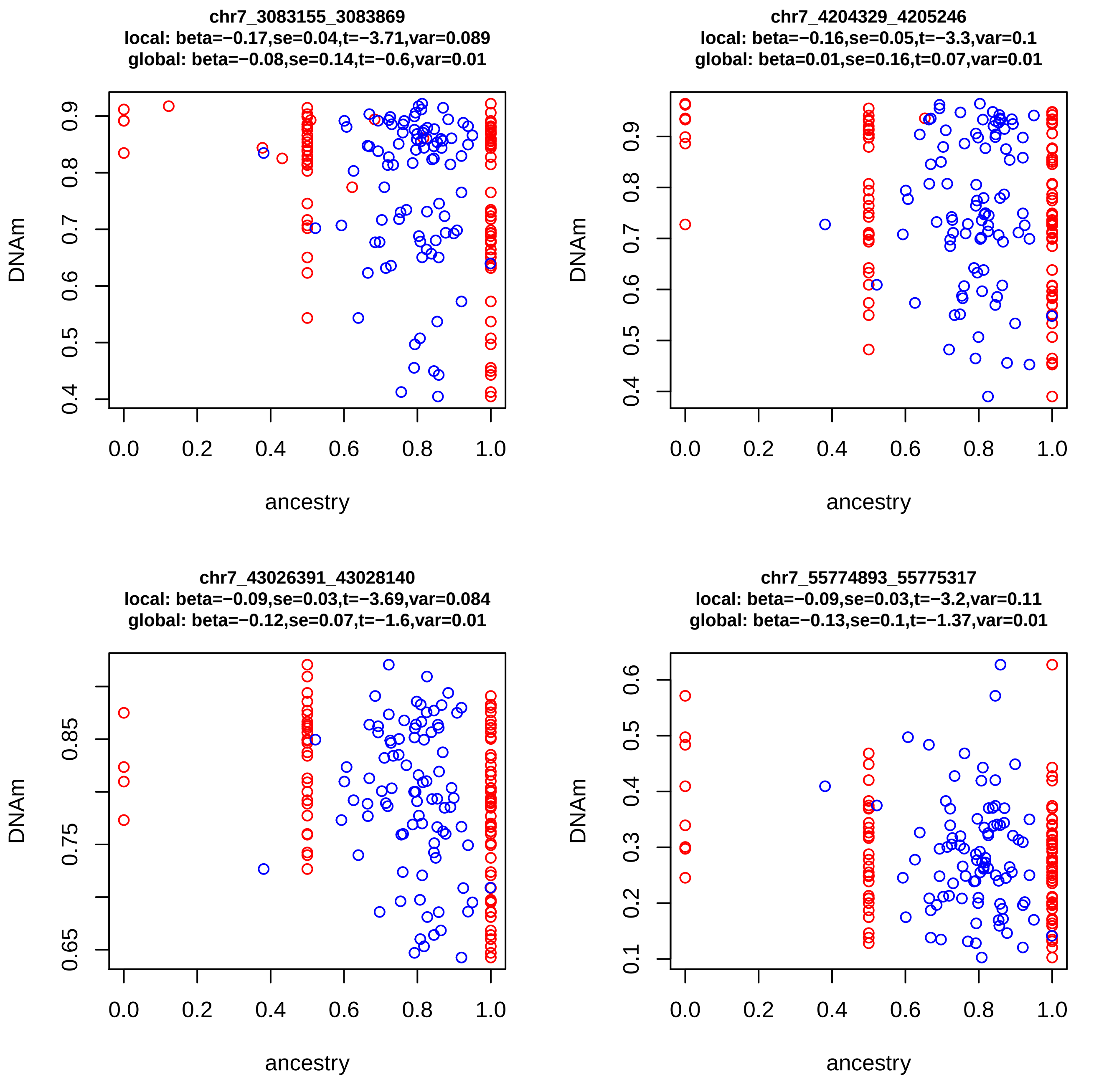
chr7_3083155_3083869
local: beta=−0.17,se=0.04,t=−3.71,var=0.089
global: beta=−0.08,se=0.14,t=−0.6,var=0.01
DNAm
ancestry
chr7_4204329_4205246
local: beta=−0.16,se=0.05,t=−3.3,var=0.1
global: beta=0.01,se=0.16,t=0.07,var=0.01
DNAm
ancestry
chr7_43026391_43028140
local: beta=−0.09,se=0.03,t=−3.69,var=0.084
global: beta=−0.12,se=0.07,t=−1.6,var=0.01
DNAm
ancestry
chr7_55774893_55775317
local: beta=−0.09,se=0.03,t=−3.2,var=0.11
global: beta=−0.13,se=0.1,t=−1.37,var=0.01
DNAm
ancestry

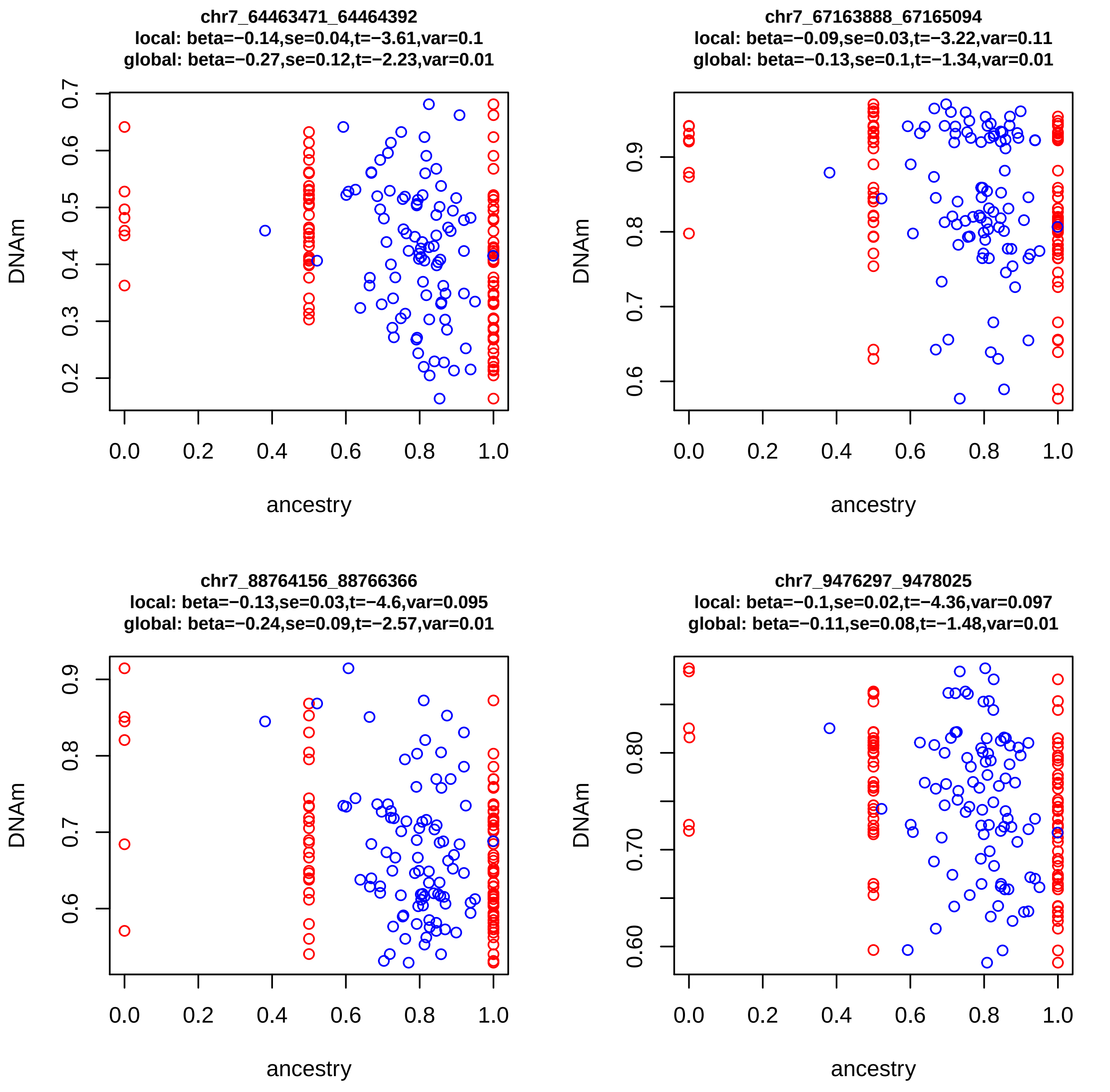
chr7_64463471_64464392
local: beta=−0.14,se=0.04,t=−3.61,var=0.1
global: beta=−0.27,se=0.12,t=−2.23,var=0.01
DNAm
ancestry
chr7_67163888_67165094
local: beta=−0.09,se=0.03,t=−3.22,var=0.11
global: beta=−0.13,se=0.1,t=−1.34,var=0.01
DNAm
ancestry
chr7_88764156_88766366
local: beta=−0.13,se=0.03,t=−4.6,var=0.095
global: beta=−0.24,se=0.09,t=−2.57,var=0.01
DNAm
ancestry
chr7_9476297_9478025
local: beta=−0.1,se=0.02,t=−4.36,var=0.097
global: beta=−0.11,se=0.08,t=−1.48,var=0.01
DNAm
ancestry

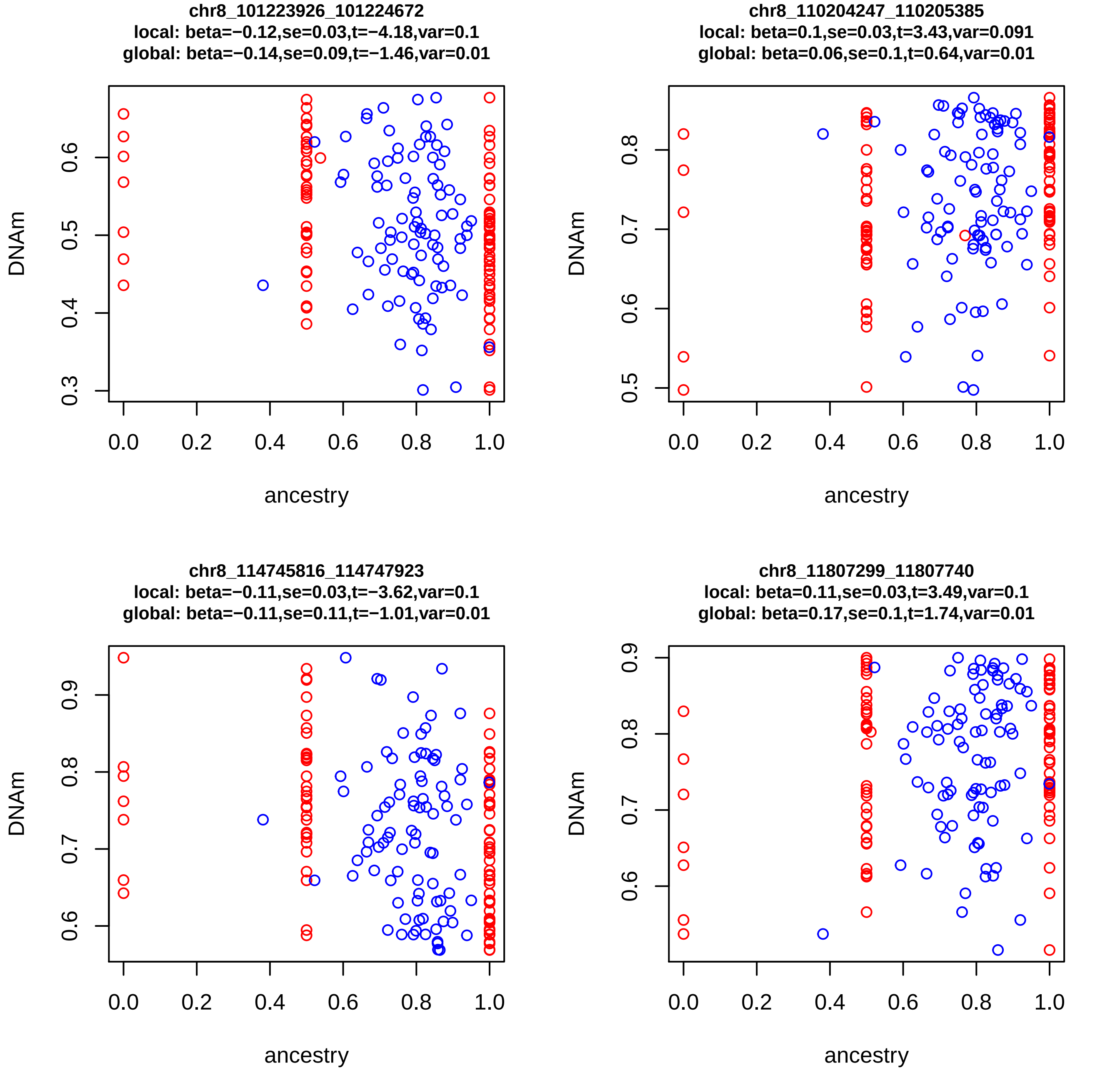
chr8_101223926_101224672
local: beta=−0.12,se=0.03,t=−4.18,var=0.1
global: beta=−0.14,se=0.09,t=−1.46,var=0.01
DNAm
ancestry
chr8_110204247_110205385
local: beta=0.1,se=0.03,t=3.43,var=0.091
global: beta=0.06,se=0.1,t=0.64,var=0.01
DNAm
ancestry
chr8_114745816_114747923
local: beta=−0.11,se=0.03,t=−3.62,var=0.1
global: beta=−0.11,se=0.11,t=−1.01,var=0.01
DNAm
ancestry
chr8_11807299_11807740
local: beta=0.11,se=0.03,t=3.49,var=0.1
global: beta=0.17,se=0.1,t=1.74,var=0.01
DNAm
ancestry

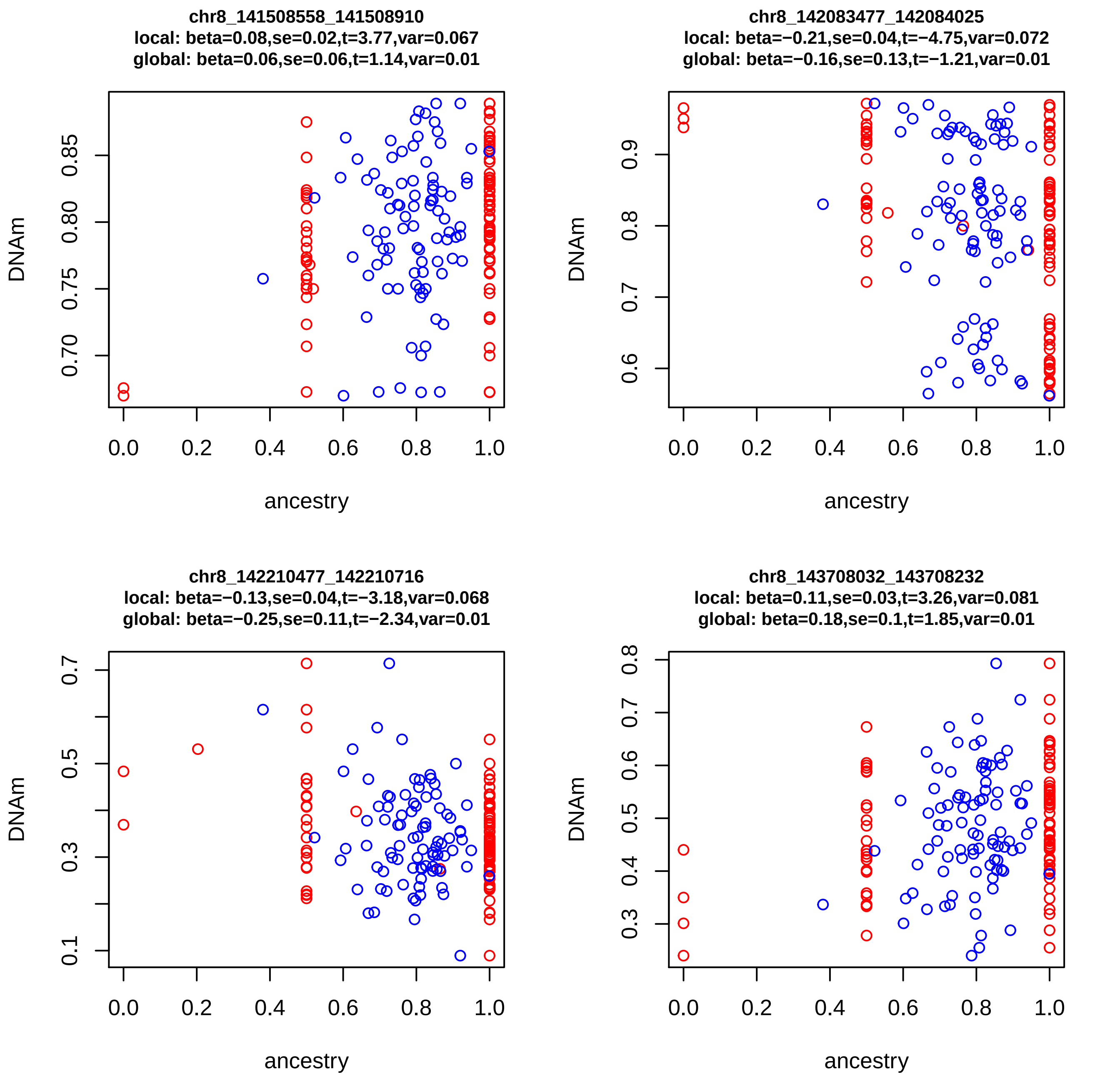
chr8_141508558_141508910
local: beta=0.08,se=0.02,t=3.77,var=0.067
global: beta=0.06,se=0.06,t=1.14,var=0.01
DNAm
ancestry
chr8_142083477_142084025
local: beta=−0.21,se=0.04,t=−4.75,var=0.072
global: beta=−0.16,se=0.13,t=−1.21,var=0.01
DNAm
ancestry
chr8_142210477_142210716
local: beta=−0.13,se=0.04,t=−3.18,var=0.068
global: beta=−0.25,se=0.11,t=−2.34,var=0.01
DNAm
ancestry
chr8_143708032_143708232
local: beta=0.11,se=0.03,t=3.26,var=0.081
global: beta=0.18,se=0.1,t=1.85,var=0.01
DNAm
ancestry

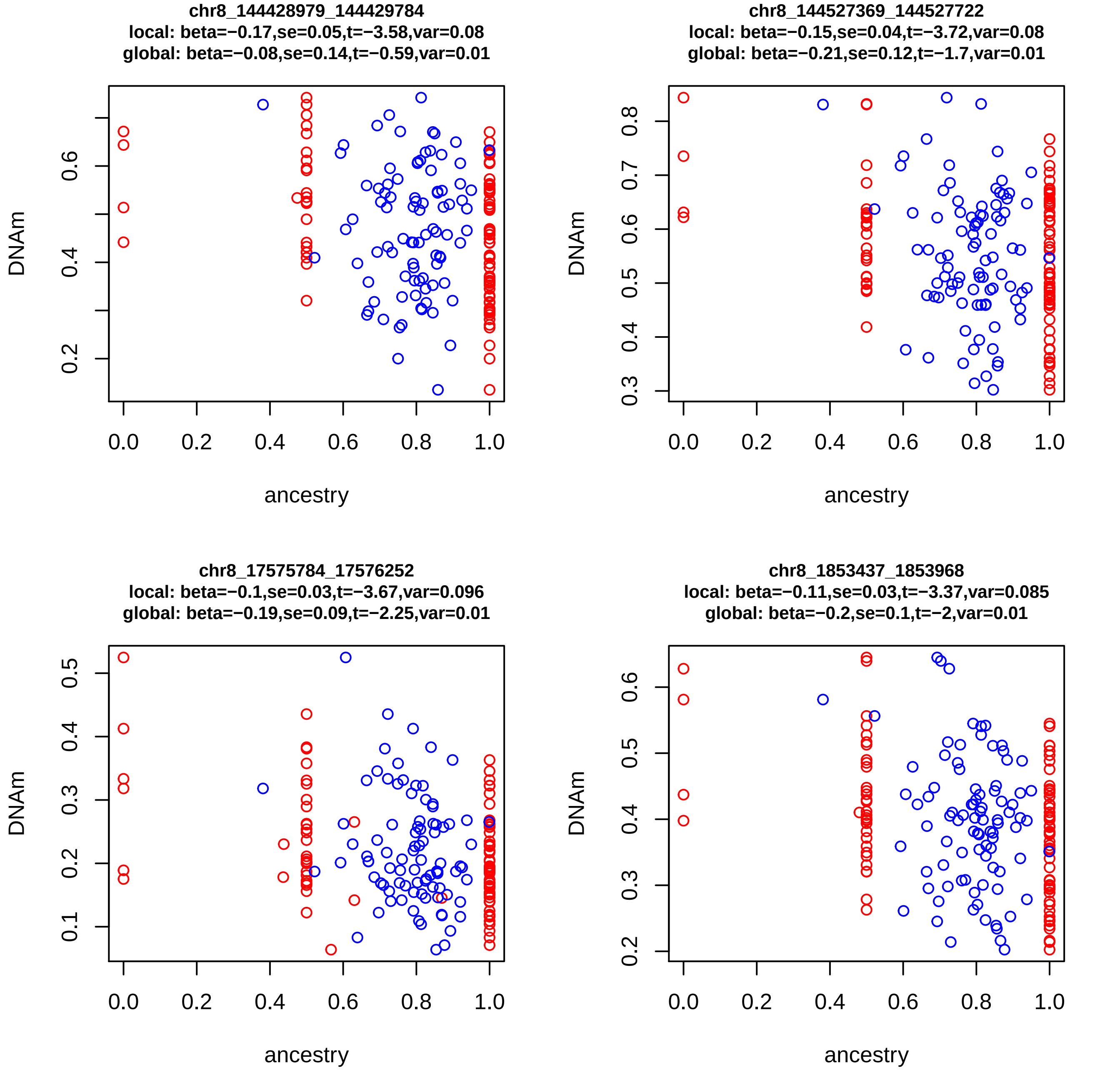
chr8_144428979_144429784
local: beta=−0.17,se=0.05,t=−3.58,var=0.08
global: beta=−0.08,se=0.14,t=−0.59,var=0.01
DNAm
ancestry
chr8_144527369_144527722
local: beta=−0.15,se=0.04,t=−3.72,var=0.08
global: beta=−0.21,se=0.12,t=−1.7,var=0.01
DNAm
ancestry
chr8_17575784_17576252
local: beta=−0.1,se=0.03,t=−3.67,var=0.096
global: beta=−0.19,se=0.09,t=−2.25,var=0.01
DNAm
ancestry
chr8_1853437_1853968
local: beta=−0.11,se=0.03,t=−3.37,var=0.085
global: beta=−0.2,se=0.1,t=−2,var=0.01
DNAm
ancestry

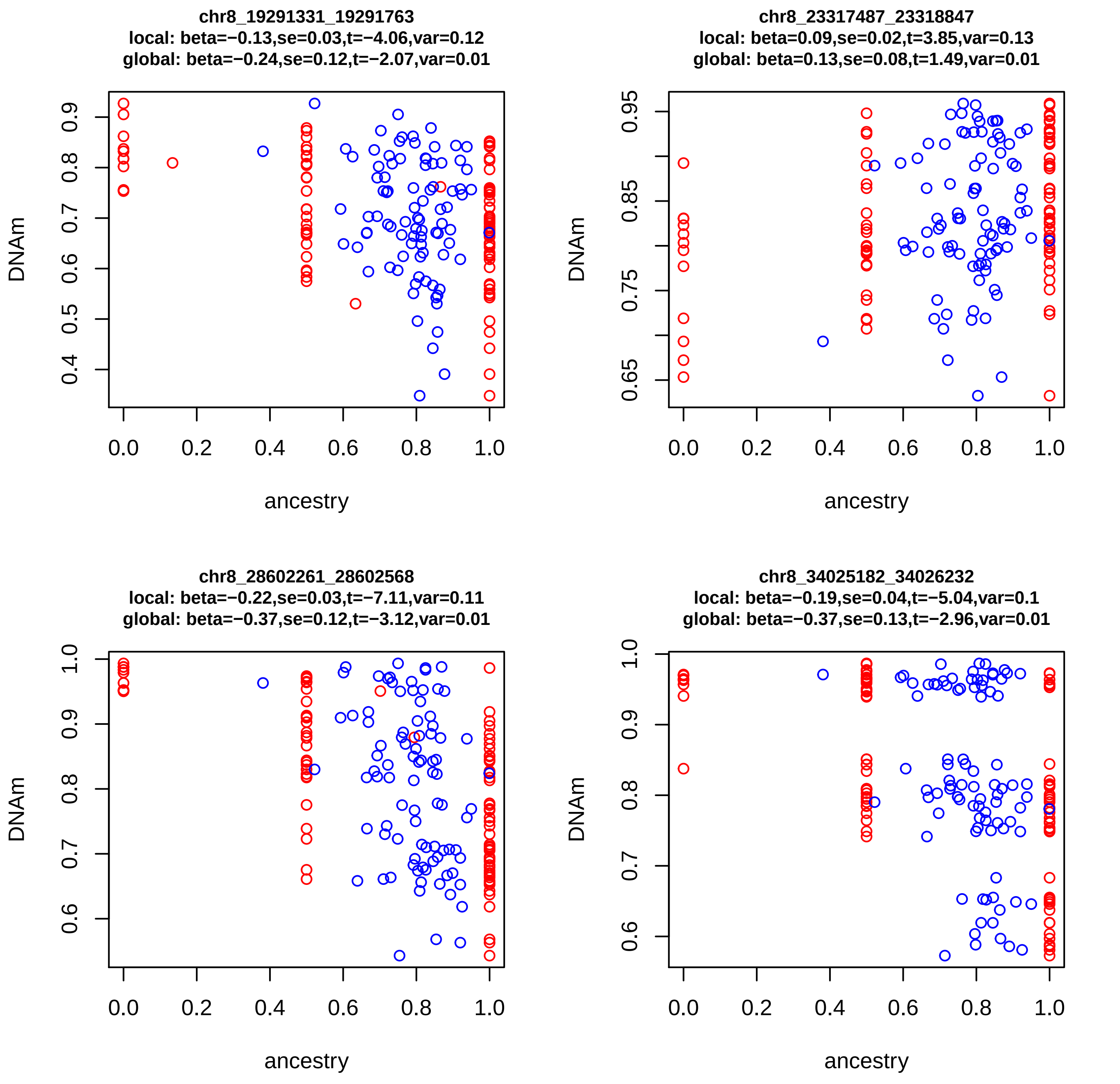
chr8_19291331_19291763
local: beta=−0.13,se=0.03,t=−4.06,var=0.12
global: beta=−0.24,se=0.12,t=−2.07,var=0.01
DNAm
ancestry
chr8_23317487_23318847
local: beta=0.09,se=0.02,t=3.85,var=0.13
global: beta=0.13,se=0.08,t=1.49,var=0.01
DNAm
ancestry
chr8_28602261_28602568
local: beta=−0.22,se=0.03,t=−7.11,var=0.11
global: beta=−0.37,se=0.12,t=−3.12,var=0.01
DNAm
ancestry
chr8_34025182_34026232
local: beta=−0.19,se=0.04,t=−5.04,var=0.1
global: beta=−0.37,se=0.13,t=−2.96,var=0.01
DNAm
ancestry

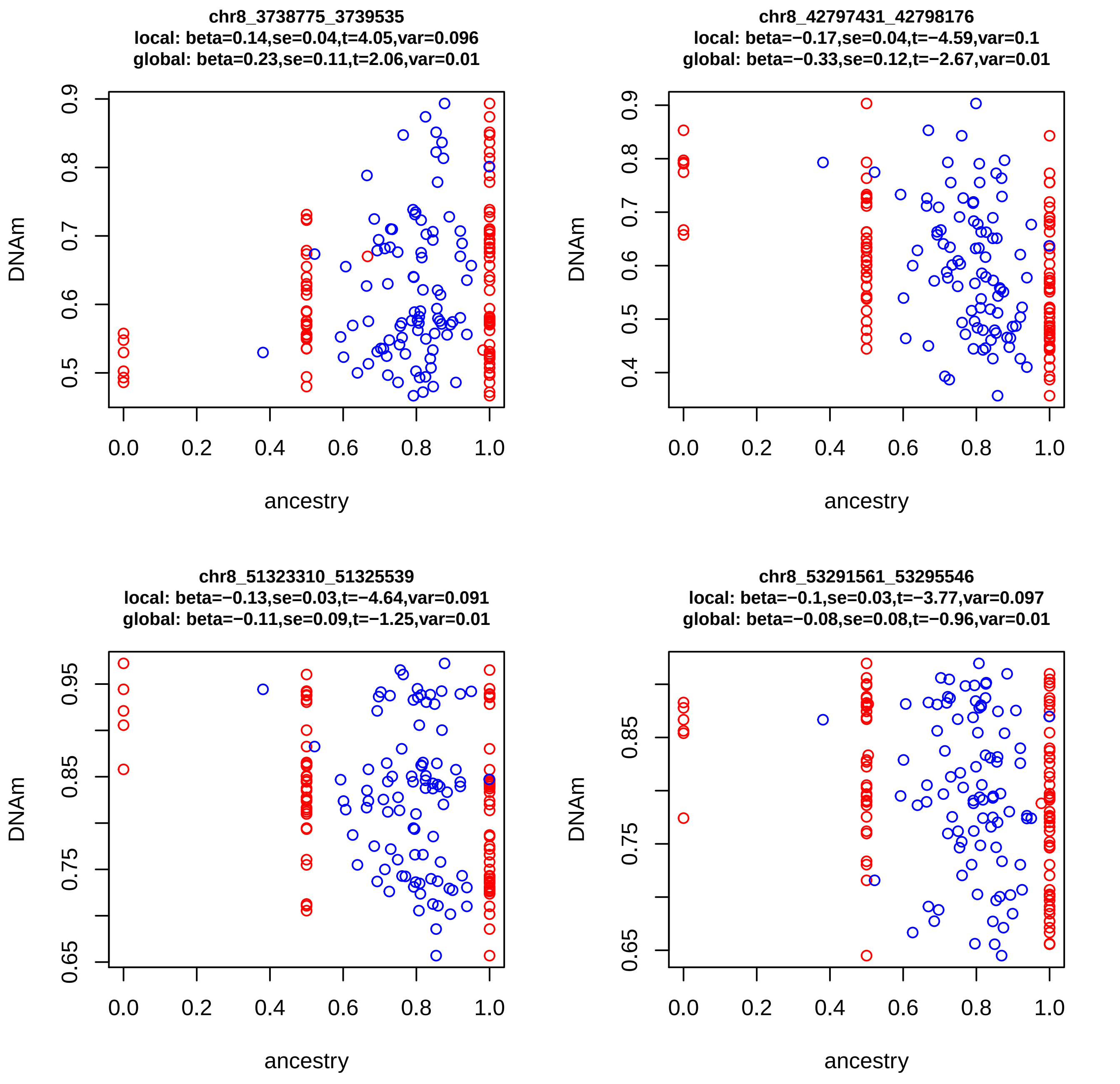
chr8_3738775_3739535
local: beta=0.14,se=0.04,t=4.05,var=0.096
global: beta=0.23,se=0.11,t=2.06,var=0.01
chr8_42797431_42798176
local: beta=−0.17,se=0.04,t=−4.59,var=0.1
global: beta=−0.33,se=0.12,t=−2.67,var=0.01
chr8_51323310_51325539
local: beta=−0.13,se=0.03,t=−4.64,var=0.091
global: beta=−0.11,se=0.09,t=−1.25,var=0.01
chr8_53291561_53295546
local: beta=−0.1,se=0.03,t=−3.77,var=0.097
global: beta=−0.08,se=0.08,t=−0.96,var=0.01
DNAm
ancestry

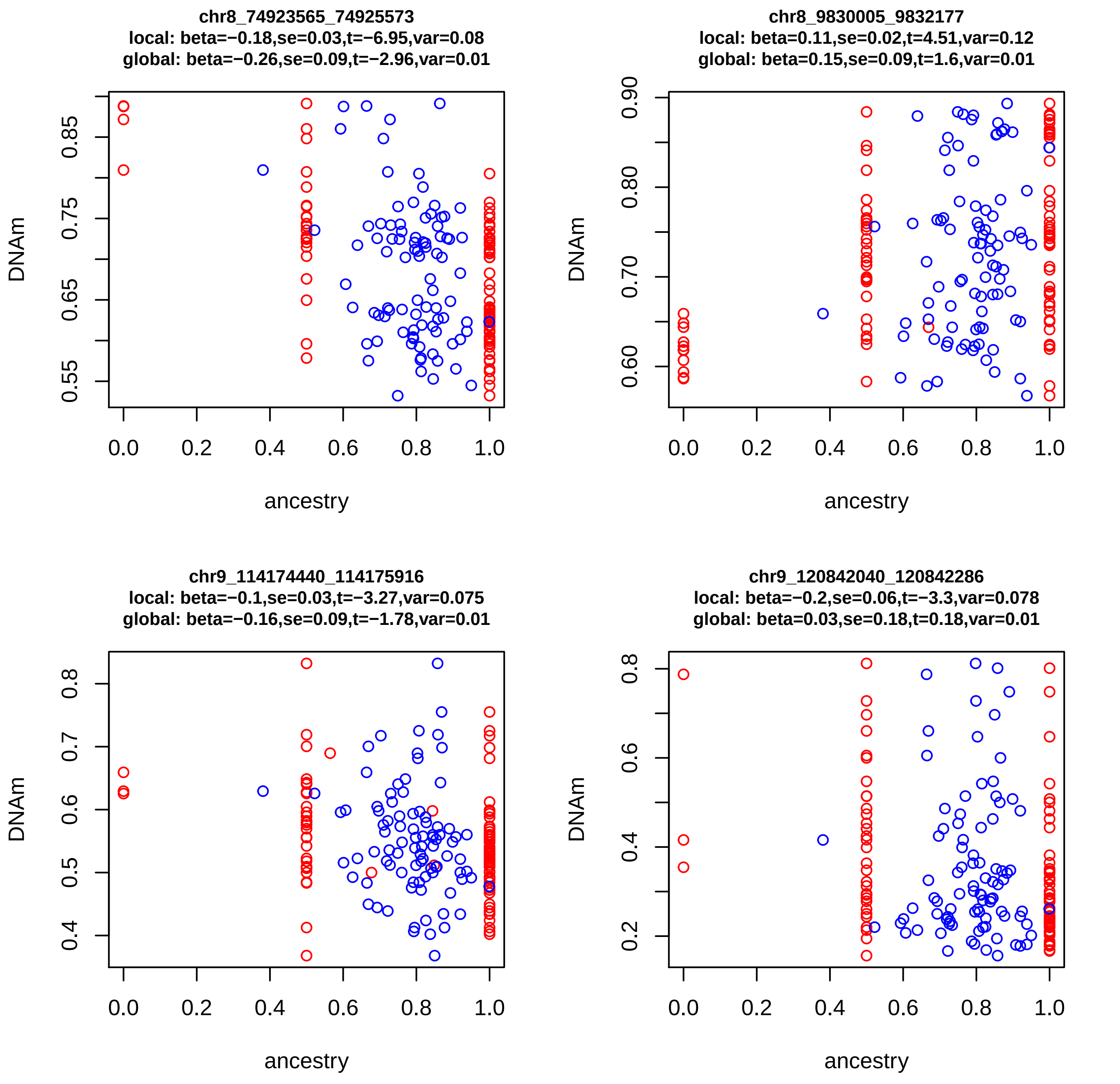
chr8_74923565_74925573
local: beta=−0.18,se=0.03,t=−6.95,var=0.08
global: beta=−0.26,se=0.09,t=−2.96,var=0.01
DNAm
ancestry
chr8_9830005_9832177
local: beta=0.11,se=0.02,t=4.51,var=0.12
global: beta=0.15,se=0.09,t=1.6,var=0.01
DNAm
ancestry
chr9_114174440_114175916
local: beta=−0.1,se=0.03,t=−3.27,var=0.075
global: beta=−0.16,se=0.09,t=−1.78,var=0.01
DNAm
ancestry
chr9_120842040_120842286
local: beta=−0.2,se=0.06,t=−3.3,var=0.078
global: beta=0.03,se=0.18,t=0.18,var=0.01
DNAm
ancestry

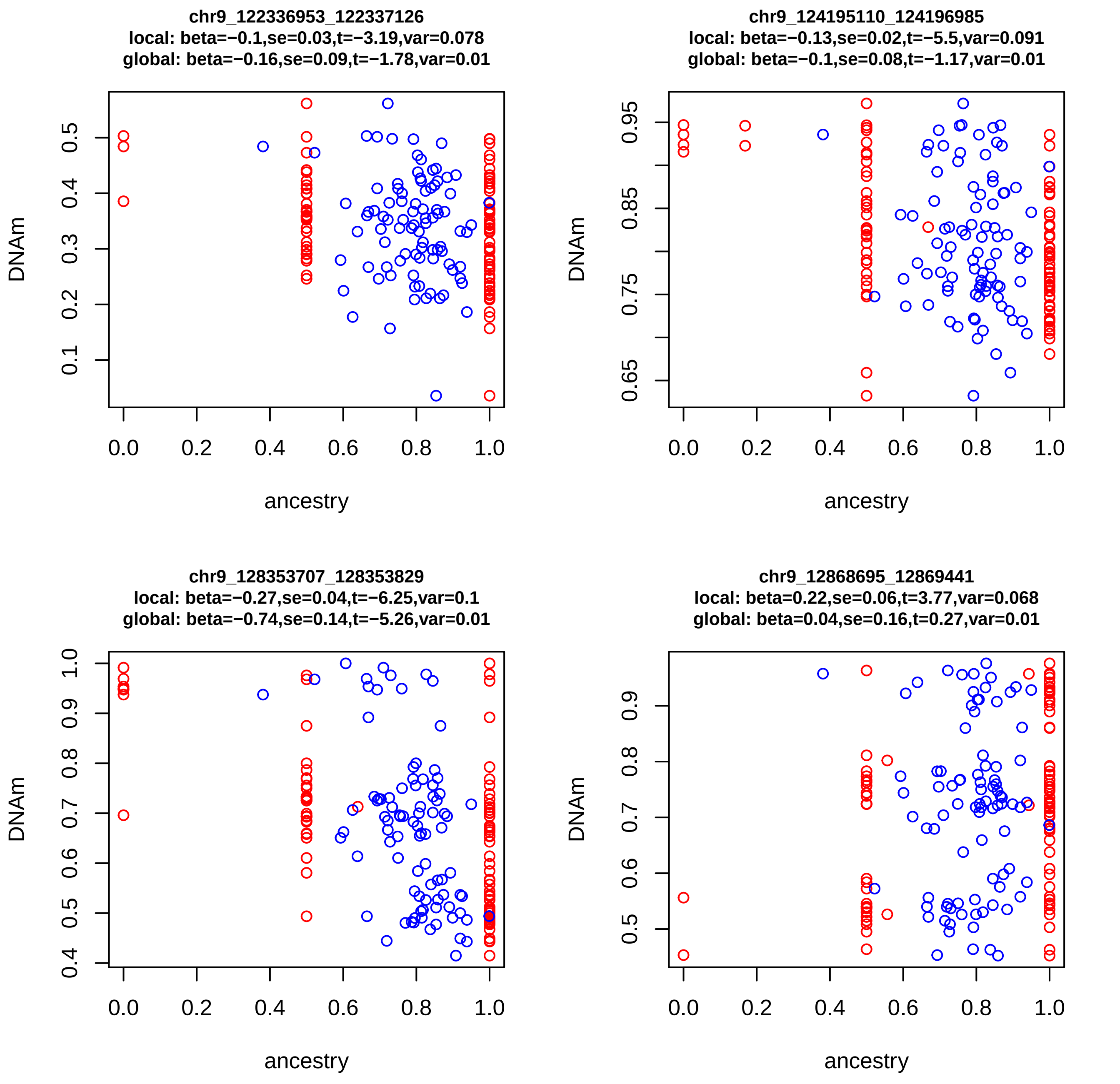
chr9_122336953_122337126
local: beta=−0.1,se=0.03,t=−3.19,var=0.078
global: beta=−0.16,se=0.09,t=−1.78,var=0.01
DNAm
ancestry
chr9_124195110_124196985
local: beta=−0.13,se=0.02,t=−5.5,var=0.091
global: beta=−0.1,se=0.08,t=−1.17,var=0.01
DNAm
ancestry
chr9_128353707_128353829
local: beta=−0.27,se=0.04,t=−6.25,var=0.1
global: beta=−0.74,se=0.14,t=−5.26,var=0.01
DNAm
ancestry
chr9_12868695_12869441
local: beta=0.22,se=0.06,t=3.77,var=0.068
global: beta=0.04,se=0.16,t=0.27,var=0.01
DNAm
ancestry

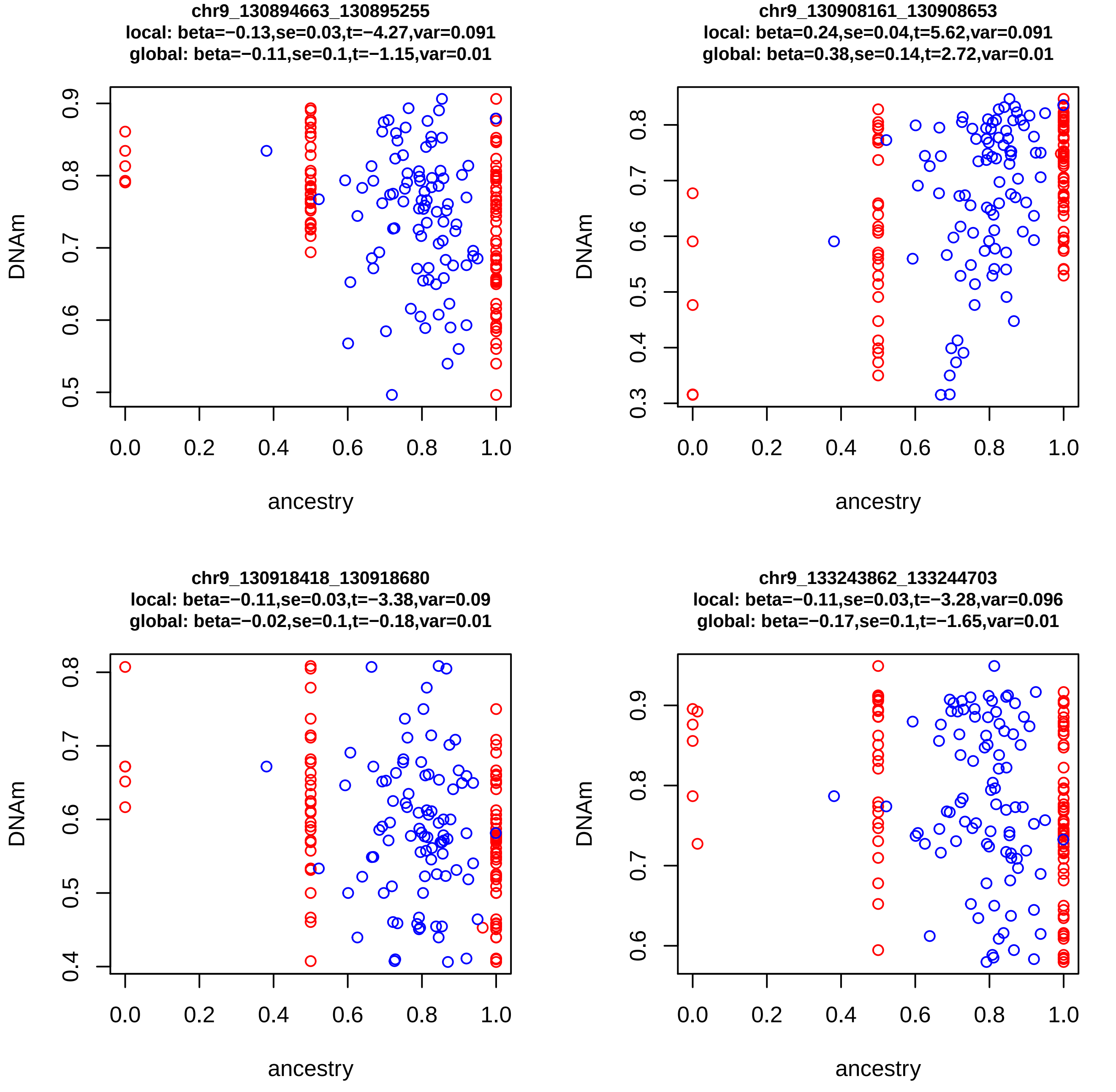
chr9_130894663_130895255
local: beta=−0.13,se=0.03,t=−4.27,var=0.091
global: beta=−0.11,se=0.1,t=−1.15,var=0.01
DNAm
ancestry
chr9_130908161_130908653
local: beta=0.24,se=0.04,t=5.62,var=0.091
global: beta=0.38,se=0.14,t=2.72,var=0.01
DNAm
ancestry
chr9_130918418_130918680
local: beta=−0.11,se=0.03,t=−3.38,var=0.09
global: beta=−0.02,se=0.1,t=−0.18,var=0.01
DNAm
ancestry
chr9_133243862_133244703
local: beta=−0.11,se=0.03,t=−3.28,var=0.096
global: beta=−0.17,se=0.1,t=−1.65,var=0.01
DNAm
ancestry

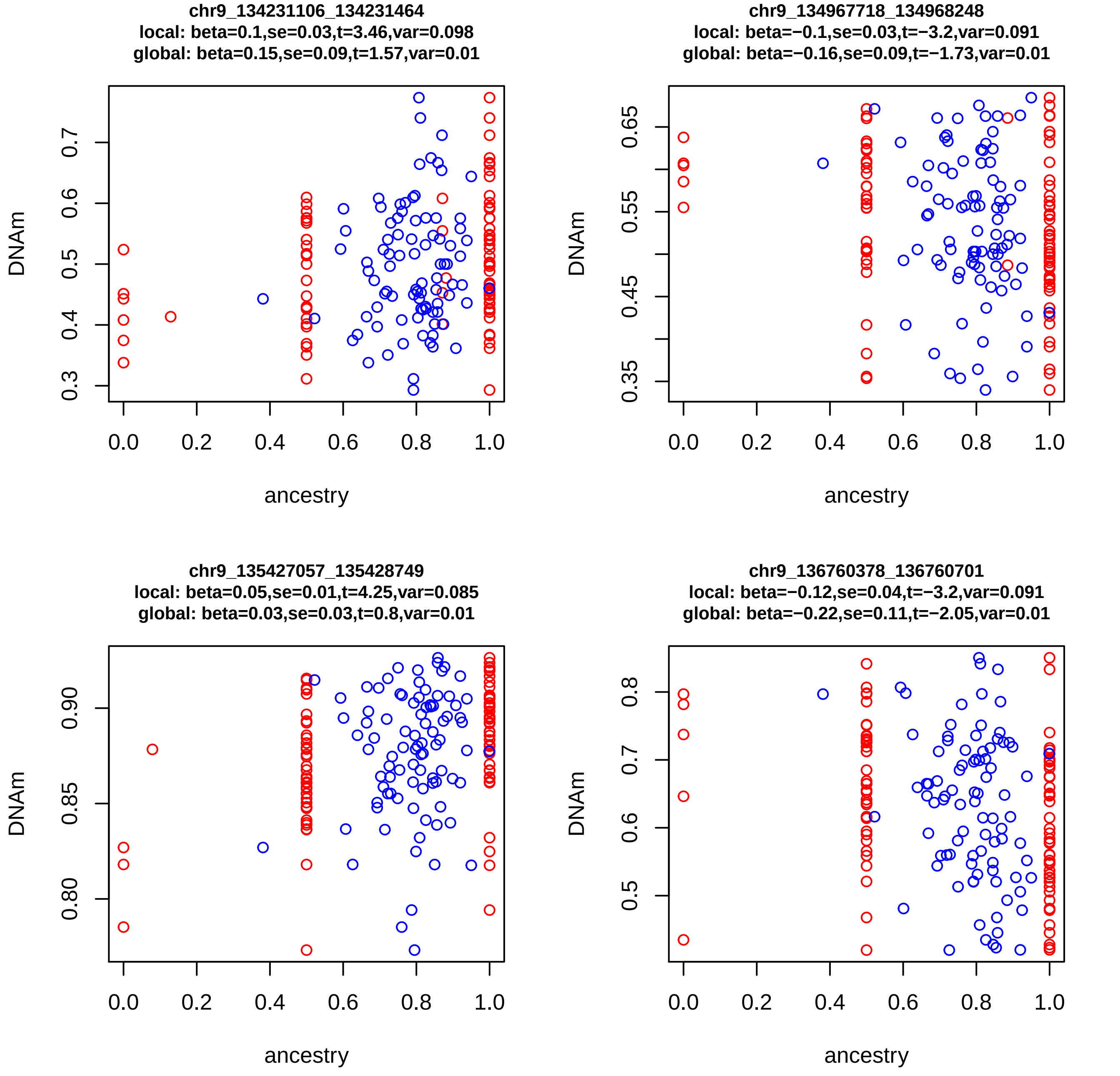
chr9_134231106_134231464
local: beta=0.1,se=0.03,t=3.46,var=0.098
global: beta=0.15,se=0.09,t=1.57,var=0.01
DNAm
ancestry
0.3
0.4
0.5
0.6
0.7
0.0
0.2
0.4
0.6
0.8
1.0
chr9_134967718_134968248
local: beta=−0.1,se=0.03,t=−3.2,var=0.091
global: beta=−0.16,se=0.09,t=−1.73,var=0.01
DNAm
ancestry
0.35
0.45
0.55
0.65
0.0
0.2
0.4
0.6
0.8
1.0
chr9_135427057_135428749
local: beta=0.05,se=0.01,t=4.25,var=0.085
global: beta=0.03,se=0.03,t=0.8,var=0.01
DNAm
ancestry
0.80
0.85
0.90
0.0
0.2
0.4
0.6
0.8
1.0
chr9_136760378_136760701
local: beta=−0.12,se=0.04,t=−3.2,var=0.091
global: beta=−0.22,se=0.11,t=−2.05,var=0.01
DNAm
ancestry
0.5
0.6
0.7
0.8
0.0
0.2
0.4
0.6
0.8
1.0

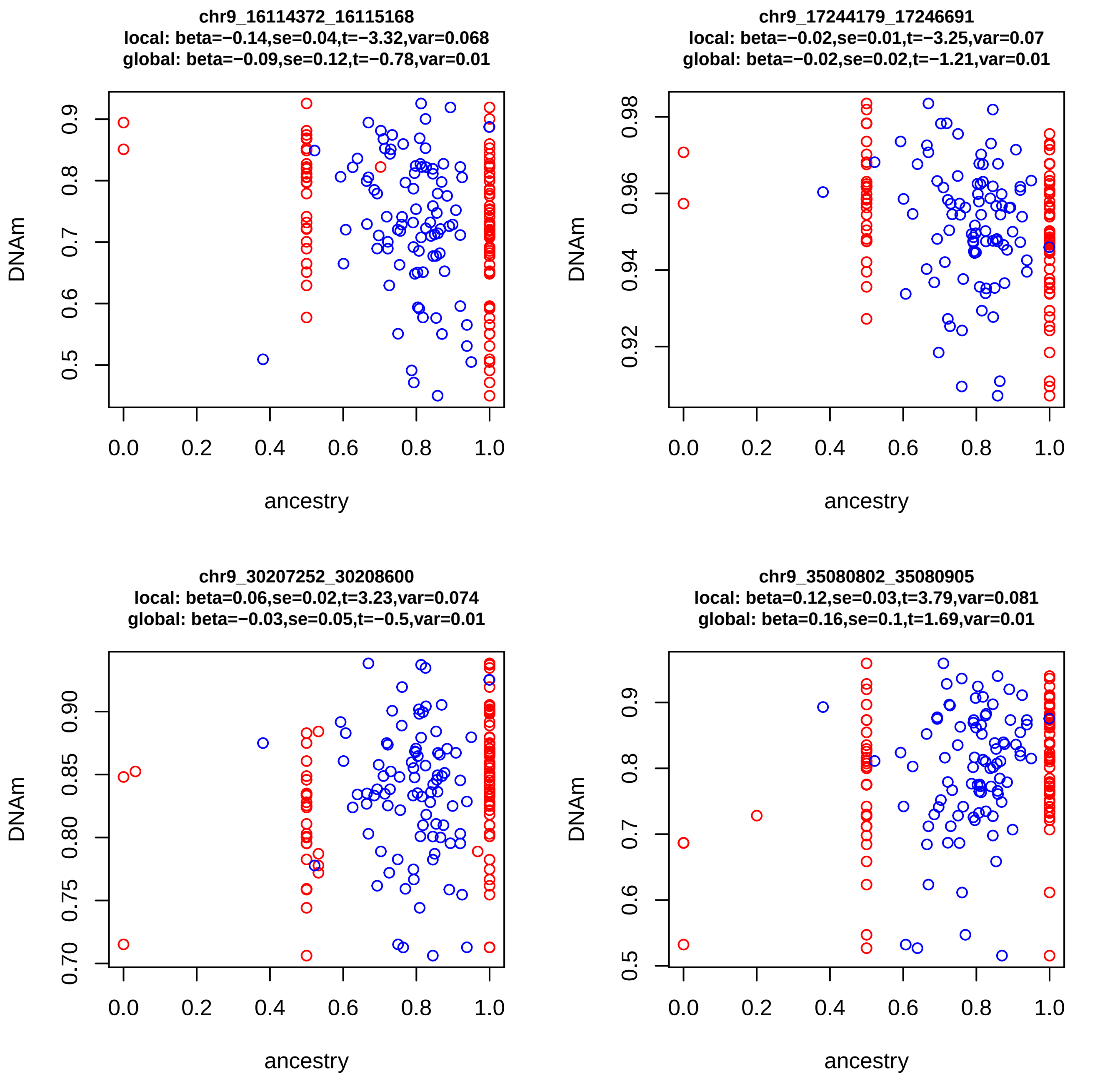
chr9_16114372_16115168
local: beta=−0.14,se=0.04,t=−3.32,var=0.068
global: beta=−0.09,se=0.12,t=−0.78,var=0.01
chr9_17244179_17246691
local: beta=−0.02,se=0.01,t=−3.25,var=0.07
global: beta=−0.02,se=0.02,t=−1.21,var=0.01
chr9_30207252_30208600
local: beta=0.06,se=0.02,t=3.23,var=0.074
global: beta=−0.03,se=0.05,t=−0.5,var=0.01
chr9_35080802_35080905
local: beta=0.12,se=0.03,t=3.79,var=0.081
global: beta=0.16,se=0.1,t=1.69,var=0.01
DNAm
ancestry
0.0
0.2
0.4
0.6
0.8
1.0

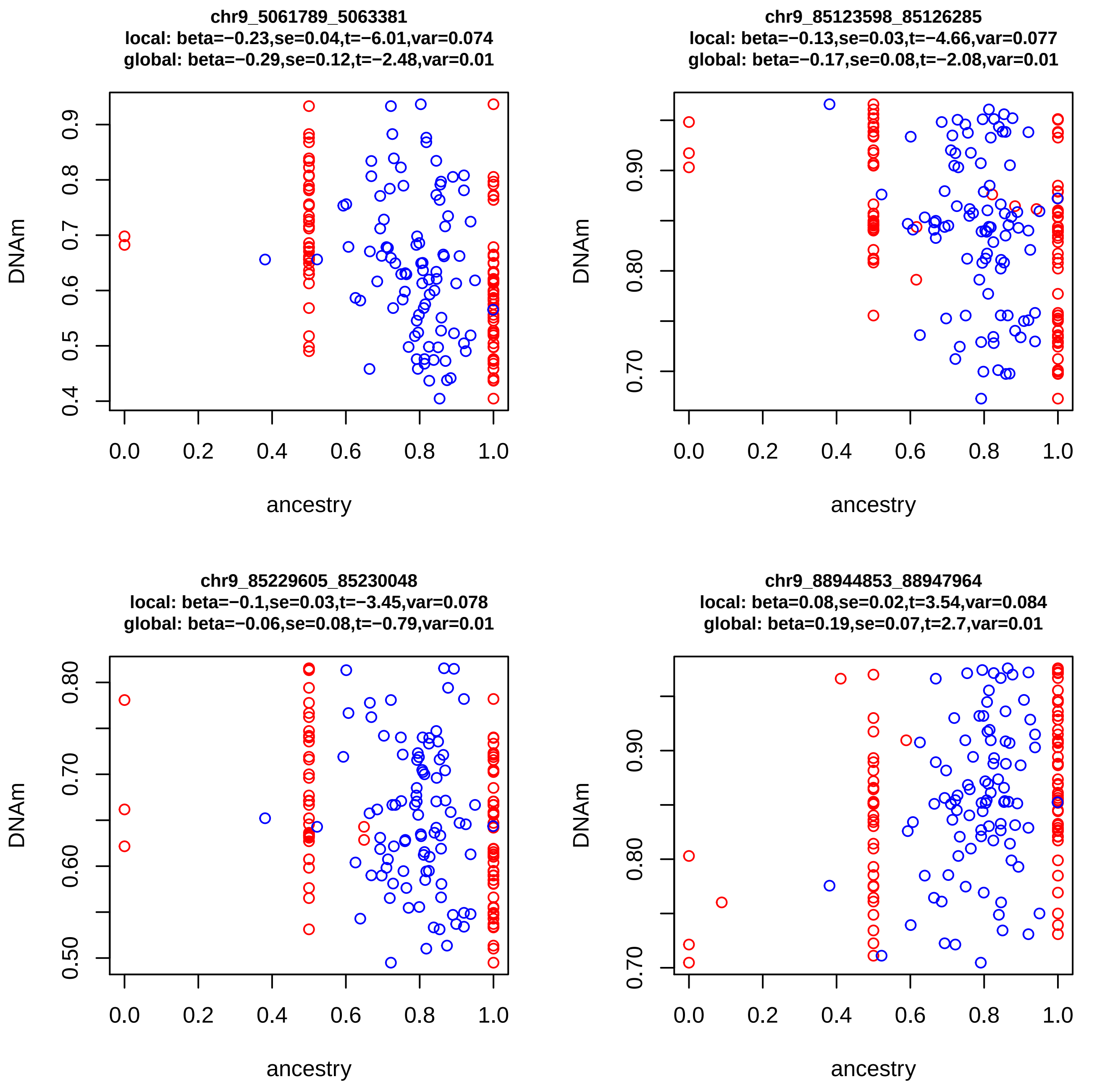

chr9_5061789_5063381
local: beta=−0.23,se=0.04,t=−6.01,var=0.074
global: beta=−0.29,se=0.12,t=−2.48,var=0.01
chr9_85123598_85126285
local: beta=−0.13,se=0.03,t=−4.66,var=0.077
global: beta=−0.17,se=0.08,t=−2.08,var=0.01
chr9_85229605_85230048
local: beta=−0.1,se=0.03,t=−3.45,var=0.078
global: beta=−0.06,se=0.08,t=−0.79,var=0.01
chr9_88944853_88947964
local: beta=0.08,se=0.02,t=3.54,var=0.084
global: beta=0.19,se=0.07,t=2.7,var=0.01
DNAm
ancestry
0.0
0.2
0.4
0.6
0.8
1.0
0.4
0.5
0.6
0.7
0.8
0.9
0.70
0.80
0.90
0.50
0.60

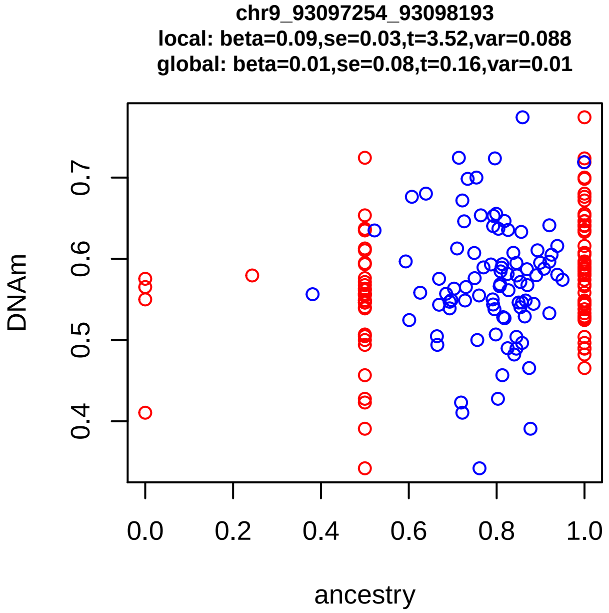
chr9_93097254_93098193
local: beta=0.09,se=0.03,t=3.52,var=0.088
global: beta=0.01,se=0.08,t=0.16,var=0.01
DNAm
0.4
0.5
0.6
0.7
0.0
0.2
0.4
0.6
0.8
1.0
ancestry

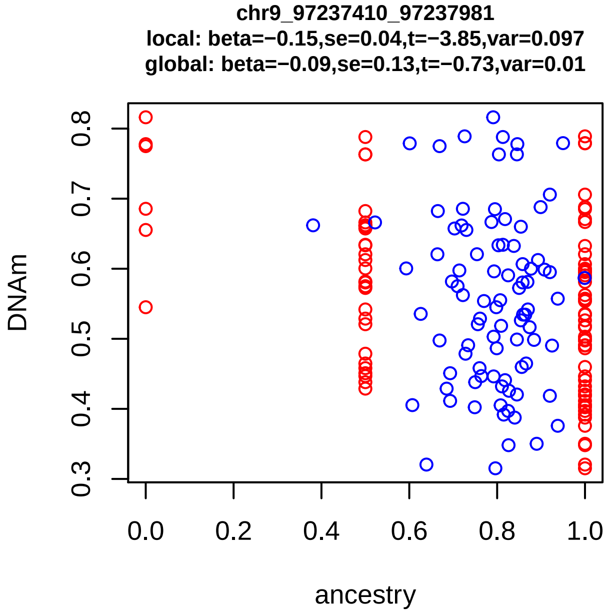
chr9_97237410_97237981
local: beta=−0.15,se=0.04,t=−3.85,var=0.097
global: beta=−0.09,se=0.13,t=−0.73,var=0.01
DNAm
0.3
0.4
0.5
0.6
0.7
0.8
0.0
0.2
0.4
0.6
0.8
1.0
ancestry
