# Supplementary material for: Analysis of gene expression in the postmortem brain of neurotypical Black Americans reveals contributions of genetic ancestry
Source: Nat Neurosci. 2024 May 20;27(6):1064–74. doi: 10.1038/s41593-024-01636-0 (PMC11156587; doi:10.1038/s41593-024-01636-0)
Supplement: Supplementary file 5 — Compressed directory of ancestry-associated DEGs enriched for WGCNA module functional enrichment results (that is, GO term enrichment) for the caudate nucleus, dentate gyrus, DLPFC and hippocampus. [file 41593_2024_1636_MOESM5_ESM.gz › wgcna_functional_enrichment/hippocampus/module_grey_go_enrichment.pdf]

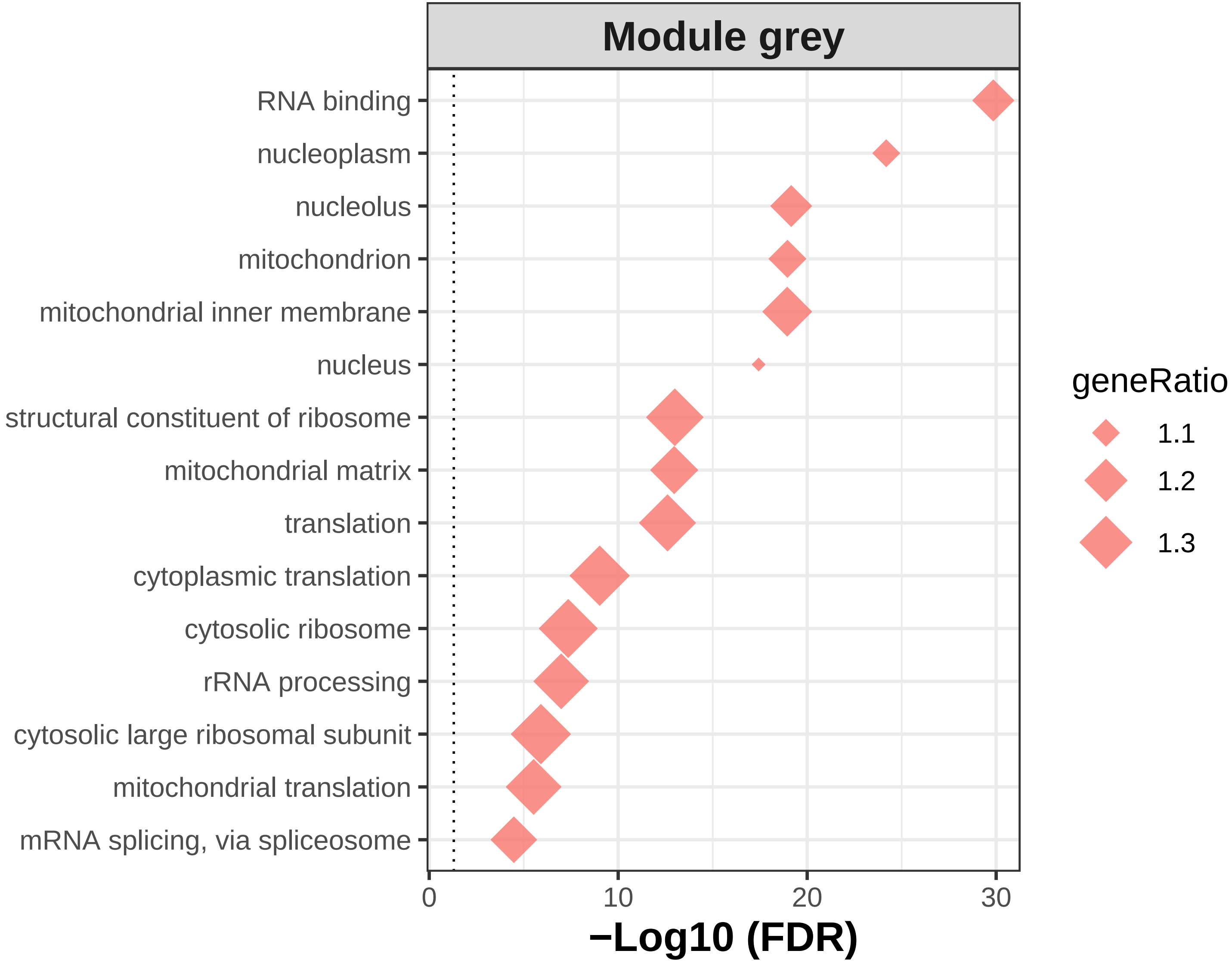

Module grey
RNA binding
nucleoplasm
nucleolus
mitochondrion
mitochondrial inner membrane
nucleus
structural constituent of ribosome
mitochondrial matrix
translation
cytoplasmic translation
cytosolic ribosome
rRNA processing
cytosolic large ribosomal subunit
mitochondrial translation
mRNA splicing, via spliceosome
0
10
20
30
−Log10 (FDR)
geneRatio
1.1
1.2
1.3
