# Supplementary material for: Analysis of gene expression in the postmortem brain of neurotypical Black Americans reveals contributions of genetic ancestry
Source: Nat Neurosci. 2024 May 20;27(6):1064–74. doi: 10.1038/s41593-024-01636-0 (PMC11156587; doi:10.1038/s41593-024-01636-0)
Supplement: Supplementary file 5 — Compressed directory of ancestry-associated DEGs enriched for WGCNA module functional enrichment results (that is, GO term enrichment) for the caudate nucleus, dentate gyrus, DLPFC and hippocampus. [file 41593_2024_1636_MOESM5_ESM.gz › wgcna_functional_enrichment/hippocampus/module_bisque4_go_enrichment.pdf]

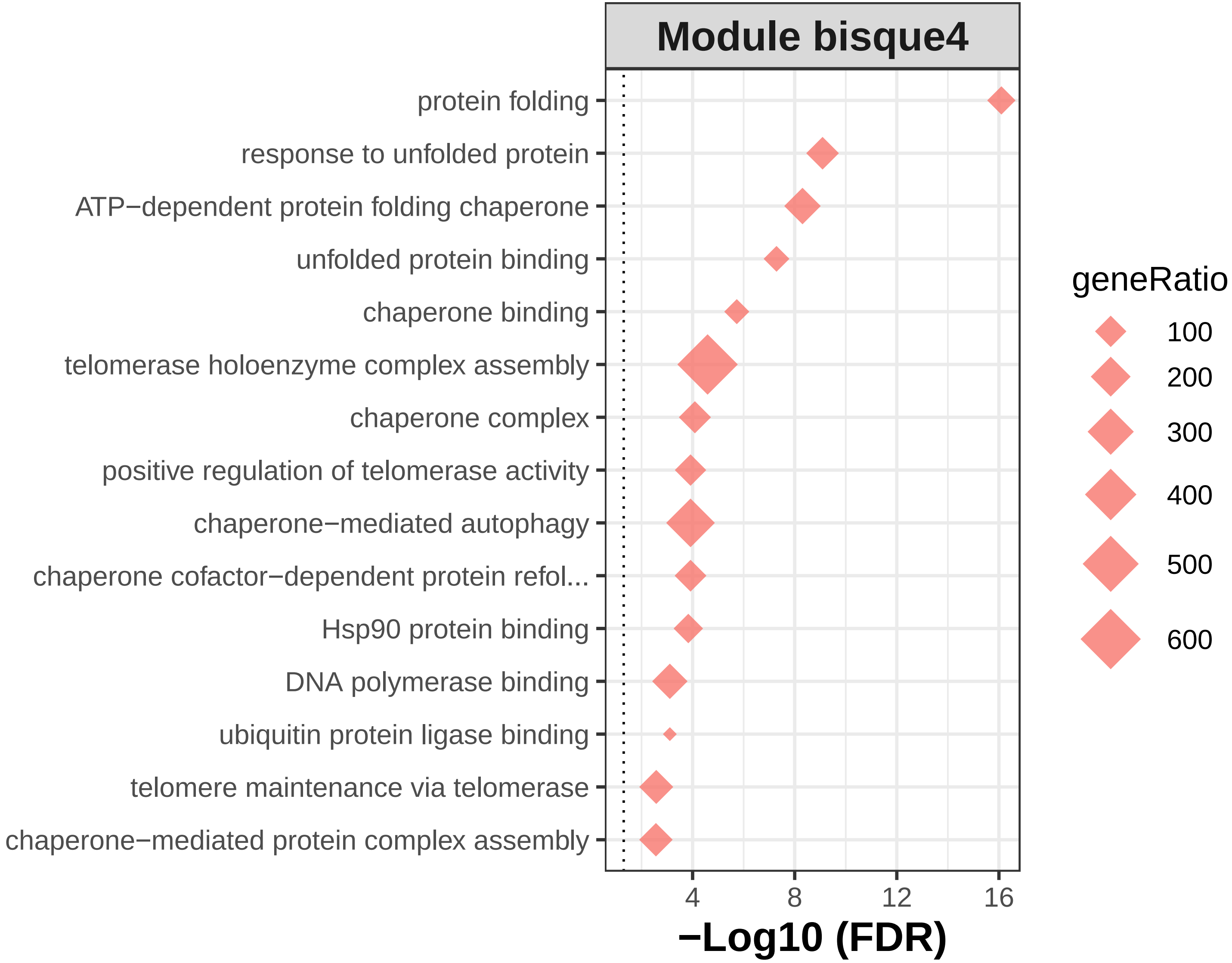

Module bisque4
protein folding
response to unfolded protein
ATP−dependent protein folding chaperone
unfolded protein binding
chaperone binding
telomerase holoenzyme complex assembly
chaperone complex
positive regulation of telomerase activity
chaperone−mediated autophagy
chaperone cofactor−dependent protein refol...
Hsp90 protein binding
DNA polymerase binding
ubiquitin protein ligase binding
telomere maintenance via telomerase
chaperone−mediated protein complex assembly
4
8
12
16
−Log10 (FDR)
geneRatio
100
200
300
400
500
600
