# Supplementary material for: Analysis of gene expression in the postmortem brain of neurotypical Black Americans reveals contributions of genetic ancestry
Source: Nat Neurosci. 2024 May 20;27(6):1064–74. doi: 10.1038/s41593-024-01636-0 (PMC11156587; doi:10.1038/s41593-024-01636-0)
Supplement: Supplementary file 5 — Compressed directory of ancestry-associated DEGs enriched for WGCNA module functional enrichment results (that is, GO term enrichment) for the caudate nucleus, dentate gyrus, DLPFC and hippocampus. [file 41593_2024_1636_MOESM5_ESM.gz › wgcna_functional_enrichment/hippocampus/module_plum1_go_enrichment.pdf]

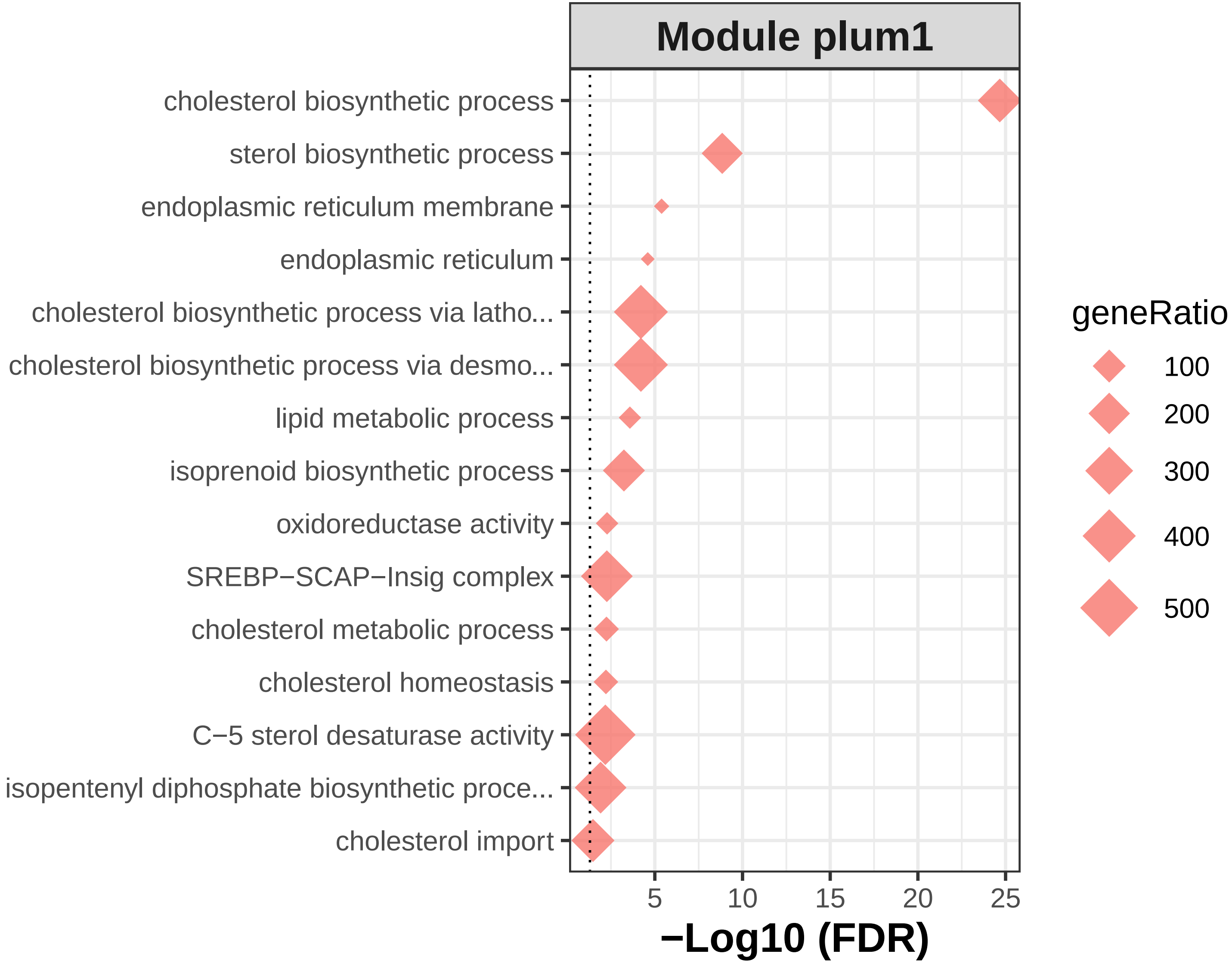
Module plum1
cholesterol biosynthetic process
sterol biosynthetic process
endoplasmic reticulum membrane
endoplasmic reticulum
cholesterol biosynthetic process via latho...
cholesterol biosynthetic process via desmo...
lipid metabolic process
isoprenoid biosynthetic process
oxidoreductase activity
SREBP−SCAP−Insig complex
cholesterol metabolic process
cholesterol homeostasis
C−5 sterol desaturase activity
isopentenyl diphosphate biosynthetic proce...
cholesterol import
5
10
15
20
25
−Log10 (FDR)
geneRatio
100
200
300
400
500
