# Supplementary material for: Analysis of gene expression in the postmortem brain of neurotypical Black Americans reveals contributions of genetic ancestry
Source: Nat Neurosci. 2024 May 20;27(6):1064–74. doi: 10.1038/s41593-024-01636-0 (PMC11156587; doi:10.1038/s41593-024-01636-0)
Supplement: Supplementary file 5 — Compressed directory of ancestry-associated DEGs enriched for WGCNA module functional enrichment results (that is, GO term enrichment) for the caudate nucleus, dentate gyrus, DLPFC and hippocampus. [file 41593_2024_1636_MOESM5_ESM.gz › wgcna_functional_enrichment/hippocampus/module_turquoise_go_enrichment.pdf]

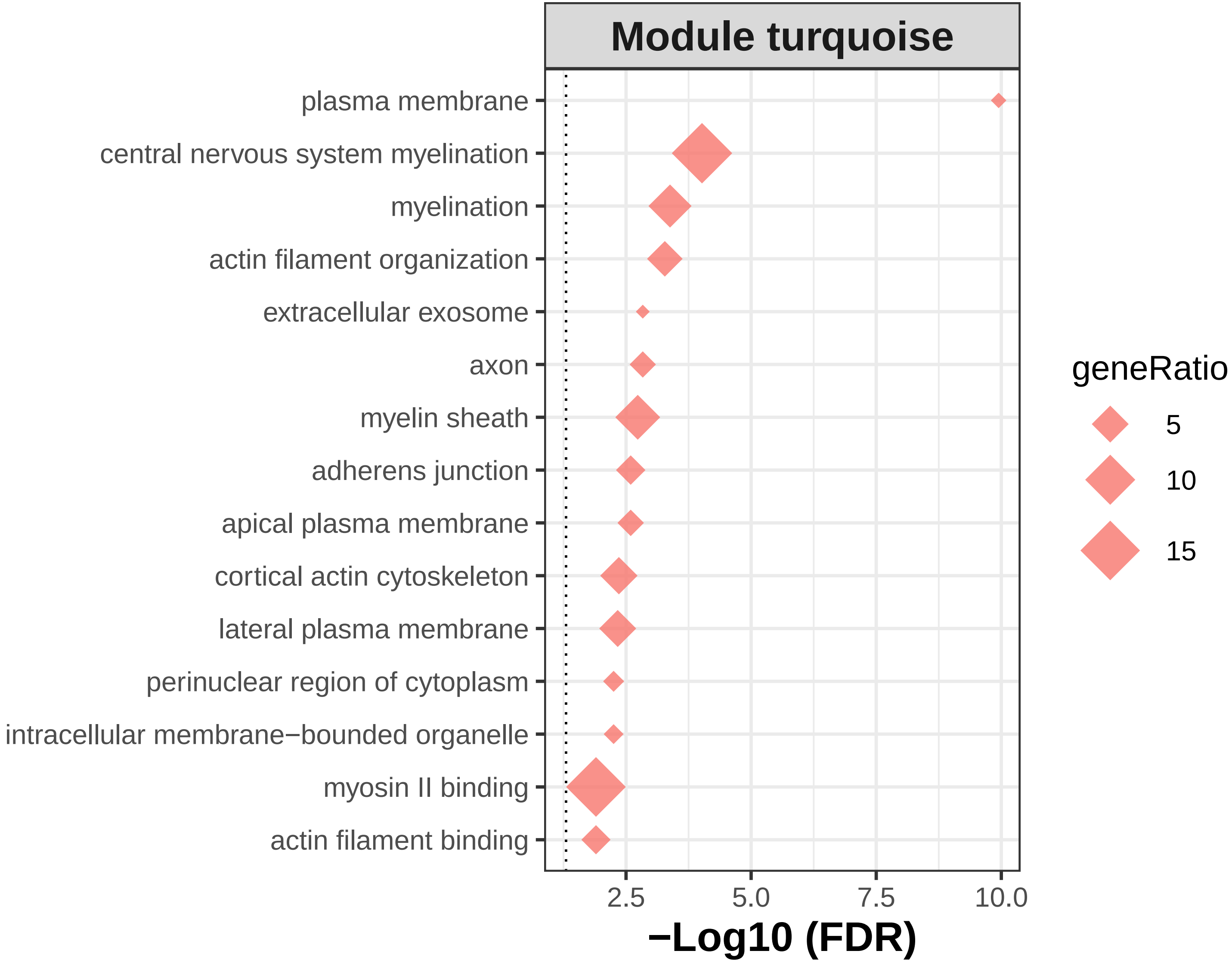
Module turquoise
plasma membrane
central nervous system myelination
myelination
actin filament organization
extracellular exosome
axon
myelin sheath
adherens junction
apical plasma membrane
cortical actin cytoskeleton
lateral plasma membrane
perinuclear region of cytoplasm
intracellular membrane−bounded organelle
myosin II binding
actin filament binding
2.5
5.0
7.5
10.0
−Log10 (FDR)
geneRatio
5
10
15
