# Supplementary material for: Analysis of gene expression in the postmortem brain of neurotypical Black Americans reveals contributions of genetic ancestry
Source: Nat Neurosci. 2024 May 20;27(6):1064–74. doi: 10.1038/s41593-024-01636-0 (PMC11156587; doi:10.1038/s41593-024-01636-0)
Supplement: Supplementary file 5 — Compressed directory of ancestry-associated DEGs enriched for WGCNA module functional enrichment results (that is, GO term enrichment) for the caudate nucleus, dentate gyrus, DLPFC and hippocampus. [file 41593_2024_1636_MOESM5_ESM.gz › wgcna_functional_enrichment/hippocampus/module_floralwhite_go_enrichment.pdf]

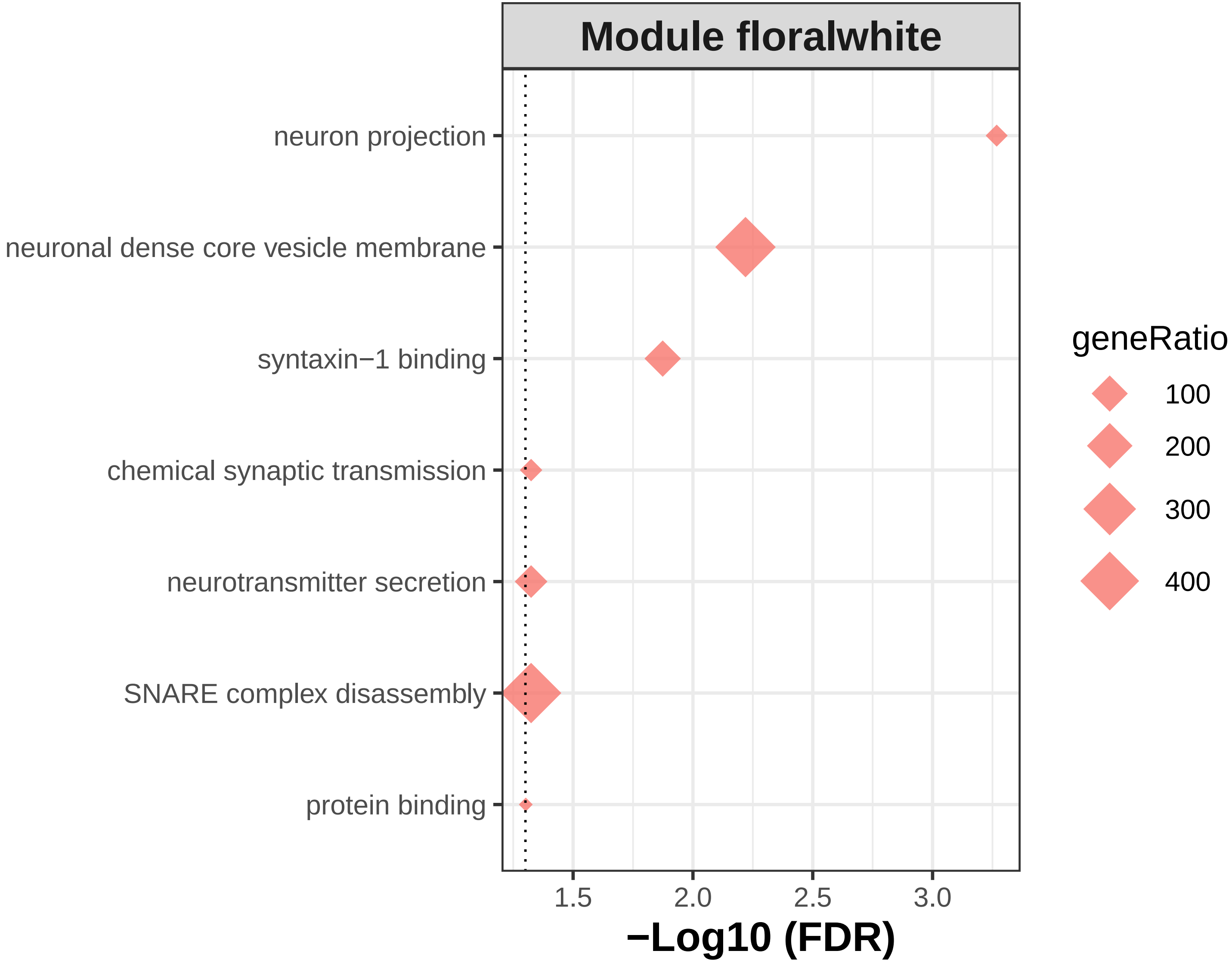
Module floralwhite
neuron projection
neuronal dense core vesicle membrane
syntaxin−1 binding
chemical synaptic transmission
neurotransmitter secretion
SNARE complex disassembly
protein binding
1.5
2.0
2.5
3.0
−Log10 (FDR)
geneRatio
100
200
300
400
