# Supplementary material for: Analysis of gene expression in the postmortem brain of neurotypical Black Americans reveals contributions of genetic ancestry
Source: Nat Neurosci. 2024 May 20;27(6):1064–74. doi: 10.1038/s41593-024-01636-0 (PMC11156587; doi:10.1038/s41593-024-01636-0)
Supplement: Supplementary file 5 — Compressed directory of ancestry-associated DEGs enriched for WGCNA module functional enrichment results (that is, GO term enrichment) for the caudate nucleus, dentate gyrus, DLPFC and hippocampus. [file 41593_2024_1636_MOESM5_ESM.gz › wgcna_functional_enrichment/hippocampus/module_lightgreen_go_enrichment.pdf]

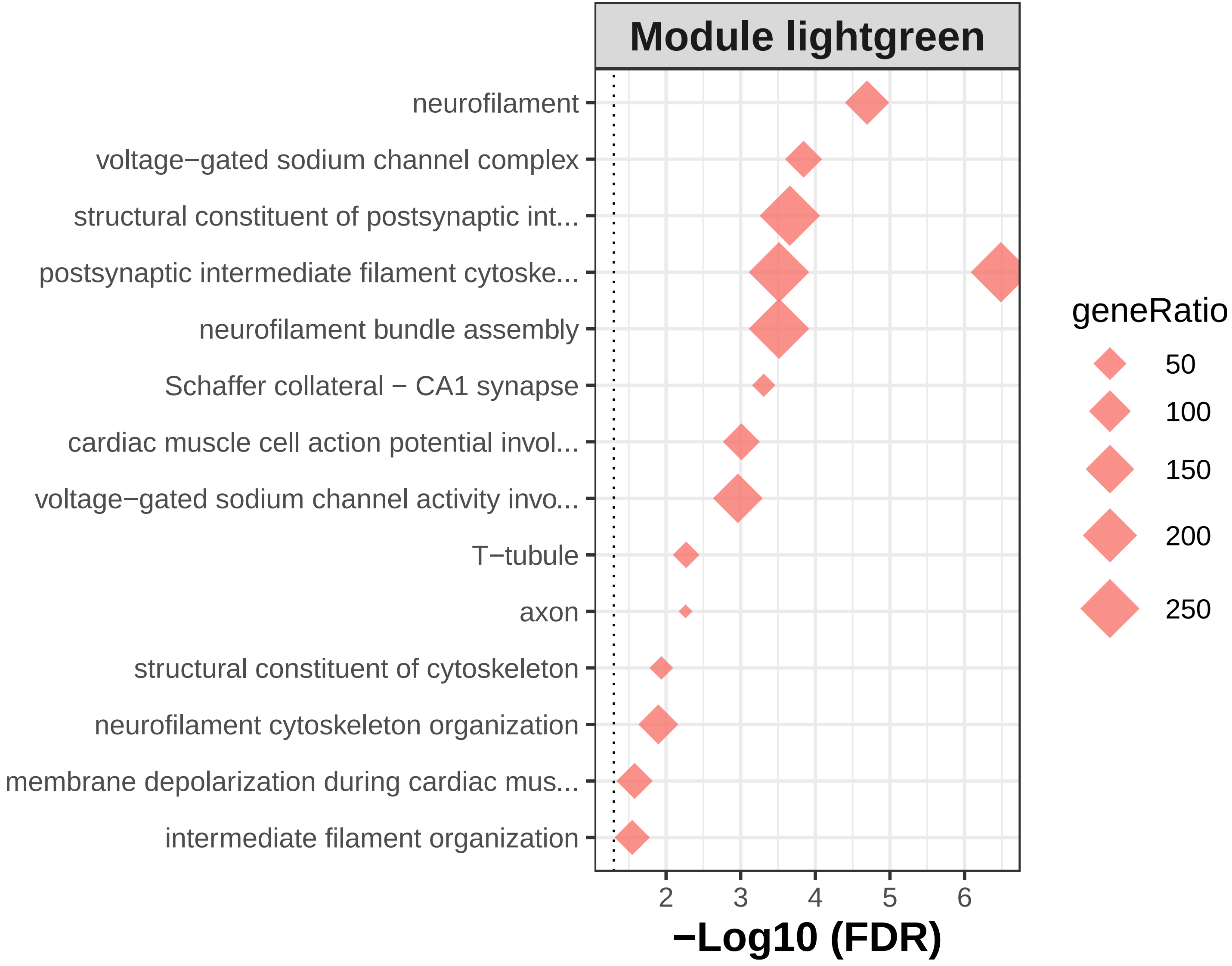
Module lightgreen
neurofilament
voltage−gated sodium channel complex
structural constituent of postsynaptic int...
postsynaptic intermediate filament cytoske...
neurofilament bundle assembly
Schaffer collateral − CA1 synapse
cardiac muscle cell action potential invol...
voltage−gated sodium channel activity invo...
T−tubule
axon
structural constituent of cytoskeleton
neurofilament cytoskeleton organization
membrane depolarization during cardiac mus...
intermediate filament organization
2
3
4
5
6
−Log10 (FDR)
geneRatio
50
100
150
200
250
