# Supplementary material for: Analysis of gene expression in the postmortem brain of neurotypical Black Americans reveals contributions of genetic ancestry
Source: Nat Neurosci. 2024 May 20;27(6):1064–74. doi: 10.1038/s41593-024-01636-0 (PMC11156587; doi:10.1038/s41593-024-01636-0)
Supplement: Supplementary file 5 — Compressed directory of ancestry-associated DEGs enriched for WGCNA module functional enrichment results (that is, GO term enrichment) for the caudate nucleus, dentate gyrus, DLPFC and hippocampus. [file 41593_2024_1636_MOESM5_ESM.gz › wgcna_functional_enrichment/caudate/module_grey_go_enrichment.pdf]

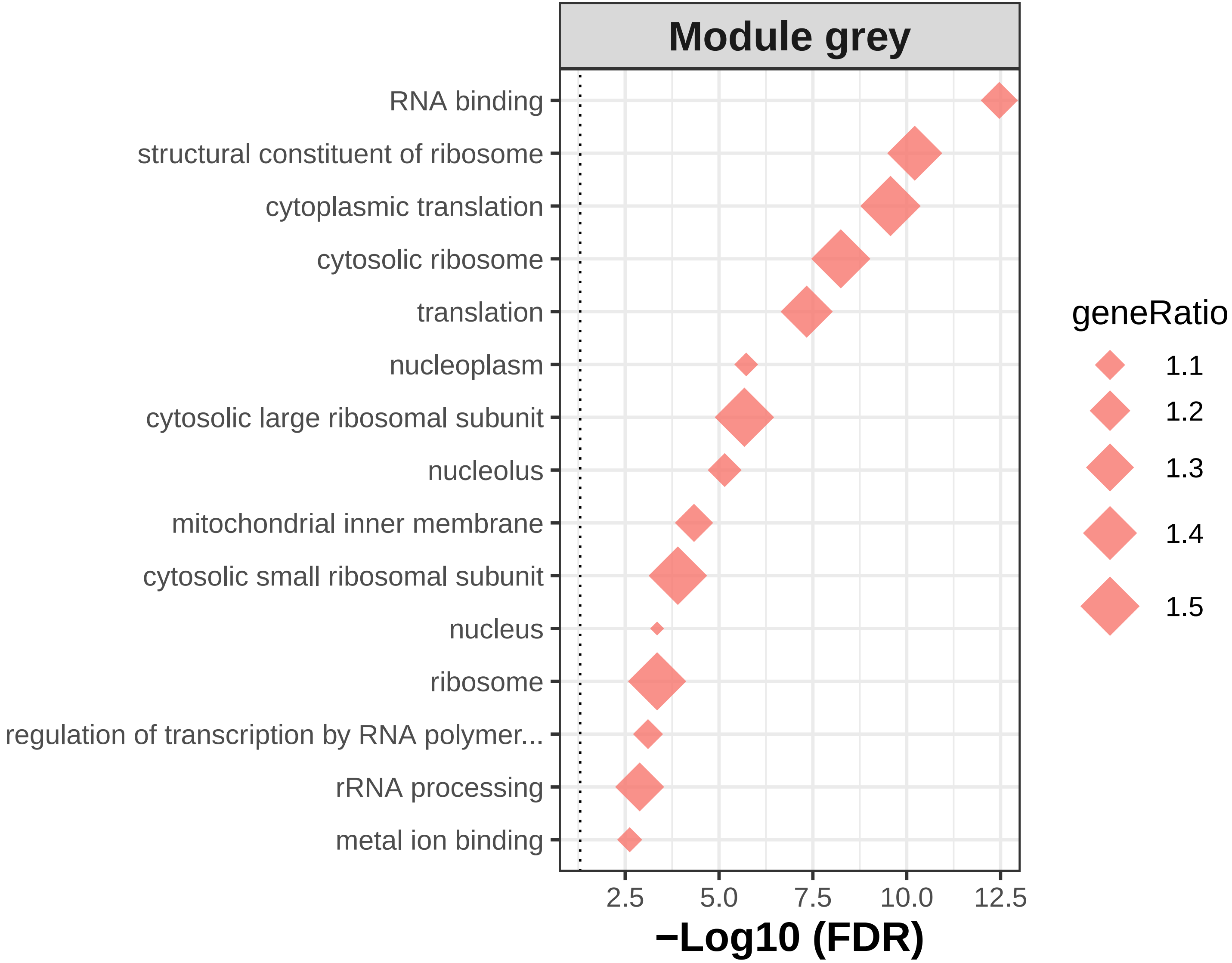

Module grey
RNA binding
structural constituent of ribosome
cytoplasmic translation
cytosolic ribosome
translation
nucleoplasm
cytosolic large ribosomal subunit
nucleolus
mitochondrial inner membrane
cytosolic small ribosomal subunit
nucleus
ribosome
regulation of transcription by RNA polymer...
rRNA processing
metal ion binding
2.5
5.0
7.5
10.0
12.5
−Log10 (FDR)
geneRatio
1.1
1.2
1.3
1.4
1.5
