# Supplementary material for: Analysis of gene expression in the postmortem brain of neurotypical Black Americans reveals contributions of genetic ancestry
Source: Nat Neurosci. 2024 May 20;27(6):1064–74. doi: 10.1038/s41593-024-01636-0 (PMC11156587; doi:10.1038/s41593-024-01636-0)
Supplement: Supplementary file 5 — Compressed directory of ancestry-associated DEGs enriched for WGCNA module functional enrichment results (that is, GO term enrichment) for the caudate nucleus, dentate gyrus, DLPFC and hippocampus. [file 41593_2024_1636_MOESM5_ESM.gz › wgcna_functional_enrichment/caudate/module_yellow_go_enrichment.pdf]

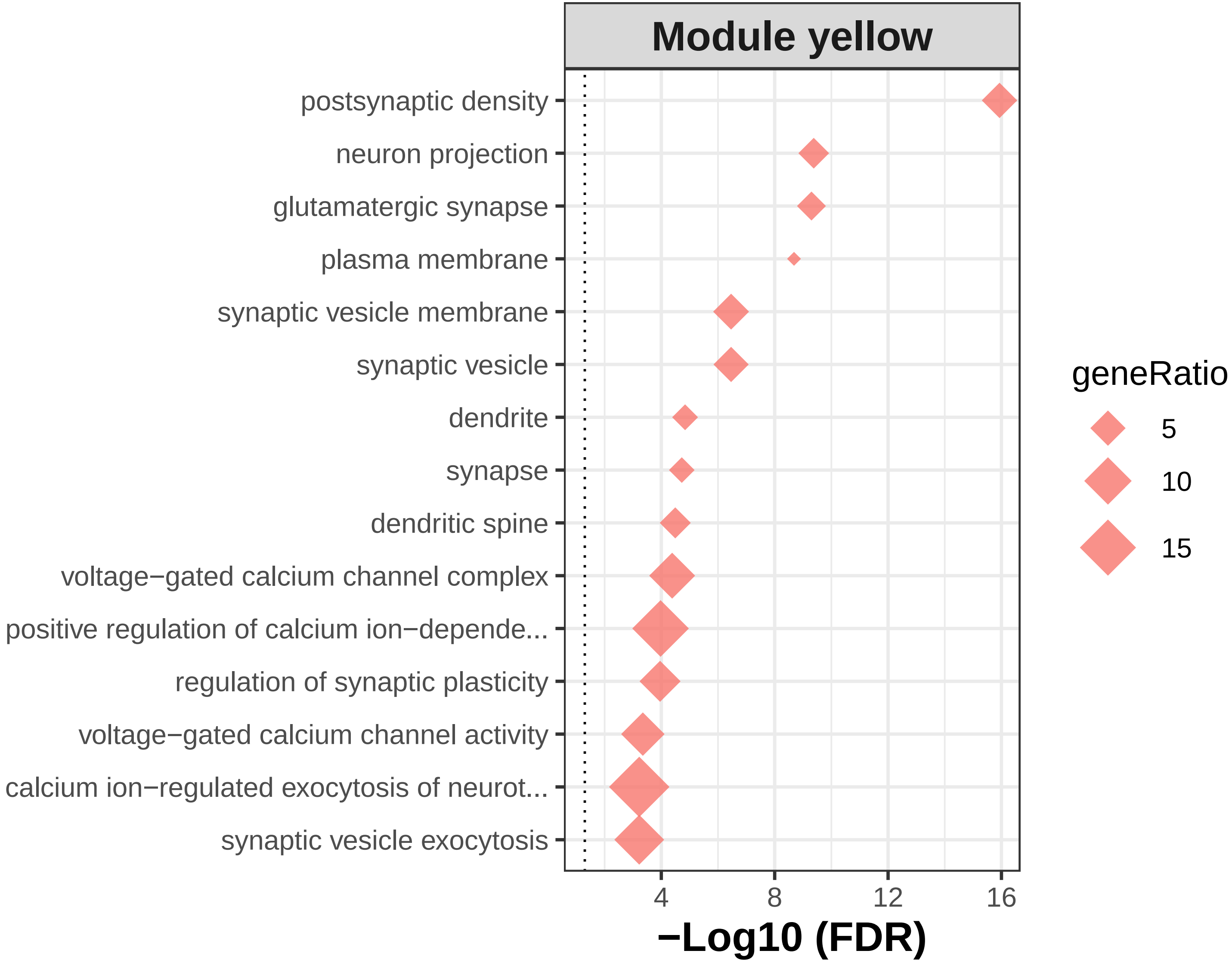

Module yellow
postsynaptic density
neuron projection
glutamatergic synapse
plasma membrane
synaptic vesicle membrane
synaptic vesicle
dendrite
synapse
dendritic spine
voltage−gated calcium channel complex
positive regulation of calcium ion−depende...
regulation of synaptic plasticity
voltage−gated calcium channel activity
calcium ion−regulated exocytosis of neurot...
synaptic vesicle exocytosis
4
8
12
16
−Log10 (FDR)
geneRatio
5
10
15
