# Supplementary material for: Analysis of gene expression in the postmortem brain of neurotypical Black Americans reveals contributions of genetic ancestry
Source: Nat Neurosci. 2024 May 20;27(6):1064–74. doi: 10.1038/s41593-024-01636-0 (PMC11156587; doi:10.1038/s41593-024-01636-0)
Supplement: Supplementary file 5 — Compressed directory of ancestry-associated DEGs enriched for WGCNA module functional enrichment results (that is, GO term enrichment) for the caudate nucleus, dentate gyrus, DLPFC and hippocampus. [file 41593_2024_1636_MOESM5_ESM.gz › wgcna_functional_enrichment/caudate/module_black_go_enrichment.pdf]

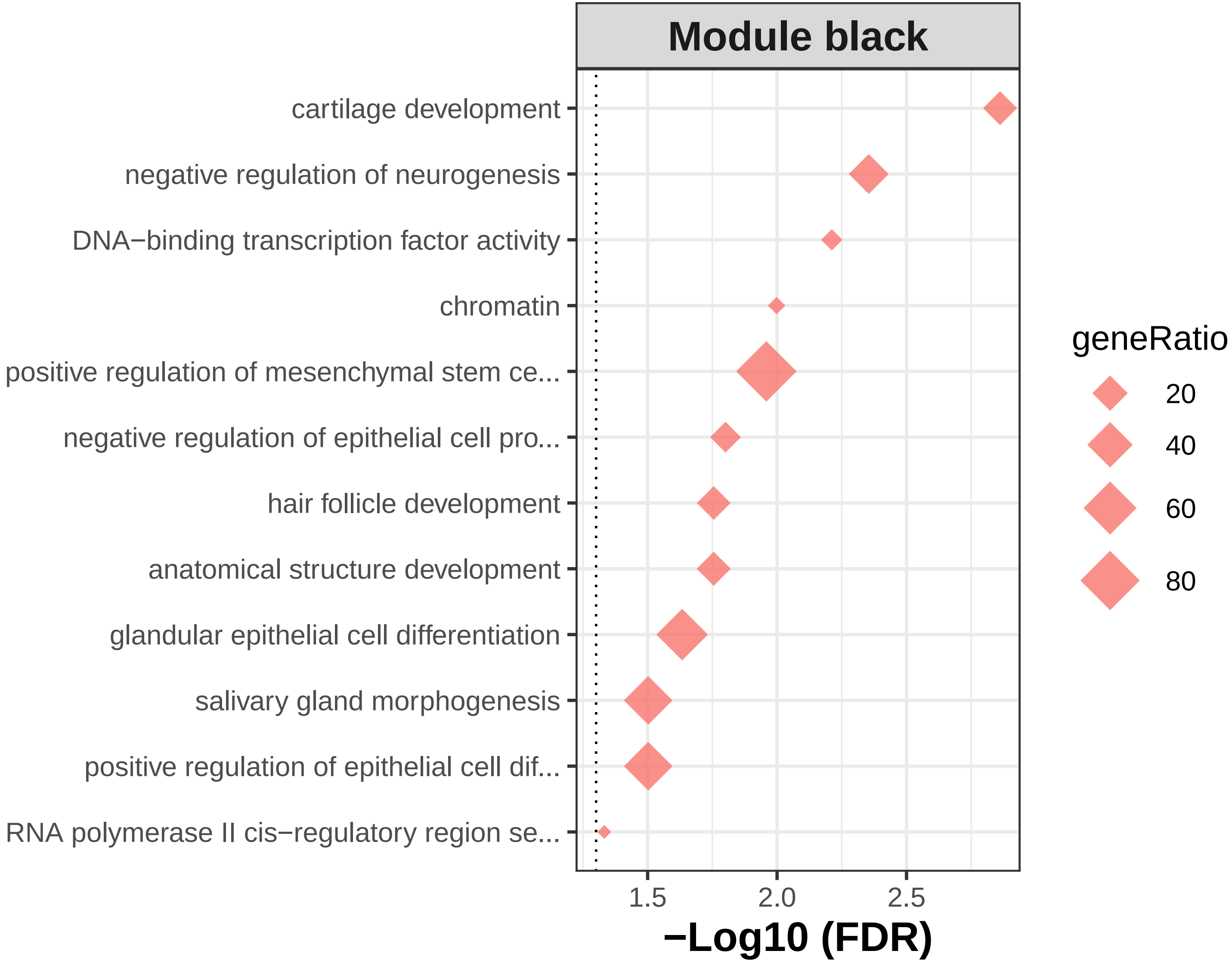
Module black
cartilage development
negative regulation of neurogenesis
DNA−binding transcription factor activity
chromatin
positive regulation of mesenchymal stem ce...
negative regulation of epithelial cell pro...
hair follicle development
anatomical structure development
glandular epithelial cell differentiation
salivary gland morphogenesis
positive regulation of epithelial cell dif...
RNA polymerase II cis−regulatory region se...
1.5
2.0
2.5
−Log10 (FDR)
geneRatio
20
40
60
80
