# Supplementary material for: Analysis of gene expression in the postmortem brain of neurotypical Black Americans reveals contributions of genetic ancestry
Source: Nat Neurosci. 2024 May 20;27(6):1064–74. doi: 10.1038/s41593-024-01636-0 (PMC11156587; doi:10.1038/s41593-024-01636-0)
Supplement: Supplementary file 5 — Compressed directory of ancestry-associated DEGs enriched for WGCNA module functional enrichment results (that is, GO term enrichment) for the caudate nucleus, dentate gyrus, DLPFC and hippocampus. [file 41593_2024_1636_MOESM5_ESM.gz › wgcna_functional_enrichment/caudate/module_brown_go_enrichment.pdf]

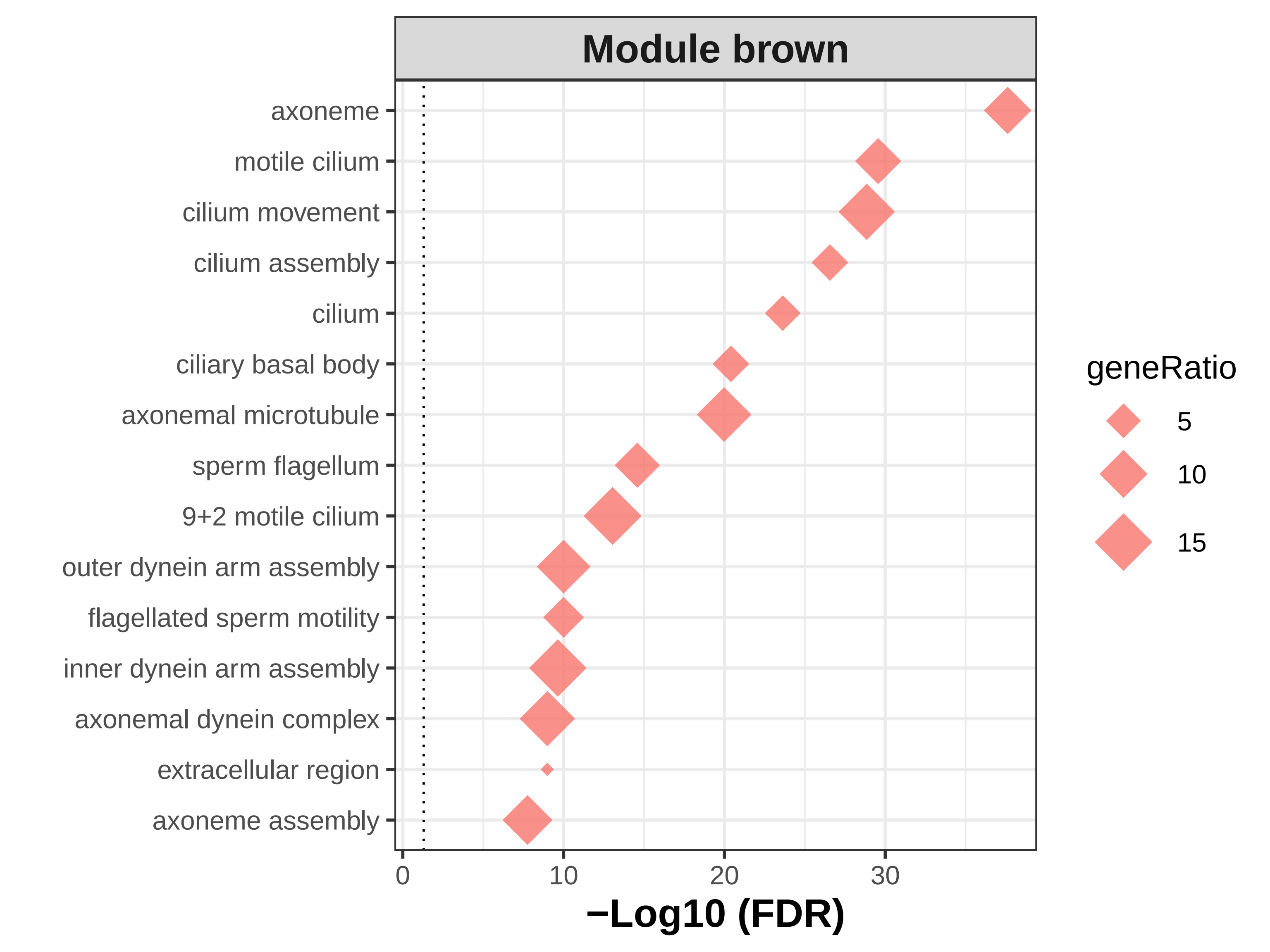

Module brown
axoneme
motile cilium
cilium movement
cilium assembly
cilium
ciliary basal body
axonemal microtubule
sperm flagellum
9+2 motile cilium
outer dynein arm assembly
flagellated sperm motility
inner dynein arm assembly
axonemal dynein complex
extracellular region
axoneme assembly
0
10
20
30
−Log10 (FDR)
geneRatio
5
10
15
