# Supplementary material for: Analysis of gene expression in the postmortem brain of neurotypical Black Americans reveals contributions of genetic ancestry
Source: Nat Neurosci. 2024 May 20;27(6):1064–74. doi: 10.1038/s41593-024-01636-0 (PMC11156587; doi:10.1038/s41593-024-01636-0)
Supplement: Supplementary file 5 — Compressed directory of ancestry-associated DEGs enriched for WGCNA module functional enrichment results (that is, GO term enrichment) for the caudate nucleus, dentate gyrus, DLPFC and hippocampus. [file 41593_2024_1636_MOESM5_ESM.gz › wgcna_functional_enrichment/caudate/module_cyan_go_enrichment.pdf]

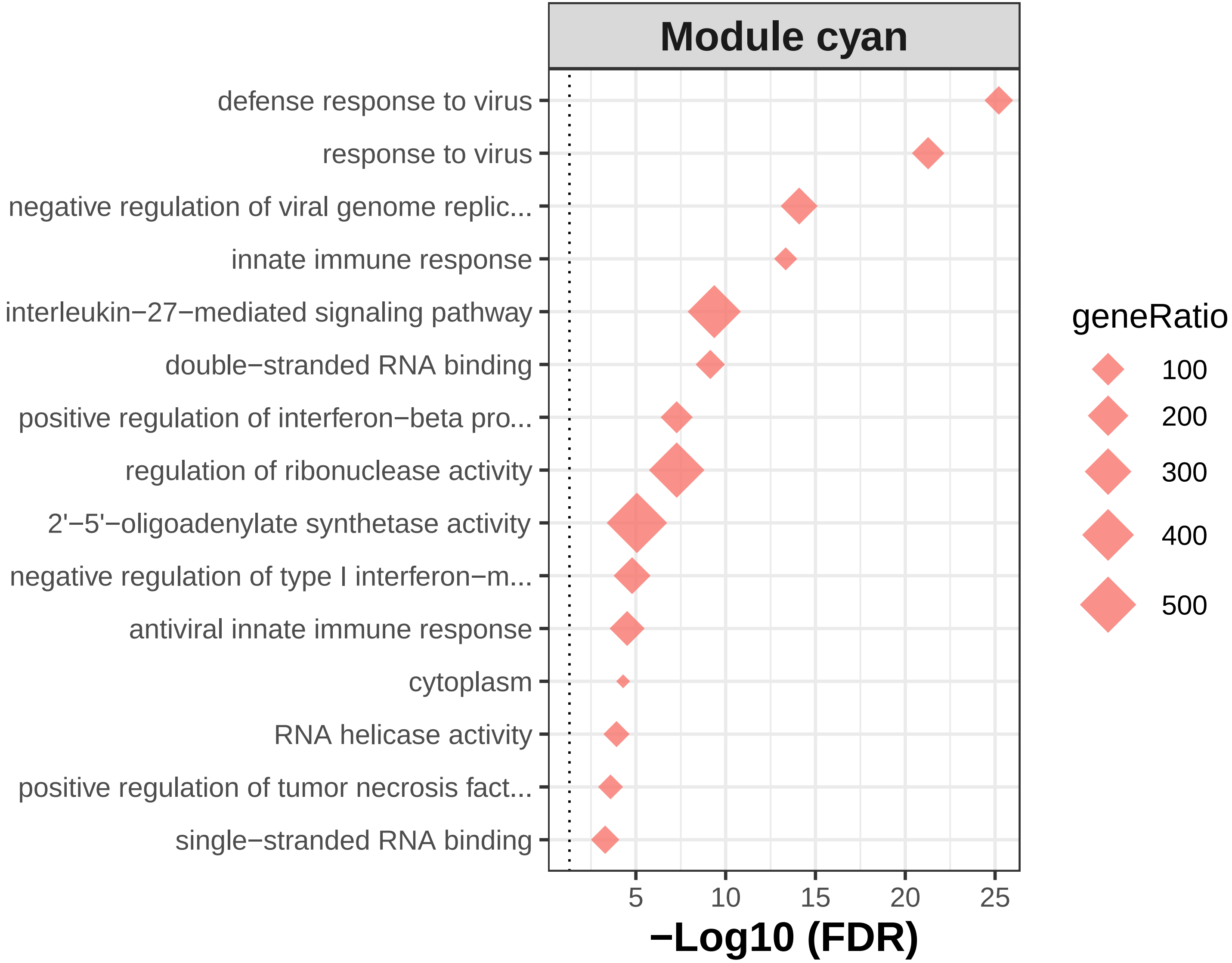
Module cyan
defense response to virus
response to virus
negative regulation of viral genome replic...
innate immune response
interleukin−27−mediated signaling pathway
double−stranded RNA binding
positive regulation of interferon−beta pro...
regulation of ribonuclease activity
2'−5'−oligoadenylate synthetase activity
negative regulation of type I interferon−m...
antiviral innate immune response
cytoplasm
RNA helicase activity
positive regulation of tumor necrosis fact...
single−stranded RNA binding
5
10
15
20
25
−Log10 (FDR)
geneRatio
100
200
300
400
500
