# Supplementary material for: Analysis of gene expression in the postmortem brain of neurotypical Black Americans reveals contributions of genetic ancestry
Source: Nat Neurosci. 2024 May 20;27(6):1064–74. doi: 10.1038/s41593-024-01636-0 (PMC11156587; doi:10.1038/s41593-024-01636-0)
Supplement: Supplementary file 5 — Compressed directory of ancestry-associated DEGs enriched for WGCNA module functional enrichment results (that is, GO term enrichment) for the caudate nucleus, dentate gyrus, DLPFC and hippocampus. [file 41593_2024_1636_MOESM5_ESM.gz › wgcna_functional_enrichment/caudate/module_turquoise_go_enrichment.pdf]

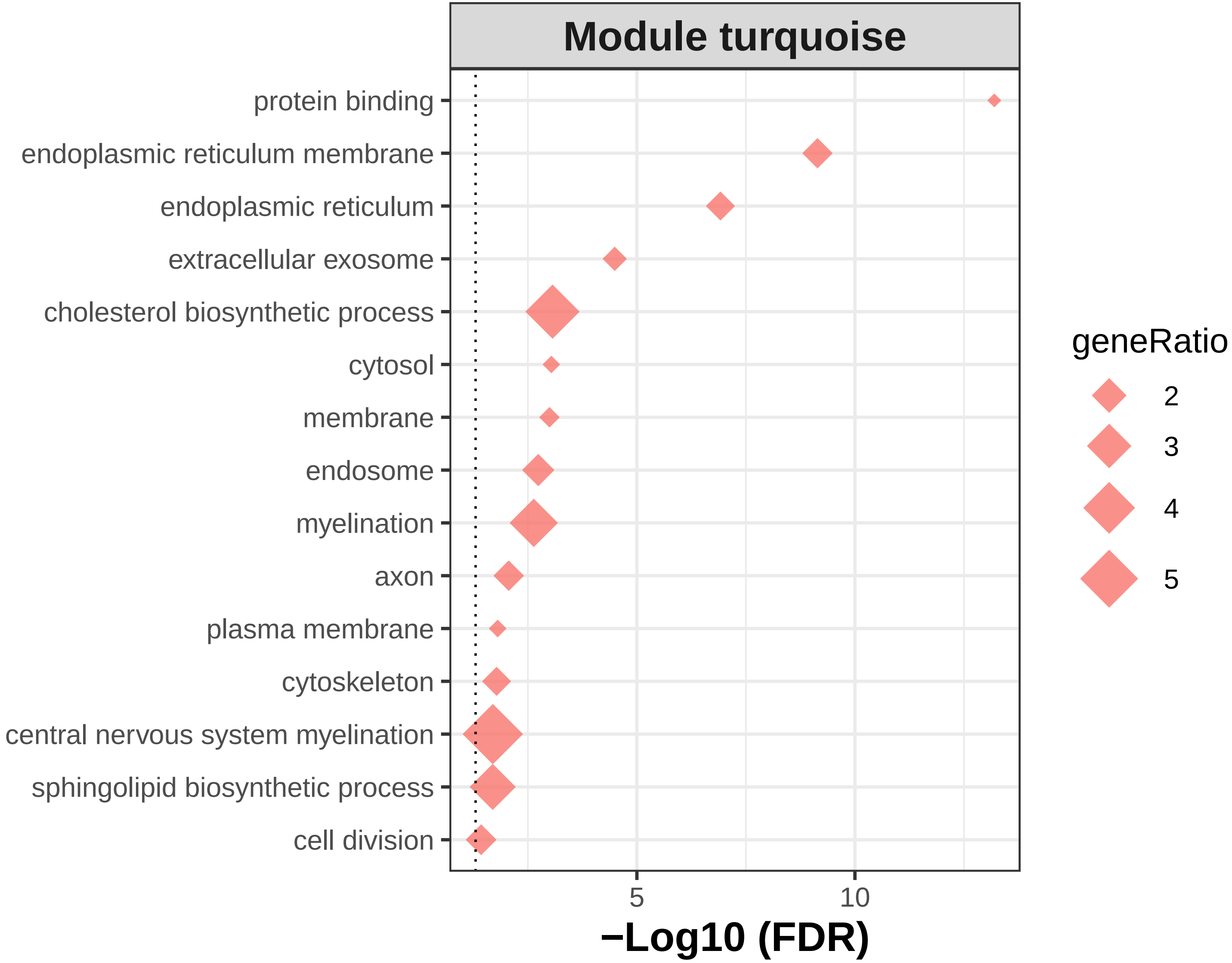
Module turquoise
protein binding
endoplasmic reticulum membrane
endoplasmic reticulum
extracellular exosome
cholesterol biosynthetic process
cytosol
membrane
endosome
myelination
axon
plasma membrane
cytoskeleton
central nervous system myelination
sphingolipid biosynthetic process
cell division
5
10
−Log10 (FDR)
geneRatio
2
3
4
5
