# Supplementary material for: Analysis of gene expression in the postmortem brain of neurotypical Black Americans reveals contributions of genetic ancestry
Source: Nat Neurosci. 2024 May 20;27(6):1064–74. doi: 10.1038/s41593-024-01636-0 (PMC11156587; doi:10.1038/s41593-024-01636-0)
Supplement: Supplementary file 5 — Compressed directory of ancestry-associated DEGs enriched for WGCNA module functional enrichment results (that is, GO term enrichment) for the caudate nucleus, dentate gyrus, DLPFC and hippocampus. [file 41593_2024_1636_MOESM5_ESM.gz › wgcna_functional_enrichment/caudate/module_greenyellow_go_enrichment.pdf]

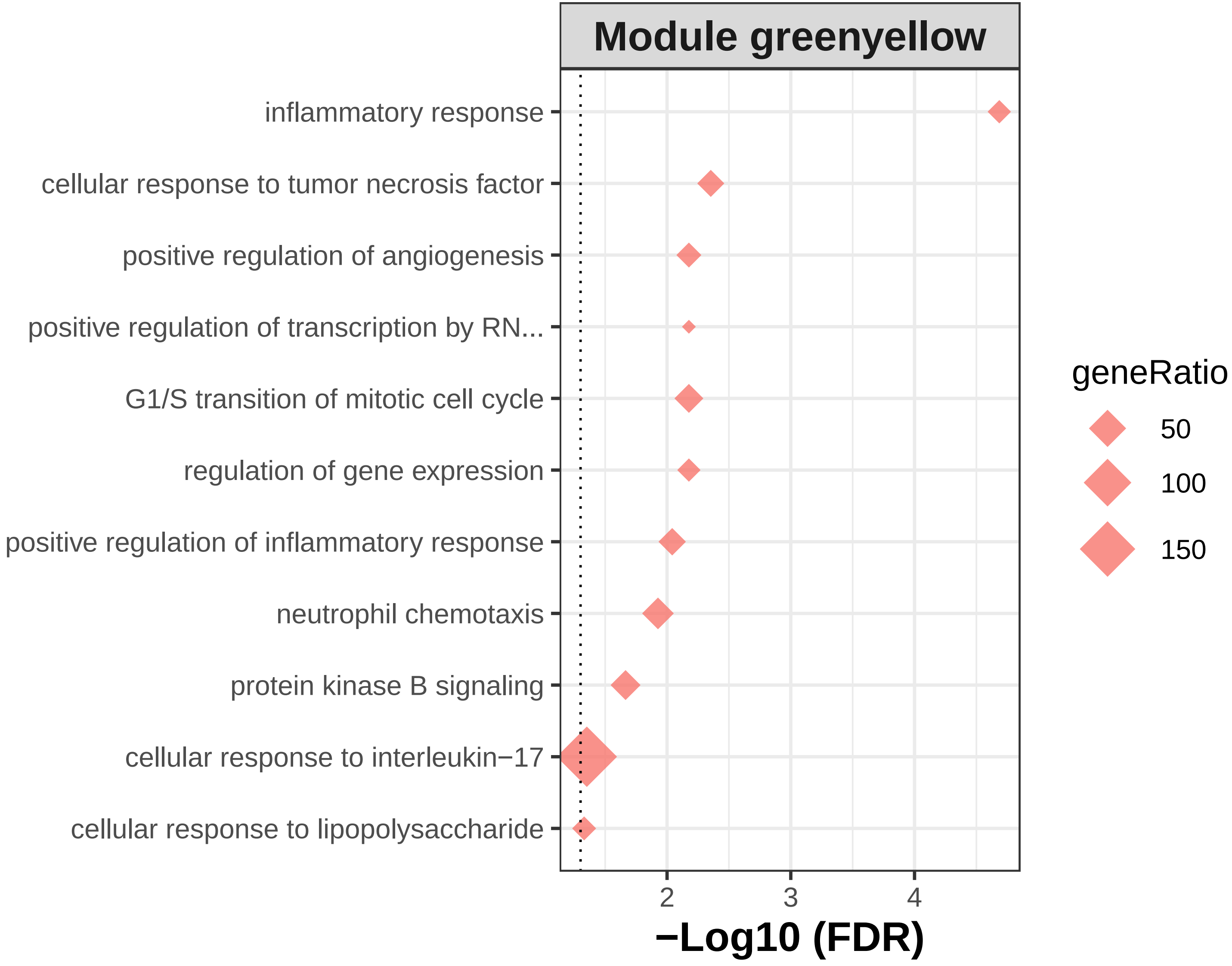
Module greenyellow
inflammatory response
cellular response to tumor necrosis factor
positive regulation of angiogenesis
positive regulation of transcription by RN...
G1/S transition of mitotic cell cycle
regulation of gene expression
positive regulation of inflammatory response
neutrophil chemotaxis
protein kinase B signaling
cellular response to interleukin−17
cellular response to lipopolysaccharide
2
3
4
−Log10 (FDR)
geneRatio
50
100
150
