# Supplementary material for: Analysis of gene expression in the postmortem brain of neurotypical Black Americans reveals contributions of genetic ancestry
Source: Nat Neurosci. 2024 May 20;27(6):1064–74. doi: 10.1038/s41593-024-01636-0 (PMC11156587; doi:10.1038/s41593-024-01636-0)
Supplement: Supplementary file 5 — Compressed directory of ancestry-associated DEGs enriched for WGCNA module functional enrichment results (that is, GO term enrichment) for the caudate nucleus, dentate gyrus, DLPFC and hippocampus. [file 41593_2024_1636_MOESM5_ESM.gz › wgcna_functional_enrichment/caudate/module_tan_go_enrichment.pdf]

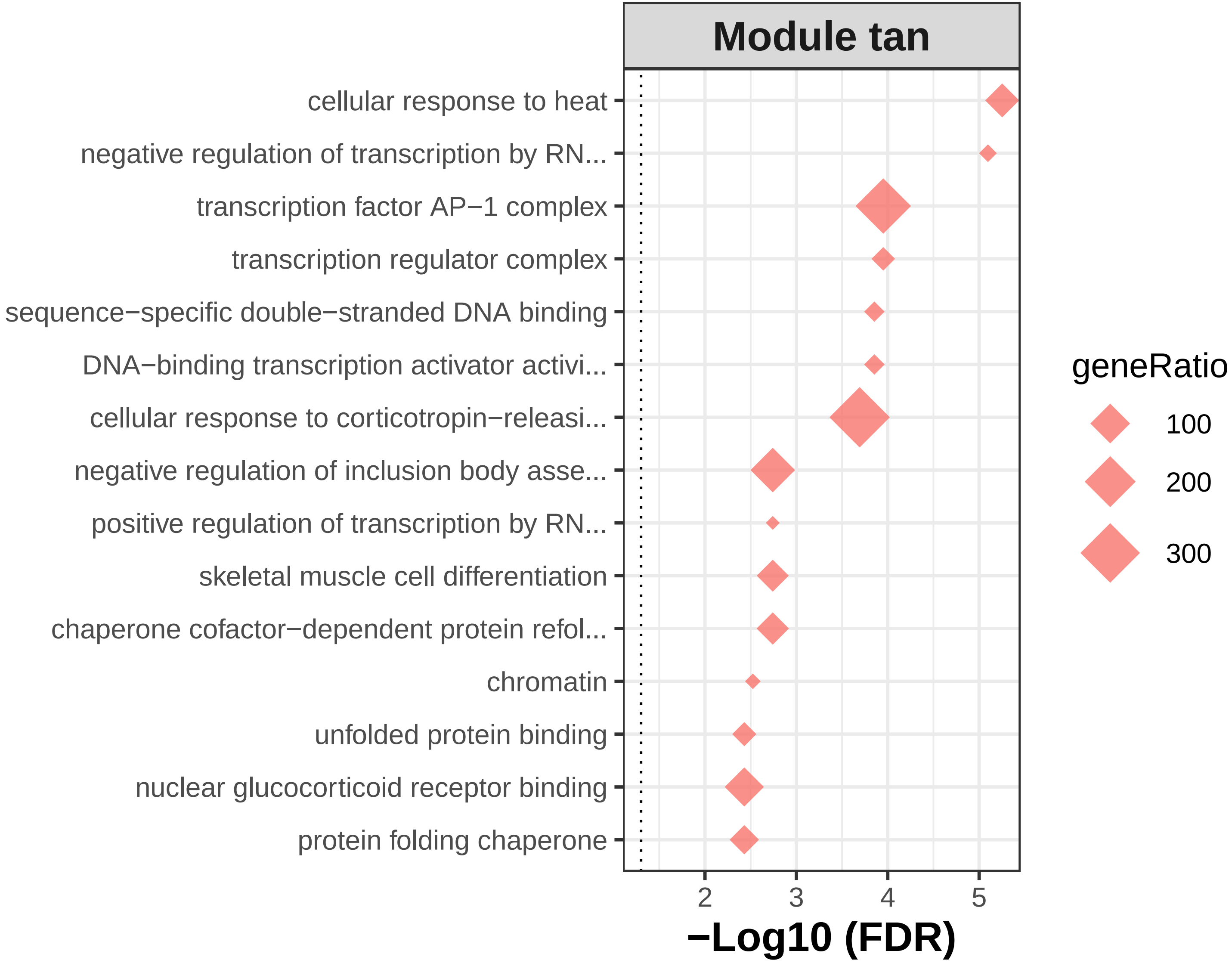
Module tan
cellular response to heat
negative regulation of transcription by RN...
transcription factor AP−1 complex
transcription regulator complex
sequence−specific double−stranded DNA binding
DNA−binding transcription activator activi...
cellular response to corticotropin−releasi...
negative regulation of inclusion body asse...
positive regulation of transcription by RN...
skeletal muscle cell differentiation
chaperone cofactor−dependent protein refol...
chromatin
unfolded protein binding
nuclear glucocorticoid receptor binding
protein folding chaperone
2
3
4
5
−Log10 (FDR)
geneRatio
100
200
300
