# Supplementary material for: Analysis of gene expression in the postmortem brain of neurotypical Black Americans reveals contributions of genetic ancestry
Source: Nat Neurosci. 2024 May 20;27(6):1064–74. doi: 10.1038/s41593-024-01636-0 (PMC11156587; doi:10.1038/s41593-024-01636-0)
Supplement: Supplementary file 5 — Compressed directory of ancestry-associated DEGs enriched for WGCNA module functional enrichment results (that is, GO term enrichment) for the caudate nucleus, dentate gyrus, DLPFC and hippocampus. [file 41593_2024_1636_MOESM5_ESM.gz › wgcna_functional_enrichment/caudate/module_blue_go_enrichment.pdf]

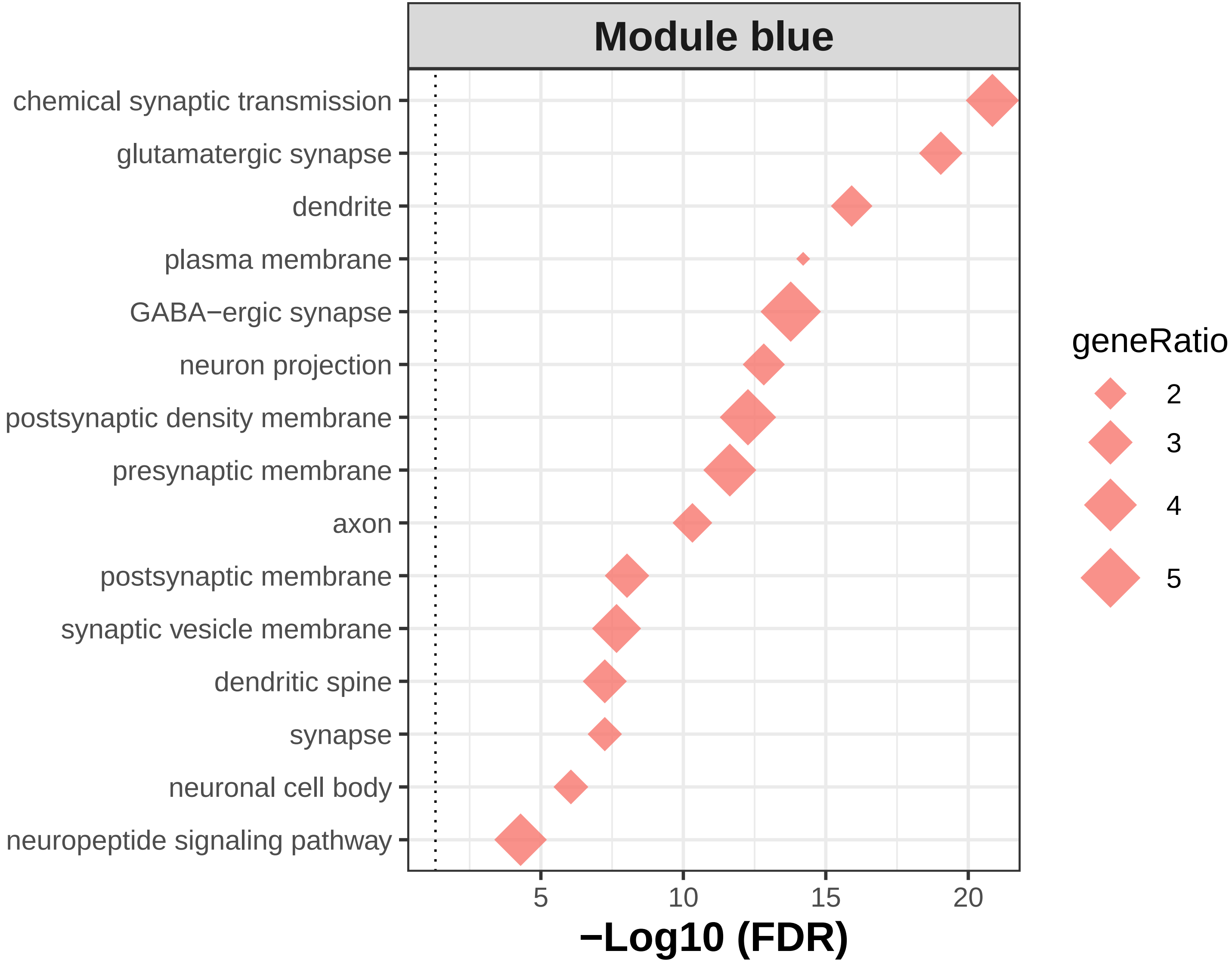
Module blue
chemical synaptic transmission
glutamatergic synapse
dendrite
plasma membrane
GABA−ergic synapse
neuron projection
postsynaptic density membrane
presynaptic membrane
axon
postsynaptic membrane
synaptic vesicle membrane
dendritic spine
synapse
neuronal cell body
neuropeptide signaling pathway
5
10
15
20
−Log10 (FDR)
geneRatio
2
3
4
5
