# Supplementary material for: Analysis of gene expression in the postmortem brain of neurotypical Black Americans reveals contributions of genetic ancestry
Source: Nat Neurosci. 2024 May 20;27(6):1064–74. doi: 10.1038/s41593-024-01636-0 (PMC11156587; doi:10.1038/s41593-024-01636-0)
Supplement: Supplementary file 5 — Compressed directory of ancestry-associated DEGs enriched for WGCNA module functional enrichment results (that is, GO term enrichment) for the caudate nucleus, dentate gyrus, DLPFC and hippocampus. [file 41593_2024_1636_MOESM5_ESM.gz › wgcna_functional_enrichment/caudate/module_pink_go_enrichment.pdf]

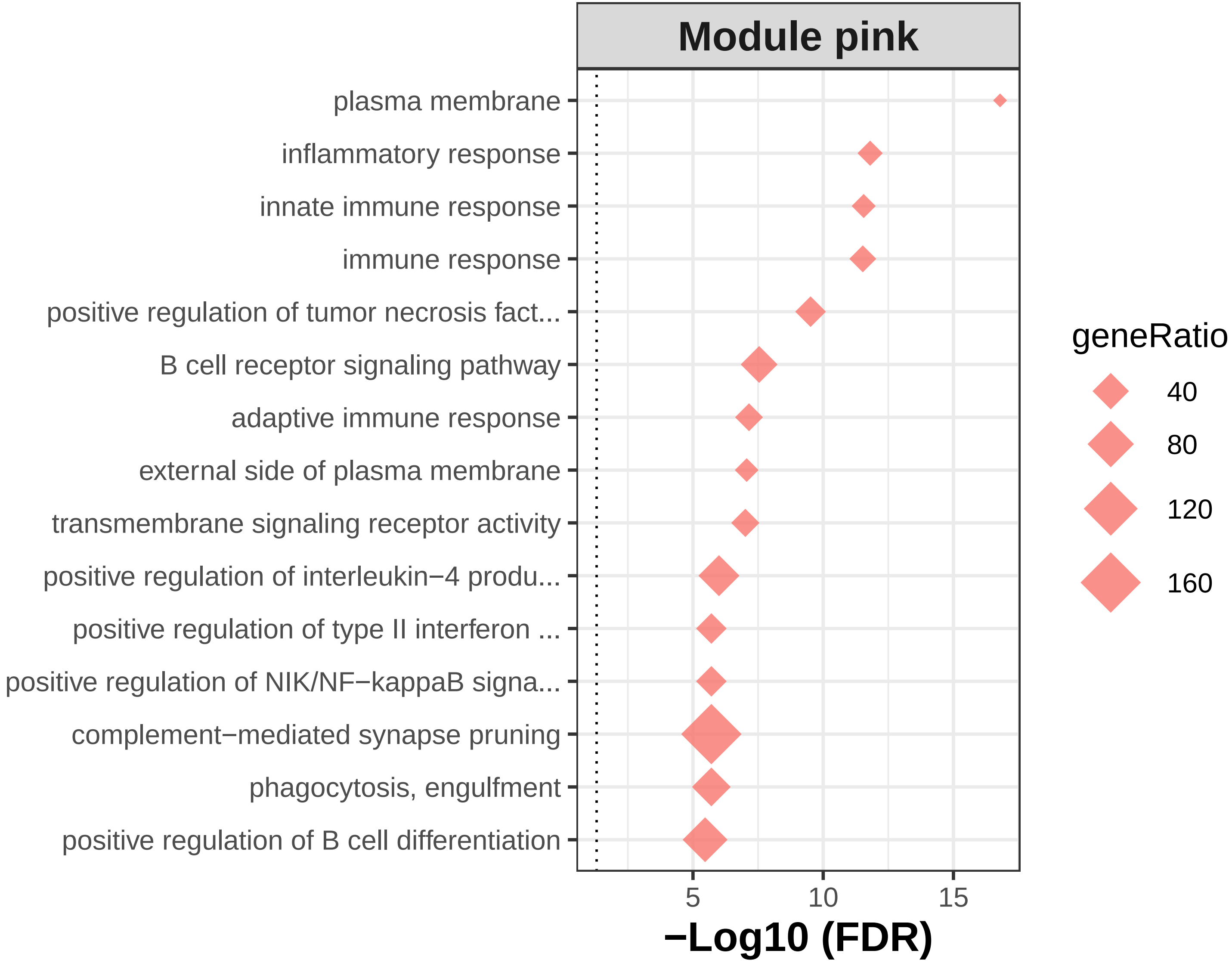

Module pink
plasma membrane
inflammatory response
innate immune response
immune response
positive regulation of tumor necrosis fact...
B cell receptor signaling pathway
adaptive immune response
external side of plasma membrane
transmembrane signaling receptor activity
positive regulation of interleukin−4 produ...
positive regulation of type II interferon ...
positive regulation of NIK/NF−kappaB signa...
complement−mediated synapse pruning
phagocytosis, engulfment
positive regulation of B cell differentiation
5
10
15
−Log10 (FDR)
geneRatio
40
80
120
160
